# c-Fos-driven metabolic switch of α-ketoglutarate orchestrates progression in prostate cancer

**DOI:** 10.1038/s41419-026-08918-4

**Published:** 2026-05-31

**Authors:** Liyan Ao, Zhiqiang Chen, Jingliang Zhang, Qi Wang, Jin Luo, Zhuoran Li, Qilong Jiao, Bobin Ning, Shiyuan Peng, Wenhao Hu, Yuqi Jia, Weimin Ci, Baojun Wang, Zhouhuan Dong, Xu Zhang, Shaoxi Niu

**Affiliations:** 1 Senior Department of Urology, Chinese PLA General Hospital, Beijing, China; 2 Graduate School of Chinese PLA General Hospital, Beijing, China; 3 Department of Urology, Xijing Hospital, Air Force Medical University, Xi’an, China; 4 School of Medicine, Nankai University, Nankai District, Tianjin, China; 5 Department of Pathology Medicine, Chinese PLA General Hospital, Beijing, China

**Keywords:** Cancer metabolism, Cancer therapeutic resistance, Prostate cancer

## Abstract

Prostate cancer is a highly heterogeneous malignancy, with distinct subtypes displaying unique molecular and metabolic profiles. This study identifies a compensatory shift in α-ketoglutarate (α-KG) metabolism in prostate cancer, where the tumor relies on IDH1 to incorporate citrate into the TCA cycle. IDH1 inhibition, leads to lower α-KG levels. Since α-KG is required for HIF-1α hydroxylation, IDH1 inhibition stabilizes HIF-1α, which subsequently upregulates c-Fos. C-Fos enhances GLUD1 transcription, promoting the conversion of glutamate to α-KG as a compensatory mechanism. Additionally, c-Fos upregulates downstream effectors, including FOXC1 and SOX2, driving neuroendocrine differentiation in prostate cancer. Targeting α-KG-metabolizing enzymes, such as IDH1 or GLUD1, presents promising therapeutic strategies for prostate cancer subtypes by inhibiting tumor proliferation and inducing oxidative stress, thus sensitizing tumors to ferroptosis. Overall, these findings uncover a metabolic adaptation in response to IDH1 inhibition and highlight the pivotal role of c-Fos in mediating this compensatory pathway, offering new insights into potential metabolic targets for prostate cancer treatment and ferroptosis-based therapies.

## Introduction

Androgen deprivation therapy (ADT) is a standard of care for prostate cancer, demonstrating effectiveness in the early stages of disease management [1, 2]. However, most patients eventually progress to castration-resistant prostate cancer (CRPC) [3, 4], for which effective therapeutic options remain scarce. In addition, a subset of patients may develop neuroendocrine prostate cancer (NEPC), characterized by neuroendocrine (NE) marker expression and the loss of androgen receptor (AR) expression [5–8], conferring resistance to ADT. Although key drivers of prostate cancer progression—such as MYC activation [9, 10], loss of tumor suppressors such as PTEN, TP53 and RB1 [11], and dysregulation of epigenetic, microenvironmental, and immune pathways [12]—have been reported, the underlying mechanisms remain incompletely understood. Once this progression occurs, treatment options are limited, posing a significant clinical challenge [13, 14].

As tumors progress, they reprogram their metabolism to enhance nutrient uptake and energy utilization, adaptations that support growth and survival but may also reveal metabolic vulnerabilities [15, 16]. Normal prostate epithelial cells exhibit a distinctive tricarboxylic acid (TCA) cycle profile, with high intracellular zinc levels inhibiting aconitase, thereby disrupting citrate production [17–19]. Physiologically, high citrate concentrations in prostatic fluid help maintain sperm viability [20, 21]. In prostate cancer, the tumor microenvironment retains elevated citrate levels even after malignant transformation, suggesting that cancer cells may preferentially exploit this abundant metabolite.

Prostate cancer utilizes cytosolic isocitrate dehydrogenase (IDH) to generate α-ketoglutarate (α-KG), replenishing the TCA cycle [22, 23]. The expression of IDH is regulated by AR, yet after ADT, IDH inhibition does not consistently suppress the TCA cycle or tumor growth [24–26]. Beyond its role in energy metabolism, α-KG has been identified as a signaling molecule that contributes to tumor progression [27, 28]. It is hypothesized that metabolic adaptations occur during prostate cancer progression under ADT, compensating for IDH inhibition and α-KG depletion. This study documents these adaptive metabolic alterations, which may provide new insights into developing targeted therapies that exploit the metabolic vulnerabilities of this disease.

## Results

### α-KG metabolism is a unique metabolic process that distinguishes distinct subgroups of prostate cancers

**Fig. 1:**
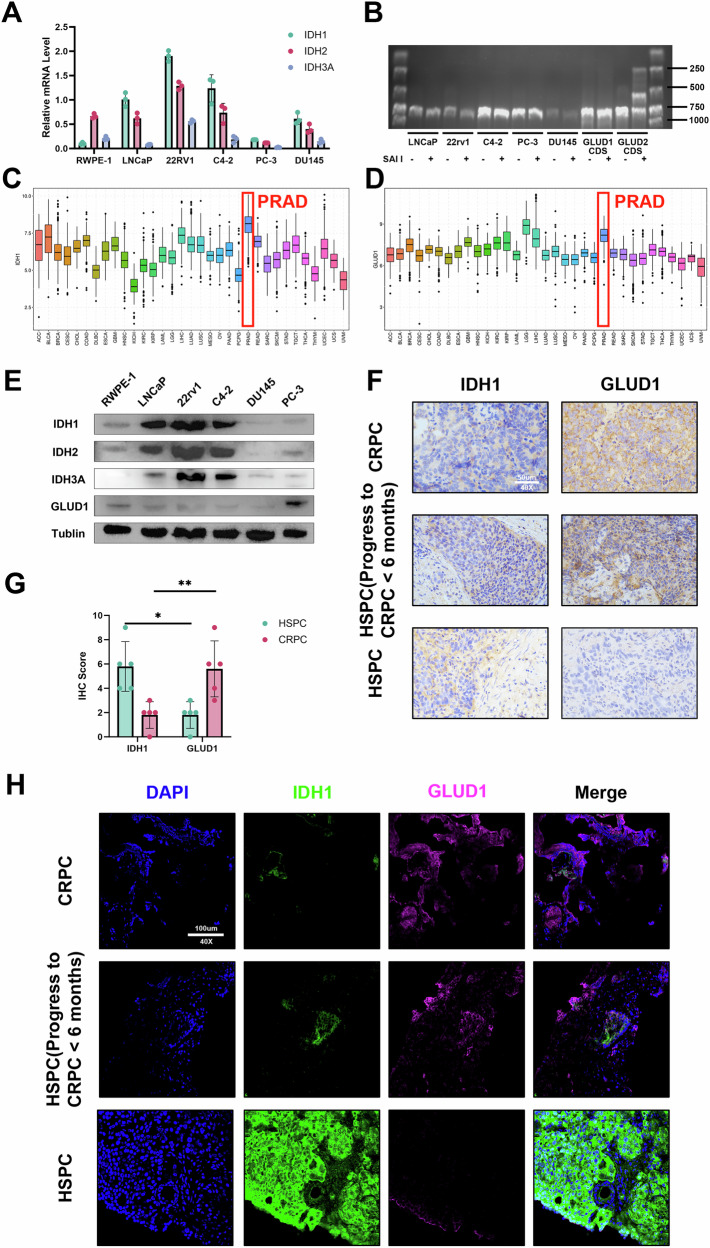
Expression of α-KG-Metabolizing enzymes in distinct prostate cancer subtypes. **A** Relative mRNA expression levels of IDH isoforms in RWPE-1, AR-positive (LNCaP, 22Rv1, C4-2) and AR-negative (PC-3, DU145) prostate cancer cell lines. **B** DNA electrophoresis results of the digestion assay performed for characterization of major GLUD isoforms in prostate cancer. **C**, **D** Pan-cancer analysis of α-KG-associated metabolic enzymes expression levels in tumors from TCGA database. **E** Immunoblots showing protein levels of the α-KG-associated metabolic enzymes in prostate cancer cell lines. **F**, **G** Immunohistochemical staining and scoring of IDH1 and GLUD1 in hormone-sensitive versus castration-resistant patients at our institution. **H** Multiplex immunofluorescence staining for IDH1 and GLUD1 in hormone-sensitive versus castration-resistant patients. IDH Isocitrate Dehydrogenase, GLUD Glutamate Dehydrogenase, α-KG α-Ketoglutarate, HSPC hormone-sensitive prostate cancer, CRPC castration-resistant prostate cancer. *p < 0.05, **p < 0.01.

Research by Costello et al. has demonstrated that prostate cancer cells thrive in a microenvironment with elevated citrate concentrations [29]. These tumor cells lose their original physiological functions and adapt by enhancing energy acquisition to support proliferation, making citrate an ideal metabolic fuel. In the TCA cycle, citrate is converted to α-KG by IDH, a key metabolic intermediate that further enters the TCA cycle for subsequent reactions. α-KG also participates in a reversible reaction catalyzed by glutamate dehydrogenase (GLUD). To investigate the expression of α-KG metabolic enzymes in prostate cancer, the specific isoforms of both IDH and GLUD were identified. mRNA expression levels were assessed to characterize the various IDH isoforms in the benign prostatic epithelial cell line RWPE-1 and several prostate cancer cell lines, including AR-positive LNCaP, 22Rv1, and C4-2 cells, as well as AR-negative PC-3 and DU145 lines. AR-positive prostate cancer cells exhibited higher IDH expression compared to RWPE-1, with IDH1 being the predominant isoform in the cancerous lines, whereas IDH2 was predominant in RWPE-1. IDH expression was higher in AR-positive cell lines and significantly lower in AR-negative cell lines (Fig. 1A). Typically, the mitochondrial isoforms IDH2/IDH3 mediate this step in most tissues, generating NADH to fuel oxidative phosphorylation for energy production. In contrast, the predominance of the cytosolic isoform IDH1 in prostate cancer highlights a distinctive metabolic feature of this malignancy. Regarding GLUD, the high homology between its isoforms GLUD1 and GLUD2 can lead to confusion with conventional RT-qPCR. Therefore, a digestion assay based on the *SAI I* restriction enzyme site difference within their cDNA confirmed that GLUD1 is the predominant isoform in prostate cancer (Supplementary Fig. S1A and Fig. 1B).

A pan-cancer analysis using the TCGA database revealed that prostate cancer ranks among the tumor types with notably high expression levels of both IDH1 and GLUD1 (Fig. 1C, D). Similarly, analysis of public databases reveals elevated expression of GLUD1 in patients with prostate cancer, and patients with high IDH1 expression exhibit a poorer prognosis (Supplementary Fig. S1B, C). Protein analysis showed that IDH1 is highly expressed in AR-positive cell lines, while other IDH isoforms were expressed at lower levels. GLUD1 expression was lower in AR-positive cell lines but elevated in the NE-like PC-3 cell line. In double-negative prostate cancer (DNPC), both IDH1 and GLUD1 were expressed at low levels (Fig. 1E). Patient specimens were collected from our center and classified into hormone-sensitive and castration-resistant groups based on their response to ADT. Notably, one patient, initially sensitive to ADT, progressed to CRPC within six months. Immunohistochemical staining revealed higher IDH1 expression in hormone-sensitive patients, while castration-resistant patients showed elevated GLUD1 expression. In the case of the rapidly progressing patient, both IDH1 and GLUD1 were expressed at significant levels (Fig. 1F, G). These findings were further corroborated by multiplex immunofluorescence (Fig. 1H). This evidence confirms that α-KG metabolic enzyme expression varies significantly across prostate cancer subtypes, with distinct characteristics of α-KG metabolism in each subtype.

### Inhibition of key enzymes in α-KG metabolism suppresses prostate cancer proliferation

**Fig. 2:**
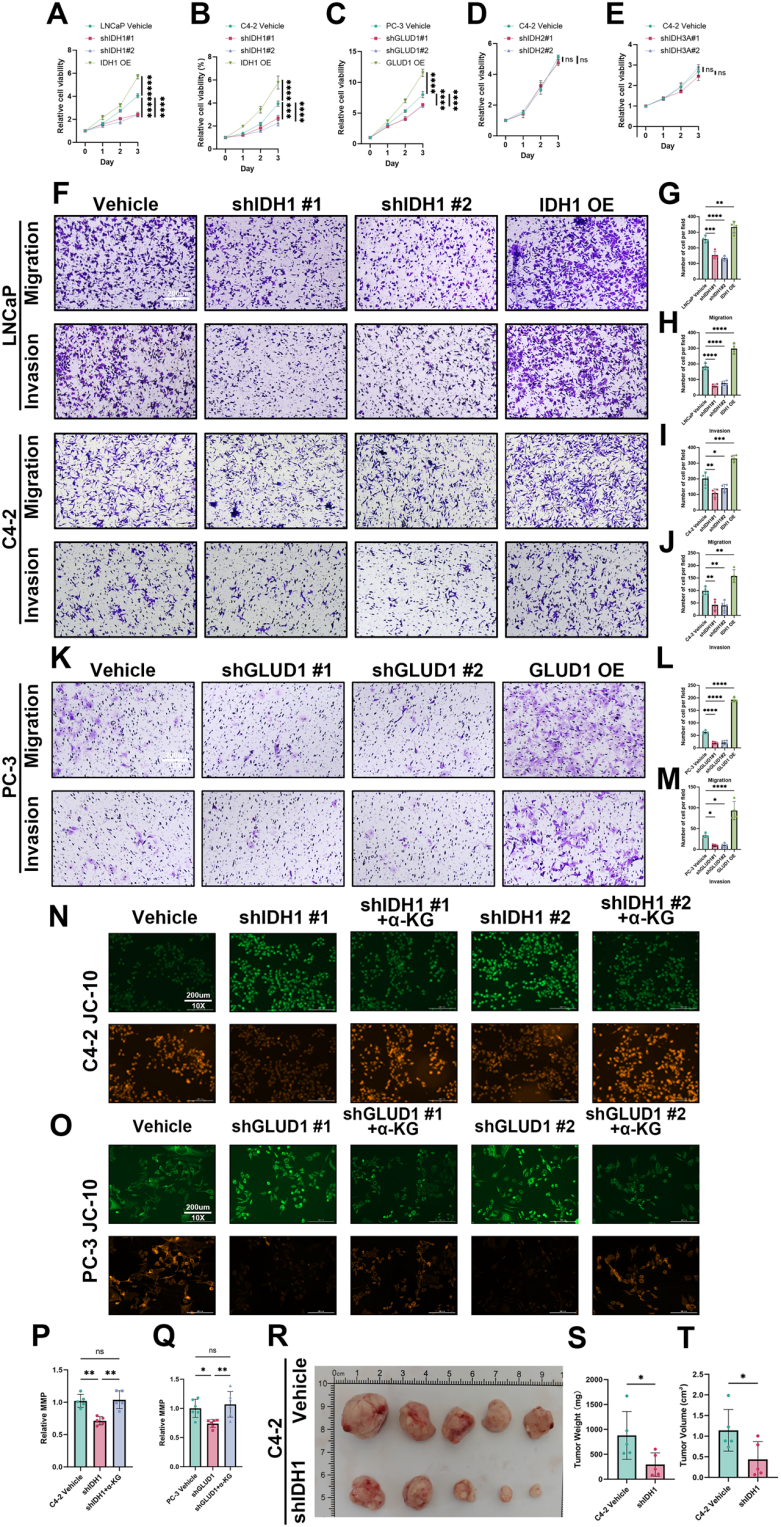
Inhibition of α-KG metabolism inhibits prostate cancer cell growth in vitro and in vivo. **A**, **B** Relative cell viability of LNCaP and C4-2 cells under different conditions: vehicle control, IDH1 knockdown and IDH1 overexpression. **C** Relative cell viability of PC-3 cells expressing either control vehicle and GLUD1 knockdown. **D**, **E** Relative cell viability of C4-2 cells under different conditions: vehicle control and IDH2 or IDH3A knockdown. **F** Representative images of transwell invasion and migration assays in LNCaP and C4-2 cells following treatments with vehicle, IDH1 knockdown and IDH1 overexpression. **G**–**J** Microscopic cell counting and quantitative analysis of the transwell assay in LNCaP and C4-2 cells. **K** Representative images of transwell invasion and migration assays in PC-3 cells following treatments with vehicle, GLUD1 knockdown and GLUD1 overexpression. **L**, **M** Microscopic cell counting and quantitative analysis of the transwell assay in PC-3 cells. **N**, **O** Fluorescence micrographs of JC-10 staining showing mitochondrial membrane potential in C4-2 and PC-3 cells under indicated treatments. **P**, **Q** Quantitative analysis of MMP in C4-2 and PC-3 cells under indicated treatments. **R**–**T** Xenograft tumors harvested from nude mice inoculated with C4-2 cells expressing either control vehicle or IDH1 knockdown and quantitative measurement of tumor weight and volume. **N**–**Q** Cells were cultured in the presence or absence of α-KG (1 mM). IDH Isocitrate Dehydrogenase, GLUD Glutamate Dehydrogenase, α-KG α-Ketoglutarate, OE overexpression, MMP mitochondrial membrane potential. ns *p* > 0.05, *p < 0.05, **p < 0.01, ***p < 0.001, ****p < 0.0001.

This study further investigated whether inhibiting α-KG metabolic enzymes could suppress prostate adenocarcinoma growth. In AR-positive prostate cancer cells (LNCaP and C4-2), knockdown of IDH1 significantly inhibited cell proliferation. Similarly, in NE-like prostate cancer cell lines (PC-3), knockdown of GLUD1 also suppressed proliferation (Fig. 2A–C). To determine whether IDH2 or IDH3 are similarly critical for prostate cancer survival, this study knocked down IDH2 or IDH3A (the catalytic subunit of IDH3), but neither affected proliferation, further confirming the dominant role of IDH1 in prostate cancer (Fig. 2D, E). Subsequent transwell migration and invasion assays revealed that inhibiting these metabolic enzymes reduced metastatic potential (Fig. 2F–M).

As α-KG is a key metabolite in the TCA cycle, its deficiency disrupts mitochondrial TCA cycling and induces mitochondrial dysfunction. After inhibiting IDH1 and GLUD1, a decrease in mitochondrial membrane potential was observed in C4-2 and PC-3 cells, indicating mitochondrial instability (Fig. 2N–Q). Finally, in vivo experiments confirmed the tumor-suppressive effects of IDH1 inhibition. Knockdown of IDH1 resulted in significantly reduced tumor weight in subcutaneous xenografts in nude mice, compared to wild-type cells (Fig. 2R–T). In summary, α-KG metabolism is critical at all stages of prostate cancer, and inhibition of IDH1 or GLUD1 effectively suppresses prostate cancer metabolism.

### Inhibition of α-KG metabolic enzymes in prostate cancer induces oxidative stress

IDH1 exhibits a distinct feature among IDH isoforms: it utilizes NADP⁺ as a coenzyme to generate α-KG while concurrently producing NADPH. In contrast, normal cells primarily utilize IDH2 or IDH3 within mitochondria, which use NAD⁺ or NADP⁺ to generate NADH and NADPH. Mitochondrial NADH fuels oxidative phosphorylation for energy metabolism [30], while NADPH serves as a critical reducing equivalent for cellular reduction reactions, maintaining redox homeostasis [31].

**Fig. 3:**
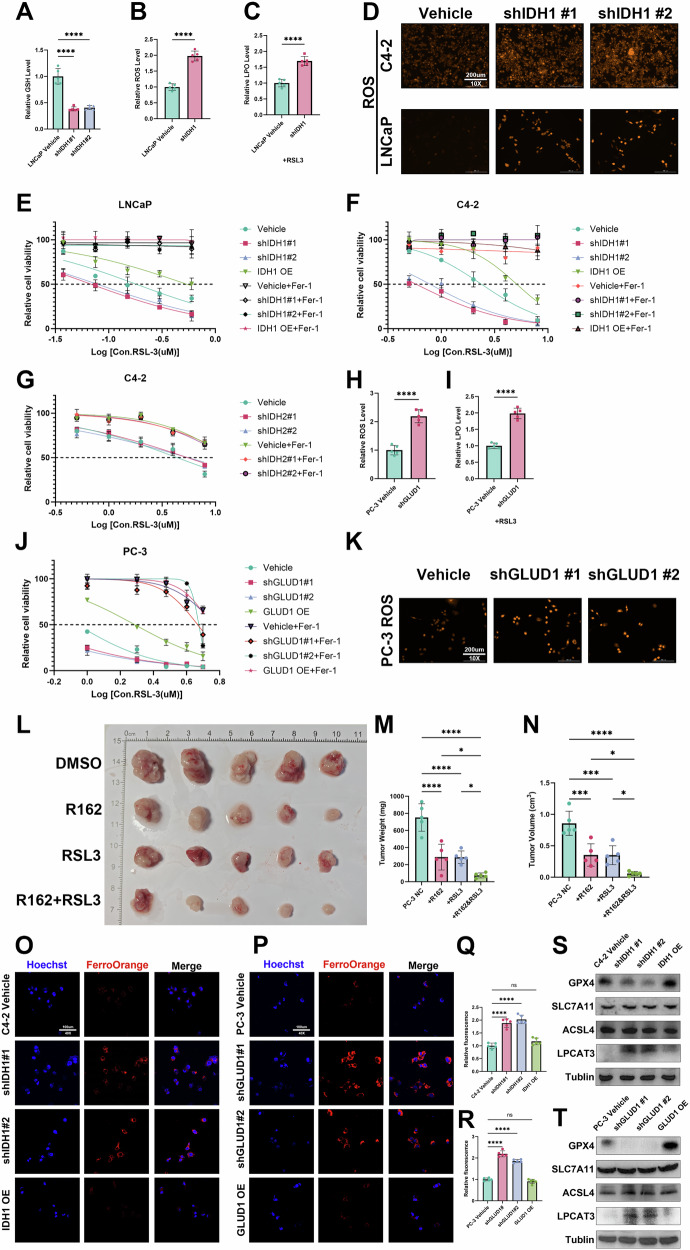
Combined inhibition of α-KG-metabolizing enzymes and ferroptosis induction effectively suppresses prostate cancer cell growth in vitro and in vivo. **A**, **B** Relative GSH and ROS levels of C4-2 cell expressing either control vehicle or IDH1 knockdown. **C** Relative LPO levels in C4-2 cells with IDH1 knockdown or vehicle expressing RSL3 treatment. **D** Representative immunofluorescence images of ROS in C4-2 and LNCaP cells with IDH1 knockdown or vehicle. **E**–**G** Relative viability of C4-2 and LNCaP cells with IDH1 or IDH2 knockdown or overexpression and vehicle treated with RSL3, in the presence or absence of Fer-1 (10 μM). **H** Relative ROS levels of PC-3 cell expressing either control vehicle or GLUD1 knockdown. **I** Relative LPO levels in PC-3 cells with GLUD1 knockdown or vehicle expressing RSL3 treatment. **J** Relative viability of PC-3 cells with GLUD1 knockdown or overexpression and vehicle treated with RSL3, in the presence or absence of Fer-1. **K** Representative immunofluorescence images of ROS in PC-3 cells with GLUD1 knockdown or vehicle. **L**–**N** Xenograft tumors harvested from nude mice inoculated with PC-3 cells expressing RSL3 or R162 and quantitative measurement of tumor weight and volume. **O**, **P** Detection of Ferrous ions in live cells using the FerroOrange probe following RSL3 treatment. **Q**, **R** Quantitative analysis of fluorescence intensity following FerroOrange probe labeling. **S**, **T** Immunoblots in C4-2 and PC-3 cells treated with RSL3 versus wild-type cells. **C**, **I** Cells were cultured in the presence of RSL3 (5 μM) for 8 h. **L**–**N** Control mice were injected intraperitoneally with an equal volume of the drug vehicle, whereas the treatment groups received daily injections of RSL3 (20 mg/kg/d) or R162 (10 mg/kg/d). **O**–**T** Cells were cultured in the presence of RSL3 (5 μM) for 8 h. GSH Glutathione, ROS Reactive Oxygen Species, LPO Lipid Peroxidation, DMSO Dimethyl Sulfoxide, IDH Isocitrate Dehydrogenase, GLUD Glutamate Dehydrogenase. ns *p* > 0.05, *p < 0.05, ***p < 0.001, ****p < 0.0001.

It is hypothesized that prostate cancer cells preferentially rely on IDH1-driven metabolism to sustain elevated reducing equivalents for redox balance. To test this, intracellular reduced glutathione (GSH), the primary reducing agent for maintaining cellular redox homeostasis [32], was measured after inhibiting IDH1. A significant decrease in GSH levels was observed, accompanied by a marked increase in reactive oxygen species (ROS) (Fig. 3A–D). This study further investigated ferroptosis, a cell death mechanism triggered by disrupted redox homeostasis [33], as a potential vulnerability in prostate cancer. IDH1 knockdown significantly increased lipid peroxide levels following short-term RSL3 treatment. The IC_50_ of RSL3 was reduced in LNCaP and C4-2 cells with IDH1 knockdown, an effect that could be rescued by the ferroptosis inhibitor Ferrostatin-1 (Fer-1) (Fig. 3E, F). While IDH2, an NADP+-dependent isoform located within the mitochondria, also generates α-KG and NADPH, our results showed that inhibiting IDH2 did not significantly affect prostate cancer cells’ sensitivity to RSL3 (Fig. 3G), confirming that IDH1 plays the primary role in maintaining redox homeostasis in prostate cancer.

GLUD1 shares functional similarities with IDH1, as both generate α-KG and NADPH. Beyond replenishing TCA cycle intermediates, GLUD1 may also compensate for redox homeostasis. In the NE-like prostate cancer line PC-3, GLUD1 knockdown similarly elevated ROS levels and enhanced sensitivity to RSL3, with a concomitant increase in lipid peroxidation (LPO) (Fig. 3H–K). Finally, in vivo experiments confirmed these findings: in PC-3 xenograft models, intraperitoneal injection of RSL3 or the GLUD1 inhibitor R162 reduced tumor volume, while combination therapy demonstrated superior efficacy (Fig. 3L–N). Our findings demonstrate that inhibition of α-KG metabolic enzymes enhances susceptibility to ferroptosis by disrupting cellular redox homeostasis. The induction of ferroptosis is also accompanied by dysregulated iron metabolism, suppression of the antioxidant defense system, and accumulation of lipid peroxidation. Following RSL3 treatment, we further examined additional ferroptosis-related indicators. First, we observed a significant increase in intracellular ferrous ions levels upon inhibition of α-KG metabolic enzymes (Fig. 3O–R). Moreover, the expression of GPX4, a key molecule in the antioxidant defense system, was downregulated after knockdown of the metabolic enzymes and upregulated upon their overexpression, whereas SLC7A11 expression remained unchanged. Similarly, the expression of LPCAT3, which mediates lipid peroxidation, was elevated following knockdown of the metabolic enzymes, while ASCL4 showed no significant alteration (Fig. 3S, T).

### Metabolic enzyme switch mediated by α-KG deficiency during prostate cancer progression

**Fig. 4:**
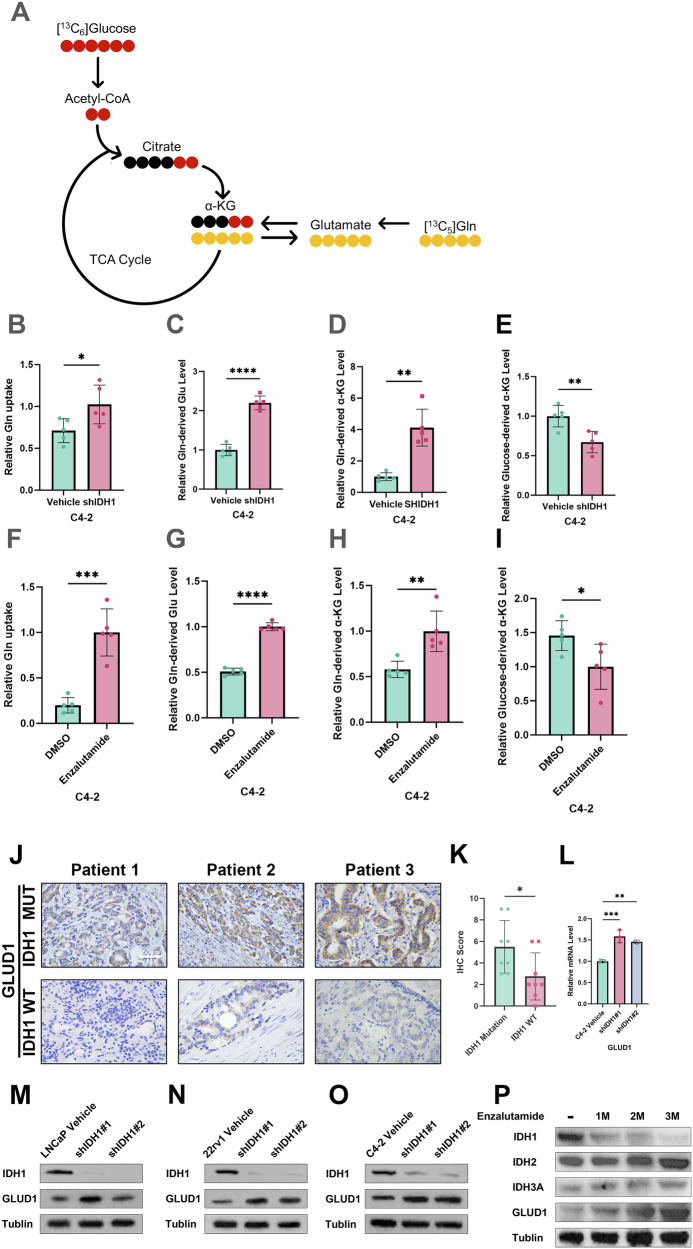
IDH1 inhibition drives a metabolic switch in prostate cancer following α-KG level reduction. **A**–**E** Tracing of 13C-labeled glutamine and glucose influx and mass isotopomer analysis of metabolites in shIDH1-C4-2 cells. **F**–**I** Tracing of 13C-labeled glutamine and glucose influx and mass isotopomer analysis of metabolites in C4-2 cells treated with enzalutamide. **J**, **K** Immunohistochemical staining of GLUD1 in IDH1-Mutated versus IDH1-Wild-Type prostate cancers from patients. **L** The relative mRNA expression level of GLUD1 in shIDH1-C4-2 cells versus vehicle control. **M**–**O** Immunoblots in shIDH1-LNCaP cells, shIDH1-22rv1 cells and shIDH1-C4-2 cells versus vehicle control. **P** Immunoblots in C4-2 cells treated with enzalutamide for various durations (1 month to 3 months) versus wild-type cells. IDH Isocitrate Dehydrogenase, GLUD Glutamate Dehydrogenase. *p < 0.05, **p < 0.01, ***p < 0.001, ****p < 0.0001.

Given the correlation between the expression trends of IDH1 and GLUD1 and AR expression levels in prostate cancer cell lines (Fig. 1E), it was hypothesized that α-KG metabolic enzyme expression and resultant α-KG levels drive disease progression. We observed a significantly higher ratio of GLUD1 to IDH1 in PC-3 cells compared to LNCaP cells (Supplementary Fig. S1D). To investigate this, ^13^C-labeled metabolic flux analysis was employed to delineate this metabolic switch (Fig. 4A). The results demonstrated that IDH1 inhibition in prostate cancer cells led to increased exogenous glutamine uptake. Consequently, the proportion of α-KG derived from glutamine increased, while the contribution from glucose decreased (Fig. 4B–E). Metabolic flux analysis of C4‑2 cells following 3‑month enzalutamide treatment versus DMSO‑treated controls revealed a consistent trend. This in vitro model, which better recapitulates the ADT process, further demonstrates the occurrence of metabolic switch during prostate cancer progression (Fig. 4F–I). The IDH1-mutant enzyme, which has a reduced ability to utilize isocitrate for α-KG and NADPH production, instead consumes α-KG and NADPH to generate 2-hydroxyglutarate (2-HG) [34]. This metabolic shift allowed for direct assessment of the alterations in patients following functional IDH1 inhibition. In a cohort of prostate patients from our hospital and Xijing Hospital of Air Force Medical University, immunohistochemical staining revealed that IDH1-mutant patients exhibited significantly elevated GLUD1 expression compared to IDH1 wild-type patients (Fig. 4J, K). We observed an upregulation of GLUD1 levels following IDH1 knockdown in various AR-positive prostate cancer cell lines (Fig. 4L–O). Furthermore, comparison of wild-type and enzalutamide-resistant C4-2 cell lines showed that enzalutamide-resistant cells exhibited both decreased IDH1 expression and increased GLUD1 expression (Fig. 4P), further supporting this conclusion.

In summary, these results demonstrate that during the progression of prostate cancer following ADT, IDH1 is downregulated, while GLUD1 is upregulated, a pattern that is also confirmed in patient samples.

### Hif-1α-regulated c-Fos promotes metabolic switch in prostate cancer following IDH1 inhibition

**Fig. 5:**
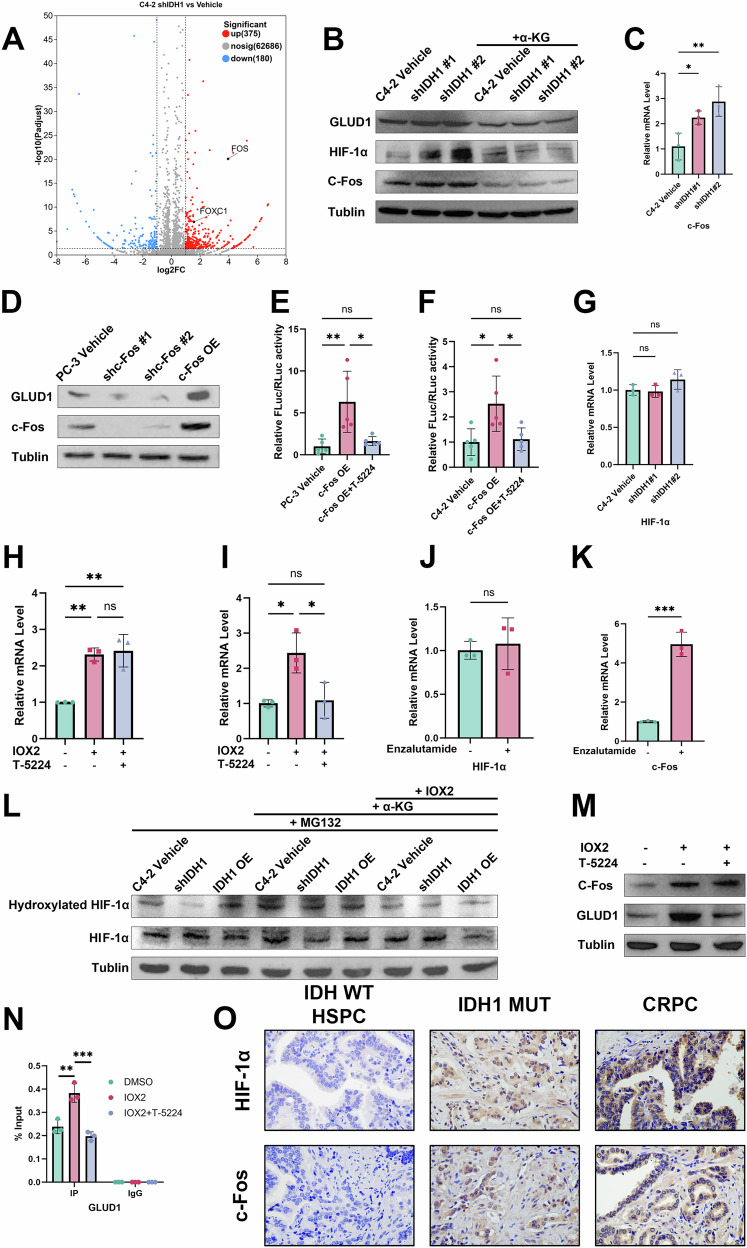
Hif-1α-regulated c-Fos promotes metabolic switch in prostate cancer. **A** Volcano plot of transcriptome sequencing differentially expressed genes between C4-2 cells with IDH1-knockdown and vehicle control. **B** Immunoblots showing protein levels in C4-2 cells with vehicle control, IDH1 knockdown, treated with or without α-KG. **C** Relative mRNA expression levels of c-Fos in C4-2 cells with IDH1 knockdown and vehicle control. **D** Immunoblots showing protein levels in C4-2 cells under different conditions: vehicle control, c-Fos knockdown and c-Fos overexpression. **E**, **F** Dual-luciferase assay results demonstrating the regulation of the GLUD1 promoter sequence by c-Fos overexpression or its inhibition by T-5224 in PC-3 and C4-2 cells. **G** Relative mRNA expression levels of HIF-1α in C4-2 cells with IDH1 knockdown and vehicle control. **H**, **I** Relative mRNA expression levels of HIF-1α and c-Fos in C4-2 cells treated with DMSO, IOX2 or T-5224. **J**, **K** Relative mRNA expression levels of HIF-1α and c-Fos in C4-2 cells treated with DMSO and enzalutamide. **L** Immunoblots showing protein levels in C4-2 cells under different conditions: vehicle control, IDH1 knockdown and IDH1 overexpression, and treatments with MG132, α-KG, or IOX2. **M** Immunoblots showing protein levels in C4-2 cells treated with DMSO, IOX2 or T-5224. **N** ChIP-qPCR analysis of the GLUD1 promoter in PC-3 cells treated with DMSO, IOX2 or T-5224. **O** Immunohistochemical staining of HIF-1α and c-Fos in patients with CRPC, IDH1-mutated disease and IDH1-wild-type HSPC. **B**, **L** Cells were cultured in the presence or absence of α-KG (1 mM). **E**, **F** Cells were cultured in the presence or absence of T-5224 (20 μM). **H**, **I**, **N** Cells were cultured in the presence or absence of IOX2 (20 μM) or T-5224 (20 μM). **J**, **K** Cells were cultured in the presence or absence of enzalutamide (20 μM).α-KG α-Ketoglutarate, IDH Isocitrate Dehydrogenase, GLUD Glutamate Dehydrogenase, OE overexpression. ns *p* > 0.05, *p < 0.05, **p < 0.01.

To further investigate the factors driving metabolic enzyme conversion in prostate cancer following IDH1 inhibition, RNA-seq analysis of IDH1-knockdown cells revealed significant upregulation of c-Fos (Fig. 5A). Protein-level assays confirmed this finding, and supplementation of α-KG to the culture medium reduced c-Fos expression, indicating that c-Fos is regulated by intracellular α-KG levels (Fig. 5B, C).

Subsequently, c-Fos was knocked down or overexpressed in C4-2 cells. c-Fos knockdown resulted in the downregulation of GLUD1, while c-Fos overexpression led to an upregulation of GLUD1 expression (Fig. 5D). As c-Fos is a key transcription factor, bioinformatic analysis was performed to predict potential binding sites on the GLUD1 promoter. Using a dual-luciferase reporter system with the GLUD1 promoter sequence, significant luminescence increase was observed upon c-Fos overexpression. This effect was rescued by T-5224, an inhibitor of c-Fos–DNA binding (Fig. 5E, F), confirming that c-Fos directly regulates GLUD1 transcription.

Given that α-KG directly regulates c-Fos, this study hypothesized the involvement of HIF-1α signaling, as α-KG modulates HIF-1α through two mechanisms: first, as a substrate for TET enzymes [35, 36], it regulates HIF-1α gene methylation to enhance HIF-1α transcription; second, as a substrate for dioxygenases, it participates in HIF-1α hydroxylation, promoting proteasomal degradation and reducing HIF-1α stability [37, 38].

HIF-1α mRNA levels were measured after IDH1 inhibition, and no significant difference was observed compared to vector controls (Fig. 5G). However, treatment with IOX2 (a HIF-1α hydroxylation inhibitor) markedly upregulated c-Fos and GLUD1, an effect that was rescued by T-5224 (Fig. 5H, I). Following Enzalutamide treatment, the mRNA level of HIF-1α in prostate cancer showed no significant change, while the mRNA level of c-Fos significantly increased, suggesting that α-KG might regulate HIF-1α levels by modulating its stability during IDH1 inhibition (Fig. 5J, K).

Moreover, inhibiting protein degradation with MG132 revealed that IDH1 knockdown reduced hydroxylated HIF-1α levels. This was reversed by exogenous α-KG supplementation, and IOX2 rescued the phenotype, confirming that IDH1–α-KG regulates HIF-1α stability and hypoxia signaling-driven c-Fos–GLUD1 activation (Fig. 5L). Similarly, the application of IOX2 led to the upregulation of c-Fos and GLUD1 expression in C4-2 cells. However, the administration of T-5224, which inhibits the DNA-binding capacity of c-Fos, resulted in a subsequent downregulation of GLUD1 expression (Fig. 5M). ChIP-qPCR analysis confirmed the binding of the transcription factor c-Fos to the GLUD1 promoter region. This interaction was enhanced by IOX2 and suppressed by T-5224, further validating the regulatory pathway identified (Fig. 5N). Based on clinical specimens from our institution, we performed immunohistochemical analysis on tumor tissue sections from patients with IDH1‑wild‑type (and HSPC), IDH1‑mutant, and CRPC. The results showed that IDH1‑mutant and CRPC samples exhibited significantly higher expression of HIF‑1α and c‑Fos, along with prominent nuclear localization (Fig. 5O). These findings from clinical specimens further confirm the upregulation of the HIF‑1α/c‑Fos axis during tumor progression.

### c-Fos–FOXC1–SOX2 to drive neuroendocrine progression in prostate cancer

**Fig. 6:**
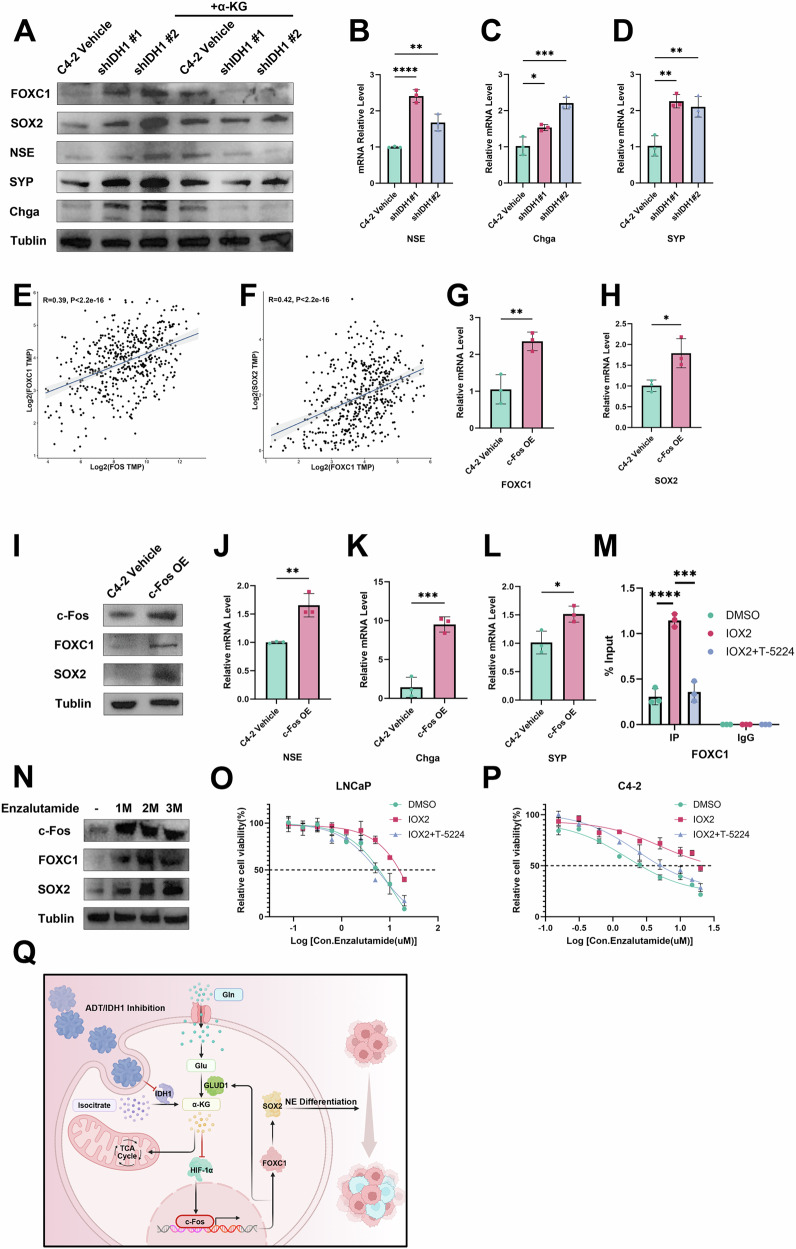
c-Fos drive progression in prostate cancer and schematic mechanism of our study. **A**: Immunoblots showing protein levels in PC-3 cells with IDH1 knockdown or vehicle control and treatments with α-KG. **B**–**D** Relative mRNA expression levels of Chga, SYP and NSE in C4-2 cells with IDH1 knockdown and vehicle control. **E**, **F** Results of mRNA correlation analysis from TCGA database. **G**, **H** Relative mRNA expression levels of FOXC1 and SOX2 in C4-2 cells with c-Fos overexpression and vehicle control. **I** Immunoblots showing protein levels in PC-3 cells with c-Fos overexpression and vehicle control. **J**–**L** Relative mRNA expression levels of Chga, SYP and NSE in PC3 cells with c-Fos overexpression. **M** ChIP-qPCR analysis of the FOXC1 promoter in PC-3 cells treated with DMSO, IOX2 or T-5224. **N** Immunoblots showing protein levels in C4-2 cells treated with enzalutamide (1 month to 3 months). **O**, **P** Relative viability of PC-3 cells cultured with DMSO, IOX2 or T-5224 treated with enzalutamide. **Q** Schematic diagram of the found regulatory axis identified in the study. A Cells were cultured in the presence or absence of α-KG (1 mM). **M**, **O** and **P** Cells were cultured in the presence or absence of IOX2 (20 μM) or T-5224 (20 μM). α-KG α-Ketoglutarate, IDH Isocitrate Dehydrogenase, GLUD Glutamate Dehydrogenase, OE Overexpression, NSE Neuron Specific Enolase, Chga Chromogranin A, SYP Synaptophysin. *p < 0.05, **p < 0.01, ***p < 0.001, ****p < 0.0001.

This study further investigated whether the expression levels of metabolic enzymes and α-KG content directly promote prostate cancer progression. In IDH1-knockdown prostate cancer cells, upregulation of NE markers (NSE, Chga, SYP) was observed (Fig. 6A), which was reversed by α-KG supplementation. mRNA-level analyses confirmed that intracellular α-KG levels modulate NE differentiation (Fig. 6B–D).

RNA-seq data revealed that IDH1 inhibition significantly upregulated FOXC1 (Fig. 5A). Correlation analysis of TCGA datasets demonstrated a positive association between FOXC1 and c-Fos mRNA expression (Fig. 6E). FOXC1, known for regulating embryonic development, drives downstream targets, including SOX2 [39], a key factor in prostate cancer NE differentiation [5, 40]. Further mRNA correlation analysis confirmed a positive link between FOXC1 and SOX2 expression in prostate cancer (Fig. 6F). The specific binding of c-Fos to the FOXC1 promoter, an interaction enhanced by IOX2 and suppressed by T-5224, was validated. In PC-3 cells, c-Fos overexpression induced upregulation of FOXC1 and SOX2 (Fig. 6G–I), along with increased NE marker expression (Fig. 6J–L), validating this regulatory axis. ChIP-qPCR results further confirmed the binding of c-Fos to the FOXC1 promoter (Fig. 6M). In C4-2 cells treated long-term with enzalutamide (1 month to 3 months), c-Fos levels progressively increased, paralleling rises in FOXC1 and SOX2 (Fig. 6N), further supporting this mechanism.

To investigate the impact of hypoxia signaling, it was enhanced in LNCaP and C4-2 cells using IOX2. Hypoxia-primed cells exhibited enhanced enzalutamide resistance, which was reversed by T-5224, restoring drug sensitivity (Fig. 6O, P). NE marker expression and enzalutamide sensitivity assays confirmed that hypoxia-driven c-Fos–FOXC1–SOX2 signaling promotes NE progression.

In summary, our research uncovers the following mechanisms: In AR-positive prostate cancer, the isocitrate dehydrogenation reaction catalyzed by the IDH1 enzyme generates α-KG, which enters the TCA cycle, while NADPH helps maintain intracellular redox homeostasis. During ADT treatment, IDH1 inhibition suppresses PHD2 function, which uses α-KG as a substrate, resulting in reduced HIF-1α hydroxylation and decreased degradation. This leads to the stabilization of HIF-1α, promoting the expression of c-Fos. Metabolically, c-Fos directly enhances GLUD1 transcription, compensating for α-KG and NADPH levels through the glutamate dehydrogenation reaction. Additionally, c-Fos promotes the expression of downstream factors FOXC1 and SOX2, driving NE differentiation and facilitating resistance to ADT. In prostate cancers with distinct α-KG metabolic profiles, inhibiting the corresponding metabolic enzymes can effectively induce oxidative stress and kill tumor cells (Fig. 6Q).

## Discussion

Prostate cancer is characterized by significant heterogeneity [41], manifested at two levels: the coexistence of distinct molecular subgroups within the primary tumor and the dynamic genomic evolution driven by ADT [3, 42]. Our research highlights the metabolic features related to α-KG in both AR-positive and NE-like prostate cancers and identifies an adaptive metabolic switch. In AR-positive prostate cancer, IDH1 is highly expressed to utilize citrate, restoring the TCA cycle. However, following AR signaling inhibition, IDH1 activity is suppressed. Despite this, tumor energy metabolism remains unaffected, as GLUD1 is compensatorily upregulated in response to IDH1 suppression, replenishing the TCA cycle through glutamate dehydrogenation. The core driver of this metabolic shift is α-KG levels. Reduced α-KG levels inhibit HIF-1α hydroxylation, resulting in its stabilization and subsequent transcriptional upregulation of c-Fos, which promotes GLUD1 expression.

During the early characterization of the GLUD1 cDNA, it was hypothesized that its promoter region contained binding sites for AP-1 proteins [43], with c-Fos as a primary subunit. The existence of this regulatory mechanism has been confirmed in prostate cancer in our research. Therefore, our findings demonstrate that c-Fos drives the metabolic adaptive switch in prostate cancer.

While the drivers of prostate cancer progression to CRPC and NEPC remain incompletely defined, current research has uncovered multiple underlying mechanisms. Among these, genomic and epigenetic alterations have been extensively studied and provide compelling evidence [44]. In recent years, dysregulation of transcription factors has emerged as a significant contributor to disease progression. Notably, transcription factors such as NMYC and ASCL1 have been identified as key regulators of the neuroendocrine phenotype [45]. N-MYC can directly regulate the expression of proteins associated with the neuroendocrine phenotype (such as NSE and SYP), while ASCL1 could govern lineage plasticity [6]. Both can directly drive the neuroendocrine phenotype. Research attempts targeting these key transcription factors have also demonstrated potential therapeutic prospects [45]. Here, our work further demonstrates that c-Fos acts as a driver of NE differentiation in prostate cancer. Various FOX family proteins’ roles in NE differentiation have been well-documented: Sylvan et al. reported sustained FOXA1 expression in NEPC, essential for maintaining NEPC gene expression [46]. Han et al. showed that FOXA2 is upregulated by ADT and facilitates the transition from adenocarcinoma to NEPC, with specific activation in NEPC [47]. Additionally, c-Fos significantly upregulates FOXC1 expression, in line with Leonie et al.‘s findings of elevated FOXC1 in advanced prostate cancer [48], although FOXC1’s role in this context was previously unreported. FOXC1 is normally expressed in embryonic tissues under physiological conditions. Cao et al.‘s research in non-small cell lung cancer indicated that FOXC1 promotes the expression of stemness-related genes, such as Oct4, NANOG, and SOX2 [39]. Li et al. demonstrated that SOX2 suppresses adenocarcinoma-related genes and participates in the upregulation of NEPC genes [49], while Kwon et al. validated SOX2’s role in driving NE differentiation in a prostate cancer mouse model [50]. In prostate cancer, FOXC1 promotes SOX2 upregulation, leading to the increased expression of NE markers. This explains how metabolic enzyme inhibition facilitates the NE differentiation phenotype through the c-Fos-FOXC1-SOX2 axis.

This research carries significant clinical implications. Current prostate cancer treatments primarily focus on AR-targeted endocrine therapy, which is associated with considerable adverse effects. A clinical study by Parker et al. reported that 14% of patients receiving short-course ADT and 19% of those on long-course therapy experienced grade 3 or higher toxicities [51]. Furthermore, the lack of precise targeting allows tumors to gradually adapt to AR loss through extensive rewiring of intracellular signaling networks, leading to the development of CRPC and even NEPC, ultimately worsening patient prognosis [52, 53]. In this context, our research identifies specific metabolic targets within prostate cancer energy metabolism. Inhibition of either IDH1 or GLUD1 effectively suppresses tumor growth across different prostate cancer subtypes. Thus, selecting the appropriate metabolic enzyme inhibitor based on the patient’s stage in ADT could potentially augment existing treatments and benefit patients. Notably, IDH1 and GLUD1 share significant functional similarities, as both enzymes generate NADPH. This contrasts with the NADH production required for oxidative phosphorylation, highlighting a high demand for reductive equivalents in prostate cancer cells. This study also explored ferroptosis, a cell death mechanism that exploits intracellular redox imbalances to kill tumor cells [54], demonstrating that it can be effectively combined with metabolic enzyme inhibition to suppress prostate cancer. Ferroptosis inducers have recently shown promising results in various cancers. Ghoochani’s research confirmed that ferroptosis inducers can effectively kill prostate tumor cells in both in vitro and in vivo settings [55]. However, applying ferroptosis in prostate cancer faces challenges, primarily due to the lack of reliable biomarkers for ferroptosis sensitivity [56], making personalized treatment difficult in the context of the disease’s high heterogeneity. By targeting molecular features of distinct prostate cancer subtypes and combining metabolic enzyme inhibition with ferroptosis inducers, the lethal concentration of ferroptosis induction can be lowered. This approach enhances tumor targeting, increasing the specificity of ferroptosis induction for prostate cancer while minimizing off-target toxicity, offering a promising therapeutic direction.

However, this study leaves several important questions unresolved. α-KG levels significantly decrease during the initial phase of IDH1 inhibition, but following compensatory GLUD1 activation, α-KG levels are substantially replenished. Whether this restored α-KG level persistently stimulates the c-Fos pathway and its downstream effects requires further validation. Our results from long-term enzalutamide-treated C4-2 cells show that c-Fos remains elevated over time, suggesting that the non-metabolic functions of α-KG in NEPC warrant additional investigation.

In conclusion, this study identified a metabolic adaptation process related to α-KG metabolism during prostate cancer progression, driven by a HIF-1α-c-Fos-centered mechanism that promotes NE differentiation. This discovery provides a novel therapeutic rationale for drug treatment in prostate cancer, potentially inhibiting NE progression to extend patient survival.

## Materials and methods

### Cell culture and reagents

RPMI 1640, ham’s F-12K and MEM medium were purchased from Eallbio Life Science. MG132 (#S2619), Enzalutamide (#S1250), IOX2 (#S2919), T-5224 (S#8966), AG-120 (#S8206), R162 (#E1170), RSL3 (#S8155), Fer-1 (#S7243) were purchased from Selleck Chemicals. The human prostate cancer cell lines LNCaP (#06.0155) were purchased from Eallbio Life Sciences, and 22RV1 (#CL-0004), C4-2 (#CL0046) PC-3 (#CL-0185) and DU145 (#CL-0075) were purchased from the Procell System, where they were recently authenticated by short tandem repeat (STR) profiling and characterized by mycoplasma detection. These cells were cultured at 37 °C with 5% CO_2_ in RPMI1640 (LNCaP, 22RV1 and C4-2), ham’s F-12K and MEM (DU145) medium with 10% FBS (Procell, #164210), 100 U/mL penicillin, and 100 mg/mL streptomycin. To avoid artefacts of long-term culture of immortalized cell lines, cell cultures were started up from an early passage vial and interrupted after 2–3 months (Excluding C4-2 cells cultured long-term with enzalutamide).

### Measurement of GSH, ROS, LPO and Ferrous ions

GSH level was quantified using a commercial GSH assay kit (Solarbio Life Science, BC1175). Briefly, cells (5 × 10⁶) were collected and lysed according to the manufacturer’s instructions. The lysate was then incubated sequentially with the provided reagents. Absorbance was measured at a wavelength of 412 nm using a microplate reader, and GSH content was calculated based on the characteristic absorption peak.

To evaluate ROS levels, cells were seeded into 96-well plates at a density of 5000 cells per well and incubated with dihydroethidium (DHE). After incubation at 37 °C for 20 min, fluorescence intensity was measured with a microplate reader at an excitation wavelength of 500 nm and an emission wavelength of 525 nm. ROS levels were quantified relative to the fluorescence signal.

Cells were treated with 5 μM RSL3 for 8 h, after which 5 × 10⁶ cells were harvested for LPO analysis using a lipid peroxidation assay kit (Solarbio Life Science, BC5245). The absorbance was recorded between 535 nm and 600 nm with a microplate reader, and LPO levels were determined based on the absorbance peak values.

Detection of intracellular ferrous ions was performed using the FerroOrange fluorescent probe (Servicebio, G1727), according to the manufacturer’s instructions. Briefly, the probe was added to the culture medium of live cells and incubated at 37 °C in the dark for 30 min. After incubation, the cells were washed with PBS to remove the residual probe. For qualitative analysis, cells cultured on confocal dishes were imaged using a fluorescence confocal microscope to capture representative fluorescent images. For quantitative analysis, the fluorescence intensity of cells in 96-well plates was measured using a microplate reader.

### Vector design and lentivirus production

For shRNAs, the lentiviral vector pLKO.1-puro was kindly provided by Miaoling Plasmid. Plasmid synthesis was performed by MiaoLing Plasmid. The shRNA and overexpression sequences were validated before use. Transfection efficiency was subsequently confirmed by RT-qPCR and immunoblots. Information on the knockdown or overexpression plasmids were as follows: shIDH1#1(P50426), shIDH1#2(P50427), IDH1 OE(P59964), shGLUD1#1(P69022), shGLUD1#2(P68591), GLUD1 OE(P67013), shc-Fos#1(P83957), shc-Fos#2(P83956), c-Fos OE(P46585), shIDH2#1(P50428), shIDH2#2(P50425), shIDH3A#1(P79166), and shIDH3#2(P79167).

To produce lentiviruses, HEK293T cells were cotransfected with shRNA or expression plasmid along with the packaging plasmids pSPAX2 and pMD2.G using JetPRIME (Starorius, #101000001). The viral supernatant was harvested 48- and 72-h post-transfection and the viral particles were filtered and concentrated by centrifugation.

### RNA extraction and RT-qPCR

Total RNA was isolated from cells and tissues using FastPure Cell/Tissue Total RNA Isolation Kit V2 (Vazyme, RC112-01) according to the manufacturer’s instructions. Complementary DNA was generated from 1 μg of total RNA using HiScript II Reverse Transcriptase Mix reverse transcriptase according to the manufacturer’s instructions (Vazyme, R201-01). Real-time PCR was conducted using SYBR Mix in quantitative PCR instrument (Bio-Rad, 1854095-IVD). Each sample was tested in triplicate. Primers used were as follows: IDH1-Forward(CTTTTGGGTTCCGTCACTTG), IDH1-Reverse (GTCGTCATGCTTATGGGGAT), IDH2-Forward (TGAACTGCCAGATAATACGGG), IDH2-Reverse (CTGACAGCCCCCACCTC), IDH3A-Forward (TCGGTGTGACACCAAGTGGCAA), IDH3A-Reverse (TTCGCCATGTCCTTGCCTGCAA

), GLUD1-Forward (ATCCTGCGGATCATCAAGCC), GLUD1-Reverse (AACGGATACCTCCCTTGCAG), NSE-Forward (TCAGGAGAGACTGAGGACAC), NSE-Reverse (AGACAGCAAGAGGTCAGGTA), Chga-Forward (CACAGCAGCTTTGAGGATGA), Chga-Reverse (ATGGGGGACTCTTGGTTAGG), SYP-Forward (AGACATGGACGTGGTGAATCA), SYP-Reverse (ACTCTCCGTCTTGTTGGCAC), HIF-1α-Forward (ACAAGTCACCACAGGACAG), HIF-1α-Reverse (AGGGAGAAAATCAAGTCG), c-Fos-Forward (CACTCCAAGCGGAGACAGAC), c-Fos-Reverse (AGGTCATCAGGGATCTTGCAG), FOXC1-Forward (AAAAAATTGGAGGCTGCTT), FOXC1-Reverse (CCAAAGAAAAATCCCCACA), SOX2-Forward (ATGGACAGTTACGCGCACA), and SOX2-Reverse (TGCGAGTAGGACATGCTGTA).

### Western blotting

Cells were lysed in RIPA buffer (Solarbio Life Science, R0020) with PMSF (Solarbio Life Science, P0100). The protein concentration was determined using a BCA assay kit. Equal amounts of protein (20–30 µg per lane) were separated by SDS-polyacrylamide gel electrophoresis (SDS-PAGE) and subsequently transferred to polyvinylidene difluoride (PVDF) membranes. The membranes were blocked with 5% non-fat milk in Tris-buffered saline containing 0.1% Tween-20 (TBST) for 1 h at room temperature. Following blocking, the membranes were incubated with primary antibodies diluted according to the manufacturer’s recommendations in the blocking buffer at 4 °C overnight. After washing with TBST, the membranes were incubated with appropriate horseradish peroxidase (HRP)-conjugated secondary antibodies for 1 h at room temperature. Protein bands were visualized using an enhanced chemiluminescence (ECL) detection system. The detailed information regarding the primary antibodies, including their names, catalog numbers, and vendors were as follows: anti-IDH1 (ab172964, Abcam), anti-IDH2 (ab131263, Abcam), anti-IDH3A (15909-1-AP, Proteintech), anti-GLUD1 (61026-1-Ig, Proteintech), anti-HIF-1α (ab179483, Abcam), anti-Hydroxy-HIF-1α (ab308637, Abcam), anti-c-Fos (340249,Zenbio), anti-FOXC1 (160347, Zenbio), anti-SXO2 (R0344, Zenbio), anti-Tublin (14555-1-AP, Proteintech), anti-NSE (ab180943, Abcam), anti-Chga (ab45179, Abcam), anti-SYP (ab32127, Abcam), anti-ACSL4 (R24265, Zenbio), anti-LPCAT3 (67882-1-Ig, Proteintech), anti-GPX4 (R371958, Zenbio), and anti-SLC7A11 (R26116, Zenbio).

### Dual-luciferase reporter assay

To assess the transcriptional activity of the GLUD1 promoter, a dual-luciferase reporter assay was performed using the pmirGLO dual-reporter vector. This vector contains both the firefly luciferase (Fluc) reporter gene and the Renillaluciferase (Rluc) gene as an internal control, situated on a single plasmid to minimize experimental variability caused by differences in transfection efficiency. A DNA fragment encompassing the human GLUD1 promoter sequence was cloned directly upstream of the Fluc gene in this vector.

Cells, transfected with the constructed pmirGLO-GLUD1-promoter plasmid, were seeded in 96-well plates. After 48 h of transfection, dual-luciferase reporter assay kit (Beyotime, RG089S) was used to detect FLuc and RLuc luciferase activities sequentially. The relative luciferase activity for each sample was calculated by normalizing the Fluc/Rluc.

### Cell viability assay

Cell viability was determined by Cell Counting Kit-8 (Eallbio Life Science, 03.17002DA) according to the manufacturer’s instructions. Briefly, 3000–5000 cells were seeded into each well of96-well plates. After 24, 48, and 72 h of incubation, 10 mL CCK-8 was added to the well and then incubated for an additional 2 h. The absorption of each well was measured at 450 nm. LNCaP, C4-2 or PC-3 cells were seeded into each well of 96-well plates. An appropriate amount of the drugs was added to the wells, and the cells were cultured for the indicated durations. The absorbance/optical density (OD) was then measured using a microplate reader. The drug cytotoxicity curve was generated using Prism 10 software.

### Animal experiments

2.5 × 10^7^ C4-2 or PC-3 cells were injected subcutaneously in intact 5-week-old female BALB/c-Nude mice (GemPharmatech). Mice were randomized into treatment groups and administered daily intraperitoneal injections of RSL3 or R162. Both compounds were solubilized in a vehicle of DMSO, PEG300, and Tween-80 in accordance with manufacturer instructions. Control group mice were administered an equal volume of the solvent. Following tumor inoculation, all mice were assigned individual identification numbers. An independent researcher, who was not involved in the subsequent procedures, then generated a randomization sequence using statistical software to allocate the animals into different treatment groups. Blinding was implemented throughout the experimental period: the personnel responsible for the daily interventions and data collection were kept unaware of the group assignments, as well as the specific composition of the injected agents. No mice were excluded from the study due to disease, mortality, or any other reasons during the experiment.

### Metabolite extraction and mass spectrometry

Materials and Reagents The standard substances used in the work were mainly purchased from Sigma-Aldrich, Aladdin Biochemical Technology, and Yuanye Bio-Technology. Liquid chromatography-mass spectrometry (LC-MS) grade acetonitrile, methanol, formic acid, amm.

Onium acetate, and ammonium hydroxide (CNW brand) were purchased from ANPEL Laboratory Technologies. Metabolic flux analysis were conducted by ProfLeader Biotech (Shanghai, China) using the following protocol. Cell pellets were extracted in 500 μL of ice-cold acetonitrile/methanol/water (4:4:2, v/v/v) containing internal standards by ultrasonication in an ice-water bath for 5 min. The extracts were centrifuged at 16,000 × *g* and 4°C for 15 min. The supernatant was evaporated to dryness under a gentle nitrogen and reconstituted in 50 μL of 50% acetonitrile. Sample analysis was performed on a Ultimate 3000 UHPLC system coupled to a Q Exactive Hybrid Quadrupole-Orbitrap Mass Spectrometer (Thermo Fisher Scientific). The redissolved extracts (2 μl) were injected into an Acquity UPLC BEH Amide column (100 mm × 2.1 mm, 1.7 μm, Waters Corporation) at a flow rate of 0.30 mL/min with mobile phase (A) 90% acetonitrile, and (B) water, both with 15 mM ammonium acetate and 0.1% ammonium hydroxide. A linear gradient elution was started from 0% B and held for 0.5 min, increased to 22% B at 8 min, to 45% B at 9.5 min and held to 10.5 min, finally returned to 0% B at 10.6 min and equilibrated to 13 min. The eluted metabolites were ionized in negative mode of Heated Electrospray Ionization. Spray voltage was set to 4000 V. Capillary and Probe Heater Temperature were 320 °C. Sheath gas flow rate was 35 (Arb, arbitrary unit), and Aux gas flow rate was 10 (Arb). S-Lens RF Level was 50 (Arb). The full scan was operated at a high-resolution of 70000 FWHM (m/z = 200) at a range of 50–1000 m/z with AGC Target setting at 3e6. Simultaneously, the fragment ions information of top 8 precursors each scan was acquired by Data-dependant acquisition (DDA) with HCD energy at 15, 30, and 45 eV, mass resolution of 17,500 FWHM, and AGC Target of 1e5. Mass isotopomer distributions (MID) were corrected for natural isotope abundance using theoretical calculations on peak areas, and expressed as a percentage of the total pool.

### IHC and IF

Immunohistochemical analysis was performed on formalin-fixed, paraffin-embedded tumor tissues. Following deparaffinization and rehydration, antigen retrieval was conducted using citrate buffer (pH 6.0) under heat-induced conditions. Endogenous peroxidase activity was quenched with 3% H₂O₂, and nonspecific binding sites were blocked with 10% normal goat serum. Sections were incubated overnight at 4 °C with primary antibodies against target proteins, followed by incubation with horseradish peroxidase (HRP)-conjugated secondary antibodies at 37 °C for 30 min. Signal visualization was achieved using 3,3′-diaminobenzidine (DAB) substrate, and nuclei were counterstained with hematoxylin. Stained sections were evaluated by two independent pathologists using a semi-quantitative scoring system based on staining intensity and the percentage of positive cells.

Multiplex immunofluorescence staining was performed on paraffin-embedded tissue sections using a standardized protocol. Following incubation with primary antibodies at 4 °C overnight, sections were incubated with fluorophore-conjugated secondary antibodies at 37 °C for 1 h. Nuclei were counterstained, and slides were mounted with an anti-fade mounting medium containing DAPI. Fluorescence images were acquired immediately after a 10-minute mounting period.

### Chromatin immunoprecipitation followed by quantitative PCR (ChIP-qPCR)

Chromatin immunoprecipitation (ChIP) was performed to analyze the enrichment of c-Fos at promoter regions of GLUD1 and FOXC1 in PC-3 cells. Approximately 4 × 10^7^ cells were crosslinked with 1% formaldehyde for 10 min at room temperature to fix protein-DNA interactions. The cross-linking was quenched by adding glycine. Cells were washed with cold phosphate-buffered saline (PBS). Following cells was isolated from the samples using ChIP kit (Beyotime, P2083S). Chromatin was fragmented by sequential digestion with MNase. A 1% aliquot of each sample was set aside as the Input control. The remaining chromatin was subjected to immunoprecipitation by incubation with a primary antibody (with IgG used as the control) at 4 °C overnight. Protein A/G magnetic beads were then added to capture the antibody-chromatin complexes. After a series of washes, the protein-DNA complexes were eluted from the beads, and proteins were digested with Proteinase K. The purified DNA was analyzed by qPCR with each sample run in triplicate. The enrichment of the target DNA was calculated as a percentage of the Input DNA.

### Statistical analysis

The sample size for both in vitro and animal experiments was determined based on a review of previous studies that reported outcomes and observed differences following the application of the relevant reagents or drugs. This preliminary assessment was subsequently validated through our own pilot experiments, leading to the establishment of an optimal number of samples for the formal study.

Data presented are derived from a minimum of three independent experiments. Normality was tested using the Shapiro-Wilk test, and homogeneity of variances was assessed with the *F*-test. For statistical evaluation, differences between two groups were assessed with the two-tailed unpaired Student’s *t* test or the non-parametric Mann–Whitney *U* test (Based on the results of normality and homogeneity of variance tests), and one-way ANOVA followed by Tukey’s post-hoc tests for multiple comparisons was applied for multi-group comparisons. Results are expressed as mean ± SD. In all graphical representations, bar heights correspond to means, and error bars indicate the SD, in the absence of specific notation. Statistical significance was defined as *p* < 0.05. GraphPad Prism (v10.1.2) was utilized for all statistical analyses.

## Supplementary information

Figure S1

Original Data

Supplementary Figure legends

## Data Availability

The original contributions presented in the study are included in the article/supplementary material. Further inquiries can be directed to the corresponding author.
