# Supplementary figures and images for: c-Fos-driven metabolic switch of α-ketoglutarate orchestrates progression in prostate cancer
Source: Cell Death Dis. 2026 May 31;17(1):574. doi: 10.1038/s41419-026-08918-4 (PMC13273188; doi:10.1038/s41419-026-08918-4)

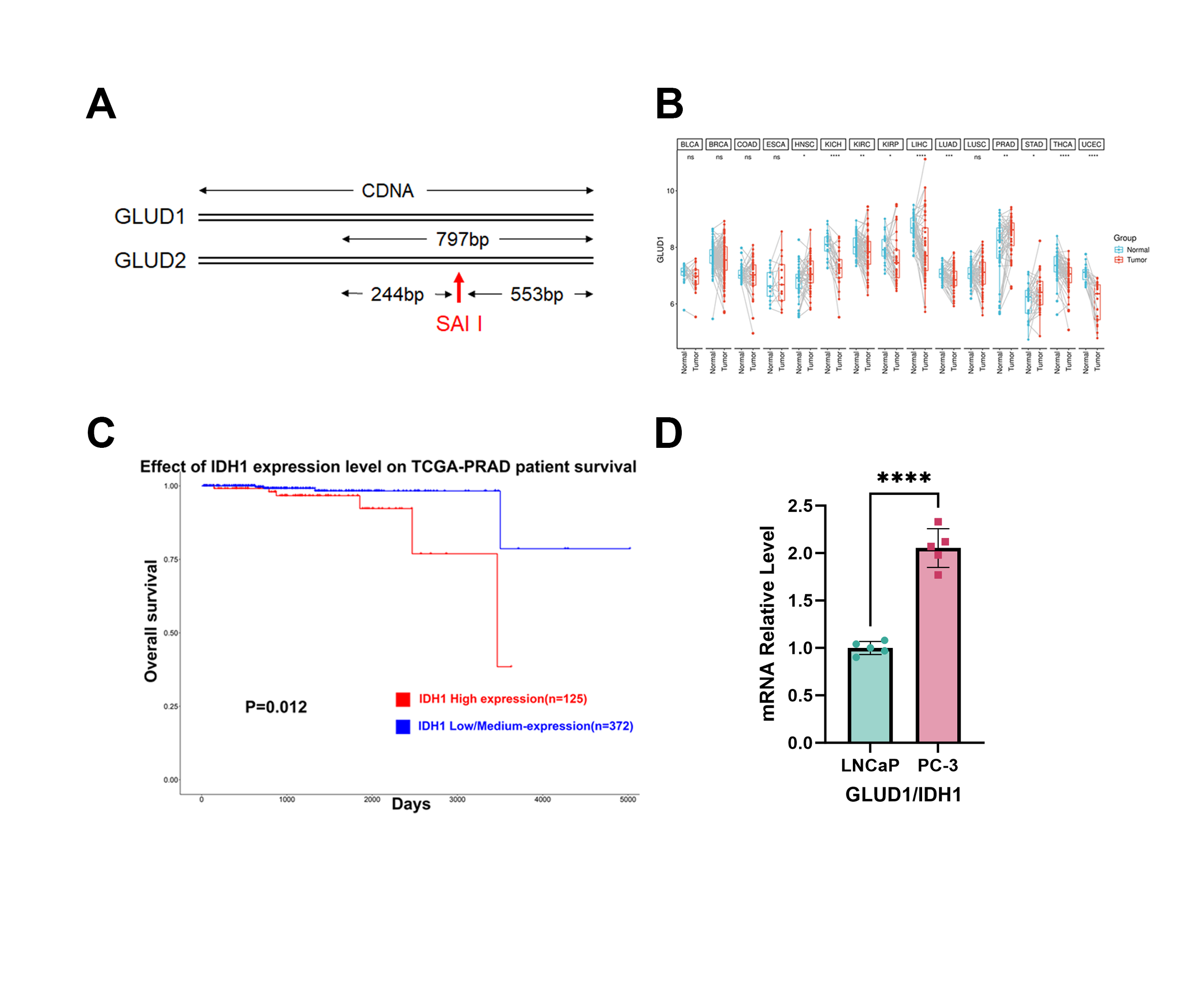

Supplement: Supplementary file 1 — Figure S1 [file 41419_2026_8918_MOESM1_ESM.tif]
