# Supplementary material for: c-Fos-driven metabolic switch of α-ketoglutarate orchestrates progression in prostate cancer
Source: Cell Death Dis. 2026 May 31;17(1):574. doi: 10.1038/s41419-026-08918-4 (PMC13273188; doi:10.1038/s41419-026-08918-4)
Supplement: Supplementary file 2 — Original Data [file 41419_2026_8918_MOESM2_ESM.pdf]

Contents

Original Data. • 1
Original Figure. • 62
Original Data of FIG.1C,D, FIG.5A and FIG.S1B. • 226

FIG.1A		RWPE-1			LNCaP			22RV1	
	1	2	3	1	2	3	1	2	3
CT-IDH1	21.13093	20.60484	21.05785	19.99364	19.76335	19.58043	17.33545	17.50195	17.41621
CT-IDH2	18.2682	18.12448	18.32651	20.76448	20.46199	20.23176	17.89161	18.07361	17.97912
CT-IDH3A	19.63928	20.10133	20.03793	23.74138	23.35304	23.35722	19.16729	19.27732	19.10587
CT-PPIA	13.37546	13.73927	14.05458	16.09739	16.15181	15.30526	14.24719	14.58804	14.41422
ΔCT-IDH1	-7.407827	-6.881735	-7.334752	-4.142143	-3.911852	-3.728932	-2.918946	-3.085447	-2.999711
ΔCT-IDH2	-4.545104	-4.401382	-4.603409	-4.912977	-4.610493	-4.380264	-3.475115	-3.657111	-3.56262
ΔCT-IDH3A	-5.916179	-6.378234	-6.314828	-7.889879	-7.501536	-7.505716	-4.750793	-4.860822	-4.689374
Δ ΔCT-IDH1 (Δ ΔCT:Norma	3.480227	2.954135	3.407152	0.214543	-0.015748	-0.198668	-1.008654	-0.842153	-0.927889
Δ ΔCT-IDH2	0.617504	0.473782	0.675809	0.985377	0.682893	0.452664	-0.452485	-0.270489	-0.36498
Δ ΔCT-IDH3A	1.988579	2.450634	2.387228	3.962279	3.573936	3.578116	0.823193	0.933222	0.761774
2^(-Δ ΔCT)-IDH1	0.089608	0.129038	0.094264	0.861819	1.010975	1.147638	2.012032	1.792724	1.90249
2^(-Δ ΔCT)-IDH2	0.651798	0.720075	0.625981	0.505094	0.622915	0.730692	1.368395	1.206216	1.287864
2^(-Δ ΔCT)-IDH3A	0.251987	0.18293	0.191149	0.064156	0.083973	0.08373	0.56519	0.523687	0.589771
		C4-2			PC-3			DU145	
	1	2	3	1	2	3	1	2	3
CT-IDH1	17.78502	18.41213	17.8173	20.66248	20.66512	20.64356	18.4879	19.00199	18.86017
CT-IDH2	18.4876	19.24402	18.56025	21.31026	21.30427	21.278	19.04788	19.66156	19.50395
CT-IDH3A	20.60118	21.39891	20.28811	23.27799	24.0297	23.86774	20.44454	21.19772	20.95716
CT-PPIA	14.47406	14.43896	14.15812	14.27361	14.24115	14.27865	14.04499	14.13201	14.22925
ΔCT-IDH1	-3.428021	-4.055132	-3.460304	-6.397977	-6.400618	-6.379062	-4.352496	-4.866595	-4.724767
ΔCT-IDH2	-4.130599	-4.887017	-4.203248	-7.045765	-7.039766	-7.013504	-4.912477	-5.526158	-5.368551
ΔCT-IDH3A	-6.244179	-7.04191	-5.931115	-9.013492	-9.765196	-9.603238	-6.309143	-7.062323	-6.821762
Δ ΔCT-IDH1 (Δ ΔCT:Norma	-0.499579	0.127532	-0.467296	2.470377	2.473018	2.451462	0.424896	0.938995	0.797167
Δ ΔCT-IDH2	0.202999	0.959417	0.275648	3.118165	3.112166	3.085904	0.984877	1.598558	1.440951
Δ ΔCT-IDH3A	2.316579	3.11431	2.003515	5.085892	5.837596	5.675638	2.381543	3.134723	2.894162
2^(-Δ ΔCT)-IDH1	1.413801	0.915396	1.382515	0.180444	0.180114	0.182825	0.744893	0.521596	0.575478
2^(-Δ ΔCT)-IDH2	0.868743	0.514265	0.826079	0.11517	0.11565	0.117774	0.505269	0.330207	0.368324
2^(-Δ ΔCT)-IDH3A	0.200743	0.115478	0.249392	0.029444	0.017487	0.019564	0.191904	0.113856	0.134515

Fig.1 G			HSPC					CRPC		
Patient	1	2	3	4	5	1	2	3	4	5
IHC Score-IDH1	6	4	9	6	4	2	0	2	2	3
IHC Score-GLUD1	2	0	3	2	2	6	9	4	6	3

Fig.2 A	NCaP-Vehicle					shIDH1#1					shIDH1#2					IDH1 OE				
	Absorbance at 450 nm of the blank control (culture medium + CCK-8 reagent): 0.095																			
OD450 (Ab	1	2	3	4	5	1	2	3	4	5	1	2	3	4	5	1	2	3	4	5
Control(Da	0.415					0.576					0.619					0.448				
Day1	0.634	0.593	0.65	0.557	0.642	0.766	0.88	0.866	0.861	0.883	0.777	0.802	0.835	0.861	0.89	0.875	0.855	0.752	0.924	0.898
Day2	1.007	1.012	0.976	0.919	0.957	1.071	1.105	1.11	1.147	1.005	0.887	0.984	1.133	1.044	1.103	1.223	1.159	1.294	1.215	1.266
Day3	1.434	1.438	1.334	1.324	1.417	1.27	1.129	1.341	1.349	1.201	1.28	1.405	1.302	1.441	1.28	2.079	2.128	2.213	2.044	2.069

Fig.2 B	C4-2-Vehicle					shIDH1#1					shIDH1#2					IDH1 OE				
	Absorbance at 450 nm of the blank control (culture medium + CCK-8 reagent): 0.096																			
OD450 (Ab	1	2	3	4	5	1	2	3	4	5	1	2	3	4	5	1	2	3	4	5
Control (D	0.472					0.391					0.403					0.55				
Day1	0.567	0.635	0.601	0.635	0.628	0.473	0.462	0.428	0.449	0.452	0.442	0.464	0.485	0.483	0.457	0.977	0.971	0.942	1.033	0.995
Day2	0.835	0.93	0.932	0.923	0.973	0.525	0.682	0.65	0.66	0.661	0.549	0.584	0.577	0.582	0.572	1.645	1.786	1.745	1.563	1.478
Day3	1.469	1.541	1.535	1.672	1.663	0.825	0.89	0.929	0.981	0.825	0.738	0.845	0.901	0.695	0.788	2.535	2.45	2.752	3.112	2.729

Fig.2 C	PC-3-Vehicle				shGLUD1#1					shGLUD1#2						GLUD1 OE				
	Absorbance at 450 nm of the blank control (culture medium + CCK-8 reagent): 0.090																			
OD450 (Ab	1	2	3	4	5	1	2	3	4	5	1	2	3	4	5	1	2	3	4	5
Control (D	0.373					0.416					0.39					0.285				
Day1	0.973	1.001	1.008	1.021	0.965	1.03	0.989	1.052	1.017	1.015	0.908	0.932	0.941	0.932	0.927	0.818	0.803	0.835	0.806	0.807
Day2	1.631	1.548	1.534	1.591	1.696	1.404	1.441	1.364	1.343	1.458	1.365	1.293	1.207	1.263	1.471	1.42	1.477	1.368	1.48	1.531
Day3	2.525	2.217	2.437	2.389	2.194	2.208	2.149	2.194	2.118	2.152	1.963	1.839	2.022	1.907	1.937	2.327	2.478	2.176	2.286	2.428

Fig.2D	C4-2-Vehicle					shIDH2#1					shIDH2#2				
	Absorbance at 450 nm of the blank control (culture medium + CCK-8 reagent): 0.100														
**OD450 (Ab**	**1**	**2**	**3**	**4**	**5**	**1**	**2**	**3**	**4**	**5**	**1**	**2**	**3**	**4**	**5**
**Control (D**	0.437					0.498					0.392				
**Day1**	0.592	0.588	0.619	0.589	0.587	0.635	0.693	0.715	0.511	0.77	0.564	0.556	0.559	0.601	0.564
**Day2**	1.051	1.117	0.974	1.211	1.213	1.314	1.351	1.571	1.234	1.501	1.026	1.117	0.977	1.109	0.971
**Day3**	1.799	1.84	1.894	1.818	1.776	1.874	2.04	2.014	2.044	1.963	1.455	1.544	1.513	1.535	1.489

Fig.2E	C4-2-Vehicle					shIDH3A#1					shIDH3A#2				
	Absorbance at 450 nm of the blank control (culture medium + CCK-8 reagent): 0.087														
OD450 (Ab	1	2	3	4	5	1	2	3	4	5	1	2	3	4	5
Control (D	0.274					0.321					0.334				
Day1	0.318	0.339	0.34	0.359	0.353	0.397	0.398	0.408	0.431	0.41	0.389	0.422	0.416	0.433	0.428
Day2	0.412	0.458	0.422	0.506	0.454	0.482	0.484	0.47	0.503	0.483	0.529	0.608	0.544	0.541	0.608
Day3	0.652	0.577	0.542	0.573	0.603	0.638	0.631	0.658	0.739	0.633	0.834	0.805	0.757	0.816	0.814

Fig.2 G				
Number of	LNCaP Vel	shIDH1#1	shIDH1#2	IDH1 OE
1	258	138	132	341
2	281	142	116	365
3	233	146	133	349
4	250	190	151	279
Migration				

Fig.2 H				
Number of	LNCaP Vel	shIDH1#1	shIDH1#2	IDH1 OE
1	209	54	63	311
2	186	69	69	335
3	167	71	94	263
4	168	48	84	281
Invasion				

Fig.2 I				
Number of	C4-2 Vehic	shIDH1#1	shIDH1#2	IDH1 OE
1	219	124	156	344
2	209	131	134	351
3	141	109	109	321
4	238	71	162	304
Migration				

Fig.2 J				
Number of	C4-2 Vehic	shIDH1#1	shIDH1#2	IDH1 OE
1	90	53	46	144
2	105	35	10	159
3	118	16	40	193
4	83	64	62	134
Invasion				

Fig.2 L				
**Number of**	**PC-3 Vehic**	**shGLUD1#**	**shGLUD1#**	**GLUD1 OE**
**1**	62	23	18	190
**2**	72	25	11	205
**3**	55	19	29	186
**4**	64	12	27	189
**Migration**				

Fig.2 M				
**Number of**	**PC-3 Vehic**	**shGLUD1#**	**shGLUD1#**	**GLUD1 OE**
**1**	29	9	10	94
**2**	33	12	4	124
**3**	31	11	16	76
**4**	41	5	11	79
**Invasion**				

Fig.2 P					
Green Fluc	C4-2 Vehic	shIDH1	shIDH1+α-KG		Control Green Fluorescence
1	1.7	2.3	2.8		0.7
2	1.9	2.1	2.8		
3	2.2	2.2	2.8		
4	1.8	2.3	2.7		
5	1.9	2.2	1.9		
Red Fluorescence					Control Red Fluorescence
1	5.6	6.1	10.8		0.4
2	6.4	5.9	12.9		
3	7.1	5.4	10.8		
4	6.7	5.4	12.3		
5	6.2	6	5.5		
Relative MMP					
1	5.2	3.5625	4.952381		
2	5	3.928571	5.952381		
3	4.466667	3.333333	4.952381		
4	5.818182	3.125	5.95		
5	4.833333	3.733333	4.25		

Fig.2 Q					
Green Fluo	PC-3 Vehic	shGLUD1	shGLUD1+α-KG		Control Green Fluorescence
1	2.7	4	3.8		0.6
2	4.7	3.9	3.1		
3	3.3	2	4.7		
4	2.1	3.1	4.5		
5	1.7	5.1	2.5		
6	2.3	2.8	3.3		
Red Fluorescence					Control Red Fluorescence
1	6.8	10.6	13.5		0.4
2	17.3	9.5	12.3		
3	12.7	4.8	13.1		
4	6	8.3	14.3		
5	5.6	11.6	10.9		
6	6.8	7.4	12.7		
Relative MMP					
1	3.047619	3	4.09375		
2	4.121951	2.757576	4.76		
3	4.555556	3.142857	3.097561		
4	3.733333	3.16	3.564103		
5	4.727273	2.488889	5.526316		
6	3.764706	3.181818	4.555556		

Fig.2 S		
**Tumor We**	**C4-2 Vehic**	**shIDH1**
**1**	1673	602
**2**	972	477
**3**	701	203
**4**	532	102
**5**	519	83

Fig.2 T		
**Tumor Vol**	**C4-2 Vehic**	**shIDH1**
**1**	1.985736	1.0048
**2**	1.2246	0.79128
**3**	0.8792	0.20096
**4**	0.732667	0.1099
**5**	0.884433	0.065417

Fig.3 A			
**Relative G**	**NCaP Vehic**	**shIDH1#1**	**shIDH1#2**
**1**	0.68	0.32	0.31
**2**	0.85	0.25	0.26
**3**	0.78	0.26	0.28
**4**	0.61	0.25	0.26
**5**	0.61	0.26	0.32

Fig.3 B		
**Relative R(**	**LNCaP Ve**	**shIDH1**
**1**	1.14	2.63
**2**	1.03	2.4
**3**	1.35	2.23
**4**	1.18	2.43
**5**	1.27	2.15

Fig.3 C		
**Relative LI**	**LNCaP Vel**	**shIDH1**
**1**	0.81	1.52
**2**	0.92	1.67
**3**	0.98	1.88
**4**	1.07	1.65
**5**	1.1	1.56

Fig.3 E	LNCaP Vehicle			shIDH1#1			shIDH1#2			IDH1 OE		
Concentrati	450 nm of the blank control (culture medium + CCK-8 r											
Control	0.653			0.771			0.683			0.815		
0.0375	0.592	0.591	0.563	0.507	0.548	0.466	0.443	0.458	0.497	0.801	0.782	0.803
0.075	0.439	0.428	0.416	0.444	0.408	0.429	0.408	0.401	0.342	0.598	0.626	0.684
0.15	0.382	0.414	0.315	0.354	0.344	0.327	0.331	0.35	0.311	0.557	0.6	0.539
0.3	0.353	0.314	0.288	0.26	0.263	0.261	0.318	0.271	0.281	0.565	0.546	0.514
0.6	0.267	0.292	0.307	0.199	0.195	0.234	0.222	0.16	0.167	0.418	0.453	0.51
	Vehicle+Fer-1			shIDH1#1+Fer-1			shIDH1#2+Fer-1			IDH1 OE+Fer-1		
Concentration(μM)												
Control	0.509			0.696			0.791			0.903		
0.0375	0.479	0.494	0.499	0.733	0.613	0.67	0.747	0.69	0.865	0.963	0.93	0.879
0.075	0.469	0.476	0.49	0.631	0.646	0.615	0.755	0.743	0.7	0.984	0.879	0.916
0.15	0.504	0.479	0.522	0.658	0.67	0.717	0.716	0.828	0.824	0.903	0.906	0.926
0.3	0.509	0.505	0.528	0.666	0.645	0.705	0.698	0.749	0.692	0.933	0.919	0.906
0.6	0.507	0.489	0.472	0.652	0.592	0.658	0.68	0.688	0.828	0.916	0.977	0.767

Fig.3 F	C4-2 Vehicle			shIDH1#1			shIDH1#2			IDH1 OE		
Concentrati	450 nm of the blank control (culture medium + CCK-8 r											
Control	0.504			0.677			0.517			0.493		
0.5	0.449	0.461	0.443	0.416	0.381	0.369	0.333	0.315	0.345	0.487	0.511	0.48
1	0.408	0.418	0.402	0.34	0.376	0.299	0.292	0.302	0.333	0.481	0.477	0.455
2	0.282	0.351	0.324	0.23	0.189	0.223	0.195	0.222	0.206	0.456	0.462	0.436
4	0.251	0.279	0.222	0.153	0.118	0.141	0.136	0.138	0.125	0.318	0.333	0.307
8	0.135	0.139	0.125	0.135	0.123	0.177	0.122	0.109	0.115	0.227	0.244	0.199
	Vehicle+Fer-1			shIDH1#1+Fer-1			shIDH1#2+Fer-1			IDH1 OE+Fer-1		
Concentration(μM)												
Control	0.514			0.685			0.59			0.519		
0.5	0.472	0.491	0.509	0.587	0.642	0.65	0.52	0.548	0.571	0.519	0.48	0.556
1	0.455	0.474	0.446	0.718	0.722	0.658	0.62	0.644	0.574	0.506	0.496	0.505
2	0.451	0.438	0.521	0.642	0.626	0.676	0.615	0.641	0.618	0.513	0.518	0.48
4	0.448	0.394	0.441	0.66	0.724	0.654	0.597	0.626	0.566	0.54	0.445	0.473
8	0.42	0.531	0.474	0.702	0.702	0.704	0.667	0.62	0.553	0.44	0.472	0.496

Fig.3 G	C4-2 Vehicle			shIDH2#1			shIDH2#2		
Concentrati	450 nm of the blank control (culture medium + CCK-8 r								
Control	0.81			0.712			0.654		
0.5	0.676	0.658	0.668	0.579	0.615	0.597	0.509	0.509	0.552
1	0.651	0.578	0.638	0.586	0.555	0.593	0.537	0.502	0.486
2	0.623	0.556	0.562	0.513	0.507	0.492	0.436	0.489	0.48
4	0.482	0.575	0.5	0.47	0.443	0.442	0.426	0.397	0.456
8	0.347	0.305	0.307	0.346	0.349	0.358	0.326	0.322	0.309
	Vehicle+Fer-1			shIDH2#1+Fer-1			shIDH2#2+Fer-1		
Concentration(μM)									
Control	0.876			0.673			0.681		
0.5	0.811	0.837	0.842	0.627	0.675	0.692	0.632	0.678	0.643
1	0.854	0.824	0.797	0.62	0.612	0.624	0.678	0.608	0.629
2	0.874	0.87	0.824	0.665	0.64	0.639	0.672	0.635	0.662
4	0.749	0.722	0.726	0.553	0.56	0.522	0.565	0.537	0.563
8	0.636	0.602	0.597	0.522	0.463	0.475	0.441	0.505	0.481

Fig.3 H		
Relative R	PC-3 Vehicl	shGLUD1
1	0.88	2.25
2	0.74	2.23
3	1.04	2.03
4	1.12	1.75
5	0.84	1.91

Fig.3 I		
**Relative LPC-3 Vehicl**	**shGLUD1**	
**1**	1.58	3.25
**2**	1.46	2.64
**3**	1.36	2.8
**4**	1.38	3.07
**5**	1.64	3.01

Fig.3 J	PC-3 Vehicle			shGLUD1#1			shGLUD1#2			GLUD1 OE		
Concentrati	450 nm of the blank control (culture medium + CCK-8											
Control	0.933			0.689			1.015			0.876		
1	0.457	0.441	0.464	0.25	0.227	0.26	0.341	0.318	0.246	0.701	0.701	0.689
2	0.284	0.265	0.327	0.189	0.169	0.153	0.191	0.176	0.201	0.455	0.466	0.475
3	0.126	0.141	0.138	0.132	0.142	0.146	0.166	0.177	0.167	0.366	0.338	0.313
4	0.13	0.144	0.152	0.144	0.142	0.143	0.164	0.162	0.16	0.343	0.282	0.277
5	0.118	0.146	0.138	0.127	0.127	0.117	0.154	0.142	0.126	0.261	0.212	0.19
	Vehicle+Fer-1			shGLUD1#1+Fer-1			shGLUD1#2+Fer-1			GLUD1 OE+Fer-1		
Concentration(μM)												
Control	0.911			0.724			0.91			1.03		
1	0.901	0.898	0.928	0.655	0.702	0.67	0.919	0.947	0.866	0.936	0.983	1.037
2	0.899	0.919	0.896	0.662	0.666	0.613	0.891	0.855	0.955	0.966	1.047	1
3	0.87	0.924	0.814	0.605	0.67	0.63	0.883	0.87	0.859	1.047	1	1.005
4	0.697	0.671	0.731	0.523	0.515	0.563	0.883	0.87	0.859	0.907	0.81	0.892
5	0.641	0.599	0.667	0.405	0.361	0.265	0.338	0.322	0.298	0.696	0.676	0.712

Fig.3 M				
**Tumor We**	**PC3-DMSC**	**R162**	**RSL3**	**R162+RSL3**
**1**	936	478	397	112
**2**	857	366	301	87
**3**	702	263	266	75
**4**	752	261	276	60
**5**	515	70	195	35

Fig.3 N				
Tumor Vol	PC-3 DMS(	R162	RSL3	R162+RSL3
1	1.172267	0.633233	0.59346	0.066987
2	0.90432	0.418667	0.334933	0.060288
3	0.70336	0.267947	0.20096	0.10048
4	0.7536	0.267947	0.256433	0.05652
5	0.7536	0.179503	0.366333	0.011723

Fig.3 Q				
**Relative Fl**	**C4-2 Vehic**	**shIDH1#1**	**shIDH1#2**	**IDH1 OE**
**1**	4.5	8.2	9.9	5.1
**2**	4.4	9.2	8.8	5
**3**	3.9	9.3	8.7	6.1
**4**	5	8.9	10.3	5.9
**5**	5.1	7.5	8.7	4.7

Fig.3 R				
**Relative Fl**	**PC-3 Vehic**	**shGLUD1#**	**shGLUD1#**	**GLUD1 OE**
**1**	6.5	13.9	12.7	5.9
**2**	6.3	14.1	11.9	6.1
**3**	6.9	14.6	12.1	6.3
**4**	7.1	13.5	12.6	6.6
**5**	6	15.9	11.5	4.8

Fig.4 B		
Relative M	C4-2 Vehic	shIDH1
1	1742010	2223954
2	1511138	1832552
3	2070029	3175831
4	2009406	2920758
5	1245357	2194188

Fig.4 C		
Relative M	C4-2 Vehic	shIDH1
1	8416821	21016350
2	12173911	22356458
3	9836788	20036570
4	10590078	24691911
5	9155428	22246383

Fig.4 D		
**Relative M**	**C4-2 Vehic**	**shIDH1**
**1**	132380.6	517341.7
**2**	116476.1	773851.5
**3**	176317.1	421785.1
**4**	115626.9	407590.8
**5**	90808.38	481012.5

Fig.4 E		
**Relative M**	**C4-2 Vehic**	**shIDH1**
**1**	208672.9	143045.7
**2**	164676.4	124089.2
**3**	218445.7	169983.1
**4**	212097.9	165005.2
**5**	243144.9	102263.8

Fig.4 F		
**Relative M**	**C4-2 DMSO**	**Enzalutamide**
**1**	426129.6	1277793
**2**	231033.8	1972369
**3**	675109.1	2124274
**4**	277573.1	2754204
**5**	404957.3	1998918

Fig.4 G		
**Relative M**	**C4-2 DMS(**	**Enzalutamide**
**1**	494760.3	967404.4
**2**	530762.7	940928.9
**3**	439760.9	936567.5
**4**	466963.4	1033289
**5**	514916.8	936777.4

Fig.4 H		
**Relative M**	**C4-2 DMS(**	**Enzalutamide**
**1**	1744358	2371149
**2**	1307918	3267337
**3**	1340273	1980906
**4**	1205177	2066632
**5**	1282685	2139049

Fig.4 I		
**Relative M**	**C4-2 DMS(**	**Enzalutamide**
**1**	32368747	20320369
**2**	23991859	21268312
**3**	36542216	27971908
**4**	30731323	25499559
**5**	29180830	9877808

Fig.4 K		
**IHC Score**	**IDH1 MUT**	**IDH1 WT**
**1**	4	0
**2**	6	1
**3**	4	3
**4**	3	6
**5**	6	6
**6**	9	2
**7**	9	2
**8**	3	2

Fig.4 L		C4-2 Vehicle			shIDH1#1			shIDH1#2	
	1	2	3	1	2	3	1	2	3
CT-GLUD1	19.92327	19.8199	19.86852	20.25742	20.37618	20.10661	22.00634	22.02398	22.08667
CT-PPIA	15.55662	15.3531	15.52144	16.54119	16.48805	16.5067	18.15023	18.20869	18.20628
ΔCT-GLUD	-4.446217	-4.342855	-4.391466	-3.745436	-3.864201	-3.594631	-3.800064	-3.817696	-3.880392
ΔΔCT-GLU	0.052707	-0.050655	-0.002044	-0.648074	-0.529309	-0.798879	-0.593446	-0.575814	-0.513118
2^(-Δ ΔCT)	0.964126	1.035735	1.001418	1.567075	1.443238	1.739749	1.508847	1.490519	1.427132

Fig.5 C		C4-2 Vehicle			shIDH1#1			shIDH1#2	
	**1**	**2**	**3**	**1**	**2**	**3**	**1**	**2**	**3**
**CT-c-Fos**	36.17711	35.73057	37.12202	36.38958	36.05021	36.22032	37.60557	37.23648	37.80177
**CT-PPIA**	15.55662	15.3531	15.52144	16.54119	16.48805	16.5067	18.15023	18.20869	18.20628
**ΔCT-c-Fos**	-20.70006	-20.25352	-21.64497	-19.8776	-19.53823	-19.70834	-19.39929	-19.0302	-19.59549
**ΔΔCT-c-Fo**	-0.169942	-0.616481	0.774972	-0.992401	-1.331768	-1.161659	-1.470706	-1.839801	-1.274512
**2^(-Δ ΔCT)**	1.125013	1.533131	0.5844	1.989493	2.51711	2.237145	2.771574	3.579606	2.41917

Fig.5 E	PC-3 Vehic	c-Fos OE	c-Fos OE+T-5224		
FLuc	1	35.8	9.4		Control Fluc:0.1
	10.7	25	17		
	8.7	47.4	23.9		
	4.5	46.6	28.8		
	14.6	28.8	25.2		
RLuc	3.1	1.4	2.3		Control Rluc:0.1
	2.5	2.4	4.5		
	5	1.5	4.9		
	2.8	3.8	8		
	2.2	3	3.4		

Fig.5 F	C4-2 Vehic	c-Fos OE	c-Fos OE+T-5224		
FLuc	30.2	390.8	52.9		Control Fluc:0.1
	25.8	131.6	105.6		
	48.9	120.5	97		
	71.7	85.2	24.4		
	6.5	86.4	26.2		
RLuc	2.5	13.2	9.3		Control Rluc:0.1
	4.6	6.5	8.5		
	6.7	8.9	13.9		
	10.3	7.2	5.5		
	3.1	7.3	3		

Fig.5G		C4-2 Vehicle			shIDH1#1			shIDH1#2	
	1	2	3	1	2	3	1	2	3
CT-Hif-1α	19.77294	19.97608	19.87895	20.80608	21.00503	21.00768	22.59903	22.31912	22.28726
CT-PPIA	15.55662	15.3531	15.52144	16.54119	16.48805	16.5067	18.15023	18.20869	18.20628
ΔCT-Hif-1α	-4.29589	-4.499033	-4.401898	-4.294096	-4.493051	-4.495696	-4.392752	-4.112835	-4.080983
ΔΔCT-Hif-1	-0.10305	0.100093	0.002958	-0.104844	0.094111	0.096756	-0.006188	-0.286105	-0.317957
2^(-Δ ΔCT)	1.074042	0.932973	0.997952	1.075378	0.936849	0.935133	1.004299	1.219344	1.246564

Fig.5 H		C4-2 DMSO			IOX2			IOX2+T-5224	
	1	2	3	1	2	3	1	2	3
CT-c-Fos	25.42367	25.42873	25.41004	25.14102	25.11849	25.32787	24.83374	24.86886	25.34279
CT-PPIA	15.35729	14.89704	15.10095	16.19561	16.04052	16.06195	15.94449	15.96673	15.98965
ΔCT-c-Fos	-10.30524	-10.3103	-10.29161	-9.041657	-9.019134	-9.228515	-8.866783	-8.901902	-9.375829
ΔΔCT-c-Fo	0.002838	0.007904	-0.01079	-1.260743	-1.283266	-1.073885	-1.435617	-1.400498	-0.926571
2^(-Δ ΔCT)	0.998035	0.994536	1.007507	2.396191	2.433893	2.105095	2.704978	2.639926	1.900753

Fig.5 I		C4-2 DMSO			IOX2			IOX2+T-5224	
	1	2	3	1	2	3	1	2	3
CT-GLUD1	18.58989	18.31222	18.35793	18.40344	18.24116	17.7781	18.57645	20.01639	19.1781
CT-PPIA	15.35729	14.89704	15.10095	16.19561	16.04052	16.06195	15.94449	15.96673	15.98965
ΔCT-GLUD	-3.471455	-3.193789	-3.239504	-2.304084	-2.141799	-1.678741	-2.609491	-4.049434	-3.211139
ΔΔCT-GLU	0.169875	-0.107791	-0.062076	-0.997496	-1.159781	-1.622839	-0.692089	0.747854	-0.090441
2^(-Δ ΔCT)	0.88892	1.077577	1.043967	1.996532	2.234235	3.079806	1.615622	0.595489	1.064696

Fig.5 J		C4-2 Vehicle			Enzalutamide	
	1	2	3	1	2	3
CT-Hif-1α	19.95012	20.19709	20.19766	21.95053	21.19602	21.18232
CT-PPIA	16.12163	16.08967	16.02379	17.59449	17.47319	17.3529
ΔCT-Hif-1α	-3.871755	-4.118715	-4.119288	-4.477004	-3.722491	-3.708785
ΔΔCT-Hif-1	-0.164835	0.082125	0.082698	0.440414	-0.314099	-0.327805
2^(-Δ ΔCT)	1.121038	0.944665	0.94429	0.736923	1.243235	1.255102

Fig.5 K		C4-2 Vehicle			Enzalutamide	
	1	2	3	1	2	3
CT-c-Fos	27.90137	28.01258	28.009	26.87221	27.21316	27.12065
CT-PPIA	16.12163	16.08967	16.02379	17.59449	17.47319	17.3529
ΔCT-c-Fos	-11.823	-11.93421	-11.93063	-9.398676	-9.739629	-9.64712
ΔΔCT-c-Fo	-0.072901	0.038305	0.034735	-2.497224	-2.156271	-2.24878
2^(-Δ ΔCT)	1.051829	0.973798	0.976211	5.64598	4.457612	4.752807

Fig.5 N			
**The input sample was diluted to 1%**			
**CT**	**DMSO**	**IOX2**	**IOX2+T-5224**
**Input1**	21.24959	22.29487	22.91609
**Input2**	21.26446	22.64136	22.72093
**Input3**	21.11029	22.35246	22.98454
**ChIP1**	23.41015	23.9248	25.36812
**ChIP2**	23.36317	23.88201	25.20029
**ChIP3**	23.08333	23.65176	25.09094
**IgG1**	0	0	0
**IgG2**	0	0	0
**IgG3**	0	0	0
**ΔCT**	**DMSO**	**IOX2**	**IOX2+T-5224**
**ChIP1**	-2.202038	-1.495227	-2.494269
**ChIP2**	-2.155057	-1.452438	-2.326441
**ChIP3**	-1.875215	-1.222185	-2.217095
**2^(-ΔCT)**	**DMSO**	**IOX2**	**IOX2+T-5224**
**ChIP1**	4.601288	2.819086	5.634426
**ChIP2**	4.453864	2.7367	5.015664
**ChIP3**	3.668564	2.332998	4.649562
**% Input**	**DMSO**	**IOX2**	**IOX2+T-5224**
**ChIP1**	0.21733	0.354725	0.17748
**ChIP2**	0.224524	0.365404	0.199375
**ChIP3**	0.272586	0.428633	0.215074

Fig.6 B		C4-2 Vehicle			shIDH1#1			shIDH1#2	
	1	2	3	1	2	3	1	2	3
CT-NSE	25.84892	25.89475	25.89613	25.55283	25.76154	25.63256	27.64748	27.86514	28.04481
CT-PPIA	15.55662	15.3531	15.52144	16.54119	16.48805	16.5067	18.15023	18.20869	18.20628
ΔCT-NSE	-10.37187	-10.4177	-10.41908	-9.040848	-9.249563	-9.120578	-9.459076	-9.676739	-9.856411
ΔΔCT-NSE	-0.031032	0.014795	0.016181	-1.362052	-1.153337	-1.282322	-0.943824	-0.726161	-0.546489
2^(-Δ ΔCT)	1.021743	0.989797	0.988847	2.570505	2.224278	2.432302	1.92362	1.654231	1.460527

Fig.6 C		C4-2 Vehicle			shIDH1#1			shIDH1#2	
	1	2	3	1	2	3	1	2	3
CT-Chga	37.6185	37.25163	37.96403	37.95313	38.02934	38.1134	39.24608	39.23879	39.0637
CT-PPIA	15.55662	15.3531	15.52144	16.54119	16.48805	16.5067	18.15023	18.20869	18.20628
ΔCT-Chga	-22.14145	-21.77458	-22.48698	-21.44115	-21.51736	-21.60142	-21.05768	-21.05039	-20.8753
ΔΔCT-Chg	0.007153	-0.359715	0.352683	-0.69315	-0.616944	-0.532877	-1.076617	-1.083909	-1.258997
2^(-Δ ΔCT)	0.995054	1.283173	0.783127	1.616809	1.533624	1.446811	2.109084	2.119772	2.393294

Fig.6 D		C4-2 Vehicle			shIDH1#1			shIDH1#2	
	1	2	3	1	2	3	1	2	3
CT-SYP	29.76046	30.0395	29.27938	29.42193	29.61251	29.63309	31.12855	31.49567	31.39619
CT-PPIA	15.55662	15.3531	15.52144	16.54119	16.48805	16.5067	18.15023	18.20869	18.20628
ΔCT-SYP	-14.28341	-14.56245	-13.80233	-12.90995	-13.10053	-13.12111	-12.94015	-13.30727	-13.20779
ΔΔCT-SYP	0.067309	0.346348	-0.413771	-1.306148	-1.115569	-1.094989	-1.275953	-0.908834	-1.008311
2^(-Δ ΔCT)	0.954416	0.786572	1.332164	2.472804	2.166804	2.136115	2.421587	1.877527	2.011554

Fig.6 G		C4-2 Vehicle			c-Fos OE	
	1	2	3	1	2	3
CT-FOXC1	30.57547	31.17168	30.0487	30.35896	30.67035	30.4694
CT-actin	13.32309	14.21738	13.43412	14.18417	15.32413	14.85681
ΔCT-FOXC	-16.91728	-17.51349	-16.39051	-15.57059	-15.88198	-15.68103
ΔΔCT-FOX	-0.023124	0.573086	-0.549893	-1.369812	-1.058416	-1.259375
2^(-Δ ΔCT)	1.016157	0.672177	1.463977	2.584369	2.082643	2.39392

Fig.6 H		C4-2 Vehicle			c-Fos OE	
	1	2	3	1	2	3
CT-SOX2	30.1847	30.58193	30.31391	30.75576	30.88786	30.36411
CT-actin	13.32309	14.21738	13.43412	14.18417	15.32413	14.85681
ΔCT-SOX2	-16.52651	-16.92374	-16.65572	-15.96739	-16.09949	-15.57574
ΔΔCT-SOX	-0.175492	0.22174	-0.046285	-0.734605	-0.602512	-1.126259
2^(-Δ ΔCT)	1.129349	0.857531	1.032602	1.663942	1.518358	2.182919

Fig.6 J		C4-2 Vehicle			c-Fos OE	
	1	2	3	1	2	3
CT-NSE	19.10867	19.06234	19.07349	19.59218	19.29172	19.59357
CT-actin	13.32309	14.21738	13.43412	14.18417	15.32413	14.85681
ΔCT-NSE	-5.450478	-5.404149	-5.4153	-4.80381	-4.503348	-4.805197
ΔΔCT-NSE	0.027168	-0.019161	-0.00801	-0.6195	-0.919962	-0.618113
2^(-Δ ΔCT)	0.981345	1.01337	1.005567	1.536343	1.892066	1.534867

Fig.6 K		C4-2 Vehicle			c-Fos OE	
	1	2	3	1	2	3
CT-Chga	38.46558	36.68323	35.31827	34.64009	34.88332	34.59927
CT-actin	13.32309	14.21738	13.43412	14.18417	15.32413	14.85681
ΔCT-Chga	-24.80739	-23.02504	-21.66008	-19.85172	-20.09495	-19.8109
ΔΔCT-Chg	1.643191	-0.139161	-1.504124	-3.312481	-3.069251	-3.353302
2^(-Δ ΔCT)	0.320148	1.101264	2.836524	9.934732	8.393374	10.21985

Fig.6 I		C4-2 Vehicle			c-Fos OE	
	1	2	3	1	2	3
CT-SYP	27.56935	27.93945	28.1156	28.42238	28.27172	28.538
CT-actin	13.32309	14.21738	13.43412	14.18417	15.32413	14.85681
ΔCT-SYP	-13.91116	-14.28126	-14.45741	-13.63401	-13.48335	-13.74963
ΔΔCT-SYP	-0.305442	0.064658	0.240815	-0.582594	-0.733245	-0.466968
2^(-Δ ΔCT)	1.235797	0.956172	0.846267	1.49754	1.662374	1.382201

	Fig.6 M		
**The input sample was diluted to 1%**			
**CT**	**DMSO**	**IOX2**	**IOX2+T-5224**
**Input1**	36.03088	37.37564	35.17415
**Input2**	36.40154	37.92814	36.46781
**Input3**	34.9557	37.01265	34.98133
**ChIP1**	37.73231	37.15818	36.60558
**ChIP2**	37.80525	37.23542	37.4871
**ChIP3**	37.09115	37.33935	37.11588
**IgG1**	0	0	0
**IgG2**	0	0	0
**IgG3**	0	0	0
**ΔCT**	**DMSO**	**IOX2**	**IOX2+T-5224**
**ChIP1**	-1.936272	0.280631	-1.064491
**ChIP2**	-2.009206	0.203386	-1.946008
**ChIP3**	-1.295112	0.099456	-1.57479
**2^(-ΔCT）**	**DMSO**	**IOX2**	**IOX2+T-5224**
**ChIP1**	3.827155	0.823231	2.091432
**ChIP2**	4.025606	0.86851	3.853069
**ChIP3**	2.45396	0.933385	2.978921
**% Input**	**DMSO**	**IOX2**	**IOX2+T-5224**
**ChIP1**	0.261291	1.214726	0.478141
**ChIP2**	0.24841	1.151398	0.259533
**ChIP3**	0.407505	1.07137	0.335692

Fig.6 O	LNCaP-DMSO			IOX2			IOX2+T-5224		
	Absorbance at 450 nm of the bla								
OD450 (Ab	1	2	3	1	2	3	1	2	3
Control	0.789			0.633			0.546		
0.078125	0.806	0.745	0.828	0.606	0.598	0.677	0.537	0.574	0.536
0.15625	0.737	0.748	0.789	0.578	0.583	0.652	0.533	0.517	0.546
0.3125	0.814	0.796	0.728	0.643	0.591	0.599	0.509	0.534	0.521
0.625	0.741	0.756	0.699	0.595	0.631	0.622	0.448	0.483	0.475
1.25	0.653	0.651	0.648	0.585	0.575	0.594	0.466	0.492	0.471
2.5	0.66	0.685	0.578	0.636	0.604	0.534	0.481	0.48	0.451
5	0.474	0.418	0.488	0.545	0.553	0.513	0.281	0.268	0.277
10	0.352	0.329	0.345	0.449	0.428	0.432	0.211	0.252	0.309
20	0.157	0.169	0.145	0.304	0.304	0.327	0.172	0.203	0.155

Fig.6 P	C4-2-DMSO			IOX2			IOX2+T-5224		
	Absorbance at 450 nm of the bla								
OD450 (Ab	1	2	3	1	2	3	1	2	3
Control	0.677			1.015			0.923		
0.15625	0.559	0.601	0.595	0.944	0.994	0.925	0.952	0.9	0.92
0.3125	0.589	0.604	0.591	0.958	0.979	1.006	0.858	0.86	0.827
0.625	0.513	0.538	0.53	0.823	0.841	0.894	0.791	0.806	0.816
1.25	0.453	0.438	0.43	0.868	0.866	0.848	0.685	0.701	0.716
2.5	0.413	0.385	0.383	0.764	0.779	0.79	0.563	0.604	0.685
5	0.338	0.319	0.309	0.816	0.724	0.732	0.483	0.516	0.562
10	0.307	0.339	0.338	0.678	0.645	0.723	0.505	0.474	0.45
15	0.267	0.284	0.287	0.696	0.652	0.665	0.393	0.403	0.402
20	0.222	0.239	0.215	0.561	0.532	0.509	0.339	0.365	0.302

Fig.S1 D		LNCaP	PC-3
Relative m	1	0.6227	1.0178
	2	0.5592	1.337
	3	0.5996	1.1891
	4	0.5189	1.2168
	5	0.5765	1.1359

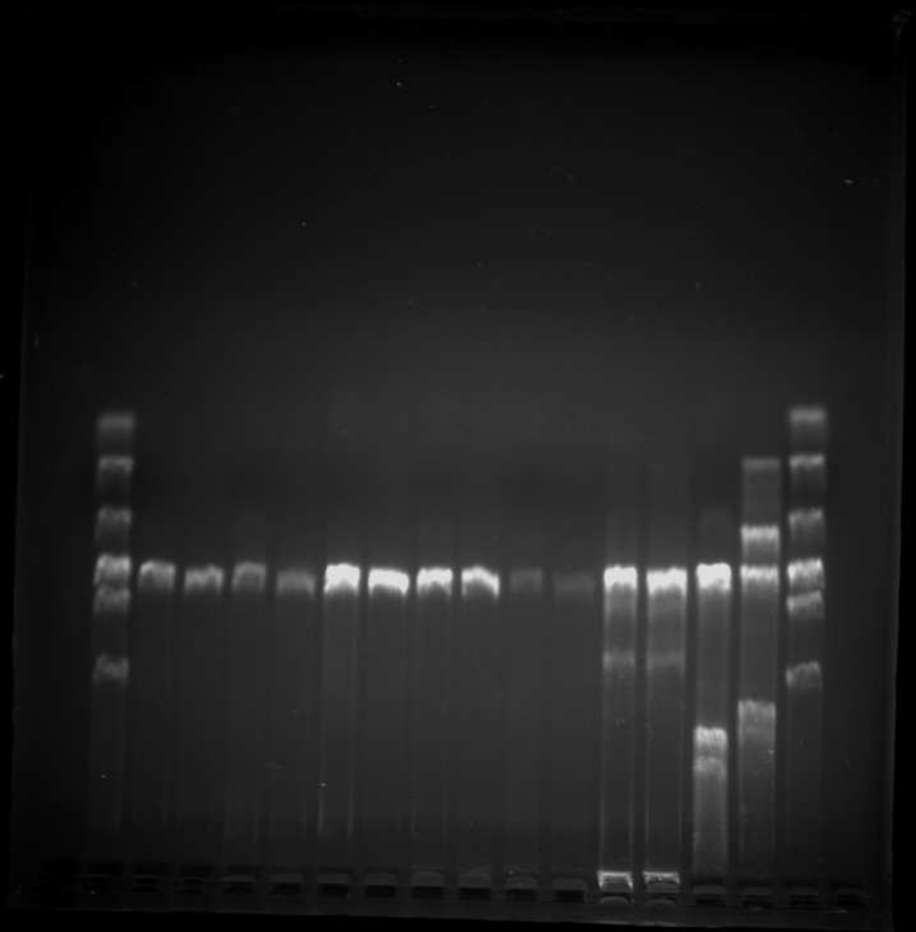

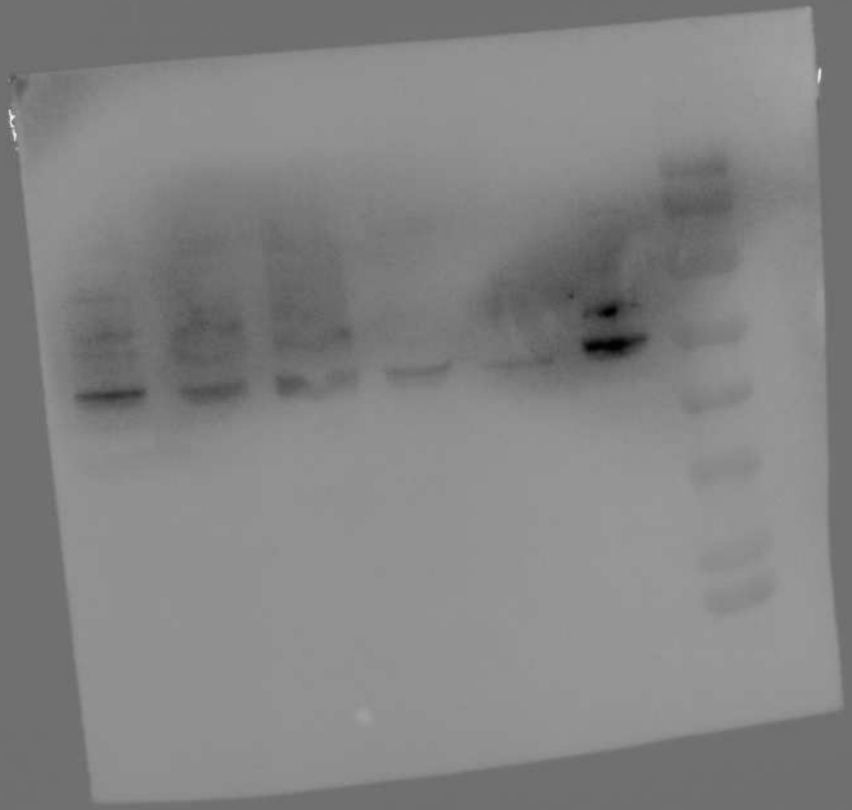

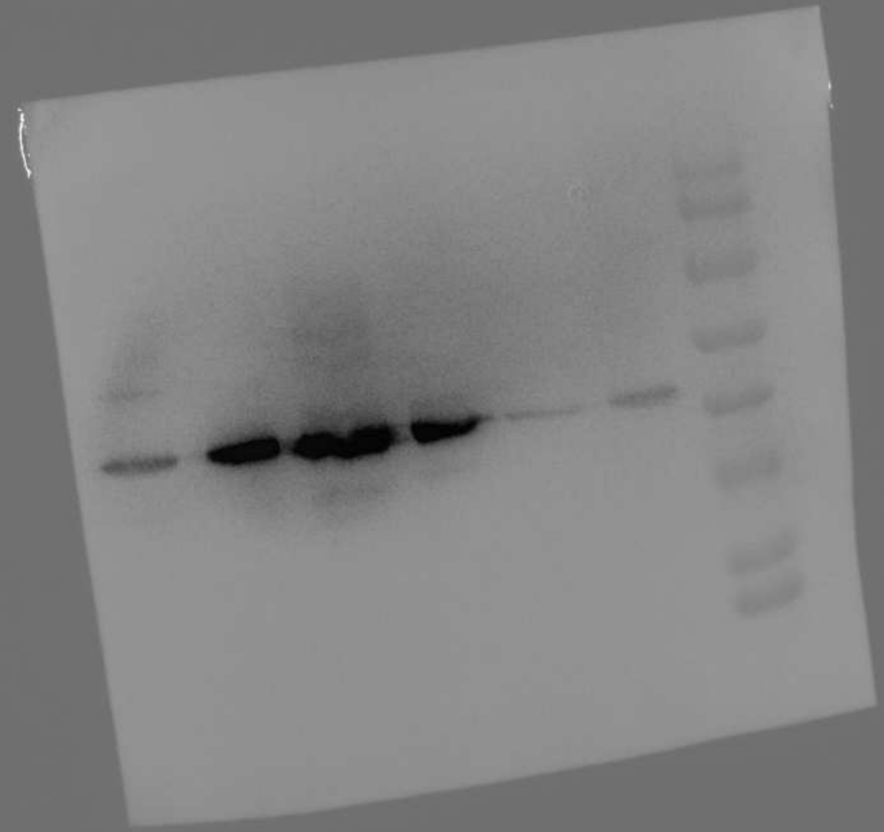

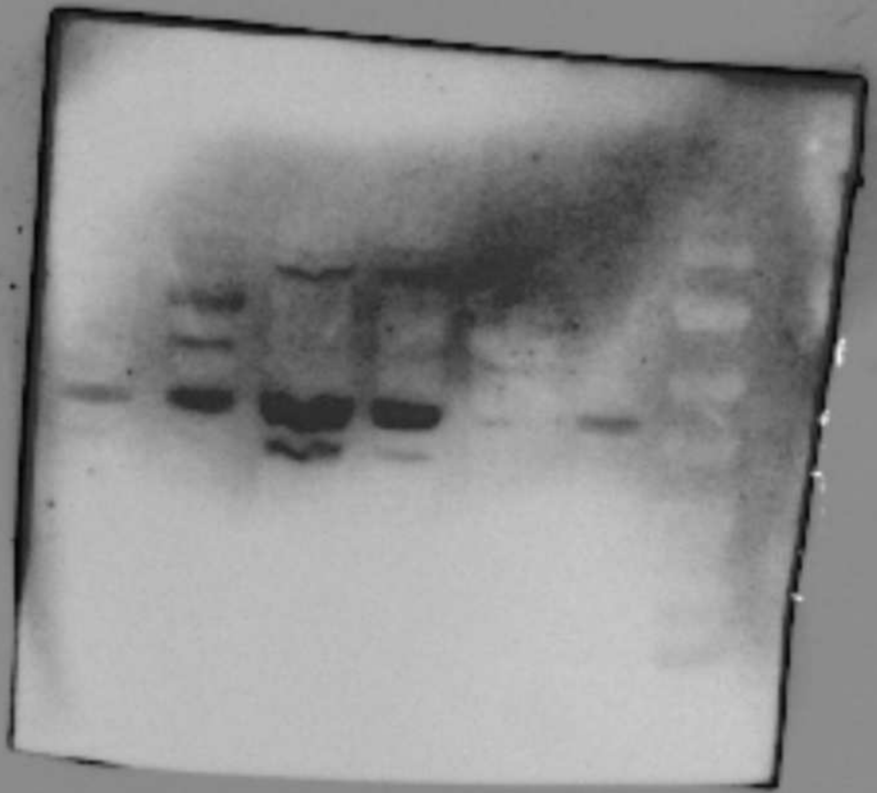

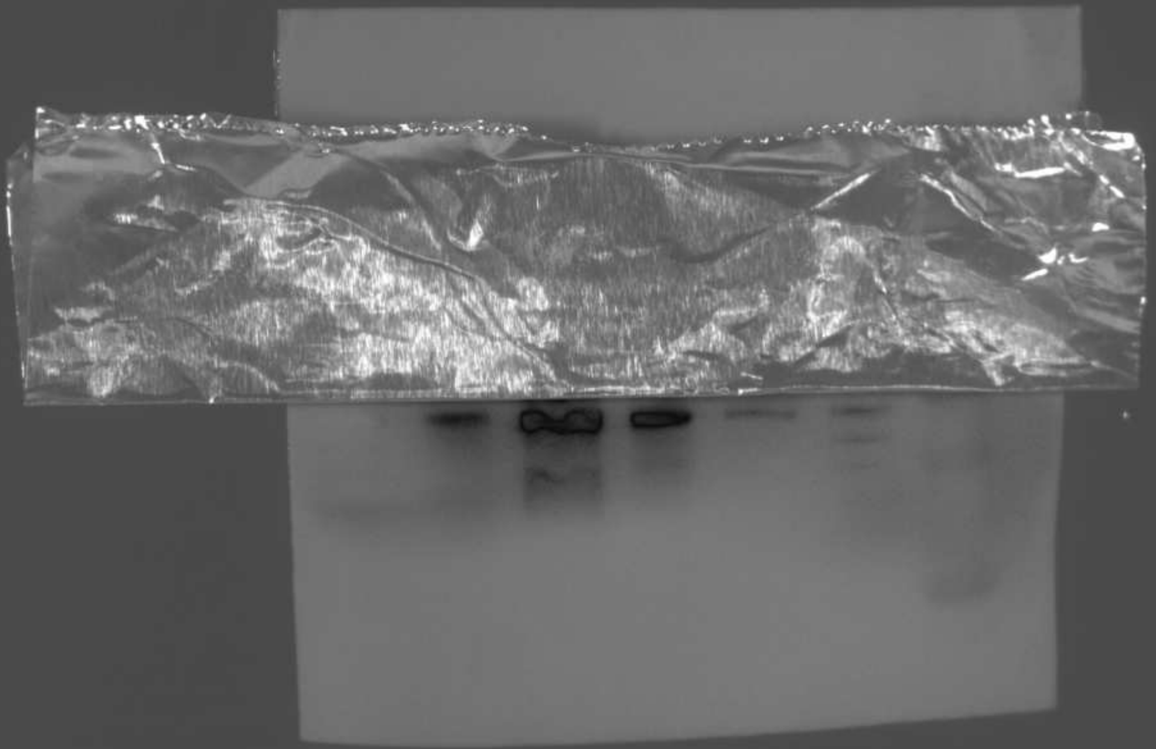

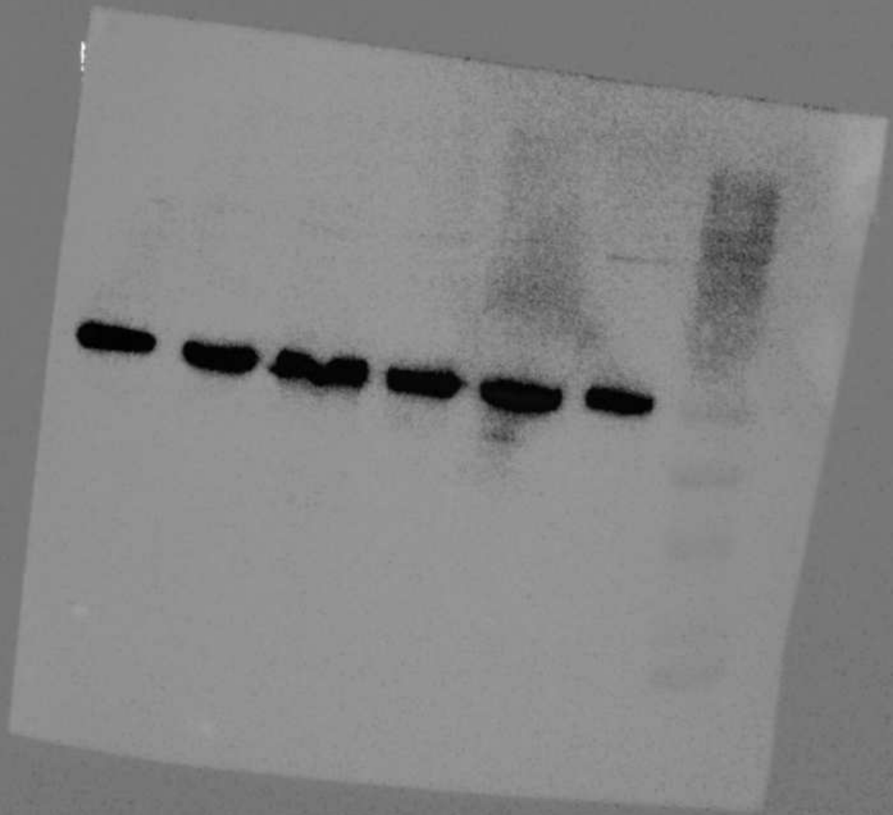

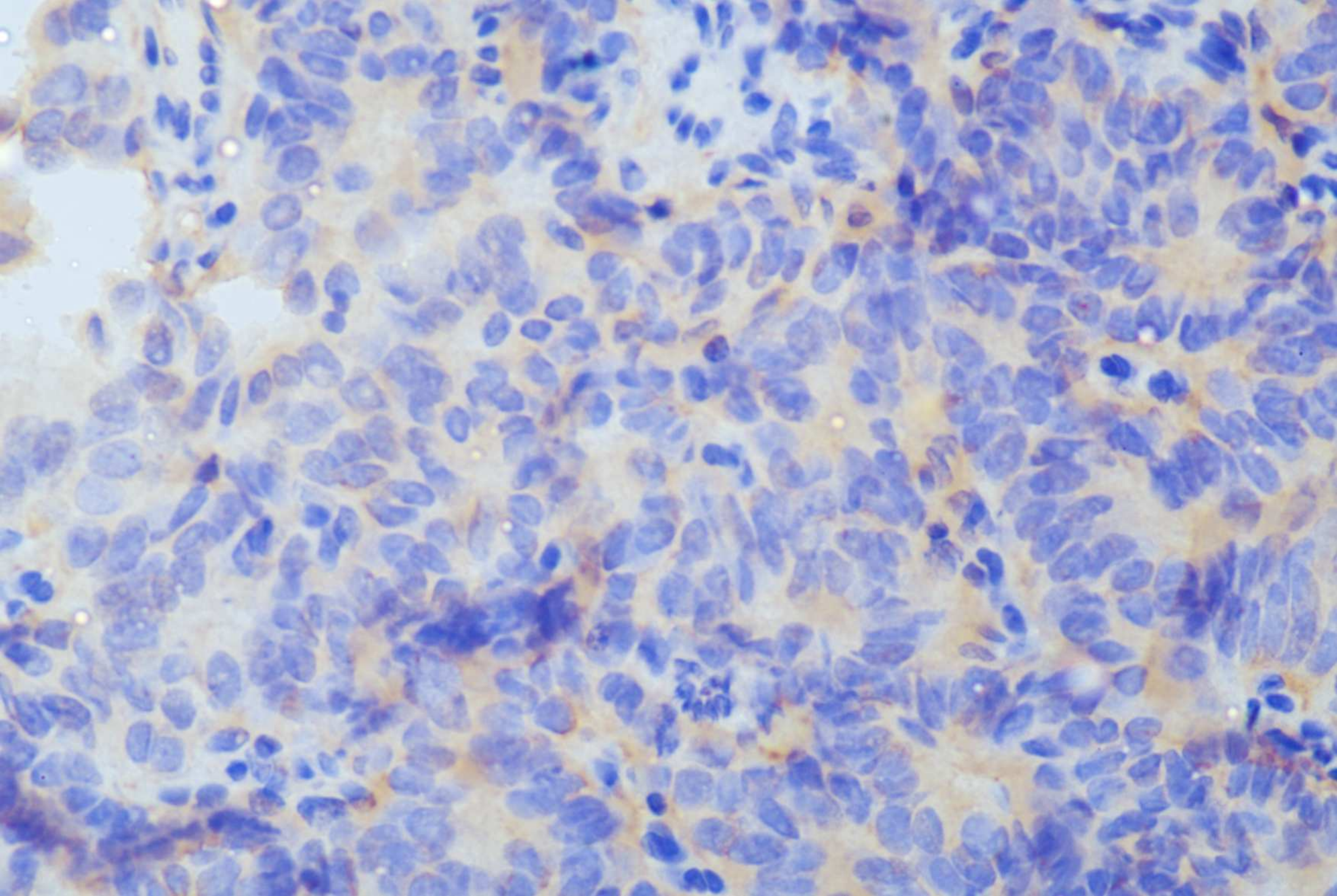

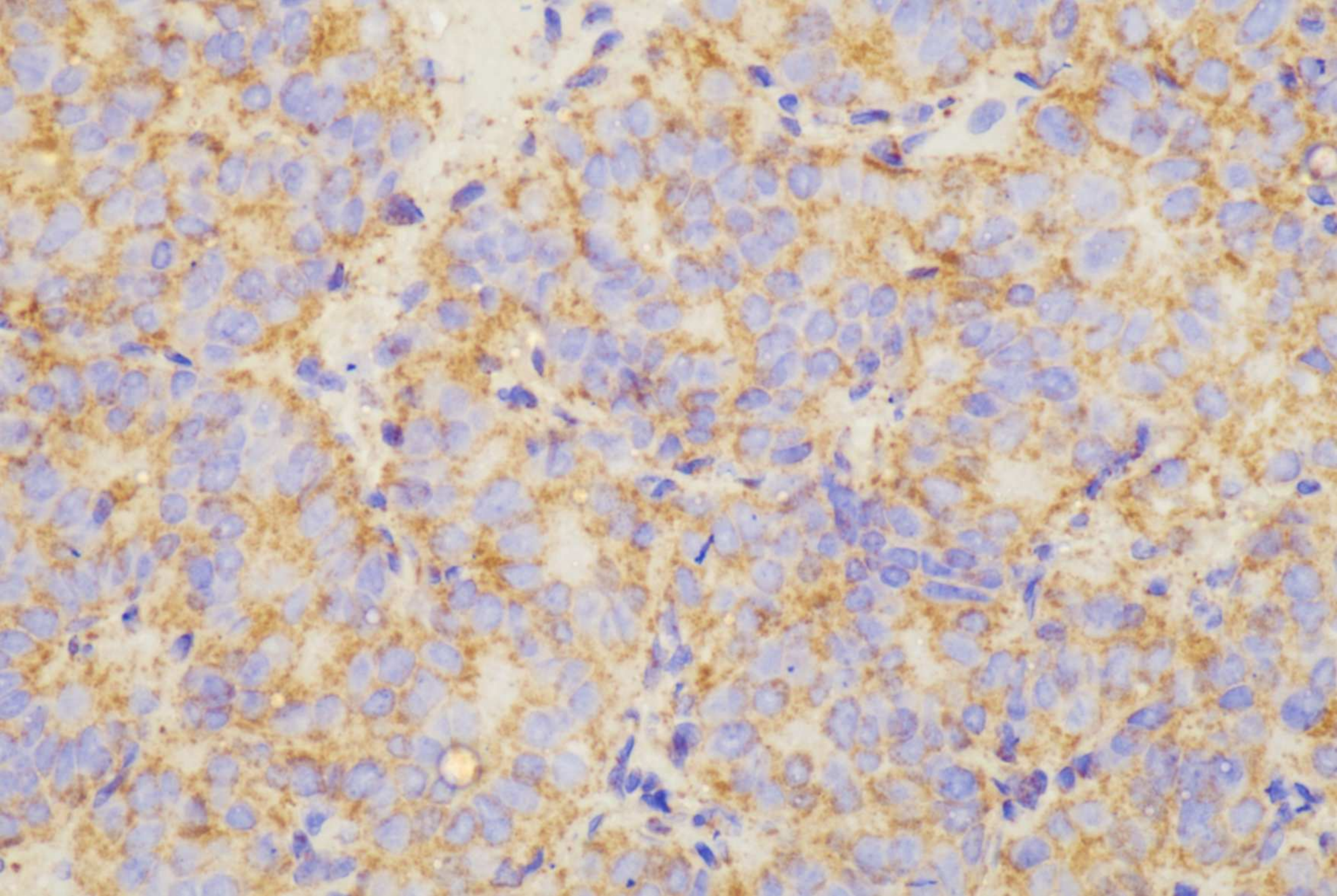

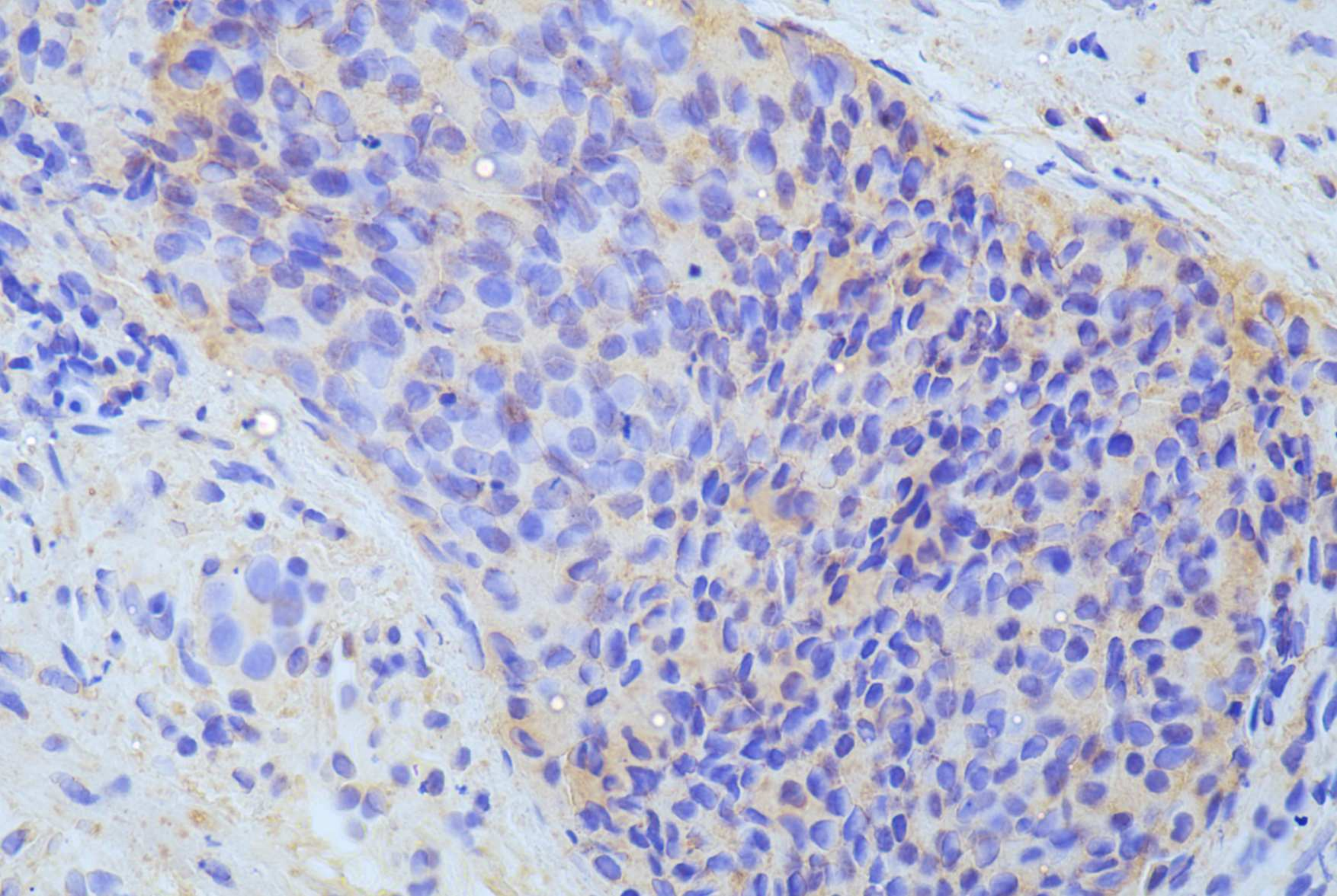

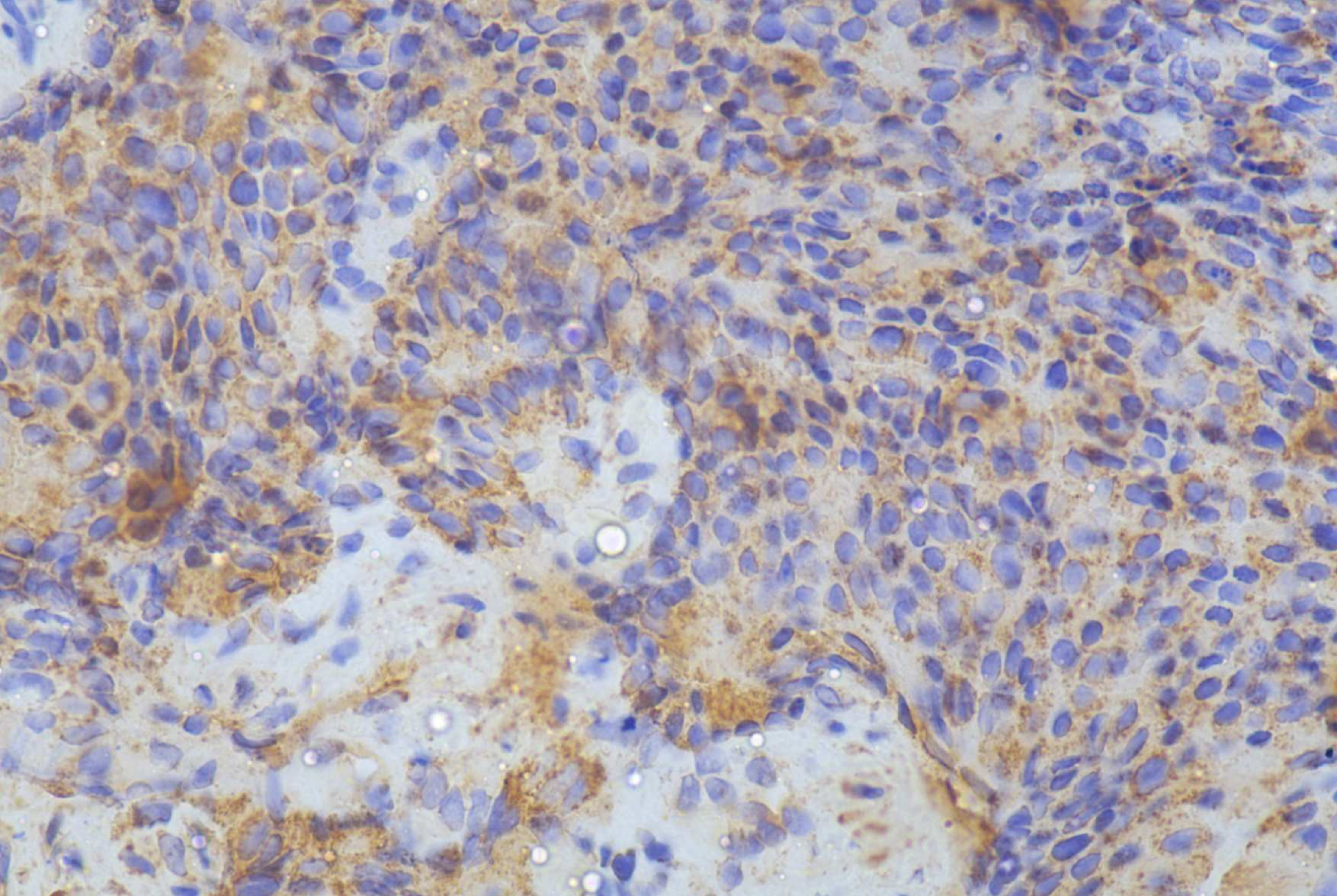

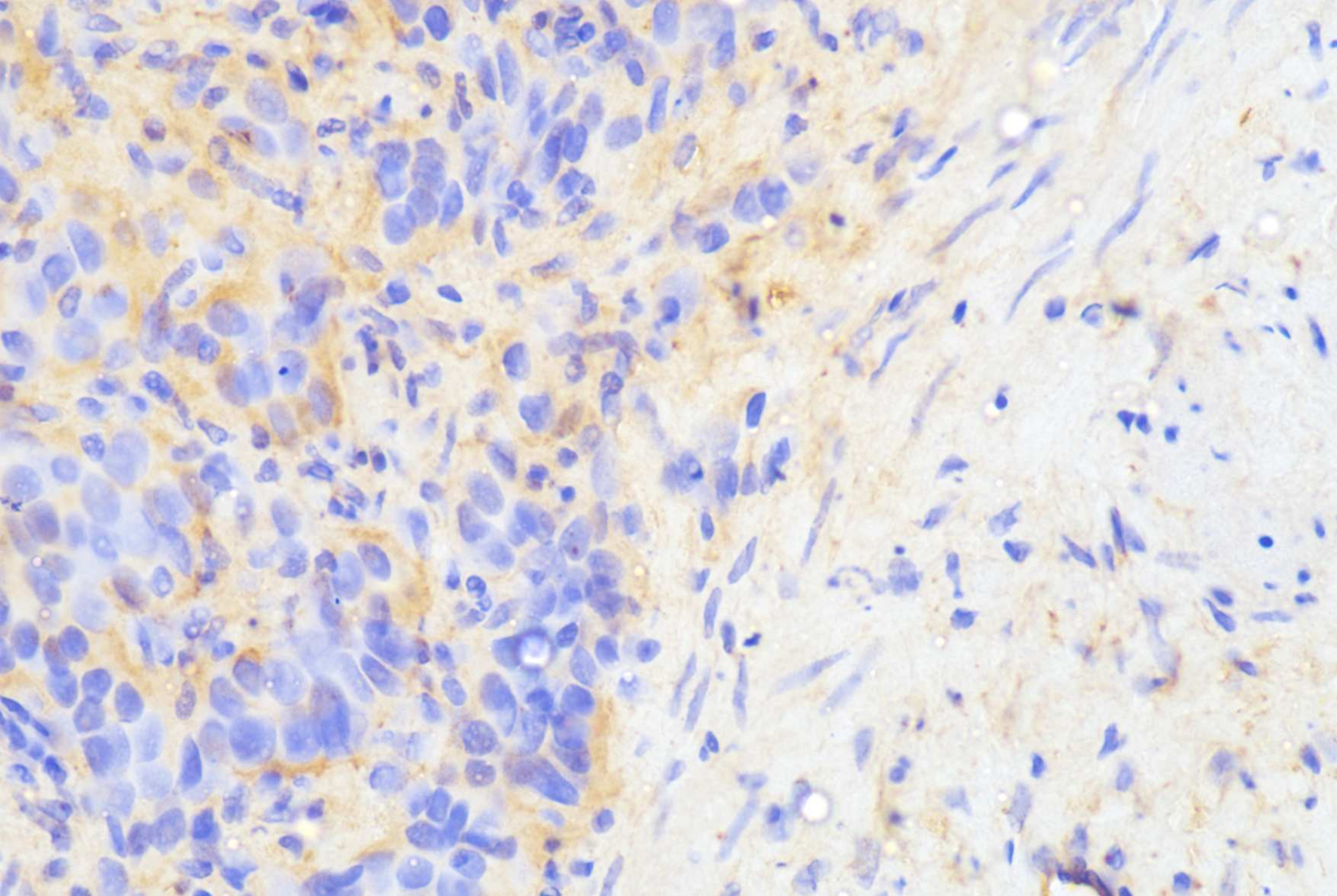

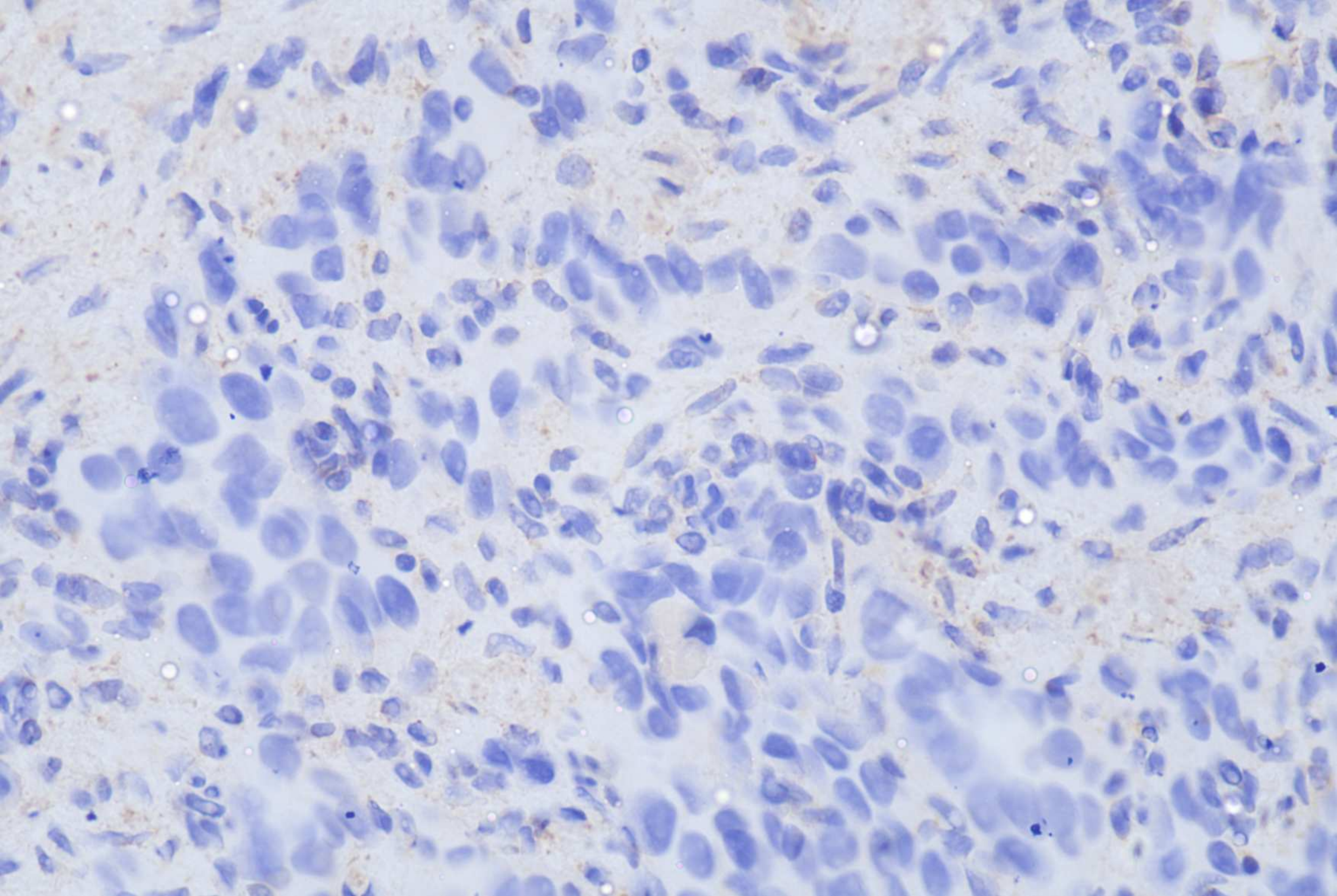

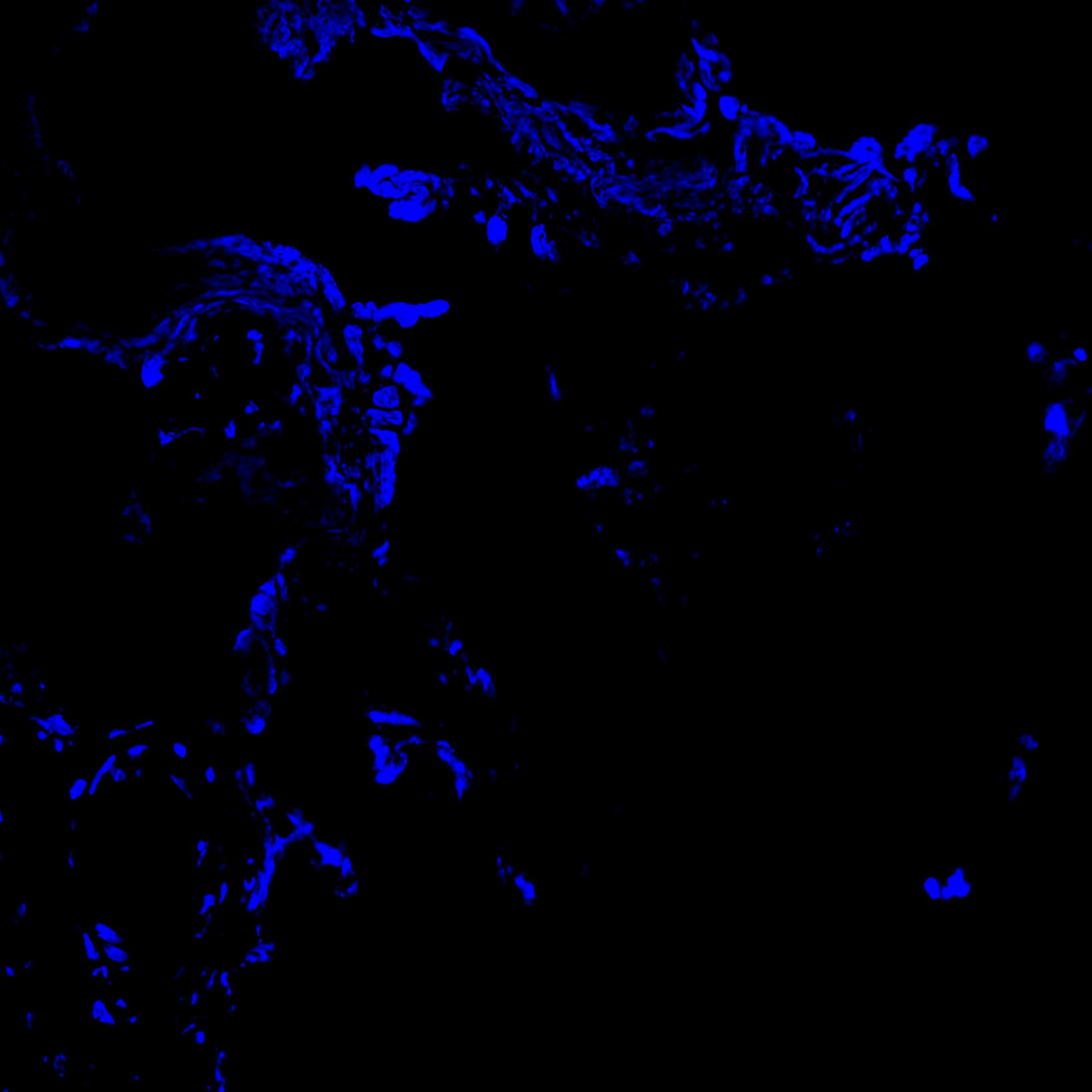

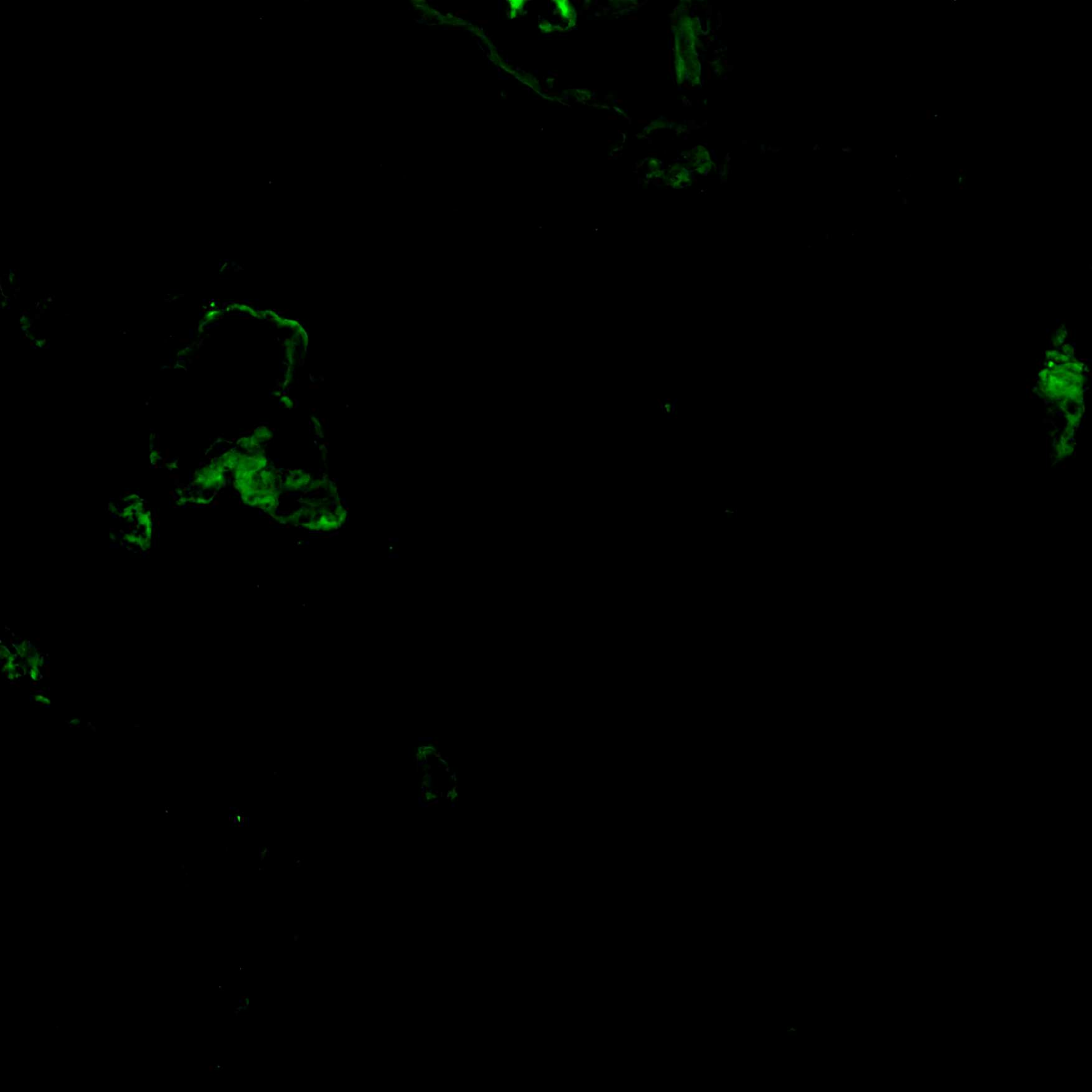

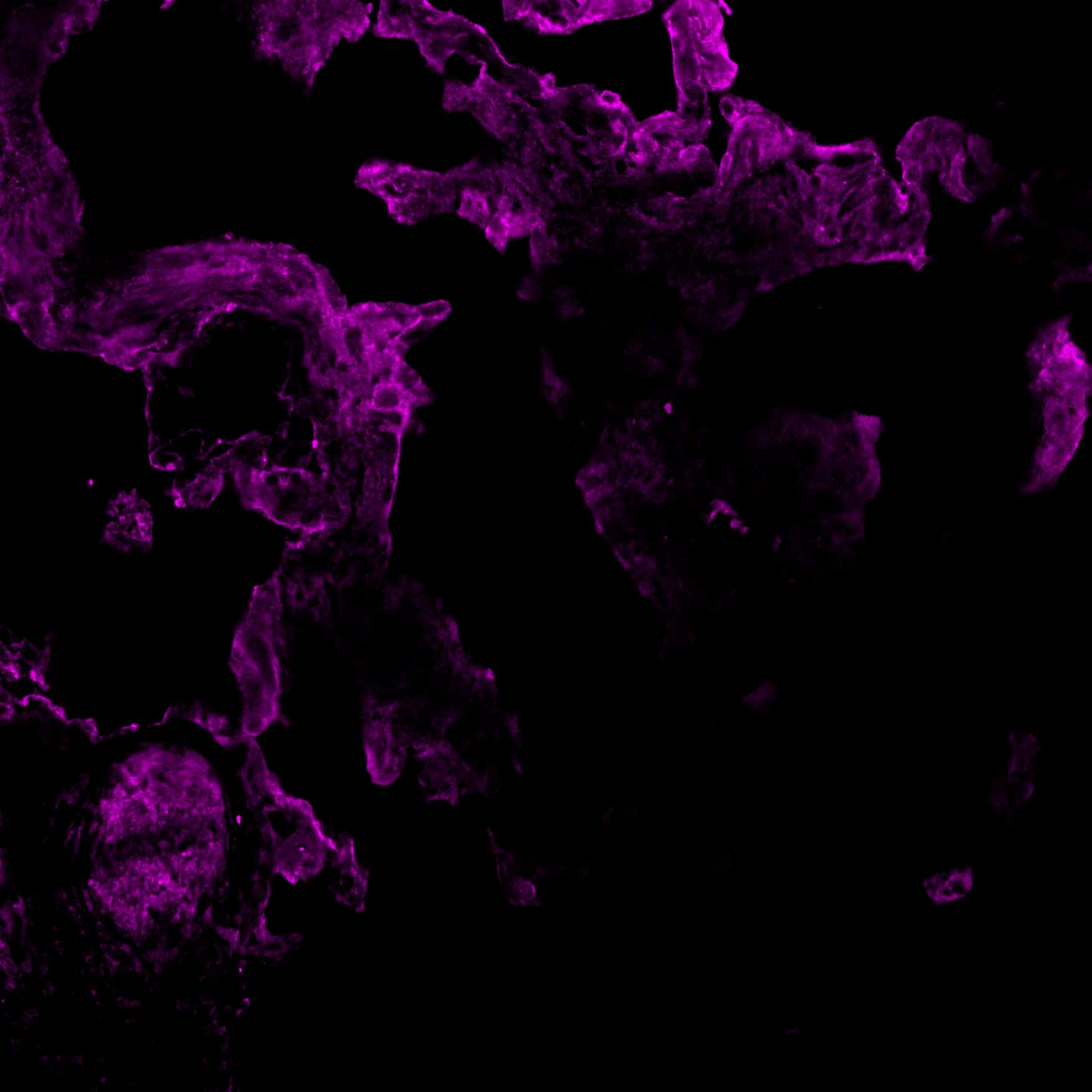

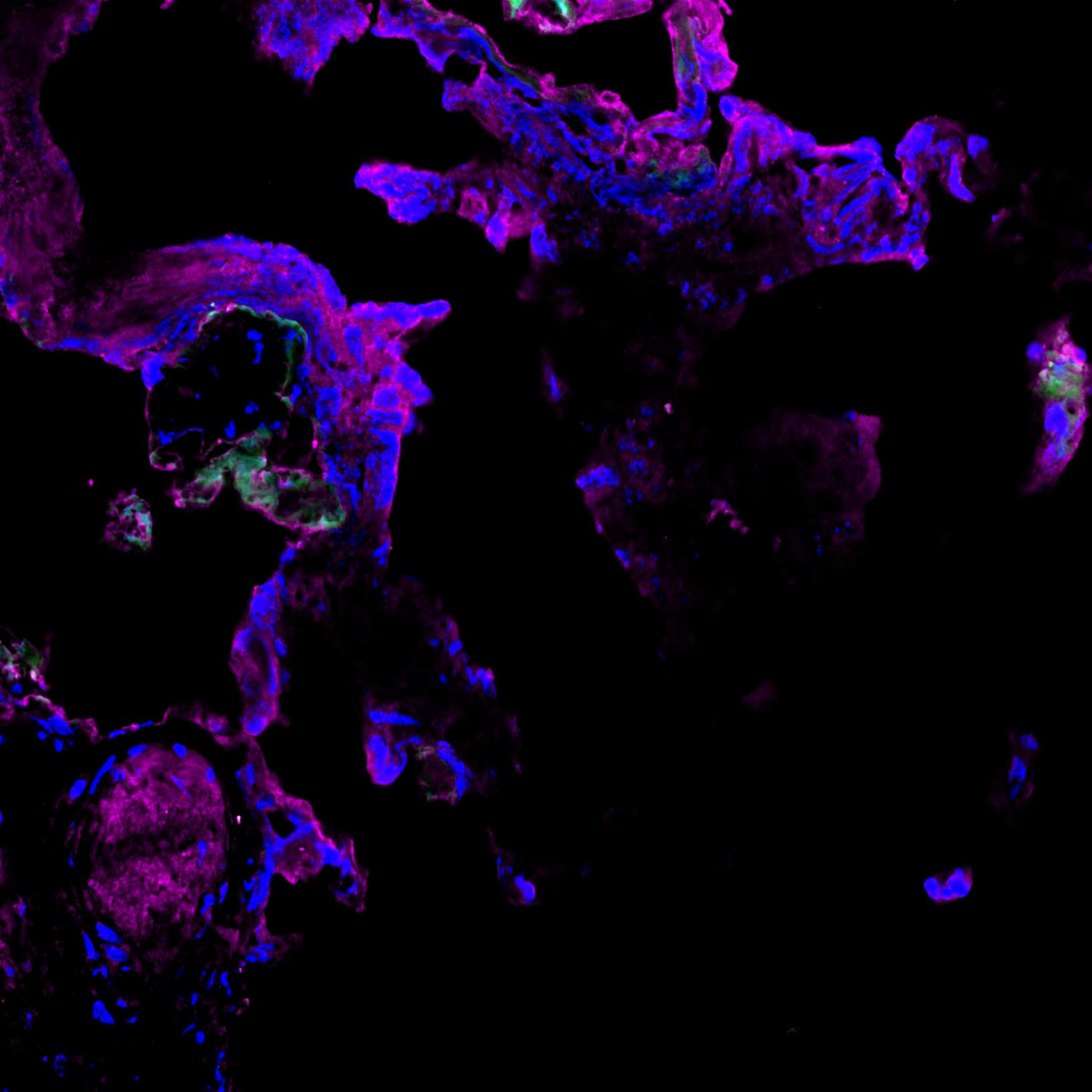

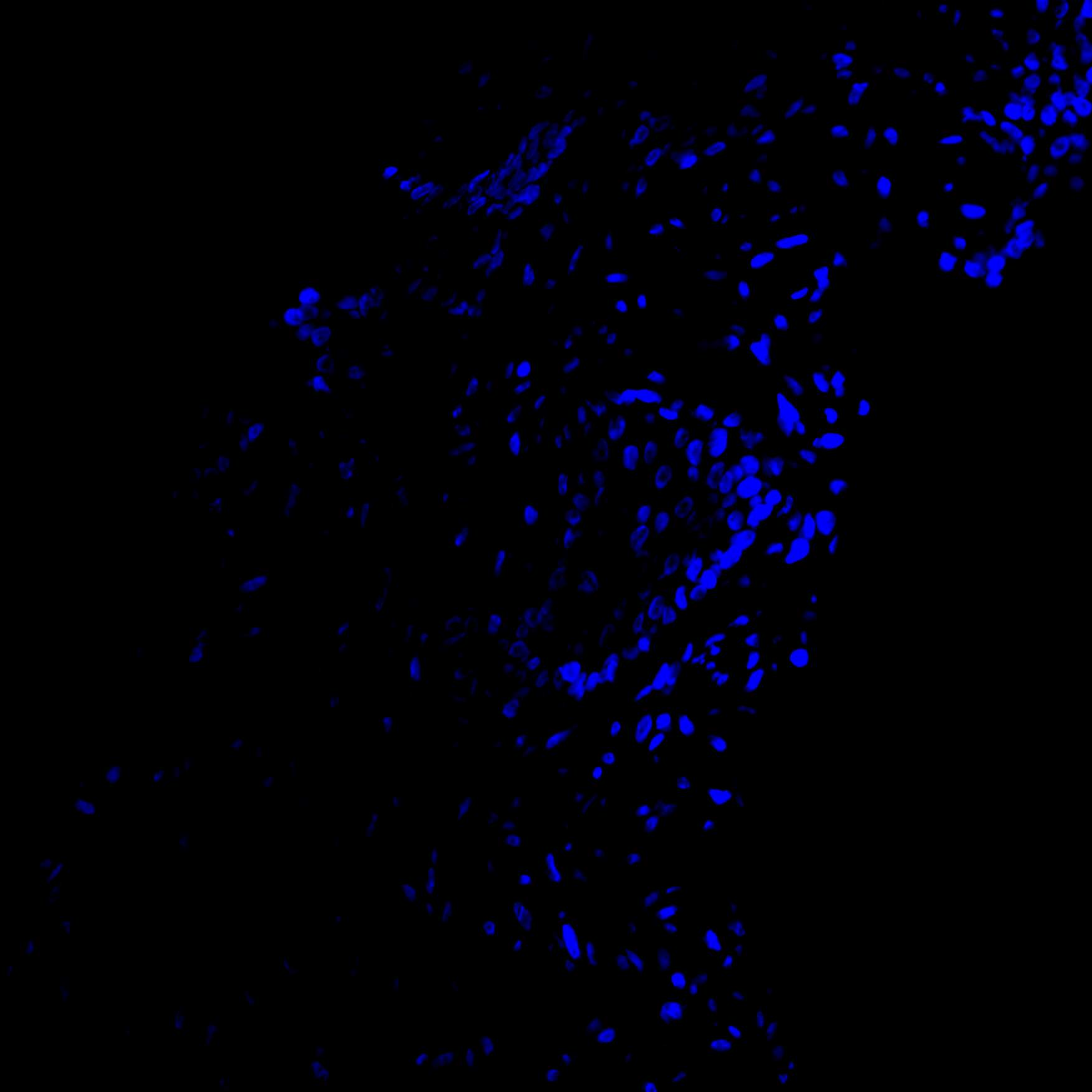

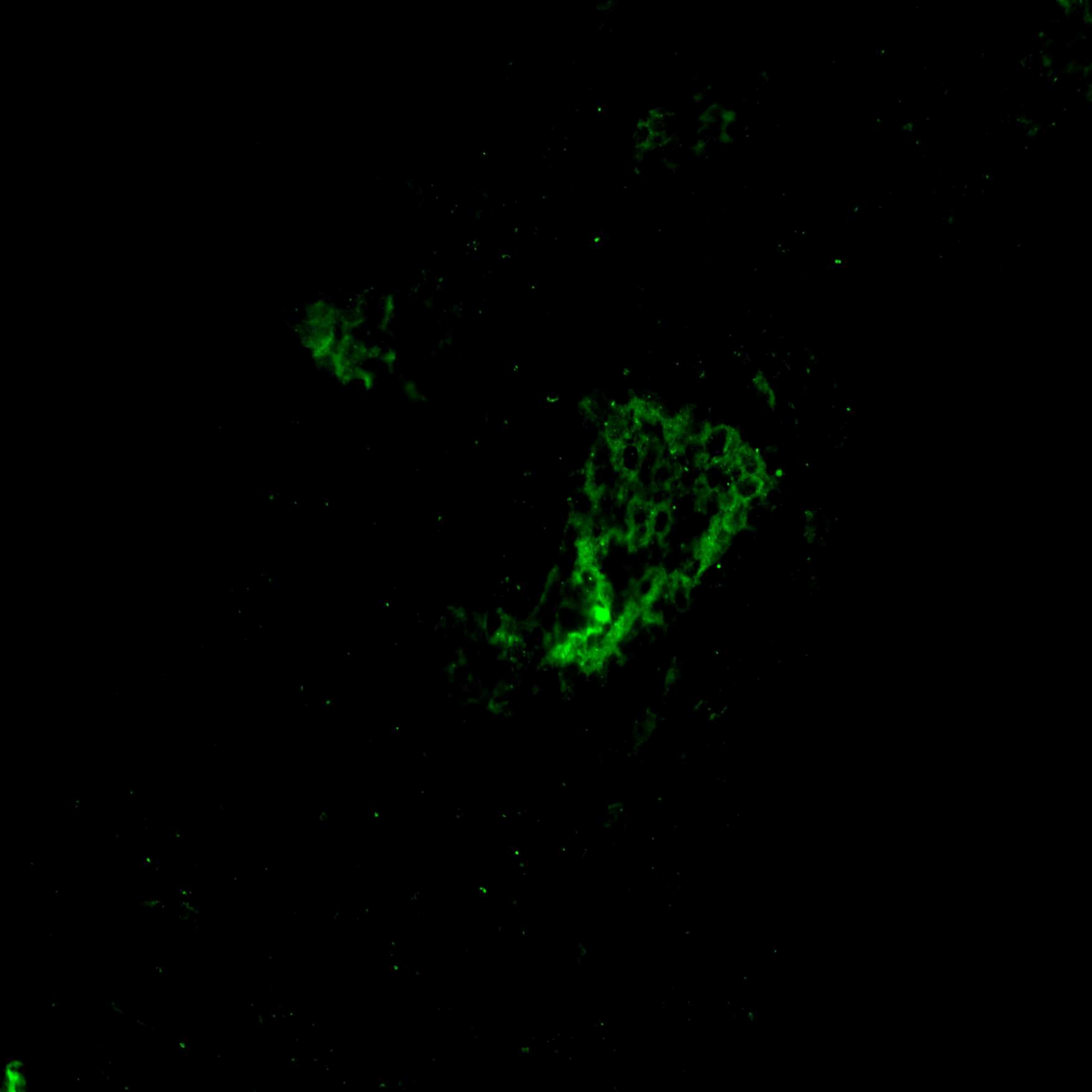

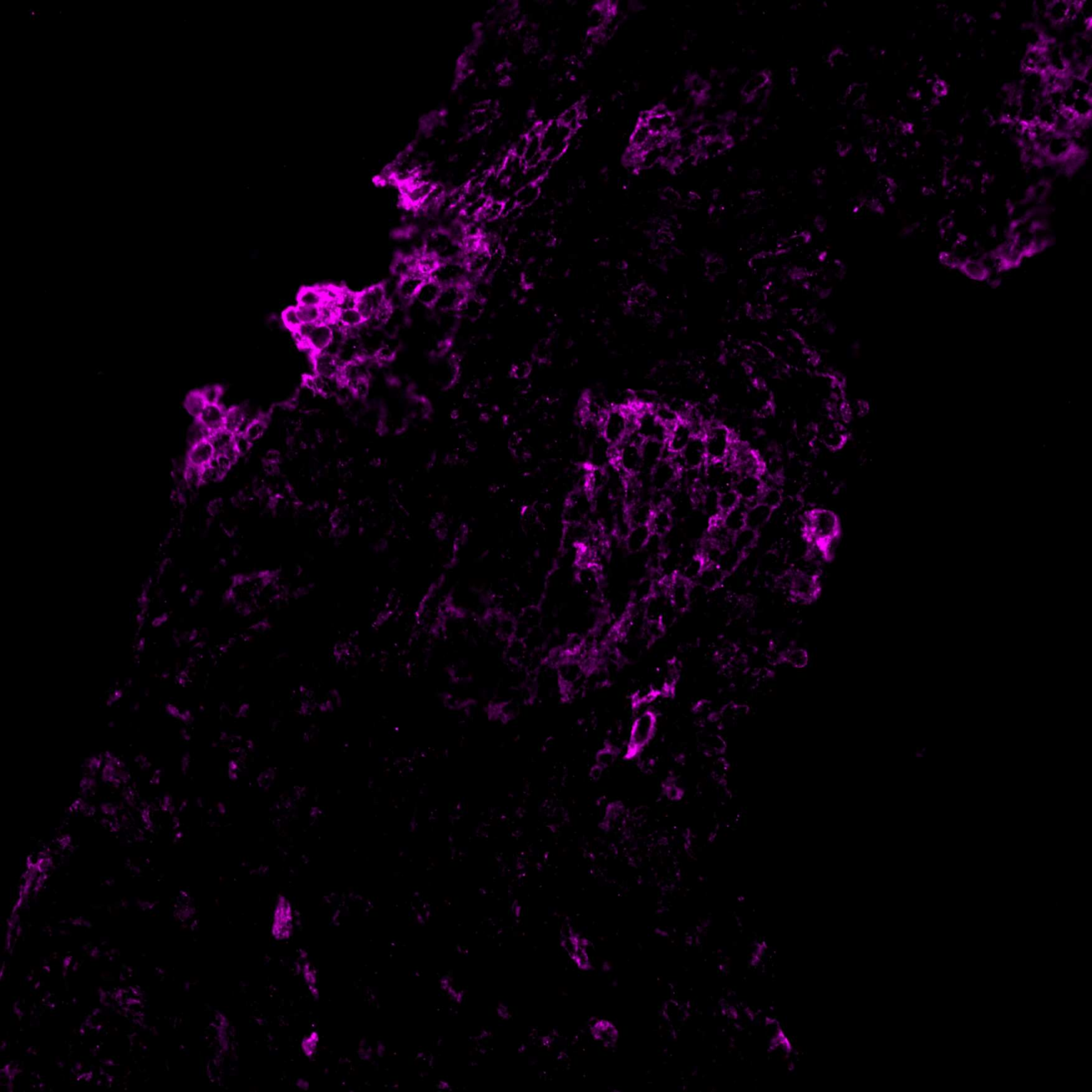

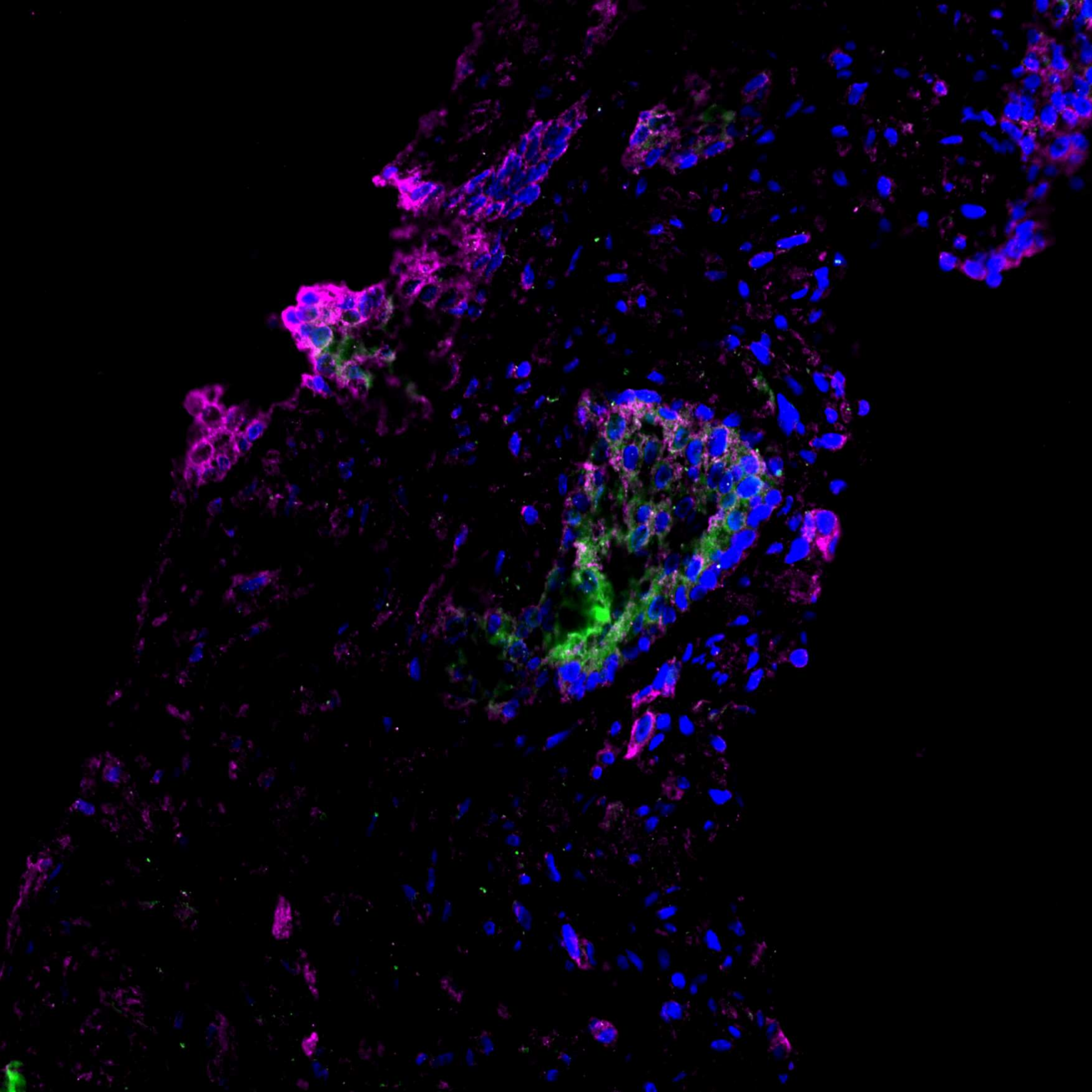

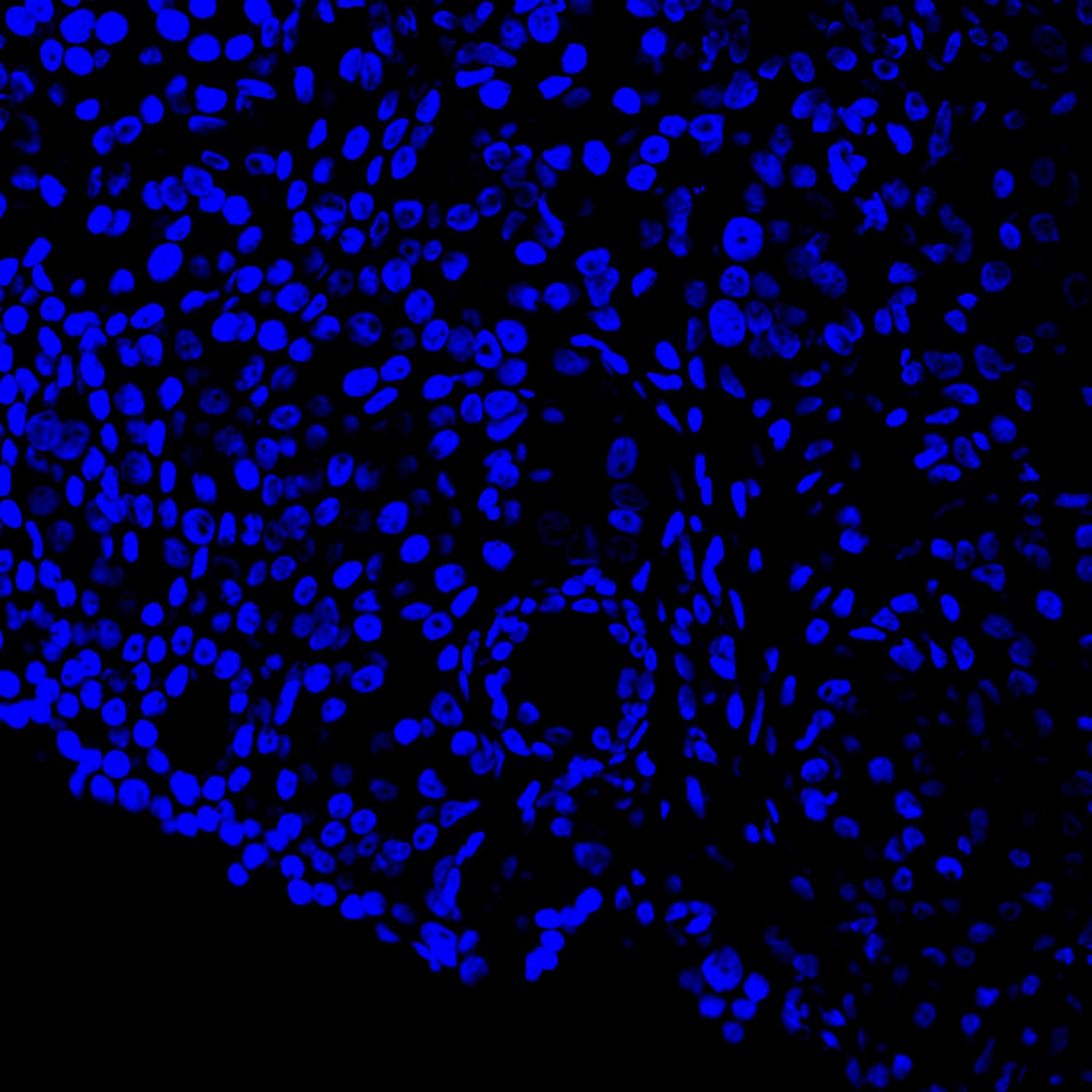

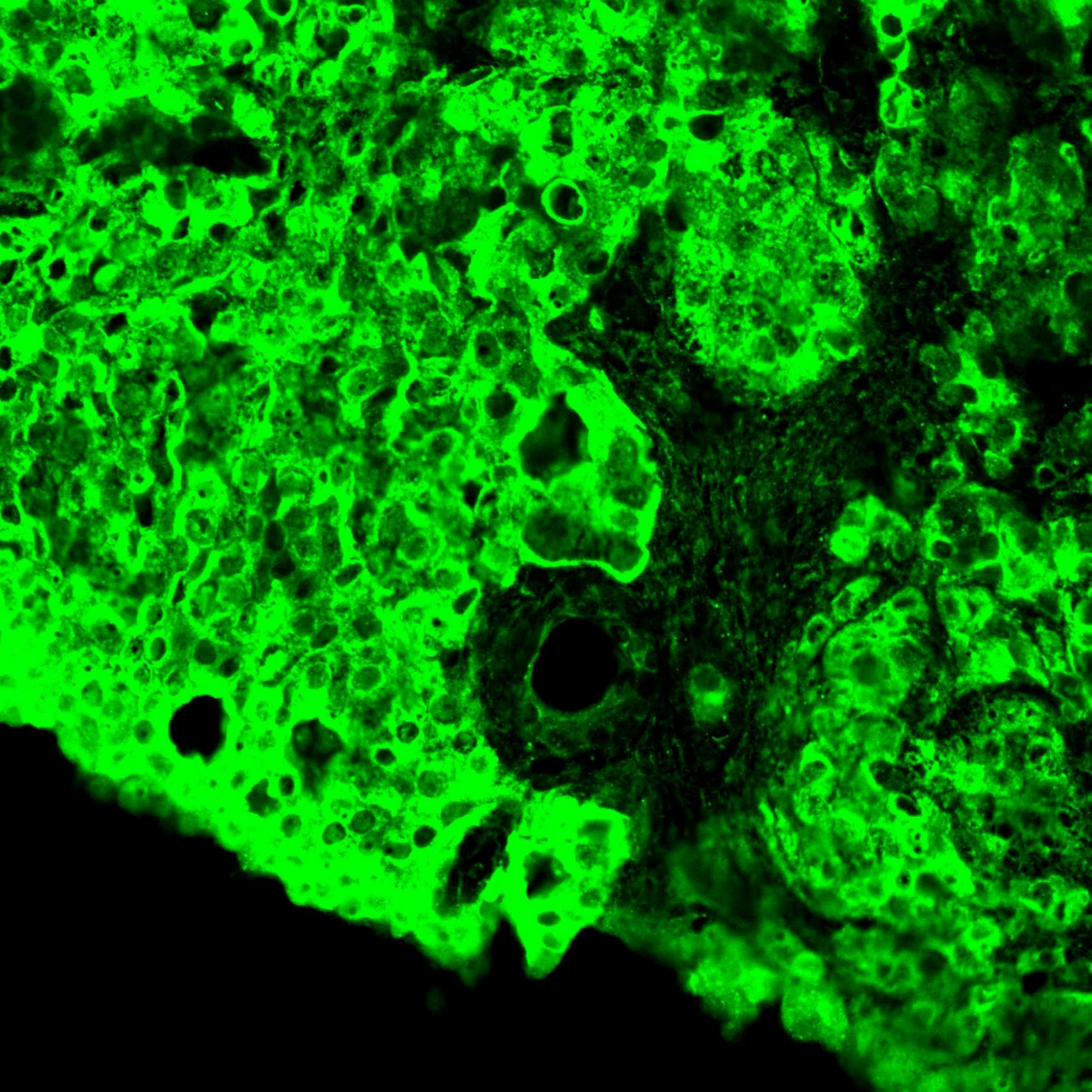

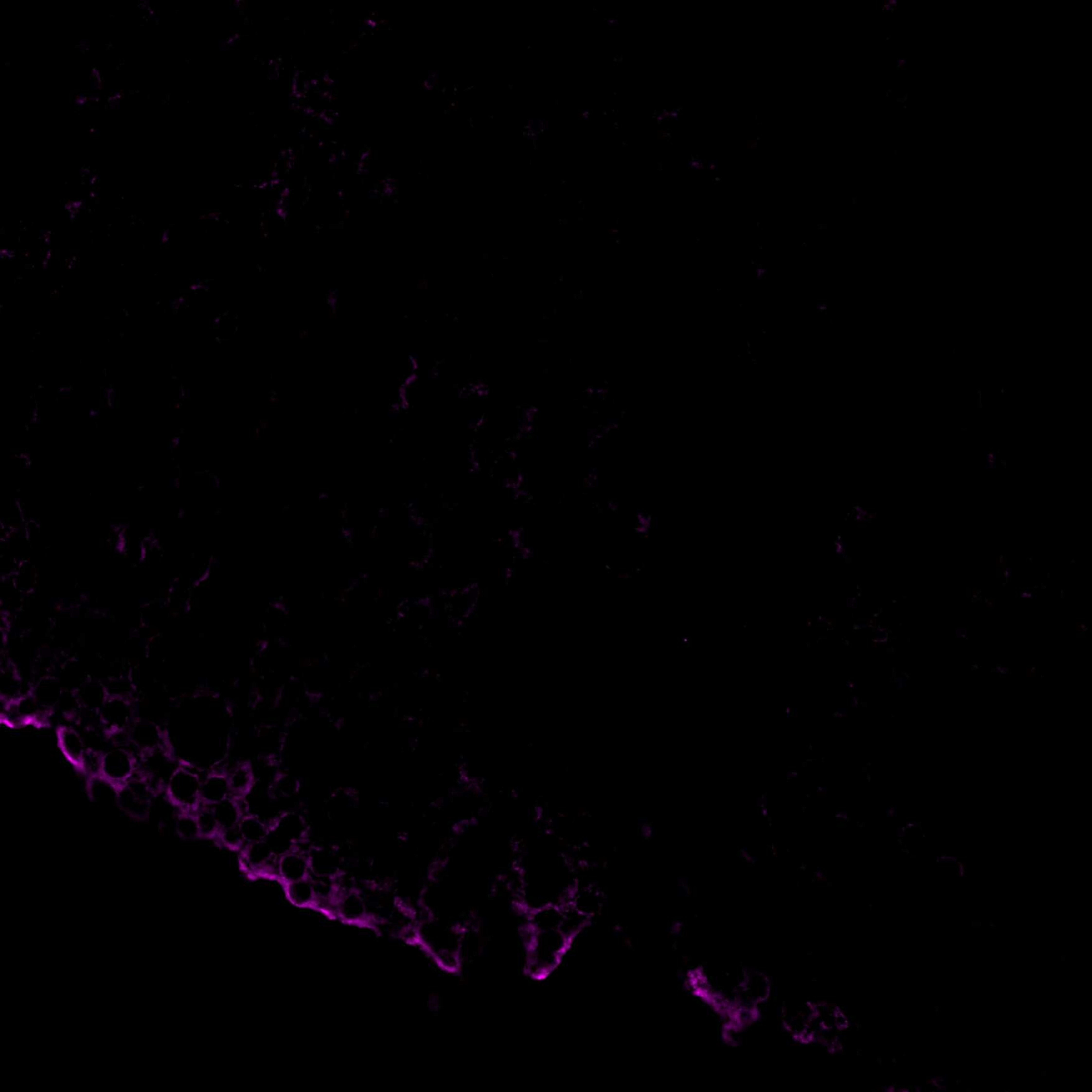

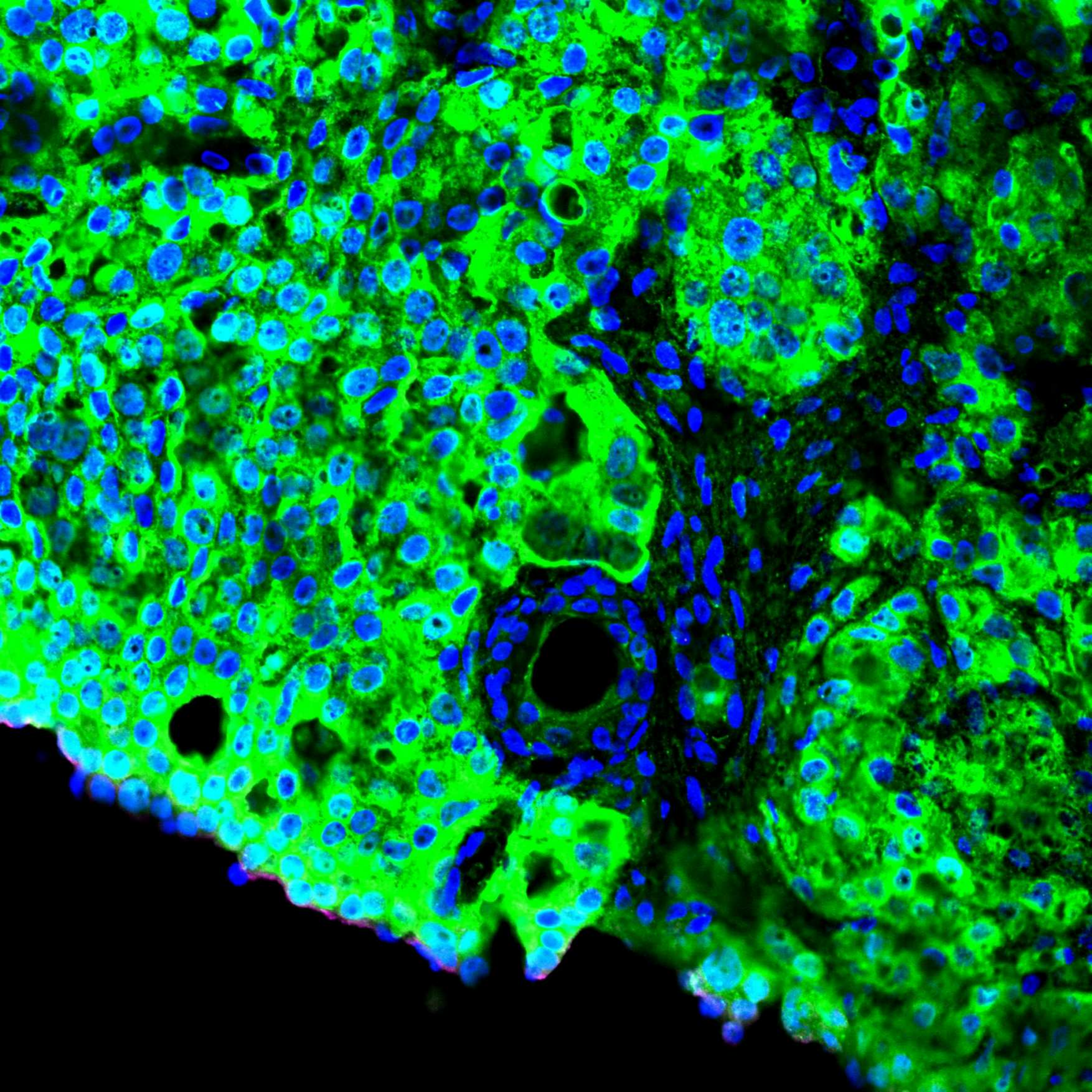

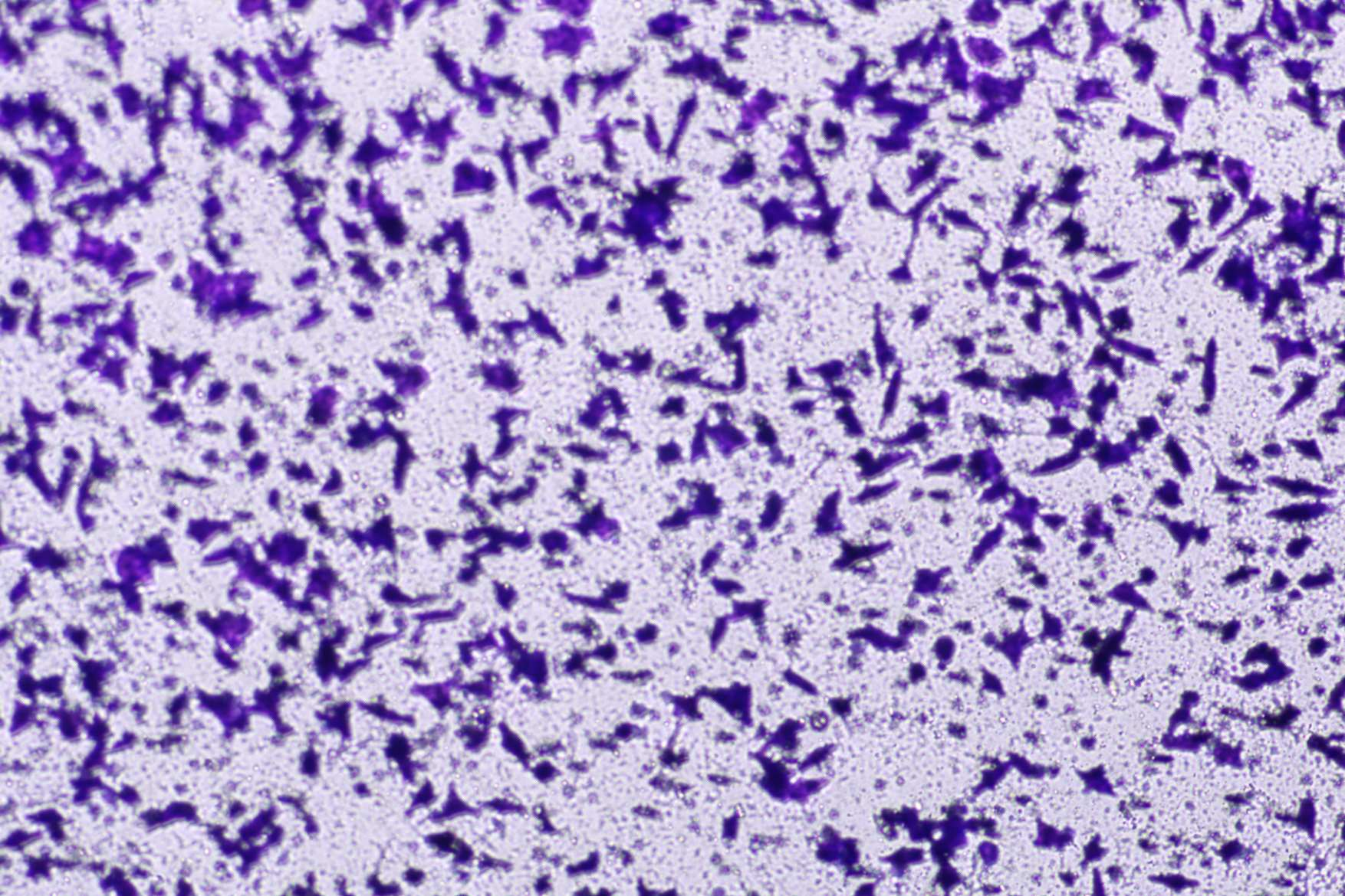

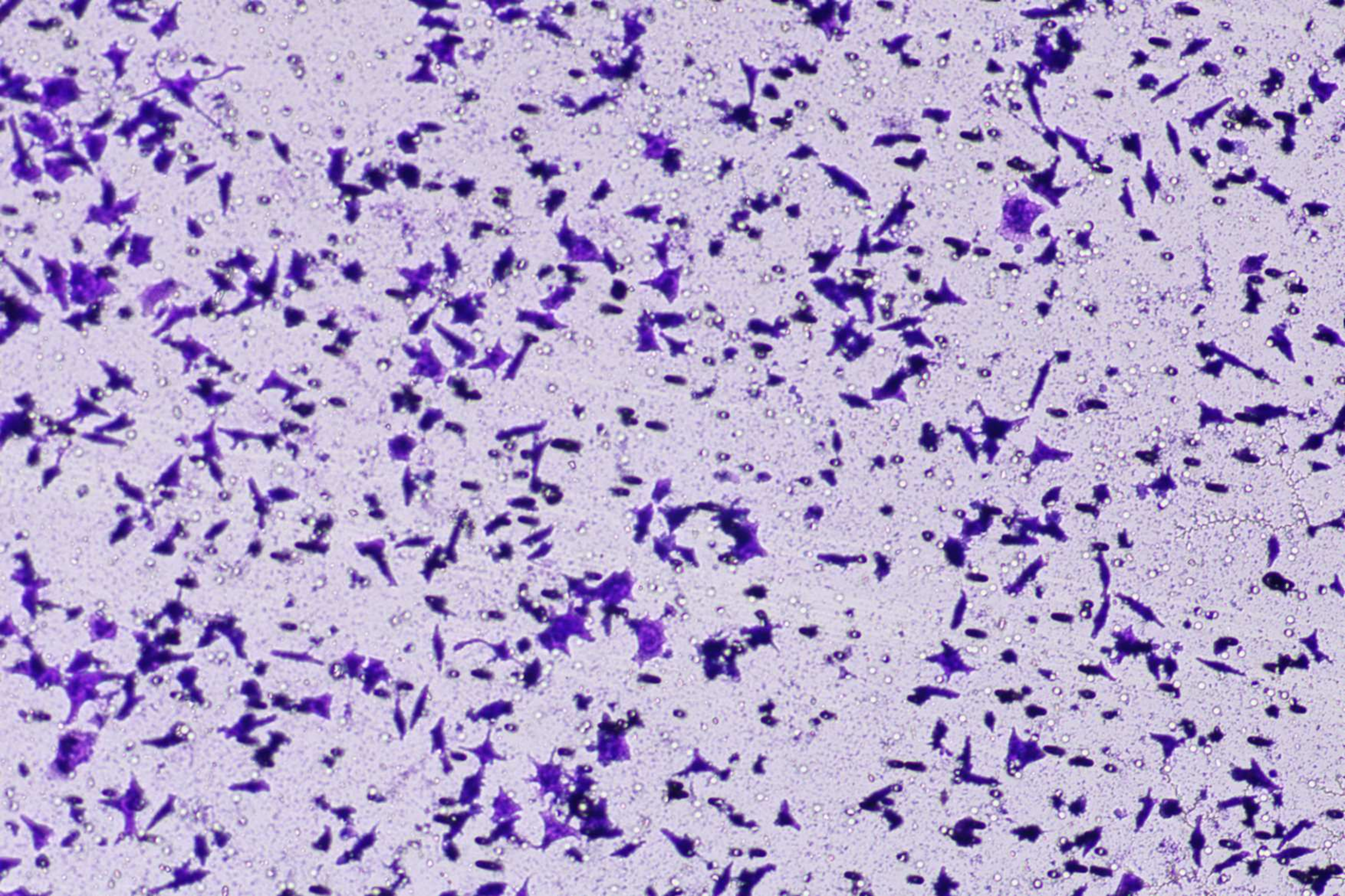

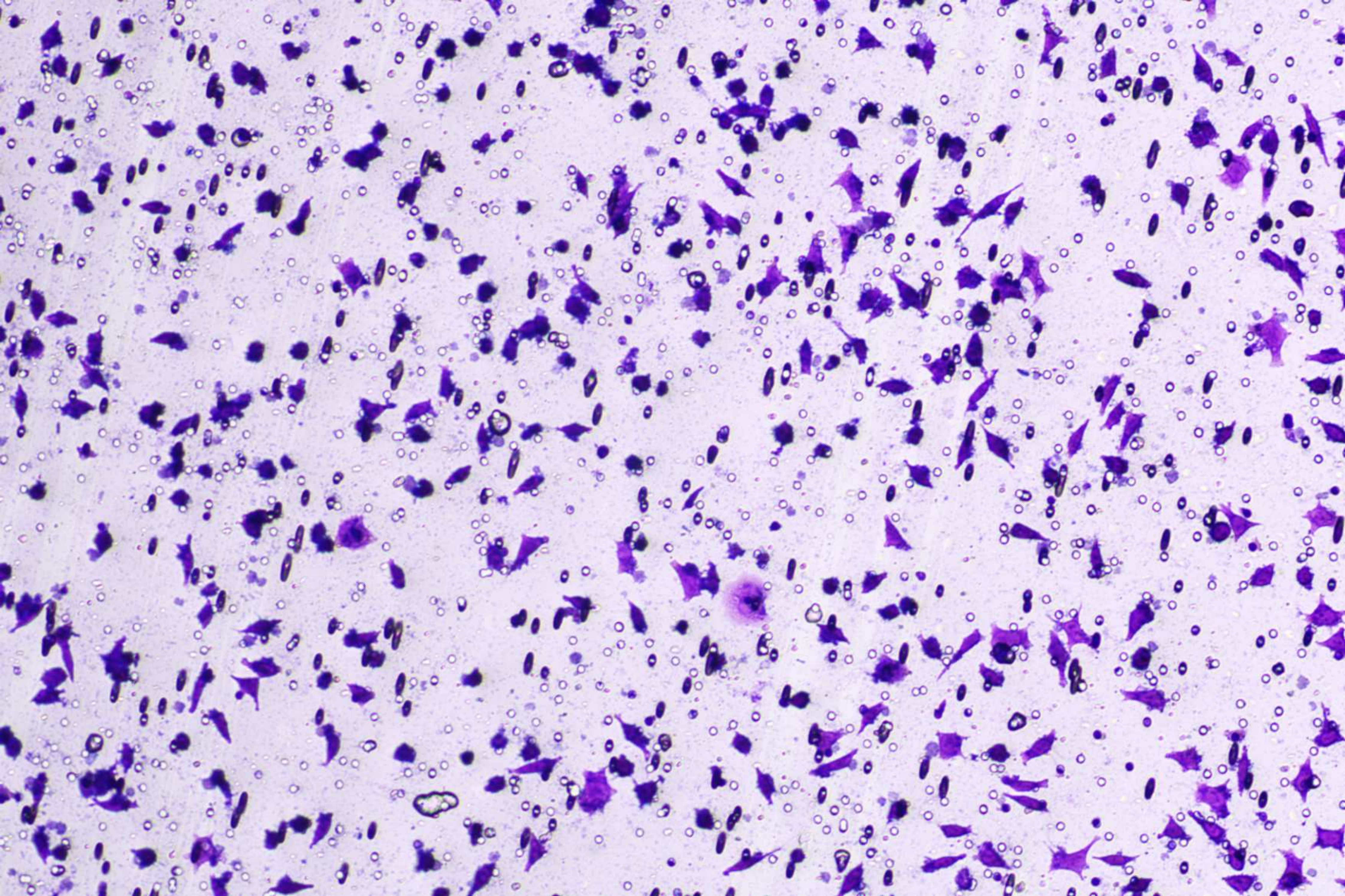

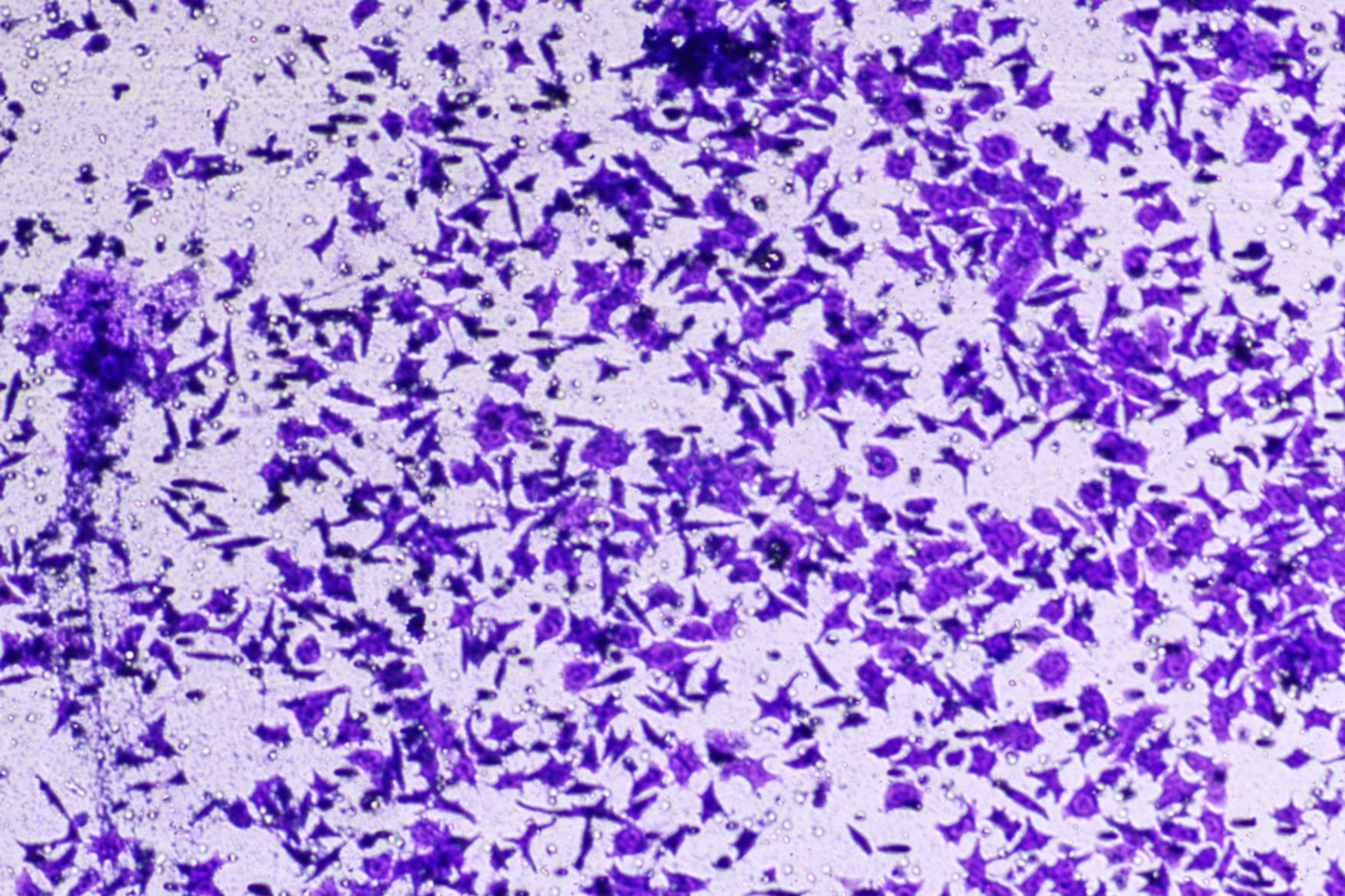

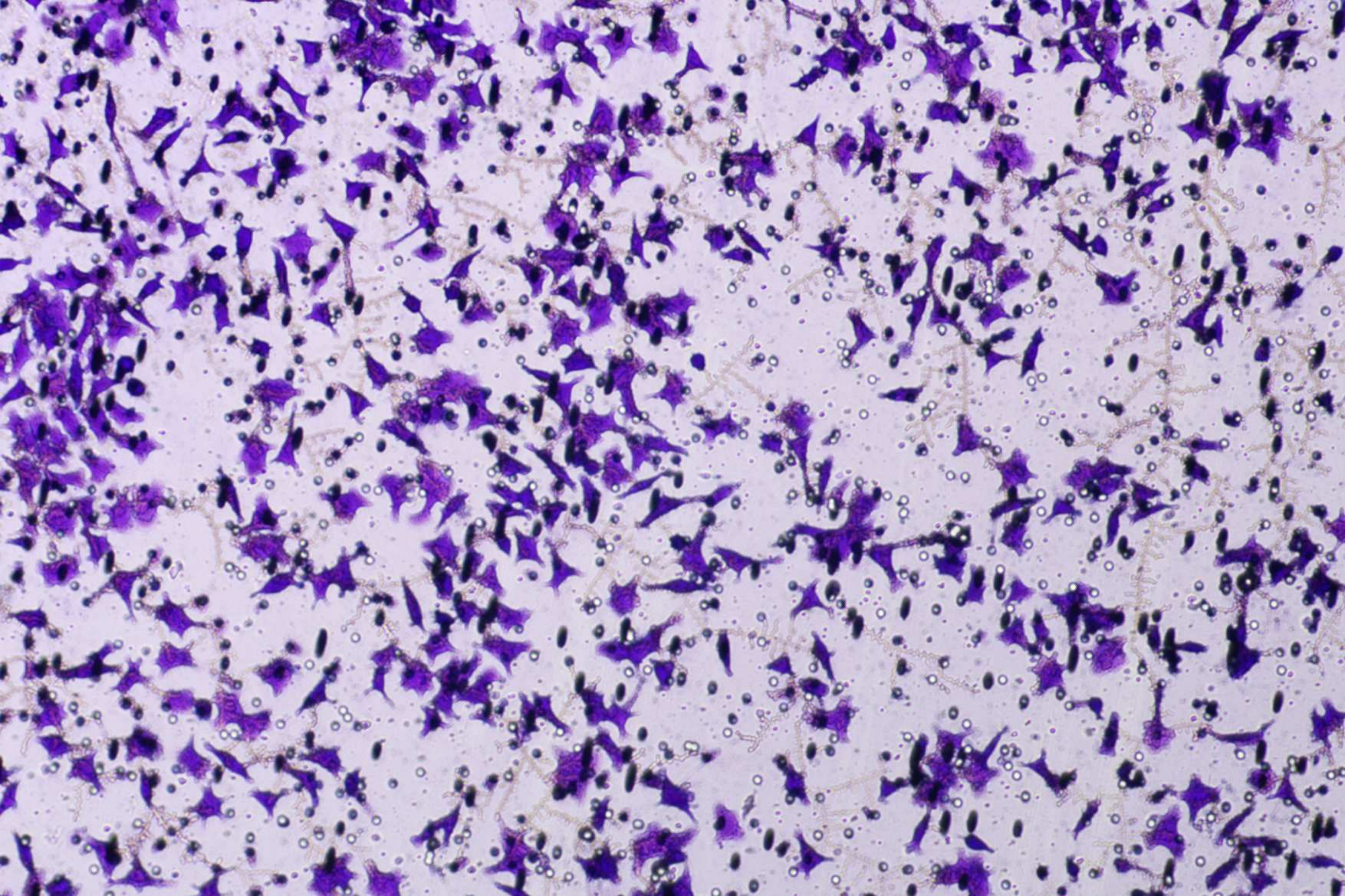

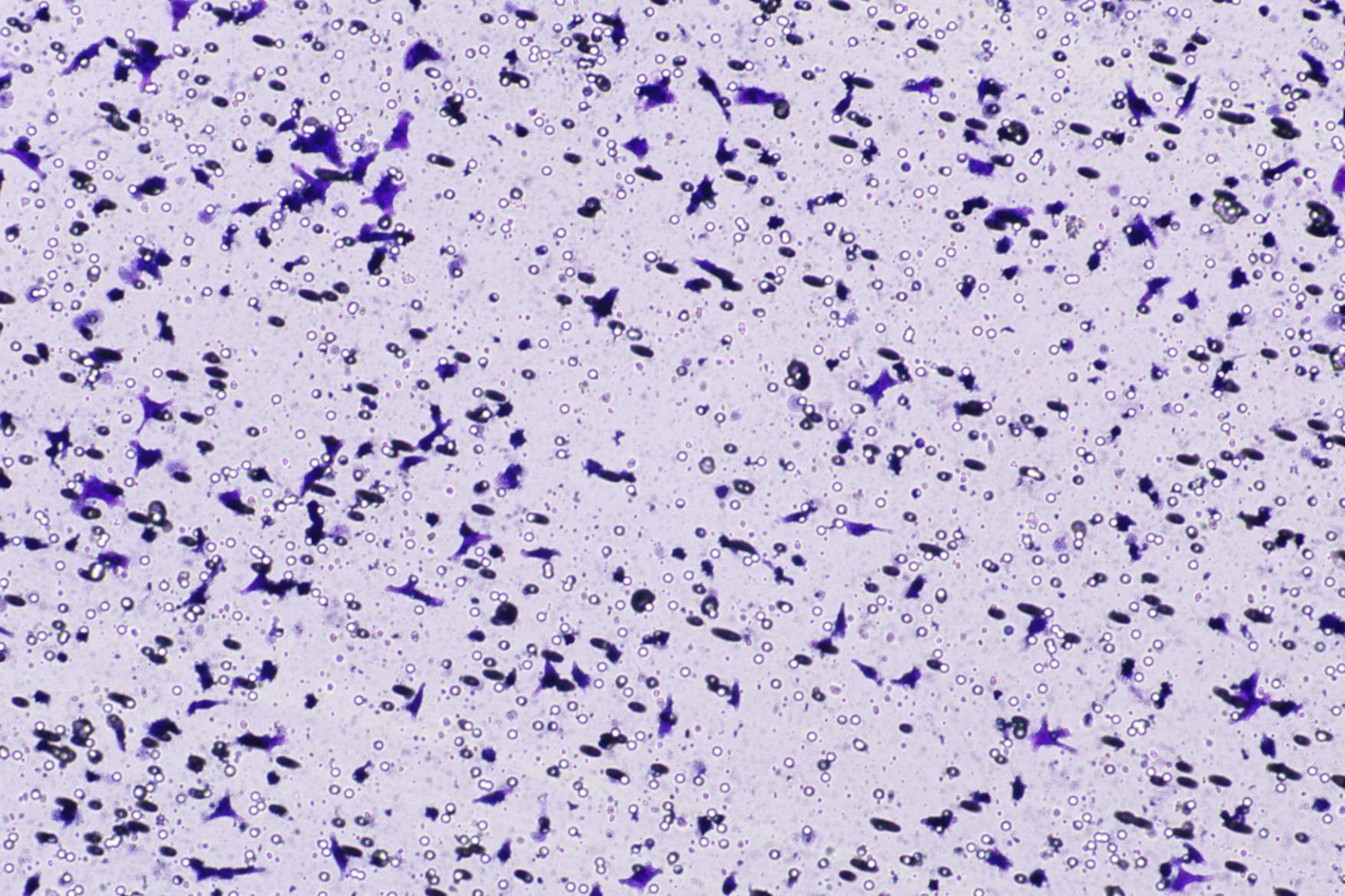

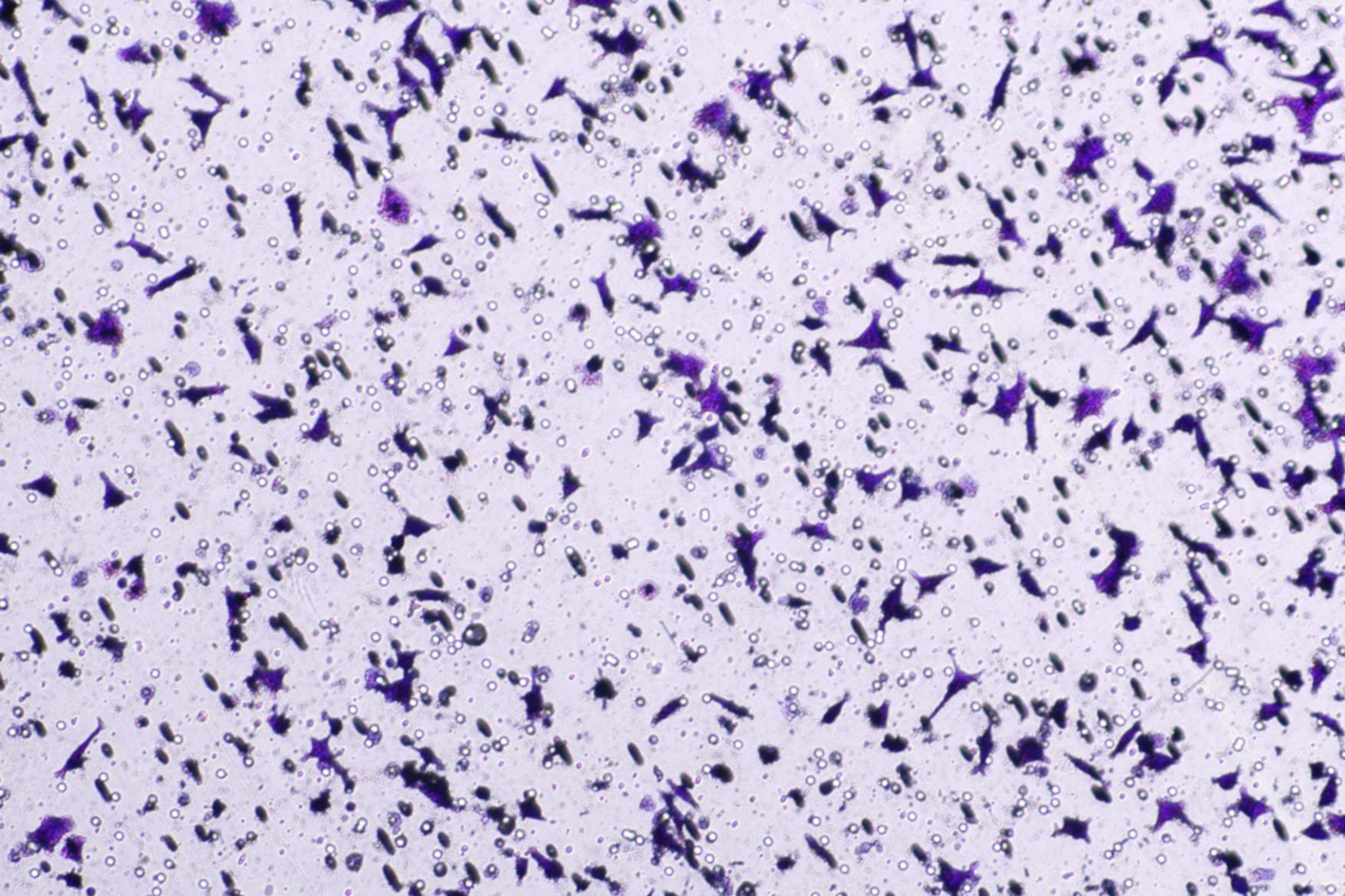

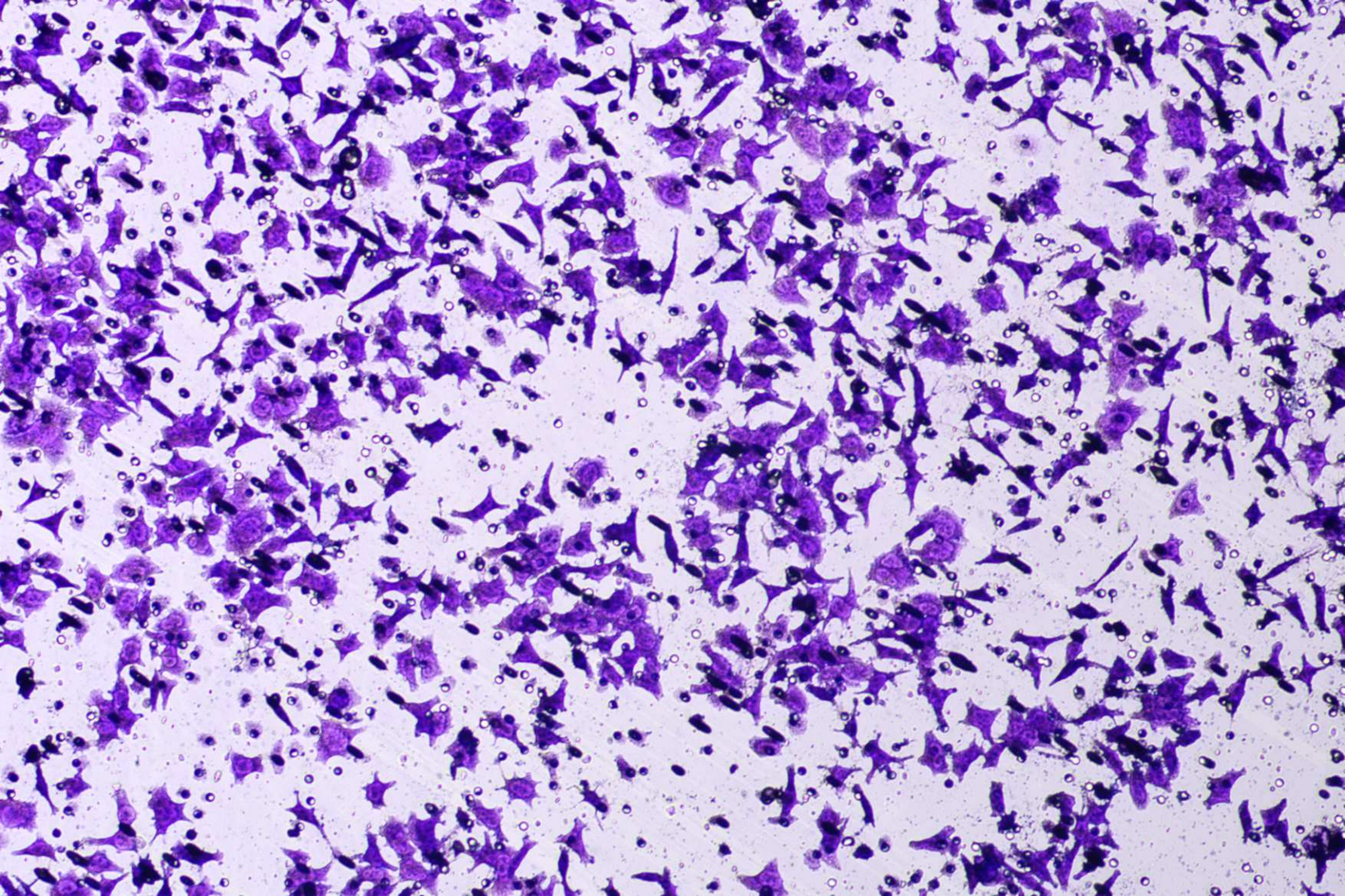

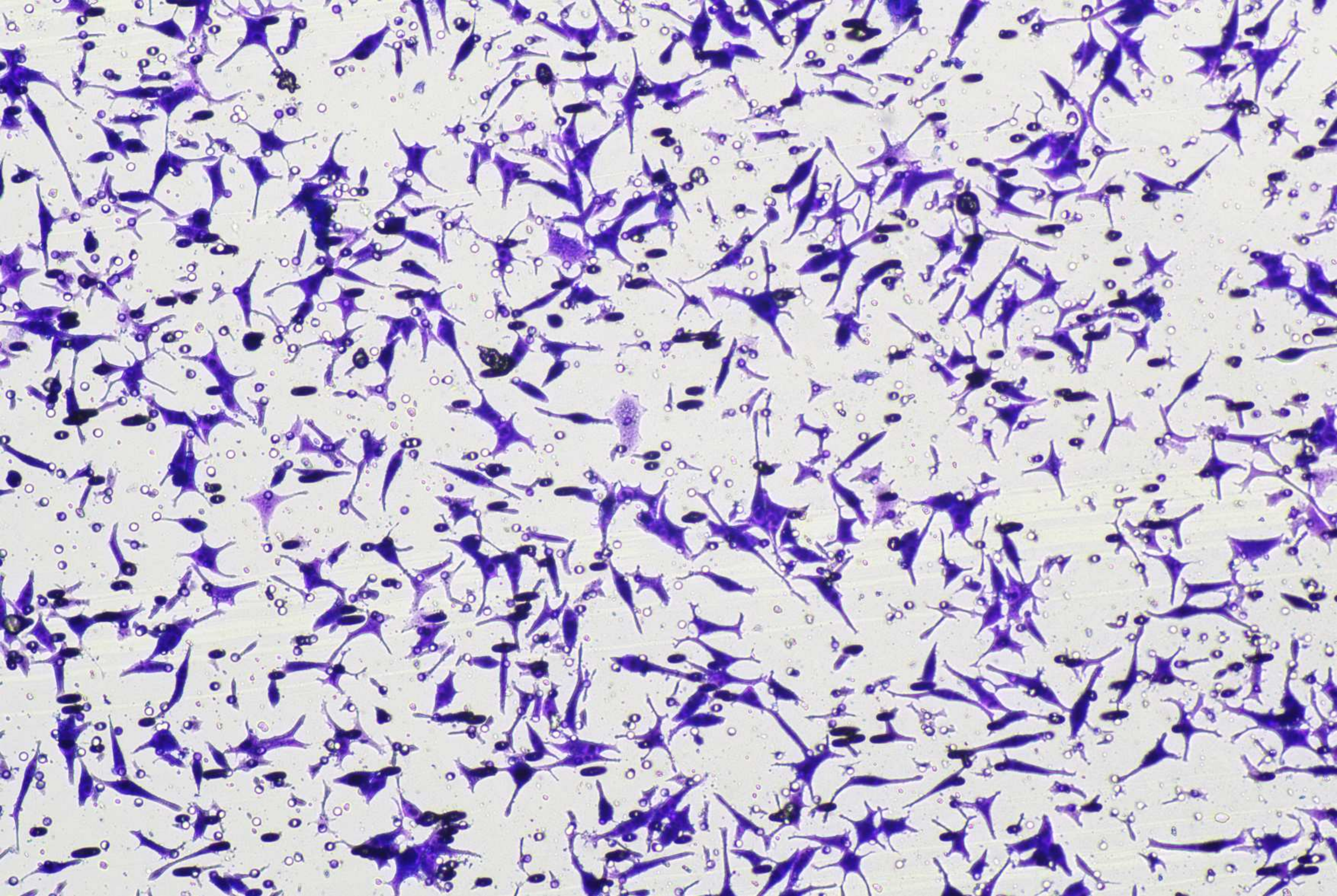

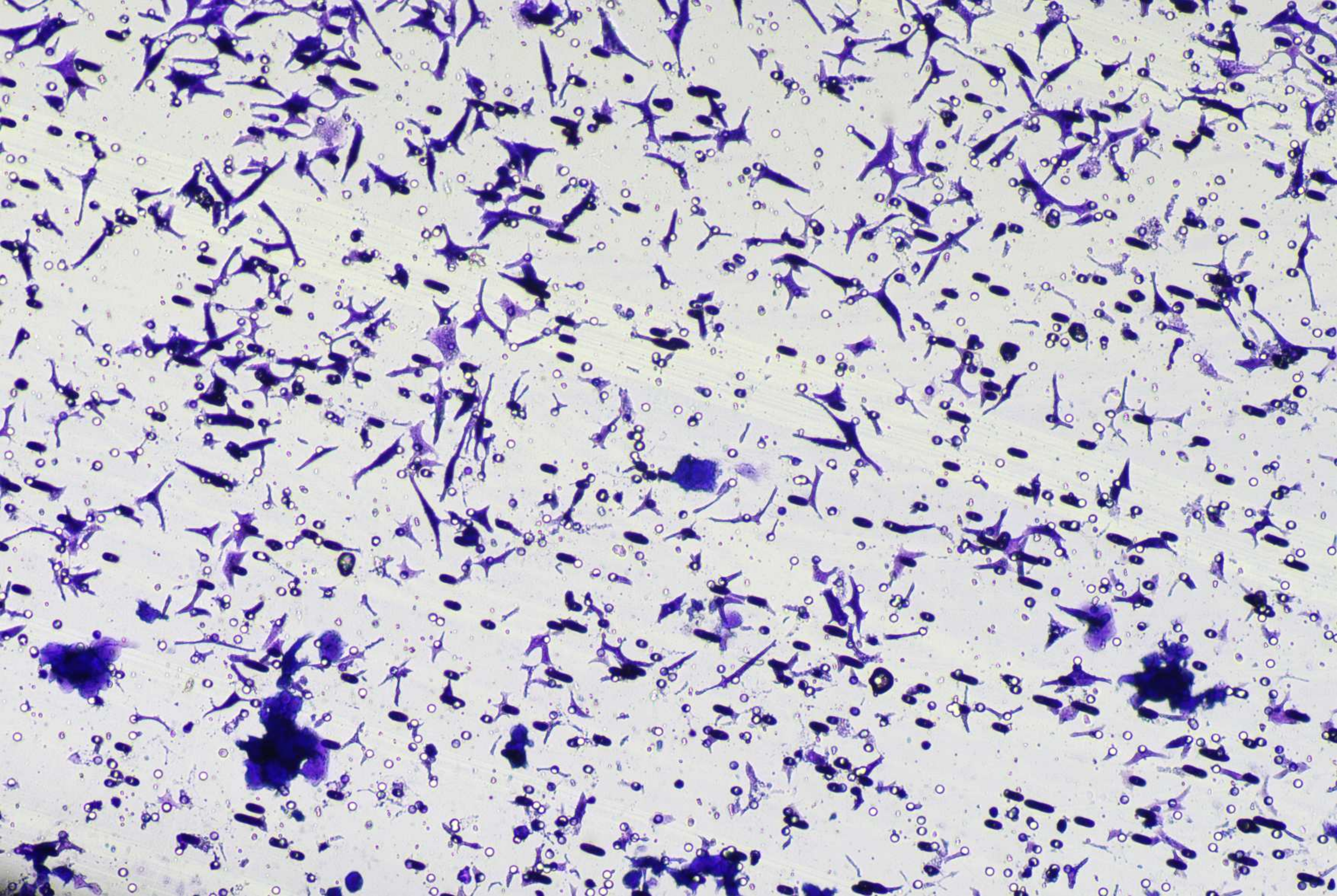

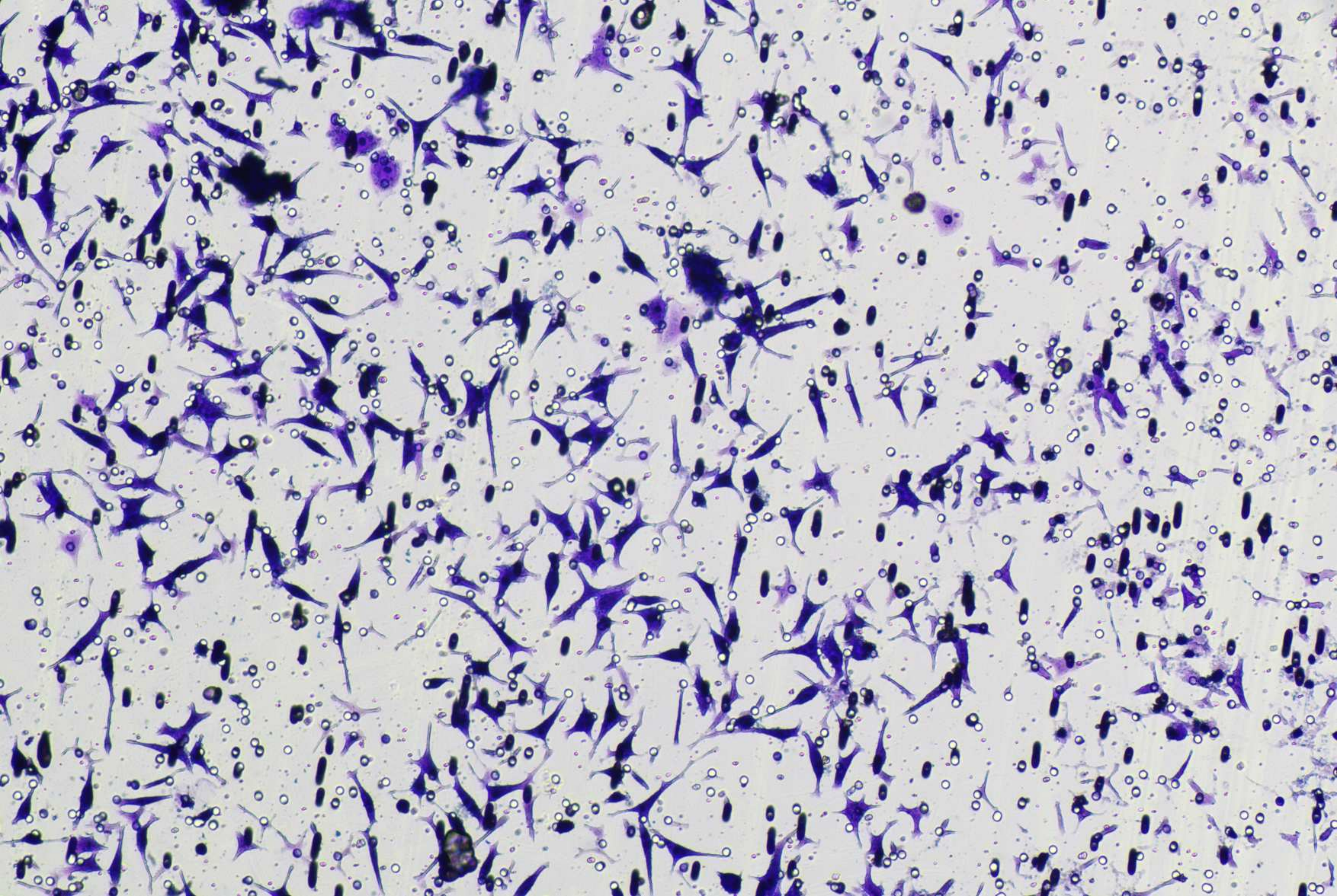

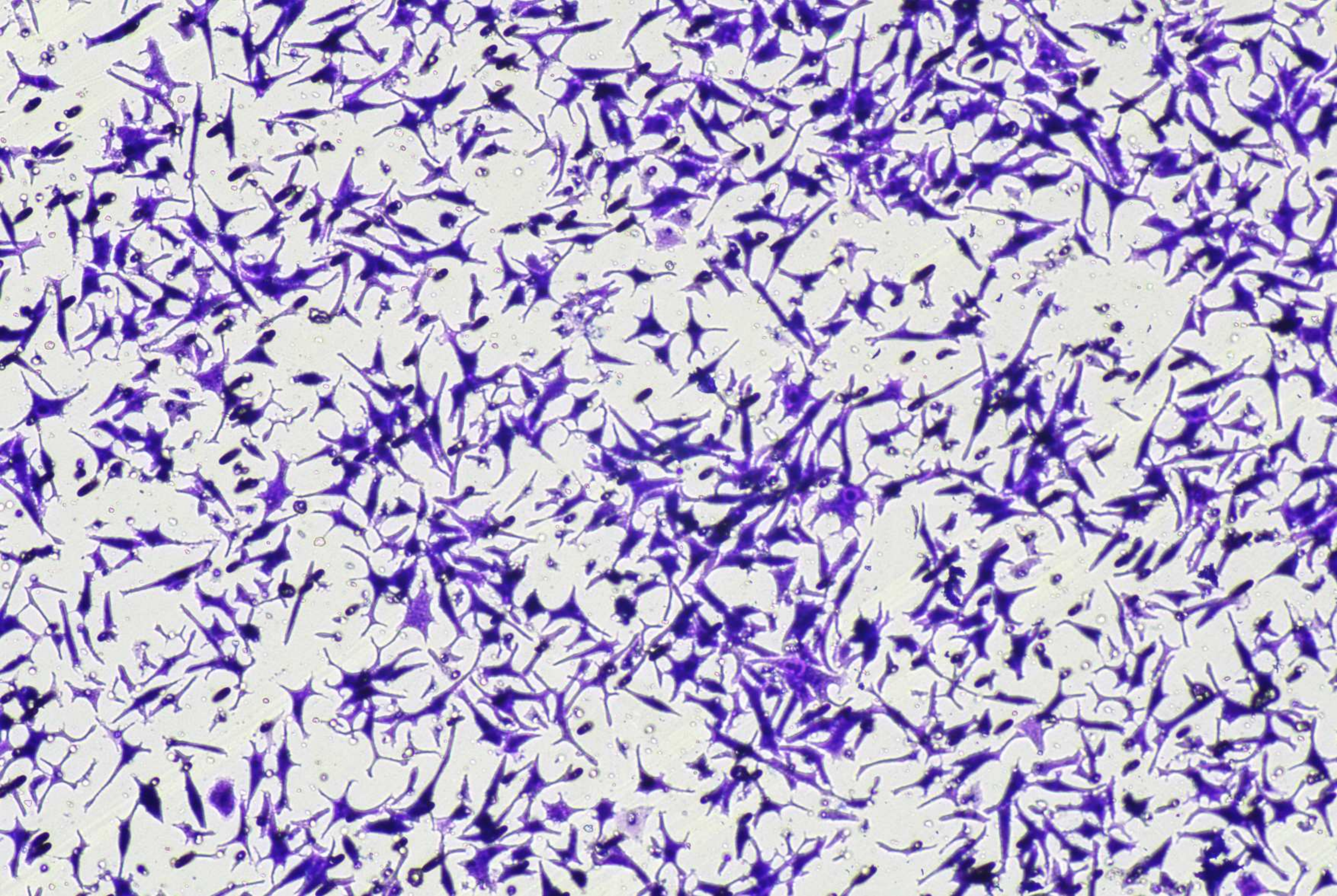

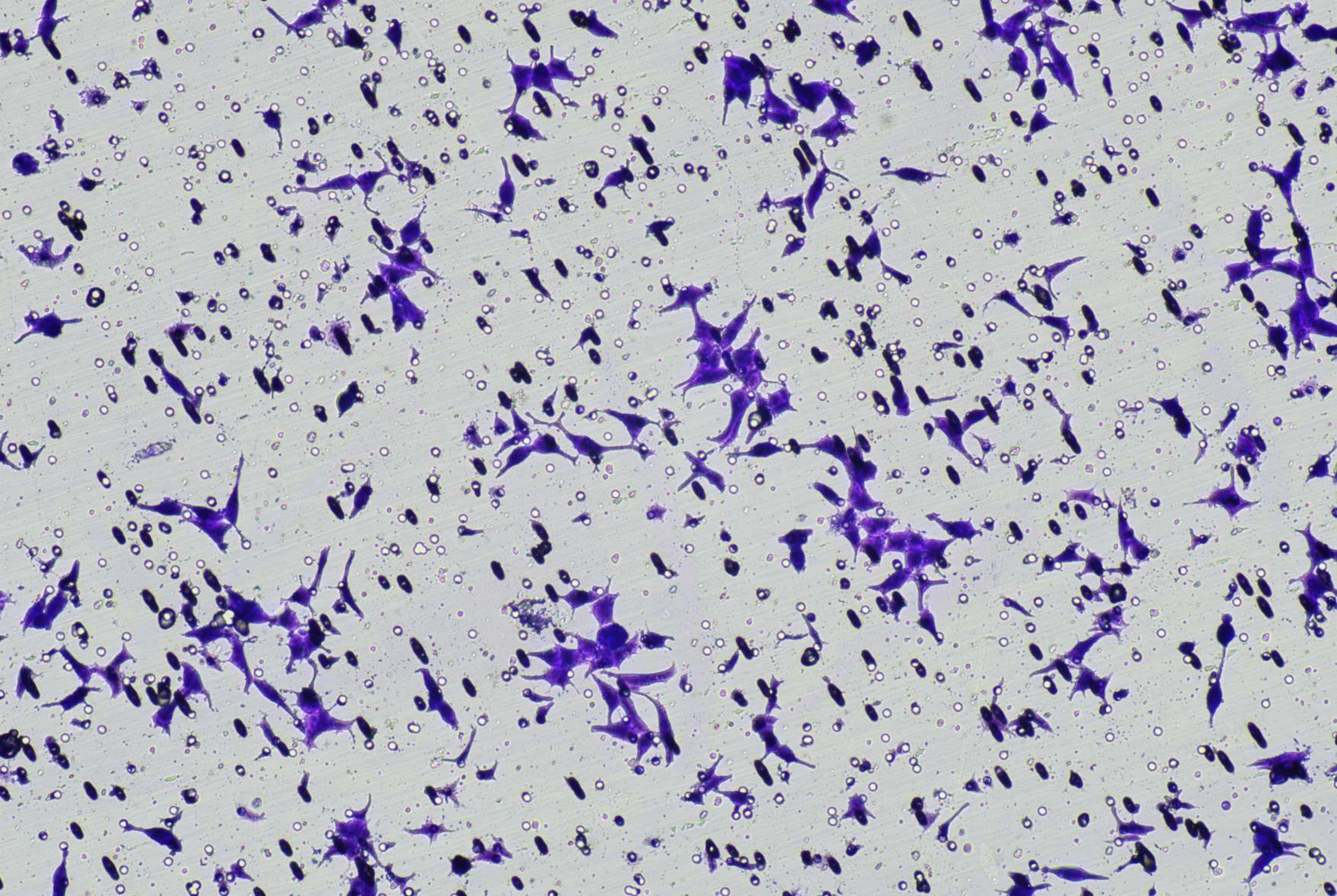

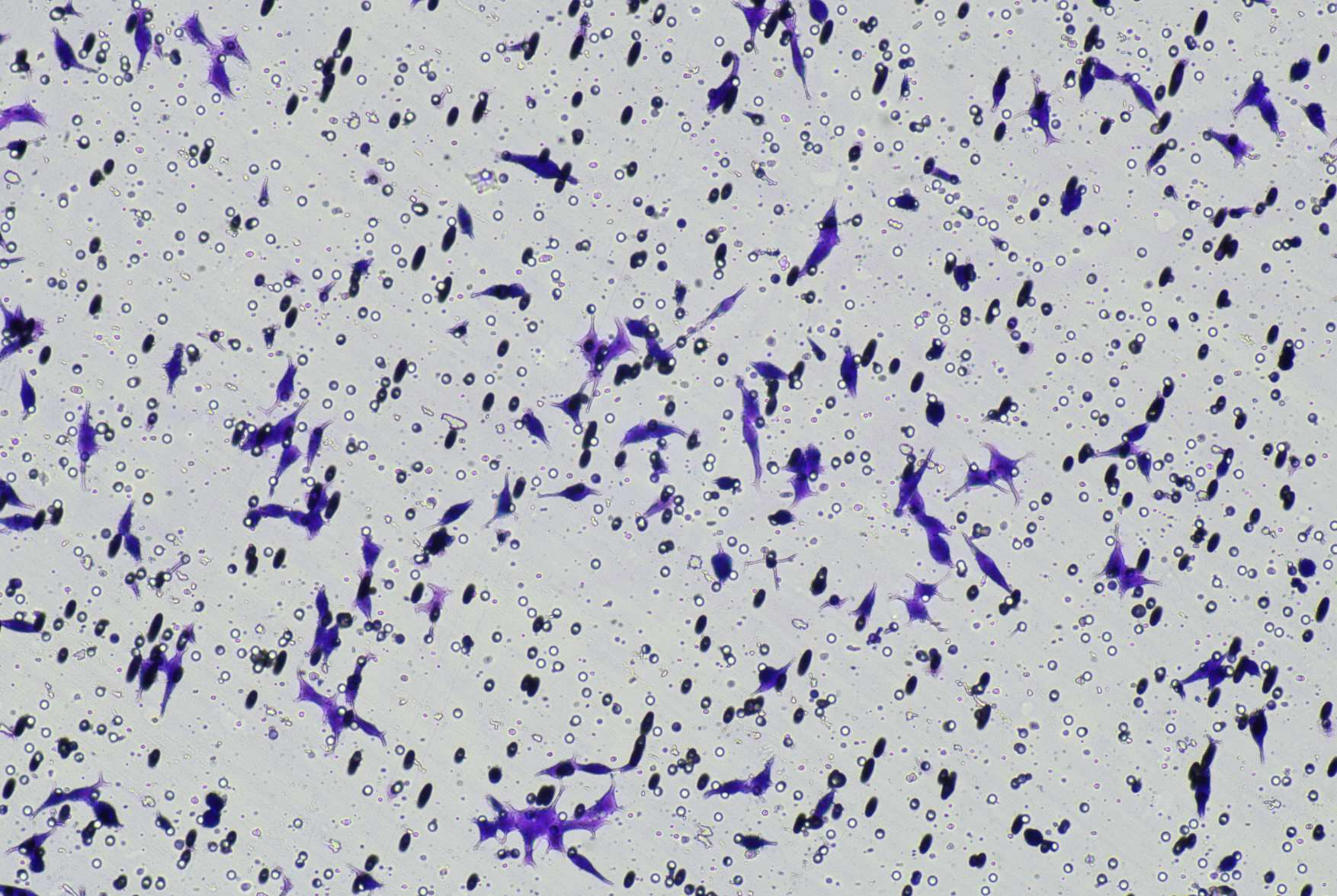

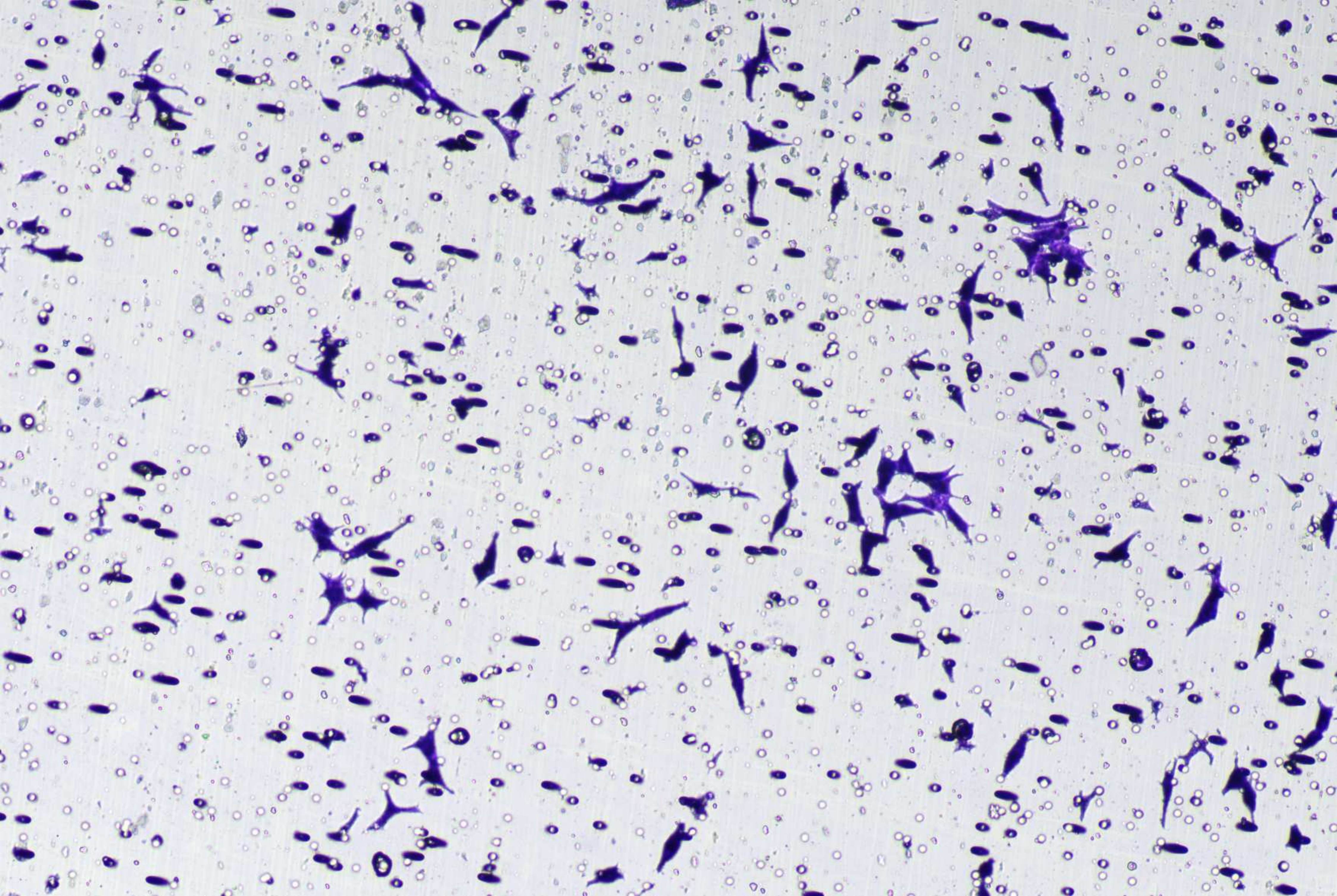

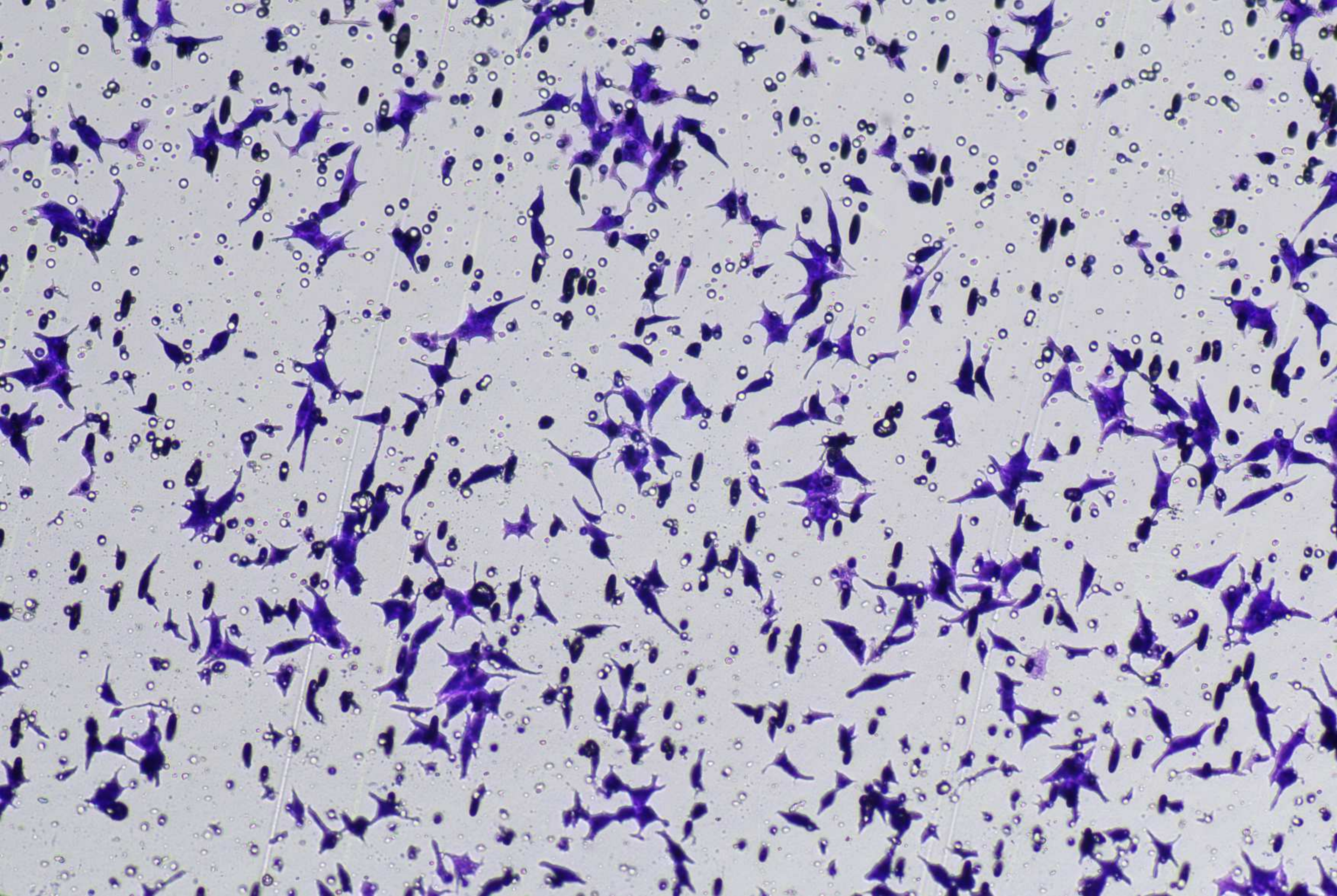

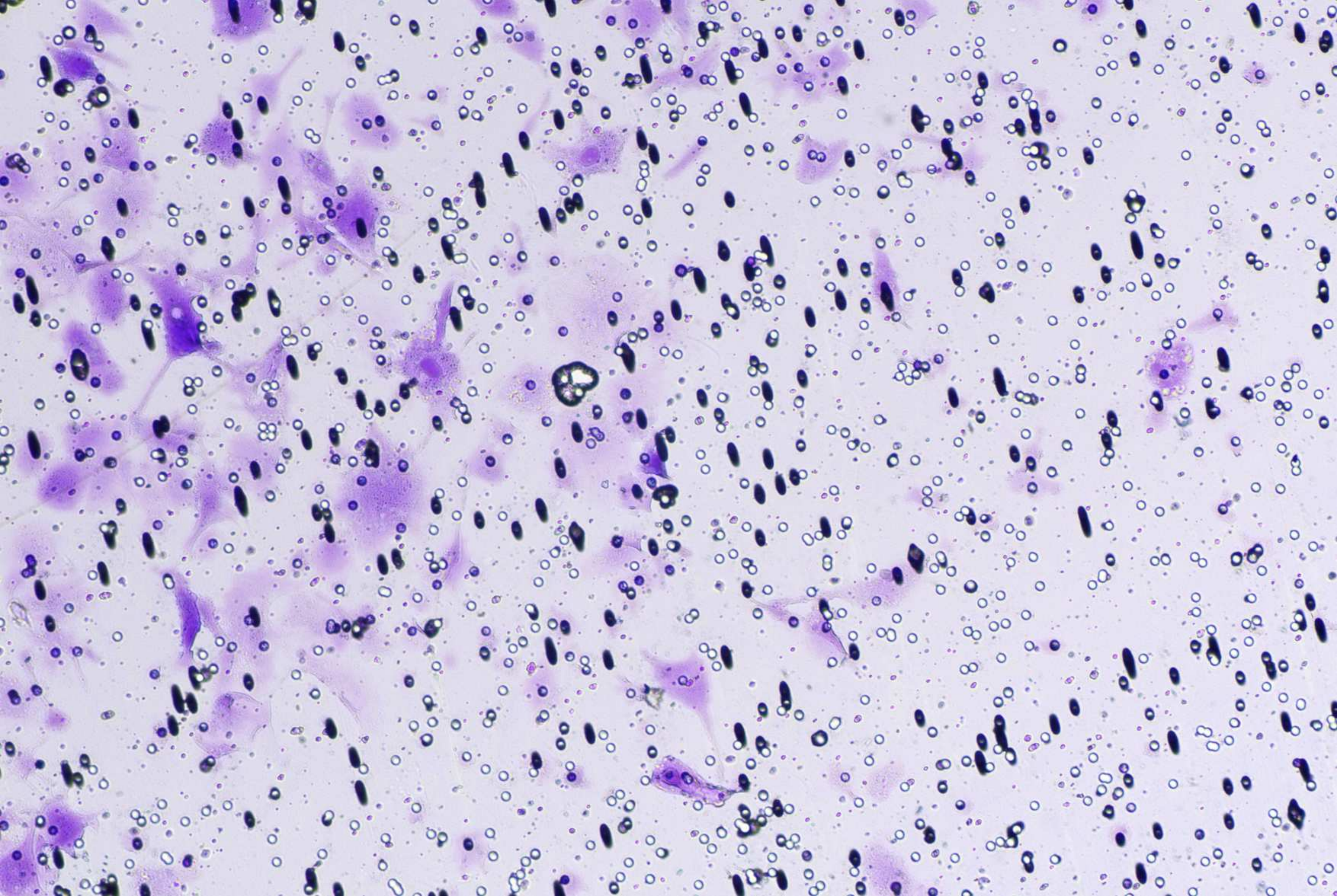

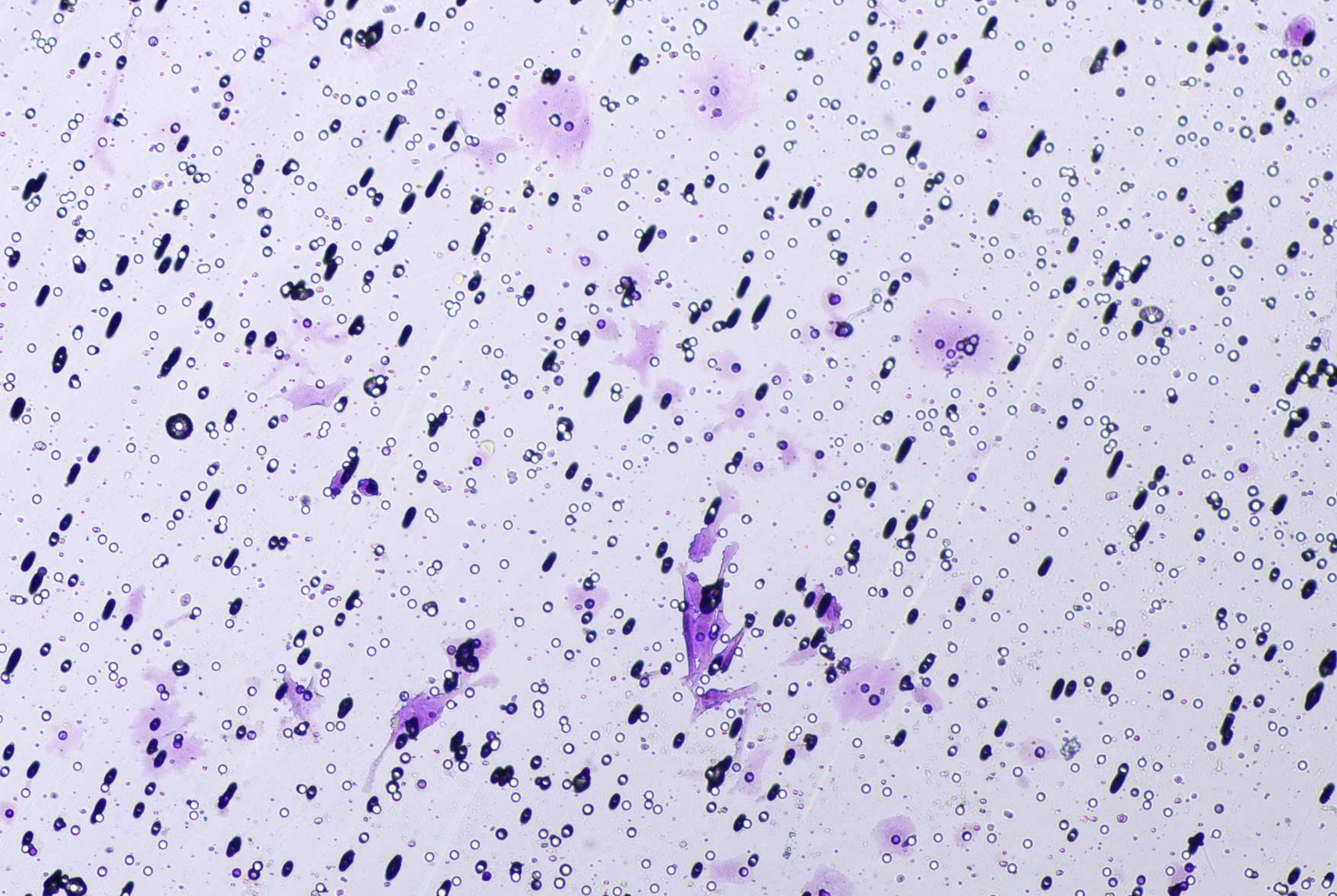

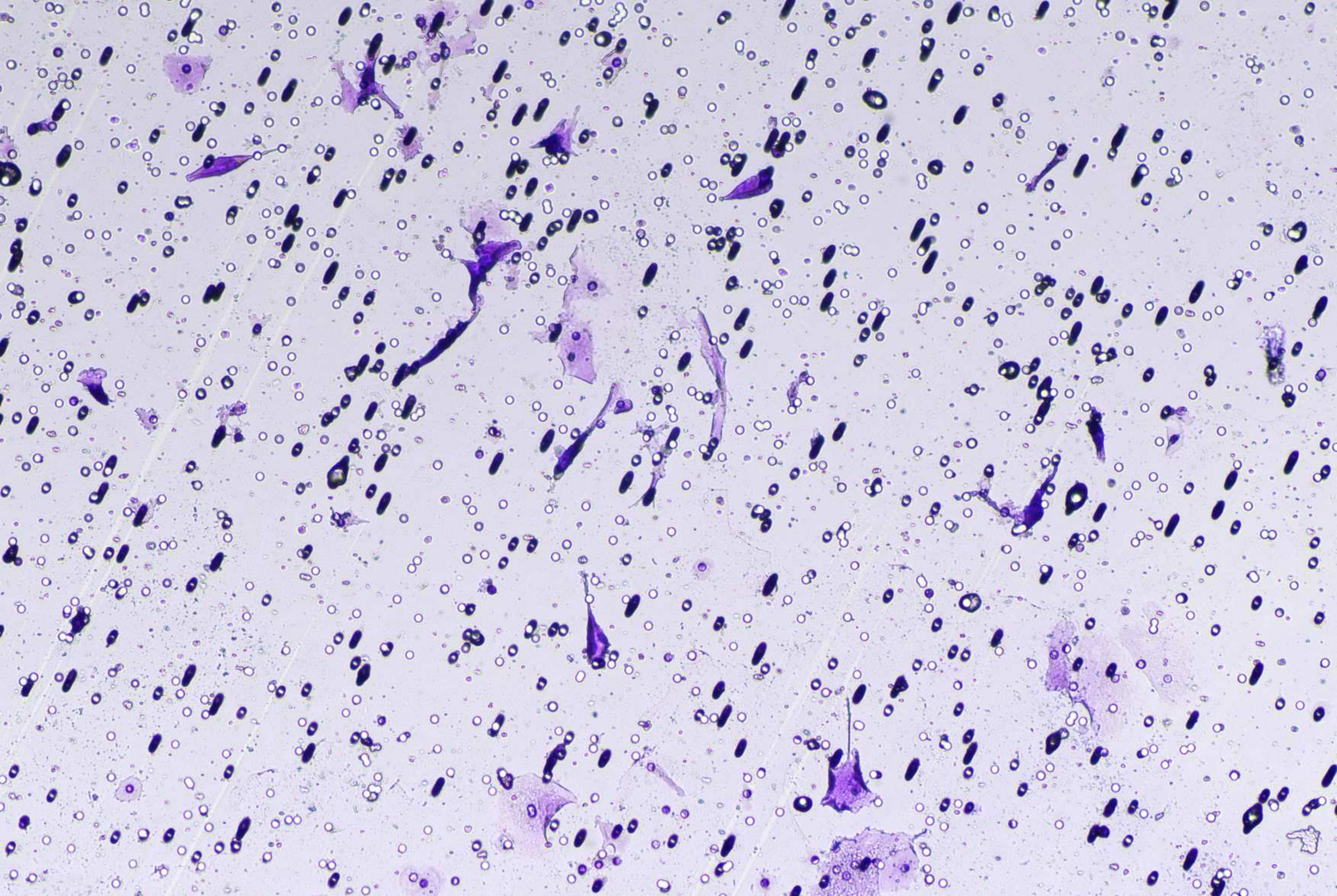

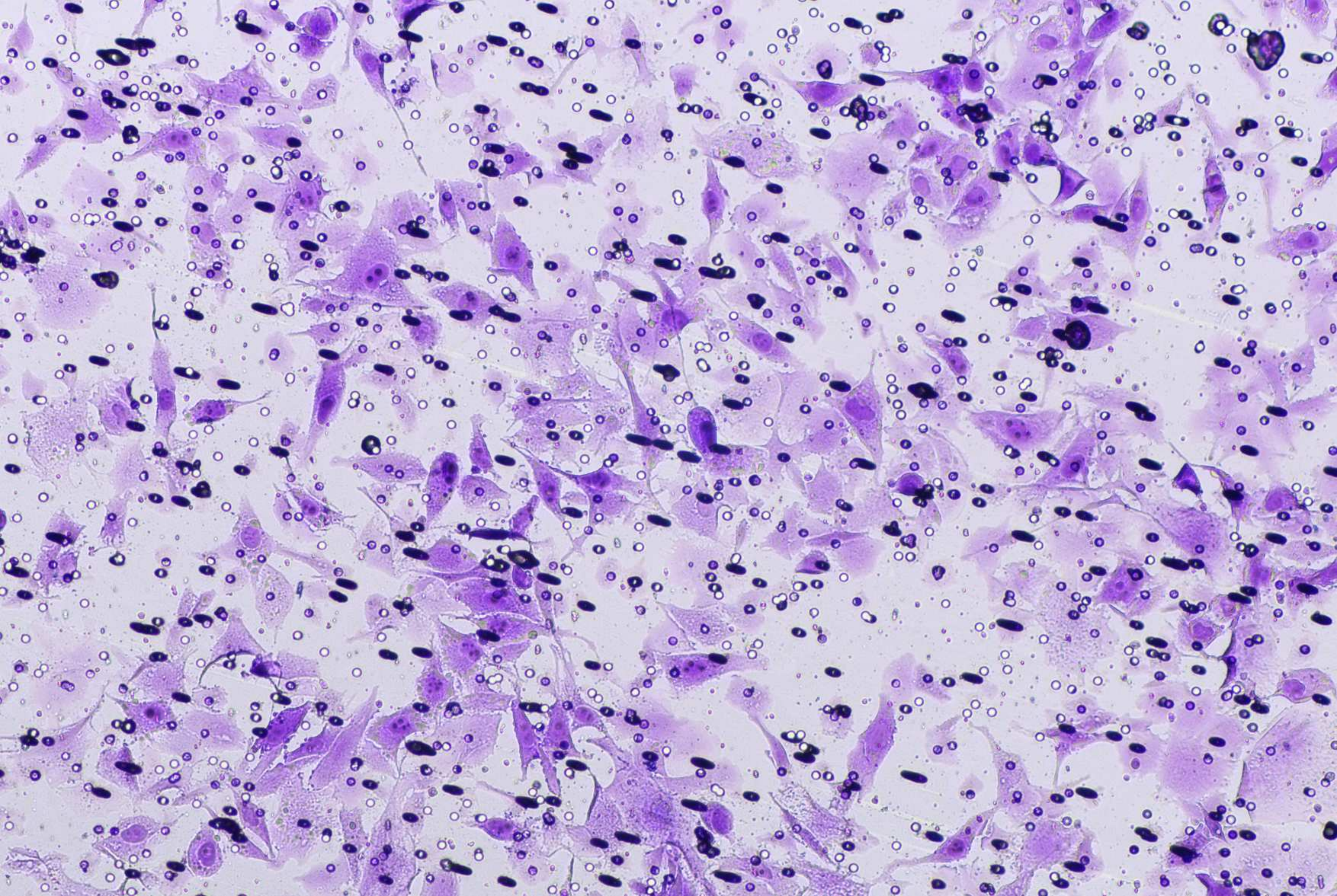

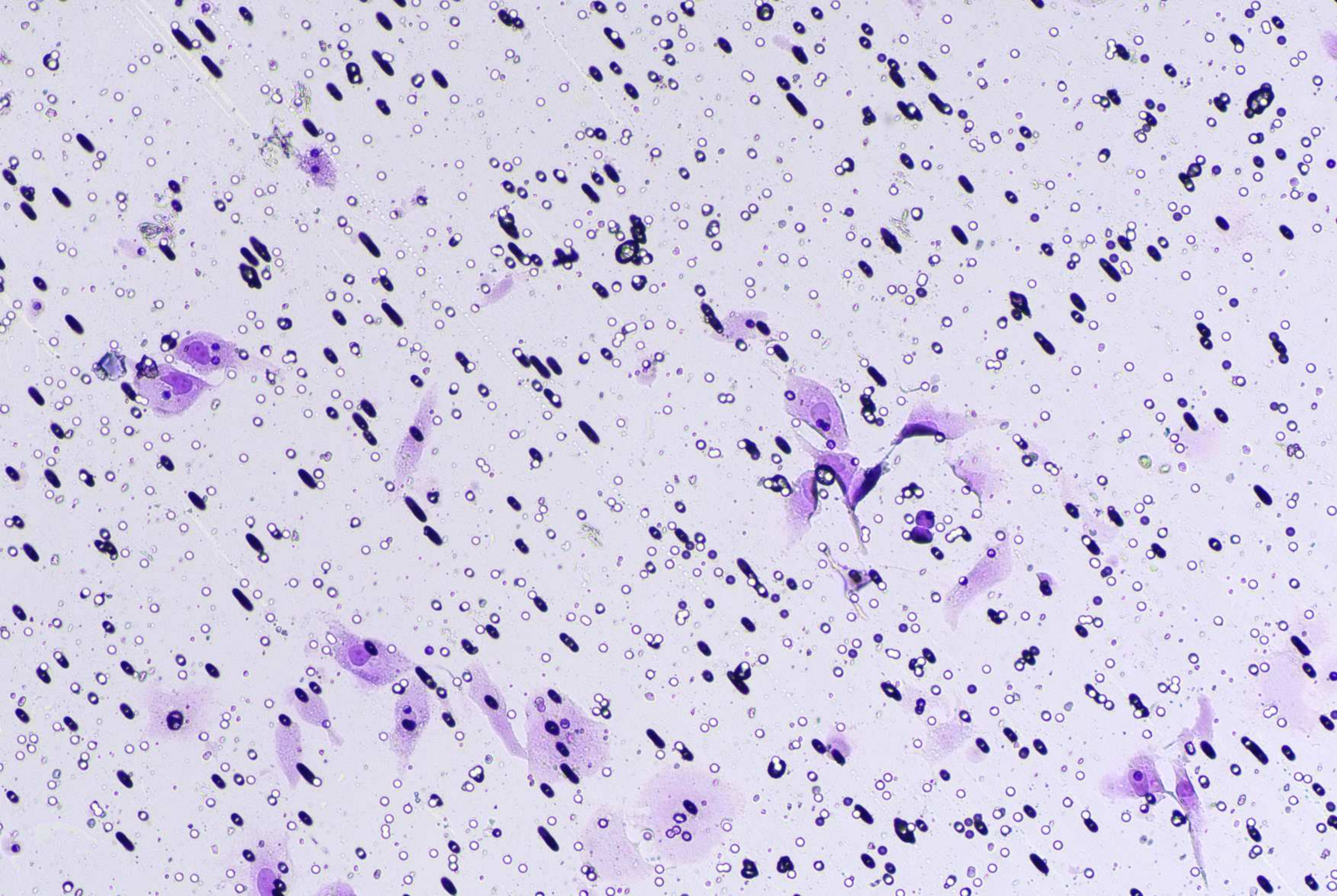

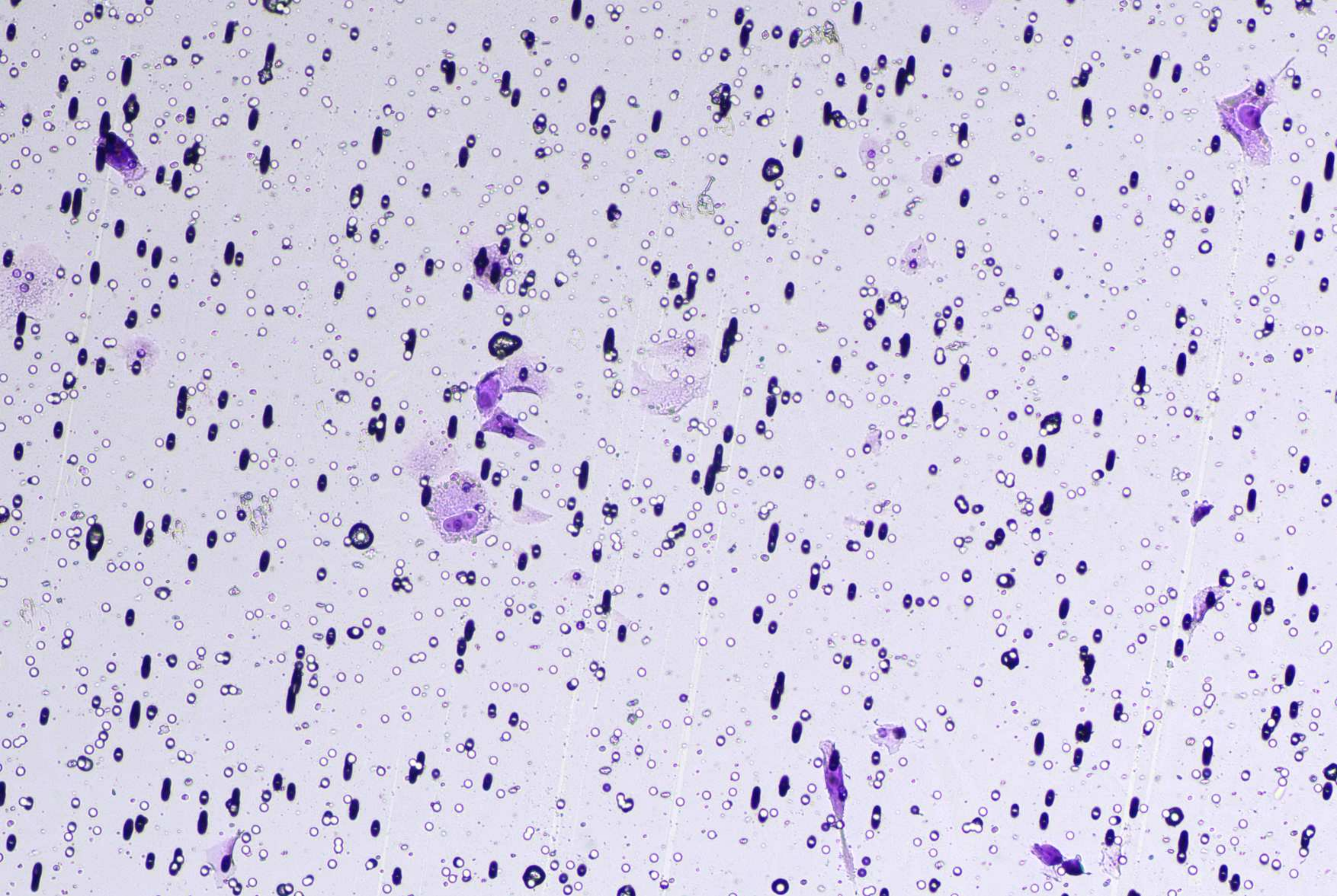

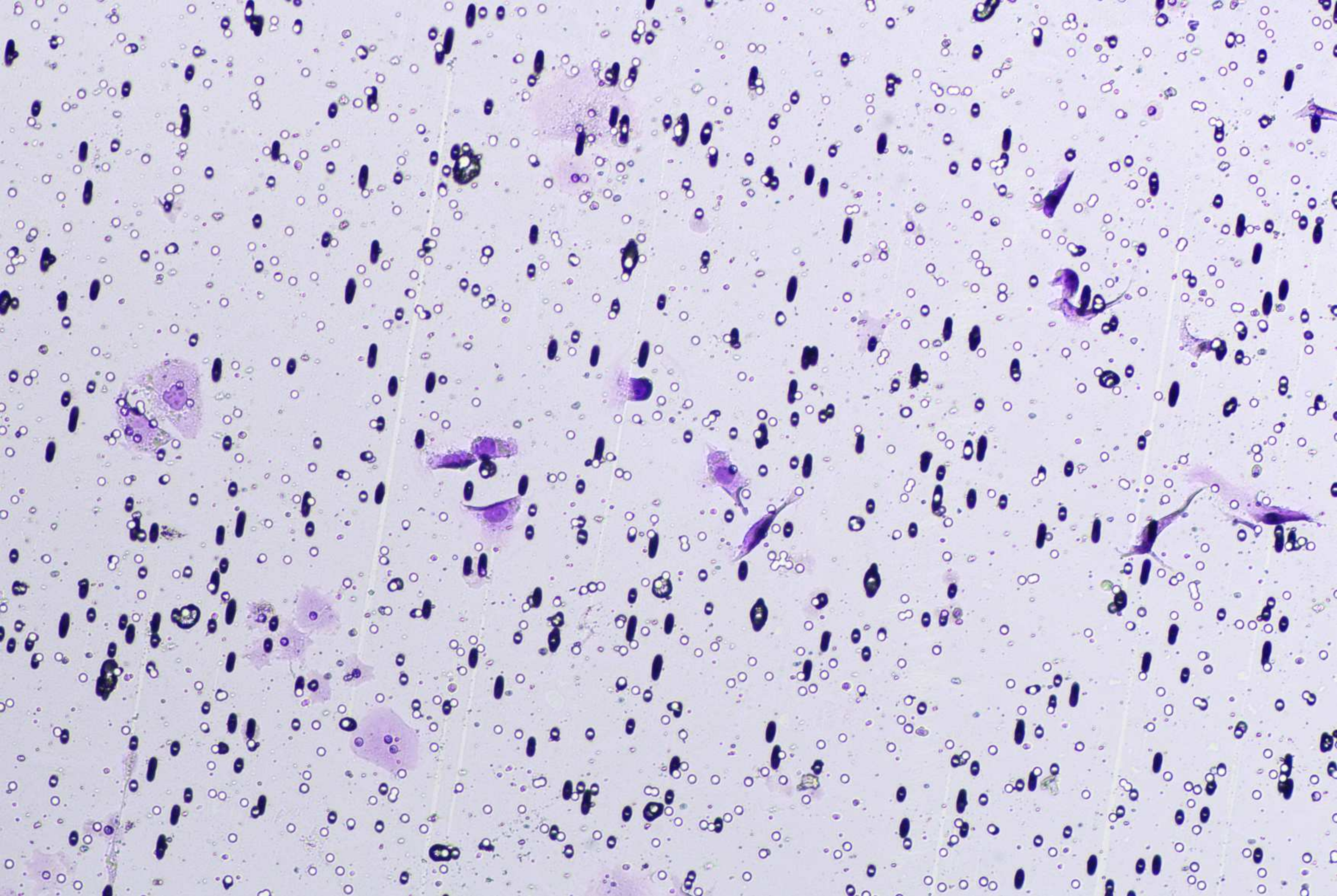

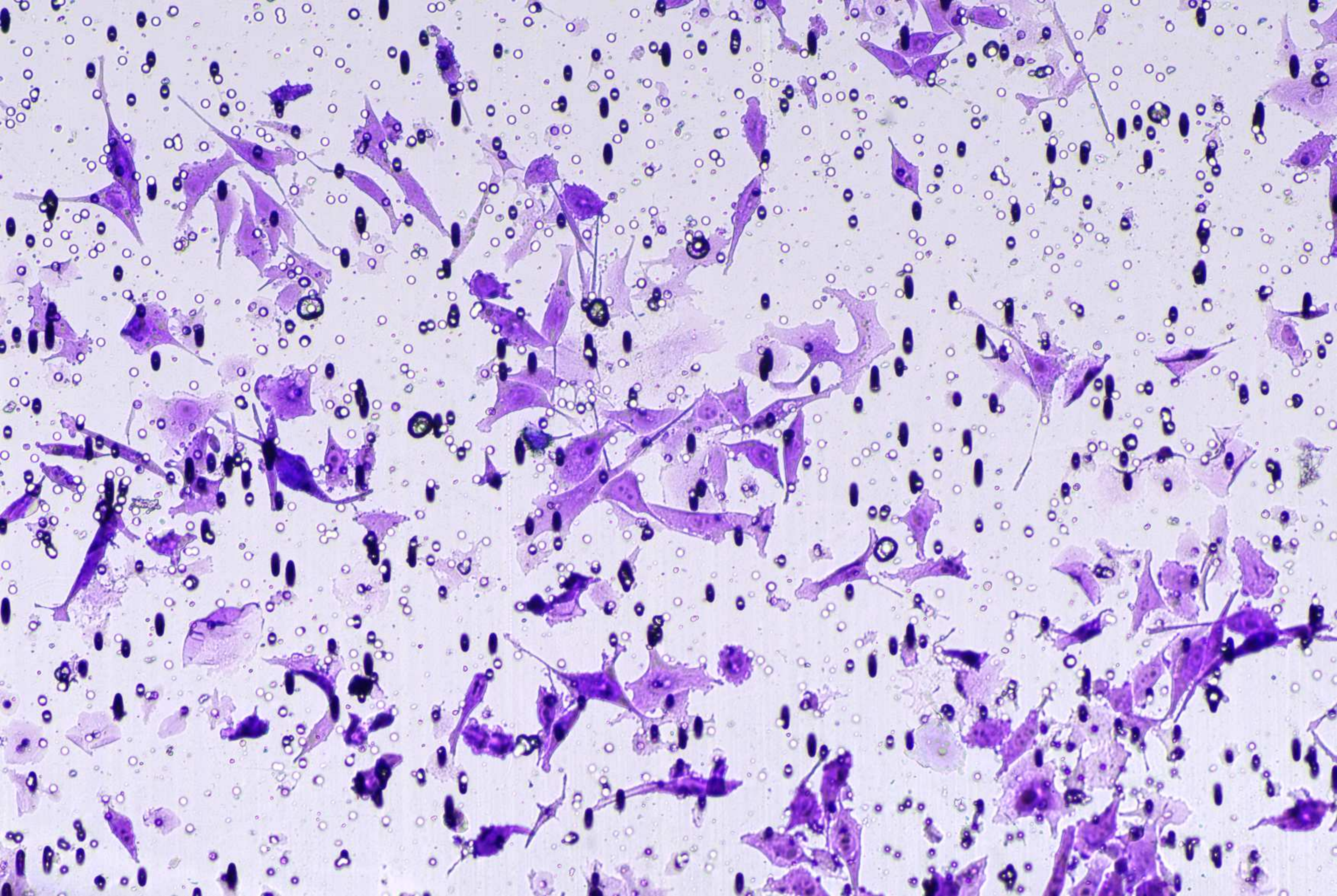

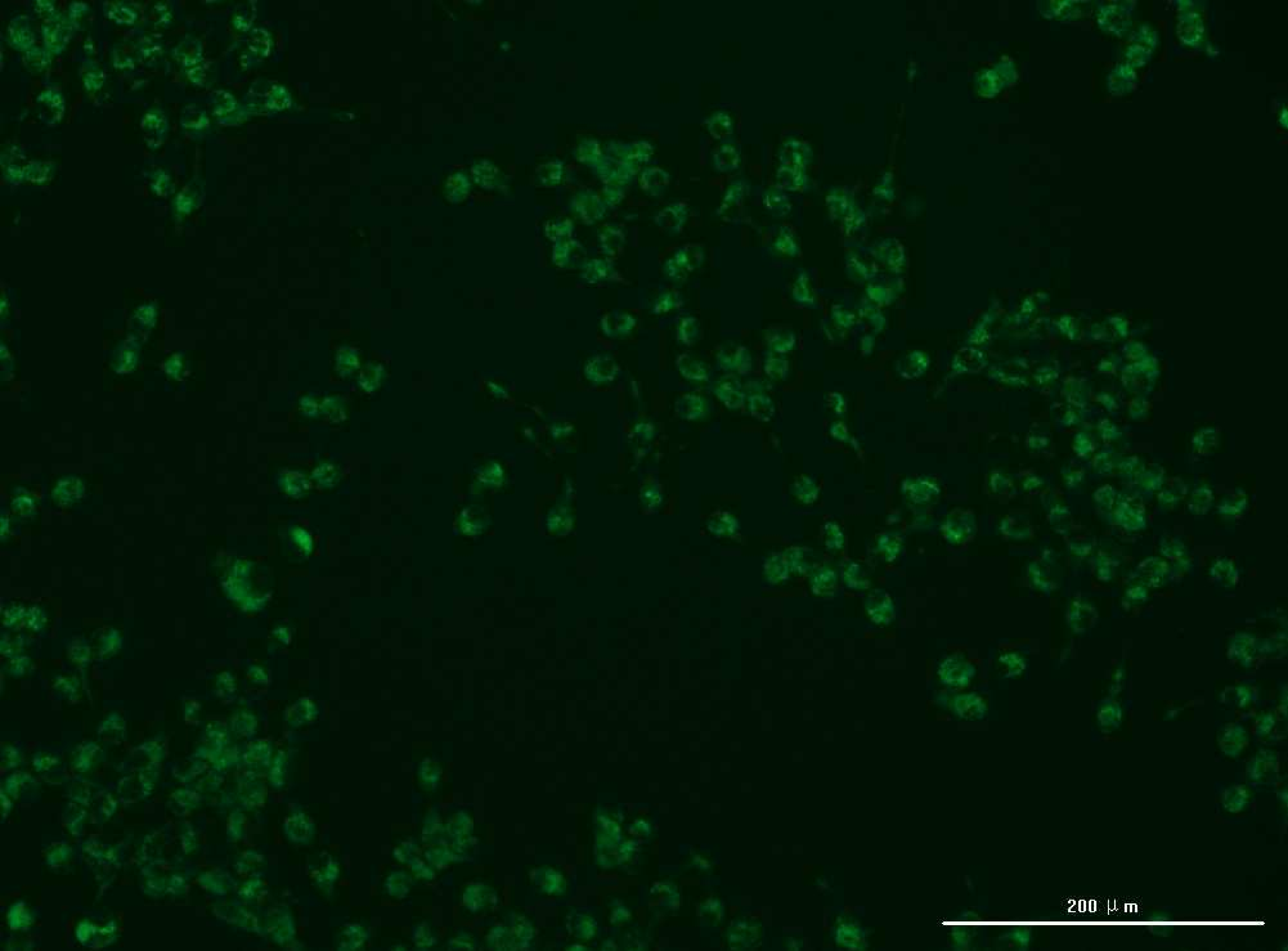
200 μm

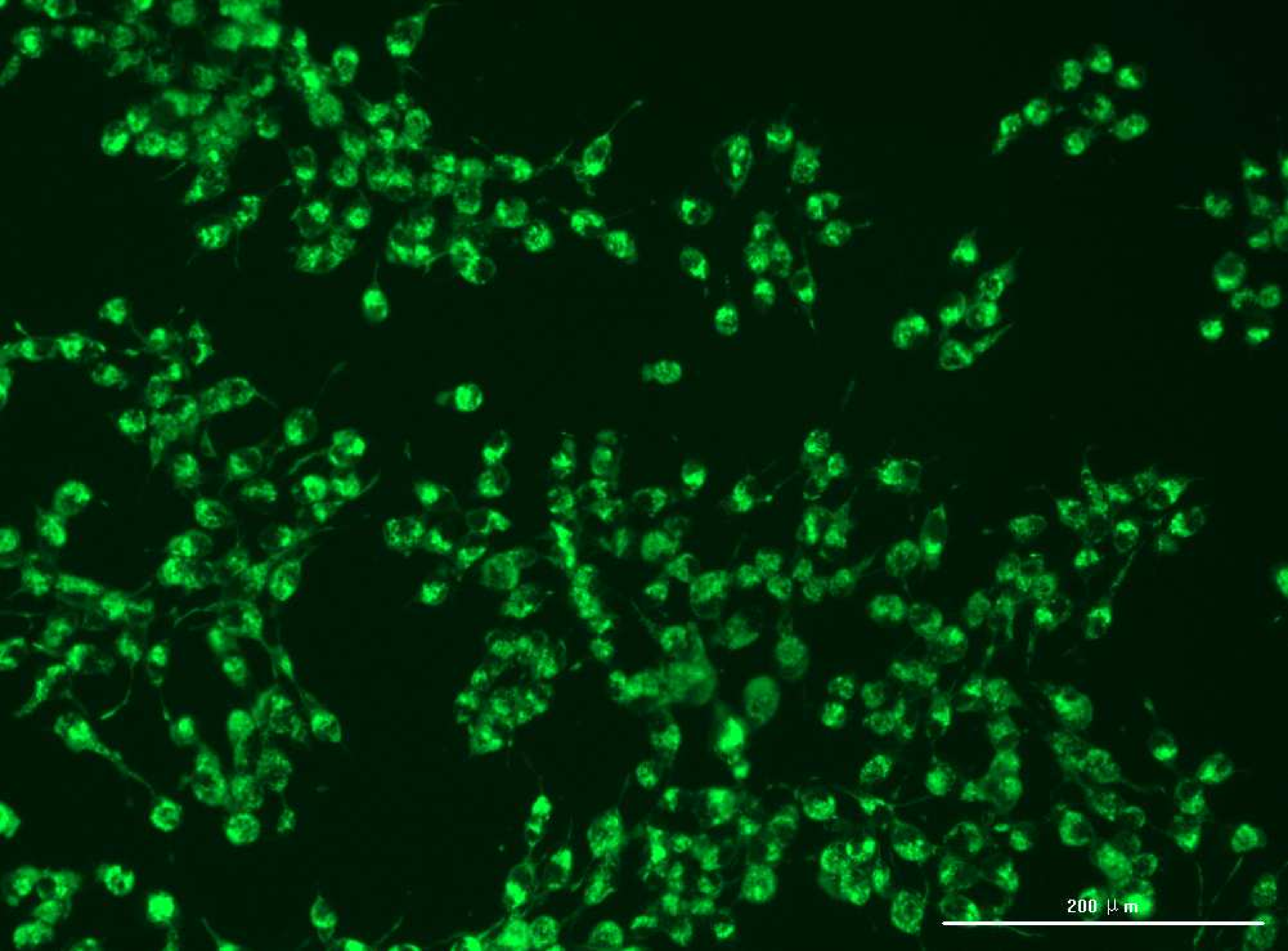
200 μm

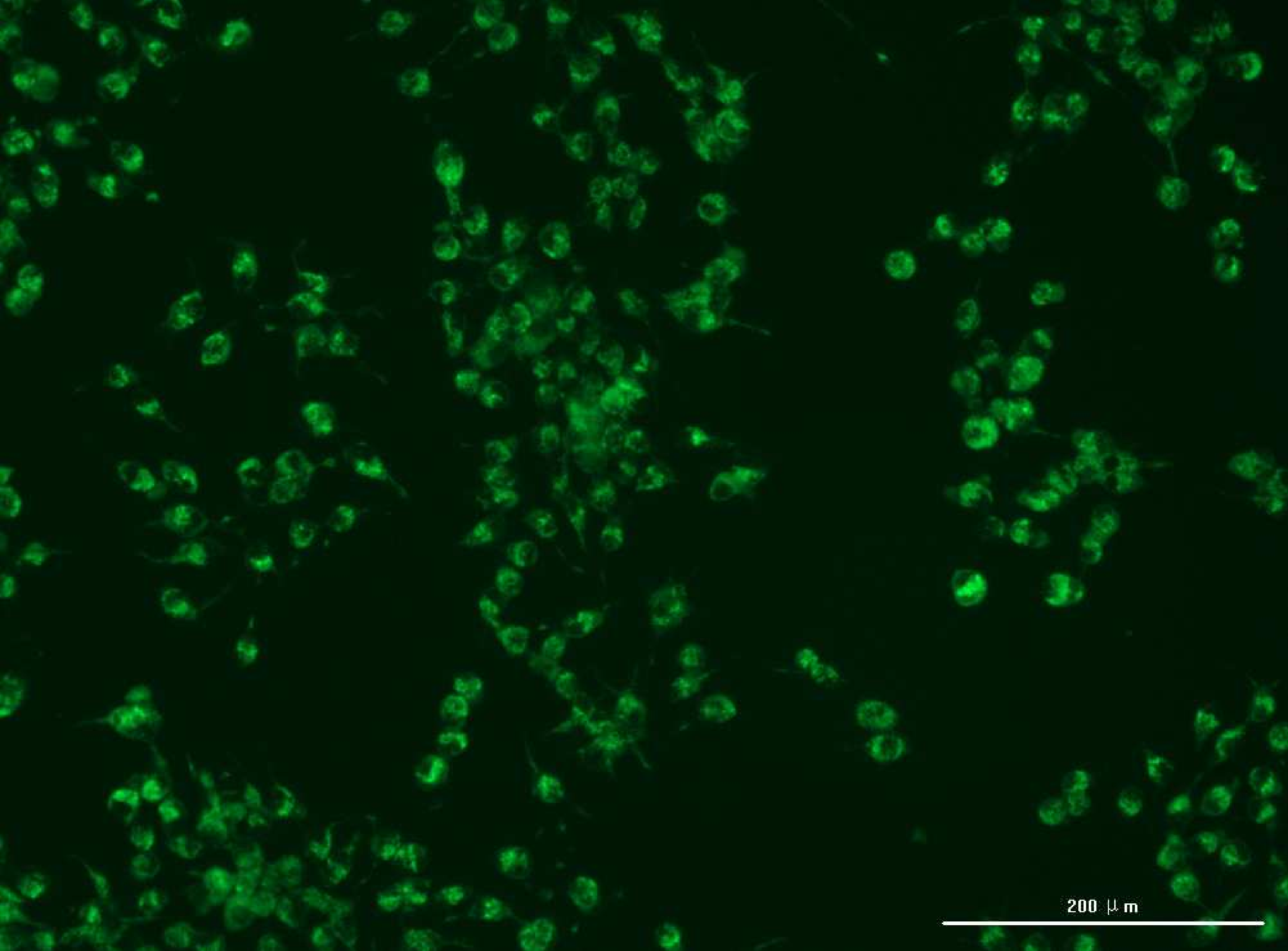
200 μm

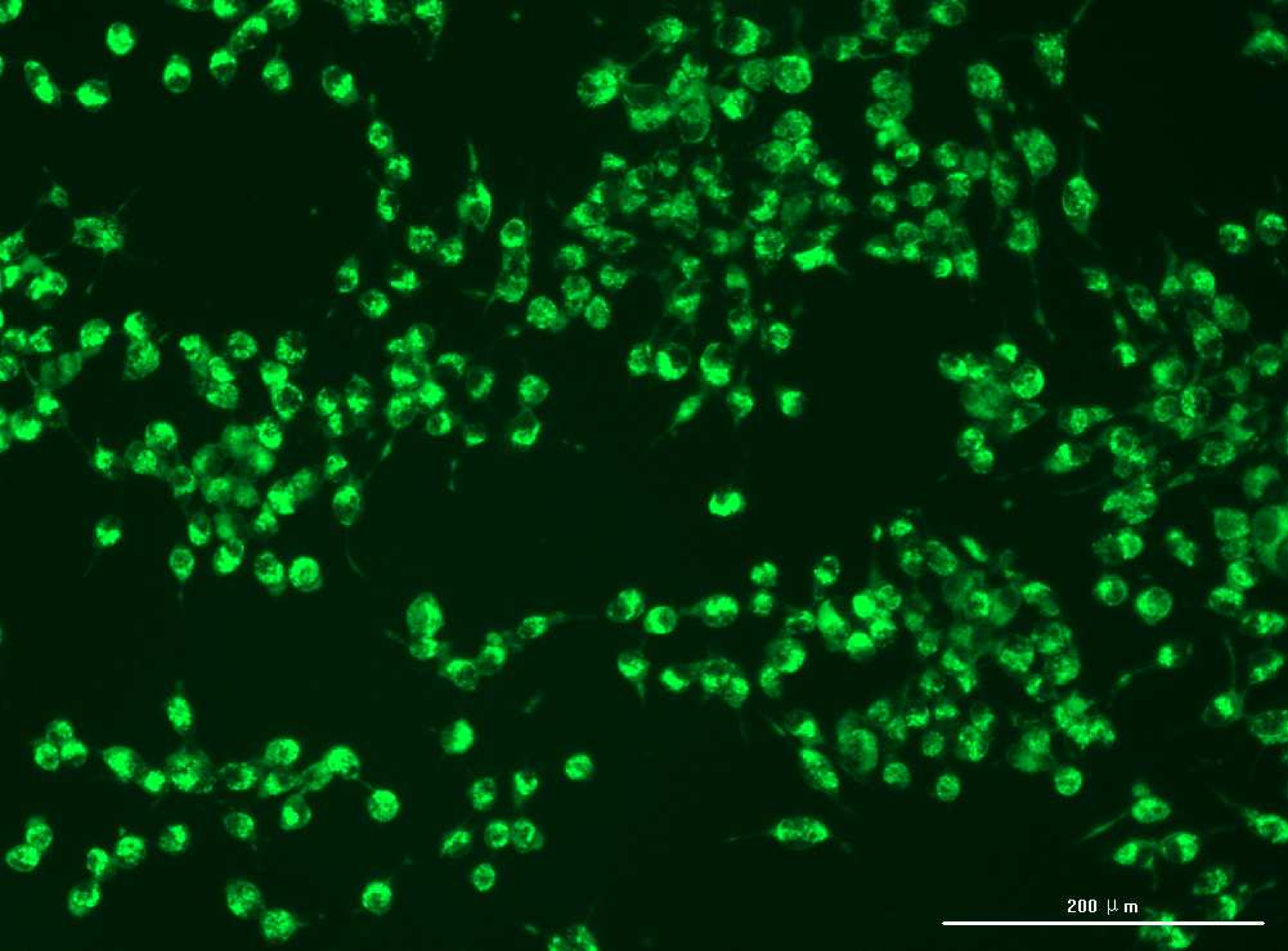
200 μm

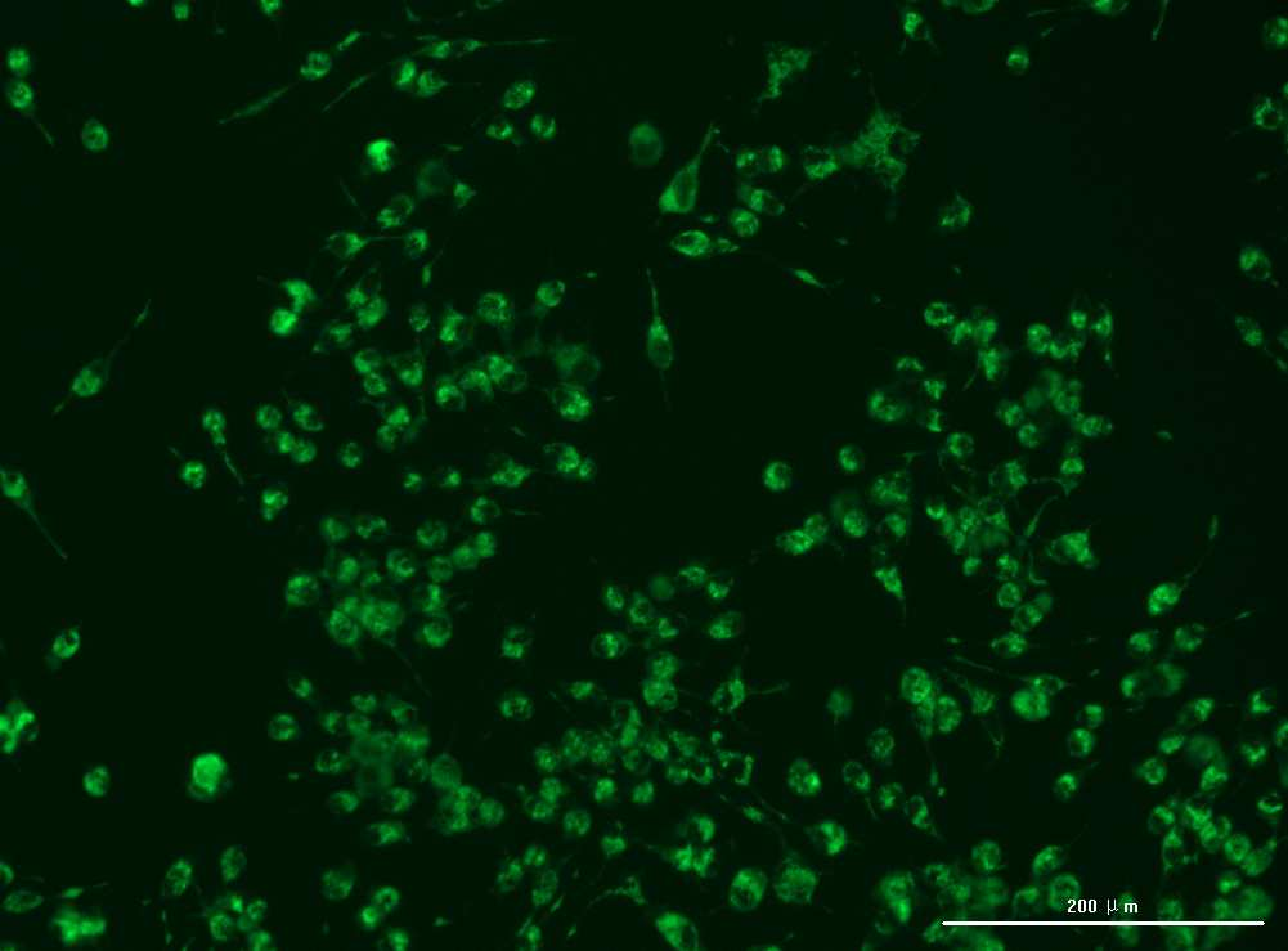
200 μm

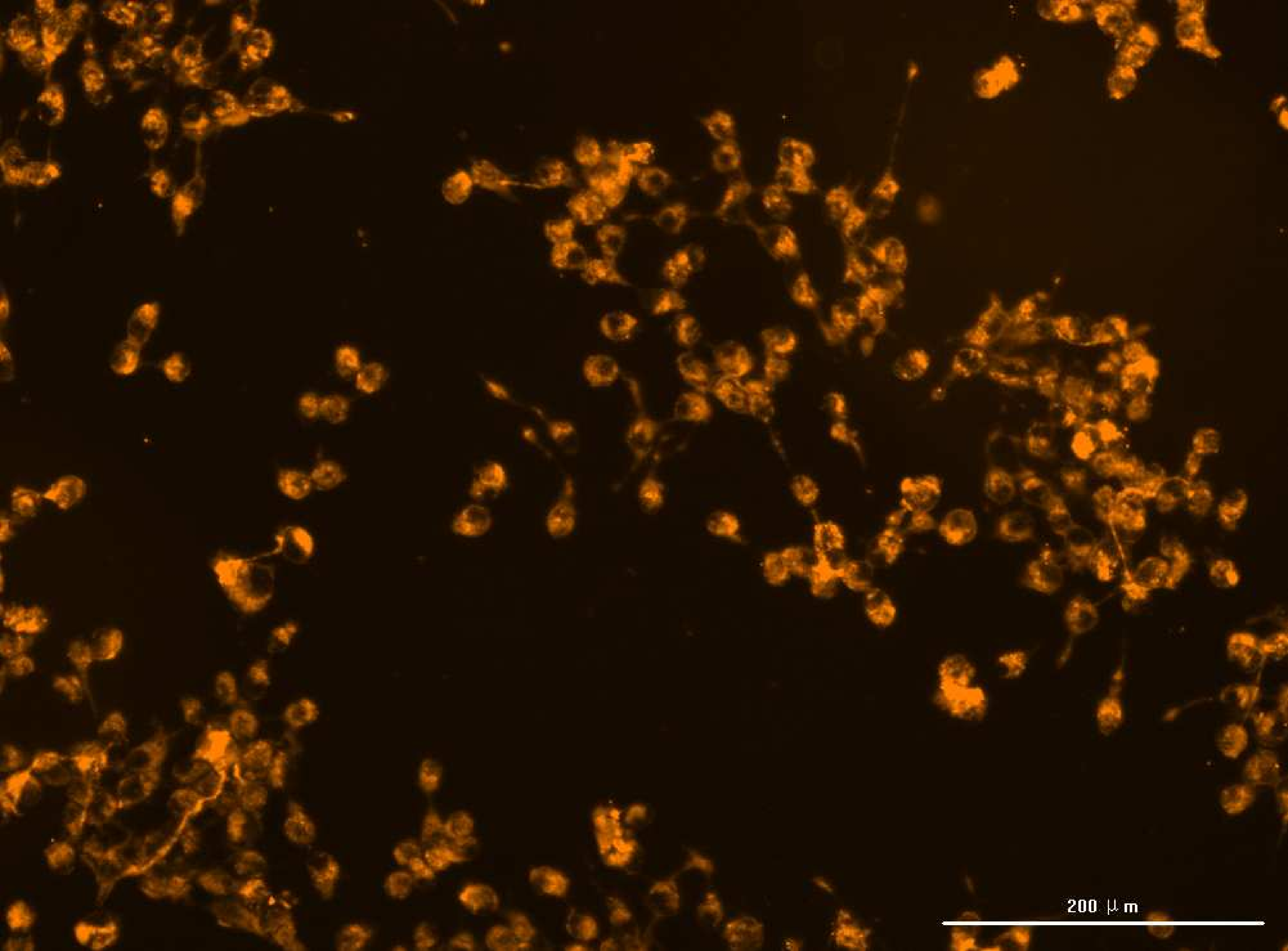
200 μm

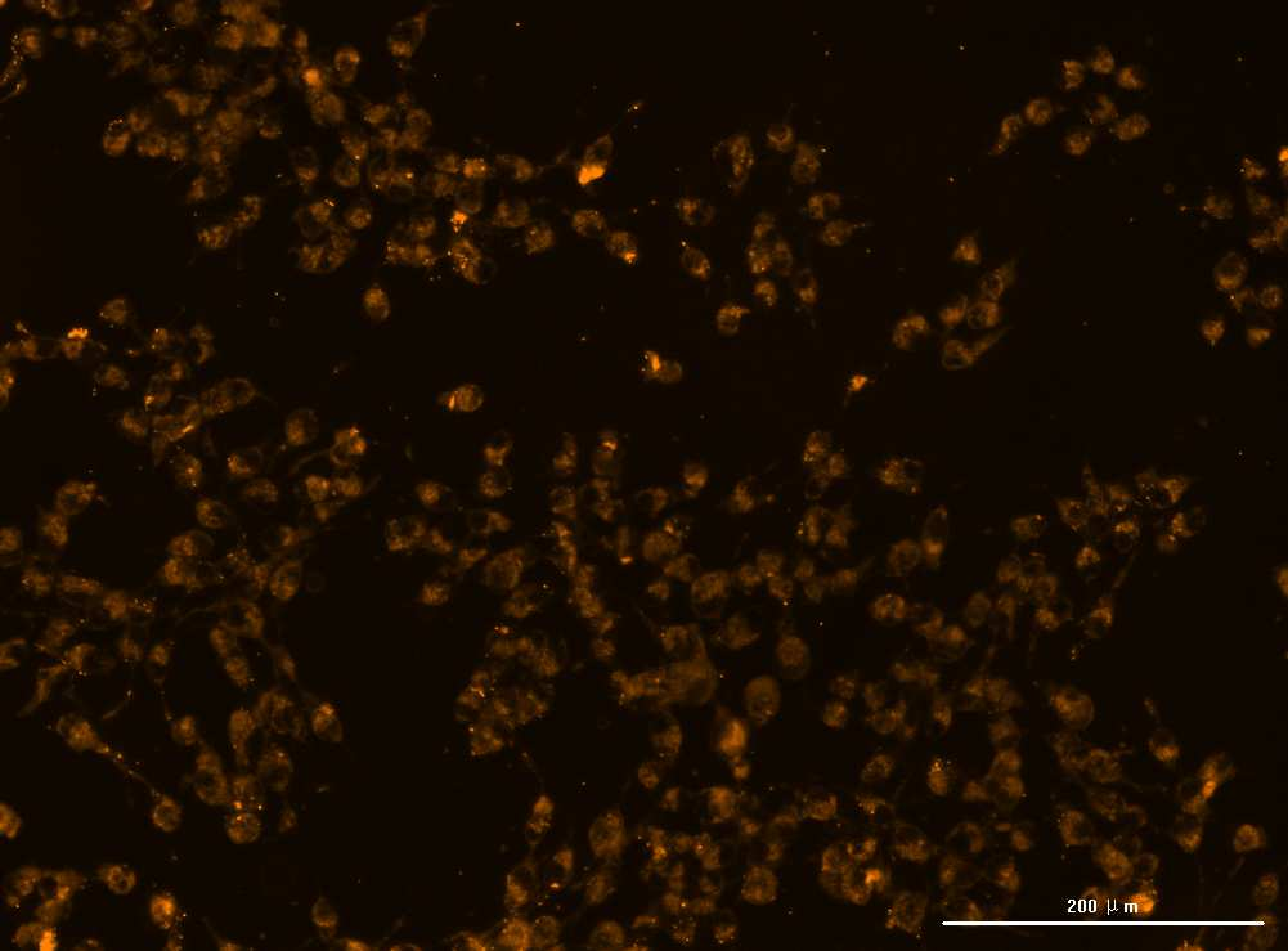
200 μm

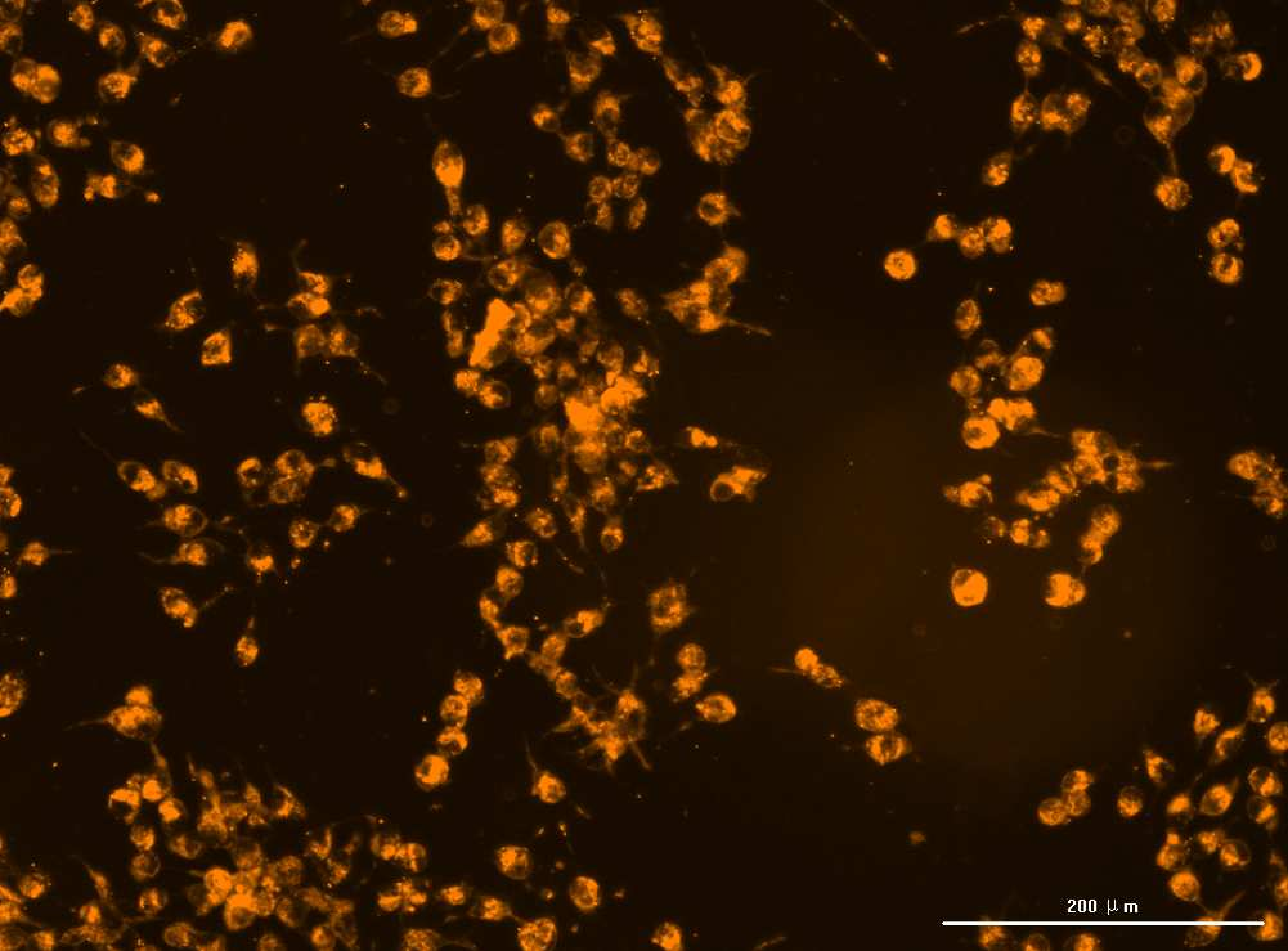
200 μm

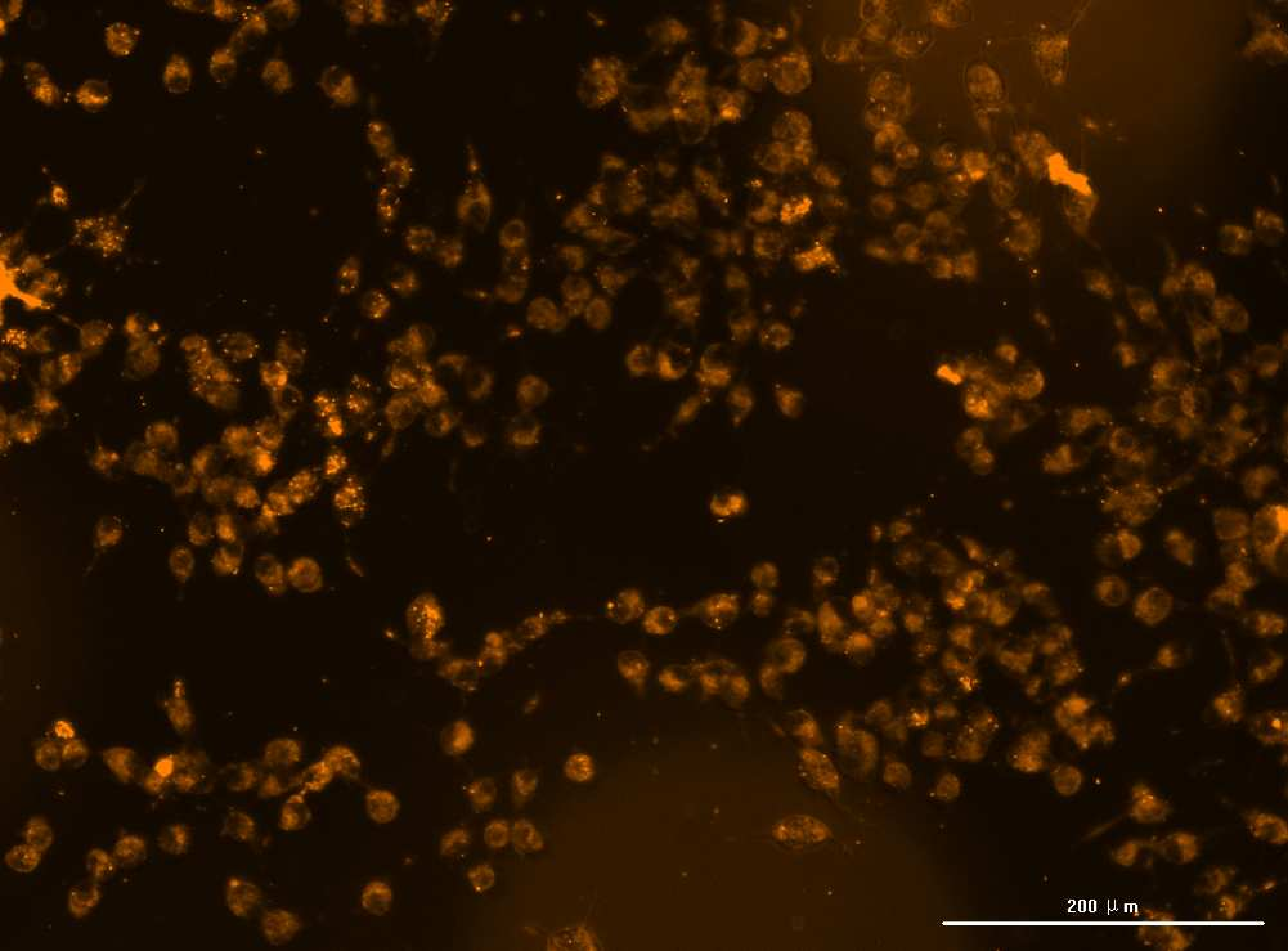
200 μm

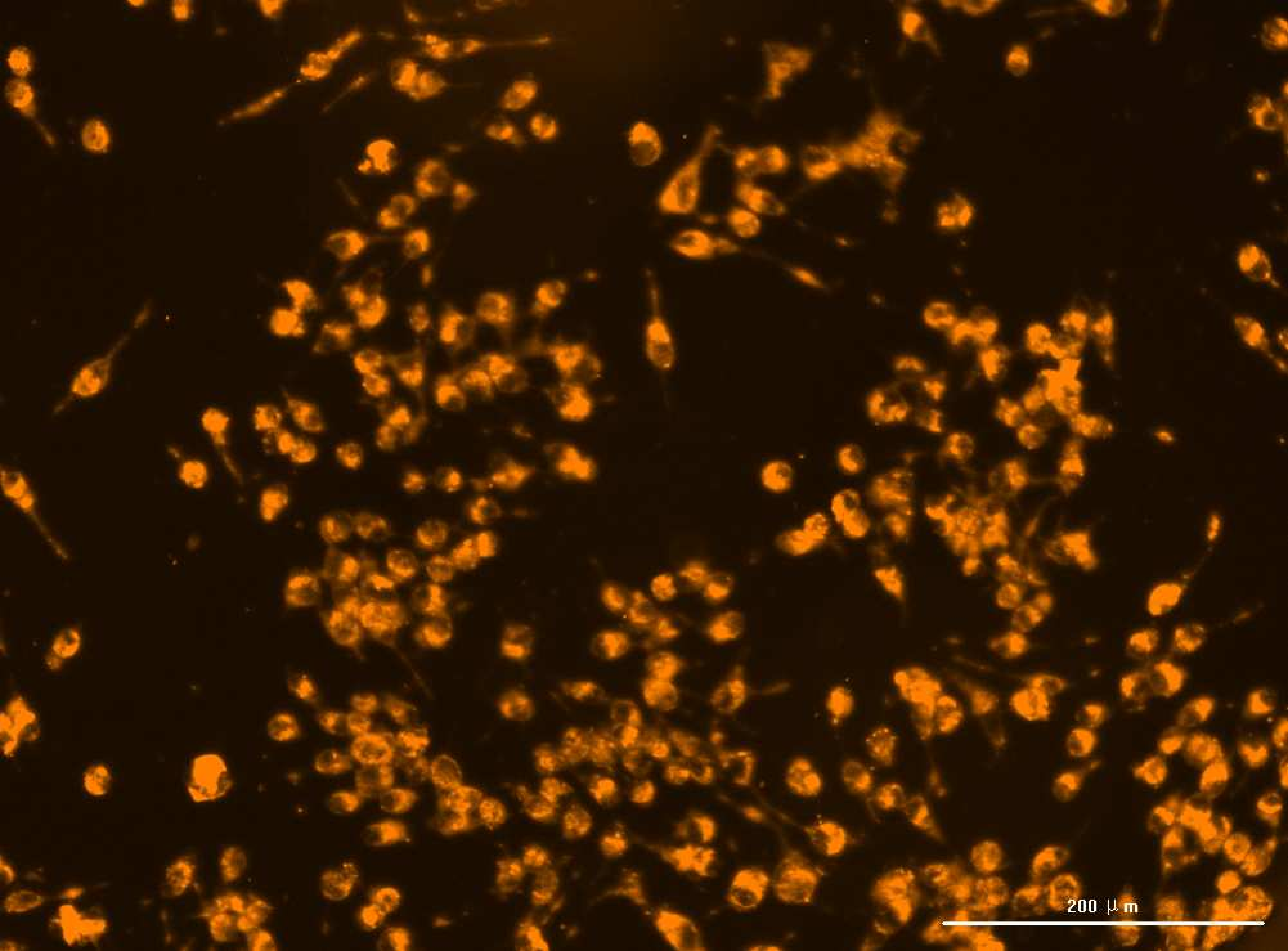
200 μm

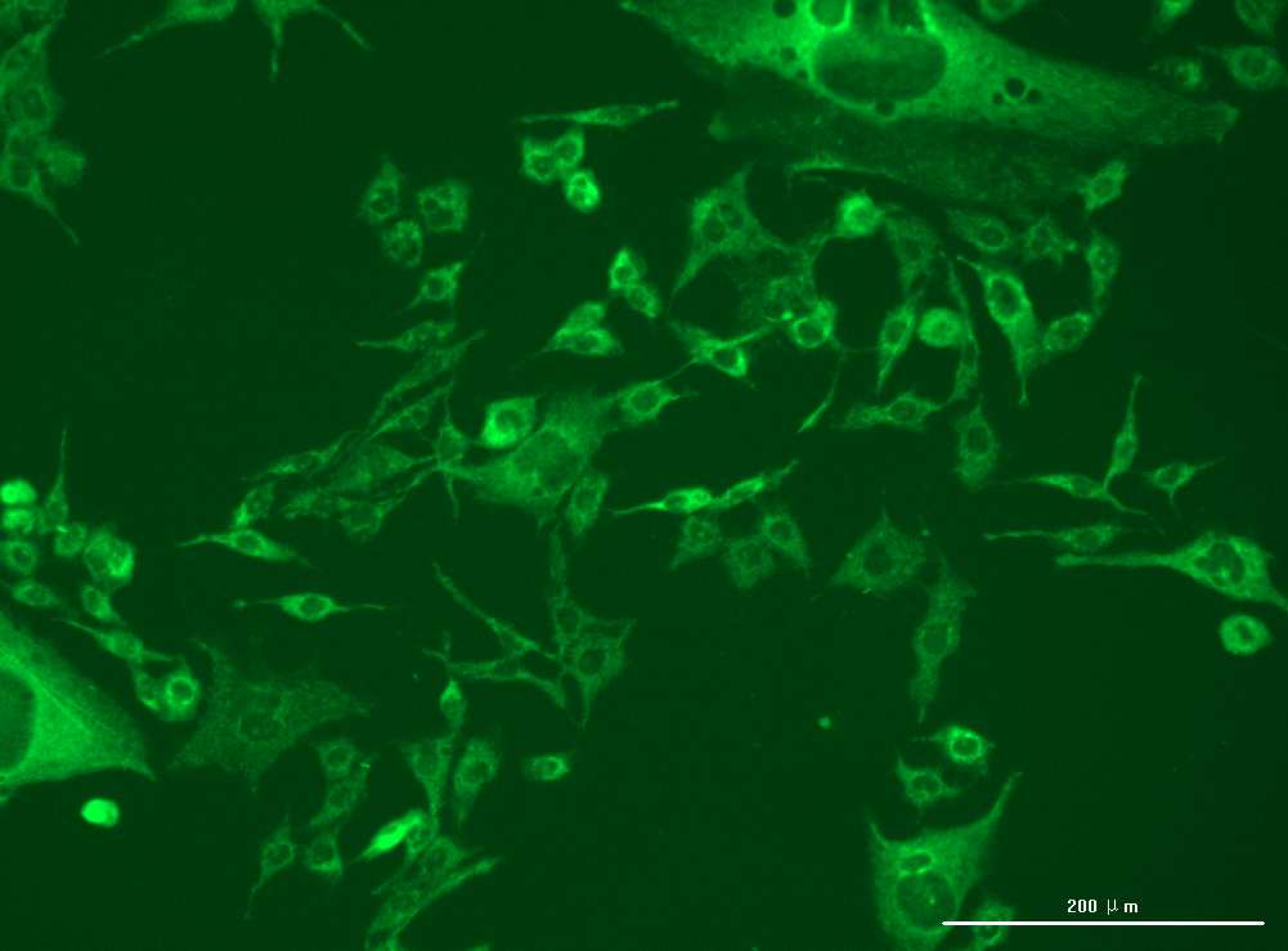
200 μm

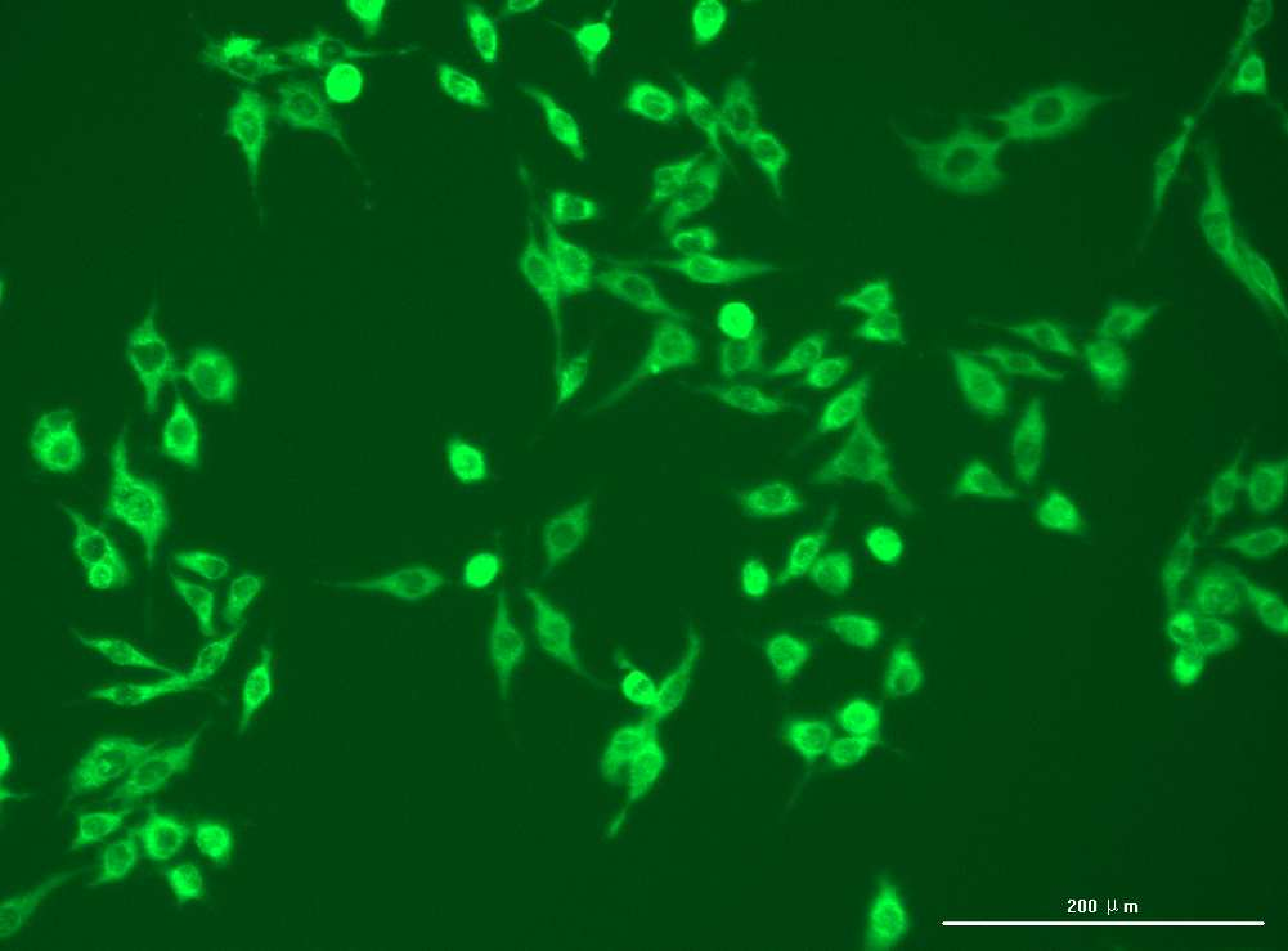
200 μm

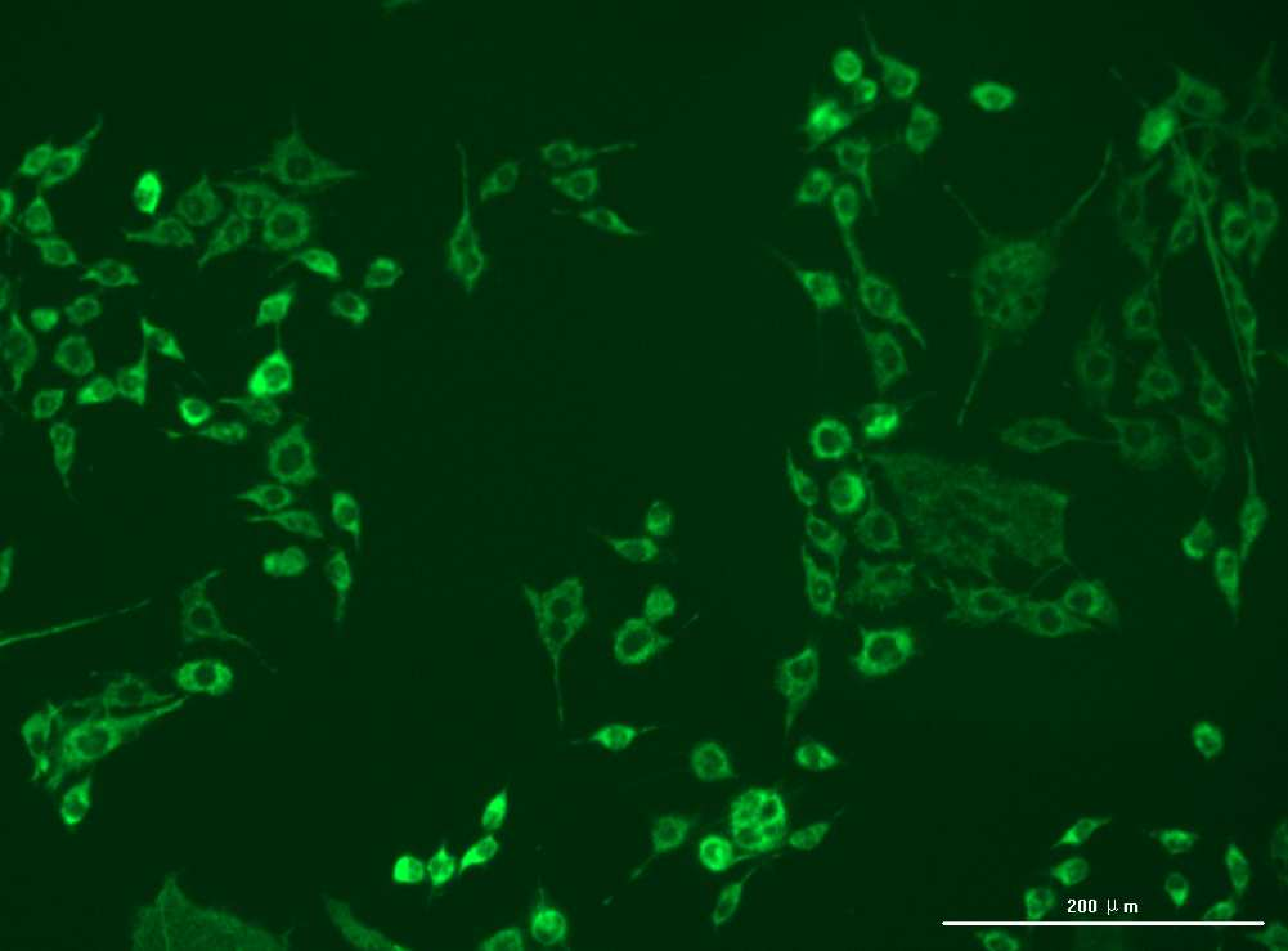
200 μm

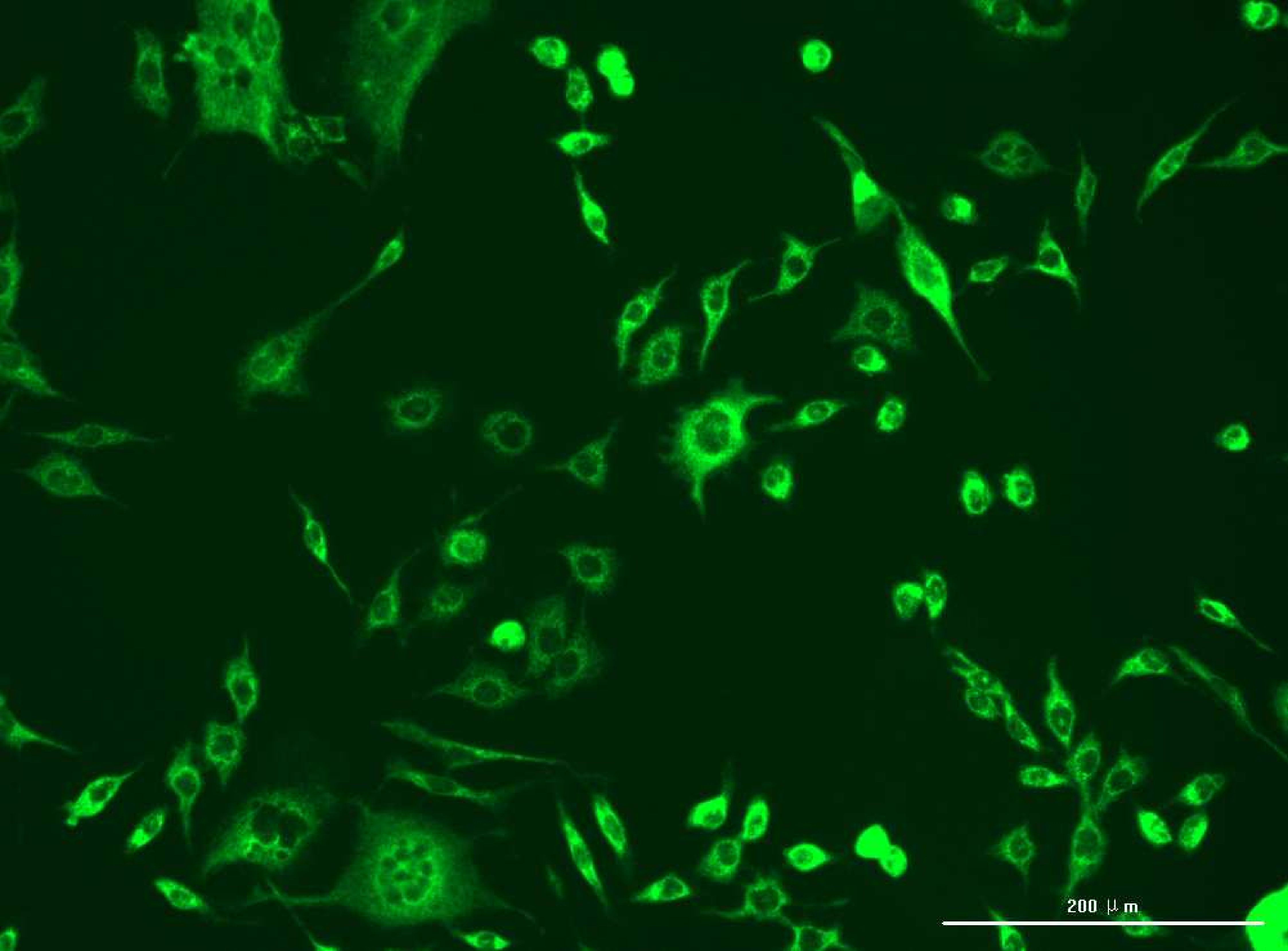
200 μm

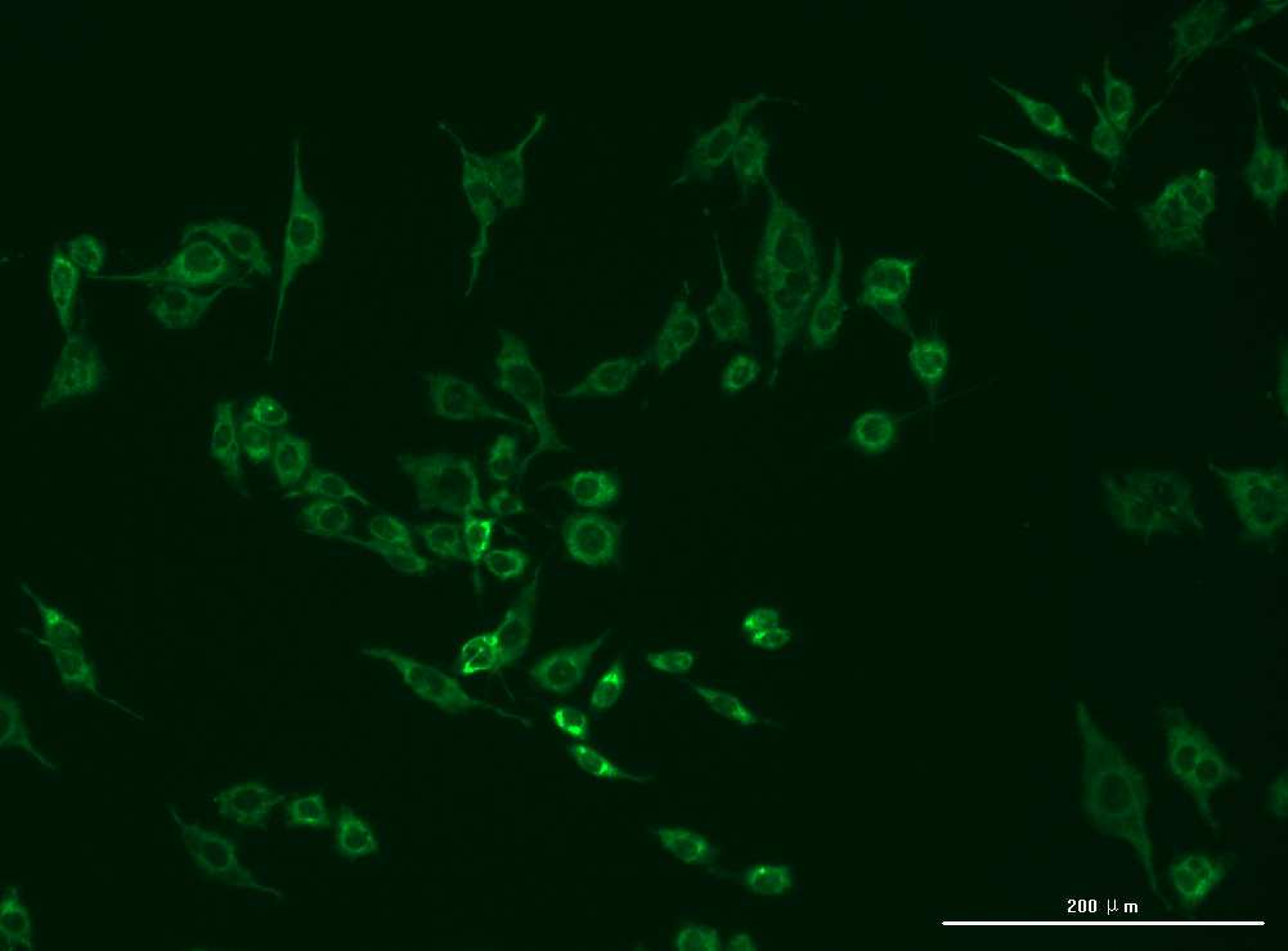
200 μm

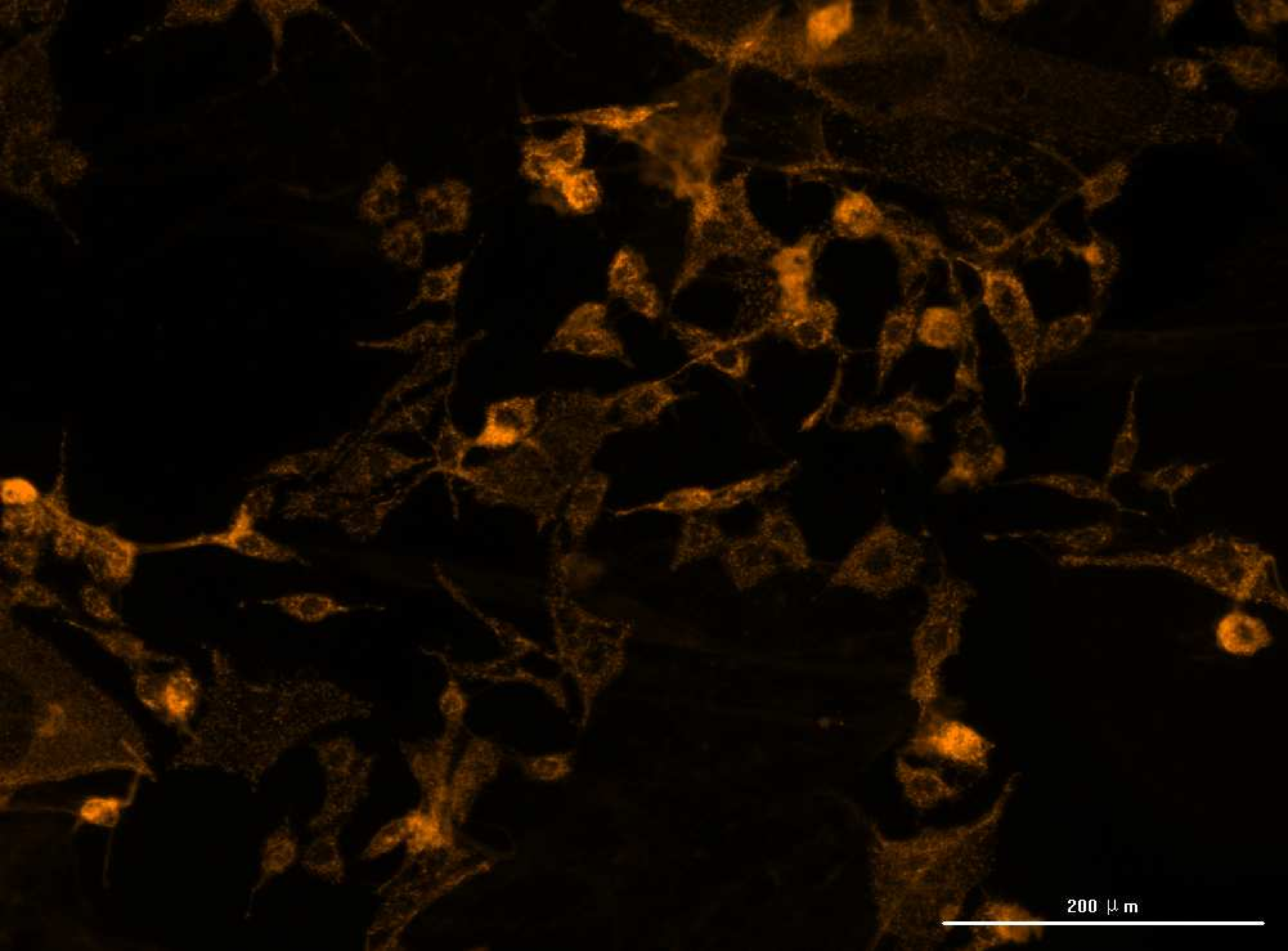
200 μm

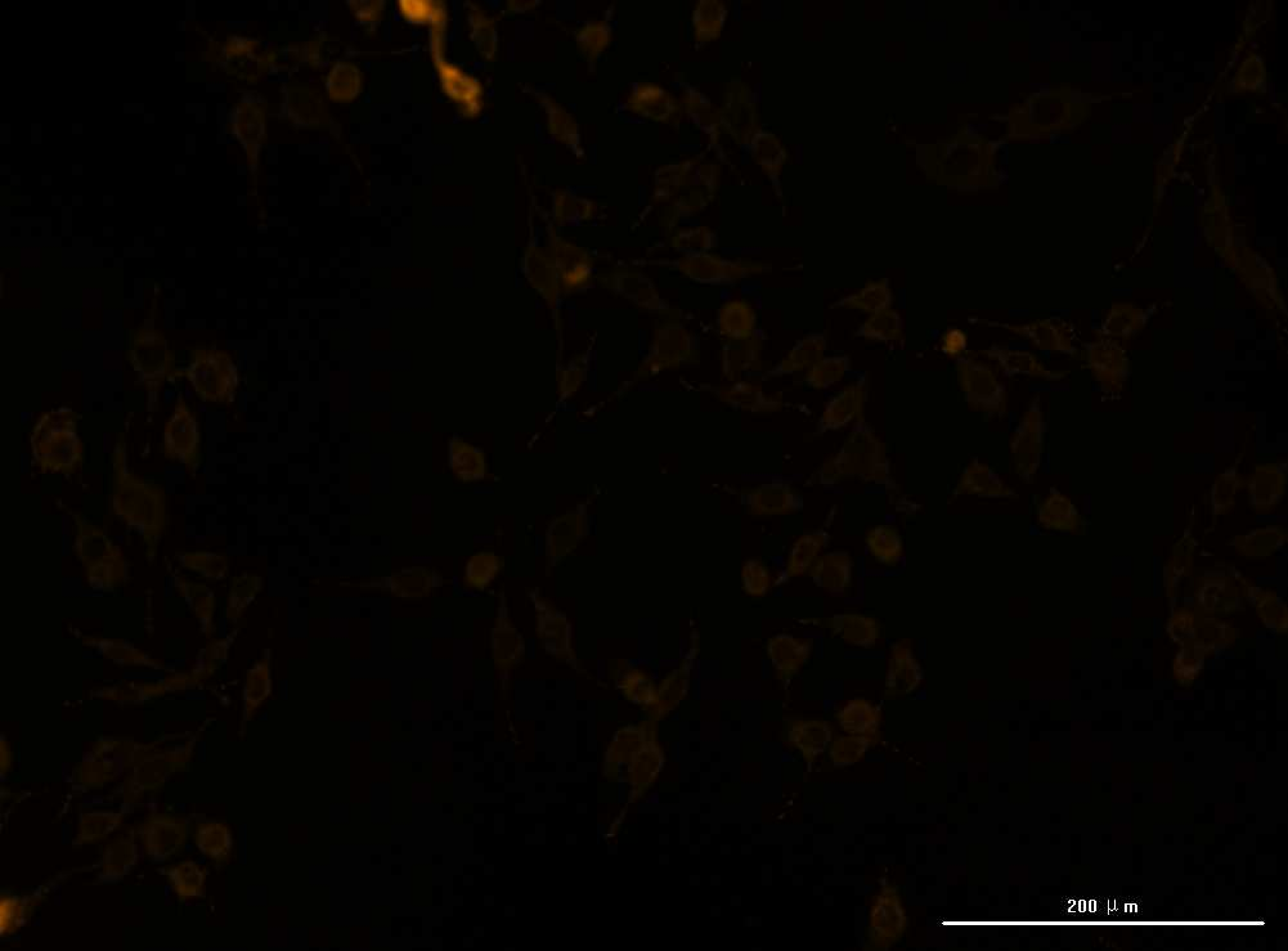
200 μm

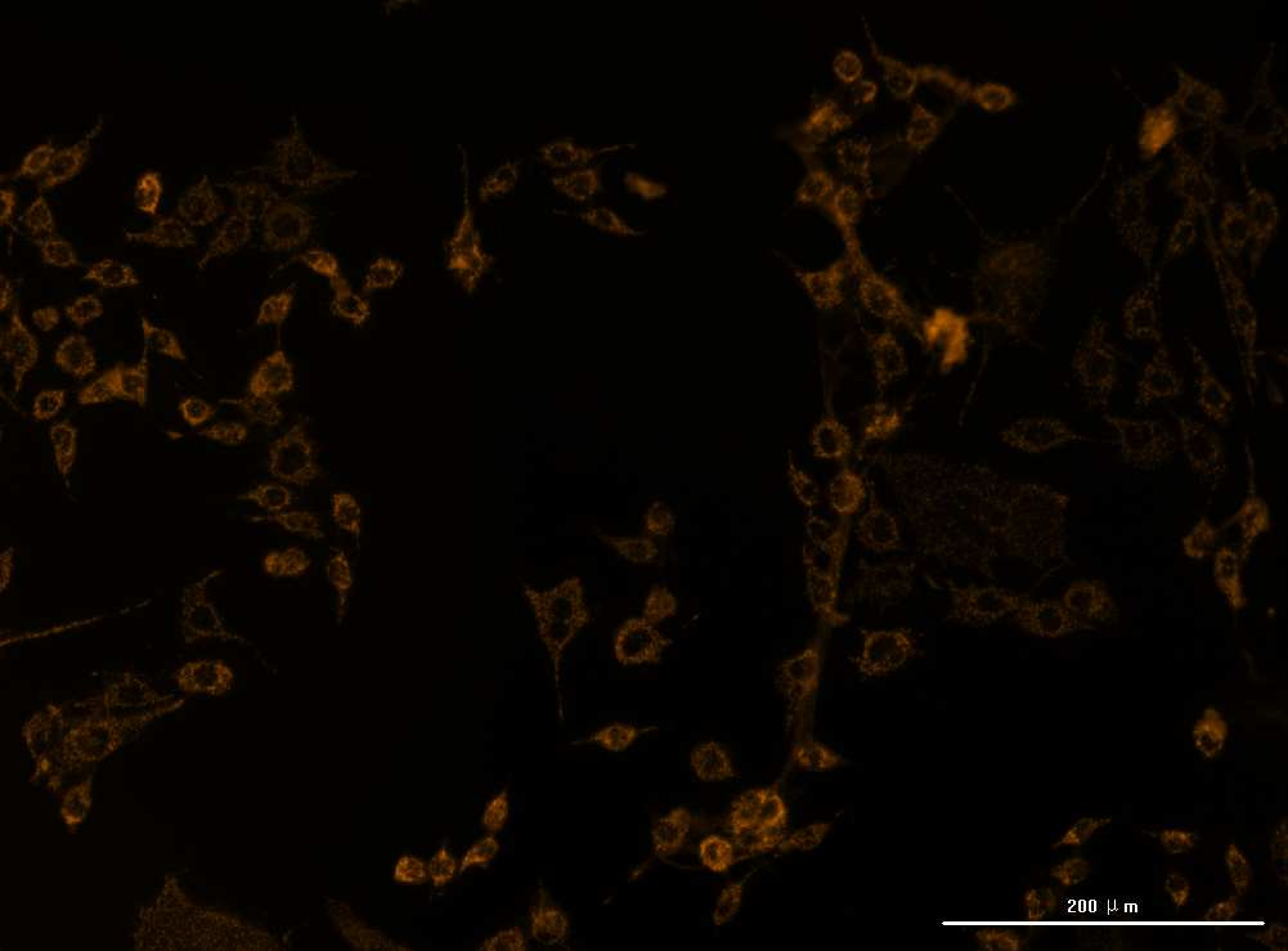
200 μm

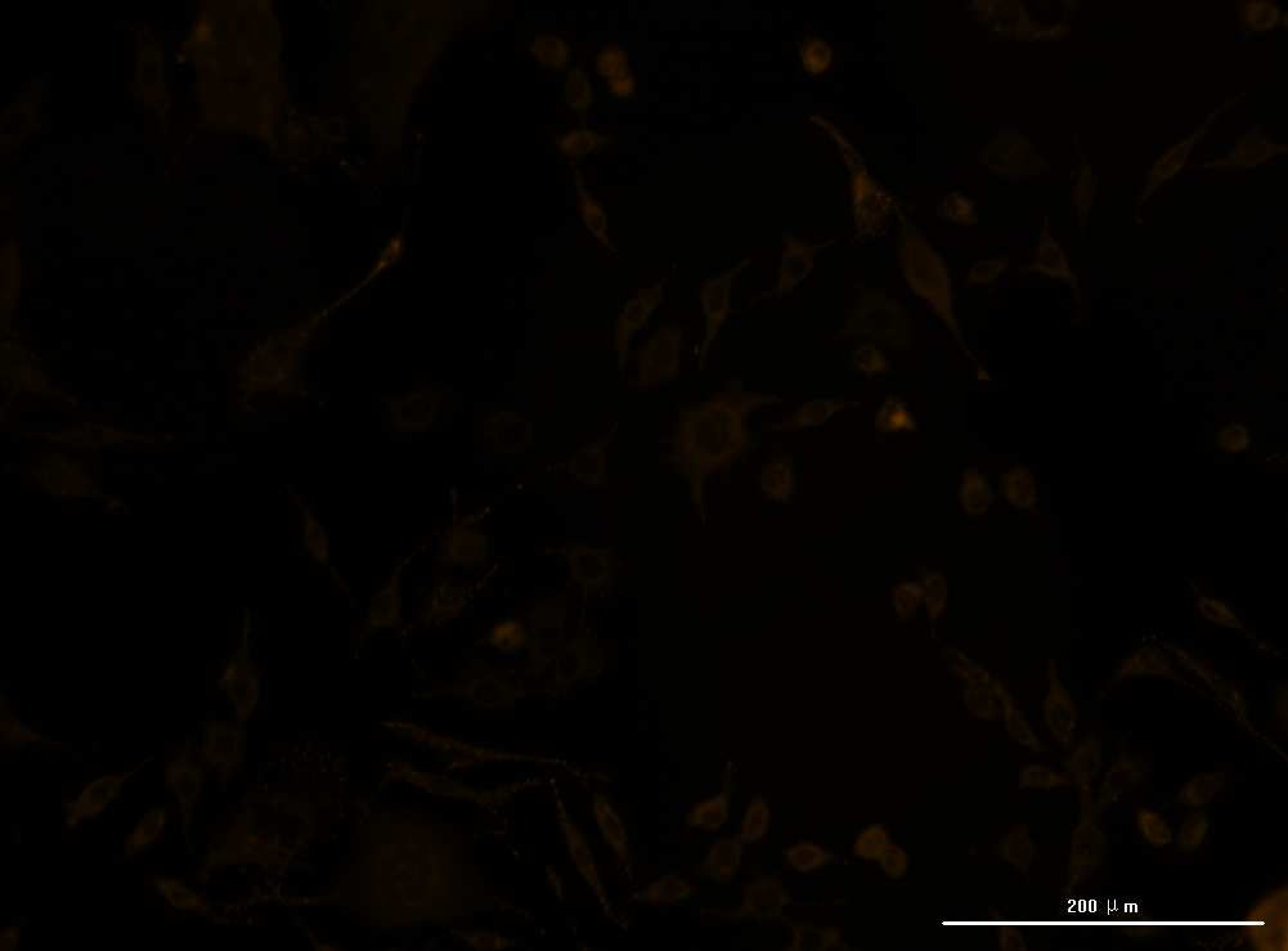
200 μm

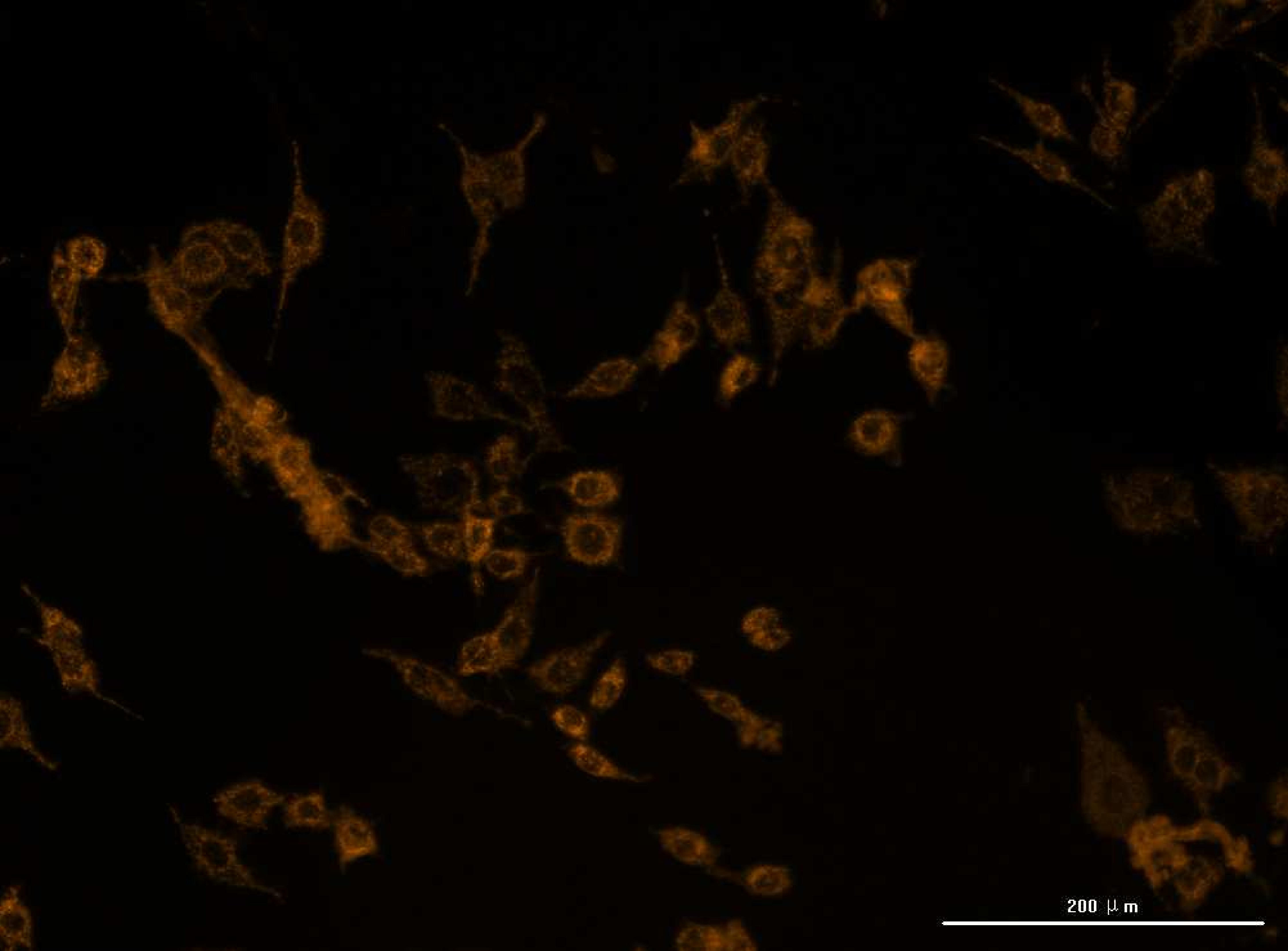
200 μm

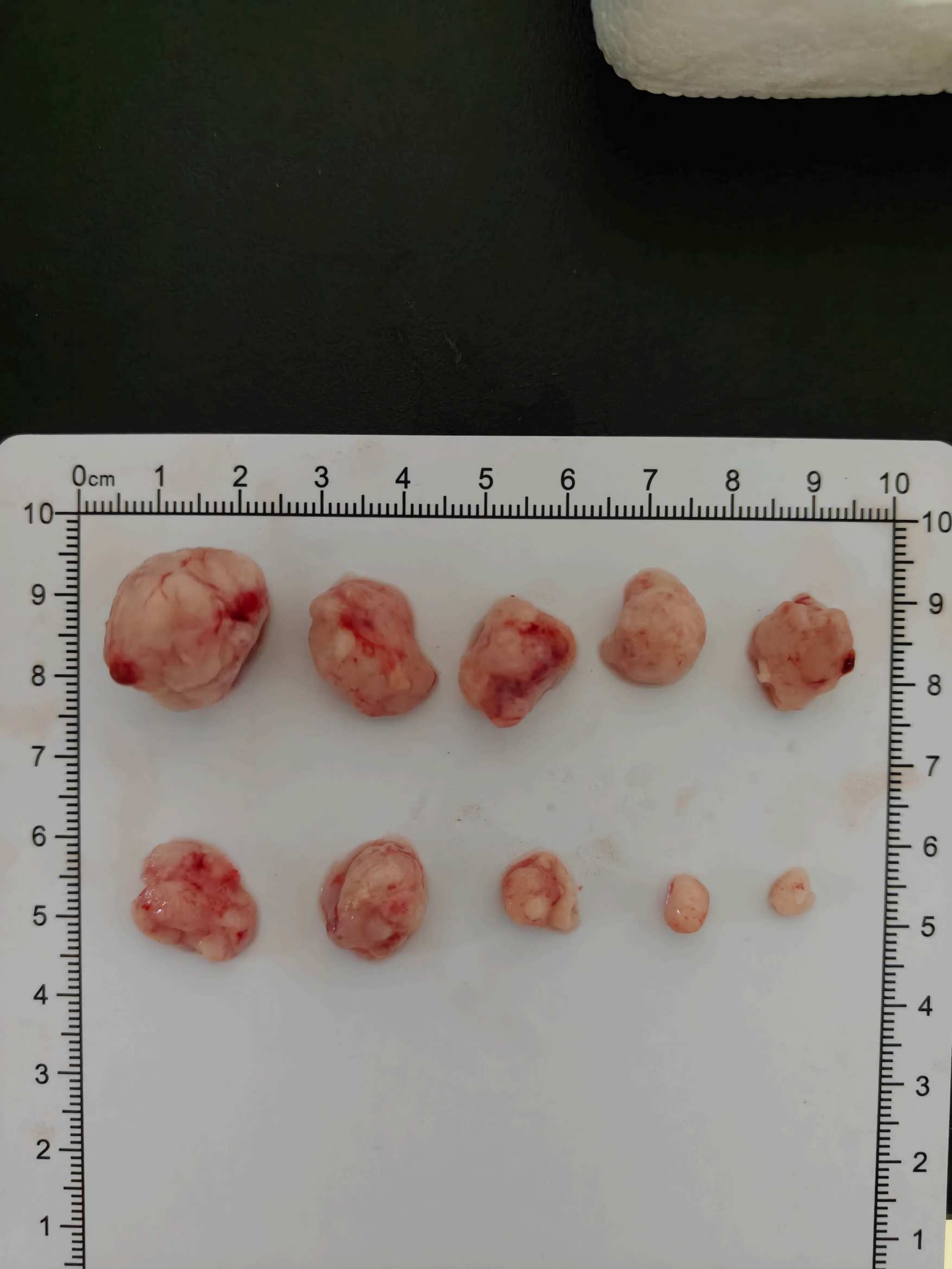
0cm 1 2 3 4 5 6 7 8 9 10

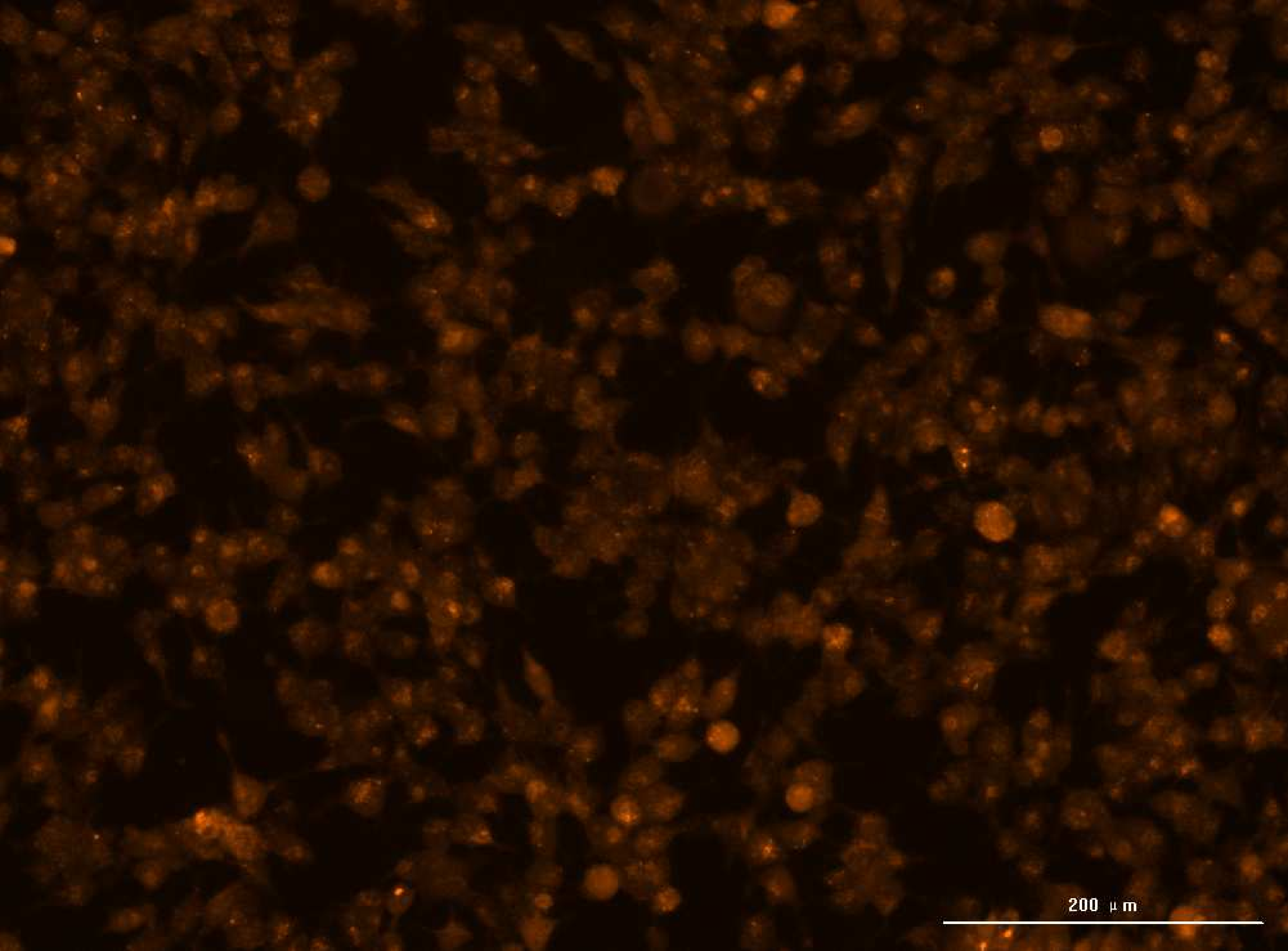
200 μm

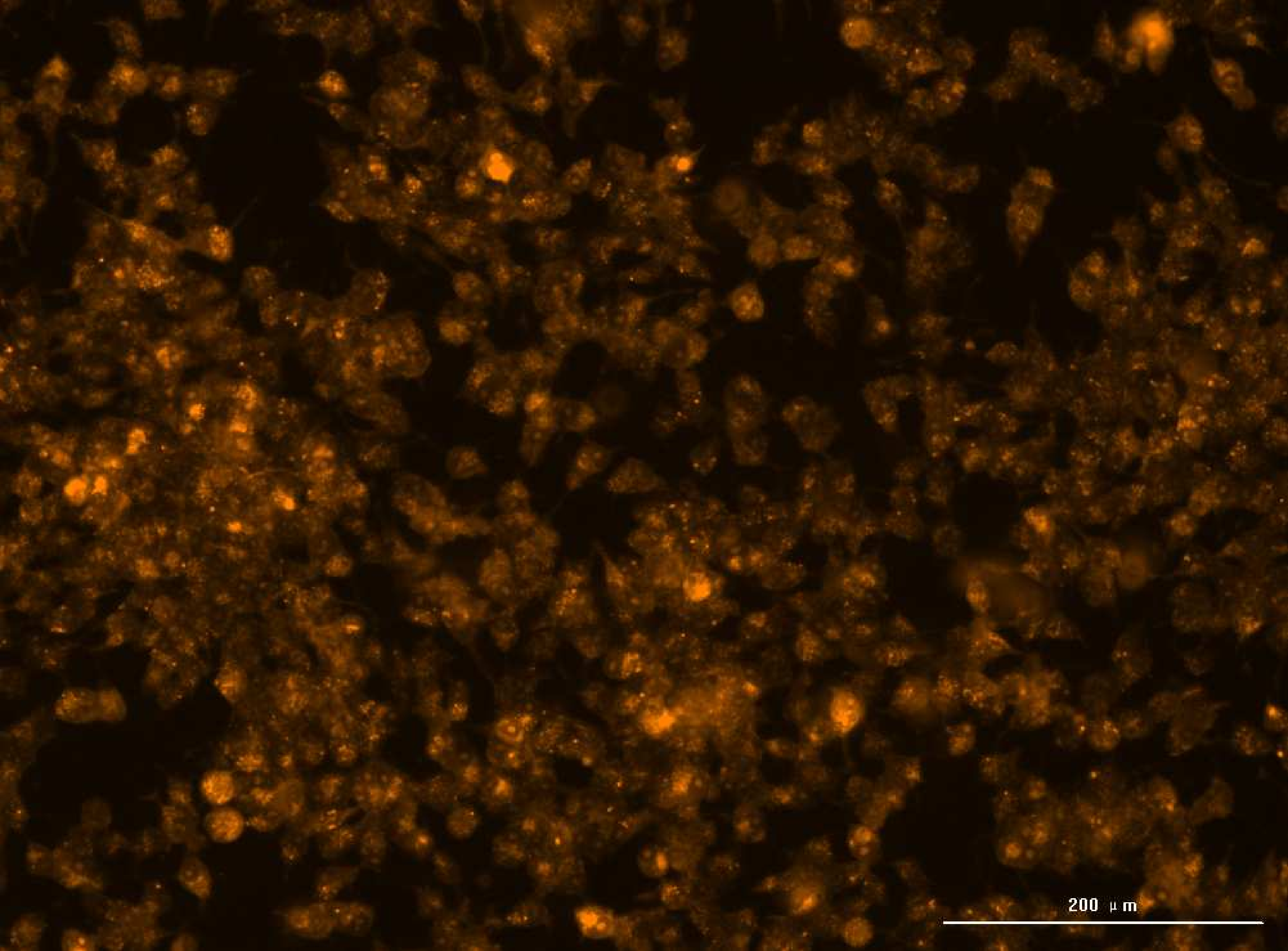
200 μm

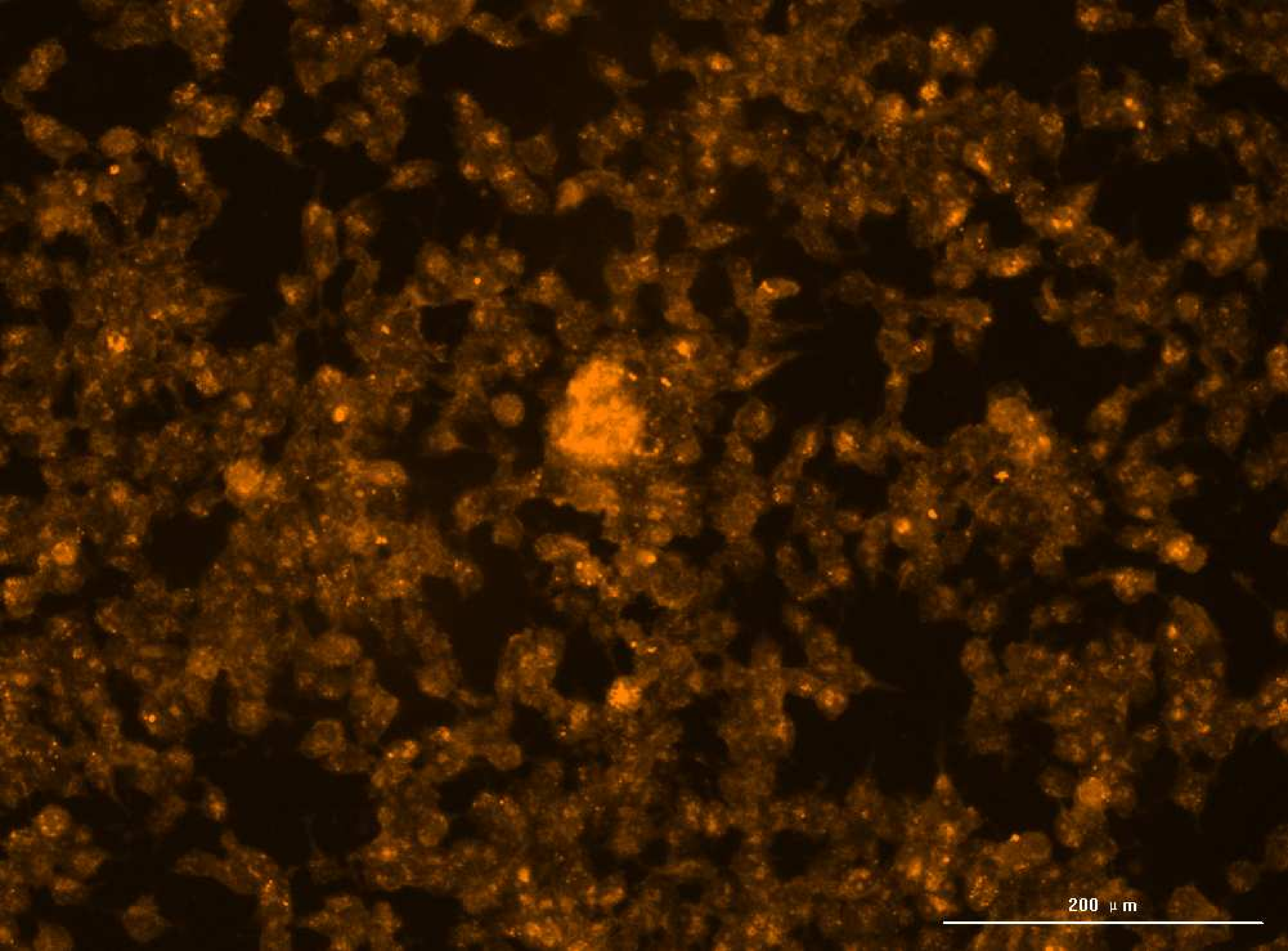
200 μm

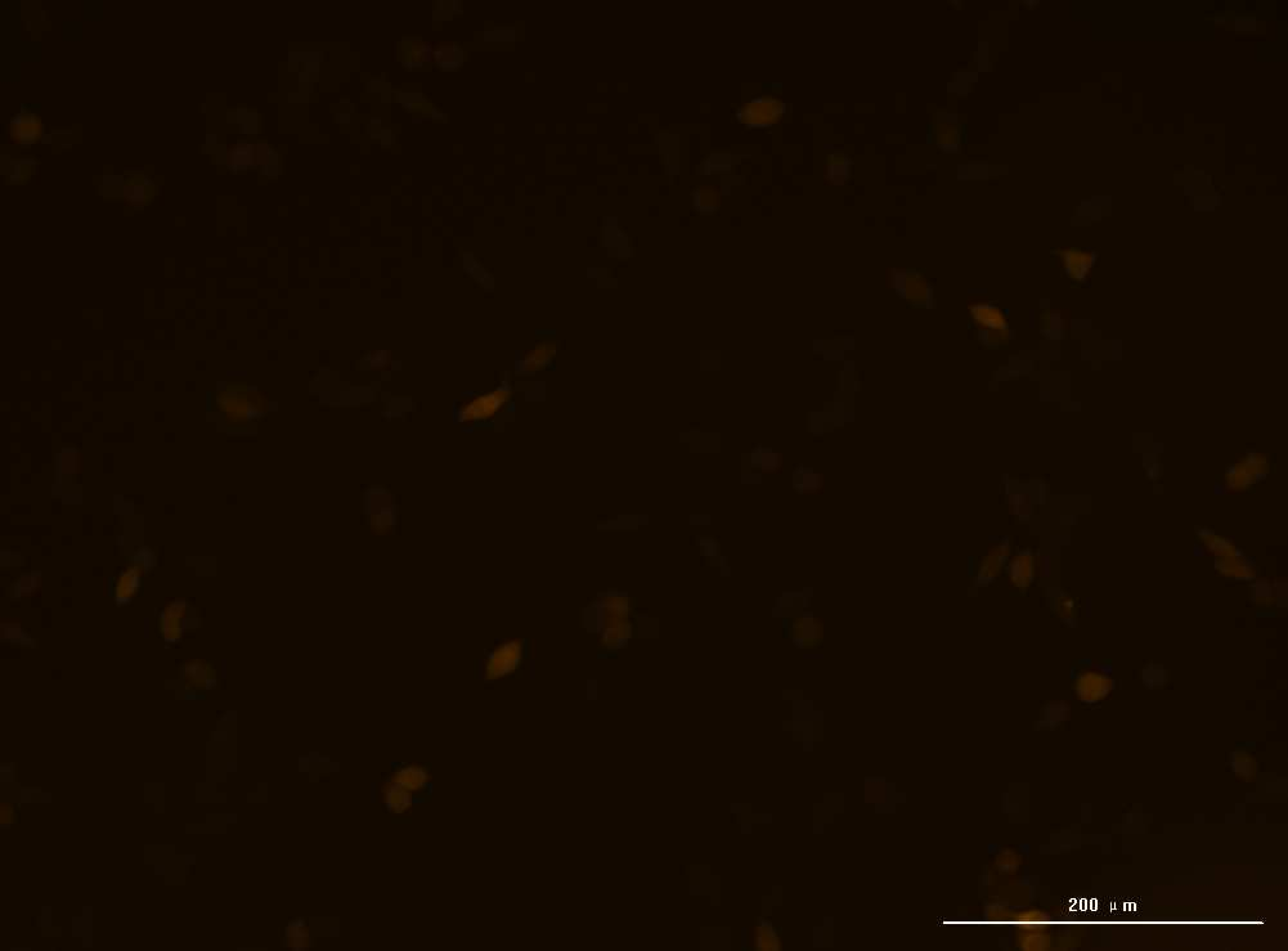
200 µm

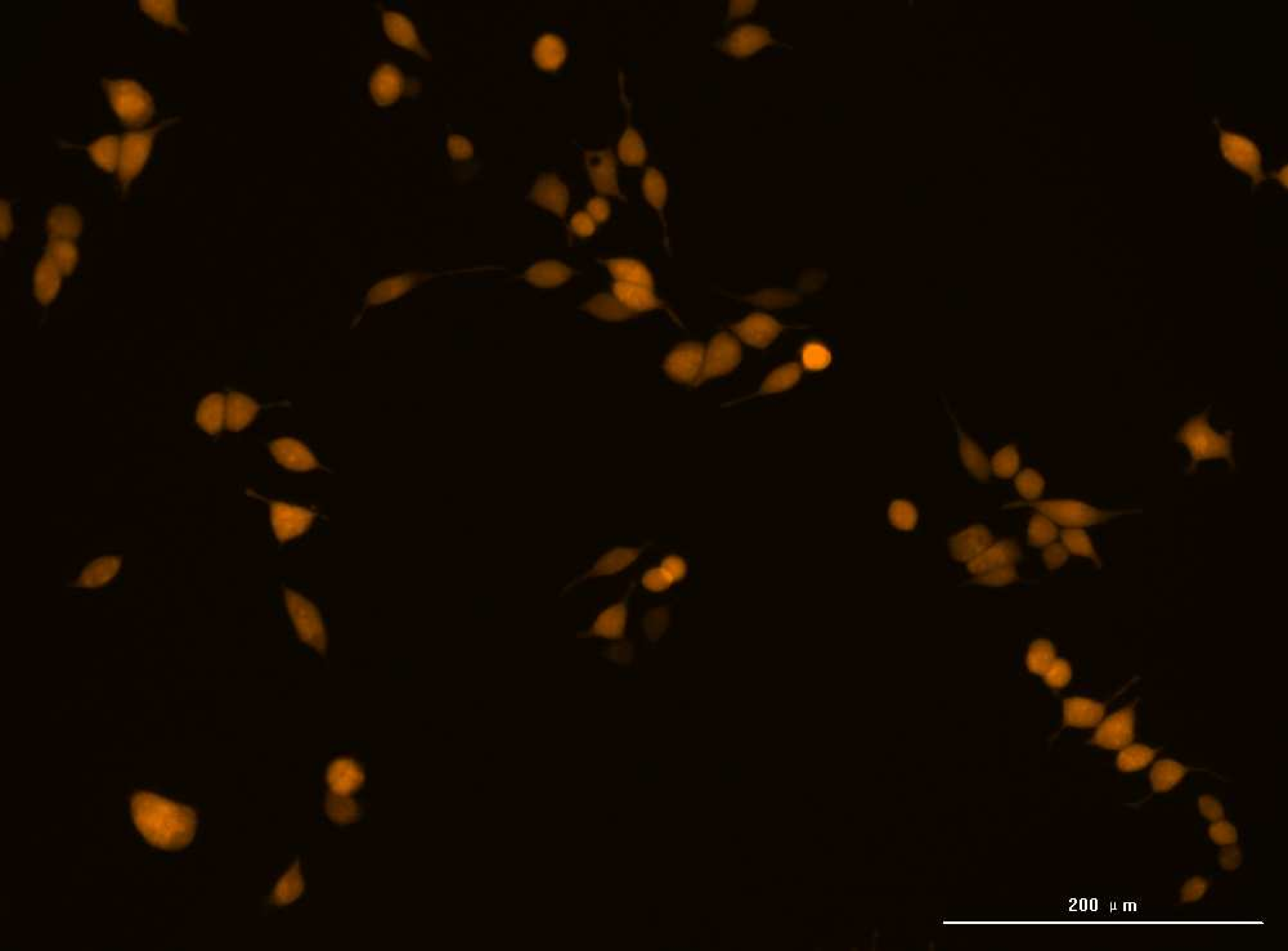
200 µm

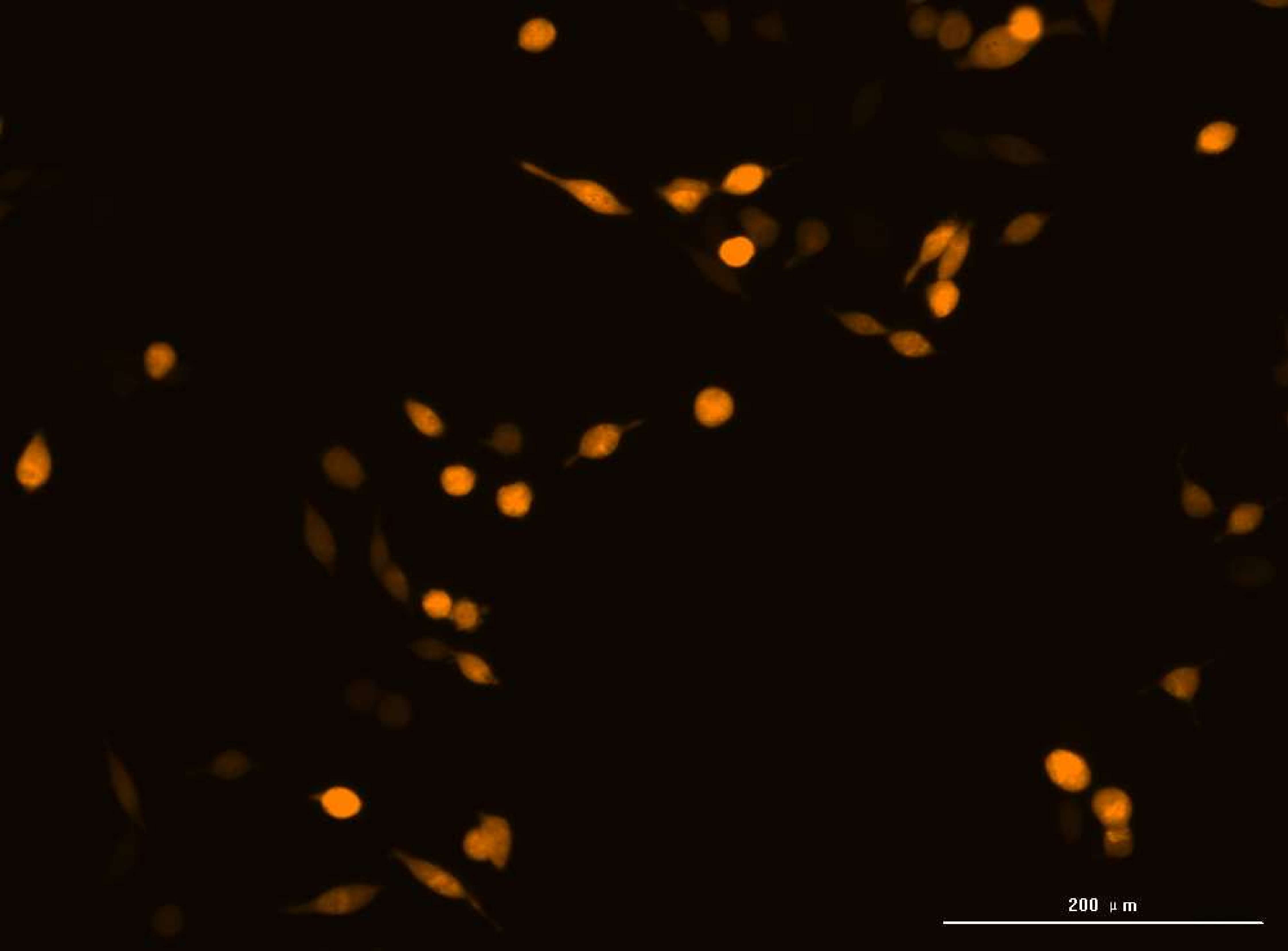
200 μm

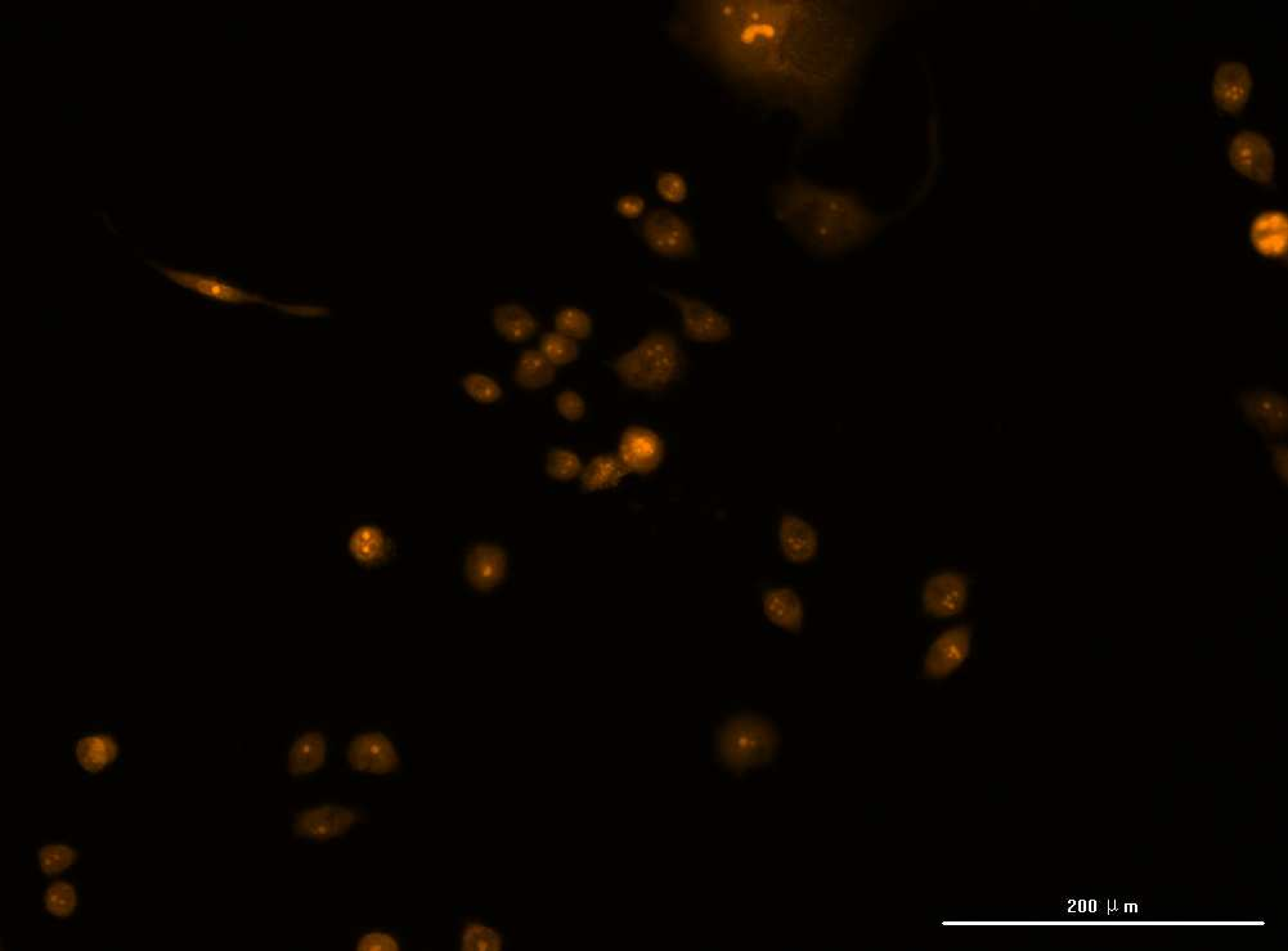
200 μm

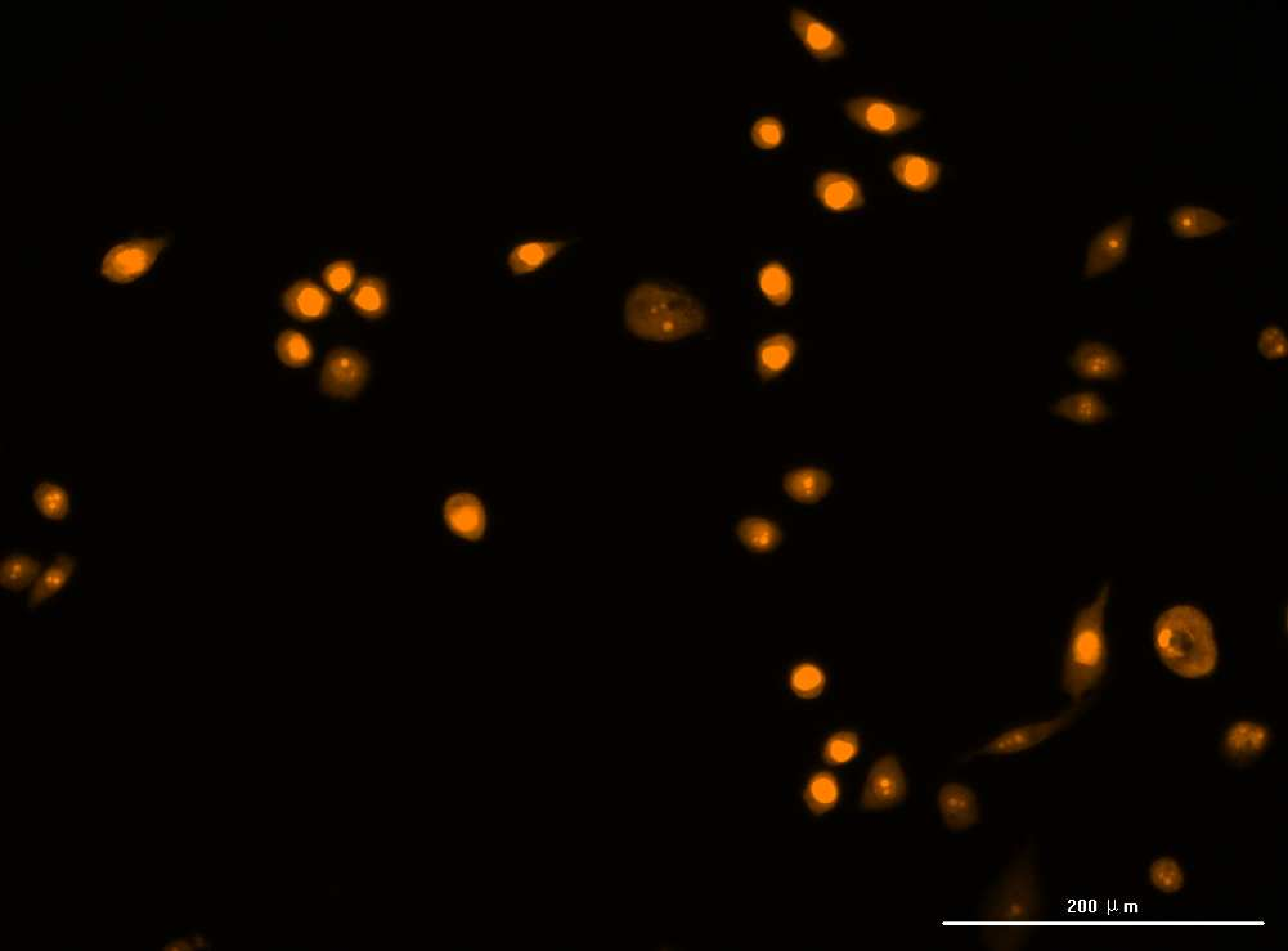
200 μm

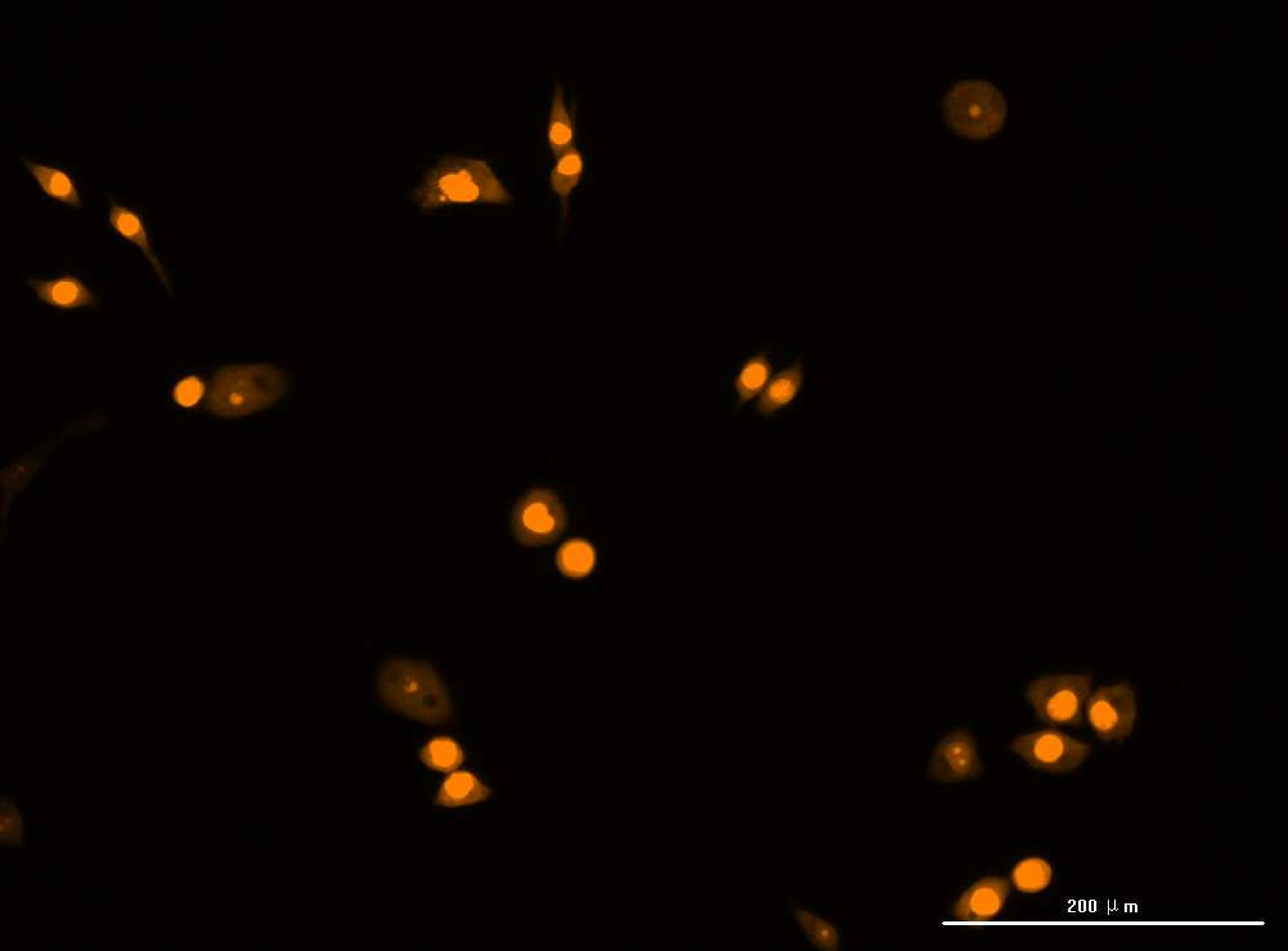
200 μm

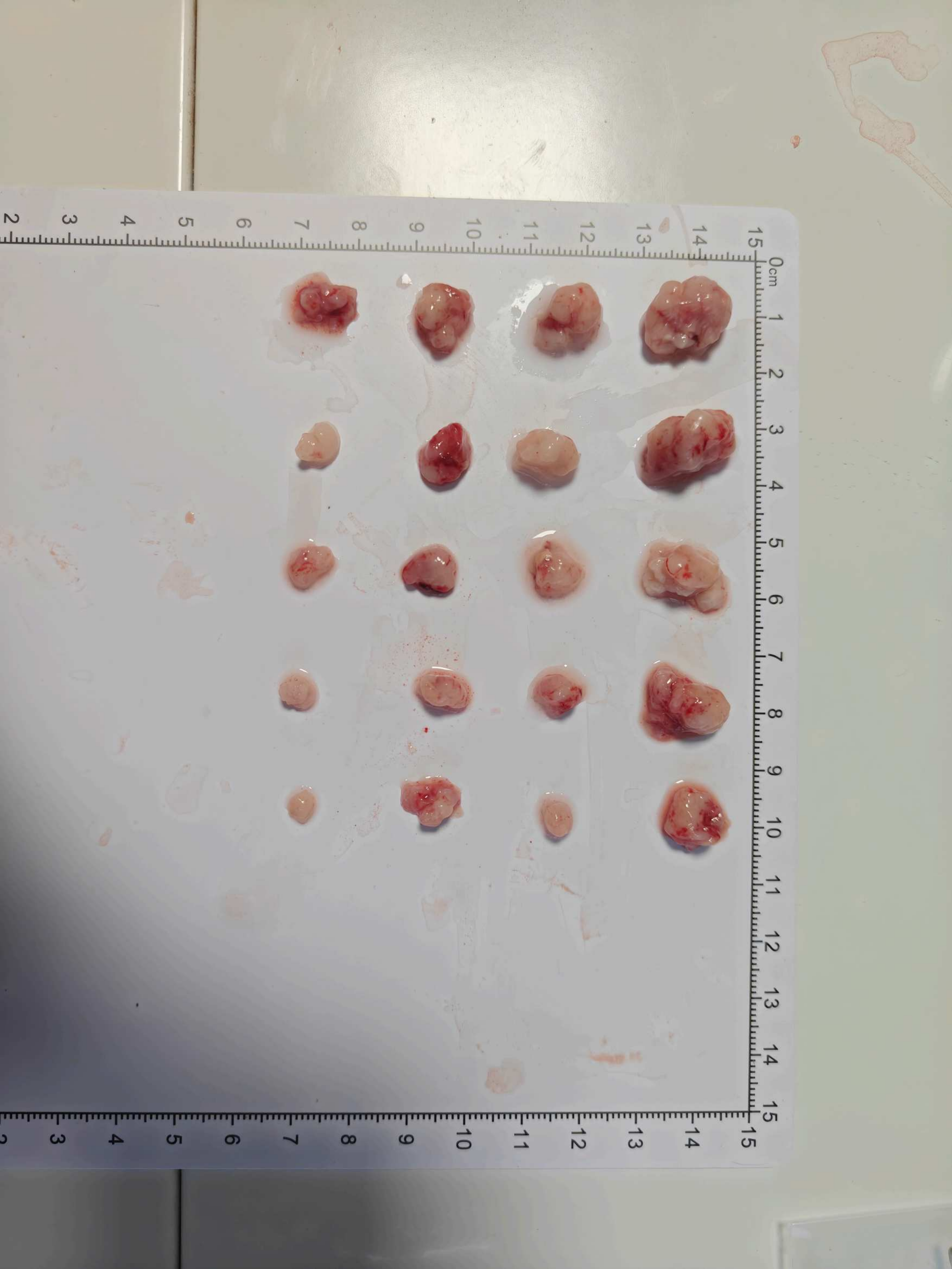

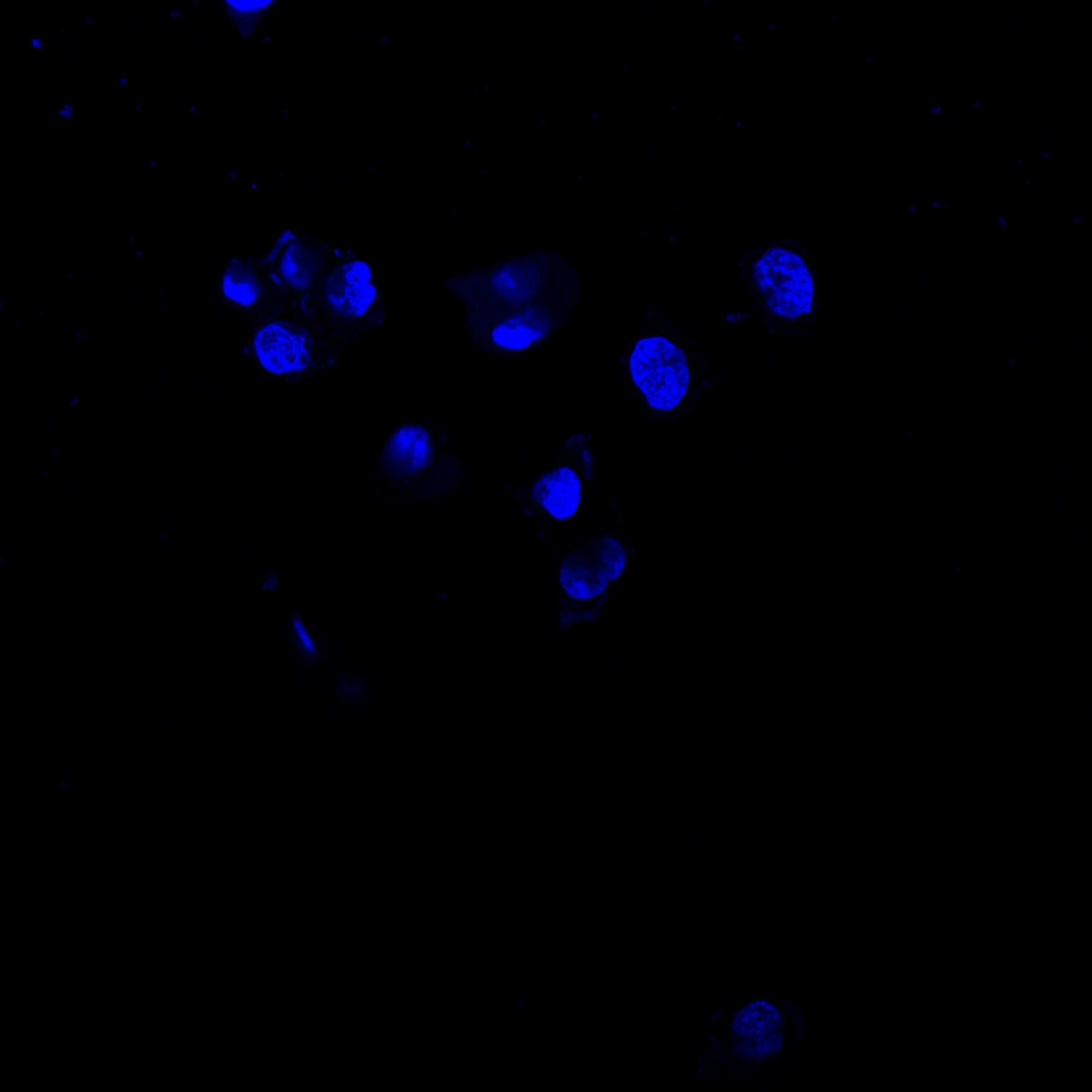

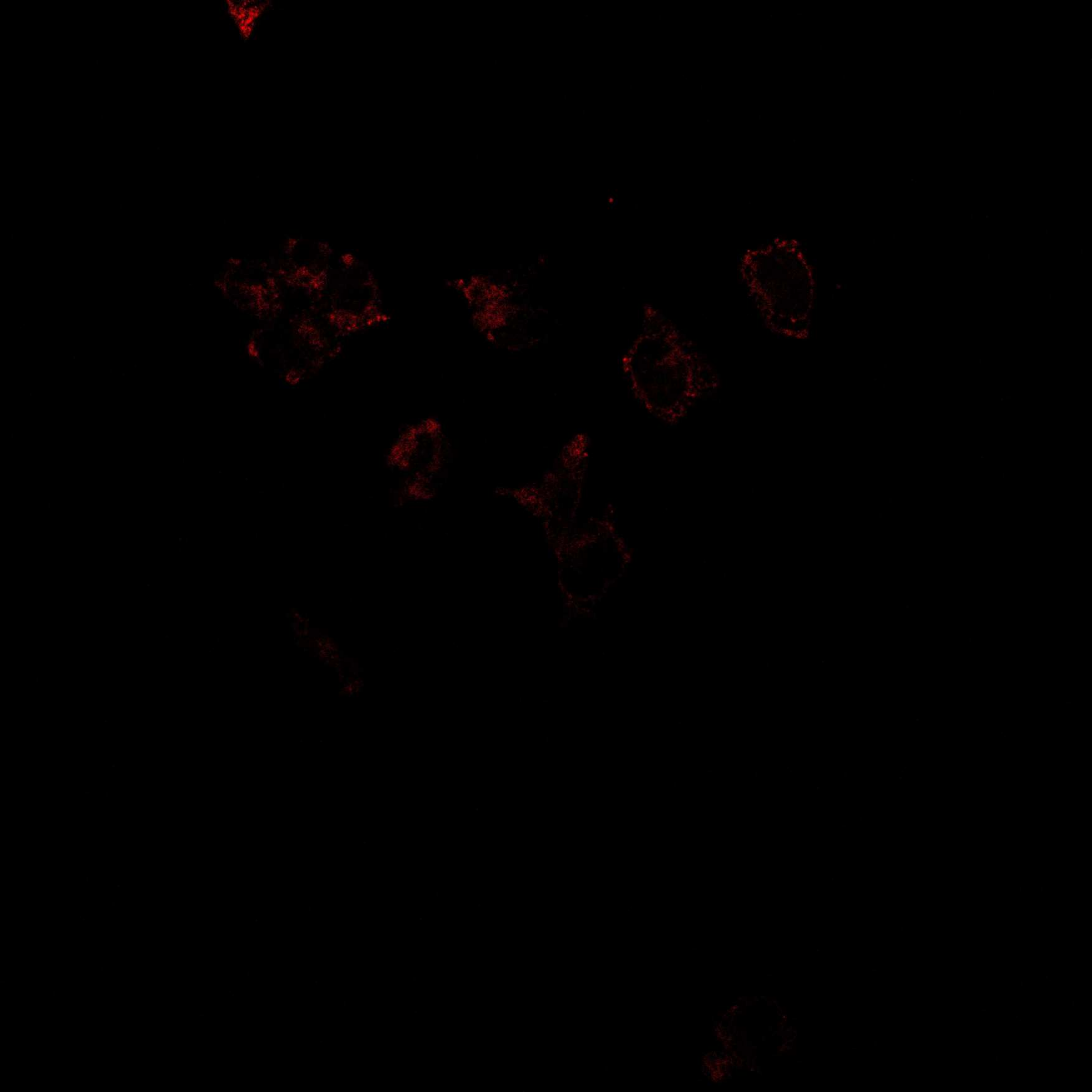

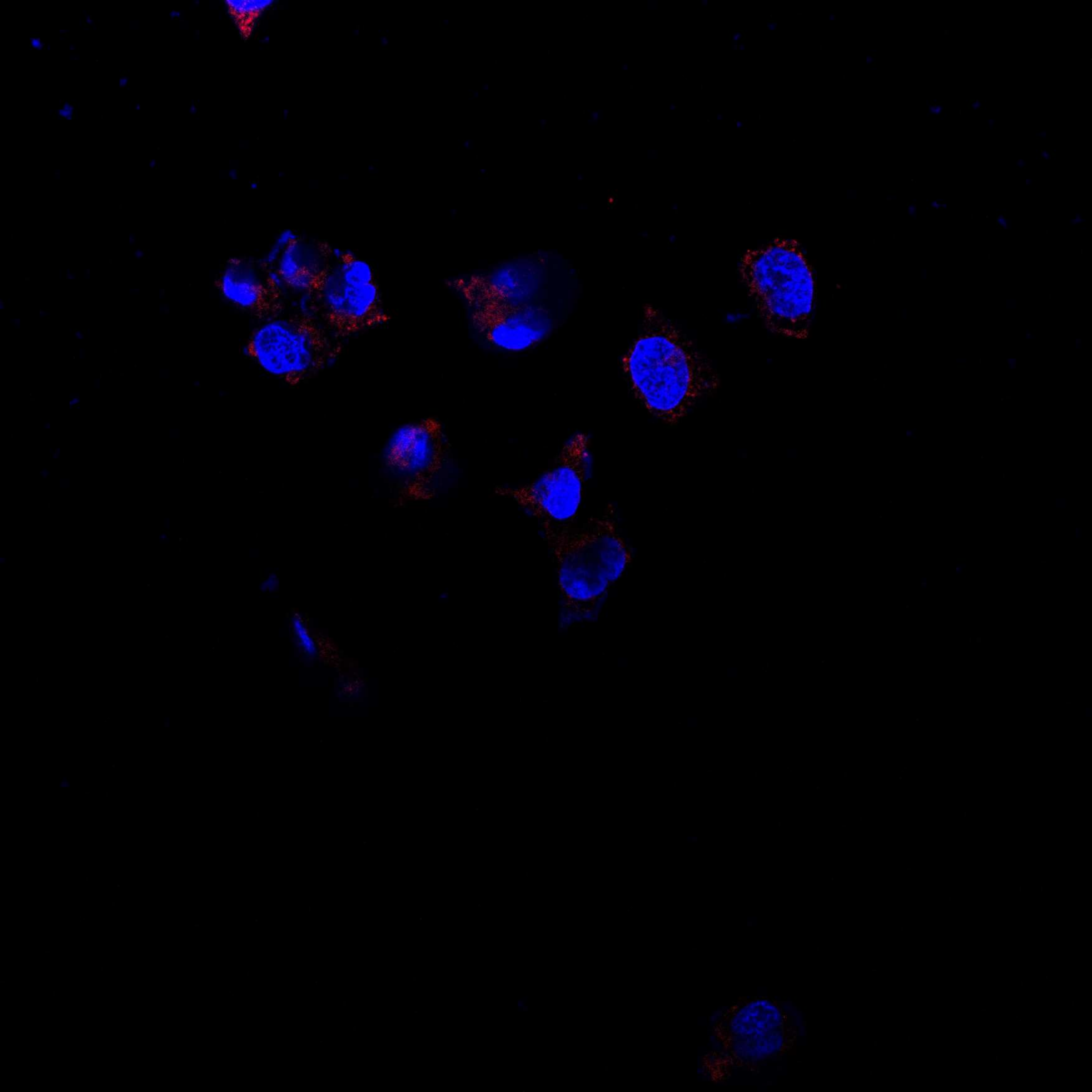

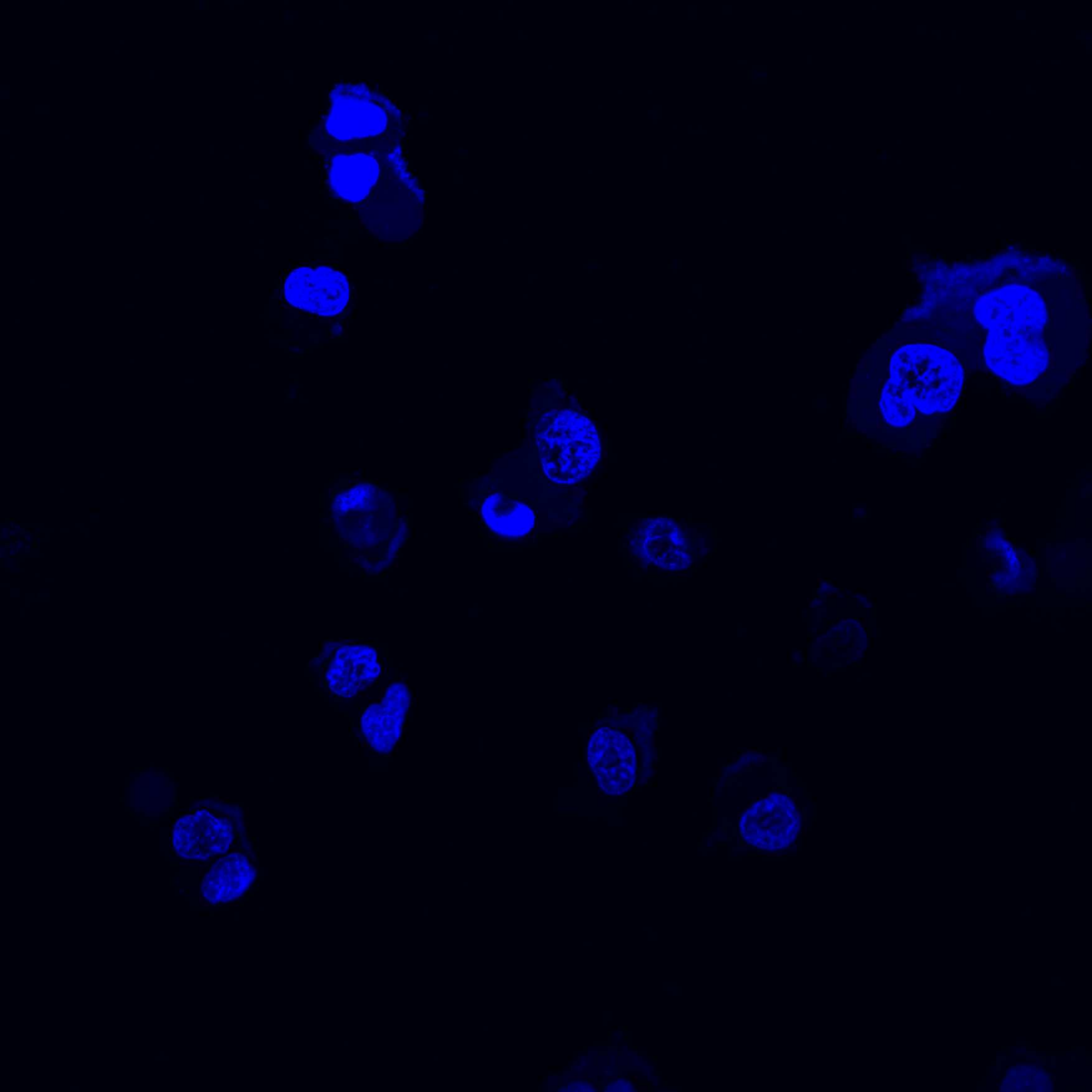

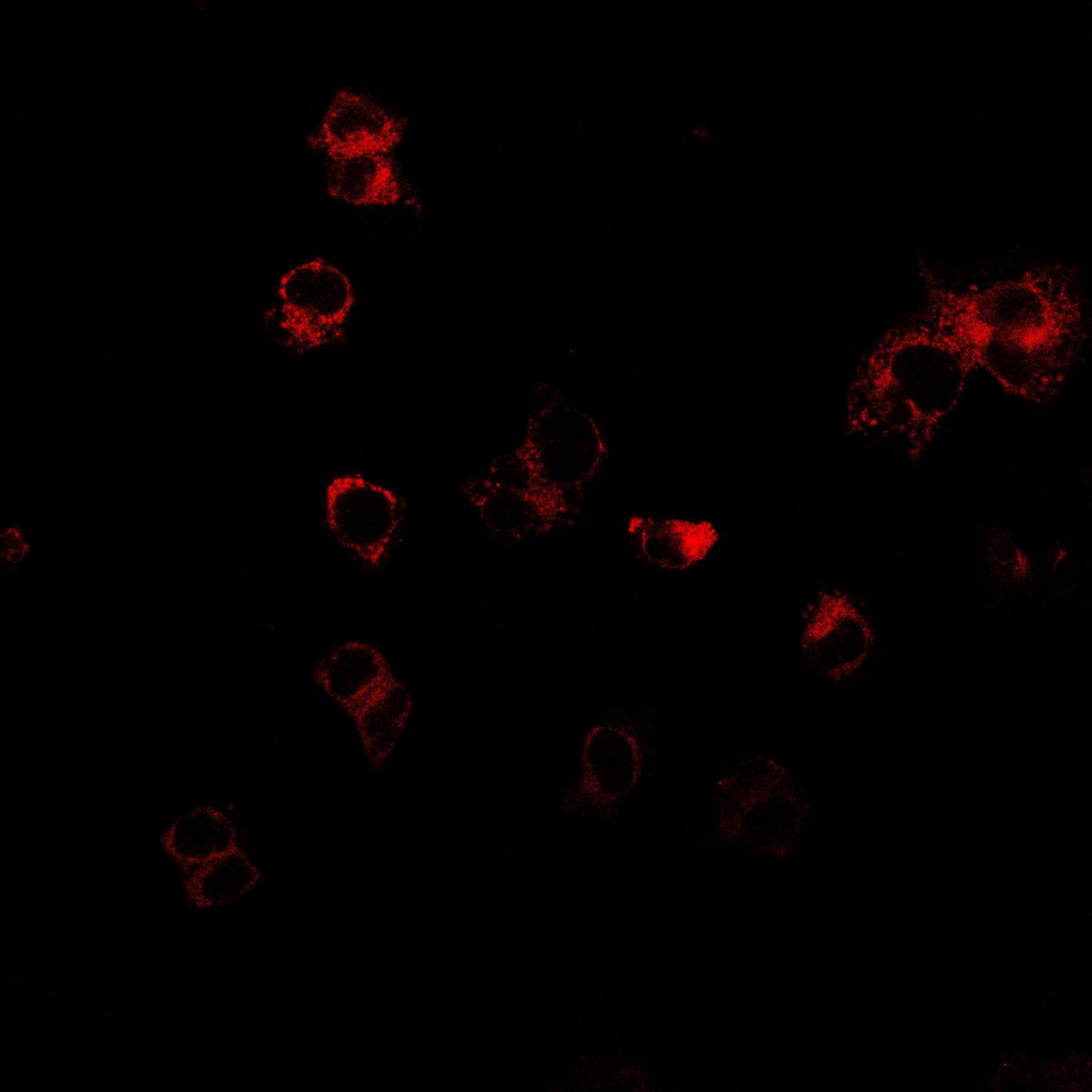

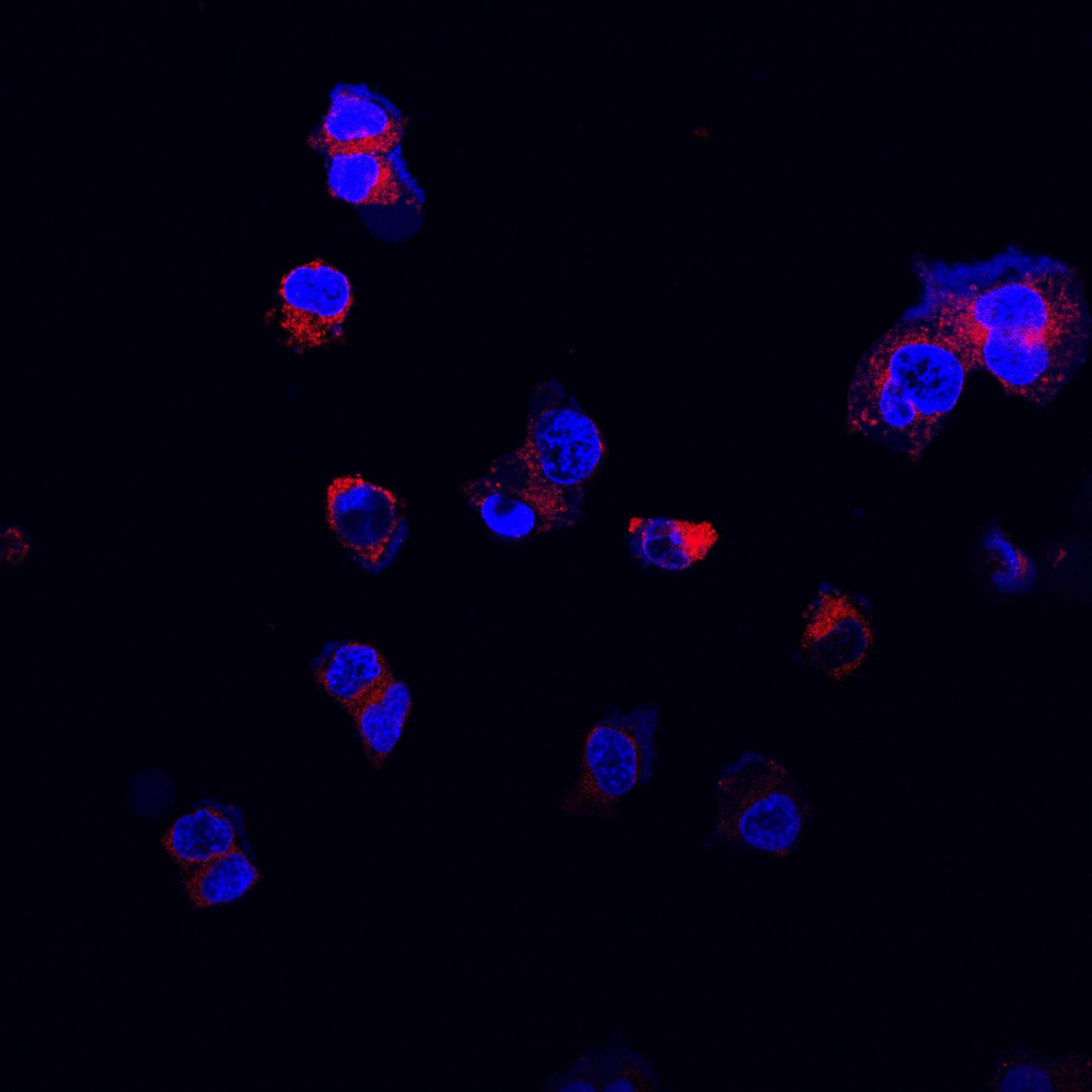

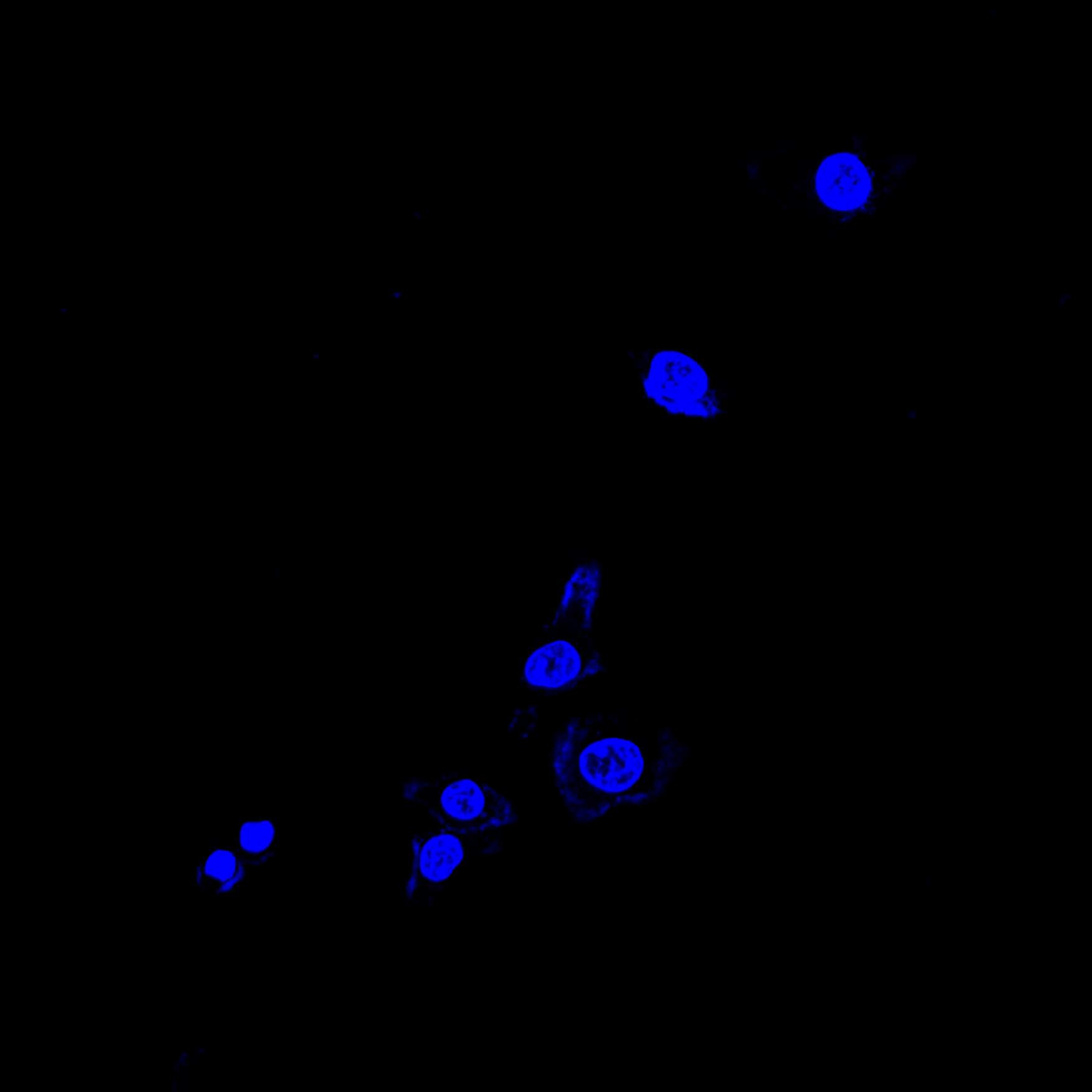

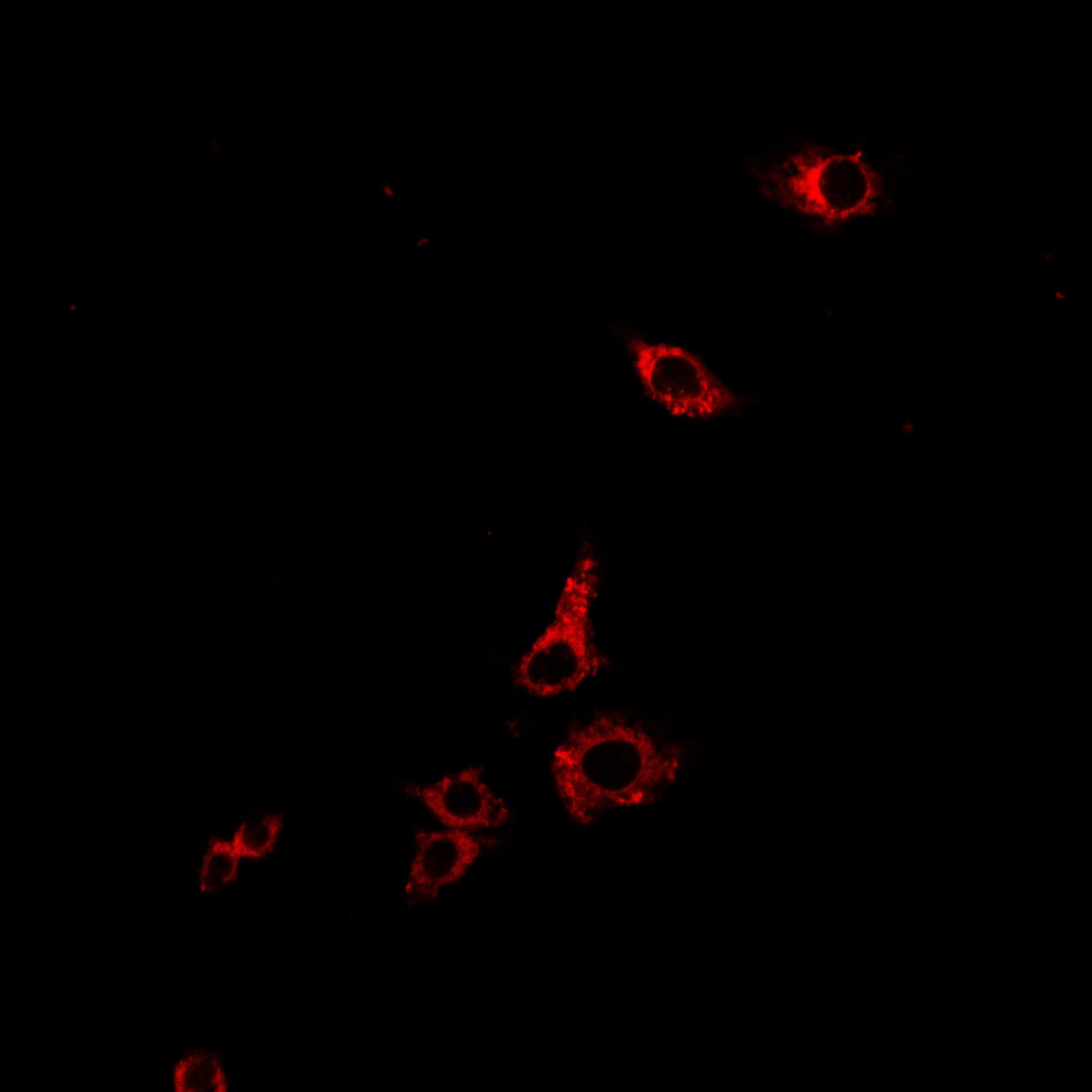

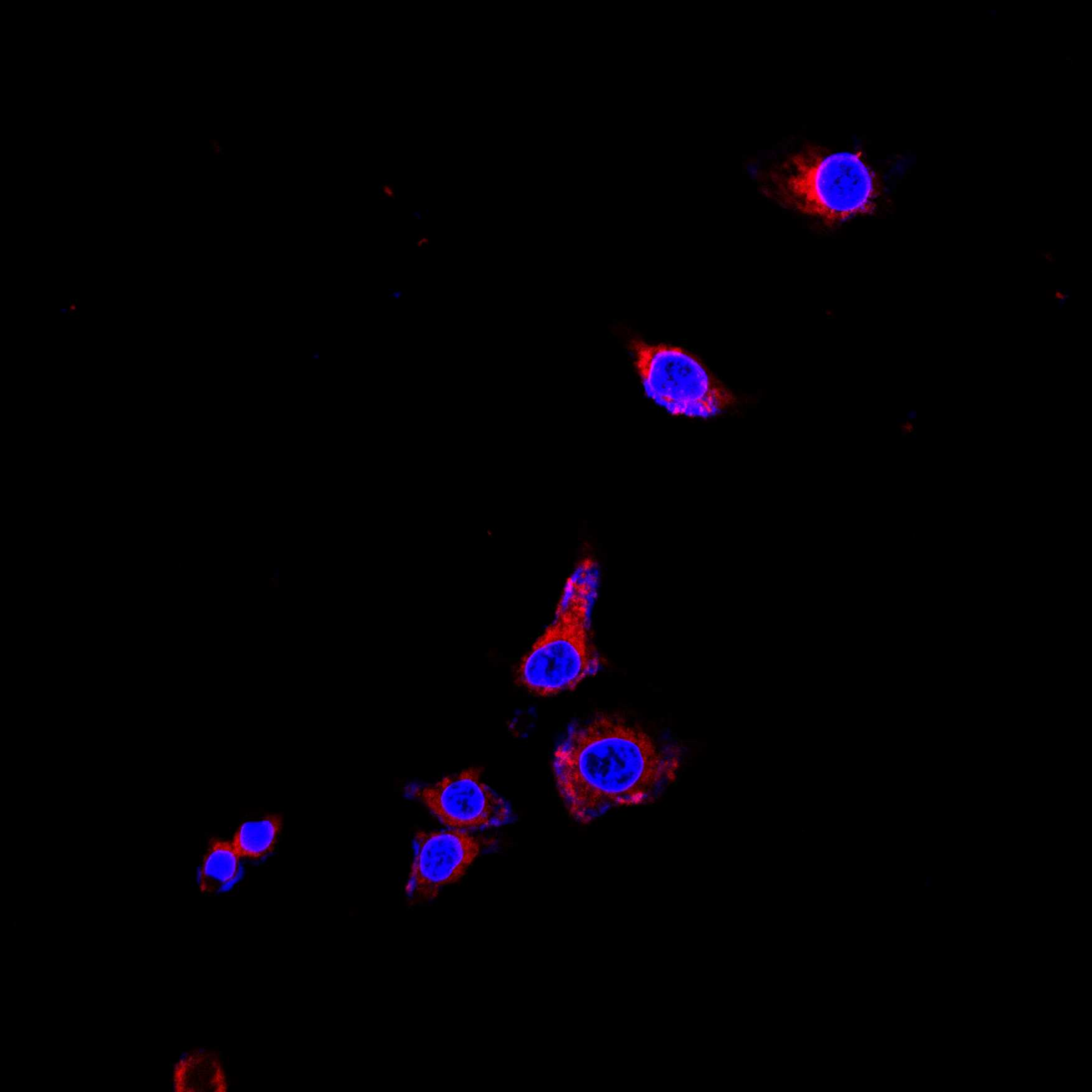

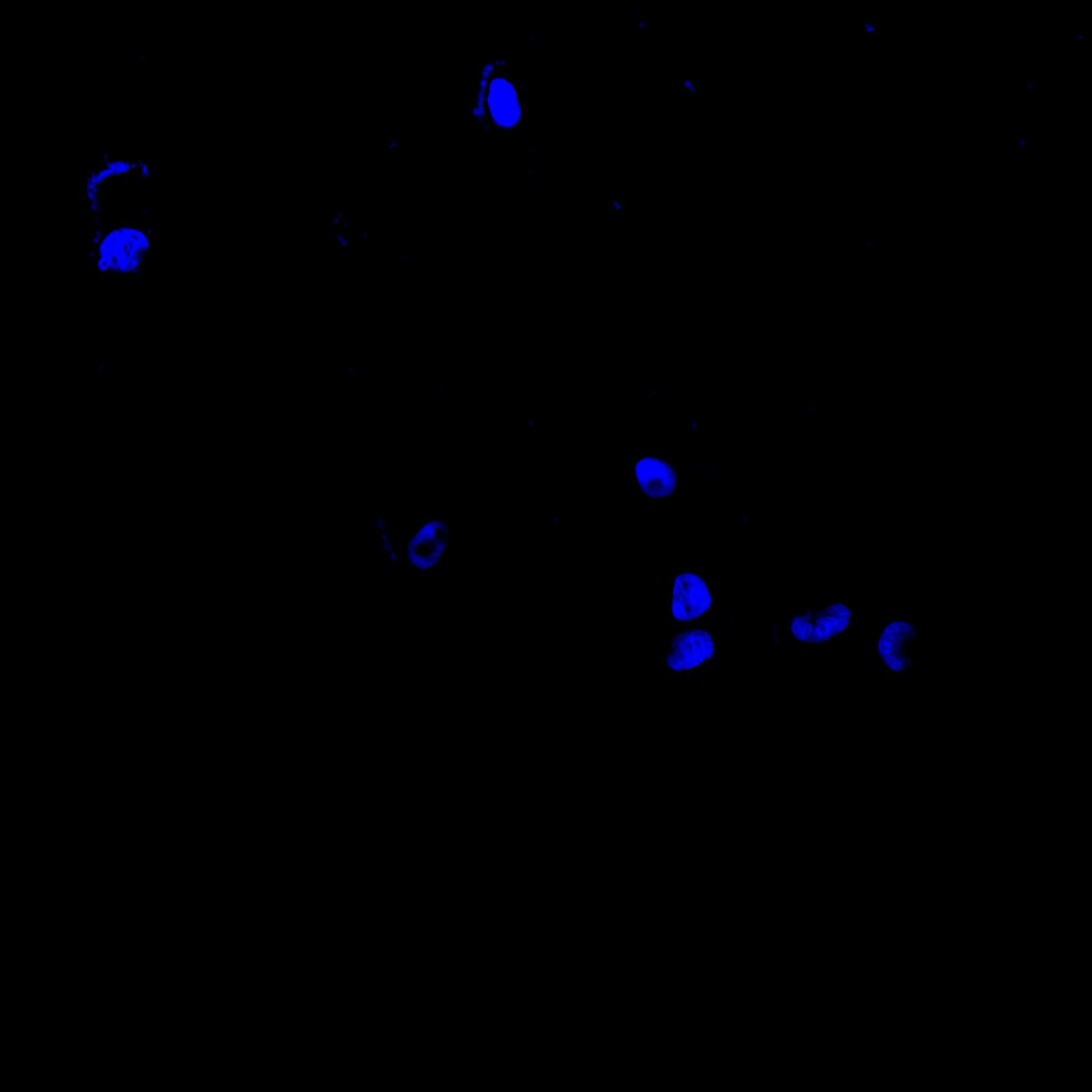

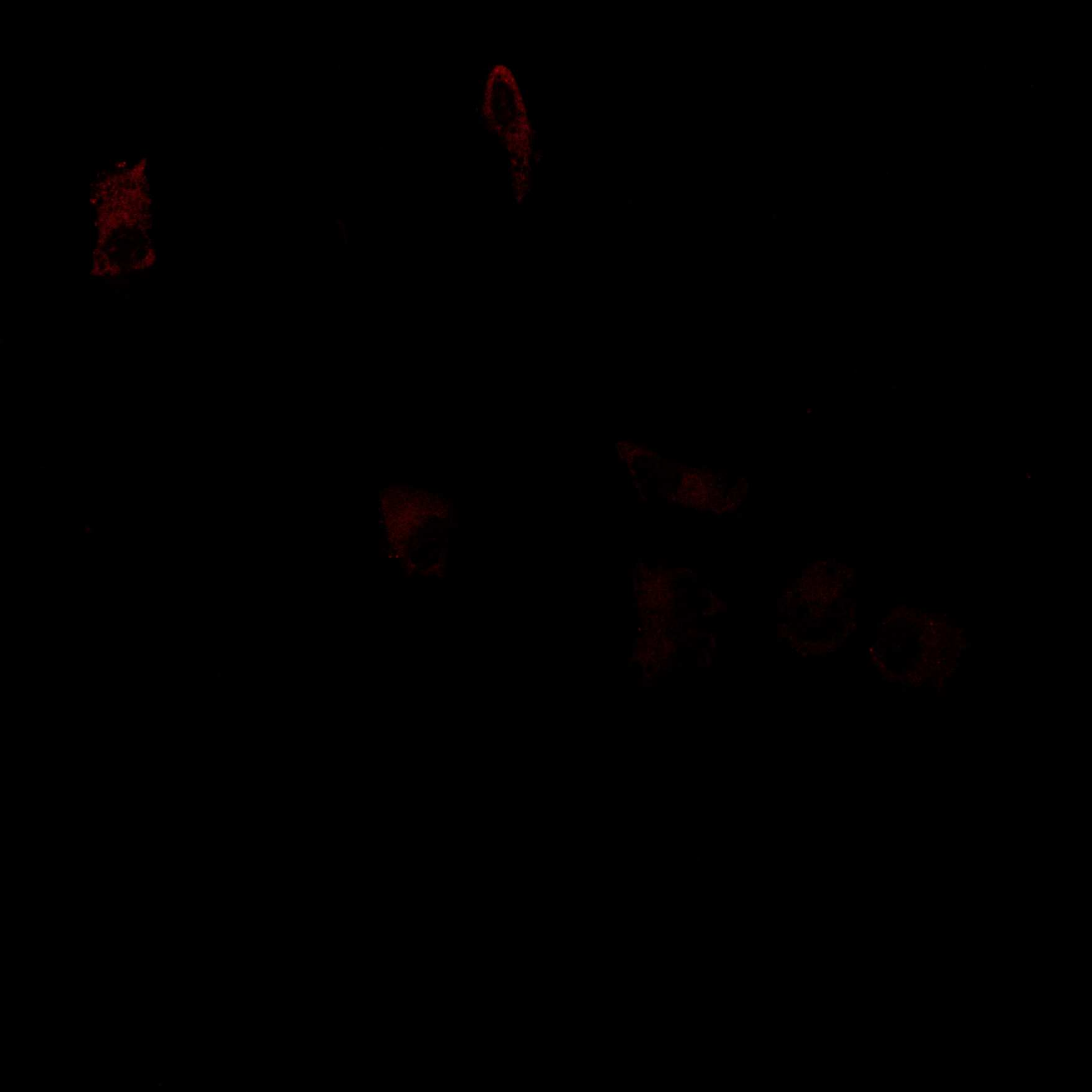

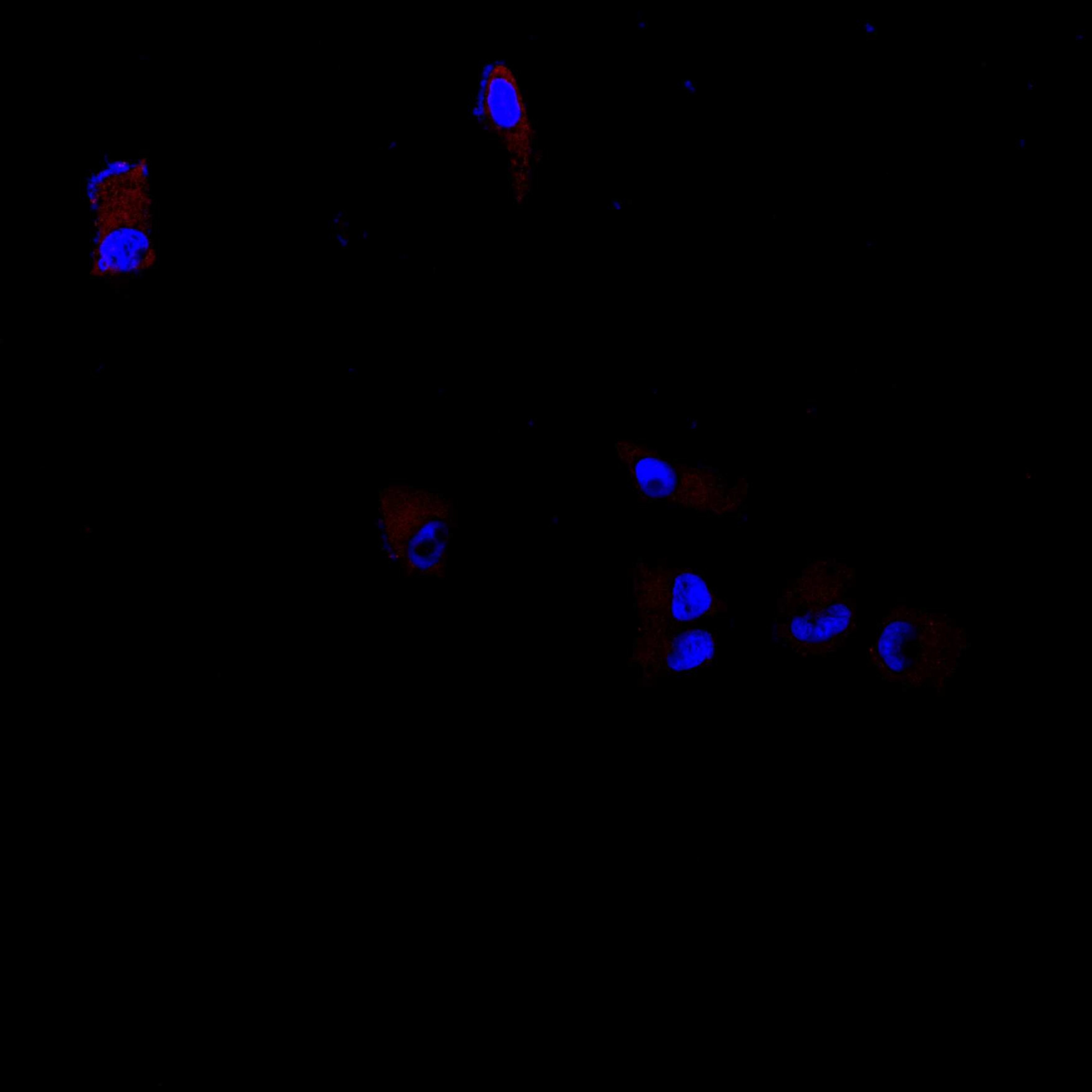

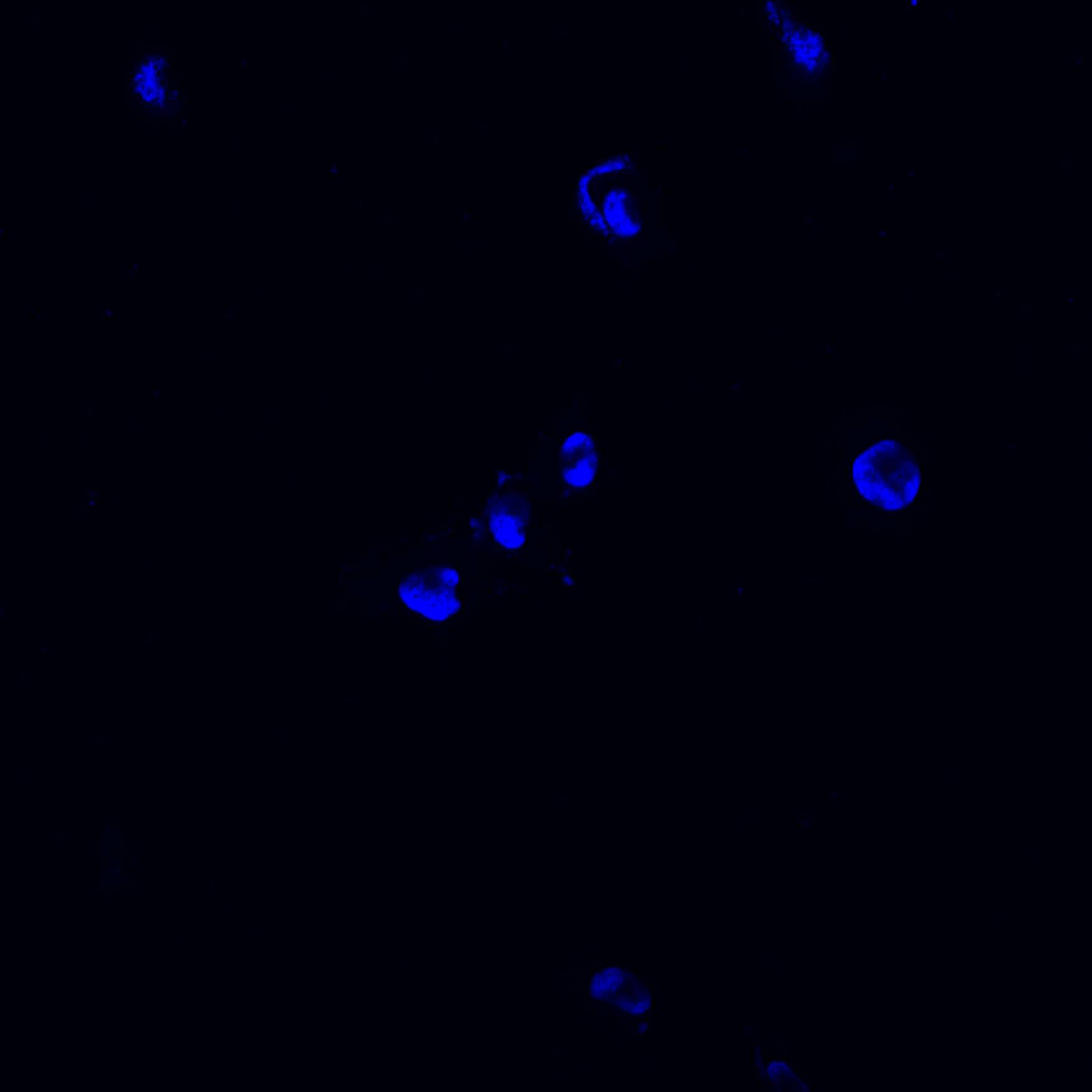

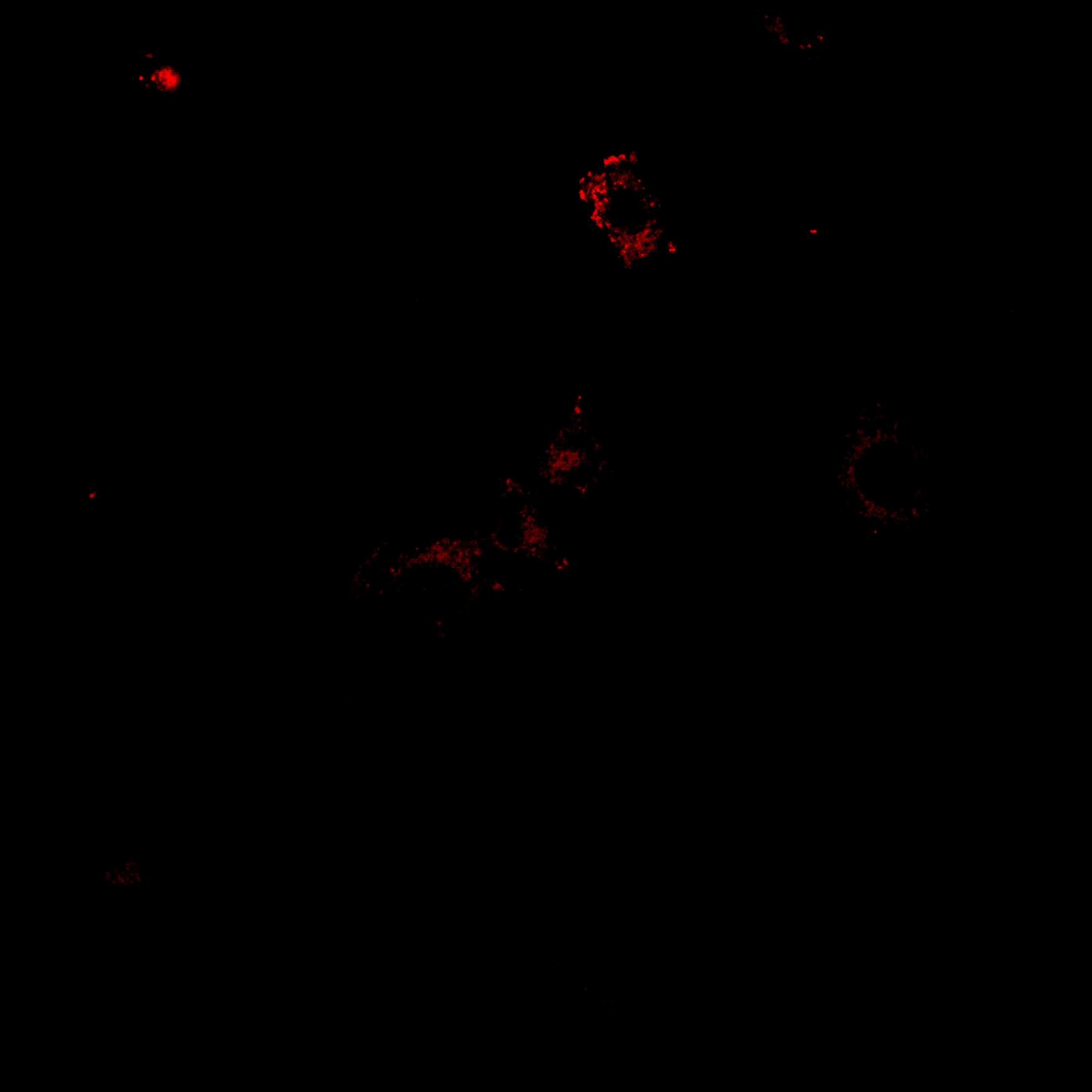

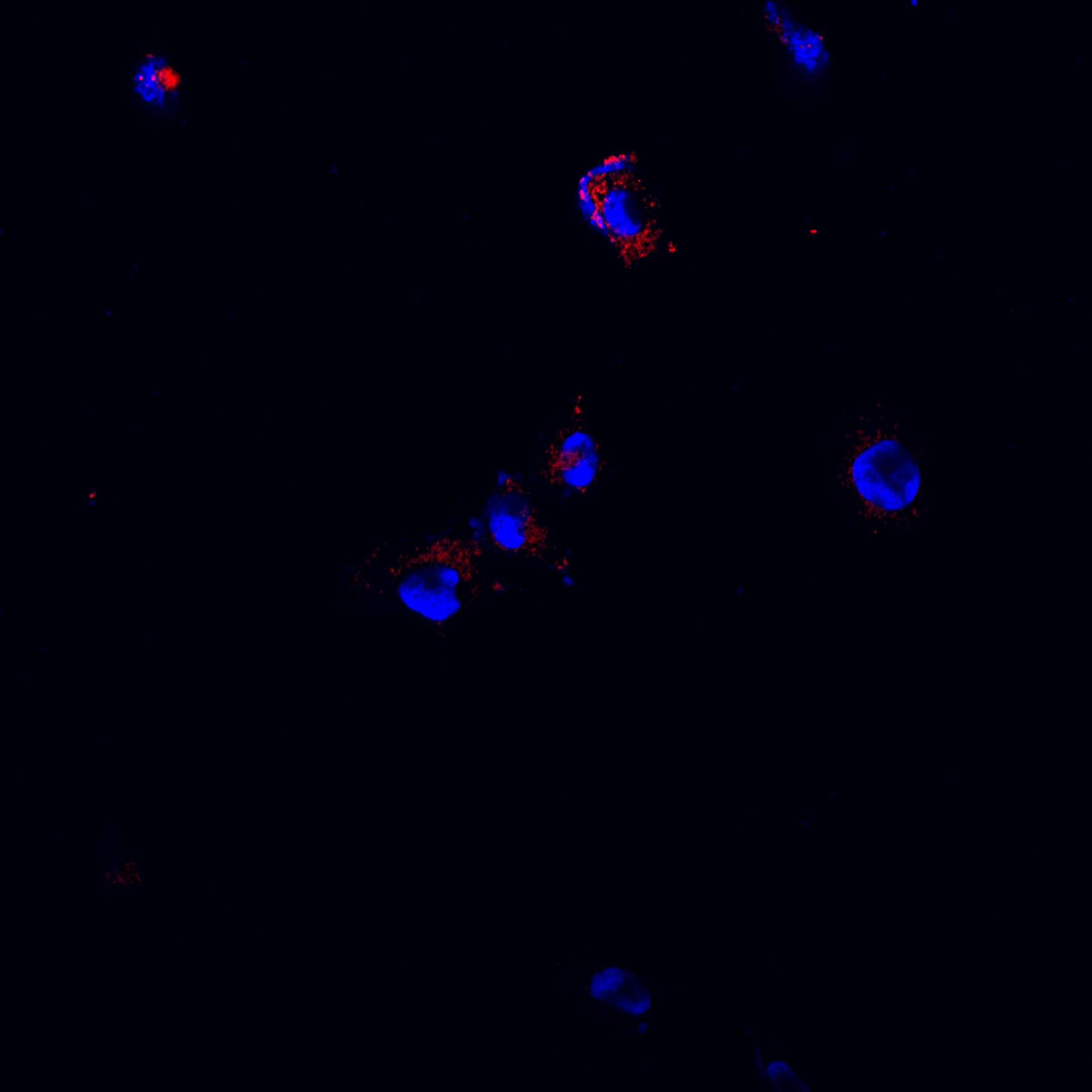

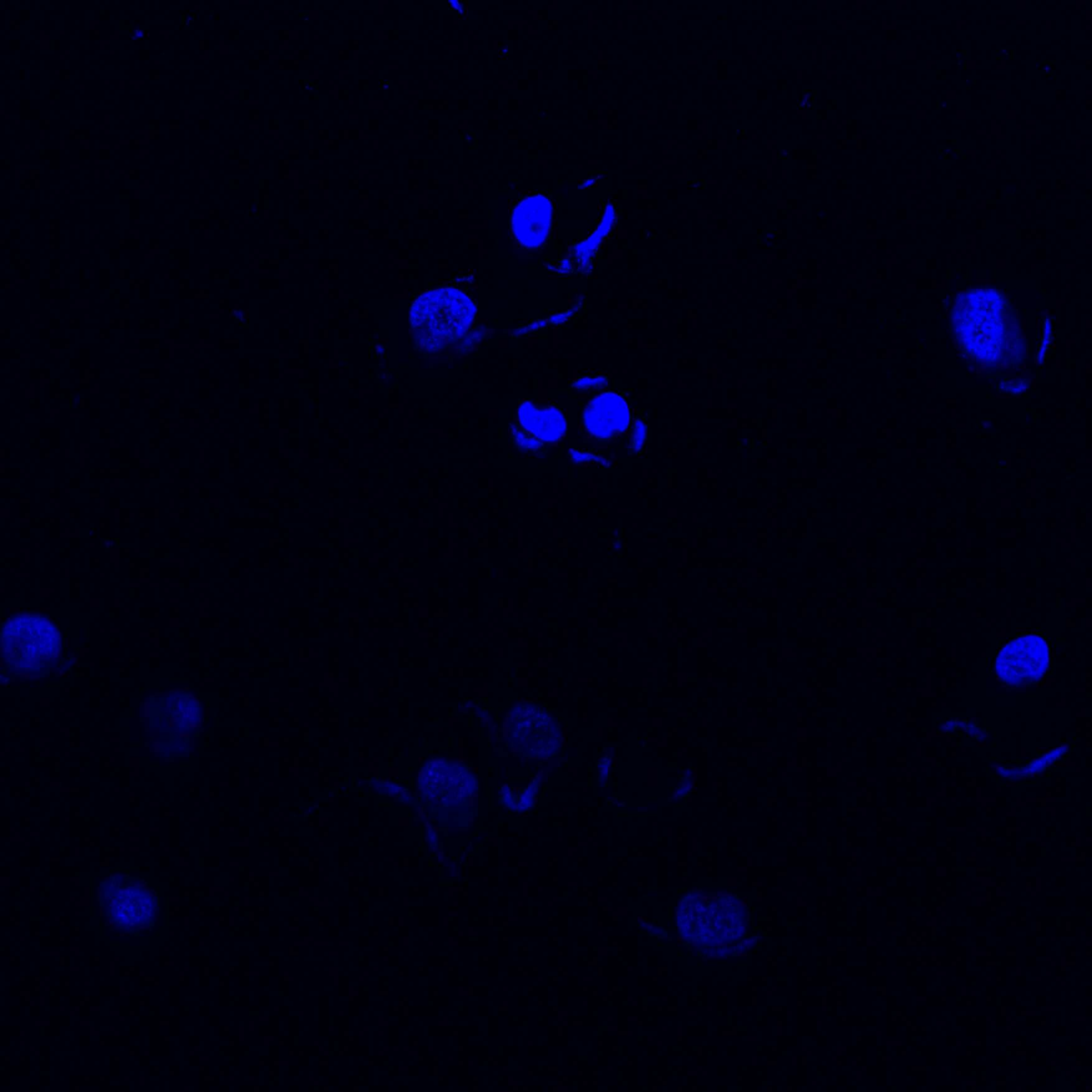

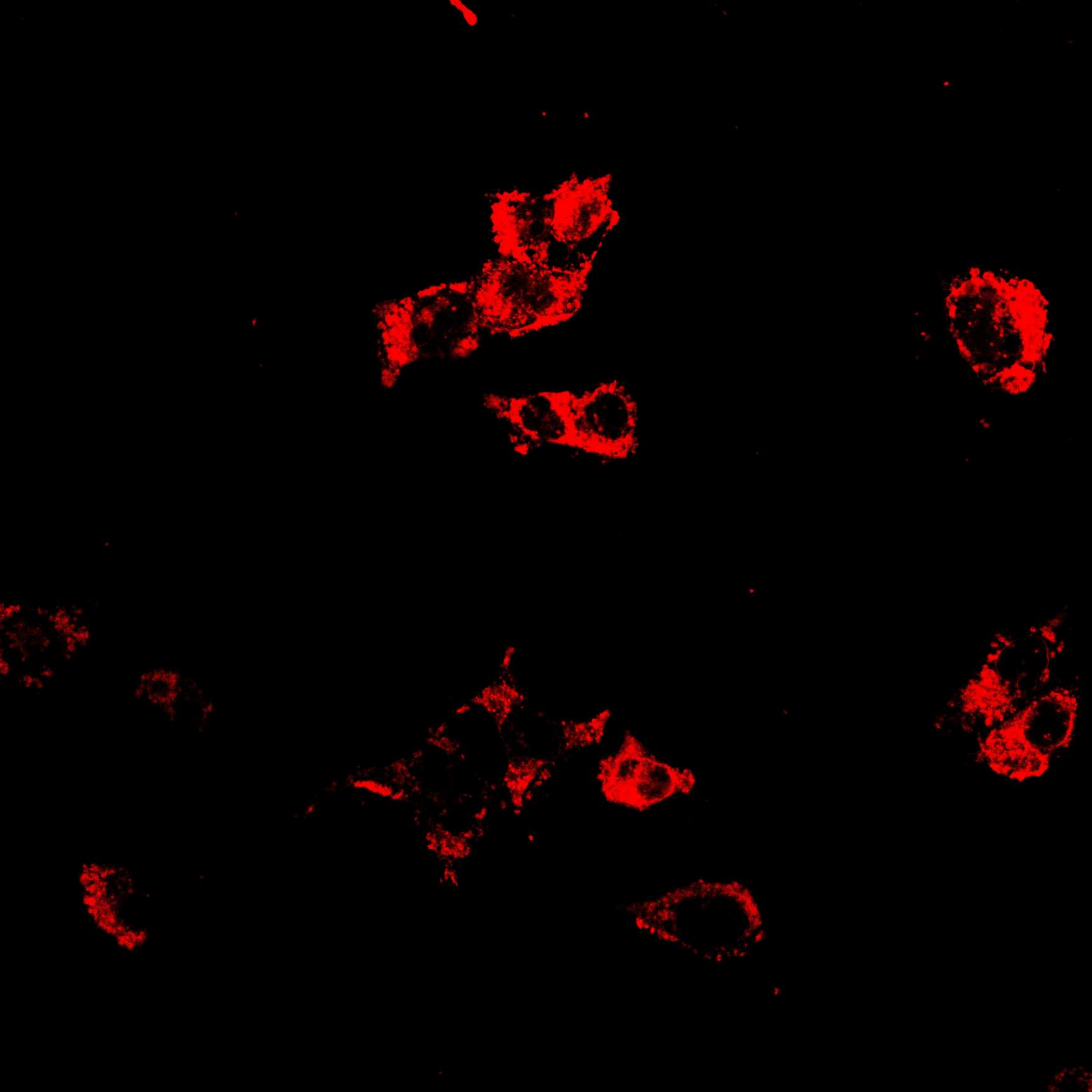

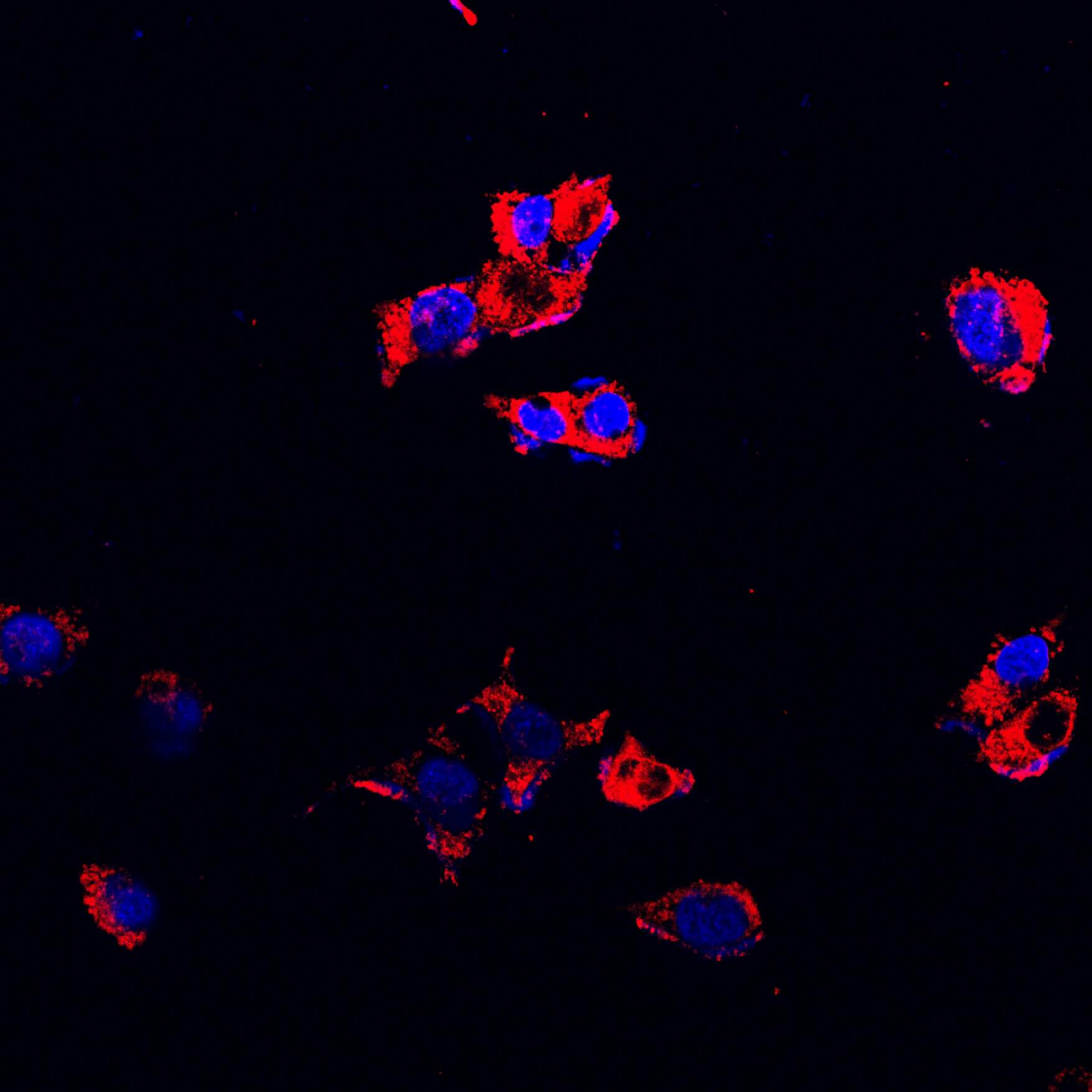

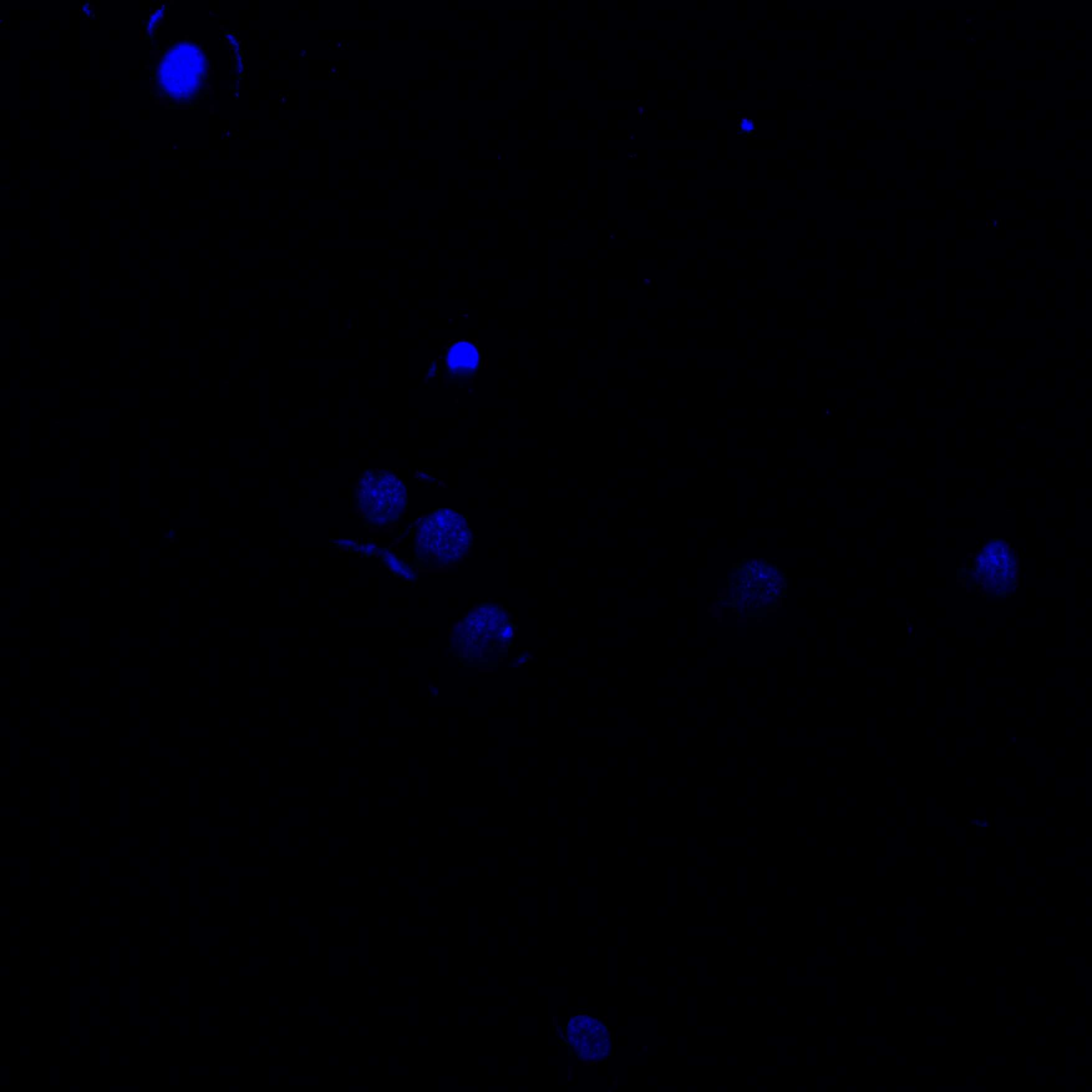

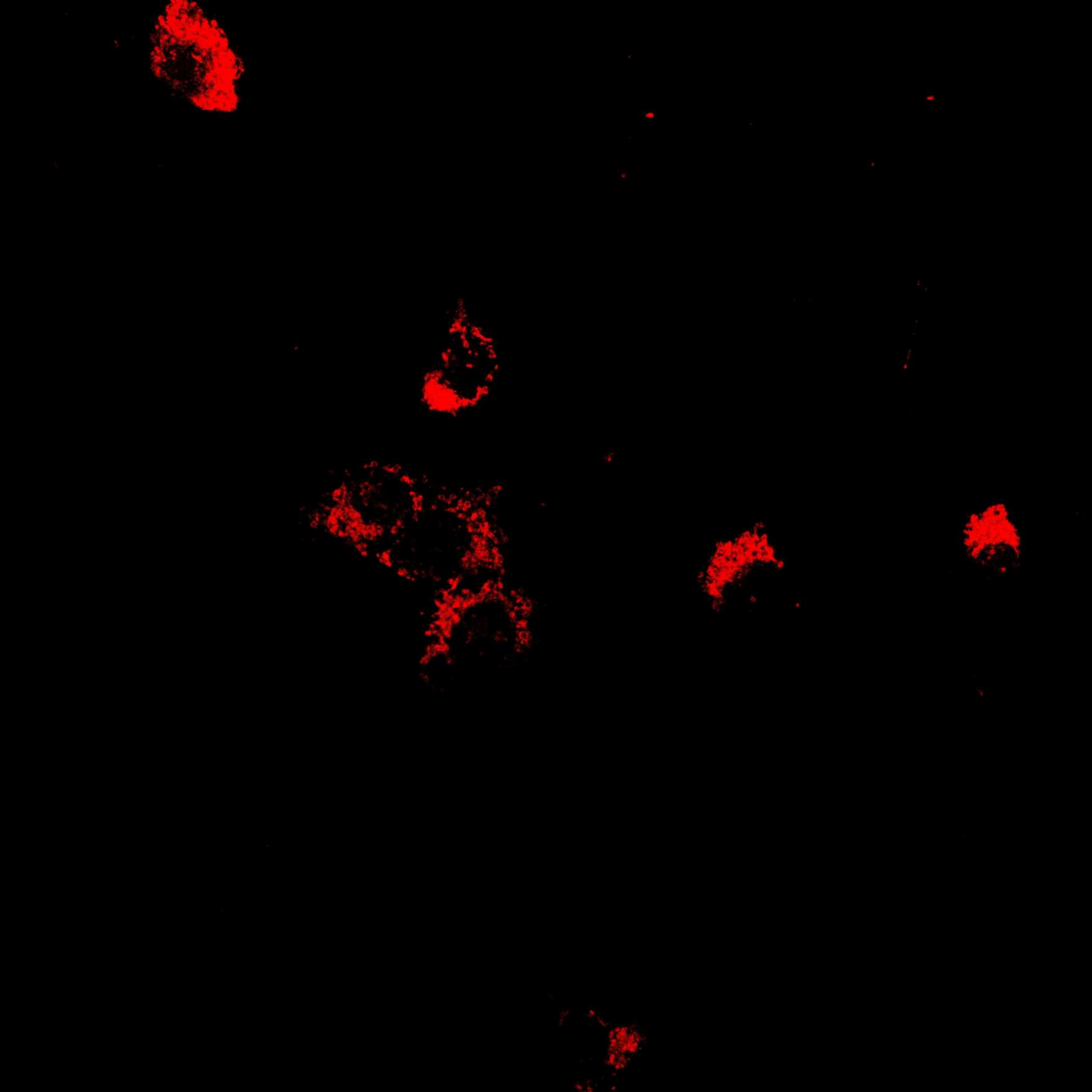

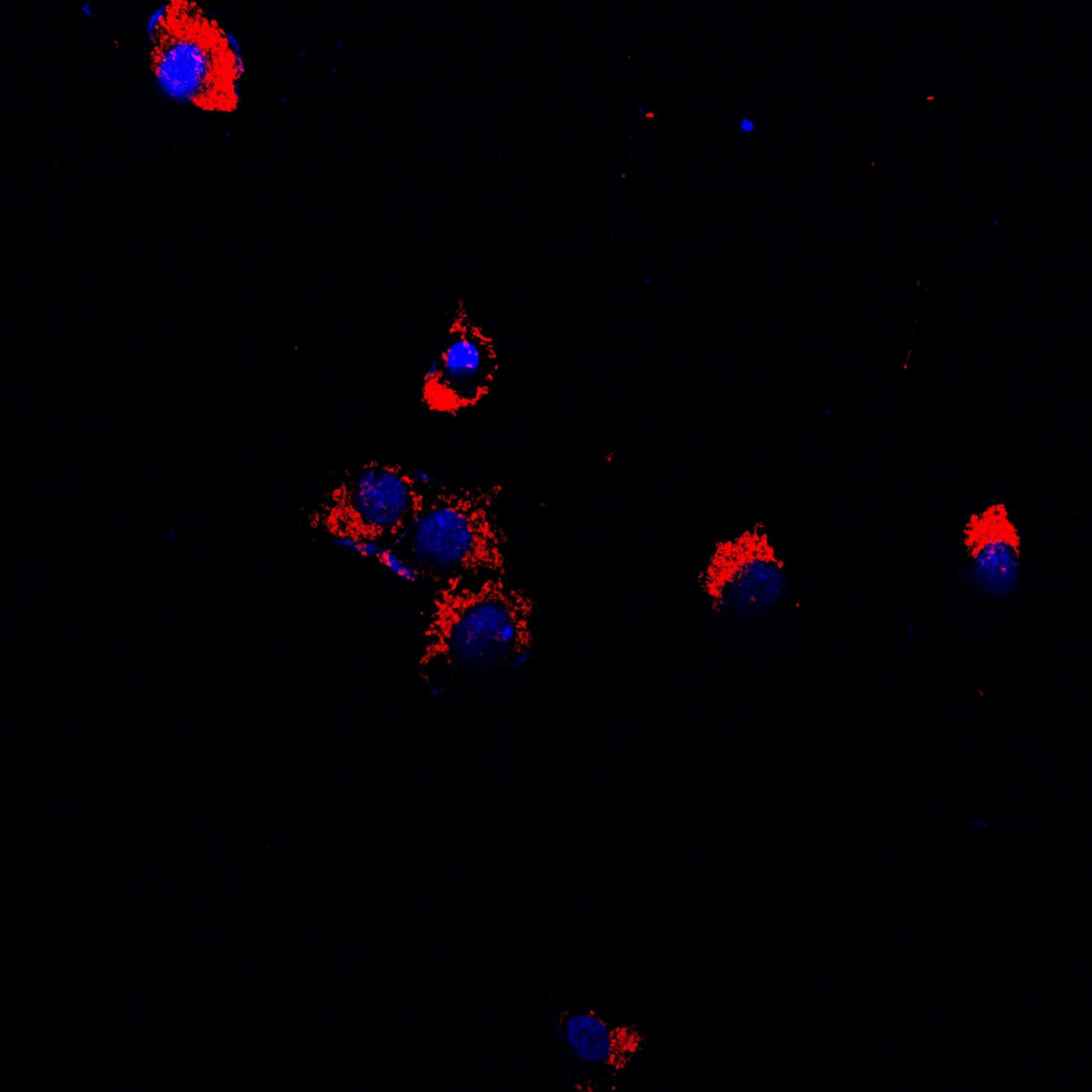

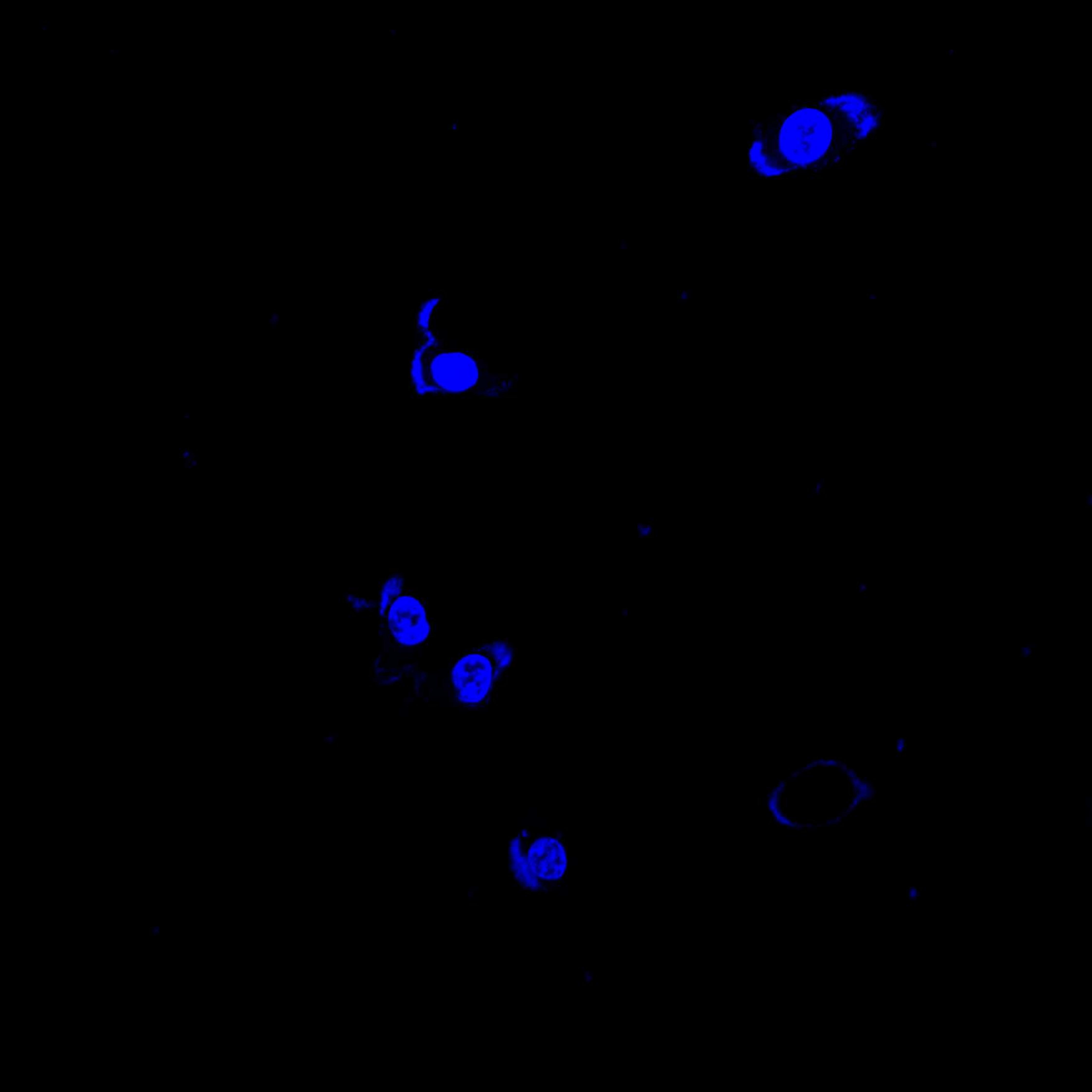

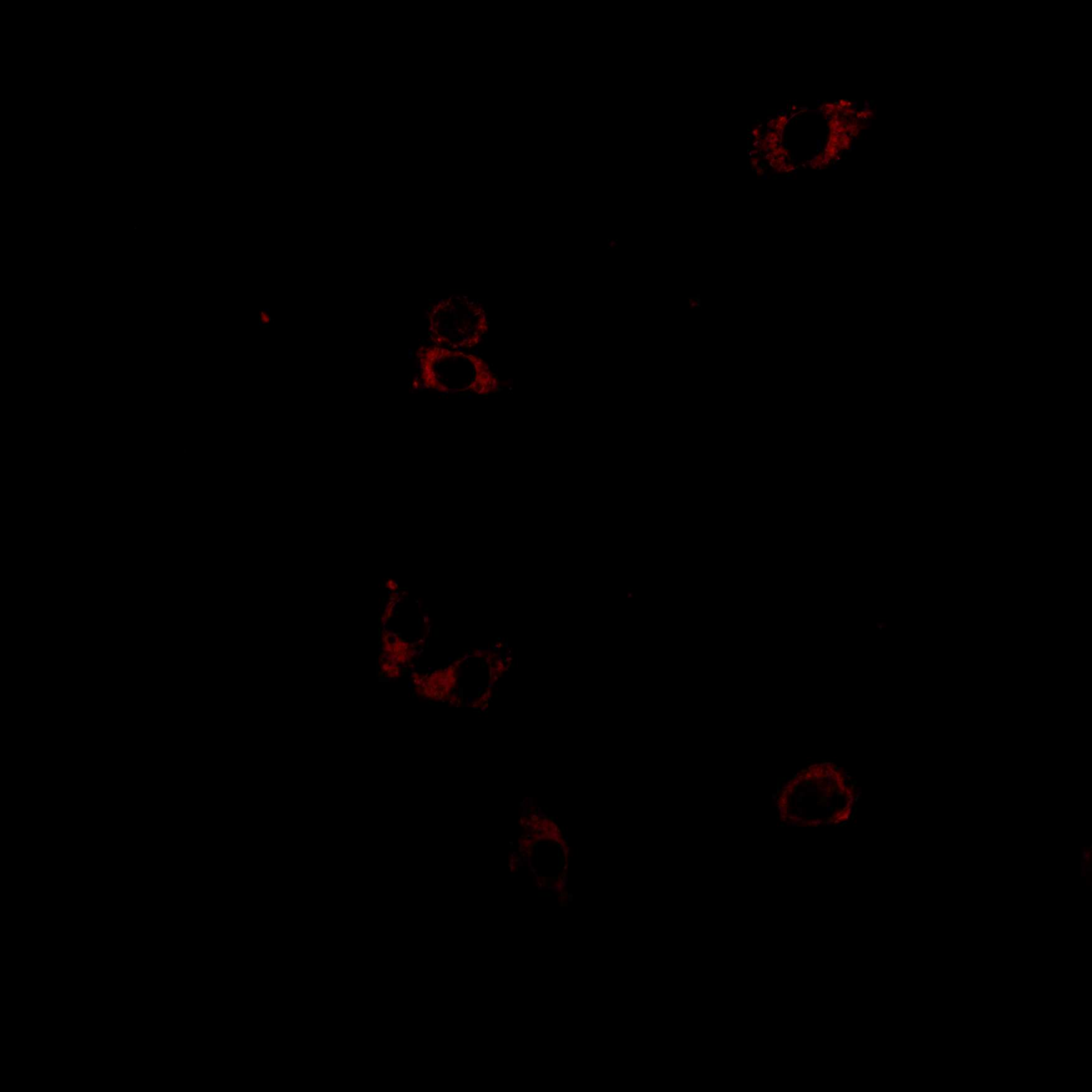

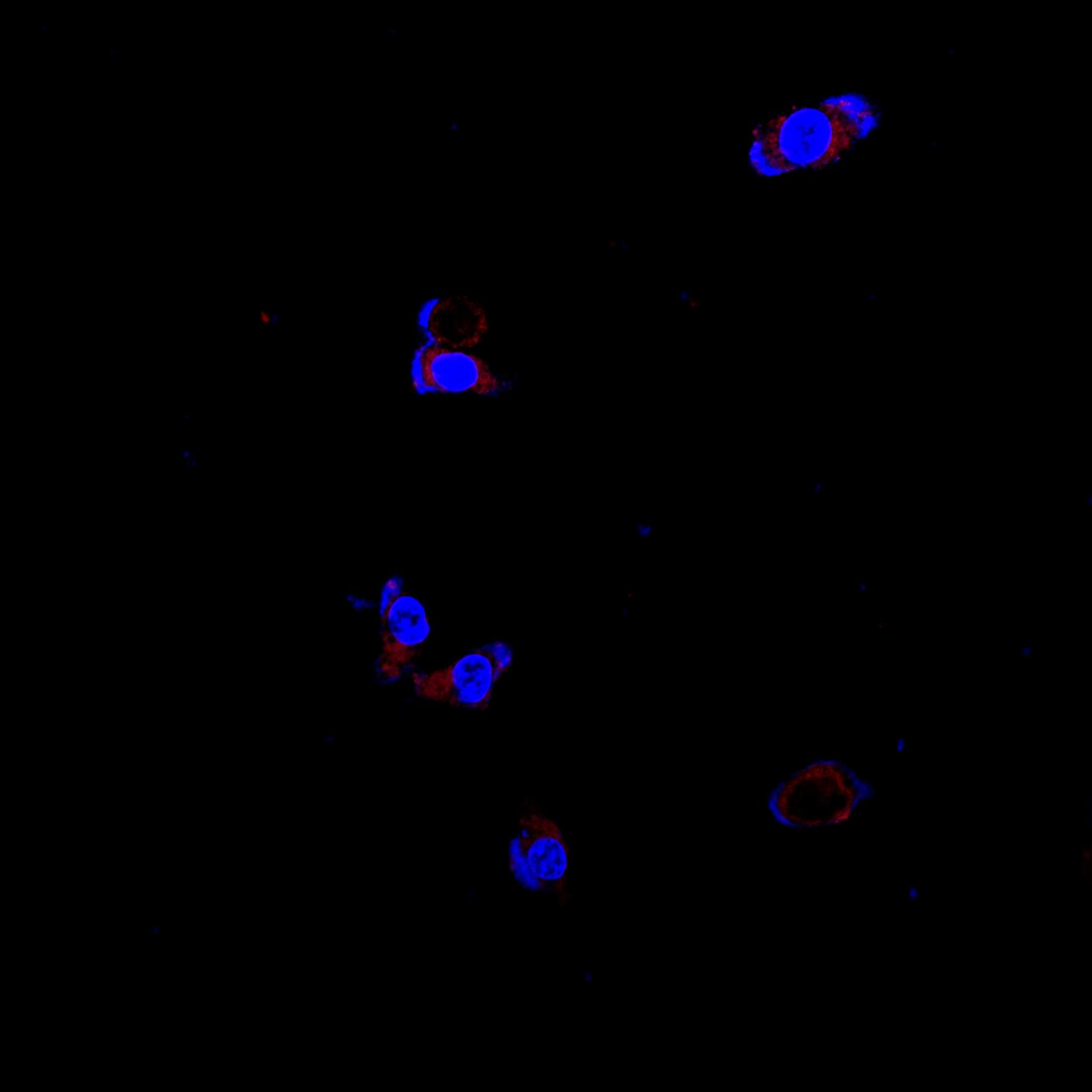

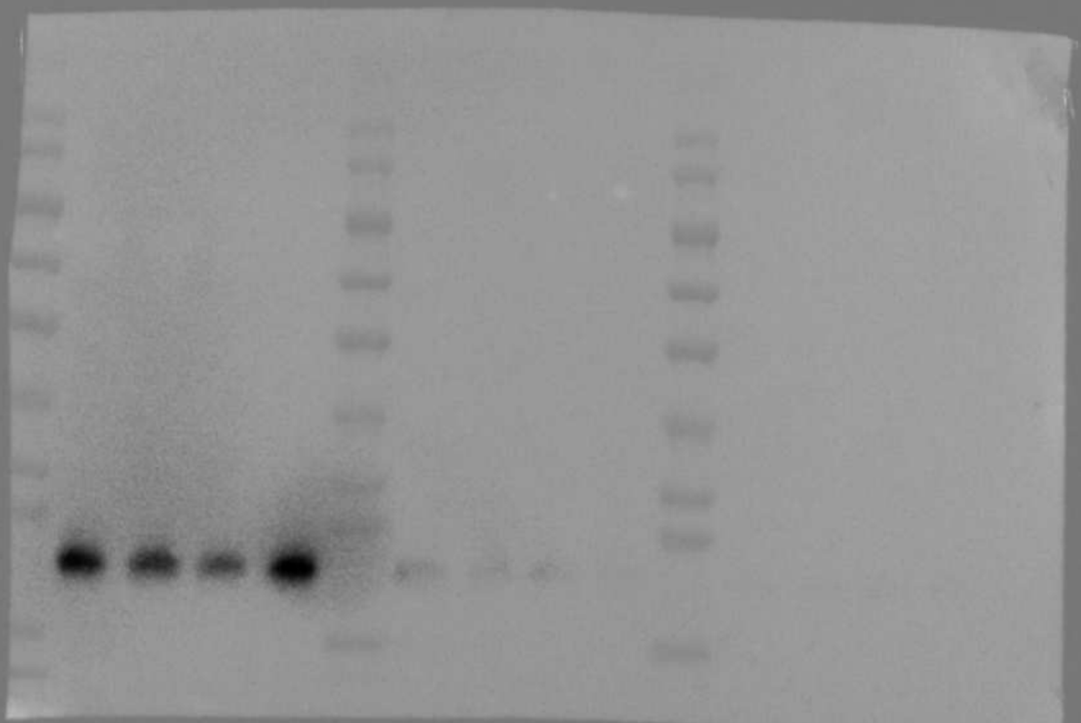

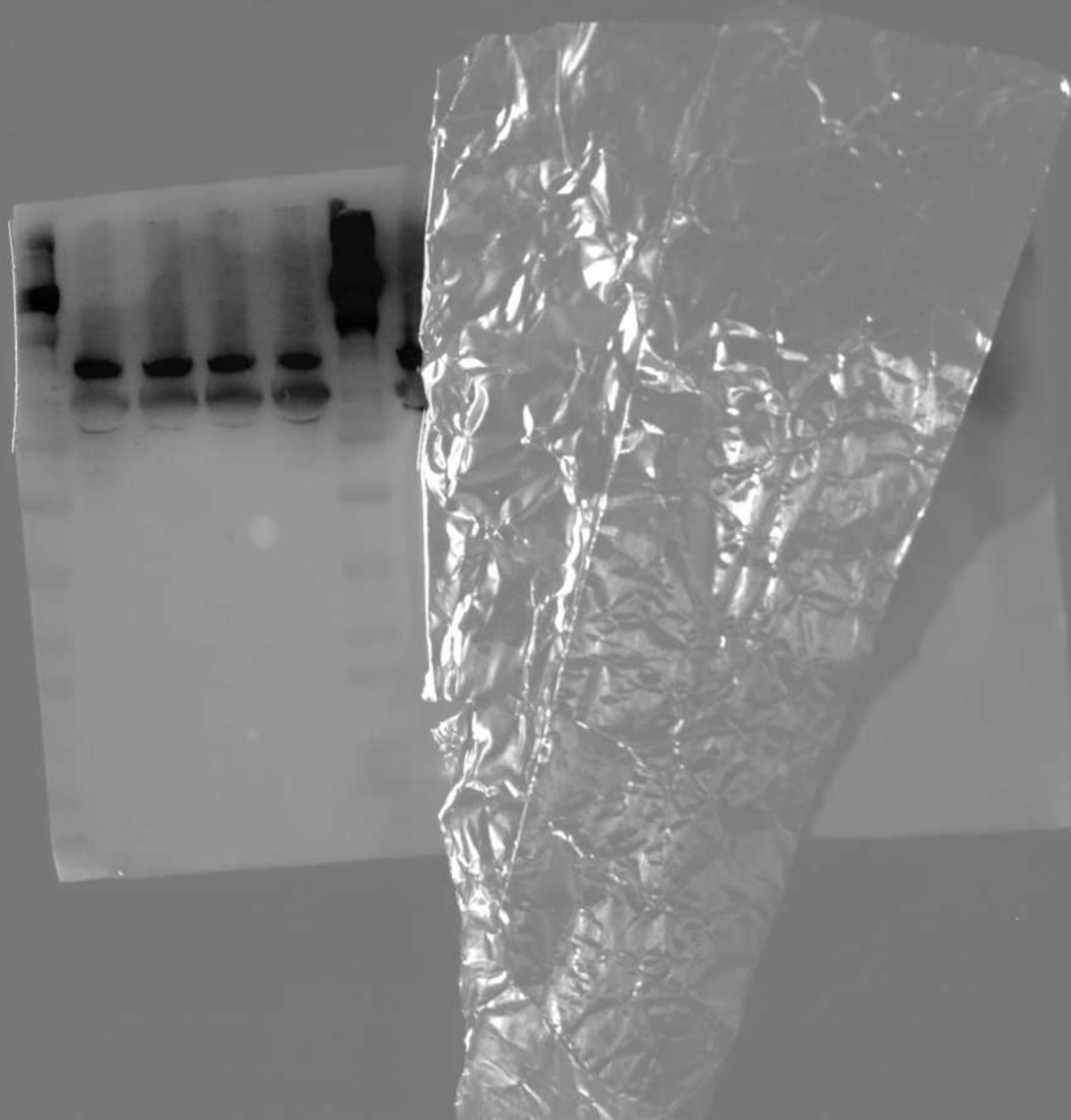

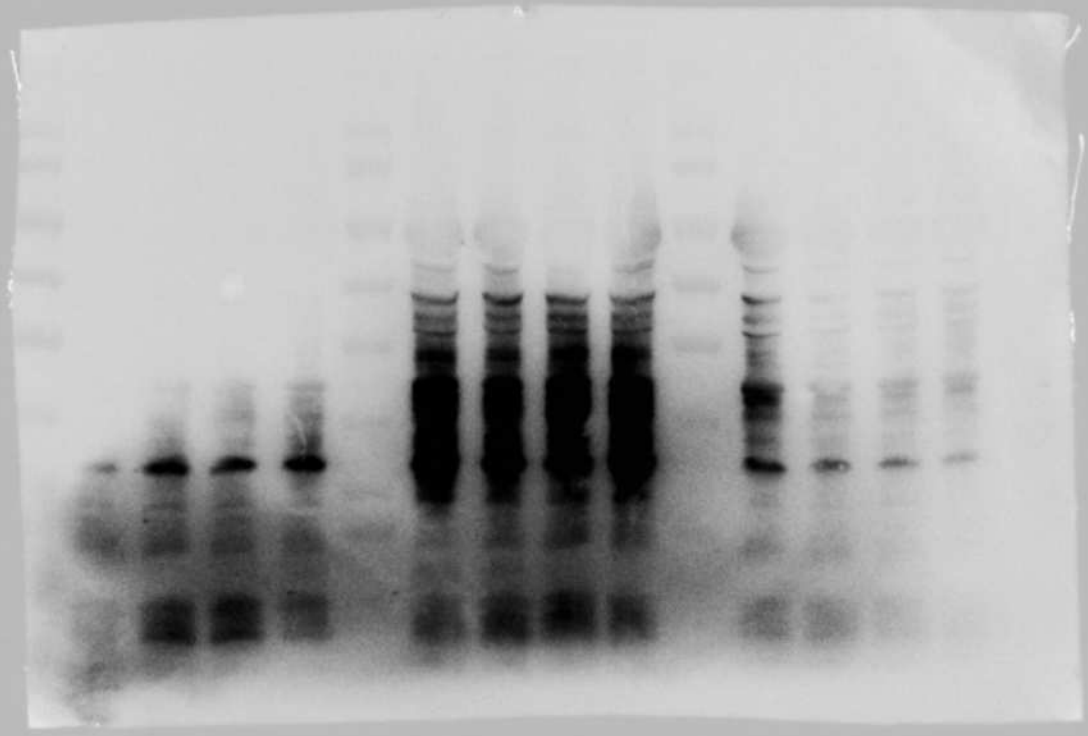

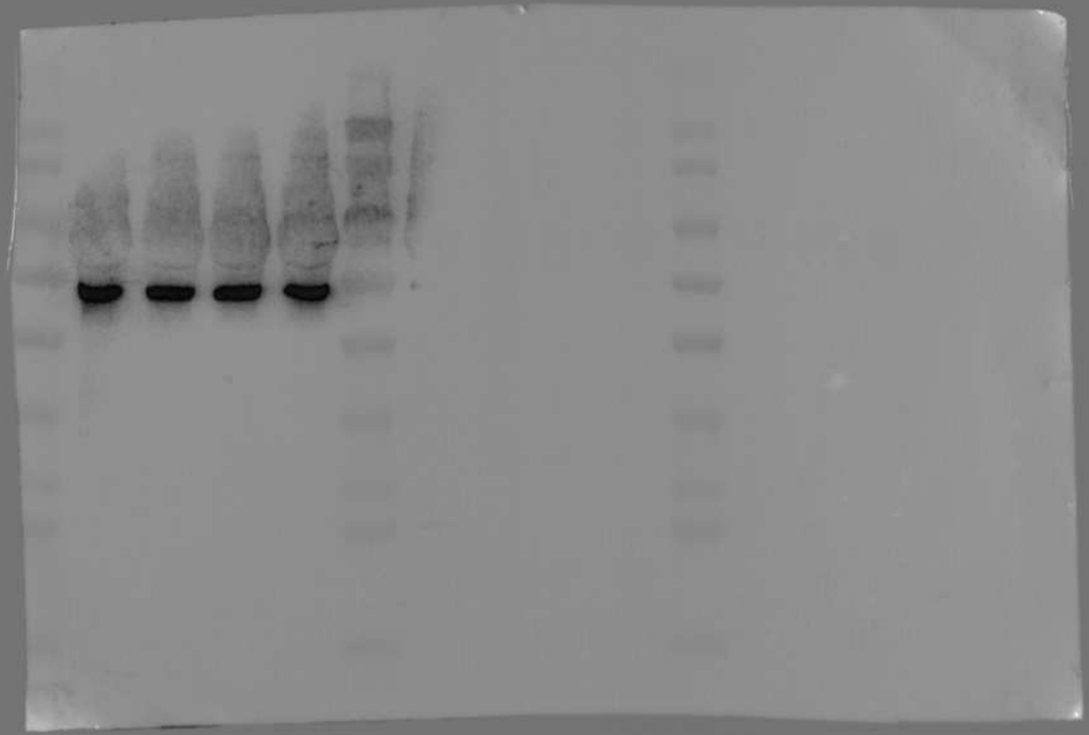

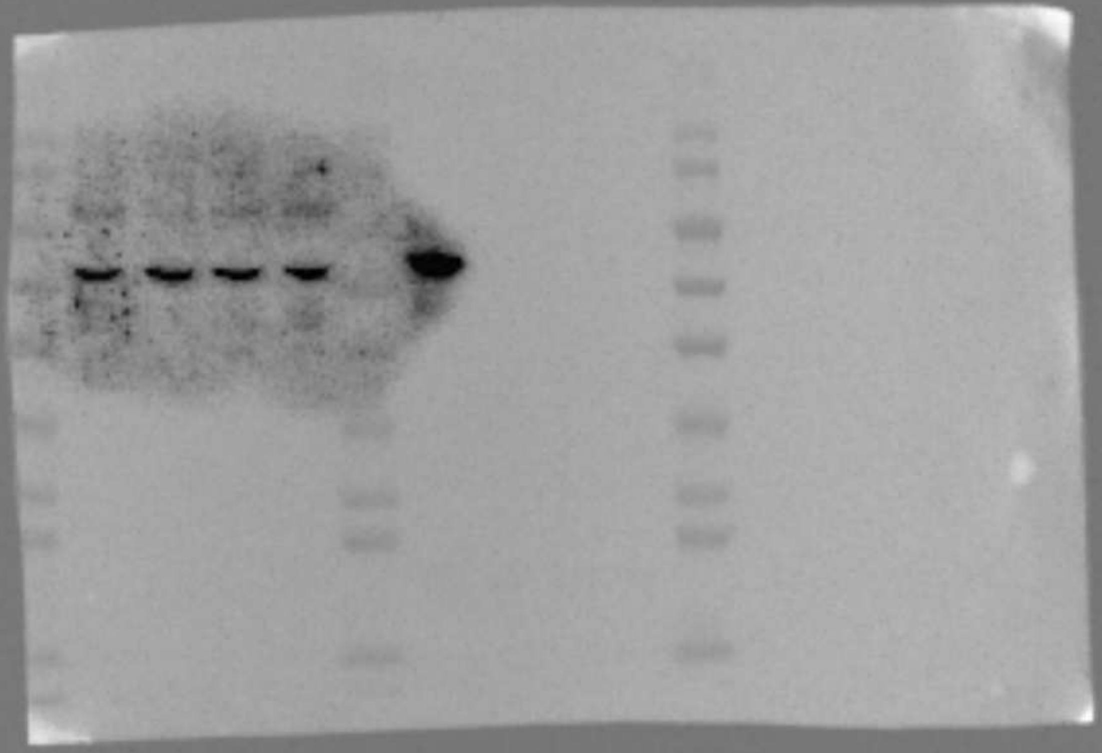

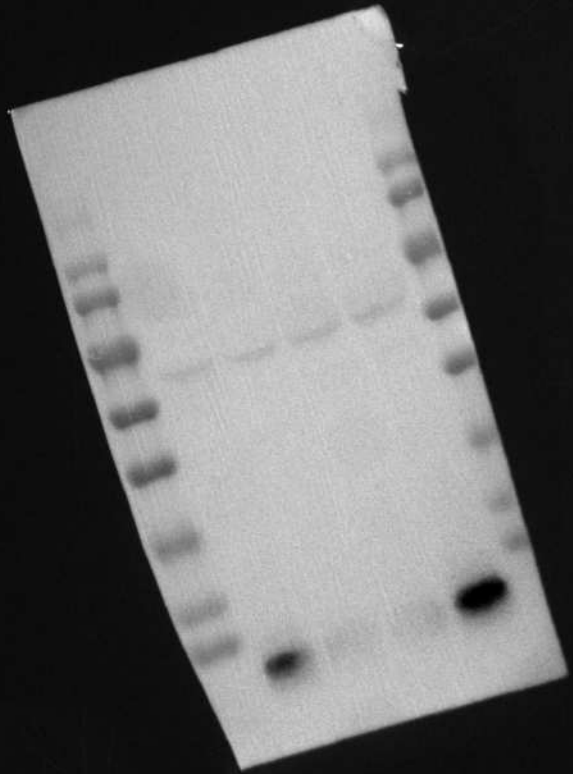

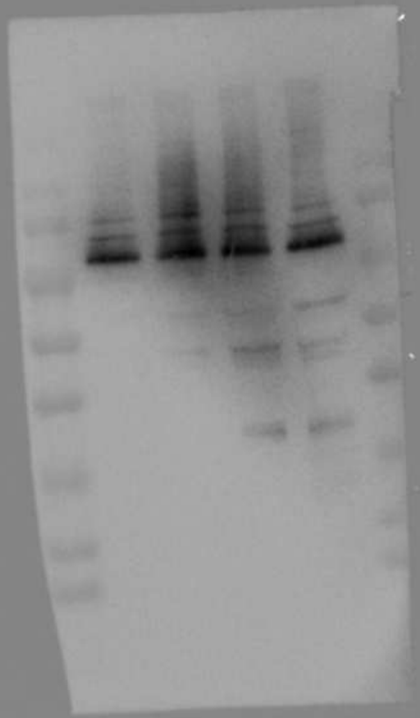

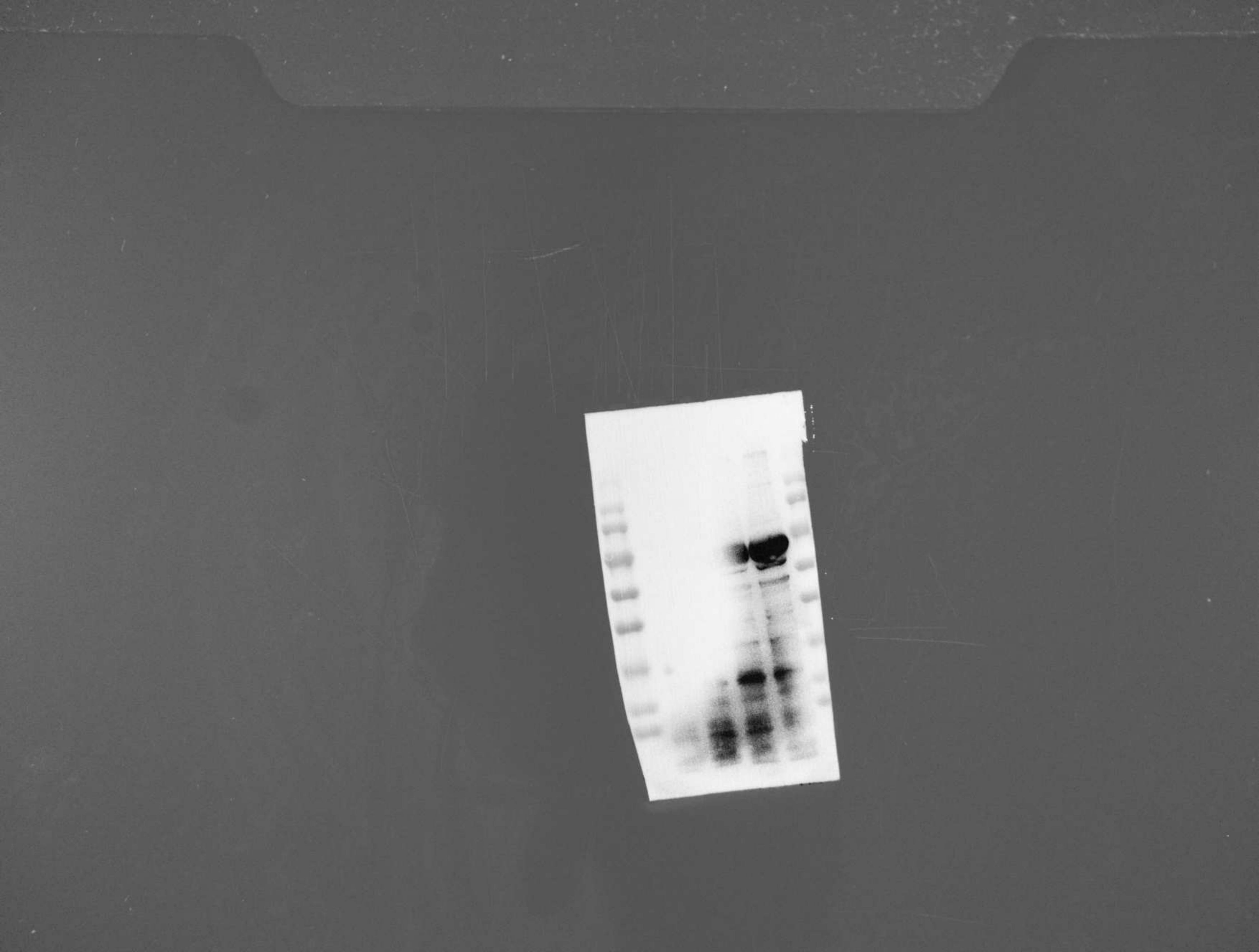

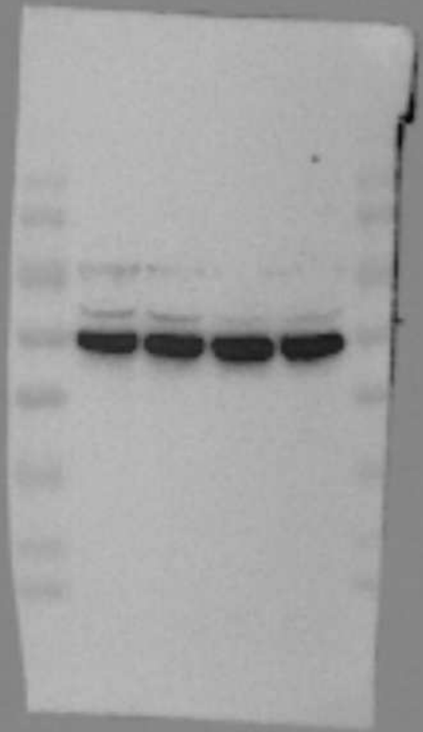

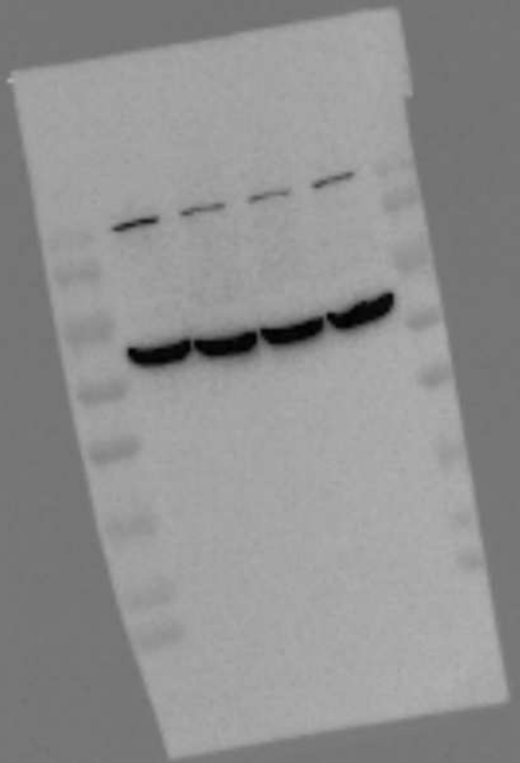

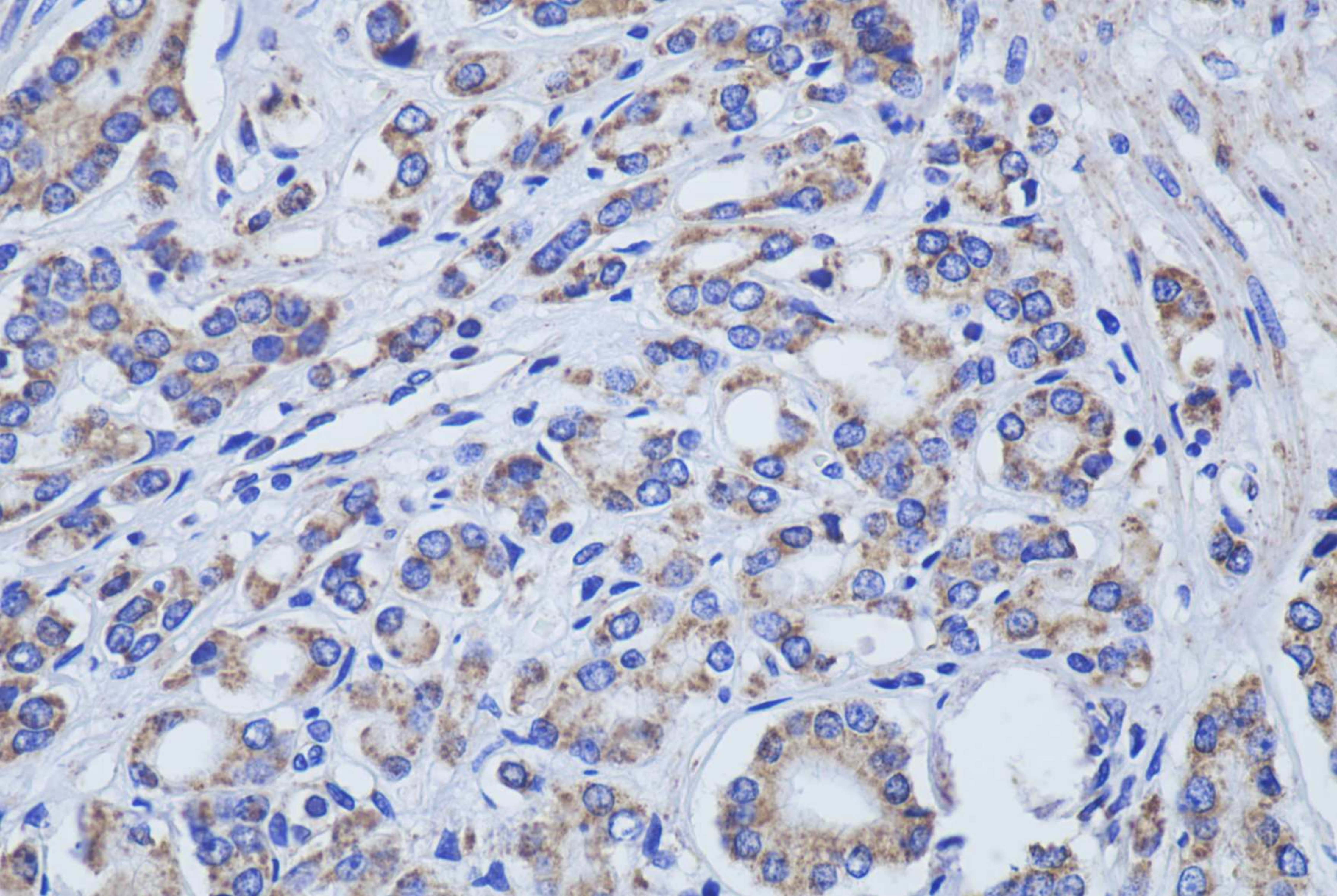

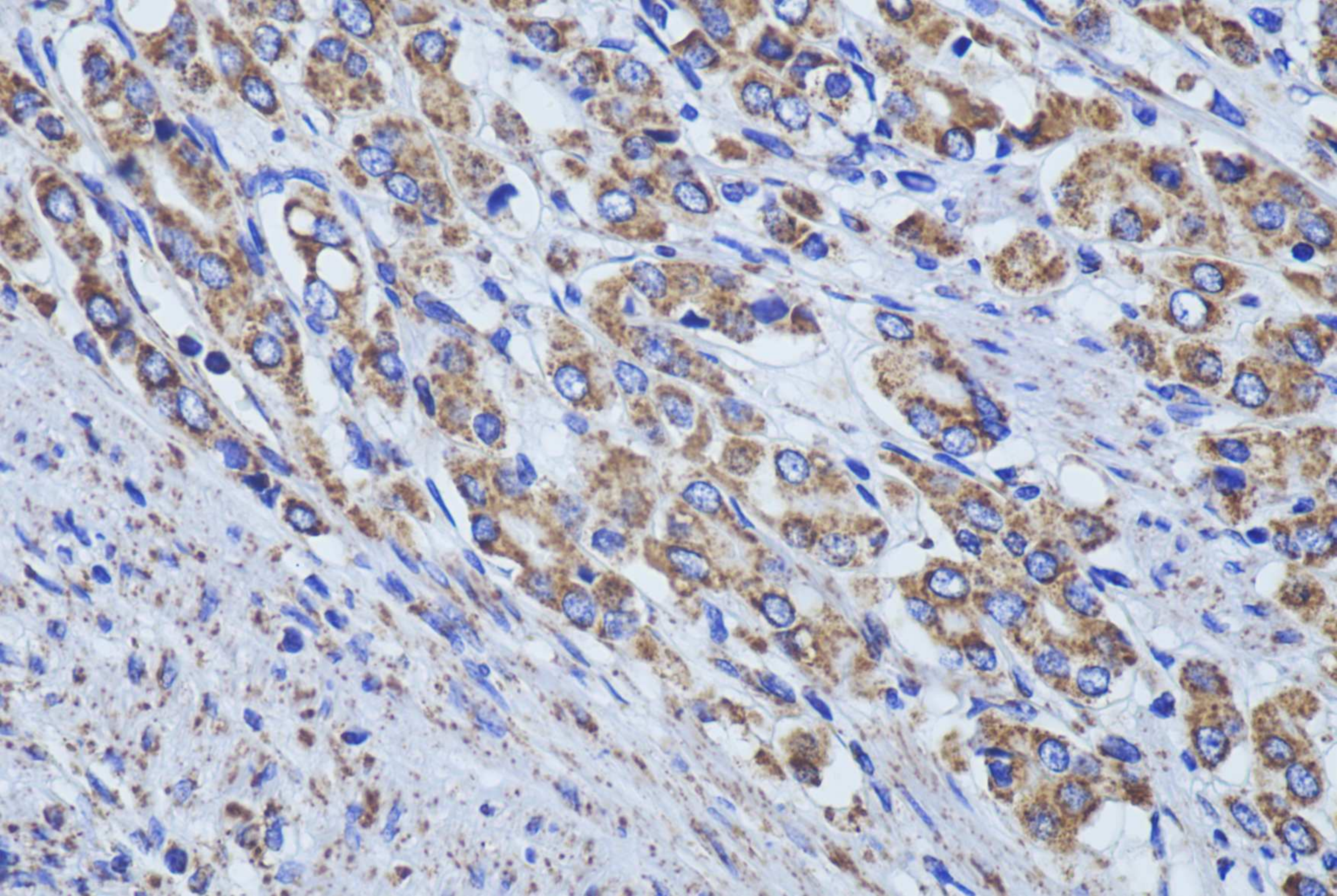

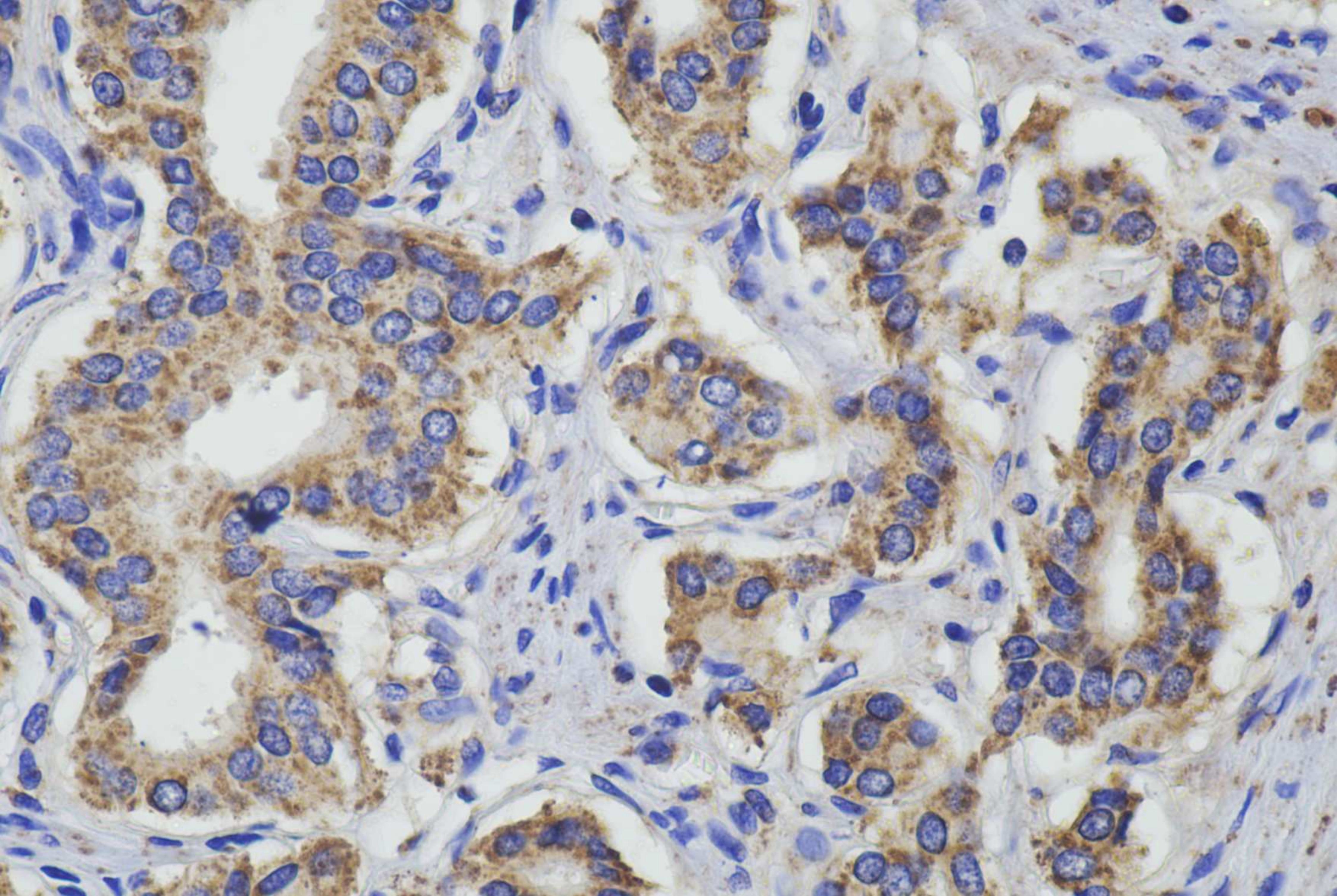

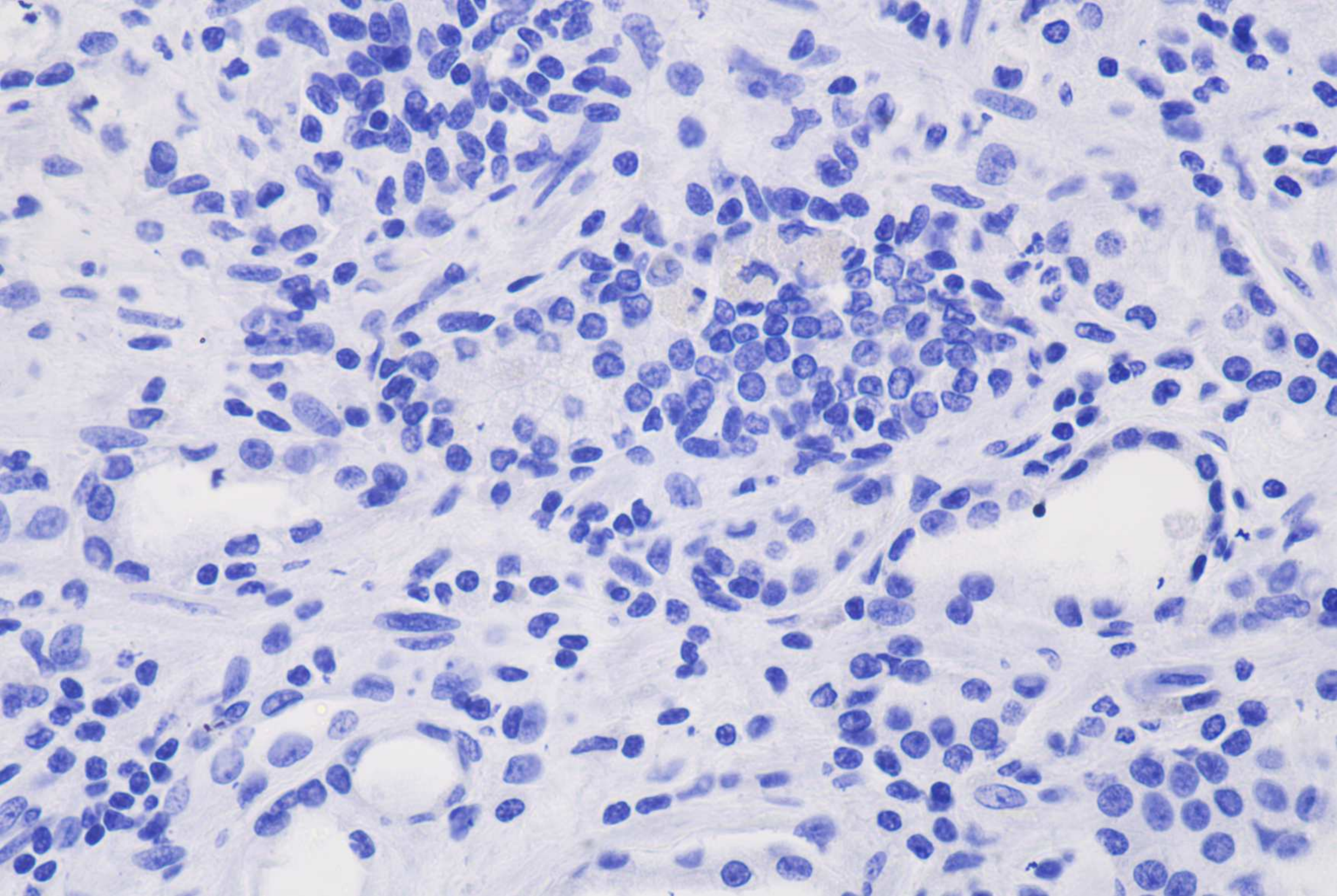

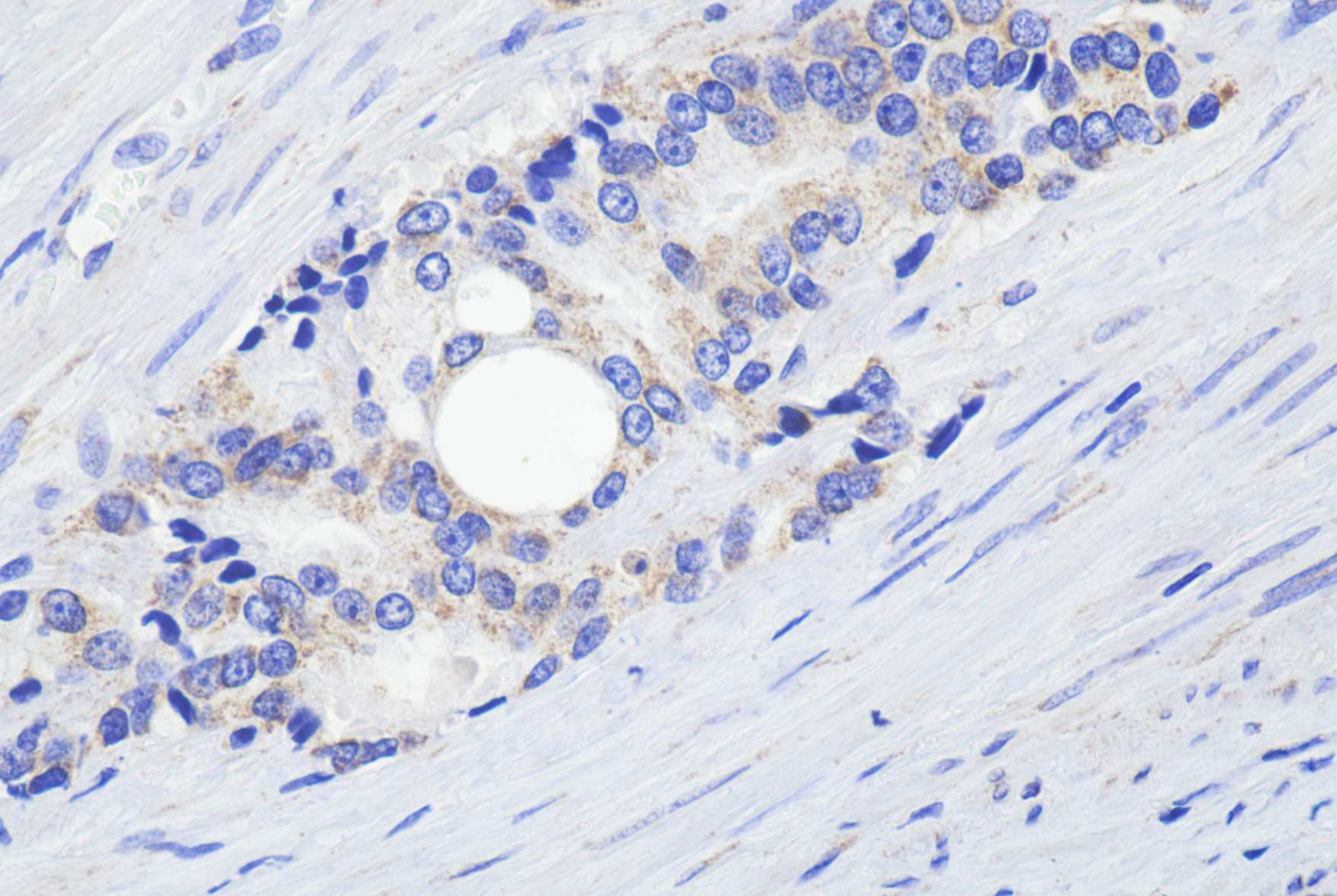

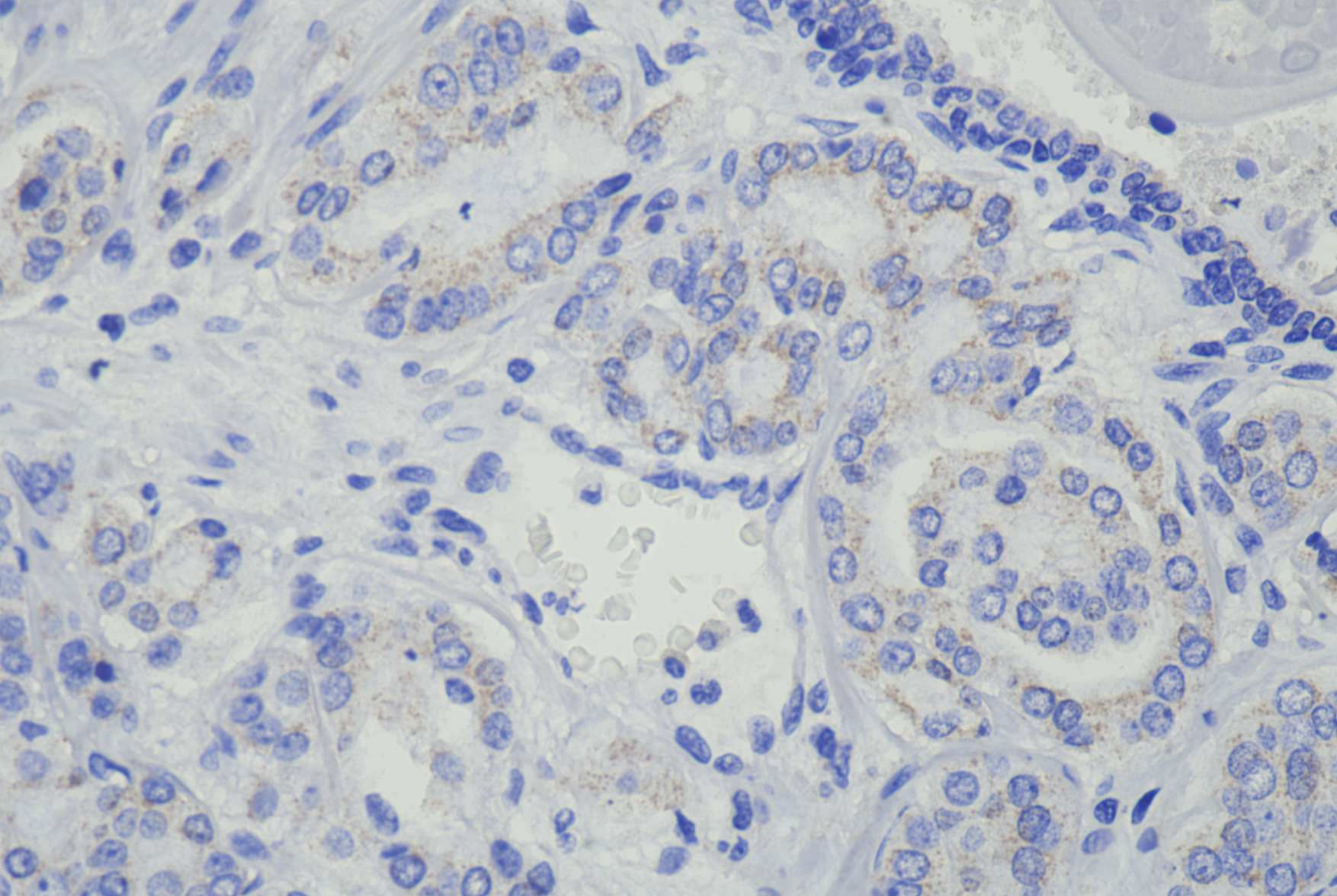

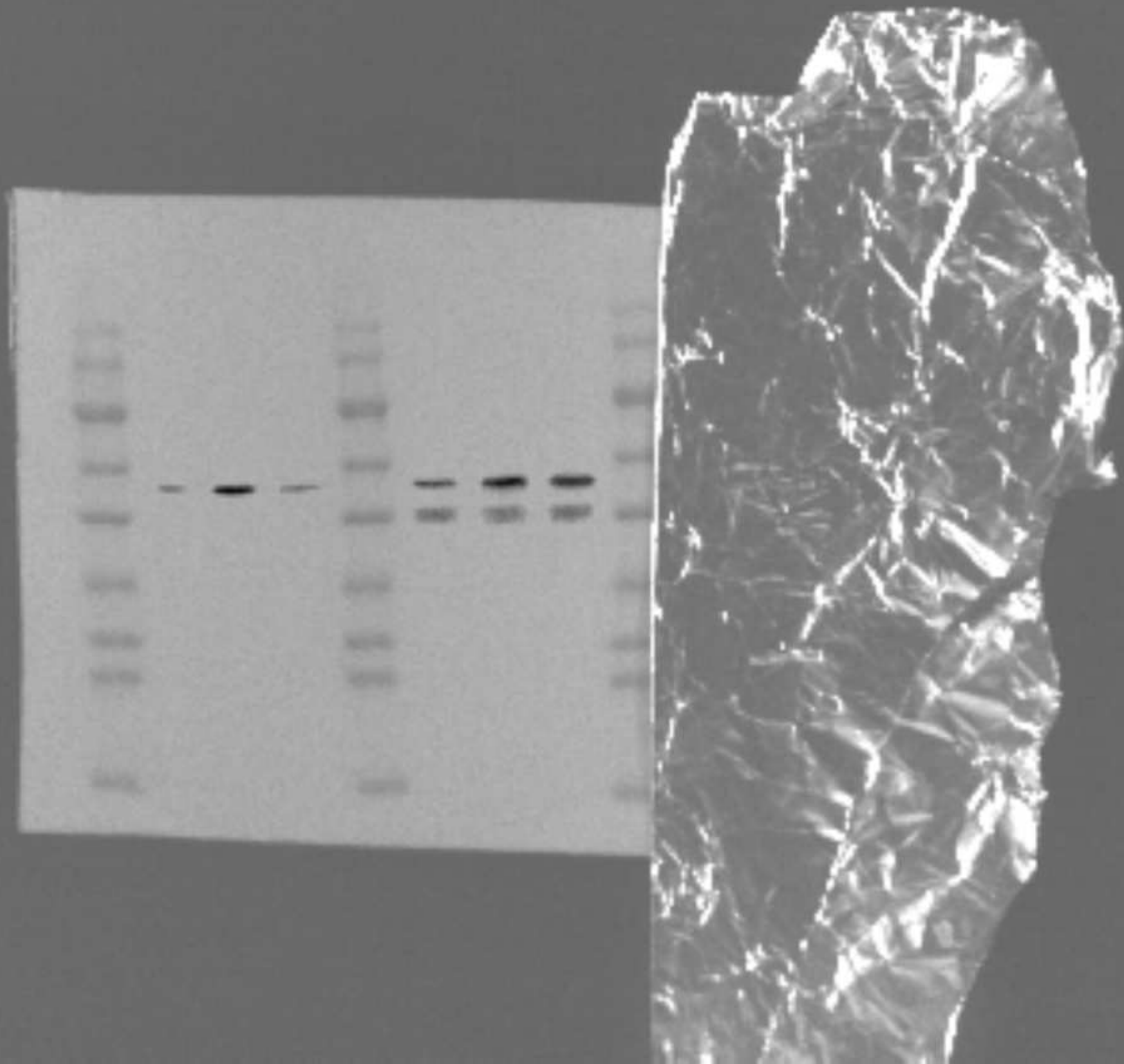

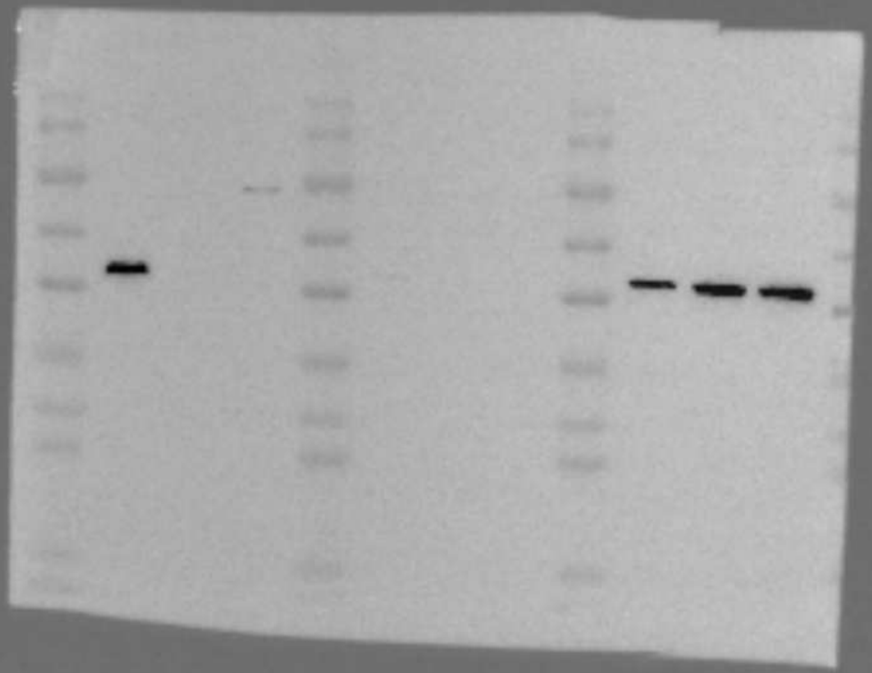

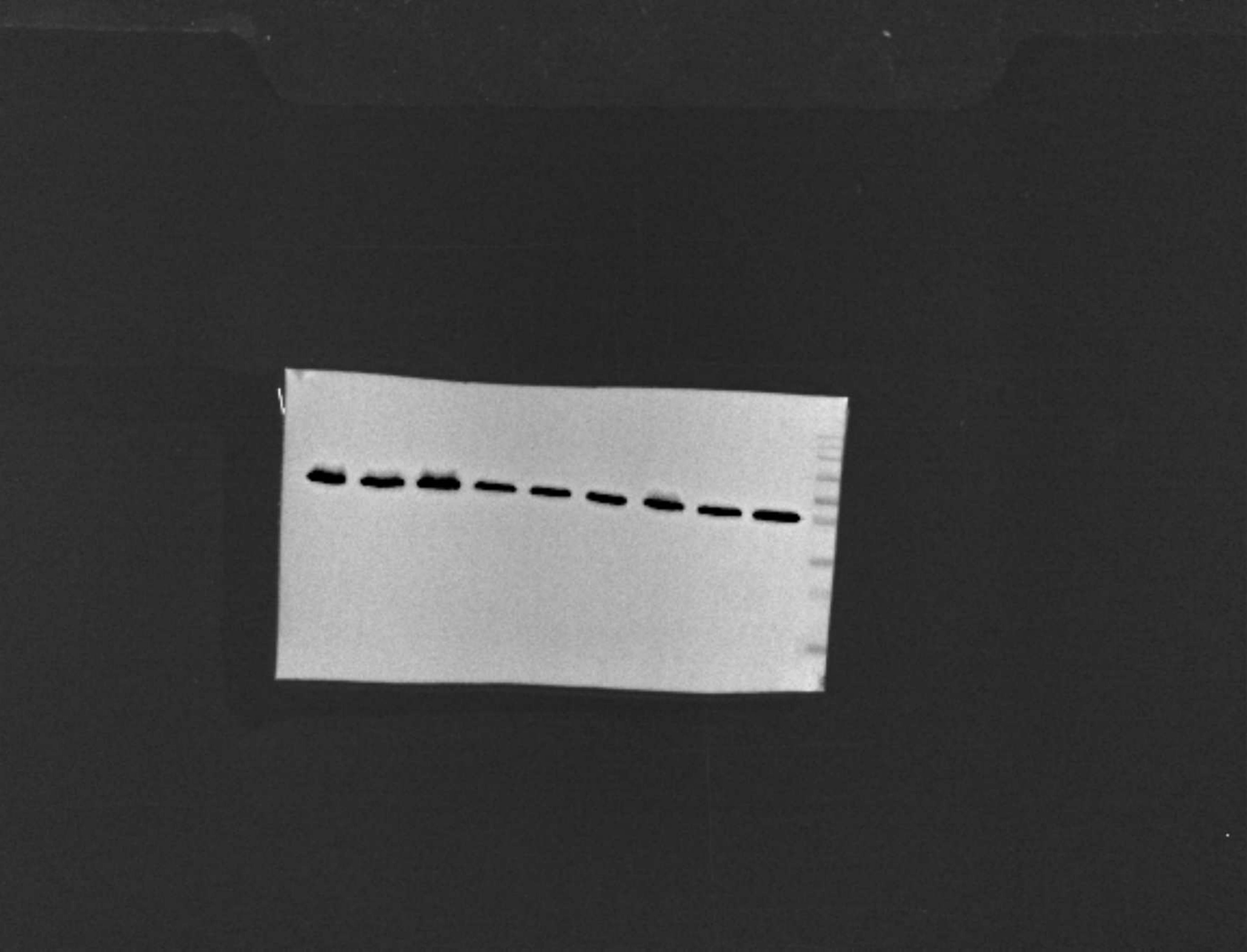

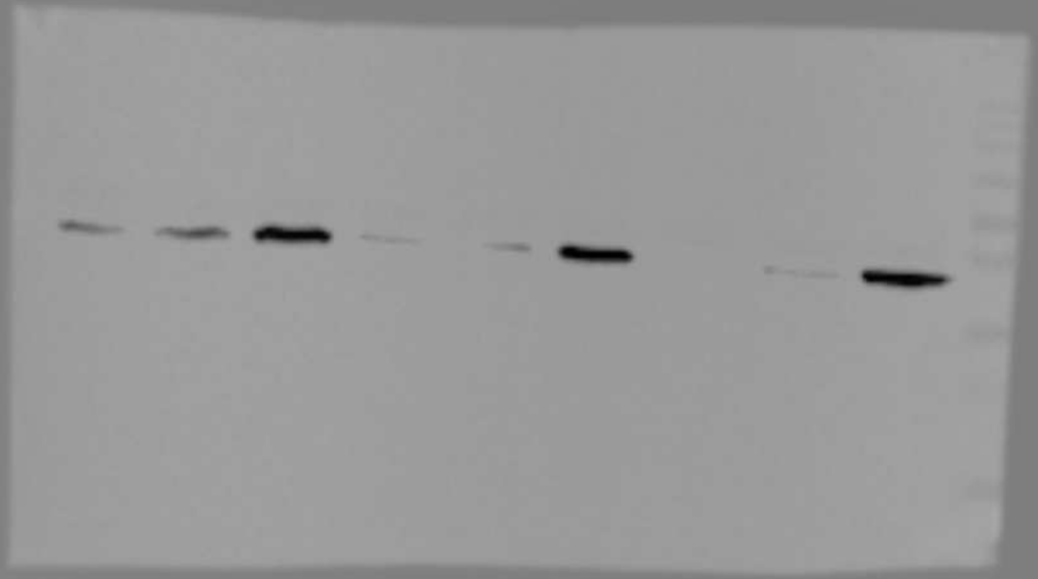

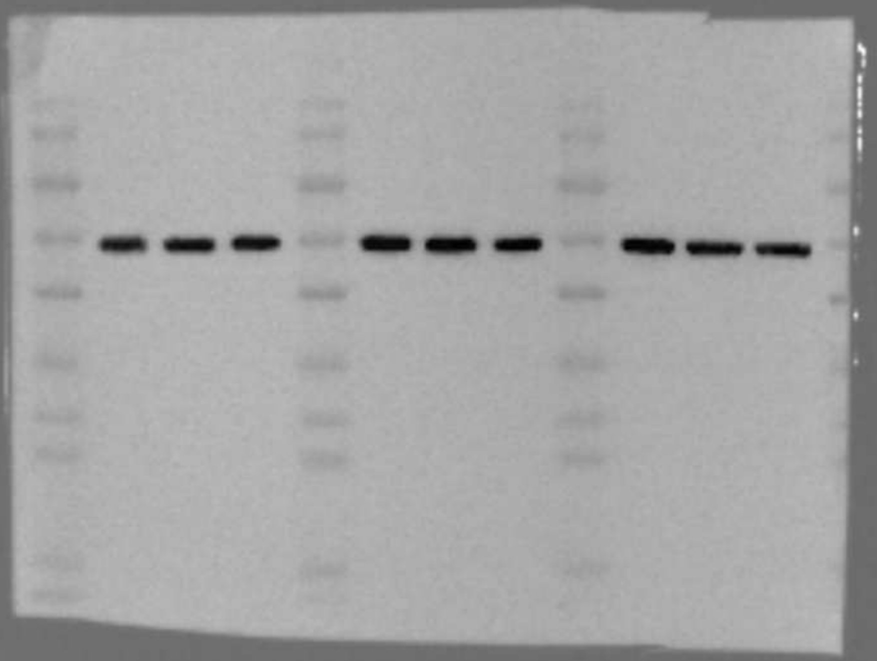

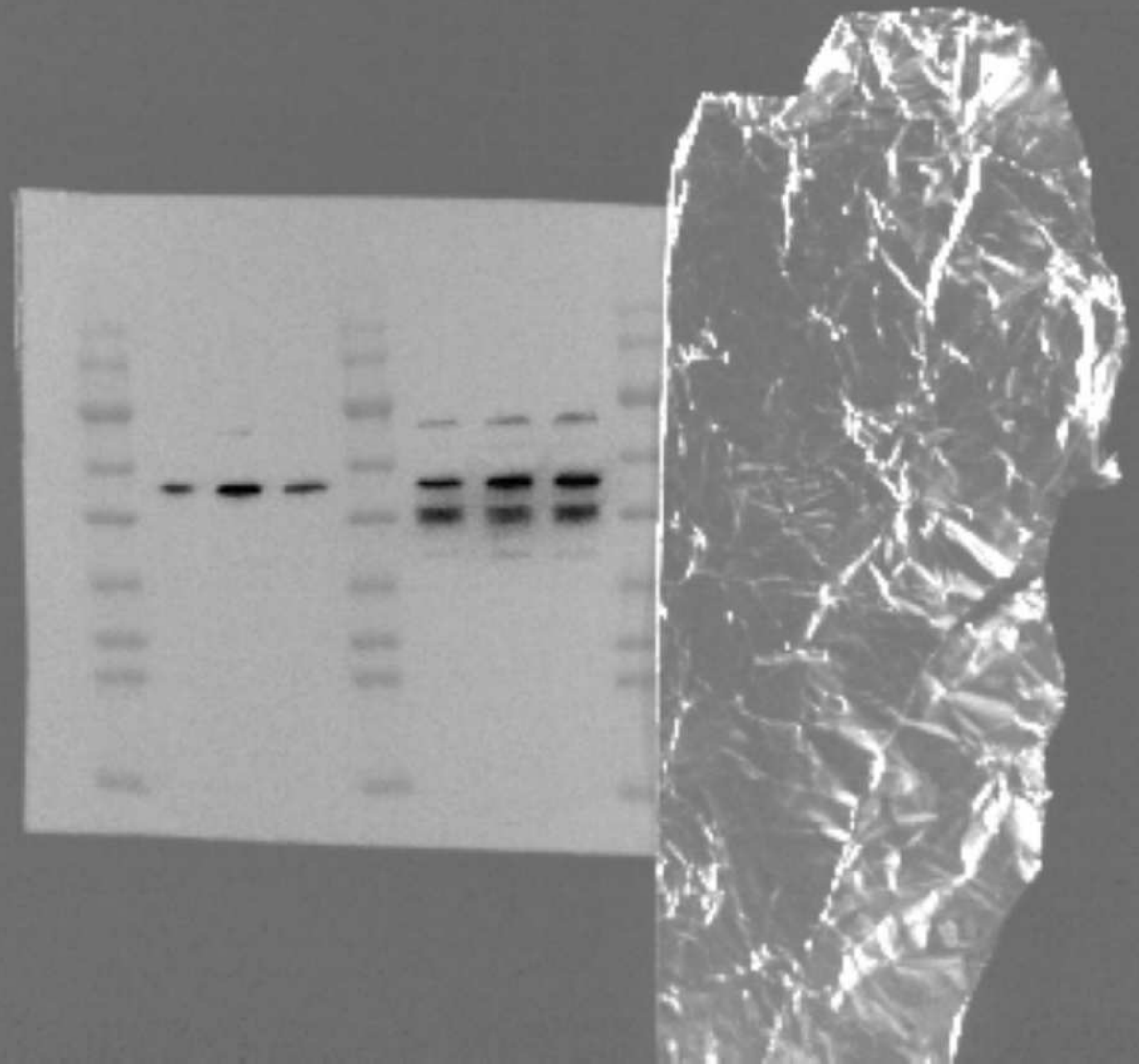

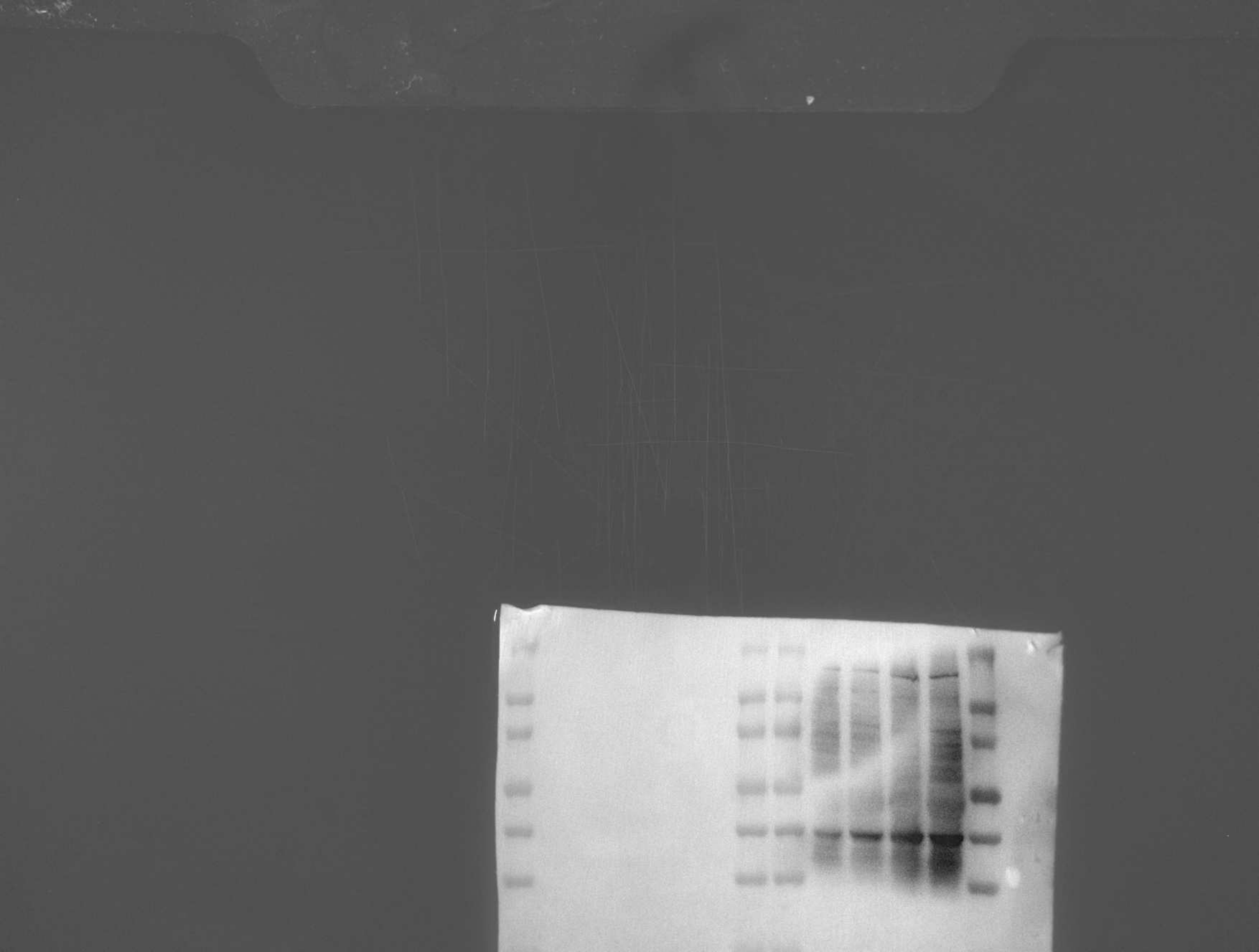

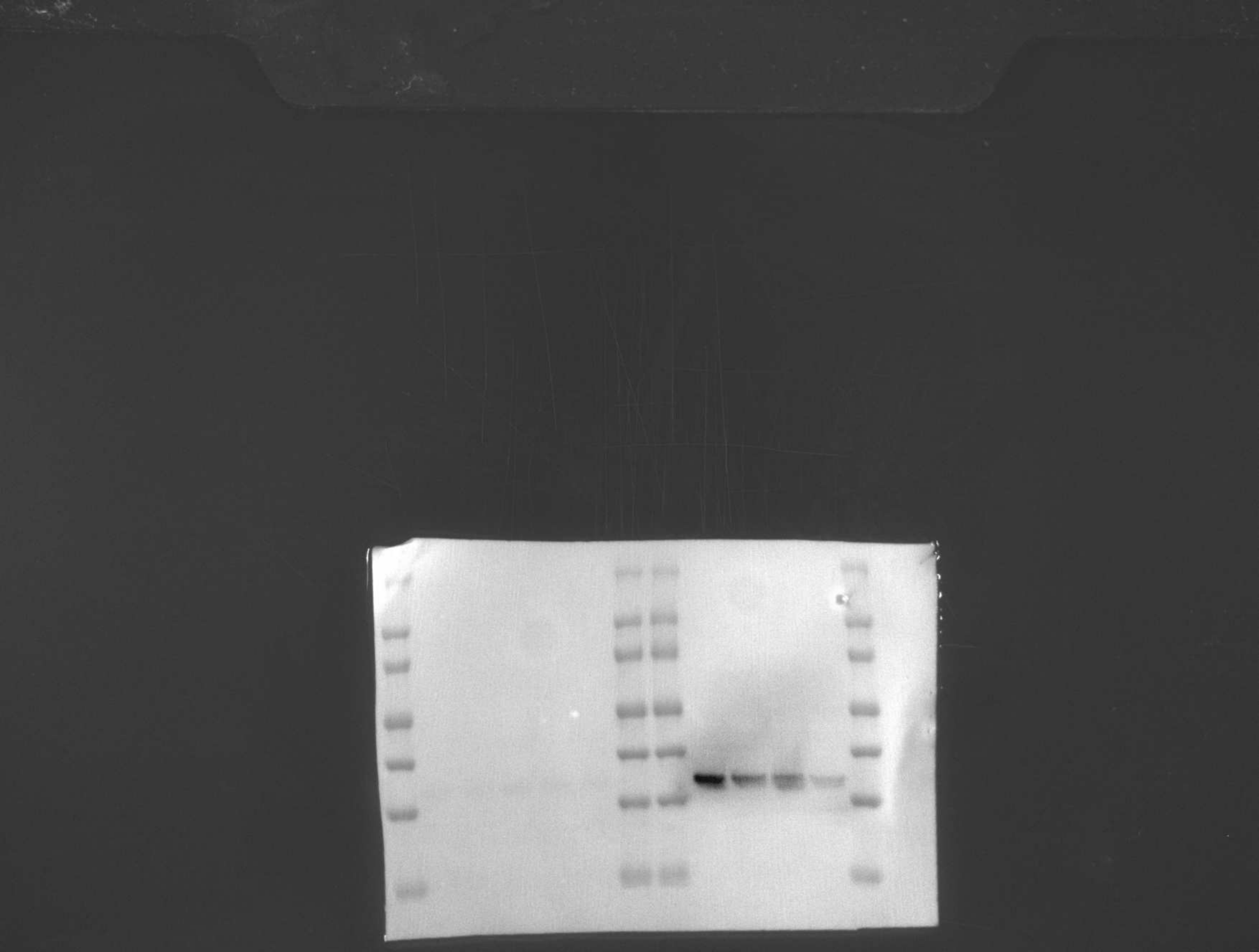

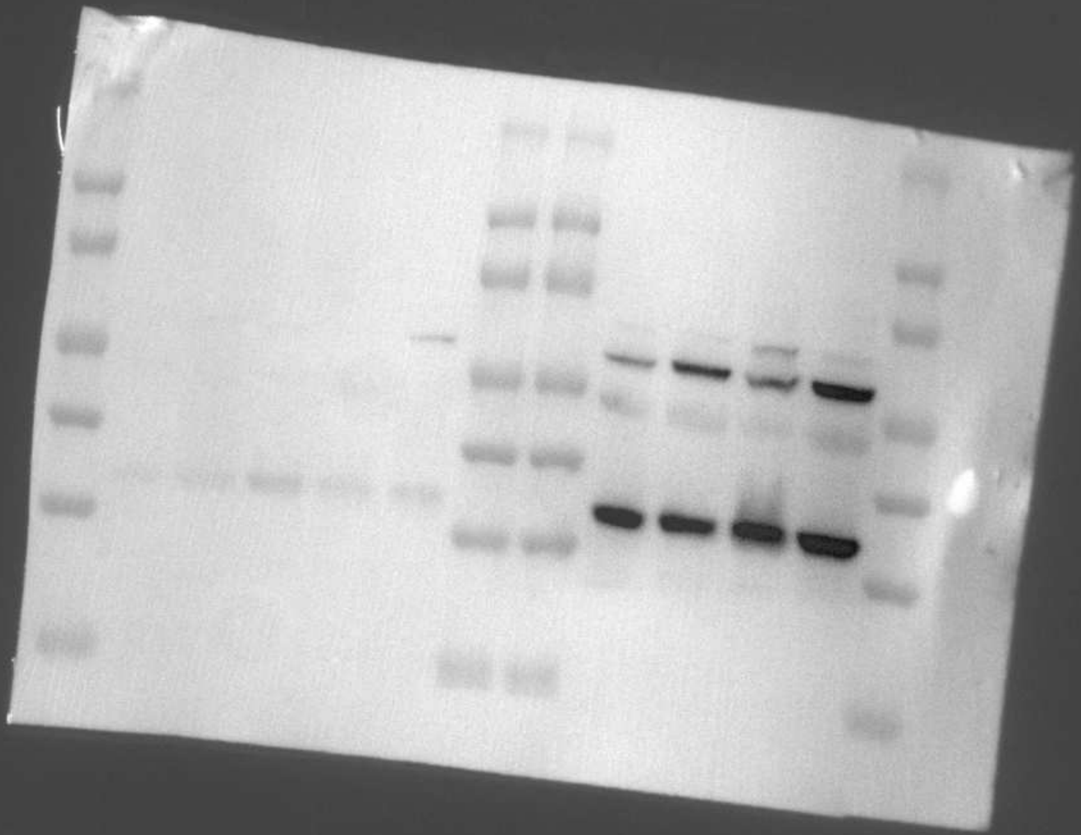

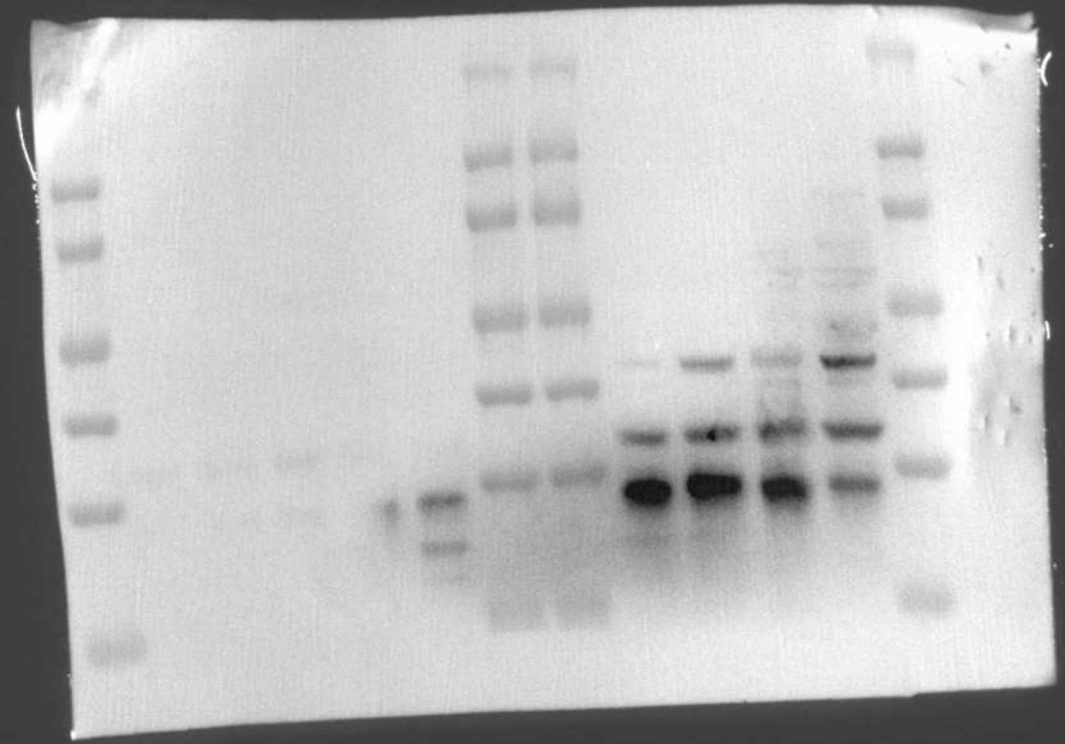

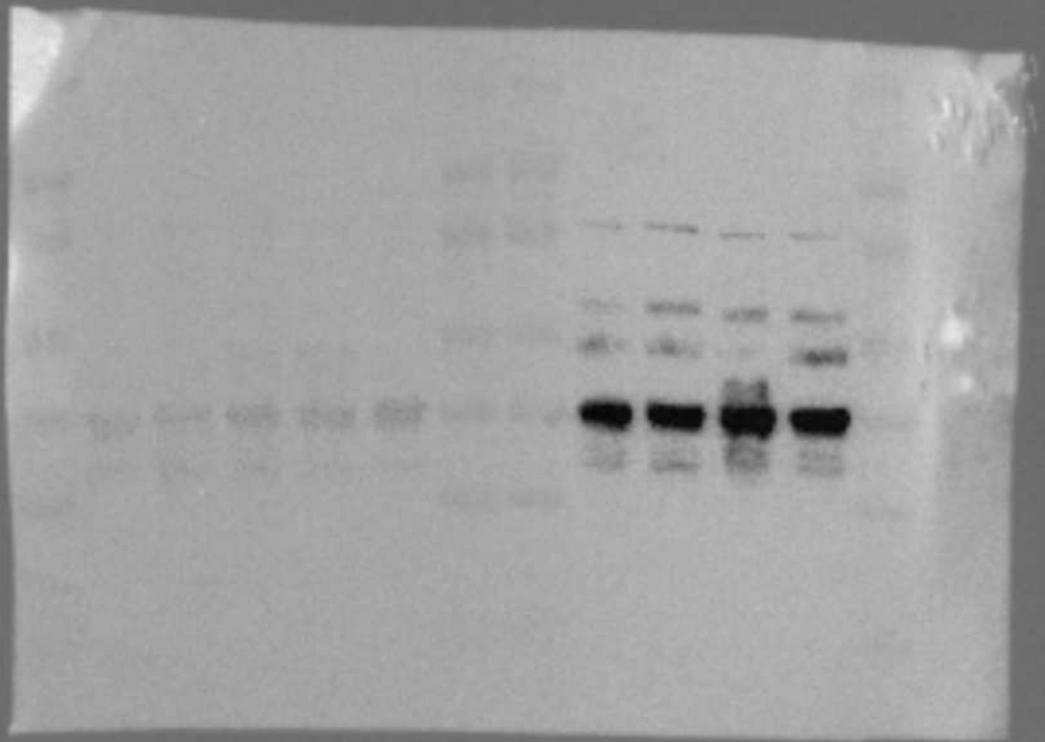

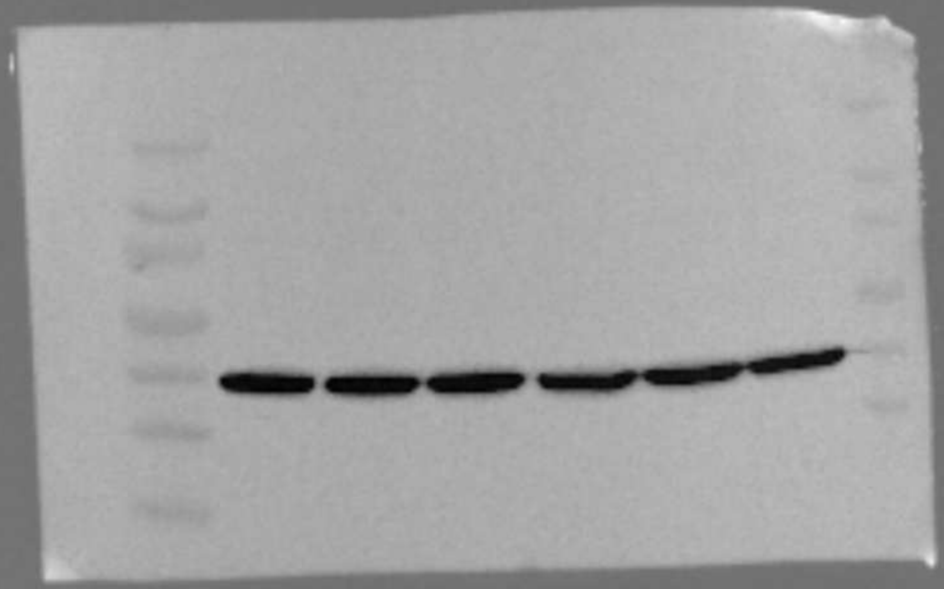

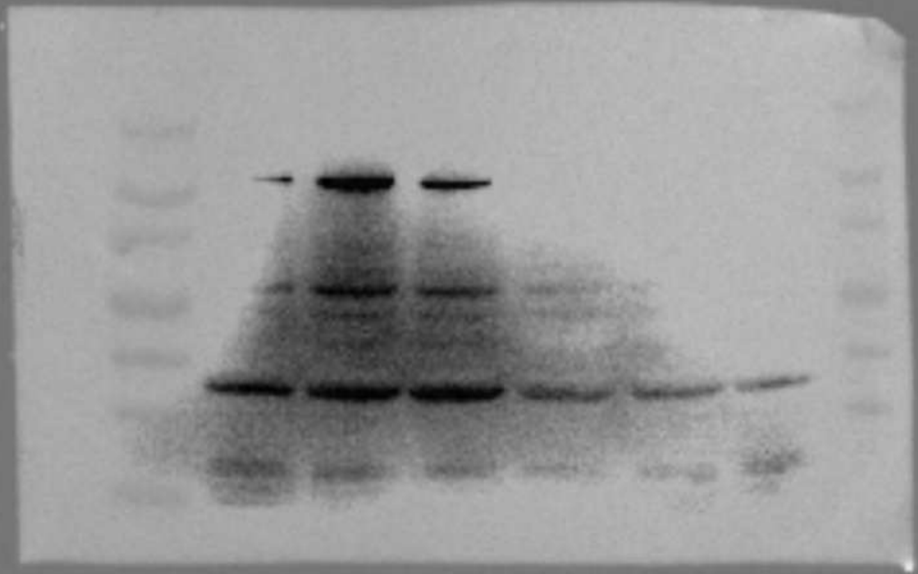

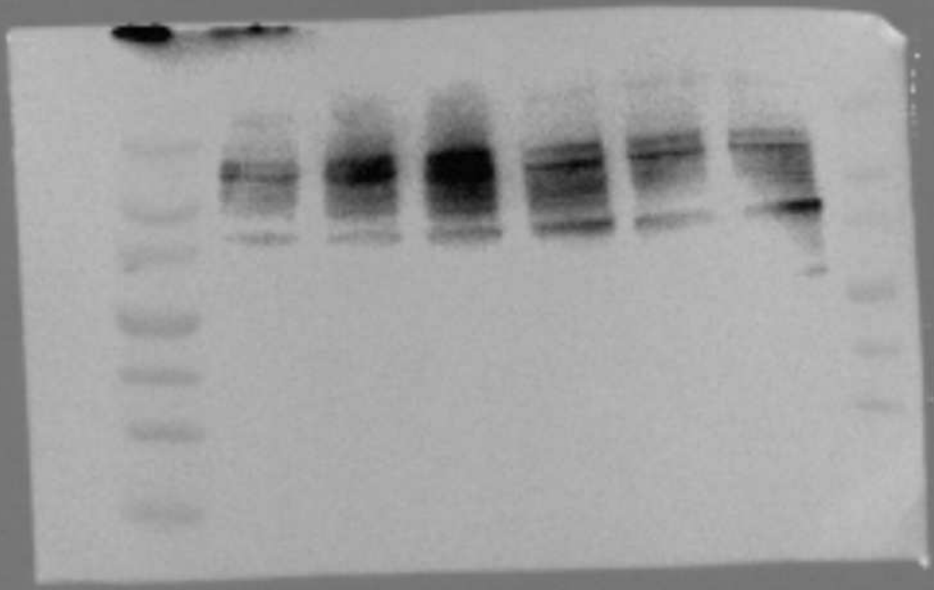

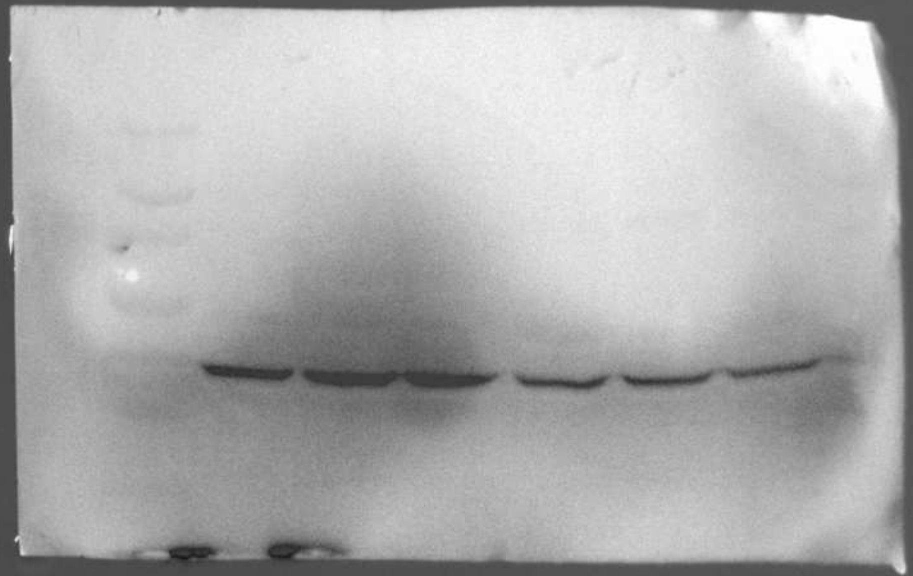

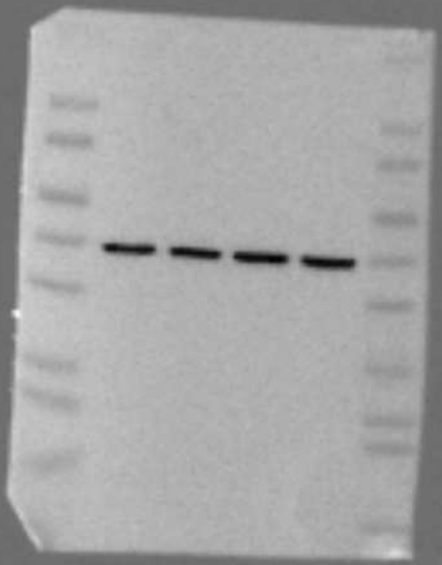

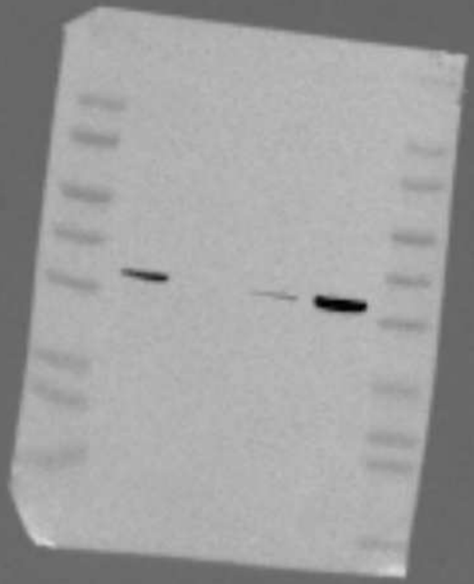

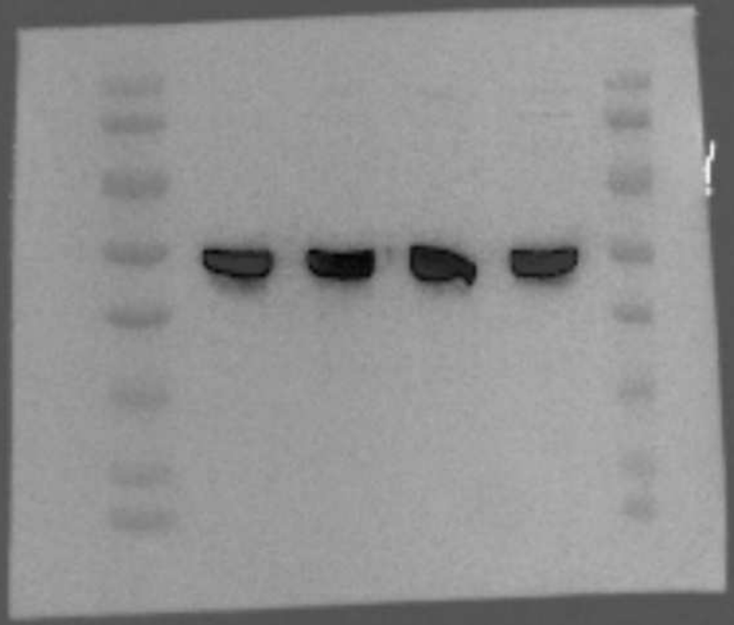

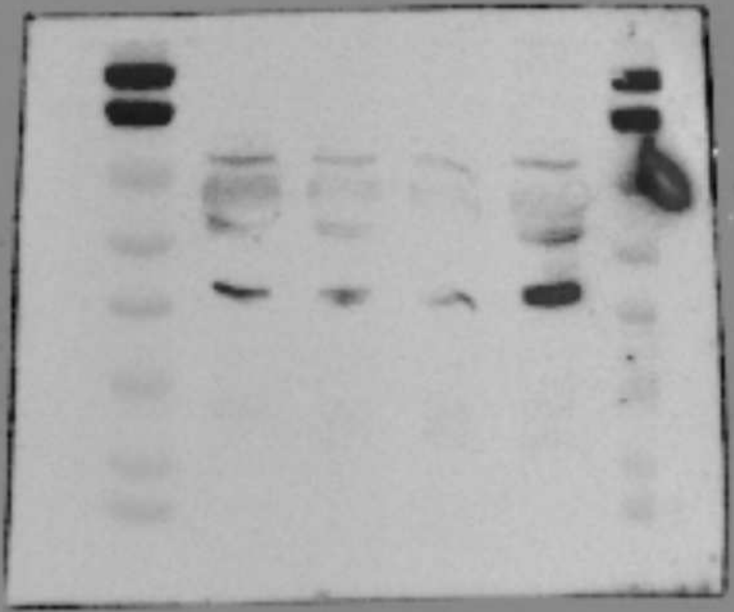

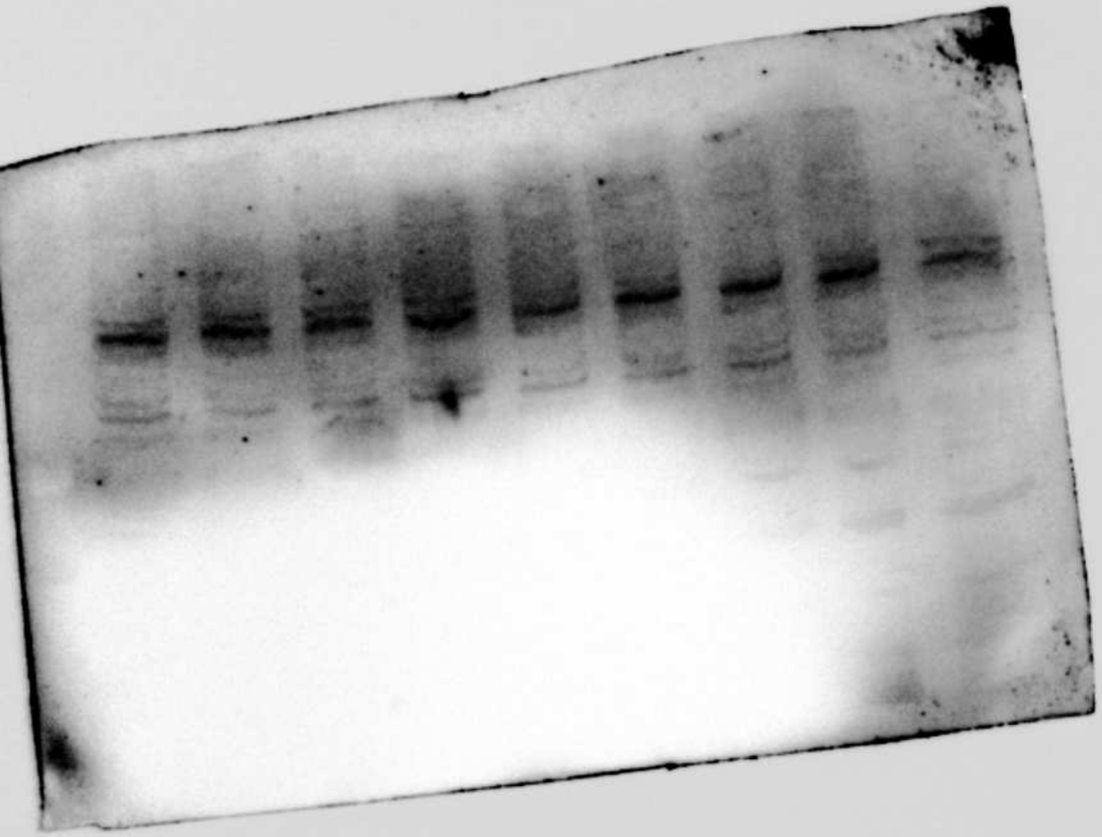

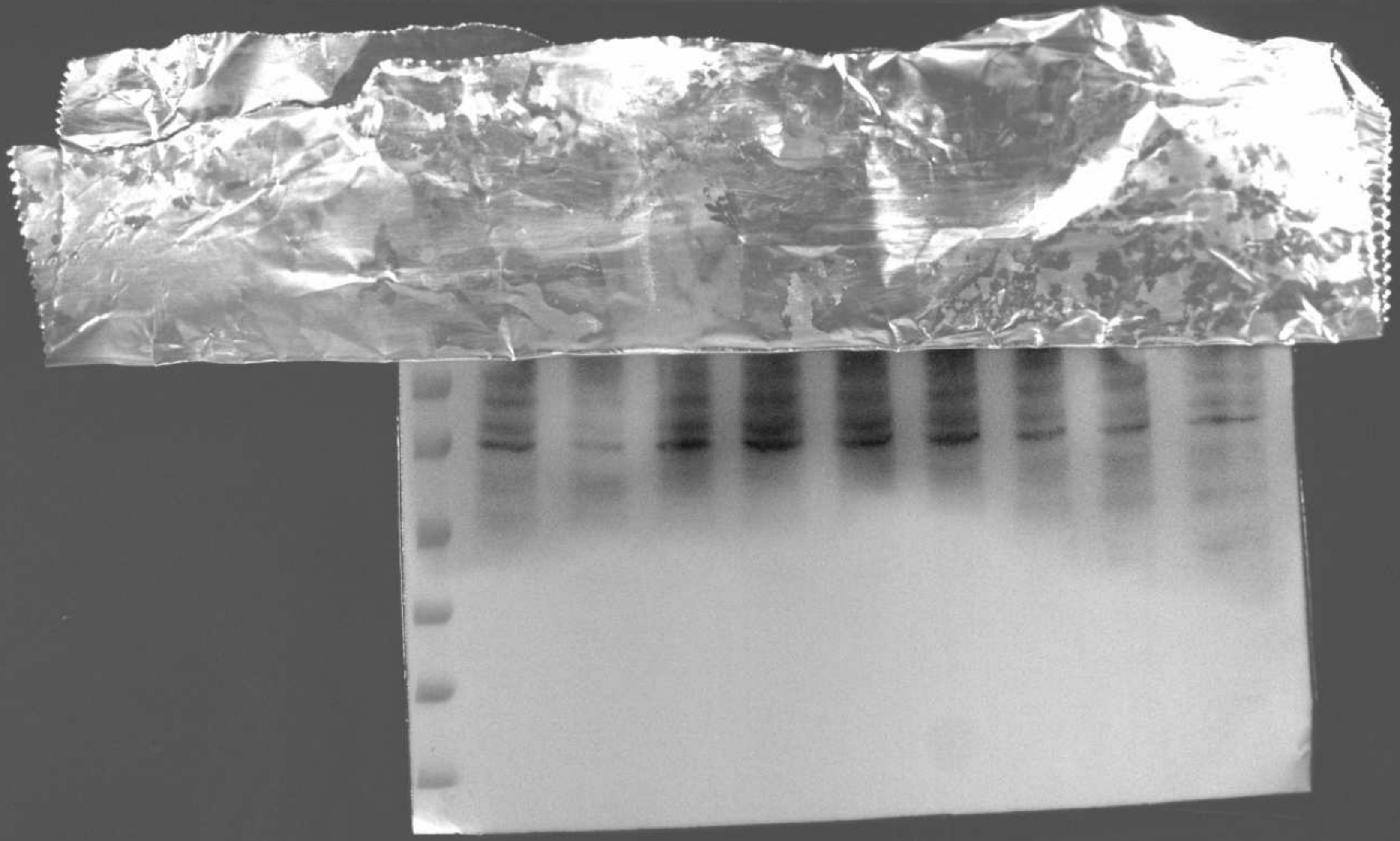

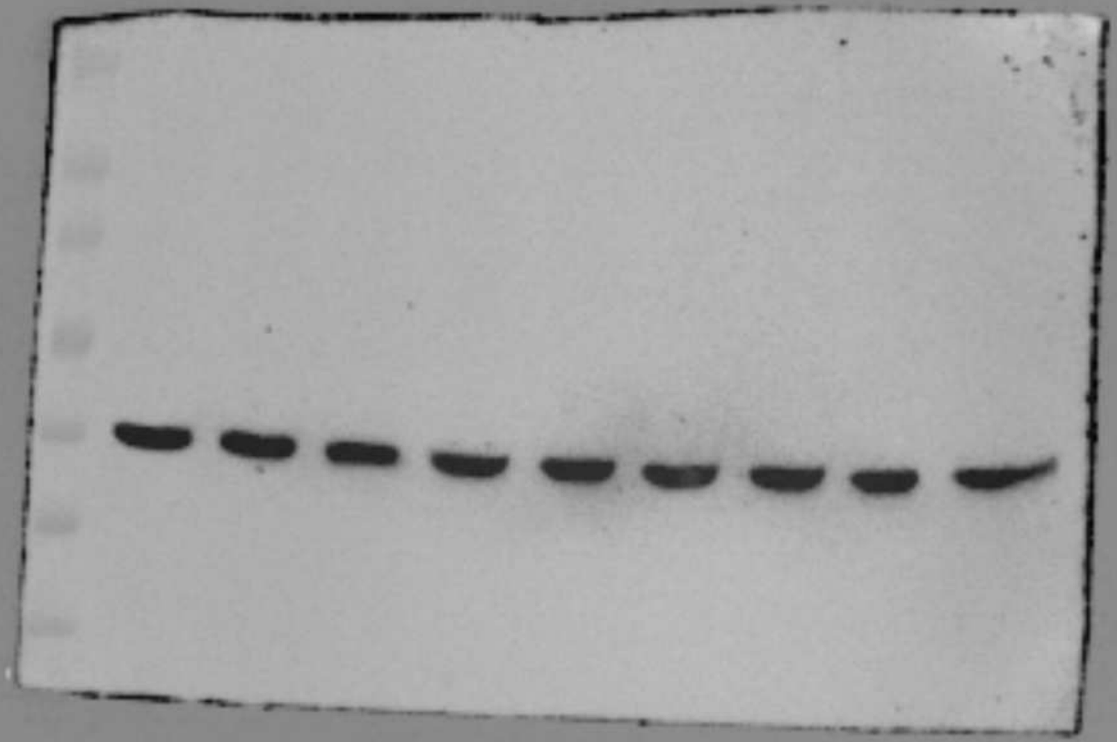

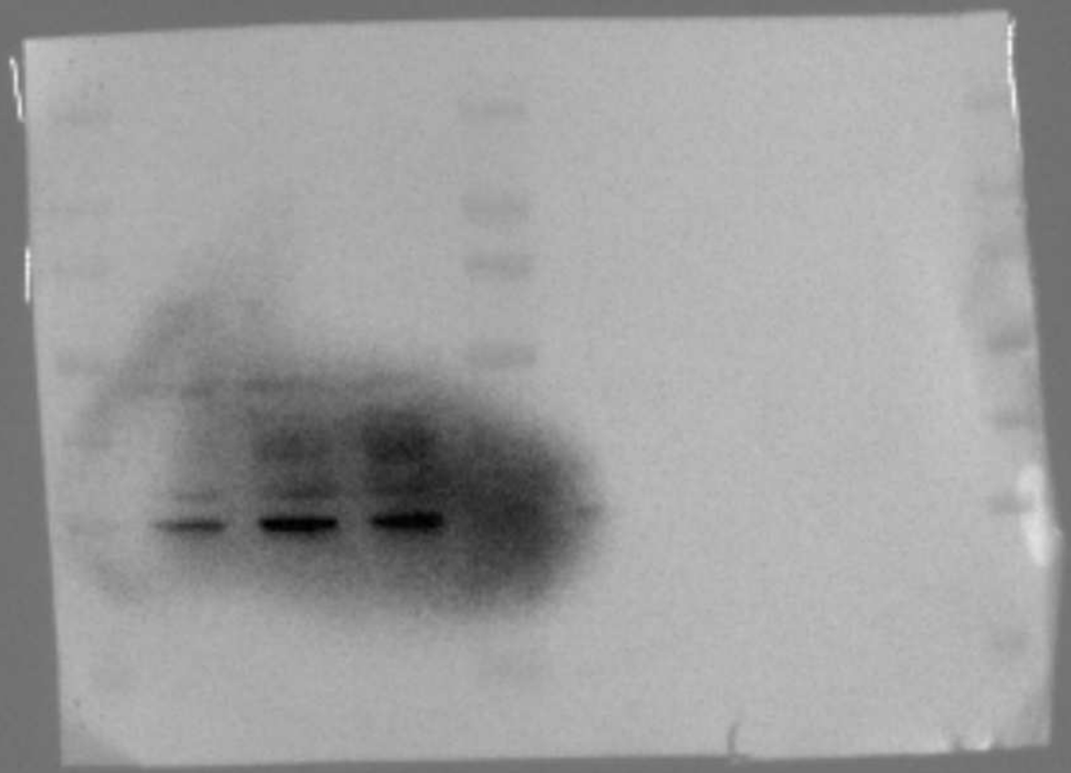

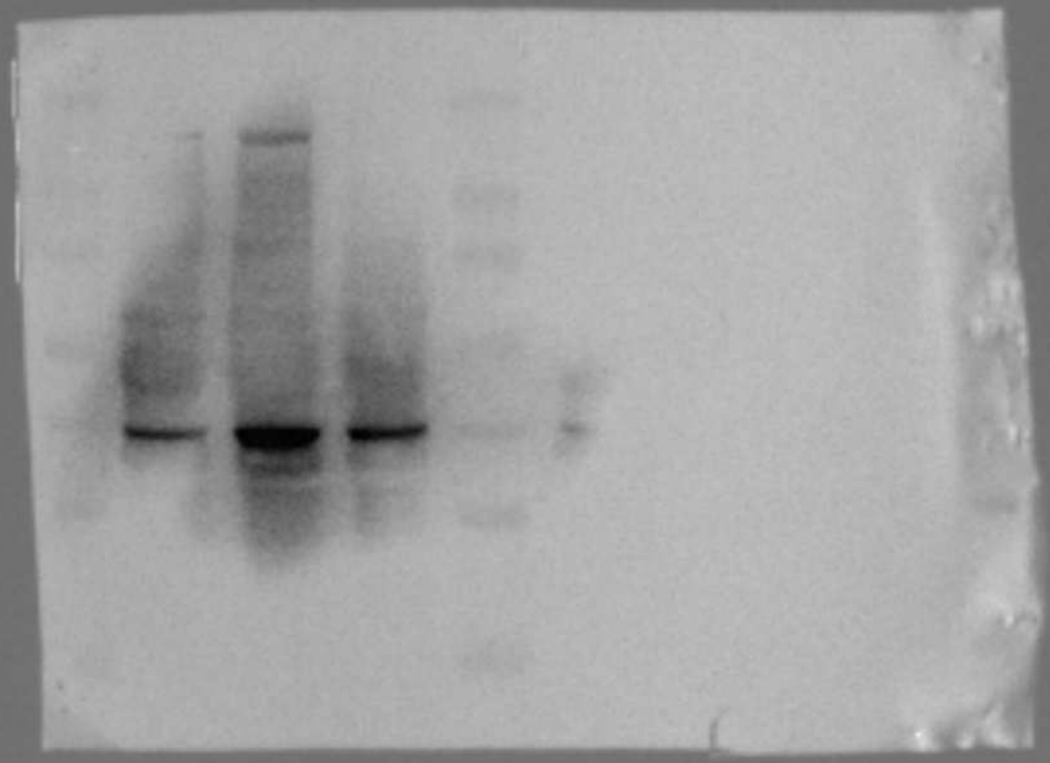

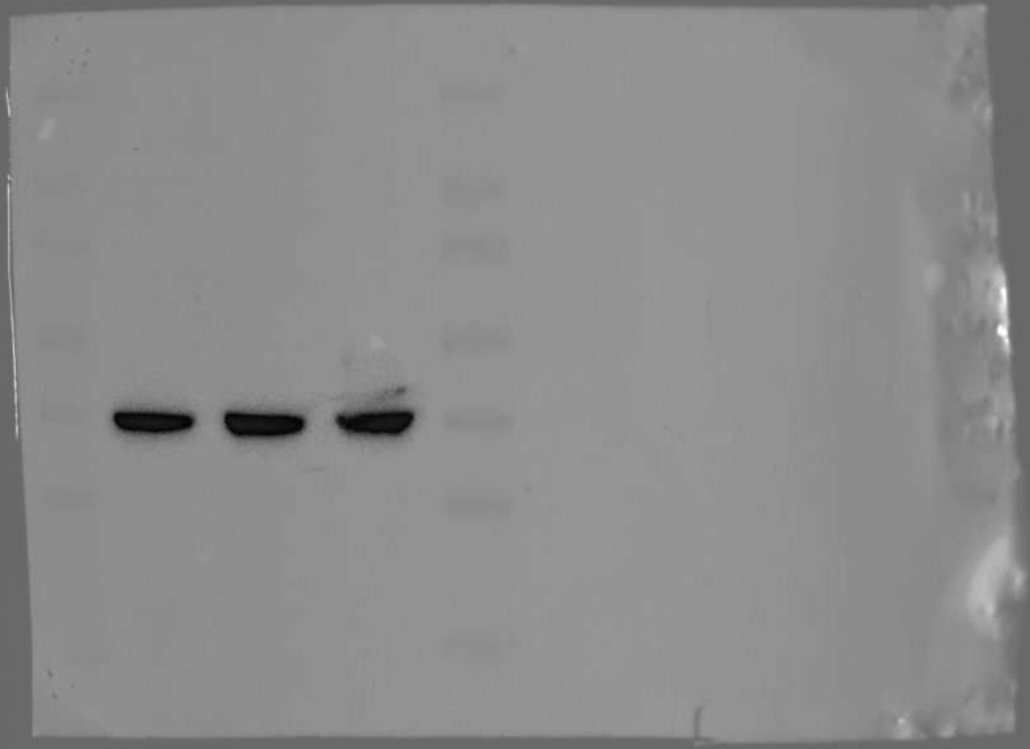

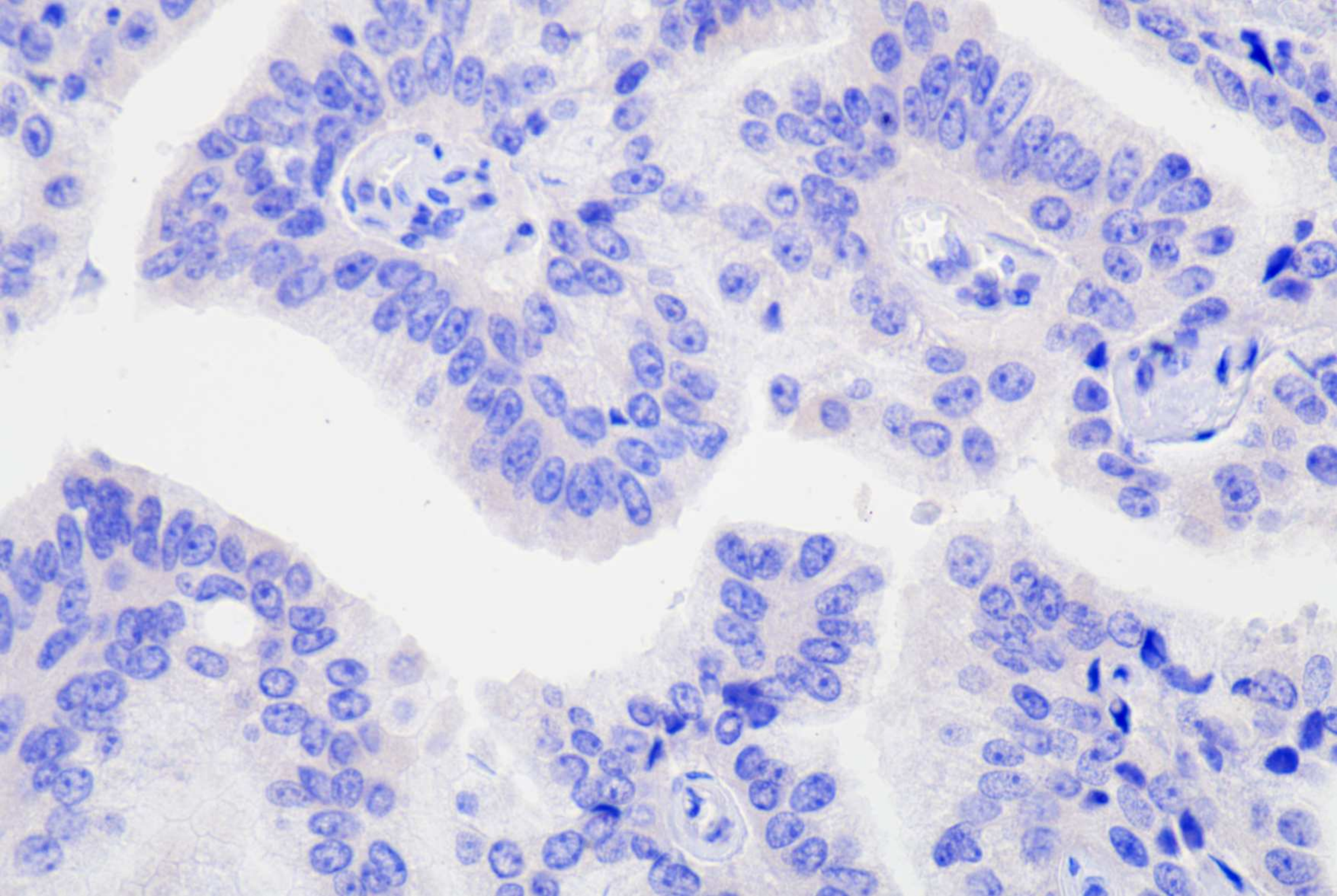

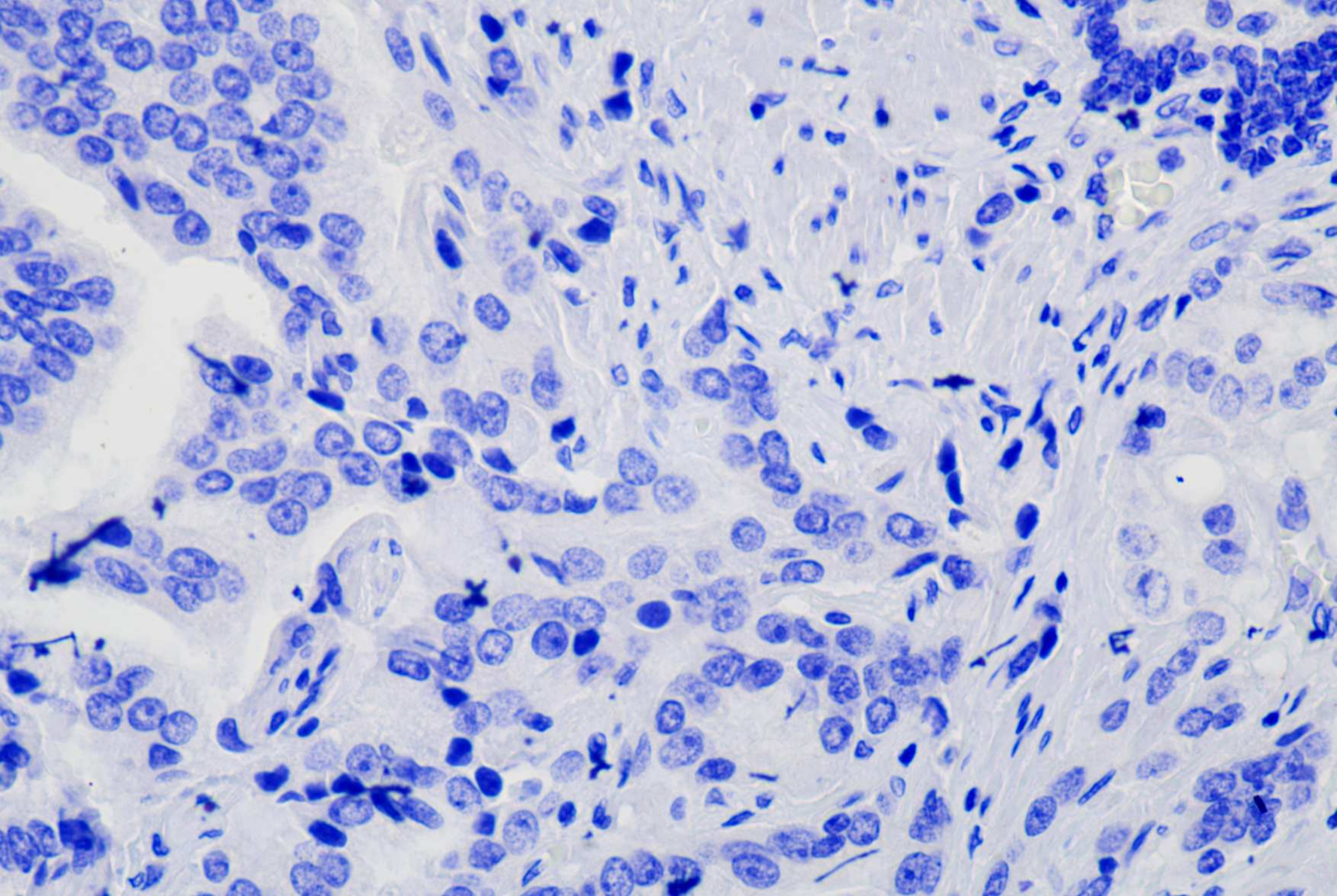

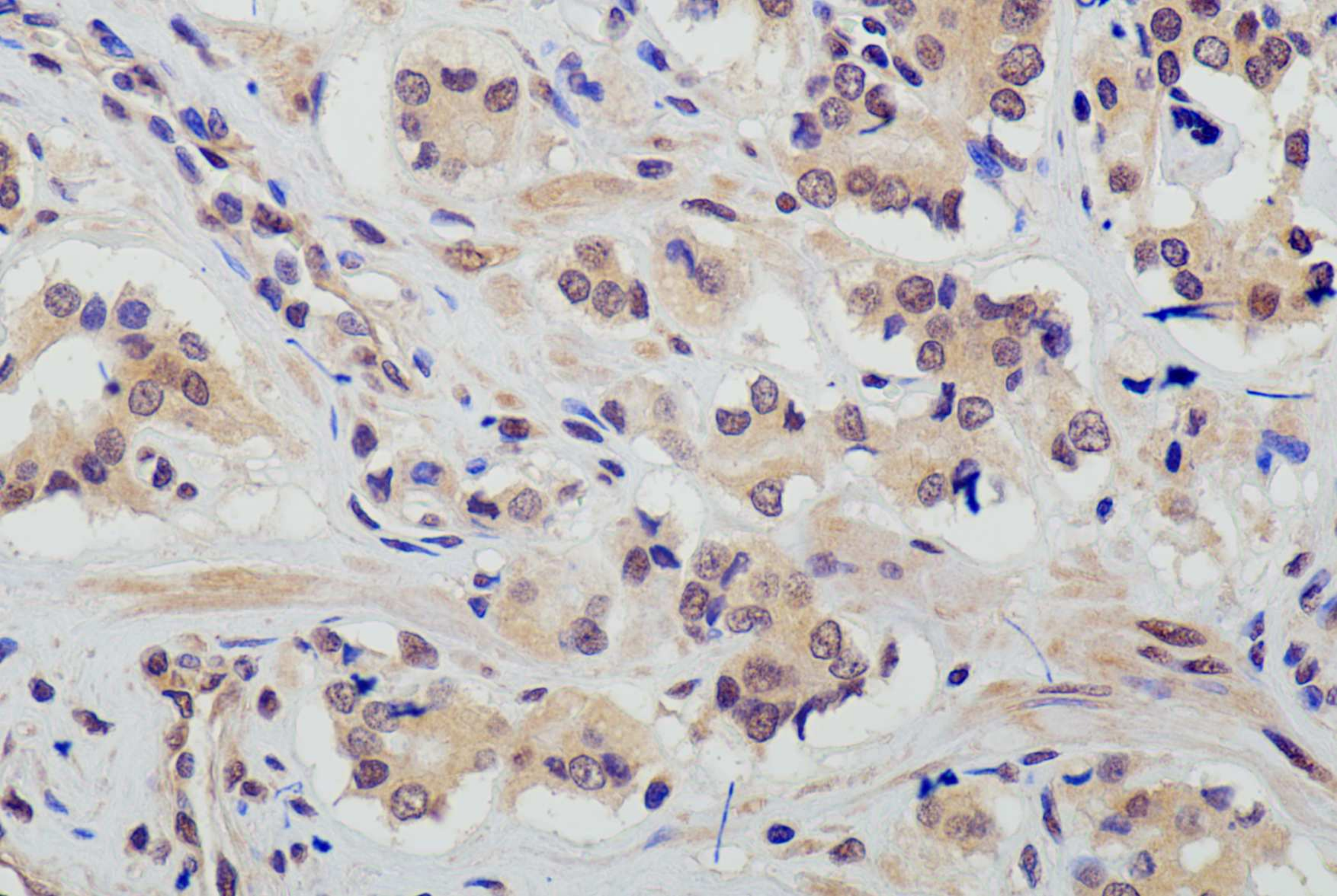

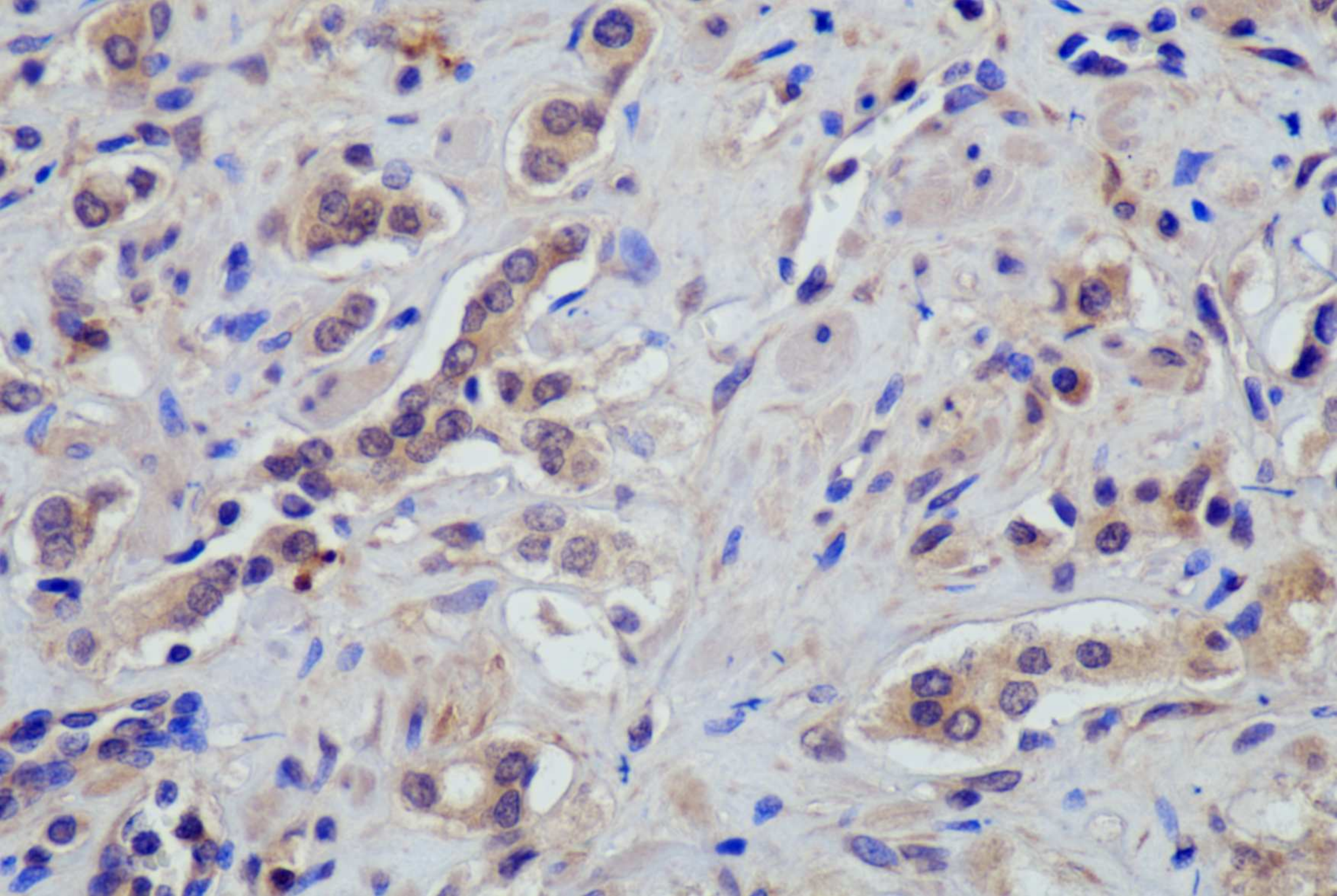

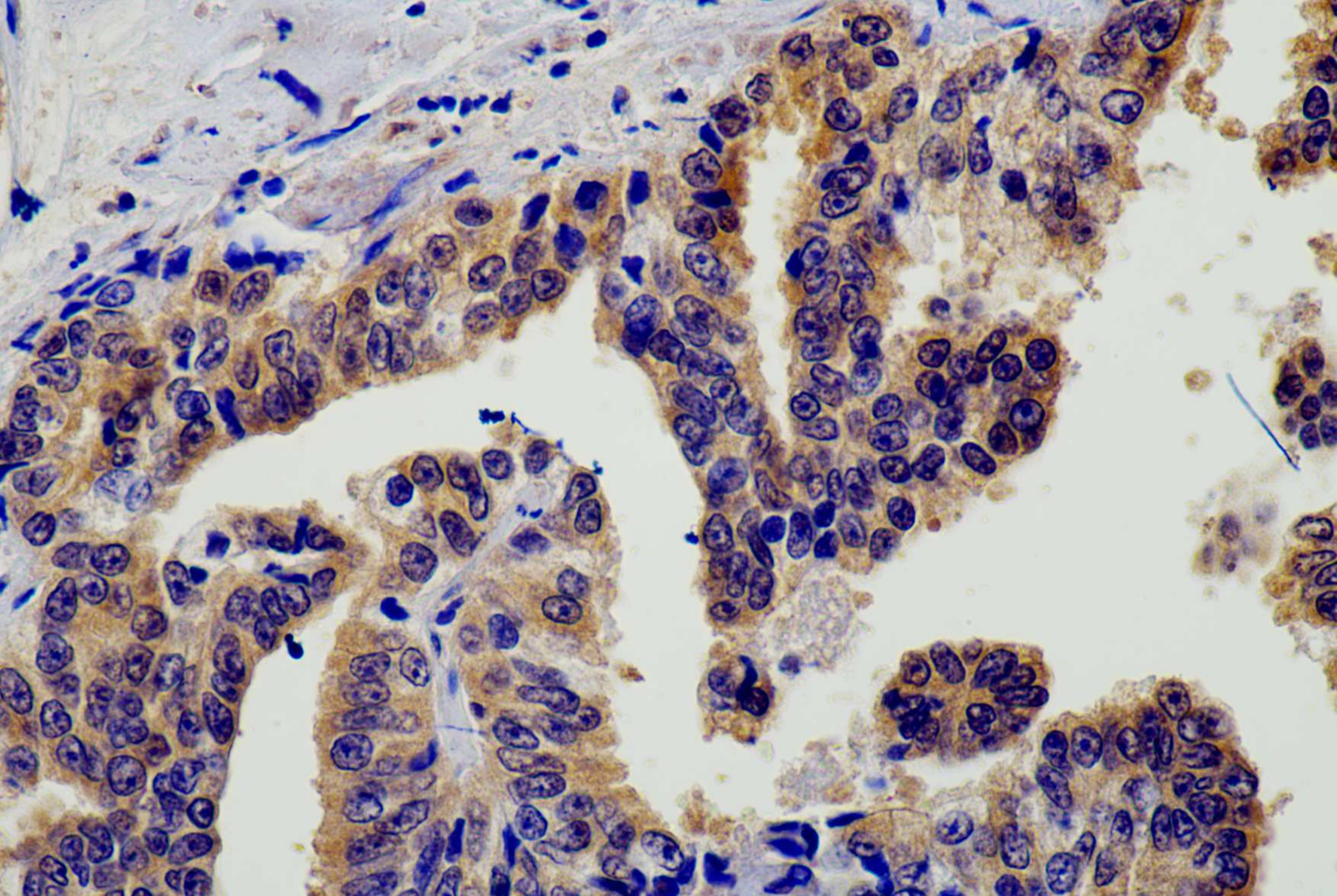

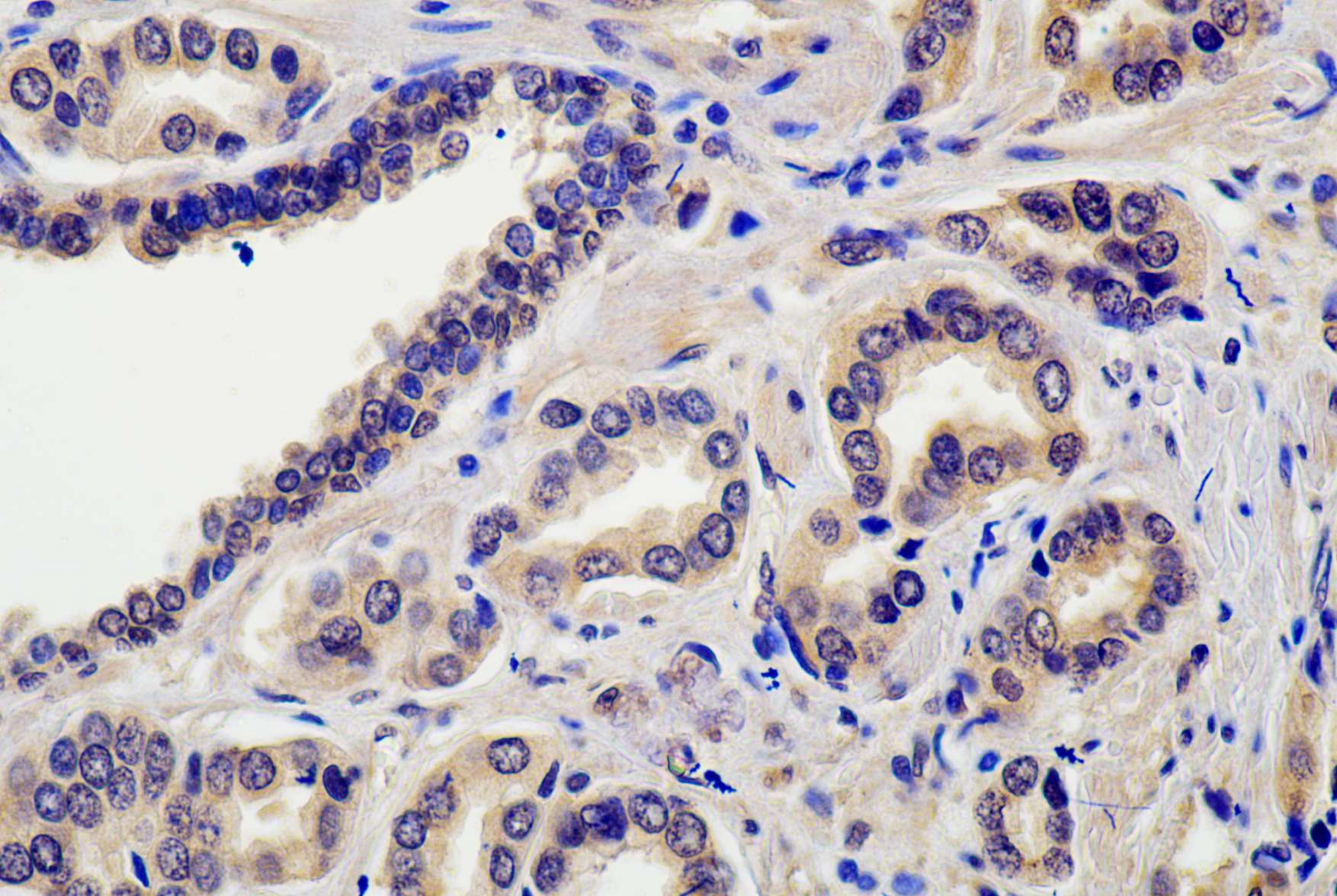

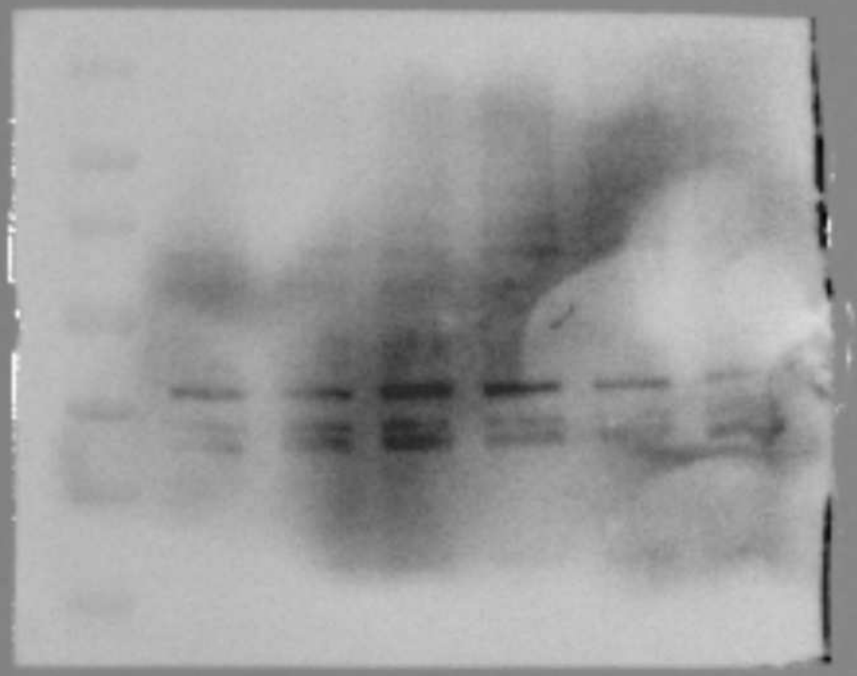

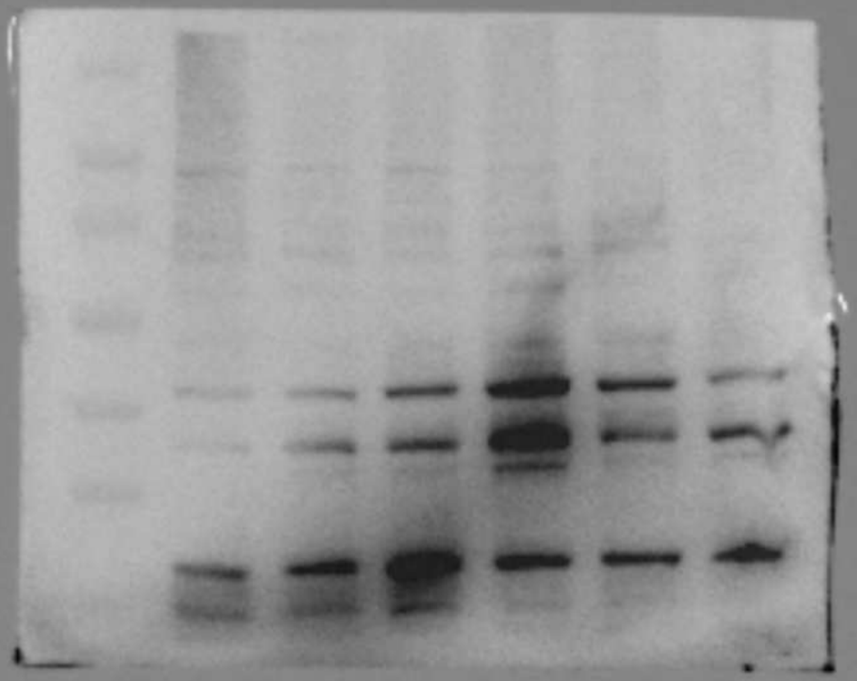

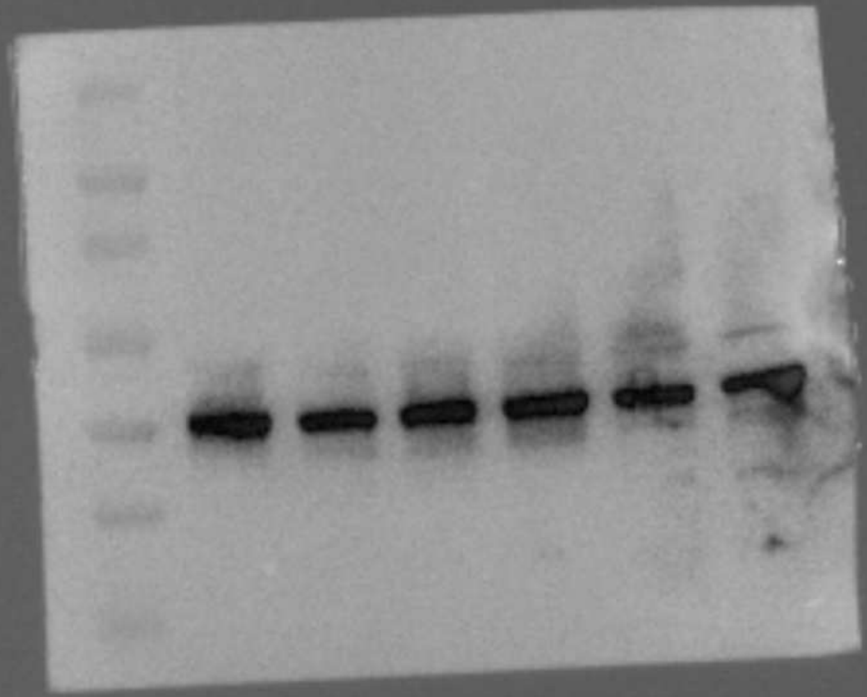

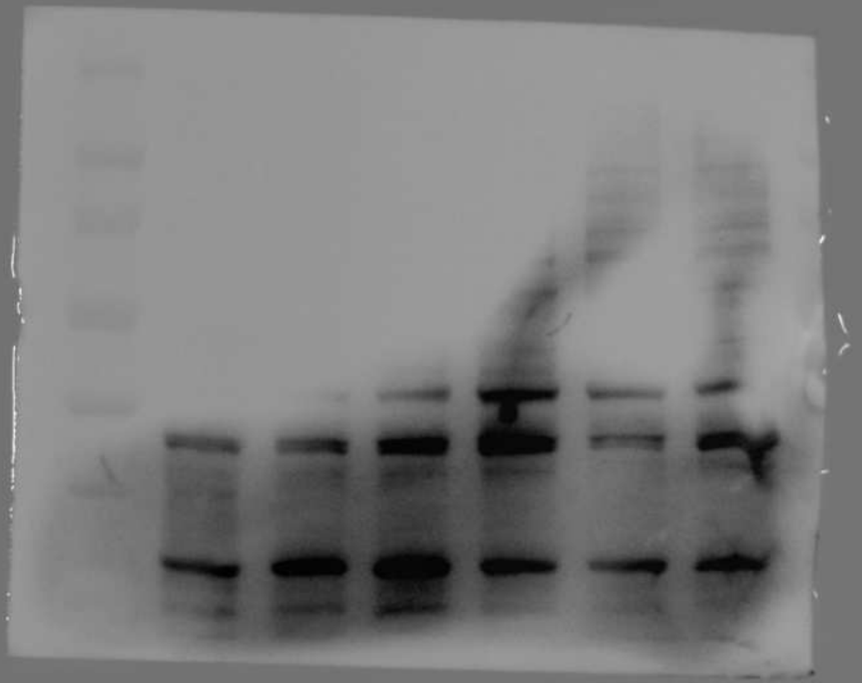

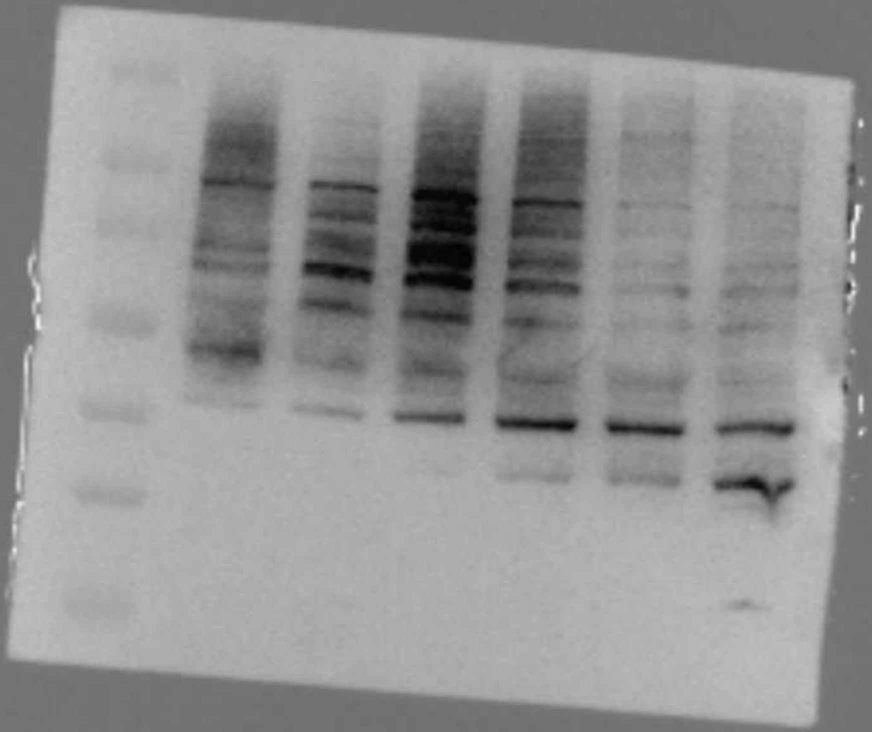

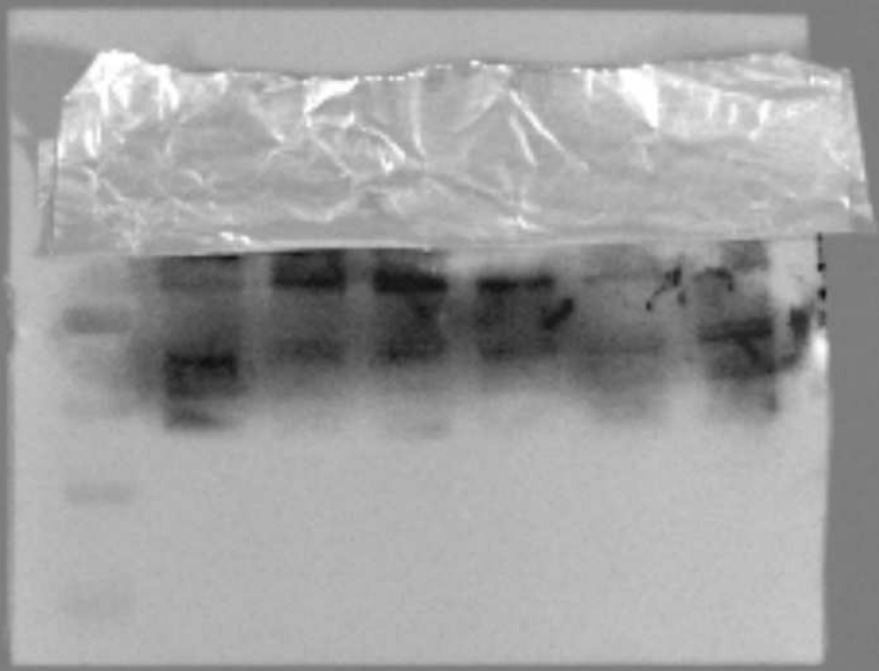

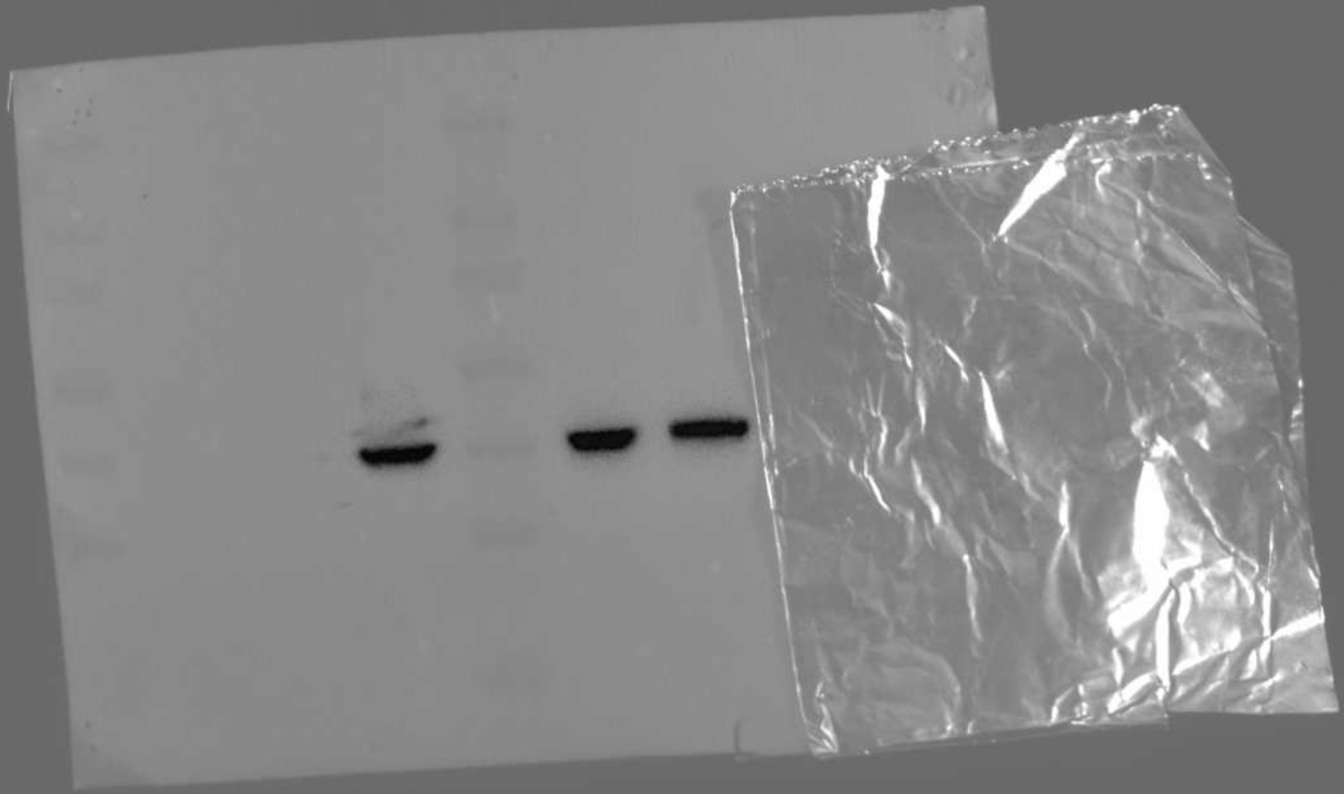

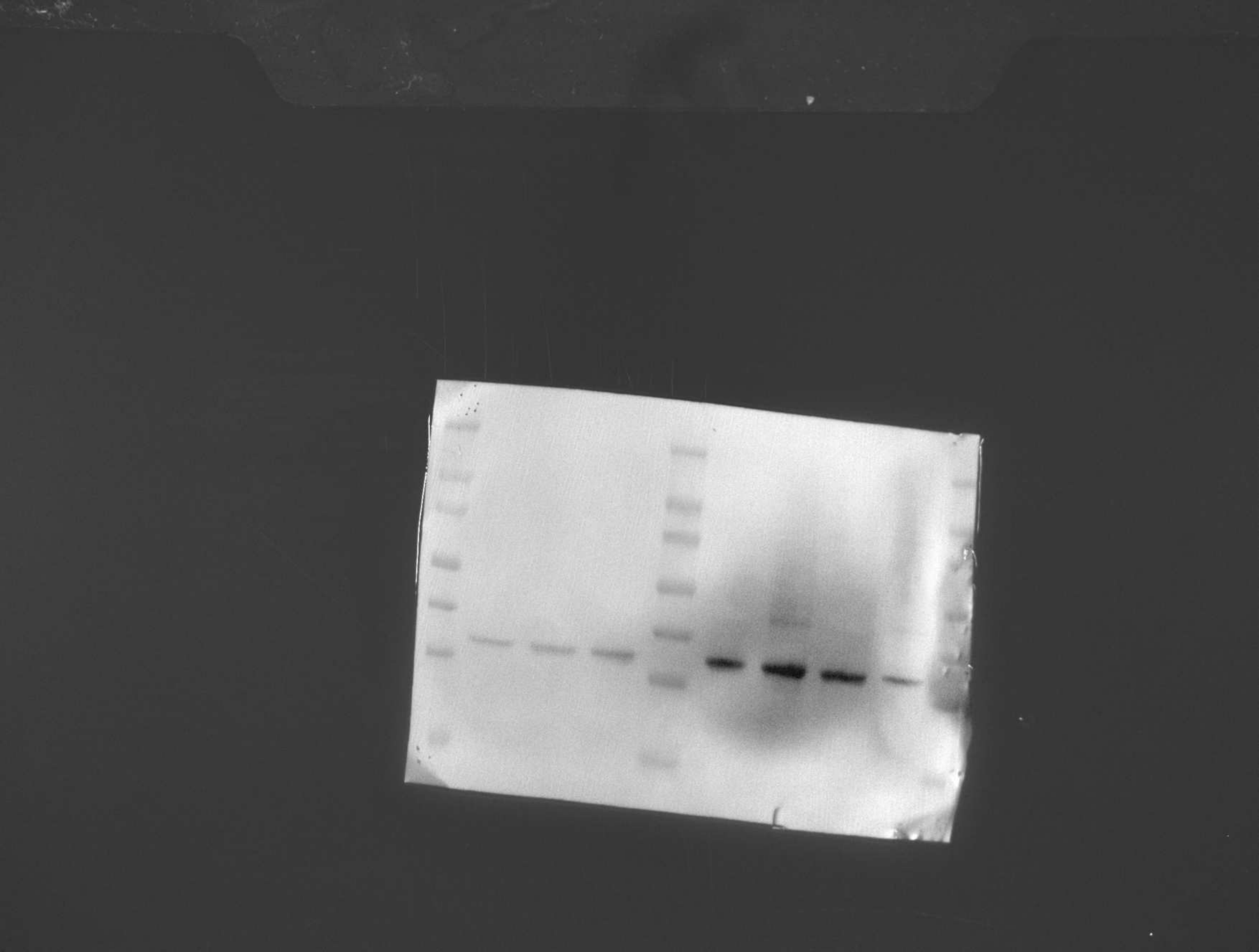

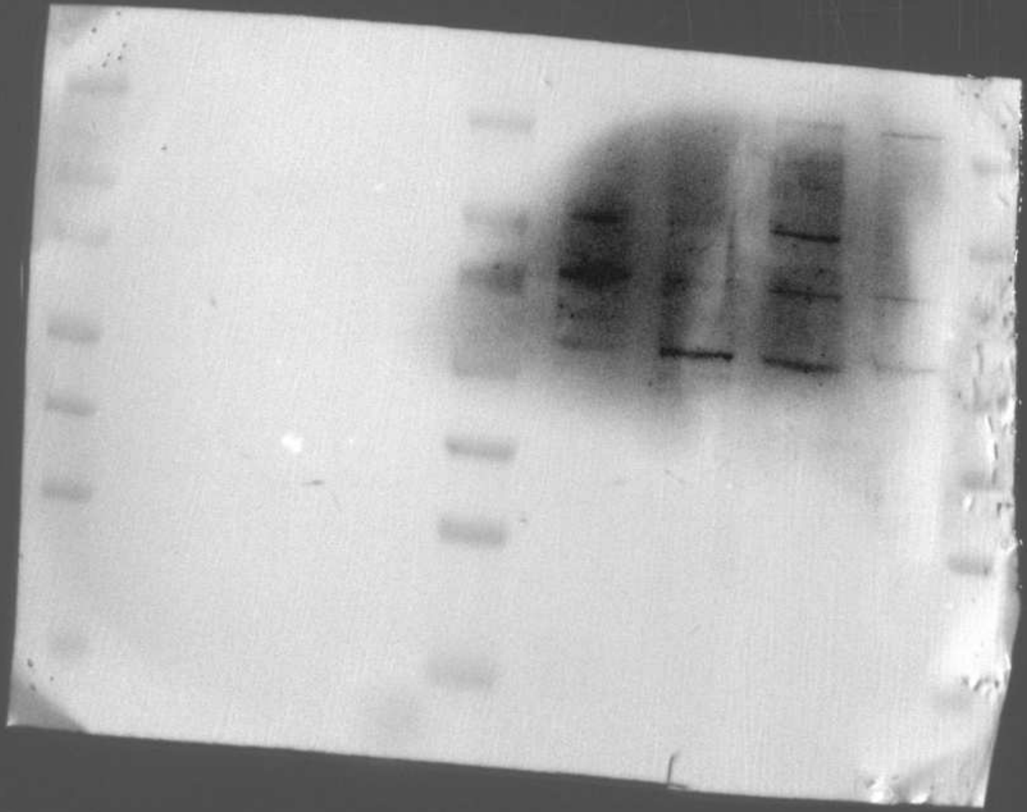

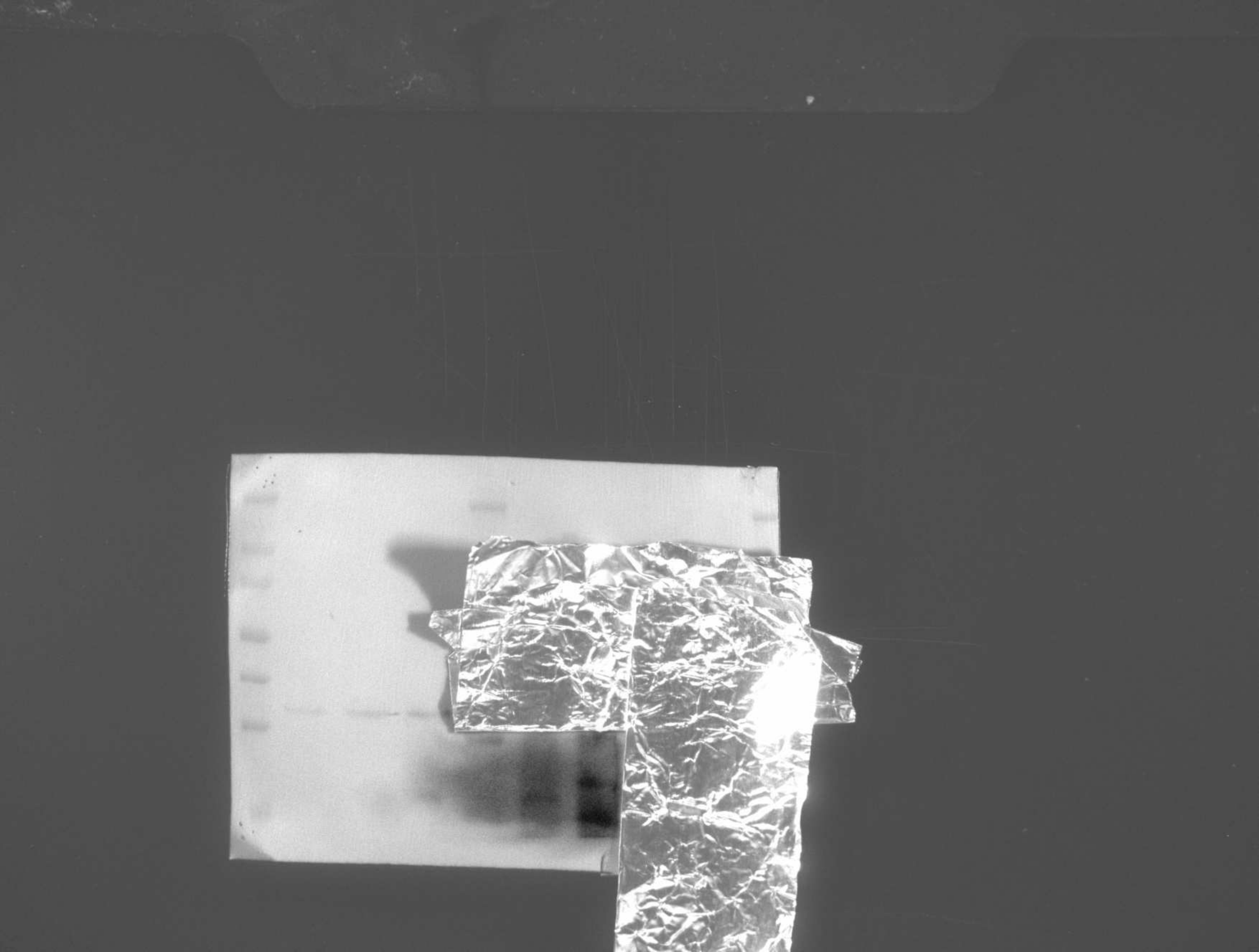

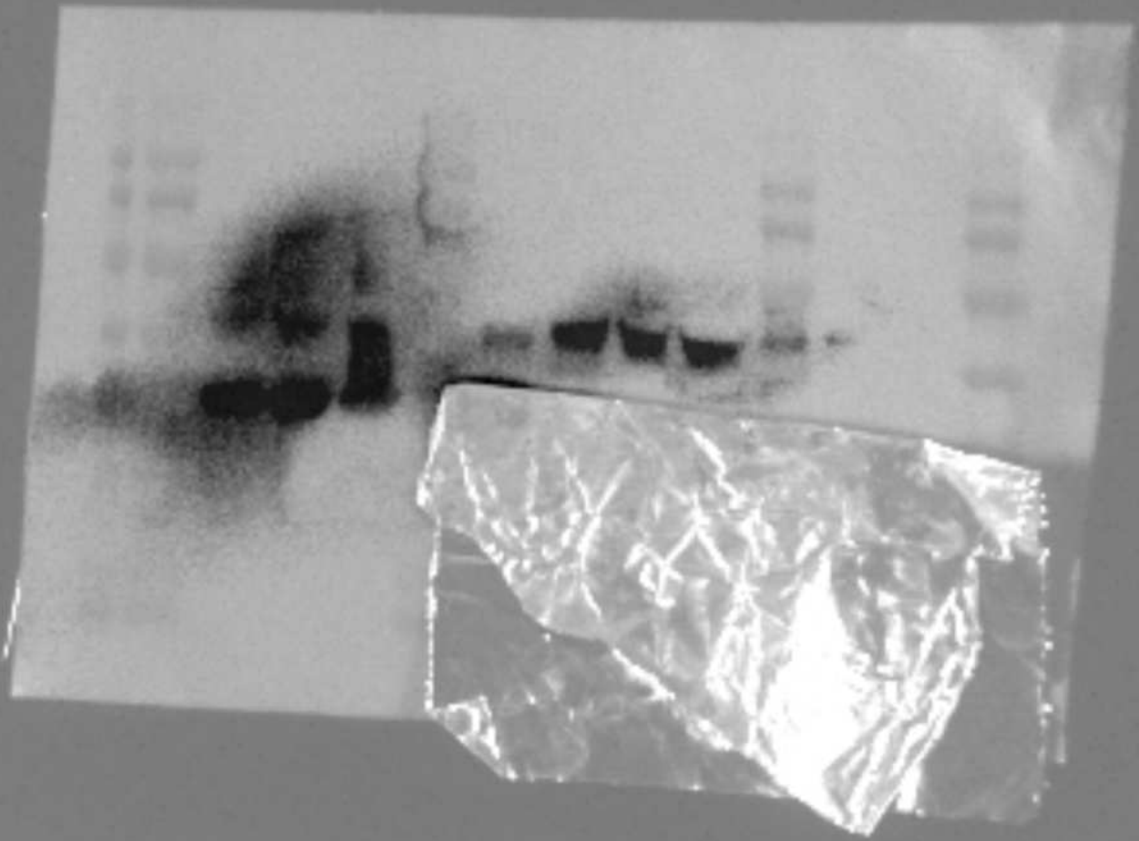

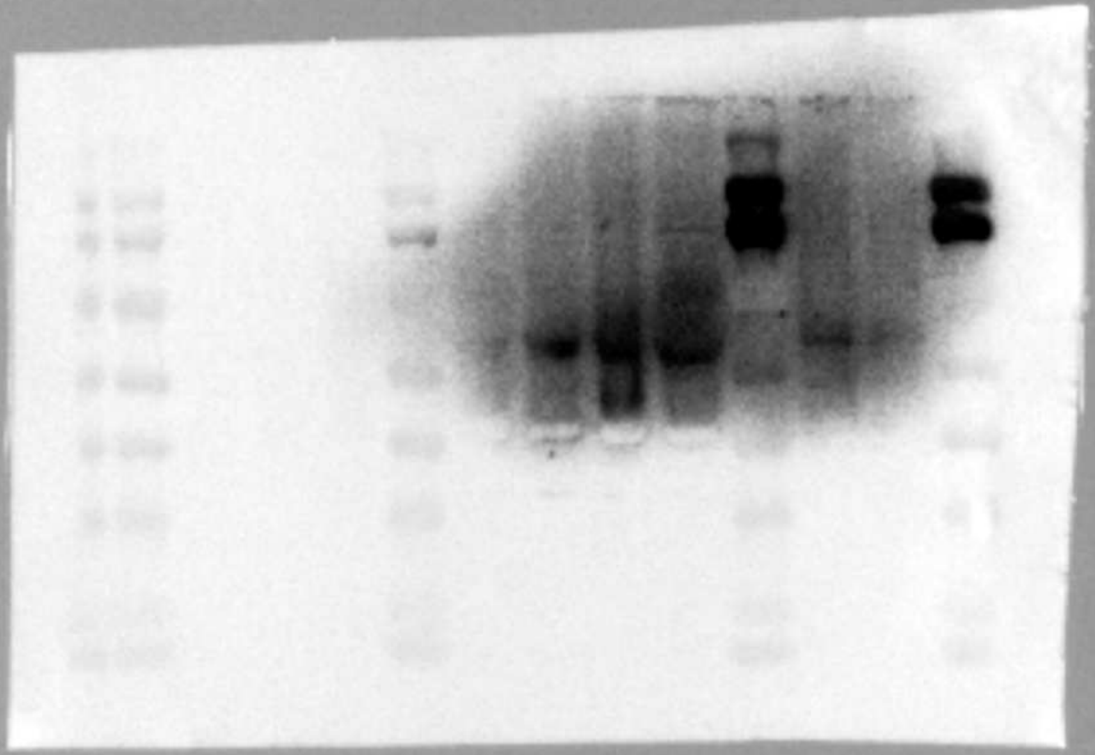

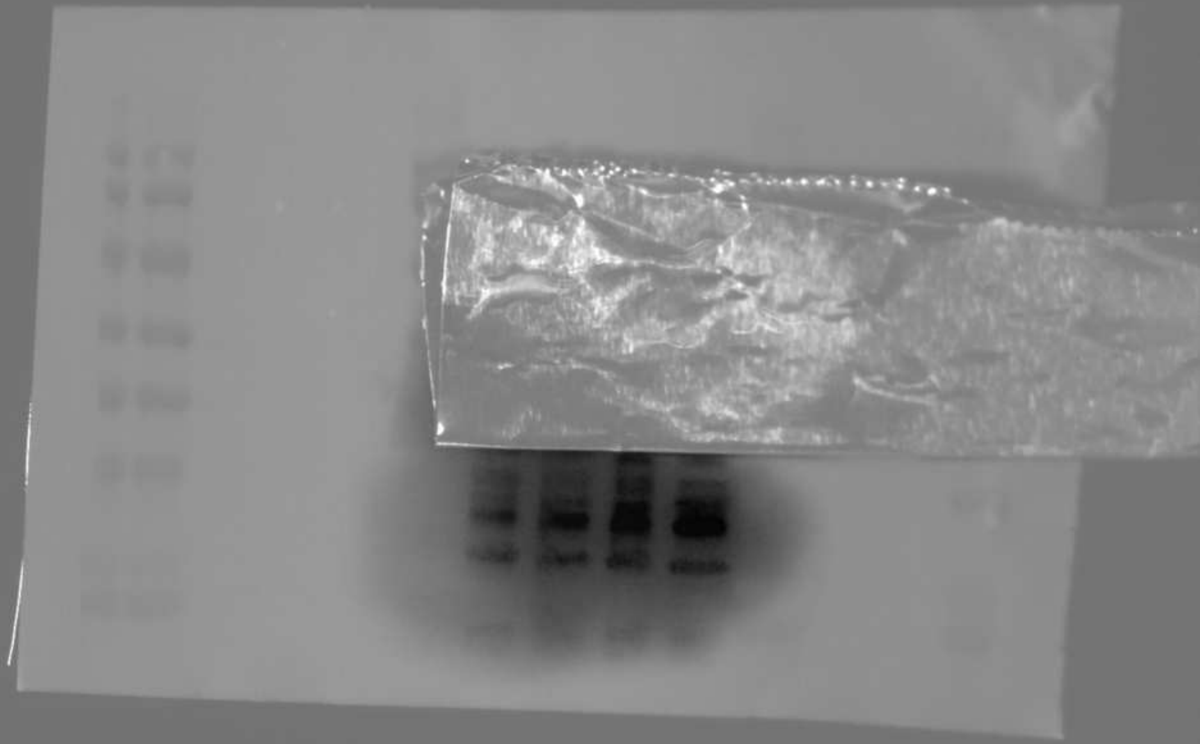

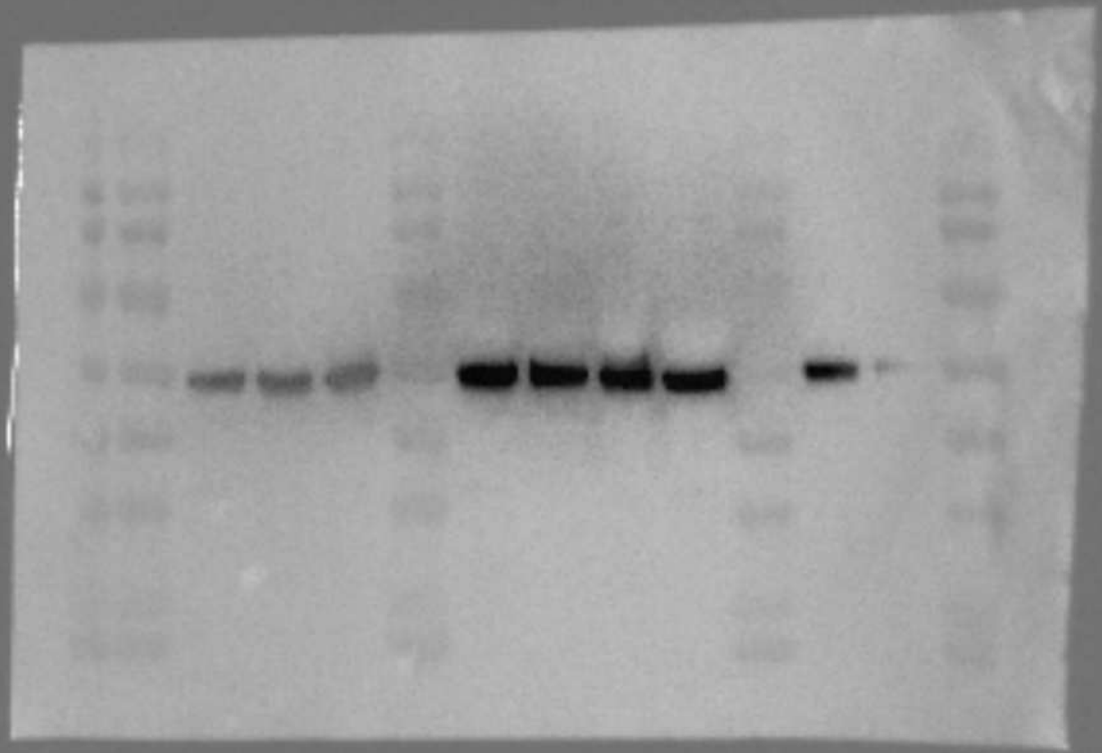

Fig.1 C

caseid	Cancer	IDH1[log2(TPM+1)]
TCGA-OR-A5LP-01A	ACC	6.71
TCGA-OR-A5K0-01A	ACC	7.69
TCGA-OR-A5JF-01A	ACC	6.8
TCGA-OR-A5JT-01A	ACC	5.63
TCGA-OR-A5K1-01A	ACC	5.36
TCGA-P6-A5OG-01A	ACC	6.06
TCGA-PK-A5H9-01A	ACC	7.2
TCGA-OR-A5KZ-01A	ACC	7.69
TCGA-OR-A5KX-01A	ACC	6.02
TCGA-OR-A5JY-01A	ACC	8.3
TCGA-PK-A5HA-01A	ACC	4.63
TCGA-OR-A5LR-01A	ACC	5.46
TCGA-OR-A5JQ-01A	ACC	5.69
TCGA-OR-A5LG-01A	ACC	7.76
TCGA-OR-A5LB-01A	ACC	6.9
TCGA-OR-A5JM-01A	ACC	8.55
TCGA-OR-A5LJ-01A	ACC	6.99
TCGA-OR-A5J3-01A	ACC	7.9
TCGA-OR-A5J6-01A	ACC	8.62
TCGA-OR-A5L3-01A	ACC	5.47
TCGA-OR-A5J1-01A	ACC	6.73
TCGA-OR-A5LA-01A	ACC	3.97
TCGA-OR-A5LH-01A	ACC	6.72
TCGA-OR-A5JR-01A	ACC	5.75
TCGA-OR-A5JK-01A	ACC	4.55
TCGA-OR-A5K9-01A	ACC	8.77
TCGA-OR-A5LC-01A	ACC	8.19
TCGA-OR-A5J9-01A	ACC	6.98
TCGA-OR-A5LE-01A	ACC	8.79
TCGA-OR-A5LL-01A	ACC	8.08
TCGA-OR-A5K8-01A	ACC	4.69
TCGA-OR-A5LO-01A	ACC	6.32
TCGA-OR-A5JD-01A	ACC	5.15
TCGA-OR-A5K6-01A	ACC	8.21
TCGA-OR-A5KW-01A	ACC	6.56
TCGA-OR-A5L5-01A	ACC	6.1
TCGA-OR-A5JP-01A	ACC	7.7
TCGA-PA-A5YG-01A	ACC	6.12
TCGA-PK-A5H8-01A	ACC	6.28
TCGA-OR-A5KY-01A	ACC	7.75
TCGA-OR-A5JX-01A	ACC	4.59
TCGA-OR-A5JW-01A	ACC	7.03
TCGA-OR-A5J5-01A	ACC	3.85
TCGA-OR-A5J8-01A	ACC	6.76
TCGA-PK-A5HB-01A	ACC	7.4
TCGA-OR-A5JL-01A	ACC	7.74
TCGA-OR-A5L8-01A	ACC	6.74
TCGA-OR-A5JG-01A	ACC	7
TCGA-OR-A5LK-01A	ACC	3.89
TCGA-OR-A5JV-01A	ACC	5.95
TCGA-OR-A5K3-01A	ACC	4.11
TCGA-OR-A5L4-01A	ACC	6.2
TCGA-OR-A5LM-01A	ACC	5.75
TCGA-OR-A5KO-01A	ACC	6.79
TCGA-OR-A5JI-01A	ACC	5.5
TCGA-OR-A5JB-01A	ACC	6.06

TCGA-OR-A5JE-01A	ACC	7.99
TCGA-OR-A5LD-01A	ACC	5.73
TCGA-OR-A5JC-01A	ACC	5.1
TCGA-OR-A5KV-01A	ACC	7.89
TCGA-OR-A5JO-01A	ACC	6.25
TCGA-OR-A5LN-01A	ACC	3.79
TCGA-P6-A5OF-01A	ACC	6.93
TCGA-OR-A5LT-01A	ACC	5.1
TCGA-OR-A5JJ-01A	ACC	6.79
TCGA-OR-A5J2-01A	ACC	7
TCGA-OR-A5KU-01A	ACC	6.04
TCGA-OR-A5J7-01A	ACC	8.79
TCGA-OR-A5JA-01A	ACC	6.74
TCGA-OR-A5JS-01A	ACC	8.14
TCGA-OR-A5K2-01A	ACC	7.89
TCGA-OR-A5K4-01A	ACC	5.83
TCGA-OR-A5JZ-01A	ACC	5.39
TCGA-OR-A5K5-01A	ACC	7.97
TCGA-OR-A5L6-01A	ACC	4.61
TCGA-OR-A5LS-01A	ACC	6.9
TCGA-OU-A5PI-01A	ACC	8
TCGA-OR-A5KT-01A	ACC	7.61
TCGA-OR-A5L9-01A	ACC	1.85
TCGA-E7-A678-01A	BLCA	7.65
TCGA-UY-A9PB-01A	BLCA	5.33
TCGA-DK-A3IN-01A	BLCA	8.08
TCGA-DK-A3IV-01A	BLCA	7.91
TCGA-XF-A9T4-01A	BLCA	6.93
TCGA-FD-A62S-01A	BLCA	6.06
TCGA-FD-A6TG-01A	BLCA	7.37
TCGA-FD-A6TI-01A	BLCA	7.84
TCGA-DK-AA6L-01A	BLCA	8.14
TCGA-FD-A5BT-01A	BLCA	4.57
TCGA-KQ-A41S-01A	BLCA	7.7
TCGA-ZF-AA4V-01A	BLCA	6.46
TCGA-FT-A3EE-01A	BLCA	6.31
TCGA-DK-AA75-01A	BLCA	8.53
TCGA-ZF-AA54-01A	BLCA	7.07
TCGA-FD-A3N5-01A	BLCA	5.66
TCGA-E7-A677-01A	BLCA	6.31
TCGA-DK-A1AB-01A	BLCA	7.92
TCGA-MV-A51V-01A	BLCA	7.66
TCGA-BT-A20N-01A	BLCA	7.81
TCGA-DK-A2I6-01A	BLCA	6.01
TCGA-ZF-AA56-01A	BLCA	8.29
TCGA-HQ-A5NE-01A	BLCA	6.86
TCGA-GV-A6ZA-01A	BLCA	7.96
TCGA-XF-A9T5-01A	BLCA	7.77
TCGA-ZF-AA5N-01A	BLCA	7.11
TCGA-ZF-A9RF-01A	BLCA	6.24
TCGA-BT-A20R-01A	BLCA	7.25
TCGA-SY-A9G5-01A	BLCA	6.45
TCGA-ZF-A9RM-01A	BLCA	9.6
TCGA-CF-A27C-01A	BLCA	8.88
TCGA-CU-A3KJ-01A	BLCA	5.15
TCGA-GC-A4ZW-01A	BLCA	5.59
TCGA-XF-AAN8-01A	BLCA	5.53
TCGA-K4-A83P-01A	BLCA	7.9

TCGA-GV-A40E-01A	BLCA	4.96
TCGA-CF-A9FL-01A	BLCA	7.04
TCGA-CF-A47Y-01A	BLCA	6.48
TCGA-DK-A1A6-01A	BLCA	8.4
TCGA-FD-A62N-01A	BLCA	5.2
TCGA-E7-A6ME-01A	BLCA	9.42
TCGA-GU-AATQ-01A	BLCA	7.99
TCGA-FD-A5BZ-01A	BLCA	5.98
TCGA-ZF-AA4N-01A	BLCA	6.99
TCGA-XF-A9SJ-01A	BLCA	6.79
TCGA-BL-A0C8-01B	BLCA	9.31
TCGA-BT-A42C-01A	BLCA	8.51
TCGA-CF-A5UA-01A	BLCA	7.99
TCGA-E5-A4TZ-01A	BLCA	5.63
TCGA-G2-A2EJ-01A	BLCA	6.18
TCGA-KQ-A41R-01A	BLCA	6.7
TCGA-2F-A9KO-01A	BLCA	8.06
TCGA-XF-AAMZ-01A	BLCA	8.47
TCGA-DK-A1AF-01A	BLCA	7.87
TCGA-DK-AA6T-01A	BLCA	5.38
TCGA-BL-A13I-01A	BLCA	6.91
TCGA-GU-A42P-01A	BLCA	7.77
TCGA-UY-A8OD-01A	BLCA	6.86
TCGA-DK-A2HX-01A	BLCA	6.68
TCGA-UY-A78P-01A	BLCA	7.41
TCGA-DK-A6AV-01A	BLCA	7.59
TCGA-BL-A0C8-01A	BLCA	9.43
TCGA-CU-A3QU-01A	BLCA	9.01
TCGA-XF-A9SX-01A	BLCA	5.28
TCGA-FJ-A871-01A	BLCA	5.04
TCGA-ZF-A9R2-01A	BLCA	7.86
TCGA-DK-A1A3-01A	BLCA	6.87
TCGA-FJ-A3ZF-01A	BLCA	8.48
TCGA-E7-A6MF-01A	BLCA	7.24
TCGA-E7-A4IJ-01A	BLCA	8.95
TCGA-FD-A6TA-01A	BLCA	8.54
TCGA-UY-A8OB-01A	BLCA	4.36
TCGA-ZF-A9RL-01A	BLCA	9.66
TCGA-FD-A6TB-01A	BLCA	6.86
TCGA-XF-AAMG-01A	BLCA	6.31
TCGA-CF-A1HR-01A	BLCA	8.05
TCGA-CF-A3MG-01A	BLCA	9.15
TCGA-ZF-AA5P-01A	BLCA	6.26
TCGA-DK-AA74-01A	BLCA	5.64
TCGA-DK-A1A7-01A	BLCA	7.36
TCGA-SY-A9G0-01A	BLCA	7.98
TCGA-UY-A78N-01A	BLCA	7.25
TCGA-DK-A3IT-01A	BLCA	8.86
TCGA-ZF-AA4U-01A	BLCA	8.62
TCGA-UY-A8OC-01A	BLCA	5.59
TCGA-4Z-AA87-01A	BLCA	7.95
TCGA-ZF-A9R0-01A	BLCA	8.23
TCGA-4Z-AA7Y-01A	BLCA	8.95
TCGA-K4-A54R-01A	BLCA	6.65
TCGA-FD-A5BV-01A	BLCA	7.86
TCGA-K4-A6FZ-01A	BLCA	7.97
TCGA-LT-A8JT-01A	BLCA	8.33
TCGA-BL-A13J-01A	BLCA	6.76

TCGA-XF-A9T0-01A	BLCA	7.17
TCGA-FD-A5BX-01A	BLCA	5.52
TCGA-DK-A3X1-01A	BLCA	7.43
TCGA-XF-A9T3-01A	BLCA	5.62
TCGA-G2-A2ES-01A	BLCA	7.06
TCGA-R3-A69X-01A	BLCA	9.36
TCGA-FD-A3SP-01A	BLCA	6.26
TCGA-K4-A6MB-01A	BLCA	8.33
TCGA-GD-A76B-01A	BLCA	9.04
TCGA-FJ-A3Z9-01A	BLCA	7.55
TCGA-BT-A3PK-01A	BLCA	5.78
TCGA-CF-A9FF-01A	BLCA	7.33
TCGA-GC-A3I6-01A	BLCA	6.84
TCGA-CF-A47X-01A	BLCA	6.93
TCGA-S5-A6DX-01A	BLCA	4.58
TCGA-4Z-AA7N-01A	BLCA	7.24
TCGA-FD-A5BU-01A	BLCA	6.76
TCGA-XF-AAN2-01A	BLCA	6.82
TCGA-G2-AA3F-01A	BLCA	9.18
TCGA-FT-A61P-01A	BLCA	5.53
TCGA-K4-A3WU-01B	BLCA	8.13
TCGA-ZF-A9R5-01A	BLCA	7.78
TCGA-CF-A9FM-01A	BLCA	8.13
TCGA-5N-A9KM-01A	BLCA	8.67
TCGA-GV-A3QK-01B	BLCA	8.01
TCGA-GU-A42Q-01A	BLCA	7.11
TCGA-C4-A0F1-01A	BLCA	5.81
TCGA-DK-A6B6-01A	BLCA	9.19
TCGA-ZF-AA53-01A	BLCA	5.77
TCGA-G2-AA3C-01A	BLCA	6.69
TCGA-E7-A3X6-01A	BLCA	8.17
TCGA-FD-A5BS-01A	BLCA	4.41
TCGA-BL-A5ZZ-01A	BLCA	6.3
TCGA-DK-A3IL-01A	BLCA	6.66
TCGA-XF-A8HC-01A	BLCA	8.9
TCGA-CF-A47T-01A	BLCA	6.53
TCGA-ZF-AA4T-01A	BLCA	8.3
TCGA-E7-A97P-01A	BLCA	6.99
TCGA-BL-A3JM-01A	BLCA	5.87
TCGA-FD-A5C1-01A	BLCA	6.03
TCGA-2F-A9KQ-01A	BLCA	7.06
TCGA-CF-A3MF-01A	BLCA	3.19
TCGA-G2-AA3B-01A	BLCA	8.86
TCGA-CF-A47W-01A	BLCA	7.68
TCGA-FD-A43U-01A	BLCA	6.28
TCGA-G2-A2EC-01A	BLCA	7.96
TCGA-FD-A3SQ-01A	BLCA	7.09
TCGA-XF-A9T2-01A	BLCA	5.14
TCGA-E7-A4XJ-01A	BLCA	6.21
TCGA-DK-A3IS-01A	BLCA	8.57
TCGA-XF-A9SH-01A	BLCA	7.57
TCGA-GC-A3RC-01A	BLCA	5.72
TCGA-PQ-A6FN-01A	BLCA	4.84
TCGA-CF-A3MH-01A	BLCA	7.41
TCGA-BT-A20X-01A	BLCA	5.2
TCGA-XF-AAME-01A	BLCA	6.19
TCGA-XF-A8HG-01A	BLCA	7.86
TCGA-XF-AAN5-01A	BLCA	7.34

TCGA-FD-A62P-01A	BLCA	6.64
TCGA-DK-A6B0-01A	BLCA	7.21
TCGA-XF-AAMR-01A	BLCA	7.6
TCGA-4Z-AA7W-01A	BLCA	5.54
TCGA-E7-A8O8-01A	BLCA	5.58
TCGA-FD-A3N6-01A	BLCA	5.23
TCGA-4Z-AA83-01A	BLCA	6.19
TCGA-E7-A6MD-01A	BLCA	8.47
TCGA-GC-A3BM-01A	BLCA	7.96
TCGA-CU-A5W6-01A	BLCA	8.67
TCGA-FD-A3SO-01A	BLCA	6.71
TCGA-KQ-A41O-01A	BLCA	7.44
TCGA-G2-A2EL-01A	BLCA	4.9
TCGA-XF-A9T8-01A	BLCA	5.79
TCGA-XF-AAN3-01A	BLCA	7.4
TCGA-GU-AATP-01A	BLCA	6.04
TCGA-E7-A7DV-01A	BLCA	7.1
TCGA-FD-A62O-01A	BLCA	8.02
TCGA-XF-A9SU-01A	BLCA	6.79
TCGA-C4-A0F0-01A	BLCA	5.91
TCGA-DK-A2I1-01A	BLCA	7.92
TCGA-FD-A3SL-01A	BLCA	8.3
TCGA-ZF-AA5H-01A	BLCA	6.56
TCGA-DK-AA6P-01A	BLCA	9.66
TCGA-BT-A2LD-01A	BLCA	5.31
TCGA-DK-A3WY-01A	BLCA	4.76
TCGA-YC-A8S6-01A	BLCA	6.31
TCGA-UY-A9PD-01A	BLCA	7.98
TCGA-GD-A6C6-01A	BLCA	6.41
TCGA-ZF-AA4R-01A	BLCA	7.5
TCGA-GU-A767-01A	BLCA	8.43
TCGA-FD-A6TD-01A	BLCA	6.22
TCGA-CU-A72E-01A	BLCA	7.3
TCGA-E7-A85H-01A	BLCA	8.73
TCGA-XF-AAN4-01A	BLCA	5.88
TCGA-2F-A9KT-01A	BLCA	6.08
TCGA-GD-A3OP-01A	BLCA	8.69
TCGA-LC-A66R-01A	BLCA	4.67
TCGA-XF-AAML-01A	BLCA	9
TCGA-UY-A9PF-01A	BLCA	7.27
TCGA-DK-A3IM-01A	BLCA	5.05
TCGA-FD-A3B5-01A	BLCA	5.9
TCGA-XF-A9SL-01A	BLCA	7.07
TCGA-XF-AAMH-01A	BLCA	5.05
TCGA-E5-A4U1-01A	BLCA	7.61
TCGA-GU-A764-01A	BLCA	5.26
TCGA-CF-A7I0-01A	BLCA	7.56
TCGA-4Z-AA7M-01A	BLCA	6.82
TCGA-E7-A7XN-01A	BLCA	6.39
TCGA-4Z-AA82-01A	BLCA	6.7
TCGA-PQ-A6FI-01A	BLCA	5.52
TCGA-C4-A0EZ-01A	BLCA	4.36
TCGA-CU-A3YL-01A	BLCA	5.95
TCGA-FD-A5C0-01A	BLCA	8.48
TCGA-HQ-A5ND-01A	BLCA	8.18
TCGA-GU-A42R-01A	BLCA	7.31
TCGA-UY-A78M-01A	BLCA	6.78
TCGA-DK-A6B1-01A	BLCA	8.06

TCGA-ZF-AA58-01A	BLCA	5.55
TCGA-ZF-AA51-01A	BLCA	7.42
TCGA-XF-AAMW-01A	BLCA	6.13
TCGA-GV-A40G-01A	BLCA	8.64
TCGA-DK-AA6U-01A	BLCA	8.67
TCGA-K4-A4AC-01A	BLCA	5.44
TCGA-BT-A20V-01A	BLCA	6.25
TCGA-DK-A6B2-01A	BLCA	8.26
TCGA-BT-A0YX-01A	BLCA	7.92
TCGA-ZF-A9RE-01A	BLCA	7.63
TCGA-GD-A3OQ-01A	BLCA	7.94
TCGA-DK-AA71-01A	BLCA	7.6
TCGA-FD-A3NA-01A	BLCA	9.13
TCGA-YF-AA3M-01A	BLCA	7.22
TCGA-GV-A3QI-01A	BLCA	8.98
TCGA-GC-A3OO-01A	BLCA	8.27
TCGA-DK-A3WW-01A	BLCA	6.19
TCGA-DK-A2I2-01A	BLCA	6.2
TCGA-K4-A5RI-01A	BLCA	9.12
TCGA-ZF-A9R7-01A	BLCA	8.03
TCGA-K4-A5RH-01A	BLCA	5.98
TCGA-G2-AA3D-01A	BLCA	8.53
TCGA-UY-A9PE-01A	BLCA	6.71
TCGA-4Z-AA7Q-01A	BLCA	6.98
TCGA-DK-A1AG-01A	BLCA	7.78
TCGA-UY-A9PH-01A	BLCA	8.52
TCGA-CF-A1HS-01A	BLCA	5.58
TCGA-H4-A2HQ-01A	BLCA	8.7
TCGA-G2-A3VY-01A	BLCA	9.17
TCGA-CF-A47S-01A	BLCA	6.92
TCGA-K4-A3WV-01A	BLCA	7.31
TCGA-XF-A9SZ-01A	BLCA	7.4
TCGA-DK-A6B5-01A	BLCA	7.05
TCGA-DK-A3IU-01A	BLCA	5.34
TCGA-DK-AA76-01A	BLCA	9.78
TCGA-KQ-A41P-01A	BLCA	6.55
TCGA-ZF-AA4X-01A	BLCA	8.16
TCGA-ZF-A9R1-01A	BLCA	8.56
TCGA-GU-A766-01A	BLCA	4.85
TCGA-XF-A8HD-01A	BLCA	7.07
TCGA-BT-A20P-01A	BLCA	7.55
TCGA-DK-AA77-01A	BLCA	7.75
TCGA-E7-A541-01A	BLCA	7.66
TCGA-BT-A3PJ-01A	BLCA	4.49
TCGA-G2-A3IE-01A	BLCA	7.83
TCGA-BL-A13I-01B	BLCA	7.63
TCGA-FD-A43Y-01A	BLCA	5.87
TCGA-LT-A5Z6-01A	BLCA	8.21
TCGA-UY-A78L-01A	BLCA	7.77
TCGA-DK-AA6M-01A	BLCA	6.03
TCGA-XF-AAN0-01A	BLCA	6.01
TCGA-FD-A5BR-01A	BLCA	7.17
TCGA-GV-A3JX-01A	BLCA	8.66
TCGA-GU-A763-01A	BLCA	6.78
TCGA-XF-A8HI-01A	BLCA	8.31
TCGA-ZF-A9RD-01A	BLCA	6.19
TCGA-DK-A3IQ-01A	BLCA	5.78
TCGA-GU-AATO-01A	BLCA	6.78

TCGA-BT-A2LB-01A	BLCA	9.15
TCGA-GC-A3WC-01A	BLCA	7.29
TCGA-XF-A9SV-01A	BLCA	9.19
TCGA-4Z-AA7S-01A	BLCA	8.63
TCGA-CF-A47V-01A	BLCA	5.84
TCGA-DK-A3WX-01A	BLCA	5.91
TCGA-CF-A3MI-01A	BLCA	5.72
TCGA-XF-AAMX-01A	BLCA	8.37
TCGA-BT-A42F-01A	BLCA	5.85
TCGA-2F-A9KR-01A	BLCA	8.02
TCGA-FD-A3B7-01A	BLCA	5.53
TCGA-ZF-A9R9-01A	BLCA	5.61
TCGA-XF-A9SW-01A	BLCA	5.42
TCGA-E7-A3Y1-01A	BLCA	6.04
TCGA-DK-A2I4-01A	BLCA	6.7
TCGA-FD-A6TE-01A	BLCA	7.86
TCGA-GD-A2C5-01A	BLCA	8.34
TCGA-XF-AAN1-01A	BLCA	7.9
TCGA-DK-AA6R-01A	BLCA	4.88
TCGA-UY-A78K-01A	BLCA	8.88
TCGA-DK-AA6W-01A	BLCA	7.01
TCGA-E7-A7DU-01A	BLCA	8.31
TCGA-YF-AA3L-01A	BLCA	6.25
TCGA-GU-A762-01A	BLCA	5.72
TCGA-E7-A519-01A	BLCA	4.93
TCGA-FD-A3SJ-01A	BLCA	9.06
TCGA-XF-A8HE-01A	BLCA	7.21
TCGA-YC-A9TC-01A	BLCA	6.24
TCGA-GC-A3RD-01A	BLCA	7.48
TCGA-XF-A9SP-01A	BLCA	6.9
TCGA-XF-A8HF-01A	BLCA	8.58
TCGA-XF-AAMQ-01A	BLCA	7.25
TCGA-FD-A43S-01A	BLCA	7.18
TCGA-CF-A9FH-01A	BLCA	8.03
TCGA-XF-AAMJ-01A	BLCA	7.52
TCGA-4Z-AA86-01A	BLCA	5.92
TCGA-DK-A1A5-01A	BLCA	7.78
TCGA-GV-A3QG-01A	BLCA	6.85
TCGA-K4-A4AB-01B	BLCA	8.19
TCGA-XF-AAMY-01A	BLCA	8.03
TCGA-FD-A3SS-01A	BLCA	6.57
TCGA-CU-A0YR-01A	BLCA	9.21
TCGA-BT-A20J-01A	BLCA	5.96
TCGA-H4-A2HO-01A	BLCA	8.85
TCGA-4Z-AA7O-01A	BLCA	7.36
TCGA-XF-A9SY-01A	BLCA	5.44
TCGA-GV-A3JZ-01A	BLCA	7.87
TCGA-CF-A8HY-01A	BLCA	6.05
TCGA-DK-A1AD-01A	BLCA	8.94
TCGA-FD-A5BY-01A	BLCA	6.48
TCGA-BT-A3PH-01A	BLCA	7.06
TCGA-FD-A3B4-01A	BLCA	5.47
TCGA-G2-A2EO-01A	BLCA	7.64
TCGA-FJ-A3ZE-01A	BLCA	7.66
TCGA-G2-A3IB-01A	BLCA	7.5
TCGA-ZF-A9R3-01A	BLCA	8.69
TCGA-GC-A3RB-01A	BLCA	8.01
TCGA-XF-A9SM-01A	BLCA	6.15

TCGA-GC-A6I3-01A	BLCA	7.97
TCGA-S5-AA26-01A	BLCA	6.66
TCGA-XF-AAMT-01A	BLCA	6.06
TCGA-BT-A20O-01A	BLCA	7.32
TCGA-C4-A0F7-01A	BLCA	7.16
TCGA-BT-A42E-01A	BLCA	5.3
TCGA-BT-A2LA-01A	BLCA	4.99
TCGA-BT-A20U-01A	BLCA	8.55
TCGA-4Z-AA84-01A	BLCA	6.1
TCGA-XF-A8HB-01A	BLCA	6.55
TCGA-KQ-A41Q-01A	BLCA	8.38
TCGA-YC-A89H-01A	BLCA	7.05
TCGA-XF-AAN7-01A	BLCA	5.88
TCGA-FD-A6TC-01A	BLCA	7.74
TCGA-CF-A5U8-01A	BLCA	7.79
TCGA-DK-A3IK-01A	BLCA	7.59
TCGA-GC-A3YS-01A	BLCA	5.55
TCGA-GV-A3JV-01A	BLCA	6.67
TCGA-FD-A3SM-01A	BLCA	8.9
TCGA-DK-A1AC-01A	BLCA	9.13
TCGA-ZF-A9R4-01A	BLCA	8.98
TCGA-XF-A8HH-01A	BLCA	7.84
TCGA-XF-A9SK-01A	BLCA	6.34
TCGA-E7-A5KE-01A	BLCA	8.35
TCGA-4Z-AA89-01A	BLCA	8.39
TCGA-4Z-AA80-01A	BLCA	8.92
TCGA-ZF-AA4W-01A	BLCA	5.36
TCGA-XF-A9SI-01A	BLCA	6.39
TCGA-BT-A0S7-01A	BLCA	6.14
TCGA-2F-A9KP-01A	BLCA	7.95
TCGA-DK-AA6Q-01A	BLCA	7.42
TCGA-ZF-A9RN-01A	BLCA	7.66
TCGA-4Z-AA7R-01A	BLCA	8.61
TCGA-ZF-AA52-01A	BLCA	6.5
TCGA-CU-A0YO-01A	BLCA	7.78
TCGA-C4-A0F6-01A	BLCA	9
TCGA-DK-A1AA-01A	BLCA	8.17
TCGA-G2-A2EF-01A	BLCA	5.58
TCGA-XF-A9ST-01A	BLCA	5.21
TCGA-2F-A9KW-01A	BLCA	6.31
TCGA-FD-A43N-01A	BLCA	7.69
TCGA-GV-A3QH-01A	BLCA	7.54
TCGA-HQ-A2OE-01A	BLCA	7.23
TCGA-GD-A3OS-01A	BLCA	4.87
TCGA-GV-A3JW-01A	BLCA	9.12
TCGA-UY-A9PA-01A	BLCA	8.23
TCGA-4Z-AA81-01A	BLCA	6.49
TCGA-K4-AAQO-01A	BLCA	8.63
TCGA-DK-A3X2-01A	BLCA	6.96
TCGA-GV-A3QF-01A	BLCA	9.38
TCGA-CF-A8HX-01A	BLCA	6.14
TCGA-E5-A2PC-01A	BLCA	9.52
TCGA-DK-A1AE-01A	BLCA	6.91
TCGA-K4-A5RJ-01A	BLCA	8.41
TCGA-DK-AA6S-01A	BLCA	6.17
TCGA-ZF-A9RC-01A	BLCA	6.79
TCGA-E7-A5KF-01A	BLCA	9.26
TCGA-5N-A9KI-01A	BLCA	7.54

TCGA-BT-A20Q-01A	BLCA	8.26
TCGA-G2-A2EK-01A	BLCA	8
TCGA-CU-A0YN-01A	BLCA	4.93
TCGA-KQ-A41N-01A	BLCA	6.3
TCGA-GC-A6I1-01A	BLCA	5.98
TCGA-XF-A9T6-01A	BLCA	5.67
TCGA-FD-A3SR-01A	BLCA	6.66
TCGA-FD-A43X-01A	BLCA	7.81
TCGA-FD-A3B3-01A	BLCA	5.15
TCGA-FD-A3B8-01A	BLCA	6.17
TCGA-FD-A6TF-01A	BLCA	5.23
TCGA-K4-A3WS-01A	BLCA	7.74
TCGA-FD-A6TK-01A	BLCA	6.22
TCGA-FJ-A3Z7-01A	BLCA	7.23
TCGA-HQ-A2OF-01A	BLCA	9.4
TCGA-BL-A13J-01B	BLCA	6.63
TCGA-FD-A43P-01A	BLCA	9.23
TCGA-FD-A3B6-01A	BLCA	6.46
TCGA-UY-A78O-01A	BLCA	8.6
TCGA-BT-A20W-01A	BLCA	8.5
TCGA-FD-A3SN-01A	BLCA	5.46
TCGA-DK-AA6X-01A	BLCA	7.86
TCGA-E7-A8O7-01A	BLCA	8.91
TCGA-E7-A7PW-01A	BLCA	8.7
TCGA-FD-A6TH-01A	BLCA	6.38
TCGA-BT-A20T-01A	BLCA	7.43
TCGA-A7-A26E-01B	BRCA	6.6
TCGA-A2-A0CU-01A	BRCA	5.67
TCGA-PL-A8LV-01A	BRCA	5.13
TCGA-BH-A0BC-01A	BRCA	6.07
TCGA-AR-A1AX-01A	BRCA	6.37
TCGA-AC-A2FO-01A	BRCA	7.31
TCGA-AQ-A0Y5-01A	BRCA	6.46
TCGA-AC-A3EH-01A	BRCA	6.12
TCGA-AC-A5EH-01A	BRCA	6.94
TCGA-D8-A142-01A	BRCA	6.27
TCGA-D8-A1XB-01A	BRCA	6.07
TCGA-D8-A1JJ-01A	BRCA	7.01
TCGA-E2-A10B-01A	BRCA	6.44
TCGA-A2-A0CP-01A	BRCA	5.33
TCGA-BH-A1FJ-01A	BRCA	7.33
TCGA-BH-A0C1-01B	BRCA	6.25
TCGA-OL-A66P-01A	BRCA	7.24
TCGA-E2-A14O-01A	BRCA	5.81
TCGA-D8-A1XJ-01A	BRCA	6.97
TCGA-AR-A24P-01A	BRCA	5.95
TCGA-AO-A1KQ-01A	BRCA	5.82
TCGA-D8-A145-01A	BRCA	6.14
TCGA-AR-A24V-01A	BRCA	5.56
TCGA-AO-A12F-01A	BRCA	6.55
TCGA-E9-A54X-01A	BRCA	5.41
TCGA-BH-A0BA-01A	BRCA	7.32
TCGA-EW-A1PH-01A	BRCA	6.05
TCGA-BH-A209-01A	BRCA	5.41
TCGA-E9-A2JS-01A	BRCA	6.28
TCGA-E9-A1R3-01A	BRCA	6.85
TCGA-E9-A1R6-01A	BRCA	5.31
TCGA-BH-A1FU-01A	BRCA	7.24

TCGA-D8-A1Y2-01A	BRCA	5.76
TCGA-A2-A4RX-01A	BRCA	5.06
TCGA-A8-A09V-01A	BRCA	6.2
TCGA-AO-A12B-01A	BRCA	6.58
TCGA-XX-A899-01A	BRCA	5.49
TCGA-A8-A08P-01A	BRCA	8
TCGA-C8-A26W-01A	BRCA	6.02
TCGA-E9-A249-01A	BRCA	5.44
TCGA-A8-A086-01A	BRCA	5.63
TCGA-B6-A0RV-01A	BRCA	7.11
TCGA-D8-A1JT-01A	BRCA	6.49
TCGA-OL-A5RV-01A	BRCA	6.66
TCGA-Z7-A8R5-01A	BRCA	5.93
TCGA-A8-A0AB-01A	BRCA	6.29
TCGA-E2-A154-01A	BRCA	5.84
TCGA-S3-A6ZG-01A	BRCA	5.57
TCGA-D8-A140-01A	BRCA	6.64
TCGA-A8-A06Q-01A	BRCA	5.95
TCGA-AR-A24Q-01A	BRCA	7.31
TCGA-A2-A1G6-01A	BRCA	7
TCGA-D8-A1XL-01A	BRCA	5.24
TCGA-A2-A1G0-01A	BRCA	5.62
TCGA-A8-A095-01A	BRCA	7.21
TCGA-AN-A0FV-01A	BRCA	7.32
TCGA-S3-AA12-01A	BRCA	5.18
TCGA-A8-A06T-01A	BRCA	5.32
TCGA-E2-A14Q-01A	BRCA	6.56
TCGA-BH-A18K-01A	BRCA	5.99
TCGA-A8-A06Y-01A	BRCA	6.38
TCGA-BH-A0AZ-01A	BRCA	6.31
TCGA-E9-A1RA-01A	BRCA	5.3
TCGA-UL-AAZ6-01A	BRCA	4.91
TCGA-BH-A1ES-01A	BRCA	6.35
TCGA-EW-A1PA-01A	BRCA	6.29
TCGA-EW-A1P4-01A	BRCA	6.42
TCGA-D8-A1JH-01A	BRCA	6.66
TCGA-B6-A409-01A	BRCA	5.12
TCGA-D8-A13Z-01A	BRCA	7.36
TCGA-AC-A7VB-01A	BRCA	8.11
TCGA-AN-A0XN-01A	BRCA	5.23
TCGA-GM-A2DN-01A	BRCA	5.52
TCGA-AO-A12G-01A	BRCA	5.73
TCGA-BH-A0B9-01A	BRCA	6.59
TCGA-C8-A273-01A	BRCA	6.85
TCGA-AR-A1AY-01A	BRCA	6.09
TCGA-AR-A1AO-01A	BRCA	7.62
TCGA-E9-A1RC-01A	BRCA	4.87
TCGA-A2-A0EN-01A	BRCA	5.84
TCGA-E9-A1RD-01A	BRCA	6.77
TCGA-A8-A08O-01A	BRCA	6.32
TCGA-AC-A2FE-01A	BRCA	6.35
TCGA-B6-A0RN-01A	BRCA	5.36
TCGA-A8-A076-01A	BRCA	8.12
TCGA-BH-A0HK-01A	BRCA	7.32
TCGA-BH-A0BO-01A	BRCA	6.31
TCGA-BH-A0HW-01A	BRCA	4.85
TCGA-D8-A1XZ-01A	BRCA	5.86
TCGA-OL-A66K-01A	BRCA	5.02

TCGA-AR-A24N-01A	BRCA	6.21
TCGA-E2-A1IU-01A	BRCA	7.63
TCGA-AR-A0TT-01A	BRCA	6.21
TCGA-B6-A0IP-01A	BRCA	6.19
TCGA-AO-A03L-01A	BRCA	6.37
TCGA-B6-A0IB-01A	BRCA	5.92
TCGA-E9-A54Y-01A	BRCA	4.98
TCGA-A2-A0T2-01A	BRCA	6.13
TCGA-AC-A3W6-01A	BRCA	5.26
TCGA-A2-A3XV-01A	BRCA	7.72
TCGA-BH-A0EA-01A	BRCA	6.53
TCGA-BH-A0B3-01A	BRCA	5.78
TCGA-5L-AAT1-01A	BRCA	5.64
TCGA-E2-A15J-01A	BRCA	5.61
TCGA-B6-A0WS-01A	BRCA	6.34
TCGA-BH-A0H7-01A	BRCA	7.32
TCGA-A2-A0YT-01A	BRCA	6.41
TCGA-AN-A0XO-01A	BRCA	6.25
TCGA-E2-A15K-06A	BRCA	6.67
TCGA-EW-A1P3-01A	BRCA	4.99
TCGA-A2-A04N-01A	BRCA	5.84
TCGA-D8-A13Y-01A	BRCA	5.56
TCGA-D8-A146-01A	BRCA	7.01
TCGA-LL-A7SZ-01A	BRCA	4.39
TCGA-AO-A0J8-01A	BRCA	5.87
TCGA-B6-A0IK-01A	BRCA	7.92
TCGA-A2-A0T3-01A	BRCA	6.12
TCGA-AO-A03M-01B	BRCA	6.24
TCGA-BH-A0W4-01A	BRCA	6.65
TCGA-E2-A15S-01A	BRCA	5.82
TCGA-D8-A27R-01A	BRCA	6.27
TCGA-B6-A0WZ-01A	BRCA	6.92
TCGA-D8-A27H-01A	BRCA	5.92
TCGA-AO-A03U-01B	BRCA	6.08
TCGA-A7-A5ZV-01A	BRCA	6.82
TCGA-BH-A1EY-01A	BRCA	7.56
TCGA-A8-A09T-01A	BRCA	5.58
TCGA-AC-A4ZE-01A	BRCA	6.03
TCGA-BH-A0H9-01A	BRCA	8.47
TCGA-LD-A7W6-01A	BRCA	6.46
TCGA-GM-A2D9-01A	BRCA	6.09
TCGA-BH-A42T-01A	BRCA	5.96
TCGA-A2-A0SX-01A	BRCA	5.85
TCGA-A1-A0SI-01A	BRCA	7.39
TCGA-A7-A0D9-01A	BRCA	5.66
TCGA-A2-A04U-01A	BRCA	5.15
TCGA-D8-A143-01A	BRCA	6.46
TCGA-BH-A8FZ-01A	BRCA	7.45
TCGA-A2-A0T6-01A	BRCA	5.58
TCGA-AC-A3BB-01A	BRCA	5.85
TCGA-BH-AB28-01A	BRCA	5.6
TCGA-A1-A0SQ-01A	BRCA	6.56
TCGA-E2-A15E-01A	BRCA	6.79
TCGA-AR-A24S-01A	BRCA	6.71
TCGA-D8-A1JS-01A	BRCA	6.67
TCGA-AO-A03O-01A	BRCA	6.15
TCGA-D8-A27F-01A	BRCA	5.27
TCGA-AR-A5QN-01A	BRCA	7.12

TCGA-B6-A0IA-01A	BRCA	6.1
TCGA-B6-A0I6-01A	BRCA	5.04
TCGA-B6-A0RE-01A	BRCA	6.66
TCGA-A8-A08F-01A	BRCA	5.75
TCGA-AR-A2LM-01A	BRCA	6.34
TCGA-AC-A62Y-01A	BRCA	6.56
TCGA-AO-A0JD-01A	BRCA	6.66
TCGA-A2-A0ST-01A	BRCA	5.58
TCGA-A2-A4RW-01A	BRCA	6.73
TCGA-B6-A0RM-01A	BRCA	5.15
TCGA-A1-A0SN-01A	BRCA	6.82
TCGA-LL-A6FR-01A	BRCA	4.07
TCGA-D8-A1JE-01A	BRCA	6.44
TCGA-A8-A09I-01A	BRCA	7.51
TCGA-BH-A0DP-01A	BRCA	6
TCGA-HN-A2NL-01A	BRCA	4.88
TCGA-AR-A24O-01A	BRCA	6.13
TCGA-BH-A1FL-01A	BRCA	5.55
TCGA-V7-A7HQ-01A	BRCA	4.42
TCGA-B6-A1KC-01B	BRCA	4.41
TCGA-A7-A3IZ-01A	BRCA	5.64
TCGA-B6-A1KI-01A	BRCA	6.69
TCGA-BH-A1FH-01A	BRCA	6.04
TCGA-B6-A0X1-01A	BRCA	6.1
TCGA-B6-A0IN-01A	BRCA	5.13
TCGA-E2-A15K-01A	BRCA	6.46
TCGA-AR-A1AL-01A	BRCA	5.81
TCGA-E2-A570-01A	BRCA	5.23
TCGA-C8-A275-01A	BRCA	7.05
TCGA-E2-A1LA-01A	BRCA	5.4
TCGA-E9-A229-01A	BRCA	6.69
TCGA-D8-A1Y0-01A	BRCA	6.1
TCGA-E9-A22D-01A	BRCA	5.33
TCGA-AN-A0XP-01A	BRCA	6.83
TCGA-A8-A07O-01A	BRCA	6.33
TCGA-S3-AA0Z-01A	BRCA	6.77
TCGA-AO-A0J2-01A	BRCA	7.8
TCGA-EW-A2FS-01A	BRCA	5.79
TCGA-BH-A0B8-01A	BRCA	6.11
TCGA-BH-A0E7-01A	BRCA	6.28
TCGA-BH-A0DO-01B	BRCA	8.52
TCGA-AR-A256-01A	BRCA	6.18
TCGA-A7-A426-01A	BRCA	5.94
TCGA-E9-A243-01A	BRCA	6.1
TCGA-BH-A1FG-01A	BRCA	7.23
TCGA-D8-A27N-01A	BRCA	6.85
TCGA-AN-A0FF-01A	BRCA	6.36
TCGA-AO-A0JL-01A	BRCA	4.78
TCGA-AC-A8OS-01A	BRCA	7.34
TCGA-D8-A1J9-01A	BRCA	7.65
TCGA-AC-A62V-01A	BRCA	5.77
TCGA-A2-A0EY-01A	BRCA	7.73
TCGA-A7-A0CJ-01A	BRCA	5.26
TCGA-GM-A2DF-01A	BRCA	4.22
TCGA-BH-A0BJ-01A	BRCA	6.36
TCGA-B6-A0WT-01A	BRCA	6.39
TCGA-E2-A159-01A	BRCA	6.7
TCGA-A8-A08Z-01A	BRCA	5.96

TCGA-A8-A096-01A	BRCA	6.12
TCGA-E2-A14Z-01A	BRCA	5.39
TCGA-A7-A4SF-01A	BRCA	7.25
TCGA-E2-A1BC-01A	BRCA	7.57
TCGA-A8-A08B-01A	BRCA	6.52
TCGA-E2-A1LH-01A	BRCA	5.83
TCGA-LL-A7T0-01A	BRCA	4.96
TCGA-AC-A5XS-01A	BRCA	7.24
TCGA-B6-A400-01A	BRCA	5.39
TCGA-EW-A3U0-01A	BRCA	5.47
TCGA-AC-A2QJ-01A	BRCA	4.5
TCGA-AR-A0TY-01A	BRCA	6
TCGA-EW-A1PG-01A	BRCA	5.86
TCGA-A7-A3J0-01A	BRCA	5.94
TCGA-E2-A15C-01A	BRCA	5.58
TCGA-BH-A0W5-01A	BRCA	5.46
TCGA-AR-A1AV-01A	BRCA	6.63
TCGA-OL-A6VR-01A	BRCA	5.6
TCGA-BH-A18Q-01A	BRCA	5.27
TCGA-BH-A5J0-01A	BRCA	5.43
TCGA-AR-A24R-01A	BRCA	6.41
TCGA-A8-A08H-01A	BRCA	7
TCGA-BH-A0DD-01A	BRCA	5.71
TCGA-E2-A1B4-01A	BRCA	6.51
TCGA-C8-A274-01A	BRCA	7.08
TCGA-A7-A4SE-01A	BRCA	6.31
TCGA-C8-A26Z-01A	BRCA	6.21
TCGA-E2-A10E-01A	BRCA	7.22
TCGA-BH-A0HU-01A	BRCA	5.06
TCGA-A2-A0SY-01A	BRCA	5.86
TCGA-C8-A8HR-01A	BRCA	5.48
TCGA-AN-A046-01A	BRCA	5.13
TCGA-D8-A1JF-01A	BRCA	8.27
TCGA-A8-A097-01A	BRCA	5.93
TCGA-C8-A12M-01A	BRCA	6.47
TCGA-E9-A1RI-01A	BRCA	6.24
TCGA-GM-A2DC-01A	BRCA	5.84
TCGA-E9-A226-01A	BRCA	7.04
TCGA-AO-A03T-01A	BRCA	4.55
TCGA-D8-A1JB-01A	BRCA	7.13
TCGA-BH-A208-01A	BRCA	6.58
TCGA-A7-A26H-01A	BRCA	6.71
TCGA-D8-A1XG-01A	BRCA	6.49
TCGA-AR-A24L-01A	BRCA	6.51
TCGA-AN-A0FY-01A	BRCA	4.31
TCGA-A2-A3XY-01A	BRCA	5.37
TCGA-AC-A8OQ-01A	BRCA	5.08
TCGA-AC-A23C-01A	BRCA	7.15
TCGA-AR-A0TU-01A	BRCA	6.53
TCGA-A8-A09E-01A	BRCA	6.5
TCGA-BH-A0HI-01A	BRCA	7.1
TCGA-E2-A14T-01A	BRCA	6.76
TCGA-E2-A1B6-01A	BRCA	5.98
TCGA-BH-A0C7-01B	BRCA	6.59
TCGA-AN-A0FW-01A	BRCA	5.81
TCGA-LL-A8F5-01A	BRCA	4.27
TCGA-AO-A0JI-01A	BRCA	5.59
TCGA-E9-A3Q9-01A	BRCA	5.36

TCGA-AC-A3HN-01A	BRCA	6.54
TCGA-E9-A5FL-01A	BRCA	5.73
TCGA-A2-A1G4-01A	BRCA	6.95
TCGA-AR-A0U0-01A	BRCA	6.65
TCGA-AR-A2LE-01A	BRCA	9.44
TCGA-E2-A14P-01A	BRCA	7.84
TCGA-AN-A04A-01A	BRCA	5.29
TCGA-BH-A1F5-01A	BRCA	6.15
TCGA-BH-A0HA-01A	BRCA	5.86
TCGA-E2-A15T-01A	BRCA	5.18
TCGA-BH-A0BF-01A	BRCA	5.97
TCGA-EW-A2FW-01A	BRCA	4.64
TCGA-E9-A1NC-01A	BRCA	4.75
TCGA-S3-AA15-01A	BRCA	5.47
TCGA-BH-A202-01A	BRCA	7.5
TCGA-AC-A23E-01A	BRCA	6.34
TCGA-WT-AB41-01A	BRCA	4.67
TCGA-BH-A0RX-01A	BRCA	5.75
TCGA-EW-A1IX-01A	BRCA	6.26
TCGA-A8-A07L-01A	BRCA	7.11
TCGA-A7-A6VY-01A	BRCA	6.05
TCGA-AR-A255-01A	BRCA	7.19
TCGA-OL-A66N-01A	BRCA	6.12
TCGA-A8-A099-01A	BRCA	7.25
TCGA-LL-A5YM-01A	BRCA	4.72
TCGA-AO-A0J6-01A	BRCA	6.08
TCGA-LL-A73Y-01A	BRCA	5.88
TCGA-D8-A73W-01A	BRCA	5.21
TCGA-E2-A14W-01A	BRCA	5.74
TCGA-A2-A25B-01A	BRCA	6.35
TCGA-E2-A15O-01A	BRCA	4.84
TCGA-A2-A0CM-01A	BRCA	6.39
TCGA-D8-A1X5-01A	BRCA	7.36
TCGA-AN-A0AR-01A	BRCA	6.57
TCGA-AR-A1AM-01A	BRCA	6.53
TCGA-E2-A1IH-01A	BRCA	5.43
TCGA-A7-A4SC-01A	BRCA	8.37
TCGA-S3-A6ZH-01A	BRCA	6.54
TCGA-BH-A0EB-01A	BRCA	6.56
TCGA-E2-A15F-01A	BRCA	6.18
TCGA-BH-A1FB-01A	BRCA	6.22
TCGA-E9-A5UO-01A	BRCA	5.68
TCGA-EW-A1J1-01A	BRCA	5.49
TCGA-D8-A1XC-01A	BRCA	6.45
TCGA-E2-A156-01A	BRCA	6.13
TCGA-EW-A1P6-01A	BRCA	5.12
TCGA-E2-A1L8-01A	BRCA	5.63
TCGA-E2-A109-01A	BRCA	6.44
TCGA-LL-A5YN-01A	BRCA	6.02
TCGA-E2-A2P5-01A	BRCA	4.59
TCGA-A7-A0DB-01A	BRCA	6.58
TCGA-A7-A0DB-01C	BRCA	6.4
TCGA-E2-A1LG-01A	BRCA	6.41
TCGA-B6-A0WW-01A	BRCA	5.56
TCGA-E2-A576-01A	BRCA	4.2
TCGA-AC-A3OD-01A	BRCA	5.5
TCGA-A2-A04R-01A	BRCA	5.59
TCGA-E9-A1R0-01A	BRCA	5.91

TCGA-B6-A1KF-01A	BRCA	7.64
TCGA-EW-A1PC-01B	BRCA	5.7
TCGA-EW-A2FV-01A	BRCA	6.21
TCGA-AO-A0JF-01A	BRCA	6.82
TCGA-AN-A0XR-01A	BRCA	6.08
TCGA-GM-A5PX-01A	BRCA	5.49
TCGA-B6-A0RH-01A	BRCA	5.16
TCGA-A2-A0CX-01A	BRCA	7.75
TCGA-AR-A0TZ-01A	BRCA	5.25
TCGA-AR-A2LR-01A	BRCA	5.2
TCGA-BH-A0B6-01A	BRCA	5.8
TCGA-A2-A3XZ-01A	BRCA	7.45
TCGA-AR-A0TR-01A	BRCA	6.19
TCGA-E9-A295-01A	BRCA	5.61
TCGA-A2-A04P-01A	BRCA	5.3
TCGA-BH-A0DS-01A	BRCA	5.23
TCGA-E9-A1N5-01A	BRCA	6.03
TCGA-S3-A6ZF-01A	BRCA	5.4
TCGA-A2-A1FV-01A	BRCA	5.78
TCGA-D8-A147-01A	BRCA	6.11
TCGA-BH-A0HP-01A	BRCA	6.95
TCGA-B6-A40C-01A	BRCA	4.91
TCGA-OK-A5Q2-01A	BRCA	6.44
TCGA-BH-A1ES-06A	BRCA	5.35
TCGA-BH-A18P-01A	BRCA	6.88
TCGA-AR-A252-01A	BRCA	6.46
TCGA-C8-A1HK-01A	BRCA	6.87
TCGA-AQ-A54N-01A	BRCA	5.54
TCGA-A2-A1FW-01A	BRCA	6.15
TCGA-3C-AALJ-01A	BRCA	5.63
TCGA-AC-A62X-01A	BRCA	6.1
TCGA-B6-A0X7-01A	BRCA	5.79
TCGA-BH-A0BW-01A	BRCA	7.38
TCGA-D8-A1XT-01A	BRCA	7.78
TCGA-AR-A254-01A	BRCA	6.39
TCGA-D8-A1Y3-01A	BRCA	4.86
TCGA-BH-A0HL-01A	BRCA	5.45
TCGA-E9-A247-01A	BRCA	5.05
TCGA-C8-A27A-01A	BRCA	6.37
TCGA-A7-A26F-01B	BRCA	5.24
TCGA-AO-A0J3-01A	BRCA	5.21
TCGA-A2-A25D-01A	BRCA	5.7
TCGA-AN-A0XW-01A	BRCA	7.23
TCGA-D8-A141-01A	BRCA	6.06
TCGA-AR-A1AN-01A	BRCA	5.31
TCGA-UU-A93S-01A	BRCA	6.78
TCGA-BH-A18S-01A	BRCA	6.69
TCGA-OL-A5S0-01A	BRCA	5.78
TCGA-LL-A442-01A	BRCA	5.48
TCGA-A8-A09W-01A	BRCA	9.27
TCGA-C8-A1HI-01A	BRCA	5.94
TCGA-BH-A0BZ-01A	BRCA	6.71
TCGA-E2-A56Z-01A	BRCA	4.99
TCGA-A2-A259-01A	BRCA	5.87
TCGA-AR-A24X-01A	BRCA	5.83
TCGA-BH-A1FD-01A	BRCA	5.21
TCGA-BH-A0DZ-01A	BRCA	7.18
TCGA-B6-A0RT-01A	BRCA	5.3

TCGA-AR-A2LQ-01A	BRCA	6.98
TCGA-E2-A150-01A	BRCA	7.45
TCGA-E2-A14R-01A	BRCA	5.28
TCGA-A8-A0A6-01A	BRCA	6.27
TCGA-A7-A26F-01A	BRCA	5.58
TCGA-AO-A0JM-01A	BRCA	6.91
TCGA-A8-A09B-01A	BRCA	6.22
TCGA-BH-A0BR-01A	BRCA	7.73
TCGA-A2-A0T7-01A	BRCA	5.84
TCGA-B6-A401-01A	BRCA	5.5
TCGA-BH-A0DH-01A	BRCA	5.75
TCGA-BH-A1EV-01A	BRCA	6.41
TCGA-EW-A6SD-01A	BRCA	7.06
TCGA-AC-A3W5-01A	BRCA	5.9
TCGA-BH-A0BG-01A	BRCA	7.19
TCGA-A2-A0CR-01A	BRCA	4.41
TCGA-3C-AALI-01A	BRCA	6.14
TCGA-B6-A0RL-01A	BRCA	4.47
TCGA-A7-A5ZW-01A	BRCA	6.41
TCGA-E2-A15H-01A	BRCA	5.98
TCGA-AQ-A1H2-01A	BRCA	7.42
TCGA-D8-A1JD-01A	BRCA	6.89
TCGA-BH-A0B2-01A	BRCA	6.01
TCGA-AR-A1AT-01A	BRCA	6.73
TCGA-D8-A1JK-01A	BRCA	8.32
TCGA-C8-A26Y-01A	BRCA	8.86
TCGA-A7-A6VV-01A	BRCA	6.41
TCGA-A2-A3XX-01A	BRCA	5.42
TCGA-GM-A2DH-01A	BRCA	6.17
TCGA-D8-A1X6-01A	BRCA	5.29
TCGA-B6-A0I1-01A	BRCA	5.66
TCGA-A8-A09M-01A	BRCA	6.07
TCGA-EW-A1IY-01A	BRCA	7.13
TCGA-A8-A07F-01A	BRCA	6.08
TCGA-BH-A8FY-01A	BRCA	6.7
TCGA-5T-A9QA-01A	BRCA	6.75
TCGA-A2-A0EM-01A	BRCA	6.81
TCGA-AO-A12D-01A	BRCA	6.39
TCGA-GI-A2C8-01A	BRCA	5.88
TCGA-OL-A6VQ-01A	BRCA	6.49
TCGA-D8-A1X9-01A	BRCA	5.99
TCGA-A1-A0SB-01A	BRCA	5.61
TCGA-BH-A28Q-01A	BRCA	6.21
TCGA-A2-A04W-01A	BRCA	6.92
TCGA-B6-A0I2-01A	BRCA	6.22
TCGA-LL-A440-01A	BRCA	6
TCGA-A7-A26E-01A	BRCA	7.06
TCGA-EW-A1P8-01A	BRCA	6.68
TCGA-OL-A66I-01A	BRCA	5.32
TCGA-AO-A1KP-01A	BRCA	4.35
TCGA-A8-A092-01A	BRCA	5.69
TCGA-E2-A15L-01A	BRCA	6.27
TCGA-BH-A0HX-01A	BRCA	6
TCGA-E9-A22G-01A	BRCA	5.44
TCGA-E9-A248-01A	BRCA	6.6
TCGA-A8-A07I-01A	BRCA	6.49
TCGA-BH-A42V-01A	BRCA	5.86
TCGA-AN-A0FJ-01A	BRCA	7.23

TCGA-AO-A0JG-01A	BRCA	6.32
TCGA-E2-A108-01A	BRCA	6.44
TCGA-GM-A2DO-01A	BRCA	4.78
TCGA-A8-A082-01A	BRCA	5.66
TCGA-EW-A6SB-01A	BRCA	5.33
TCGA-D8-A27L-01A	BRCA	6.31
TCGA-EW-A1PD-01A	BRCA	5.97
TCGA-B6-A0IM-01A	BRCA	6.17
TCGA-EW-A1P5-01A	BRCA	6.55
TCGA-B6-A0X4-01A	BRCA	5.4
TCGA-EW-A1OY-01A	BRCA	5.31
TCGA-OL-A5DA-01A	BRCA	6.15
TCGA-B6-A0X5-01A	BRCA	6.73
TCGA-EW-A1IW-01A	BRCA	6.93
TCGA-LQ-A4E4-01A	BRCA	6.69
TCGA-A2-A0YM-01A	BRCA	6.24
TCGA-A8-A07U-01A	BRCA	5.82
TCGA-BH-A8G0-01A	BRCA	5.9
TCGA-AR-A0TV-01A	BRCA	5.38
TCGA-E9-A22E-01A	BRCA	6.5
TCGA-C8-A278-01A	BRCA	7.29
TCGA-AR-A0TQ-01A	BRCA	6.34
TCGA-AO-A0JB-01A	BRCA	4.14
TCGA-D8-A1XQ-01A	BRCA	6.58
TCGA-B6-A0I9-01A	BRCA	6.05
TCGA-A2-A0D3-01A	BRCA	6.28
TCGA-A1-A0SF-01A	BRCA	6.15
TCGA-BH-A1FE-06A	BRCA	7.95
TCGA-AN-A04D-01A	BRCA	6.22
TCGA-E9-A22A-01A	BRCA	5.51
TCGA-S3-AA17-01A	BRCA	5.43
TCGA-OL-A5RX-01A	BRCA	5.91
TCGA-BH-A1EO-01A	BRCA	6.1
TCGA-BH-A6R9-01A	BRCA	5.87
TCGA-B6-A0RP-01A	BRCA	6.22
TCGA-A8-A0A1-01A	BRCA	6.92
TCGA-B6-A0IE-01A	BRCA	5.42
TCGA-C8-A12L-01A	BRCA	8.06
TCGA-E9-A1RH-01A	BRCA	8
TCGA-AN-A0XL-01A	BRCA	7.3
TCGA-AC-A2FF-01A	BRCA	6.15
TCGA-A2-A4S3-01A	BRCA	6.84
TCGA-BH-A1FE-01A	BRCA	6.62
TCGA-E9-A1NE-01A	BRCA	6.8
TCGA-E2-A10A-01A	BRCA	5.92
TCGA-C8-A12Z-01A	BRCA	8.05
TCGA-A2-A4RY-01A	BRCA	5.8
TCGA-LL-A6FP-01A	BRCA	7.21
TCGA-AR-A24K-01A	BRCA	4.64
TCGA-E2-A15M-01A	BRCA	6.85
TCGA-A2-A0CK-01A	BRCA	5.19
TCGA-HN-A2OB-01A	BRCA	6.67
TCGA-WT-AB44-01A	BRCA	4.67
TCGA-E2-A573-01A	BRCA	5.97
TCGA-E2-A1L9-01A	BRCA	6.56
TCGA-A7-A26G-01A	BRCA	6.84
TCGA-D8-A1J8-01A	BRCA	7.34
TCGA-A2-A0CL-01A	BRCA	6.39

TCGA-A2-A04V-01A	BRCA	4.44
TCGA-EW-A1J5-01A	BRCA	6.62
TCGA-BH-A0GZ-01A	BRCA	4.96
TCGA-E2-A14U-01A	BRCA	6.85
TCGA-B6-A0IJ-01A	BRCA	6.09
TCGA-B6-A1KN-01A	BRCA	6.81
TCGA-E2-A15G-01A	BRCA	5.73
TCGA-A8-A08S-01A	BRCA	5.7
TCGA-A8-A09X-01A	BRCA	6.3
TCGA-AO-A03R-01A	BRCA	5.19
TCGA-E2-A1IE-01A	BRCA	6.99
TCGA-A2-A0EQ-01A	BRCA	5.68
TCGA-E9-A1R5-01A	BRCA	6.39
TCGA-S3-AA10-01A	BRCA	5.29
TCGA-LL-A5YL-01A	BRCA	6.08
TCGA-BH-A0E0-01A	BRCA	8.06
TCGA-B6-A0I5-01A	BRCA	4.52
TCGA-BH-A18U-01A	BRCA	6.2
TCGA-A8-A06Z-01A	BRCA	6.26
TCGA-D8-A1JN-01A	BRCA	7.62
TCGA-A7-A13H-01A	BRCA	7.62
TCGA-C8-A12Y-01A	BRCA	5.86
TCGA-BH-A0BS-01A	BRCA	7.86
TCGA-A7-A13F-01A	BRCA	6.65
TCGA-AC-A7VC-01A	BRCA	4.24
TCGA-AO-A03P-01A	BRCA	5.04
TCGA-BH-A1F0-01A	BRCA	7.28
TCGA-AR-A1AJ-01A	BRCA	7.1
TCGA-BH-A0EI-01A	BRCA	5.83
TCGA-AC-A3W7-01A	BRCA	6.68
TCGA-E2-A1LL-01A	BRCA	7.83
TCGA-E9-A1R7-01A	BRCA	9.3
TCGA-A8-A0A4-01A	BRCA	5.99
TCGA-E2-A1L6-01A	BRCA	7.05
TCGA-AO-A12E-01A	BRCA	6.08
TCGA-A2-A3KC-01A	BRCA	6.84
TCGA-BH-A18T-01A	BRCA	6.61
TCGA-AN-A0FK-01A	BRCA	5.95
TCGA-B6-A0IG-01A	BRCA	6.69
TCGA-A8-A08T-01A	BRCA	5.32
TCGA-D8-A1XU-01A	BRCA	7.93
TCGA-E2-A3DX-01A	BRCA	5.58
TCGA-A2-A0YD-01A	BRCA	7.14
TCGA-BH-A42U-01A	BRCA	6.06
TCGA-A8-A07W-01A	BRCA	6.36
TCGA-A8-A06N-01A	BRCA	5.52
TCGA-A8-A079-01A	BRCA	7.21
TCGA-AN-A0XT-01A	BRCA	6.35
TCGA-AR-A5QP-01A	BRCA	6.32
TCGA-BH-A0W3-01A	BRCA	6.23
TCGA-AN-A0AM-01A	BRCA	7.41
TCGA-A8-A0A2-01A	BRCA	5.4
TCGA-A8-A09N-01A	BRCA	6.01
TCGA-A8-A07J-01A	BRCA	6.69
TCGA-E2-A15R-01A	BRCA	5.91
TCGA-AR-A1AU-01A	BRCA	6.07
TCGA-A8-A09D-01A	BRCA	6.32
TCGA-AN-A041-01A	BRCA	7.58

TCGA-A8-A084-01A	BRCA	8.24
TCGA-BH-A201-01A	BRCA	6.57
TCGA-A8-A085-01A	BRCA	6.08
TCGA-LD-A66U-01A	BRCA	5.19
TCGA-AR-A5QQ-01A	BRCA	6.17
TCGA-A7-A0DA-01A	BRCA	5.88
TCGA-AR-A2LH-01A	BRCA	6.45
TCGA-E9-A1RE-01A	BRCA	5.41
TCGA-AR-A24U-01A	BRCA	7.23
TCGA-D8-A27T-01A	BRCA	5.49
TCGA-B6-A0IH-01A	BRCA	6.16
TCGA-A2-A0YH-01A	BRCA	5.65
TCGA-BH-A0DI-01A	BRCA	6.11
TCGA-E2-A1LI-01A	BRCA	5.18
TCGA-A2-A0CW-01A	BRCA	6.8
TCGA-E2-A14V-01A	BRCA	6.82
TCGA-AQ-A54O-01A	BRCA	5.2
TCGA-C8-A135-01A	BRCA	7.03
TCGA-GM-A2DK-01A	BRCA	6.2
TCGA-BH-A0AU-01A	BRCA	6.13
TCGA-AO-A0JE-01A	BRCA	6.85
TCGA-A2-A0D0-01A	BRCA	5.54
TCGA-LL-A73Z-01A	BRCA	6.24
TCGA-A8-A09G-01A	BRCA	7.66
TCGA-AN-A0AL-01A	BRCA	8.35
TCGA-A2-A0CT-01A	BRCA	6.37
TCGA-AN-A049-01A	BRCA	6.72
TCGA-LD-A74U-01A	BRCA	6.9
TCGA-D8-A1Y1-01A	BRCA	5.39
TCGA-A2-A0T5-01A	BRCA	5.58
TCGA-D8-A1JM-01A	BRCA	7.98
TCGA-OL-A6VO-01A	BRCA	6.39
TCGA-AC-A2FG-01A	BRCA	7.2
TCGA-E9-A22H-01A	BRCA	6.63
TCGA-B6-A0X0-01A	BRCA	6.16
TCGA-D8-A73X-01A	BRCA	6
TCGA-AQ-A7U7-01A	BRCA	5.42
TCGA-A2-A3Y0-01A	BRCA	7.2
TCGA-E2-A1LK-01A	BRCA	4.45
TCGA-BH-A0B7-01A	BRCA	7.61
TCGA-AR-A2LK-01A	BRCA	5.66
TCGA-AO-A1KT-01A	BRCA	6.27
TCGA-BH-A0HF-01A	BRCA	7.22
TCGA-A8-A09R-01A	BRCA	6.54
TCGA-AR-A1AR-01A	BRCA	7.27
TCGA-A7-A0DC-01A	BRCA	4.42
TCGA-A7-A0DC-01B	BRCA	3.64
TCGA-BH-A1F8-01A	BRCA	7.24
TCGA-A7-A56D-01A	BRCA	4.89
TCGA-E9-A1ND-01A	BRCA	6.55
TCGA-C8-A27B-01A	BRCA	5.48
TCGA-C8-A134-01A	BRCA	6.18
TCGA-E2-A107-01A	BRCA	6.2
TCGA-BH-A0BP-01A	BRCA	7.54
TCGA-A8-A091-01A	BRCA	6.1
TCGA-AC-A3OD-01B	BRCA	4.97
TCGA-EW-A1PE-01A	BRCA	7.61
TCGA-BH-A0DK-01A	BRCA	5.41

TCGA-A8-A06R-01A	BRCA	6.9
TCGA-E9-A3QA-01A	BRCA	5.78
TCGA-EW-A1P1-01A	BRCA	6.59
TCGA-E2-A1IN-01A	BRCA	5.01
TCGA-BH-A0HQ-01A	BRCA	5.83
TCGA-B6-A0WY-01A	BRCA	6.53
TCGA-A8-A090-01A	BRCA	6.46
TCGA-A8-A08X-01A	BRCA	5.66
TCGA-3C-AALK-01A	BRCA	6.73
TCGA-EW-A1OW-01A	BRCA	5.88
TCGA-A2-A0CS-01A	BRCA	6.13
TCGA-BH-A18M-01A	BRCA	6.41
TCGA-E2-A1LB-01A	BRCA	9.47
TCGA-PL-A8LX-01A	BRCA	5.02
TCGA-E2-A2P6-01A	BRCA	8.16
TCGA-BH-A18R-01A	BRCA	6.08
TCGA-BH-A0B0-01A	BRCA	6.76
TCGA-GM-A2DI-01A	BRCA	6.91
TCGA-GM-A5PV-01A	BRCA	7.17
TCGA-A7-A0CG-01A	BRCA	8.18
TCGA-AC-A2QH-01B	BRCA	3.41
TCGA-GM-A4E0-01A	BRCA	5.2
TCGA-AC-A2QH-01A	BRCA	3.72
TCGA-GM-A3XL-01A	BRCA	6.83
TCGA-D8-A1XO-01A	BRCA	6.37
TCGA-BH-A1F2-01A	BRCA	8.42
TCGA-A7-A26J-01A	BRCA	6.56
TCGA-AR-A24H-01A	BRCA	6.2
TCGA-C8-A8HQ-01A	BRCA	6.45
TCGA-B6-A0RU-01A	BRCA	6.18
TCGA-OL-A5D8-01A	BRCA	5.63
TCGA-OL-A97C-01A	BRCA	6.07
TCGA-GM-A3XG-01A	BRCA	6.38
TCGA-AR-A2LJ-01A	BRCA	5.8
TCGA-GM-A3NW-01A	BRCA	6.1
TCGA-A2-A0EV-01A	BRCA	6.17
TCGA-C8-A26X-01A	BRCA	7.47
TCGA-D8-A1XF-01A	BRCA	6.74
TCGA-A2-A0YI-01A	BRCA	7.43
TCGA-D8-A27G-01A	BRCA	7.49
TCGA-OL-A66H-01A	BRCA	5.67
TCGA-B6-A0WV-01A	BRCA	7.31
TCGA-BH-A0DE-01A	BRCA	6.71
TCGA-E2-A1L7-01A	BRCA	5.75
TCGA-D8-A1JG-01B	BRCA	7.54
TCGA-A8-A075-01A	BRCA	5.83
TCGA-JL-A3YX-01A	BRCA	5.09
TCGA-A2-A4S1-01A	BRCA	6.01
TCGA-C8-A12V-01A	BRCA	6.78
TCGA-D8-A4Z1-01A	BRCA	6.05
TCGA-EW-A1P0-01A	BRCA	5.72
TCGA-BH-A0WA-01A	BRCA	5.91
TCGA-AQ-A1H3-01A	BRCA	5.92
TCGA-A7-A13E-01A	BRCA	5.95
TCGA-BH-A0HO-01A	BRCA	6.01
TCGA-D8-A1XW-01A	BRCA	5.95
TCGA-A8-A07P-01A	BRCA	7.01
TCGA-E2-A10F-01A	BRCA	6.03

TCGA-C8-A12O-01A	BRCA	6.49
TCGA-C8-A132-01A	BRCA	6.94
TCGA-E2-A1B1-01A	BRCA	6.83
TCGA-E9-A1RG-01A	BRCA	6.25
TCGA-E2-A1AZ-01A	BRCA	6
TCGA-A7-A3J1-01A	BRCA	6.01
TCGA-AR-A0TW-01A	BRCA	7.05
TCGA-C8-A8HP-01A	BRCA	7.23
TCGA-MS-A51U-01A	BRCA	6.53
TCGA-LL-A6FQ-01A	BRCA	5.45
TCGA-A8-A094-01A	BRCA	7.27
TCGA-AR-A1AI-01A	BRCA	6.09
TCGA-C8-A3M7-01A	BRCA	6.63
TCGA-AN-A0AJ-01A	BRCA	6.44
TCGA-BH-A1EW-01A	BRCA	6.92
TCGA-AR-A24T-01A	BRCA	6.25
TCGA-AR-A1AW-01A	BRCA	5.04
TCGA-GI-A2C9-01A	BRCA	6.65
TCGA-A7-A0CE-01A	BRCA	5.33
TCGA-A2-A0YC-01A	BRCA	6.58
TCGA-AN-A0XU-01A	BRCA	7.06
TCGA-AR-A24Z-01A	BRCA	7.25
TCGA-AR-A24M-01A	BRCA	7.02
TCGA-C8-A1HL-01A	BRCA	6.84
TCGA-EW-A1OV-01A	BRCA	5.4
TCGA-A1-A0SH-01A	BRCA	6.53
TCGA-A2-A1FX-01A	BRCA	6.11
TCGA-D8-A1JL-01A	BRCA	6.69
TCGA-AC-A3YI-01A	BRCA	5.01
TCGA-AO-A03V-01A	BRCA	6.31
TCGA-AR-A1AH-01A	BRCA	6.76
TCGA-A2-A25A-01A	BRCA	5.73
TCGA-BH-A0DQ-01A	BRCA	6.09
TCGA-BH-A1F6-01A	BRCA	5.96
TCGA-EW-A6SA-01A	BRCA	7.05
TCGA-A7-A4SB-01A	BRCA	4.56
TCGA-E9-A1N8-01A	BRCA	5.43
TCGA-Z7-A8R6-01A	BRCA	6.16
TCGA-A8-A09A-01A	BRCA	7.27
TCGA-A7-A5ZX-01A	BRCA	6.92
TCGA-C8-A12N-01A	BRCA	6.2
TCGA-PE-A5DD-01A	BRCA	6.63
TCGA-BH-A2L8-01A	BRCA	6.18
TCGA-AC-A2FM-01A	BRCA	5.85
TCGA-A1-A0SE-01A	BRCA	6.87
TCGA-AC-A8OP-01A	BRCA	7.61
TCGA-GM-A3NY-01A	BRCA	6.58
TCGA-E2-A153-01A	BRCA	5.53
TCGA-A2-A3XW-01A	BRCA	4.98
TCGA-E9-A1RF-01A	BRCA	6.4
TCGA-AC-A2QI-01A	BRCA	6.47
TCGA-BH-A0BM-01A	BRCA	6.56
TCGA-AO-A129-01A	BRCA	6.19
TCGA-XX-A89A-01A	BRCA	5.59
TCGA-A2-A0YG-01A	BRCA	7.42
TCGA-EW-A3E8-01B	BRCA	5.93
TCGA-4H-AAAK-01A	BRCA	5.44
TCGA-A1-A0SO-01A	BRCA	6.69

TCGA-BH-A18N-01A	BRCA	5.71
TCGA-A8-A06P-01A	BRCA	5.83
TCGA-EW-A1OX-01A	BRCA	6.73
TCGA-A2-A0EP-01A	BRCA	5.9
TCGA-AR-A24W-01A	BRCA	6.22
TCGA-E2-A15I-01A	BRCA	6.95
TCGA-D8-A27E-01A	BRCA	7.41
TCGA-A8-A08J-01A	BRCA	5.37
TCGA-C8-A12X-01A	BRCA	6.21
TCGA-AO-A12C-01A	BRCA	5.31
TCGA-BH-A0H3-01A	BRCA	6.06
TCGA-LL-A441-01A	BRCA	6.32
TCGA-3C-AAAU-01A	BRCA	5.67
TCGA-E2-A1IO-01A	BRCA	7.25
TCGA-BH-A18V-06A	BRCA	6.34
TCGA-E2-A15A-01A	BRCA	7.34
TCGA-A7-A3RF-01A	BRCA	5.99
TCGA-GM-A2DM-01A	BRCA	5.37
TCGA-AC-A6IX-01A	BRCA	6.59
TCGA-D8-A3Z5-01A	BRCA	8.21
TCGA-AC-A2FK-01A	BRCA	6.67
TCGA-AR-A1AS-01A	BRCA	7.07
TCGA-AQ-A04L-01B	BRCA	7.25
TCGA-AC-A3TN-01A	BRCA	6.32
TCGA-AC-A23H-01A	BRCA	7.14
TCGA-A8-A08A-01A	BRCA	5.89
TCGA-A2-A0YE-01A	BRCA	7.92
TCGA-A2-A1FZ-01A	BRCA	7.22
TCGA-BH-A28O-01A	BRCA	6.83
TCGA-E9-A3HO-01A	BRCA	4.83
TCGA-E9-A245-01A	BRCA	5.77
TCGA-BH-A1EX-01A	BRCA	6.5
TCGA-D8-A1XV-01A	BRCA	5.47
TCGA-AO-A125-01A	BRCA	6.24
TCGA-AC-A6IW-01A	BRCA	5.32
TCGA-A7-A13E-01B	BRCA	5.58
TCGA-OL-A5RY-01A	BRCA	6.79
TCGA-AC-A3YJ-01A	BRCA	5.36
TCGA-E2-A1LS-01A	BRCA	5.87
TCGA-A2-A0EW-01A	BRCA	6.34
TCGA-C8-A1HM-01A	BRCA	7.11
TCGA-AR-A1AK-01A	BRCA	6.29
TCGA-E9-A1N6-01A	BRCA	7.26
TCGA-B6-A0RS-01A	BRCA	7.48
TCGA-BH-A0DV-01A	BRCA	6.33
TCGA-BH-A1EU-01A	BRCA	7.13
TCGA-BH-A18I-01A	BRCA	5.46
TCGA-E9-A24A-01A	BRCA	6.56
TCGA-E2-A1B0-01A	BRCA	6.97
TCGA-A2-A4S2-01A	BRCA	5.59
TCGA-A1-A0SJ-01A	BRCA	6.91
TCGA-JL-A3YW-01A	BRCA	6.87
TCGA-EW-A1J2-01A	BRCA	6.88
TCGA-OL-A66L-01A	BRCA	4.79
TCGA-A2-A0T4-01A	BRCA	6.39
TCGA-C8-A12P-01A	BRCA	7.45
TCGA-AN-A03Y-01A	BRCA	4.33
TCGA-C8-A1HO-01A	BRCA	6.36

TCGA-A1-A0SP-01A	BRCA	5.6
TCGA-B6-A0IC-01A	BRCA	7.1
TCGA-E9-A2JT-01A	BRCA	6
TCGA-A2-A0YL-01A	BRCA	5.81
TCGA-E9-A1NH-01A	BRCA	6.78
TCGA-A8-A07C-01A	BRCA	7.09
TCGA-C8-A138-01A	BRCA	5.53
TCGA-OL-A5D6-01A	BRCA	7.86
TCGA-E9-A1R4-01A	BRCA	6.38
TCGA-B6-A0RI-01A	BRCA	5.97
TCGA-A2-A0SU-01A	BRCA	4.87
TCGA-AO-A1KO-01A	BRCA	6.05
TCGA-D8-A27P-01A	BRCA	8.03
TCGA-AO-A0JJ-01A	BRCA	6.71
TCGA-OL-A5D7-01A	BRCA	5.44
TCGA-BH-A1FR-01A	BRCA	6.28
TCGA-A2-A3XT-01A	BRCA	5.62
TCGA-EW-A1IZ-01A	BRCA	5.87
TCGA-AO-A126-01A	BRCA	5.45
TCGA-A8-A08R-01A	BRCA	6.15
TCGA-BH-A0E6-01A	BRCA	5.47
TCGA-A7-A26J-01B	BRCA	5.83
TCGA-A8-A06O-01A	BRCA	5.58
TCGA-A7-A13G-01B	BRCA	5.49
TCGA-B6-A0I8-01A	BRCA	7.43
TCGA-AC-A2BK-01A	BRCA	6.6
TCGA-C8-A12K-01A	BRCA	6.18
TCGA-BH-A1FN-01A	BRCA	5.99
TCGA-A2-A04X-01A	BRCA	6.62
TCGA-A8-A08G-01A	BRCA	6.39
TCGA-A8-A06X-01A	BRCA	7
TCGA-AC-A3QP-01A	BRCA	5.22
TCGA-E2-A15A-06A	BRCA	6.47
TCGA-D8-A1JP-01A	BRCA	5.06
TCGA-C8-A12T-01A	BRCA	6.04
TCGA-AN-A0AS-01A	BRCA	5
TCGA-AC-A6IX-06A	BRCA	6.81
TCGA-A2-A0YJ-01A	BRCA	6.44
TCGA-D8-A27K-01A	BRCA	6.28
TCGA-B6-A40B-01A	BRCA	5.03
TCGA-A8-A08C-01A	BRCA	6.56
TCGA-E9-A1R2-01A	BRCA	6.49
TCGA-BH-A1EN-01A	BRCA	7.5
TCGA-C8-A1HN-01A	BRCA	6.18
TCGA-BH-A0C3-01A	BRCA	6.98
TCGA-BH-A0BV-01A	BRCA	6.15
TCGA-D8-A1X8-01A	BRCA	8.47
TCGA-E9-A227-01A	BRCA	6.42
TCGA-5L-AAT0-01A	BRCA	6.2
TCGA-AC-A8OR-01A	BRCA	5.75
TCGA-A2-A0YK-01A	BRCA	6.99
TCGA-C8-A130-01A	BRCA	6.29
TCGA-BH-A0E2-01A	BRCA	6.14
TCGA-EW-A1OZ-01A	BRCA	5.85
TCGA-A7-A13D-01A	BRCA	5.36
TCGA-C8-A26V-01A	BRCA	6.82
TCGA-BH-A18H-01A	BRCA	5.72
TCGA-OL-A66J-01A	BRCA	6.34

TCGA-AR-A1AP-01A	BRCA	6.15
TCGA-A2-A0ET-01A	BRCA	5.05
TCGA-AO-A0J7-01A	BRCA	5.71
TCGA-LD-A9QF-01A	BRCA	6.47
TCGA-AR-A1AQ-01A	BRCA	6.28
TCGA-B6-A0RG-01A	BRCA	6.54
TCGA-A2-A0EX-01A	BRCA	5.88
TCGA-PL-A8LY-01A	BRCA	6.73
TCGA-A2-A0ES-01A	BRCA	6.96
TCGA-C8-A1HF-01A	BRCA	5.48
TCGA-E9-A5UP-01A	BRCA	5.32
TCGA-AN-A0XV-01A	BRCA	6.54
TCGA-BH-A0HN-01A	BRCA	4.6
TCGA-A2-A0YF-01A	BRCA	8.33
TCGA-BH-A0H6-01A	BRCA	5.69
TCGA-A2-A3XU-01A	BRCA	4.88
TCGA-A2-A0CV-01A	BRCA	5.1
TCGA-A8-A0AD-01A	BRCA	5.86
TCGA-AO-A0J4-01A	BRCA	5.58
TCGA-D8-A27M-01A	BRCA	7.68
TCGA-A2-A04T-01A	BRCA	5.03
TCGA-E9-A1NA-01A	BRCA	6.79
TCGA-EW-A2FR-01A	BRCA	6.36
TCGA-LL-A5YO-01A	BRCA	6.54
TCGA-E9-A22B-01A	BRCA	6.62
TCGA-E2-A1IL-01A	BRCA	7.69
TCGA-A8-A07B-01A	BRCA	6.28
TCGA-A7-A26I-01A	BRCA	6.46
TCGA-BH-A0B1-01A	BRCA	6.1
TCGA-EW-A423-01A	BRCA	6.09
TCGA-A1-A0SD-01A	BRCA	6.46
TCGA-D8-A1XD-01A	BRCA	5.11
TCGA-E2-A1IK-01A	BRCA	6.15
TCGA-AN-A0FN-01A	BRCA	5.72
TCGA-E2-A15D-01A	BRCA	5.96
TCGA-BH-A0AW-01A	BRCA	6.36
TCGA-AR-A0U3-01A	BRCA	7.04
TCGA-D8-A1X7-01A	BRCA	7.42
TCGA-E2-A14X-01A	BRCA	4.79
TCGA-LL-A9Q3-01A	BRCA	6.28
TCGA-GM-A2DB-01A	BRCA	6.78
TCGA-AR-A0U4-01A	BRCA	6.1
TCGA-D8-A1XA-01A	BRCA	5.3
TCGA-E2-A1B5-01A	BRCA	5.53
TCGA-A8-A06U-01A	BRCA	6.41
TCGA-A8-A07R-01A	BRCA	6.12
TCGA-A2-A0ER-01A	BRCA	5.82
TCGA-E2-A1LE-01A	BRCA	6.76
TCGA-A1-A0SM-01A	BRCA	6.87
TCGA-A2-A1G1-01A	BRCA	6.35
TCGA-AQ-A04H-01B	BRCA	7.42
TCGA-E9-A6HE-01A	BRCA	5.67
TCGA-AN-A0FZ-01A	BRCA	8.76
TCGA-PE-A5DE-01A	BRCA	6.52
TCGA-EW-A1J6-01A	BRCA	3.69
TCGA-A7-A13D-01B	BRCA	5.42
TCGA-AC-A6NO-01A	BRCA	5.44
TCGA-A7-A2KD-01A	BRCA	6.75

TCGA-A8-A08L-01A	BRCA	7.1
TCGA-A7-A6VW-01A	BRCA	5.88
TCGA-EW-A6SC-01A	BRCA	5.89
TCGA-D8-A1XK-01A	BRCA	5.84
TCGA-BH-A0AY-01A	BRCA	5.44
TCGA-C8-A12U-01A	BRCA	7.31
TCGA-AR-A0U2-01A	BRCA	6.83
TCGA-D8-A1JA-01A	BRCA	7.63
TCGA-A7-A0CH-01A	BRCA	6.93
TCGA-A8-A0A7-01A	BRCA	7.13
TCGA-AR-A250-01A	BRCA	7.52
TCGA-B6-A408-01A	BRCA	4.19
TCGA-E2-A15P-01A	BRCA	8.23
TCGA-AR-A5QM-01A	BRCA	6.26
TCGA-A8-A07Z-01A	BRCA	6.01
TCGA-BH-A0W7-01A	BRCA	6.06
TCGA-A7-A4SA-01A	BRCA	8.58
TCGA-AO-A0J9-01A	BRCA	4.67
TCGA-E9-A244-01A	BRCA	7.11
TCGA-A2-A4S0-01A	BRCA	5.8
TCGA-EW-A424-01A	BRCA	5.04
TCGA-OL-A66O-01A	BRCA	5.69
TCGA-OL-A5RW-01A	BRCA	4.8
TCGA-A2-A0SV-01A	BRCA	5.71
TCGA-E9-A1NF-01A	BRCA	6.71
TCGA-E2-A105-01A	BRCA	6.54
TCGA-D8-A1JU-01A	BRCA	6.77
TCGA-A7-A425-01A	BRCA	5.93
TCGA-GM-A2DD-01A	BRCA	6.52
TCGA-BH-A0BD-01A	BRCA	6.53
TCGA-BH-A0BQ-01A	BRCA	6.07
TCGA-E9-A228-01A	BRCA	5.57
TCGA-D8-A27W-01A	BRCA	6.3
TCGA-E2-A1IG-01A	BRCA	6.1
TCGA-D8-A1JI-01A	BRCA	7.29
TCGA-AN-A0FT-01A	BRCA	5.45
TCGA-A2-A3KD-01A	BRCA	6.22
TCGA-C8-A3M8-01A	BRCA	6.92
TCGA-A2-A0EO-01A	BRCA	5.35
TCGA-BH-A0B4-01A	BRCA	5.93
TCGA-E2-A14S-01A	BRCA	6.69
TCGA-E9-A1NG-01A	BRCA	6.32
TCGA-AC-A3TM-01A	BRCA	5.07
TCGA-AN-A04C-01A	BRCA	5.83
TCGA-BH-A0E1-01A	BRCA	5.87
TCGA-C8-A12W-01A	BRCA	6.32
TCGA-A8-A09K-01A	BRCA	6.33
TCGA-E9-A1N4-01A	BRCA	6.24
TCGA-BH-A18V-01A	BRCA	6.85
TCGA-E2-A9RU-01A	BRCA	4.68
TCGA-A2-A0D4-01A	BRCA	7.35
TCGA-D8-A1XR-01A	BRCA	5.98
TCGA-E2-A14N-01A	BRCA	6.69
TCGA-PE-A5DC-01A	BRCA	5.43
TCGA-AR-A2LL-01A	BRCA	5.96
TCGA-BH-A0E9-01B	BRCA	8.15
TCGA-A2-A0CY-01A	BRCA	3.68
TCGA-BH-A0AV-01A	BRCA	5.38

TCGA-BH-A0GY-01A	BRCA	5.63
TCGA-BH-A0HY-01A	BRCA	7.48
TCGA-A8-A08I-01A	BRCA	6.17
TCGA-A2-A25C-01A	BRCA	6.53
TCGA-OL-A5RZ-01A	BRCA	5.39
TCGA-A8-A0A9-01A	BRCA	6.11
TCGA-A7-A6VX-01A	BRCA	6.07
TCGA-E2-A106-01A	BRCA	6.88
TCGA-AC-A6IV-01A	BRCA	6.07
TCGA-AN-A03X-01A	BRCA	5.83
TCGA-EW-A1PB-01A	BRCA	6.44
TCGA-BH-A6R8-01A	BRCA	6.4
TCGA-PL-A8LZ-01A	BRCA	6.6
TCGA-D8-A1XM-01A	BRCA	6.6
TCGA-BH-A1ET-01A	BRCA	6.58
TCGA-B6-A3ZX-01A	BRCA	5.62
TCGA-W8-A86G-01A	BRCA	6.96
TCGA-AO-A03N-01B	BRCA	6
TCGA-D8-A73U-01A	BRCA	6.23
TCGA-E9-A1RB-01A	BRCA	5.49
TCGA-AR-A0TS-01A	BRCA	5.25
TCGA-AO-A12A-01A	BRCA	5.94
TCGA-AN-A0XS-01A	BRCA	5.74
TCGA-AN-A0G0-01A	BRCA	8.15
TCGA-E2-A10C-01A	BRCA	5.66
TCGA-BH-A0H0-01A	BRCA	7.76
TCGA-AQ-A04J-01A	BRCA	5.78
TCGA-AN-A0AK-01A	BRCA	7.41
TCGA-AR-A2LN-01A	BRCA	6.67
TCGA-A8-A081-01A	BRCA	7.35
TCGA-A2-A04Y-01A	BRCA	5.26
TCGA-BH-A0B5-01A	BRCA	6.41
TCGA-A2-A0CZ-01A	BRCA	4.91
TCGA-B6-A0WX-01A	BRCA	5.86
TCGA-D8-A3Z6-01A	BRCA	6.69
TCGA-A8-A07S-01A	BRCA	7.24
TCGA-E2-A14Y-01A	BRCA	6.87
TCGA-BH-A18L-01A	BRCA	5.84
TCGA-BH-A0BT-01A	BRCA	6.69
TCGA-LD-A7W5-01A	BRCA	5.87
TCGA-A2-A3XS-01A	BRCA	4.93
TCGA-E2-A152-01A	BRCA	6.41
TCGA-BH-A5IZ-01A	BRCA	5.75
TCGA-LL-A740-01A	BRCA	7.89
TCGA-C8-A12Q-01A	BRCA	8.45
TCGA-A2-A0CQ-01A	BRCA	5.05
TCGA-AO-A12H-01A	BRCA	5.39
TCGA-C8-A133-01A	BRCA	5.24
TCGA-A2-A04Q-01A	BRCA	6.15
TCGA-AO-A124-01A	BRCA	5.77
TCGA-AN-A0FL-01A	BRCA	7.25
TCGA-BH-A1FC-01A	BRCA	5.45
TCGA-AN-A0FD-01A	BRCA	5.39
TCGA-C8-A131-01A	BRCA	6.08
TCGA-A2-A25F-01A	BRCA	6.45
TCGA-E9-A1NI-01A	BRCA	5.8
TCGA-GM-A2DA-01A	BRCA	5.22
TCGA-B6-A0RQ-01A	BRCA	6.49

TCGA-BH-A0H5-01A	BRCA	6.14
TCGA-AC-A2B8-01A	BRCA	6.17
TCGA-A7-A13G-01A	BRCA	6.21
TCGA-A7-A3IY-01A	BRCA	6.12
TCGA-LL-A5YP-01A	BRCA	5.94
TCGA-E2-A1IF-01A	BRCA	6.04
TCGA-BH-A18G-01A	BRCA	5.97
TCGA-B6-A402-01A	BRCA	5.17
TCGA-BH-A0DT-01A	BRCA	6.23
TCGA-EW-A1PF-01A	BRCA	6.33
TCGA-A8-A09Z-01A	BRCA	6.74
TCGA-BH-A0C0-01A	BRCA	6.52
TCGA-BH-A1FM-01A	BRCA	5.95
TCGA-A8-A07E-01A	BRCA	6.89
TCGA-A8-A09C-01A	BRCA	5.56
TCGA-AC-A2BM-01A	BRCA	6.02
TCGA-AO-A0JA-01A	BRCA	6.74
TCGA-A2-A0CO-01A	BRCA	6.01
TCGA-A8-A07G-01A	BRCA	6.58
TCGA-LL-A50Y-01A	BRCA	5.84
TCGA-A7-A4SD-01A	BRCA	6.26
TCGA-A2-A0T0-01A	BRCA	6.62
TCGA-S3-AA14-01A	BRCA	7.19
TCGA-AR-A251-01A	BRCA	6.59
TCGA-BH-A0BL-01A	BRCA	6.83
TCGA-AC-A3QQ-01B	BRCA	3.73
TCGA-AC-A5XU-01A	BRCA	5.46
TCGA-A2-A0D2-01A	BRCA	5.68
TCGA-A2-A0SW-01A	BRCA	6.19
TCGA-E9-A3X8-01A	BRCA	3.75
TCGA-B6-A2IU-01A	BRCA	6.19
TCGA-EW-A6S9-01A	BRCA	6.75
TCGA-E9-A1N9-01A	BRCA	5.72
TCGA-D8-A27I-01A	BRCA	6.34
TCGA-E2-A158-01A	BRCA	5.11
TCGA-A1-A0SK-01A	BRCA	4.44
TCGA-AC-A23G-01A	BRCA	5.37
TCGA-C8-A1HJ-01A	BRCA	6.35
TCGA-GM-A2DL-01A	BRCA	5.73
TCGA-AR-A2LO-01A	BRCA	5.4
TCGA-E2-A1IJ-01A	BRCA	5.55
TCGA-AR-A0TP-01A	BRCA	5.54
TCGA-D8-A27V-01A	BRCA	6.88
TCGA-E9-A5FK-01A	BRCA	5.14
TCGA-BH-A203-01A	BRCA	8.02
TCGA-BH-A0DL-01A	BRCA	7.24
TCGA-OL-A5RU-01A	BRCA	8.05
TCGA-A8-A083-01A	BRCA	6.72
TCGA-A2-A25E-01A	BRCA	6.85
TCGA-C8-A1HE-01A	BRCA	7.99
TCGA-A7-A26I-01B	BRCA	5.66
TCGA-AO-A1KS-01A	BRCA	5.38
TCGA-E9-A1QZ-01A	BRCA	6.36
TCGA-AR-A0TX-01A	BRCA	7.24
TCGA-BH-A18F-01A	BRCA	6.28
TCGA-AO-A0J5-01A	BRCA	6.64
TCGA-AO-A1KR-01A	BRCA	5.52
TCGA-EW-A1J3-01A	BRCA	6.9

TCGA-A2-A0EU-01A	BRCA	5.77
TCGA-BH-A0EE-01A	BRCA	6.9
TCGA-BH-A18J-01A	BRCA	6.66
TCGA-D8-A1XY-01A	BRCA	6.19
TCGA-EW-A1P7-01A	BRCA	6.03
TCGA-AO-A128-01A	BRCA	7.1
TCGA-A8-A093-01A	BRCA	5.8
TCGA-B6-A0IQ-01A	BRCA	5.89
TCGA-E2-A155-01A	BRCA	6.76
TCGA-BH-A0DX-01A	BRCA	7.48
TCGA-B6-A0IO-01A	BRCA	6.08
TCGA-C8-A137-01A	BRCA	7.47
TCGA-AC-A2FB-01A	BRCA	6.8
TCGA-A8-A09Q-01A	BRCA	9.68
TCGA-A2-A0D1-01A	BRCA	8.15
TCGA-BH-A0DG-01A	BRCA	6.69
TCGA-GM-A3XN-01A	BRCA	5.11
TCGA-BH-A0HB-01A	BRCA	5.92
TCGA-E2-A572-01A	BRCA	5.2
TCGA-A7-A0CD-01A	BRCA	6.08
TCGA-C8-A1HG-01A	BRCA	6.09
TCGA-A1-A0SG-01A	BRCA	5.84
TCGA-AN-A0FS-01A	BRCA	6.66
TCGA-S3-AA11-01A	BRCA	6.39
TCGA-E2-A1BD-01A	BRCA	5.71
TCGA-E2-A15E-06A	BRCA	5.58
TCGA-B6-A0RO-01A	BRCA	6.73
TCGA-E2-A1II-01A	BRCA	6.42
TCGA-D8-A1XS-01A	BRCA	6.54
TCGA-AN-A0FX-01A	BRCA	5.87
TCGA-D8-A1JC-01A	BRCA	6.96
TCGA-AO-A0JC-01A	BRCA	5.2
TCGA-E2-A574-01A	BRCA	5.52
TCGA-E9-A1N3-01A	BRCA	6.02
TCGA-AN-A0AT-01A	BRCA	6.15
TCGA-BH-A204-01A	BRCA	7.18
TCGA-A2-A0T1-01A	BRCA	6.48
TCGA-VS-A9UL-01A	CESC	6.04
TCGA-VS-A8EI-01A	CESC	4.72
TCGA-JX-A5QV-01A	CESC	5.27
TCGA-EK-A2RA-01A	CESC	5.05
TCGA-VS-A9U5-01A	CESC	5.44
TCGA-C5-A1MI-01A	CESC	6.64
TCGA-ZJ-AAXD-01A	CESC	5.7
TCGA-C5-A8XI-01A	CESC	5.82
TCGA-VS-A8EG-01A	CESC	6.4
TCGA-FU-A57G-01A	CESC	6.77
TCGA-Q1-A73S-01A	CESC	7.1
TCGA-C5-A1M6-01A	CESC	7.74
TCGA-VS-A9UY-01A	CESC	5.32
TCGA-VS-A9V1-01A	CESC	6.43
TCGA-JW-A5VL-01A	CESC	6.89
TCGA-VS-A9UC-01A	CESC	5
TCGA-EK-A2RC-01A	CESC	5.45
TCGA-C5-A7UI-01A	CESC	3.78
TCGA-VS-A9V5-01A	CESC	6.14
TCGA-VS-A9UV-01A	CESC	6.41
TCGA-ZJ-AAXU-01A	CESC	4.86

TCGA-EK-A2R8-01A	CESC	5.59
TCGA-C5-A2LZ-01A	CESC	4.91
TCGA-2W-A8YY-01A	CESC	7.54
TCGA-C5-A7UC-01A	CESC	6.21
TCGA-Q1-A73Q-01A	CESC	6.18
TCGA-Q1-A6DW-01A	CESC	5.48
TCGA-EA-A5ZE-01A	CESC	7.83
TCGA-IR-A3L7-01A	CESC	6.63
TCGA-GH-A9DA-01A	CESC	5.31
TCGA-JW-A5VH-01A	CESC	6.71
TCGA-C5-A3HF-01A	CESC	6.91
TCGA-EA-A3HQ-01A	CESC	6.49
TCGA-Q1-A5R3-01A	CESC	4.67
TCGA-EA-A50E-01A	CESC	5.64
TCGA-EK-A2RL-01A	CESC	7.33
TCGA-JW-A5VI-01A	CESC	5.61
TCGA-DG-A2KH-01A	CESC	6.39
TCGA-IR-A3LL-01A	CESC	5.24
TCGA-EA-A411-01A	CESC	5.58
TCGA-C5-A1BI-01B	CESC	5
TCGA-EK-A2IP-01A	CESC	4.95
TCGA-MA-AA3X-01A	CESC	5.62
TCGA-VS-A9UR-01A	CESC	6.55
TCGA-C5-A7X5-01A	CESC	6.3
TCGA-VS-A8EC-01A	CESC	5.01
TCGA-VS-A954-01A	CESC	4.98
TCGA-VS-A9UI-01A	CESC	6.67
TCGA-FU-A5XV-01A	CESC	6.34
TCGA-MA-AA41-01A	CESC	5.44
TCGA-C5-A1MK-01A	CESC	6.37
TCGA-EK-A2IR-01A	CESC	7.21
TCGA-IR-A3LK-01A	CESC	5.36
TCGA-C5-A907-01A	CESC	6.39
TCGA-C5-A3HE-01A	CESC	6.63
TCGA-VS-A9UT-01A	CESC	6.15
TCGA-EK-A2RO-01A	CESC	5.59
TCGA-C5-A8YR-01A	CESC	6.09
TCGA-EK-A2GZ-01A	CESC	7.24
TCGA-UC-A7PD-01A	CESC	5.21
TCGA-JX-A3Q0-01A	CESC	7.27
TCGA-C5-A0TN-01A	CESC	5.09
TCGA-C5-A7CK-01A	CESC	6.67
TCGA-EA-A5O9-01A	CESC	5.03
TCGA-VS-A8EB-01A	CESC	5.24
TCGA-VS-A94X-01A	CESC	6.52
TCGA-C5-A8ZZ-01A	CESC	6.43
TCGA-VS-A8QM-01A	CESC	5.67
TCGA-JX-A3Q8-01A	CESC	7.83
TCGA-C5-A8XH-01A	CESC	5.8
TCGA-DS-A1OB-01A	CESC	6.76
TCGA-VS-A950-01A	CESC	5.68
TCGA-C5-A1MH-01A	CESC	6.36
TCGA-WL-A834-01A	CESC	5.56
TCGA-C5-A7X3-01A	CESC	5.64
TCGA-C5-A902-01A	CESC	5.36
TCGA-IR-A3LC-01A	CESC	5.6
TCGA-DS-A1OA-01A	CESC	5.35
TCGA-FU-A2QG-01A	CESC	4.91

TCGA-DS-A0VM-01A	CESC	7.32
TCGA-EA-A44S-01A	CESC	4.7
TCGA-VS-A9UM-01A	CESC	5.3
TCGA-IR-A3LH-01A	CESC	5.77
TCGA-EK-A2RB-01A	CESC	4.88
TCGA-VS-A8EK-01A	CESC	5.58
TCGA-EX-A3L1-01A	CESC	5.82
TCGA-C5-A7CO-01A	CESC	5.63
TCGA-C5-A7CJ-01A	CESC	6.47
TCGA-DS-A7WH-01A	CESC	5.54
TCGA-ZJ-A8QR-01A	CESC	6.57
TCGA-EK-A2R9-01A	CESC	4.89
TCGA-VS-AA62-01A	CESC	6.91
TCGA-VS-A8EJ-01A	CESC	7.32
TCGA-EA-A3HT-01A	CESC	6.36
TCGA-BI-A0VS-01A	CESC	5.51
TCGA-BI-A0VR-01A	CESC	5.95
TCGA-VS-A8Q9-01A	CESC	6.17
TCGA-EK-A2PI-01A	CESC	5.65
TCGA-EK-A2RM-01A	CESC	7.08
TCGA-DS-A7WI-01A	CESC	6.14
TCGA-IR-A3LB-01A	CESC	6.46
TCGA-DG-A2KM-01A	CESC	6.04
TCGA-C5-A7CH-01A	CESC	4.87
TCGA-EK-A2R7-01A	CESC	7.51
TCGA-DS-A1OD-01A	CESC	5.09
TCGA-C5-A8XJ-01A	CESC	6.75
TCGA-ZJ-AAXA-01A	CESC	5.51
TCGA-EK-A3GJ-01A	CESC	5.68
TCGA-EA-A5FO-01A	CESC	6.86
TCGA-C5-A7XC-01A	CESC	5.55
TCGA-C5-A1M7-01A	CESC	6.2
TCGA-VS-A8EL-01A	CESC	4.68
TCGA-VS-A9UP-01A	CESC	7.79
TCGA-LP-A4AU-01A	CESC	6.86
TCGA-DS-A1OC-01A	CESC	6.7
TCGA-Q1-A73R-01A	CESC	6.51
TCGA-BI-A20A-01A	CESC	5.76
TCGA-C5-A2LY-01A	CESC	6.27
TCGA-UC-A7PF-01A	CESC	5.66
TCGA-VS-A8QF-01A	CESC	6.19
TCGA-C5-A1MJ-01A	CESC	6.55
TCGA-EA-A5ZD-01A	CESC	6.66
TCGA-EX-A69M-01A	CESC	7.52
TCGA-LP-A5U2-01A	CESC	4.82
TCGA-DR-A0ZL-01A	CESC	5.67
TCGA-VS-A94W-01A	CESC	6.21
TCGA-EA-A439-01A	CESC	7.99
TCGA-C5-A7X8-01A	CESC	5.89
TCGA-EK-A2PL-01A	CESC	5.67
TCGA-MU-A5YI-01A	CESC	6.08
TCGA-EX-A449-01A	CESC	8.22
TCGA-VS-A959-01A	CESC	6.41
TCGA-ZJ-AB0H-01A	CESC	5.79
TCGA-HG-A2PA-01A	CESC	7.1
TCGA-Q1-A5R2-01A	CESC	5.72
TCGA-C5-A1BQ-01C	CESC	7.57
TCGA-C5-A7UE-01A	CESC	4.67

TCGA-MA-AA42-01A	CESC	6.51
TCGA-EK-A3GN-01A	CESC	6.07
TCGA-MA-AA3W-01A	CESC	5.04
TCGA-VS-A953-01A	CESC	5.51
TCGA-MY-A5BE-01A	CESC	5.46
TCGA-DS-A0VN-01A	CESC	4.97
TCGA-XS-A8TJ-01A	CESC	6.05
TCGA-EK-A2PM-01A	CESC	7.69
TCGA-LP-A7HU-01A	CESC	5.66
TCGA-MU-A51Y-01A	CESC	7.16
TCGA-DR-A0ZM-01A	CESC	6.3
TCGA-IR-A3LA-01A	CESC	9.91
TCGA-VS-A9UJ-01A	CESC	6.43
TCGA-FU-A40J-01A	CESC	5.83
TCGA-C5-A1BJ-01A	CESC	6.39
TCGA-HM-A6W2-06A	CESC	7.47
TCGA-C5-A1BK-01B	CESC	4.62
TCGA-ZJ-A8QQ-01A	CESC	5.65
TCGA-EA-A4BA-01A	CESC	6.9
TCGA-VS-A958-01A	CESC	5.72
TCGA-C5-A7UH-01A	CESC	4.94
TCGA-C5-A7CL-01A	CESC	6.28
TCGA-VS-A8QH-01A	CESC	5.96
TCGA-VS-A957-01A	CESC	6.99
TCGA-C5-A1ME-01A	CESC	4.75
TCGA-JW-A5VK-01A	CESC	6.34
TCGA-DS-A7WF-01A	CESC	5.97
TCGA-Q1-A73O-01A	CESC	6
TCGA-RA-A741-01A	CESC	7.1
TCGA-C5-A7CM-01A	CESC	7.09
TCGA-DS-A1O9-01A	CESC	7.58
TCGA-C5-A3HL-01A	CESC	6.61
TCGA-C5-A3HD-01B	CESC	6.08
TCGA-FU-A770-01A	CESC	4.73
TCGA-JW-A5VG-01A	CESC	4.81
TCGA-EA-A5ZF-01A	CESC	9.01
TCGA-LP-A5U3-01A	CESC	6.13
TCGA-ZJ-AAX4-01A	CESC	5.36
TCGA-EX-A1H6-01B	CESC	4.49
TCGA-VS-A94Y-01A	CESC	5.47
TCGA-C5-A2LS-01A	CESC	7.08
TCGA-R2-A69V-01A	CESC	5.49
TCGA-VS-A8QA-01A	CESC	7.14
TCGA-LP-A4AV-01A	CESC	3.45
TCGA-VS-A9UB-01A	CESC	6.54
TCGA-C5-A1M5-01A	CESC	5.9
TCGA-VS-A9V3-01A	CESC	6.42
TCGA-EA-A43B-01A	CESC	5.12
TCGA-EA-A556-01A	CESC	6.98
TCGA-MA-AA3Y-01A	CESC	5.29
TCGA-ZJ-AAXT-01A	CESC	5.49
TCGA-C5-A1BF-01B	CESC	5.32
TCGA-C5-A1MN-01A	CESC	6.84
TCGA-JX-A3PZ-01A	CESC	5.26
TCGA-C5-A1ML-01A	CESC	6.59
TCGA-C5-A2LV-01A	CESC	5.39
TCGA-LP-A4AX-01A	CESC	5.95
TCGA-Q1-A73P-01A	CESC	6.42

TCGA-C5-A1MF-01A	CESC	4.87
TCGA-C5-A1BL-01A	CESC	6.79
TCGA-ZJ-AAXB-01A	CESC	5.47
TCGA-C5-A1BM-01A	CESC	6.36
TCGA-C5-A2M1-01A	CESC	7.23
TCGA-C5-A1BN-01B	CESC	6.28
TCGA-EA-A6QX-01A	CESC	5.69
TCGA-DG-A2KK-01A	CESC	7.51
TCGA-VS-A9U6-01A	CESC	6.16
TCGA-C5-A1M9-01A	CESC	5.72
TCGA-MA-AA3Z-01A	CESC	7.38
TCGA-UC-A7PI-01A	CESC	6.76
TCGA-VS-A8QC-01A	CESC	4.85
TCGA-ZJ-AB0I-01A	CESC	4.45
TCGA-Q1-A6DV-01A	CESC	6.3
TCGA-C5-A2LX-01A	CESC	5.27
TCGA-EK-A2RJ-01A	CESC	4.85
TCGA-EK-A2RK-01A	CESC	6.94
TCGA-EA-A1QT-01A	CESC	5.49
TCGA-ZJ-AAX8-01A	CESC	6.54
TCGA-EA-A3QE-01A	CESC	5.27
TCGA-FU-A3TX-01A	CESC	6.56
TCGA-VS-A9V0-01A	CESC	6
TCGA-EK-A3GM-01A	CESC	6.96
TCGA-FU-A3EO-01A	CESC	4.88
TCGA-C5-A905-01A	CESC	4.98
TCGA-C5-A8YT-01A	CESC	6.96
TCGA-C5-A8YQ-01A	CESC	6.06
TCGA-VS-A8EH-01A	CESC	7.53
TCGA-VS-A9UU-01A	CESC	5.29
TCGA-FU-A3HY-01A	CESC	5.91
TCGA-DS-A0VK-01A	CESC	7.94
TCGA-UC-A7PG-01A	CESC	4.98
TCGA-VS-A9V4-01A	CESC	7.09
TCGA-VS-A9UH-01A	CESC	5.44
TCGA-VS-A8Q8-01A	CESC	5.62
TCGA-VS-A94Z-01A	CESC	6.46
TCGA-VS-A9UD-01A	CESC	6.54
TCGA-EX-A69L-01A	CESC	5.69
TCGA-HM-A3JK-01A	CESC	5.81
TCGA-IR-A3LI-01A	CESC	5.92
TCGA-DS-A3LQ-01A	CESC	7.72
TCGA-DG-A2KJ-01A	CESC	3.36
TCGA-EA-A97N-01A	CESC	5.87
TCGA-ZX-AA5X-01A	CESC	5.31
TCGA-ZJ-AAXJ-01A	CESC	5.8
TCGA-EK-A2PK-01A	CESC	5.81
TCGA-EX-A1H5-01A	CESC	6.8
TCGA-FU-A3HZ-01A	CESC	6.65
TCGA-FU-A3WB-01A	CESC	5.32
TCGA-UC-A7PG-06A	CESC	5.79
TCGA-ZJ-AAXN-01A	CESC	6.09
TCGA-VS-A9U7-01A	CESC	4.7
TCGA-C5-A2M2-01A	CESC	7.35
TCGA-4J-AA1J-01A	CESC	6.13
TCGA-IR-A3LF-01A	CESC	6.33
TCGA-FU-A3YQ-01A	CESC	6.2
TCGA-EA-A1QS-01A	CESC	5.46

TCGA-FU-A23L-01A	CESC	6.63
TCGA-EA-A3HU-01A	CESC	6.59
TCGA-HM-A6W2-01A	CESC	6.29
TCGA-C5-A1MP-01A	CESC	4.73
TCGA-ZJ-AAXF-01A	CESC	5.42
TCGA-HM-A4S6-01A	CESC	6.76
TCGA-VS-A9UZ-01A	CESC	6.99
TCGA-EK-A2H1-01A	CESC	4.66
TCGA-EA-A3HR-01A	CESC	5.79
TCGA-FU-A23K-01A	CESC	5.26
TCGA-EA-A78R-01A	CESC	5.42
TCGA-JW-A852-01A	CESC	5.35
TCGA-JW-AAVH-01A	CESC	5.82
TCGA-C5-A1M8-01A	CESC	4.41
TCGA-VS-A9UQ-01A	CESC	6.21
TCGA-MU-A8JM-01A	CESC	5.01
TCGA-EX-A8YF-01A	CESC	7.06
TCGA-DG-A2KL-01A	CESC	6.18
TCGA-C5-A1BE-01B	CESC	6.74
TCGA-VS-A9UO-01A	CESC	7.62
TCGA-PN-A8MA-01A	CESC	5.93
TCGA-ZJ-AAXI-01A	CESC	5.06
TCGA-VS-A9V2-01A	CESC	6.41
TCGA-MY-A5BF-01A	CESC	5.24
TCGA-Q1-A6DT-01A	CESC	5.12
TCGA-EA-A3Y4-01A	CESC	4.69
TCGA-EK-A2PG-01A	CESC	4.18
TCGA-FU-A3TQ-01A	CESC	6.79
TCGA-EA-A410-01A	CESC	7
TCGA-LP-A4AW-01A	CESC	6.45
TCGA-EA-A3HS-01A	CESC	5.54
TCGA-EK-A2RE-01A	CESC	4.9
TCGA-C5-A8XK-01A	CESC	6.49
TCGA-EA-A3QD-01A	CESC	5.83
TCGA-DS-A5RQ-01A	CESC	6.44
TCGA-VS-A952-01A	CESC	7.11
TCGA-EK-A2RN-01A	CESC	3.47
TCGA-EK-A3GK-01A	CESC	5.15
TCGA-EK-A2H0-01A	CESC	5.89
TCGA-MY-A5BD-01A	CESC	6.07
TCGA-C5-A901-01A	CESC	5.06
TCGA-DS-A0VL-01A	CESC	5.85
TCGA-MA-AA43-01A	CESC	4.33
TCGA-ZJ-A8QO-01A	CESC	5.69
TCGA-JW-A5VJ-01A	CESC	5.75
TCGA-C5-A1MQ-01A	CESC	5.05
TCGA-C5-A7CG-01A	CESC	5.62
TCGA-HM-A3JJ-01A	CESC	6.19
TCGA-MY-A913-01A	CESC	5.2
TCGA-Q1-A5R1-01A	CESC	6.28
TCGA-JW-A69B-01A	CESC	6.97
TCGA-C5-A2LT-01A	CESC	4.52
TCGA-FU-A3NI-01A	CESC	5.59
TCGA-W5-AA33-01A	CHOL	6.89
TCGA-YR-A95A-01A	CHOL	6.21
TCGA-ZH-A8Y6-01A	CHOL	7.48
TCGA-3X-AAVB-01A	CHOL	6.37
TCGA-WD-A7RX-01A	CHOL	6.78

TCGA-W5-AA2Z-01A	CHOL	6.28
TCGA-W5-AA38-01A	CHOL	7.76
TCGA-W5-AA2X-01A	CHOL	7.95
TCGA-W6-AA0S-01A	CHOL	6.17
TCGA-W5-AA2Q-01A	CHOL	7.07
TCGA-W5-AA36-01A	CHOL	6.44
TCGA-W5-AA39-01A	CHOL	6.69
TCGA-W5-AA2I-01A	CHOL	6.41
TCGA-3X-AAV9-01A	CHOL	5.11
TCGA-ZH-A8Y2-01A	CHOL	6.85
TCGA-W5-AA2G-01A	CHOL	7.23
TCGA-ZU-A8S4-01A	CHOL	6.7
TCGA-W5-AA2O-01A	CHOL	6.81
TCGA-W5-AA31-01A	CHOL	7.22
TCGA-3X-AAVC-01A	CHOL	7.6
TCGA-ZH-A8Y1-01A	CHOL	7.51
TCGA-3X-AAVE-01A	CHOL	6.9
TCGA-ZH-A8Y5-01A	CHOL	6.23
TCGA-ZH-A8Y4-01A	CHOL	6.48
TCGA-4G-AAZT-01A	CHOL	5.11
TCGA-W5-AA30-01A	CHOL	6.94
TCGA-W5-AA2T-01A	CHOL	6.1
TCGA-W5-AA34-01A	CHOL	6.21
TCGA-W5-AA2H-01A	CHOL	3.81
TCGA-ZD-A8I3-01A	CHOL	7.07
TCGA-W5-AA2W-01A	CHOL	5.43
TCGA-4G-AAZO-01A	CHOL	5.45
TCGA-ZH-A8Y8-01A	CHOL	6.31
TCGA-3X-AAVA-01A	CHOL	6.3
TCGA-W5-AA2U-01A	CHOL	6.41
TCGA-AA-A00K-01A	COAD	7.38
TCGA-D5-5538-01A	COAD	6.33
TCGA-AA-A00A-01A	COAD	6.69
TCGA-AA-A02K-01A	COAD	7.88
TCGA-D5-6922-01A	COAD	7.51
TCGA-AD-5900-01A	COAD	7.24
TCGA-AA-3495-01A	COAD	7.11
TCGA-AD-6888-01A	COAD	8.11
TCGA-CA-5256-01A	COAD	7.51
TCGA-G4-6299-01A	COAD	7.12
TCGA-AZ-4313-01A	COAD	6.56
TCGA-AA-3872-01A	COAD	7.59
TCGA-F4-6854-01A	COAD	7.09
TCGA-AA-3977-01A	COAD	7.7
TCGA-CA-5254-01A	COAD	7.59
TCGA-CK-5916-01A	COAD	7.84
TCGA-AA-A03F-01A	COAD	5.42
TCGA-AA-A01X-01A	COAD	7.01
TCGA-G4-6304-01A	COAD	7.23
TCGA-AA-3866-01A	COAD	7.28
TCGA-AY-4071-01A	COAD	5.94
TCGA-AA-3655-01A	COAD	7.43
TCGA-AA-A02E-01A	COAD	7.93
TCGA-AA-3510-01A	COAD	6.81
TCGA-AA-3561-01A	COAD	7.23
TCGA-D5-6930-01A	COAD	7.07
TCGA-AU-6004-01A	COAD	6.97
TCGA-F4-6461-01A	COAD	6.56

TCGA-AA-3675-01A	COAD	7.7
TCGA-D5-6926-01A	COAD	6.94
TCGA-A6-2684-01A	COAD	6.72
TCGA-CM-5868-01A	COAD	7.25
TCGA-AA-A00O-01A	COAD	6.98
TCGA-AA-3527-01A	COAD	6.22
TCGA-AA-3712-01A	COAD	6.92
TCGA-D5-5540-01A	COAD	8.03
TCGA-A6-6137-01A	COAD	7.04
TCGA-A6-5667-01A	COAD	7.65
TCGA-G4-6628-01A	COAD	7.32
TCGA-AY-6196-01A	COAD	6.39
TCGA-AA-3693-01A	COAD	6.92
TCGA-CK-5913-01A	COAD	7.07
TCGA-AA-3688-01A	COAD	6.83
TCGA-F4-6460-01A	COAD	6.24
TCGA-AA-3548-01A	COAD	6.89
TCGA-CM-6172-01A	COAD	7.24
TCGA-AA-3844-01A	COAD	6.56
TCGA-AZ-4308-01A	COAD	5.94
TCGA-A6-3808-01A	COAD	7.66
TCGA-AD-6963-01A	COAD	7.33
TCGA-AA-A00L-01A	COAD	6.86
TCGA-CM-6161-01A	COAD	7.2
TCGA-AA-3980-01A	COAD	6.9
TCGA-CM-5341-01A	COAD	7.26
TCGA-CK-4951-01A	COAD	6.91
TCGA-A6-2678-01A	COAD	7.79
TCGA-AA-3869-01A	COAD	7.72
TCGA-QG-A5YX-01A	COAD	7.88
TCGA-F4-6808-01A	COAD	6.51
TCGA-G4-6306-01A	COAD	7.09
TCGA-G4-6626-01A	COAD	6.33
TCGA-G4-6294-01A	COAD	6.47
TCGA-AA-3858-01A	COAD	6.42
TCGA-DM-A28F-01A	COAD	7.19
TCGA-CA-5797-01A	COAD	7.88
TCGA-A6-6138-01A	COAD	7.21
TCGA-G4-6307-01A	COAD	6.61
TCGA-AY-6386-01A	COAD	8.44
TCGA-AZ-4614-01A	COAD	6.9
TCGA-AA-3555-01A	COAD	8.2
TCGA-AM-5820-01A	COAD	7.83
TCGA-AA-3560-01A	COAD	7.13
TCGA-D5-6541-01A	COAD	7.03
TCGA-CM-6676-01A	COAD	7.72
TCGA-A6-6649-01A	COAD	7.69
TCGA-A6-2671-01A	COAD	6.53
TCGA-AA-3812-01A	COAD	6.03
TCGA-A6-3807-01A	COAD	7.02
TCGA-AA-A01Q-01A	COAD	5.82
TCGA-AA-3956-01A	COAD	6.78
TCGA-A6-2681-01A	COAD	6.75
TCGA-AA-3841-01A	COAD	6.33
TCGA-CM-4752-01A	COAD	6.46
TCGA-WS-AB45-01A	COAD	7.01
TCGA-DM-A280-01A	COAD	7.1
TCGA-QG-A5Z2-01A	COAD	7.24

TCGA-AA-3496-01A	COAD	6.55
TCGA-A6-A56B-01A	COAD	6.21
TCGA-A6-6780-01A	COAD	8.91
TCGA-4N-A93T-01A	COAD	6.66
TCGA-AA-3930-01A	COAD	7.25
TCGA-CK-6747-01A	COAD	7.83
TCGA-CA-6715-01A	COAD	8.15
TCGA-A6-3809-01B	COAD	5.01
TCGA-G4-6588-01A	COAD	7.29
TCGA-AZ-6605-01A	COAD	6.91
TCGA-DM-A1D4-01A	COAD	6.94
TCGA-F4-6807-01A	COAD	7.32
TCGA-QG-A5YW-01A	COAD	6.84
TCGA-CK-6748-01A	COAD	6.4
TCGA-A6-5662-01A	COAD	6.88
TCGA-CM-6163-01A	COAD	7.27
TCGA-AA-3664-01A	COAD	6.67
TCGA-A6-6652-01A	COAD	6.73
TCGA-D5-6538-01A	COAD	7.2
TCGA-CM-5860-01A	COAD	7.48
TCGA-AA-A01K-01A	COAD	5.99
TCGA-5M-AATA-01A	COAD	7.3
TCGA-AA-3532-01A	COAD	7.62
TCGA-AA-3696-01A	COAD	7.66
TCGA-G4-6298-01A	COAD	7.1
TCGA-AA-3681-01A	COAD	6.77
TCGA-G4-6314-01A	COAD	6.93
TCGA-D5-6532-01A	COAD	7.65
TCGA-CM-5862-01A	COAD	6.79
TCGA-CM-5861-01A	COAD	7.4
TCGA-AA-A01R-01A	COAD	6.61
TCGA-AA-3525-01A	COAD	7.32
TCGA-AA-A00Z-01A	COAD	6.55
TCGA-A6-5659-01A	COAD	7.18
TCGA-A6-4107-01A	COAD	7.02
TCGA-AZ-6598-01A	COAD	7.24
TCGA-AA-3845-01A	COAD	7.24
TCGA-G4-6322-01A	COAD	7.61
TCGA-AA-3692-01A	COAD	7.1
TCGA-AZ-4323-01A	COAD	5.64
TCGA-A6-6781-01B	COAD	5.76
TCGA-AZ-4315-01A	COAD	8.27
TCGA-AA-3534-01A	COAD	7.77
TCGA-CA-6718-01A	COAD	7.07
TCGA-G4-6295-01A	COAD	7.45
TCGA-A6-2677-01A	COAD	7.39
TCGA-AD-A5EK-01A	COAD	6.52
TCGA-AZ-4616-01A	COAD	6.85
TCGA-D5-6920-01A	COAD	7.23
TCGA-F4-6809-01A	COAD	7.25
TCGA-AZ-6599-01A	COAD	6.59
TCGA-DM-A1DA-01A	COAD	6.89
TCGA-DM-A28K-01A	COAD	7.41
TCGA-A6-6782-01A	COAD	6.43
TCGA-AA-3531-01A	COAD	7.13
TCGA-A6-2674-01B	COAD	6.35
TCGA-AA-3877-01A	COAD	7.2
TCGA-NH-A6GA-01A	COAD	7.3

TCGA-AA-3986-01A	COAD	6.82
TCGA-CK-4952-01A	COAD	7.29
TCGA-A6-6141-01A	COAD	7.46
TCGA-G4-6625-01A	COAD	6.93
TCGA-AA-A02H-01A	COAD	6.36
TCGA-CK-4948-01B	COAD	7.13
TCGA-AD-6964-01A	COAD	6.74
TCGA-AA-A022-01A	COAD	6.05
TCGA-AA-3673-01A	COAD	7.36
TCGA-AA-3947-01A	COAD	7.73
TCGA-AA-3488-01A	COAD	7.15
TCGA-AA-3970-01A	COAD	6.96
TCGA-F4-6704-01A	COAD	7.46
TCGA-AY-A8YK-01A	COAD	6.73
TCGA-CM-4750-01A	COAD	6.65
TCGA-A6-6142-01A	COAD	6.42
TCGA-AA-3864-01A	COAD	8.78
TCGA-AA-3870-01A	COAD	5.93
TCGA-NH-A5IV-01A	COAD	6.81
TCGA-CK-5912-01A	COAD	7.65
TCGA-AA-3667-01A	COAD	5.43
TCGA-CM-6167-01A	COAD	6.71
TCGA-AA-3867-01A	COAD	7.63
TCGA-CM-4743-01A	COAD	6.64
TCGA-DM-A28M-01A	COAD	6.83
TCGA-AA-3554-01A	COAD	6.97
TCGA-AA-3517-01A	COAD	5.99
TCGA-AA-3968-01A	COAD	6.63
TCGA-AA-3949-01A	COAD	6.87
TCGA-A6-5657-01A	COAD	6.59
TCGA-G4-6309-01A	COAD	7.86
TCGA-A6-5664-01A	COAD	7.17
TCGA-CM-6162-01A	COAD	7.41
TCGA-AA-3814-01A	COAD	7.05
TCGA-AA-3680-01A	COAD	7.47
TCGA-D5-6530-01A	COAD	6.93
TCGA-AA-3556-01A	COAD	7.41
TCGA-CM-6165-01A	COAD	7.25
TCGA-AA-3710-01A	COAD	7.01
TCGA-D5-6533-01A	COAD	7.32
TCGA-AA-3818-01A	COAD	7.4
TCGA-AA-A00W-01A	COAD	6.74
TCGA-A6-2680-01A	COAD	6.89
TCGA-DM-A1D7-01A	COAD	7.44
TCGA-DM-A28G-01A	COAD	7.55
TCGA-A6-2683-01A	COAD	6.07
TCGA-G4-6320-01A	COAD	6.94
TCGA-AA-3549-01A	COAD	6.17
TCGA-AA-3685-01A	COAD	7.17
TCGA-DM-A282-01A	COAD	7.06
TCGA-CM-6171-01A	COAD	8.13
TCGA-F4-6463-01A	COAD	7.9
TCGA-D5-6539-01A	COAD	7.03
TCGA-AA-3544-01A	COAD	6.52
TCGA-A6-2685-01A	COAD	6.21
TCGA-CM-6680-01A	COAD	6.83
TCGA-AA-A01T-01A	COAD	5.54
TCGA-AA-3973-01A	COAD	6.06

TCGA-F4-6570-01A	COAD	7.04
TCGA-AA-3984-01A	COAD	7.06
TCGA-AD-6890-01A	COAD	6.82
TCGA-AA-A01D-01A	COAD	7.01
TCGA-AD-6899-01A	COAD	7.15
TCGA-D5-6928-01A	COAD	6.07
TCGA-DM-A1D6-01A	COAD	6.08
TCGA-AA-A02F-01A	COAD	6.94
TCGA-AU-3779-01A	COAD	6.62
TCGA-A6-5656-01A	COAD	7.15
TCGA-A6-5656-01B	COAD	7.02
TCGA-D5-6536-01A	COAD	6.26
TCGA-F4-6805-01A	COAD	7.26
TCGA-AA-A00N-01A	COAD	7.14
TCGA-AY-6197-01A	COAD	7.14
TCGA-G4-6321-01A	COAD	6.9
TCGA-DM-A28C-01A	COAD	7.57
TCGA-AZ-4682-01B	COAD	7.05
TCGA-AZ-6608-01A	COAD	7.05
TCGA-CM-6675-01A	COAD	7.63
TCGA-AA-3660-01A	COAD	6.94
TCGA-D5-6531-01A	COAD	6.31
TCGA-AA-3846-01A	COAD	6.96
TCGA-D5-6537-01A	COAD	7.44
TCGA-A6-5661-01B	COAD	3.81
TCGA-CM-5864-01A	COAD	6.62
TCGA-AA-A00J-01A	COAD	6.98
TCGA-RU-A8FL-01A	COAD	7.19
TCGA-D5-6929-01A	COAD	7.24
TCGA-AA-3861-01A	COAD	7.48
TCGA-AD-A5EJ-01A	COAD	7.59
TCGA-G4-6586-01A	COAD	6.49
TCGA-A6-6650-01A	COAD	7.59
TCGA-G4-6302-01A	COAD	6.37
TCGA-A6-5661-01A	COAD	7.82
TCGA-A6-2674-01A	COAD	6.75
TCGA-A6-5665-01A	COAD	7
TCGA-A6-2684-01C	COAD	6.36
TCGA-A6-5665-01B	COAD	4.91
TCGA-AA-3862-01A	COAD	6.77
TCGA-AD-6895-01A	COAD	6.35
TCGA-AA-3875-01A	COAD	7.43
TCGA-NH-A8F7-01A	COAD	6.86
TCGA-AA-3955-01A	COAD	7.13
TCGA-A6-6653-01A	COAD	7.54
TCGA-CA-5255-01A	COAD	7.3
TCGA-NH-A8F7-06A	COAD	7.14
TCGA-AA-A00F-01A	COAD	6.14
TCGA-AA-A02O-01A	COAD	6.41
TCGA-AA-3852-01A	COAD	6.59
TCGA-DM-A0X9-01A	COAD	7.42
TCGA-AA-A02W-01A	COAD	7.27
TCGA-AA-A01G-01A	COAD	7.3
TCGA-DM-A1HA-01A	COAD	6.71
TCGA-5M-AAT5-01A	COAD	6.83
TCGA-NH-A50T-01A	COAD	7.07
TCGA-AA-3494-01A	COAD	7.12
TCGA-CM-4747-01A	COAD	6.28

TCGA-D5-6931-01A	COAD	7.72
TCGA-AA-3713-01A	COAD	6.88
TCGA-AZ-4681-01A	COAD	6.53
TCGA-G4-6297-01A	COAD	6.5
TCGA-AZ-6601-01A	COAD	7.4
TCGA-AA-A01S-01A	COAD	7.3
TCGA-CM-6679-01A	COAD	6.41
TCGA-A6-A567-01A	COAD	7.01
TCGA-AA-A02J-01A	COAD	6.94
TCGA-CK-4947-01B	COAD	7.64
TCGA-A6-3809-01A	COAD	7.3
TCGA-DM-A1D8-01A	COAD	6.53
TCGA-A6-5659-01B	COAD	5.06
TCGA-A6-4105-01A	COAD	7.42
TCGA-AA-3542-01A	COAD	7.05
TCGA-AA-A029-01A	COAD	6.77
TCGA-DM-A1HB-01A	COAD	6.74
TCGA-DM-A285-01A	COAD	7.77
TCGA-SS-A7HO-01A	COAD	7.98
TCGA-AA-3842-01A	COAD	7.01
TCGA-AA-A01C-01A	COAD	6.23
TCGA-AA-3509-01A	COAD	7.6
TCGA-AA-3939-01A	COAD	7.53
TCGA-T9-A92H-01A	COAD	7.28
TCGA-DM-A28E-01A	COAD	6.65
TCGA-CM-6169-01A	COAD	6.6
TCGA-AD-6889-01A	COAD	7.61
TCGA-AA-A01I-01A	COAD	7.9
TCGA-4T-AA8H-01A	COAD	6.39
TCGA-AA-3524-01A	COAD	7.48
TCGA-A6-A5ZU-01A	COAD	6.25
TCGA-AA-3982-01A	COAD	7.24
TCGA-AZ-6603-01A	COAD	6.74
TCGA-CM-6164-01A	COAD	7.34
TCGA-CK-5914-01A	COAD	7.67
TCGA-AY-4070-01A	COAD	7.23
TCGA-AZ-6607-01A	COAD	5.87
TCGA-A6-6650-01B	COAD	6.79
TCGA-AA-3854-01A	COAD	6.99
TCGA-CA-5796-01A	COAD	7.53
TCGA-A6-2682-01A	COAD	7.04
TCGA-AZ-5403-01A	COAD	7.95
TCGA-A6-A566-01A	COAD	7.29
TCGA-A6-6780-01B	COAD	6.89
TCGA-AA-3492-01A	COAD	7.83
TCGA-AA-3941-01A	COAD	7.12
TCGA-AA-3526-01A	COAD	8.15
TCGA-F4-6459-01A	COAD	7.91
TCGA-AA-3511-01A	COAD	6.56
TCGA-CK-6746-01A	COAD	6.68
TCGA-A6-6654-01A	COAD	7.3
TCGA-AA-3994-01A	COAD	7.04
TCGA-AA-3543-01A	COAD	6.69
TCGA-AA-3502-01A	COAD	6.55
TCGA-CA-6716-01A	COAD	8.17
TCGA-AA-3972-01A	COAD	6.51
TCGA-G4-6311-01A	COAD	7.18
TCGA-A6-2676-01A	COAD	7.25

TCGA-DM-A0XD-01A	COAD	6.23
TCGA-D5-5541-01A	COAD	8
TCGA-AA-3520-01A	COAD	7.43
TCGA-CM-6674-01A	COAD	7.55
TCGA-AM-5821-01A	COAD	6.76
TCGA-F4-6856-01A	COAD	8.26
TCGA-A6-6648-01A	COAD	7.39
TCGA-CM-6168-01A	COAD	7.71
TCGA-CK-6751-01A	COAD	7.57
TCGA-AA-A00Q-01A	COAD	7.13
TCGA-AA-A02R-01A	COAD	6.91
TCGA-CM-5863-01A	COAD	6.75
TCGA-CM-4748-01A	COAD	6.63
TCGA-5M-AAT6-01A	COAD	6.95
TCGA-G4-6317-01A	COAD	7.65
TCGA-G4-6317-02A	COAD	6.85
TCGA-AA-3672-01A	COAD	6.28
TCGA-AA-3684-01A	COAD	6.06
TCGA-AA-3679-01A	COAD	7.12
TCGA-D5-6534-01A	COAD	6.61
TCGA-AA-3989-01A	COAD	7.05
TCGA-AY-A54L-01A	COAD	7.1
TCGA-D5-5537-01A	COAD	7.92
TCGA-AA-A02Y-01A	COAD	7.49
TCGA-AA-3666-01A	COAD	6.8
TCGA-A6-3810-01A	COAD	6.16
TCGA-CM-4746-01A	COAD	6.91
TCGA-QG-A5Z1-01A	COAD	7.51
TCGA-AA-3975-01A	COAD	5.89
TCGA-CA-6719-01A	COAD	6.79
TCGA-AA-3678-01A	COAD	7.43
TCGA-A6-6781-01A	COAD	6.64
TCGA-A6-2672-01A	COAD	6.98
TCGA-D5-6535-01A	COAD	6.64
TCGA-AA-3521-01A	COAD	6.63
TCGA-DM-A28H-01A	COAD	7.3
TCGA-5M-AATE-01A	COAD	6.61
TCGA-AA-A017-01A	COAD	6.11
TCGA-AA-3815-01A	COAD	6.84
TCGA-D5-6927-01A	COAD	7.31
TCGA-AZ-5407-01A	COAD	6.71
TCGA-F4-6806-01A	COAD	6.92
TCGA-D5-6540-01A	COAD	6.36
TCGA-D5-6529-01A	COAD	7.13
TCGA-AA-3966-01A	COAD	6.15
TCGA-A6-3810-01B	COAD	6.28
TCGA-AA-3952-01A	COAD	6.54
TCGA-CM-6170-01A	COAD	6.82
TCGA-AA-A004-01A	COAD	3.67
TCGA-AZ-4615-01A	COAD	7.96
TCGA-AA-3529-01A	COAD	7.83
TCGA-F4-6569-01A	COAD	7.03
TCGA-NH-A50V-01A	COAD	5.94
TCGA-AA-3950-01A	COAD	7.9
TCGA-CK-4950-01A	COAD	7.76
TCGA-AZ-6606-01A	COAD	7.14
TCGA-AA-3489-01A	COAD	6.61
TCGA-DM-A1DB-01A	COAD	7.6

TCGA-AA-A03J-01A	COAD	5.69
TCGA-AA-3860-01A	COAD	7.34
TCGA-AY-A69D-01A	COAD	7.78
TCGA-CM-6166-01A	COAD	8.1
TCGA-AZ-6600-01A	COAD	7.06
TCGA-AA-3979-01A	COAD	7.26
TCGA-CM-5344-01A	COAD	6.59
TCGA-D5-6923-01A	COAD	6.89
TCGA-CM-6677-01A	COAD	7.17
TCGA-AA-3855-01A	COAD	6.75
TCGA-AA-3518-01A	COAD	7.14
TCGA-NH-A50U-01A	COAD	6.99
TCGA-CK-5915-01A	COAD	7.68
TCGA-A6-2672-01B	COAD	5.79
TCGA-DM-A28A-01A	COAD	7.62
TCGA-AD-6901-01A	COAD	6.91
TCGA-AA-3663-01A	COAD	7.9
TCGA-CM-5348-01A	COAD	6.06
TCGA-QG-A5YV-01A	COAD	7.23
TCGA-AD-6965-01A	COAD	7.12
TCGA-AA-A01Z-01A	COAD	6.11
TCGA-AA-3715-01A	COAD	6.3
TCGA-AA-3811-01A	COAD	7.15
TCGA-QL-A97D-01A	COAD	6.81
TCGA-D5-7000-01A	COAD	7.01
TCGA-DM-A1D9-01A	COAD	7.39
TCGA-AA-3514-01A	COAD	6.55
TCGA-A6-2677-01B	COAD	6.58
TCGA-AY-A71X-01A	COAD	6.57
TCGA-AA-A024-01A	COAD	6.62
TCGA-AA-3553-01A	COAD	7.29
TCGA-AA-3538-01A	COAD	7.57
TCGA-G4-6315-01A	COAD	7.81
TCGA-NH-A6GB-01A	COAD	6.02
TCGA-AA-3697-01A	COAD	6.8
TCGA-AA-3516-01A	COAD	6.52
TCGA-AZ-4684-01A	COAD	8.23
TCGA-3L-AA1B-01A	COAD	6.53
TCGA-AD-6548-01A	COAD	7.01
TCGA-AA-3831-01A	COAD	7.12
TCGA-DM-A0XF-01A	COAD	8
TCGA-D5-6924-01A	COAD	6.83
TCGA-CM-4744-01A	COAD	6.72
TCGA-CM-6678-01A	COAD	7.11
TCGA-AA-A00R-01A	COAD	6.02
TCGA-AA-3522-01A	COAD	7.45
TCGA-A6-A565-01A	COAD	6.28
TCGA-AA-3662-01A	COAD	5.79
TCGA-CM-4751-01A	COAD	7.26
TCGA-AA-3848-01A	COAD	5.46
TCGA-AA-3971-01A	COAD	6.98
TCGA-A6-5660-01A	COAD	7
TCGA-AY-5543-01A	COAD	7.71
TCGA-AA-3530-01A	COAD	6.86
TCGA-F4-6855-01A	COAD	7.1
TCGA-DM-A288-01A	COAD	6.63
TCGA-G4-6310-01A	COAD	6.88
TCGA-G4-6303-01A	COAD	6.88

TCGA-D5-6898-01A	COAD	8.42
TCGA-A6-6140-01A	COAD	7.11
TCGA-AA-3821-01A	COAD	7.75
TCGA-AA-3562-01A	COAD	6.96
TCGA-G4-6323-01A	COAD	6.84
TCGA-D5-6932-01A	COAD	6.46
TCGA-AA-A01P-01A	COAD	6.13
TCGA-AA-3851-01A	COAD	7.92
TCGA-G4-6293-01A	COAD	6.71
TCGA-CM-5349-01A	COAD	7.19
TCGA-AA-A01F-01A	COAD	6.59
TCGA-D5-5539-01A	COAD	6.6
TCGA-AA-3819-01A	COAD	6.96
TCGA-NH-A8F8-01A	COAD	6.8
TCGA-AA-3695-01A	COAD	7.07
TCGA-AA-3850-01A	COAD	6.66
TCGA-AA-3552-01A	COAD	7.3
TCGA-AA-3506-01A	COAD	6.67
TCGA-CA-6717-01A	COAD	7.74
TCGA-5M-AAT4-01A	COAD	7.12
TCGA-A6-6651-01A	COAD	6.28
TCGA-AA-A00E-01A	COAD	6.82
TCGA-AA-3856-01A	COAD	6.86
TCGA-AA-3976-01A	COAD	7.09
TCGA-A6-2679-01A	COAD	5.09
TCGA-AA-3837-01A	COAD	6.88
TCGA-DM-A1D0-01A	COAD	7.58
TCGA-G4-6627-01A	COAD	6.95
TCGA-A6-2686-01A	COAD	5.85
TCGA-A6-5666-01A	COAD	7.65
TCGA-AA-3833-01A	COAD	6.48
TCGA-NH-A6GC-01A	COAD	6.56
TCGA-AA-A010-01A	COAD	7.74
TCGA-A6-2675-01A	COAD	7.05
TCGA-AA-A00D-01A	COAD	6.73
TCGA-AA-A00U-01A	COAD	6.8
TCGA-AA-A01V-01A	COAD	6.54
TCGA-AA-3519-01A	COAD	6.76
TCGA-F4-6703-01A	COAD	7.07
TCGA-GS-A9TY-01A	DLBC	4.5
TCGA-G8-6914-01A	DLBC	5.02
TCGA-FF-8041-01A	DLBC	4.35
TCGA-RQ-A68N-01A	DLBC	4.79
TCGA-G8-6907-01A	DLBC	2.93
TCGA-GS-A9TT-01A	DLBC	5.05
TCGA-FF-8046-01A	DLBC	4.51
TCGA-FA-A6HO-01A	DLBC	5.13
TCGA-G8-6324-01A	DLBC	3.94
TCGA-G8-6326-01A	DLBC	5.24
TCGA-FM-8000-01A	DLBC	3.99
TCGA-GS-A9TQ-01A	DLBC	5.16
TCGA-G8-6906-01A	DLBC	4.34
TCGA-GS-A9U4-01A	DLBC	5.81
TCGA-GR-A4D9-01B	DLBC	5.93
TCGA-GS-A9TW-01A	DLBC	4.94
TCGA-GS-A9TZ-01A	DLBC	6.49
TCGA-FA-A82F-01A	DLBC	5.01
TCGA-FF-A7CQ-01A	DLBC	5.93

TCGA-GS-A9U3-01A	DLBC	5.53
TCGA-FF-A7CW-01A	DLBC	4.61
TCGA-FA-A4BB-01A	DLBC	4.71
TCGA-FA-A6HN-01A	DLBC	4.48
TCGA-FF-8062-01A	DLBC	4.6
TCGA-FF-A7CX-01A	DLBC	5.12
TCGA-G8-6909-01A	DLBC	4.86
TCGA-FA-A86F-01A	DLBC	5.32
TCGA-GR-A4D6-01A	DLBC	3.75
TCGA-GR-7351-01A	DLBC	4.37
TCGA-FA-8693-01A	DLBC	5.13
TCGA-VB-A8QN-01A	DLBC	5.03
TCGA-GR-7353-01A	DLBC	3.34
TCGA-GS-A9TV-01A	DLBC	6.32
TCGA-FF-8043-01A	DLBC	4.41
TCGA-GR-A4D4-01A	DLBC	5.46
TCGA-GS-A9TU-01A	DLBC	5.43
TCGA-RQ-AAAT-01A	DLBC	3.21
TCGA-GS-A9TX-01A	DLBC	5.04
TCGA-FA-A7Q1-01A	DLBC	5.38
TCGA-FA-A7DS-01A	DLBC	4.66
TCGA-FF-8042-01A	DLBC	4.81
TCGA-G8-6325-01A	DLBC	5.3
TCGA-FF-A7CR-01A	DLBC	6.63
TCGA-FF-8047-01A	DLBC	4.19
TCGA-GR-A4D5-01A	DLBC	5.29
TCGA-FA-A4XK-01A	DLBC	5.89
TCGA-FF-8061-01A	DLBC	4.23
TCGA-RQ-A6JB-01A	DLBC	5.66
TCGA-LN-A49Y-01A	ESCA	5.41
TCGA-JY-A6FB-01A	ESCA	7.57
TCGA-VR-A8EY-01A	ESCA	7.12
TCGA-L5-A8NV-01A	ESCA	5.99
TCGA-Q9-A6FW-01A	ESCA	6.43
TCGA-LN-A49L-01A	ESCA	8.02
TCGA-LN-A49P-01A	ESCA	6.86
TCGA-L5-A4ON-01A	ESCA	7.54
TCGA-LN-A9FO-01A	ESCA	5.17
TCGA-L5-A4OI-01A	ESCA	6.85
TCGA-R6-A6L4-01A	ESCA	6.73
TCGA-L5-A8NW-01A	ESCA	5.92
TCGA-L5-A4OT-01A	ESCA	6.69
TCGA-L5-A88W-01A	ESCA	5.3
TCGA-L5-A4OS-01A	ESCA	6.42
TCGA-V5-A7RB-01A	ESCA	6.36
TCGA-2H-A9GR-01A	ESCA	5.61
TCGA-VR-A8EP-01A	ESCA	5.9
TCGA-2H-A9GI-01A	ESCA	6.38
TCGA-IG-A51D-01A	ESCA	5.39
TCGA-LN-A49W-01A	ESCA	5.59
TCGA-L7-A6VZ-01A	ESCA	7.14
TCGA-2H-A9GJ-01A	ESCA	7.55
TCGA-VR-A8EZ-01A	ESCA	6.46
TCGA-ZR-A9CJ-01B	ESCA	5.82
TCGA-2H-A9GL-01A	ESCA	5.87
TCGA-R6-A6DN-01B	ESCA	6.73
TCGA-L5-A88V-01A	ESCA	8.75
TCGA-VR-A8EU-01A	ESCA	5.38

TCGA-LN-A49N-01A	ESCA	6.07
TCGA-X8-AAAR-01A	ESCA	5.22
TCGA-VR-AA7B-01A	ESCA	6.16
TCGA-L5-A8NJ-01A	ESCA	6.4
TCGA-2H-A9GQ-01A	ESCA	6.54
TCGA-R6-A8W8-01B	ESCA	7.46
TCGA-LN-A49S-01A	ESCA	6.69
TCGA-L5-A8NR-01A	ESCA	5.81
TCGA-LN-A4A6-01A	ESCA	6.72
TCGA-LN-A4A2-01A	ESCA	7.27
TCGA-IC-A6RF-01A	ESCA	6.15
TCGA-LN-A8HZ-01A	ESCA	6.83
TCGA-L5-A4OJ-01A	ESCA	6.12
TCGA-IG-A5S3-01A	ESCA	5.47
TCGA-LN-A49U-01A	ESCA	6.94
TCGA-L5-A891-01A	ESCA	7.01
TCGA-LN-A4A4-01A	ESCA	6.15
TCGA-L5-A8NU-01A	ESCA	5.14
TCGA-LN-A9FR-01A	ESCA	6.99
TCGA-L5-A88Y-01A	ESCA	5.99
TCGA-KH-A6WC-01A	ESCA	6.02
TCGA-R6-A6DQ-01B	ESCA	6.28
TCGA-LN-A7HZ-01A	ESCA	6.49
TCGA-2H-A9GK-01A	ESCA	5.22
TCGA-L5-A43H-01A	ESCA	5.65
TCGA-IG-A3YC-01A	ESCA	5.23
TCGA-IG-A4P3-01A	ESCA	5.79
TCGA-VR-A8EO-01A	ESCA	5.19
TCGA-VR-A8EW-01A	ESCA	6.67
TCGA-R6-A6Y0-01B	ESCA	6
TCGA-L5-A8NE-01A	ESCA	7.4
TCGA-L5-A4OO-01A	ESCA	6.38
TCGA-L5-A88T-01A	ESCA	6.31
TCGA-IG-A97H-01A	ESCA	6.75
TCGA-IG-A50L-01A	ESCA	7.24
TCGA-LN-A7HW-01A	ESCA	8.13
TCGA-L5-A8NN-01A	ESCA	8.03
TCGA-L5-A4OM-01A	ESCA	6.5
TCGA-Z6-A9VB-01A	ESCA	6.05
TCGA-JY-A939-01A	ESCA	5.82
TCGA-L5-A43M-01A	ESCA	6.84
TCGA-L5-A43I-01A	ESCA	7.3
TCGA-LN-A7HV-01A	ESCA	5.98
TCGA-L5-A88S-01A	ESCA	4.79
TCGA-L5-A4OW-01A	ESCA	6.96
TCGA-Z6-A8JE-01A	ESCA	6.4
TCGA-LN-A9FQ-01A	ESCA	7.54
TCGA-L5-A4OH-01A	ESCA	8.13
TCGA-LN-A4A3-01A	ESCA	5.43
TCGA-LN-A8I0-01A	ESCA	5.28
TCGA-IC-A6RE-01A	ESCA	6.11
TCGA-V5-AASX-01A	ESCA	7.32
TCGA-L5-A4OR-01A	ESCA	7.22
TCGA-L5-A4OE-01A	ESCA	5.38
TCGA-L5-A8NK-01A	ESCA	5.9
TCGA-IG-A3YA-01A	ESCA	6.22
TCGA-IG-A97I-01A	ESCA	5.65
TCGA-V5-AASV-01A	ESCA	6.96

TCGA-R6-A6KZ-01A	ESCA	7.19
TCGA-LN-A4A8-01A	ESCA	7.18
TCGA-R6-A6XQ-01B	ESCA	6.35
TCGA-IG-A5B8-01A	ESCA	5.24
TCGA-RE-A7BO-01A	ESCA	4.88
TCGA-R6-A8WC-01A	ESCA	7.32
TCGA-L5-A8NL-01A	ESCA	7.09
TCGA-2H-A9GN-01A	ESCA	6.04
TCGA-Q9-A6FU-01A	ESCA	6.29
TCGA-LN-A5U5-01A	ESCA	8.33
TCGA-2H-A9GG-01A	ESCA	5.11
TCGA-2H-A9GM-01A	ESCA	6.86
TCGA-VR-A8EQ-01A	ESCA	6.17
TCGA-JY-A6FG-01A	ESCA	4.35
TCGA-L5-A43E-01A	ESCA	6.55
TCGA-JY-A93D-01A	ESCA	6.64
TCGA-L5-A4OP-01A	ESCA	6.08
TCGA-LN-A7HX-01A	ESCA	6.33
TCGA-IG-A3YB-01A	ESCA	5.92
TCGA-IG-A8O2-01A	ESCA	7.34
TCGA-LN-A9FP-01A	ESCA	5.84
TCGA-VR-AA7I-01A	ESCA	5.67
TCGA-JY-A6F8-01A	ESCA	6.45
TCGA-LN-A49X-01A	ESCA	5.32
TCGA-LN-A8I1-01A	ESCA	6
TCGA-L5-A8NQ-01A	ESCA	4.79
TCGA-VR-A8EX-01A	ESCA	6.08
TCGA-L5-A4OF-01A	ESCA	7.95
TCGA-L5-A43J-01A	ESCA	6.24
TCGA-M9-A5M8-01A	ESCA	6.55
TCGA-R6-A6XG-01B	ESCA	7.43
TCGA-VR-A8Q7-01A	ESCA	7.39
TCGA-IG-A4QT-01A	ESCA	4.58
TCGA-V5-A7RE-01A	ESCA	7.73
TCGA-L5-A88Z-01A	ESCA	6.56
TCGA-VR-A8ER-01A	ESCA	6.76
TCGA-JY-A6FD-01A	ESCA	4.6
TCGA-LN-A4MQ-01A	ESCA	7.98
TCGA-Z6-AAPN-01A	ESCA	5.41
TCGA-LN-A49O-01A	ESCA	4.99
TCGA-LN-A49R-01A	ESCA	6.12
TCGA-IG-A7DP-01A	ESCA	4.71
TCGA-S8-A6BV-01A	ESCA	5.8
TCGA-R6-A6L6-01B	ESCA	4.97
TCGA-R6-A8WG-01A	ESCA	7.09
TCGA-L5-A4OX-01A	ESCA	6.89
TCGA-IG-A4QS-01A	ESCA	6.22
TCGA-R6-A6Y2-01B	ESCA	6.19
TCGA-L7-A56G-01A	ESCA	5.63
TCGA-LN-A49V-01A	ESCA	5.68
TCGA-L5-A8NT-01A	ESCA	7.46
TCGA-LN-A7HY-01A	ESCA	5.37
TCGA-XP-A8T8-01A	ESCA	6.62
TCGA-JY-A938-01A	ESCA	6.4
TCGA-L5-A8NG-01A	ESCA	6.29
TCGA-V5-A7RC-06A	ESCA	6.61
TCGA-V5-A7RC-01B	ESCA	6.49
TCGA-LN-A4MR-01A	ESCA	5.05

TCGA-LN-A5U7-01A	ESCA	6.92
TCGA-XP-A8T6-01A	ESCA	6.3
TCGA-L5-A893-01A	ESCA	6.1
TCGA-L5-A4OG-01A	ESCA	6.98
TCGA-VR-A8ET-01A	ESCA	4.66
TCGA-LN-A4A9-01A	ESCA	4.68
TCGA-V5-AASW-01A	ESCA	6.83
TCGA-2H-A9GO-01A	ESCA	6.79
TCGA-L5-A4OU-01A	ESCA	5.95
TCGA-L5-A43C-01A	ESCA	5.53
TCGA-VR-AA4G-01A	ESCA	4.98
TCGA-S8-A6BW-01A	ESCA	5.38
TCGA-L5-A8NH-01A	ESCA	5.99
TCGA-IG-A3I8-01A	ESCA	5.43
TCGA-IG-A625-01A	ESCA	6.72
TCGA-2H-A9GH-01A	ESCA	8.81
TCGA-JY-A93F-01A	ESCA	4.32
TCGA-R6-A8W5-01B	ESCA	8.11
TCGA-L5-A8NI-01A	ESCA	7.2
TCGA-LN-A5U6-01A	ESCA	6.47
TCGA-LN-A4A1-01A	ESCA	6.01
TCGA-LN-A4A5-01A	ESCA	6.19
TCGA-VR-AA7D-01A	ESCA	5.88
TCGA-JY-A93E-01A	ESCA	5.88
TCGA-LN-A49K-01A	ESCA	6.13
TCGA-L5-A8NS-01A	ESCA	6.41
TCGA-JY-A6FA-01A	ESCA	7.18
TCGA-JY-A6FE-01A	ESCA	5.56
TCGA-IG-A3Y9-01A	ESCA	4.9
TCGA-L5-A4OQ-01A	ESCA	7.56
TCGA-2H-A9GF-01A	ESCA	6.59
TCGA-IG-A6QS-01A	ESCA	4.93
TCGA-LN-A49M-01A	ESCA	5.65
TCGA-Z6-A8JD-01A	ESCA	4.87
TCGA-L5-A8NM-01A	ESCA	6.73
TCGA-JY-A93C-01A	ESCA	7.3
TCGA-JY-A6FH-01A	ESCA	7.9
TCGA-L5-A8NF-01A	ESCA	7.39
TCGA-VR-AA4D-01A	ESCA	7.05
TCGA-IG-A3QL-01A	ESCA	6.23
TCGA-27-2523-01A	GBM	7.93
TCGA-06-0190-02A	GBM	6.49
TCGA-06-0130-01A	GBM	6.46
TCGA-76-4926-01B	GBM	6.22
TCGA-06-0125-02A	GBM	6.42
TCGA-26-5136-01B	GBM	6.59
TCGA-12-0619-01A	GBM	6.49
TCGA-06-5411-01A	GBM	6.84
TCGA-06-5418-01A	GBM	7.05
TCGA-16-0846-01A	GBM	5.86
TCGA-27-2521-01A	GBM	6.03
TCGA-27-2526-01A	GBM	7.45
TCGA-06-0138-01A	GBM	6.32
TCGA-41-2571-01A	GBM	6.1
TCGA-06-0649-01B	GBM	6.1
TCGA-14-0871-01A	GBM	5.13
TCGA-27-2524-01A	GBM	7.04
TCGA-76-4927-01A	GBM	7.65

TCGA-06-0747-01A	GBM	5.54
TCGA-28-2514-01A	GBM	6.46
TCGA-19-1787-01B	GBM	5.87
TCGA-06-0141-01A	GBM	6.26
TCGA-32-2632-01A	GBM	6.38
TCGA-06-5859-01A	GBM	6.53
TCGA-14-0789-01A	GBM	6.94
TCGA-06-0645-01A	GBM	7.57
TCGA-14-2554-01A	GBM	6.82
TCGA-06-2557-01A	GBM	6.63
TCGA-06-0211-02A	GBM	7.45
TCGA-06-0211-01A	GBM	7.4
TCGA-27-1835-01A	GBM	6.04
TCGA-14-0781-01B	GBM	6.16
TCGA-19-4065-02A	GBM	5.54
TCGA-41-4097-01A	GBM	6.44
TCGA-06-0139-01A	GBM	7.55
TCGA-14-0790-01B	GBM	7.31
TCGA-41-2572-01A	GBM	7.22
TCGA-06-0882-01A	GBM	6.55
TCGA-06-0156-01A	GBM	6.53
TCGA-06-0184-01A	GBM	6.59
TCGA-26-5132-01A	GBM	6.48
TCGA-19-2624-01A	GBM	7.28
TCGA-28-1747-01C	GBM	7.16
TCGA-32-2638-01A	GBM	6.59
TCGA-19-1389-02A	GBM	6.21
TCGA-06-0219-01A	GBM	6.67
TCGA-28-2513-01A	GBM	6.63
TCGA-06-5416-01A	GBM	6.7
TCGA-02-0047-01A	GBM	6.53
TCGA-06-0750-01A	GBM	6.73
TCGA-19-1390-01A	GBM	6.56
TCGA-32-2634-01A	GBM	6.15
TCGA-06-0187-01A	GBM	7.3
TCGA-26-5134-01A	GBM	6.48
TCGA-12-3653-01A	GBM	7.75
TCGA-08-0386-01A	GBM	7.22
TCGA-32-4213-01A	GBM	6.94
TCGA-32-2615-01A	GBM	6.58
TCGA-15-1444-01A	GBM	5.94
TCGA-28-5208-01A	GBM	7.6
TCGA-02-2485-01A	GBM	6.34
TCGA-12-3650-01A	GBM	7.99
TCGA-14-1034-02B	GBM	7.65
TCGA-12-1597-01B	GBM	6.35
TCGA-16-1045-01B	GBM	7.46
TCGA-14-1402-02A	GBM	6.81
TCGA-28-5215-01A	GBM	6.99
TCGA-06-2558-01A	GBM	7.09
TCGA-27-1832-01A	GBM	5.98
TCGA-06-2565-01A	GBM	6.29
TCGA-06-5410-01A	GBM	7.66
TCGA-76-4931-01A	GBM	7.84
TCGA-06-5415-01A	GBM	7.18
TCGA-28-5220-01A	GBM	6.15
TCGA-76-4925-01A	GBM	7.17
TCGA-06-5413-01A	GBM	8.28

TCGA-06-2564-01A	GBM	7.67
TCGA-06-0878-01A	GBM	7.33
TCGA-28-5216-01A	GBM	6.32
TCGA-14-0736-02A	GBM	5.99
TCGA-32-1982-01A	GBM	5.99
TCGA-27-2528-01A	GBM	6.65
TCGA-76-4929-01A	GBM	6.71
TCGA-28-5218-01A	GBM	7.13
TCGA-06-2559-01A	GBM	7.39
TCGA-41-5651-01A	GBM	6.81
TCGA-28-1753-01A	GBM	7.31
TCGA-28-2509-01A	GBM	6
TCGA-06-0132-01A	GBM	6.6
TCGA-02-2483-01A	GBM	6.64
TCGA-06-0190-01A	GBM	7.19
TCGA-02-2486-01A	GBM	6.67
TCGA-06-0743-01A	GBM	5.85
TCGA-32-5222-01A	GBM	6.79
TCGA-12-0616-01A	GBM	7.13
TCGA-06-0157-01A	GBM	6.79
TCGA-06-0152-02A	GBM	8
TCGA-27-1831-01A	GBM	7.42
TCGA-19-2619-01A	GBM	7.56
TCGA-06-0646-01A	GBM	6.63
TCGA-12-0618-01A	GBM	6.11
TCGA-12-0821-01A	GBM	6.22
TCGA-06-5856-01A	GBM	7.03
TCGA-06-0210-01A	GBM	6.3
TCGA-76-4928-01B	GBM	6.63
TCGA-06-0238-01A	GBM	6.47
TCGA-14-1825-01A	GBM	6.9
TCGA-76-4932-01A	GBM	7.41
TCGA-06-0749-01A	GBM	5.37
TCGA-26-5139-01A	GBM	6.77
TCGA-28-2499-01A	GBM	5.63
TCGA-06-2561-01A	GBM	6.07
TCGA-02-0055-01A	GBM	6.61
TCGA-32-1980-01A	GBM	6
TCGA-19-2625-01A	GBM	5.23
TCGA-06-0745-01A	GBM	6.5
TCGA-06-2567-01A	GBM	6.83
TCGA-06-0744-01A	GBM	6.87
TCGA-06-0171-02A	GBM	7.7
TCGA-06-0178-01A	GBM	6.24
TCGA-32-2616-01A	GBM	6.97
TCGA-14-1829-01A	GBM	6.81
TCGA-14-0817-01A	GBM	6.9
TCGA-14-1823-01A	GBM	6.63
TCGA-19-0957-02A	GBM	5.85
TCGA-06-0129-01A	GBM	6.66
TCGA-26-1442-01A	GBM	5.44
TCGA-06-0686-01A	GBM	6.48
TCGA-12-5295-01A	GBM	7.48
TCGA-06-1804-01A	GBM	6.81
TCGA-19-5960-01A	GBM	6.08
TCGA-06-0125-01A	GBM	7.47
TCGA-06-5408-01A	GBM	7.8
TCGA-14-0787-01A	GBM	7.59

TCGA-19-4065-01A	GBM	6.9
TCGA-06-2570-01A	GBM	6.17
TCGA-26-5133-01A	GBM	6.62
TCGA-27-1837-01A	GBM	7.49
TCGA-06-5858-01A	GBM	7.26
TCGA-19-2629-01A	GBM	6.02
TCGA-06-2563-01A	GBM	7.61
TCGA-06-0158-01A	GBM	6.7
TCGA-06-2562-01A	GBM	6.34
TCGA-06-0210-02A	GBM	6.53
TCGA-06-5412-01A	GBM	6.27
TCGA-28-5207-01A	GBM	6.73
TCGA-06-0174-01A	GBM	7.56
TCGA-15-0742-01A	GBM	7.67
TCGA-06-0168-01A	GBM	6.22
TCGA-19-2620-01A	GBM	5.91
TCGA-26-5135-01A	GBM	6.02
TCGA-06-2569-01A	GBM	6.31
TCGA-32-1970-01A	GBM	6.56
TCGA-06-0211-01B	GBM	7.89
TCGA-12-5299-01A	GBM	7.87
TCGA-27-1830-01A	GBM	6.34
TCGA-27-1834-01A	GBM	6.34
TCGA-27-2519-01A	GBM	6.52
TCGA-28-5204-01A	GBM	8.23
TCGA-41-3915-01A	GBM	6.68
TCGA-06-0644-01A	GBM	6.61
TCGA-28-2510-01A	GBM	5.58
TCGA-28-5213-01A	GBM	7.37
TCGA-06-5414-01A	GBM	7.42
TCGA-06-5417-01A	GBM	7.21
TCGA-06-0221-02A	GBM	6.32
TCGA-28-5209-01A	GBM	7.49
TCGA-12-3652-01A	GBM	6.54
TCGA-14-1034-01A	GBM	6.88
TCGA-CV-A464-01A	HNSC	5.49
TCGA-CV-6960-01A	HNSC	5.89
TCGA-CV-A45W-01A	HNSC	5.99
TCGA-BA-A4IF-01A	HNSC	6.44
TCGA-CN-5366-01A	HNSC	5.85
TCGA-MT-A51X-01A	HNSC	5.19
TCGA-P3-A5QE-01A	HNSC	6.32
TCGA-CV-6942-01A	HNSC	4.85
TCGA-CN-6022-01A	HNSC	5.53
TCGA-CV-7406-01A	HNSC	5.76
TCGA-UF-A71A-06A	HNSC	6.75
TCGA-CV-7263-01A	HNSC	6.23
TCGA-CV-5430-01A	HNSC	7.16
TCGA-P3-A6T2-01A	HNSC	6.05
TCGA-UF-A7JA-01A	HNSC	5.83
TCGA-CV-7097-01A	HNSC	5.45
TCGA-HD-A634-01A	HNSC	5.83
TCGA-CV-A6K2-01A	HNSC	5.82
TCGA-HL-7533-01A	HNSC	5.67
TCGA-CR-6474-01A	HNSC	5.35
TCGA-CX-7086-01A	HNSC	6
TCGA-CV-6962-01A	HNSC	7.03
TCGA-CN-A63T-01A	HNSC	6.43

TCGA-CV-7245-01A	HNSC	5.34
TCGA-CN-5358-01A	HNSC	5.18
TCGA-QK-A8ZA-01A	HNSC	7.39
TCGA-KU-A6H7-06A	HNSC	5.79
TCGA-CR-7376-01A	HNSC	6.16
TCGA-IQ-A61I-01A	HNSC	4.92
TCGA-CR-6480-01A	HNSC	7.51
TCGA-CN-4739-01A	HNSC	5.76
TCGA-CV-6952-01A	HNSC	5.36
TCGA-BB-7872-01A	HNSC	5.63
TCGA-P3-A5QA-01A	HNSC	5.48
TCGA-CR-7370-01A	HNSC	7.67
TCGA-CV-7424-01A	HNSC	7.38
TCGA-CN-A6V3-01A	HNSC	5.36
TCGA-4P-AA8J-01A	HNSC	4.93
TCGA-CQ-5324-01A	HNSC	8.1
TCGA-CR-6467-01A	HNSC	5.44
TCGA-CR-7402-01A	HNSC	5.17
TCGA-P3-A6T7-01A	HNSC	6.12
TCGA-CV-6436-01A	HNSC	4.37
TCGA-CR-7369-01A	HNSC	5.63
TCGA-F7-A50G-01A	HNSC	5.36
TCGA-CQ-6220-01A	HNSC	5.17
TCGA-CV-6936-01A	HNSC	5.41
TCGA-CN-4738-01A	HNSC	5.71
TCGA-BB-A5HZ-01A	HNSC	4.74
TCGA-CN-6021-01A	HNSC	6.36
TCGA-BA-5153-01A	HNSC	6.8
TCGA-CV-7104-01A	HNSC	5.9
TCGA-CQ-A4CH-01A	HNSC	5.33
TCGA-CN-4722-01A	HNSC	5.51
TCGA-HD-8635-01A	HNSC	5.44
TCGA-BA-A6D8-01A	HNSC	5.3
TCGA-CN-5359-01A	HNSC	6.67
TCGA-BA-A6DG-01A	HNSC	4.98
TCGA-BB-4225-01A	HNSC	5.2
TCGA-CQ-A4CB-01A	HNSC	5.47
TCGA-TN-A7HI-01A	HNSC	3.69
TCGA-CV-6956-01A	HNSC	6.08
TCGA-CQ-7068-01A	HNSC	5.97
TCGA-CV-7178-01A	HNSC	6.22
TCGA-F7-A50I-01A	HNSC	5.31
TCGA-CV-7100-01A	HNSC	5.93
TCGA-CV-7428-01A	HNSC	5.61
TCGA-CQ-6221-01A	HNSC	5.65
TCGA-BA-4076-01A	HNSC	4.91
TCGA-F7-A622-01A	HNSC	4.56
TCGA-CN-6012-01A	HNSC	6.58
TCGA-D6-6515-01A	HNSC	5.97
TCGA-UF-A7J9-01A	HNSC	6.89
TCGA-IQ-A61H-01A	HNSC	7.47
TCGA-P3-A6T8-01A	HNSC	4.38
TCGA-HD-A6HZ-01A	HNSC	5.85
TCGA-CV-7416-01A	HNSC	6.86
TCGA-CQ-5330-01A	HNSC	5.79
TCGA-DQ-5630-01A	HNSC	6.35
TCGA-CQ-6229-01A	HNSC	5
TCGA-T2-A6X2-01A	HNSC	5.3

TCGA-CN-A497-01A	HNSC	6.99
TCGA-F7-7848-01A	HNSC	6.19
TCGA-BA-5555-01A	HNSC	6.75
TCGA-F7-A61S-01A	HNSC	4.85
TCGA-F7-A61W-01A	HNSC	5.95
TCGA-CV-6943-01A	HNSC	5.22
TCGA-CQ-6225-01A	HNSC	5.05
TCGA-D6-8569-01A	HNSC	5.96
TCGA-CN-4723-01A	HNSC	6.18
TCGA-CV-6951-01A	HNSC	6.79
TCGA-CN-6020-01A	HNSC	6.03
TCGA-P3-A6T6-01A	HNSC	4.52
TCGA-UF-A71E-01A	HNSC	7
TCGA-CV-7438-01A	HNSC	5.13
TCGA-CV-6945-01A	HNSC	4.33
TCGA-CN-4736-01A	HNSC	6.08
TCGA-UF-A7JT-01A	HNSC	5.62
TCGA-CQ-A4CD-01A	HNSC	5.05
TCGA-CV-A6JN-01A	HNSC	4.07
TCGA-CR-7383-01A	HNSC	7.08
TCGA-BB-7866-01A	HNSC	6.81
TCGA-CV-5440-01A	HNSC	5.61
TCGA-CV-A468-01A	HNSC	5.14
TCGA-CR-7367-01A	HNSC	4.83
TCGA-CV-5443-01A	HNSC	5.86
TCGA-KU-A66T-01A	HNSC	6.48
TCGA-P3-A6SX-01A	HNSC	5.21
TCGA-HD-7831-01A	HNSC	5.4
TCGA-UF-A7JH-01A	HNSC	4.42
TCGA-F7-A61V-01A	HNSC	4.52
TCGA-CV-5966-01A	HNSC	5.14
TCGA-CN-4727-01A	HNSC	6.01
TCGA-CQ-A4C7-01A	HNSC	5.45
TCGA-BB-7861-01A	HNSC	5.53
TCGA-CV-5979-01A	HNSC	5.58
TCGA-CV-5970-01A	HNSC	5.24
TCGA-BA-A6DA-01A	HNSC	3.09
TCGA-CV-A45P-01A	HNSC	4.37
TCGA-CV-7103-01A	HNSC	5.76
TCGA-CV-5976-01A	HNSC	6.52
TCGA-MT-A67D-01A	HNSC	5.06
TCGA-CQ-6219-01A	HNSC	6.18
TCGA-UF-A7JV-01A	HNSC	5.59
TCGA-D6-A6EQ-01A	HNSC	4.67
TCGA-DQ-5631-01A	HNSC	5.59
TCGA-CQ-5334-01A	HNSC	6.28
TCGA-BA-A6DE-01A	HNSC	5.18
TCGA-F7-A50J-01A	HNSC	3.63
TCGA-BA-A6DJ-01A	HNSC	4.58
TCGA-UF-A7JS-01A	HNSC	3.49
TCGA-CV-5971-01A	HNSC	4.97
TCGA-BA-5559-01A	HNSC	5.88
TCGA-CN-4737-01A	HNSC	5.58
TCGA-BA-A6DB-01A	HNSC	4.91
TCGA-UF-A7JK-01A	HNSC	6.11
TCGA-HD-8634-01A	HNSC	4.36
TCGA-UF-A718-01A	HNSC	4.74
TCGA-P3-A6T3-01A	HNSC	5.08

TCGA-CN-6997-01A	HNSC	6.39
TCGA-HD-7754-01A	HNSC	7.32
TCGA-CX-A4AQ-01A	HNSC	5.13
TCGA-CV-7418-01A	HNSC	6.62
TCGA-CQ-7065-01A	HNSC	5.34
TCGA-CQ-A4CI-01A	HNSC	7.52
TCGA-QK-A6IF-01A	HNSC	4.83
TCGA-UF-A7JD-01A	HNSC	5.36
TCGA-CV-7253-01A	HNSC	6
TCGA-CQ-6228-01A	HNSC	7.76
TCGA-CR-7379-01A	HNSC	5.52
TCGA-CQ-A4CE-01A	HNSC	4.6
TCGA-CR-5243-01A	HNSC	6.65
TCGA-H7-8502-01A	HNSC	6.33
TCGA-CV-7423-01A	HNSC	5.67
TCGA-MT-A67A-01A	HNSC	4.72
TCGA-BA-7269-01A	HNSC	6.07
TCGA-IQ-7630-01A	HNSC	5.8
TCGA-CV-7254-01A	HNSC	6.39
TCGA-CV-A463-01A	HNSC	5.13
TCGA-BA-6869-01A	HNSC	6.29
TCGA-DQ-7595-01A	HNSC	7.21
TCGA-CR-7374-01A	HNSC	6.06
TCGA-CV-6948-01A	HNSC	6.67
TCGA-CV-6938-01A	HNSC	6.86
TCGA-MZ-A5BI-01A	HNSC	5.69
TCGA-HD-7917-01A	HNSC	4.95
TCGA-CV-7437-01A	HNSC	7.41
TCGA-CV-6953-01A	HNSC	3.96
TCGA-BA-5151-01A	HNSC	5.18
TCGA-T2-A6WZ-01A	HNSC	3.2
TCGA-CV-A45V-01A	HNSC	5.74
TCGA-CV-5436-01A	HNSC	4.31
TCGA-BA-4074-01A	HNSC	6.36
TCGA-CR-7395-01A	HNSC	5.61
TCGA-CR-6481-01A	HNSC	6.16
TCGA-CR-5250-01A	HNSC	5.7
TCGA-BA-A8YP-01A	HNSC	6.68
TCGA-CR-5248-01A	HNSC	5.74
TCGA-CN-A499-01A	HNSC	5.26
TCGA-MZ-A6I9-01A	HNSC	6.07
TCGA-CN-6010-01A	HNSC	7.44
TCGA-TN-A7HJ-01A	HNSC	6.16
TCGA-HD-A633-01A	HNSC	4.89
TCGA-CV-7433-01A	HNSC	5.63
TCGA-CR-6493-01A	HNSC	4.87
TCGA-D6-A6ES-01A	HNSC	2.92
TCGA-DQ-5624-01A	HNSC	5.24
TCGA-F7-8298-01A	HNSC	5.17
TCGA-CV-7180-01A	HNSC	5.24
TCGA-IQ-A61E-01A	HNSC	5.06
TCGA-IQ-A61G-01A	HNSC	5.46
TCGA-CV-A6K0-01B	HNSC	5.52
TCGA-CV-6433-01A	HNSC	5.51
TCGA-IQ-A61J-01A	HNSC	5.74
TCGA-RS-A6TO-01A	HNSC	5.4
TCGA-CQ-5323-01A	HNSC	4.88
TCGA-CV-A45Z-01A	HNSC	6.48

TCGA-CR-7401-01A	HNSC	5.26
TCGA-CQ-6223-01A	HNSC	6.4
TCGA-P3-A6T0-01A	HNSC	6.22
TCGA-HD-A6I0-01A	HNSC	4.02
TCGA-CR-7404-01A	HNSC	6.06
TCGA-D6-A6EN-01A	HNSC	4.87
TCGA-HD-8314-01A	HNSC	6.57
TCGA-CN-6019-01A	HNSC	6.93
TCGA-CV-7568-01A	HNSC	5.85
TCGA-CN-6989-01A	HNSC	6.26
TCGA-BB-7864-01A	HNSC	4.02
TCGA-D6-6823-01A	HNSC	6
TCGA-CV-6937-01A	HNSC	5.73
TCGA-CN-4735-01A	HNSC	6.02
TCGA-DQ-5629-01A	HNSC	6.96
TCGA-CV-7421-01A	HNSC	5.78
TCGA-CQ-7067-01A	HNSC	6.52
TCGA-CN-4740-01A	HNSC	5.45
TCGA-CN-4733-01A	HNSC	4.95
TCGA-CQ-6222-01A	HNSC	5.68
TCGA-CV-6941-01A	HNSC	5.99
TCGA-D6-6516-01A	HNSC	5.93
TCGA-CR-7394-01A	HNSC	5.41
TCGA-CV-6955-01A	HNSC	6.54
TCGA-D6-A6EK-01A	HNSC	4.19
TCGA-CR-7377-01A	HNSC	5.95
TCGA-BA-6868-01B	HNSC	5.08
TCGA-WA-A7GZ-01A	HNSC	5.36
TCGA-T2-A6X0-01A	HNSC	7.09
TCGA-CV-7425-01A	HNSC	5.6
TCGA-CV-7177-01A	HNSC	7.17
TCGA-CV-5441-01A	HNSC	5.5
TCGA-CV-7414-01A	HNSC	7.34
TCGA-BA-A4IH-01A	HNSC	6.19
TCGA-T3-A92M-01A	HNSC	6.08
TCGA-BA-A6DL-01A	HNSC	6.57
TCGA-CQ-A4CA-01A	HNSC	5.63
TCGA-CQ-5326-01A	HNSC	5.86
TCGA-CQ-5331-01A	HNSC	5.68
TCGA-QK-A6V9-01A	HNSC	6.16
TCGA-CV-5435-01A	HNSC	7.31
TCGA-IQ-7631-01A	HNSC	4.46
TCGA-CN-5356-01A	HNSC	5.37
TCGA-UF-A71B-01A	HNSC	7.17
TCGA-DQ-7590-01A	HNSC	5.95
TCGA-CR-6487-01A	HNSC	6.28
TCGA-CN-A642-01A	HNSC	5.72
TCGA-CV-A45O-01A	HNSC	4.2
TCGA-CV-7409-01A	HNSC	5.44
TCGA-CR-7385-01A	HNSC	5.93
TCGA-CR-6484-01A	HNSC	5.47
TCGA-CV-A6JU-01A	HNSC	6.15
TCGA-CN-A49C-01A	HNSC	6.35
TCGA-BB-4227-01A	HNSC	6.45
TCGA-CN-5369-01A	HNSC	6.21
TCGA-CV-7432-01A	HNSC	5.42
TCGA-CV-7415-01A	HNSC	5.55
TCGA-D6-6517-01A	HNSC	6.13

TCGA-H7-A6C4-01A	HNSC	4.57
TCGA-CV-6939-01A	HNSC	6.36
TCGA-CV-7243-01A	HNSC	5.8
TCGA-D6-A74Q-01A	HNSC	5.39
TCGA-C9-A480-01A	HNSC	5.04
TCGA-D6-A6EM-01A	HNSC	4.98
TCGA-F7-A623-01A	HNSC	4.9
TCGA-CN-6016-01A	HNSC	5.5
TCGA-T3-A92N-01A	HNSC	5.69
TCGA-CR-7399-01A	HNSC	5.74
TCGA-CR-6471-01A	HNSC	5.21
TCGA-CR-6488-01A	HNSC	5.7
TCGA-CQ-A4CG-01A	HNSC	6.65
TCGA-BB-7862-01A	HNSC	6.52
TCGA-CV-6959-01A	HNSC	5.5
TCGA-HD-7753-01A	HNSC	5.01
TCGA-CR-6473-01A	HNSC	5.26
TCGA-H7-8501-01A	HNSC	6.11
TCGA-H7-7774-01A	HNSC	6.56
TCGA-CN-6011-01A	HNSC	5.61
TCGA-BB-A5HU-01A	HNSC	5.71
TCGA-CR-7382-01A	HNSC	6.32
TCGA-UP-A6WW-01A	HNSC	6.16
TCGA-CR-7371-01A	HNSC	6.27
TCGA-CN-4741-01A	HNSC	5.85
TCGA-CR-7368-01A	HNSC	6.79
TCGA-CR-7373-01A	HNSC	4.51
TCGA-BB-4217-01A	HNSC	5.16
TCGA-QK-A64Z-01A	HNSC	6.28
TCGA-CV-7235-01A	HNSC	4.91
TCGA-CN-6024-01A	HNSC	6.11
TCGA-CN-4730-01A	HNSC	4.59
TCGA-UF-A7JF-01A	HNSC	6.19
TCGA-HD-7832-01A	HNSC	5.33
TCGA-CV-6933-01A	HNSC	6.56
TCGA-CV-A45X-01A	HNSC	4.41
TCGA-CQ-7063-01A	HNSC	5.03
TCGA-CV-7236-01A	HNSC	5.86
TCGA-CN-6995-01A	HNSC	5.43
TCGA-CV-A6K1-01A	HNSC	4.31
TCGA-CN-6998-01A	HNSC	4.43
TCGA-QK-A6VC-01A	HNSC	6.45
TCGA-CN-6988-01A	HNSC	6.67
TCGA-DQ-7589-01A	HNSC	7.48
TCGA-QK-A8Z7-01A	HNSC	5.86
TCGA-CQ-5325-01A	HNSC	5.63
TCGA-CN-4731-01A	HNSC	5.6
TCGA-CV-7247-01A	HNSC	6.01
TCGA-BA-4077-01B	HNSC	6.64
TCGA-CN-5373-01A	HNSC	5.59
TCGA-BB-8596-01A	HNSC	6.75
TCGA-CV-7099-01A	HNSC	6.45
TCGA-CV-7255-01A	HNSC	5.14
TCGA-CR-7380-01A	HNSC	5.73
TCGA-CN-5367-01A	HNSC	6.44
TCGA-CQ-6227-01A	HNSC	5.2
TCGA-DQ-7594-01A	HNSC	6.18
TCGA-CN-5370-01A	HNSC	6.18

TCGA-CV-7434-01A	HNSC	5.4
TCGA-CN-A49A-01A	HNSC	5.51
TCGA-CN-6023-01A	HNSC	7.67
TCGA-CV-7440-01A	HNSC	5.02
TCGA-CV-7411-01A	HNSC	5.44
TCGA-CN-A6UY-01A	HNSC	6.05
TCGA-CV-5431-01A	HNSC	4.12
TCGA-CN-4728-01A	HNSC	5.5
TCGA-CV-A6JZ-01A	HNSC	6.39
TCGA-CN-A63W-01A	HNSC	4.53
TCGA-CN-6992-01A	HNSC	7.35
TCGA-BA-A4IG-01A	HNSC	5.66
TCGA-CQ-A4C6-01A	HNSC	5.41
TCGA-CV-5444-01A	HNSC	5.81
TCGA-CN-6018-01A	HNSC	4.92
TCGA-CN-6994-01A	HNSC	5.7
TCGA-CQ-6218-01A	HNSC	4.09
TCGA-IQ-7632-01A	HNSC	5.11
TCGA-CR-7364-01A	HNSC	6.48
TCGA-CV-A465-01A	HNSC	4.64
TCGA-CN-A641-01A	HNSC	4.71
TCGA-CV-A6JT-01A	HNSC	4.01
TCGA-CN-6013-01A	HNSC	6.51
TCGA-CV-6441-01A	HNSC	6.37
TCGA-BA-5557-01A	HNSC	4.55
TCGA-CN-5365-01A	HNSC	5.47
TCGA-CN-4726-01A	HNSC	5.46
TCGA-CV-A45Q-01A	HNSC	2.44
TCGA-CR-6491-01A	HNSC	4.78
TCGA-CQ-5332-01A	HNSC	6.54
TCGA-UF-A7JJ-01A	HNSC	5.3
TCGA-CR-5247-01A	HNSC	5.53
TCGA-BA-4075-01A	HNSC	6.01
TCGA-C9-A47Z-01A	HNSC	5
TCGA-CQ-7071-01A	HNSC	5.27
TCGA-CR-7391-01A	HNSC	5.18
TCGA-CV-A45T-01A	HNSC	4.59
TCGA-QK-A6VB-01A	HNSC	7.82
TCGA-CV-7435-01A	HNSC	6
TCGA-CN-5360-01A	HNSC	6.03
TCGA-CQ-A4C9-01A	HNSC	6.89
TCGA-QK-AA3J-01A	HNSC	7.4
TCGA-BB-4224-01A	HNSC	4.98
TCGA-BA-5558-01A	HNSC	4.68
TCGA-DQ-7593-01A	HNSC	5.19
TCGA-CN-A49B-01A	HNSC	6.21
TCGA-BA-6870-01A	HNSC	5.57
TCGA-D6-6824-01A	HNSC	6.64
TCGA-IQ-A6SH-01A	HNSC	4.86
TCGA-CN-A6V7-01A	HNSC	6.38
TCGA-BA-5556-01A	HNSC	5.85
TCGA-MT-A51W-01A	HNSC	4.65
TCGA-HD-A4C1-01A	HNSC	6.73
TCGA-CR-7390-01A	HNSC	6.01
TCGA-D6-6826-01A	HNSC	6.1
TCGA-CX-7085-01A	HNSC	4.9
TCGA-BA-6872-01A	HNSC	5.25
TCGA-CQ-5329-01A	HNSC	6.08

TCGA-P3-A6SW-01A	HNSC	6.31
TCGA-QK-A8ZB-01A	HNSC	5.28
TCGA-CQ-5333-01A	HNSC	6.09
TCGA-QK-AA3K-01A	HNSC	5.05
TCGA-CV-6935-01A	HNSC	8.16
TCGA-CN-6017-01A	HNSC	4.58
TCGA-CV-7101-01A	HNSC	6.2
TCGA-BA-A4II-01A	HNSC	6.25
TCGA-CV-7252-01A	HNSC	5.16
TCGA-CN-4729-01A	HNSC	5.5
TCGA-D6-6827-01A	HNSC	4.73
TCGA-CV-5439-01A	HNSC	6.68
TCGA-BB-A5HY-01A	HNSC	5.9
TCGA-CV-A461-01A	HNSC	6.64
TCGA-P3-A5QF-01A	HNSC	7.19
TCGA-CN-5355-01A	HNSC	5.09
TCGA-D6-6825-01A	HNSC	5.99
TCGA-CV-6961-01A	HNSC	6.54
TCGA-CN-4742-01A	HNSC	5.6
TCGA-CN-A498-01A	HNSC	6.17
TCGA-CN-6996-01A	HNSC	5.75
TCGA-CV-A45Y-01A	HNSC	5.42
TCGA-QK-A652-01A	HNSC	5.4
TCGA-P3-A5Q6-01A	HNSC	5.44
TCGA-CV-7242-01A	HNSC	5.83
TCGA-F7-A624-01A	HNSC	5.74
TCGA-TN-A7HL-01A	HNSC	5.21
TCGA-CV-7102-01A	HNSC	5.83
TCGA-MT-A67F-01A	HNSC	5.03
TCGA-CN-A6V6-01A	HNSC	5.54
TCGA-CN-A63U-01A	HNSC	7.13
TCGA-CN-5363-01A	HNSC	5.88
TCGA-BB-8601-01A	HNSC	7.94
TCGA-D6-A6EP-01A	HNSC	4.21
TCGA-CV-6950-01A	HNSC	6.23
TCGA-UF-A71D-01A	HNSC	5.09
TCGA-DQ-7591-01A	HNSC	6.62
TCGA-CX-7219-01A	HNSC	6.63
TCGA-CR-7386-01A	HNSC	6.21
TCGA-P3-A5Q5-01A	HNSC	6.34
TCGA-CV-7422-01A	HNSC	7.08
TCGA-BB-7863-01A	HNSC	5.43
TCGA-CV-7091-01A	HNSC	5.11
TCGA-QK-A6II-01A	HNSC	3.93
TCGA-CN-4725-01A	HNSC	5.62
TCGA-QK-A6IG-01A	HNSC	5.17
TCGA-BA-5152-01A	HNSC	5.64
TCGA-CV-5442-01A	HNSC	4.95
TCGA-UF-A71A-01A	HNSC	6.67
TCGA-CR-7372-01A	HNSC	5.37
TCGA-D6-A6EO-01A	HNSC	6.8
TCGA-CV-A6JD-01A	HNSC	5.28
TCGA-CR-7392-01A	HNSC	4.95
TCGA-CR-6478-01A	HNSC	5.65
TCGA-WA-A7H4-01A	HNSC	5.2
TCGA-D6-A4Z9-01A	HNSC	5.56
TCGA-H7-A76A-01A	HNSC	4.88
TCGA-CR-7389-01A	HNSC	5.88

TCGA-CQ-5327-01A	HNSC	5.06
TCGA-HD-7229-01A	HNSC	5.5
TCGA-D6-8568-01A	HNSC	4.94
TCGA-CV-6954-01A	HNSC	5.43
TCGA-CV-7089-01A	HNSC	5.29
TCGA-CV-6003-01A	HNSC	6.37
TCGA-CN-5361-01A	HNSC	6.4
TCGA-CV-5977-01A	HNSC	4.45
TCGA-UF-A7JO-01A	HNSC	8.15
TCGA-CR-7388-01A	HNSC	5.83
TCGA-F7-8489-01A	HNSC	5.99
TCGA-CV-7248-01A	HNSC	6.2
TCGA-P3-A6T5-01A	HNSC	5.72
TCGA-CV-7429-01A	HNSC	7.27
TCGA-CV-6934-01A	HNSC	5.74
TCGA-BB-4223-01A	HNSC	5.44
TCGA-CX-7082-01A	HNSC	7.1
TCGA-RS-A6TP-01A	HNSC	6.23
TCGA-KU-A66S-01A	HNSC	5.46
TCGA-CV-7090-01A	HNSC	5.8
TCGA-CQ-7069-01A	HNSC	5.31
TCGA-CV-A6JY-01A	HNSC	5.04
TCGA-MZ-A7D7-01A	HNSC	5.61
TCGA-CR-6472-01A	HNSC	5.9
TCGA-CV-7410-01A	HNSC	5.9
TCGA-CV-7250-01A	HNSC	6.76
TCGA-CR-7393-01A	HNSC	5.45
TCGA-HD-8224-01A	HNSC	7.17
TCGA-CN-A6V1-01A	HNSC	5.12
TCGA-BB-A6UM-01A	HNSC	6.15
TCGA-CR-6482-01A	HNSC	5.38
TCGA-DQ-5625-01A	HNSC	6.02
TCGA-CV-7261-01A	HNSC	5.54
TCGA-UF-A7JC-01A	HNSC	5.85
TCGA-CV-7427-01A	HNSC	6.31
TCGA-CQ-7072-01A	HNSC	4.94
TCGA-BB-4228-01A	HNSC	5.24
TCGA-BA-6871-01A	HNSC	5.64
TCGA-BA-A6DD-01A	HNSC	5.43
TCGA-CV-7430-01A	HNSC	6.15
TCGA-BA-5149-01A	HNSC	5.17
TCGA-CV-A6JE-01A	HNSC	3.47
TCGA-BB-A6UO-01A	HNSC	4.8
TCGA-CV-A460-01A	HNSC	6.26
TCGA-BB-7871-01A	HNSC	7.41
TCGA-CR-7365-01A	HNSC	5.59
TCGA-QK-A8Z8-01A	HNSC	6.69
TCGA-CR-6492-01A	HNSC	5.6
TCGA-CR-7397-01A	HNSC	5.2
TCGA-UF-A719-01A	HNSC	7.09
TCGA-BA-6873-01A	HNSC	5.97
TCGA-CR-6477-01A	HNSC	5.77
TCGA-CV-7446-01A	HNSC	7.04
TCGA-T2-A6WX-01A	HNSC	5.49
TCGA-CV-7238-01A	HNSC	4.83
TCGA-QK-A8Z9-01B	HNSC	6.22
TCGA-DQ-7588-01A	HNSC	6.29
TCGA-CV-A45U-01A	HNSC	6.02

TCGA-CV-6940-01A	HNSC	7.67
TCGA-CN-4734-01A	HNSC	5.15
TCGA-IQ-A6SG-01A	HNSC	4.78
TCGA-CN-5374-01A	HNSC	5.07
TCGA-CN-A63V-01A	HNSC	5.42
TCGA-CV-7095-01A	HNSC	5.07
TCGA-IQ-A61O-01A	HNSC	5.25
TCGA-P3-A6T4-01A	HNSC	5.48
TCGA-DQ-7592-01A	HNSC	5.82
TCGA-CR-5249-01A	HNSC	6.14
TCGA-CQ-6224-01A	HNSC	6
TCGA-CV-5978-01A	HNSC	7.64
TCGA-CV-7183-01A	HNSC	4.68
TCGA-BA-4078-01A	HNSC	6.38
TCGA-QK-A6IJ-01A	HNSC	4.17
TCGA-CV-5432-01A	HNSC	6.16
TCGA-CR-7398-01A	HNSC	6.28
TCGA-CV-5973-01A	HNSC	4.03
TCGA-CV-7407-01A	HNSC	7.01
TCGA-CV-A45R-01A	HNSC	5.24
TCGA-KU-A6H8-01A	HNSC	5.25
TCGA-CV-A6JO-01B	HNSC	5.13
TCGA-BA-A6DI-01A	HNSC	5.88
TCGA-BB-7870-01A	HNSC	5.57
TCGA-CV-5434-01A	HNSC	7
TCGA-D6-A4ZB-01A	HNSC	5.12
TCGA-CR-6470-01A	HNSC	6.29
TCGA-F7-A620-01A	HNSC	4.49
TCGA-KU-A6H7-01A	HNSC	5.51
TCGA-CV-7413-01A	HNSC	5
TCGA-MT-A7BN-01A	HNSC	4.79
TCGA-QK-A6IH-01A	HNSC	4.34
TCGA-CV-A6JM-01A	HNSC	3.74
TCGA-DQ-7596-01A	HNSC	7.17
TCGA-CN-5364-01A	HNSC	6.32
TCGA-KL-8325-01A	KICH	5.3
TCGA-KO-8403-01A	KICH	3.75
TCGA-KL-8341-01A	KICH	6.17
TCGA-KL-8336-01A	KICH	5.21
TCGA-KN-8421-01A	KICH	3.22
TCGA-KN-8422-01A	KICH	3.92
TCGA-KL-8345-01A	KICH	3.91
TCGA-KN-8428-01A	KICH	4.37
TCGA-KM-8476-01A	KICH	2.13
TCGA-KL-8337-01A	KICH	3.2
TCGA-KN-8423-01A	KICH	3.76
TCGA-KL-8340-01A	KICH	3.85
TCGA-KN-8429-01A	KICH	4.1
TCGA-KM-8442-01A	KICH	3.88
TCGA-KL-8328-01A	KICH	3.77
TCGA-KM-8439-01A	KICH	4.07
TCGA-KL-8333-01A	KICH	3.97
TCGA-KO-8407-01A	KICH	3.63
TCGA-KO-8417-01A	KICH	3.99
TCGA-KO-8404-01A	KICH	5.46
TCGA-KL-8330-01A	KICH	3.46
TCGA-KL-8327-01A	KICH	1.4
TCGA-KO-8416-01A	KICH	2.96

TCGA-KL-8326-01A	KICH	1.78
TCGA-KL-8324-01A	KICH	3.11
TCGA-KM-8438-01A	KICH	3.4
TCGA-KO-8413-01A	KICH	3.07
TCGA-KM-8443-01A	KICH	3.66
TCGA-KL-8338-01A	KICH	3.75
TCGA-KL-8332-01A	KICH	3.52
TCGA-KN-8431-01A	KICH	2.12
TCGA-KM-8639-01A	KICH	5.36
TCGA-KN-8424-01A	KICH	5.13
TCGA-KL-8331-01A	KICH	4.4
TCGA-KN-8427-01A	KICH	5.13
TCGA-KN-8430-01A	KICH	4.51
TCGA-KO-8408-01A	KICH	3.81
TCGA-KN-8433-01A	KICH	3.88
TCGA-KL-8346-01A	KICH	4.12
TCGA-KL-8344-01A	KICH	3.84
TCGA-KO-8414-01A	KICH	3.37
TCGA-KN-8418-01A	KICH	4
TCGA-KL-8323-01A	KICH	4.16
TCGA-KM-8440-01A	KICH	2.97
TCGA-KO-8406-01A	KICH	3.62
TCGA-KM-8477-01A	KICH	2.69
TCGA-KO-8410-01A	KICH	4.69
TCGA-KL-8342-01A	KICH	4.23
TCGA-KN-8434-01A	KICH	4.57
TCGA-KO-8411-01A	KICH	3.89
TCGA-KN-8437-01A	KICH	5.01
TCGA-KO-8415-01A	KICH	4.2
TCGA-KL-8343-01A	KICH	4.71
TCGA-KL-8335-01A	KICH	3.6
TCGA-KL-8339-01A	KICH	5.07
TCGA-KL-8334-01A	KICH	3.87
TCGA-KN-8432-01A	KICH	4
TCGA-KN-8426-01A	KICH	3.66
TCGA-KN-8425-01A	KICH	4.15
TCGA-KN-8419-01A	KICH	3.15
TCGA-KO-8405-01A	KICH	4.06
TCGA-KL-8329-01A	KICH	4.25
TCGA-KN-8436-01A	KICH	4.57
TCGA-KN-8435-01A	KICH	5
TCGA-KM-8441-01A	KICH	5.26
TCGA-KO-8409-01A	KICH	1.88
TCGA-BP-4756-01A	KIRC	5.55
TCGA-BP-4999-01A	KIRC	5.18
TCGA-CJ-5684-01A	KIRC	5.31
TCGA-B0-5692-01A	KIRC	4.53
TCGA-BP-4988-01A	KIRC	5.17
TCGA-BP-5010-01A	KIRC	4.18
TCGA-CZ-5451-01A	KIRC	5.2
TCGA-CJ-4899-01A	KIRC	5.89
TCGA-CW-5590-01A	KIRC	5.67
TCGA-B8-5545-01A	KIRC	6.29
TCGA-CJ-4902-01A	KIRC	5.3
TCGA-B0-5102-01A	KIRC	4.98
TCGA-BP-4801-01A	KIRC	6.15
TCGA-CZ-5460-01A	KIRC	6.01
TCGA-BP-4347-01A	KIRC	5.39

TCGA-CJ-4635-01A	KIRC	5.67
TCGA-A3-A6NJ-01A	KIRC	4.91
TCGA-BP-4962-01A	KIRC	5.34
TCGA-B4-5834-01A	KIRC	5.51
TCGA-BP-4963-01A	KIRC	5.72
TCGA-B0-5116-01A	KIRC	4.76
TCGA-B2-4101-01A	KIRC	5.57
TCGA-B0-5707-01A	KIRC	4.89
TCGA-B0-5108-01A	KIRC	5.91
TCGA-A3-3313-01A	KIRC	5.7
TCGA-B0-4843-01A	KIRC	4.73
TCGA-CW-5585-01A	KIRC	5.72
TCGA-B8-A8YJ-01A	KIRC	5.74
TCGA-B8-5164-01A	KIRC	5.92
TCGA-CJ-4637-01A	KIRC	5.76
TCGA-B0-4841-01A	KIRC	2.99
TCGA-CJ-4870-01A	KIRC	4.52
TCGA-B0-5400-01A	KIRC	5.84
TCGA-BP-4784-01A	KIRC	5.54
TCGA-A3-3363-01A	KIRC	5.41
TCGA-B8-4621-01A	KIRC	5.22
TCGA-B0-5690-01A	KIRC	5.26
TCGA-B0-5702-01A	KIRC	3.28
TCGA-B0-5083-01A	KIRC	4.71
TCGA-B0-4688-01A	KIRC	6.09
TCGA-BP-4781-01A	KIRC	6.1
TCGA-B4-5832-01A	KIRC	7.84
TCGA-BP-4766-01A	KIRC	5.87
TCGA-B0-5121-01A	KIRC	3.91
TCGA-CJ-4872-01A	KIRC	5.61
TCGA-B2-5633-01B	KIRC	5.81
TCGA-A3-3317-01A	KIRC	5.88
TCGA-CZ-4853-01A	KIRC	5.1
TCGA-BP-4991-01A	KIRC	5.86
TCGA-B0-4691-01A	KIRC	4.55
TCGA-B0-5713-01A	KIRC	5.19
TCGA-CJ-6032-01A	KIRC	5.88
TCGA-B8-4146-01B	KIRC	6.89
TCGA-AK-3445-01A	KIRC	5.57
TCGA-A3-3335-01A	KIRC	5.41
TCGA-BP-4342-01A	KIRC	5.99
TCGA-B8-5163-01A	KIRC	5.98
TCGA-B0-5092-01A	KIRC	4.26
TCGA-B0-4703-01A	KIRC	5.86
TCGA-B8-5162-01A	KIRC	6.37
TCGA-CJ-5677-01A	KIRC	5.2
TCGA-BP-4797-01A	KIRC	5.33
TCGA-B0-4693-01A	KIRC	5.43
TCGA-BP-4985-01A	KIRC	4.86
TCGA-6D-AA2E-01A	KIRC	5.92
TCGA-BP-4770-01A	KIRC	5.19
TCGA-B0-5693-01A	KIRC	5.03
TCGA-BP-4332-01A	KIRC	5.31
TCGA-A3-3382-01A	KIRC	6.22
TCGA-BP-4334-01A	KIRC	3
TCGA-BP-4971-01A	KIRC	4.46
TCGA-AK-3428-01A	KIRC	5.05
TCGA-A3-3387-01A	KIRC	5.61

TCGA-BP-4327-01A	KIRC	4.9
TCGA-A3-3306-01A	KIRC	5.37
TCGA-B0-5100-01A	KIRC	4.26
TCGA-BP-4995-01A	KIRC	5.61
TCGA-CJ-4642-01B	KIRC	4.57
TCGA-B2-5641-01A	KIRC	5.12
TCGA-B0-5117-01A	KIRC	3.81
TCGA-CJ-4912-01A	KIRC	3.79
TCGA-CZ-5982-01A	KIRC	5.3
TCGA-MW-A4EC-01A	KIRC	5.31
TCGA-A3-3325-01A	KIRC	5.2
TCGA-CJ-4886-01A	KIRC	5.57
TCGA-B2-4102-01A	KIRC	5.42
TCGA-B0-4699-01A	KIRC	5.86
TCGA-B0-5120-01A	KIRC	5.35
TCGA-B0-4824-01A	KIRC	4.85
TCGA-B0-5077-01A	KIRC	5.21
TCGA-CJ-6033-01A	KIRC	6.18
TCGA-A3-3357-01A	KIRC	5.46
TCGA-A3-3320-01A	KIRC	5.22
TCGA-CJ-4916-01A	KIRC	4.63
TCGA-BP-4346-01A	KIRC	4.96
TCGA-B0-5712-01A	KIRC	4.57
TCGA-CW-5589-01A	KIRC	5.65
TCGA-EU-5906-01A	KIRC	5.7
TCGA-MM-A564-01A	KIRC	4.21
TCGA-BP-4983-01A	KIRC	6.13
TCGA-CZ-4860-01A	KIRC	7.52
TCGA-BP-4765-01A	KIRC	5.1
TCGA-CZ-5455-01A	KIRC	5.86
TCGA-B0-4690-01A	KIRC	7.21
TCGA-CZ-5988-01A	KIRC	6.45
TCGA-B8-A7U6-01A	KIRC	5.11
TCGA-CJ-6031-01A	KIRC	5.87
TCGA-BP-4355-01A	KIRC	4.31
TCGA-B0-5110-01A	KIRC	5.31
TCGA-DV-5574-01A	KIRC	6.06
TCGA-BP-4176-01A	KIRC	6.14
TCGA-B0-5695-01A	KIRC	5.06
TCGA-DV-5569-01A	KIRC	4.82
TCGA-CZ-5454-01A	KIRC	6.24
TCGA-BP-5183-01A	KIRC	4.94
TCGA-BP-5168-01A	KIRC	4.89
TCGA-B8-4619-01A	KIRC	4.39
TCGA-A3-3378-01A	KIRC	6.21
TCGA-BP-5178-01A	KIRC	5.65
TCGA-B8-5546-01A	KIRC	5.02
TCGA-CZ-5469-01A	KIRC	4.85
TCGA-CJ-4892-01A	KIRC	4.94
TCGA-CJ-4875-01A	KIRC	4.19
TCGA-BP-4326-01A	KIRC	5.4
TCGA-CJ-4920-01A	KIRC	5.45
TCGA-CZ-4863-01A	KIRC	5.47
TCGA-CJ-6030-01A	KIRC	5.69
TCGA-CJ-4634-01A	KIRC	5.8
TCGA-CZ-5459-01A	KIRC	5.12
TCGA-BP-4341-01A	KIRC	4.12
TCGA-CZ-5468-01A	KIRC	6.44

TCGA-BP-5176-01A	KIRC	4.87
TCGA-CZ-5986-01A	KIRC	5.65
TCGA-A3-3346-01A	KIRC	7.04
TCGA-B0-5402-01A	KIRC	5.68
TCGA-CJ-4871-01A	KIRC	5.58
TCGA-BP-4989-01A	KIRC	4.55
TCGA-AK-3447-01A	KIRC	2.54
TCGA-A3-3359-01A	KIRC	5.15
TCGA-B0-5084-01A	KIRC	5.05
TCGA-CJ-5675-01A	KIRC	5.3
TCGA-B0-4845-01A	KIRC	4.61
TCGA-B0-4945-01A	KIRC	5.11
TCGA-A3-3351-01A	KIRC	5.26
TCGA-B8-A54G-01A	KIRC	5.05
TCGA-BP-4961-01A	KIRC	5.61
TCGA-T7-A92I-01A	KIRC	5.98
TCGA-BP-4974-01A	KIRC	5.35
TCGA-BP-5001-01A	KIRC	4.12
TCGA-BP-5199-01A	KIRC	5.34
TCGA-B0-4846-01A	KIRC	5.32
TCGA-B0-5697-01A	KIRC	4.91
TCGA-BP-5185-01A	KIRC	2.68
TCGA-A3-A8OX-01A	KIRC	6.01
TCGA-CZ-5462-01A	KIRC	5.55
TCGA-A3-3323-01A	KIRC	5.81
TCGA-CJ-5689-01A	KIRC	5.78
TCGA-BP-4965-01A	KIRC	5.84
TCGA-BP-5173-01A	KIRC	5.3
TCGA-CJ-4641-01A	KIRC	5.64
TCGA-BP-4981-01A	KIRC	3.31
TCGA-CZ-5457-01A	KIRC	6
TCGA-CJ-4643-01A	KIRC	5.05
TCGA-B0-5096-01A	KIRC	6.43
TCGA-CJ-4901-01A	KIRC	5.73
TCGA-DV-5573-01A	KIRC	5.46
TCGA-A3-3362-01A	KIRC	5.36
TCGA-B0-5699-01A	KIRC	5.36
TCGA-B0-5709-01A	KIRC	5.57
TCGA-BP-4970-01A	KIRC	5.55
TCGA-AK-3443-01A	KIRC	3.49
TCGA-A3-3343-01A	KIRC	5.05
TCGA-BP-4964-01A	KIRC	5.26
TCGA-CZ-4857-01A	KIRC	6.03
TCGA-CJ-4894-01A	KIRC	5.4
TCGA-CJ-4891-01A	KIRC	5.23
TCGA-CZ-4859-01A	KIRC	5.38
TCGA-BP-4769-01A	KIRC	5.38
TCGA-AK-3458-01A	KIRC	4.97
TCGA-CJ-4893-01A	KIRC	5.65
TCGA-BP-4354-01A	KIRC	7.51
TCGA-B0-5698-01A	KIRC	4.53
TCGA-B8-4143-01A	KIRC	5.55
TCGA-CJ-4876-01A	KIRC	4.54
TCGA-BP-4161-01A	KIRC	6.33
TCGA-CZ-5458-01A	KIRC	5.15
TCGA-BP-4763-01A	KIRC	6.46
TCGA-CZ-5984-01A	KIRC	5.72
TCGA-B0-4834-01A	KIRC	3.03

TCGA-B8-A54K-01A	KIRC	4.48
TCGA-CJ-4895-01A	KIRC	5.69
TCGA-BP-5191-01A	KIRC	3.51
TCGA-AK-3427-01A	KIRC	4.98
TCGA-BP-5008-01A	KIRC	5.04
TCGA-B8-5549-01A	KIRC	5.16
TCGA-BP-4977-01A	KIRC	5.3
TCGA-AK-3426-01A	KIRC	4.79
TCGA-B4-5377-01A	KIRC	5.58
TCGA-BP-5195-01A	KIRC	5.44
TCGA-BP-4775-01A	KIRC	5.83
TCGA-B8-5165-01A	KIRC	5.98
TCGA-A3-3358-01A	KIRC	5.81
TCGA-B8-A54D-01A	KIRC	4.95
TCGA-B8-4153-01B	KIRC	4.3
TCGA-B8-5159-01A	KIRC	5.69
TCGA-DV-5567-01A	KIRC	4.71
TCGA-A3-A8OW-01A	KIRC	5.19
TCGA-B0-5109-01A	KIRC	4.96
TCGA-A3-A8CQ-01A	KIRC	5.74
TCGA-B0-4822-01A	KIRC	4.46
TCGA-CZ-4862-01A	KIRC	5.83
TCGA-B8-4622-01A	KIRC	5.45
TCGA-BP-4164-01A	KIRC	5.05
TCGA-BP-4776-01A	KIRC	3.94
TCGA-BP-5006-01A	KIRC	4.86
TCGA-B0-5710-01A	KIRC	5.36
TCGA-BP-5180-01A	KIRC	5.08
TCGA-CJ-4897-01A	KIRC	5.71
TCGA-BP-4986-01A	KIRC	5.88
TCGA-BP-4973-01A	KIRC	4.86
TCGA-B0-4823-01A	KIRC	5.82
TCGA-BP-4968-01A	KIRC	5.27
TCGA-CZ-4861-01A	KIRC	6.45
TCGA-AK-3444-01A	KIRC	5.67
TCGA-A3-3352-01A	KIRC	5.64
TCGA-CJ-4868-01A	KIRC	5.83
TCGA-EU-5907-01A	KIRC	6.4
TCGA-A3-3326-01A	KIRC	5.05
TCGA-A3-3373-01A	KIRC	5.18
TCGA-B0-4842-01A	KIRC	4.27
TCGA-CW-5581-01A	KIRC	5.74
TCGA-BP-4337-01A	KIRC	5.04
TCGA-CZ-4854-01A	KIRC	5.76
TCGA-B0-5097-01A	KIRC	6.65
TCGA-BP-4338-01A	KIRC	6.77
TCGA-A3-3324-01A	KIRC	5.21
TCGA-BP-5009-01A	KIRC	4.56
TCGA-CJ-5682-01A	KIRC	5.07
TCGA-A3-A6NL-01A	KIRC	5.32
TCGA-B8-5550-01A	KIRC	6.41
TCGA-B2-5635-01A	KIRC	5.45
TCGA-CJ-4885-01A	KIRC	4.88
TCGA-B0-4811-01A	KIRC	3.58
TCGA-CZ-5453-01A	KIRC	4.11
TCGA-B4-5836-01A	KIRC	5.76
TCGA-B8-A54F-01A	KIRC	5.36
TCGA-B0-5085-01A	KIRC	2.94

TCGA-B2-3924-01A	KIRC	4.92
TCGA-B4-5378-01A	KIRC	5.19
TCGA-BP-4787-01A	KIRC	6.16
TCGA-AK-3456-01A	KIRC	5.23
TCGA-B0-4707-01A	KIRC	4.38
TCGA-BP-5181-01A	KIRC	5.28
TCGA-CW-6090-01A	KIRC	6.26
TCGA-B8-A54E-01A	KIRC	7.02
TCGA-B0-5703-01A	KIRC	4.56
TCGA-CJ-4873-01A	KIRC	4.69
TCGA-CW-5587-01A	KIRC	6.13
TCGA-BP-5194-01A	KIRC	5.64
TCGA-BP-4993-01A	KIRC	4.62
TCGA-B0-5705-01A	KIRC	5.16
TCGA-A3-3370-01A	KIRC	5.23
TCGA-BP-5198-01A	KIRC	5.81
TCGA-CJ-4644-01A	KIRC	6.05
TCGA-DV-5576-01A	KIRC	4.82
TCGA-AK-3436-01A	KIRC	5.65
TCGA-BP-5202-01A	KIRC	5.16
TCGA-AK-3429-01A	KIRC	5.73
TCGA-BP-4351-01A	KIRC	5.32
TCGA-B0-4817-01A	KIRC	5.07
TCGA-BP-4987-01A	KIRC	5.52
TCGA-BP-4774-01A	KIRC	4.43
TCGA-MM-A84U-01A	KIRC	3.85
TCGA-BP-4325-01A	KIRC	5.45
TCGA-A3-3331-01A	KIRC	5.21
TCGA-B4-5843-01A	KIRC	6.06
TCGA-B4-5844-01A	KIRC	5.23
TCGA-CW-6087-01A	KIRC	7.02
TCGA-BP-4177-01A	KIRC	4.66
TCGA-B0-4714-01A	KIRC	4.32
TCGA-B0-4814-01A	KIRC	5.02
TCGA-A3-3385-01A	KIRC	4.88
TCGA-CJ-4890-01A	KIRC	6.09
TCGA-CZ-5985-01A	KIRC	5.68
TCGA-B0-5081-01A	KIRC	4.66
TCGA-CJ-4882-01A	KIRC	5.1
TCGA-B2-4098-01A	KIRC	5.42
TCGA-B0-4819-01A	KIRC	4.76
TCGA-DV-5566-01A	KIRC	5.17
TCGA-BP-4760-01A	KIRC	5.09
TCGA-B2-5635-01B	KIRC	5.2
TCGA-BP-4173-01A	KIRC	5.46
TCGA-BP-5192-01A	KIRC	5.04
TCGA-AK-3460-01A	KIRC	4.95
TCGA-CJ-4903-01A	KIRC	5.24
TCGA-B0-4818-01A	KIRC	4.38
TCGA-CW-6097-01A	KIRC	6.05
TCGA-BP-4343-01A	KIRC	6.81
TCGA-CZ-4864-01A	KIRC	5.03
TCGA-B0-4852-01A	KIRC	5.24
TCGA-CJ-4874-01A	KIRC	5.15
TCGA-CJ-5678-01A	KIRC	5.19
TCGA-B0-4701-01A	KIRC	5.13
TCGA-B2-5633-01A	KIRC	5.86
TCGA-CW-5580-01A	KIRC	5.67

TCGA-BP-4994-01A	KIRC	5.33
TCGA-BP-4174-01A	KIRC	6.1
TCGA-BP-4782-01A	KIRC	5.84
TCGA-CZ-5463-01A	KIRC	5.51
TCGA-CJ-5671-01A	KIRC	6.21
TCGA-B0-4844-01A	KIRC	5.52
TCGA-CJ-4884-01A	KIRC	4.88
TCGA-BP-4959-01A	KIRC	5.6
TCGA-CZ-5466-01A	KIRC	5.15
TCGA-B0-5700-01A	KIRC	4.46
TCGA-BP-4344-01A	KIRC	5.23
TCGA-B0-4698-01A	KIRC	6.12
TCGA-B0-4836-01A	KIRC	6.13
TCGA-BP-4998-01A	KIRC	5.57
TCGA-BP-4329-01A	KIRC	5.55
TCGA-CJ-5680-01A	KIRC	4.7
TCGA-BP-4777-01A	KIRC	5.35
TCGA-CW-5583-01A	KIRC	5.53
TCGA-B4-5838-01A	KIRC	6.55
TCGA-B0-5711-01A	KIRC	5.45
TCGA-CJ-4878-01A	KIRC	3.51
TCGA-B8-5553-01A	KIRC	5.43
TCGA-B0-5094-01A	KIRC	5.82
TCGA-B8-A54I-01A	KIRC	4.45
TCGA-B0-4813-01A	KIRC	4.94
TCGA-CJ-4905-01A	KIRC	5.44
TCGA-B0-4821-01A	KIRC	4.26
TCGA-A3-3380-01A	KIRC	4.98
TCGA-BP-4795-01A	KIRC	5.61
TCGA-CZ-5461-01A	KIRC	5.73
TCGA-CJ-4907-01A	KIRC	5.49
TCGA-BP-4331-01A	KIRC	5.45
TCGA-BP-5000-01A	KIRC	4.22
TCGA-BP-4969-01A	KIRC	5.46
TCGA-EU-5904-01A	KIRC	5.01
TCGA-A3-3308-01A	KIRC	5.83
TCGA-DV-A4W0-01A	KIRC	4.94
TCGA-CZ-5456-01A	KIRC	6.83
TCGA-B0-4816-01A	KIRC	4.75
TCGA-BP-5004-01A	KIRC	5.43
TCGA-B0-4810-01A	KIRC	6.17
TCGA-BP-4758-01A	KIRC	3.9
TCGA-BP-4352-01A	KIRC	5
TCGA-B2-3923-01A	KIRC	4.22
TCGA-CJ-5686-01A	KIRC	5.63
TCGA-GK-A6C7-01A	KIRC	5.24
TCGA-AK-3434-01A	KIRC	4.7
TCGA-B8-4148-01A	KIRC	5.12
TCGA-BP-4976-01A	KIRC	5.49
TCGA-BP-5189-01A	KIRC	5.55
TCGA-BP-4972-01A	KIRC	6.17
TCGA-BP-5175-01A	KIRC	4.07
TCGA-BP-4967-01A	KIRC	5.52
TCGA-3Z-A93Z-01A	KIRC	5.3
TCGA-AK-3454-01A	KIRC	4.46
TCGA-BP-4761-01A	KIRC	4.7
TCGA-CZ-4866-01A	KIRC	6.01
TCGA-B0-5706-01A	KIRC	5.56

TCGA-BP-4166-01A	KIRC	5.37
TCGA-BP-4771-01A	KIRC	4.7
TCGA-A3-3376-01A	KIRC	5.78
TCGA-B0-4710-01A	KIRC	5.76
TCGA-B0-5113-01A	KIRC	5.1
TCGA-CJ-5679-01A	KIRC	6.03
TCGA-BP-5196-01A	KIRC	5.42
TCGA-BP-4759-01A	KIRC	4.39
TCGA-BP-4158-01A	KIRC	4.75
TCGA-CZ-5989-01A	KIRC	6.05
TCGA-B0-4828-01A	KIRC	4.59
TCGA-A3-3329-01A	KIRC	5.7
TCGA-DV-5568-01A	KIRC	5.39
TCGA-A3-3319-01A	KIRC	6.01
TCGA-AK-3455-01A	KIRC	5.44
TCGA-AK-3425-01A	KIRC	5.99
TCGA-BP-5186-01A	KIRC	5.2
TCGA-B0-5696-01A	KIRC	5.42
TCGA-G6-A8L7-01A	KIRC	4.67
TCGA-G6-A8L6-01A	KIRC	4.31
TCGA-BP-5169-01A	KIRC	4.78
TCGA-B0-5691-01A	KIRC	5.83
TCGA-BP-4803-01A	KIRC	5.33
TCGA-A3-3374-01A	KIRC	5.32
TCGA-AK-3450-01A	KIRC	5.8
TCGA-A3-3372-01A	KIRC	5.32
TCGA-BP-4960-01A	KIRC	4.23
TCGA-BP-4165-01A	KIRC	4.17
TCGA-CJ-5676-01A	KIRC	5.53
TCGA-BP-4762-01A	KIRC	5.85
TCGA-B0-4712-01A	KIRC	6.36
TCGA-CJ-4900-01A	KIRC	4.51
TCGA-B0-5115-01A	KIRC	5.19
TCGA-CJ-6027-01A	KIRC	6.15
TCGA-B0-4839-01A	KIRC	4.1
TCGA-B0-4718-01A	KIRC	4.93
TCGA-B0-5095-01A	KIRC	5.69
TCGA-A3-3307-01A	KIRC	5.53
TCGA-CJ-4640-01A	KIRC	5.76
TCGA-BP-4170-01A	KIRC	5.63
TCGA-CW-5588-01A	KIRC	6.4
TCGA-BP-4160-01A	KIRC	5.89
TCGA-G6-A8L8-01A	KIRC	4.35
TCGA-AS-3778-01A	KIRC	5.64
TCGA-B0-5107-01A	KIRC	4.19
TCGA-CZ-5464-01A	KIRC	5.46
TCGA-BP-5177-01A	KIRC	4.7
TCGA-BP-4345-01A	KIRC	5.4
TCGA-B0-5701-01A	KIRC	5.9
TCGA-B0-4849-01A	KIRC	4.31
TCGA-CJ-4908-01A	KIRC	5.33
TCGA-B0-5088-01A	KIRC	4.96
TCGA-B2-3923-01B	KIRC	4.29
TCGA-CJ-4869-01A	KIRC	4.95
TCGA-AK-3461-01A	KIRC	4.73
TCGA-B0-4848-01A	KIRC	6.09
TCGA-B8-A54J-01A	KIRC	4.82
TCGA-AK-3465-01A	KIRC	4.63

TCGA-A3-3316-01A	KIRC	6.05
TCGA-DV-5575-01A	KIRC	5.13
TCGA-B0-4827-01A	KIRC	5.8
TCGA-CJ-4918-01A	KIRC	5.38
TCGA-B0-4706-01A	KIRC	4.41
TCGA-A3-3322-01A	KIRC	4.93
TCGA-A3-A6NI-01A	KIRC	5.04
TCGA-A3-A6NN-01A	KIRC	5.24
TCGA-CJ-4638-01A	KIRC	3.86
TCGA-A3-3349-01A	KIRC	5.67
TCGA-CW-5584-01A	KIRC	5.24
TCGA-A3-3311-01A	KIRC	4.95
TCGA-B0-5694-01A	KIRC	4.74
TCGA-B8-5551-01A	KIRC	6.99
TCGA-B0-4694-01A	KIRC	6.52
TCGA-B0-4696-01A	KIRC	4.99
TCGA-A3-3365-01A	KIRC	5.42
TCGA-B4-5835-01A	KIRC	7.23
TCGA-EU-5905-01A	KIRC	6.02
TCGA-BP-5201-01A	KIRC	4.46
TCGA-B0-5075-01A	KIRC	5.34
TCGA-B2-5639-01A	KIRC	5.75
TCGA-CZ-5465-01A	KIRC	5.83
TCGA-CJ-5683-01A	KIRC	5.09
TCGA-A3-3367-01A	KIRC	5.18
TCGA-CJ-4881-01A	KIRC	5.4
TCGA-CJ-4923-01A	KIRC	5.49
TCGA-A3-A8OV-01A	KIRC	4.85
TCGA-B0-4697-01A	KIRC	3.98
TCGA-CJ-6028-01A	KIRC	6.76
TCGA-B2-4099-01A	KIRC	5.35
TCGA-CJ-5681-01A	KIRC	8.85
TCGA-BP-5170-01A	KIRC	5.35
TCGA-B0-5812-01A	KIRC	5.51
TCGA-CJ-4636-01A	KIRC	5.35
TCGA-B0-5098-01A	KIRC	6.07
TCGA-B8-A54H-01A	KIRC	5.1
TCGA-BP-4807-01A	KIRC	6.2
TCGA-CW-6093-01A	KIRC	5.46
TCGA-CZ-4865-01A	KIRC	5.83
TCGA-B0-4837-01A	KIRC	6.1
TCGA-BP-4169-01A	KIRC	5.76
TCGA-A3-3347-01A	KIRC	6.77
TCGA-B2-3924-01B	KIRC	4.9
TCGA-DV-A4W0-05A	KIRC	4.49
TCGA-AK-3431-01A	KIRC	5.81
TCGA-BP-4162-01A	KIRC	5.66
TCGA-BP-5174-01A	KIRC	5.1
TCGA-CW-6088-01A	KIRC	5.57
TCGA-CJ-4639-01A	KIRC	5.52
TCGA-BP-5184-01A	KIRC	5.43
TCGA-AK-3440-01A	KIRC	3.99
TCGA-BP-5182-01A	KIRC	6.1
TCGA-G6-A5PC-01A	KIRC	3.33
TCGA-BP-4340-01A	KIRC	5.8
TCGA-A3-3383-01A	KIRC	4.62
TCGA-BP-4975-01A	KIRC	5.39
TCGA-B0-5106-01A	KIRC	4.74

TCGA-BP-5007-01A	KIRC	5.11
TCGA-B2-5636-01A	KIRC	5.95
TCGA-CZ-4858-01A	KIRC	6.1
TCGA-CZ-5452-01A	KIRC	5.19
TCGA-BP-4167-01A	KIRC	5.43
TCGA-BP-4353-01A	KIRC	6.72
TCGA-AK-3451-01A	KIRC	4.85
TCGA-DV-5565-01A	KIRC	3.81
TCGA-BP-4982-01A	KIRC	5.53
TCGA-AK-3433-01A	KIRC	2.88
TCGA-BP-5190-01A	KIRC	4.68
TCGA-BP-4330-01A	KIRC	5.16
TCGA-MM-A563-01A	KIRC	5.35
TCGA-CW-5591-01A	KIRC	5.82
TCGA-B0-4833-01A	KIRC	4.2
TCGA-B0-5119-01A	KIRC	5.36
TCGA-DV-A4VZ-01A	KIRC	4.5
TCGA-BP-5187-01A	KIRC	5.34
TCGA-B0-4815-01A	KIRC	5.64
TCGA-CJ-4888-01A	KIRC	5.67
TCGA-CJ-5672-01A	KIRC	5.49
TCGA-DV-A4VX-01A	KIRC	6.17
TCGA-A3-A8OU-01A	KIRC	4.78
TCGA-B0-4838-01A	KIRC	4.29
TCGA-BP-4163-01A	KIRC	5.45
TCGA-B0-5399-01A	KIRC	5.1
TCGA-BP-4349-01A	KIRC	4.66
TCGA-BP-4159-01A	KIRC	4.56
TCGA-CZ-4856-01A	KIRC	5.17
TCGA-B0-5080-01A	KIRC	5.3
TCGA-BP-5200-01A	KIRC	5.44
TCGA-B2-A4SR-01A	KIRC	5.59
TCGA-CZ-5987-01A	KIRC	4.56
TCGA-CJ-4889-01A	KIRC	5.52
TCGA-AK-3453-01A	KIRC	3.89
TCGA-CJ-4904-01A	KIRC	5.1
TCGA-B0-5104-01A	KIRC	4.69
TCGA-BP-4790-01A	KIRC	6.28
TCGA-B8-5552-01B	KIRC	6
TCGA-BP-4335-01A	KIRC	4.19
TCGA-BP-4799-01A	KIRC	4.9
TCGA-B8-5158-01A	KIRC	6.27
TCGA-B0-4700-01A	KIRC	5.91
TCGA-B8-4620-01A	KIRC	5.77
TCGA-AS-3777-01A	KIRC	6.34
TCGA-BP-4804-01A	KIRC	5.21
TCGA-B8-4151-01A	KIRC	5.07
TCGA-BP-4992-01A	KIRC	3.04
TCGA-BP-4789-01A	KIRC	5.6
TCGA-BP-4798-01A	KIRC	5.71
TCGA-CJ-4887-01A	KIRC	5.13
TCGA-CZ-5467-01A	KIRC	5.1
TCGA-B0-5099-01A	KIRC	4.6
TCGA-B0-4847-01A	KIRC	3.59
TCGA-B8-4154-01A	KIRC	5.3
TCGA-A3-3328-01A	KIRC	4.7
TCGA-B0-4713-01A	KIRC	5.03
TCGA-BP-4768-01A	KIRC	4.74

TCGA-CZ-5470-01A	KIRC	5.39
TCGA-DW-7834-01A	KIRP	4.21
TCGA-2Z-A9J8-01A	KIRP	6.73
TCGA-IA-A83T-01A	KIRP	5.17
TCGA-B9-4114-01A	KIRP	4.77
TCGA-UZ-A9PL-01A	KIRP	4.72
TCGA-2Z-A9JG-01A	KIRP	5.56
TCGA-MH-A561-01A	KIRP	4.77
TCGA-HE-A5NL-01A	KIRP	4.58
TCGA-G7-6790-01A	KIRP	6.15
TCGA-A4-A772-01A	KIRP	5.14
TCGA-KV-A6GE-01A	KIRP	4.58
TCGA-EV-5901-01A	KIRP	5.23
TCGA-EV-5902-01A	KIRP	5.25
TCGA-DW-7841-01A	KIRP	4.42
TCGA-MH-A55Z-01A	KIRP	4.53
TCGA-A4-8517-01A	KIRP	4.61
TCGA-A4-7996-01A	KIRP	6
TCGA-B9-A5W7-01A	KIRP	5.21
TCGA-HE-A5NK-01A	KIRP	5.33
TCGA-B9-4117-01A	KIRP	5.8
TCGA-IA-A83S-01A	KIRP	4.92
TCGA-5P-A9K2-01A	KIRP	2.96
TCGA-HE-A5NJ-01A	KIRP	4.12
TCGA-P4-A5E8-01A	KIRP	6.48
TCGA-SX-A71V-01A	KIRP	5.46
TCGA-A4-8310-01A	KIRP	5.51
TCGA-A4-A4ZT-01A	KIRP	5.68
TCGA-DW-5561-01A	KIRP	4.41
TCGA-A4-A7UZ-01A	KIRP	4.91
TCGA-5P-A9JZ-01A	KIRP	5.27
TCGA-AL-3473-01A	KIRP	5.62
TCGA-UN-AAZ9-01A	KIRP	5.79
TCGA-B9-A8YI-01A	KIRP	5
TCGA-UZ-A9PZ-01A	KIRP	4.55
TCGA-SX-A71R-01A	KIRP	3.93
TCGA-J7-8537-01A	KIRP	6.62
TCGA-Y8-A8S0-01A	KIRP	5
TCGA-J7-A8I2-01A	KIRP	5.24
TCGA-5P-A9KC-01A	KIRP	5.83
TCGA-BQ-5883-01A	KIRP	5.84
TCGA-BQ-7055-01A	KIRP	3.32
TCGA-V9-A7HT-01A	KIRP	5.67
TCGA-BQ-7061-01A	KIRP	5.49
TCGA-AL-3467-01A	KIRP	4.63
TCGA-B1-5398-01A	KIRP	5.58
TCGA-BQ-7053-01A	KIRP	4.69
TCGA-2K-A9WE-01A	KIRP	5.95
TCGA-IA-A40U-01A	KIRP	4.84
TCGA-KV-A74V-01A	KIRP	5.7
TCGA-B9-A8YH-01A	KIRP	5.14
TCGA-AL-3468-01A	KIRP	4.4
TCGA-A4-8312-01A	KIRP	4.67
TCGA-B3-3926-01A	KIRP	5.67
TCGA-EV-5903-01A	KIRP	5.14
TCGA-IA-A40Y-01A	KIRP	5.33
TCGA-J7-6720-01A	KIRP	5.29
TCGA-BQ-5887-01A	KIRP	5.67

TCGA-UZ-A9PO-01A	KIRP	5.18
TCGA-UZ-A9Q0-01A	KIRP	3.36
TCGA-2Z-A9JQ-01A	KIRP	4.05
TCGA-B1-7332-01A	KIRP	5.25
TCGA-BQ-5892-01A	KIRP	5.46
TCGA-SX-A71W-01A	KIRP	4.79
TCGA-G7-A8LC-01A	KIRP	4.73
TCGA-G7-6793-01A	KIRP	6.23
TCGA-P4-A5E6-01A	KIRP	4.47
TCGA-BQ-5882-01A	KIRP	6.89
TCGA-B9-A5W9-01A	KIRP	4.98
TCGA-BQ-7051-01A	KIRP	6.11
TCGA-G7-6789-01A	KIRP	4.97
TCGA-AL-7173-01A	KIRP	5.05
TCGA-4A-A93Y-01A	KIRP	5.58
TCGA-UZ-A9PJ-01A	KIRP	4.75
TCGA-2Z-A9J1-01A	KIRP	4.87
TCGA-MH-A560-01A	KIRP	3.93
TCGA-DZ-6132-01A	KIRP	6.04
TCGA-2Z-A9JR-01A	KIRP	5.54
TCGA-5P-A9JV-01A	KIRP	5.55
TCGA-BQ-7049-01A	KIRP	4.94
TCGA-SX-A7SN-01A	KIRP	5.35
TCGA-IZ-A6M8-01A	KIRP	5.08
TCGA-2Z-A9J2-01A	KIRP	3.6
TCGA-IZ-8195-01A	KIRP	5.25
TCGA-BQ-5880-01A	KIRP	4.85
TCGA-BQ-5879-01A	KIRP	5.34
TCGA-B9-4115-01A	KIRP	5.12
TCGA-P4-A5EB-01A	KIRP	5.96
TCGA-BQ-7062-01A	KIRP	4.83
TCGA-GL-A59R-01A	KIRP	4.83
TCGA-G7-A4TM-01A	KIRP	4.58
TCGA-F9-A8NY-01A	KIRP	7.73
TCGA-AL-3466-01A	KIRP	5.49
TCGA-A4-8098-01A	KIRP	4.58
TCGA-B1-A656-01A	KIRP	5.25
TCGA-SX-A7SS-01A	KIRP	4.07
TCGA-A4-A48D-01A	KIRP	5.19
TCGA-UZ-A9PK-01A	KIRP	4.99
TCGA-HE-A5NH-01A	KIRP	5.37
TCGA-DZ-6133-01A	KIRP	6.37
TCGA-B9-7268-01A	KIRP	5.05
TCGA-MH-A857-01A	KIRP	5
TCGA-BQ-5893-01A	KIRP	6.22
TCGA-A4-7286-01A	KIRP	5.39
TCGA-Y8-A8RY-01A	KIRP	4.42
TCGA-Y8-A896-01A	KIRP	5.34
TCGA-SX-A7SQ-01A	KIRP	5.19
TCGA-UZ-A9PM-01A	KIRP	5.38
TCGA-5P-A9JU-01A	KIRP	5.43
TCGA-G7-6797-01A	KIRP	4.8
TCGA-BQ-7056-01A	KIRP	3.91
TCGA-BQ-7058-01A	KIRP	4.13
TCGA-SX-A7SL-01A	KIRP	4.35
TCGA-HE-7129-01A	KIRP	5.31
TCGA-GL-A4EM-01A	KIRP	4.92
TCGA-BQ-7045-01A	KIRP	4.05

TCGA-PJ-A5Z8-01A	KIRP	5.04
TCGA-WN-A9G9-01A	KIRP	4.56
TCGA-B9-4617-01A	KIRP	3.95
TCGA-4A-A93W-01A	KIRP	4.79
TCGA-BQ-7048-01A	KIRP	4.39
TCGA-BQ-5878-01A	KIRP	6
TCGA-A4-8518-01A	KIRP	5
TCGA-5P-A9K4-01A	KIRP	5.01
TCGA-A4-A5DU-01A	KIRP	5.66
TCGA-WN-AB4C-01A	KIRP	3.72
TCGA-SX-A7SR-01A	KIRP	5.12
TCGA-MH-A55W-01A	KIRP	5.12
TCGA-A4-A6HP-01A	KIRP	4.18
TCGA-B1-A654-01A	KIRP	3.87
TCGA-B9-5156-01A	KIRP	5.79
TCGA-DW-7836-01A	KIRP	4.8
TCGA-B3-4103-01A	KIRP	5.67
TCGA-GL-A59T-01A	KIRP	5.62
TCGA-A4-A5Y0-01A	KIRP	3.97
TCGA-BQ-7059-01A	KIRP	5.68
TCGA-A4-7997-01A	KIRP	5.78
TCGA-AT-A5NU-01A	KIRP	4.69
TCGA-UZ-A9PR-01A	KIRP	4.92
TCGA-Y8-A8RZ-01A	KIRP	4
TCGA-MH-A562-01A	KIRP	4.02
TCGA-2Z-A9J3-01A	KIRP	4.74
TCGA-5P-A9JY-01A	KIRP	4.74
TCGA-P4-AAVM-01A	KIRP	5.46
TCGA-Y8-A894-01A	KIRP	4.06
TCGA-P4-A5EA-01A	KIRP	4.29
TCGA-DW-5560-01A	KIRP	3.84
TCGA-G7-7501-01A	KIRP	5.98
TCGA-BQ-5888-01A	KIRP	4.16
TCGA-B9-5155-01A	KIRP	5.45
TCGA-BQ-7060-01A	KIRP	5.18
TCGA-A4-A5Y1-01A	KIRP	6.15
TCGA-DZ-6131-01A	KIRP	5.2
TCGA-UZ-A9PS-05A	KIRP	6.58
TCGA-B1-A47O-01A	KIRP	3.99
TCGA-B9-4113-01A	KIRP	5.05
TCGA-DW-7963-01B	KIRP	4.39
TCGA-B3-3925-01A	KIRP	5.88
TCGA-B1-A655-01A	KIRP	5.75
TCGA-MH-A856-01A	KIRP	3.74
TCGA-BQ-5875-01A	KIRP	5.97
TCGA-Q2-A5QZ-01A	KIRP	3.89
TCGA-5P-A9KH-01A	KIRP	4.25
TCGA-P4-AAVO-01A	KIRP	3.86
TCGA-UZ-A9PV-01A	KIRP	5.2
TCGA-B9-4116-01A	KIRP	5.46
TCGA-GL-A9DD-01A	KIRP	5.48
TCGA-GL-A9DE-01A	KIRP	5.18
TCGA-MH-A855-01A	KIRP	4.44
TCGA-A4-7915-01A	KIRP	5.6
TCGA-GL-8500-01A	KIRP	5.27
TCGA-HE-7130-01A	KIRP	8.91
TCGA-P4-AAVL-01A	KIRP	4.47
TCGA-A4-7828-01A	KIRP	5.17

TCGA-SX-A7SU-01A	KIRP	4.02
TCGA-G7-A8LE-01A	KIRP	4.58
TCGA-Y8-A898-01A	KIRP	5.17
TCGA-Y8-A8S1-01A	KIRP	5.78
TCGA-SX-A7SM-01A	KIRP	4.8
TCGA-5P-A9K3-01A	KIRP	5.48
TCGA-BQ-5877-01A	KIRP	5.84
TCGA-AL-3472-01A	KIRP	3.71
TCGA-IA-A83W-01A	KIRP	4.76
TCGA-P4-AAVK-01A	KIRP	5.63
TCGA-F9-A97G-01A	KIRP	6.91
TCGA-BQ-7050-01A	KIRP	4.98
TCGA-B1-A657-01A	KIRP	5.86
TCGA-F9-A4JJ-01A	KIRP	6.07
TCGA-5P-A9KF-01A	KIRP	5.2
TCGA-UZ-A9PN-01A	KIRP	5.42
TCGA-5P-A9KE-01A	KIRP	4.74
TCGA-DW-7840-01A	KIRP	4.34
TCGA-5P-A9K0-01A	KIRP	4.62
TCGA-F9-A7Q0-01A	KIRP	5.32
TCGA-HE-A5NF-01A	KIRP	5.76
TCGA-BQ-5881-01A	KIRP	3.43
TCGA-2Z-A9JN-01A	KIRP	4.83
TCGA-2Z-A9JI-01A	KIRP	5.24
TCGA-G7-6792-01A	KIRP	4.83
TCGA-GL-6846-01A	KIRP	4.9
TCGA-2Z-A9JJ-01A	KIRP	5.83
TCGA-B1-A47M-01A	KIRP	4.73
TCGA-2Z-A9J6-01A	KIRP	5.08
TCGA-DW-7839-01A	KIRP	4.45
TCGA-5P-A9JW-01A	KIRP	4.53
TCGA-SX-A7SO-01A	KIRP	4.46
TCGA-UZ-A9PS-01A	KIRP	5.88
TCGA-IZ-8196-01A	KIRP	5.09
TCGA-A4-7732-01A	KIRP	4.73
TCGA-2Z-A9J9-01A	KIRP	4.83
TCGA-G7-6796-01A	KIRP	3.89
TCGA-2Z-A9JO-01A	KIRP	4.93
TCGA-F9-A7VF-01A	KIRP	4.18
TCGA-GL-7773-01A	KIRP	4.45
TCGA-2Z-A9JT-01A	KIRP	3.24
TCGA-DZ-6135-01A	KIRP	4.94
TCGA-BQ-5889-01A	KIRP	4.59
TCGA-B9-A69E-01A	KIRP	4.64
TCGA-B9-A44B-01A	KIRP	4.82
TCGA-HE-7128-01A	KIRP	5.39
TCGA-UZ-A9PU-01A	KIRP	5.14
TCGA-4A-A93X-01A	KIRP	5.15
TCGA-A4-7288-01A	KIRP	5.47
TCGA-2Z-A9JP-01A	KIRP	4.23
TCGA-DZ-6134-01A	KIRP	5.29
TCGA-B3-4104-01A	KIRP	7.34
TCGA-KV-A6GD-01A	KIRP	5.17
TCGA-A4-7583-01A	KIRP	4.62
TCGA-BQ-5890-01A	KIRP	5.13
TCGA-BQ-5894-01A	KIRP	5.92
TCGA-A4-8311-01A	KIRP	4.05
TCGA-Y8-A895-01A	KIRP	4.9

TCGA-B3-8121-01A	KIRP	5.95
TCGA-2Z-A9J7-01A	KIRP	4.54
TCGA-A4-A57E-01A	KIRP	5.43
TCGA-IA-A83V-01A	KIRP	5.89
TCGA-DW-7838-01A	KIRP	5.51
TCGA-2Z-A9J5-01A	KIRP	4.91
TCGA-BQ-5885-01A	KIRP	4.98
TCGA-HE-A5NI-01A	KIRP	4.28
TCGA-B1-A47N-01A	KIRP	3.44
TCGA-G7-A8LD-01A	KIRP	4.8
TCGA-5P-A9K9-01A	KIRP	5.53
TCGA-PJ-A5Z9-01A	KIRP	5.58
TCGA-AL-3471-01A	KIRP	4.58
TCGA-UZ-A9PX-01A	KIRP	4.71
TCGA-A4-A5XZ-01A	KIRP	6.05
TCGA-2Z-A9JK-01A	KIRP	5.19
TCGA-P4-A5E7-01A	KIRP	5.34
TCGA-BQ-5891-01A	KIRP	5.4
TCGA-2Z-A9JE-01A	KIRP	4.55
TCGA-SX-A71U-01A	KIRP	5.22
TCGA-2Z-A9JD-01A	KIRP	4.91
TCGA-BQ-5884-01A	KIRP	7.25
TCGA-BQ-5886-01A	KIRP	3.93
TCGA-2Z-A9JL-01A	KIRP	4.61
TCGA-SX-A71S-01A	KIRP	5.04
TCGA-SX-A7SP-01A	KIRP	4.9
TCGA-O9-A75Z-01A	KIRP	4.57
TCGA-IZ-A6M9-01A	KIRP	4.34
TCGA-A4-8630-01A	KIRP	5.39
TCGA-A4-7287-01A	KIRP	7.44
TCGA-2Z-A9JS-01A	KIRP	4.58
TCGA-B9-A5W8-01A	KIRP	5.47
TCGA-AL-A5DJ-01A	KIRP	4.87
TCGA-UZ-A9PQ-01A	KIRP	5.2
TCGA-5P-A9K8-01A	KIRP	4.72
TCGA-G7-7502-01A	KIRP	5.54
TCGA-A4-7734-01A	KIRP	5.52
TCGA-MH-A854-01A	KIRP	3.71
TCGA-A4-8515-01A	KIRP	5.63
TCGA-5P-A9K6-01A	KIRP	4.81
TCGA-G7-A8LB-01A	KIRP	3.49
TCGA-B3-A6W5-01A	KIRP	5.48
TCGA-Y8-A897-01A	KIRP	5.27
TCGA-UZ-A9Q1-01A	KIRP	5.38
TCGA-A4-7585-01A	KIRP	5.57
TCGA-BQ-7044-01A	KIRP	4.59
TCGA-A4-7584-01A	KIRP	4.81
TCGA-UZ-A9PP-01A	KIRP	4.36
TCGA-GL-A9DC-01A	KIRP	5.21
TCGA-2Z-A9JM-01A	KIRP	5
TCGA-BQ-5876-01A	KIRP	7.08
TCGA-5P-A9KA-01A	KIRP	4.33
TCGA-BQ-7046-01A	KIRP	5.69
TCGA-P4-A5ED-01A	KIRP	4.5
TCGA-DW-7837-01A	KIRP	5.15
TCGA-A4-8516-01A	KIRP	5.14
TCGA-IA-A40X-01A	KIRP	4.52
TCGA-G7-6795-01A	KIRP	3.84

TCGA-DW-7842-01A	KIRP	4.81
TCGA-GL-7966-01A	KIRP	6.43
TCGA-AB-2937-03A	LAML	4.47
TCGA-AB-2863-03A	LAML	6.5
TCGA-AB-2941-03A	LAML	5.64
TCGA-AB-2910-03A	LAML	7.53
TCGA-AB-2856-03A	LAML	7.11
TCGA-AB-2874-03A	LAML	6.87
TCGA-AB-2812-03A	LAML	5.89
TCGA-AB-2891-03A	LAML	6.74
TCGA-AB-2851-03A	LAML	6.59
TCGA-AB-2811-03B	LAML	4.85
TCGA-AB-2894-03A	LAML	5.25
TCGA-AB-2876-03A	LAML	5.65
TCGA-AB-2975-03A	LAML	5.34
TCGA-AB-2980-03A	LAML	5.15
TCGA-AB-2949-03B	LAML	5.16
TCGA-AB-2909-03A	LAML	5.96
TCGA-AB-2921-03A	LAML	7.41
TCGA-AB-2936-03A	LAML	5.45
TCGA-AB-2966-03A	LAML	5.67
TCGA-AB-2849-03A	LAML	5.3
TCGA-AB-2998-03A	LAML	6.07
TCGA-AB-2981-03B	LAML	6.93
TCGA-AB-2825-03A	LAML	7.9
TCGA-AB-2927-03A	LAML	5.29
TCGA-AB-2881-03A	LAML	5.85
TCGA-AB-2931-03A	LAML	7.38
TCGA-AB-3007-03A	LAML	5.38
TCGA-AB-2828-03A	LAML	6.52
TCGA-AB-2948-03A	LAML	6.13
TCGA-AB-2963-03A	LAML	6.53
TCGA-AB-2935-03A	LAML	6.22
TCGA-AB-2818-03A	LAML	6.97
TCGA-AB-2883-03A	LAML	5.08
TCGA-AB-2869-03A	LAML	6.55
TCGA-AB-2880-03A	LAML	6.73
TCGA-AB-2834-03A	LAML	6.82
TCGA-AB-2984-03A	LAML	3.91
TCGA-AB-2914-03A	LAML	5.97
TCGA-AB-2861-03A	LAML	5.82
TCGA-AB-2882-03A	LAML	4.65
TCGA-AB-2823-03A	LAML	5.71
TCGA-AB-2813-03A	LAML	6.55
TCGA-AB-2898-03A	LAML	5.37
TCGA-AB-2853-03A	LAML	6.62
TCGA-AB-2893-03A	LAML	7.15
TCGA-AB-2871-03A	LAML	5.8
TCGA-AB-2859-03A	LAML	6.5
TCGA-AB-2920-03B	LAML	4.65
TCGA-AB-2822-03A	LAML	5.79
TCGA-AB-2846-03A	LAML	6.39
TCGA-AB-2885-03A	LAML	6.16
TCGA-AB-2913-03A	LAML	6.03
TCGA-AB-2899-03A	LAML	6.4
TCGA-AB-2970-03A	LAML	5.96
TCGA-AB-2847-03A	LAML	5.38
TCGA-AB-2971-03A	LAML	5.76

TCGA-AB-2986-03A	LAML	5.71
TCGA-AB-2826-03A	LAML	5.81
TCGA-AB-2895-03A	LAML	6.96
TCGA-AB-2924-03A	LAML	6.73
TCGA-AB-2991-03A	LAML	4.42
TCGA-AB-2912-03A	LAML	5.89
TCGA-AB-2911-03A	LAML	4.67
TCGA-AB-2959-03A	LAML	5.63
TCGA-AB-2965-03A	LAML	6.89
TCGA-AB-2872-03A	LAML	6.84
TCGA-AB-2983-03A	LAML	6.41
TCGA-AB-2999-03B	LAML	5.14
TCGA-AB-3002-03A	LAML	5.02
TCGA-AB-2996-03A	LAML	6
TCGA-AB-2944-03A	LAML	5.62
TCGA-AB-2930-03A	LAML	7.15
TCGA-AB-2901-03A	LAML	6.58
TCGA-AB-2842-03A	LAML	6.07
TCGA-AB-2890-03A	LAML	5.56
TCGA-AB-2844-03A	LAML	6.63
TCGA-AB-2925-03A	LAML	6.89
TCGA-AB-2933-03A	LAML	4.84
TCGA-AB-2889-03A	LAML	6.19
TCGA-AB-3001-03A	LAML	5.35
TCGA-AB-2940-03A	LAML	6.09
TCGA-AB-2897-03A	LAML	6.89
TCGA-AB-2908-03A	LAML	5.33
TCGA-AB-2987-03A	LAML	5.64
TCGA-AB-2973-03A	LAML	5.49
TCGA-AB-2896-03B	LAML	6.51
TCGA-AB-2841-03B	LAML	4.75
TCGA-AB-3000-03A	LAML	6.9
TCGA-AB-2810-03A	LAML	3.93
TCGA-AB-2943-03A	LAML	5.63
TCGA-AB-2840-03A	LAML	5.27
TCGA-AB-2888-03B	LAML	6.79
TCGA-AB-3008-03A	LAML	5.85
TCGA-AB-2830-03A	LAML	6.1
TCGA-AB-2858-03A	LAML	5.74
TCGA-AB-2815-03A	LAML	6.27
TCGA-AB-2886-03A	LAML	6.13
TCGA-AB-2820-03A	LAML	4.83
TCGA-AB-2942-03A	LAML	5.47
TCGA-AB-2865-03A	LAML	5.52
TCGA-AB-2915-03A	LAML	7.35
TCGA-AB-3012-03A	LAML	5.11
TCGA-AB-2952-03B	LAML	5.05
TCGA-AB-2877-03A	LAML	6.29
TCGA-AB-2873-03A	LAML	6.94
TCGA-AB-2946-03A	LAML	6.23
TCGA-AB-2836-03A	LAML	6.63
TCGA-AB-2988-03B	LAML	7.36
TCGA-AB-2939-03A	LAML	6.85
TCGA-AB-2892-03A	LAML	5.91
TCGA-AB-2839-03A	LAML	5.69
TCGA-AB-2862-03A	LAML	5.82
TCGA-AB-2929-03A	LAML	5.84
TCGA-AB-2916-03A	LAML	6.01

TCGA-AB-2806-03A	LAML	6.92
TCGA-AB-2990-03B	LAML	5.42
TCGA-AB-2995-03A	LAML	5.02
TCGA-AB-2835-03A	LAML	6.66
TCGA-AB-2808-03A	LAML	6.25
TCGA-AB-2843-03A	LAML	5.66
TCGA-AB-3011-03A	LAML	5.94
TCGA-AB-2857-03A	LAML	5.2
TCGA-AB-2884-03A	LAML	6.73
TCGA-AB-2875-03A	LAML	6.26
TCGA-AB-2992-03A	LAML	5.99
TCGA-AB-2866-03A	LAML	6.99
TCGA-AB-2917-03A	LAML	5.06
TCGA-AB-3009-03A	LAML	6.53
TCGA-AB-2870-03A	LAML	6.19
TCGA-AB-2817-03A	LAML	6.09
TCGA-AB-2950-03A	LAML	5.78
TCGA-AB-2932-03A	LAML	6.28
TCGA-AB-2819-03A	LAML	5.88
TCGA-AB-2900-03A	LAML	6.29
TCGA-AB-2928-03A	LAML	5.63
TCGA-AB-2845-03B	LAML	6.53
TCGA-AB-2814-03A	LAML	5.68
TCGA-AB-2821-03A	LAML	6.11
TCGA-AB-2977-03B	LAML	5.73
TCGA-AB-2938-03A	LAML	6.04
TCGA-AB-2867-03A	LAML	4.9
TCGA-AB-2878-03A	LAML	5.54
TCGA-AB-2934-03A	LAML	6.03
TCGA-AB-2919-03A	LAML	5.85
TCGA-AB-2976-03A	LAML	6.46
TCGA-AB-2805-03A	LAML	6.71
TCGA-AB-2994-03A	LAML	6.04
TCGA-AB-2956-03A	LAML	7.25
TCGA-AB-2955-03A	LAML	6.01
TCGA-AB-2979-03B	LAML	5.77
TCGA-AB-2918-03A	LAML	6.8
TCGA-HT-7691-01A	LGG	5.45
TCGA-S9-A7J0-01A	LGG	7.09
TCGA-HT-7605-01A	LGG	5.5
TCGA-HT-A4DS-01A	LGG	6.04
TCGA-P5-A5EW-01A	LGG	6.6
TCGA-QH-A6CS-01A	LGG	4.95
TCGA-DU-6407-02A	LGG	5.75
TCGA-DU-8162-01A	LGG	5.18
TCGA-CS-4944-01A	LGG	5.22
TCGA-TM-A84I-01A	LGG	5.89
TCGA-E1-A7YS-01A	LGG	6.22
TCGA-S9-A6TU-01A	LGG	5.12
TCGA-RY-A83X-01A	LGG	6.05
TCGA-W9-A837-01A	LGG	5.42
TCGA-DU-A5TP-01A	LGG	5.36
TCGA-DU-A5TT-01A	LGG	4.77
TCGA-TM-A84M-01A	LGG	6.58
TCGA-HT-A61C-01A	LGG	7.12
TCGA-HT-7692-01A	LGG	5.33
TCGA-S9-A6U5-01A	LGG	5.66
TCGA-HT-7485-01A	LGG	5.5

TCGA-HT-7884-01B	LGG	5.92
TCGA-FG-6689-01A	LGG	5.72
TCGA-DU-5847-01A	LGG	6.72
TCGA-DH-A7UT-01A	LGG	5.3
TCGA-FG-7636-01A	LGG	5.64
TCGA-TM-A84J-01A	LGG	6.52
TCGA-DH-A7UR-01A	LGG	5.86
TCGA-VM-A8CD-01A	LGG	6.38
TCGA-HT-7603-01A	LGG	5.29
TCGA-QH-A6CZ-01A	LGG	5.58
TCGA-QH-A6X3-01A	LGG	5.8
TCGA-DU-A7TJ-01A	LGG	8.06
TCGA-FG-6690-01A	LGG	5.35
TCGA-S9-A7R3-01A	LGG	5.07
TCGA-QH-A65V-01A	LGG	5.21
TCGA-DB-5270-01A	LGG	5.82
TCGA-HW-8319-01A	LGG	5.96
TCGA-FG-A60J-01A	LGG	5.34
TCGA-WY-A85B-01A	LGG	6.47
TCGA-FG-7637-01A	LGG	6.8
TCGA-DU-7304-02A	LGG	4.25
TCGA-DH-A66F-01A	LGG	5.87
TCGA-VM-A8CA-01A	LGG	4.25
TCGA-CS-6669-01A	LGG	3.55
TCGA-S9-A6UB-01A	LGG	6.76
TCGA-TQ-A7RO-01A	LGG	5.7
TCGA-QH-A6CY-01A	LGG	5.44
TCGA-TQ-A7RQ-01A	LGG	5.65
TCGA-DU-5870-01A	LGG	5.82
TCGA-DU-7018-01A	LGG	6.64
TCGA-DU-5870-02A	LGG	5.62
TCGA-HT-7681-01A	LGG	5.48
TCGA-TM-A84H-01A	LGG	5.69
TCGA-HT-7860-01A	LGG	7.14
TCGA-HT-7467-01A	LGG	5.33
TCGA-TQ-A7RF-01A	LGG	5.13
TCGA-E1-A7YE-01A	LGG	5.67
TCGA-EZ-7264-01A	LGG	6.04
TCGA-RY-A847-01A	LGG	5.29
TCGA-DU-6408-01A	LGG	5.15
TCGA-DU-6393-01A	LGG	6.35
TCGA-DU-A7TI-01A	LGG	5.44
TCGA-FG-7641-01B	LGG	5.59
TCGA-DU-6402-01A	LGG	6.56
TCGA-HT-A614-01A	LGG	6.02
TCGA-DU-7006-01A	LGG	7.11
TCGA-P5-A72Z-01A	LGG	6.29
TCGA-DB-A4XE-01A	LGG	6.6
TCGA-P5-A737-01A	LGG	5.67
TCGA-RY-A83Y-01A	LGG	5.29
TCGA-DU-5874-01A	LGG	6.58
TCGA-DB-A4XD-01A	LGG	5.81
TCGA-DU-A6S3-01A	LGG	5.68
TCGA-HT-7607-01A	LGG	4.86
TCGA-FG-6691-01A	LGG	6.08
TCGA-WY-A859-01A	LGG	5.58
TCGA-HT-8013-01A	LGG	6.24
TCGA-HW-7493-01A	LGG	5.26

TCGA-DU-A7TD-01A	LGG	7.42
TCGA-HT-7877-01A	LGG	5.56
TCGA-HT-A616-01A	LGG	4.83
TCGA-WY-A85C-01A	LGG	5.29
TCGA-QH-A6X5-01A	LGG	5.31
TCGA-TM-A84B-01A	LGG	7.24
TCGA-FG-A4MY-01A	LGG	5.32
TCGA-DU-8164-01A	LGG	6.82
TCGA-HT-7606-01A	LGG	7.34
TCGA-TM-A84Q-01A	LGG	6.43
TCGA-S9-A89V-01A	LGG	6.54
TCGA-S9-A6WL-01A	LGG	5.66
TCGA-HT-A617-01A	LGG	6.02
TCGA-HT-8105-01A	LGG	5.9
TCGA-P5-A780-01A	LGG	5.73
TCGA-P5-A733-01A	LGG	4.29
TCGA-DU-6397-01A	LGG	5.95
TCGA-DB-A64L-01A	LGG	4.97
TCGA-DB-5277-01A	LGG	5.81
TCGA-DU-5855-01A	LGG	5.74
TCGA-HT-7468-01A	LGG	6.71
TCGA-DU-A7TB-01A	LGG	6.24
TCGA-DU-8166-01A	LGG	6.86
TCGA-DB-A4XB-01A	LGG	6.53
TCGA-HT-7601-01A	LGG	6.71
TCGA-S9-A6WM-01A	LGG	6.75
TCGA-HT-A74H-01A	LGG	4.37
TCGA-CS-4941-01A	LGG	6.41
TCGA-E1-A7YU-01A	LGG	5.68
TCGA-DU-8168-01A	LGG	7.09
TCGA-TM-A84T-01A	LGG	6.17
TCGA-DU-7007-01A	LGG	6.41
TCGA-S9-A7R2-01A	LGG	4.6
TCGA-TM-A84O-01A	LGG	5.94
TCGA-DB-A64Q-01A	LGG	6
TCGA-QH-A65S-01A	LGG	5.34
TCGA-DU-6405-01A	LGG	7.51
TCGA-S9-A7R1-01A	LGG	5.28
TCGA-HT-8111-01A	LGG	5.36
TCGA-CS-6668-01A	LGG	6.02
TCGA-TM-A84L-01A	LGG	5.64
TCGA-P5-A781-01A	LGG	5.75
TCGA-HT-7686-01A	LGG	6.52
TCGA-S9-A6WD-01A	LGG	5.38
TCGA-HT-7687-01A	LGG	6.22
TCGA-HT-7695-01A	LGG	5.98
TCGA-E1-5303-01A	LGG	5.95
TCGA-P5-A72U-01A	LGG	5.07
TCGA-S9-A7R4-01A	LGG	5.87
TCGA-HT-7478-01A	LGG	5.92
TCGA-HW-7486-01A	LGG	5.84
TCGA-DU-8167-01A	LGG	5.89
TCGA-CS-6290-01A	LGG	5.89
TCGA-HT-7857-01A	LGG	6.73
TCGA-DU-5849-01A	LGG	5.39
TCGA-HT-7475-01A	LGG	6.3
TCGA-DU-5872-01A	LGG	5.79
TCGA-DU-5872-02A	LGG	5.75

TCGA-TQ-A7RV-02A	LGG	4.78
TCGA-HT-7693-01A	LGG	5.72
TCGA-VM-A8CE-01A	LGG	5.72
TCGA-S9-A6U6-01A	LGG	5.79
TCGA-P5-A77X-01A	LGG	5.54
TCGA-DB-A75P-01A	LGG	5.65
TCGA-HT-7882-01A	LGG	6.9
TCGA-S9-A7QY-01A	LGG	5.37
TCGA-HT-8558-01A	LGG	3.93
TCGA-HT-8108-01A	LGG	6.84
TCGA-E1-A7Z2-01A	LGG	4.51
TCGA-IK-8125-01A	LGG	5.7
TCGA-E1-5305-01A	LGG	6.45
TCGA-S9-A6WO-01A	LGG	5.82
TCGA-E1-A7YO-01A	LGG	5.46
TCGA-E1-A7YH-01A	LGG	5.85
TCGA-DB-A4XA-01A	LGG	5.64
TCGA-HW-A5KK-01A	LGG	5.38
TCGA-CS-6666-01A	LGG	6.04
TCGA-DB-5279-01A	LGG	4.9
TCGA-TQ-A7RI-01A	LGG	5.84
TCGA-DU-A7T6-01A	LGG	5.6
TCGA-DU-A6S8-01A	LGG	6.43
TCGA-E1-A7Z4-01A	LGG	5.3
TCGA-TM-A84C-01A	LGG	5.2
TCGA-DB-5276-01A	LGG	5.65
TCGA-DU-7300-01A	LGG	5.87
TCGA-DB-A64U-01A	LGG	6.34
TCGA-CS-6186-01A	LGG	7.08
TCGA-FG-A6J3-01A	LGG	5.23
TCGA-P5-A735-01A	LGG	6.28
TCGA-WY-A858-01A	LGG	4.83
TCGA-HT-7879-01A	LGG	6.03
TCGA-DU-7309-01A	LGG	5.47
TCGA-S9-A89Z-01A	LGG	6.38
TCGA-HT-A619-01A	LGG	6.59
TCGA-HT-7473-01A	LGG	5.71
TCGA-DU-7013-01A	LGG	6.81
TCGA-HT-A61B-01A	LGG	6.26
TCGA-R8-A73M-01A	LGG	6.08
TCGA-DU-7012-01A	LGG	6.14
TCGA-DU-5871-01A	LGG	5.38
TCGA-S9-A6TY-01A	LGG	6.12
TCGA-DH-5143-01A	LGG	5.66
TCGA-HT-A5R9-01A	LGG	6.09
TCGA-FG-8186-01A	LGG	5.9
TCGA-P5-A5ET-01A	LGG	6.07
TCGA-FG-A4MT-01A	LGG	5.86
TCGA-FG-A4MW-01A	LGG	6.21
TCGA-FG-A4MT-02A	LGG	5.92
TCGA-TQ-A7RJ-01A	LGG	5.67
TCGA-P5-A77W-01A	LGG	6.1
TCGA-HT-8564-01A	LGG	6.75
TCGA-HT-A61A-01A	LGG	4.69
TCGA-HT-7689-01A	LGG	6.04
TCGA-S9-A6WE-01A	LGG	6.63
TCGA-FG-5964-01A	LGG	5.94
TCGA-DU-6407-02B	LGG	8.02

TCGA-HT-A5R5-01A	LGG	4.55
TCGA-E1-A7YQ-01A	LGG	5.86
TCGA-HT-7688-01A	LGG	5.73
TCGA-DU-7010-01A	LGG	6.36
TCGA-KT-A7W1-01A	LGG	7.5
TCGA-DB-A4XG-01A	LGG	5.81
TCGA-RY-A83Z-01A	LGG	5.75
TCGA-DB-A64P-01A	LGG	5.84
TCGA-DU-A5TU-01A	LGG	5.96
TCGA-DB-5281-01A	LGG	5.94
TCGA-TM-A84R-01A	LGG	4.84
TCGA-WY-A85E-01A	LGG	5.78
TCGA-HT-7476-01A	LGG	5.18
TCGA-DU-7298-01A	LGG	7.05
TCGA-E1-A7YV-01A	LGG	5.58
TCGA-P5-A5EX-01A	LGG	5.94
TCGA-HT-7482-01A	LGG	5.88
TCGA-QH-A65X-01A	LGG	5.32
TCGA-E1-A7YD-01A	LGG	6.78
TCGA-HT-7472-01A	LGG	6.47
TCGA-DB-A4XF-01A	LGG	5.34
TCGA-P5-A72W-01A	LGG	5.2
TCGA-FG-6692-01A	LGG	6.44
TCGA-DB-A64S-01A	LGG	5.85
TCGA-FN-7833-01A	LGG	6.38
TCGA-TQ-A7RR-01A	LGG	5.44
TCGA-FG-8182-01A	LGG	6.88
TCGA-DU-7302-01A	LGG	6.3
TCGA-E1-A7YM-01A	LGG	5.19
TCGA-QH-A65R-01A	LGG	5.47
TCGA-VM-A8C9-01A	LGG	4.67
TCGA-HT-7854-01A	LGG	4.54
TCGA-DU-A5TR-01A	LGG	4.71
TCGA-DU-A5TW-01A	LGG	6.08
TCGA-HT-7471-01A	LGG	6.82
TCGA-VM-A8CH-01A	LGG	5.64
TCGA-HT-A5RA-01A	LGG	6.81
TCGA-CS-6670-01A	LGG	5.7
TCGA-TQ-A7RH-01A	LGG	5.71
TCGA-DB-A4X9-01A	LGG	5.99
TCGA-FG-A60L-01A	LGG	5.3
TCGA-HT-A74O-01A	LGG	5.13
TCGA-R8-A6MK-01A	LGG	5.12
TCGA-FG-5963-02A	LGG	6.73
TCGA-DH-A7US-01A	LGG	6.21
TCGA-TQ-A7RG-01A	LGG	6.09
TCGA-FG-A87Q-01A	LGG	5.91
TCGA-HT-A5RC-01A	LGG	6.65
TCGA-DH-A669-02A	LGG	6.34
TCGA-FG-A87N-01A	LGG	7.03
TCGA-DU-6407-01A	LGG	6.35
TCGA-RY-A840-01A	LGG	5.72
TCGA-TQ-A7RP-01A	LGG	3.73
TCGA-E1-A7YY-01A	LGG	4.67
TCGA-HW-8322-01A	LGG	6.77
TCGA-HT-7474-01A	LGG	5.23
TCGA-FG-5963-01A	LGG	6.49
TCGA-HT-7602-01A	LGG	5.19

TCGA-P5-A5EZ-01A	LGG	5.33
TCGA-QH-A6XC-01A	LGG	5.51
TCGA-P5-A5F0-01A	LGG	5.97
TCGA-DU-7301-01A	LGG	6.53
TCGA-DU-6403-01A	LGG	6.61
TCGA-F6-A8O3-01A	LGG	6.79
TCGA-HT-8012-01A	LGG	5.79
TCGA-HW-A5KJ-01A	LGG	4.92
TCGA-S9-A7IY-01A	LGG	5.5
TCGA-QH-A6X4-01A	LGG	5.6
TCGA-S9-A7R8-01A	LGG	6.27
TCGA-TM-A7C5-01A	LGG	6.28
TCGA-DU-A6S7-01A	LGG	5.66
TCGA-S9-A6U1-01A	LGG	6.29
TCGA-DB-A64X-01A	LGG	5.32
TCGA-HT-7676-01A	LGG	6.52
TCGA-HT-7479-01A	LGG	5.13
TCGA-VM-A8C8-01A	LGG	5.31
TCGA-P5-A736-01A	LGG	4.87
TCGA-CS-4943-01A	LGG	6.89
TCGA-DH-A7UU-01A	LGG	6.39
TCGA-S9-A7QZ-01A	LGG	5.53
TCGA-DU-A76R-01A	LGG	5.13
TCGA-HT-8019-01A	LGG	3.51
TCGA-FG-A70Z-01A	LGG	6.53
TCGA-HT-8113-01A	LGG	4.05
TCGA-CS-6667-01A	LGG	5.34
TCGA-QH-A86X-01A	LGG	5.82
TCGA-IK-7675-01A	LGG	5.8
TCGA-E1-5322-01A	LGG	7.08
TCGA-E1-A7YW-01A	LGG	5.93
TCGA-F6-A8O4-01A	LGG	5.51
TCGA-E1-5304-01A	LGG	7.56
TCGA-S9-A7QW-01A	LGG	6.06
TCGA-HW-7489-01A	LGG	5.13
TCGA-QH-A6CU-01A	LGG	4.43
TCGA-DH-A669-01A	LGG	6.3
TCGA-HT-A5R7-01A	LGG	5.12
TCGA-E1-5311-01A	LGG	5.94
TCGA-TQ-A7RK-02A	LGG	6.31
TCGA-DU-A76K-01A	LGG	5.77
TCGA-HT-7881-01A	LGG	4.92
TCGA-S9-A7IZ-01A	LGG	5.31
TCGA-HT-7855-01A	LGG	5.5
TCGA-FG-8191-01A	LGG	5.59
TCGA-S9-A7J3-01A	LGG	6.02
TCGA-P5-A5F1-01A	LGG	4.98
TCGA-S9-A6WH-01A	LGG	5.71
TCGA-S9-A6TV-01A	LGG	6.3
TCGA-HW-A5KL-01A	LGG	5.5
TCGA-HT-7611-01A	LGG	5.92
TCGA-WY-A85D-01A	LGG	5.38
TCGA-VV-A829-01A	LGG	6.47
TCGA-S9-A6WQ-01A	LGG	5.77
TCGA-DU-6396-01A	LGG	6.54
TCGA-CS-4942-01A	LGG	5.95
TCGA-DB-A64V-01A	LGG	5.38
TCGA-P5-A731-01A	LGG	5.47

TCGA-VM-A8CB-01A	LGG	6.11
TCGA-DB-A4XC-01A	LGG	5.82
TCGA-FG-7634-01A	LGG	6.65
TCGA-P5-A5EY-01A	LGG	5.37
TCGA-TQ-A7RK-01A	LGG	5.5
TCGA-QH-A870-01A	LGG	4.88
TCGA-P5-A72X-01A	LGG	5.13
TCGA-RY-A845-01A	LGG	6.05
TCGA-FG-5965-01B	LGG	5.78
TCGA-DU-6410-01A	LGG	6.71
TCGA-HT-7880-01A	LGG	4.69
TCGA-TM-A84F-01A	LGG	5.6
TCGA-P5-A730-01A	LGG	5.75
TCGA-S9-A6WI-01A	LGG	3.5
TCGA-S9-A7J2-01A	LGG	6.08
TCGA-TQ-A8XE-01A	LGG	5.9
TCGA-DU-7019-01A	LGG	6.19
TCGA-R8-A6MO-01A	LGG	6.14
TCGA-QH-A6CV-01A	LGG	5.31
TCGA-P5-A5F6-01A	LGG	5.58
TCGA-S9-A7J1-01A	LGG	5.73
TCGA-DH-5140-01A	LGG	7.28
TCGA-VM-A8CF-01A	LGG	4.13
TCGA-DH-5141-01A	LGG	5.14
TCGA-HT-7477-01B	LGG	6.1
TCGA-S9-A6U9-01A	LGG	4.82
TCGA-CS-6188-01A	LGG	7.5
TCGA-S9-A6TX-01A	LGG	5.4
TCGA-R8-A6ML-01A	LGG	5.73
TCGA-S9-A7QX-01A	LGG	6.06
TCGA-FG-8189-01B	LGG	3.89
TCGA-HT-A74L-01A	LGG	5.81
TCGA-HT-7610-01A	LGG	5.79
TCGA-DU-5851-01A	LGG	5.51
TCGA-FG-A710-01A	LGG	5.99
TCGA-VW-A8FI-01A	LGG	6.22
TCGA-E1-A7YI-01A	LGG	7.46
TCGA-DH-5142-01A	LGG	6.22
TCGA-DB-A75K-01A	LGG	5.84
TCGA-HT-8104-01A	LGG	7.33
TCGA-TQ-A7RM-01A	LGG	6.45
TCGA-RY-A843-01A	LGG	5.26
TCGA-VW-A7QS-01A	LGG	5.83
TCGA-DB-5278-01A	LGG	6.5
TCGA-HT-A4DV-01A	LGG	5.31
TCGA-HW-8321-01A	LGG	5.99
TCGA-HT-7604-01A	LGG	5.86
TCGA-S9-A6U2-01A	LGG	6.13
TCGA-FG-5965-02A	LGG	6.24
TCGA-CS-4938-01B	LGG	5.56
TCGA-DU-7009-01A	LGG	6.52
TCGA-DB-5280-01A	LGG	6.42
TCGA-DU-6400-01A	LGG	6.33
TCGA-FG-8181-01A	LGG	3.4
TCGA-DU-7292-01A	LGG	6.65
TCGA-WY-A85A-01A	LGG	6.37
TCGA-DU-8165-01A	LGG	6.35
TCGA-TQ-A8XE-02A	LGG	6.25

TCGA-DB-5274-01A	LGG	6.06
TCGA-HT-A74J-01A	LGG	5.5
TCGA-P5-A5EU-01A	LGG	6.67
TCGA-QH-A6X9-01A	LGG	6.23
TCGA-VV-A86M-01A	LGG	6.1
TCGA-TM-A7CA-01A	LGG	6.51
TCGA-DU-5853-01A	LGG	6.15
TCGA-TQ-A7RK-02B	LGG	6.11
TCGA-FG-7638-01B	LGG	5.5
TCGA-DU-A5TY-01A	LGG	5.99
TCGA-FG-A711-01A	LGG	5.56
TCGA-DU-7294-01A	LGG	6.87
TCGA-KT-A74X-01A	LGG	6.47
TCGA-HW-7495-01A	LGG	5.38
TCGA-DH-A7UV-01A	LGG	5.73
TCGA-E1-5318-01A	LGG	6.42
TCGA-TQ-A7RS-01A	LGG	5.89
TCGA-HT-7694-01A	LGG	5.15
TCGA-DU-A6S6-01A	LGG	5.68
TCGA-DU-7008-01A	LGG	6.57
TCGA-HT-7608-01A	LGG	4.82
TCGA-QH-A6XA-01A	LGG	6
TCGA-DU-6394-01A	LGG	6.1
TCGA-HT-7680-01A	LGG	5.08
TCGA-TQ-A7RV-01A	LGG	5.98
TCGA-DB-A4XH-01A	LGG	5.61
TCGA-DU-A7TC-01A	LGG	5.56
TCGA-TM-A84G-01A	LGG	7.08
TCGA-DU-A6S2-01A	LGG	5.29
TCGA-CS-5396-01A	LGG	6.58
TCGA-HT-8110-01A	LGG	6.36
TCGA-E1-A7YK-01A	LGG	5.95
TCGA-QH-A65Z-01A	LGG	6.39
TCGA-DU-7306-01A	LGG	5.48
TCGA-DU-8161-01A	LGG	6.2
TCGA-TQ-A7RN-01A	LGG	5.81
TCGA-CS-5390-01A	LGG	5.91
TCGA-DU-8163-01A	LGG	6.27
TCGA-HT-8015-01B	LGG	5.09
TCGA-E1-A7Z6-01A	LGG	5.06
TCGA-DU-7299-01A	LGG	6.13
TCGA-CS-5394-01A	LGG	6.31
TCGA-E1-A7YL-01A	LGG	6.32
TCGA-DU-6401-01A	LGG	5.7
TCGA-HT-A618-01A	LGG	5.83
TCGA-FG-A60K-01A	LGG	6.13
TCGA-HW-7491-01A	LGG	6.95
TCGA-FG-A4MX-01A	LGG	4.46
TCGA-S9-A6WG-01A	LGG	5.35
TCGA-DU-5852-01A	LGG	7.01
TCGA-DB-5273-01A	LGG	6.06
TCGA-HT-7616-01A	LGG	6.17
TCGA-S9-A6U0-01A	LGG	6.15
TCGA-S9-A7R7-01A	LGG	6.36
TCGA-FG-7643-01A	LGG	4.52
TCGA-S9-A6WN-01A	LGG	5.19
TCGA-HT-7856-01A	LGG	5.26
TCGA-DU-8158-01A	LGG	6.31

TCGA-S9-A6U8-01A	LGG	5.73
TCGA-E1-A7YJ-01A	LGG	6.03
TCGA-S9-A6TZ-01A	LGG	6.11
TCGA-DU-6404-02A	LGG	5.68
TCGA-HT-7858-01A	LGG	6.33
TCGA-HT-A74K-01A	LGG	4.75
TCGA-HT-8106-01A	LGG	7.16
TCGA-HT-7609-01A	LGG	5.6
TCGA-HT-7481-01A	LGG	5.82
TCGA-E1-5307-01A	LGG	6
TCGA-DB-5275-01A	LGG	6.33
TCGA-DU-5854-01A	LGG	6.33
TCGA-HT-7684-01A	LGG	4.23
TCGA-DU-A76O-01A	LGG	6.17
TCGA-E1-A7YN-01A	LGG	6.62
TCGA-TQ-A7RU-01A	LGG	6.79
TCGA-DH-A66B-01A	LGG	6.25
TCGA-DU-6406-01A	LGG	7.45
TCGA-HT-7677-01A	LGG	7.12
TCGA-HT-7480-01A	LGG	6.05
TCGA-DU-A76L-01A	LGG	7.21
TCGA-E1-A7Z3-01A	LGG	5.49
TCGA-FG-5965-02B	LGG	4.94
TCGA-WH-A86K-01A	LGG	4.95
TCGA-HW-7487-01A	LGG	5.62
TCGA-DU-7014-01A	LGG	5.54
TCGA-S9-A7IX-01A	LGG	6.23
TCGA-R8-A6YH-01A	LGG	5.78
TCGA-HW-7490-01A	LGG	5.44
TCGA-DU-6404-02B	LGG	5.45
TCGA-P5-A5F2-01A	LGG	4.88
TCGA-S9-A6TW-01A	LGG	5.77
TCGA-HT-7875-01A	LGG	6.16
TCGA-HT-8011-01A	LGG	6.85
TCGA-DU-6399-01A	LGG	6.38
TCGA-S9-A6TS-01A	LGG	5.42
TCGA-DU-A7TG-01A	LGG	4.62
TCGA-DU-6395-01A	LGG	5.93
TCGA-DB-A64R-01A	LGG	6.4
TCGA-HT-7873-01B	LGG	6.74
TCGA-FG-A70Y-01A	LGG	5.7
TCGA-HT-7469-01A	LGG	6.52
TCGA-DU-6404-01A	LGG	5.49
TCGA-CS-5397-01A	LGG	6.54
TCGA-HT-8109-01A	LGG	5.49
TCGA-DB-A75L-01A	LGG	5.65
TCGA-DU-7011-01A	LGG	5.89
TCGA-HT-7483-01A	LGG	6.42
TCGA-FG-8187-01A	LGG	5.14
TCGA-FG-A4MU-01B	LGG	7.23
TCGA-E1-5302-01A	LGG	6.18
TCGA-HT-8018-01A	LGG	5.59
TCGA-HT-7874-01A	LGG	5.05
TCGA-E1-5319-01A	LGG	5.96
TCGA-HT-7902-01A	LGG	5.4
TCGA-P5-A5F4-01A	LGG	6.2
TCGA-TM-A7CF-01A	LGG	5.54
TCGA-TM-A7CF-02A	LGG	5.71

TCGA-HT-8114-01A	LGG	5.99
TCGA-HT-7620-01A	LGG	5.46
TCGA-HW-A5KM-01A	LGG	6.72
TCGA-HT-A5RB-01A	LGG	6.35
TCGA-S9-A6WP-01A	LGG	6.25
TCGA-HT-8010-01A	LGG	4.17
TCGA-DB-A75M-01A	LGG	4.65
TCGA-DB-A75O-01A	LGG	4.98
TCGA-DU-A7T8-01A	LGG	6.05
TCGA-DB-A64W-01A	LGG	6.78
TCGA-DH-A66G-01A	LGG	5.8
TCGA-DU-A5TS-01A	LGG	4.66
TCGA-DU-6397-02A	LGG	5.75
TCGA-DU-7290-01A	LGG	5.43
TCGA-QH-A6X8-01A	LGG	6.44
TCGA-CS-5393-01A	LGG	5.8
TCGA-DU-A7TA-01A	LGG	5.87
TCGA-DH-A66D-01A	LGG	6.42
TCGA-P5-A5EV-01A	LGG	6.17
TCGA-S9-A7IS-01A	LGG	7.36
TCGA-HT-8107-01A	LGG	3.83
TCGA-QH-A6CW-01A	LGG	5.52
TCGA-FG-8188-01A	LGG	5.55
TCGA-DH-5144-01A	LGG	6.96
TCGA-DB-A64O-01A	LGG	4.89
TCGA-S9-A7IQ-01A	LGG	4.78
TCGA-TM-A84S-01A	LGG	5.71
TCGA-FG-6688-01A	LGG	5.32
TCGA-DU-6542-01A	LGG	6.22
TCGA-CS-6665-01A	LGG	6.52
TCGA-HT-8563-01A	LGG	7.12
TCGA-QH-A6CX-01A	LGG	5.52
TCGA-FG-A6IZ-01A	LGG	5.98
TCGA-TM-A7C3-01A	LGG	4.75
TCGA-HT-A615-01A	LGG	6.2
TCGA-S9-A6UA-01A	LGG	6.42
TCGA-FG-5962-01B	LGG	5.65
TCGA-FG-A6J1-01A	LGG	5.72
TCGA-HW-8320-01A	LGG	6.04
TCGA-HT-7690-01A	LGG	7.1
TCGA-FG-8185-01A	LGG	5.44
TCGA-DU-7015-01A	LGG	6.15
TCGA-DU-7304-01A	LGG	5.21
TCGA-CS-5395-01A	LGG	6.76
TCGA-TM-A7C4-01A	LGG	5.95
TCGA-HT-7470-01A	LGG	5
TCGA-TQ-A7RW-01A	LGG	5.7
TCGA-DU-6392-01A	LGG	7.13
TCGA-FG-A713-01A	LGG	4.19
TCGA-RC-A7SF-01A	LIHC	6
TCGA-EP-A2KC-01A	LIHC	7.15
TCGA-ES-A2HS-01A	LIHC	6.47
TCGA-CC-5259-01A	LIHC	5.89
TCGA-ED-A97K-01A	LIHC	7.69
TCGA-DD-A3A1-01A	LIHC	7.54
TCGA-G3-A6UC-01A	LIHC	8.09
TCGA-DD-AACU-01A	LIHC	8.02
TCGA-DD-A1EG-01A	LIHC	8.1

TCGA-DD-A4NJ-01A	LIHC	6.91
TCGA-ZS-A9CF-02A	LIHC	7.98
TCGA-BD-A3ER-01A	LIHC	7.17
TCGA-G3-A5SL-01A	LIHC	7.83
TCGA-DD-A73F-01A	LIHC	7.48
TCGA-CC-A3M9-01A	LIHC	6.29
TCGA-ED-A627-01A	LIHC	6.95
TCGA-CC-A8HT-01A	LIHC	8.29
TCGA-UB-A7MF-01A	LIHC	7.06
TCGA-2Y-A9H1-01A	LIHC	5.73
TCGA-DD-AADO-01A	LIHC	6.52
TCGA-ZP-A9CV-01A	LIHC	8.09
TCGA-4R-AA8I-01A	LIHC	7.8
TCGA-DD-A113-01A	LIHC	6.94
TCGA-DD-AADL-01A	LIHC	7.82
TCGA-RC-A7SK-01A	LIHC	6.75
TCGA-DD-AAEA-01A	LIHC	6.9
TCGA-FV-A495-01A	LIHC	6.53
TCGA-EP-A26S-01A	LIHC	8.73
TCGA-DD-AAEG-01A	LIHC	7.5
TCGA-G3-A25S-01A	LIHC	7.92
TCGA-G3-A3CJ-01A	LIHC	7.15
TCGA-G3-A3CI-01A	LIHC	7.54
TCGA-2Y-A9H2-01A	LIHC	6.45
TCGA-DD-A4NG-01A	LIHC	7.05
TCGA-K7-A6G5-01A	LIHC	7.44
TCGA-DD-AADG-01A	LIHC	7.6
TCGA-LG-A9QC-01A	LIHC	7.69
TCGA-DD-AACJ-01A	LIHC	6.7
TCGA-DD-A1EA-01A	LIHC	8.12
TCGA-LG-A9QD-01A	LIHC	6.87
TCGA-ED-A7PY-01A	LIHC	7.34
TCGA-NI-A4U2-01A	LIHC	6.75
TCGA-DD-A1EK-01A	LIHC	7.67
TCGA-DD-AADP-01A	LIHC	7.28
TCGA-DD-AAVS-01A	LIHC	8.35
TCGA-ED-A82E-01A	LIHC	6.82
TCGA-CC-A7II-01A	LIHC	5.25
TCGA-EP-A3JL-01A	LIHC	6.44
TCGA-DD-A3A4-01A	LIHC	9.2
TCGA-DD-AACP-01A	LIHC	7.22
TCGA-2Y-A9H6-01A	LIHC	7.19
TCGA-UB-A7MB-01A	LIHC	7.29
TCGA-UB-A7MD-01A	LIHC	8.25
TCGA-CC-5260-01A	LIHC	7.14
TCGA-DD-A1EH-01A	LIHC	8.28
TCGA-ED-A4XI-01A	LIHC	8.08
TCGA-DD-AADR-01A	LIHC	7.74
TCGA-FV-A496-01A	LIHC	7.29
TCGA-G3-A3CG-01A	LIHC	7.12
TCGA-DD-AACS-01A	LIHC	7.89
TCGA-DD-AAVR-01A	LIHC	7.33
TCGA-BC-A10T-01A	LIHC	7.32
TCGA-3K-AAZ8-01A	LIHC	7.58
TCGA-DD-AAVV-01A	LIHC	6.88
TCGA-2Y-A9GS-01A	LIHC	8.78
TCGA-RC-A6M6-01A	LIHC	7.12
TCGA-G3-AAV6-01A	LIHC	7.92

TCGA-CC-5263-01A	LIHC	8.25
TCGA-ZS-A9CE-01A	LIHC	7.82
TCGA-DD-A4ND-01A	LIHC	7.22
TCGA-DD-AAVX-01A	LIHC	7.49
TCGA-DD-A4NE-01A	LIHC	8.37
TCGA-2Y-A9HB-01A	LIHC	7.96
TCGA-CC-A9FV-01A	LIHC	5.54
TCGA-DD-A39Y-01A	LIHC	6.95
TCGA-CC-A5UC-01A	LIHC	7.69
TCGA-DD-AACB-01A	LIHC	7.12
TCGA-HP-A5N0-01A	LIHC	8.16
TCGA-ED-A8O6-01A	LIHC	7.39
TCGA-ZS-A9CF-01A	LIHC	8.61
TCGA-2Y-A9H4-01A	LIHC	7.91
TCGA-DD-A116-01A	LIHC	7.07
TCGA-G3-A7M5-01A	LIHC	7
TCGA-BC-A112-01A	LIHC	7.49
TCGA-DD-A4NV-01A	LIHC	7.2
TCGA-DD-AAE6-01A	LIHC	8.74
TCGA-CC-A7IL-01A	LIHC	7.73
TCGA-5C-AAPD-01A	LIHC	7.31
TCGA-DD-AAC9-01A	LIHC	7.49
TCGA-MI-A75C-01A	LIHC	7.3
TCGA-FV-A23B-01A	LIHC	7.27
TCGA-DD-AAVY-01A	LIHC	8.54
TCGA-DD-A3A6-01A	LIHC	4.04
TCGA-DD-AADD-01A	LIHC	8.19
TCGA-G3-A25U-01A	LIHC	6.98
TCGA-DD-AAE2-01A	LIHC	7.49
TCGA-DD-AAW3-01A	LIHC	7.59
TCGA-BW-A5NP-01A	LIHC	8.2
TCGA-DD-AA3A-01A	LIHC	6.35
TCGA-XR-A8TD-01A	LIHC	8.06
TCGA-BC-A10S-01A	LIHC	6.18
TCGA-T1-A6J8-01A	LIHC	7.56
TCGA-KR-A7K0-01A	LIHC	7.96
TCGA-DD-A4NK-01A	LIHC	7.8
TCGA-DD-A3A9-01A	LIHC	7.3
TCGA-K7-A5RG-01A	LIHC	7.92
TCGA-DD-A4NR-01A	LIHC	7.61
TCGA-FV-A4ZP-01A	LIHC	5.86
TCGA-CC-A9FU-01A	LIHC	7.78
TCGA-DD-AACW-01A	LIHC	7.49
TCGA-DD-A1EC-01A	LIHC	6.78
TCGA-WJ-A86L-01A	LIHC	6.89
TCGA-DD-AAEK-01A	LIHC	7.75
TCGA-FV-A4ZQ-01A	LIHC	7.89
TCGA-5C-A9VH-01A	LIHC	6.73
TCGA-DD-AACA-02B	LIHC	6.96
TCGA-DD-AACA-02A	LIHC	7.41
TCGA-DD-AACA-01A	LIHC	7.68
TCGA-FV-A2QQ-01A	LIHC	7.56
TCGA-2Y-A9GX-01A	LIHC	7.27
TCGA-G3-AAUZ-01A	LIHC	6.22
TCGA-DD-AACK-01A	LIHC	8.78
TCGA-DD-A3A5-01A	LIHC	8.1
TCGA-DD-AACY-01A	LIHC	6.42
TCGA-BD-A3EP-01A	LIHC	7.08

TCGA-BC-A10Q-01A	LIHC	7.29
TCGA-CC-A7IF-01A	LIHC	5.96
TCGA-DD-AADY-01A	LIHC	7.86
TCGA-DD-AACI-01A	LIHC	7.52
TCGA-CC-A7IH-01A	LIHC	7.89
TCGA-UB-AA0V-01A	LIHC	6.84
TCGA-DD-AAEB-01A	LIHC	6.81
TCGA-DD-AAEH-01A	LIHC	7.85
TCGA-CC-A8HS-01A	LIHC	7.37
TCGA-MR-A8JO-01A	LIHC	5.67
TCGA-DD-AACV-01A	LIHC	8.35
TCGA-ED-A7PZ-01A	LIHC	6.96
TCGA-EP-A12J-01A	LIHC	8.34
TCGA-2Y-A9H3-01A	LIHC	4.6
TCGA-FV-A3I0-01A	LIHC	6.66
TCGA-DD-AADI-01A	LIHC	5.4
TCGA-DD-A4NS-01A	LIHC	7.56
TCGA-2Y-A9HA-01A	LIHC	8.1
TCGA-DD-AACG-01A	LIHC	6.76
TCGA-DD-A73A-01A	LIHC	7.41
TCGA-KR-A7K7-01A	LIHC	6.76
TCGA-QA-A7B7-01A	LIHC	8.02
TCGA-BC-A110-01A	LIHC	6.2
TCGA-UB-A7ME-01A	LIHC	6.63
TCGA-G3-AAV2-01A	LIHC	7.81
TCGA-DD-A39X-01A	LIHC	6.51
TCGA-DD-AAD3-01A	LIHC	7.98
TCGA-FV-A3R2-01A	LIHC	8.45
TCGA-2Y-A9H7-01A	LIHC	6.75
TCGA-ED-A7XO-01A	LIHC	6.49
TCGA-DD-AAVZ-01A	LIHC	6.76
TCGA-ZP-A9CZ-01A	LIHC	7.28
TCGA-G3-A5SI-01A	LIHC	6.21
TCGA-MR-A520-01A	LIHC	7.58
TCGA-BC-A10U-01A	LIHC	8.25
TCGA-G3-A25Z-01A	LIHC	6.87
TCGA-DD-A39V-01A	LIHC	6.39
TCGA-DD-A4NA-01A	LIHC	8.36
TCGA-FV-A3I1-01A	LIHC	8.2
TCGA-DD-A4NL-01A	LIHC	7.73
TCGA-DD-A4NQ-01A	LIHC	8.31
TCGA-CC-A7IJ-01A	LIHC	7.15
TCGA-LG-A6GG-01A	LIHC	7.72
TCGA-CC-A123-01A	LIHC	5.46
TCGA-DD-AAE4-01A	LIHC	6.79
TCGA-GJ-A3OU-01A	LIHC	7.36
TCGA-EP-A3RK-01A	LIHC	8.16
TCGA-MI-A75H-01A	LIHC	7.52
TCGA-FV-A2QR-01A	LIHC	7.69
TCGA-ED-A5KG-01A	LIHC	7.27
TCGA-CC-A9FS-01A	LIHC	6.37
TCGA-G3-A25T-01A	LIHC	6.36
TCGA-UB-A7MC-01A	LIHC	8.11
TCGA-YA-A8S7-01A	LIHC	7.63
TCGA-DD-AACN-01A	LIHC	6.63
TCGA-5C-A9VG-01A	LIHC	6.79
TCGA-BC-A10R-01A	LIHC	7.73
TCGA-BC-A3KF-01A	LIHC	6.74

TCGA-CC-A5UE-01A	LIHC	8
TCGA-MI-A75E-01A	LIHC	7.72
TCGA-G3-A7M8-01A	LIHC	6.77
TCGA-CC-A5UD-01A	LIHC	6.61
TCGA-CC-A9FW-01A	LIHC	7.56
TCGA-BC-A10W-01A	LIHC	8.9
TCGA-2Y-A9GW-01A	LIHC	6.33
TCGA-WX-AA47-01A	LIHC	6.92
TCGA-DD-AADC-01A	LIHC	6.79
TCGA-DD-AADQ-01A	LIHC	7.37
TCGA-DD-AAE9-01A	LIHC	7.49
TCGA-ZP-A9D1-01A	LIHC	7.87
TCGA-G3-AAV5-01A	LIHC	7.39
TCGA-2Y-A9GZ-01A	LIHC	7.89
TCGA-RG-A7D4-01A	LIHC	6.97
TCGA-EP-A2KB-01A	LIHC	6.5
TCGA-DD-AAW0-01A	LIHC	7.35
TCGA-DD-AACQ-01A	LIHC	8.12
TCGA-CC-A8HU-01A	LIHC	6.78
TCGA-MI-A75G-01A	LIHC	8.35
TCGA-DD-AAD1-01A	LIHC	6.82
TCGA-CC-A8HV-01A	LIHC	7.71
TCGA-DD-A1EJ-01A	LIHC	7.71
TCGA-WQ-A9G7-01A	LIHC	7.14
TCGA-NI-A8LF-01A	LIHC	7.09
TCGA-BC-4072-01B	LIHC	8.05
TCGA-DD-AAD5-01A	LIHC	6.8
TCGA-2Y-A9H5-01A	LIHC	7.23
TCGA-BC-A217-01A	LIHC	6.91
TCGA-DD-A3A8-01A	LIHC	7.36
TCGA-CC-5258-01A	LIHC	7
TCGA-BC-A5W4-01A	LIHC	8.07
TCGA-DD-AADF-01A	LIHC	6.5
TCGA-DD-AAE1-01A	LIHC	6.95
TCGA-2V-A95S-01A	LIHC	6.69
TCGA-DD-A73D-01A	LIHC	8.07
TCGA-ZP-A9D0-01A	LIHC	7.74
TCGA-G3-A5SK-01A	LIHC	7.11
TCGA-DD-AADN-01A	LIHC	6.59
TCGA-CC-A3MC-01A	LIHC	8.02
TCGA-DD-AADS-01A	LIHC	6.33
TCGA-DD-AACX-01A	LIHC	7.91
TCGA-5R-AAAM-01A	LIHC	7.99
TCGA-EP-A2KA-01A	LIHC	7.38
TCGA-XR-A8TC-01A	LIHC	6.7
TCGA-DD-A39W-01A	LIHC	7.99
TCGA-G3-A3CH-01A	LIHC	6.14
TCGA-DD-AACO-01A	LIHC	5.86
TCGA-K7-A5RF-01A	LIHC	7.23
TCGA-DD-A4NP-01A	LIHC	7.73
TCGA-ZP-A9CY-01A	LIHC	7.56
TCGA-BC-4073-01B	LIHC	8.61
TCGA-DD-AAEE-01A	LIHC	7.96
TCGA-CC-A3MB-01A	LIHC	8.17
TCGA-RC-A7SH-01A	LIHC	5.82
TCGA-ZP-A9D4-01A	LIHC	8.05
TCGA-ED-A66Y-01A	LIHC	6.12
TCGA-DD-A114-01A	LIHC	8.32

TCGA-5R-AA1C-01A	LIHC	9.63
TCGA-BC-A10X-01A	LIHC	7.34
TCGA-DD-AACT-01A	LIHC	7.32
TCGA-DD-AAE7-01A	LIHC	7.04
TCGA-G3-A3CK-01A	LIHC	8.27
TCGA-BC-A216-01A	LIHC	6.8
TCGA-DD-A39Z-01A	LIHC	7.15
TCGA-DD-AADV-01A	LIHC	8.24
TCGA-DD-AADK-01A	LIHC	6.71
TCGA-2Y-A9H0-01A	LIHC	7.7
TCGA-DD-AAC8-01A	LIHC	8.3
TCGA-CC-A7IG-01A	LIHC	6.09
TCGA-KR-A7K8-01A	LIHC	7.18
TCGA-CC-5264-01A	LIHC	7.51
TCGA-GJ-A9DB-01A	LIHC	8.43
TCGA-DD-AADM-01A	LIHC	7.23
TCGA-CC-A1HT-01A	LIHC	7.31
TCGA-G3-AAV0-01A	LIHC	5.74
TCGA-DD-A1EE-01A	LIHC	8.88
TCGA-DD-A3A7-01A	LIHC	7.2
TCGA-DD-AAED-01A	LIHC	6.54
TCGA-DD-AACF-01A	LIHC	6.15
TCGA-2Y-A9GY-01A	LIHC	7.73
TCGA-DD-A4NO-01A	LIHC	7.85
TCGA-DD-A118-01A	LIHC	6.06
TCGA-O8-A75V-01A	LIHC	7.01
TCGA-DD-AAVQ-01A	LIHC	6.41
TCGA-BC-A69H-01A	LIHC	6.57
TCGA-DD-A73C-01A	LIHC	8.35
TCGA-ZS-A9CD-01A	LIHC	6.55
TCGA-UB-AA0U-01A	LIHC	7.41
TCGA-DD-AACE-01A	LIHC	6.45
TCGA-ED-A66X-01A	LIHC	7.07
TCGA-BW-A5NQ-01A	LIHC	8.24
TCGA-2Y-A9GU-01A	LIHC	6.59
TCGA-DD-AADU-01A	LIHC	6.93
TCGA-DD-A4NN-01A	LIHC	7.86
TCGA-DD-A73E-01A	LIHC	8.97
TCGA-RC-A6M3-01A	LIHC	7.07
TCGA-DD-AADA-01A	LIHC	7.08
TCGA-G3-AAV3-01A	LIHC	8.36
TCGA-CC-A7IK-01A	LIHC	8.36
TCGA-XR-A8TE-01A	LIHC	8.41
TCGA-2Y-A9GV-01A	LIHC	7.74
TCGA-G3-A25V-01A	LIHC	7.71
TCGA-G3-A5SM-01A	LIHC	7.2
TCGA-BW-A5NO-01A	LIHC	8.07
TCGA-G3-AAV1-01A	LIHC	7.62
TCGA-2Y-A9H9-01A	LIHC	7.59
TCGA-RC-A7S9-01A	LIHC	7.35
TCGA-RC-A6M5-01A	LIHC	6.24
TCGA-WQ-AB4B-01A	LIHC	6.06
TCGA-ED-A8O5-01A	LIHC	7.6
TCGA-2Y-A9GT-01A	LIHC	7.59
TCGA-DD-A3A2-01A	LIHC	6.79
TCGA-DD-AAW1-01A	LIHC	7.51
TCGA-G3-A7M7-01A	LIHC	6.6
TCGA-DD-AACL-01A	LIHC	7.07

TCGA-ZS-A9CG-01A	LIHC	7.25
TCGA-ED-A7PX-01A	LIHC	7.58
TCGA-FV-A3R3-01A	LIHC	6.91
TCGA-DD-A4NH-01A	LIHC	7.8
TCGA-DD-A73G-01A	LIHC	5.75
TCGA-DD-AAD6-01A	LIHC	7
TCGA-DD-AACD-01A	LIHC	5.97
TCGA-G3-A25X-01A	LIHC	5.85
TCGA-DD-A11D-01A	LIHC	8.12
TCGA-DD-A4NB-01A	LIHC	8.43
TCGA-CC-A7IE-01A	LIHC	6.67
TCGA-RC-A7SB-01A	LIHC	7.57
TCGA-RC-A6M4-01A	LIHC	7.73
TCGA-DD-A4NF-01A	LIHC	8.79
TCGA-G3-A7M9-01A	LIHC	8.33
TCGA-DD-AAVW-01A	LIHC	7.69
TCGA-PD-A5DF-01A	LIHC	8.25
TCGA-BD-A2L6-01A	LIHC	7
TCGA-XR-A8TG-01A	LIHC	7.95
TCGA-XR-A8TF-01A	LIHC	5.86
TCGA-G3-A7M6-01A	LIHC	6.34
TCGA-G3-A25Y-01A	LIHC	6.78
TCGA-UB-A7MA-01A	LIHC	6.66
TCGA-GJ-A6C0-01A	LIHC	8.3
TCGA-DD-AAVP-01A	LIHC	7.15
TCGA-DD-AADW-01A	LIHC	7.49
TCGA-DD-A1EF-01A	LIHC	7.41
TCGA-CC-5261-01A	LIHC	7.46
TCGA-DD-A11B-01A	LIHC	7.85
TCGA-DD-AAD8-01A	LIHC	8.5
TCGA-DD-AAVU-01A	LIHC	6.53
TCGA-CC-5262-01A	LIHC	7.84
TCGA-G3-AAV4-01A	LIHC	8.3
TCGA-DD-AACH-01A	LIHC	8.75
TCGA-DD-AADJ-01A	LIHC	5.86
TCGA-G3-A5SJ-01A	LIHC	7.6
TCGA-K7-AAU7-01A	LIHC	7.13
TCGA-HP-A5MZ-01A	LIHC	6.83
TCGA-DD-A1EL-01A	LIHC	8.24
TCGA-DD-A1EI-01A	LIHC	6.98
TCGA-DD-AAEI-01A	LIHC	6.97
TCGA-2Y-A9H8-01A	LIHC	7.57
TCGA-DD-A11C-01A	LIHC	6.94
TCGA-DD-AAE3-01A	LIHC	7.07
TCGA-WX-AA46-01A	LIHC	7.22
TCGA-DD-AAW2-01A	LIHC	7.42
TCGA-ZP-A9D2-01A	LIHC	8.32
TCGA-KR-A7K2-01A	LIHC	6.26
TCGA-DD-AACZ-01A	LIHC	7.35
TCGA-BC-A8YO-01A	LIHC	8.53
TCGA-DD-A3A3-01A	LIHC	6.04
TCGA-WX-AA44-01A	LIHC	7.48
TCGA-DD-A73B-01A	LIHC	6.54
TCGA-DD-A4NI-01A	LIHC	7.24
TCGA-MI-A75I-01A	LIHC	7.39
TCGA-DD-AAE0-01A	LIHC	6.92
TCGA-DD-AADB-01A	LIHC	6.86
TCGA-ED-A459-01A	LIHC	7.52

TCGA-BC-A10Y-01A	LIHC	7.51
TCGA-DD-A1EB-01A	LIHC	7.69
TCGA-DD-A11A-01A	LIHC	7.98
TCGA-BC-A3KG-01A	LIHC	8.54
TCGA-DD-A119-01A	LIHC	6.97
TCGA-DD-A115-01A	LIHC	7.51
TCGA-G3-AAV7-01A	LIHC	7.82
TCGA-BC-A10Z-01A	LIHC	9.55
TCGA-BC-A69I-01A	LIHC	7.04
TCGA-CC-A3MA-01A	LIHC	5.95
TCGA-ES-A2HT-01A	LIHC	6.25
TCGA-DD-AAD2-01A	LIHC	7.24
TCGA-DD-AAD0-01A	LIHC	7.43
TCGA-DD-A1ED-01A	LIHC	7.72
TCGA-ED-A7XP-01A	LIHC	7.51
TCGA-DD-AACC-01A	LIHC	6.49
TCGA-5R-AA1D-01A	LIHC	7.11
TCGA-69-7980-01A	LUAD	6.21
TCGA-55-1592-01A	LUAD	8.26
TCGA-55-7727-01A	LUAD	6.65
TCGA-55-6972-01A	LUAD	8.83
TCGA-49-AARE-01A	LUAD	7.5
TCGA-86-7713-01A	LUAD	6.61
TCGA-55-7913-01B	LUAD	6.48
TCGA-44-A47G-01A	LUAD	6.47
TCGA-44-2662-01B	LUAD	7.02
TCGA-44-5645-01A	LUAD	6.74
TCGA-95-A4VK-01A	LUAD	6.56
TCGA-62-A46O-01A	LUAD	7.69
TCGA-62-A46S-01A	LUAD	7.28
TCGA-05-4434-01A	LUAD	7.28
TCGA-53-7626-01A	LUAD	7.09
TCGA-55-8087-01A	LUAD	6.57
TCGA-86-8668-01A	LUAD	6.33
TCGA-55-A493-01A	LUAD	5.94
TCGA-55-8616-01A	LUAD	6.44
TCGA-55-7570-01A	LUAD	7.72
TCGA-64-1678-01A	LUAD	6.76
TCGA-05-4250-01A	LUAD	6.68
TCGA-86-8278-01A	LUAD	6.98
TCGA-75-6203-01A	LUAD	6.69
TCGA-05-4433-01A	LUAD	7.44
TCGA-86-8673-01A	LUAD	6.6
TCGA-NJ-A55A-01A	LUAD	5.88
TCGA-55-8097-01A	LUAD	5.74
TCGA-73-4668-01A	LUAD	6.8
TCGA-MP-A4TE-01A	LUAD	6.82
TCGA-86-8671-01A	LUAD	6.22
TCGA-55-8508-01A	LUAD	5.39
TCGA-44-2659-01A	LUAD	5.87
TCGA-50-5055-01A	LUAD	5.79
TCGA-78-7153-01A	LUAD	6.5
TCGA-05-4415-01A	LUAD	8.3
TCGA-78-7154-01A	LUAD	8.91
TCGA-55-8507-01A	LUAD	7.33
TCGA-55-8091-01A	LUAD	6.51
TCGA-50-5946-01A	LUAD	5.71
TCGA-75-6206-01A	LUAD	7.98

TCGA-78-7536-01A	LUAD	9.61
TCGA-38-4627-01A	LUAD	6.92
TCGA-91-6831-01A	LUAD	5.77
TCGA-55-1595-01A	LUAD	5.79
TCGA-44-5645-01B	LUAD	6.01
TCGA-62-A472-01A	LUAD	7.22
TCGA-67-6215-01A	LUAD	7.07
TCGA-55-6985-01A	LUAD	6.77
TCGA-55-7815-01A	LUAD	7.53
TCGA-78-7148-01A	LUAD	7.41
TCGA-97-A4LX-01A	LUAD	6.53
TCGA-50-5936-01A	LUAD	7.42
TCGA-05-4432-01A	LUAD	6.53
TCGA-55-6984-01A	LUAD	6.8
TCGA-78-7167-01A	LUAD	8.09
TCGA-44-2657-01A	LUAD	6.37
TCGA-49-4510-01A	LUAD	6.41
TCGA-44-7671-01A	LUAD	8.43
TCGA-97-8175-01A	LUAD	6.37
TCGA-69-8254-01A	LUAD	6.97
TCGA-55-8505-01A	LUAD	8.23
TCGA-62-A46V-01A	LUAD	6.58
TCGA-38-4630-01A	LUAD	7.35
TCGA-44-2665-01B	LUAD	6.21
TCGA-44-2665-01A	LUAD	6.33
TCGA-4B-A93V-01A	LUAD	6.47
TCGA-44-A4SS-01A	LUAD	8.68
TCGA-97-7937-01A	LUAD	7.09
TCGA-64-5774-01A	LUAD	6.01
TCGA-50-5044-01A	LUAD	5.7
TCGA-78-7220-01A	LUAD	9.12
TCGA-L9-A743-01A	LUAD	6
TCGA-64-5815-01A	LUAD	6.45
TCGA-55-7574-01A	LUAD	5.71
TCGA-97-8552-01A	LUAD	6.03
TCGA-44-7659-01A	LUAD	6.4
TCGA-93-8067-01A	LUAD	8.59
TCGA-95-7944-01A	LUAD	5.86
TCGA-49-4506-01A	LUAD	8.01
TCGA-55-7995-01A	LUAD	8.68
TCGA-97-8547-01A	LUAD	5.8
TCGA-55-7283-01A	LUAD	6.8
TCGA-44-7661-01A	LUAD	6.61
TCGA-95-7043-01A	LUAD	9.4
TCGA-50-8457-01A	LUAD	5.79
TCGA-MN-A4N4-01A	LUAD	6.9
TCGA-91-6840-01A	LUAD	7.11
TCGA-50-5946-02A	LUAD	5.22
TCGA-80-5611-01A	LUAD	7.03
TCGA-99-AA5R-01A	LUAD	5.45
TCGA-86-6562-01A	LUAD	6.83
TCGA-86-A4JF-01A	LUAD	6.84
TCGA-67-3770-01A	LUAD	6.91
TCGA-62-A470-01A	LUAD	7.67
TCGA-78-7542-01A	LUAD	6.44
TCGA-93-7347-01A	LUAD	7.01
TCGA-91-A4BC-01A	LUAD	5.83
TCGA-44-3398-01A	LUAD	6.76

TCGA-38-6178-01A	LUAD	6.63
TCGA-05-4405-01A	LUAD	7.28
TCGA-49-AAR2-01A	LUAD	5.86
TCGA-78-7155-01A	LUAD	8.44
TCGA-73-4658-01A	LUAD	6.52
TCGA-69-7765-01A	LUAD	5.87
TCGA-49-4487-01A	LUAD	6.13
TCGA-97-A4M1-01A	LUAD	6.31
TCGA-44-2668-01B	LUAD	5.61
TCGA-86-8672-01A	LUAD	8.15
TCGA-91-7771-01A	LUAD	6.91
TCGA-73-4659-01A	LUAD	7.68
TCGA-97-A4M7-01A	LUAD	6.03
TCGA-44-2668-01A	LUAD	6.48
TCGA-86-8073-01A	LUAD	7.61
TCGA-64-5775-01A	LUAD	7.05
TCGA-05-4410-01A	LUAD	6.5
TCGA-44-2662-01A	LUAD	6.76
TCGA-50-6593-01A	LUAD	7.32
TCGA-97-A4M0-01A	LUAD	7.53
TCGA-55-7728-01A	LUAD	5.98
TCGA-44-2666-01B	LUAD	5.83
TCGA-J2-A4AE-01A	LUAD	5.99
TCGA-95-7947-01A	LUAD	8.14
TCGA-55-6543-01A	LUAD	6.64
TCGA-MP-A4TI-01A	LUAD	6.47
TCGA-78-7146-01A	LUAD	6.61
TCGA-95-7039-01A	LUAD	6.3
TCGA-50-6673-01A	LUAD	6.24
TCGA-55-8301-01A	LUAD	6.02
TCGA-93-A4JN-01A	LUAD	6.11
TCGA-86-7701-01A	LUAD	5.59
TCGA-55-A490-01A	LUAD	4.51
TCGA-05-4390-01A	LUAD	5.8
TCGA-44-6779-01A	LUAD	7.57
TCGA-97-8172-01A	LUAD	6.08
TCGA-64-1681-01A	LUAD	6.82
TCGA-78-7160-01A	LUAD	8.88
TCGA-44-A4SU-01A	LUAD	5.92
TCGA-38-4628-01A	LUAD	7.22
TCGA-55-6969-01A	LUAD	5.57
TCGA-44-2666-01A	LUAD	6.3
TCGA-75-7030-01A	LUAD	5.75
TCGA-50-5051-01A	LUAD	7.15
TCGA-J2-A4AG-01A	LUAD	5.72
TCGA-97-7938-01A	LUAD	7.18
TCGA-35-3615-01A	LUAD	7.99
TCGA-44-2661-01A	LUAD	7.12
TCGA-50-5941-01A	LUAD	7.01
TCGA-64-1676-01A	LUAD	6.4
TCGA-50-5932-01A	LUAD	7.24
TCGA-55-8207-01A	LUAD	6.53
TCGA-L9-A50W-01A	LUAD	6.68
TCGA-97-7941-01A	LUAD	6.31
TCGA-97-7554-01A	LUAD	5.92
TCGA-55-7910-01A	LUAD	6.73
TCGA-50-8459-01A	LUAD	5.8
TCGA-44-7662-01A	LUAD	7.17

TCGA-78-7166-01A	LUAD	5.97
TCGA-55-8513-01A	LUAD	6.96
TCGA-49-4514-01A	LUAD	7.92
TCGA-78-7152-01A	LUAD	6.67
TCGA-67-6216-01A	LUAD	7.09
TCGA-55-7907-01A	LUAD	7.04
TCGA-44-6776-01A	LUAD	7.52
TCGA-49-4494-01A	LUAD	6.99
TCGA-05-4403-01A	LUAD	7.03
TCGA-75-5147-01A	LUAD	7.19
TCGA-62-A46R-01A	LUAD	6.72
TCGA-05-4418-01A	LUAD	6.94
TCGA-49-4488-01A	LUAD	6.41
TCGA-69-7973-01A	LUAD	7.01
TCGA-86-8280-01A	LUAD	6.84
TCGA-05-5715-01A	LUAD	7.05
TCGA-93-7348-01A	LUAD	6.59
TCGA-78-7147-01A	LUAD	6.4
TCGA-69-7978-01A	LUAD	6.35
TCGA-55-6712-01A	LUAD	6.54
TCGA-MP-A4TK-01A	LUAD	5.63
TCGA-44-2656-01A	LUAD	7.25
TCGA-75-7031-01A	LUAD	6.97
TCGA-78-7158-01A	LUAD	6.32
TCGA-49-4507-01A	LUAD	7.38
TCGA-55-8092-01A	LUAD	6.6
TCGA-NJ-A4YQ-01A	LUAD	7.86
TCGA-91-8499-01A	LUAD	6.96
TCGA-91-8497-01A	LUAD	6.05
TCGA-49-6761-01A	LUAD	5.3
TCGA-53-7624-01A	LUAD	7.54
TCGA-44-3919-01A	LUAD	6.56
TCGA-86-8281-01A	LUAD	8.6
TCGA-67-3774-01A	LUAD	6.26
TCGA-05-4426-01A	LUAD	5.99
TCGA-MP-A4TD-01A	LUAD	6.43
TCGA-86-8055-01A	LUAD	6.8
TCGA-05-5428-01A	LUAD	8.81
TCGA-75-6214-01A	LUAD	6.55
TCGA-55-6642-01A	LUAD	5.14
TCGA-69-8253-01A	LUAD	7
TCGA-L9-A443-01A	LUAD	6.31
TCGA-49-AAR9-01A	LUAD	7.19
TCGA-91-6848-01A	LUAD	6.35
TCGA-MP-A4SV-01A	LUAD	6.79
TCGA-38-4632-01A	LUAD	7.18
TCGA-05-4395-01A	LUAD	7.67
TCGA-49-AAR4-01A	LUAD	6.14
TCGA-95-8039-01A	LUAD	7.25
TCGA-64-1677-01A	LUAD	5.41
TCGA-86-8075-01A	LUAD	6.9
TCGA-05-4427-01A	LUAD	6.8
TCGA-50-6594-01A	LUAD	7.44
TCGA-53-A4EZ-01A	LUAD	7.02
TCGA-38-7271-01A	LUAD	6.01
TCGA-MP-A4TJ-01A	LUAD	6.15
TCGA-86-8585-01A	LUAD	6.03
TCGA-55-6982-01A	LUAD	6.58

TCGA-05-4398-01A	LUAD	7.78
TCGA-97-A4M6-01A	LUAD	6.27
TCGA-44-6775-01A	LUAD	6.87
TCGA-L4-A4E5-01A	LUAD	5.24
TCGA-49-AAR0-01A	LUAD	7.66
TCGA-35-4122-01A	LUAD	6.35
TCGA-O1-A52J-01A	LUAD	7.04
TCGA-NJ-A7XG-01A	LUAD	6.38
TCGA-86-8054-01A	LUAD	5.58
TCGA-50-5931-01A	LUAD	5.71
TCGA-55-8620-01A	LUAD	6.29
TCGA-69-8255-01A	LUAD	7.07
TCGA-62-A46Y-01A	LUAD	7.51
TCGA-80-5607-01A	LUAD	6.39
TCGA-86-A4P8-01A	LUAD	6.66
TCGA-91-8496-01A	LUAD	7.21
TCGA-93-A4JQ-01A	LUAD	6.31
TCGA-69-A59K-01A	LUAD	7.09
TCGA-55-7281-01A	LUAD	6.21
TCGA-05-4397-01A	LUAD	7.16
TCGA-49-6767-01A	LUAD	6.72
TCGA-35-4123-01A	LUAD	6.11
TCGA-78-8648-01A	LUAD	5.85
TCGA-62-8394-01A	LUAD	6.53
TCGA-55-8204-01A	LUAD	5.86
TCGA-MP-A4T9-01A	LUAD	6.54
TCGA-MN-A4N1-01A	LUAD	6.22
TCGA-50-5942-01A	LUAD	6.24
TCGA-44-6148-01A	LUAD	6.41
TCGA-55-7903-01A	LUAD	6.41
TCGA-NJ-A55O-01A	LUAD	6.68
TCGA-44-6147-01A	LUAD	6.2
TCGA-78-7539-01A	LUAD	5.94
TCGA-55-6971-01A	LUAD	6.06
TCGA-75-5122-01A	LUAD	7.31
TCGA-62-A46U-01A	LUAD	6.79
TCGA-86-8076-01A	LUAD	6.15
TCGA-55-7573-01A	LUAD	6.97
TCGA-67-3772-01A	LUAD	6.98
TCGA-97-7547-01A	LUAD	7.65
TCGA-44-4112-01A	LUAD	6.23
TCGA-44-3917-01B	LUAD	6.87
TCGA-78-7163-01A	LUAD	6.44
TCGA-55-6970-01A	LUAD	8.32
TCGA-99-8032-01A	LUAD	5.63
TCGA-MP-A4T6-01A	LUAD	5.38
TCGA-55-8085-01A	LUAD	7.73
TCGA-78-7537-01A	LUAD	6.7
TCGA-55-7994-01A	LUAD	7.17
TCGA-55-8094-01A	LUAD	7.03
TCGA-05-4425-01A	LUAD	6.64
TCGA-44-3917-01A	LUAD	6.04
TCGA-55-7914-01A	LUAD	5.93
TCGA-55-8514-01A	LUAD	5.98
TCGA-05-4402-01A	LUAD	7.06
TCGA-69-7761-01A	LUAD	6.71
TCGA-50-5933-01A	LUAD	7.86
TCGA-97-A4M5-01A	LUAD	6.43

TCGA-55-1594-01A	LUAD	6.75
TCGA-44-4112-01B	LUAD	5.59
TCGA-44-8117-01A	LUAD	6.71
TCGA-75-7025-01A	LUAD	6.74
TCGA-44-3918-01B	LUAD	6.07
TCGA-73-7499-01A	LUAD	6.82
TCGA-80-5608-01A	LUAD	7.2
TCGA-MP-A4TH-01A	LUAD	6.07
TCGA-05-5429-01A	LUAD	7.86
TCGA-91-6830-01A	LUAD	6.6
TCGA-55-8619-01A	LUAD	5.83
TCGA-55-6979-01A	LUAD	7.27
TCGA-86-8359-01A	LUAD	6.65
TCGA-95-8494-01A	LUAD	5.87
TCGA-50-6590-01A	LUAD	5.49
TCGA-91-6829-01A	LUAD	6.58
TCGA-55-7576-01A	LUAD	7.14
TCGA-55-8615-01A	LUAD	5.93
TCGA-83-5908-01A	LUAD	6.33
TCGA-95-A4VN-01A	LUAD	6.7
TCGA-55-A492-01A	LUAD	7.45
TCGA-44-7672-01A	LUAD	5.58
TCGA-78-7633-01A	LUAD	8.45
TCGA-05-4430-01A	LUAD	6.75
TCGA-55-A48X-01A	LUAD	7.09
TCGA-NJ-A4YP-01A	LUAD	7.19
TCGA-05-4420-01A	LUAD	5.68
TCGA-44-2656-01B	LUAD	6.01
TCGA-44-6146-01A	LUAD	7.02
TCGA-44-6146-01B	LUAD	6.67
TCGA-62-A46P-01A	LUAD	6.29
TCGA-73-4676-01A	LUAD	8.09
TCGA-55-1596-01A	LUAD	9.53
TCGA-55-8511-01A	LUAD	6.43
TCGA-44-5644-01A	LUAD	5.11
TCGA-49-6742-01A	LUAD	7.77
TCGA-55-8512-01A	LUAD	6.7
TCGA-97-8179-01A	LUAD	8.06
TCGA-95-7562-01A	LUAD	7.67
TCGA-50-5072-01A	LUAD	8.08
TCGA-78-8662-01A	LUAD	7.51
TCGA-75-5125-01A	LUAD	6.81
TCGA-MN-A4N5-01A	LUAD	6.87
TCGA-86-8279-01A	LUAD	7.38
TCGA-55-7724-01A	LUAD	6.75
TCGA-73-4662-01A	LUAD	6.94
TCGA-73-A9RS-01A	LUAD	7.18
TCGA-78-8640-01A	LUAD	8.29
TCGA-NJ-A4YI-01A	LUAD	7.28
TCGA-38-4631-01A	LUAD	7.54
TCGA-50-5930-01A	LUAD	6.31
TCGA-50-5066-02A	LUAD	8.49
TCGA-05-4382-01A	LUAD	6.95
TCGA-L9-A5IP-01A	LUAD	7.73
TCGA-64-5779-01A	LUAD	5.06
TCGA-75-6212-01A	LUAD	7.3
TCGA-55-A4DF-01A	LUAD	6.72
TCGA-44-6774-01A	LUAD	6.32

TCGA-78-7149-01A	LUAD	7.08
TCGA-97-A4M2-01A	LUAD	6.9
TCGA-38-A44F-01A	LUAD	5.92
TCGA-38-4629-01A	LUAD	6.71
TCGA-38-4625-01A	LUAD	8.07
TCGA-50-5939-01A	LUAD	7.4
TCGA-55-A48Y-01A	LUAD	7.84
TCGA-MP-A4SW-01A	LUAD	7.78
TCGA-97-8176-01A	LUAD	8.31
TCGA-55-8089-01A	LUAD	6.3
TCGA-64-1680-01A	LUAD	7.18
TCGA-97-7552-01A	LUAD	6.75
TCGA-73-4670-01A	LUAD	7.43
TCGA-93-A4JP-01A	LUAD	6.63
TCGA-69-7764-01A	LUAD	7.3
TCGA-55-8096-01A	LUAD	5.9
TCGA-55-6987-01A	LUAD	6.51
TCGA-S2-AA1A-01A	LUAD	5.14
TCGA-55-6980-01A	LUAD	6.38
TCGA-J2-8192-01A	LUAD	6.7
TCGA-49-6744-01A	LUAD	6.26
TCGA-75-5146-01A	LUAD	6.69
TCGA-50-5935-01A	LUAD	6.29
TCGA-78-7162-01A	LUAD	7.74
TCGA-55-7911-01A	LUAD	6.62
TCGA-L4-A4E6-01A	LUAD	6.76
TCGA-MP-A4T4-01A	LUAD	5.96
TCGA-64-5781-01A	LUAD	7.42
TCGA-93-A4JO-01A	LUAD	5.48
TCGA-05-4422-01A	LUAD	7.45
TCGA-44-3396-01A	LUAD	5.86
TCGA-55-A4DG-01A	LUAD	6.73
TCGA-75-7027-01A	LUAD	8.71
TCGA-55-8506-01A	LUAD	6.26
TCGA-MP-A4T7-01A	LUAD	6.7
TCGA-86-A4D0-01A	LUAD	7.86
TCGA-86-7953-01A	LUAD	7.67
TCGA-78-7540-01A	LUAD	6.71
TCGA-55-7816-01A	LUAD	6.71
TCGA-78-7159-01A	LUAD	6.4
TCGA-44-6778-01A	LUAD	6.7
TCGA-86-8074-01A	LUAD	6.78
TCGA-69-7974-01A	LUAD	7.32
TCGA-78-7535-01A	LUAD	5.94
TCGA-73-4666-01A	LUAD	8.11
TCGA-50-5068-01A	LUAD	6.84
TCGA-35-5375-01A	LUAD	7.49
TCGA-55-7227-01A	LUAD	6.02
TCGA-L9-A7SV-01A	LUAD	6.79
TCGA-91-6835-01A	LUAD	7.17
TCGA-71-8520-01A	LUAD	7.02
TCGA-44-6777-01A	LUAD	6.75
TCGA-97-7553-01A	LUAD	7.07
TCGA-62-8398-01A	LUAD	7.83
TCGA-53-7813-01A	LUAD	6.12
TCGA-44-6775-01C	LUAD	6.6
TCGA-97-8171-01A	LUAD	7.07
TCGA-62-8402-01A	LUAD	6.6

TCGA-55-A57B-01A	LUAD	7.31
TCGA-67-3773-01A	LUAD	6.32
TCGA-95-7567-01A	LUAD	6.37
TCGA-38-4626-01A	LUAD	7.54
TCGA-J2-8194-01A	LUAD	8.53
TCGA-50-5944-01A	LUAD	7.22
TCGA-75-6211-01A	LUAD	7.9
TCGA-50-5066-01A	LUAD	6.69
TCGA-55-6986-01A	LUAD	6.52
TCGA-86-8056-01A	LUAD	7.14
TCGA-73-4677-01A	LUAD	8.55
TCGA-97-A4M3-01A	LUAD	7.73
TCGA-05-4249-01A	LUAD	6.54
TCGA-78-7145-01A	LUAD	7.27
TCGA-55-6978-01A	LUAD	6.72
TCGA-49-AARO-01A	LUAD	5.55
TCGA-67-4679-01B	LUAD	7.03
TCGA-71-6725-01A	LUAD	5.95
TCGA-50-8460-01A	LUAD	6.7
TCGA-86-8358-01A	LUAD	6.48
TCGA-78-7143-01A	LUAD	6.81
TCGA-55-6983-01A	LUAD	6.89
TCGA-49-4486-01A	LUAD	7.82
TCGA-L9-A444-01A	LUAD	5.83
TCGA-86-6851-01A	LUAD	6.29
TCGA-86-7714-01A	LUAD	7.29
TCGA-49-AAR3-01A	LUAD	6.04
TCGA-75-6207-01A	LUAD	7.19
TCGA-44-7669-01A	LUAD	7.22
TCGA-44-6145-01A	LUAD	6.93
TCGA-62-8395-01A	LUAD	7.99
TCGA-05-4424-01A	LUAD	9.06
TCGA-55-8090-01A	LUAD	5.89
TCGA-62-8399-01A	LUAD	6.68
TCGA-NJ-A4YF-01A	LUAD	5.38
TCGA-44-8119-01A	LUAD	5.73
TCGA-55-8299-01A	LUAD	6.33
TCGA-05-5423-01A	LUAD	7.47
TCGA-05-4244-01A	LUAD	6.87
TCGA-95-A4VP-01A	LUAD	5.51
TCGA-86-A4P7-01A	LUAD	5.8
TCGA-50-5045-01A	LUAD	6
TCGA-50-5049-01A	LUAD	6.42
TCGA-69-8453-01A	LUAD	6.85
TCGA-05-4396-01A	LUAD	8.36
TCGA-78-7161-01A	LUAD	5.4
TCGA-55-8205-01A	LUAD	7.28
TCGA-55-8203-01A	LUAD	5.95
TCGA-55-A491-01A	LUAD	5.97
TCGA-91-6828-01A	LUAD	6.79
TCGA-55-A494-01A	LUAD	6.24
TCGA-49-AARN-01A	LUAD	6.2
TCGA-55-5899-01A	LUAD	7.26
TCGA-MP-A4TC-01A	LUAD	6.31
TCGA-55-7725-01A	LUAD	6.55
TCGA-05-4384-01A	LUAD	8.03
TCGA-78-7150-01A	LUAD	9.4
TCGA-99-7458-01A	LUAD	6.63

TCGA-49-4512-01A	LUAD	5.61
TCGA-44-7660-01A	LUAD	6.24
TCGA-49-4505-01A	LUAD	6.15
TCGA-50-6597-01A	LUAD	6.19
TCGA-62-A471-01A	LUAD	8.24
TCGA-64-5778-01A	LUAD	7.08
TCGA-55-7726-01A	LUAD	5.69
TCGA-49-AARQ-01A	LUAD	6.56
TCGA-99-8033-01A	LUAD	7.8
TCGA-62-8397-01A	LUAD	6.28
TCGA-50-6591-01A	LUAD	5.01
TCGA-55-8206-01A	LUAD	7.01
TCGA-49-AARR-01A	LUAD	5.56
TCGA-55-8614-01A	LUAD	6.52
TCGA-99-8028-01A	LUAD	5.71
TCGA-91-6847-01A	LUAD	5.97
TCGA-86-7711-01A	LUAD	7.36
TCGA-55-8208-01A	LUAD	6.72
TCGA-05-5420-01A	LUAD	6.85
TCGA-78-7156-01A	LUAD	8.95
TCGA-97-8174-01A	LUAD	7.06
TCGA-MP-A4SY-01A	LUAD	6.17
TCGA-44-6147-01B	LUAD	5.82
TCGA-55-8621-01A	LUAD	6.32
TCGA-91-6849-01A	LUAD	7.57
TCGA-44-7670-01A	LUAD	6.97
TCGA-MP-A4TF-01A	LUAD	6.46
TCGA-86-8669-01A	LUAD	7.31
TCGA-44-8120-01A	LUAD	7.4
TCGA-99-8025-01A	LUAD	6
TCGA-NJ-A55R-01A	LUAD	7.1
TCGA-55-8302-01A	LUAD	6.69
TCGA-64-1679-01A	LUAD	6.73
TCGA-86-A456-01A	LUAD	6.18
TCGA-50-6595-01A	LUAD	6.17
TCGA-78-8660-01A	LUAD	6.25
TCGA-50-7109-01A	LUAD	5.96
TCGA-44-A479-01A	LUAD	6.16
TCGA-75-6205-01A	LUAD	6.9
TCGA-05-5425-01A	LUAD	7.3
TCGA-05-4417-01A	LUAD	6.72
TCGA-49-6743-01A	LUAD	8.69
TCGA-69-7979-01A	LUAD	8
TCGA-78-8655-01A	LUAD	6.75
TCGA-05-4389-01A	LUAD	7.38
TCGA-55-6975-01A	LUAD	7.82
TCGA-44-A47B-01A	LUAD	5.44
TCGA-75-5126-01A	LUAD	7.17
TCGA-95-7948-01A	LUAD	8.17
TCGA-49-AAQV-01A	LUAD	6.82
TCGA-55-8510-01A	LUAD	6.78
TCGA-L9-A8F4-01A	LUAD	6.25
TCGA-73-7498-01A	LUAD	7.73
TCGA-49-6745-01A	LUAD	7.27
TCGA-73-4675-01A	LUAD	6.74
TCGA-44-3918-01A	LUAD	6.23
TCGA-49-4490-01A	LUAD	6.1
TCGA-55-6968-01A	LUAD	6.27

TCGA-44-A47A-01A	LUAD	7.16
TCGA-44-7667-01A	LUAD	5.91
TCGA-NJ-A4YG-01A	LUAD	6.39
TCGA-MP-A4T8-01A	LUAD	6.01
TCGA-69-7760-01A	LUAD	5.6
TCGA-MP-A5C7-01A	LUAD	7.63
TCGA-MP-A4TA-01A	LUAD	8.75
TCGA-49-4501-01A	LUAD	6.86
TCGA-55-6981-01A	LUAD	6.31
TCGA-44-2655-01A	LUAD	7.89
TCGA-91-A4BD-01A	LUAD	6.46
TCGA-97-8177-01A	LUAD	7.75
TCGA-67-6217-01A	LUAD	6.55
TCGA-55-7284-01B	LUAD	6.94
TCGA-86-7955-01A	LUAD	7.07
TCGA-91-6836-01A	LUAD	7.17
TCGA-67-3771-01A	LUAD	7.65
TCGA-50-6592-01A	LUAD	7.43
TCGA-86-8674-01A	LUAD	8.28
TCGA-86-7954-01A	LUAD	7.04
TCGA-J2-A4AD-01A	LUAD	7.44
TCGA-97-7546-01A	LUAD	6.3
TCGA-55-A48Z-01A	LUAD	5.55
TCGA-69-7763-01A	LUAD	6.1
TCGA-44-5643-01A	LUAD	7.6
TCGA-92-7340-01A	LUSC	5.88
TCGA-O2-A52Q-01A	LUSC	6.73
TCGA-85-8479-01A	LUSC	6.53
TCGA-22-4594-01A	LUSC	7.55
TCGA-63-A5MB-01A	LUSC	7.46
TCGA-21-5783-01A	LUSC	6.17
TCGA-18-4083-01A	LUSC	5.21
TCGA-85-A53L-01A	LUSC	6.62
TCGA-77-8148-01A	LUSC	6.23
TCGA-77-A5G7-01B	LUSC	5.54
TCGA-22-4593-01A	LUSC	6.95
TCGA-68-8250-01A	LUSC	7.27
TCGA-77-8150-01A	LUSC	5.56
TCGA-66-2793-01A	LUSC	6.64
TCGA-33-A4WN-01A	LUSC	7.93
TCGA-34-8456-01A	LUSC	6.54
TCGA-77-A5FZ-01A	LUSC	5.72
TCGA-34-2596-01A	LUSC	8.49
TCGA-O2-A5IB-01A	LUSC	6.56
TCGA-66-2773-01A	LUSC	7.88
TCGA-43-5670-01A	LUSC	6.13
TCGA-NC-A5HK-01A	LUSC	6.3
TCGA-66-2789-01A	LUSC	7.2
TCGA-85-A50Z-01A	LUSC	6.84
TCGA-22-1017-01A	LUSC	7.01
TCGA-85-A4JB-01A	LUSC	6.12
TCGA-33-AASB-01A	LUSC	7.4
TCGA-NK-A5CR-01A	LUSC	6.72
TCGA-34-5234-01A	LUSC	6.55
TCGA-85-6175-01A	LUSC	6.35
TCGA-77-7338-01A	LUSC	7.27
TCGA-NK-A7XE-01A	LUSC	6.6
TCGA-63-A5MY-01A	LUSC	6.46

TCGA-56-7222-01A	LUSC	6.59
TCGA-33-4586-01A	LUSC	8.48
TCGA-51-4079-01A	LUSC	7.85
TCGA-22-1005-01A	LUSC	5.01
TCGA-22-5485-01A	LUSC	6.51
TCGA-60-2704-01A	LUSC	6.45
TCGA-56-8083-01A	LUSC	6
TCGA-85-6561-01A	LUSC	6.57
TCGA-66-2737-01A	LUSC	7.67
TCGA-39-5036-01A	LUSC	6.98
TCGA-21-5784-01A	LUSC	6.65
TCGA-85-8287-01A	LUSC	5.81
TCGA-33-4589-01A	LUSC	6.05
TCGA-60-2707-01A	LUSC	6.86
TCGA-63-5131-01A	LUSC	7.04
TCGA-63-A5MH-01A	LUSC	5.48
TCGA-22-4609-01A	LUSC	6.68
TCGA-90-7766-01A	LUSC	6.33
TCGA-98-A53J-01A	LUSC	7.49
TCGA-58-8388-01A	LUSC	5.89
TCGA-34-2608-01A	LUSC	6.85
TCGA-77-8154-01A	LUSC	7.26
TCGA-56-8623-01A	LUSC	6.42
TCGA-77-8140-01A	LUSC	6.34
TCGA-33-4587-01A	LUSC	7.04
TCGA-NC-A5HP-01A	LUSC	6.31
TCGA-60-2709-01A	LUSC	6.65
TCGA-56-1622-01A	LUSC	7.33
TCGA-NC-A5HQ-01A	LUSC	6.37
TCGA-NC-A5HO-01A	LUSC	6.7
TCGA-85-A4JC-01A	LUSC	5.68
TCGA-66-2792-01A	LUSC	7.23
TCGA-63-A5MT-01A	LUSC	6.33
TCGA-34-A5IX-01A	LUSC	5.65
TCGA-43-6647-01A	LUSC	5.77
TCGA-98-8023-01A	LUSC	6.25
TCGA-43-A56U-01A	LUSC	4.22
TCGA-58-8392-01A	LUSC	7.72
TCGA-22-5492-01A	LUSC	7.17
TCGA-22-5478-01A	LUSC	6.67
TCGA-92-7341-01A	LUSC	7.24
TCGA-60-2708-01A	LUSC	6.02
TCGA-68-A59J-01A	LUSC	7.51
TCGA-94-A4VJ-01A	LUSC	5.78
TCGA-60-2720-01A	LUSC	4.92
TCGA-96-8170-01A	LUSC	6.86
TCGA-94-8035-01A	LUSC	6.54
TCGA-39-5029-01A	LUSC	7.49
TCGA-85-8052-01A	LUSC	6.92
TCGA-60-2697-01A	LUSC	6.07
TCGA-34-5236-01A	LUSC	7.63
TCGA-39-5024-01A	LUSC	6.65
TCGA-51-4081-01A	LUSC	6.66
TCGA-52-7622-01A	LUSC	7.71
TCGA-60-2726-01A	LUSC	6.63
TCGA-21-1076-01A	LUSC	7.85
TCGA-21-1070-01A	LUSC	6.11
TCGA-56-A5DR-01A	LUSC	6.81

TCGA-22-4605-01A	LUSC	6.81
TCGA-63-7023-01A	LUSC	7.17
TCGA-77-8138-01A	LUSC	6.65
TCGA-85-7843-01A	LUSC	6.5
TCGA-34-8455-01A	LUSC	6.41
TCGA-66-2765-01A	LUSC	6.04
TCGA-56-8624-01A	LUSC	6.74
TCGA-43-2578-01A	LUSC	6.65
TCGA-63-7020-01A	LUSC	6.8
TCGA-85-8481-01A	LUSC	6.22
TCGA-43-6770-01A	LUSC	7.05
TCGA-77-7142-01A	LUSC	7.12
TCGA-94-7033-01A	LUSC	6.24
TCGA-18-3421-01A	LUSC	6.2
TCGA-85-8666-01A	LUSC	7.7
TCGA-22-1016-01A	LUSC	6.93
TCGA-22-5480-01A	LUSC	6.33
TCGA-79-5596-01A	LUSC	6.28
TCGA-NC-A5HJ-01A	LUSC	6.55
TCGA-21-1081-01A	LUSC	6.7
TCGA-22-4613-01A	LUSC	6.1
TCGA-18-5592-01A	LUSC	7.29
TCGA-77-8153-01A	LUSC	6.41
TCGA-39-5037-01A	LUSC	6.93
TCGA-63-A5MW-01A	LUSC	5.44
TCGA-56-8201-01A	LUSC	5.85
TCGA-56-7223-01A	LUSC	7.61
TCGA-68-7755-01A	LUSC	6.88
TCGA-18-4086-01A	LUSC	6.25
TCGA-66-2756-01A	LUSC	6.69
TCGA-85-8353-01A	LUSC	6.27
TCGA-96-7545-01A	LUSC	7.38
TCGA-77-8008-01A	LUSC	5.79
TCGA-66-2800-01A	LUSC	7.4
TCGA-NC-A5HF-01A	LUSC	6.31
TCGA-39-5011-01A	LUSC	8.26
TCGA-98-A53C-01A	LUSC	5.76
TCGA-18-5595-01A	LUSC	7.17
TCGA-22-4601-01A	LUSC	6.17
TCGA-22-5491-01A	LUSC	7.73
TCGA-94-A5I4-01A	LUSC	8.39
TCGA-33-4547-01A	LUSC	6.76
TCGA-92-8063-01A	LUSC	7.43
TCGA-33-AASI-01A	LUSC	5.19
TCGA-37-4141-01A	LUSC	6.16
TCGA-34-5239-01A	LUSC	5.99
TCGA-85-8070-01A	LUSC	7.68
TCGA-66-2769-01A	LUSC	6.52
TCGA-NC-A5HL-01A	LUSC	7.06
TCGA-63-A5MV-01A	LUSC	6.31
TCGA-85-A4CL-01A	LUSC	6.16
TCGA-85-A50M-01A	LUSC	6.74
TCGA-60-2716-01A	LUSC	7.24
TCGA-6A-AB49-01A	LUSC	5.66
TCGA-85-A513-01A	LUSC	5.5
TCGA-34-5928-01A	LUSC	6.03
TCGA-90-7769-01A	LUSC	6.91
TCGA-O2-A52V-01A	LUSC	7.62

TCGA-60-2722-01A	LUSC	7.96
TCGA-90-A4ED-01A	LUSC	5.31
TCGA-39-5030-01A	LUSC	6.39
TCGA-85-6560-01A	LUSC	7.75
TCGA-56-A62T-01A	LUSC	6.43
TCGA-51-4080-01A	LUSC	7.83
TCGA-56-8304-01A	LUSC	6.48
TCGA-66-2780-01A	LUSC	6.19
TCGA-85-A4CN-01A	LUSC	7.04
TCGA-O2-A52W-01A	LUSC	6.73
TCGA-56-A49D-01A	LUSC	6.31
TCGA-66-2771-01A	LUSC	7.16
TCGA-85-8288-01A	LUSC	5.64
TCGA-LA-A446-01A	LUSC	6.91
TCGA-56-7221-01A	LUSC	6.71
TCGA-66-2763-01A	LUSC	6.58
TCGA-96-7544-01A	LUSC	8.85
TCGA-77-7465-01A	LUSC	5.9
TCGA-63-A5MI-01A	LUSC	5.87
TCGA-77-8139-01A	LUSC	5.98
TCGA-22-4591-01A	LUSC	8.33
TCGA-37-4132-01A	LUSC	5.42
TCGA-52-7811-01A	LUSC	6.59
TCGA-98-8021-01A	LUSC	7.42
TCGA-70-6723-01A	LUSC	8.03
TCGA-60-2711-01A	LUSC	7.17
TCGA-18-4721-01A	LUSC	8.18
TCGA-56-7579-01A	LUSC	7.76
TCGA-98-A53D-01A	LUSC	5.91
TCGA-56-8629-01A	LUSC	6.62
TCGA-37-A5EM-01A	LUSC	5.74
TCGA-56-8625-01A	LUSC	6.96
TCGA-66-2777-01A	LUSC	7.07
TCGA-56-8628-01A	LUSC	6.52
TCGA-66-2744-01A	LUSC	6.27
TCGA-77-A5G6-01A	LUSC	6.68
TCGA-66-2767-01A	LUSC	6.5
TCGA-39-5016-01A	LUSC	8.59
TCGA-58-8386-01A	LUSC	6.5
TCGA-39-5019-01A	LUSC	6.89
TCGA-68-A59I-01A	LUSC	6.5
TCGA-58-A46L-01A	LUSC	6.32
TCGA-85-7844-01A	LUSC	7.55
TCGA-37-4135-01A	LUSC	6.47
TCGA-LA-A7SW-01A	LUSC	5.68
TCGA-43-8115-01A	LUSC	6.55
TCGA-96-A4JL-01A	LUSC	7.17
TCGA-85-7699-01A	LUSC	5.82
TCGA-21-1082-01A	LUSC	5.24
TCGA-60-2723-01A	LUSC	7.8
TCGA-O2-A52N-01A	LUSC	5.99
TCGA-21-1083-01A	LUSC	7.45
TCGA-18-3419-01A	LUSC	6.44
TCGA-77-A5GF-01A	LUSC	6.95
TCGA-34-8454-01A	LUSC	6.69
TCGA-21-1078-01A	LUSC	8.43
TCGA-22-4604-01A	LUSC	5.85
TCGA-63-A5MG-01A	LUSC	5.79

TCGA-90-6837-01A	LUSC	5.65
TCGA-43-2576-01A	LUSC	7.14
TCGA-56-8082-01A	LUSC	7.28
TCGA-63-7021-01A	LUSC	5.52
TCGA-66-2734-01A	LUSC	6.91
TCGA-22-1011-01A	LUSC	5.92
TCGA-58-8393-01A	LUSC	7.07
TCGA-77-7140-01A	LUSC	8.31
TCGA-85-8352-01A	LUSC	6.81
TCGA-34-5927-01A	LUSC	7.48
TCGA-NC-A5HR-01A	LUSC	7.11
TCGA-85-8354-01A	LUSC	7.17
TCGA-43-3920-01A	LUSC	6.55
TCGA-37-3783-01A	LUSC	6.37
TCGA-56-7730-01A	LUSC	7.45
TCGA-NK-A5D1-01A	LUSC	6.64
TCGA-43-8116-01A	LUSC	6.34
TCGA-63-A5MR-01A	LUSC	5.24
TCGA-37-4129-01A	LUSC	6.11
TCGA-18-3410-01A	LUSC	6.84
TCGA-J1-A4AH-01A	LUSC	6.77
TCGA-33-A5GW-01A	LUSC	8.41
TCGA-77-8156-01A	LUSC	6.2
TCGA-77-8145-01A	LUSC	7.22
TCGA-22-1000-01A	LUSC	6.36
TCGA-77-8144-01A	LUSC	6.27
TCGA-90-A59Q-01A	LUSC	5.46
TCGA-85-8048-01A	LUSC	6.63
TCGA-52-7810-01A	LUSC	6
TCGA-66-2782-01A	LUSC	6.37
TCGA-22-5474-01A	LUSC	7.92
TCGA-66-2794-01A	LUSC	7.79
TCGA-77-8130-01A	LUSC	5.47
TCGA-85-8277-01A	LUSC	7.11
TCGA-NC-A5HT-01A	LUSC	6.45
TCGA-18-3406-01A	LUSC	5.57
TCGA-56-8504-01A	LUSC	7.49
TCGA-22-4607-01A	LUSC	6.72
TCGA-37-4133-01A	LUSC	7.76
TCGA-77-7463-01A	LUSC	7.5
TCGA-56-8308-01A	LUSC	7.21
TCGA-18-3407-01A	LUSC	7.52
TCGA-37-3792-01A	LUSC	7.23
TCGA-51-6867-01A	LUSC	6.9
TCGA-63-A5MS-01A	LUSC	6.12
TCGA-77-7141-01A	LUSC	7.6
TCGA-77-8146-01A	LUSC	7.06
TCGA-60-2719-01A	LUSC	8.32
TCGA-90-7964-01A	LUSC	7.8
TCGA-37-5819-01A	LUSC	6.9
TCGA-18-3416-01A	LUSC	7.93
TCGA-33-4532-01A	LUSC	6.68
TCGA-77-A5G8-01B	LUSC	6.01
TCGA-85-8584-01A	LUSC	6.94
TCGA-22-1012-01A	LUSC	6.43
TCGA-21-1072-01A	LUSC	8.35
TCGA-63-A5MJ-01A	LUSC	6.22
TCGA-46-3767-01A	LUSC	6.41

TCGA-58-8390-01A	LUSC	6.69
TCGA-22-A5C4-01A	LUSC	8.75
TCGA-85-A4PA-01A	LUSC	4.59
TCGA-22-0940-01A	LUSC	6.66
TCGA-77-6842-01A	LUSC	7.67
TCGA-52-7812-01A	LUSC	7.15
TCGA-66-2754-01A	LUSC	7.54
TCGA-52-7809-01A	LUSC	6.43
TCGA-85-8580-01A	LUSC	7.12
TCGA-58-8387-01A	LUSC	5.68
TCGA-77-8131-01A	LUSC	6.09
TCGA-63-A5MU-01A	LUSC	7.26
TCGA-56-5898-01A	LUSC	6.9
TCGA-66-2727-01A	LUSC	7.37
TCGA-85-6798-01A	LUSC	6.79
TCGA-46-6025-01A	LUSC	7.39
TCGA-94-8490-01A	LUSC	7.34
TCGA-60-2714-01A	LUSC	5.55
TCGA-34-5241-01A	LUSC	6.66
TCGA-NK-A5CX-01A	LUSC	6.14
TCGA-56-7823-01B	LUSC	7.63
TCGA-94-A5I6-01A	LUSC	7.73
TCGA-96-A4JK-01A	LUSC	7.24
TCGA-66-2742-01A	LUSC	6.82
TCGA-43-6773-01A	LUSC	5.18
TCGA-60-2724-01A	LUSC	5.45
TCGA-46-3768-01A	LUSC	8.67
TCGA-22-1002-01A	LUSC	6.13
TCGA-85-7698-01A	LUSC	6.53
TCGA-90-7767-01A	LUSC	7.37
TCGA-56-8503-01A	LUSC	6.15
TCGA-43-7656-01A	LUSC	5.86
TCGA-37-A5EL-01A	LUSC	7.53
TCGA-21-1079-01A	LUSC	6.28
TCGA-85-8350-01A	LUSC	5.86
TCGA-94-7943-01A	LUSC	8.12
TCGA-NC-A5HH-01A	LUSC	6.54
TCGA-43-A474-01A	LUSC	7.3
TCGA-39-5034-01A	LUSC	7.48
TCGA-77-8007-01A	LUSC	6.28
TCGA-34-7107-01A	LUSC	7.44
TCGA-98-A539-01A	LUSC	6.08
TCGA-85-8351-01A	LUSC	6.15
TCGA-56-A4ZJ-01A	LUSC	6.55
TCGA-66-2753-01A	LUSC	6.67
TCGA-60-2696-01A	LUSC	7.2
TCGA-56-8305-01A	LUSC	8.36
TCGA-22-5482-01A	LUSC	6.74
TCGA-66-2781-01A	LUSC	6.43
TCGA-39-5027-01A	LUSC	7.54
TCGA-NC-A5HM-01A	LUSC	6.67
TCGA-63-6202-01A	LUSC	6
TCGA-60-2713-01A	LUSC	6.22
TCGA-O2-A52S-01A	LUSC	6.61
TCGA-77-7138-01A	LUSC	4.85
TCGA-43-7657-01A	LUSC	5.76
TCGA-22-5489-01A	LUSC	6.2
TCGA-66-2759-01A	LUSC	7.33

TCGA-92-8064-01A	LUSC	6.2
TCGA-98-A53H-01A	LUSC	5.91
TCGA-34-5929-01A	LUSC	6.64
TCGA-33-AASD-01A	LUSC	7.27
TCGA-92-8065-01A	LUSC	5.86
TCGA-56-A5DS-01A	LUSC	6.49
TCGA-98-A53I-01A	LUSC	7.78
TCGA-21-5786-01A	LUSC	5.55
TCGA-MF-A522-01A	LUSC	8.43
TCGA-43-7658-01A	LUSC	6.16
TCGA-60-2695-01A	LUSC	5.6
TCGA-39-5022-01A	LUSC	6.8
TCGA-63-7022-01A	LUSC	5.2
TCGA-56-7582-01A	LUSC	7.26
TCGA-77-8128-01A	LUSC	7.07
TCGA-33-AASL-01A	LUSC	7.37
TCGA-56-A4BW-01A	LUSC	6.5
TCGA-43-6771-01A	LUSC	5.86
TCGA-33-4533-01A	LUSC	5.75
TCGA-22-5471-01A	LUSC	6.53
TCGA-85-A512-01A	LUSC	6.83
TCGA-21-1080-01A	LUSC	6.33
TCGA-85-A5B5-01A	LUSC	6.21
TCGA-46-3769-01A	LUSC	7.55
TCGA-56-A4BY-01A	LUSC	7.21
TCGA-96-8169-01A	LUSC	5.89
TCGA-63-5128-01A	LUSC	5.75
TCGA-22-4596-01A	LUSC	6.52
TCGA-34-5232-01A	LUSC	6.82
TCGA-21-5787-01A	LUSC	6.07
TCGA-46-6026-01A	LUSC	5.79
TCGA-18-3417-01A	LUSC	6.62
TCGA-46-3765-01A	LUSC	7.77
TCGA-22-5473-01A	LUSC	7.82
TCGA-34-5240-01A	LUSC	8.04
TCGA-60-2725-01A	LUSC	7.15
TCGA-66-2790-01A	LUSC	6.99
TCGA-56-A4BX-01A	LUSC	7.43
TCGA-60-2698-01A	LUSC	7.43
TCGA-21-1075-01A	LUSC	7.93
TCGA-58-A46N-01A	LUSC	8.09
TCGA-L3-A524-01A	LUSC	7.01
TCGA-98-8020-01A	LUSC	5.23
TCGA-37-4130-01A	LUSC	7.61
TCGA-66-2757-01A	LUSC	6.98
TCGA-94-8491-01A	LUSC	7
TCGA-66-2786-01A	LUSC	6.91
TCGA-43-2581-01A	LUSC	5.56
TCGA-18-3409-01A	LUSC	6.69
TCGA-77-A5GH-01A	LUSC	6.37
TCGA-90-A4EE-01A	LUSC	6.27
TCGA-22-5481-01A	LUSC	4.48
TCGA-77-8143-01A	LUSC	7.79
TCGA-85-8582-01A	LUSC	6.33
TCGA-43-A475-01A	LUSC	6.97
TCGA-85-7696-01A	LUSC	7.11
TCGA-18-3412-01A	LUSC	7.96
TCGA-43-3394-01A	LUSC	7

TCGA-60-2721-01A	LUSC	6.08
TCGA-22-5477-01A	LUSC	7.95
TCGA-85-8071-01A	LUSC	7.43
TCGA-85-A510-01A	LUSC	6.45
TCGA-60-2703-01A	LUSC	5.95
TCGA-85-7697-01A	LUSC	6.03
TCGA-56-6546-01A	LUSC	6.31
TCGA-85-8355-01A	LUSC	7.24
TCGA-18-3408-01A	LUSC	8.19
TCGA-L3-A4E7-01A	LUSC	6.31
TCGA-22-4599-01A	LUSC	6.91
TCGA-85-8072-01A	LUSC	6.49
TCGA-60-2712-01A	LUSC	5.19
TCGA-56-8622-01A	LUSC	5.59
TCGA-NC-A5HD-01A	LUSC	7.01
TCGA-56-8309-01A	LUSC	6.5
TCGA-21-1077-01A	LUSC	7.21
TCGA-56-8626-01A	LUSC	5.87
TCGA-98-7454-01A	LUSC	6.84
TCGA-66-2791-01A	LUSC	7.23
TCGA-22-5483-01A	LUSC	7.4
TCGA-98-8022-01A	LUSC	6.02
TCGA-56-5897-01A	LUSC	7.83
TCGA-39-5035-01A	LUSC	5.39
TCGA-60-2706-01A	LUSC	8.52
TCGA-66-2766-01A	LUSC	7.16
TCGA-77-6843-01A	LUSC	8.27
TCGA-NC-A5HI-01A	LUSC	7.3
TCGA-56-8307-01A	LUSC	6.36
TCGA-77-A5G1-01A	LUSC	6.64
TCGA-33-6738-01A	LUSC	8.37
TCGA-98-A538-01A	LUSC	7.5
TCGA-58-A46K-01A	LUSC	7.61
TCGA-66-2768-01A	LUSC	6.19
TCGA-85-A511-01A	LUSC	7.24
TCGA-NC-A5HE-01A	LUSC	6.64
TCGA-56-7580-01A	LUSC	6.31
TCGA-66-2755-01A	LUSC	6.98
TCGA-39-5028-01A	LUSC	5.65
TCGA-37-A5EN-01A	LUSC	7.11
TCGA-34-2600-01A	LUSC	5.98
TCGA-58-A46J-01A	LUSC	6.3
TCGA-XC-AA0X-01A	LUSC	6.58
TCGA-66-2795-01A	LUSC	7.41
TCGA-66-2787-01A	LUSC	7
TCGA-66-2758-01A	LUSC	7.33
TCGA-70-6722-01A	LUSC	6.35
TCGA-58-A46M-01A	LUSC	8.34
TCGA-60-2715-01A	LUSC	6.07
TCGA-39-5040-01A	LUSC	6.74
TCGA-63-A5M9-01A	LUSC	7.75
TCGA-77-6844-01A	LUSC	6.74
TCGA-33-AASJ-01A	LUSC	6.08
TCGA-37-3789-01A	LUSC	6.27
TCGA-56-7822-01A	LUSC	6.12
TCGA-66-2770-01A	LUSC	7.38
TCGA-68-7757-01B	LUSC	6.98
TCGA-18-3415-01A	LUSC	7.47

TCGA-66-2778-01A	LUSC	6.96
TCGA-85-7710-01A	LUSC	6.08
TCGA-58-8391-01A	LUSC	8.24
TCGA-43-8118-01A	LUSC	7.28
TCGA-85-8276-01A	LUSC	6.74
TCGA-60-2710-01A	LUSC	7.62
TCGA-18-3414-01A	LUSC	6.4
TCGA-NC-A5HG-01A	LUSC	5.66
TCGA-33-6737-01A	LUSC	7.22
TCGA-43-A56V-01A	LUSC	7.05
TCGA-77-8136-01A	LUSC	5.55
TCGA-22-5472-01A	LUSC	7.43
TCGA-68-7756-01A	LUSC	7.44
TCGA-77-A5GA-01A	LUSC	7.24
TCGA-66-2783-01A	LUSC	7.9
TCGA-63-A5MN-01A	LUSC	5.46
TCGA-98-A53B-01A	LUSC	6.02
TCGA-21-5782-01A	LUSC	5.57
TCGA-22-0944-01A	LUSC	6.68
TCGA-43-5668-01A	LUSC	9.67
TCGA-85-8664-01A	LUSC	5.59
TCGA-94-7557-01A	LUSC	8.3
TCGA-77-7139-01A	LUSC	6.2
TCGA-39-5021-01A	LUSC	7.81
TCGA-77-6845-01A	LUSC	7.05
TCGA-66-2788-01A	LUSC	6.77
TCGA-63-A5MP-01A	LUSC	5.99
TCGA-85-8049-01A	LUSC	6.69
TCGA-33-4566-01A	LUSC	7.73
TCGA-39-5031-01A	LUSC	6.45
TCGA-56-A4ZK-01A	LUSC	6.73
TCGA-21-A5DI-01A	LUSC	5.99
TCGA-33-AAS8-01A	LUSC	7.37
TCGA-63-A5MM-01A	LUSC	5.68
TCGA-77-8009-01A	LUSC	7.3
TCGA-43-6143-01A	LUSC	7.15
TCGA-77-7335-01A	LUSC	6.95
TCGA-22-4595-01A	LUSC	6.62
TCGA-56-6545-01A	LUSC	5.99
TCGA-77-A5G3-01A	LUSC	6.99
TCGA-77-8133-01A	LUSC	6.72
TCGA-56-7731-01A	LUSC	6.33
TCGA-66-2785-01A	LUSC	6.8
TCGA-33-4538-01A	LUSC	6.99
TCGA-77-7337-01A	LUSC	6.42
TCGA-98-A53A-01A	LUSC	7.11
TCGA-68-8251-01A	LUSC	6.24
TCGA-63-A5ML-01A	LUSC	7.22
TCGA-85-A4QQ-01A	LUSC	6.05
TCGA-21-1071-01A	LUSC	8.17
TCGA-85-7950-01A	LUSC	7.11
TCGA-NK-A5CT-01A	LUSC	6.62
TCGA-33-4583-01A	LUSC	8.65
TCGA-NC-A5HN-01A	LUSC	5.94
TCGA-34-5231-01A	LUSC	6.13
TCGA-22-5479-01A	LUSC	6.18
TCGA-46-3766-01A	LUSC	6.37
TCGA-18-3411-01A	LUSC	8.36

TCGA-39-5039-01A	LUSC	6.96
TCGA-85-A4QR-01A	LUSC	7.2
TCGA-33-4582-01A	LUSC	6.33
TCGA-77-A5GB-01B	LUSC	4.66
TCGA-NQ-A638-01A	MESO	5.93
TCGA-LK-A4O4-01A	MESO	5.01
TCGA-TS-A7P6-01A	MESO	5.61
TCGA-TS-A8AS-01A	MESO	5.64
TCGA-UD-AAC4-01A	MESO	5.84
TCGA-MQ-A4LI-01A	MESO	6.08
TCGA-MQ-A6BN-01A	MESO	4.64
TCGA-MQ-A6BS-01A	MESO	5.96
TCGA-TS-A7P1-01A	MESO	5.01
TCGA-ZN-A9VW-01A	MESO	7.6
TCGA-SC-A6LQ-01A	MESO	6.09
TCGA-YS-AA4M-01A	MESO	6.44
TCGA-3U-A98F-01A	MESO	6.03
TCGA-3H-AB3O-01A	MESO	6.31
TCGA-3H-AB3T-01A	MESO	5.65
TCGA-3U-A98H-01A	MESO	5.71
TCGA-ZN-A9VS-01A	MESO	7.81
TCGA-UT-A88E-01B	MESO	6.19
TCGA-TS-A7P3-01A	MESO	5.74
TCGA-MQ-A4LM-01A	MESO	6.05
TCGA-UD-AAC7-01A	MESO	6.67
TCGA-SC-AA5Z-01A	MESO	6.09
TCGA-NQ-A57I-01A	MESO	5.88
TCGA-MQ-A6BL-01A	MESO	5.34
TCGA-UT-A88D-01B	MESO	6.01
TCGA-TS-A8AY-01A	MESO	6.54
TCGA-TS-A7OY-01A	MESO	6.18
TCGA-SC-A6LN-01A	MESO	5.39
TCGA-YS-A95B-01A	MESO	6.17
TCGA-3H-AB3S-01A	MESO	5.43
TCGA-LK-A4O0-01A	MESO	5.73
TCGA-LK-A4NW-01A	MESO	6.08
TCGA-TS-A7P7-01A	MESO	5.87
TCGA-SH-A9CT-01A	MESO	6.29
TCGA-SH-A9CU-01A	MESO	6.44
TCGA-ZN-A9VO-01A	MESO	6.28
TCGA-UD-AABZ-01A	MESO	5.52
TCGA-LK-A4O5-01A	MESO	5.55
TCGA-3H-AB3L-01A	MESO	6.08
TCGA-3U-A98G-01A	MESO	5.63
TCGA-MQ-A4LJ-01A	MESO	5.93
TCGA-MQ-A4LP-01A	MESO	7.4
TCGA-SH-A7BH-01A	MESO	4.04
TCGA-LK-A4O2-01A	MESO	6.58
TCGA-SH-A7BD-01A	MESO	5.17
TCGA-TS-A7OU-01B	MESO	7.44
TCGA-TS-A8AI-01A	MESO	6.05
TCGA-SC-A6LR-01A	MESO	6.15
TCGA-3U-A98D-01A	MESO	6.2
TCGA-3U-A98I-01A	MESO	5.57
TCGA-TS-A7PB-01A	MESO	5.63
TCGA-MQ-A6BQ-01A	MESO	5.95
TCGA-MQ-A4LC-01A	MESO	6.29
TCGA-ZN-A9VU-01A	MESO	7.42

TCGA-UT-A88G-01B	MESO	6.26
TCGA-MQ-A4KX-01A	MESO	6.02
TCGA-UT-A88C-01B	MESO	6.15
TCGA-MQ-A4LV-01A	MESO	5.74
TCGA-TS-A8AF-01A	MESO	5.75
TCGA-3H-AB3U-01A	MESO	7.21
TCGA-YS-A95C-01A	MESO	6.37
TCGA-UD-AAC5-01A	MESO	6.44
TCGA-LK-A4NY-01A	MESO	5.12
TCGA-LK-A4NZ-01A	MESO	5.5
TCGA-UD-AAC1-01A	MESO	5.67
TCGA-TS-A7P8-01A	MESO	5.62
TCGA-UD-AAC6-01A	MESO	6.87
TCGA-UD-AABY-01A	MESO	5.88
TCGA-SH-A7BC-01A	MESO	6.34
TCGA-3H-AB3X-01A	MESO	5.84
TCGA-LK-A4O6-01A	MESO	4.42
TCGA-SC-A6LP-01A	MESO	5.58
TCGA-3U-A98E-01A	MESO	6.07
TCGA-TS-A7P0-01A	MESO	6.12
TCGA-3H-AB3K-01A	MESO	5.47
TCGA-TS-A8AV-01A	MESO	6.39
TCGA-XT-AASU-01A	MESO	6.01
TCGA-3U-A98J-01A	MESO	5.6
TCGA-UT-A97Y-01A	MESO	5.3
TCGA-ZN-A9VV-01A	MESO	4.38
TCGA-LK-A4O7-01A	MESO	6.12
TCGA-SC-A6LM-01A	MESO	6.73
TCGA-TS-A7OZ-01A	MESO	5.91
TCGA-3H-AB3M-01A	MESO	6.52
TCGA-ZN-A9VP-01A	MESO	6.24
TCGA-ZN-A9VQ-01A	MESO	6.21
TCGA-MQ-A6BR-01A	MESO	4.6
TCGA-10-0933-01A	OV	6.33
TCGA-23-1024-01A	OV	4.92
TCGA-13-0886-01A	OV	6.4
TCGA-29-1769-01A	OV	6.32
TCGA-13-1505-01A	OV	5.99
TCGA-61-1736-01B	OV	5.63
TCGA-61-1737-01A	OV	5.96
TCGA-25-1871-01A	OV	6.14
TCGA-29-1705-02A	OV	4.31
TCGA-29-1705-01A	OV	4.61
TCGA-24-1104-01A	OV	5.99
TCGA-13-0720-01A	OV	4.5
TCGA-61-1728-01A	OV	6.2
TCGA-WR-A838-01A	OV	6.11
TCGA-23-1122-01A	OV	5.76
TCGA-13-1487-01A	OV	6.04
TCGA-23-1027-01A	OV	6.71
TCGA-29-1774-01A	OV	6.99
TCGA-30-1866-01A	OV	5.99
TCGA-23-1030-01A	OV	4.85
TCGA-25-1627-01A	OV	6.09
TCGA-13-1410-01A	OV	6.65
TCGA-24-1564-01A	OV	6.82
TCGA-10-0926-01A	OV	6.32
TCGA-13-1510-01A	OV	6.58

TCGA-31-1956-01A	OV	5.68
TCGA-24-2020-01A	OV	6.47
TCGA-23-1028-01A	OV	6.16
TCGA-13-1407-01A	OV	6
TCGA-04-1655-01A	OV	6.06
TCGA-09-1661-01B	OV	6.09
TCGA-24-1416-01A	OV	6.18
TCGA-61-1911-01A	OV	5.74
TCGA-61-1995-01A	OV	6.25
TCGA-10-0927-01A	OV	6.08
TCGA-20-1684-01A	OV	6.85
TCGA-31-1951-01A	OV	5.98
TCGA-13-1411-01A	OV	6.48
TCGA-24-1425-01A	OV	6.56
TCGA-09-0367-01A	OV	5.41
TCGA-30-1718-01A	OV	6.12
TCGA-20-1687-01A	OV	4.74
TCGA-61-2094-01A	OV	5.82
TCGA-61-1743-01A	OV	6.34
TCGA-30-1892-01A	OV	4.78
TCGA-09-1665-01B	OV	6.69
TCGA-13-0801-01A	OV	5.82
TCGA-04-1343-01A	OV	5.48
TCGA-61-2092-01A	OV	7.28
TCGA-5X-AA5U-01A	OV	5.26
TCGA-61-1740-01A	OV	5.31
TCGA-09-1674-01A	OV	5.27
TCGA-61-2088-01A	OV	6.03
TCGA-24-1567-01A	OV	6.02
TCGA-25-1633-01A	OV	6.04
TCGA-23-2084-01A	OV	5.8
TCGA-59-2348-01A	OV	6.8
TCGA-OY-A56P-01A	OV	5.81
TCGA-24-1544-01A	OV	6.03
TCGA-04-1338-01A	OV	5.24
TCGA-36-1576-01A	OV	6.61
TCGA-13-0885-01A	OV	5.94
TCGA-13-0913-01A	OV	5.38
TCGA-13-0724-01A	OV	5.8
TCGA-09-1669-01A	OV	7.19
TCGA-20-1683-01A	OV	5.66
TCGA-23-1107-01A	OV	5.8
TCGA-13-0795-01A	OV	4.96
TCGA-25-2399-01A	OV	6.83
TCGA-29-1703-01A	OV	5.73
TCGA-24-1560-01A	OV	6.87
TCGA-29-2414-02A	OV	6.57
TCGA-24-2289-01A	OV	5.67
TCGA-61-1725-01A	OV	5.58
TCGA-25-1632-01A	OV	6.08
TCGA-13-1488-01A	OV	6.19
TCGA-24-2036-01A	OV	6.76
TCGA-13-1497-01A	OV	6.24
TCGA-59-2354-01A	OV	6.03
TCGA-24-1474-01A	OV	5.65
TCGA-24-1426-01A	OV	5.08
TCGA-13-1496-01A	OV	5.53
TCGA-13-0890-01A	OV	5.8

TCGA-04-1514-01A	OV	5.96
TCGA-36-1577-01A	OV	6.7
TCGA-59-2350-01A	OV	7.68
TCGA-29-A5NZ-01A	OV	6.05
TCGA-61-2098-01A	OV	6.1
TCGA-23-2077-01A	OV	5.88
TCGA-23-1120-01A	OV	6.73
TCGA-24-1565-01A	OV	5.16
TCGA-24-1423-01A	OV	5.88
TCGA-61-1900-01A	OV	6.15
TCGA-29-1781-01A	OV	5.64
TCGA-20-1685-01A	OV	8.73
TCGA-09-2054-01A	OV	5.66
TCGA-25-1313-01A	OV	6.04
TCGA-13-0760-01A	OV	5.47
TCGA-10-0931-01A	OV	5.4
TCGA-13-0768-01A	OV	5.19
TCGA-3P-A9WA-01A	OV	7.43
TCGA-24-1545-01A	OV	6.55
TCGA-13-0906-01A	OV	6.27
TCGA-13-1482-01A	OV	5.75
TCGA-36-1581-01A	OV	7.07
TCGA-29-1711-01A	OV	6.48
TCGA-13-1499-01A	OV	5.33
TCGA-23-1023-01A	OV	6.12
TCGA-29-2428-01A	OV	5.49
TCGA-24-1844-01A	OV	6.66
TCGA-25-1635-01A	OV	6.28
TCGA-23-1114-01B	OV	6.65
TCGA-OY-A56Q-01A	OV	7
TCGA-31-1946-01A	OV	5.83
TCGA-23-2078-01A	OV	5.51
TCGA-61-2109-01A	OV	5.93
TCGA-57-1584-01A	OV	6.8
TCGA-25-2409-01A	OV	5.81
TCGA-04-1347-01A	OV	4.81
TCGA-36-1568-01A	OV	5.93
TCGA-09-2056-01B	OV	6.66
TCGA-24-2261-01A	OV	6.05
TCGA-24-1471-01A	OV	4.69
TCGA-13-0893-01B	OV	6.53
TCGA-25-1634-01A	OV	6.87
TCGA-25-1870-01A	OV	5.72
TCGA-13-0797-01A	OV	5.77
TCGA-31-1959-01A	OV	7.99
TCGA-57-1993-01A	OV	6.7
TCGA-13-1409-01A	OV	5.4
TCGA-09-1668-01B	OV	5.94
TCGA-13-1477-01A	OV	4.39
TCGA-24-1418-01A	OV	5.9
TCGA-13-0887-01A	OV	6.38
TCGA-13-2060-01A	OV	6.13
TCGA-24-2288-01A	OV	5.17
TCGA-29-1784-01A	OV	5.27
TCGA-57-1585-01A	OV	5.65
TCGA-13-0916-01A	OV	5.65
TCGA-09-1670-01A	OV	6.72
TCGA-57-1994-01A	OV	6.19

TCGA-23-1123-01A	OV	6.28
TCGA-24-0968-01A	OV	6.15
TCGA-25-1321-01A	OV	7.61
TCGA-24-1930-01A	OV	5.36
TCGA-29-1693-01A	OV	5.44
TCGA-30-1853-01A	OV	4.78
TCGA-61-2016-01A	OV	5.47
TCGA-23-1118-01A	OV	6.42
TCGA-24-1604-01A	OV	7.05
TCGA-23-1109-01A	OV	4.36
TCGA-25-1631-01A	OV	6.69
TCGA-13-0897-01A	OV	5.16
TCGA-24-0975-01A	OV	5.15
TCGA-61-1738-01A	OV	7.43
TCGA-61-2008-02A	OV	5.74
TCGA-61-2008-01A	OV	5.03
TCGA-24-1549-01A	OV	6.63
TCGA-25-2401-01A	OV	6.37
TCGA-29-1761-01A	OV	7.39
TCGA-09-2045-01A	OV	6.42
TCGA-29-2425-01A	OV	5.77
TCGA-24-1551-01A	OV	6.56
TCGA-13-1498-01A	OV	5.47
TCGA-25-1316-01A	OV	5.42
TCGA-25-1320-01A	OV	5.98
TCGA-30-1891-01A	OV	4.86
TCGA-24-1434-01A	OV	6.46
TCGA-13-1507-01A	OV	5.9
TCGA-29-1707-02A	OV	5.79
TCGA-25-1623-01A	OV	6.12
TCGA-04-1364-01A	OV	6.3
TCGA-25-2042-01A	OV	6.71
TCGA-13-1512-01A	OV	5.84
TCGA-24-1923-01A	OV	6.38
TCGA-13-1404-01A	OV	5.84
TCGA-61-2102-01A	OV	7.17
TCGA-VG-A8LO-01A	OV	7.34
TCGA-04-1356-01A	OV	6.49
TCGA-29-1701-01A	OV	5.21
TCGA-13-0900-01B	OV	6.81
TCGA-29-1768-01A	OV	5.81
TCGA-57-1583-01A	OV	6.86
TCGA-13-0765-01A	OV	4.69
TCGA-24-2298-01A	OV	6.86
TCGA-61-2111-01A	OV	5.96
TCGA-29-1696-01A	OV	6.69
TCGA-24-2267-01A	OV	6.01
TCGA-13-0727-01A	OV	4.64
TCGA-20-1682-01A	OV	5.39
TCGA-13-0911-01A	OV	5.77
TCGA-20-0991-01A	OV	5.99
TCGA-57-1586-01A	OV	6.24
TCGA-24-1417-01A	OV	6.46
TCGA-24-1464-01A	OV	5.34
TCGA-25-2397-01A	OV	6.1
TCGA-13-1485-01A	OV	5.09
TCGA-30-1857-01A	OV	5.41
TCGA-25-2400-01A	OV	6.04

TCGA-29-1762-01A	OV	6.41
TCGA-57-1582-01A	OV	6.59
TCGA-23-1113-01A	OV	5.63
TCGA-24-1552-01A	OV	5.58
TCGA-25-2396-01A	OV	6.55
TCGA-25-1626-01A	OV	6.37
TCGA-29-1690-01A	OV	5.57
TCGA-25-1315-01A	OV	6.35
TCGA-13-0884-01B	OV	5.52
TCGA-09-1659-01B	OV	4.8
TCGA-25-2392-01A	OV	5.35
TCGA-25-1312-01A	OV	5.74
TCGA-13-0888-01A	OV	5.21
TCGA-29-1776-01A	OV	7.02
TCGA-13-1495-01A	OV	6.18
TCGA-24-1846-01A	OV	5.11
TCGA-09-2044-01B	OV	6.23
TCGA-29-1770-02A	OV	5.02
TCGA-13-1483-01A	OV	5.77
TCGA-31-1953-01A	OV	6.67
TCGA-10-0934-01A	OV	6.4
TCGA-23-1111-01A	OV	5.17
TCGA-59-2355-01A	OV	6.52
TCGA-04-1331-01A	OV	7.24
TCGA-24-1556-01A	OV	5.91
TCGA-36-1569-01A	OV	5.31
TCGA-24-1557-01A	OV	7.84
TCGA-61-2002-01A	OV	6.06
TCGA-61-1914-01A	OV	5.91
TCGA-13-0804-01A	OV	5
TCGA-13-1408-01A	OV	5.46
TCGA-24-0979-01A	OV	5.79
TCGA-24-2254-01A	OV	7.38
TCGA-25-1319-01A	OV	6.87
TCGA-04-1341-01A	OV	5.05
TCGA-25-1328-01A	OV	5.03
TCGA-24-1105-01A	OV	6.84
TCGA-61-1907-01A	OV	5.77
TCGA-61-2110-01A	OV	6
TCGA-25-1625-01A	OV	5.33
TCGA-23-1023-01R	OV	5.89
TCGA-24-1843-01A	OV	6.6
TCGA-24-1849-01A	OV	6.04
TCGA-29-1763-01A	OV	5.74
TCGA-04-1536-01A	OV	4.03
TCGA-24-2026-01A	OV	5.5
TCGA-61-1918-01A	OV	6.59
TCGA-24-1427-01A	OV	5.49
TCGA-24-2290-01A	OV	5.21
TCGA-24-1430-01A	OV	6.76
TCGA-24-1431-01A	OV	5.35
TCGA-13-0726-01A	OV	5.28
TCGA-24-2297-01A	OV	6.4
TCGA-04-1517-01A	OV	7.65
TCGA-59-2351-01A	OV	6.57
TCGA-25-1314-01A	OV	4.21
TCGA-13-1506-01A	OV	6.84
TCGA-04-1350-01A	OV	6.43

TCGA-24-1463-01A	OV	5.57
TCGA-24-2262-01A	OV	6.56
TCGA-13-0714-01A	OV	5.39
TCGA-09-2048-01A	OV	6.52
TCGA-29-1702-01A	OV	5.04
TCGA-25-1318-01A	OV	6.01
TCGA-24-2038-01A	OV	4.73
TCGA-09-0366-01A	OV	7.08
TCGA-61-1721-01A	OV	5.71
TCGA-61-2012-01A	OV	7.29
TCGA-13-0905-01B	OV	5.56
TCGA-24-1847-01A	OV	6.11
TCGA-24-2027-01A	OV	5.95
TCGA-24-1924-01A	OV	6.55
TCGA-61-2000-01A	OV	5.12
TCGA-25-2404-01A	OV	6.46
TCGA-10-0928-01A	OV	6.3
TCGA-24-1467-01A	OV	5.83
TCGA-13-1403-01A	OV	5.38
TCGA-04-1362-01A	OV	7.78
TCGA-13-0901-01B	OV	5.8
TCGA-24-0982-01A	OV	6.41
TCGA-29-1785-01A	OV	5.63
TCGA-24-1550-01A	OV	6
TCGA-29-1698-01A	OV	6.37
TCGA-09-0369-01A	OV	5.42
TCGA-29-1694-01A	OV	6.28
TCGA-25-1630-01A	OV	7.12
TCGA-13-0730-01A	OV	7.03
TCGA-09-2051-01A	OV	5.9
TCGA-20-0987-01A	OV	5.95
TCGA-13-0913-02A	OV	4.27
TCGA-04-1365-01A	OV	5.16
TCGA-09-1667-01C	OV	6.24
TCGA-61-1724-01A	OV	6.79
TCGA-29-1695-01A	OV	6.99
TCGA-13-0800-01A	OV	6.72
TCGA-23-1119-01A	OV	5.77
TCGA-29-1766-01A	OV	6.37
TCGA-25-1323-01A	OV	5.99
TCGA-13-1405-01A	OV	6.66
TCGA-25-1628-01A	OV	6.43
TCGA-59-2363-01A	OV	6.01
TCGA-04-1519-01A	OV	6.19
TCGA-09-2053-01C	OV	5.02
TCGA-13-A5FT-01A	OV	5.43
TCGA-61-1919-01A	OV	6.4
TCGA-04-1357-01A	OV	5.47
TCGA-24-0966-01A	OV	4.81
TCGA-20-1686-01A	OV	6.44
TCGA-23-1032-01A	OV	5.66
TCGA-04-1337-01A	OV	4.67
TCGA-04-1348-01A	OV	6.53
TCGA-13-0924-01A	OV	6.12
TCGA-25-2398-01A	OV	5.29
TCGA-23-1110-01A	OV	5.88
TCGA-24-1428-01A	OV	5.01
TCGA-61-2009-01A	OV	5.73

TCGA-10-0937-01A	OV	5.01
TCGA-24-1928-01A	OV	6.69
TCGA-04-1332-01A	OV	6.18
TCGA-24-2293-01A	OV	6.36
TCGA-25-1322-01A	OV	6.77
TCGA-23-1021-01B	OV	5.29
TCGA-24-1845-01A	OV	4.77
TCGA-13-0799-01A	OV	6.02
TCGA-24-1558-01A	OV	7.32
TCGA-23-1026-01B	OV	5.78
TCGA-25-1324-01A	OV	6.56
TCGA-24-2271-01A	OV	5.76
TCGA-29-1691-01A	OV	7.12
TCGA-29-2414-01A	OV	6.48
TCGA-04-1361-01A	OV	6.28
TCGA-25-2393-01A	OV	5.55
TCGA-24-1470-01A	OV	5.82
TCGA-25-1329-01A	OV	5.54
TCGA-24-2019-01A	OV	6.02
TCGA-24-2280-01A	OV	5.22
TCGA-23-1809-01A	OV	6.16
TCGA-24-1850-01A	OV	7.9
TCGA-24-1424-01A	OV	6.74
TCGA-13-0920-01A	OV	6.06
TCGA-09-1662-01A	OV	6.13
TCGA-59-A5PD-01A	OV	6.65
TCGA-13-0766-01A	OV	6.28
TCGA-24-2033-01A	OV	5.47
TCGA-24-1546-01A	OV	6.26
TCGA-36-1574-01A	OV	6.76
TCGA-24-1103-01A	OV	6.29
TCGA-25-2391-01A	OV	6.09
TCGA-04-1648-01A	OV	6.63
TCGA-29-1699-01A	OV	5.73
TCGA-23-1022-01A	OV	5.29
TCGA-13-1509-01A	OV	4.87
TCGA-36-1578-01A	OV	6.1
TCGA-31-1944-01A	OV	6.06
TCGA-24-1616-01A	OV	6.64
TCGA-09-0364-01A	OV	5.52
TCGA-23-1116-01A	OV	4.19
TCGA-13-1511-01A	OV	6.82
TCGA-24-1436-01A	OV	6.07
TCGA-61-2113-01A	OV	6.82
TCGA-59-2352-01A	OV	6.56
TCGA-61-1910-01A	OV	5.52
TCGA-24-1413-01A	OV	5.74
TCGA-29-1697-01A	OV	6.88
TCGA-29-1688-01A	OV	5.61
TCGA-61-2003-01A	OV	5.76
TCGA-61-2104-01A	OV	6.23
TCGA-29-1710-01A	OV	5.49
TCGA-24-2023-01A	OV	6.63
TCGA-24-1562-01A	OV	6.17
TCGA-61-1998-01A	OV	7.38
TCGA-31-1950-01A	OV	6.54
TCGA-29-1783-01A	OV	5.08
TCGA-61-1733-01A	OV	5.67

TCGA-29-1778-01A	OV	5.27
TCGA-13-1501-01A	OV	5.93
TCGA-30-1860-01A	OV	5.54
TCGA-24-1563-01A	OV	6.28
TCGA-36-1580-01A	OV	5.39
TCGA-61-2097-01A	OV	7.41
TCGA-30-1862-01A	OV	5.73
TCGA-04-1651-01A	OV	5.56
TCGA-23-2081-01A	OV	6.46
TCGA-13-0923-01A	OV	6.4
TCGA-13-0725-01A	OV	7.37
TCGA-04-1530-01A	OV	5.3
TCGA-13-0883-01A	OV	5.55
TCGA-09-1666-01A	OV	5.9
TCGA-13-0899-01A	OV	6.75
TCGA-24-2281-01A	OV	5.74
TCGA-29-1777-01A	OV	5.22
TCGA-61-2101-01A	OV	5.91
TCGA-24-1555-01A	OV	7.83
TCGA-24-1548-01A	OV	6.9
TCGA-13-1492-01A	OV	6.44
TCGA-13-0762-01A	OV	5.3
TCGA-24-1419-01A	OV	7.11
TCGA-25-1317-01A	OV	6.28
TCGA-10-0938-01A	OV	6.12
TCGA-36-1575-01A	OV	4.88
TCGA-29-2427-01A	OV	6.28
TCGA-30-1855-01A	OV	6.04
TCGA-13-0919-01A	OV	5.64
TCGA-24-1435-01A	OV	5.5
TCGA-23-1029-01B	OV	6.95
TCGA-09-1673-01A	OV	5.48
TCGA-29-1770-01A	OV	5.07
TCGA-10-0936-01A	OV	6.6
TCGA-61-2095-01A	OV	5.59
TCGA-13-1489-01A	OV	6.45
TCGA-13-1489-02A	OV	5.06
TCGA-36-1571-01A	OV	6.41
TCGA-61-1741-01A	OV	6.08
TCGA-24-1603-01A	OV	6.79
TCGA-13-1481-01A	OV	6.16
TCGA-24-0970-01B	OV	5.72
TCGA-24-1469-01A	OV	5.76
TCGA-24-1422-01A	OV	6.42
TCGA-24-2035-01A	OV	6.03
TCGA-24-1553-01A	OV	5.92
TCGA-30-1861-01A	OV	5.37
TCGA-13-0908-01B	OV	5.05
TCGA-25-1877-01A	OV	5.23
TCGA-04-1542-01A	OV	6.81
TCGA-25-1326-01A	OV	6.5
TCGA-24-2024-01A	OV	5.72
TCGA-36-1570-01A	OV	6.94
TCGA-30-1714-01A	OV	5.17
TCGA-13-0891-01A	OV	5.77
TCGA-24-1842-01A	OV	5.8
TCGA-FB-AAPP-01A	PAAD	7.14
TCGA-HZ-7289-01A	PAAD	6.92

TCGA-2L-AAQL-01A	PAAD	5.95
TCGA-3A-A9IL-01A	PAAD	4.41
TCGA-FB-AAPS-01A	PAAD	5.66
TCGA-2J-AAB8-01A	PAAD	7.22
TCGA-2J-AABT-01A	PAAD	6.26
TCGA-OE-A75W-01A	PAAD	5.69
TCGA-IB-8127-01A	PAAD	7.18
TCGA-HZ-8002-01A	PAAD	6.9
TCGA-HV-A5A6-01A	PAAD	6.71
TCGA-US-A779-01A	PAAD	6.47
TCGA-3A-A9IR-01A	PAAD	7.8
TCGA-HZ-8637-01A	PAAD	7.06
TCGA-HZ-7923-01A	PAAD	6.13
TCGA-IB-AAUN-01A	PAAD	5.97
TCGA-LB-A7SX-01A	PAAD	7.02
TCGA-HV-A7OP-01A	PAAD	4.66
TCGA-IB-A5ST-01A	PAAD	6.45
TCGA-3A-A9IC-01A	PAAD	6.36
TCGA-2J-AAB6-01A	PAAD	5.91
TCGA-HZ-8003-01A	PAAD	5.38
TCGA-H6-8124-01A	PAAD	6.24
TCGA-HZ-A9TJ-01A	PAAD	6.55
TCGA-FB-AAPY-01A	PAAD	6.09
TCGA-FB-A545-01A	PAAD	6.13
TCGA-2J-AABH-01A	PAAD	6.22
TCGA-HZ-8317-01A	PAAD	6.06
TCGA-2J-AABO-01A	PAAD	6.81
TCGA-3A-A9IS-01A	PAAD	4.74
TCGA-IB-7891-01A	PAAD	6.55
TCGA-2J-AABA-01A	PAAD	6.5
TCGA-US-A776-01A	PAAD	6.27
TCGA-IB-7886-01A	PAAD	6.84
TCGA-PZ-A5RE-01A	PAAD	6.03
TCGA-XD-AAUG-01A	PAAD	5.61
TCGA-HV-A5A3-01A	PAAD	6.95
TCGA-2L-AAQJ-01A	PAAD	6.37
TCGA-HZ-A77O-01A	PAAD	6.16
TCGA-FB-AAQ2-01A	PAAD	6.96
TCGA-FB-AAQ1-01A	PAAD	5.65
TCGA-HV-AA8V-01A	PAAD	6.07
TCGA-3A-A9IH-01A	PAAD	7
TCGA-3A-A9IO-01A	PAAD	5.09
TCGA-IB-AAUW-01A	PAAD	6.44
TCGA-HV-AA8X-01A	PAAD	8.25
TCGA-F2-A7TX-01A	PAAD	6.08
TCGA-IB-7644-01A	PAAD	6.92
TCGA-IB-A5SS-01A	PAAD	6.81
TCGA-IB-7897-01A	PAAD	6.43
TCGA-IB-7889-01A	PAAD	6.22
TCGA-US-A77J-01A	PAAD	5.43
TCGA-3A-A9IZ-01A	PAAD	7.4
TCGA-IB-8126-01A	PAAD	4.63
TCGA-HZ-8001-01A	PAAD	5.77
TCGA-2J-AABR-01A	PAAD	6.5
TCGA-F2-A44H-01A	PAAD	5.93
TCGA-Z5-AAPL-01A	PAAD	5.3
TCGA-IB-AAUR-01A	PAAD	5.5
TCGA-F2-A8YN-01A	PAAD	6.97

TCGA-3A-A9IJ-01A	PAAD	4.86
TCGA-US-A77G-01A	PAAD	6.87
TCGA-FB-AAPU-01A	PAAD	7.16
TCGA-IB-7885-01A	PAAD	6.91
TCGA-IB-AAUO-01A	PAAD	5.62
TCGA-IB-7645-01A	PAAD	6.95
TCGA-F2-6879-01A	PAAD	7.36
TCGA-FB-AAQ6-01A	PAAD	6.46
TCGA-HZ-A9TJ-06A	PAAD	7.08
TCGA-IB-AAUU-01A	PAAD	7.15
TCGA-HZ-7924-01A	PAAD	7.07
TCGA-XD-AAUH-01A	PAAD	5.78
TCGA-IB-AAUM-01A	PAAD	5.17
TCGA-S4-A8RM-01A	PAAD	6.94
TCGA-S4-A8RP-01A	PAAD	6.34
TCGA-HZ-A49I-01A	PAAD	6.68
TCGA-2J-AABI-01A	PAAD	5.27
TCGA-3A-A9I9-01A	PAAD	5.75
TCGA-HZ-8636-01A	PAAD	7.24
TCGA-HZ-8005-01A	PAAD	6.2
TCGA-HV-A5A4-01A	PAAD	6.34
TCGA-2J-AAB4-01A	PAAD	6.49
TCGA-HV-A7OL-01A	PAAD	5.61
TCGA-IB-AAUP-01A	PAAD	6.04
TCGA-FB-A5VM-01A	PAAD	5.86
TCGA-IB-A5SQ-01A	PAAD	5.98
TCGA-2J-AABE-01A	PAAD	6.87
TCGA-YH-A8SY-01A	PAAD	6.3
TCGA-HZ-8638-01A	PAAD	7.23
TCGA-IB-A6UF-01A	PAAD	6.12
TCGA-HZ-7918-01A	PAAD	7.4
TCGA-US-A77E-01A	PAAD	6.16
TCGA-RB-A7B8-01A	PAAD	6.2
TCGA-IB-A7M4-01A	PAAD	6.45
TCGA-IB-7654-01A	PAAD	6.41
TCGA-HZ-7922-01A	PAAD	7.07
TCGA-2J-AAB1-01A	PAAD	6.34
TCGA-IB-AAUS-01A	PAAD	6.39
TCGA-IB-AAUT-01A	PAAD	5.82
TCGA-2L-AAQM-01A	PAAD	4.27
TCGA-3A-A9IX-01A	PAAD	6.28
TCGA-2L-AAQE-01A	PAAD	7.14
TCGA-3A-A9I5-01A	PAAD	6.49
TCGA-FB-AAQ0-01A	PAAD	5.71
TCGA-2J-AABP-01A	PAAD	6.25
TCGA-FB-A4P6-01A	PAAD	6.38
TCGA-XD-AAUI-01A	PAAD	6.78
TCGA-3E-AAAZ-01A	PAAD	6.5
TCGA-RB-AA9M-01A	PAAD	6.55
TCGA-3E-AAAY-01A	PAAD	6.37
TCGA-FB-A4P5-01A	PAAD	6.17
TCGA-RL-AAAS-01A	PAAD	5.91
TCGA-S4-A8RO-01A	PAAD	7.89
TCGA-YB-A89D-01A	PAAD	6.69
TCGA-IB-A5SO-01A	PAAD	5.97
TCGA-HZ-A8P1-01A	PAAD	6.93
TCGA-XN-A8T3-01A	PAAD	6.2
TCGA-IB-A5SP-01A	PAAD	7.04

TCGA-IB-7646-01A	PAAD	6.49
TCGA-3A-A9IU-01A	PAAD	6.26
TCGA-2J-AABV-01A	PAAD	3.32
TCGA-3A-A9IV-01A	PAAD	4.84
TCGA-Q3-AA2A-01A	PAAD	5.82
TCGA-F2-A44G-01A	PAAD	6.21
TCGA-IB-7652-01A	PAAD	7.61
TCGA-LB-A9Q5-01A	PAAD	5.12
TCGA-FB-A78T-01A	PAAD	6.19
TCGA-IB-7893-01A	PAAD	7.13
TCGA-3A-A9IB-01A	PAAD	5.87
TCGA-IB-A6UG-01A	PAAD	5.45
TCGA-HZ-A49G-01A	PAAD	6.25
TCGA-IB-7651-01A	PAAD	6.37
TCGA-F2-7273-01A	PAAD	6.16
TCGA-IB-7890-01A	PAAD	6.75
TCGA-3A-A9I7-01A	PAAD	6.33
TCGA-FB-AAQ3-01A	PAAD	6.22
TCGA-HZ-A77P-01A	PAAD	6.32
TCGA-HZ-A4BK-01A	PAAD	6.45
TCGA-XD-AAUL-01A	PAAD	6.37
TCGA-IB-AAUV-01A	PAAD	5.68
TCGA-H6-A45N-01A	PAAD	6.7
TCGA-HZ-8519-01A	PAAD	6.6
TCGA-2J-AABF-01A	PAAD	6.94
TCGA-IB-A7LX-01A	PAAD	6.08
TCGA-3A-A9J0-01A	PAAD	7.33
TCGA-HZ-A8P0-01A	PAAD	6.39
TCGA-2J-AAB9-01A	PAAD	6.05
TCGA-IB-AAUQ-01A	PAAD	6.19
TCGA-2L-AAQI-01A	PAAD	7.12
TCGA-F2-6880-01A	PAAD	3.84
TCGA-L1-A7W4-01A	PAAD	6.92
TCGA-IB-7647-01A	PAAD	7.18
TCGA-F2-7276-01A	PAAD	6.51
TCGA-FB-A7DR-01A	PAAD	5.95
TCGA-IB-7649-01A	PAAD	6.02
TCGA-HZ-7925-01A	PAAD	6.53
TCGA-2L-AAQA-01A	PAAD	7.19
TCGA-YY-A8LH-01A	PAAD	6.44
TCGA-M8-A5N4-01A	PAAD	5.33
TCGA-IB-7887-01A	PAAD	7.48
TCGA-FB-AAPZ-01A	PAAD	6.21
TCGA-HV-A5A5-01A	PAAD	6.23
TCGA-HZ-A49H-01A	PAAD	5.91
TCGA-Q3-A5QY-01A	PAAD	5.24
TCGA-HZ-7920-01A	PAAD	5.93
TCGA-HZ-7919-01A	PAAD	8.02
TCGA-HZ-8315-01A	PAAD	6.43
TCGA-HZ-7926-01A	PAAD	6.14
TCGA-IB-7888-01A	PAAD	6.77
TCGA-HZ-A4BH-01A	PAAD	7.8
TCGA-US-A774-01A	PAAD	6.76
TCGA-H8-A6C1-01A	PAAD	6.07
TCGA-XN-A8T5-01A	PAAD	5.81
TCGA-2J-AABU-01A	PAAD	6.77
TCGA-2J-AABK-01A	PAAD	6.11
TCGA-LB-A8F3-01A	PAAD	5.57

TCGA-HZ-A77Q-01A	PAAD	6.11
TCGA-FB-AAPQ-01A	PAAD	7.22
TCGA-3A-A9IN-01A	PAAD	4.46
TCGA-QR-A70H-01A	PCPG	3.94
TCGA-QR-A70X-01A	PCPG	4.67
TCGA-S7-A7WW-01A	PCPG	5.11
TCGA-PR-A5PF-01A	PCPG	4.33
TCGA-RW-A689-01A	PCPG	4.87
TCGA-QR-A6GR-01A	PCPG	4.64
TCGA-QR-A6GZ-05A	PCPG	4.59
TCGA-RW-A681-01A	PCPG	4.26
TCGA-WB-A81W-01A	PCPG	4.98
TCGA-TT-A6YO-01A	PCPG	7.43
TCGA-WB-A81J-01A	PCPG	4.93
TCGA-QR-A6GT-01A	PCPG	5.01
TCGA-RW-A68G-01A	PCPG	5.11
TCGA-RW-A68D-01A	PCPG	5.34
TCGA-WB-A81R-01A	PCPG	4.93
TCGA-RW-A68C-01A	PCPG	4.87
TCGA-QR-A70U-01A	PCPG	4.03
TCGA-WB-A81K-01A	PCPG	5.46
TCGA-QR-A70O-01A	PCPG	2.24
TCGA-TT-A6YJ-01A	PCPG	5.15
TCGA-RW-A68B-01A	PCPG	5.06
TCGA-RW-A68A-01A	PCPG	5.06
TCGA-S7-A7WL-01A	PCPG	4.86
TCGA-QR-A6H2-01A	PCPG	3.42
TCGA-QT-A5XO-01A	PCPG	5.34
TCGA-RT-A6Y9-01A	PCPG	4.58
TCGA-WB-A80K-01A	PCPG	4.34
TCGA-SR-A6N0-01A	PCPG	7.66
TCGA-SR-A6MX-05A	PCPG	2.94
TCGA-WB-A81M-01A	PCPG	4.87
TCGA-TT-A6YN-01A	PCPG	4.83
TCGA-S7-A7WP-01A	PCPG	5.2
TCGA-QR-A707-01A	PCPG	4.37
TCGA-QR-A6GS-01A	PCPG	5.09
TCGA-WB-A80V-01A	PCPG	4.11
TCGA-RW-A67X-01A	PCPG	4.5
TCGA-QR-A7IP-01A	PCPG	4.16
TCGA-SR-A6MR-01A	PCPG	4.39
TCGA-QT-A5XP-01A	PCPG	4.61
TCGA-S7-A7WM-01A	PCPG	4.26
TCGA-RW-A68F-01A	PCPG	4.14
TCGA-WB-A822-01A	PCPG	4.12
TCGA-WB-A815-01A	PCPG	4.49
TCGA-XG-A823-01A	PCPG	4.88
TCGA-W2-A7H7-01A	PCPG	4.4
TCGA-S7-A7WX-01A	PCPG	5.24
TCGA-SP-A6QF-01A	PCPG	3.65
TCGA-QR-A70J-01A	PCPG	4.65
TCGA-QR-A708-01A	PCPG	3.02
TCGA-SQ-A6I4-01A	PCPG	4.91
TCGA-WB-A81N-01A	PCPG	4.9
TCGA-QR-A6H1-01A	PCPG	4.81
TCGA-QR-A6ZZ-01A	PCPG	4.77
TCGA-S7-A7WU-01A	PCPG	4.3
TCGA-QR-A70G-01B	PCPG	6.34

TCGA-QT-A5XN-01A	PCPG	4.27
TCGA-QT-A5XL-01A	PCPG	5.55
TCGA-QR-A703-01A	PCPG	4.65
TCGA-W2-A7HD-01A	PCPG	4.97
TCGA-QT-A69Q-01A	PCPG	4.2
TCGA-QR-A70R-01A	PCPG	3.52
TCGA-WB-A816-01A	PCPG	6.8
TCGA-RT-A6YA-01A	PCPG	3.26
TCGA-WB-A820-01A	PCPG	5.93
TCGA-QR-A70V-01A	PCPG	4.58
TCGA-S7-A7WQ-01A	PCPG	5.29
TCGA-QR-A702-01A	PCPG	3.98
TCGA-QR-A70W-01A	PCPG	3.65
TCGA-QR-A6H0-01A	PCPG	4.34
TCGA-SP-A6QK-01A	PCPG	5.57
TCGA-QR-A70D-01A	PCPG	5.27
TCGA-SR-A6MT-01A	PCPG	4.18
TCGA-WB-A81F-01A	PCPG	5.61
TCGA-QR-A70Q-01A	PCPG	3.56
TCGA-W2-A7HF-01A	PCPG	4.75
TCGA-WB-A81S-01A	PCPG	4.99
TCGA-SR-A6MU-01A	PCPG	4.77
TCGA-QT-A5XM-01A	PCPG	5.37
TCGA-WB-A81A-01A	PCPG	4.32
TCGA-SR-A6MX-01A	PCPG	3.66
TCGA-QR-A70M-01A	PCPG	4.9
TCGA-SR-A6MP-01A	PCPG	4.93
TCGA-WB-A80P-01A	PCPG	4.82
TCGA-RW-A8AZ-01A	PCPG	3.8
TCGA-QR-A6GO-01A	PCPG	4.53
TCGA-QR-A6H5-01A	PCPG	5.17
TCGA-WB-A81V-01A	PCPG	4.5
TCGA-QR-A70I-01A	PCPG	4.43
TCGA-PR-A5PH-01A	PCPG	4.44
TCGA-QR-A6GX-01A	PCPG	4.36
TCGA-WB-A81D-01A	PCPG	4.89
TCGA-RW-A67Y-01A	PCPG	4.92
TCGA-RW-A67V-01A	PCPG	2.99
TCGA-W2-A7HE-01A	PCPG	6.88
TCGA-W2-A7HA-01B	PCPG	5.21
TCGA-SR-A6MZ-01A	PCPG	6.3
TCGA-QR-A6GZ-01A	PCPG	4.5
TCGA-P7-A5NX-01A	PCPG	5.39
TCGA-QR-A6H3-01A	PCPG	3.47
TCGA-SR-A6MQ-01A	PCPG	5
TCGA-TT-A6YK-01A	PCPG	3.64
TCGA-S7-A7WT-01A	PCPG	3.67
TCGA-S7-A7X2-01A	PCPG	4.99
TCGA-SP-A6QJ-01A	PCPG	4.59
TCGA-P7-A5NY-01A	PCPG	5.25
TCGA-RW-A680-01A	PCPG	2.97
TCGA-RW-A67W-01A	PCPG	3.67
TCGA-QR-A6H4-01A	PCPG	4.22
TCGA-S7-A7WV-01A	PCPG	5.86
TCGA-WB-A817-01A	PCPG	6.31
TCGA-SR-A6MX-06A	PCPG	3.55
TCGA-S7-A7X1-01A	PCPG	4.66
TCGA-W2-A7UY-01A	PCPG	3.95

TCGA-TT-A6YP-01A	PCPG	5.4
TCGA-WB-A80N-01A	PCPG	4.71
TCGA-WB-A80Q-01A	PCPG	2.83
TCGA-SR-A6MY-01A	PCPG	5.35
TCGA-QR-A6GY-01A	PCPG	3.59
TCGA-SP-A6QH-01A	PCPG	4.41
TCGA-S7-A7WN-01A	PCPG	5.1
TCGA-S7-A7WO-01A	PCPG	7.81
TCGA-P8-A5KD-01A	PCPG	5.97
TCGA-W2-A7HB-01A	PCPG	5.37
TCGA-RW-A684-01A	PCPG	4.37
TCGA-PR-A5PG-01A	PCPG	5.45
TCGA-RM-A68T-01A	PCPG	4.76
TCGA-SR-A6MS-01A	PCPG	4.47
TCGA-WB-A80O-01A	PCPG	5.2
TCGA-QT-A5XJ-01A	PCPG	6.24
TCGA-P7-A5NY-05A	PCPG	6.06
TCGA-W2-A7HH-01A	PCPG	4.64
TCGA-P8-A6RY-01A	PCPG	3.54
TCGA-RM-A68W-01A	PCPG	4.64
TCGA-RW-A685-01A	PCPG	5.18
TCGA-WB-A81T-01A	PCPG	5.57
TCGA-QR-A6GU-01A	PCPG	4.69
TCGA-QR-A70T-01A	PCPG	3.37
TCGA-RW-A7D0-01A	PCPG	4.97
TCGA-QR-A70E-01A	PCPG	4.62
TCGA-RW-A7CZ-01A	PCPG	4.66
TCGA-WB-A81I-01A	PCPG	4.5
TCGA-QR-A70K-01A	PCPG	3.84
TCGA-SP-A6QD-01A	PCPG	3.44
TCGA-QR-A7IN-01A	PCPG	4.26
TCGA-S7-A7X0-01A	PCPG	4.66
TCGA-WB-A81P-01A	PCPG	4.64
TCGA-QR-A705-01A	PCPG	3.52
TCGA-W2-A7H5-01B	PCPG	4.67
TCGA-RT-A6YC-01A	PCPG	4.96
TCGA-QR-A706-01A	PCPG	4.44
TCGA-QR-A700-01A	PCPG	5.05
TCGA-RX-A8JQ-01A	PCPG	5.3
TCGA-QR-A70C-01A	PCPG	3.16
TCGA-WB-A818-01A	PCPG	4.92
TCGA-WB-A81Q-01A	PCPG	5.8
TCGA-SQ-A6I6-01A	PCPG	4.93
TCGA-WB-A819-01A	PCPG	5.55
TCGA-W2-A7HC-01A	PCPG	5.79
TCGA-QT-A5XK-01A	PCPG	4.86
TCGA-SR-A6MV-01A	PCPG	3.61
TCGA-WB-A81H-01A	PCPG	6.29
TCGA-WB-A81G-01A	PCPG	4.82
TCGA-WB-A80Y-01A	PCPG	4.34
TCGA-QR-A6GW-01A	PCPG	5.35
TCGA-QR-A70A-01A	PCPG	3.82
TCGA-SP-A6QG-01A	PCPG	3.61
TCGA-RW-A686-01A	PCPG	4.17
TCGA-QR-A70P-01A	PCPG	4.34
TCGA-SP-A6QC-01A	PCPG	3.39
TCGA-P8-A6RX-01A	PCPG	5.41
TCGA-SA-A6C2-01A	PCPG	5.13

TCGA-WB-A814-01A	PCPG	4.66
TCGA-P8-A5KC-01A	PCPG	4.74
TCGA-RW-A686-06A	PCPG	3.87
TCGA-QR-A70N-01A	PCPG	3.98
TCGA-RW-A688-01A	PCPG	4.67
TCGA-QT-A7U0-01A	PCPG	4.73
TCGA-WB-A81E-01A	PCPG	5.36
TCGA-WB-A821-01A	PCPG	4.14
TCGA-WB-A80M-01A	PCPG	4.57
TCGA-QR-A6H6-01A	PCPG	3.49
TCGA-S7-A7WR-01A	PCPG	5.47
TCGA-SP-A6QI-01A	PCPG	4.56
TCGA-WB-A80L-01A	PCPG	4.94
TCGA-J9-A8CL-01A	PRAD	7.53
TCGA-EJ-A46B-01A	PRAD	8.28
TCGA-EJ-5509-01A	PRAD	7.83
TCGA-EJ-7782-01A	PRAD	7.91
TCGA-J9-A52C-01A	PRAD	6.69
TCGA-G9-6356-01A	PRAD	7.9
TCGA-EJ-7783-01A	PRAD	8.52
TCGA-KK-A8IG-01A	PRAD	7.12
TCGA-EJ-5505-01A	PRAD	9.62
TCGA-EJ-7789-01A	PRAD	7.12
TCGA-XK-AAK1-01A	PRAD	8.7
TCGA-YL-A9WH-01A	PRAD	8.17
TCGA-EJ-5516-01A	PRAD	9.77
TCGA-YL-A8SL-01B	PRAD	8.47
TCGA-YL-A8SQ-01B	PRAD	8.46
TCGA-J4-A83K-01A	PRAD	8.18
TCGA-KK-A6E7-01A	PRAD	8.72
TCGA-KK-A7B2-01A	PRAD	7.69
TCGA-HC-7213-01A	PRAD	8.96
TCGA-HC-8257-01A	PRAD	7.65
TCGA-G9-6343-01A	PRAD	7.86
TCGA-HC-A8D1-01A	PRAD	7.98
TCGA-YL-A8HK-01A	PRAD	8.58
TCGA-XK-AAJP-01A	PRAD	9.03
TCGA-CH-5751-01A	PRAD	7.69
TCGA-KK-A7B1-01A	PRAD	8.28
TCGA-G9-6354-01A	PRAD	7.6
TCGA-G9-6333-01A	PRAD	8.6
TCGA-EJ-5498-01A	PRAD	8.64
TCGA-KK-A8ID-01A	PRAD	7.63
TCGA-V1-A8ML-01A	PRAD	8.33
TCGA-EJ-A65G-01A	PRAD	8.23
TCGA-KC-A7F3-01A	PRAD	8.56
TCGA-G9-7522-01A	PRAD	8.72
TCGA-KC-A7FD-01A	PRAD	8.53
TCGA-EJ-5501-01A	PRAD	8.53
TCGA-HC-A6AO-01A	PRAD	6.59
TCGA-CH-5744-01A	PRAD	8.63
TCGA-KC-A7FA-01A	PRAD	7.98
TCGA-V1-A9O7-01A	PRAD	6.95
TCGA-EJ-7328-01A	PRAD	7.26
TCGA-HC-8213-01A	PRAD	8.16
TCGA-CH-5737-01A	PRAD	8.42
TCGA-EJ-5525-01A	PRAD	8.59
TCGA-HC-A9TH-01A	PRAD	8.25

TCGA-X4-A8KQ-01A	PRAD	5.73
TCGA-XJ-A83H-01A	PRAD	8.2
TCGA-4L-AA1F-01A	PRAD	7.97
TCGA-XJ-A9DK-01A	PRAD	8.62
TCGA-EJ-5542-01A	PRAD	9.14
TCGA-J9-A8CP-01A	PRAD	8.59
TCGA-EJ-5496-01A	PRAD	8.36
TCGA-HI-7170-01A	PRAD	7.25
TCGA-J4-A67M-01A	PRAD	6.79
TCGA-EJ-7791-01A	PRAD	9.21
TCGA-KK-A8IM-01A	PRAD	8.89
TCGA-M7-A720-01A	PRAD	8.79
TCGA-EJ-7325-01B	PRAD	6.45
TCGA-2A-A8VL-01A	PRAD	7.91
TCGA-XQ-A8TB-01A	PRAD	7.32
TCGA-YL-A8S8-01A	PRAD	7.51
TCGA-HC-7075-01A	PRAD	8.47
TCGA-CH-5738-01A	PRAD	8.57
TCGA-EJ-7318-01B	PRAD	8.43
TCGA-EJ-7784-01A	PRAD	8.41
TCGA-ZG-A9L2-01A	PRAD	8.67
TCGA-KK-A59X-01A	PRAD	8.23
TCGA-KK-A59Y-01A	PRAD	7.84
TCGA-J4-AATZ-01A	PRAD	8.81
TCGA-VN-A88P-01A	PRAD	7.85
TCGA-HC-A6AS-01A	PRAD	7.53
TCGA-EJ-AB20-01A	PRAD	6.37
TCGA-KK-A8I6-01A	PRAD	8.78
TCGA-VN-A88L-01A	PRAD	7.44
TCGA-G9-6361-01A	PRAD	7.92
TCGA-VP-A87C-01A	PRAD	7.36
TCGA-HC-7738-01A	PRAD	7.94
TCGA-HC-8216-01A	PRAD	8.37
TCGA-EJ-7321-01A	PRAD	7.77
TCGA-HC-7077-01A	PRAD	8.16
TCGA-KK-A7B0-01A	PRAD	6.8
TCGA-HC-7737-01A	PRAD	7.96
TCGA-2A-A8VV-01A	PRAD	8.01
TCGA-FC-A66V-01A	PRAD	7.81
TCGA-CH-5739-01A	PRAD	8.54
TCGA-H9-A6BY-01A	PRAD	7.27
TCGA-G9-7509-01A	PRAD	8.22
TCGA-EJ-7317-01A	PRAD	8.36
TCGA-KK-A7AZ-01A	PRAD	7.39
TCGA-V1-A9ZK-01A	PRAD	8.48
TCGA-J4-8200-01A	PRAD	8.56
TCGA-CH-5761-01A	PRAD	9.2
TCGA-KC-A7FE-01A	PRAD	7.97
TCGA-EJ-A65F-01A	PRAD	8.14
TCGA-CH-5767-01A	PRAD	8.9
TCGA-EJ-A65J-01A	PRAD	7.73
TCGA-ZG-A9LM-01A	PRAD	6.39
TCGA-YJ-A8SW-01A	PRAD	7.86
TCGA-VP-A87H-01A	PRAD	8.29
TCGA-V1-A9OH-01A	PRAD	7.62
TCGA-TP-A8TV-01A	PRAD	8.11
TCGA-HC-7745-01A	PRAD	7.38
TCGA-VP-A876-01A	PRAD	8.16

TCGA-YL-A8HJ-01A	PRAD	6.84
TCGA-KK-A8I5-01A	PRAD	8.57
TCGA-CH-5791-01A	PRAD	8.89
TCGA-EJ-7330-01A	PRAD	8.37
TCGA-FC-A8O0-01A	PRAD	7.1
TCGA-HC-8256-01A	PRAD	8.91
TCGA-J9-A52D-01A	PRAD	9.05
TCGA-EJ-5512-01A	PRAD	8.32
TCGA-HC-A8CY-01A	PRAD	7.12
TCGA-EJ-AB27-01A	PRAD	8.7
TCGA-G9-6373-01A	PRAD	7.69
TCGA-G9-6332-01A	PRAD	8.53
TCGA-EJ-8474-01A	PRAD	7.85
TCGA-J4-A83M-01A	PRAD	8.02
TCGA-G9-7521-01A	PRAD	8.46
TCGA-XJ-A83F-01A	PRAD	7.66
TCGA-EJ-7218-01B	PRAD	7.95
TCGA-EJ-5521-01A	PRAD	9.36
TCGA-CH-5750-01A	PRAD	8.61
TCGA-YL-A9WY-01A	PRAD	7.88
TCGA-V1-A9OF-01A	PRAD	9.11
TCGA-V1-A8WW-01A	PRAD	9.19
TCGA-EJ-A65M-01A	PRAD	8.84
TCGA-XJ-A83G-01A	PRAD	8.68
TCGA-XK-AAJA-01A	PRAD	8.12
TCGA-EJ-5497-01A	PRAD	9.35
TCGA-EJ-A7NK-01A	PRAD	8.36
TCGA-EJ-A8FO-01A	PRAD	8.54
TCGA-YL-A8HO-01A	PRAD	7.78
TCGA-G9-6498-01A	PRAD	7.35
TCGA-VN-A88I-01A	PRAD	7.08
TCGA-EJ-5527-01A	PRAD	8.38
TCGA-G9-6353-01A	PRAD	8
TCGA-HC-A9TE-01A	PRAD	8.03
TCGA-QU-A6IM-01A	PRAD	6.86
TCGA-EJ-A7NN-01A	PRAD	8.26
TCGA-KK-A8II-01A	PRAD	8.77
TCGA-CH-5789-01A	PRAD	8.9
TCGA-YL-A8SR-01B	PRAD	8.63
TCGA-V1-A8WS-01A	PRAD	7.24
TCGA-KK-A6E4-01A	PRAD	6.8
TCGA-EJ-5506-01A	PRAD	9.17
TCGA-G9-6369-01A	PRAD	8.67
TCGA-J4-A83J-01A	PRAD	8.4
TCGA-KK-A8IJ-01A	PRAD	8.35
TCGA-G9-6348-01A	PRAD	7.74
TCGA-G9-6329-01A	PRAD	7.44
TCGA-HC-7752-01A	PRAD	5.25
TCGA-HC-7736-01A	PRAD	9.78
TCGA-VN-A88K-01A	PRAD	7.57
TCGA-J4-8198-01A	PRAD	8.09
TCGA-MG-AAMC-01A	PRAD	7.65
TCGA-KK-A59V-01A	PRAD	6.7
TCGA-XK-AAJR-01A	PRAD	8.26
TCGA-EJ-A65D-01A	PRAD	6.53
TCGA-V1-A8WV-01A	PRAD	7.11
TCGA-HC-7231-01A	PRAD	8.91
TCGA-EJ-5531-01A	PRAD	9.22

TCGA-YL-A9WK-01A	PRAD	7.42
TCGA-CH-5741-01A	PRAD	8.39
TCGA-WW-A8ZI-01A	PRAD	7.96
TCGA-VN-A88O-01A	PRAD	8.24
TCGA-ZG-A9LB-01A	PRAD	6.08
TCGA-YL-A8HM-01A	PRAD	9.57
TCGA-VP-A875-01A	PRAD	7.41
TCGA-EJ-7327-01A	PRAD	7.89
TCGA-VN-A88N-01A	PRAD	9.15
TCGA-KK-A59Z-01A	PRAD	6.88
TCGA-HC-7747-01A	PRAD	8.25
TCGA-2A-A8W1-01A	PRAD	8.55
TCGA-V1-A9O5-01A	PRAD	8.67
TCGA-EJ-A7NF-01A	PRAD	7.69
TCGA-HC-8266-01A	PRAD	7.79
TCGA-KK-A7AY-01A	PRAD	7.48
TCGA-HC-7744-01A	PRAD	9.62
TCGA-V1-A9O9-01A	PRAD	8.49
TCGA-V1-A8MJ-01A	PRAD	8.32
TCGA-HC-A4ZV-01A	PRAD	7.05
TCGA-EJ-5532-01A	PRAD	9.13
TCGA-ZG-A8QX-01A	PRAD	8.18
TCGA-XQ-A8TA-01A	PRAD	9.21
TCGA-FC-7961-01A	PRAD	8.91
TCGA-G9-6347-01A	PRAD	6.48
TCGA-ZG-A8QZ-01A	PRAD	8.51
TCGA-KK-A7AQ-01A	PRAD	7.07
TCGA-EJ-7115-01A	PRAD	8.82
TCGA-J4-A67R-01A	PRAD	7.55
TCGA-HC-A631-01A	PRAD	8.37
TCGA-VP-A87E-01A	PRAD	8.03
TCGA-CH-5740-01A	PRAD	8.58
TCGA-KK-A6E1-01A	PRAD	9.2
TCGA-G9-6385-01A	PRAD	8.06
TCGA-HC-7211-01A	PRAD	8.36
TCGA-ZG-A9L5-01A	PRAD	7.69
TCGA-HC-8261-01B	PRAD	7.11
TCGA-EJ-A8FN-01A	PRAD	8.29
TCGA-QU-A6IL-01A	PRAD	5.51
TCGA-HC-A632-01A	PRAD	6.77
TCGA-HC-A6HX-01A	PRAD	7.03
TCGA-J4-AATV-01A	PRAD	7.49
TCGA-M7-A724-01A	PRAD	7.76
TCGA-YL-A8HL-01A	PRAD	9.03
TCGA-G9-6364-01A	PRAD	7.98
TCGA-EJ-7331-01A	PRAD	8.38
TCGA-KC-A4BV-01A	PRAD	8.19
TCGA-J4-A67K-01A	PRAD	6.63
TCGA-YL-A8SP-01B	PRAD	7.05
TCGA-EJ-7794-01A	PRAD	8.71
TCGA-YL-A8SI-01A	PRAD	8.72
TCGA-EJ-A46H-01A	PRAD	7.86
TCGA-HC-7819-01A	PRAD	8.93
TCGA-G9-6371-01A	PRAD	8.25
TCGA-ZG-A9L9-01A	PRAD	6.83
TCGA-EJ-5508-01A	PRAD	8.53
TCGA-G9-6339-01A	PRAD	8.07
TCGA-YL-A9WL-01A	PRAD	8.19

TCGA-V1-A8WN-01A	PRAD	8.01
TCGA-KK-A6E0-01A	PRAD	10.1
TCGA-Y6-A9XI-01A	PRAD	8.62
TCGA-G9-6496-01A	PRAD	7.54
TCGA-HC-7232-01A	PRAD	8.97
TCGA-ZG-A9L4-01A	PRAD	7.82
TCGA-G9-6379-01A	PRAD	6.96
TCGA-ZG-A9LZ-01A	PRAD	8.17
TCGA-QU-A6IN-01A	PRAD	6.28
TCGA-KK-A8IK-01A	PRAD	6.73
TCGA-CH-5752-01A	PRAD	9.2
TCGA-KK-A7AV-01A	PRAD	8.53
TCGA-V1-A9ZG-01A	PRAD	7.44
TCGA-J9-A8CM-01A	PRAD	7.18
TCGA-YL-A8SF-01A	PRAD	6.54
TCGA-EJ-5530-01A	PRAD	9.19
TCGA-V1-A9O5-06A	PRAD	7.66
TCGA-EJ-A7NJ-01A	PRAD	9.23
TCGA-KC-A7F6-01A	PRAD	7.58
TCGA-EJ-7797-01A	PRAD	8.15
TCGA-VN-A88R-01A	PRAD	8.15
TCGA-XK-AAJ3-01A	PRAD	8.67
TCGA-HC-8259-01A	PRAD	8.31
TCGA-G9-6494-01A	PRAD	7.69
TCGA-G9-A9S0-01A	PRAD	9.69
TCGA-EJ-A46F-01A	PRAD	7.6
TCGA-XK-AAIV-01A	PRAD	9.26
TCGA-KK-A6E5-01A	PRAD	7.99
TCGA-YL-A8SK-01B	PRAD	7.42
TCGA-HC-7749-01A	PRAD	8.44
TCGA-J9-A8CN-01A	PRAD	7.8
TCGA-EJ-5515-01A	PRAD	8.83
TCGA-EJ-8470-01A	PRAD	8.74
TCGA-EJ-A8FU-01A	PRAD	8.15
TCGA-ZG-A9LN-01A	PRAD	7.13
TCGA-YL-A8SB-01A	PRAD	7.49
TCGA-XK-AAIW-01A	PRAD	8.94
TCGA-ZG-A9N3-01A	PRAD	6.83
TCGA-EJ-7785-01A	PRAD	8.74
TCGA-J4-A67L-01A	PRAD	8.52
TCGA-V1-A9ZR-01A	PRAD	6.8
TCGA-KK-A8IL-01A	PRAD	8.02
TCGA-ZG-A9ND-01A	PRAD	8.56
TCGA-G9-6377-01A	PRAD	7.88
TCGA-M7-A722-01A	PRAD	6.26
TCGA-XJ-A9DI-01A	PRAD	6.96
TCGA-HC-8265-01A	PRAD	6.99
TCGA-KC-A7F5-01A	PRAD	7.48
TCGA-YL-A8SC-01A	PRAD	8.09
TCGA-G9-6338-01A	PRAD	6.59
TCGA-HI-7168-01A	PRAD	7.07
TCGA-KK-A8I9-01A	PRAD	8.21
TCGA-EJ-7315-01A	PRAD	8.55
TCGA-HC-7750-01A	PRAD	8.03
TCGA-V1-A8MG-01A	PRAD	7.89
TCGA-CH-5771-01A	PRAD	7.32
TCGA-KK-A6E3-01A	PRAD	8
TCGA-EJ-5518-01A	PRAD	9.85

TCGA-V1-A9ZI-01A	PRAD	7.14
TCGA-EJ-7312-01B	PRAD	9.14
TCGA-HC-8260-01A	PRAD	8.1
TCGA-HC-8265-01B	PRAD	5.57
TCGA-CH-5743-01A	PRAD	7
TCGA-FC-A4JI-01A	PRAD	7.26
TCGA-EJ-7792-01A	PRAD	8.43
TCGA-EJ-7781-01A	PRAD	7.93
TCGA-ZG-A9L0-01A	PRAD	7.2
TCGA-KK-A7AU-01A	PRAD	8.05
TCGA-HC-7081-01A	PRAD	9.37
TCGA-G9-6342-01A	PRAD	8.4
TCGA-HC-A6AQ-01A	PRAD	7.53
TCGA-HC-A6AN-01A	PRAD	9.81
TCGA-J4-A67N-01A	PRAD	7.36
TCGA-KC-A4BR-01A	PRAD	7.04
TCGA-VP-A879-01A	PRAD	7.81
TCGA-CH-5754-01A	PRAD	8.71
TCGA-M7-A71Y-01A	PRAD	8.03
TCGA-V1-A9OQ-01A	PRAD	7.65
TCGA-G9-6378-01A	PRAD	7.6
TCGA-VP-A87B-01A	PRAD	7.65
TCGA-CH-5763-01A	PRAD	8.71
TCGA-HI-7169-01A	PRAD	5.43
TCGA-G9-7519-01A	PRAD	8.01
TCGA-V1-A9OT-01A	PRAD	6.04
TCGA-HC-A76X-01A	PRAD	8.88
TCGA-J4-A67Q-01A	PRAD	6.42
TCGA-FC-7708-01A	PRAD	9.04
TCGA-EJ-5522-01A	PRAD	9.24
TCGA-VP-A87K-01A	PRAD	8.47
TCGA-SU-A7E7-01A	PRAD	8
TCGA-YL-A9WI-01A	PRAD	8.25
TCGA-J9-A52E-01A	PRAD	5.95
TCGA-G9-A9S7-01A	PRAD	8.57
TCGA-VN-A88M-01A	PRAD	7.32
TCGA-KK-A8I8-01A	PRAD	6.36
TCGA-J9-A52B-01A	PRAD	8.23
TCGA-YL-A8SA-01A	PRAD	8.76
TCGA-KK-A7AW-01A	PRAD	5.84
TCGA-EJ-A65E-01A	PRAD	9.63
TCGA-EJ-8468-01A	PRAD	9.02
TCGA-H9-A6BX-01A	PRAD	7.25
TCGA-HC-A6HY-01A	PRAD	7.01
TCGA-CH-5790-01A	PRAD	9.4
TCGA-KK-A8IF-01A	PRAD	7.22
TCGA-ZG-A9LY-01A	PRAD	7.29
TCGA-EJ-A8FP-01A	PRAD	8.19
TCGA-J4-A67T-01A	PRAD	8.25
TCGA-KK-A8I4-01A	PRAD	7.53
TCGA-EJ-A46D-01A	PRAD	8.15
TCGA-G9-6367-01A	PRAD	8.58
TCGA-2A-A8VT-01A	PRAD	8.41
TCGA-HC-7078-01A	PRAD	8.01
TCGA-HI-7171-01A	PRAD	8.01
TCGA-J4-A67S-01A	PRAD	7.82
TCGA-ZG-A8QY-01A	PRAD	8.73
TCGA-EJ-A46G-01A	PRAD	8.05

TCGA-G9-A9S4-01A	PRAD	7.43
TCGA-KK-A8IB-01A	PRAD	6.89
TCGA-EJ-7314-01A	PRAD	8.82
TCGA-V1-A8MK-01A	PRAD	8.55
TCGA-TP-A8TT-01A	PRAD	8.01
TCGA-QU-A6IP-01A	PRAD	8.1
TCGA-V1-A9Z8-01A	PRAD	7.69
TCGA-CH-5753-01A	PRAD	7.84
TCGA-HC-7821-01A	PRAD	8.72
TCGA-G9-6384-01A	PRAD	8.56
TCGA-ZG-A9L6-01A	PRAD	7.82
TCGA-CH-5745-01A	PRAD	8.7
TCGA-V1-A8MU-01A	PRAD	8.07
TCGA-EJ-7125-01A	PRAD	7.76
TCGA-EJ-7123-01A	PRAD	8.68
TCGA-G9-6363-01A	PRAD	9.59
TCGA-EJ-7786-01A	PRAD	8.9
TCGA-J4-A6G3-01A	PRAD	8.96
TCGA-EJ-A46I-01A	PRAD	7.81
TCGA-VN-A943-01A	PRAD	8.29
TCGA-ZG-A9M4-01A	PRAD	6.75
TCGA-YL-A9WX-01A	PRAD	7.5
TCGA-Y6-A8TL-01A	PRAD	9.12
TCGA-2A-A8VX-01A	PRAD	7.19
TCGA-2A-AAYU-01A	PRAD	8.19
TCGA-EJ-5503-01A	PRAD	8.7
TCGA-CH-5766-01A	PRAD	9.08
TCGA-EJ-A46E-01A	PRAD	7.3
TCGA-KK-A6DY-01A	PRAD	8.44
TCGA-KK-A7B3-01A	PRAD	8.07
TCGA-V1-A9OA-01A	PRAD	7.37
TCGA-VN-A88Q-01A	PRAD	8.88
TCGA-J4-A67O-01A	PRAD	6.75
TCGA-EJ-A6RA-01A	PRAD	8.37
TCGA-KK-A7B4-01A	PRAD	8.78
TCGA-TK-A8OK-01A	PRAD	6.95
TCGA-KK-A5A1-01A	PRAD	8.7
TCGA-HC-7209-01A	PRAD	9.73
TCGA-KK-A6E2-01A	PRAD	7.5
TCGA-KC-A4BN-01A	PRAD	8.06
TCGA-V1-A8MF-01A	PRAD	7.18
TCGA-EJ-5519-01A	PRAD	9.45
TCGA-QU-A6IO-01A	PRAD	6.83
TCGA-EJ-5510-01A	PRAD	8.14
TCGA-2A-A8W3-01A	PRAD	7.56
TCGA-HC-A76W-01A	PRAD	7.22
TCGA-HC-7820-01A	PRAD	9.22
TCGA-G9-7523-01A	PRAD	7.4
TCGA-V1-A8MM-01A	PRAD	7.55
TCGA-VP-A87D-01A	PRAD	7.82
TCGA-EJ-A7NM-01A	PRAD	7.43
TCGA-CH-5769-01A	PRAD	9.27
TCGA-ZG-A9LS-01A	PRAD	8.69
TCGA-EJ-A8FS-01A	PRAD	7.87
TCGA-EJ-5495-01A	PRAD	8.41
TCGA-ZG-A9KY-01A	PRAD	7.33
TCGA-EJ-8472-01A	PRAD	8.32
TCGA-ZG-A9NI-01A	PRAD	7.95

TCGA-HC-7230-01A	PRAD	8.85
TCGA-EJ-5524-01A	PRAD	8.53
TCGA-V1-A9Z7-01A	PRAD	8.28
TCGA-VP-A878-01A	PRAD	8.47
TCGA-2A-AAYF-01A	PRAD	8.91
TCGA-J4-A83I-01A	PRAD	8.09
TCGA-VP-A87J-01A	PRAD	9.16
TCGA-EJ-5502-01A	PRAD	9.62
TCGA-EJ-5517-01A	PRAD	8.63
TCGA-CH-5746-01A	PRAD	8.07
TCGA-XK-AAJT-01A	PRAD	6.34
TCGA-KK-A8IC-01A	PRAD	8.02
TCGA-G9-6365-01A	PRAD	7.93
TCGA-XJ-A9DX-01A	PRAD	7.22
TCGA-CH-5765-01A	PRAD	8.23
TCGA-ZG-A9MC-01A	PRAD	7.17
TCGA-V1-A8X3-01A	PRAD	8.58
TCGA-VP-A872-01A	PRAD	9.15
TCGA-2A-A8VO-01A	PRAD	7.76
TCGA-HC-7740-01B	PRAD	6.11
TCGA-KK-A8IH-01A	PRAD	8.81
TCGA-CH-5792-01A	PRAD	8.09
TCGA-XA-A8JR-01A	PRAD	7.88
TCGA-ZG-A9LU-01A	PRAD	6.87
TCGA-YL-A8SO-01B	PRAD	8.38
TCGA-XJ-A9DQ-01A	PRAD	8.15
TCGA-M7-A71Z-01A	PRAD	7.15
TCGA-M7-A721-01A	PRAD	8.12
TCGA-EJ-5507-01A	PRAD	9.37
TCGA-EJ-A7NG-01A	PRAD	8.25
TCGA-KK-A6E8-01A	PRAD	7.34
TCGA-2A-AAYO-01A	PRAD	7.68
TCGA-V1-A9OY-01A	PRAD	8.06
TCGA-HC-7210-01A	PRAD	8.94
TCGA-HC-7740-01A	PRAD	7.79
TCGA-J4-A83L-01A	PRAD	8.14
TCGA-HC-8264-01B	PRAD	7.68
TCGA-YL-A8SH-01B	PRAD	8.64
TCGA-J4-A83N-01A	PRAD	8.2
TCGA-CH-5788-01A	PRAD	9.67
TCGA-EJ-7793-01A	PRAD	8.58
TCGA-HC-8258-01B	PRAD	5.66
TCGA-HC-8258-01A	PRAD	8.05
TCGA-EJ-5526-01A	PRAD	8.47
TCGA-J9-A8CK-01A	PRAD	7.76
TCGA-HC-7818-01A	PRAD	8.9
TCGA-J4-A6M7-01A	PRAD	8.43
TCGA-CH-5764-01A	PRAD	7.97
TCGA-HC-7233-01A	PRAD	9.26
TCGA-VP-AA1N-01A	PRAD	8.02
TCGA-G9-7510-01A	PRAD	8.14
TCGA-G9-6362-01A	PRAD	7.2
TCGA-EJ-5511-01A	PRAD	7.84
TCGA-HC-7748-01A	PRAD	8.97
TCGA-G9-7525-01A	PRAD	7.33
TCGA-HC-8262-01A	PRAD	7.96
TCGA-M7-A725-01A	PRAD	8.27
TCGA-CH-5768-01A	PRAD	8.36

TCGA-V1-A9OL-01A	PRAD	8.02
TCGA-J4-A6G1-01A	PRAD	7.61
TCGA-KK-A8I7-01A	PRAD	9.04
TCGA-ZG-A9L1-01A	PRAD	6.95
TCGA-HC-7742-01A	PRAD	8.59
TCGA-HC-A8D0-01A	PRAD	6.76
TCGA-HC-7079-01A	PRAD	7.12
TCGA-YL-A9WJ-01A	PRAD	9.46
TCGA-EJ-A7NH-01A	PRAD	7.95
TCGA-V1-A9Z9-01A	PRAD	8.94
TCGA-H9-7775-01A	PRAD	8.35
TCGA-EJ-5504-01A	PRAD	8.59
TCGA-ZG-A8QW-01A	PRAD	9.21
TCGA-CH-5772-01A	PRAD	9.05
TCGA-J4-AAU2-01A	PRAD	8.32
TCGA-M7-A723-01A	PRAD	8.31
TCGA-XK-AAJU-01A	PRAD	8.45
TCGA-EJ-A65B-01A	PRAD	8.59
TCGA-G9-6366-01A	PRAD	8.94
TCGA-EJ-8469-01A	PRAD	8.88
TCGA-EJ-7788-01A	PRAD	8.62
TCGA-HC-A6AP-01A	PRAD	7.51
TCGA-KK-A6E6-01A	PRAD	8.53
TCGA-EJ-5514-01A	PRAD	9.53
TCGA-KK-A7AP-01A	PRAD	5.78
TCGA-G9-6370-01A	PRAD	8.21
TCGA-FC-A6HD-01A	PRAD	7.1
TCGA-FC-A5OB-01A	PRAD	9.42
TCGA-V1-A8WL-01A	PRAD	7.84
TCGA-EJ-5494-01A	PRAD	9.12
TCGA-HC-8261-01A	PRAD	8.87
TCGA-HC-7817-01B	PRAD	8.52
TCGA-G9-6351-01A	PRAD	8.64
TCGA-HC-A48F-01A	PRAD	8.73
TCGA-YL-A8SJ-01B	PRAD	7.08
TCGA-CH-5794-01A	PRAD	8.19
TCGA-G9-6499-01A	PRAD	8.46
TCGA-YL-A8S9-01A	PRAD	6.56
TCGA-EJ-A6RC-01A	PRAD	7.74
TCGA-HC-7212-01A	PRAD	9.15
TCGA-HC-A6AL-01A	PRAD	8.2
TCGA-CH-5762-01A	PRAD	8.29
TCGA-KC-A4BL-01A	PRAD	7.44
TCGA-X4-A8KS-01A	PRAD	6.62
TCGA-G9-6336-01A	PRAD	8.07
TCGA-EJ-5499-01A	PRAD	7.88
TCGA-HC-7080-01A	PRAD	8.01
TCGA-CH-5748-01A	PRAD	9.02
TCGA-KK-A8IA-01A	PRAD	8.43
TCGA-V1-A9OX-01A	PRAD	7.65
TCGA-XK-AAIR-01A	PRAD	8.33
TCGA-G5-6572-01A	READ	7.7
TCGA-AG-A023-01A	READ	5.99
TCGA-DC-5337-01A	READ	7.96
TCGA-AF-2693-01A	READ	6.84
TCGA-AG-3898-01A	READ	7.02
TCGA-AG-3894-01A	READ	7.34
TCGA-EI-6885-01A	READ	7.77

TCGA-EI-6510-01A	READ	6.67
TCGA-F5-6810-01A	READ	6.9
TCGA-AG-3575-01A	READ	6.21
TCGA-AF-6672-01A	READ	6.74
TCGA-AG-3896-01A	READ	7.32
TCGA-AG-A011-01A	READ	6.95
TCGA-DC-6154-01A	READ	7.27
TCGA-AG-3581-01A	READ	6.48
TCGA-DT-5265-01A	READ	6.43
TCGA-AG-3578-01A	READ	7.33
TCGA-AG-3583-01A	READ	6.56
TCGA-AF-5654-01A	READ	7.68
TCGA-AG-A00C-01A	READ	6.3
TCGA-DC-6155-01A	READ	4.9
TCGA-AG-A036-01A	READ	7.39
TCGA-AF-A56K-01A	READ	6.26
TCGA-AG-3602-01A	READ	7.03
TCGA-AH-6547-01A	READ	6.27
TCGA-AH-6644-01A	READ	7.15
TCGA-EI-6917-01A	READ	6.98
TCGA-AG-3574-01A	READ	7.57
TCGA-AG-A032-01A	READ	6.08
TCGA-CL-5918-01A	READ	6.94
TCGA-AF-2687-01A	READ	6.71
TCGA-AG-3611-01A	READ	6.77
TCGA-EI-6507-01A	READ	6.58
TCGA-EI-6508-01A	READ	7.48
TCGA-AF-A56L-01A	READ	7.28
TCGA-F5-6811-01A	READ	7.02
TCGA-EI-6511-01A	READ	6.19
TCGA-EI-6883-01A	READ	6.73
TCGA-EF-5831-01A	READ	7.24
TCGA-DY-A1DE-01A	READ	7.18
TCGA-F5-6464-01A	READ	6.64
TCGA-F5-6863-01A	READ	7.04
TCGA-AG-3887-01A	READ	6.68
TCGA-CL-4957-01A	READ	7.69
TCGA-CI-6623-01B	READ	7.12
TCGA-BM-6198-01A	READ	6.68
TCGA-EI-6506-01A	READ	6.94
TCGA-AG-3594-01A	READ	7.16
TCGA-AG-A002-01A	READ	7.34
TCGA-CL-5917-01A	READ	8
TCGA-AG-A014-01A	READ	6.33
TCGA-AG-3598-01A	READ	6.82
TCGA-AF-A56N-01A	READ	7.32
TCGA-G5-6641-01A	READ	7.15
TCGA-DY-A1DD-01A	READ	5.45
TCGA-EF-5830-01A	READ	7.49
TCGA-AG-3609-01A	READ	6.31
TCGA-CI-6624-01C	READ	7.38
TCGA-AG-A016-01A	READ	6.95
TCGA-AG-A01L-01A	READ	6.78
TCGA-AG-3732-01A	READ	7.7
TCGA-AG-A00H-01A	READ	6.63
TCGA-AG-4007-01A	READ	6.8
TCGA-AG-3591-01A	READ	7.51
TCGA-AG-A02X-01A	READ	7.58

TCGA-DY-A1DF-01A	READ	5.28
TCGA-EI-6884-01A	READ	7.34
TCGA-AG-3902-01A	READ	6.08
TCGA-AG-A015-01A	READ	7.36
TCGA-AF-4110-01A	READ	6.48
TCGA-AF-2691-01A	READ	6.9
TCGA-AG-3731-01A	READ	6.74
TCGA-DY-A1DG-01A	READ	6.47
TCGA-CI-6619-01B	READ	6.88
TCGA-DC-6157-01A	READ	7.63
TCGA-DC-4745-01A	READ	6.8
TCGA-AG-3584-01A	READ	6.72
TCGA-AG-4022-01A	READ	6.69
TCGA-AG-3893-01A	READ	6.23
TCGA-AG-3881-01A	READ	5.68
TCGA-DY-A1H8-01A	READ	5.38
TCGA-AG-3883-01A	READ	5.57
TCGA-AG-3882-01A	READ	5.31
TCGA-AG-3601-01A	READ	7.17
TCGA-F5-6813-01A	READ	7.08
TCGA-AG-3608-01A	READ	6.39
TCGA-F5-6702-01A	READ	6.93
TCGA-AH-6643-01A	READ	7.37
TCGA-CI-6622-01A	READ	7.26
TCGA-G5-6233-01A	READ	7.05
TCGA-AG-3742-01A	READ	6.95
TCGA-AG-3725-01A	READ	6.95
TCGA-AG-A00Y-01A	READ	6.37
TCGA-AG-3878-01A	READ	5.83
TCGA-AG-3582-01A	READ	6.7
TCGA-AG-3892-01A	READ	7.03
TCGA-AG-3587-01A	READ	6.5
TCGA-AF-3400-01A	READ	6.53
TCGA-AG-3890-01A	READ	6.27
TCGA-EI-6882-01A	READ	8.12
TCGA-F5-6812-01A	READ	5.99
TCGA-DC-5869-01A	READ	7.54
TCGA-AG-3885-01A	READ	5.96
TCGA-AF-6655-01A	READ	6.68
TCGA-AG-3727-01A	READ	6.16
TCGA-AG-A008-01A	READ	7.05
TCGA-F5-6465-01A	READ	7.13
TCGA-AG-4001-01A	READ	6.61
TCGA-F5-6861-01A	READ	8.03
TCGA-AG-4021-01A	READ	8.52
TCGA-AG-3580-01A	READ	7.18
TCGA-AG-3999-01A	READ	6.97
TCGA-AF-2692-01A	READ	7.26
TCGA-AG-4008-01A	READ	6.66
TCGA-AH-6544-01A	READ	7.71
TCGA-AG-A025-01A	READ	6.48
TCGA-AG-3728-01A	READ	5.35
TCGA-DY-A0XA-01A	READ	7.17
TCGA-F5-6571-01A	READ	6.02
TCGA-AG-A01W-01A	READ	7.69
TCGA-EI-6513-01A	READ	7.08
TCGA-AG-A01J-01A	READ	6.46
TCGA-AG-A01Y-01A	READ	6.84

TCGA-AG-3593-01A	READ	6.93
TCGA-AH-6549-01A	READ	6.72
TCGA-AF-3911-01A	READ	7.22
TCGA-AG-4015-01A	READ	6.51
TCGA-DC-6681-01A	READ	7
TCGA-F5-6864-01A	READ	7.81
TCGA-AF-2690-01A	READ	6.73
TCGA-EI-6509-01A	READ	6.9
TCGA-AG-A01N-01A	READ	6.82
TCGA-AG-3600-01A	READ	7.96
TCGA-AG-3599-01A	READ	7.34
TCGA-AG-A020-01A	READ	7.07
TCGA-EI-7002-01A	READ	7.22
TCGA-CI-6621-01A	READ	7.1
TCGA-DC-6160-01A	READ	7.44
TCGA-AG-3726-01A	READ	6.23
TCGA-G5-6572-02A	READ	6.91
TCGA-EI-6512-01A	READ	7.02
TCGA-AF-3913-01A	READ	7.02
TCGA-DC-6682-01A	READ	6.99
TCGA-AH-6897-01A	READ	8.12
TCGA-AG-A026-01A	READ	6.58
TCGA-AF-6136-01A	READ	7.78
TCGA-EI-6514-01A	READ	6.75
TCGA-AG-3901-01A	READ	7.43
TCGA-DC-6683-01A	READ	7.39
TCGA-DC-6158-01A	READ	7.67
TCGA-AG-A02G-01A	READ	6.65
TCGA-DC-6156-01A	READ	7.4
TCGA-AG-3592-01A	READ	6.91
TCGA-AG-3612-01A	READ	6.28
TCGA-AH-6903-01A	READ	7.43
TCGA-DY-A1DC-01A	READ	6.77
TCGA-AG-4005-01A	READ	6.55
TCGA-EI-6881-01A	READ	6.87
TCGA-AG-A02N-01A	READ	7.17
TCGA-AG-3605-01A	READ	6.51
TCGA-AG-3909-01A	READ	6.58
TCGA-AG-3586-01A	READ	7.27
TCGA-F5-6814-01A	READ	8.21
TCGA-EI-7004-01A	READ	6.51
TCGA-DC-4749-01A	READ	7.94
TCGA-G5-6235-01A	READ	6.76
TCGA-CI-6620-01A	READ	7.09
TCGA-HS-A5N7-01A	SARC	4.7
TCGA-DX-A3LS-01A	SARC	6.06
TCGA-IE-A3OV-01A	SARC	4.84
TCGA-FX-A2QS-01A	SARC	7.2
TCGA-HS-A5N9-01A	SARC	5.29
TCGA-FX-A8OO-01A	SARC	5
TCGA-SI-AA8B-01A	SARC	5.33
TCGA-DX-AB2W-01A	SARC	5.97
TCGA-K1-A6RT-01A	SARC	4.72
TCGA-MB-A5YA-01A	SARC	5.31
TCGA-UE-A6QU-01A	SARC	5.74
TCGA-DX-A3U9-01A	SARC	3.28
TCGA-DX-AB37-01A	SARC	5.96
TCGA-DX-A6YR-01A	SARC	5.73

TCGA-HS-A5NA-01A	SARC	4.56
TCGA-QQ-A8VH-01A	SARC	5.75
TCGA-IS-A3K7-01A	SARC	4.86
TCGA-MJ-A68H-01A	SARC	4.52
TCGA-DX-A8BK-01A	SARC	5.25
TCGA-DX-AATS-01A	SARC	6.56
TCGA-IF-A3RQ-01A	SARC	5.92
TCGA-IE-A6BZ-01A	SARC	6.14
TCGA-DX-A1L0-01A	SARC	5.61
TCGA-X6-A7WA-01A	SARC	5.38
TCGA-DX-AB3B-01A	SARC	5.78
TCGA-DX-A8BJ-01A	SARC	5.5
TCGA-X6-A8C2-01A	SARC	5.45
TCGA-WK-A8XQ-01A	SARC	5.96
TCGA-QQ-A8VD-01A	SARC	5.46
TCGA-K1-A42X-01A	SARC	5.3
TCGA-HS-A5N8-01A	SARC	6.01
TCGA-DX-A1L2-01A	SARC	6.42
TCGA-DX-A3UB-01A	SARC	3.61
TCGA-MO-A47P-01A	SARC	5.96
TCGA-PC-A5DO-01A	SARC	5.47
TCGA-DX-AB2F-01A	SARC	6.06
TCGA-FX-A3RE-01A	SARC	7.34
TCGA-DX-A8BG-01A	SARC	6.32
TCGA-DX-A23Y-01A	SARC	6.22
TCGA-QC-A7B5-01A	SARC	5.07
TCGA-3B-A9HZ-01A	SARC	5.84
TCGA-X6-A8C5-01A	SARC	5.23
TCGA-X6-A8C3-01A	SARC	6.24
TCGA-3B-A9HI-01A	SARC	5.48
TCGA-DX-A8BS-01A	SARC	5.84
TCGA-DX-A3LY-01B	SARC	7.05
TCGA-DX-A6BB-01A	SARC	3.72
TCGA-VT-AB3D-01A	SARC	5.57
TCGA-DX-A7EN-01A	SARC	3.81
TCGA-VT-A80J-02A	SARC	7.12
TCGA-DX-AB2P-01A	SARC	5.11
TCGA-3B-A9HV-01A	SARC	5.05
TCGA-DX-A8BR-01A	SARC	7.16
TCGA-QQ-A8VB-01A	SARC	6.31
TCGA-DX-A1KZ-01A	SARC	6.28
TCGA-DX-A2J0-01A	SARC	4.94
TCGA-IS-A3KA-01A	SARC	3.89
TCGA-K1-A6RU-01A	SARC	6.11
TCGA-3B-A9HS-01A	SARC	5.24
TCGA-LI-A67I-01A	SARC	5.61
TCGA-WK-A8XY-01A	SARC	4.92
TCGA-IW-A3M6-01A	SARC	5.43
TCGA-WK-A8XX-01A	SARC	5.93
TCGA-DX-A3UF-01A	SARC	5.07
TCGA-DX-A8BZ-01A	SARC	4.84
TCGA-DX-AB2J-01A	SARC	5.63
TCGA-WP-A9GB-01A	SARC	4.42
TCGA-IE-A4EJ-01A	SARC	6.06
TCGA-Z4-A9VC-01A	SARC	7.48
TCGA-DX-A6YQ-01A	SARC	6.27
TCGA-IE-A4EK-01A	SARC	5.06
TCGA-DX-AB2H-01A	SARC	4.64

TCGA-HB-A3L4-01A	SARC	5.08
TCGA-X2-A95T-01A	SARC	5.27
TCGA-K1-A42W-01A	SARC	4.83
TCGA-DX-A48J-01A	SARC	4.75
TCGA-3B-A9HQ-01A	SARC	4.62
TCGA-X6-A7WB-01A	SARC	5.07
TCGA-QQ-A5VA-01A	SARC	5.12
TCGA-DX-AB36-01A	SARC	5.46
TCGA-DX-A7EF-01A	SARC	5.9
TCGA-VT-A80G-01A	SARC	4.99
TCGA-N1-A6IA-01A	SARC	5.45
TCGA-K1-A42X-02A	SARC	5.14
TCGA-X6-A8C6-01A	SARC	5.57
TCGA-DX-A6B8-01A	SARC	5.43
TCGA-DX-A7ES-01A	SARC	5.46
TCGA-DX-AB2X-01A	SARC	6.56
TCGA-QQ-A8VG-01A	SARC	5.65
TCGA-DX-A48K-01A	SARC	5.57
TCGA-IE-A4EH-01A	SARC	4.51
TCGA-DX-A6BH-01A	SARC	4.51
TCGA-DX-AB2G-01A	SARC	5.89
TCGA-SI-A71P-01A	SARC	5.48
TCGA-DX-A23Z-01A	SARC	5.55
TCGA-DX-A240-01A	SARC	5.23
TCGA-IF-A4AJ-01A	SARC	4.86
TCGA-PT-A8TR-01A	SARC	5.49
TCGA-Z4-AAPG-01A	SARC	6.06
TCGA-DX-A3U6-01A	SARC	6.1
TCGA-DX-AB30-01A	SARC	5.56
TCGA-DX-A2IZ-01A	SARC	4.6
TCGA-DX-A1KX-01A	SARC	6.19
TCGA-DX-A3M2-01A	SARC	5.97
TCGA-MO-A47R-01A	SARC	5.94
TCGA-SI-AA8C-01A	SARC	5.35
TCGA-LI-A9QH-01A	SARC	5.33
TCGA-MB-A8JL-01A	SARC	6.23
TCGA-FX-A3NJ-01A	SARC	5.34
TCGA-DX-AB3C-01A	SARC	4.68
TCGA-DX-A6BG-01A	SARC	5.82
TCGA-UE-A6QT-01A	SARC	5.84
TCGA-DX-A6YV-01A	SARC	5.75
TCGA-MB-A8JK-01A	SARC	4.98
TCGA-DX-A48N-01A	SARC	5.49
TCGA-DX-A3UC-01A	SARC	4.5
TCGA-DX-A1L4-01A	SARC	6.79
TCGA-DX-A23U-01A	SARC	5.55
TCGA-X9-A973-01A	SARC	5.81
TCGA-PC-A5DL-01A	SARC	4.69
TCGA-DX-A3U5-01A	SARC	7.43
TCGA-DX-A8BP-01A	SARC	6.23
TCGA-DX-A1L3-01A	SARC	6.66
TCGA-DX-AB2Z-01A	SARC	6.29
TCGA-DX-A3UD-01A	SARC	3.73
TCGA-DX-A48R-01A	SARC	3.65
TCGA-DX-A7EO-01A	SARC	5.14
TCGA-QQ-A5V9-01A	SARC	5.31
TCGA-SI-A71O-06A	SARC	5.48
TCGA-QQ-A5V2-01A	SARC	4.45

TCGA-DX-A6Z0-01A	SARC	6.14
TCGA-KF-A41W-01A	SARC	5.66
TCGA-KD-A5QT-01A	SARC	5.96
TCGA-3B-A9HU-01A	SARC	5.39
TCGA-DX-AB3A-01A	SARC	5.47
TCGA-DX-A6B9-01A	SARC	4.75
TCGA-3B-A9I3-01A	SARC	6.01
TCGA-PC-A5DK-01A	SARC	5.11
TCGA-DX-A1KW-01A	SARC	5.82
TCGA-JV-A5VF-01A	SARC	4.82
TCGA-X6-A8C7-01A	SARC	5.53
TCGA-Z4-AAPF-01A	SARC	4.71
TCGA-PC-A5DN-01A	SARC	5.04
TCGA-HB-A5W3-01A	SARC	5.21
TCGA-X9-A971-01A	SARC	4.26
TCGA-IE-A4EI-01A	SARC	4.78
TCGA-IS-A3K8-01A	SARC	4.77
TCGA-X6-A8C4-01A	SARC	6.38
TCGA-DX-AB2Q-01A	SARC	5.82
TCGA-Z4-A8JB-01A	SARC	5.25
TCGA-MB-A5Y9-01A	SARC	5.86
TCGA-DX-A23T-01A	SARC	6.22
TCGA-3B-A9I0-01A	SARC	4.39
TCGA-3B-A9HJ-01A	SARC	4.79
TCGA-3B-A9HL-01A	SARC	5.87
TCGA-JV-A5VE-01A	SARC	6.23
TCGA-DX-A23R-01A	SARC	6.23
TCGA-KD-A5QS-01A	SARC	5.78
TCGA-K1-A3PN-02A	SARC	3.55
TCGA-WK-A8XO-01A	SARC	5.87
TCGA-IS-A3K6-01A	SARC	4.24
TCGA-DX-A7EM-01A	SARC	4.34
TCGA-DX-A6YX-01A	SARC	6.88
TCGA-DX-A8BL-01A	SARC	4.63
TCGA-3B-A9HR-01A	SARC	4.18
TCGA-DX-A6BF-01A	SARC	5.82
TCGA-FX-A3NK-01A	SARC	5.07
TCGA-3B-A9I1-01A	SARC	5.17
TCGA-PC-A5DM-01A	SARC	5.05
TCGA-QC-AA9N-01A	SARC	4.62
TCGA-DX-A7ER-01A	SARC	4.07
TCGA-SI-A71O-01A	SARC	5.27
TCGA-RN-A68Q-01A	SARC	5.5
TCGA-WK-A8Y0-01A	SARC	5.8
TCGA-SG-A849-01A	SARC	5.97
TCGA-IW-A3M5-01A	SARC	5.22
TCGA-JV-A75J-01A	SARC	4.56
TCGA-WK-A8XS-01A	SARC	4.91
TCGA-DX-A8BQ-01A	SARC	6.23
TCGA-DX-A48L-01A	SARC	4.66
TCGA-DX-AB32-01A	SARC	5.68
TCGA-DX-AB35-01A	SARC	5.22
TCGA-QQ-A5VD-01A	SARC	4.86
TCGA-DX-A3U7-01A	SARC	4.17
TCGA-3B-A9HO-01A	SARC	5.8
TCGA-DX-AB2O-01A	SARC	4.86
TCGA-DX-A7EL-01A	SARC	4.8
TCGA-DX-A2J4-01A	SARC	5.54

TCGA-DX-A6YT-01A	SARC	5.96
TCGA-DX-A6BE-01A	SARC	5.25
TCGA-DX-A3U8-01A	SARC	6.75
TCGA-DX-AB2E-01A	SARC	6.36
TCGA-DX-A3UA-01A	SARC	5.71
TCGA-DX-A7EU-01A	SARC	5.97
TCGA-X6-A7WD-01A	SARC	5.05
TCGA-DX-A48P-01A	SARC	5.09
TCGA-DX-A1KU-01A	SARC	6.28
TCGA-SI-A71Q-01A	SARC	5.33
TCGA-DX-A3M1-01A	SARC	5.19
TCGA-RN-AAAQ-01A	SARC	5.64
TCGA-DX-A6YS-01A	SARC	5.32
TCGA-X6-A7W8-01A	SARC	4.62
TCGA-DX-A48U-01A	SARC	4.8
TCGA-3R-A8YX-01A	SARC	5.91
TCGA-K1-A3PN-01A	SARC	3.73
TCGA-DX-A48O-01A	SARC	3.92
TCGA-DX-A3LU-01A	SARC	5.48
TCGA-DX-A3LT-01A	SARC	5.85
TCGA-DX-AB2S-01A	SARC	5.52
TCGA-DX-A8BT-01A	SARC	6.55
TCGA-DX-A1KY-01A	SARC	5.93
TCGA-PC-A5DP-01A	SARC	3.39
TCGA-3B-A9HP-01A	SARC	5.21
TCGA-K1-A6RV-01A	SARC	5.21
TCGA-IF-A4AK-01A	SARC	4.65
TCGA-DX-A8BM-01A	SARC	5.84
TCGA-3B-A9HX-01A	SARC	5.9
TCGA-HB-A43Z-01A	SARC	6.03
TCGA-HB-A2OT-01A	SARC	6.59
TCGA-QC-A6FX-01A	SARC	5.9
TCGA-DX-AB2V-01A	SARC	6.03
TCGA-WK-A8XZ-01A	SARC	4.54
TCGA-DX-A6B7-01A	SARC	3.38
TCGA-FX-A48G-01A	SARC	4.49
TCGA-DX-A8BO-01A	SARC	7.1
TCGA-IW-A3M4-01A	SARC	5.81
TCGA-MJ-A850-01A	SARC	3.95
TCGA-K1-A3PO-01A	SARC	5.98
TCGA-DX-AB2T-01A	SARC	6.09
TCGA-DX-A3LW-01A	SARC	5.87
TCGA-MJ-A68J-01A	SARC	5.4
TCGA-DX-A7EQ-01A	SARC	3.44
TCGA-FX-A3TO-01A	SARC	5.4
TCGA-X6-A7WC-01A	SARC	5.65
TCGA-QQ-A5VB-01A	SARC	5.74
TCGA-DX-A23V-01A	SARC	6.74
TCGA-DX-A2J1-01A	SARC	6.16
TCGA-SG-A6Z7-01A	SARC	4.08
TCGA-DX-A8BV-01A	SARC	6.25
TCGA-DX-A6YU-01A	SARC	6.78
TCGA-SG-A6Z4-01A	SARC	5.28
TCGA-HB-A3YV-01A	SARC	4.66
TCGA-DX-A6Z2-01A	SARC	5.15
TCGA-DX-AB2L-01A	SARC	5.44
TCGA-FX-A76Y-01A	SARC	4.75
TCGA-QQ-A8VF-01A	SARC	6

TCGA-WK-A8XT-01A	SARC	5.51
TCGA-DX-A7ET-01A	SARC	4.9
TCGA-MB-A5Y8-01A	SARC	6.03
TCGA-DX-A6YZ-01A	SARC	6.23
TCGA-DX-A8BX-01A	SARC	6.51
TCGA-3B-A9HY-01A	SARC	4.59
TCGA-DX-A7EI-01A	SARC	4.66
TCGA-KD-A5QU-01A	SARC	5.04
TCGA-DX-A6BA-01A	SARC	4.93
TCGA-QQ-A5VC-01A	SARC	4.94
TCGA-DX-A8BN-01A	SARC	5.53
TCGA-VT-A80J-01A	SARC	6.74
TCGA-DX-A8BU-01A	SARC	4.43
TCGA-DX-A3UE-01A	SARC	4.5
TCGA-DX-A8BH-01A	SARC	6.17
TCGA-DX-A1L1-01A	SARC	6.69
TCGA-3B-A9HT-01A	SARC	6.63
TCGA-LH-A9QB-06A	SKCM	5.7
TCGA-GN-A268-06A	SKCM	6.35
TCGA-FR-A69P-06A	SKCM	5.57
TCGA-EE-A2A1-06A	SKCM	6.35
TCGA-YD-A9TA-06A	SKCM	6.08
TCGA-D3-A1QB-06A	SKCM	6.3
TCGA-EE-A2A6-06A	SKCM	6.23
TCGA-FS-A1YY-06A	SKCM	4.83
TCGA-D3-A5GN-06A	SKCM	6.11
TCGA-RP-A690-06A	SKCM	5.02
TCGA-EE-A2ME-06A	SKCM	5.76
TCGA-WE-AAA3-06A	SKCM	5.49
TCGA-QB-A6FS-06A	SKCM	5.3
TCGA-EE-A17X-06A	SKCM	5.93
TCGA-GN-A4U4-06A	SKCM	5.24
TCGA-XV-AAZW-01A	SKCM	5.65
TCGA-D3-A8GK-06A	SKCM	6.68
TCGA-WE-A8ZR-06A	SKCM	6.14
TCGA-GN-A8LL-06A	SKCM	6.11
TCGA-EB-A551-01A	SKCM	6.26
TCGA-GN-A264-06A	SKCM	5.35
TCGA-FS-A4FB-06A	SKCM	5.15
TCGA-WE-A8ZQ-06A	SKCM	5.23
TCGA-EE-A2GJ-06A	SKCM	6.27
TCGA-EE-A3AE-06A	SKCM	5.55
TCGA-EE-A2GL-06A	SKCM	5.64
TCGA-FS-A1ZH-06A	SKCM	5.81
TCGA-EE-A185-06A	SKCM	7.13
TCGA-FS-A1ZM-06A	SKCM	5.82
TCGA-EB-A5SH-06A	SKCM	4.37
TCGA-EB-A44N-01A	SKCM	5.41
TCGA-FS-A1ZC-06A	SKCM	4.98
TCGA-EE-A3AB-06A	SKCM	5.86
TCGA-BF-A1PX-01A	SKCM	5.52
TCGA-D3-A8GL-06A	SKCM	7.34
TCGA-D3-A8GC-06A	SKCM	7.44
TCGA-D3-A2JO-06A	SKCM	6.96
TCGA-EB-A57M-01A	SKCM	5.14
TCGA-EE-A2A2-06A	SKCM	6.21
TCGA-D3-A8GS-06A	SKCM	5.36
TCGA-EE-A2M6-06A	SKCM	6.17

TCGA-Z2-A8RT-06A	SKCM	5.73
TCGA-WE-A8ZT-06A	SKCM	5.79
TCGA-EB-A82C-01A	SKCM	5.97
TCGA-EE-A183-06A	SKCM	6.1
TCGA-EB-A44R-06A	SKCM	5.98
TCGA-EE-A2GD-06A	SKCM	6.72
TCGA-EE-A29S-06A	SKCM	6.54
TCGA-D3-A5GU-06A	SKCM	4.74
TCGA-EB-A44P-01A	SKCM	5.9
TCGA-ER-A2NH-06A	SKCM	6.1
TCGA-EB-A3XF-01A	SKCM	6.77
TCGA-D3-A3CE-06A	SKCM	5.77
TCGA-EE-A182-06A	SKCM	5.14
TCGA-D9-A3Z3-06A	SKCM	5.45
TCGA-Z2-AA3S-06A	SKCM	5.37
TCGA-FS-A4FD-06A	SKCM	4.97
TCGA-D3-A8GD-06A	SKCM	5.61
TCGA-FR-A3R1-01A	SKCM	5.79
TCGA-XV-A9W5-01A	SKCM	5.54
TCGA-EE-A2M7-06A	SKCM	6.76
TCGA-DA-A1I4-06A	SKCM	6.63
TCGA-EE-A2MJ-06A	SKCM	5.72
TCGA-ER-A42K-06A	SKCM	6.63
TCGA-DA-A1I1-06A	SKCM	5.83
TCGA-EE-A2MT-06A	SKCM	6.33
TCGA-ER-A19O-06A	SKCM	5.11
TCGA-EE-A29N-06A	SKCM	6.5
TCGA-YD-A9TB-06A	SKCM	5.36
TCGA-W3-AA1Q-06A	SKCM	4.3
TCGA-YD-A89C-06A	SKCM	5.69
TCGA-D3-A1Q1-06A	SKCM	7.23
TCGA-BF-A5EO-01A	SKCM	4.26
TCGA-D3-A1Q8-06A	SKCM	6.03
TCGA-EE-A29E-06A	SKCM	5.95
TCGA-D3-A3MO-06A	SKCM	6.56
TCGA-EE-A29G-06A	SKCM	6.68
TCGA-DA-A1IB-06A	SKCM	5.1
TCGA-BF-AAP2-01A	SKCM	4.95
TCGA-FS-A1ZU-06A	SKCM	6.3
TCGA-EE-A2GO-06A	SKCM	5.23
TCGA-FS-A1ZR-06A	SKCM	5.03
TCGA-WE-AAA4-06A	SKCM	5.64
TCGA-EE-A20I-06A	SKCM	6.07
TCGA-GN-A26D-06A	SKCM	5.24
TCGA-EB-A5VU-01A	SKCM	6.31
TCGA-D3-A51N-06A	SKCM	4.89
TCGA-D3-A3CB-06A	SKCM	5.88
TCGA-ER-A19T-06A	SKCM	5.39
TCGA-EE-A3J3-06A	SKCM	5.26
TCGA-EE-A2GI-06A	SKCM	5.55
TCGA-FS-A1ZQ-06A	SKCM	6.12
TCGA-D9-A4Z6-06A	SKCM	6.95
TCGA-ER-A3ES-06A	SKCM	4.45
TCGA-ER-A2NC-06A	SKCM	6.11
TCGA-FS-A1ZY-06A	SKCM	4.97
TCGA-FS-A4FC-06A	SKCM	6.12
TCGA-EB-A550-01A	SKCM	5.53
TCGA-D3-A3BZ-06A	SKCM	6.46

TCGA-ER-A196-01A	SKCM	4.2
TCGA-D3-A3C6-06A	SKCM	5.14
TCGA-GF-A769-01A	SKCM	7.64
TCGA-EE-A3AC-06A	SKCM	6.05
TCGA-D3-A2JF-06A	SKCM	4.26
TCGA-DA-A95X-06A	SKCM	6.93
TCGA-EE-A29H-06A	SKCM	5.63
TCGA-EE-A29D-06A	SKCM	6.66
TCGA-EE-A2GK-06A	SKCM	4.95
TCGA-EE-A29P-06A	SKCM	5.76
TCGA-EB-A5SE-01A	SKCM	6.62
TCGA-D9-A4Z2-01A	SKCM	5.16
TCGA-D3-A8GR-06A	SKCM	4.47
TCGA-ER-A19P-06A	SKCM	5.13
TCGA-D9-A148-06A	SKCM	5.67
TCGA-WE-A8ZM-06A	SKCM	4.63
TCGA-GF-A3OT-06A	SKCM	6.24
TCGA-ER-A19K-01A	SKCM	6.65
TCGA-GN-A26C-01A	SKCM	6.58
TCGA-ER-A19S-06A	SKCM	5.59
TCGA-D9-A1JX-06A	SKCM	5.8
TCGA-FS-A1ZG-06A	SKCM	6.15
TCGA-FR-A729-06A	SKCM	5.57
TCGA-D3-A3MV-06A	SKCM	5.53
TCGA-EB-A5UL-06A	SKCM	6.29
TCGA-EE-A20B-06A	SKCM	5.85
TCGA-FS-A1ZZ-06A	SKCM	7.25
TCGA-D9-A6EA-06A	SKCM	4.47
TCGA-D3-A8GO-06A	SKCM	3.52
TCGA-ER-A19C-06A	SKCM	5.08
TCGA-EB-A44Q-06A	SKCM	6.55
TCGA-EB-A3XE-01A	SKCM	4.28
TCGA-D3-A8GE-06A	SKCM	5.05
TCGA-EE-A29R-06A	SKCM	6.83
TCGA-FR-A8YC-06A	SKCM	4.76
TCGA-DA-A1I5-06A	SKCM	5.57
TCGA-D3-A1Q4-06A	SKCM	5.35
TCGA-EE-A181-06A	SKCM	5.99
TCGA-BF-A3DN-01A	SKCM	6.01
TCGA-D3-A2J8-06A	SKCM	5.85
TCGA-ER-A19W-06A	SKCM	7.95
TCGA-GF-A4EO-06A	SKCM	5.31
TCGA-W3-A824-06A	SKCM	5.34
TCGA-FR-A2OS-01A	SKCM	5.25
TCGA-EB-A41A-01A	SKCM	5.13
TCGA-EE-A29C-06A	SKCM	5.83
TCGA-EE-A2MD-06A	SKCM	6.61
TCGA-ER-A2NF-01A	SKCM	5.38
TCGA-ER-A2NF-06A	SKCM	6.06
TCGA-EB-A3Y7-01A	SKCM	4.88
TCGA-GN-A4U8-06A	SKCM	5.7
TCGA-EB-A430-01A	SKCM	5.71
TCGA-EB-A41B-01A	SKCM	5.82
TCGA-EE-A2MM-06A	SKCM	5.52
TCGA-D3-A2JC-06A	SKCM	6.63
TCGA-W3-AA1O-06A	SKCM	7.02
TCGA-FS-A1ZF-06A	SKCM	4.92
TCGA-D3-A51K-06A	SKCM	3.57

TCGA-ER-A19H-06A	SKCM	6.24
TCGA-ER-A199-06A	SKCM	5.79
TCGA-DA-A95V-06A	SKCM	5.56
TCGA-ER-A3EV-06A	SKCM	6.94
TCGA-EB-A4P0-01A	SKCM	4.74
TCGA-GN-A262-06A	SKCM	4.72
TCGA-FR-A7UA-06A	SKCM	5.77
TCGA-EB-A24D-01A	SKCM	4.34
TCGA-GN-A9SD-06A	SKCM	5.36
TCGA-EE-A17Z-06A	SKCM	6.59
TCGA-ER-A198-06A	SKCM	7.19
TCGA-ER-A193-06A	SKCM	5.8
TCGA-EB-A6QZ-01A	SKCM	4.99
TCGA-XV-A9W2-01A	SKCM	4.91
TCGA-DA-A1IC-06A	SKCM	6.46
TCGA-EE-A29B-06A	SKCM	6.05
TCGA-ER-A19G-06A	SKCM	6.05
TCGA-D3-A8GP-06A	SKCM	4.64
TCGA-EB-A553-01A	SKCM	4.37
TCGA-XV-AAZV-01A	SKCM	5.25
TCGA-GN-A8LN-01A	SKCM	4.95
TCGA-BF-AAP6-01A	SKCM	6.36
TCGA-FR-A8YE-06A	SKCM	4.85
TCGA-XV-AAZY-01A	SKCM	4.37
TCGA-D3-A2JD-06A	SKCM	5.93
TCGA-EE-A2A0-06A	SKCM	6.18
TCGA-EB-A3Y6-01A	SKCM	5.56
TCGA-EB-A6QY-01A	SKCM	6.04
TCGA-FS-A4F5-06A	SKCM	5.38
TCGA-EE-A2GS-06A	SKCM	5.19
TCGA-EE-A180-06A	SKCM	6.05
TCGA-ER-A3PL-06A	SKCM	5.5
TCGA-EE-A29T-06A	SKCM	6.5
TCGA-EB-A431-01A	SKCM	6.46
TCGA-EE-A2GT-06A	SKCM	5.16
TCGA-W3-AA1V-06B	SKCM	6.18
TCGA-DA-A3F2-06A	SKCM	4.31
TCGA-GN-A267-06A	SKCM	3.51
TCGA-ER-A19N-06A	SKCM	5.81
TCGA-WE-A8ZO-06A	SKCM	5.82
TCGA-WE-A8K1-06A	SKCM	5.83
TCGA-ER-A19T-01A	SKCM	6.2
TCGA-EE-A3JD-06A	SKCM	6.01
TCGA-BF-A5ER-01A	SKCM	4.81
TCGA-3N-A9WD-06A	SKCM	4.84
TCGA-W3-A828-06A	SKCM	5.94
TCGA-EE-A20C-06A	SKCM	5.52
TCGA-EE-A2MH-06A	SKCM	4.74
TCGA-ER-A2NB-01A	SKCM	3.71
TCGA-EB-A3XB-01A	SKCM	5.96
TCGA-D3-A5GR-06A	SKCM	5.86
TCGA-D3-A8GI-06A	SKCM	5.01
TCGA-WE-AAA0-06A	SKCM	5.61
TCGA-RP-A694-06A	SKCM	6.62
TCGA-EE-A29W-06A	SKCM	5.62
TCGA-EB-A3HV-01A	SKCM	4.69
TCGA-D9-A4Z5-01A	SKCM	5.32
TCGA-EE-A2MQ-06A	SKCM	6.03

TCGA-EE-A2M5-06A	SKCM	7.04
TCGA-EE-A29L-06A	SKCM	6.27
TCGA-DA-A3F8-06A	SKCM	6.11
TCGA-D3-A5GS-06A	SKCM	5.3
TCGA-GN-A4U3-06A	SKCM	5.22
TCGA-WE-A8K6-06A	SKCM	4.45
TCGA-GN-A8LK-06A	SKCM	6.36
TCGA-FW-A5DX-01A	SKCM	4.81
TCGA-BF-AAP8-01A	SKCM	4.44
TCGA-DA-A960-01A	SKCM	3.5
TCGA-EB-A82B-01A	SKCM	5.28
TCGA-D3-A51T-06A	SKCM	4.87
TCGA-EB-A4XL-01A	SKCM	4.72
TCGA-IH-A3EA-01A	SKCM	4.61
TCGA-EE-A3AA-06A	SKCM	6.04
TCGA-FS-A1ZN-01A	SKCM	6.12
TCGA-FW-A5DY-06A	SKCM	4.87
TCGA-DA-A3F5-06A	SKCM	5.85
TCGA-EB-A5UN-06A	SKCM	6.12
TCGA-EE-A3J5-06A	SKCM	6.46
TCGA-BF-AAOU-01A	SKCM	4.72
TCGA-D3-A2JB-06A	SKCM	5.82
TCGA-WE-A8K4-01A	SKCM	7.01
TCGA-D3-A1Q7-06A	SKCM	6.66
TCGA-FS-A1Z7-06A	SKCM	5.97
TCGA-DA-A1I8-06A	SKCM	6.02
TCGA-D3-A5GO-06A	SKCM	4.76
TCGA-EE-A2GH-06A	SKCM	4.82
TCGA-EE-A2GR-06A	SKCM	5.49
TCGA-EE-A2A5-06A	SKCM	5.61
TCGA-EB-A299-01A	SKCM	5.06
TCGA-D3-A51E-06A	SKCM	6.01
TCGA-D9-A1JW-06A	SKCM	6.05
TCGA-BF-A5EP-01A	SKCM	5.91
TCGA-EE-A3AH-06A	SKCM	6.15
TCGA-D3-A5GL-06A	SKCM	6.73
TCGA-EE-A2MG-06A	SKCM	5.54
TCGA-ER-A42H-01A	SKCM	5.18
TCGA-D3-A2JP-06A	SKCM	5.36
TCGA-EE-A2GB-06A	SKCM	6.86
TCGA-D3-A2JK-06A	SKCM	4.59
TCGA-OD-A75X-06A	SKCM	6.67
TCGA-EB-A85J-01A	SKCM	5.87
TCGA-FR-A3YN-06A	SKCM	6.16
TCGA-D9-A1X3-06A	SKCM	5.88
TCGA-EE-A2GP-06A	SKCM	6.14
TCGA-EB-A5VV-06A	SKCM	4.56
TCGA-EE-A3J8-06A	SKCM	5.28
TCGA-GN-A4U7-06A	SKCM	6.33
TCGA-EE-A3AD-06A	SKCM	6.37
TCGA-EB-A5SG-06A	SKCM	5.45
TCGA-FS-A1YX-06A	SKCM	6.22
TCGA-DA-A95Y-06A	SKCM	6.15
TCGA-ER-A19A-06A	SKCM	6.53
TCGA-D9-A3Z1-06A	SKCM	7.13
TCGA-DA-A95W-06A	SKCM	5.31
TCGA-EE-A2MR-06A	SKCM	6.16
TCGA-D3-A8GM-06A	SKCM	5.35

TCGA-D3-A51G-06A	SKCM	5.93
TCGA-BF-A1PZ-01A	SKCM	6.34
TCGA-FR-A726-01A	SKCM	5.82
TCGA-D3-A2JG-06A	SKCM	5.16
TCGA-D3-A8GB-06A	SKCM	4.64
TCGA-D3-A8GJ-06A	SKCM	5.56
TCGA-EE-A2GE-06A	SKCM	6.15
TCGA-D3-A51H-06A	SKCM	4.98
TCGA-BF-A5EQ-01A	SKCM	4.87
TCGA-EB-A6R0-01A	SKCM	5.5
TCGA-EE-A29Q-06A	SKCM	6.78
TCGA-FS-A1ZB-06A	SKCM	5.94
TCGA-FS-A1Z0-06A	SKCM	5.72
TCGA-D3-A2J6-06A	SKCM	6.52
TCGA-EE-A2MP-06A	SKCM	5.66
TCGA-EB-A3XD-01A	SKCM	4.68
TCGA-WE-A8ZY-06A	SKCM	6.15
TCGA-BF-A3DJ-01A	SKCM	5.67
TCGA-EB-A4OY-01A	SKCM	3.89
TCGA-DA-A1HW-06A	SKCM	5.68
TCGA-Z2-AA3V-06A	SKCM	5.08
TCGA-GN-A26A-06A	SKCM	5.92
TCGA-W3-AA1R-06A	SKCM	6.43
TCGA-EB-A51B-01A	SKCM	5.74
TCGA-D3-A8GN-06A	SKCM	5.02
TCGA-EE-A20F-06A	SKCM	5.82
TCGA-3N-A9WB-06A	SKCM	5.61
TCGA-EB-A5UM-01A	SKCM	4.2
TCGA-FR-A3YO-06A	SKCM	4.88
TCGA-DA-A95Z-06A	SKCM	4.84
TCGA-FS-A4F4-06A	SKCM	4.42
TCGA-DA-A1I0-06A	SKCM	5.51
TCGA-FS-A1Z4-06A	SKCM	5.73
TCGA-RP-A693-06A	SKCM	6.4
TCGA-EE-A3AF-06A	SKCM	6.09
TCGA-EE-A3JA-06A	SKCM	5.31
TCGA-XV-A9VZ-01A	SKCM	4.78
TCGA-EE-A29V-06A	SKCM	6.51
TCGA-EB-A85I-01A	SKCM	4.69
TCGA-EE-A29A-06A	SKCM	4.82
TCGA-FW-A3TU-06A	SKCM	4.91
TCGA-RP-A6K9-06A	SKCM	5.14
TCGA-ER-A19L-06A	SKCM	5.1
TCGA-D3-A5GT-01A	SKCM	3.54
TCGA-HR-A2OH-06A	SKCM	5.83
TCGA-WE-A8JZ-06A	SKCM	5.29
TCGA-EE-A3JB-06A	SKCM	7.25
TCGA-BF-AAP1-01A	SKCM	5.77
TCGA-D3-A1Q9-06A	SKCM	7.87
TCGA-EE-A2M8-06A	SKCM	5.43
TCGA-BF-AAP0-06A	SKCM	5.96
TCGA-3N-A9WC-06A	SKCM	5.43
TCGA-D3-A3MU-06A	SKCM	4.5
TCGA-BF-A1Q0-01A	SKCM	4.51
TCGA-BF-AAP4-01A	SKCM	7.31
TCGA-ER-A19M-06A	SKCM	6.31
TCGA-ER-A19Q-06A	SKCM	6.33
TCGA-FS-A1ZW-06A	SKCM	5.21

TCGA-D3-A2JH-06A	SKCM	6.84
TCGA-EE-A2MK-06A	SKCM	5.76
TCGA-W3-AA21-06A	SKCM	4.47
TCGA-BF-A1PU-01A	SKCM	5.98
TCGA-ER-A42L-06A	SKCM	7.9
TCGA-FW-A3I3-06A	SKCM	5.9
TCGA-FS-A4F2-06A	SKCM	6.23
TCGA-EB-A97M-01A	SKCM	5.08
TCGA-XV-AB01-06A	SKCM	2.24
TCGA-DA-A1HY-06A	SKCM	5.32
TCGA-DA-A1I2-06A	SKCM	5.48
TCGA-HR-A2OG-06A	SKCM	5.25
TCGA-EE-A3J7-06A	SKCM	5.49
TCGA-BF-AAP7-01A	SKCM	5
TCGA-ER-A3ET-06A	SKCM	6.99
TCGA-ER-A197-06A	SKCM	6.57
TCGA-FS-A1ZT-06A	SKCM	6.78
TCGA-FS-A4F0-06A	SKCM	6.38
TCGA-FS-A1YW-06A	SKCM	4.7
TCGA-GF-A6C9-06A	SKCM	5.54
TCGA-D3-A3C7-06A	SKCM	5.17
TCGA-EE-A2GM-06B	SKCM	5.72
TCGA-EE-A2MS-06A	SKCM	5.2
TCGA-EE-A2ML-06A	SKCM	6.88
TCGA-FS-A1ZP-06A	SKCM	5.99
TCGA-ER-A195-06A	SKCM	6.11
TCGA-FW-A3TV-06A	SKCM	6.31
TCGA-EB-A4IQ-01A	SKCM	4.68
TCGA-EB-A3XC-01A	SKCM	6.27
TCGA-GN-A265-06A	SKCM	6.04
TCGA-EE-A2MN-06A	SKCM	5.48
TCGA-FS-A4F9-06A	SKCM	5.46
TCGA-GF-A6C8-06A	SKCM	6.53
TCGA-EE-A2GC-06A	SKCM	5.39
TCGA-EB-A5KH-06A	SKCM	4.39
TCGA-ER-A19F-06A	SKCM	6.51
TCGA-FS-A1ZK-06A	SKCM	7.27
TCGA-FR-A44A-06A	SKCM	5.83
TCGA-BF-A9VF-01A	SKCM	5.74
TCGA-YG-AA3O-06A	SKCM	6.29
TCGA-D3-A1Q6-06A	SKCM	5.55
TCGA-ER-A2ND-06A	SKCM	4.58
TCGA-FR-A7U9-06A	SKCM	5.54
TCGA-BF-A3DM-01A	SKCM	6.61
TCGA-D9-A6E9-06A	SKCM	5.98
TCGA-D3-A2JN-06A	SKCM	5.62
TCGA-FS-A1ZJ-06A	SKCM	5.7
TCGA-D3-A3CC-06A	SKCM	6.24
TCGA-DA-A1HV-06A	SKCM	5.75
TCGA-D3-A51F-06A	SKCM	6.04
TCGA-FR-A7U8-06A	SKCM	5.33
TCGA-GF-A2C7-01A	SKCM	5.73
TCGA-W3-A825-06A	SKCM	5.42
TCGA-D3-A1Q5-06A	SKCM	5.67
TCGA-D3-A3C1-06A	SKCM	5.55
TCGA-FS-A1ZS-06A	SKCM	6.57
TCGA-EE-A2MC-06A	SKCM	6.16
TCGA-RP-A695-06A	SKCM	6.8

TCGA-EE-A2MU-06A	SKCM	6.45
TCGA-EE-A2MF-06A	SKCM	6.52
TCGA-WE-AA9Y-06A	SKCM	4.83
TCGA-GN-A263-01A	SKCM	6.67
TCGA-D9-A149-06A	SKCM	6.28
TCGA-D3-A3CF-06A	SKCM	5.87
TCGA-WE-A8K5-06A	SKCM	5.39
TCGA-D3-A3C8-06A	SKCM	5.79
TCGA-FS-A1ZA-06A	SKCM	6.08
TCGA-YG-AA3N-01A	SKCM	4.88
TCGA-EE-A3JI-06A	SKCM	6.6
TCGA-FS-A1Z3-06A	SKCM	5.39
TCGA-ER-A194-01A	SKCM	3.41
TCGA-DA-A1IA-06A	SKCM	5.07
TCGA-HR-A5NC-01A	SKCM	5.84
TCGA-EB-A24C-01A	SKCM	4.83
TCGA-EB-A1NK-01A	SKCM	4.93
TCGA-D3-A51R-06A	SKCM	5.74
TCGA-QB-AA9O-06A	SKCM	5.24
TCGA-D3-A2JE-06A	SKCM	5.23
TCGA-EB-A5SF-01A	SKCM	4.43
TCGA-D3-A51J-06A	SKCM	5.52
TCGA-D3-A2JA-06A	SKCM	7.32
TCGA-D3-A3ML-06A	SKCM	5.41
TCGA-EB-A4OZ-01A	SKCM	3.18
TCGA-ER-A19D-06A	SKCM	6.14
TCGA-GN-A266-06A	SKCM	6.16
TCGA-EB-A5FP-01A	SKCM	6.55
TCGA-W3-AA1W-06A	SKCM	5.75
TCGA-EE-A184-06A	SKCM	6
TCGA-FS-A1ZE-06A	SKCM	5.11
TCGA-EE-A3JH-06A	SKCM	5.54
TCGA-EE-A20H-06A	SKCM	6.58
TCGA-D3-A8GV-06A	SKCM	6.66
TCGA-D3-A8GQ-06A	SKCM	5.42
TCGA-ER-A2NE-06A	SKCM	6.22
TCGA-D3-A3MR-06A	SKCM	4.13
TCGA-ER-A1A1-06A	SKCM	4.99
TCGA-D3-A2J7-06A	SKCM	6.2
TCGA-EE-A29M-06A	SKCM	7.73
TCGA-EB-A4IS-01A	SKCM	5.37
TCGA-EE-A2GU-06A	SKCM	7.25
TCGA-D9-A4Z3-01A	SKCM	5.19
TCGA-EE-A29X-06A	SKCM	5.66
TCGA-FS-A4F8-06A	SKCM	4.66
TCGA-FR-A8YD-06A	SKCM	5.04
TCGA-DA-A3F3-06A	SKCM	5.91
TCGA-BF-AAOX-01A	SKCM	5.71
TCGA-ER-A19E-06A	SKCM	6.55
TCGA-D3-A2JL-06A	SKCM	5.64
TCGA-EE-A3J4-06A	SKCM	5.44
TCGA-D3-A1Q3-06A	SKCM	6.12
TCGA-EE-A3AG-06A	SKCM	5.06
TCGA-BF-A5ES-01A	SKCM	5.81
TCGA-ER-A19B-06A	SKCM	6.56
TCGA-ER-A2NG-06A	SKCM	6.95
TCGA-EB-A6L9-06A	SKCM	4.32
TCGA-D3-A1QA-06A	SKCM	7.07

TCGA-FW-A3R5-06A	SKCM	6.68
TCGA-D3-A3C3-06A	SKCM	5.52
TCGA-WE-A8ZX-06A	SKCM	6
TCGA-EB-A42Z-01A	SKCM	5.4
TCGA-YG-AA3P-06A	SKCM	3.7
TCGA-FS-A1ZD-06A	SKCM	7.11
TCGA-GN-A4U5-01A	SKCM	5.06
TCGA-EE-A2GN-06A	SKCM	5.71
TCGA-GN-A4U9-06A	SKCM	4.83
TCGA-EE-A3JE-06A	SKCM	5.26
TCGA-D9-A3Z4-01A	SKCM	5.05
TCGA-EE-A2MI-06A	SKCM	6.25
TCGA-ER-A19J-06A	SKCM	5.92
TCGA-D9-A6EG-06A	SKCM	8.28
TCGA-EB-A44O-01A	SKCM	7.64
TCGA-D9-A6EC-06A	SKCM	6.99
TCGA-EE-A17Y-06A	SKCM	6.21
TCGA-EB-A42Y-01A	SKCM	5.21
TCGA-FR-A728-01A	SKCM	4.8
TCGA-DA-A1I7-06A	SKCM	5.57
TCGA-D3-A2J9-06A	SKCM	5.91
TCGA-BF-A3DL-01A	SKCM	4.69
TCGA-BF-A1PV-01A	SKCM	6.2
TCGA-WE-A8ZN-06A	SKCM	3.93
TCGA-D3-A1QA-07A	SKCM	7.02
TCGA-BR-6457-01A	STAD	5.38
TCGA-BR-A4QM-01A	STAD	4.31
TCGA-CG-5723-01A	STAD	5.87
TCGA-BR-8591-01A	STAD	6.51
TCGA-BR-7957-01A	STAD	6.45
TCGA-BR-8368-01A	STAD	6.76
TCGA-BR-A4J2-01A	STAD	6.07
TCGA-CD-5813-01A	STAD	5
TCGA-FP-7735-01A	STAD	6.31
TCGA-CD-8528-01A	STAD	7.18
TCGA-D7-8570-01A	STAD	6.57
TCGA-BR-8485-01A	STAD	7.4
TCGA-BR-8060-01A	STAD	5.41
TCGA-RD-A8N4-01A	STAD	4.38
TCGA-VQ-A8PC-01A	STAD	6.47
TCGA-BR-7958-01A	STAD	5.77
TCGA-CD-8527-01A	STAD	5.57
TCGA-BR-6456-01A	STAD	6.58
TCGA-F1-6875-01A	STAD	5.91
TCGA-IN-A6RJ-01A	STAD	5.23
TCGA-BR-7703-01A	STAD	6.13
TCGA-BR-A4CS-01A	STAD	5.77
TCGA-CG-4469-01A	STAD	5.68
TCGA-FP-8211-01A	STAD	6.42
TCGA-CG-5724-01A	STAD	4.87
TCGA-HU-A4GJ-01A	STAD	5.08
TCGA-RD-A8MW-01A	STAD	5.71
TCGA-BR-7851-01A	STAD	6.82
TCGA-BR-A4PF-01A	STAD	7.78
TCGA-FP-A4BF-01A	STAD	5.25
TCGA-F1-6874-01A	STAD	6.25
TCGA-RD-A8N2-01A	STAD	3.99
TCGA-HU-A4G8-01A	STAD	6.02

TCGA-D7-A4YY-01A	STAD	6.82
TCGA-CG-4477-01A	STAD	5.64
TCGA-D7-8575-01A	STAD	6.59
TCGA-BR-8081-01A	STAD	6.32
TCGA-BR-8291-01A	STAD	5.88
TCGA-CD-A4MH-01A	STAD	6.42
TCGA-IN-A7NU-01A	STAD	4.94
TCGA-VQ-A8PB-01A	STAD	6.88
TCGA-VQ-A91K-01A	STAD	6.97
TCGA-BR-4361-01A	STAD	6.68
TCGA-IN-A6RO-01A	STAD	7.56
TCGA-FP-8631-01A	STAD	6.94
TCGA-BR-6452-01A	STAD	8.43
TCGA-CG-5719-01A	STAD	5.49
TCGA-RD-A7BT-01A	STAD	6.78
TCGA-BR-8296-01A	STAD	5.97
TCGA-CD-A4MJ-01A	STAD	7.03
TCGA-CG-4438-01A	STAD	7.27
TCGA-D7-A4YX-01A	STAD	6.97
TCGA-HU-A4HB-01A	STAD	6.6
TCGA-B7-A5TI-01A	STAD	6.36
TCGA-D7-A74A-01A	STAD	7.17
TCGA-VQ-A8PE-01A	STAD	6.43
TCGA-BR-4253-01A	STAD	7.05
TCGA-MX-A5UJ-01A	STAD	6.13
TCGA-HU-A4G2-01A	STAD	6.68
TCGA-HU-A4GY-01A	STAD	5.11
TCGA-EQ-8122-01A	STAD	7.19
TCGA-CG-4476-01A	STAD	5.95
TCGA-VQ-AA6I-01A	STAD	7.96
TCGA-RD-A8MV-01A	STAD	5.54
TCGA-BR-8678-01A	STAD	6.43
TCGA-BR-6801-01A	STAD	5.88
TCGA-IN-AB1X-01A	STAD	6.42
TCGA-D7-A6EX-01A	STAD	5.33
TCGA-BR-7196-01A	STAD	6.42
TCGA-KB-A6F7-01A	STAD	6.34
TCGA-VQ-A91A-01A	STAD	6.13
TCGA-BR-8371-01A	STAD	4.28
TCGA-VQ-AA6B-01A	STAD	7.27
TCGA-IN-A7NT-01A	STAD	8.18
TCGA-D7-6520-01A	STAD	6.57
TCGA-HF-7134-01A	STAD	6.78
TCGA-RD-A8N0-01A	STAD	5.59
TCGA-D7-A6EZ-01A	STAD	7.04
TCGA-FP-8209-01A	STAD	4.45
TCGA-VQ-A922-01A	STAD	6.28
TCGA-ZA-A8F6-01A	STAD	5.79
TCGA-BR-7901-01A	STAD	6.08
TCGA-BR-8680-01A	STAD	7.47
TCGA-VQ-A94R-01A	STAD	6.19
TCGA-BR-6564-01A	STAD	4.37
TCGA-BR-6454-01A	STAD	5.82
TCGA-HU-8238-01A	STAD	5.45
TCGA-FP-7998-01A	STAD	5.89
TCGA-BR-8367-01A	STAD	7.07
TCGA-CD-A48A-01A	STAD	6.9
TCGA-R5-A7ZI-01A	STAD	4.76

TCGA-BR-7959-01A	STAD	5.32
TCGA-BR-8059-01A	STAD	5.19
TCGA-BR-7723-01A	STAD	6.12
TCGA-VQ-AA6G-01A	STAD	7.22
TCGA-HU-A4H2-01A	STAD	6.62
TCGA-R5-A7ZE-01B	STAD	6.44
TCGA-BR-8686-01A	STAD	6.11
TCGA-D7-A6EV-01A	STAD	7.27
TCGA-BR-6802-01A	STAD	5.83
TCGA-CG-4301-01A	STAD	6.89
TCGA-HU-A4GX-01A	STAD	6.06
TCGA-HU-8243-01A	STAD	6.89
TCGA-VQ-A927-01A	STAD	6.46
TCGA-HJ-7597-01A	STAD	5.1
TCGA-BR-7707-01A	STAD	6.45
TCGA-BR-8289-01A	STAD	7.58
TCGA-CG-4440-01A	STAD	7.37
TCGA-BR-A4J4-01A	STAD	6.46
TCGA-HU-A4G9-01A	STAD	6.82
TCGA-D7-8579-01A	STAD	5.83
TCGA-FP-8210-01A	STAD	5.28
TCGA-BR-8361-01A	STAD	6.14
TCGA-VQ-A94U-01A	STAD	5.31
TCGA-CG-4442-01A	STAD	7
TCGA-HU-A4GD-01A	STAD	5.5
TCGA-BR-4255-01A	STAD	6.3
TCGA-BR-6563-01A	STAD	5.07
TCGA-BR-7704-01A	STAD	6.54
TCGA-BR-4294-01A	STAD	5.59
TCGA-BR-8588-01A	STAD	6.22
TCGA-D7-8574-01A	STAD	5.2
TCGA-SW-A7EB-01A	STAD	6.3
TCGA-BR-8284-01A	STAD	6.91
TCGA-D7-6526-01A	STAD	6.8
TCGA-VQ-A8E2-01A	STAD	7.34
TCGA-BR-8487-01A	STAD	6.42
TCGA-BR-A4QL-01A	STAD	5.68
TCGA-BR-8690-01A	STAD	5.53
TCGA-CD-5803-01A	STAD	5.28
TCGA-VQ-A91D-01A	STAD	6.39
TCGA-BR-A4IV-01A	STAD	4.7
TCGA-D7-6528-01A	STAD	4.75
TCGA-BR-8297-01A	STAD	5.91
TCGA-FP-A9TM-01A	STAD	6.8
TCGA-CG-5734-01A	STAD	5.05
TCGA-VQ-AA68-01A	STAD	6.97
TCGA-BR-A4J7-01A	STAD	4.53
TCGA-HU-8604-01A	STAD	5.29
TCGA-BR-8058-01A	STAD	5.86
TCGA-R5-A7O7-01A	STAD	5.96
TCGA-CG-4460-01A	STAD	6.41
TCGA-CG-5720-01A	STAD	5.16
TCGA-D7-6525-01A	STAD	5.63
TCGA-IN-7806-01A	STAD	6.83
TCGA-BR-8683-01A	STAD	6.54
TCGA-BR-7197-01A	STAD	6.22
TCGA-CD-5804-01A	STAD	6.44
TCGA-BR-6706-01A	STAD	5.09

TCGA-BR-8381-01A	STAD	6.12
TCGA-R5-A7ZF-01A	STAD	5.69
TCGA-BR-A4IU-01A	STAD	5.36
TCGA-IN-8462-01A	STAD	5.06
TCGA-VQ-A94P-01A	STAD	4.63
TCGA-D7-A748-01A	STAD	5.62
TCGA-D7-A6F0-01A	STAD	7.5
TCGA-IN-A6RS-01A	STAD	6.82
TCGA-VQ-A924-01A	STAD	6.84
TCGA-VQ-AA6K-01A	STAD	6.96
TCGA-BR-A44U-01A	STAD	5.83
TCGA-VQ-A8DT-01A	STAD	7.47
TCGA-BR-7722-01A	STAD	4.97
TCGA-CG-5726-01A	STAD	6.91
TCGA-VQ-A8E7-01B	STAD	6.81
TCGA-BR-4191-01A	STAD	6.66
TCGA-HU-A4G3-01A	STAD	6.23
TCGA-D7-6524-01A	STAD	6.32
TCGA-SW-A7EA-01A	STAD	5.43
TCGA-VQ-A91Y-01A	STAD	6.26
TCGA-D7-8572-01A	STAD	7.08
TCGA-BR-4201-01A	STAD	6.21
TCGA-CG-4443-01A	STAD	6.94
TCGA-3M-AB47-01A	STAD	5.39
TCGA-BR-A44T-01A	STAD	4.12
TCGA-CG-4304-01A	STAD	6.08
TCGA-VQ-A8P2-01A	STAD	7.92
TCGA-BR-6455-01A	STAD	8.37
TCGA-BR-8286-01A	STAD	5.54
TCGA-CD-A489-01A	STAD	4.67
TCGA-VQ-A91W-01A	STAD	8.3
TCGA-VQ-A8PD-01A	STAD	6.82
TCGA-RD-A8N9-01A	STAD	5.6
TCGA-HU-A4H8-01A	STAD	7.31
TCGA-D7-6522-01A	STAD	4.76
TCGA-BR-7717-01A	STAD	6.42
TCGA-BR-A4QI-01A	STAD	7.59
TCGA-CD-8532-01A	STAD	6.33
TCGA-BR-A4IY-01A	STAD	5.79
TCGA-CD-8536-01A	STAD	6.61
TCGA-BR-8589-01A	STAD	7.06
TCGA-HF-A5NB-01A	STAD	7.28
TCGA-BR-6453-01A	STAD	5.53
TCGA-BR-4357-01A	STAD	8.05
TCGA-RD-A7BW-01A	STAD	4.8
TCGA-VQ-A8PM-01A	STAD	6.35
TCGA-VQ-AA6J-01A	STAD	7.7
TCGA-D7-6818-01A	STAD	5.21
TCGA-BR-A4IZ-01A	STAD	5.91
TCGA-VQ-A91Z-01A	STAD	6.82
TCGA-VQ-A8PU-01A	STAD	6.65
TCGA-D7-6527-01A	STAD	6.43
TCGA-BR-4367-01A	STAD	7.04
TCGA-VQ-A91S-01A	STAD	5.79
TCGA-RD-A7C1-01A	STAD	6.27
TCGA-BR-8080-01A	STAD	5.23
TCGA-CG-4465-01A	STAD	7.06
TCGA-BR-7716-01A	STAD	7.92

TCGA-HU-8249-01A	STAD	5.36
TCGA-BR-8382-01A	STAD	6.14
TCGA-CD-5798-01A	STAD	5.35
TCGA-VQ-A8PX-01A	STAD	6.64
TCGA-CD-A48C-01A	STAD	6.77
TCGA-CG-5722-01A	STAD	5.12
TCGA-IN-7808-01A	STAD	4.24
TCGA-RD-A7BS-01A	STAD	5.62
TCGA-VQ-A8PF-01A	STAD	6.87
TCGA-VQ-A92D-01A	STAD	6.41
TCGA-BR-8380-01A	STAD	5.17
TCGA-BR-8592-01A	STAD	5.43
TCGA-BR-6705-01A	STAD	5.43
TCGA-BR-8676-01A	STAD	7.29
TCGA-HU-8608-01A	STAD	6.62
TCGA-CG-5717-01A	STAD	6.09
TCGA-BR-4257-01A	STAD	8.03
TCGA-BR-A4PD-01A	STAD	6.18
TCGA-BR-8590-01A	STAD	6.73
TCGA-KB-A93H-01A	STAD	7.04
TCGA-3M-AB46-01A	STAD	6.07
TCGA-VQ-AA6F-01A	STAD	5.96
TCGA-MX-A663-01A	STAD	6.73
TCGA-BR-6707-01A	STAD	6.9
TCGA-BR-8682-01A	STAD	6.7
TCGA-ZQ-A9CR-01A	STAD	6.3
TCGA-VQ-A8E0-01A	STAD	6.62
TCGA-VQ-A91N-01A	STAD	7.35
TCGA-BR-4371-01A	STAD	5.93
TCGA-F1-6177-01A	STAD	7.06
TCGA-VQ-A8DV-01A	STAD	6.21
TCGA-F1-A448-01A	STAD	6.11
TCGA-HU-A4HD-01A	STAD	6.61
TCGA-CD-A4MI-01A	STAD	5.67
TCGA-HU-A4GP-01A	STAD	6.08
TCGA-CD-5799-01A	STAD	6.4
TCGA-IN-A6RL-01A	STAD	6.59
TCGA-IN-A6RN-01A	STAD	6.1
TCGA-D7-A4Z0-01A	STAD	5.99
TCGA-IN-8663-01A	STAD	7.25
TCGA-CG-4475-01A	STAD	7.73
TCGA-BR-8483-01A	STAD	7.03
TCGA-BR-6458-01A	STAD	6.18
TCGA-CD-5800-01A	STAD	6.99
TCGA-R5-A7ZR-01A	STAD	7.02
TCGA-BR-8078-01A	STAD	6.34
TCGA-B7-5816-01A	STAD	6.25
TCGA-D7-8573-01A	STAD	6.56
TCGA-BR-4369-01A	STAD	7.06
TCGA-FP-A8CX-01A	STAD	7.34
TCGA-BR-8679-01A	STAD	6.84
TCGA-VQ-A8P8-01A	STAD	6.6
TCGA-CD-A4MG-01A	STAD	5.86
TCGA-VQ-A94T-01A	STAD	7.33
TCGA-BR-8677-01A	STAD	5.33
TCGA-BR-A4J1-01A	STAD	7.62
TCGA-VQ-A8E3-01A	STAD	6.11
TCGA-D7-A6F2-01A	STAD	6.37

TCGA-MX-A5UG-01A	STAD	5.75
TCGA-IN-A7NR-01A	STAD	6.18
TCGA-RD-A8NB-01A	STAD	6.25
TCGA-HU-A4GN-01A	STAD	6.16
TCGA-BR-8077-01A	STAD	7.09
TCGA-IN-A6RI-01A	STAD	7.26
TCGA-IP-7968-01A	STAD	6.97
TCGA-VQ-AA64-01A	STAD	7.4
TCGA-BR-A4PE-01A	STAD	7.21
TCGA-HU-A4H5-01A	STAD	7.55
TCGA-HU-A4GH-01A	STAD	6.22
TCGA-CG-5718-01A	STAD	5.75
TCGA-D7-6822-01A	STAD	6.31
TCGA-CD-5801-01A	STAD	6.34
TCGA-BR-4280-01A	STAD	6.35
TCGA-HU-8244-01A	STAD	6.11
TCGA-MX-A666-01A	STAD	7.4
TCGA-BR-8362-01A	STAD	7.4
TCGA-BR-6565-01A	STAD	6.56
TCGA-VQ-A94O-01A	STAD	7.26
TCGA-VQ-A8PP-01A	STAD	6.81
TCGA-HU-8602-01A	STAD	6.96
TCGA-HU-A4GF-01A	STAD	6.67
TCGA-CG-4462-01A	STAD	6.17
TCGA-D7-6519-01A	STAD	5.78
TCGA-RD-A8N6-01A	STAD	6.34
TCGA-CD-8531-01A	STAD	4.13
TCGA-VQ-AA6A-01A	STAD	7.72
TCGA-FP-7916-01A	STAD	6.34
TCGA-IN-AB1V-01A	STAD	6.47
TCGA-KB-A93G-01A	STAD	5.87
TCGA-BR-8369-01A	STAD	6.5
TCGA-CG-5732-01A	STAD	6.76
TCGA-VQ-A91Q-01A	STAD	6.92
TCGA-CD-A487-01A	STAD	5.71
TCGA-BR-4370-01A	STAD	5.86
TCGA-VQ-A8DZ-01A	STAD	7.22
TCGA-CD-A486-01A	STAD	6.51
TCGA-D7-5578-01A	STAD	5.99
TCGA-BR-8486-01A	STAD	6.7
TCGA-CD-8533-01A	STAD	8.22
TCGA-HU-A4GU-01A	STAD	6.98
TCGA-BR-4368-01A	STAD	5.33
TCGA-VQ-A8P3-01A	STAD	6.07
TCGA-BR-7715-01A	STAD	6.18
TCGA-BR-8295-01A	STAD	7.46
TCGA-F1-A72C-01A	STAD	6.1
TCGA-HF-7133-01A	STAD	6.02
TCGA-D7-8578-01A	STAD	6.24
TCGA-BR-4292-01A	STAD	6.82
TCGA-BR-4366-01A	STAD	7.11
TCGA-HU-A4GQ-01A	STAD	6.32
TCGA-VQ-A8PH-01A	STAD	5.51
TCGA-B7-5818-01A	STAD	7.11
TCGA-VQ-A8PQ-01A	STAD	5.5
TCGA-RD-A8N5-01A	STAD	4.56
TCGA-D7-5577-01A	STAD	6.99
TCGA-HU-A4G6-01A	STAD	7.11

TCGA-VQ-A91E-01A	STAD	7.33
TCGA-CD-8525-01A	STAD	5
TCGA-VQ-A928-01A	STAD	6.82
TCGA-D7-A4YU-01A	STAD	5.9
TCGA-CG-4441-01A	STAD	7.04
TCGA-D7-A747-01A	STAD	4.67
TCGA-D7-6518-01A	STAD	4.74
TCGA-VQ-AA69-01A	STAD	7.44
TCGA-RD-A8N1-01A	STAD	6.92
TCGA-D7-A4YV-01A	STAD	6.29
TCGA-HU-A4H6-01A	STAD	5.52
TCGA-FP-A4BE-01A	STAD	6.88
TCGA-BR-6852-01A	STAD	5.84
TCGA-CG-5716-01A	STAD	5.42
TCGA-VQ-A8PO-01A	STAD	6.85
TCGA-VQ-A91V-01A	STAD	7.42
TCGA-B7-A5TK-01A	STAD	5.5
TCGA-BR-A4J5-01A	STAD	6.19
TCGA-BR-8372-01A	STAD	7.32
TCGA-CD-8529-01A	STAD	6.77
TCGA-B7-A5TJ-01A	STAD	6.78
TCGA-VQ-A91X-01A	STAD	6.77
TCGA-CG-4437-01A	STAD	5.79
TCGA-BR-8364-01A	STAD	5.75
TCGA-VQ-A8DU-01A	STAD	6.03
TCGA-CG-5721-01A	STAD	6.24
TCGA-KB-A93J-01A	STAD	7.67
TCGA-VQ-A91U-01A	STAD	7.86
TCGA-VQ-A923-01A	STAD	6.18
TCGA-BR-6566-01A	STAD	5.5
TCGA-BR-8365-01A	STAD	4.86
TCGA-BR-A4J8-01A	STAD	5.06
TCGA-HU-A4H4-01A	STAD	6.15
TCGA-VQ-A925-01A	STAD	7.33
TCGA-BR-6710-01A	STAD	6.08
TCGA-CG-4466-01A	STAD	7.09
TCGA-BR-6803-01A	STAD	5.35
TCGA-CG-4444-01A	STAD	6.59
TCGA-BR-8687-01A	STAD	5.91
TCGA-BR-8366-01A	STAD	5.53
TCGA-CD-8535-01A	STAD	5.47
TCGA-D7-6817-01A	STAD	8.19
TCGA-BR-A4J9-01A	STAD	3.92
TCGA-BR-4363-01A	STAD	6.45
TCGA-B7-A5TN-01A	STAD	6.16
TCGA-BR-8373-01A	STAD	7.45
TCGA-VQ-AA6D-01A	STAD	7.35
TCGA-BR-8384-01A	STAD	4.57
TCGA-CD-8526-01A	STAD	5.96
TCGA-HF-7136-01A	STAD	6.64
TCGA-FP-8099-01A	STAD	6.45
TCGA-D7-A4YT-01A	STAD	6.64
TCGA-BR-8363-01A	STAD	5.74
TCGA-D7-6815-01A	STAD	7.48
TCGA-CD-8524-01A	STAD	7.5
TCGA-FP-7829-01A	STAD	7.91
TCGA-HU-A4H3-01A	STAD	6.29
TCGA-VQ-A8PK-01A	STAD	6.07

TCGA-BR-6709-01A	STAD	5.53
TCGA-R5-A805-01A	STAD	6.5
TCGA-CD-8534-01A	STAD	4.78
TCGA-D7-A6EY-01A	STAD	6.53
TCGA-IN-A6RR-01A	STAD	6.91
TCGA-BR-A452-01A	STAD	6.5
TCGA-HF-7132-01A	STAD	6.52
TCGA-D7-6521-01A	STAD	6.35
TCGA-CG-4306-01A	STAD	6.24
TCGA-BR-4256-01A	STAD	6.5
TCGA-BR-A4J6-01A	STAD	7.19
TCGA-BR-A453-01A	STAD	4.22
TCGA-BR-4279-01A	STAD	6.17
TCGA-BR-4187-01A	STAD	5.73
TCGA-CG-5725-01A	STAD	5.02
TCGA-HU-8610-01A	STAD	6.67
TCGA-HU-A4GC-01A	STAD	6.87
TCGA-VQ-A8P5-01A	STAD	7.06
TCGA-D7-6820-01A	STAD	6.46
TCGA-CG-4436-01A	STAD	6.61
TCGA-BR-A4CR-01A	STAD	4.97
TCGA-HU-A4GT-01A	STAD	7.18
TCGA-VQ-A8PJ-01A	STAD	7.49
TCGA-HF-7131-01A	STAD	6.95
TCGA-BR-4267-01A	STAD	7.3
TCGA-CD-8530-01A	STAD	5.48
TCGA-CG-4305-01A	STAD	6.69
TCGA-BR-8484-01A	STAD	6.33
TCGA-EQ-A4SO-01A	STAD	6.64
TCGA-HU-A4H0-01A	STAD	7.27
TCGA-D7-8576-01A	STAD	7.51
TCGA-2G-AAFE-01A	TGCT	6.67
TCGA-S6-A8JY-01A	TGCT	6.76
TCGA-2G-AAGY-01A	TGCT	6.12
TCGA-2G-AAG0-01A	TGCT	8.1
TCGA-2X-A9D6-01A	TGCT	6.3
TCGA-2G-AAKO-01A	TGCT	5.66
TCGA-2G-AAG9-01A	TGCT	6.25
TCGA-4K-AA1H-01A	TGCT	5.8
TCGA-2G-AAGT-01A	TGCT	8.59
TCGA-2G-AAFH-01A	TGCT	7.29
TCGA-2G-AALT-01A	TGCT	6.12
TCGA-VF-A8AC-01A	TGCT	5.91
TCGA-2G-AAKG-05A	TGCT	5.02
TCGA-XY-A8S3-01B	TGCT	8.25
TCGA-XE-AANV-01A	TGCT	5.86
TCGA-W4-A7U2-01A	TGCT	6.38
TCGA-2X-A9D5-01A	TGCT	6.12
TCGA-2G-AAH2-01A	TGCT	7.04
TCGA-VF-A8A8-01A	TGCT	7.77
TCGA-2G-AAG6-01A	TGCT	5.77
TCGA-2G-AALS-01A	TGCT	6.09
TCGA-2G-AAGW-01A	TGCT	6.93
TCGA-2G-AAKG-01A	TGCT	7.54
TCGA-ZM-AA0F-01A	TGCT	4.69
TCGA-2G-AAFO-01A	TGCT	6.95
TCGA-2G-AAGA-01A	TGCT	6.89
TCGA-XE-AAOD-01A	TGCT	6.55

TCGA-WZ-A8D5-01A	TGCT	6.7
TCGA-2G-AAFL-01A	TGCT	6.37
TCGA-YU-A912-01A	TGCT	6.68
TCGA-2G-AAFJ-01A	TGCT	7.05
TCGA-2G-AAGN-01A	TGCT	8.09
TCGA-2G-AAHL-01A	TGCT	6.26
TCGA-XE-AAOF-01A	TGCT	6.14
TCGA-2G-AAL7-01A	TGCT	7.31
TCGA-XE-A8H5-01A	TGCT	6.71
TCGA-2G-AALQ-01A	TGCT	6.55
TCGA-ZM-AA0D-01A	TGCT	5.32
TCGA-W4-A7U3-01A	TGCT	8.08
TCGA-2G-AAFN-01A	TGCT	7.27
TCGA-2G-AAEW-01A	TGCT	6.78
TCGA-XE-AAO4-01A	TGCT	6.82
TCGA-YU-AA61-01A	TGCT	8.36
TCGA-SN-A6IS-01A	TGCT	6.67
TCGA-SN-A84X-01A	TGCT	6.02
TCGA-2G-AAGV-01A	TGCT	6.84
TCGA-SN-A84W-01A	TGCT	6.6
TCGA-2G-AAGE-01A	TGCT	7.76
TCGA-2G-AAH8-01A	TGCT	6.65
TCGA-XE-A9SE-01A	TGCT	6.67
TCGA-2G-AAGI-05A	TGCT	6.18
TCGA-XE-A8H4-01A	TGCT	6.59
TCGA-XY-A9T9-01A	TGCT	6.14
TCGA-2G-AAHT-01A	TGCT	6.98
TCGA-SN-A84Y-01A	TGCT	7.85
TCGA-2G-AALG-01A	TGCT	5.69
TCGA-2G-AAGG-01A	TGCT	7.69
TCGA-VF-A8A9-01A	TGCT	6.13
TCGA-XE-AAOB-01A	TGCT	6.09
TCGA-XE-A8H1-01A	TGCT	7.35
TCGA-SB-A76C-01A	TGCT	6.29
TCGA-2G-AAGX-01A	TGCT	6.81
TCGA-S6-A8JW-01A	TGCT	6.46
TCGA-ZM-AA0E-01A	TGCT	4.98
TCGA-2G-AAHC-01A	TGCT	6.56
TCGA-2G-AAF8-01A	TGCT	7.19
TCGA-2G-AAKD-01A	TGCT	7.15
TCGA-2G-AAGY-05A	TGCT	7.71
TCGA-2G-AAKO-05A	TGCT	6.21
TCGA-2G-AAEX-01A	TGCT	6.43
TCGA-2G-AAF4-01A	TGCT	6.87
TCGA-2G-AAGC-01A	TGCT	7.8
TCGA-YU-AA4L-01A	TGCT	8.17
TCGA-ZM-AA05-01A	TGCT	3.82
TCGA-4K-AAAL-01A	TGCT	6.35
TCGA-ZM-AA06-01A	TGCT	4.25
TCGA-XE-AAO6-01A	TGCT	5.57
TCGA-2G-AAFI-01A	TGCT	8.14
TCGA-2G-AAFM-01A	TGCT	6.16
TCGA-2G-AAG3-01A	TGCT	6.17
TCGA-2G-AAGO-01A	TGCT	8.3
TCGA-2G-AAM3-01A	TGCT	6.2
TCGA-2G-AAM4-01A	TGCT	7.34
TCGA-YU-A90W-01A	TGCT	6.85
TCGA-2G-AAFG-05A	TGCT	6.46

TCGA-2G-AAFG-01A	TGCT	6.55
TCGA-XE-AAOC-01A	TGCT	6.49
TCGA-YU-A90Q-01A	TGCT	6.17
TCGA-SB-A6J6-01A	TGCT	6.29
TCGA-ZM-AA0H-01A	TGCT	4.9
TCGA-2G-AAGK-01A	TGCT	7.51
TCGA-WZ-A7V4-01A	TGCT	6.55
TCGA-XE-AANJ-01A	TGCT	6.56
TCGA-2G-AALO-01A	TGCT	7.62
TCGA-2G-AAFY-01A	TGCT	7.29
TCGA-2G-AALY-01A	TGCT	7.98
TCGA-YU-A90S-01A	TGCT	6.02
TCGA-2G-AAG5-01A	TGCT	5.64
TCGA-2G-AAL5-01A	TGCT	6.9
TCGA-2G-AAGJ-01A	TGCT	6.99
TCGA-XY-A8S2-01A	TGCT	7.8
TCGA-2G-AAGZ-01A	TGCT	6.48
TCGA-2G-AAFV-01A	TGCT	7.99
TCGA-2G-AAF1-01A	TGCT	6.47
TCGA-YU-A94D-01A	TGCT	6.76
TCGA-W4-A7U4-01A	TGCT	7.68
TCGA-YU-A90P-01A	TGCT	7.46
TCGA-2G-AAH3-01A	TGCT	6.69
TCGA-VF-A8AA-01A	TGCT	6.04
TCGA-2G-AALZ-01A	TGCT	6.61
TCGA-2G-AAFZ-01A	TGCT	7.93
TCGA-2G-AAHG-01A	TGCT	6.86
TCGA-VF-A8AE-01A	TGCT	5.95
TCGA-2G-AAGP-01A	TGCT	7.02
TCGA-ZM-AA0B-01A	TGCT	5.38
TCGA-2G-AAHA-01A	TGCT	6.52
TCGA-4K-AA1G-01A	TGCT	7.24
TCGA-VF-A8AB-01A	TGCT	6.59
TCGA-2G-AALN-01A	TGCT	7.68
TCGA-YU-A94I-01A	TGCT	8.31
TCGA-2G-AAGM-01A	TGCT	7.31
TCGA-ZM-AA0N-01A	TGCT	4.53
TCGA-VF-A8AD-01A	TGCT	7.94
TCGA-2G-AAHP-05A	TGCT	5.31
TCGA-2G-AAG7-01A	TGCT	8.07
TCGA-WZ-A7V5-01A	TGCT	5.45
TCGA-2G-AALP-01A	TGCT	7.23
TCGA-2G-AAKM-01A	TGCT	8.02
TCGA-XE-AANI-01A	TGCT	6.42
TCGA-XE-AAOJ-01A	TGCT	7.03
TCGA-2G-AAG8-01A	TGCT	7.54
TCGA-XE-AANR-01A	TGCT	6.71
TCGA-2G-AALR-01A	TGCT	6.99
TCGA-2G-AAKL-01A	TGCT	7.76
TCGA-XY-A89B-01A	TGCT	6.12
TCGA-2G-AAH0-01A	TGCT	7.03
TCGA-2G-AAH4-01A	TGCT	7.06
TCGA-2G-AAGS-01A	TGCT	7.51
TCGA-SO-A8JP-01A	TGCT	6.54
TCGA-XE-AAOL-01A	TGCT	5.13
TCGA-XE-AAO3-01A	TGCT	7.18
TCGA-2G-AAGF-01A	TGCT	6.55
TCGA-2G-AAGI-01A	TGCT	8

TCGA-YU-A90Y-01A	TGCT	6.95
TCGA-2G-AALX-01A	TGCT	5.83
TCGA-2G-AAM2-01A	TGCT	8.84
TCGA-2G-AAHP-01A	TGCT	6.83
TCGA-2G-AALF-01A	TGCT	7.14
TCGA-2G-AAF6-01A	TGCT	6.7
TCGA-2G-AALW-01A	TGCT	7.44
TCGA-WZ-A7V3-01A	TGCT	6.28
TCGA-2G-AAKH-01A	TGCT	6.7
TCGA-2G-AAHN-01A	TGCT	6.24
TCGA-4K-AA1I-01A	TGCT	6.16
TCGA-X3-A8G4-01A	TGCT	7.53
TCGA-S6-A8JX-01A	TGCT	6.5
TCGA-EM-A2CO-01A	THCA	3.79
TCGA-EL-A3ZM-01A	THCA	5.94
TCGA-BJ-A190-01A	THCA	4.6
TCGA-DJ-A2PP-01A	THCA	6.72
TCGA-DJ-A2Q9-01A	THCA	5.89
TCGA-E8-A437-01A	THCA	4.93
TCGA-BJ-A2N9-01A	THCA	4.88
TCGA-DJ-A3VI-01A	THCA	6.13
TCGA-DJ-A3VM-01A	THCA	4.76
TCGA-EL-A3ZN-01A	THCA	4.66
TCGA-EL-A3CM-01A	THCA	5.2
TCGA-EM-A1CW-01A	THCA	5.57
TCGA-FE-A3PB-01A	THCA	4.59
TCGA-EL-A3CT-01A	THCA	7.47
TCGA-BJ-A0ZA-01A	THCA	5.73
TCGA-BJ-A3PU-01A	THCA	5.59
TCGA-DE-A0XZ-01A	THCA	5.64
TCGA-IM-A3EB-01A	THCA	6.35
TCGA-EL-A3GQ-01A	THCA	5.82
TCGA-EM-A3FN-01A	THCA	5.32
TCGA-FY-A3TY-01A	THCA	6.13
TCGA-DJ-A3UY-01A	THCA	5.89
TCGA-BJ-A28S-01A	THCA	5.2
TCGA-EL-A3CN-01A	THCA	5.77
TCGA-EM-A4FQ-01A	THCA	5.45
TCGA-J8-A3O0-01A	THCA	6.57
TCGA-EM-A4FV-01A	THCA	6.33
TCGA-ET-A3DS-01A	THCA	5.98
TCGA-EL-A4KH-01A	THCA	4.84
TCGA-DJ-A3UW-01A	THCA	5.14
TCGA-DJ-A3V3-01A	THCA	5.25
TCGA-FE-A231-01A	THCA	5.69
TCGA-EM-A2CU-01A	THCA	5.52
TCGA-DJ-A3V2-01A	THCA	5.86
TCGA-ET-A40P-01A	THCA	4.83
TCGA-DJ-A3UN-01A	THCA	6.53
TCGA-ET-A25M-01A	THCA	5.49
TCGA-EM-A2P0-01A	THCA	6.36
TCGA-ET-A2N5-01A	THCA	5.38
TCGA-FY-A76V-01A	THCA	5.46
TCGA-GE-A2C6-01A	THCA	6.11
TCGA-FY-A3W9-01A	THCA	5.85
TCGA-EM-A2CQ-01A	THCA	5.8
TCGA-DJ-A3V6-01A	THCA	5.66
TCGA-BJ-A0ZJ-01A	THCA	5.6

TCGA-EL-A3MY-01A	THCA	6.06
TCGA-BJ-A0ZH-01A	THCA	5.21
TCGA-EL-A3CU-01A	THCA	6.02
TCGA-KS-A4ID-01A	THCA	5.03
TCGA-EM-A2CK-01A	THCA	5.38
TCGA-EM-A4FR-01A	THCA	4.21
TCGA-DJ-A4UL-01A	THCA	6.1
TCGA-DJ-A2Q0-01A	THCA	4.63
TCGA-EL-A3CX-01A	THCA	6.36
TCGA-FY-A3ON-01A	THCA	6.43
TCGA-KS-A4IC-01A	THCA	5.48
TCGA-EL-A3ZO-01A	THCA	5.51
TCGA-FY-A4B3-01A	THCA	5.92
TCGA-J8-A3YF-01A	THCA	5.58
TCGA-EL-A3ZK-01A	THCA	5.2
TCGA-DE-A4MB-01A	THCA	5.66
TCGA-ET-A25L-01A	THCA	5.77
TCGA-EL-A4JW-01A	THCA	6.07
TCGA-EL-A4K6-01A	THCA	4.01
TCGA-EM-A4FU-01A	THCA	5
TCGA-ET-A2MX-01A	THCA	5.05
TCGA-EL-A3ZT-01A	THCA	5.86
TCGA-EM-A2CS-06A	THCA	6.83
TCGA-EM-A2CS-01A	THCA	7
TCGA-EL-A4K0-01A	THCA	6.32
TCGA-DJ-A13T-01A	THCA	6.25
TCGA-ET-A25R-01A	THCA	5.67
TCGA-BJ-A18Z-01A	THCA	5.83
TCGA-ET-A25P-01A	THCA	5.79
TCGA-EM-A3FK-01A	THCA	6.7
TCGA-ET-A39J-01A	THCA	6.57
TCGA-DJ-A2QC-01A	THCA	5.03
TCGA-EM-A3AO-01A	THCA	4.99
TCGA-DJ-A3V5-01A	THCA	5.82
TCGA-EL-A4JX-01A	THCA	5.05
TCGA-FK-A4UB-01A	THCA	4.62
TCGA-FE-A237-01A	THCA	5.92
TCGA-H2-A421-01A	THCA	5.17
TCGA-CE-A483-01A	THCA	5.28
TCGA-CE-A27D-01A	THCA	5.38
TCGA-ET-A4KN-01A	THCA	3.2
TCGA-IM-A3U2-01A	THCA	5.6
TCGA-FK-A3SG-01A	THCA	5.58
TCGA-EM-A3AR-01A	THCA	6.02
TCGA-EL-A3T0-01A	THCA	5.15
TCGA-BJ-A2N8-01A	THCA	5.65
TCGA-BJ-A45J-01A	THCA	6.17
TCGA-DE-A4M9-01A	THCA	6.81
TCGA-BJ-A45H-01A	THCA	6.13
TCGA-FY-A40M-01A	THCA	6.36
TCGA-FK-A3SB-01A	THCA	5.91
TCGA-FY-A40K-01A	THCA	6.4
TCGA-DE-A2OL-01A	THCA	6.3
TCGA-DJ-A13S-01A	THCA	5.17
TCGA-ET-A39K-01A	THCA	5.29
TCGA-BJ-A0Z9-01A	THCA	6.96
TCGA-EL-A4K9-01A	THCA	6.92
TCGA-IM-A41Z-01A	THCA	6.03

TCGA-DJ-A3V7-01A	THCA	5.48
TCGA-ET-A39T-01A	THCA	6.28
TCGA-E8-A417-01A	THCA	5.39
TCGA-EL-A3T6-01A	THCA	6.18
TCGA-FY-A40L-01A	THCA	5.2
TCGA-EL-A3H5-01A	THCA	4.39
TCGA-E8-A242-01A	THCA	5.94
TCGA-EM-A1CU-01A	THCA	6.72
TCGA-E3-A3DZ-01A	THCA	6.14
TCGA-ET-A25J-01A	THCA	5.55
TCGA-ET-A3BQ-01B	THCA	6.15
TCGA-EM-A4FH-01A	THCA	5.96
TCGA-FK-A3SD-01A	THCA	6.13
TCGA-EM-A3AK-01A	THCA	5.95
TCGA-J8-A42S-01A	THCA	5.97
TCGA-EL-A3D4-01A	THCA	4.76
TCGA-EL-A3CL-01A	THCA	6.59
TCGA-KS-A4I1-01A	THCA	5.91
TCGA-KS-A41I-01A	THCA	2.73
TCGA-BJ-A0ZG-01A	THCA	5.91
TCGA-FE-A3PC-01A	THCA	4.08
TCGA-BJ-A0ZE-01A	THCA	4.73
TCGA-DJ-A3V8-01A	THCA	5.88
TCGA-E8-A432-01A	THCA	6.23
TCGA-BJ-A3F0-01A	THCA	5.51
TCGA-DJ-A3UZ-01A	THCA	5.36
TCGA-DJ-A2Q2-01A	THCA	5.74
TCGA-EM-A2OX-01A	THCA	6.92
TCGA-EL-A3T7-01A	THCA	6.82
TCGA-EM-A3FO-01A	THCA	5.95
TCGA-L6-A4EU-01A	THCA	5.77
TCGA-FY-A3NN-01A	THCA	5.57
TCGA-H2-A3RI-01A	THCA	5.51
TCGA-FY-A3BL-01A	THCA	4.95
TCGA-DO-A2HM-01B	THCA	6.4
TCGA-DE-A69K-01A	THCA	6.35
TCGA-EL-A3GX-01A	THCA	6.47
TCGA-DE-A4MC-01A	THCA	5.51
TCGA-EM-A22Q-01A	THCA	4.96
TCGA-E8-A416-01A	THCA	5.63
TCGA-EL-A3CS-01A	THCA	6.49
TCGA-KS-A41F-01A	THCA	4.12
TCGA-FK-A3S3-01A	THCA	6.03
TCGA-E3-A3E1-01A	THCA	6.35
TCGA-FE-A22Z-01A	THCA	5.78
TCGA-BJ-A45K-01A	THCA	5.76
TCGA-E8-A2EA-01A	THCA	6.04
TCGA-ET-A3BP-01A	THCA	5.83
TCGA-EM-A1CV-01A	THCA	6.1
TCGA-BJ-A0Z0-01A	THCA	6.21
TCGA-BJ-A28Z-01A	THCA	5.71
TCGA-DE-A4MA-01A	THCA	4.63
TCGA-DJ-A13O-01A	THCA	5.54
TCGA-ET-A3BX-01A	THCA	7.53
TCGA-DJ-A13U-01A	THCA	5.6
TCGA-EM-A1YD-01A	THCA	5.61
TCGA-EM-A3FM-01A	THCA	5.97
TCGA-EL-A3T1-01A	THCA	5.14

TCGA-EM-A2P1-01A	THCA	5.32
TCGA-J8-A3YH-01A	THCA	6.73
TCGA-J8-A3YH-06A	THCA	6.81
TCGA-EL-A3TB-01A	THCA	5.84
TCGA-BJ-A45C-01A	THCA	5.5
TCGA-CE-A13K-01A	THCA	5.66
TCGA-DJ-A2PX-01A	THCA	5.74
TCGA-EL-A3ZS-01A	THCA	5.58
TCGA-DJ-A3UX-01A	THCA	5.87
TCGA-EM-A2P1-06A	THCA	5.58
TCGA-EM-A22M-01A	THCA	5.22
TCGA-L6-A4EP-01A	THCA	5.3
TCGA-BJ-A18Y-01A	THCA	5.82
TCGA-EL-A4K2-01A	THCA	5.84
TCGA-DJ-A4UT-01A	THCA	5.51
TCGA-DJ-A2Q3-01A	THCA	6.13
TCGA-FK-A3SE-01A	THCA	6.56
TCGA-FY-A2QD-01A	THCA	5.94
TCGA-ET-A3DR-01A	THCA	6.25
TCGA-DJ-A13M-01A	THCA	5.25
TCGA-ET-A25O-01A	THCA	5.79
TCGA-DJ-A4V4-01A	THCA	4.86
TCGA-EL-A3CZ-01A	THCA	5.93
TCGA-EM-A4FF-01A	THCA	5.51
TCGA-FY-A3R7-01A	THCA	6.02
TCGA-BJ-A4O9-01A	THCA	6.03
TCGA-EL-A3GW-01A	THCA	6.32
TCGA-DJ-A13P-01A	THCA	5.46
TCGA-EM-A3FL-01A	THCA	5.34
TCGA-EM-A2CT-01A	THCA	7.47
TCGA-IM-A3ED-01A	THCA	6.64
TCGA-KS-A4IB-01A	THCA	4.78
TCGA-DJ-A2PW-01A	THCA	6.06
TCGA-L6-A4EQ-01A	THCA	5.74
TCGA-ET-A3BO-01A	THCA	5.93
TCGA-DJ-A3VD-01A	THCA	6.02
TCGA-IM-A3U3-01A	THCA	5.91
TCGA-EM-A2CJ-01A	THCA	6.07
TCGA-DJ-A2Q1-01A	THCA	5.49
TCGA-EL-A3MW-01A	THCA	6.17
TCGA-FE-A233-01A	THCA	6.31
TCGA-BJ-A0Z2-01A	THCA	5.98
TCGA-EL-A3CV-01A	THCA	6.37
TCGA-BJ-A2NA-01A	THCA	6.2
TCGA-DJ-A13L-01A	THCA	5.88
TCGA-ET-A39L-01A	THCA	5.39
TCGA-BJ-A2N7-01A	THCA	5.18
TCGA-ET-A3BS-01A	THCA	6.11
TCGA-ET-A39O-01A	THCA	6.53
TCGA-FY-A3YR-01A	THCA	5.18
TCGA-EL-A3TA-01A	THCA	5.7
TCGA-J8-A3YE-01A	THCA	6.16
TCGA-DJ-A3UR-01A	THCA	5.63
TCGA-BJ-A0ZB-01A	THCA	5.37
TCGA-FE-A238-01A	THCA	6.25
TCGA-E8-A413-01A	THCA	5.82
TCGA-DJ-A3UK-01A	THCA	6.07
TCGA-KS-A4I7-01A	THCA	5.8

TCGA-EL-A3ZL-01A	THCA	4.53
TCGA-IM-A420-01A	THCA	5.41
TCGA-KS-A4I5-01A	THCA	5.82
TCGA-DJ-A3VE-01A	THCA	6.73
TCGA-DJ-A1QL-01A	THCA	5.97
TCGA-EM-A1YE-01A	THCA	5.72
TCGA-FE-A234-01A	THCA	6.38
TCGA-DJ-A1QH-01A	THCA	6.41
TCGA-E3-A3E2-01A	THCA	5.76
TCGA-EM-A4G1-01A	THCA	4.88
TCGA-DE-A4MD-06A	THCA	4.74
TCGA-DE-A4MD-01A	THCA	5.19
TCGA-FY-A3I4-01A	THCA	5.78
TCGA-DJ-A3VL-01A	THCA	4.85
TCGA-FE-A235-01A	THCA	4.76
TCGA-DJ-A1QG-01A	THCA	5.23
TCGA-EM-A2OY-01A	THCA	4.14
TCGA-DJ-A4UR-01A	THCA	4.31
TCGA-ET-A40S-01A	THCA	6.14
TCGA-BJ-A45I-01A	THCA	5.81
TCGA-DJ-A3UO-01A	THCA	6.13
TCGA-E8-A436-01A	THCA	5.83
TCGA-ET-A3BT-01A	THCA	6.42
TCGA-4C-A93U-01A	THCA	6.14
TCGA-EM-A3AJ-01A	THCA	6.75
TCGA-DJ-A1QE-01A	THCA	6.5
TCGA-L6-A4ET-01A	THCA	5.17
TCGA-BJ-A45F-01A	THCA	4.38
TCGA-MK-A4N6-01A	THCA	7.18
TCGA-DJ-A1QD-01A	THCA	6.14
TCGA-ET-A25N-01A	THCA	5.74
TCGA-EL-A4K7-01A	THCA	5.35
TCGA-DJ-A3VJ-01A	THCA	6.15
TCGA-EL-A3D5-01A	THCA	6.78
TCGA-H2-A26U-01A	THCA	5.91
TCGA-FY-A3R6-01A	THCA	5.63
TCGA-E8-A419-01A	THCA	5.84
TCGA-EL-A3H1-01A	THCA	5.82
TCGA-DE-A4M8-01A	THCA	5.31
TCGA-ET-A2N3-01B	THCA	5.72
TCGA-DE-A69J-01A	THCA	6.92
TCGA-EL-A3GO-01A	THCA	6.23
TCGA-EL-A4K1-01A	THCA	4.36
TCGA-EM-A2OW-01A	THCA	4.69
TCGA-DJ-A2PS-01A	THCA	6.52
TCGA-ET-A3DT-01A	THCA	5.91
TCGA-DJ-A2QA-01A	THCA	4.73
TCGA-EL-A3T8-01A	THCA	6.28
TCGA-DJ-A3VF-01A	THCA	5.55
TCGA-EL-A3CY-01A	THCA	6.31
TCGA-EL-A3GR-01A	THCA	6.95
TCGA-BJ-A192-01A	THCA	5.72
TCGA-DJ-A3VB-01A	THCA	5.69
TCGA-EM-A3FP-01A	THCA	4.78
TCGA-DJ-A2QB-01A	THCA	5.3
TCGA-DJ-A4UW-01A	THCA	5.38
TCGA-BJ-A28V-01A	THCA	6.39
TCGA-DE-A0Y3-01B	THCA	5.69

TCGA-EM-A22N-01A	THCA	4.15
TCGA-EM-A3FJ-01A	THCA	5.42
TCGA-DJ-A3VG-01A	THCA	4.92
TCGA-BJ-A291-01A	THCA	4.72
TCGA-CE-A3ME-01A	THCA	6.42
TCGA-DJ-A3UM-01A	THCA	5.87
TCGA-E3-A3DY-01A	THCA	6.18
TCGA-FY-A3R9-01A	THCA	4.43
TCGA-EL-A3GP-01A	THCA	6
TCGA-EL-A3GV-01A	THCA	6.11
TCGA-EM-A22I-01A	THCA	5.48
TCGA-DJ-A3UV-01A	THCA	5.66
TCGA-DJ-A1QF-01A	THCA	5.46
TCGA-BJ-A45D-01A	THCA	5.29
TCGA-EL-A3ZP-01A	THCA	5.56
TCGA-E3-A3E5-01A	THCA	6.25
TCGA-BJ-A28T-01A	THCA	5.32
TCGA-DJ-A3UT-01A	THCA	4.81
TCGA-CE-A482-01A	THCA	5.89
TCGA-E8-A44K-01A	THCA	4.82
TCGA-DJ-A2Q6-01A	THCA	6.12
TCGA-EM-A22L-01A	THCA	5.52
TCGA-EM-A3SY-01A	THCA	4.11
TCGA-EM-A2P2-01A	THCA	6.9
TCGA-ET-A3BV-01A	THCA	6.25
TCGA-EL-A3D1-01A	THCA	5.59
TCGA-BJ-A28X-01A	THCA	5.84
TCGA-EM-A1YB-01A	THCA	4.43
TCGA-DJ-A3V0-01A	THCA	5.12
TCGA-EM-A22J-01A	THCA	5.67
TCGA-EM-A2P3-01A	THCA	5.93
TCGA-ET-A3DU-01A	THCA	5.07
TCGA-EL-A4KG-01A	THCA	6.18
TCGA-EM-A3ST-01A	THCA	5.81
TCGA-ET-A39S-01A	THCA	6.33
TCGA-DJ-A2PO-01A	THCA	6.22
TCGA-ET-A25G-01A	THCA	6.43
TCGA-EL-A3H4-01A	THCA	6.22
TCGA-EL-A3GU-01A	THCA	5.62
TCGA-EM-A1CS-01A	THCA	5.91
TCGA-DJ-A2PQ-01A	THCA	6.15
TCGA-EM-A4FO-01A	THCA	5.14
TCGA-BJ-A0YZ-01A	THCA	6.45
TCGA-BJ-A28W-01A	THCA	3.94
TCGA-FE-A230-01A	THCA	5.48
TCGA-EL-A3ZR-01A	THCA	5.7
TCGA-ET-A39R-01A	THCA	6.13
TCGA-EM-A3O8-01A	THCA	5.67
TCGA-DO-A1K0-01A	THCA	5.65
TCGA-ET-A3DV-01A	THCA	5.83
TCGA-EL-A3H2-01A	THCA	6.23
TCGA-ET-A40Q-01A	THCA	5.72
TCGA-DJ-A4V5-01A	THCA	6.85
TCGA-ET-A39N-01A	THCA	6.76
TCGA-BJ-A0ZF-01A	THCA	6.61
TCGA-EL-A3T9-01A	THCA	6.24
TCGA-EL-A3T2-01A	THCA	5.52
TCGA-EM-A3OB-01A	THCA	5.66

TCGA-DJ-A2Q7-01A	THCA	6.02
TCGA-DJ-A3VK-01A	THCA	5.9
TCGA-DJ-A13V-01A	THCA	6.46
TCGA-DJ-A1QQ-01A	THCA	6.61
TCGA-J8-A3YG-01A	THCA	5.78
TCGA-BJ-A3EZ-01A	THCA	6.05
TCGA-QD-A8IV-01A	THCA	5.55
TCGA-E8-A415-01A	THCA	5.68
TCGA-BJ-A45G-01A	THCA	5.23
TCGA-EL-A4KD-01A	THCA	5.44
TCGA-FY-A3R8-01A	THCA	6.24
TCGA-DJ-A4V2-01A	THCA	6.2
TCGA-CE-A484-01A	THCA	5.16
TCGA-EM-A22K-01A	THCA	5.78
TCGA-FK-A3SH-01A	THCA	6.11
TCGA-ET-A39M-01A	THCA	5.16
TCGA-EL-A3MX-01A	THCA	5.47
TCGA-E8-A3X7-01A	THCA	5.24
TCGA-FE-A239-01A	THCA	5.8
TCGA-J8-A3YD-01A	THCA	5.63
TCGA-J8-A3NZ-01A	THCA	6.26
TCGA-ET-A39P-01A	THCA	5.54
TCGA-EL-A4JZ-01A	THCA	6.23
TCGA-EL-A3CW-01A	THCA	5.51
TCGA-FY-A3NP-01A	THCA	5.68
TCGA-H2-A3RH-01A	THCA	6
TCGA-EM-A1YA-01A	THCA	5.16
TCGA-J8-A3O2-06A	THCA	6.86
TCGA-EM-A2CN-01A	THCA	5.11
TCGA-BJ-A28R-01A	THCA	5.64
TCGA-EM-A3AI-01A	THCA	5.92
TCGA-EM-A22O-01A	THCA	6.39
TCGA-BJ-A0ZC-01A	THCA	6.38
TCGA-DE-A0Y2-01A	THCA	5.48
TCGA-FY-A4B0-01A	THCA	6.26
TCGA-DJ-A1QN-01A	THCA	6.33
TCGA-EL-A3D6-01A	THCA	6.14
TCGA-ET-A2N0-01A	THCA	5.01
TCGA-EM-A3FR-01A	THCA	5.74
TCGA-ET-A4KQ-01A	THCA	5.46
TCGA-ET-A3BW-01A	THCA	5.59
TCGA-EL-A3ZG-01A	THCA	5.66
TCGA-EL-A3N2-01A	THCA	6.19
TCGA-J8-A4HY-01A	THCA	4.99
TCGA-EM-A3OA-01A	THCA	6.6
TCGA-DE-A3KN-01A	THCA	5.74
TCGA-EM-A3AL-01A	THCA	6.1
TCGA-EM-A3AP-01A	THCA	6.46
TCGA-ET-A3DO-01A	THCA	5.87
TCGA-EM-A4FK-01A	THCA	7.2
TCGA-DJ-A2PU-01A	THCA	6.26
TCGA-BJ-A0Z5-01A	THCA	5.51
TCGA-ET-A25I-01A	THCA	4.47
TCGA-DJ-A2Q5-01A	THCA	5.87
TCGA-EM-A3SX-01A	THCA	6.04
TCGA-EM-A2OZ-01A	THCA	6.69
TCGA-BJ-A191-01A	THCA	6.16
TCGA-ET-A40R-01A	THCA	5.32

TCGA-DJ-A4UP-01A	THCA	5.41
TCGA-CE-A481-01A	THCA	5.49
TCGA-DJ-A3UP-01A	THCA	6.02
TCGA-EL-A3GZ-01A	THCA	7
TCGA-EM-A3O9-01A	THCA	4.51
TCGA-EL-A3N3-01A	THCA	5.79
TCGA-EM-A3O6-01A	THCA	5.84
TCGA-EM-A2CP-01A	THCA	5.61
TCGA-ET-A25K-01A	THCA	5.61
TCGA-EL-A3H3-01A	THCA	5.91
TCGA-DJ-A1QM-01A	THCA	5.95
TCGA-DJ-A2PT-01A	THCA	6.01
TCGA-BJ-A290-01A	THCA	7.29
TCGA-EM-A3O7-01A	THCA	5.57
TCGA-ET-A3DW-01A	THCA	6.22
TCGA-KS-A4I9-01A	THCA	5.51
TCGA-IM-A41Y-01A	THCA	5.86
TCGA-FE-A232-01A	THCA	5.51
TCGA-ET-A2MZ-01A	THCA	6.62
TCGA-DJ-A3UU-01A	THCA	5.9
TCGA-ET-A3BU-01A	THCA	6.3
TCGA-J8-A3O2-01A	THCA	6.75
TCGA-EL-A3D0-01A	THCA	5.96
TCGA-FY-A3I5-01B	THCA	5.34
TCGA-EM-A3AN-01A	THCA	6.35
TCGA-DJ-A2PZ-01A	THCA	5.56
TCGA-E3-A3E3-01A	THCA	6.18
TCGA-DJ-A4UQ-01A	THCA	5.99
TCGA-BJ-A3PT-01A	THCA	4.87
TCGA-KS-A41J-01A	THCA	5.55
TCGA-DJ-A3UQ-01A	THCA	6.12
TCGA-E8-A414-01A	THCA	5.99
TCGA-IM-A4EB-01A	THCA	5.35
TCGA-EL-A3GS-01A	THCA	6.06
TCGA-DJ-A1QI-01A	THCA	6.53
TCGA-ET-A3BN-01A	THCA	5.97
TCGA-FE-A236-01A	THCA	6.17
TCGA-E8-A44M-01A	THCA	5.63
TCGA-EM-A3O3-01A	THCA	5.25
TCGA-EM-A2CM-01A	THCA	4.62
TCGA-ET-A39I-01A	THCA	5.89
TCGA-EL-A3CP-01A	THCA	6.06
TCGA-FE-A3PD-01A	THCA	5.73
TCGA-ET-A2N4-01A	THCA	6.07
TCGA-FY-A40N-01A	THCA	5.21
TCGA-DJ-A13R-01A	THCA	3.37
TCGA-EL-A4K4-01A	THCA	6.43
TCGA-DJ-A2PR-01A	THCA	6.13
TCGA-BJ-A0Z3-01A	THCA	5.65
TCGA-BJ-A45E-01A	THCA	4.01
TCGA-DJ-A13X-01A	THCA	6.19
TCGA-DJ-A13W-01A	THCA	2.76
TCGA-MK-A4N9-01A	THCA	5.84
TCGA-EM-A2CL-01A	THCA	5.87
TCGA-DJ-A3VA-01A	THCA	5.88
TCGA-EL-A3CR-01A	THCA	5.54
TCGA-H2-A2K9-01A	THCA	6.05
TCGA-EM-A1CT-01A	THCA	5.36

TCGA-ET-A40T-01A	THCA	6.02
TCGA-DE-A7U5-01A	THCA	6.02
TCGA-EL-A3ZQ-01A	THCA	4.16
TCGA-DJ-A3V9-01A	THCA	6.3
TCGA-DJ-A3V4-01A	THCA	5.91
TCGA-EM-A3SZ-01A	THCA	6.03
TCGA-FE-A3PA-01A	THCA	5.07
TCGA-DO-A1JZ-01A	THCA	5.94
TCGA-EL-A3ZH-01A	THCA	5.47
TCGA-ET-A3DP-01A	THCA	6.25
TCGA-EM-A2CR-01A	THCA	5.11
TCGA-MK-A84Z-01A	THCA	5.17
TCGA-EM-A22P-01A	THCA	6.09
TCGA-EL-A4KI-01A	THCA	5.03
TCGA-E8-A2JQ-01A	THCA	5.92
TCGA-FY-A3NM-01A	THCA	5.09
TCGA-EL-A3MZ-01A	THCA	5.79
TCGA-EM-A4FN-01A	THCA	4.98
TCGA-ET-A3DQ-01A	THCA	5.35
TCGA-BJ-A3PR-01A	THCA	5.31
TCGA-EM-A3FQ-01A	THCA	6.31
TCGA-ET-A2MY-01A	THCA	5.72
TCGA-E8-A418-01A	THCA	5.92
TCGA-DJ-A4V0-01A	THCA	5.17
TCGA-DJ-A2PY-01A	THCA	6.33
TCGA-J8-A4HW-06A	THCA	6.6
TCGA-KS-A4I3-01A	THCA	6.14
TCGA-EL-A3H7-01A	THCA	5.83
TCGA-BJ-A2P4-01A	THCA	4.86
TCGA-J8-A4HW-01A	THCA	6.54
TCGA-EL-A3CO-01A	THCA	5.83
TCGA-FE-A23A-01A	THCA	4.88
TCGA-EM-A3SU-06A	THCA	6.05
TCGA-EM-A3SU-01A	THCA	5.17
TCGA-E8-A434-01A	THCA	5.89
TCGA-DJ-A2Q4-01A	THCA	6.66
TCGA-EL-A3H8-01A	THCA	6.52
TCGA-MK-A4N7-01A	THCA	6.54
TCGA-EM-A3AQ-01A	THCA	5
TCGA-DJ-A1QO-01A	THCA	6.06
TCGA-DJ-A2PN-01A	THCA	6.42
TCGA-EM-A2OV-01A	THCA	5.08
TCGA-DJ-A2PV-01A	THCA	6.15
TCGA-E3-A3E0-01A	THCA	6.08
TCGA-EL-A3T3-01A	THCA	5.77
TCGA-FY-A4B4-01A	THCA	6.15
TCGA-FY-A3RA-01A	THCA	6.45
TCGA-FY-A3WA-01A	THCA	5.66
TCGA-KS-A41L-01A	THCA	5.95
TCGA-E8-A433-01A	THCA	5.04
TCGA-EL-A4JV-01A	THCA	5.18
TCGA-DJ-A3US-01A	THCA	5.94
TCGA-H2-A422-01A	THCA	5.54
TCGA-CE-A485-01A	THCA	5.86
TCGA-EM-A1YC-01A	THCA	4.89
TCGA-EM-A4FM-01A	THCA	5.31
TCGA-EL-A3GY-01A	THCA	6.45
TCGA-CE-A3MD-01A	THCA	5.65

TCGA-BJ-A4O8-01A	THCA	5.94
TCGA-EM-A3FQ-06A	THCA	4.2
TCGA-E8-A438-01A	THCA	4.47
TCGA-J8-A3O1-01A	THCA	5.98
TCGA-X7-A8D7-01A	THYM	4.62
TCGA-XM-A8RH-01A	THYM	4.7
TCGA-X7-A8DE-01A	THYM	4.73
TCGA-ZB-A96G-01A	THYM	4.13
TCGA-5G-A9ZZ-01A	THYM	6.23
TCGA-4V-A9QR-01A	THYM	3.64
TCGA-4V-A9QU-01A	THYM	4.53
TCGA-XM-A8RF-01A	THYM	5.41
TCGA-3T-AA9L-01A	THYM	4.58
TCGA-4X-A9F9-01A	THYM	3.22
TCGA-ZB-A96M-01A	THYM	5.4
TCGA-XU-AAXW-01A	THYM	5.22
TCGA-3G-AB0Q-01A	THYM	4.7
TCGA-ZT-A8OM-01A	THYM	2.9
TCGA-ZB-A96H-01A	THYM	5.44
TCGA-ZB-A961-01A	THYM	5.39
TCGA-5U-AB0F-01A	THYM	3.02
TCGA-X7-A8DB-01A	THYM	4
TCGA-XM-AAZ1-01A	THYM	4.25
TCGA-XM-A8RE-01A	THYM	4.2
TCGA-X7-A8DG-01A	THYM	4.13
TCGA-3G-AB14-01A	THYM	5.28
TCGA-4X-A9FA-01A	THYM	4.33
TCGA-ZB-A96C-01A	THYM	5.43
TCGA-XH-A853-01A	THYM	4.79
TCGA-ZB-A96D-01A	THYM	5.06
TCGA-YT-A95F-01A	THYM	5.22
TCGA-X7-A8D9-01A	THYM	5.22
TCGA-XU-A92O-01A	THYM	4.35
TCGA-X7-A8M8-01A	THYM	4.35
TCGA-5V-A9RR-01A	THYM	3.69
TCGA-4X-A9FC-01A	THYM	5
TCGA-XU-A92W-01A	THYM	5.01
TCGA-ZB-A96P-01A	THYM	4.95
TCGA-ZC-AAAF-01A	THYM	4.17
TCGA-4V-A9QI-01A	THYM	4.27
TCGA-ZB-A96Q-01A	THYM	5.59
TCGA-4V-A9QX-01A	THYM	4.61
TCGA-ZB-A96F-01A	THYM	4.52
TCGA-X7-A8M1-01A	THYM	5.24
TCGA-X7-A8M4-01A	THYM	4.31
TCGA-XM-AAZ3-01A	THYM	5.3
TCGA-ZB-A964-01A	THYM	5.58
TCGA-ZL-A9V6-01A	THYM	5.44
TCGA-ZB-A965-01A	THYM	5.23
TCGA-XU-AAY1-01A	THYM	3.79
TCGA-4V-A9QT-01A	THYM	4.71
TCGA-X7-A8M7-01A	THYM	3.5
TCGA-XU-AAXZ-01A	THYM	1.76
TCGA-XM-AAZ2-01A	THYM	5.61
TCGA-X7-A8M0-01A	THYM	5.13
TCGA-XM-A8R9-01A	THYM	4.56
TCGA-X7-A8M5-01A	THYM	4.02
TCGA-XM-A8R8-01A	THYM	4.66

TCGA-YT-A95H-01A	THYM	4.76
TCGA-ZB-A966-01A	THYM	6.25
TCGA-ZC-AAAH-01A	THYM	3.76
TCGA-4V-A9QN-01A	THYM	4.78
TCGA-3G-AB0O-01A	THYM	4.3
TCGA-ZB-A96V-01A	THYM	6.06
TCGA-4V-A9QJ-01A	THYM	4.36
TCGA-XM-A8RC-01A	THYM	4.94
TCGA-4V-A9QW-01A	THYM	3.13
TCGA-X7-A8DD-01A	THYM	3.26
TCGA-XM-A8RL-01A	THYM	4.35
TCGA-XU-A92T-01A	THYM	5.7
TCGA-ZB-A96A-01A	THYM	4.6
TCGA-XU-A92U-01A	THYM	4.69
TCGA-3Q-A9WF-01A	THYM	4.45
TCGA-XM-A8RG-01A	THYM	5.58
TCGA-XU-A92Y-01A	THYM	4.59
TCGA-ZB-A96L-01A	THYM	5.79
TCGA-XU-A92X-01A	THYM	3.76
TCGA-X7-A8DJ-01A	THYM	4.83
TCGA-ZB-A962-01A	THYM	5.76
TCGA-XM-A8RB-01A	THYM	5.04
TCGA-XU-A930-01A	THYM	5.43
TCGA-XU-A932-01A	THYM	4.47
TCGA-4X-A9FD-01A	THYM	5.32
TCGA-3S-AAYX-01A	THYM	5.18
TCGA-ZB-A96B-01A	THYM	4.28
TCGA-XU-A92V-01A	THYM	5.26
TCGA-ZC-AAAA-01A	THYM	4.11
TCGA-X7-A8M3-01A	THYM	4.41
TCGA-XU-AAXY-01A	THYM	3.51
TCGA-XU-AAXX-01A	THYM	5.03
TCGA-X7-A8M6-01A	THYM	3.25
TCGA-XM-A8RI-01A	THYM	5.05
TCGA-5K-AAAP-01A	THYM	4.42
TCGA-YT-A95G-01A	THYM	5.87
TCGA-XU-AAY0-01A	THYM	3.68
TCGA-ZC-AAA7-01A	THYM	5.3
TCGA-YT-A95D-01A	THYM	5.85
TCGA-5U-AB0D-01A	THYM	3.71
TCGA-3S-A8YW-01A	THYM	5.88
TCGA-XU-A936-01A	THYM	5.37
TCGA-5U-AB0E-01A	THYM	3.79
TCGA-4X-A9FB-01A	THYM	4.33
TCGA-X7-A8D6-01A	THYM	4.95
TCGA-4V-A9QM-01A	THYM	5.17
TCGA-ZB-A96K-01A	THYM	5.24
TCGA-3G-AB0T-01A	THYM	3.43
TCGA-ZB-A96O-01A	THYM	3.84
TCGA-X7-A8D8-01A	THYM	5.12
TCGA-XM-A8RD-01A	THYM	4.95
TCGA-ZB-A96R-01A	THYM	3.98
TCGA-XU-AAXV-01A	THYM	5.42
TCGA-X7-A8DF-01A	THYM	4.25
TCGA-XU-A931-01A	THYM	5.08
TCGA-3G-AB19-01A	THYM	5.17
TCGA-ZB-A96E-01A	THYM	5.02
TCGA-XU-A92Z-01A	THYM	5.65

TCGA-ZB-A96I-01A	THYM	5.12
TCGA-XU-A933-01A	THYM	6.63
TCGA-4V-A9QS-01A	THYM	4.9
TCGA-YT-A95E-01A	THYM	4.45
TCGA-4V-A9QL-01A	THYM	4.74
TCGA-ZB-A969-01A	THYM	6.27
TCGA-XU-A92Q-01A	THYM	4.88
TCGA-ZB-A963-01A	THYM	5.07
TCGA-D1-A17H-01A	UCEC	7.22
TCGA-D1-A176-01A	UCEC	6.7
TCGA-AX-A3G8-01A	UCEC	6.31
TCGA-AX-A3FX-01A	UCEC	5.23
TCGA-D1-A17C-01A	UCEC	4.98
TCGA-D1-A2G0-01A	UCEC	6.51
TCGA-AX-A2HK-01A	UCEC	6.46
TCGA-AJ-A23O-01A	UCEC	7.26
TCGA-EY-A1GR-01A	UCEC	6.26
TCGA-AJ-A3NH-01A	UCEC	7.03
TCGA-EO-A1Y7-01A	UCEC	6.77
TCGA-AX-A3G7-01A	UCEC	6.39
TCGA-A5-A0GA-01A	UCEC	6.57
TCGA-AP-A056-01A	UCEC	7.31
TCGA-AJ-A23N-01A	UCEC	6.68
TCGA-B5-A0JX-01A	UCEC	5.73
TCGA-BG-A0MO-01A	UCEC	7.86
TCGA-EO-A3KW-01A	UCEC	7.65
TCGA-AP-A0LV-01A	UCEC	6.07
TCGA-D1-A16F-01A	UCEC	6.43
TCGA-AP-A0LF-01A	UCEC	3.28
TCGA-KP-A3W4-01A	UCEC	6.87
TCGA-AJ-A3BG-01A	UCEC	7.84
TCGA-A5-A0GR-01A	UCEC	3.89
TCGA-AP-A0LH-01A	UCEC	7.23
TCGA-D1-A3DG-01A	UCEC	5.39
TCGA-AX-A2IO-01A	UCEC	4.61
TCGA-A5-AB3J-01A	UCEC	6.99
TCGA-B5-A0JU-01B	UCEC	6.8
TCGA-A5-A0G3-01A	UCEC	4.96
TCGA-AX-A2HG-01A	UCEC	6.12
TCGA-EY-A1GQ-01A	UCEC	6.98
TCGA-DF-A2KU-01A	UCEC	5.63
TCGA-BG-A0MG-01A	UCEC	5.84
TCGA-BK-A0C9-01A	UCEC	6.98
TCGA-EY-A72D-01A	UCEC	7.06
TCGA-AX-A2H2-01A	UCEC	5.84
TCGA-D1-A16E-01A	UCEC	6.59
TCGA-E6-A8L9-01A	UCEC	6.05
TCGA-B5-A3FC-01A	UCEC	6.11
TCGA-AX-A0J1-01A	UCEC	8.9
TCGA-BG-A0VZ-01A	UCEC	6.27
TCGA-DI-A2QT-01A	UCEC	5.38
TCGA-B5-A3FD-01A	UCEC	6.22
TCGA-AX-A063-01A	UCEC	7.47
TCGA-D1-A17T-01A	UCEC	5.75
TCGA-D1-A177-01A	UCEC	9.42
TCGA-EY-A210-01A	UCEC	6.81
TCGA-EC-A1QX-01A	UCEC	5.76
TCGA-BG-A2AE-01A	UCEC	6.66

TCGA-D1-A1NZ-01A	UCEC	7.99
TCGA-AX-A06L-01A	UCEC	4.8
TCGA-D1-A15V-01A	UCEC	6.57
TCGA-D1-A17B-01A	UCEC	6.09
TCGA-EY-A1GW-01A	UCEC	4.54
TCGA-D1-A102-01A	UCEC	6.62
TCGA-BS-A0U8-01A	UCEC	7.18
TCGA-AX-A1CI-01A	UCEC	5.94
TCGA-BG-A0MA-01A	UCEC	5.69
TCGA-D1-A174-01A	UCEC	5.53
TCGA-H5-A2HR-01A	UCEC	5.23
TCGA-EY-A4KR-01A	UCEC	5.88
TCGA-AX-A0IW-01A	UCEC	6.29
TCGA-PG-A914-01A	UCEC	5.44
TCGA-B5-A11O-01A	UCEC	6.3
TCGA-AP-A0L8-01A	UCEC	6.76
TCGA-D1-A16O-01A	UCEC	8.22
TCGA-BG-A0MU-01A	UCEC	6.68
TCGA-A5-A7WJ-01A	UCEC	9.73
TCGA-EY-A1GH-01A	UCEC	8.57
TCGA-4E-A92E-01A	UCEC	7.52
TCGA-B5-A0K4-01A	UCEC	5.02
TCGA-AP-A052-01A	UCEC	6.06
TCGA-FI-A2CY-01A	UCEC	6.58
TCGA-B5-A1MS-01B	UCEC	5.65
TCGA-BG-A0MH-01A	UCEC	6.43
TCGA-BK-A26L-01A	UCEC	7.06
TCGA-D1-A0ZO-01A	UCEC	8.03
TCGA-D1-A16B-01A	UCEC	6.48
TCGA-AP-A0LS-01A	UCEC	7.99
TCGA-D1-A15Z-01A	UCEC	6.5
TCGA-A5-A2K5-01A	UCEC	6.5
TCGA-AP-A1DM-01A	UCEC	8.27
TCGA-E6-A2P8-01A	UCEC	6.69
TCGA-D1-A169-01A	UCEC	6.6
TCGA-EO-A22S-01A	UCEC	8.68
TCGA-B5-A0JR-01A	UCEC	9.01
TCGA-AX-A3FS-01A	UCEC	6.54
TCGA-D1-A1NS-01A	UCEC	6.12
TCGA-AX-A1CJ-01A	UCEC	4.91
TCGA-AX-A2HD-01A	UCEC	6.3
TCGA-A5-A0RA-01A	UCEC	7.59
TCGA-AP-A0LJ-01A	UCEC	5.09
TCGA-EC-A24G-01A	UCEC	7.47
TCGA-AP-A051-01A	UCEC	8.43
TCGA-BS-A0TA-01A	UCEC	5.89
TCGA-BS-A0UV-01A	UCEC	6.99
TCGA-EY-A2ON-01A	UCEC	6.72
TCGA-EO-A2CG-01A	UCEC	6.1
TCGA-EC-A1NJ-01A	UCEC	6.74
TCGA-AX-A1C8-01A	UCEC	5.08
TCGA-AJ-A5DW-01A	UCEC	7.41
TCGA-AX-A1CC-01A	UCEC	5.99
TCGA-A5-A2K4-01A	UCEC	5.76
TCGA-SL-A6J9-01A	UCEC	5.92
TCGA-AP-A0LL-01A	UCEC	3.72
TCGA-A5-A0GQ-01A	UCEC	5.97
TCGA-B5-A11Z-01A	UCEC	7.83

TCGA-D1-A15W-01A	UCEC	7.23
TCGA-D1-A0ZU-01A	UCEC	7.38
TCGA-DI-A1BY-01A	UCEC	4.82
TCGA-AP-A054-01A	UCEC	6.6
TCGA-BG-A0MS-01A	UCEC	6.38
TCGA-BG-A0LX-01A	UCEC	7.66
TCGA-AJ-A3BH-01A	UCEC	6.57
TCGA-EY-A1GX-01A	UCEC	5.75
TCGA-B5-A0JN-01A	UCEC	7.28
TCGA-BS-A0V6-01A	UCEC	6.33
TCGA-PG-A917-01A	UCEC	6.38
TCGA-BG-A0MI-01A	UCEC	6.26
TCGA-B5-A11G-01A	UCEC	5.07
TCGA-KP-A3W0-01A	UCEC	6.24
TCGA-B5-A11S-01A	UCEC	7.33
TCGA-BS-A0U5-01A	UCEC	6.62
TCGA-EY-A1H0-01A	UCEC	6.04
TCGA-DI-A1NO-01A	UCEC	6.59
TCGA-AP-A05A-01A	UCEC	4.19
TCGA-EY-A1GL-01A	UCEC	5.17
TCGA-B5-A0JT-01A	UCEC	7.76
TCGA-AJ-A3NF-01A	UCEC	6.14
TCGA-BG-A0VT-01A	UCEC	6.15
TCGA-EY-A1G8-01A	UCEC	5.9
TCGA-D1-A17N-01A	UCEC	4.34
TCGA-BG-A0M6-01A	UCEC	6.35
TCGA-BK-A0CC-01A	UCEC	4.9
TCGA-D1-A2G5-01A	UCEC	6.8
TCGA-D1-A1O7-01A	UCEC	6.91
TCGA-AP-A05J-01A	UCEC	4.59
TCGA-EY-A214-01A	UCEC	8.47
TCGA-B5-A11J-01A	UCEC	8
TCGA-DI-A0WH-01A	UCEC	4.7
TCGA-AJ-A6NU-01A	UCEC	7.15
TCGA-B5-A0K9-01A	UCEC	7.94
TCGA-EO-A3L0-01A	UCEC	6.54
TCGA-AW-A1PO-01A	UCEC	5.87
TCGA-AP-A1E3-01A	UCEC	6.5
TCGA-D1-A3JP-01A	UCEC	7.91
TCGA-AP-A1DK-01A	UCEC	6.29
TCGA-AJ-A8CV-01A	UCEC	7.25
TCGA-AX-A1C4-01A	UCEC	8.01
TCGA-AX-A3FZ-01A	UCEC	6.11
TCGA-D1-A16I-01A	UCEC	5.09
TCGA-DF-A2KY-01A	UCEC	5.58
TCGA-B5-A0K6-01A	UCEC	5.4
TCGA-EY-A548-01A	UCEC	6.06
TCGA-BS-A0TE-01A	UCEC	7.93
TCGA-EO-A2CH-01A	UCEC	5.84
TCGA-EO-A22X-01A	UCEC	5.94
TCGA-AP-A1E4-01A	UCEC	5.53
TCGA-D1-A0ZR-01A	UCEC	6.83
TCGA-AJ-A3EK-01A	UCEC	8.71
TCGA-B5-A1MV-01A	UCEC	6.3
TCGA-AX-A3G3-01A	UCEC	5.58
TCGA-5B-A90C-01A	UCEC	8.13
TCGA-AX-A1CF-01A	UCEC	7
TCGA-D1-A16G-01A	UCEC	7.09

TCGA-D1-A160-01A	UCEC	7.39
TCGA-AX-A05T-01A	UCEC	6.57
TCGA-BK-A0CC-01B	UCEC	4.61
TCGA-SJ-A6ZI-01A	UCEC	6.92
TCGA-KP-A3W3-01A	UCEC	6.55
TCGA-D1-A168-01A	UCEC	5.2
TCGA-B5-A1MR-01A	UCEC	4.91
TCGA-DI-A1C3-01A	UCEC	4.54
TCGA-BK-A0CA-01A	UCEC	7
TCGA-FI-A2EU-01A	UCEC	6.88
TCGA-AP-A059-01A	UCEC	6.32
TCGA-D1-A16R-01A	UCEC	6.88
TCGA-AP-A05O-01A	UCEC	6.44
TCGA-AX-A1CK-01A	UCEC	6.5
TCGA-BG-A2AD-01A	UCEC	8.26
TCGA-BS-A0T9-01A	UCEC	5.32
TCGA-FI-A2D2-01A	UCEC	7.22
TCGA-DF-A2KS-01A	UCEC	5.94
TCGA-PG-A916-01A	UCEC	6.31
TCGA-B5-A0JY-01A	UCEC	6.52
TCGA-D1-A16D-01A	UCEC	6.47
TCGA-AX-A1CP-01A	UCEC	6.77
TCGA-BS-A0UT-01A	UCEC	4.77
TCGA-B5-A1MW-01A	UCEC	8.08
TCGA-AX-A3G6-01A	UCEC	6.12
TCGA-E6-A1M0-01A	UCEC	6.82
TCGA-EY-A1GV-01A	UCEC	4.75
TCGA-BS-A0V4-01A	UCEC	4.95
TCGA-EY-A1GC-01A	UCEC	5.61
TCGA-BK-A13B-01A	UCEC	8.25
TCGA-BG-A221-01A	UCEC	6.86
TCGA-AP-A0LP-01A	UCEC	6.17
TCGA-PG-A6IB-01A	UCEC	6.34
TCGA-B5-A11R-01A	UCEC	6.51
TCGA-DI-A2QU-01A	UCEC	5.63
TCGA-D1-A1O5-01A	UCEC	5.78
TCGA-D1-A175-01A	UCEC	8.65
TCGA-PG-A915-01A	UCEC	5.53
TCGA-AX-A06F-01A	UCEC	7.09
TCGA-BG-A0RY-01A	UCEC	6.41
TCGA-B5-A11W-01A	UCEC	7.45
TCGA-A5-A2K7-01A	UCEC	5.87
TCGA-B5-A11I-01A	UCEC	7.85
TCGA-QF-A5YT-01A	UCEC	5.69
TCGA-B5-A1MU-01A	UCEC	5.79
TCGA-B5-A11X-01A	UCEC	6.04
TCGA-D1-A1NU-01A	UCEC	6.6
TCGA-EY-A2OP-01A	UCEC	7
TCGA-BS-A0TJ-01A	UCEC	6.07
TCGA-AJ-A3I9-01A	UCEC	7.37
TCGA-B5-A0KB-01B	UCEC	6.78
TCGA-AP-A05D-01A	UCEC	4.72
TCGA-SJ-A6ZJ-01A	UCEC	5.36
TCGA-B5-A1N2-01A	UCEC	7.18
TCGA-BG-A0YV-01A	UCEC	6.87
TCGA-AP-A05H-01A	UCEC	5.45
TCGA-BS-A0TI-01A	UCEC	6.35
TCGA-BG-A18A-01A	UCEC	5.54

TCGA-FI-A2CX-01A	UCEC	6.61
TCGA-BG-A0M8-01A	UCEC	4.75
TCGA-BS-A0TD-01A	UCEC	6.39
TCGA-BS-A0WQ-01A	UCEC	6.18
TCGA-EY-A1GT-01A	UCEC	6.48
TCGA-AJ-A2QO-01A	UCEC	9.26
TCGA-BG-A0MT-01A	UCEC	7.26
TCGA-EY-A1GO-01A	UCEC	6.98
TCGA-FI-A2EW-01A	UCEC	3.9
TCGA-DI-A1NN-01A	UCEC	6.36
TCGA-A5-A0GJ-01A	UCEC	5.45
TCGA-BG-A0YU-01A	UCEC	6.88
TCGA-2E-A9G8-01A	UCEC	6.65
TCGA-AX-A2H7-01A	UCEC	5.78
TCGA-BS-A0UA-01A	UCEC	8.08
TCGA-EY-A1GE-01A	UCEC	6.4
TCGA-A5-A0GN-01A	UCEC	5.15
TCGA-AP-A5FX-01A	UCEC	6.45
TCGA-QS-A744-01A	UCEC	6.49
TCGA-A5-A1OH-01A	UCEC	6.51
TCGA-AJ-A2QL-01A	UCEC	6.86
TCGA-AX-A06D-01A	UCEC	6.18
TCGA-A5-A0GE-01A	UCEC	5.88
TCGA-A5-A0GG-01A	UCEC	7.9
TCGA-AP-A053-01A	UCEC	6.87
TCGA-B5-A11Q-01A	UCEC	7.67
TCGA-BS-A0UM-01A	UCEC	6.97
TCGA-B5-A3FA-01A	UCEC	6.38
TCGA-AP-A1DQ-01A	UCEC	6.16
TCGA-AX-A06J-01A	UCEC	5
TCGA-A5-A0GU-01A	UCEC	7.09
TCGA-B5-A5OC-01A	UCEC	9.12
TCGA-BK-A26L-01C	UCEC	6.35
TCGA-AJ-A3NC-01A	UCEC	7.33
TCGA-BK-A13C-01A	UCEC	6.12
TCGA-EO-A3AV-01A	UCEC	6.01
TCGA-EY-A1GS-01A	UCEC	5.87
TCGA-AJ-A23M-01A	UCEC	5.49
TCGA-D1-A101-01A	UCEC	6.44
TCGA-A5-A2K3-01A	UCEC	4.41
TCGA-B5-A0JZ-01A	UCEC	8.41
TCGA-D1-A17Q-01A	UCEC	7.6
TCGA-AX-A05Y-01A	UCEC	5.03
TCGA-B5-A11P-01B	UCEC	5.95
TCGA-AX-A2H5-01A	UCEC	5.39
TCGA-B5-A5OE-01A	UCEC	5.91
TCGA-K6-A3WQ-01A	UCEC	6.88
TCGA-D1-A1NW-01A	UCEC	5.53
TCGA-D1-A1NX-01A	UCEC	5.68
TCGA-AP-A0LI-01A	UCEC	4.18
TCGA-EY-A1GK-01A	UCEC	7.68
TCGA-BG-A0M2-01A	UCEC	6.78
TCGA-A5-A0GM-01A	UCEC	5.44
TCGA-BG-A3EW-01A	UCEC	7.6
TCGA-BK-A6W4-01A	UCEC	7.1
TCGA-D1-A165-01A	UCEC	6.6
TCGA-D1-A163-01A	UCEC	7.55
TCGA-A5-A3LP-01A	UCEC	8.04

TCGA-AX-A3G1-01A	UCEC	6.71
TCGA-AX-A2H4-01A	UCEC	4.33
TCGA-D1-A0ZP-01A	UCEC	7.56
TCGA-AP-A1DP-01A	UCEC	7.72
TCGA-D1-A1O0-01A	UCEC	6.26
TCGA-B5-A0K2-01A	UCEC	7.4
TCGA-AX-A3GB-01A	UCEC	5.18
TCGA-AJ-A3BD-01A	UCEC	5.37
TCGA-AX-A05Z-01A	UCEC	5.28
TCGA-B5-A11Y-01A	UCEC	7.16
TCGA-AP-A0LG-01A	UCEC	7.9
TCGA-B5-A11N-01A	UCEC	5.34
TCGA-D1-A17A-01A	UCEC	6.06
TCGA-BS-A0V7-01A	UCEC	3.04
TCGA-AX-A1C5-01A	UCEC	7.48
TCGA-AX-A060-01A	UCEC	8.12
TCGA-BG-A3PP-01A	UCEC	7.24
TCGA-AX-A3FV-01A	UCEC	7.15
TCGA-EO-A3KU-01A	UCEC	6.36
TCGA-AX-A3GI-01A	UCEC	6.64
TCGA-FI-A3PX-01A	UCEC	6.61
TCGA-B5-A11F-01A	UCEC	5.74
TCGA-EY-A549-01A	UCEC	7.4
TCGA-AX-A1CA-01A	UCEC	4.7
TCGA-B5-A5OD-01A	UCEC	6.03
TCGA-BK-A0CA-01B	UCEC	6.88
TCGA-B5-A0K1-01A	UCEC	7.23
TCGA-AX-A1CE-01A	UCEC	6.96
TCGA-A5-A0G9-01A	UCEC	6.52
TCGA-D1-A2G6-01A	UCEC	5.83
TCGA-A5-A0G1-01A	UCEC	5.35
TCGA-BG-A18C-01A	UCEC	4.69
TCGA-BK-A4ZD-01A	UCEC	7.36
TCGA-AX-A0J0-01A	UCEC	7.08
TCGA-BG-A0M7-01A	UCEC	7.11
TCGA-B5-A0K8-01A	UCEC	6.01
TCGA-BK-A56F-01A	UCEC	6.78
TCGA-EO-A22U-01A	UCEC	7.36
TCGA-AX-A1C9-01A	UCEC	6.4
TCGA-AX-A0IS-01A	UCEC	6.74
TCGA-AX-A2HJ-01A	UCEC	6.98
TCGA-A5-A0GH-01A	UCEC	7.08
TCGA-SL-A6JA-01A	UCEC	8.58
TCGA-EY-A2OM-01A	UCEC	7
TCGA-B5-A3S1-01A	UCEC	7.02
TCGA-KP-A3W1-01A	UCEC	4.69
TCGA-EY-A2OO-01A	UCEC	7.28
TCGA-AP-A05N-01A	UCEC	7.24
TCGA-D1-A0ZV-01A	UCEC	6.44
TCGA-B5-A3FB-01A	UCEC	7.26
TCGA-D1-A1NY-01A	UCEC	6.22
TCGA-BK-A6W3-01A	UCEC	6.85
TCGA-AP-A1DR-01A	UCEC	7.12
TCGA-A5-A1OJ-01A	UCEC	5.15
TCGA-B5-A121-01A	UCEC	7.09
TCGA-AJ-A3NG-01A	UCEC	6.95
TCGA-B5-A0JV-01A	UCEC	7.83
TCGA-D1-A17S-01A	UCEC	3.85

TCGA-D1-A16X-01A	UCEC	6.1
TCGA-B5-A0K7-01A	UCEC	7.37
TCGA-A5-A1OF-01A	UCEC	6.16
TCGA-DF-A2KZ-01A	UCEC	8.29
TCGA-D1-A0ZQ-01A	UCEC	7.25
TCGA-JU-AAVI-01A	UCEC	5.17
TCGA-A5-A0R6-01A	UCEC	6.16
TCGA-AP-A05P-01A	UCEC	6.85
TCGA-D1-A167-01A	UCEC	6.9
TCGA-BG-A222-01A	UCEC	7
TCGA-AJ-A8CW-01A	UCEC	7.02
TCGA-D1-A162-01A	UCEC	6.83
TCGA-FI-A2F4-01A	UCEC	8.37
TCGA-AJ-A3BI-01A	UCEC	5.05
TCGA-D1-A16J-01A	UCEC	5.8
TCGA-AP-A0LT-01A	UCEC	6.12
TCGA-A5-A0GW-01A	UCEC	6.4
TCGA-A5-A0G2-01A	UCEC	5.79
TCGA-AJ-A3BK-01A	UCEC	6.75
TCGA-PG-A7D5-01A	UCEC	5.42
TCGA-QS-A8F1-01A	UCEC	3.87
TCGA-A5-A0GX-01A	UCEC	5.26
TCGA-AX-A062-01A	UCEC	6.21
TCGA-D1-A17K-01A	UCEC	7.32
TCGA-BG-A220-01A	UCEC	6.61
TCGA-AX-A05S-01A	UCEC	5.7
TCGA-EY-A2OQ-01A	UCEC	4.65
TCGA-AP-A0LM-01A	UCEC	5.28
TCGA-AJ-A3BF-01A	UCEC	6.38
TCGA-A5-A0G5-01A	UCEC	4.56
TCGA-EY-A215-01A	UCEC	7.37
TCGA-FI-A2D4-01A	UCEC	6.56
TCGA-E6-A1LX-01A	UCEC	7.21
TCGA-AX-A2HH-01A	UCEC	6.68
TCGA-BG-A0M9-01A	UCEC	6.14
TCGA-EO-A22R-01A	UCEC	8.01
TCGA-BS-A0UL-01A	UCEC	7.04
TCGA-AP-A1DO-01A	UCEC	6.7
TCGA-B5-A0K0-01A	UCEC	6.15
TCGA-BS-A0VI-01A	UCEC	5.61
TCGA-EY-A547-01A	UCEC	6.29
TCGA-FI-A2EX-01A	UCEC	6.67
TCGA-BS-A0V8-01A	UCEC	4.01
TCGA-EO-A3AZ-01A	UCEC	3.78
TCGA-EO-A3B0-01A	UCEC	7.7
TCGA-D1-A0ZZ-01A	UCEC	5.93
TCGA-DI-A1BU-01A	UCEC	7.58
TCGA-AX-A2HC-01A	UCEC	6.69
TCGA-A5-A1OG-01A	UCEC	5.5
TCGA-QS-A5YR-01A	UCEC	6.87
TCGA-EO-A1Y5-01A	UCEC	5.22
TCGA-EO-A3AS-01A	UCEC	6.33
TCGA-AJ-A3EL-01A	UCEC	6.37
TCGA-EO-A3B1-01A	UCEC	5.91
TCGA-DF-A2KR-01A	UCEC	7.6
TCGA-D1-A2G7-01A	UCEC	5.99
TCGA-EY-A1GD-01A	UCEC	6.52
TCGA-A5-A3LO-01A	UCEC	10.09

TCGA-BS-A0TG-01A	UCEC	5.5
TCGA-A5-A0R8-01A	UCEC	6.28
TCGA-B5-A0JS-01A	UCEC	6.17
TCGA-A5-A0GD-01A	UCEC	7.71
TCGA-BG-A187-01A	UCEC	6.09
TCGA-KJ-A3U4-01A	UCEC	7.75
TCGA-BG-A0MK-01A	UCEC	6.36
TCGA-BS-A0U9-01B	UCEC	7.86
TCGA-BG-A0M4-01A	UCEC	7.33
TCGA-BK-A0CB-01A	UCEC	6.08
TCGA-D1-A103-01A	UCEC	7.02
TCGA-AJ-A3EJ-01A	UCEC	7.42
TCGA-FI-A2EY-01A	UCEC	3
TCGA-D1-A3DH-01A	UCEC	6.1
TCGA-FI-A2D6-01A	UCEC	5.52
TCGA-B5-A0K3-01A	UCEC	6.19
TCGA-AX-A2HF-01A	UCEC	6.2
TCGA-EY-A1G7-01A	UCEC	7.48
TCGA-D1-A16Q-01A	UCEC	4.72
TCGA-BK-A139-02A	UCEC	7.46
TCGA-AJ-A3OL-01A	UCEC	7.09
TCGA-D1-A0ZN-01A	UCEC	8.19
TCGA-EY-A3QX-01A	UCEC	6.68
TCGA-AX-A06B-01A	UCEC	7.02
TCGA-BK-A139-01C	UCEC	9.03
TCGA-AX-A2HA-01A	UCEC	6.17
TCGA-EO-A3AY-01A	UCEC	5.46
TCGA-B5-A11U-01A	UCEC	5.71
TCGA-D1-A16Y-01A	UCEC	5.03
TCGA-E6-A2P9-01A	UCEC	8.48
TCGA-D1-A15X-01A	UCEC	6.15
TCGA-B5-A11L-01B	UCEC	5.46
TCGA-AP-A0LD-01A	UCEC	4.86
TCGA-AJ-A3NE-01A	UCEC	7.36
TCGA-AP-A1DV-01A	UCEC	6.64
TCGA-B5-A1MY-01A	UCEC	5.67
TCGA-D1-A17R-01A	UCEC	7.07
TCGA-DF-A2KV-01A	UCEC	6.81
TCGA-FI-A2F8-01A	UCEC	6.34
TCGA-A5-A0GV-01A	UCEC	5.74
TCGA-EY-A1GP-01A	UCEC	6.16
TCGA-FI-A3PV-01A	UCEC	5.96
TCGA-DF-A2L0-01A	UCEC	5.4
TCGA-AP-A0LN-01A	UCEC	6.09
TCGA-AX-A1C7-01A	UCEC	4.6
TCGA-AP-A0L9-01A	UCEC	6.55
TCGA-BG-A0LW-01A	UCEC	7.08
TCGA-D1-A17D-01A	UCEC	6.2
TCGA-DI-A2QY-01A	UCEC	5.24
TCGA-AX-A0IU-01A	UCEC	5.24
TCGA-E6-A1LZ-01A	UCEC	4.81
TCGA-BK-A139-01A	UCEC	8.81
TCGA-BG-A0MC-01A	UCEC	4.83
TCGA-EY-A1GU-01A	UCEC	7.34
TCGA-BG-A18B-01A	UCEC	7.33
TCGA-AJ-A3OJ-01A	UCEC	6.95
TCGA-BS-A0TC-01A	UCEC	6.18
TCGA-AJ-A3IA-01A	UCEC	7.74

TCGA-D1-A16V-01A	UCEC	7.41
TCGA-AX-A3FT-01A	UCEC	4.89
TCGA-BG-A0VX-01A	UCEC	6.44
TCGA-BG-A0M0-01A	UCEC	6.5
TCGA-EO-A3KX-01A	UCEC	7.63
TCGA-AX-A2IN-01A	UCEC	5.56
TCGA-AJ-A2QM-01A	UCEC	7.74
TCGA-AX-A2H8-01A	UCEC	6.7
TCGA-AJ-A2QN-01A	UCEC	6.6
TCGA-BS-A0UJ-01A	UCEC	4.74
TCGA-D1-A3DA-01A	UCEC	6.57
TCGA-FI-A2D5-01A	UCEC	7.45
TCGA-AP-A0LE-01A	UCEC	6.66
TCGA-EO-A1Y8-01A	UCEC	5.34
TCGA-AJ-A2QK-01A	UCEC	6.39
TCGA-A5-A7WK-01A	UCEC	5.94
TCGA-B5-A11H-01A	UCEC	7.32
TCGA-BG-A0W2-01A	UCEC	6.03
TCGA-AX-A05U-01A	UCEC	6.67
TCGA-AX-A3G9-01A	UCEC	8.02
TCGA-AX-A06H-01A	UCEC	8
TCGA-BS-A0UF-01A	UCEC	6.68
TCGA-EO-A22Y-01A	UCEC	6.12
TCGA-B5-A11M-01A	UCEC	6.08
TCGA-BG-A186-01A	UCEC	6.21
TCGA-BG-A0VW-01A	UCEC	5.6
TCGA-EY-A1GF-01A	UCEC	9.04
TCGA-EO-A22T-01A	UCEC	7.01
TCGA-AX-A064-01A	UCEC	6.72
TCGA-D1-A17L-01A	UCEC	5.81
TCGA-AX-A3G4-01A	UCEC	8.29
TCGA-KP-A3VZ-01A	UCEC	6.45
TCGA-5S-A9Q8-01A	UCEC	5.92
TCGA-BG-A0M3-01A	UCEC	5.93
TCGA-EY-A54A-01A	UCEC	6.44
TCGA-BG-A0VV-01A	UCEC	6.45
TCGA-B5-A1MZ-01A	UCEC	6.63
TCGA-D1-A17U-01A	UCEC	6.8
TCGA-AJ-A3QS-01A	UCEC	6.51
TCGA-A5-A2K2-01A	UCEC	5.19
TCGA-AJ-A8CT-01A	UCEC	4.97
TCGA-AP-A1E0-01A	UCEC	7.39
TCGA-D1-A0ZS-01A	UCEC	6.15
TCGA-EO-A3AU-01A	UCEC	8.04
TCGA-AX-A05W-01A	UCEC	5.57
TCGA-FI-A2F9-01A	UCEC	6.01
TCGA-BG-A0W1-01A	UCEC	5.58
TCGA-A5-A0VP-01A	UCEC	8.41
TCGA-B5-A3F9-01A	UCEC	6.34
TCGA-D1-A1O8-01A	UCEC	5.02
TCGA-BS-A0U7-01A	UCEC	8.99
TCGA-PG-A5BC-01A	UCEC	6.55
TCGA-A5-A0GB-01A	UCEC	9.25
TCGA-A5-A1OK-01A	UCEC	6.07
TCGA-B5-A1MX-01A	UCEC	7
TCGA-D1-A179-01A	UCEC	6.02
TCGA-AX-A3FW-01A	UCEC	8.57
TCGA-B5-A11E-01A	UCEC	6.97

TCGA-AP-A1E1-01A	UCEC	8.09
TCGA-A5-A0R7-01A	UCEC	5.84
TCGA-D1-A17M-01A	UCEC	7.78
TCGA-D1-A161-01A	UCEC	6.26
TCGA-QS-A5YQ-01A	UCEC	5.48
TCGA-D1-A3JQ-01A	UCEC	5.88
TCGA-AP-A1DH-01A	UCEC	7.81
TCGA-A5-A0GP-01A	UCEC	7.35
TCGA-A5-A0VO-01A	UCEC	6.23
TCGA-EY-A3L3-01A	UCEC	6.88
TCGA-B5-A11V-01A	UCEC	6.83
TCGA-AJ-A5DV-01A	UCEC	6.44
TCGA-AJ-A3TW-01A	UCEC	5.2
TCGA-DF-A2KN-01A	UCEC	7.59
TCGA-AJ-A3EM-01A	UCEC	8.47
TCGA-AX-A1CN-01A	UCEC	6.93
TCGA-AP-A0LO-01A	UCEC	6.37
TCGA-D1-A16N-01A	UCEC	6.9
TCGA-QF-A5YS-01A	UCEC	5.76
TCGA-D1-A17F-01A	UCEC	5.47
TCGA-EY-A1GM-01A	UCEC	6.03
TCGA-BG-A0MQ-01A	UCEC	7.81
TCGA-AJ-A3OK-01A	UCEC	5.82
TCGA-EY-A1GI-01A	UCEC	7.76
TCGA-FI-A2D0-01A	UCEC	5.48
TCGA-B5-A3FH-01A	UCEC	7.5
TCGA-BG-A2L7-01A	UCEC	6.24
TCGA-A5-A0VQ-01A	UCEC	6.75
TCGA-D1-A16S-01A	UCEC	5.01
TCGA-AX-A0IZ-01A	UCEC	5.71
TCGA-EY-A5W2-01A	UCEC	7.31
TCGA-AP-A3K1-01A	UCEC	4.96
TCGA-A5-A0R9-01A	UCEC	5.36
TCGA-EY-A212-01A	UCEC	5.66
TCGA-AX-A1CR-01A	UCEC	4.07
TCGA-A5-A0GI-01A	UCEC	6.39
TCGA-N6-A4VF-01A	UCS	6.66
TCGA-N9-A4PZ-01A	UCS	5.61
TCGA-N9-A4Q8-01A	UCS	5.65
TCGA-N8-A4PQ-01A	UCS	5.67
TCGA-N5-A4RO-01A	UCS	5.17
TCGA-QM-A5NM-01A	UCS	5.51
TCGA-N5-A4RJ-01A	UCS	6.61
TCGA-NA-A4QW-01A	UCS	5.31
TCGA-N5-A4RS-01A	UCS	5.92
TCGA-NF-A4WX-01A	UCS	5.82
TCGA-NG-A4VU-01A	UCS	4.84
TCGA-NA-A4QX-01A	UCS	5.68
TCGA-ND-A4WF-01A	UCS	5.01
TCGA-N6-A4V9-01A	UCS	5.89
TCGA-ND-A4WC-01A	UCS	7
TCGA-ND-A4WA-01A	UCS	6.15
TCGA-N5-A4RF-01A	UCS	5.83
TCGA-N9-A4Q4-01A	UCS	5.62
TCGA-N6-A4VE-01A	UCS	5.97
TCGA-N8-A56S-01A	UCS	6.09
TCGA-NA-A5I1-01A	UCS	5.99
TCGA-N5-A4RU-01A	UCS	5.05

TCGA-N5-A4RV-01A	UCS	3.69
TCGA-N5-A4RA-01A	UCS	5.5
TCGA-N5-A4RM-01A	UCS	4.61
TCGA-N8-A4PM-01A	UCS	5.41
TCGA-N6-A4VC-01A	UCS	7.06
TCGA-N5-A59F-01A	UCS	6
TCGA-N7-A4Y5-01A	UCS	4.16
TCGA-N5-A4RT-01A	UCS	6.77
TCGA-N7-A59B-01A	UCS	5.86
TCGA-N8-A4PO-01A	UCS	5.71
TCGA-N5-A4RN-01A	UCS	4.61
TCGA-NF-A4X2-01A	UCS	5.23
TCGA-N7-A4Y8-01A	UCS	4.21
TCGA-N8-A4PL-01A	UCS	6.05
TCGA-N9-A4Q1-01A	UCS	4.53
TCGA-N5-A4RD-01A	UCS	5.66
TCGA-N5-A4R8-01A	UCS	4.96
TCGA-N8-A4PN-01A	UCS	5.07
TCGA-QN-A5NN-01A	UCS	6.24
TCGA-NA-A4R1-01A	UCS	5.86
TCGA-N9-A4Q7-01A	UCS	4.96
TCGA-NF-A5CP-01A	UCS	6.67
TCGA-N8-A4PI-01A	UCS	6.1
TCGA-NA-A4R0-01A	UCS	5.59
TCGA-N5-A59E-01A	UCS	6.02
TCGA-NG-A4VW-01A	UCS	6.35
TCGA-N6-A4VG-01A	UCS	5.49
TCGA-NA-A4QV-01A	UCS	6.15
TCGA-N6-A4VD-01A	UCS	6.19
TCGA-ND-A4W6-01A	UCS	5.09
TCGA-N8-A4PP-01A	UCS	5.4
TCGA-NF-A4WU-01A	UCS	6.86
TCGA-N9-A4Q3-01A	UCS	5.63
TCGA-N7-A4Y0-01A	UCS	5.11
TCGA-NA-A4QY-01A	UCS	6.54
TCGA-VD-A8KJ-01A	UVM	5.54
TCGA-WC-A87W-01A	UVM	4.9
TCGA-V4-A9EW-01A	UVM	4.81
TCGA-V4-A9F8-01A	UVM	3.08
TCGA-YZ-A984-01A	UVM	3.64
TCGA-WC-A888-01A	UVM	4.49
TCGA-VD-A8KH-01A	UVM	4.91
TCGA-V4-A9EZ-01A	UVM	2.36
TCGA-V4-A9F2-01A	UVM	3.7
TCGA-V4-A9F4-01A	UVM	4.38
TCGA-WC-A881-01A	UVM	3.58
TCGA-WC-A87U-01A	UVM	4.49
TCGA-WC-A88A-01A	UVM	4.29
TCGA-V4-A9ET-01A	UVM	3.33
TCGA-V3-A9ZX-01A	UVM	5.09
TCGA-V4-A9E9-01A	UVM	3.05
TCGA-V4-A9EK-01A	UVM	5.84
TCGA-WC-AA9E-01A	UVM	5.11
TCGA-VD-A8KF-01A	UVM	4.34
TCGA-V4-A9EM-01A	UVM	4.12
TCGA-VD-A8KD-01A	UVM	4.99
TCGA-V4-A9EH-01A	UVM	4.49
TCGA-V4-A9F7-01A	UVM	4.49

TCGA-V4-A9E5-01A	UVM	2.95
TCGA-V4-A9EY-01A	UVM	2.43
TCGA-VD-A8KM-01A	UVM	5.11
TCGA-VD-A8KO-01A	UVM	4.75
TCGA-VD-AA8N-01A	UVM	3.72
TCGA-VD-A8KB-01A	UVM	3.98
TCGA-V4-A9ED-01A	UVM	4.1
TCGA-WC-A87Y-01A	UVM	5.06
TCGA-V3-A9ZY-01A	UVM	3.3
TCGA-VD-A8KI-01A	UVM	4.16
TCGA-RZ-AB0B-01A	UVM	4.67
TCGA-WC-A87T-01A	UVM	4.3
TCGA-VD-AA8Q-01A	UVM	2.33
TCGA-V4-A9F0-01A	UVM	4.27
TCGA-VD-AA8M-01A	UVM	4.09
TCGA-WC-A884-01A	UVM	5.29
TCGA-V4-A9EX-01A	UVM	4.99
TCGA-V4-A9EL-01A	UVM	4.91
TCGA-WC-A882-01A	UVM	2.81
TCGA-VD-AA8S-01B	UVM	4.81
TCGA-VD-AA8O-01A	UVM	4.7
TCGA-V4-A9E7-01A	UVM	3.64
TCGA-V4-A9ES-01A	UVM	3.93
TCGA-YZ-A983-01A	UVM	4.39
TCGA-V4-A9F1-01A	UVM	5.03
TCGA-WC-A883-01A	UVM	3.9
TCGA-VD-A8KL-01A	UVM	3.81
TCGA-VD-A8KN-01A	UVM	4.27
TCGA-V4-A9EO-01A	UVM	4.98
TCGA-WC-A880-01A	UVM	5.3
TCGA-V4-A9EV-01A	UVM	5
TCGA-V4-A9E8-01A	UVM	4.26
TCGA-V4-A9EE-01A	UVM	5.16
TCGA-WC-A885-01A	UVM	3.59
TCGA-VD-AA8T-01A	UVM	4.54
TCGA-VD-A8K8-01A	UVM	4.51
TCGA-V4-A9EF-01A	UVM	3.69
TCGA-V4-A9EA-01A	UVM	2.7
TCGA-VD-A8KA-01B	UVM	4.3
TCGA-VD-AA8P-01A	UVM	4
TCGA-YZ-A980-01A	UVM	5.56
TCGA-VD-A8K9-01A	UVM	5.42
TCGA-VD-A8KG-01A	UVM	4.98
TCGA-V4-A9EQ-01A	UVM	3.21
TCGA-V4-A9F5-01A	UVM	3.29
TCGA-YZ-A982-01A	UVM	4.61
TCGA-V4-A9EJ-01A	UVM	4.82
TCGA-V4-A9EU-01A	UVM	4.86
TCGA-YZ-A985-01A	UVM	2.93
TCGA-VD-A8KK-01A	UVM	3.08
TCGA-VD-AA8R-01A	UVM	4.43
TCGA-V4-A9EI-01A	UVM	5.53
TCGA-VD-A8KE-01A	UVM	3.96
TCGA-V4-A9F3-01A	UVM	5.35
TCGA-V4-A9EC-01A	UVM	3.12
TCGA-VD-A8K7-01B	UVM	4.45
TCGA-WC-AA9A-01A	UVM	3.7

Fig.1 D

caseid	Cancer	GLUD1[log2(TPM+1)]
TCGA-OR-A5LP-01A	ACC	6.24
TCGA-OR-A5K0-01A	ACC	6.78
TCGA-OR-A5JF-01A	ACC	6.5
TCGA-OR-A5JT-01A	ACC	7.19
TCGA-OR-A5K1-01A	ACC	6.79
TCGA-P6-A5OG-01A	ACC	7.33
TCGA-PK-A5H9-01A	ACC	7.96
TCGA-OR-A5KZ-01A	ACC	7.48
TCGA-OR-A5KX-01A	ACC	8.1
TCGA-OR-A5JY-01A	ACC	6.5
TCGA-PK-A5HA-01A	ACC	6.19
TCGA-OR-A5LR-01A	ACC	6.78
TCGA-OR-A5JQ-01A	ACC	7
TCGA-OR-A5LG-01A	ACC	6.46
TCGA-OR-A5LB-01A	ACC	7.13
TCGA-OR-A5JM-01A	ACC	8.04
TCGA-OR-A5LJ-01A	ACC	7.02
TCGA-OR-A5J3-01A	ACC	6.3
TCGA-OR-A5J6-01A	ACC	7.74
TCGA-OR-A5L3-01A	ACC	5.39
TCGA-OR-A5J1-01A	ACC	6.82
TCGA-OR-A5LA-01A	ACC	5.34
TCGA-OR-A5LH-01A	ACC	6.47
TCGA-OR-A5JR-01A	ACC	7.2
TCGA-OR-A5JK-01A	ACC	6.12
TCGA-OR-A5K9-01A	ACC	7.25
TCGA-OR-A5LC-01A	ACC	6.86
TCGA-OR-A5J9-01A	ACC	7.94
TCGA-OR-A5LE-01A	ACC	7.95
TCGA-OR-A5LL-01A	ACC	7.31
TCGA-OR-A5K8-01A	ACC	6.04
TCGA-OR-A5LO-01A	ACC	6.23
TCGA-OR-A5JD-01A	ACC	6.28
TCGA-OR-A5K6-01A	ACC	6.7
TCGA-OR-A5KW-01A	ACC	6.32
TCGA-OR-A5L5-01A	ACC	6.48
TCGA-OR-A5JP-01A	ACC	7.15
TCGA-PA-A5YG-01A	ACC	6.63
TCGA-PK-A5H8-01A	ACC	6.49
TCGA-OR-A5KY-01A	ACC	7.19
TCGA-OR-A5JX-01A	ACC	6.42
TCGA-OR-A5JW-01A	ACC	7.13
TCGA-OR-A5J5-01A	ACC	5.2
TCGA-OR-A5J8-01A	ACC	6.6
TCGA-PK-A5HB-01A	ACC	8.37
TCGA-OR-A5JL-01A	ACC	6.43
TCGA-OR-A5L8-01A	ACC	7.62
TCGA-OR-A5JG-01A	ACC	7.47
TCGA-OR-A5LK-01A	ACC	4.54
TCGA-OR-A5JV-01A	ACC	6.05
TCGA-OR-A5K3-01A	ACC	5.04
TCGA-OR-A5L4-01A	ACC	6.46
TCGA-OR-A5LM-01A	ACC	6.08
TCGA-OR-A5KO-01A	ACC	6.16
TCGA-OR-A5JI-01A	ACC	5.99
TCGA-OR-A5JB-01A	ACC	6.65

TCGA-OR-A5JE-01A	ACC	7.29
TCGA-OR-A5LD-01A	ACC	6.65
TCGA-OR-A5JC-01A	ACC	6.76
TCGA-OR-A5KV-01A	ACC	7.27
TCGA-OR-A5JO-01A	ACC	6.94
TCGA-OR-A5LN-01A	ACC	6.04
TCGA-P6-A5OF-01A	ACC	7.42
TCGA-OR-A5LT-01A	ACC	7.3
TCGA-OR-A5JJ-01A	ACC	6.29
TCGA-OR-A5J2-01A	ACC	7.13
TCGA-OR-A5KU-01A	ACC	6.12
TCGA-OR-A5J7-01A	ACC	7.79
TCGA-OR-A5JA-01A	ACC	5.93
TCGA-OR-A5JS-01A	ACC	7.23
TCGA-OR-A5K2-01A	ACC	8.03
TCGA-OR-A5K4-01A	ACC	7.62
TCGA-OR-A5JZ-01A	ACC	7.19
TCGA-OR-A5K5-01A	ACC	6.81
TCGA-OR-A5L6-01A	ACC	5.66
TCGA-OR-A5LS-01A	ACC	6.99
TCGA-OU-A5PI-01A	ACC	7.4
TCGA-OR-A5KT-01A	ACC	7.71
TCGA-OR-A5L9-01A	ACC	3.99
TCGA-E7-A678-01A	BLCA	7.15
TCGA-UY-A9PB-01A	BLCA	6.91
TCGA-DK-A3IN-01A	BLCA	6.63
TCGA-DK-A3IV-01A	BLCA	6.56
TCGA-XF-A9T4-01A	BLCA	7.31
TCGA-FD-A62S-01A	BLCA	6.85
TCGA-FD-A6TG-01A	BLCA	6.66
TCGA-FD-A6TI-01A	BLCA	6.68
TCGA-DK-AA6L-01A	BLCA	6.87
TCGA-FD-A5BT-01A	BLCA	6.09
TCGA-KQ-A41S-01A	BLCA	6.31
TCGA-ZF-AA4V-01A	BLCA	6.53
TCGA-FT-A3EE-01A	BLCA	8.22
TCGA-DK-AA75-01A	BLCA	6.3
TCGA-ZF-AA54-01A	BLCA	7.51
TCGA-FD-A3N5-01A	BLCA	6.24
TCGA-E7-A677-01A	BLCA	6.85
TCGA-DK-A1AB-01A	BLCA	7.21
TCGA-MV-A51V-01A	BLCA	6.56
TCGA-BT-A20N-01A	BLCA	7.51
TCGA-DK-A2I6-01A	BLCA	6.17
TCGA-ZF-AA56-01A	BLCA	7.17
TCGA-HQ-A5NE-01A	BLCA	7.38
TCGA-GV-A6ZA-01A	BLCA	6.39
TCGA-XF-A9T5-01A	BLCA	6.42
TCGA-ZF-AA5N-01A	BLCA	6.69
TCGA-ZF-A9RF-01A	BLCA	6.91
TCGA-BT-A20R-01A	BLCA	6.53
TCGA-SY-A9G5-01A	BLCA	6.89
TCGA-ZF-A9RM-01A	BLCA	7.53
TCGA-CF-A27C-01A	BLCA	6.97
TCGA-CU-A3KJ-01A	BLCA	6.24
TCGA-GC-A4ZW-01A	BLCA	6.83
TCGA-XF-AAN8-01A	BLCA	7
TCGA-K4-A83P-01A	BLCA	6.02

TCGA-GV-A40E-01A	BLCA	6.55
TCGA-CF-A9FL-01A	BLCA	6.51
TCGA-CF-A47Y-01A	BLCA	5.96
TCGA-DK-A1A6-01A	BLCA	7.54
TCGA-FD-A62N-01A	BLCA	6.72
TCGA-E7-A6ME-01A	BLCA	6.2
TCGA-GU-AATQ-01A	BLCA	6.6
TCGA-FD-A5BZ-01A	BLCA	6.89
TCGA-ZF-AA4N-01A	BLCA	7.02
TCGA-XF-A9SJ-01A	BLCA	6.31
TCGA-BL-A0C8-01B	BLCA	8.24
TCGA-BT-A42C-01A	BLCA	7.17
TCGA-CF-A5UA-01A	BLCA	7.18
TCGA-E5-A4TZ-01A	BLCA	7.67
TCGA-G2-A2EJ-01A	BLCA	6.7
TCGA-KQ-A41R-01A	BLCA	6.87
TCGA-2F-A9KO-01A	BLCA	6.79
TCGA-XF-AAMZ-01A	BLCA	7.4
TCGA-DK-A1AF-01A	BLCA	7.42
TCGA-DK-AA6T-01A	BLCA	6.52
TCGA-BL-A13I-01A	BLCA	6.35
TCGA-GU-A42P-01A	BLCA	8.17
TCGA-UY-A8OD-01A	BLCA	7.04
TCGA-DK-A2HX-01A	BLCA	7.11
TCGA-UY-A78P-01A	BLCA	7.41
TCGA-DK-A6AV-01A	BLCA	7.2
TCGA-BL-A0C8-01A	BLCA	8.37
TCGA-CU-A3QU-01A	BLCA	6.99
TCGA-XF-A9SX-01A	BLCA	6.18
TCGA-FJ-A871-01A	BLCA	6.58
TCGA-ZF-A9R2-01A	BLCA	6.91
TCGA-DK-A1A3-01A	BLCA	7.32
TCGA-FJ-A3ZF-01A	BLCA	7.14
TCGA-E7-A6MF-01A	BLCA	7.32
TCGA-E7-A4IJ-01A	BLCA	6.32
TCGA-FD-A6TA-01A	BLCA	6.26
TCGA-UY-A8OB-01A	BLCA	6.97
TCGA-ZF-A9RL-01A	BLCA	7.04
TCGA-FD-A6TB-01A	BLCA	7.07
TCGA-XF-AAMG-01A	BLCA	6.05
TCGA-CF-A1HR-01A	BLCA	6.58
TCGA-CF-A3MG-01A	BLCA	7.02
TCGA-ZF-AA5P-01A	BLCA	6.49
TCGA-DK-AA74-01A	BLCA	5.95
TCGA-DK-A1A7-01A	BLCA	4.92
TCGA-SY-A9G0-01A	BLCA	6.94
TCGA-UY-A78N-01A	BLCA	7.48
TCGA-DK-A3IT-01A	BLCA	7.04
TCGA-ZF-AA4U-01A	BLCA	7.43
TCGA-UY-A8OC-01A	BLCA	7.1
TCGA-4Z-AA87-01A	BLCA	7.19
TCGA-ZF-A9R0-01A	BLCA	5.86
TCGA-4Z-AA7Y-01A	BLCA	7.59
TCGA-K4-A54R-01A	BLCA	6.58
TCGA-FD-A5BV-01A	BLCA	7.21
TCGA-K4-A6FZ-01A	BLCA	7.22
TCGA-LT-A8JT-01A	BLCA	7.08
TCGA-BL-A13J-01A	BLCA	6.98

TCGA-XF-A9T0-01A	BLCA	8.95
TCGA-FD-A5BX-01A	BLCA	6.97
TCGA-DK-A3X1-01A	BLCA	5.86
TCGA-XF-A9T3-01A	BLCA	7.01
TCGA-G2-A2ES-01A	BLCA	8.12
TCGA-R3-A69X-01A	BLCA	7.49
TCGA-FD-A3SP-01A	BLCA	6.63
TCGA-K4-A6MB-01A	BLCA	7.39
TCGA-GD-A76B-01A	BLCA	5.56
TCGA-FJ-A3Z9-01A	BLCA	6.78
TCGA-BT-A3PK-01A	BLCA	6.13
TCGA-CF-A9FF-01A	BLCA	6.07
TCGA-GC-A3I6-01A	BLCA	7.46
TCGA-CF-A47X-01A	BLCA	6.97
TCGA-S5-A6DX-01A	BLCA	6.35
TCGA-4Z-AA7N-01A	BLCA	6.77
TCGA-FD-A5BU-01A	BLCA	6.46
TCGA-XF-AAN2-01A	BLCA	6.85
TCGA-G2-AA3F-01A	BLCA	6.39
TCGA-FT-A61P-01A	BLCA	6.75
TCGA-K4-A3WU-01B	BLCA	7.13
TCGA-ZF-A9R5-01A	BLCA	7.28
TCGA-CF-A9FM-01A	BLCA	7.45
TCGA-5N-A9KM-01A	BLCA	6.96
TCGA-GV-A3QK-01B	BLCA	6.87
TCGA-GU-A42Q-01A	BLCA	7.64
TCGA-C4-A0F1-01A	BLCA	7.29
TCGA-DK-A6B6-01A	BLCA	6.13
TCGA-ZF-AA53-01A	BLCA	6.42
TCGA-G2-AA3C-01A	BLCA	6.86
TCGA-E7-A3X6-01A	BLCA	7.06
TCGA-FD-A5BS-01A	BLCA	5.11
TCGA-BL-A5ZZ-01A	BLCA	6.76
TCGA-DK-A3IL-01A	BLCA	5.75
TCGA-XF-A8HC-01A	BLCA	6.19
TCGA-CF-A47T-01A	BLCA	7.09
TCGA-ZF-AA4T-01A	BLCA	6.33
TCGA-E7-A97P-01A	BLCA	7.81
TCGA-BL-A3JM-01A	BLCA	6.75
TCGA-FD-A5C1-01A	BLCA	5.8
TCGA-2F-A9KQ-01A	BLCA	7.67
TCGA-CF-A3MF-01A	BLCA	5.07
TCGA-G2-AA3B-01A	BLCA	6.69
TCGA-CF-A47W-01A	BLCA	6.79
TCGA-FD-A43U-01A	BLCA	5.99
TCGA-G2-A2EC-01A	BLCA	7.12
TCGA-FD-A3SQ-01A	BLCA	6.77
TCGA-XF-A9T2-01A	BLCA	6.41
TCGA-E7-A4XJ-01A	BLCA	6.34
TCGA-DK-A3IS-01A	BLCA	7.31
TCGA-XF-A9SH-01A	BLCA	6.88
TCGA-GC-A3RC-01A	BLCA	6.28
TCGA-PQ-A6FN-01A	BLCA	7.7
TCGA-CF-A3MH-01A	BLCA	6.8
TCGA-BT-A20X-01A	BLCA	7.34
TCGA-XF-AAME-01A	BLCA	6.82
TCGA-XF-A8HG-01A	BLCA	6.67
TCGA-XF-AAN5-01A	BLCA	7.99

TCGA-FD-A62P-01A	BLCA	6.83
TCGA-DK-A6B0-01A	BLCA	7.03
TCGA-XF-AAMR-01A	BLCA	7.16
TCGA-4Z-AA7W-01A	BLCA	6.09
TCGA-E7-A8O8-01A	BLCA	6.83
TCGA-FD-A3N6-01A	BLCA	7.42
TCGA-4Z-AA83-01A	BLCA	6.95
TCGA-E7-A6MD-01A	BLCA	6.94
TCGA-GC-A3BM-01A	BLCA	7.07
TCGA-CU-A5W6-01A	BLCA	6.3
TCGA-FD-A3SO-01A	BLCA	6.81
TCGA-KQ-A41O-01A	BLCA	6.01
TCGA-G2-A2EL-01A	BLCA	6.47
TCGA-XF-A9T8-01A	BLCA	6.73
TCGA-XF-AAN3-01A	BLCA	7.22
TCGA-GU-AATP-01A	BLCA	6.51
TCGA-E7-A7DV-01A	BLCA	6.99
TCGA-FD-A62O-01A	BLCA	7.43
TCGA-XF-A9SU-01A	BLCA	6.08
TCGA-C4-A0F0-01A	BLCA	8.14
TCGA-DK-A2I1-01A	BLCA	6.71
TCGA-FD-A3SL-01A	BLCA	7.19
TCGA-ZF-AA5H-01A	BLCA	7.21
TCGA-DK-AA6P-01A	BLCA	6.78
TCGA-BT-A2LD-01A	BLCA	6.47
TCGA-DK-A3WY-01A	BLCA	6.4
TCGA-YC-A8S6-01A	BLCA	7.37
TCGA-UY-A9PD-01A	BLCA	7.12
TCGA-GD-A6C6-01A	BLCA	5.83
TCGA-ZF-AA4R-01A	BLCA	6.22
TCGA-GU-A767-01A	BLCA	6.8
TCGA-FD-A6TD-01A	BLCA	6.18
TCGA-CU-A72E-01A	BLCA	6.84
TCGA-E7-A85H-01A	BLCA	7.84
TCGA-XF-AAN4-01A	BLCA	7.22
TCGA-2F-A9KT-01A	BLCA	7.11
TCGA-GD-A3OP-01A	BLCA	6.72
TCGA-LC-A66R-01A	BLCA	6.58
TCGA-XF-AAML-01A	BLCA	5.58
TCGA-UY-A9PF-01A	BLCA	7.44
TCGA-DK-A3IM-01A	BLCA	6.44
TCGA-FD-A3B5-01A	BLCA	7.39
TCGA-XF-A9SL-01A	BLCA	6.53
TCGA-XF-AAMH-01A	BLCA	6.35
TCGA-E5-A4U1-01A	BLCA	7.14
TCGA-GU-A764-01A	BLCA	5.53
TCGA-CF-A7I0-01A	BLCA	6.78
TCGA-4Z-AA7M-01A	BLCA	6.33
TCGA-E7-A7XN-01A	BLCA	7.44
TCGA-4Z-AA82-01A	BLCA	6.99
TCGA-PQ-A6FI-01A	BLCA	7.17
TCGA-C4-A0EZ-01A	BLCA	6.36
TCGA-CU-A3YL-01A	BLCA	6.14
TCGA-FD-A5C0-01A	BLCA	6.75
TCGA-HQ-A5ND-01A	BLCA	7.37
TCGA-GU-A42R-01A	BLCA	7.65
TCGA-UY-A78M-01A	BLCA	6.58
TCGA-DK-A6B1-01A	BLCA	7.49

TCGA-ZF-AA58-01A	BLCA	7.19
TCGA-ZF-AA51-01A	BLCA	6.54
TCGA-XF-AAMW-01A	BLCA	7.24
TCGA-GV-A40G-01A	BLCA	6.74
TCGA-DK-AA6U-01A	BLCA	6.43
TCGA-K4-A4AC-01A	BLCA	6.22
TCGA-BT-A20V-01A	BLCA	7.19
TCGA-DK-A6B2-01A	BLCA	7.24
TCGA-BT-A0YX-01A	BLCA	6.51
TCGA-ZF-A9RE-01A	BLCA	7.31
TCGA-GD-A3OQ-01A	BLCA	7.22
TCGA-DK-AA71-01A	BLCA	7.1
TCGA-FD-A3NA-01A	BLCA	7.56
TCGA-YF-AA3M-01A	BLCA	6.73
TCGA-GV-A3QI-01A	BLCA	6.19
TCGA-GC-A3OO-01A	BLCA	7.55
TCGA-DK-A3WW-01A	BLCA	7.54
TCGA-DK-A2I2-01A	BLCA	6.15
TCGA-K4-A5RI-01A	BLCA	7.09
TCGA-ZF-A9R7-01A	BLCA	6.3
TCGA-K4-A5RH-01A	BLCA	6.95
TCGA-G2-AA3D-01A	BLCA	6.81
TCGA-UY-A9PE-01A	BLCA	6.19
TCGA-4Z-AA7Q-01A	BLCA	5.28
TCGA-DK-A1AG-01A	BLCA	7.51
TCGA-UY-A9PH-01A	BLCA	7.15
TCGA-CF-A1HS-01A	BLCA	7.13
TCGA-H4-A2HQ-01A	BLCA	7.5
TCGA-G2-A3VY-01A	BLCA	7.15
TCGA-CF-A47S-01A	BLCA	6.71
TCGA-K4-A3WV-01A	BLCA	6.67
TCGA-XF-A9SZ-01A	BLCA	6.56
TCGA-DK-A6B5-01A	BLCA	7.55
TCGA-DK-A3IU-01A	BLCA	7.13
TCGA-DK-AA76-01A	BLCA	7.24
TCGA-KQ-A41P-01A	BLCA	7.22
TCGA-ZF-AA4X-01A	BLCA	6.72
TCGA-ZF-A9R1-01A	BLCA	7.61
TCGA-GU-A766-01A	BLCA	6.42
TCGA-XF-A8HD-01A	BLCA	6.45
TCGA-BT-A20P-01A	BLCA	7.13
TCGA-DK-AA77-01A	BLCA	7.72
TCGA-E7-A541-01A	BLCA	5.94
TCGA-BT-A3PJ-01A	BLCA	6.97
TCGA-G2-A3IE-01A	BLCA	7.14
TCGA-BL-A13I-01B	BLCA	7.01
TCGA-FD-A43Y-01A	BLCA	7.46
TCGA-LT-A5Z6-01A	BLCA	6.03
TCGA-UY-A78L-01A	BLCA	6.29
TCGA-DK-AA6M-01A	BLCA	6.46
TCGA-XF-AAN0-01A	BLCA	5.85
TCGA-FD-A5BR-01A	BLCA	7.91
TCGA-GV-A3JX-01A	BLCA	6.34
TCGA-GU-A763-01A	BLCA	6.77
TCGA-XF-A8HI-01A	BLCA	7.14
TCGA-ZF-A9RD-01A	BLCA	6.75
TCGA-DK-A3IQ-01A	BLCA	6.94
TCGA-GU-AATO-01A	BLCA	6.19

TCGA-BT-A2LB-01A	BLCA	7.24
TCGA-GC-A3WC-01A	BLCA	6.39
TCGA-XF-A9SV-01A	BLCA	6.37
TCGA-4Z-AA7S-01A	BLCA	7.02
TCGA-CF-A47V-01A	BLCA	6.04
TCGA-DK-A3WX-01A	BLCA	6.41
TCGA-CF-A3MI-01A	BLCA	5.96
TCGA-XF-AAMX-01A	BLCA	6.41
TCGA-BT-A42F-01A	BLCA	6.16
TCGA-2F-A9KR-01A	BLCA	6.65
TCGA-FD-A3B7-01A	BLCA	6.22
TCGA-ZF-A9R9-01A	BLCA	6.62
TCGA-XF-A9SW-01A	BLCA	7
TCGA-E7-A3Y1-01A	BLCA	6.41
TCGA-DK-A2I4-01A	BLCA	6.91
TCGA-FD-A6TE-01A	BLCA	7.6
TCGA-GD-A2C5-01A	BLCA	7.48
TCGA-XF-AAN1-01A	BLCA	7.15
TCGA-DK-AA6R-01A	BLCA	7.23
TCGA-UY-A78K-01A	BLCA	7.02
TCGA-DK-AA6W-01A	BLCA	7.02
TCGA-E7-A7DU-01A	BLCA	6.76
TCGA-YF-AA3L-01A	BLCA	6.51
TCGA-GU-A762-01A	BLCA	7.2
TCGA-E7-A519-01A	BLCA	6.32
TCGA-FD-A3SJ-01A	BLCA	7
TCGA-XF-A8HE-01A	BLCA	6.8
TCGA-YC-A9TC-01A	BLCA	8.53
TCGA-GC-A3RD-01A	BLCA	6.76
TCGA-XF-A9SP-01A	BLCA	6.76
TCGA-XF-A8HF-01A	BLCA	7.2
TCGA-XF-AAMQ-01A	BLCA	7.63
TCGA-FD-A43S-01A	BLCA	6.36
TCGA-CF-A9FH-01A	BLCA	7.12
TCGA-XF-AAMJ-01A	BLCA	6.38
TCGA-4Z-AA86-01A	BLCA	7.25
TCGA-DK-A1A5-01A	BLCA	6.52
TCGA-GV-A3QG-01A	BLCA	7.53
TCGA-K4-A4AB-01B	BLCA	7.62
TCGA-XF-AAMY-01A	BLCA	7.24
TCGA-FD-A3SS-01A	BLCA	6.34
TCGA-CU-A0YR-01A	BLCA	7.6
TCGA-BT-A20J-01A	BLCA	7.13
TCGA-H4-A2HO-01A	BLCA	8.13
TCGA-4Z-AA7O-01A	BLCA	7.04
TCGA-XF-A9SY-01A	BLCA	7.17
TCGA-GV-A3JZ-01A	BLCA	6.17
TCGA-CF-A8HY-01A	BLCA	5.86
TCGA-DK-A1AD-01A	BLCA	6.69
TCGA-FD-A5BY-01A	BLCA	7.25
TCGA-BT-A3PH-01A	BLCA	7.62
TCGA-FD-A3B4-01A	BLCA	6.76
TCGA-G2-A2EO-01A	BLCA	6.71
TCGA-FJ-A3ZE-01A	BLCA	6.6
TCGA-G2-A3IB-01A	BLCA	7.78
TCGA-ZF-A9R3-01A	BLCA	7.01
TCGA-GC-A3RB-01A	BLCA	7.74
TCGA-XF-A9SM-01A	BLCA	6.57

TCGA-GC-A6I3-01A	BLCA	5.54
TCGA-S5-AA26-01A	BLCA	6.5
TCGA-XF-AAMT-01A	BLCA	6.71
TCGA-BT-A20O-01A	BLCA	6.99
TCGA-C4-A0F7-01A	BLCA	8.08
TCGA-BT-A42E-01A	BLCA	6.31
TCGA-BT-A2LA-01A	BLCA	6.98
TCGA-BT-A20U-01A	BLCA	7.2
TCGA-4Z-AA84-01A	BLCA	6.94
TCGA-XF-A8HB-01A	BLCA	8.15
TCGA-KQ-A41Q-01A	BLCA	5.62
TCGA-YC-A89H-01A	BLCA	6.77
TCGA-XF-AAN7-01A	BLCA	6.72
TCGA-FD-A6TC-01A	BLCA	6.67
TCGA-CF-A5U8-01A	BLCA	6.7
TCGA-DK-A3IK-01A	BLCA	6.15
TCGA-GC-A3YS-01A	BLCA	6.56
TCGA-GV-A3JV-01A	BLCA	7.64
TCGA-FD-A3SM-01A	BLCA	6.82
TCGA-DK-A1AC-01A	BLCA	7.04
TCGA-ZF-A9R4-01A	BLCA	7.08
TCGA-XF-A8HH-01A	BLCA	6.74
TCGA-XF-A9SK-01A	BLCA	7.19
TCGA-E7-A5KE-01A	BLCA	7.59
TCGA-4Z-AA89-01A	BLCA	7.31
TCGA-4Z-AA80-01A	BLCA	7.45
TCGA-ZF-AA4W-01A	BLCA	7.73
TCGA-XF-A9SI-01A	BLCA	6.35
TCGA-BT-A0S7-01A	BLCA	7.54
TCGA-2F-A9KP-01A	BLCA	7.24
TCGA-DK-AA6Q-01A	BLCA	7.33
TCGA-ZF-A9RN-01A	BLCA	6.41
TCGA-4Z-AA7R-01A	BLCA	5.88
TCGA-ZF-AA52-01A	BLCA	6.44
TCGA-CU-A0YO-01A	BLCA	6.89
TCGA-C4-A0F6-01A	BLCA	6.9
TCGA-DK-A1AA-01A	BLCA	7.99
TCGA-G2-A2EF-01A	BLCA	7.41
TCGA-XF-A9ST-01A	BLCA	6.32
TCGA-2F-A9KW-01A	BLCA	6.91
TCGA-FD-A43N-01A	BLCA	6.35
TCGA-GV-A3QH-01A	BLCA	6.6
TCGA-HQ-A2OE-01A	BLCA	8.6
TCGA-GD-A3OS-01A	BLCA	6.59
TCGA-GV-A3JW-01A	BLCA	7.25
TCGA-UY-A9PA-01A	BLCA	7
TCGA-4Z-AA81-01A	BLCA	7.39
TCGA-K4-AAQO-01A	BLCA	7.33
TCGA-DK-A3X2-01A	BLCA	7.35
TCGA-GV-A3QF-01A	BLCA	6.75
TCGA-CF-A8HX-01A	BLCA	5.58
TCGA-E5-A2PC-01A	BLCA	6.3
TCGA-DK-A1AE-01A	BLCA	7.32
TCGA-K4-A5RJ-01A	BLCA	7.15
TCGA-DK-AA6S-01A	BLCA	6.1
TCGA-ZF-A9RC-01A	BLCA	5.98
TCGA-E7-A5KF-01A	BLCA	7.8
TCGA-5N-A9KI-01A	BLCA	6.19

TCGA-BT-A20Q-01A	BLCA	6.81
TCGA-G2-A2EK-01A	BLCA	6.5
TCGA-CU-A0YN-01A	BLCA	6.76
TCGA-KQ-A41N-01A	BLCA	6.29
TCGA-GC-A6I1-01A	BLCA	6.89
TCGA-XF-A9T6-01A	BLCA	6.92
TCGA-FD-A3SR-01A	BLCA	6.79
TCGA-FD-A43X-01A	BLCA	6.43
TCGA-FD-A3B3-01A	BLCA	6.53
TCGA-FD-A3B8-01A	BLCA	6.74
TCGA-FD-A6TF-01A	BLCA	6.68
TCGA-K4-A3WS-01A	BLCA	6.89
TCGA-FD-A6TK-01A	BLCA	7.57
TCGA-FJ-A3Z7-01A	BLCA	6.4
TCGA-HQ-A2OF-01A	BLCA	6.91
TCGA-BL-A13J-01B	BLCA	6.92
TCGA-FD-A43P-01A	BLCA	6.19
TCGA-FD-A3B6-01A	BLCA	6.98
TCGA-UY-A78O-01A	BLCA	6.51
TCGA-BT-A20W-01A	BLCA	6.94
TCGA-FD-A3SN-01A	BLCA	7.16
TCGA-DK-AA6X-01A	BLCA	6.54
TCGA-E7-A8O7-01A	BLCA	6.56
TCGA-E7-A7PW-01A	BLCA	5.23
TCGA-FD-A6TH-01A	BLCA	7.71
TCGA-BT-A20T-01A	BLCA	6.81
TCGA-A7-A26E-01B	BRCA	7.18
TCGA-A2-A0CU-01A	BRCA	7.28
TCGA-PL-A8LV-01A	BRCA	6.09
TCGA-BH-A0BC-01A	BRCA	7.83
TCGA-AR-A1AX-01A	BRCA	7.39
TCGA-AC-A2FO-01A	BRCA	9
TCGA-AQ-A0Y5-01A	BRCA	7.96
TCGA-AC-A3EH-01A	BRCA	7.13
TCGA-AC-A5EH-01A	BRCA	8.28
TCGA-D8-A142-01A	BRCA	6.16
TCGA-D8-A1XB-01A	BRCA	7.65
TCGA-D8-A1JJ-01A	BRCA	7.59
TCGA-E2-A10B-01A	BRCA	7.74
TCGA-A2-A0CP-01A	BRCA	7.26
TCGA-BH-A1FJ-01A	BRCA	8.93
TCGA-BH-A0C1-01B	BRCA	8.37
TCGA-OL-A66P-01A	BRCA	5.15
TCGA-E2-A14O-01A	BRCA	8.15
TCGA-D8-A1XJ-01A	BRCA	6.91
TCGA-AR-A24P-01A	BRCA	7.63
TCGA-AO-A1KQ-01A	BRCA	7.7
TCGA-D8-A145-01A	BRCA	8.08
TCGA-AR-A24V-01A	BRCA	8.1
TCGA-AO-A12F-01A	BRCA	6.59
TCGA-E9-A54X-01A	BRCA	7.73
TCGA-BH-A0BA-01A	BRCA	7.73
TCGA-EW-A1PH-01A	BRCA	6.92
TCGA-BH-A209-01A	BRCA	7.35
TCGA-E9-A2JS-01A	BRCA	6.92
TCGA-E9-A1R3-01A	BRCA	7.35
TCGA-E9-A1R6-01A	BRCA	8.81
TCGA-BH-A1FU-01A	BRCA	7.28

TCGA-D8-A1Y2-01A	BRCA	7.41
TCGA-A2-A4RX-01A	BRCA	7.17
TCGA-A8-A09V-01A	BRCA	8.32
TCGA-AO-A12B-01A	BRCA	8.36
TCGA-XX-A899-01A	BRCA	7.67
TCGA-A8-A08P-01A	BRCA	7.44
TCGA-C8-A26W-01A	BRCA	6.77
TCGA-E9-A249-01A	BRCA	8.1
TCGA-A8-A086-01A	BRCA	7.85
TCGA-B6-A0RV-01A	BRCA	8.17
TCGA-D8-A1JT-01A	BRCA	7.99
TCGA-OL-A5RV-01A	BRCA	7.77
TCGA-Z7-A8R5-01A	BRCA	7.01
TCGA-A8-A0AB-01A	BRCA	8.18
TCGA-E2-A154-01A	BRCA	8.37
TCGA-S3-A6ZG-01A	BRCA	7.82
TCGA-D8-A140-01A	BRCA	7.25
TCGA-A8-A06Q-01A	BRCA	7.19
TCGA-AR-A24Q-01A	BRCA	6.81
TCGA-A2-A1G6-01A	BRCA	8.34
TCGA-D8-A1XL-01A	BRCA	7.74
TCGA-A2-A1G0-01A	BRCA	8.44
TCGA-A8-A095-01A	BRCA	7.53
TCGA-AN-A0FV-01A	BRCA	7.63
TCGA-S3-AA12-01A	BRCA	8.62
TCGA-A8-A06T-01A	BRCA	8.88
TCGA-E2-A14Q-01A	BRCA	7.91
TCGA-BH-A18K-01A	BRCA	7.8
TCGA-A8-A06Y-01A	BRCA	7.57
TCGA-BH-A0AZ-01A	BRCA	7.19
TCGA-E9-A1RA-01A	BRCA	7.45
TCGA-UL-AAZ6-01A	BRCA	6.66
TCGA-BH-A1ES-01A	BRCA	8.82
TCGA-EW-A1PA-01A	BRCA	6.93
TCGA-EW-A1P4-01A	BRCA	7.15
TCGA-D8-A1JH-01A	BRCA	8
TCGA-B6-A409-01A	BRCA	6.02
TCGA-D8-A13Z-01A	BRCA	7.68
TCGA-AC-A7VB-01A	BRCA	7.49
TCGA-AN-A0XN-01A	BRCA	7.84
TCGA-GM-A2DN-01A	BRCA	7.14
TCGA-AO-A12G-01A	BRCA	6.73
TCGA-BH-A0B9-01A	BRCA	7.08
TCGA-C8-A273-01A	BRCA	7.22
TCGA-AR-A1AY-01A	BRCA	6.89
TCGA-AR-A1AO-01A	BRCA	7.2
TCGA-E9-A1RC-01A	BRCA	7.75
TCGA-A2-A0EN-01A	BRCA	7.27
TCGA-E9-A1RD-01A	BRCA	8.31
TCGA-A8-A08O-01A	BRCA	8.39
TCGA-AC-A2FE-01A	BRCA	7.56
TCGA-B6-A0RN-01A	BRCA	8.24
TCGA-A8-A076-01A	BRCA	7.04
TCGA-BH-A0HK-01A	BRCA	7.09
TCGA-BH-A0BO-01A	BRCA	8.06
TCGA-BH-A0HW-01A	BRCA	8.7
TCGA-D8-A1XZ-01A	BRCA	7.54
TCGA-OL-A66K-01A	BRCA	7.65

TCGA-AR-A24N-01A	BRCA	8.08
TCGA-E2-A1IU-01A	BRCA	8.64
TCGA-AR-A0TT-01A	BRCA	6.66
TCGA-B6-A0IP-01A	BRCA	7.86
TCGA-AO-A03L-01A	BRCA	7.51
TCGA-B6-A0IB-01A	BRCA	7.1
TCGA-E9-A54Y-01A	BRCA	6.63
TCGA-A2-A0T2-01A	BRCA	6.21
TCGA-AC-A3W6-01A	BRCA	7.32
TCGA-A2-A3XV-01A	BRCA	6.88
TCGA-BH-A0EA-01A	BRCA	7.75
TCGA-BH-A0B3-01A	BRCA	6.27
TCGA-5L-AAT1-01A	BRCA	8.07
TCGA-E2-A15J-01A	BRCA	7.87
TCGA-B6-A0WS-01A	BRCA	7.93
TCGA-BH-A0H7-01A	BRCA	7.32
TCGA-A2-A0YT-01A	BRCA	7.69
TCGA-AN-A0XO-01A	BRCA	7.64
TCGA-E2-A15K-06A	BRCA	8.28
TCGA-EW-A1P3-01A	BRCA	7.35
TCGA-A2-A04N-01A	BRCA	8.32
TCGA-D8-A13Y-01A	BRCA	6.65
TCGA-D8-A146-01A	BRCA	7.7
TCGA-LL-A7SZ-01A	BRCA	5.76
TCGA-AO-A0J8-01A	BRCA	7.68
TCGA-B6-A0IK-01A	BRCA	6.87
TCGA-A2-A0T3-01A	BRCA	7.5
TCGA-AO-A03M-01B	BRCA	7.32
TCGA-BH-A0W4-01A	BRCA	7.54
TCGA-E2-A15S-01A	BRCA	8.97
TCGA-D8-A27R-01A	BRCA	7.86
TCGA-B6-A0WZ-01A	BRCA	7.43
TCGA-D8-A27H-01A	BRCA	7.09
TCGA-AO-A03U-01B	BRCA	6.66
TCGA-A7-A5ZV-01A	BRCA	6.8
TCGA-BH-A1EY-01A	BRCA	6.93
TCGA-A8-A09T-01A	BRCA	8.27
TCGA-AC-A4ZE-01A	BRCA	7.78
TCGA-BH-A0H9-01A	BRCA	7.58
TCGA-LD-A7W6-01A	BRCA	7.86
TCGA-GM-A2D9-01A	BRCA	7.46
TCGA-BH-A42T-01A	BRCA	6.95
TCGA-A2-A0SX-01A	BRCA	7.27
TCGA-A1-A0SI-01A	BRCA	7.11
TCGA-A7-A0D9-01A	BRCA	7.9
TCGA-A2-A04U-01A	BRCA	6.99
TCGA-D8-A143-01A	BRCA	6.37
TCGA-BH-A8FZ-01A	BRCA	7.84
TCGA-A2-A0T6-01A	BRCA	8.16
TCGA-AC-A3BB-01A	BRCA	7.63
TCGA-BH-AB28-01A	BRCA	8.18
TCGA-A1-A0SQ-01A	BRCA	8.25
TCGA-E2-A15E-01A	BRCA	7.76
TCGA-AR-A24S-01A	BRCA	8.21
TCGA-D8-A1JS-01A	BRCA	8.5
TCGA-AO-A03O-01A	BRCA	7.92
TCGA-D8-A27F-01A	BRCA	6.28
TCGA-AR-A5QN-01A	BRCA	8.3

TCGA-B6-A0IA-01A	BRCA	7.89
TCGA-B6-A0I6-01A	BRCA	6.93
TCGA-B6-A0RE-01A	BRCA	7.09
TCGA-A8-A08F-01A	BRCA	6.9
TCGA-AR-A2LM-01A	BRCA	7.84
TCGA-AC-A62Y-01A	BRCA	7.24
TCGA-AO-A0JD-01A	BRCA	7.29
TCGA-A2-A0ST-01A	BRCA	7.07
TCGA-A2-A4RW-01A	BRCA	6.91
TCGA-B6-A0RM-01A	BRCA	9.13
TCGA-A1-A0SN-01A	BRCA	7.19
TCGA-LL-A6FR-01A	BRCA	6.22
TCGA-D8-A1JE-01A	BRCA	8.1
TCGA-A8-A09I-01A	BRCA	7.39
TCGA-BH-A0DP-01A	BRCA	8.26
TCGA-HN-A2NL-01A	BRCA	6.83
TCGA-AR-A24O-01A	BRCA	7.63
TCGA-BH-A1FL-01A	BRCA	8.22
TCGA-V7-A7HQ-01A	BRCA	7.23
TCGA-B6-A1KC-01B	BRCA	8.61
TCGA-A7-A3IZ-01A	BRCA	9.15
TCGA-B6-A1KI-01A	BRCA	7.6
TCGA-BH-A1FH-01A	BRCA	7.31
TCGA-B6-A0X1-01A	BRCA	7.28
TCGA-B6-A0IN-01A	BRCA	7.01
TCGA-E2-A15K-01A	BRCA	7.6
TCGA-AR-A1AL-01A	BRCA	7.92
TCGA-E2-A570-01A	BRCA	7.23
TCGA-C8-A275-01A	BRCA	7.74
TCGA-E2-A1LA-01A	BRCA	7.89
TCGA-E9-A229-01A	BRCA	7.56
TCGA-D8-A1Y0-01A	BRCA	8.01
TCGA-E9-A22D-01A	BRCA	7.37
TCGA-AN-A0XP-01A	BRCA	7.58
TCGA-A8-A07O-01A	BRCA	8.03
TCGA-S3-AA0Z-01A	BRCA	6.74
TCGA-AO-A0J2-01A	BRCA	6.28
TCGA-EW-A2FS-01A	BRCA	6.46
TCGA-BH-A0B8-01A	BRCA	8.62
TCGA-BH-A0E7-01A	BRCA	8.35
TCGA-BH-A0DO-01B	BRCA	8.16
TCGA-AR-A256-01A	BRCA	7.7
TCGA-A7-A426-01A	BRCA	7.17
TCGA-E9-A243-01A	BRCA	6.92
TCGA-BH-A1FG-01A	BRCA	8.18
TCGA-D8-A27N-01A	BRCA	7.24
TCGA-AN-A0FF-01A	BRCA	7.63
TCGA-AO-A0JL-01A	BRCA	5.01
TCGA-AC-A8OS-01A	BRCA	7.18
TCGA-D8-A1J9-01A	BRCA	8.04
TCGA-AC-A62V-01A	BRCA	6.86
TCGA-A2-A0EY-01A	BRCA	7.27
TCGA-A7-A0CJ-01A	BRCA	6.08
TCGA-GM-A2DF-01A	BRCA	5.92
TCGA-BH-A0BJ-01A	BRCA	7.22
TCGA-B6-A0WT-01A	BRCA	7.28
TCGA-E2-A159-01A	BRCA	7.08
TCGA-A8-A08Z-01A	BRCA	7.59

TCGA-A8-A096-01A	BRCA	8.15
TCGA-E2-A14Z-01A	BRCA	7.14
TCGA-A7-A4SF-01A	BRCA	7.33
TCGA-E2-A1BC-01A	BRCA	8.02
TCGA-A8-A08B-01A	BRCA	7.27
TCGA-E2-A1LH-01A	BRCA	5.69
TCGA-LL-A7T0-01A	BRCA	7.04
TCGA-AC-A5XS-01A	BRCA	6.98
TCGA-B6-A400-01A	BRCA	7.13
TCGA-EW-A3U0-01A	BRCA	6.28
TCGA-AC-A2QJ-01A	BRCA	5.37
TCGA-AR-A0TY-01A	BRCA	8.36
TCGA-EW-A1PG-01A	BRCA	7.72
TCGA-A7-A3J0-01A	BRCA	7.93
TCGA-E2-A15C-01A	BRCA	8.2
TCGA-BH-A0W5-01A	BRCA	7.7
TCGA-AR-A1AV-01A	BRCA	7.86
TCGA-OL-A6VR-01A	BRCA	8.54
TCGA-BH-A18Q-01A	BRCA	7.41
TCGA-BH-A5J0-01A	BRCA	7.43
TCGA-AR-A24R-01A	BRCA	7.61
TCGA-A8-A08H-01A	BRCA	7.63
TCGA-BH-A0DD-01A	BRCA	7.75
TCGA-E2-A1B4-01A	BRCA	8.79
TCGA-C8-A274-01A	BRCA	7.97
TCGA-A7-A4SE-01A	BRCA	6.76
TCGA-C8-A26Z-01A	BRCA	9.07
TCGA-E2-A10E-01A	BRCA	8.78
TCGA-BH-A0HU-01A	BRCA	7.58
TCGA-A2-A0SY-01A	BRCA	7.76
TCGA-C8-A8HR-01A	BRCA	7.26
TCGA-AN-A046-01A	BRCA	7.72
TCGA-D8-A1JF-01A	BRCA	7.51
TCGA-A8-A097-01A	BRCA	6.95
TCGA-C8-A12M-01A	BRCA	7.99
TCGA-E9-A1RI-01A	BRCA	8.29
TCGA-GM-A2DC-01A	BRCA	6.35
TCGA-E9-A226-01A	BRCA	8.33
TCGA-AO-A03T-01A	BRCA	5.52
TCGA-D8-A1JB-01A	BRCA	7.22
TCGA-BH-A208-01A	BRCA	7.16
TCGA-A7-A26H-01A	BRCA	7.79
TCGA-D8-A1XG-01A	BRCA	8.63
TCGA-AR-A24L-01A	BRCA	7.07
TCGA-AN-A0FY-01A	BRCA	7.59
TCGA-A2-A3XY-01A	BRCA	6.56
TCGA-AC-A8OQ-01A	BRCA	6.24
TCGA-AC-A23C-01A	BRCA	7.1
TCGA-AR-A0TU-01A	BRCA	7.44
TCGA-A8-A09E-01A	BRCA	7.49
TCGA-BH-A0HI-01A	BRCA	7.81
TCGA-E2-A14T-01A	BRCA	7.2
TCGA-E2-A1B6-01A	BRCA	6.92
TCGA-BH-A0C7-01B	BRCA	7.78
TCGA-AN-A0FW-01A	BRCA	6.66
TCGA-LL-A8F5-01A	BRCA	5.51
TCGA-AO-A0JI-01A	BRCA	9.54
TCGA-E9-A3Q9-01A	BRCA	7.17

TCGA-AC-A3HN-01A	BRCA	7.47
TCGA-E9-A5FL-01A	BRCA	6.52
TCGA-A2-A1G4-01A	BRCA	8.15
TCGA-AR-A0U0-01A	BRCA	6.93
TCGA-AR-A2LE-01A	BRCA	7.32
TCGA-E2-A14P-01A	BRCA	7.15
TCGA-AN-A04A-01A	BRCA	7.54
TCGA-BH-A1F5-01A	BRCA	8.82
TCGA-BH-A0HA-01A	BRCA	7.19
TCGA-E2-A15T-01A	BRCA	8.57
TCGA-BH-A0BF-01A	BRCA	7.66
TCGA-EW-A2FW-01A	BRCA	8.57
TCGA-E9-A1NC-01A	BRCA	6.93
TCGA-S3-AA15-01A	BRCA	5.39
TCGA-BH-A202-01A	BRCA	6.69
TCGA-AC-A23E-01A	BRCA	8.37
TCGA-WT-AB41-01A	BRCA	7.22
TCGA-BH-A0RX-01A	BRCA	6.66
TCGA-EW-A1IX-01A	BRCA	7.94
TCGA-A8-A07L-01A	BRCA	8.18
TCGA-A7-A6VY-01A	BRCA	6.27
TCGA-AR-A255-01A	BRCA	8.26
TCGA-OL-A66N-01A	BRCA	7.06
TCGA-A8-A099-01A	BRCA	8.77
TCGA-LL-A5YM-01A	BRCA	5.89
TCGA-AO-A0J6-01A	BRCA	6.38
TCGA-LL-A73Y-01A	BRCA	7.6
TCGA-D8-A73W-01A	BRCA	7.78
TCGA-E2-A14W-01A	BRCA	7.76
TCGA-A2-A25B-01A	BRCA	7.69
TCGA-E2-A15O-01A	BRCA	7.26
TCGA-A2-A0CM-01A	BRCA	7.55
TCGA-D8-A1X5-01A	BRCA	7.59
TCGA-AN-A0AR-01A	BRCA	6.75
TCGA-AR-A1AM-01A	BRCA	6.71
TCGA-E2-A1IH-01A	BRCA	7.44
TCGA-A7-A4SC-01A	BRCA	6.41
TCGA-S3-A6ZH-01A	BRCA	7.54
TCGA-BH-A0EB-01A	BRCA	7.57
TCGA-E2-A15F-01A	BRCA	8.11
TCGA-BH-A1FB-01A	BRCA	8.04
TCGA-E9-A5UO-01A	BRCA	6.56
TCGA-EW-A1J1-01A	BRCA	7.64
TCGA-D8-A1XC-01A	BRCA	8.66
TCGA-E2-A156-01A	BRCA	8.91
TCGA-EW-A1P6-01A	BRCA	8.32
TCGA-E2-A1L8-01A	BRCA	8.03
TCGA-E2-A109-01A	BRCA	8.07
TCGA-LL-A5YN-01A	BRCA	6.61
TCGA-E2-A2P5-01A	BRCA	8.01
TCGA-A7-A0DB-01A	BRCA	7.04
TCGA-A7-A0DB-01C	BRCA	7.23
TCGA-E2-A1LG-01A	BRCA	7.73
TCGA-B6-A0WW-01A	BRCA	8.06
TCGA-E2-A576-01A	BRCA	6.4
TCGA-AC-A3OD-01A	BRCA	6.42
TCGA-A2-A04R-01A	BRCA	7.74
TCGA-E9-A1R0-01A	BRCA	7.91

TCGA-B6-A1KF-01A	BRCA	6.4
TCGA-EW-A1PC-01B	BRCA	6.51
TCGA-EW-A2FV-01A	BRCA	7.2
TCGA-AO-A0JF-01A	BRCA	8.08
TCGA-AN-A0XR-01A	BRCA	7.58
TCGA-GM-A5PX-01A	BRCA	7.74
TCGA-B6-A0RH-01A	BRCA	6.95
TCGA-A2-A0CX-01A	BRCA	7.05
TCGA-AR-A0TZ-01A	BRCA	7.7
TCGA-AR-A2LR-01A	BRCA	6.54
TCGA-BH-A0B6-01A	BRCA	7.79
TCGA-A2-A3XZ-01A	BRCA	6.56
TCGA-AR-A0TR-01A	BRCA	7.94
TCGA-E9-A295-01A	BRCA	8.05
TCGA-A2-A04P-01A	BRCA	5.52
TCGA-BH-A0DS-01A	BRCA	7.84
TCGA-E9-A1N5-01A	BRCA	7.73
TCGA-S3-A6ZF-01A	BRCA	7.16
TCGA-A2-A1FV-01A	BRCA	8.24
TCGA-D8-A147-01A	BRCA	7.72
TCGA-BH-A0HP-01A	BRCA	9.14
TCGA-B6-A40C-01A	BRCA	6.78
TCGA-OK-A5Q2-01A	BRCA	7.41
TCGA-BH-A1ES-06A	BRCA	7.72
TCGA-BH-A18P-01A	BRCA	8.01
TCGA-AR-A252-01A	BRCA	7.84
TCGA-C8-A1HK-01A	BRCA	7.75
TCGA-AQ-A54N-01A	BRCA	7.41
TCGA-A2-A1FW-01A	BRCA	8.89
TCGA-3C-AALJ-01A	BRCA	6.83
TCGA-AC-A62X-01A	BRCA	6.84
TCGA-B6-A0X7-01A	BRCA	8.06
TCGA-BH-A0BW-01A	BRCA	6.7
TCGA-D8-A1XT-01A	BRCA	7.2
TCGA-AR-A254-01A	BRCA	7.32
TCGA-D8-A1Y3-01A	BRCA	7.25
TCGA-BH-A0HL-01A	BRCA	8.18
TCGA-E9-A247-01A	BRCA	9.19
TCGA-C8-A27A-01A	BRCA	7.2
TCGA-A7-A26F-01B	BRCA	5.85
TCGA-AO-A0J3-01A	BRCA	8.18
TCGA-A2-A25D-01A	BRCA	7.71
TCGA-AN-A0XW-01A	BRCA	7.11
TCGA-D8-A141-01A	BRCA	7.61
TCGA-AR-A1AN-01A	BRCA	7.75
TCGA-UU-A93S-01A	BRCA	6.25
TCGA-BH-A18S-01A	BRCA	8.58
TCGA-OL-A5S0-01A	BRCA	6.91
TCGA-LL-A442-01A	BRCA	7.85
TCGA-A8-A09W-01A	BRCA	8.27
TCGA-C8-A1HI-01A	BRCA	7.46
TCGA-BH-A0BZ-01A	BRCA	7.24
TCGA-E2-A56Z-01A	BRCA	7
TCGA-A2-A259-01A	BRCA	8.48
TCGA-AR-A24X-01A	BRCA	8.06
TCGA-BH-A1FD-01A	BRCA	7.96
TCGA-BH-A0DZ-01A	BRCA	7.54
TCGA-B6-A0RT-01A	BRCA	7.06

TCGA-AR-A2LQ-01A	BRCA	7.3
TCGA-E2-A150-01A	BRCA	6.51
TCGA-E2-A14R-01A	BRCA	6.13
TCGA-A8-A0A6-01A	BRCA	7.63
TCGA-A7-A26F-01A	BRCA	5.81
TCGA-AO-A0JM-01A	BRCA	7.18
TCGA-A8-A09B-01A	BRCA	7.48
TCGA-BH-A0BR-01A	BRCA	7.68
TCGA-A2-A0T7-01A	BRCA	7.46
TCGA-B6-A401-01A	BRCA	6.78
TCGA-BH-A0DH-01A	BRCA	7.47
TCGA-BH-A1EV-01A	BRCA	7.52
TCGA-EW-A6SD-01A	BRCA	6.61
TCGA-AC-A3W5-01A	BRCA	6.9
TCGA-BH-A0BG-01A	BRCA	7.55
TCGA-A2-A0CR-01A	BRCA	6.87
TCGA-3C-AALI-01A	BRCA	7.04
TCGA-B6-A0RL-01A	BRCA	8.19
TCGA-A7-A5ZW-01A	BRCA	8.04
TCGA-E2-A15H-01A	BRCA	7.68
TCGA-AQ-A1H2-01A	BRCA	8.47
TCGA-D8-A1JD-01A	BRCA	8.41
TCGA-BH-A0B2-01A	BRCA	7.73
TCGA-AR-A1AT-01A	BRCA	7.13
TCGA-D8-A1JK-01A	BRCA	6.34
TCGA-C8-A26Y-01A	BRCA	7.48
TCGA-A7-A6VV-01A	BRCA	6.45
TCGA-A2-A3XX-01A	BRCA	6.57
TCGA-GM-A2DH-01A	BRCA	7.2
TCGA-D8-A1X6-01A	BRCA	8.01
TCGA-B6-A0I1-01A	BRCA	6.25
TCGA-A8-A09M-01A	BRCA	6.96
TCGA-EW-A1IY-01A	BRCA	7.77
TCGA-A8-A07F-01A	BRCA	8.23
TCGA-BH-A8FY-01A	BRCA	7.54
TCGA-5T-A9QA-01A	BRCA	8.51
TCGA-A2-A0EM-01A	BRCA	8.18
TCGA-AO-A12D-01A	BRCA	6.73
TCGA-GI-A2C8-01A	BRCA	7.93
TCGA-OL-A6VQ-01A	BRCA	7.69
TCGA-D8-A1X9-01A	BRCA	7.57
TCGA-A1-A0SB-01A	BRCA	7.46
TCGA-BH-A28Q-01A	BRCA	8.56
TCGA-A2-A04W-01A	BRCA	6.35
TCGA-B6-A0I2-01A	BRCA	6.55
TCGA-LL-A440-01A	BRCA	7.26
TCGA-A7-A26E-01A	BRCA	7.37
TCGA-EW-A1P8-01A	BRCA	6.42
TCGA-OL-A66I-01A	BRCA	6.94
TCGA-AO-A1KP-01A	BRCA	8.99
TCGA-A8-A092-01A	BRCA	7.81
TCGA-E2-A15L-01A	BRCA	8.47
TCGA-BH-A0HX-01A	BRCA	8.13
TCGA-E9-A22G-01A	BRCA	6.77
TCGA-E9-A248-01A	BRCA	6.71
TCGA-A8-A07I-01A	BRCA	7.7
TCGA-BH-A42V-01A	BRCA	7.76
TCGA-AN-A0FJ-01A	BRCA	7.83

TCGA-AO-A0JG-01A	BRCA	8.11
TCGA-E2-A108-01A	BRCA	7.15
TCGA-GM-A2DO-01A	BRCA	7.02
TCGA-A8-A082-01A	BRCA	7.45
TCGA-EW-A6SB-01A	BRCA	6.08
TCGA-D8-A27L-01A	BRCA	7.69
TCGA-EW-A1PD-01A	BRCA	7.7
TCGA-B6-A0IM-01A	BRCA	7.05
TCGA-EW-A1P5-01A	BRCA	7.07
TCGA-B6-A0X4-01A	BRCA	7.67
TCGA-EW-A1OY-01A	BRCA	7.69
TCGA-OL-A5DA-01A	BRCA	8
TCGA-B6-A0X5-01A	BRCA	8.39
TCGA-EW-A1IW-01A	BRCA	7.98
TCGA-LQ-A4E4-01A	BRCA	7.27
TCGA-A2-A0YM-01A	BRCA	7.37
TCGA-A8-A07U-01A	BRCA	6.27
TCGA-BH-A8G0-01A	BRCA	7.03
TCGA-AR-A0TV-01A	BRCA	7.53
TCGA-E9-A22E-01A	BRCA	7.23
TCGA-C8-A278-01A	BRCA	7.32
TCGA-AR-A0TQ-01A	BRCA	7.68
TCGA-AO-A0JB-01A	BRCA	5.8
TCGA-D8-A1XQ-01A	BRCA	6.26
TCGA-B6-A0I9-01A	BRCA	7.11
TCGA-A2-A0D3-01A	BRCA	8.34
TCGA-A1-A0SF-01A	BRCA	8.12
TCGA-BH-A1FE-06A	BRCA	7.34
TCGA-AN-A04D-01A	BRCA	6.63
TCGA-E9-A22A-01A	BRCA	7.54
TCGA-S3-AA17-01A	BRCA	7.27
TCGA-OL-A5RX-01A	BRCA	7.52
TCGA-BH-A1EO-01A	BRCA	7.96
TCGA-BH-A6R9-01A	BRCA	6.97
TCGA-B6-A0RP-01A	BRCA	8.46
TCGA-A8-A0A1-01A	BRCA	7.93
TCGA-B6-A0IE-01A	BRCA	7.94
TCGA-C8-A12L-01A	BRCA	6.49
TCGA-E9-A1RH-01A	BRCA	6.91
TCGA-AN-A0XL-01A	BRCA	7.68
TCGA-AC-A2FF-01A	BRCA	7.83
TCGA-A2-A4S3-01A	BRCA	7.49
TCGA-BH-A1FE-01A	BRCA	7.86
TCGA-E9-A1NE-01A	BRCA	6.91
TCGA-E2-A10A-01A	BRCA	7.8
TCGA-C8-A12Z-01A	BRCA	7.91
TCGA-A2-A4RY-01A	BRCA	7.49
TCGA-LL-A6FP-01A	BRCA	7.54
TCGA-AR-A24K-01A	BRCA	8.53
TCGA-E2-A15M-01A	BRCA	7.66
TCGA-A2-A0CK-01A	BRCA	7.19
TCGA-HN-A2OB-01A	BRCA	7.64
TCGA-WT-AB44-01A	BRCA	7.38
TCGA-E2-A573-01A	BRCA	6.45
TCGA-E2-A1L9-01A	BRCA	7.67
TCGA-A7-A26G-01A	BRCA	6.58
TCGA-D8-A1J8-01A	BRCA	7.9
TCGA-A2-A0CL-01A	BRCA	6.83

TCGA-A2-A04V-01A	BRCA	8
TCGA-EW-A1J5-01A	BRCA	7.25
TCGA-BH-A0GZ-01A	BRCA	6.71
TCGA-E2-A14U-01A	BRCA	8.38
TCGA-B6-A0IJ-01A	BRCA	6.78
TCGA-B6-A1KN-01A	BRCA	7.3
TCGA-E2-A15G-01A	BRCA	8.44
TCGA-A8-A08S-01A	BRCA	7.91
TCGA-A8-A09X-01A	BRCA	7.03
TCGA-AO-A03R-01A	BRCA	6.8
TCGA-E2-A1IE-01A	BRCA	7.34
TCGA-A2-A0EQ-01A	BRCA	7.4
TCGA-E9-A1R5-01A	BRCA	8.59
TCGA-S3-AA10-01A	BRCA	7.35
TCGA-LL-A5YL-01A	BRCA	7.32
TCGA-BH-A0E0-01A	BRCA	6.32
TCGA-B6-A0I5-01A	BRCA	6.95
TCGA-BH-A18U-01A	BRCA	7.28
TCGA-A8-A06Z-01A	BRCA	7.89
TCGA-D8-A1JN-01A	BRCA	8.61
TCGA-A7-A13H-01A	BRCA	7.36
TCGA-C8-A12Y-01A	BRCA	7.95
TCGA-BH-A0BS-01A	BRCA	8.54
TCGA-A7-A13F-01A	BRCA	7.36
TCGA-AC-A7VC-01A	BRCA	6.74
TCGA-AO-A03P-01A	BRCA	6.74
TCGA-BH-A1F0-01A	BRCA	6.98
TCGA-AR-A1AJ-01A	BRCA	6.72
TCGA-BH-A0EI-01A	BRCA	8.25
TCGA-AC-A3W7-01A	BRCA	7.51
TCGA-E2-A1LL-01A	BRCA	7.14
TCGA-E9-A1R7-01A	BRCA	6.63
TCGA-A8-A0A4-01A	BRCA	8.04
TCGA-E2-A1L6-01A	BRCA	7.86
TCGA-AO-A12E-01A	BRCA	7.88
TCGA-A2-A3KC-01A	BRCA	7.75
TCGA-BH-A18T-01A	BRCA	6.62
TCGA-AN-A0FK-01A	BRCA	8.62
TCGA-B6-A0IG-01A	BRCA	7.74
TCGA-A8-A08T-01A	BRCA	7.9
TCGA-D8-A1XU-01A	BRCA	7.18
TCGA-E2-A3DX-01A	BRCA	7.54
TCGA-A2-A0YD-01A	BRCA	7.54
TCGA-BH-A42U-01A	BRCA	7.14
TCGA-A8-A07W-01A	BRCA	7.57
TCGA-A8-A06N-01A	BRCA	8.22
TCGA-A8-A079-01A	BRCA	7.14
TCGA-AN-A0XT-01A	BRCA	8.14
TCGA-AR-A5QP-01A	BRCA	8.31
TCGA-BH-A0W3-01A	BRCA	8.62
TCGA-AN-A0AM-01A	BRCA	7.66
TCGA-A8-A0A2-01A	BRCA	7.12
TCGA-A8-A09N-01A	BRCA	8.2
TCGA-A8-A07J-01A	BRCA	7.93
TCGA-E2-A15R-01A	BRCA	9.18
TCGA-AR-A1AU-01A	BRCA	7.6
TCGA-A8-A09D-01A	BRCA	7.85
TCGA-AN-A041-01A	BRCA	7.55

TCGA-A8-A084-01A	BRCA	7.48
TCGA-BH-A201-01A	BRCA	7.75
TCGA-A8-A085-01A	BRCA	7.95
TCGA-LD-A66U-01A	BRCA	6.96
TCGA-AR-A5QQ-01A	BRCA	6.58
TCGA-A7-A0DA-01A	BRCA	6.81
TCGA-AR-A2LH-01A	BRCA	6.94
TCGA-E9-A1RE-01A	BRCA	9.28
TCGA-AR-A24U-01A	BRCA	6.78
TCGA-D8-A27T-01A	BRCA	7.44
TCGA-B6-A0IH-01A	BRCA	7.89
TCGA-A2-A0YH-01A	BRCA	7.56
TCGA-BH-A0DI-01A	BRCA	7.21
TCGA-E2-A1LI-01A	BRCA	6.25
TCGA-A2-A0CW-01A	BRCA	7.96
TCGA-E2-A14V-01A	BRCA	7.95
TCGA-AQ-A54O-01A	BRCA	6.66
TCGA-C8-A135-01A	BRCA	7.85
TCGA-GM-A2DK-01A	BRCA	6.77
TCGA-BH-A0AU-01A	BRCA	8.63
TCGA-AO-A0JE-01A	BRCA	7.21
TCGA-A2-A0D0-01A	BRCA	6.5
TCGA-LL-A73Z-01A	BRCA	6.59
TCGA-A8-A09G-01A	BRCA	7.3
TCGA-AN-A0AL-01A	BRCA	6
TCGA-A2-A0CT-01A	BRCA	8.39
TCGA-AN-A049-01A	BRCA	7.26
TCGA-LD-A74U-01A	BRCA	7.54
TCGA-D8-A1Y1-01A	BRCA	8.12
TCGA-A2-A0T5-01A	BRCA	7.52
TCGA-D8-A1JM-01A	BRCA	8.86
TCGA-OL-A6VO-01A	BRCA	6.51
TCGA-AC-A2FG-01A	BRCA	8.3
TCGA-E9-A22H-01A	BRCA	7.78
TCGA-B6-A0X0-01A	BRCA	8.02
TCGA-D8-A73X-01A	BRCA	8.24
TCGA-AQ-A7U7-01A	BRCA	6.67
TCGA-A2-A3Y0-01A	BRCA	5.51
TCGA-E2-A1LK-01A	BRCA	7.06
TCGA-BH-A0B7-01A	BRCA	6.97
TCGA-AR-A2LK-01A	BRCA	7.76
TCGA-AO-A1KT-01A	BRCA	8.26
TCGA-BH-A0HF-01A	BRCA	8.07
TCGA-A8-A09R-01A	BRCA	7.01
TCGA-AR-A1AR-01A	BRCA	7.14
TCGA-A7-A0DC-01A	BRCA	8.36
TCGA-A7-A0DC-01B	BRCA	7.81
TCGA-BH-A1F8-01A	BRCA	7.84
TCGA-A7-A56D-01A	BRCA	6.14
TCGA-E9-A1ND-01A	BRCA	6.09
TCGA-C8-A27B-01A	BRCA	6.69
TCGA-C8-A134-01A	BRCA	4.91
TCGA-E2-A107-01A	BRCA	7.52
TCGA-BH-A0BP-01A	BRCA	7.68
TCGA-A8-A091-01A	BRCA	8.33
TCGA-AC-A3OD-01B	BRCA	6.43
TCGA-EW-A1PE-01A	BRCA	7.84
TCGA-BH-A0DK-01A	BRCA	6.71

TCGA-A8-A06R-01A	BRCA	6.55
TCGA-E9-A3QA-01A	BRCA	6.71
TCGA-EW-A1P1-01A	BRCA	7.54
TCGA-E2-A1IN-01A	BRCA	7.64
TCGA-BH-A0HQ-01A	BRCA	7.66
TCGA-B6-A0WY-01A	BRCA	7.8
TCGA-A8-A090-01A	BRCA	8.82
TCGA-A8-A08X-01A	BRCA	5.7
TCGA-3C-AALK-01A	BRCA	6.92
TCGA-EW-A1OW-01A	BRCA	6.33
TCGA-A2-A0CS-01A	BRCA	8.16
TCGA-BH-A18M-01A	BRCA	7.6
TCGA-E2-A1LB-01A	BRCA	8.21
TCGA-PL-A8LX-01A	BRCA	6.54
TCGA-E2-A2P6-01A	BRCA	7.51
TCGA-BH-A18R-01A	BRCA	8.61
TCGA-BH-A0B0-01A	BRCA	8
TCGA-GM-A2DI-01A	BRCA	7.78
TCGA-GM-A5PV-01A	BRCA	8.43
TCGA-A7-A0CG-01A	BRCA	8.24
TCGA-AC-A2QH-01B	BRCA	5.38
TCGA-GM-A4E0-01A	BRCA	6.89
TCGA-AC-A2QH-01A	BRCA	5.33
TCGA-GM-A3XL-01A	BRCA	6.18
TCGA-D8-A1XO-01A	BRCA	8.23
TCGA-BH-A1F2-01A	BRCA	7.39
TCGA-A7-A26J-01A	BRCA	7.89
TCGA-AR-A24H-01A	BRCA	8.02
TCGA-C8-A8HQ-01A	BRCA	7.32
TCGA-B6-A0RU-01A	BRCA	7.01
TCGA-OL-A5D8-01A	BRCA	7.59
TCGA-OL-A97C-01A	BRCA	7.46
TCGA-GM-A3XG-01A	BRCA	7.55
TCGA-AR-A2LJ-01A	BRCA	7.09
TCGA-GM-A3NW-01A	BRCA	8.86
TCGA-A2-A0EV-01A	BRCA	6.81
TCGA-C8-A26X-01A	BRCA	6.36
TCGA-D8-A1XF-01A	BRCA	7.46
TCGA-A2-A0YI-01A	BRCA	7.66
TCGA-D8-A27G-01A	BRCA	7.65
TCGA-OL-A66H-01A	BRCA	7.37
TCGA-B6-A0WV-01A	BRCA	8.26
TCGA-BH-A0DE-01A	BRCA	7.56
TCGA-E2-A1L7-01A	BRCA	6.75
TCGA-D8-A1JG-01B	BRCA	6.95
TCGA-A8-A075-01A	BRCA	7.75
TCGA-JL-A3YX-01A	BRCA	7.39
TCGA-A2-A4S1-01A	BRCA	6.49
TCGA-C8-A12V-01A	BRCA	6.33
TCGA-D8-A4Z1-01A	BRCA	8.04
TCGA-EW-A1P0-01A	BRCA	8.36
TCGA-BH-A0WA-01A	BRCA	6.9
TCGA-AQ-A1H3-01A	BRCA	7.88
TCGA-A7-A13E-01A	BRCA	7.74
TCGA-BH-A0HO-01A	BRCA	7.73
TCGA-D8-A1XW-01A	BRCA	6.89
TCGA-A8-A07P-01A	BRCA	8.21
TCGA-E2-A10F-01A	BRCA	7.66

TCGA-C8-A12O-01A	BRCA	8.24
TCGA-C8-A132-01A	BRCA	8.68
TCGA-E2-A1B1-01A	BRCA	7.97
TCGA-E9-A1RG-01A	BRCA	7.49
TCGA-E2-A1AZ-01A	BRCA	7.76
TCGA-A7-A3J1-01A	BRCA	7.73
TCGA-AR-A0TW-01A	BRCA	5.84
TCGA-C8-A8HP-01A	BRCA	6.32
TCGA-MS-A51U-01A	BRCA	7.73
TCGA-LL-A6FQ-01A	BRCA	7.05
TCGA-A8-A094-01A	BRCA	7.14
TCGA-AR-A1AI-01A	BRCA	6.38
TCGA-C8-A3M7-01A	BRCA	8.04
TCGA-AN-A0AJ-01A	BRCA	7.32
TCGA-BH-A1EW-01A	BRCA	7.95
TCGA-AR-A24T-01A	BRCA	8.23
TCGA-AR-A1AW-01A	BRCA	8.01
TCGA-GI-A2C9-01A	BRCA	6.96
TCGA-A7-A0CE-01A	BRCA	7.21
TCGA-A2-A0YC-01A	BRCA	7.86
TCGA-AN-A0XU-01A	BRCA	7.36
TCGA-AR-A24Z-01A	BRCA	8.16
TCGA-AR-A24M-01A	BRCA	8.29
TCGA-C8-A1HL-01A	BRCA	7.41
TCGA-EW-A1OV-01A	BRCA	7.74
TCGA-A1-A0SH-01A	BRCA	7.7
TCGA-A2-A1FX-01A	BRCA	7.83
TCGA-D8-A1JL-01A	BRCA	7.03
TCGA-AC-A3YI-01A	BRCA	7
TCGA-AO-A03V-01A	BRCA	7.72
TCGA-AR-A1AH-01A	BRCA	7.42
TCGA-A2-A25A-01A	BRCA	7.6
TCGA-BH-A0DQ-01A	BRCA	7.51
TCGA-BH-A1F6-01A	BRCA	6.72
TCGA-EW-A6SA-01A	BRCA	8.03
TCGA-A7-A4SB-01A	BRCA	7.38
TCGA-E9-A1N8-01A	BRCA	7.61
TCGA-Z7-A8R6-01A	BRCA	6.16
TCGA-A8-A09A-01A	BRCA	7.92
TCGA-A7-A5ZX-01A	BRCA	7.47
TCGA-C8-A12N-01A	BRCA	7.73
TCGA-PE-A5DD-01A	BRCA	7.74
TCGA-BH-A2L8-01A	BRCA	7.47
TCGA-AC-A2FM-01A	BRCA	8.15
TCGA-A1-A0SE-01A	BRCA	7.66
TCGA-AC-A8OP-01A	BRCA	6.98
TCGA-GM-A3NY-01A	BRCA	6.59
TCGA-E2-A153-01A	BRCA	7.53
TCGA-A2-A3XW-01A	BRCA	6.53
TCGA-E9-A1RF-01A	BRCA	6.46
TCGA-AC-A2QI-01A	BRCA	7.11
TCGA-BH-A0BM-01A	BRCA	7.75
TCGA-AO-A129-01A	BRCA	6.9
TCGA-XX-A89A-01A	BRCA	7.24
TCGA-A2-A0YG-01A	BRCA	6.7
TCGA-EW-A3E8-01B	BRCA	7.45
TCGA-4H-AAAK-01A	BRCA	7.91
TCGA-A1-A0SO-01A	BRCA	6.65

TCGA-BH-A18N-01A	BRCA	8.22
TCGA-A8-A06P-01A	BRCA	7.8
TCGA-EW-A1OX-01A	BRCA	7.37
TCGA-A2-A0EP-01A	BRCA	7.1
TCGA-AR-A24W-01A	BRCA	7.91
TCGA-E2-A15I-01A	BRCA	8.24
TCGA-D8-A27E-01A	BRCA	8.69
TCGA-A8-A08J-01A	BRCA	7.77
TCGA-C8-A12X-01A	BRCA	7.86
TCGA-AO-A12C-01A	BRCA	7.71
TCGA-BH-A0H3-01A	BRCA	8.07
TCGA-LL-A441-01A	BRCA	6.39
TCGA-3C-AAAU-01A	BRCA	6.84
TCGA-E2-A1IO-01A	BRCA	8.05
TCGA-BH-A18V-06A	BRCA	6.56
TCGA-E2-A15A-01A	BRCA	8.01
TCGA-A7-A3RF-01A	BRCA	7.69
TCGA-GM-A2DM-01A	BRCA	7.73
TCGA-AC-A6IX-01A	BRCA	7.71
TCGA-D8-A3Z5-01A	BRCA	7.45
TCGA-AC-A2FK-01A	BRCA	8.02
TCGA-AR-A1AS-01A	BRCA	7.61
TCGA-AQ-A04L-01B	BRCA	8.34
TCGA-AC-A3TN-01A	BRCA	7.61
TCGA-AC-A23H-01A	BRCA	7.55
TCGA-A8-A08A-01A	BRCA	7.68
TCGA-A2-A0YE-01A	BRCA	6.25
TCGA-A2-A1FZ-01A	BRCA	7.89
TCGA-BH-A28O-01A	BRCA	7.3
TCGA-E9-A3HO-01A	BRCA	6.84
TCGA-E9-A245-01A	BRCA	7.44
TCGA-BH-A1EX-01A	BRCA	7.5
TCGA-D8-A1XV-01A	BRCA	7.73
TCGA-AO-A125-01A	BRCA	7.82
TCGA-AC-A6IW-01A	BRCA	6.68
TCGA-A7-A13E-01B	BRCA	7.27
TCGA-OL-A5RY-01A	BRCA	6.86
TCGA-AC-A3YJ-01A	BRCA	7.14
TCGA-E2-A1LS-01A	BRCA	5.73
TCGA-A2-A0EW-01A	BRCA	7.87
TCGA-C8-A1HM-01A	BRCA	8.88
TCGA-AR-A1AK-01A	BRCA	7.52
TCGA-E9-A1N6-01A	BRCA	7.05
TCGA-B6-A0RS-01A	BRCA	7.2
TCGA-BH-A0DV-01A	BRCA	7.82
TCGA-BH-A1EU-01A	BRCA	7.73
TCGA-BH-A18I-01A	BRCA	7.58
TCGA-E9-A24A-01A	BRCA	8.2
TCGA-E2-A1B0-01A	BRCA	7.44
TCGA-A2-A4S2-01A	BRCA	7.45
TCGA-A1-A0SJ-01A	BRCA	7.83
TCGA-JL-A3YW-01A	BRCA	6.54
TCGA-EW-A1J2-01A	BRCA	8.16
TCGA-OL-A66L-01A	BRCA	6.71
TCGA-A2-A0T4-01A	BRCA	7.92
TCGA-C8-A12P-01A	BRCA	6.53
TCGA-AN-A03Y-01A	BRCA	7.54
TCGA-C8-A1HO-01A	BRCA	8.04

TCGA-A1-A0SP-01A	BRCA	6.85
TCGA-B6-A0IC-01A	BRCA	8.39
TCGA-E9-A2JT-01A	BRCA	7.02
TCGA-A2-A0YL-01A	BRCA	7.61
TCGA-E9-A1NH-01A	BRCA	7.62
TCGA-A8-A07C-01A	BRCA	7.12
TCGA-C8-A138-01A	BRCA	7.09
TCGA-OL-A5D6-01A	BRCA	7.18
TCGA-E9-A1R4-01A	BRCA	7
TCGA-B6-A0RI-01A	BRCA	7.32
TCGA-A2-A0SU-01A	BRCA	8.79
TCGA-AO-A1KO-01A	BRCA	7.12
TCGA-D8-A27P-01A	BRCA	8.34
TCGA-AO-A0JJ-01A	BRCA	7.97
TCGA-OL-A5D7-01A	BRCA	6.41
TCGA-BH-A1FR-01A	BRCA	8.54
TCGA-A2-A3XT-01A	BRCA	6.13
TCGA-EW-A1IZ-01A	BRCA	7.59
TCGA-AO-A126-01A	BRCA	8.05
TCGA-A8-A08R-01A	BRCA	7.02
TCGA-BH-A0E6-01A	BRCA	6.88
TCGA-A7-A26J-01B	BRCA	7.33
TCGA-A8-A06O-01A	BRCA	7.46
TCGA-A7-A13G-01B	BRCA	6.83
TCGA-B6-A0I8-01A	BRCA	7.31
TCGA-AC-A2BK-01A	BRCA	7.71
TCGA-C8-A12K-01A	BRCA	7.12
TCGA-BH-A1FN-01A	BRCA	8.34
TCGA-A2-A04X-01A	BRCA	7.96
TCGA-A8-A08G-01A	BRCA	7.45
TCGA-A8-A06X-01A	BRCA	7.64
TCGA-AC-A3QP-01A	BRCA	7.33
TCGA-E2-A15A-06A	BRCA	7.39
TCGA-D8-A1JP-01A	BRCA	7.45
TCGA-C8-A12T-01A	BRCA	8.01
TCGA-AN-A0AS-01A	BRCA	7.08
TCGA-AC-A6IX-06A	BRCA	7.92
TCGA-A2-A0YJ-01A	BRCA	7.51
TCGA-D8-A27K-01A	BRCA	8.08
TCGA-B6-A40B-01A	BRCA	7.26
TCGA-A8-A08C-01A	BRCA	8.04
TCGA-E9-A1R2-01A	BRCA	6.32
TCGA-BH-A1EN-01A	BRCA	8.07
TCGA-C8-A1HN-01A	BRCA	7.65
TCGA-BH-A0C3-01A	BRCA	7.14
TCGA-BH-A0BV-01A	BRCA	7.23
TCGA-D8-A1X8-01A	BRCA	8.17
TCGA-E9-A227-01A	BRCA	7.6
TCGA-5L-AAT0-01A	BRCA	7.35
TCGA-AC-A8OR-01A	BRCA	8.35
TCGA-A2-A0YK-01A	BRCA	7.56
TCGA-C8-A130-01A	BRCA	7.43
TCGA-BH-A0E2-01A	BRCA	8.1
TCGA-EW-A1OZ-01A	BRCA	8.07
TCGA-A7-A13D-01A	BRCA	5.94
TCGA-C8-A26V-01A	BRCA	7.17
TCGA-BH-A18H-01A	BRCA	7.89
TCGA-OL-A66J-01A	BRCA	7.39

TCGA-AR-A1AP-01A	BRCA	7.81
TCGA-A2-A0ET-01A	BRCA	8.29
TCGA-AO-A0J7-01A	BRCA	8.09
TCGA-LD-A9QF-01A	BRCA	6.82
TCGA-AR-A1AQ-01A	BRCA	6.6
TCGA-B6-A0RG-01A	BRCA	7.53
TCGA-A2-A0EX-01A	BRCA	8.31
TCGA-PL-A8LY-01A	BRCA	7.31
TCGA-A2-A0ES-01A	BRCA	8.31
TCGA-C8-A1HF-01A	BRCA	7.11
TCGA-E9-A5UP-01A	BRCA	7.67
TCGA-AN-A0XV-01A	BRCA	8.34
TCGA-BH-A0HN-01A	BRCA	7.53
TCGA-A2-A0YF-01A	BRCA	8.41
TCGA-BH-A0H6-01A	BRCA	8.6
TCGA-A2-A3XU-01A	BRCA	6.8
TCGA-A2-A0CV-01A	BRCA	7.63
TCGA-A8-A0AD-01A	BRCA	7.78
TCGA-AO-A0J4-01A	BRCA	7.02
TCGA-D8-A27M-01A	BRCA	7.01
TCGA-A2-A04T-01A	BRCA	6.64
TCGA-E9-A1NA-01A	BRCA	7.14
TCGA-EW-A2FR-01A	BRCA	7.44
TCGA-LL-A5YO-01A	BRCA	6.61
TCGA-E9-A22B-01A	BRCA	6.95
TCGA-E2-A1IL-01A	BRCA	8.07
TCGA-A8-A07B-01A	BRCA	7.26
TCGA-A7-A26I-01A	BRCA	6.31
TCGA-BH-A0B1-01A	BRCA	7.84
TCGA-EW-A423-01A	BRCA	7.09
TCGA-A1-A0SD-01A	BRCA	7.77
TCGA-D8-A1XD-01A	BRCA	8.33
TCGA-E2-A1IK-01A	BRCA	8.16
TCGA-AN-A0FN-01A	BRCA	7.39
TCGA-E2-A15D-01A	BRCA	7.68
TCGA-BH-A0AW-01A	BRCA	7.28
TCGA-AR-A0U3-01A	BRCA	8.22
TCGA-D8-A1X7-01A	BRCA	8.18
TCGA-E2-A14X-01A	BRCA	7.32
TCGA-LL-A9Q3-01A	BRCA	6.12
TCGA-GM-A2DB-01A	BRCA	6.47
TCGA-AR-A0U4-01A	BRCA	6.8
TCGA-D8-A1XA-01A	BRCA	7.96
TCGA-E2-A1B5-01A	BRCA	7.26
TCGA-A8-A06U-01A	BRCA	7.02
TCGA-A8-A07R-01A	BRCA	5.93
TCGA-A2-A0ER-01A	BRCA	8.49
TCGA-E2-A1LE-01A	BRCA	6.92
TCGA-A1-A0SM-01A	BRCA	8.04
TCGA-A2-A1G1-01A	BRCA	7.59
TCGA-AQ-A04H-01B	BRCA	7.44
TCGA-E9-A6HE-01A	BRCA	6.7
TCGA-AN-A0FZ-01A	BRCA	6.82
TCGA-PE-A5DE-01A	BRCA	6.84
TCGA-EW-A1J6-01A	BRCA	6.54
TCGA-A7-A13D-01B	BRCA	5.86
TCGA-AC-A6NO-01A	BRCA	7.58
TCGA-A7-A2KD-01A	BRCA	7.15

TCGA-A8-A08L-01A	BRCA	6.93
TCGA-A7-A6VW-01A	BRCA	6.21
TCGA-EW-A6SC-01A	BRCA	7.02
TCGA-D8-A1XK-01A	BRCA	7.76
TCGA-BH-A0AY-01A	BRCA	7.09
TCGA-C8-A12U-01A	BRCA	6.43
TCGA-AR-A0U2-01A	BRCA	7.85
TCGA-D8-A1JA-01A	BRCA	7.93
TCGA-A7-A0CH-01A	BRCA	8.26
TCGA-A8-A0A7-01A	BRCA	6.48
TCGA-AR-A250-01A	BRCA	7.88
TCGA-B6-A408-01A	BRCA	7.24
TCGA-E2-A15P-01A	BRCA	7.35
TCGA-AR-A5QM-01A	BRCA	7.33
TCGA-A8-A07Z-01A	BRCA	7.89
TCGA-BH-A0W7-01A	BRCA	7.05
TCGA-A7-A4SA-01A	BRCA	6.11
TCGA-AO-A0J9-01A	BRCA	6.81
TCGA-E9-A244-01A	BRCA	7.72
TCGA-A2-A4S0-01A	BRCA	8.03
TCGA-EW-A424-01A	BRCA	8.02
TCGA-OL-A66O-01A	BRCA	7.11
TCGA-OL-A5RW-01A	BRCA	6.3
TCGA-A2-A0SV-01A	BRCA	6.86
TCGA-E9-A1NF-01A	BRCA	7.38
TCGA-E2-A105-01A	BRCA	7.62
TCGA-D8-A1JU-01A	BRCA	8.12
TCGA-A7-A425-01A	BRCA	6.88
TCGA-GM-A2DD-01A	BRCA	6.69
TCGA-BH-A0BD-01A	BRCA	7.27
TCGA-BH-A0BQ-01A	BRCA	7.45
TCGA-E9-A228-01A	BRCA	8.4
TCGA-D8-A27W-01A	BRCA	8.32
TCGA-E2-A1IG-01A	BRCA	7.44
TCGA-D8-A1JI-01A	BRCA	7.36
TCGA-AN-A0FT-01A	BRCA	7.24
TCGA-A2-A3KD-01A	BRCA	7.84
TCGA-C8-A3M8-01A	BRCA	7.34
TCGA-A2-A0EO-01A	BRCA	8.01
TCGA-BH-A0B4-01A	BRCA	6.82
TCGA-E2-A14S-01A	BRCA	7.7
TCGA-E9-A1NG-01A	BRCA	7.85
TCGA-AC-A3TM-01A	BRCA	6.88
TCGA-AN-A04C-01A	BRCA	7.19
TCGA-BH-A0E1-01A	BRCA	7.45
TCGA-C8-A12W-01A	BRCA	7.71
TCGA-A8-A09K-01A	BRCA	8.16
TCGA-E9-A1N4-01A	BRCA	8.16
TCGA-BH-A18V-01A	BRCA	7.82
TCGA-E2-A9RU-01A	BRCA	6.82
TCGA-A2-A0D4-01A	BRCA	6.69
TCGA-D8-A1XR-01A	BRCA	7.93
TCGA-E2-A14N-01A	BRCA	6.69
TCGA-PE-A5DC-01A	BRCA	7.39
TCGA-AR-A2LL-01A	BRCA	8.31
TCGA-BH-A0E9-01B	BRCA	7.39
TCGA-A2-A0CY-01A	BRCA	7.33
TCGA-BH-A0AV-01A	BRCA	6.83

TCGA-BH-A0GY-01A	BRCA	7.27
TCGA-BH-A0HY-01A	BRCA	7.42
TCGA-A8-A08I-01A	BRCA	8.72
TCGA-A2-A25C-01A	BRCA	8.01
TCGA-OL-A5RZ-01A	BRCA	7.74
TCGA-A8-A0A9-01A	BRCA	7.53
TCGA-A7-A6VX-01A	BRCA	8.04
TCGA-E2-A106-01A	BRCA	7.62
TCGA-AC-A6IV-01A	BRCA	7.13
TCGA-AN-A03X-01A	BRCA	8.08
TCGA-EW-A1PB-01A	BRCA	6.8
TCGA-BH-A6R8-01A	BRCA	7.2
TCGA-PL-A8LZ-01A	BRCA	6.38
TCGA-D8-A1XM-01A	BRCA	7.4
TCGA-BH-A1ET-01A	BRCA	8.5
TCGA-B6-A3ZX-01A	BRCA	6.34
TCGA-W8-A86G-01A	BRCA	7.84
TCGA-AO-A03N-01B	BRCA	6.1
TCGA-D8-A73U-01A	BRCA	6.82
TCGA-E9-A1RB-01A	BRCA	7.49
TCGA-AR-A0TS-01A	BRCA	6.71
TCGA-AO-A12A-01A	BRCA	7.84
TCGA-AN-A0XS-01A	BRCA	7.65
TCGA-AN-A0G0-01A	BRCA	6.57
TCGA-E2-A10C-01A	BRCA	6.79
TCGA-BH-A0H0-01A	BRCA	8.97
TCGA-AQ-A04J-01A	BRCA	5.48
TCGA-AN-A0AK-01A	BRCA	8.15
TCGA-AR-A2LN-01A	BRCA	7.62
TCGA-A8-A081-01A	BRCA	8.5
TCGA-A2-A04Y-01A	BRCA	6.65
TCGA-BH-A0B5-01A	BRCA	7.67
TCGA-A2-A0CZ-01A	BRCA	6.72
TCGA-B6-A0WX-01A	BRCA	6.72
TCGA-D8-A3Z6-01A	BRCA	6.32
TCGA-A8-A07S-01A	BRCA	8.06
TCGA-E2-A14Y-01A	BRCA	8.25
TCGA-BH-A18L-01A	BRCA	8.3
TCGA-BH-A0BT-01A	BRCA	8.49
TCGA-LD-A7W5-01A	BRCA	6.87
TCGA-A2-A3XS-01A	BRCA	5.98
TCGA-E2-A152-01A	BRCA	7.37
TCGA-BH-A5IZ-01A	BRCA	7.46
TCGA-LL-A740-01A	BRCA	7.38
TCGA-C8-A12Q-01A	BRCA	7.57
TCGA-A2-A0CQ-01A	BRCA	7.59
TCGA-AO-A12H-01A	BRCA	8.22
TCGA-C8-A133-01A	BRCA	7.89
TCGA-A2-A04Q-01A	BRCA	6.32
TCGA-AO-A124-01A	BRCA	5.29
TCGA-AN-A0FL-01A	BRCA	6.21
TCGA-BH-A1FC-01A	BRCA	6.94
TCGA-AN-A0FD-01A	BRCA	7.96
TCGA-C8-A131-01A	BRCA	6.94
TCGA-A2-A25F-01A	BRCA	7.38
TCGA-E9-A1NI-01A	BRCA	7.74
TCGA-GM-A2DA-01A	BRCA	6.72
TCGA-B6-A0RQ-01A	BRCA	8.21

TCGA-BH-A0H5-01A	BRCA	7.73
TCGA-AC-A2B8-01A	BRCA	7.85
TCGA-A7-A13G-01A	BRCA	6.98
TCGA-A7-A3IY-01A	BRCA	7.85
TCGA-LL-A5YP-01A	BRCA	6.59
TCGA-E2-A1IF-01A	BRCA	7.31
TCGA-BH-A18G-01A	BRCA	6.5
TCGA-B6-A402-01A	BRCA	6.33
TCGA-BH-A0DT-01A	BRCA	8.2
TCGA-EW-A1PF-01A	BRCA	7.48
TCGA-A8-A09Z-01A	BRCA	8.66
TCGA-BH-A0C0-01A	BRCA	6.88
TCGA-BH-A1FM-01A	BRCA	7.12
TCGA-A8-A07E-01A	BRCA	7.94
TCGA-A8-A09C-01A	BRCA	8.1
TCGA-AC-A2BM-01A	BRCA	7.88
TCGA-AO-A0JA-01A	BRCA	7.19
TCGA-A2-A0CO-01A	BRCA	7.04
TCGA-A8-A07G-01A	BRCA	7.48
TCGA-LL-A50Y-01A	BRCA	7.75
TCGA-A7-A4SD-01A	BRCA	5.11
TCGA-A2-A0T0-01A	BRCA	6.49
TCGA-S3-AA14-01A	BRCA	8.07
TCGA-AR-A251-01A	BRCA	7.31
TCGA-BH-A0BL-01A	BRCA	6.79
TCGA-AC-A3QQ-01B	BRCA	5.31
TCGA-AC-A5XU-01A	BRCA	7.66
TCGA-A2-A0D2-01A	BRCA	6.82
TCGA-A2-A0SW-01A	BRCA	6.24
TCGA-E9-A3X8-01A	BRCA	6.76
TCGA-B6-A2IU-01A	BRCA	7.92
TCGA-EW-A6S9-01A	BRCA	7.28
TCGA-E9-A1N9-01A	BRCA	7.91
TCGA-D8-A27I-01A	BRCA	7.22
TCGA-E2-A158-01A	BRCA	8.01
TCGA-A1-A0SK-01A	BRCA	7.93
TCGA-AC-A23G-01A	BRCA	7.06
TCGA-C8-A1HJ-01A	BRCA	6.23
TCGA-GM-A2DL-01A	BRCA	7.6
TCGA-AR-A2LO-01A	BRCA	7.01
TCGA-E2-A1IJ-01A	BRCA	7.37
TCGA-AR-A0TP-01A	BRCA	6.9
TCGA-D8-A27V-01A	BRCA	8.77
TCGA-E9-A5FK-01A	BRCA	6.85
TCGA-BH-A203-01A	BRCA	7.77
TCGA-BH-A0DL-01A	BRCA	6.29
TCGA-OL-A5RU-01A	BRCA	7.66
TCGA-A8-A083-01A	BRCA	8.54
TCGA-A2-A25E-01A	BRCA	8.52
TCGA-C8-A1HE-01A	BRCA	8.34
TCGA-A7-A26I-01B	BRCA	5.94
TCGA-AO-A1KS-01A	BRCA	8.49
TCGA-E9-A1QZ-01A	BRCA	7.77
TCGA-AR-A0TX-01A	BRCA	7.77
TCGA-BH-A18F-01A	BRCA	7.99
TCGA-AO-A0J5-01A	BRCA	7.93
TCGA-AO-A1KR-01A	BRCA	5.99
TCGA-EW-A1J3-01A	BRCA	7.86

TCGA-A2-A0EU-01A	BRCA	9.07
TCGA-BH-A0EE-01A	BRCA	6.55
TCGA-BH-A18J-01A	BRCA	7.4
TCGA-D8-A1XY-01A	BRCA	8.43
TCGA-EW-A1P7-01A	BRCA	6.97
TCGA-AO-A128-01A	BRCA	7.67
TCGA-A8-A093-01A	BRCA	8.04
TCGA-B6-A0IQ-01A	BRCA	5.63
TCGA-E2-A155-01A	BRCA	7.89
TCGA-BH-A0DX-01A	BRCA	8.27
TCGA-B6-A0IO-01A	BRCA	6.72
TCGA-C8-A137-01A	BRCA	7.75
TCGA-AC-A2FB-01A	BRCA	7.52
TCGA-A8-A09Q-01A	BRCA	8.26
TCGA-A2-A0D1-01A	BRCA	6.98
TCGA-BH-A0DG-01A	BRCA	7.34
TCGA-GM-A3XN-01A	BRCA	6.7
TCGA-BH-A0HB-01A	BRCA	7.92
TCGA-E2-A572-01A	BRCA	6.97
TCGA-A7-A0CD-01A	BRCA	8.77
TCGA-C8-A1HG-01A	BRCA	7.52
TCGA-A1-A0SG-01A	BRCA	8.06
TCGA-AN-A0FS-01A	BRCA	8.62
TCGA-S3-AA11-01A	BRCA	6.5
TCGA-E2-A1BD-01A	BRCA	8.28
TCGA-E2-A15E-06A	BRCA	7.76
TCGA-B6-A0RO-01A	BRCA	8.95
TCGA-E2-A1II-01A	BRCA	7.45
TCGA-D8-A1XS-01A	BRCA	8.75
TCGA-AN-A0FX-01A	BRCA	7.66
TCGA-D8-A1JC-01A	BRCA	8.81
TCGA-AO-A0JC-01A	BRCA	7.07
TCGA-E2-A574-01A	BRCA	6.41
TCGA-E9-A1N3-01A	BRCA	8.09
TCGA-AN-A0AT-01A	BRCA	7.72
TCGA-BH-A204-01A	BRCA	8.81
TCGA-A2-A0T1-01A	BRCA	6.78
TCGA-VS-A9UL-01A	CESC	5.71
TCGA-VS-A8EI-01A	CESC	7.04
TCGA-JX-A5QV-01A	CESC	7.43
TCGA-EK-A2RA-01A	CESC	6.79
TCGA-VS-A9U5-01A	CESC	7.15
TCGA-C5-A1MI-01A	CESC	7.14
TCGA-ZJ-AAXD-01A	CESC	6.9
TCGA-C5-A8XI-01A	CESC	6.73
TCGA-VS-A8EG-01A	CESC	6.91
TCGA-FU-A57G-01A	CESC	6.77
TCGA-Q1-A73S-01A	CESC	8.62
TCGA-C5-A1M6-01A	CESC	6.63
TCGA-VS-A9UY-01A	CESC	7.21
TCGA-VS-A9V1-01A	CESC	6.35
TCGA-JW-A5VL-01A	CESC	6.73
TCGA-VS-A9UC-01A	CESC	5.86
TCGA-EK-A2RC-01A	CESC	6.91
TCGA-C5-A7UI-01A	CESC	5.94
TCGA-VS-A9V5-01A	CESC	6.73
TCGA-VS-A9UV-01A	CESC	7.27
TCGA-ZJ-AAXU-01A	CESC	6.2

TCGA-EK-A2R8-01A	CESC	6.66
TCGA-C5-A2LZ-01A	CESC	7.18
TCGA-2W-A8YY-01A	CESC	6.82
TCGA-C5-A7UC-01A	CESC	7.12
TCGA-Q1-A73Q-01A	CESC	7.88
TCGA-Q1-A6DW-01A	CESC	6.78
TCGA-EA-A5ZE-01A	CESC	7.09
TCGA-IR-A3L7-01A	CESC	7.59
TCGA-GH-A9DA-01A	CESC	6.8
TCGA-JW-A5VH-01A	CESC	6.55
TCGA-C5-A3HF-01A	CESC	6.87
TCGA-EA-A3HQ-01A	CESC	6.99
TCGA-Q1-A5R3-01A	CESC	7.14
TCGA-EA-A50E-01A	CESC	7.99
TCGA-EK-A2RL-01A	CESC	6.26
TCGA-JW-A5VI-01A	CESC	7.31
TCGA-DG-A2KH-01A	CESC	7.04
TCGA-IR-A3LL-01A	CESC	6.04
TCGA-EA-A411-01A	CESC	7.16
TCGA-C5-A1BI-01B	CESC	6.94
TCGA-EK-A2IP-01A	CESC	6.08
TCGA-MA-AA3X-01A	CESC	6.13
TCGA-VS-A9UR-01A	CESC	5.82
TCGA-C5-A7X5-01A	CESC	7.18
TCGA-VS-A8EC-01A	CESC	7.49
TCGA-VS-A954-01A	CESC	7.75
TCGA-VS-A9UI-01A	CESC	6.8
TCGA-FU-A5XV-01A	CESC	7.42
TCGA-MA-AA41-01A	CESC	7.27
TCGA-C5-A1MK-01A	CESC	6.32
TCGA-EK-A2IR-01A	CESC	6.25
TCGA-IR-A3LK-01A	CESC	7.73
TCGA-C5-A907-01A	CESC	6.5
TCGA-C5-A3HE-01A	CESC	6.02
TCGA-VS-A9UT-01A	CESC	5.98
TCGA-EK-A2RO-01A	CESC	6.97
TCGA-C5-A8YR-01A	CESC	5.75
TCGA-EK-A2GZ-01A	CESC	6.71
TCGA-UC-A7PD-01A	CESC	6.52
TCGA-JX-A3Q0-01A	CESC	7.2
TCGA-C5-A0TN-01A	CESC	6.28
TCGA-C5-A7CK-01A	CESC	6.81
TCGA-EA-A5O9-01A	CESC	6.38
TCGA-VS-A8EB-01A	CESC	6.79
TCGA-VS-A94X-01A	CESC	6.76
TCGA-C5-A8ZZ-01A	CESC	7.53
TCGA-VS-A8QM-01A	CESC	6.77
TCGA-JX-A3Q8-01A	CESC	5.48
TCGA-C5-A8XH-01A	CESC	7.62
TCGA-DS-A1OB-01A	CESC	6.68
TCGA-VS-A950-01A	CESC	6.23
TCGA-C5-A1MH-01A	CESC	6.61
TCGA-WL-A834-01A	CESC	8.19
TCGA-C5-A7X3-01A	CESC	6.3
TCGA-C5-A902-01A	CESC	6.73
TCGA-IR-A3LC-01A	CESC	7.07
TCGA-DS-A1OA-01A	CESC	7.24
TCGA-FU-A2QG-01A	CESC	7.17

TCGA-DS-A0VM-01A	CESC	7.9
TCGA-EA-A44S-01A	CESC	6.59
TCGA-VS-A9UM-01A	CESC	6.51
TCGA-IR-A3LH-01A	CESC	6.25
TCGA-EK-A2RB-01A	CESC	6.27
TCGA-VS-A8EK-01A	CESC	6.79
TCGA-EX-A3L1-01A	CESC	6.52
TCGA-C5-A7CO-01A	CESC	6.7
TCGA-C5-A7CJ-01A	CESC	6.84
TCGA-DS-A7WH-01A	CESC	6.93
TCGA-ZJ-A8QR-01A	CESC	6.98
TCGA-EK-A2R9-01A	CESC	6.2
TCGA-VS-AA62-01A	CESC	6.64
TCGA-VS-A8EJ-01A	CESC	5.48
TCGA-EA-A3HT-01A	CESC	6.95
TCGA-BI-A0VS-01A	CESC	6.39
TCGA-BI-A0VR-01A	CESC	6.89
TCGA-VS-A8Q9-01A	CESC	6.26
TCGA-EK-A2PI-01A	CESC	6.55
TCGA-EK-A2RM-01A	CESC	6.2
TCGA-DS-A7WI-01A	CESC	5.81
TCGA-IR-A3LB-01A	CESC	6.59
TCGA-DG-A2KM-01A	CESC	6.81
TCGA-C5-A7CH-01A	CESC	6.69
TCGA-EK-A2R7-01A	CESC	6.44
TCGA-DS-A1OD-01A	CESC	6.68
TCGA-C5-A8XJ-01A	CESC	4.1
TCGA-ZJ-AAXA-01A	CESC	7.73
TCGA-EK-A3GJ-01A	CESC	7.08
TCGA-EA-A5FO-01A	CESC	7.32
TCGA-C5-A7XC-01A	CESC	6.69
TCGA-C5-A1M7-01A	CESC	7.01
TCGA-VS-A8EL-01A	CESC	5.97
TCGA-VS-A9UP-01A	CESC	6.1
TCGA-LP-A4AU-01A	CESC	6.4
TCGA-DS-A1OC-01A	CESC	6.7
TCGA-Q1-A73R-01A	CESC	6.28
TCGA-BI-A20A-01A	CESC	6.92
TCGA-C5-A2LY-01A	CESC	6.38
TCGA-UC-A7PF-01A	CESC	6.71
TCGA-VS-A8QF-01A	CESC	6.7
TCGA-C5-A1MJ-01A	CESC	6.36
TCGA-EA-A5ZD-01A	CESC	7.22
TCGA-EX-A69M-01A	CESC	6.13
TCGA-LP-A5U2-01A	CESC	7.13
TCGA-DR-A0ZL-01A	CESC	7.72
TCGA-VS-A94W-01A	CESC	6.54
TCGA-EA-A439-01A	CESC	6.31
TCGA-C5-A7X8-01A	CESC	5.8
TCGA-EK-A2PL-01A	CESC	7.15
TCGA-MU-A5YI-01A	CESC	6.8
TCGA-EX-A449-01A	CESC	5.82
TCGA-VS-A959-01A	CESC	6.61
TCGA-ZJ-AB0H-01A	CESC	7.22
TCGA-HG-A2PA-01A	CESC	6.93
TCGA-Q1-A5R2-01A	CESC	6.14
TCGA-C5-A1BQ-01C	CESC	5.69
TCGA-C5-A7UE-01A	CESC	6.06

TCGA-MA-AA42-01A	CESC	6.18
TCGA-EK-A3GN-01A	CESC	6.58
TCGA-MA-AA3W-01A	CESC	7.71
TCGA-VS-A953-01A	CESC	6.99
TCGA-MY-A5BE-01A	CESC	6.39
TCGA-DS-A0VN-01A	CESC	6.91
TCGA-XS-A8TJ-01A	CESC	7.11
TCGA-EK-A2PM-01A	CESC	7.28
TCGA-LP-A7HU-01A	CESC	6.62
TCGA-MU-A51Y-01A	CESC	7.01
TCGA-DR-A0ZM-01A	CESC	6.57
TCGA-IR-A3LA-01A	CESC	6.74
TCGA-VS-A9UJ-01A	CESC	6.63
TCGA-FU-A40J-01A	CESC	6.2
TCGA-C5-A1BJ-01A	CESC	7.1
TCGA-HM-A6W2-06A	CESC	6.15
TCGA-C5-A1BK-01B	CESC	7.19
TCGA-ZJ-A8QQ-01A	CESC	6.97
TCGA-EA-A4BA-01A	CESC	6.07
TCGA-VS-A958-01A	CESC	6.35
TCGA-C5-A7UH-01A	CESC	7.38
TCGA-C5-A7CL-01A	CESC	6.01
TCGA-VS-A8QH-01A	CESC	6.76
TCGA-VS-A957-01A	CESC	7.09
TCGA-C5-A1ME-01A	CESC	7.6
TCGA-JW-A5VK-01A	CESC	1.8
TCGA-DS-A7WF-01A	CESC	6.71
TCGA-Q1-A73O-01A	CESC	6.64
TCGA-RA-A741-01A	CESC	6.46
TCGA-C5-A7CM-01A	CESC	5.26
TCGA-DS-A1O9-01A	CESC	5.83
TCGA-C5-A3HL-01A	CESC	5.84
TCGA-C5-A3HD-01B	CESC	8.32
TCGA-FU-A770-01A	CESC	6.64
TCGA-JW-A5VG-01A	CESC	7.23
TCGA-EA-A5ZF-01A	CESC	6.08
TCGA-LP-A5U3-01A	CESC	7.55
TCGA-ZJ-AAX4-01A	CESC	6.07
TCGA-EX-A1H6-01B	CESC	6.69
TCGA-VS-A94Y-01A	CESC	6.7
TCGA-C5-A2LS-01A	CESC	6.98
TCGA-R2-A69V-01A	CESC	6.55
TCGA-VS-A8QA-01A	CESC	6.17
TCGA-LP-A4AV-01A	CESC	5.79
TCGA-VS-A9UB-01A	CESC	6.57
TCGA-C5-A1M5-01A	CESC	7.01
TCGA-VS-A9V3-01A	CESC	7.08
TCGA-EA-A43B-01A	CESC	6.41
TCGA-EA-A556-01A	CESC	6.52
TCGA-MA-AA3Y-01A	CESC	6.95
TCGA-ZJ-AAXT-01A	CESC	6.49
TCGA-C5-A1BF-01B	CESC	6.7
TCGA-C5-A1MN-01A	CESC	7.5
TCGA-JX-A3PZ-01A	CESC	6.6
TCGA-C5-A1ML-01A	CESC	6.05
TCGA-C5-A2LV-01A	CESC	6.72
TCGA-LP-A4AX-01A	CESC	6.44
TCGA-Q1-A73P-01A	CESC	7.32

TCGA-C5-A1MF-01A	CESC	6.18
TCGA-C5-A1BL-01A	CESC	7.22
TCGA-ZJ-AAXB-01A	CESC	6.71
TCGA-C5-A1BM-01A	CESC	5.62
TCGA-C5-A2M1-01A	CESC	7.13
TCGA-C5-A1BN-01B	CESC	7.56
TCGA-EA-A6QX-01A	CESC	6.61
TCGA-DG-A2KK-01A	CESC	7.01
TCGA-VS-A9U6-01A	CESC	6.49
TCGA-C5-A1M9-01A	CESC	7.34
TCGA-MA-AA3Z-01A	CESC	7.41
TCGA-UC-A7PI-01A	CESC	6.49
TCGA-VS-A8QC-01A	CESC	6.43
TCGA-ZJ-AB0I-01A	CESC	7.25
TCGA-Q1-A6DV-01A	CESC	7.2
TCGA-C5-A2LX-01A	CESC	5.93
TCGA-EK-A2RJ-01A	CESC	5.98
TCGA-EK-A2RK-01A	CESC	6.4
TCGA-EA-A1QT-01A	CESC	7.18
TCGA-ZJ-AAX8-01A	CESC	6.29
TCGA-EA-A3QE-01A	CESC	6.83
TCGA-FU-A3TX-01A	CESC	5.66
TCGA-VS-A9V0-01A	CESC	5.66
TCGA-EK-A3GM-01A	CESC	5.57
TCGA-FU-A3EO-01A	CESC	7.25
TCGA-C5-A905-01A	CESC	7.73
TCGA-C5-A8YT-01A	CESC	6.53
TCGA-C5-A8YQ-01A	CESC	7.22
TCGA-VS-A8EH-01A	CESC	6.03
TCGA-VS-A9UU-01A	CESC	6.27
TCGA-FU-A3HY-01A	CESC	8.04
TCGA-DS-A0VK-01A	CESC	6.9
TCGA-UC-A7PG-01A	CESC	6.33
TCGA-VS-A9V4-01A	CESC	6.89
TCGA-VS-A9UH-01A	CESC	5.78
TCGA-VS-A8Q8-01A	CESC	6.96
TCGA-VS-A94Z-01A	CESC	7.23
TCGA-VS-A9UD-01A	CESC	5.85
TCGA-EX-A69L-01A	CESC	6.48
TCGA-HM-A3JK-01A	CESC	6.19
TCGA-IR-A3LI-01A	CESC	6.2
TCGA-DS-A3LQ-01A	CESC	7.13
TCGA-DG-A2KJ-01A	CESC	5.11
TCGA-EA-A97N-01A	CESC	7.62
TCGA-ZX-AA5X-01A	CESC	6.35
TCGA-ZJ-AAXJ-01A	CESC	6.9
TCGA-EK-A2PK-01A	CESC	6.04
TCGA-EX-A1H5-01A	CESC	6.31
TCGA-FU-A3HZ-01A	CESC	6.5
TCGA-FU-A3WB-01A	CESC	7.08
TCGA-UC-A7PG-06A	CESC	6.46
TCGA-ZJ-AAXN-01A	CESC	7.32
TCGA-VS-A9U7-01A	CESC	6.91
TCGA-C5-A2M2-01A	CESC	7.57
TCGA-4J-AA1J-01A	CESC	7.67
TCGA-IR-A3LF-01A	CESC	7.57
TCGA-FU-A3YQ-01A	CESC	6.71
TCGA-EA-A1QS-01A	CESC	6.53

TCGA-FU-A23L-01A	CESC	6.92
TCGA-EA-A3HU-01A	CESC	6.47
TCGA-HM-A6W2-01A	CESC	5.69
TCGA-C5-A1MP-01A	CESC	6.63
TCGA-ZJ-AAXF-01A	CESC	6.04
TCGA-HM-A4S6-01A	CESC	6.42
TCGA-VS-A9UZ-01A	CESC	6.92
TCGA-EK-A2H1-01A	CESC	6.69
TCGA-EA-A3HR-01A	CESC	7.47
TCGA-FU-A23K-01A	CESC	6.03
TCGA-EA-A78R-01A	CESC	5.98
TCGA-JW-A852-01A	CESC	5.83
TCGA-JW-AAVH-01A	CESC	7.35
TCGA-C5-A1M8-01A	CESC	6.69
TCGA-VS-A9UQ-01A	CESC	7.74
TCGA-MU-A8JM-01A	CESC	7.14
TCGA-EX-A8YF-01A	CESC	6.33
TCGA-DG-A2KL-01A	CESC	6.75
TCGA-C5-A1BE-01B	CESC	7.21
TCGA-VS-A9UO-01A	CESC	6.21
TCGA-PN-A8MA-01A	CESC	6.79
TCGA-ZJ-AAXI-01A	CESC	6.79
TCGA-VS-A9V2-01A	CESC	6.73
TCGA-MY-A5BF-01A	CESC	7.93
TCGA-Q1-A6DT-01A	CESC	6.29
TCGA-EA-A3Y4-01A	CESC	6.84
TCGA-EK-A2PG-01A	CESC	6.74
TCGA-FU-A3TQ-01A	CESC	5.89
TCGA-EA-A410-01A	CESC	6.17
TCGA-LP-A4AW-01A	CESC	7.17
TCGA-EA-A3HS-01A	CESC	7.66
TCGA-EK-A2RE-01A	CESC	7.35
TCGA-C5-A8XK-01A	CESC	7.12
TCGA-EA-A3QD-01A	CESC	6.6
TCGA-DS-A5RQ-01A	CESC	7.01
TCGA-VS-A952-01A	CESC	6.53
TCGA-EK-A2RN-01A	CESC	4.89
TCGA-EK-A3GK-01A	CESC	6.87
TCGA-EK-A2H0-01A	CESC	7.12
TCGA-MY-A5BD-01A	CESC	7.84
TCGA-C5-A901-01A	CESC	6.88
TCGA-DS-A0VL-01A	CESC	7.47
TCGA-MA-AA43-01A	CESC	6.73
TCGA-ZJ-A8QO-01A	CESC	6.52
TCGA-JW-A5VJ-01A	CESC	6.33
TCGA-C5-A1MQ-01A	CESC	6.21
TCGA-C5-A7CG-01A	CESC	7.2
TCGA-HM-A3JJ-01A	CESC	7.07
TCGA-MY-A913-01A	CESC	6.76
TCGA-Q1-A5R1-01A	CESC	7.18
TCGA-JW-A69B-01A	CESC	5.5
TCGA-C5-A2LT-01A	CESC	6.86
TCGA-FU-A3NI-01A	CESC	7.82
TCGA-W5-AA33-01A	CHOL	7.46
TCGA-YR-A95A-01A	CHOL	7.24
TCGA-ZH-A8Y6-01A	CHOL	7.95
TCGA-3X-AAVB-01A	CHOL	7.08
TCGA-WD-A7RX-01A	CHOL	7.88

TCGA-W5-AA2Z-01A	CHOL	7.7
TCGA-W5-AA38-01A	CHOL	7.18
TCGA-W5-AA2X-01A	CHOL	6.66
TCGA-W6-AA0S-01A	CHOL	7.31
TCGA-W5-AA2Q-01A	CHOL	7.01
TCGA-W5-AA36-01A	CHOL	7.1
TCGA-W5-AA39-01A	CHOL	7.43
TCGA-W5-AA2I-01A	CHOL	7.03
TCGA-3X-AAV9-01A	CHOL	6.51
TCGA-ZH-A8Y2-01A	CHOL	7.29
TCGA-W5-AA2G-01A	CHOL	7.65
TCGA-ZU-A8S4-01A	CHOL	7.64
TCGA-W5-AA2O-01A	CHOL	7.55
TCGA-W5-AA31-01A	CHOL	7.18
TCGA-3X-AAVC-01A	CHOL	7.52
TCGA-ZH-A8Y1-01A	CHOL	6.92
TCGA-3X-AAVE-01A	CHOL	6.53
TCGA-ZH-A8Y5-01A	CHOL	7.56
TCGA-ZH-A8Y4-01A	CHOL	6.31
TCGA-4G-AAZT-01A	CHOL	7.13
TCGA-W5-AA30-01A	CHOL	6.31
TCGA-W5-AA2T-01A	CHOL	7.17
TCGA-W5-AA34-01A	CHOL	6.88
TCGA-W5-AA2H-01A	CHOL	5.91
TCGA-ZD-A8I3-01A	CHOL	7
TCGA-W5-AA2W-01A	CHOL	6.5
TCGA-4G-AAZO-01A	CHOL	7.16
TCGA-ZH-A8Y8-01A	CHOL	7.21
TCGA-3X-AAVA-01A	CHOL	6.79
TCGA-W5-AA2U-01A	CHOL	7.31
TCGA-AA-A00K-01A	COAD	6.58
TCGA-D5-5538-01A	COAD	7.37
TCGA-AA-A00A-01A	COAD	6.99
TCGA-AA-A02K-01A	COAD	6.61
TCGA-D5-6922-01A	COAD	6.69
TCGA-AD-5900-01A	COAD	7.06
TCGA-AA-3495-01A	COAD	7.13
TCGA-AD-6888-01A	COAD	6.78
TCGA-CA-5256-01A	COAD	7.55
TCGA-G4-6299-01A	COAD	8.53
TCGA-AZ-4313-01A	COAD	6.51
TCGA-AA-3872-01A	COAD	6.66
TCGA-F4-6854-01A	COAD	7.47
TCGA-AA-3977-01A	COAD	7.25
TCGA-CA-5254-01A	COAD	7.46
TCGA-CK-5916-01A	COAD	7.48
TCGA-AA-A03F-01A	COAD	5.38
TCGA-AA-A01X-01A	COAD	6.02
TCGA-G4-6304-01A	COAD	7.11
TCGA-AA-3866-01A	COAD	7.27
TCGA-AY-4071-01A	COAD	6.71
TCGA-AA-3655-01A	COAD	7.26
TCGA-AA-A02E-01A	COAD	7.15
TCGA-AA-3510-01A	COAD	6.69
TCGA-AA-3561-01A	COAD	7.76
TCGA-D5-6930-01A	COAD	6.82
TCGA-AU-6004-01A	COAD	7.22
TCGA-F4-6461-01A	COAD	6.71

TCGA-AA-3675-01A	COAD	7.53
TCGA-D5-6926-01A	COAD	6.64
TCGA-A6-2684-01A	COAD	7.03
TCGA-CM-5868-01A	COAD	7.29
TCGA-AA-A00O-01A	COAD	6.84
TCGA-AA-3527-01A	COAD	5.49
TCGA-AA-3712-01A	COAD	7.43
TCGA-D5-5540-01A	COAD	7.79
TCGA-A6-6137-01A	COAD	7.05
TCGA-A6-5667-01A	COAD	6.94
TCGA-G4-6628-01A	COAD	7.74
TCGA-AY-6196-01A	COAD	6.4
TCGA-AA-3693-01A	COAD	7.91
TCGA-CK-5913-01A	COAD	7.02
TCGA-AA-3688-01A	COAD	7.32
TCGA-F4-6460-01A	COAD	7.09
TCGA-AA-3548-01A	COAD	6.19
TCGA-CM-6172-01A	COAD	7.34
TCGA-AA-3844-01A	COAD	7.38
TCGA-AZ-4308-01A	COAD	5.76
TCGA-A6-3808-01A	COAD	6.57
TCGA-AD-6963-01A	COAD	7.04
TCGA-AA-A00L-01A	COAD	6.57
TCGA-CM-6161-01A	COAD	6.79
TCGA-AA-3980-01A	COAD	6.6
TCGA-CM-5341-01A	COAD	7.22
TCGA-CK-4951-01A	COAD	6.87
TCGA-A6-2678-01A	COAD	7.74
TCGA-AA-3869-01A	COAD	7.09
TCGA-QG-A5YX-01A	COAD	6.95
TCGA-F4-6808-01A	COAD	6.14
TCGA-G4-6306-01A	COAD	6.87
TCGA-G4-6626-01A	COAD	6.67
TCGA-G4-6294-01A	COAD	7.1
TCGA-AA-3858-01A	COAD	6.91
TCGA-DM-A28F-01A	COAD	7.13
TCGA-CA-5797-01A	COAD	7.59
TCGA-A6-6138-01A	COAD	7.31
TCGA-G4-6307-01A	COAD	6.29
TCGA-AY-6386-01A	COAD	7.16
TCGA-AZ-4614-01A	COAD	6.59
TCGA-AA-3555-01A	COAD	7.52
TCGA-AM-5820-01A	COAD	7.03
TCGA-AA-3560-01A	COAD	7.37
TCGA-D5-6541-01A	COAD	6.8
TCGA-CM-6676-01A	COAD	6.95
TCGA-A6-6649-01A	COAD	6.94
TCGA-A6-2671-01A	COAD	6.16
TCGA-AA-3812-01A	COAD	5.97
TCGA-A6-3807-01A	COAD	7.32
TCGA-AA-A01Q-01A	COAD	6.1
TCGA-AA-3956-01A	COAD	7.03
TCGA-A6-2681-01A	COAD	7.02
TCGA-AA-3841-01A	COAD	6.44
TCGA-CM-4752-01A	COAD	6.8
TCGA-WS-AB45-01A	COAD	6.97
TCGA-DM-A280-01A	COAD	6.83
TCGA-QG-A5Z2-01A	COAD	7.21

TCGA-AA-3496-01A	COAD	6.66
TCGA-A6-A56B-01A	COAD	7.08
TCGA-A6-6780-01A	COAD	7.64
TCGA-4N-A93T-01A	COAD	7.04
TCGA-AA-3930-01A	COAD	7.16
TCGA-CK-6747-01A	COAD	7.05
TCGA-CA-6715-01A	COAD	7.26
TCGA-A6-3809-01B	COAD	5.39
TCGA-G4-6588-01A	COAD	7.37
TCGA-AZ-6605-01A	COAD	6.92
TCGA-DM-A1D4-01A	COAD	7.11
TCGA-F4-6807-01A	COAD	7.32
TCGA-QG-A5YW-01A	COAD	5.31
TCGA-CK-6748-01A	COAD	7.38
TCGA-A6-5662-01A	COAD	7.79
TCGA-CM-6163-01A	COAD	7.33
TCGA-AA-3664-01A	COAD	5.91
TCGA-A6-6652-01A	COAD	6.89
TCGA-D5-6538-01A	COAD	6.97
TCGA-CM-5860-01A	COAD	8.05
TCGA-AA-A01K-01A	COAD	6.7
TCGA-5M-AATA-01A	COAD	6.82
TCGA-AA-3532-01A	COAD	7.43
TCGA-AA-3696-01A	COAD	6.81
TCGA-G4-6298-01A	COAD	7.05
TCGA-AA-3681-01A	COAD	7.09
TCGA-G4-6314-01A	COAD	7.06
TCGA-D5-6532-01A	COAD	6.66
TCGA-CM-5862-01A	COAD	7.35
TCGA-CM-5861-01A	COAD	7.29
TCGA-AA-A01R-01A	COAD	6.04
TCGA-AA-3525-01A	COAD	6.83
TCGA-AA-A00Z-01A	COAD	6.55
TCGA-A6-5659-01A	COAD	7.32
TCGA-A6-4107-01A	COAD	6.82
TCGA-AZ-6598-01A	COAD	7.55
TCGA-AA-3845-01A	COAD	7.39
TCGA-G4-6322-01A	COAD	6.64
TCGA-AA-3692-01A	COAD	6.75
TCGA-AZ-4323-01A	COAD	6.39
TCGA-A6-6781-01B	COAD	6.52
TCGA-AZ-4315-01A	COAD	7.16
TCGA-AA-3534-01A	COAD	6.84
TCGA-CA-6718-01A	COAD	7.6
TCGA-G4-6295-01A	COAD	7.66
TCGA-A6-2677-01A	COAD	7.41
TCGA-AD-A5EK-01A	COAD	6.96
TCGA-AZ-4616-01A	COAD	7
TCGA-D5-6920-01A	COAD	6.93
TCGA-F4-6809-01A	COAD	7.19
TCGA-AZ-6599-01A	COAD	7.25
TCGA-DM-A1DA-01A	COAD	7.45
TCGA-DM-A28K-01A	COAD	6.8
TCGA-A6-6782-01A	COAD	6.54
TCGA-AA-3531-01A	COAD	7
TCGA-A6-2674-01B	COAD	7.15
TCGA-AA-3877-01A	COAD	7.36
TCGA-NH-A6GA-01A	COAD	7.73

TCGA-AA-3986-01A	COAD	6.8
TCGA-CK-4952-01A	COAD	7.25
TCGA-A6-6141-01A	COAD	6.86
TCGA-G4-6625-01A	COAD	7.02
TCGA-AA-A02H-01A	COAD	6.62
TCGA-CK-4948-01B	COAD	6.84
TCGA-AD-6964-01A	COAD	6.9
TCGA-AA-A022-01A	COAD	6.66
TCGA-AA-3673-01A	COAD	7.28
TCGA-AA-3947-01A	COAD	8.54
TCGA-AA-3488-01A	COAD	7.26
TCGA-AA-3970-01A	COAD	7.3
TCGA-F4-6704-01A	COAD	6.98
TCGA-AY-A8YK-01A	COAD	7.1
TCGA-CM-4750-01A	COAD	6.37
TCGA-A6-6142-01A	COAD	6.98
TCGA-AA-3864-01A	COAD	7.39
TCGA-AA-3870-01A	COAD	7.43
TCGA-NH-A5IV-01A	COAD	7.16
TCGA-CK-5912-01A	COAD	7.5
TCGA-AA-3667-01A	COAD	6.08
TCGA-CM-6167-01A	COAD	6.9
TCGA-AA-3867-01A	COAD	7.28
TCGA-CM-4743-01A	COAD	7.2
TCGA-DM-A28M-01A	COAD	7.04
TCGA-AA-3554-01A	COAD	7.03
TCGA-AA-3517-01A	COAD	6.65
TCGA-AA-3968-01A	COAD	7.24
TCGA-AA-3949-01A	COAD	7.43
TCGA-A6-5657-01A	COAD	7.13
TCGA-G4-6309-01A	COAD	7.24
TCGA-A6-5664-01A	COAD	6.84
TCGA-CM-6162-01A	COAD	7.08
TCGA-AA-3814-01A	COAD	7.02
TCGA-AA-3680-01A	COAD	6.67
TCGA-D5-6530-01A	COAD	7.01
TCGA-AA-3556-01A	COAD	6.67
TCGA-CM-6165-01A	COAD	7.5
TCGA-AA-3710-01A	COAD	7.48
TCGA-D5-6533-01A	COAD	7.36
TCGA-AA-3818-01A	COAD	6.97
TCGA-AA-A00W-01A	COAD	6.82
TCGA-A6-2680-01A	COAD	6.73
TCGA-DM-A1D7-01A	COAD	6.81
TCGA-DM-A28G-01A	COAD	6.67
TCGA-A6-2683-01A	COAD	6.34
TCGA-G4-6320-01A	COAD	6.94
TCGA-AA-3549-01A	COAD	7.06
TCGA-AA-3685-01A	COAD	7.47
TCGA-DM-A282-01A	COAD	6.55
TCGA-CM-6171-01A	COAD	7.35
TCGA-F4-6463-01A	COAD	7.1
TCGA-D5-6539-01A	COAD	6.64
TCGA-AA-3544-01A	COAD	6.31
TCGA-A6-2685-01A	COAD	6.63
TCGA-CM-6680-01A	COAD	7.06
TCGA-AA-A01T-01A	COAD	4.89
TCGA-AA-3973-01A	COAD	7.2

TCGA-F4-6570-01A	COAD	7.33
TCGA-AA-3984-01A	COAD	7.25
TCGA-AD-6890-01A	COAD	6.54
TCGA-AA-A01D-01A	COAD	6.59
TCGA-AD-6899-01A	COAD	7.24
TCGA-D5-6928-01A	COAD	6.86
TCGA-DM-A1D6-01A	COAD	7.24
TCGA-AA-A02F-01A	COAD	6.41
TCGA-AU-3779-01A	COAD	7.15
TCGA-A6-5656-01A	COAD	6.98
TCGA-A6-5656-01B	COAD	7.26
TCGA-D5-6536-01A	COAD	5.63
TCGA-F4-6805-01A	COAD	7.44
TCGA-AA-A00N-01A	COAD	6.46
TCGA-AY-6197-01A	COAD	7.38
TCGA-G4-6321-01A	COAD	6.58
TCGA-DM-A28C-01A	COAD	6.73
TCGA-AZ-4682-01B	COAD	7.34
TCGA-AZ-6608-01A	COAD	7.05
TCGA-CM-6675-01A	COAD	6.5
TCGA-AA-3660-01A	COAD	6.97
TCGA-D5-6531-01A	COAD	6.41
TCGA-AA-3846-01A	COAD	7.4
TCGA-D5-6537-01A	COAD	7.22
TCGA-A6-5661-01B	COAD	4.85
TCGA-CM-5864-01A	COAD	7.45
TCGA-AA-A00J-01A	COAD	7.43
TCGA-RU-A8FL-01A	COAD	6.88
TCGA-D5-6929-01A	COAD	6.76
TCGA-AA-3861-01A	COAD	7.26
TCGA-AD-A5EJ-01A	COAD	7.1
TCGA-G4-6586-01A	COAD	6.97
TCGA-A6-6650-01A	COAD	7.8
TCGA-G4-6302-01A	COAD	6.86
TCGA-A6-5661-01A	COAD	7.84
TCGA-A6-2674-01A	COAD	6.94
TCGA-A6-5665-01A	COAD	7.93
TCGA-A6-2684-01C	COAD	6.77
TCGA-A6-5665-01B	COAD	6.43
TCGA-AA-3862-01A	COAD	7.67
TCGA-AD-6895-01A	COAD	7.13
TCGA-AA-3875-01A	COAD	6.85
TCGA-NH-A8F7-01A	COAD	6.27
TCGA-AA-3955-01A	COAD	7.38
TCGA-A6-6653-01A	COAD	6.83
TCGA-CA-5255-01A	COAD	7.18
TCGA-NH-A8F7-06A	COAD	6.84
TCGA-AA-A00F-01A	COAD	6.14
TCGA-AA-A02O-01A	COAD	6.01
TCGA-AA-3852-01A	COAD	6.78
TCGA-DM-A0X9-01A	COAD	7.11
TCGA-AA-A02W-01A	COAD	6.65
TCGA-AA-A01G-01A	COAD	6.7
TCGA-DM-A1HA-01A	COAD	8.05
TCGA-5M-AAT5-01A	COAD	6.42
TCGA-NH-A50T-01A	COAD	6.75
TCGA-AA-3494-01A	COAD	6.73
TCGA-CM-4747-01A	COAD	6.35

TCGA-D5-6931-01A	COAD	7.31
TCGA-AA-3713-01A	COAD	7.48
TCGA-AZ-4681-01A	COAD	6.6
TCGA-G4-6297-01A	COAD	7.28
TCGA-AZ-6601-01A	COAD	7.22
TCGA-AA-A01S-01A	COAD	6.19
TCGA-CM-6679-01A	COAD	6.77
TCGA-A6-A567-01A	COAD	6.7
TCGA-AA-A02J-01A	COAD	7
TCGA-CK-4947-01B	COAD	7.35
TCGA-A6-3809-01A	COAD	7.9
TCGA-DM-A1D8-01A	COAD	7.07
TCGA-A6-5659-01B	COAD	5.23
TCGA-A6-4105-01A	COAD	7.4
TCGA-AA-3542-01A	COAD	6.26
TCGA-AA-A029-01A	COAD	6.69
TCGA-DM-A1HB-01A	COAD	7.1
TCGA-DM-A285-01A	COAD	6.56
TCGA-SS-A7HO-01A	COAD	6.95
TCGA-AA-3842-01A	COAD	7.94
TCGA-AA-A01C-01A	COAD	6.54
TCGA-AA-3509-01A	COAD	7.57
TCGA-AA-3939-01A	COAD	7.81
TCGA-T9-A92H-01A	COAD	7.12
TCGA-DM-A28E-01A	COAD	7.2
TCGA-CM-6169-01A	COAD	7.06
TCGA-AD-6889-01A	COAD	7.48
TCGA-AA-A01I-01A	COAD	6.97
TCGA-4T-AA8H-01A	COAD	7.07
TCGA-AA-3524-01A	COAD	7.32
TCGA-A6-A5ZU-01A	COAD	5.82
TCGA-AA-3982-01A	COAD	7.25
TCGA-AZ-6603-01A	COAD	6.85
TCGA-CM-6164-01A	COAD	7.08
TCGA-CK-5914-01A	COAD	7.28
TCGA-AY-4070-01A	COAD	6.06
TCGA-AZ-6607-01A	COAD	7.48
TCGA-A6-6650-01B	COAD	7.4
TCGA-AA-3854-01A	COAD	7.1
TCGA-CA-5796-01A	COAD	7.61
TCGA-A6-2682-01A	COAD	6.11
TCGA-AZ-5403-01A	COAD	7.66
TCGA-A6-A566-01A	COAD	7.05
TCGA-A6-6780-01B	COAD	5.93
TCGA-AA-3492-01A	COAD	8.17
TCGA-AA-3941-01A	COAD	7.57
TCGA-AA-3526-01A	COAD	6.62
TCGA-F4-6459-01A	COAD	7.02
TCGA-AA-3511-01A	COAD	6.66
TCGA-CK-6746-01A	COAD	7.98
TCGA-A6-6654-01A	COAD	6.83
TCGA-AA-3994-01A	COAD	6.86
TCGA-AA-3543-01A	COAD	7.18
TCGA-AA-3502-01A	COAD	7.48
TCGA-CA-6716-01A	COAD	7.09
TCGA-AA-3972-01A	COAD	6.95
TCGA-G4-6311-01A	COAD	6.88
TCGA-A6-2676-01A	COAD	7.16

TCGA-DM-A0XD-01A	COAD	6.32
TCGA-D5-5541-01A	COAD	7.26
TCGA-AA-3520-01A	COAD	7.42
TCGA-CM-6674-01A	COAD	7.6
TCGA-AM-5821-01A	COAD	7.68
TCGA-F4-6856-01A	COAD	7.52
TCGA-A6-6648-01A	COAD	7.15
TCGA-CM-6168-01A	COAD	7.32
TCGA-CK-6751-01A	COAD	6.79
TCGA-AA-A00Q-01A	COAD	6.65
TCGA-AA-A02R-01A	COAD	7.45
TCGA-CM-5863-01A	COAD	6.97
TCGA-CM-4748-01A	COAD	6.79
TCGA-5M-AAT6-01A	COAD	7.05
TCGA-G4-6317-01A	COAD	7.1
TCGA-G4-6317-02A	COAD	7.1
TCGA-AA-3672-01A	COAD	6.21
TCGA-AA-3684-01A	COAD	6.1
TCGA-AA-3679-01A	COAD	7.13
TCGA-D5-6534-01A	COAD	6.7
TCGA-AA-3989-01A	COAD	6.72
TCGA-AY-A54L-01A	COAD	6.03
TCGA-D5-5537-01A	COAD	7.12
TCGA-AA-A02Y-01A	COAD	6.87
TCGA-AA-3666-01A	COAD	7.88
TCGA-A6-3810-01A	COAD	6.82
TCGA-CM-4746-01A	COAD	6.53
TCGA-QG-A5Z1-01A	COAD	6.79
TCGA-AA-3975-01A	COAD	6.59
TCGA-CA-6719-01A	COAD	7.13
TCGA-AA-3678-01A	COAD	7.26
TCGA-A6-6781-01A	COAD	6.99
TCGA-A6-2672-01A	COAD	6.84
TCGA-D5-6535-01A	COAD	7.27
TCGA-AA-3521-01A	COAD	6.82
TCGA-DM-A28H-01A	COAD	7.31
TCGA-5M-AATE-01A	COAD	6.78
TCGA-AA-A017-01A	COAD	6.45
TCGA-AA-3815-01A	COAD	7.45
TCGA-D5-6927-01A	COAD	7.43
TCGA-AZ-5407-01A	COAD	7.63
TCGA-F4-6806-01A	COAD	7.09
TCGA-D5-6540-01A	COAD	6.94
TCGA-D5-6529-01A	COAD	7.09
TCGA-AA-3966-01A	COAD	6.69
TCGA-A6-3810-01B	COAD	7.22
TCGA-AA-3952-01A	COAD	5.76
TCGA-CM-6170-01A	COAD	7.28
TCGA-AA-A004-01A	COAD	4.12
TCGA-AZ-4615-01A	COAD	7.68
TCGA-AA-3529-01A	COAD	7.33
TCGA-F4-6569-01A	COAD	7.03
TCGA-NH-A50V-01A	COAD	7.14
TCGA-AA-3950-01A	COAD	7.48
TCGA-CK-4950-01A	COAD	7.04
TCGA-AZ-6606-01A	COAD	7.14
TCGA-AA-3489-01A	COAD	7.1
TCGA-DM-A1DB-01A	COAD	7

TCGA-AA-A03J-01A	COAD	5.75
TCGA-AA-3860-01A	COAD	6.51
TCGA-AY-A69D-01A	COAD	6.86
TCGA-CM-6166-01A	COAD	7.51
TCGA-AZ-6600-01A	COAD	7.47
TCGA-AA-3979-01A	COAD	7.39
TCGA-CM-5344-01A	COAD	6.81
TCGA-D5-6923-01A	COAD	7.34
TCGA-CM-6677-01A	COAD	7.23
TCGA-AA-3855-01A	COAD	6.5
TCGA-AA-3518-01A	COAD	7.34
TCGA-NH-A50U-01A	COAD	6.71
TCGA-CK-5915-01A	COAD	7.65
TCGA-A6-2672-01B	COAD	6.04
TCGA-DM-A28A-01A	COAD	7.45
TCGA-AD-6901-01A	COAD	6.86
TCGA-AA-3663-01A	COAD	7.67
TCGA-CM-5348-01A	COAD	6.93
TCGA-QG-A5YV-01A	COAD	7.08
TCGA-AD-6965-01A	COAD	7.36
TCGA-AA-A01Z-01A	COAD	6.51
TCGA-AA-3715-01A	COAD	7.46
TCGA-AA-3811-01A	COAD	7.11
TCGA-QL-A97D-01A	COAD	7.4
TCGA-D5-7000-01A	COAD	7.25
TCGA-DM-A1D9-01A	COAD	6.3
TCGA-AA-3514-01A	COAD	6.09
TCGA-A6-2677-01B	COAD	7.14
TCGA-AY-A71X-01A	COAD	6.74
TCGA-AA-A024-01A	COAD	6.07
TCGA-AA-3553-01A	COAD	7.35
TCGA-AA-3538-01A	COAD	7.45
TCGA-G4-6315-01A	COAD	7.03
TCGA-NH-A6GB-01A	COAD	6.41
TCGA-AA-3697-01A	COAD	7.29
TCGA-AA-3516-01A	COAD	7.32
TCGA-AZ-4684-01A	COAD	7.16
TCGA-3L-AA1B-01A	COAD	6.77
TCGA-AD-6548-01A	COAD	7
TCGA-AA-3831-01A	COAD	6.91
TCGA-DM-A0XF-01A	COAD	7.42
TCGA-D5-6924-01A	COAD	7.14
TCGA-CM-4744-01A	COAD	7
TCGA-CM-6678-01A	COAD	7.33
TCGA-AA-A00R-01A	COAD	6.73
TCGA-AA-3522-01A	COAD	8.08
TCGA-A6-A565-01A	COAD	6.56
TCGA-AA-3662-01A	COAD	6.64
TCGA-CM-4751-01A	COAD	7.12
TCGA-AA-3848-01A	COAD	6.06
TCGA-AA-3971-01A	COAD	6.73
TCGA-A6-5660-01A	COAD	7.56
TCGA-AY-5543-01A	COAD	7.48
TCGA-AA-3530-01A	COAD	7.29
TCGA-F4-6855-01A	COAD	7.33
TCGA-DM-A288-01A	COAD	6.87
TCGA-G4-6310-01A	COAD	7.16
TCGA-G4-6303-01A	COAD	7.74

TCGA-D5-6898-01A	COAD	7.37
TCGA-A6-6140-01A	COAD	7
TCGA-AA-3821-01A	COAD	7.11
TCGA-AA-3562-01A	COAD	7.12
TCGA-G4-6323-01A	COAD	6.02
TCGA-D5-6932-01A	COAD	7.43
TCGA-AA-A01P-01A	COAD	6.6
TCGA-AA-3851-01A	COAD	7.05
TCGA-G4-6293-01A	COAD	6.55
TCGA-CM-5349-01A	COAD	6.36
TCGA-AA-A01F-01A	COAD	6.91
TCGA-D5-5539-01A	COAD	7.05
TCGA-AA-3819-01A	COAD	7.1
TCGA-NH-A8F8-01A	COAD	6.77
TCGA-AA-3695-01A	COAD	7.18
TCGA-AA-3850-01A	COAD	6.72
TCGA-AA-3552-01A	COAD	6.57
TCGA-AA-3506-01A	COAD	6.82
TCGA-CA-6717-01A	COAD	7.22
TCGA-5M-AAT4-01A	COAD	7.07
TCGA-A6-6651-01A	COAD	6.91
TCGA-AA-A00E-01A	COAD	6.97
TCGA-AA-3856-01A	COAD	6.71
TCGA-AA-3976-01A	COAD	7.87
TCGA-A6-2679-01A	COAD	4.96
TCGA-AA-3837-01A	COAD	7.01
TCGA-DM-A1D0-01A	COAD	5.97
TCGA-G4-6627-01A	COAD	7.05
TCGA-A6-2686-01A	COAD	7.38
TCGA-A6-5666-01A	COAD	7.53
TCGA-AA-3833-01A	COAD	7.2
TCGA-NH-A6GC-01A	COAD	5.92
TCGA-AA-A010-01A	COAD	6.75
TCGA-A6-2675-01A	COAD	7.24
TCGA-AA-A00D-01A	COAD	6.71
TCGA-AA-A00U-01A	COAD	6.64
TCGA-AA-A01V-01A	COAD	6.43
TCGA-AA-3519-01A	COAD	6.55
TCGA-F4-6703-01A	COAD	7.09
TCGA-GS-A9TY-01A	DLBC	5.75
TCGA-G8-6914-01A	DLBC	5.84
TCGA-FF-8041-01A	DLBC	5.64
TCGA-RQ-A68N-01A	DLBC	6.71
TCGA-G8-6907-01A	DLBC	5.31
TCGA-GS-A9TT-01A	DLBC	7.3
TCGA-FF-8046-01A	DLBC	6.11
TCGA-FA-A6HO-01A	DLBC	6.81
TCGA-G8-6324-01A	DLBC	6.13
TCGA-G8-6326-01A	DLBC	5.96
TCGA-FM-8000-01A	DLBC	6.36
TCGA-GS-A9TQ-01A	DLBC	7.26
TCGA-G8-6906-01A	DLBC	6.46
TCGA-GS-A9U4-01A	DLBC	6.78
TCGA-GR-A4D9-01B	DLBC	7.15
TCGA-GS-A9TW-01A	DLBC	6.56
TCGA-GS-A9TZ-01A	DLBC	6.69
TCGA-FA-A82F-01A	DLBC	7.05
TCGA-FF-A7CQ-01A	DLBC	7.4

TCGA-GS-A9U3-01A	DLBC	6.6
TCGA-FF-A7CW-01A	DLBC	6.49
TCGA-FA-A4BB-01A	DLBC	6.48
TCGA-FA-A6HN-01A	DLBC	6.39
TCGA-FF-8062-01A	DLBC	6.31
TCGA-FF-A7CX-01A	DLBC	6.86
TCGA-G8-6909-01A	DLBC	5.61
TCGA-FA-A86F-01A	DLBC	7.06
TCGA-GR-A4D6-01A	DLBC	5.6
TCGA-GR-7351-01A	DLBC	6.37
TCGA-FA-8693-01A	DLBC	6.32
TCGA-VB-A8QN-01A	DLBC	6.8
TCGA-GR-7353-01A	DLBC	5.11
TCGA-GS-A9TV-01A	DLBC	6.74
TCGA-FF-8043-01A	DLBC	6.06
TCGA-GR-A4D4-01A	DLBC	7.12
TCGA-GS-A9TU-01A	DLBC	6.94
TCGA-RQ-AAAT-01A	DLBC	6.81
TCGA-GS-A9TX-01A	DLBC	6.81
TCGA-FA-A7Q1-01A	DLBC	7.02
TCGA-FA-A7DS-01A	DLBC	6.97
TCGA-FF-8042-01A	DLBC	6.57
TCGA-G8-6325-01A	DLBC	6.09
TCGA-FF-A7CR-01A	DLBC	7.29
TCGA-FF-8047-01A	DLBC	6
TCGA-GR-A4D5-01A	DLBC	6.81
TCGA-FA-A4XK-01A	DLBC	6.94
TCGA-FF-8061-01A	DLBC	6.14
TCGA-RQ-A6JB-01A	DLBC	6.8
TCGA-LN-A49Y-01A	ESCA	6.44
TCGA-JY-A6FB-01A	ESCA	7.19
TCGA-VR-A8EY-01A	ESCA	8.21
TCGA-L5-A8NV-01A	ESCA	6.87
TCGA-Q9-A6FW-01A	ESCA	7.51
TCGA-LN-A49L-01A	ESCA	6.45
TCGA-LN-A49P-01A	ESCA	7.6
TCGA-L5-A4ON-01A	ESCA	6.91
TCGA-LN-A9FO-01A	ESCA	7.14
TCGA-L5-A4OI-01A	ESCA	7.38
TCGA-R6-A6L4-01A	ESCA	6.69
TCGA-L5-A8NW-01A	ESCA	6.86
TCGA-L5-A4OT-01A	ESCA	6.73
TCGA-L5-A88W-01A	ESCA	7.46
TCGA-L5-A4OS-01A	ESCA	6.5
TCGA-V5-A7RB-01A	ESCA	6.74
TCGA-2H-A9GR-01A	ESCA	6.33
TCGA-VR-A8EP-01A	ESCA	7.05
TCGA-2H-A9GI-01A	ESCA	6.11
TCGA-IG-A51D-01A	ESCA	7.12
TCGA-LN-A49W-01A	ESCA	7.24
TCGA-L7-A6VZ-01A	ESCA	7.28
TCGA-2H-A9GJ-01A	ESCA	7.14
TCGA-VR-A8EZ-01A	ESCA	7
TCGA-ZR-A9CJ-01B	ESCA	7.76
TCGA-2H-A9GL-01A	ESCA	6.44
TCGA-R6-A6DN-01B	ESCA	7.16
TCGA-L5-A88V-01A	ESCA	7.46
TCGA-VR-A8EU-01A	ESCA	7.88

TCGA-LN-A49N-01A	ESCA	7.92
TCGA-X8-AAAR-01A	ESCA	7.43
TCGA-VR-AA7B-01A	ESCA	7.6
TCGA-L5-A8NJ-01A	ESCA	7.36
TCGA-2H-A9GQ-01A	ESCA	6.15
TCGA-R6-A8W8-01B	ESCA	6.65
TCGA-LN-A49S-01A	ESCA	6.89
TCGA-L5-A8NR-01A	ESCA	7.03
TCGA-LN-A4A6-01A	ESCA	7.85
TCGA-LN-A4A2-01A	ESCA	7.75
TCGA-IC-A6RF-01A	ESCA	8.56
TCGA-LN-A8HZ-01A	ESCA	7.42
TCGA-L5-A4OJ-01A	ESCA	6.1
TCGA-IG-A5S3-01A	ESCA	7.1
TCGA-LN-A49U-01A	ESCA	8.09
TCGA-L5-A891-01A	ESCA	7.43
TCGA-LN-A4A4-01A	ESCA	7.55
TCGA-L5-A8NU-01A	ESCA	5.63
TCGA-LN-A9FR-01A	ESCA	7.79
TCGA-L5-A88Y-01A	ESCA	6.25
TCGA-KH-A6WC-01A	ESCA	6.59
TCGA-R6-A6DQ-01B	ESCA	6.41
TCGA-LN-A7HZ-01A	ESCA	7.27
TCGA-2H-A9GK-01A	ESCA	6.56
TCGA-L5-A43H-01A	ESCA	7.04
TCGA-IG-A3YC-01A	ESCA	6.9
TCGA-IG-A4P3-01A	ESCA	7.46
TCGA-VR-A8EO-01A	ESCA	6.69
TCGA-VR-A8EW-01A	ESCA	7.66
TCGA-R6-A6Y0-01B	ESCA	6.47
TCGA-L5-A8NE-01A	ESCA	6.4
TCGA-L5-A4OO-01A	ESCA	5.8
TCGA-L5-A88T-01A	ESCA	6.98
TCGA-IG-A97H-01A	ESCA	6.26
TCGA-IG-A50L-01A	ESCA	7.15
TCGA-LN-A7HW-01A	ESCA	7.09
TCGA-L5-A8NN-01A	ESCA	7.56
TCGA-L5-A4OM-01A	ESCA	7.9
TCGA-Z6-A9VB-01A	ESCA	7.49
TCGA-JY-A939-01A	ESCA	6.29
TCGA-L5-A43M-01A	ESCA	6.92
TCGA-L5-A43I-01A	ESCA	7.15
TCGA-LN-A7HV-01A	ESCA	6.58
TCGA-L5-A88S-01A	ESCA	6.96
TCGA-L5-A4OW-01A	ESCA	6.64
TCGA-Z6-A8JE-01A	ESCA	8.05
TCGA-LN-A9FQ-01A	ESCA	8.38
TCGA-L5-A4OH-01A	ESCA	6.58
TCGA-LN-A4A3-01A	ESCA	7.35
TCGA-LN-A8I0-01A	ESCA	6.97
TCGA-IC-A6RE-01A	ESCA	6.68
TCGA-V5-AASX-01A	ESCA	7.2
TCGA-L5-A4OR-01A	ESCA	5.69
TCGA-L5-A4OE-01A	ESCA	7.36
TCGA-L5-A8NK-01A	ESCA	7.06
TCGA-IG-A3YA-01A	ESCA	7.11
TCGA-IG-A97I-01A	ESCA	7.83
TCGA-V5-AASV-01A	ESCA	6.2

TCGA-R6-A6KZ-01A	ESCA	7.34
TCGA-LN-A4A8-01A	ESCA	6.98
TCGA-R6-A6XQ-01B	ESCA	6.69
TCGA-IG-A5B8-01A	ESCA	7.56
TCGA-RE-A7BO-01A	ESCA	6.71
TCGA-R6-A8WC-01A	ESCA	7.1
TCGA-L5-A8NL-01A	ESCA	7.03
TCGA-2H-A9GN-01A	ESCA	7.63
TCGA-Q9-A6FU-01A	ESCA	7.01
TCGA-LN-A5U5-01A	ESCA	7.61
TCGA-2H-A9GG-01A	ESCA	5.63
TCGA-2H-A9GM-01A	ESCA	6.47
TCGA-VR-A8EQ-01A	ESCA	7.59
TCGA-JY-A6FG-01A	ESCA	6.72
TCGA-L5-A43E-01A	ESCA	6.52
TCGA-JY-A93D-01A	ESCA	6.91
TCGA-L5-A4OP-01A	ESCA	6.63
TCGA-LN-A7HX-01A	ESCA	8.29
TCGA-IG-A3YB-01A	ESCA	6.25
TCGA-IG-A8O2-01A	ESCA	7.8
TCGA-LN-A9FP-01A	ESCA	7.29
TCGA-VR-AA7I-01A	ESCA	7.01
TCGA-JY-A6F8-01A	ESCA	7.08
TCGA-LN-A49X-01A	ESCA	7.93
TCGA-LN-A8I1-01A	ESCA	5.51
TCGA-L5-A8NQ-01A	ESCA	6.43
TCGA-VR-A8EX-01A	ESCA	6.92
TCGA-L5-A4OF-01A	ESCA	7.69
TCGA-L5-A43J-01A	ESCA	7.25
TCGA-M9-A5M8-01A	ESCA	7.35
TCGA-R6-A6XG-01B	ESCA	6.66
TCGA-VR-A8Q7-01A	ESCA	7.83
TCGA-IG-A4QT-01A	ESCA	5.89
TCGA-V5-A7RE-01A	ESCA	6.36
TCGA-L5-A88Z-01A	ESCA	7.15
TCGA-VR-A8ER-01A	ESCA	7.86
TCGA-JY-A6FD-01A	ESCA	7.76
TCGA-LN-A4MQ-01A	ESCA	6.91
TCGA-Z6-AAPN-01A	ESCA	7.14
TCGA-LN-A49O-01A	ESCA	7.79
TCGA-LN-A49R-01A	ESCA	5.98
TCGA-IG-A7DP-01A	ESCA	5.97
TCGA-S8-A6BV-01A	ESCA	6.51
TCGA-R6-A6L6-01B	ESCA	5.8
TCGA-R6-A8WG-01A	ESCA	7.92
TCGA-L5-A4OX-01A	ESCA	7.18
TCGA-IG-A4QS-01A	ESCA	6.28
TCGA-R6-A6Y2-01B	ESCA	6.55
TCGA-L7-A56G-01A	ESCA	7
TCGA-LN-A49V-01A	ESCA	6.66
TCGA-L5-A8NT-01A	ESCA	7.24
TCGA-LN-A7HY-01A	ESCA	6.47
TCGA-XP-A8T8-01A	ESCA	7.75
TCGA-JY-A938-01A	ESCA	7.19
TCGA-L5-A8NG-01A	ESCA	6.88
TCGA-V5-A7RC-06A	ESCA	6.96
TCGA-V5-A7RC-01B	ESCA	6.85
TCGA-LN-A4MR-01A	ESCA	5.77

TCGA-LN-A5U7-01A	ESCA	7.4
TCGA-XP-A8T6-01A	ESCA	7.46
TCGA-L5-A893-01A	ESCA	6.67
TCGA-L5-A4OG-01A	ESCA	6.93
TCGA-VR-A8ET-01A	ESCA	5.97
TCGA-LN-A4A9-01A	ESCA	6.65
TCGA-V5-AASW-01A	ESCA	6.54
TCGA-2H-A9GO-01A	ESCA	6.24
TCGA-L5-A4OU-01A	ESCA	6.71
TCGA-L5-A43C-01A	ESCA	6.44
TCGA-VR-AA4G-01A	ESCA	7.3
TCGA-S8-A6BW-01A	ESCA	7.88
TCGA-L5-A8NH-01A	ESCA	6.63
TCGA-IG-A3I8-01A	ESCA	7.39
TCGA-IG-A625-01A	ESCA	7.84
TCGA-2H-A9GH-01A	ESCA	7.47
TCGA-JY-A93F-01A	ESCA	6.55
TCGA-R6-A8W5-01B	ESCA	7.28
TCGA-L5-A8NI-01A	ESCA	6.68
TCGA-LN-A5U6-01A	ESCA	7.41
TCGA-LN-A4A1-01A	ESCA	6.81
TCGA-LN-A4A5-01A	ESCA	7.03
TCGA-VR-AA7D-01A	ESCA	7.68
TCGA-JY-A93E-01A	ESCA	6.89
TCGA-LN-A49K-01A	ESCA	7.77
TCGA-L5-A8NS-01A	ESCA	7.16
TCGA-JY-A6FA-01A	ESCA	7.67
TCGA-JY-A6FE-01A	ESCA	7.37
TCGA-IG-A3Y9-01A	ESCA	6.42
TCGA-L5-A4OQ-01A	ESCA	6.12
TCGA-2H-A9GF-01A	ESCA	6.54
TCGA-IG-A6QS-01A	ESCA	7.09
TCGA-LN-A49M-01A	ESCA	6.51
TCGA-Z6-A8JD-01A	ESCA	6.95
TCGA-L5-A8NM-01A	ESCA	7.85
TCGA-JY-A93C-01A	ESCA	5.75
TCGA-JY-A6FH-01A	ESCA	7.08
TCGA-L5-A8NF-01A	ESCA	7.13
TCGA-VR-AA4D-01A	ESCA	6.79
TCGA-IG-A3QL-01A	ESCA	7.5
TCGA-27-2523-01A	GBM	8.02
TCGA-06-0190-02A	GBM	7.09
TCGA-06-0130-01A	GBM	7.43
TCGA-76-4926-01B	GBM	5.8
TCGA-06-0125-02A	GBM	7.43
TCGA-26-5136-01B	GBM	8.13
TCGA-12-0619-01A	GBM	6.65
TCGA-06-5411-01A	GBM	8.02
TCGA-06-5418-01A	GBM	7.32
TCGA-16-0846-01A	GBM	8.58
TCGA-27-2521-01A	GBM	8.85
TCGA-27-2526-01A	GBM	7.6
TCGA-06-0138-01A	GBM	7.49
TCGA-41-2571-01A	GBM	6.95
TCGA-06-0649-01B	GBM	7.49
TCGA-14-0871-01A	GBM	6.25
TCGA-27-2524-01A	GBM	7.71
TCGA-76-4927-01A	GBM	7.72

TCGA-06-0747-01A	GBM	7.93
TCGA-28-2514-01A	GBM	8.14
TCGA-19-1787-01B	GBM	7.52
TCGA-06-0141-01A	GBM	6.76
TCGA-32-2632-01A	GBM	7.57
TCGA-06-5859-01A	GBM	8.11
TCGA-14-0789-01A	GBM	7.31
TCGA-06-0645-01A	GBM	7.65
TCGA-14-2554-01A	GBM	8.09
TCGA-06-2557-01A	GBM	7.58
TCGA-06-0211-02A	GBM	7.01
TCGA-06-0211-01A	GBM	7.52
TCGA-27-1835-01A	GBM	7.91
TCGA-14-0781-01B	GBM	6.68
TCGA-19-4065-02A	GBM	6.97
TCGA-41-4097-01A	GBM	7.65
TCGA-06-0139-01A	GBM	6.66
TCGA-14-0790-01B	GBM	8.4
TCGA-41-2572-01A	GBM	7.42
TCGA-06-0882-01A	GBM	7.49
TCGA-06-0156-01A	GBM	7.5
TCGA-06-0184-01A	GBM	7.27
TCGA-26-5132-01A	GBM	7.58
TCGA-19-2624-01A	GBM	7.32
TCGA-28-1747-01C	GBM	8.73
TCGA-32-2638-01A	GBM	7.79
TCGA-19-1389-02A	GBM	6.87
TCGA-06-0219-01A	GBM	8.18
TCGA-28-2513-01A	GBM	7.34
TCGA-06-5416-01A	GBM	7.83
TCGA-02-0047-01A	GBM	8.7
TCGA-06-0750-01A	GBM	6.83
TCGA-19-1390-01A	GBM	7.62
TCGA-32-2634-01A	GBM	7.76
TCGA-06-0187-01A	GBM	7.99
TCGA-26-5134-01A	GBM	7.06
TCGA-12-3653-01A	GBM	7.65
TCGA-08-0386-01A	GBM	7.7
TCGA-32-4213-01A	GBM	7.04
TCGA-32-2615-01A	GBM	7.8
TCGA-15-1444-01A	GBM	8.52
TCGA-28-5208-01A	GBM	8.07
TCGA-02-2485-01A	GBM	8.66
TCGA-12-3650-01A	GBM	7.8
TCGA-14-1034-02B	GBM	7.95
TCGA-12-1597-01B	GBM	7.87
TCGA-16-1045-01B	GBM	7.5
TCGA-14-1402-02A	GBM	8.43
TCGA-28-5215-01A	GBM	7.63
TCGA-06-2558-01A	GBM	8.05
TCGA-27-1832-01A	GBM	7.16
TCGA-06-2565-01A	GBM	7.94
TCGA-06-5410-01A	GBM	7.19
TCGA-76-4931-01A	GBM	7.91
TCGA-06-5415-01A	GBM	8.52
TCGA-28-5220-01A	GBM	8.12
TCGA-76-4925-01A	GBM	7.7
TCGA-06-5413-01A	GBM	8.54

TCGA-06-2564-01A	GBM	7.76
TCGA-06-0878-01A	GBM	7.69
TCGA-28-5216-01A	GBM	8.41
TCGA-14-0736-02A	GBM	7.45
TCGA-32-1982-01A	GBM	7.37
TCGA-27-2528-01A	GBM	8.61
TCGA-76-4929-01A	GBM	7.78
TCGA-28-5218-01A	GBM	6.74
TCGA-06-2559-01A	GBM	7.47
TCGA-41-5651-01A	GBM	7.68
TCGA-28-1753-01A	GBM	7.29
TCGA-28-2509-01A	GBM	8.32
TCGA-06-0132-01A	GBM	7.53
TCGA-02-2483-01A	GBM	7.78
TCGA-06-0190-01A	GBM	7.23
TCGA-02-2486-01A	GBM	7.25
TCGA-06-0743-01A	GBM	7.51
TCGA-32-5222-01A	GBM	8.26
TCGA-12-0616-01A	GBM	6.91
TCGA-06-0157-01A	GBM	7.67
TCGA-06-0152-02A	GBM	7.83
TCGA-27-1831-01A	GBM	8.39
TCGA-19-2619-01A	GBM	7.5
TCGA-06-0646-01A	GBM	7.63
TCGA-12-0618-01A	GBM	7.59
TCGA-12-0821-01A	GBM	8.12
TCGA-06-5856-01A	GBM	8.24
TCGA-06-0210-01A	GBM	6.78
TCGA-76-4928-01B	GBM	7.39
TCGA-06-0238-01A	GBM	7.24
TCGA-14-1825-01A	GBM	8.07
TCGA-76-4932-01A	GBM	8
TCGA-06-0749-01A	GBM	7.75
TCGA-26-5139-01A	GBM	7.93
TCGA-28-2499-01A	GBM	8.09
TCGA-06-2561-01A	GBM	7.43
TCGA-02-0055-01A	GBM	7.45
TCGA-32-1980-01A	GBM	7.96
TCGA-19-2625-01A	GBM	6.91
TCGA-06-0745-01A	GBM	7.88
TCGA-06-2567-01A	GBM	7.07
TCGA-06-0744-01A	GBM	7.53
TCGA-06-0171-02A	GBM	6.81
TCGA-06-0178-01A	GBM	8.57
TCGA-32-2616-01A	GBM	7.99
TCGA-14-1829-01A	GBM	7.63
TCGA-14-0817-01A	GBM	7.64
TCGA-14-1823-01A	GBM	7.21
TCGA-19-0957-02A	GBM	7.5
TCGA-06-0129-01A	GBM	8.42
TCGA-26-1442-01A	GBM	8.85
TCGA-06-0686-01A	GBM	7.56
TCGA-12-5295-01A	GBM	8.42
TCGA-06-1804-01A	GBM	7.22
TCGA-19-5960-01A	GBM	8.09
TCGA-06-0125-01A	GBM	7.63
TCGA-06-5408-01A	GBM	7.72
TCGA-14-0787-01A	GBM	7.79

TCGA-19-4065-01A	GBM	7.32
TCGA-06-2570-01A	GBM	8.6
TCGA-26-5133-01A	GBM	7.54
TCGA-27-1837-01A	GBM	7.69
TCGA-06-5858-01A	GBM	7.68
TCGA-19-2629-01A	GBM	7.62
TCGA-06-2563-01A	GBM	8.86
TCGA-06-0158-01A	GBM	8.27
TCGA-06-2562-01A	GBM	7.09
TCGA-06-0210-02A	GBM	7.2
TCGA-06-5412-01A	GBM	7.16
TCGA-28-5207-01A	GBM	8.45
TCGA-06-0174-01A	GBM	7.66
TCGA-15-0742-01A	GBM	7.24
TCGA-06-0168-01A	GBM	7.45
TCGA-19-2620-01A	GBM	7.83
TCGA-26-5135-01A	GBM	6.98
TCGA-06-2569-01A	GBM	7.13
TCGA-32-1970-01A	GBM	7.59
TCGA-06-0211-01B	GBM	7.82
TCGA-12-5299-01A	GBM	8.33
TCGA-27-1830-01A	GBM	7.42
TCGA-27-1834-01A	GBM	7.98
TCGA-27-2519-01A	GBM	8.42
TCGA-28-5204-01A	GBM	7.78
TCGA-41-3915-01A	GBM	7.6
TCGA-06-0644-01A	GBM	7.23
TCGA-28-2510-01A	GBM	7.94
TCGA-28-5213-01A	GBM	7.47
TCGA-06-5414-01A	GBM	8.15
TCGA-06-5417-01A	GBM	9.38
TCGA-06-0221-02A	GBM	8.79
TCGA-28-5209-01A	GBM	7.74
TCGA-12-3652-01A	GBM	7.93
TCGA-14-1034-01A	GBM	8
TCGA-CV-A464-01A	HNSC	6.59
TCGA-CV-6960-01A	HNSC	6.41
TCGA-CV-A45W-01A	HNSC	6.11
TCGA-BA-A4IF-01A	HNSC	7.76
TCGA-CN-5366-01A	HNSC	7.3
TCGA-MT-A51X-01A	HNSC	6.8
TCGA-P3-A5QE-01A	HNSC	4.09
TCGA-CV-6942-01A	HNSC	6.67
TCGA-CN-6022-01A	HNSC	6.69
TCGA-CV-7406-01A	HNSC	6.63
TCGA-UF-A71A-06A	HNSC	7.34
TCGA-CV-7263-01A	HNSC	7.02
TCGA-CV-5430-01A	HNSC	7.35
TCGA-P3-A6T2-01A	HNSC	8.56
TCGA-UF-A7JA-01A	HNSC	6.8
TCGA-CV-7097-01A	HNSC	7.01
TCGA-HD-A634-01A	HNSC	6.65
TCGA-CV-A6K2-01A	HNSC	6.53
TCGA-HL-7533-01A	HNSC	6.96
TCGA-CR-6474-01A	HNSC	7.23
TCGA-CX-7086-01A	HNSC	7.1
TCGA-CV-6962-01A	HNSC	6.96
TCGA-CN-A63T-01A	HNSC	7.61

TCGA-CV-7245-01A	HNSC	6.87
TCGA-CN-5358-01A	HNSC	6.86
TCGA-QK-A8ZA-01A	HNSC	6.6
TCGA-KU-A6H7-06A	HNSC	6.01
TCGA-CR-7376-01A	HNSC	7.31
TCGA-IQ-A61I-01A	HNSC	6.41
TCGA-CR-6480-01A	HNSC	6.95
TCGA-CN-4739-01A	HNSC	6.95
TCGA-CV-6952-01A	HNSC	6.88
TCGA-BB-7872-01A	HNSC	7.38
TCGA-P3-A5QA-01A	HNSC	6.8
TCGA-CR-7370-01A	HNSC	7.09
TCGA-CV-7424-01A	HNSC	6.63
TCGA-CN-A6V3-01A	HNSC	7.88
TCGA-4P-AA8J-01A	HNSC	6.58
TCGA-CQ-5324-01A	HNSC	8.43
TCGA-CR-6467-01A	HNSC	7.34
TCGA-CR-7402-01A	HNSC	7.26
TCGA-P3-A6T7-01A	HNSC	6.88
TCGA-CV-6436-01A	HNSC	6.2
TCGA-CR-7369-01A	HNSC	6.69
TCGA-F7-A50G-01A	HNSC	6.33
TCGA-CQ-6220-01A	HNSC	6.34
TCGA-CV-6936-01A	HNSC	6.26
TCGA-CN-4738-01A	HNSC	7.57
TCGA-BB-A5HZ-01A	HNSC	7.05
TCGA-CN-6021-01A	HNSC	5.86
TCGA-BA-5153-01A	HNSC	7.93
TCGA-CV-7104-01A	HNSC	7.24
TCGA-CQ-A4CH-01A	HNSC	7.21
TCGA-CN-4722-01A	HNSC	6.75
TCGA-HD-8635-01A	HNSC	7.26
TCGA-BA-A6D8-01A	HNSC	6.75
TCGA-CN-5359-01A	HNSC	7.09
TCGA-BA-A6DG-01A	HNSC	6.08
TCGA-BB-4225-01A	HNSC	7.26
TCGA-CQ-A4CB-01A	HNSC	7.8
TCGA-TN-A7HI-01A	HNSC	6.77
TCGA-CV-6956-01A	HNSC	8.62
TCGA-CQ-7068-01A	HNSC	7.55
TCGA-CV-7178-01A	HNSC	6.85
TCGA-F7-A50I-01A	HNSC	7.84
TCGA-CV-7100-01A	HNSC	8.11
TCGA-CV-7428-01A	HNSC	7.88
TCGA-CQ-6221-01A	HNSC	5.98
TCGA-BA-4076-01A	HNSC	6.92
TCGA-F7-A622-01A	HNSC	6.52
TCGA-CN-6012-01A	HNSC	6.7
TCGA-D6-6515-01A	HNSC	6.79
TCGA-UF-A7J9-01A	HNSC	7.26
TCGA-IQ-A61H-01A	HNSC	7.36
TCGA-P3-A6T8-01A	HNSC	7.44
TCGA-HD-A6HZ-01A	HNSC	7.1
TCGA-CV-7416-01A	HNSC	7.54
TCGA-CQ-5330-01A	HNSC	7.8
TCGA-DQ-5630-01A	HNSC	7.31
TCGA-CQ-6229-01A	HNSC	6.95
TCGA-T2-A6X2-01A	HNSC	7.01

TCGA-CN-A497-01A	HNSC	7.78
TCGA-F7-7848-01A	HNSC	7.59
TCGA-BA-5555-01A	HNSC	6.96
TCGA-F7-A61S-01A	HNSC	6.8
TCGA-F7-A61W-01A	HNSC	7.87
TCGA-CV-6943-01A	HNSC	6.97
TCGA-CQ-6225-01A	HNSC	7.63
TCGA-D6-8569-01A	HNSC	7.57
TCGA-CN-4723-01A	HNSC	7.49
TCGA-CV-6951-01A	HNSC	7.1
TCGA-CN-6020-01A	HNSC	7.7
TCGA-P3-A6T6-01A	HNSC	7.08
TCGA-UF-A71E-01A	HNSC	7.11
TCGA-CV-7438-01A	HNSC	6.88
TCGA-CV-6945-01A	HNSC	5.49
TCGA-CN-4736-01A	HNSC	7.52
TCGA-UF-A7JT-01A	HNSC	7.07
TCGA-CQ-A4CD-01A	HNSC	7.22
TCGA-CV-A6JN-01A	HNSC	5.9
TCGA-CR-7383-01A	HNSC	6.64
TCGA-BB-7866-01A	HNSC	7.18
TCGA-CV-5440-01A	HNSC	7.07
TCGA-CV-A468-01A	HNSC	6.6
TCGA-CR-7367-01A	HNSC	7.07
TCGA-CV-5443-01A	HNSC	6.76
TCGA-KU-A66T-01A	HNSC	6.89
TCGA-P3-A6SX-01A	HNSC	6.48
TCGA-HD-7831-01A	HNSC	6.82
TCGA-UF-A7JH-01A	HNSC	6.6
TCGA-F7-A61V-01A	HNSC	6.96
TCGA-CV-5966-01A	HNSC	8.02
TCGA-CN-4727-01A	HNSC	7.88
TCGA-CQ-A4C7-01A	HNSC	7.99
TCGA-BB-7861-01A	HNSC	6.31
TCGA-CV-5979-01A	HNSC	7.14
TCGA-CV-5970-01A	HNSC	6.28
TCGA-BA-A6DA-01A	HNSC	5.61
TCGA-CV-A45P-01A	HNSC	6.52
TCGA-CV-7103-01A	HNSC	7.63
TCGA-CV-5976-01A	HNSC	7.58
TCGA-MT-A67D-01A	HNSC	6.4
TCGA-CQ-6219-01A	HNSC	7.33
TCGA-UF-A7JV-01A	HNSC	7.05
TCGA-D6-A6EQ-01A	HNSC	6.51
TCGA-DQ-5631-01A	HNSC	7.56
TCGA-CQ-5334-01A	HNSC	7.27
TCGA-BA-A6DE-01A	HNSC	7.22
TCGA-F7-A50J-01A	HNSC	6.12
TCGA-BA-A6DJ-01A	HNSC	7.34
TCGA-UF-A7JS-01A	HNSC	6
TCGA-CV-5971-01A	HNSC	6.59
TCGA-BA-5559-01A	HNSC	6.72
TCGA-CN-4737-01A	HNSC	7.33
TCGA-BA-A6DB-01A	HNSC	6.84
TCGA-UF-A7JK-01A	HNSC	6.96
TCGA-HD-8634-01A	HNSC	7.06
TCGA-UF-A718-01A	HNSC	6.13
TCGA-P3-A6T3-01A	HNSC	7.08

TCGA-CN-6997-01A	HNSC	8.37
TCGA-HD-7754-01A	HNSC	7.71
TCGA-CX-A4AQ-01A	HNSC	6.12
TCGA-CV-7418-01A	HNSC	6.83
TCGA-CQ-7065-01A	HNSC	7.52
TCGA-CQ-A4CI-01A	HNSC	8.59
TCGA-QK-A6IF-01A	HNSC	6.65
TCGA-UF-A7JD-01A	HNSC	6.83
TCGA-CV-7253-01A	HNSC	7.28
TCGA-CQ-6228-01A	HNSC	7.79
TCGA-CR-7379-01A	HNSC	7.38
TCGA-CQ-A4CE-01A	HNSC	6.22
TCGA-CR-5243-01A	HNSC	7.36
TCGA-H7-8502-01A	HNSC	8.2
TCGA-CV-7423-01A	HNSC	6.75
TCGA-MT-A67A-01A	HNSC	5.87
TCGA-BA-7269-01A	HNSC	6.53
TCGA-IQ-7630-01A	HNSC	6.94
TCGA-CV-7254-01A	HNSC	6.99
TCGA-CV-A463-01A	HNSC	6.82
TCGA-BA-6869-01A	HNSC	6.89
TCGA-DQ-7595-01A	HNSC	7.57
TCGA-CR-7374-01A	HNSC	6.81
TCGA-CV-6948-01A	HNSC	7.67
TCGA-CV-6938-01A	HNSC	7.5
TCGA-MZ-A5BI-01A	HNSC	5.38
TCGA-HD-7917-01A	HNSC	7.77
TCGA-CV-7437-01A	HNSC	7.17
TCGA-CV-6953-01A	HNSC	6.82
TCGA-BA-5151-01A	HNSC	7.68
TCGA-T2-A6WZ-01A	HNSC	6.08
TCGA-CV-A45V-01A	HNSC	6.36
TCGA-CV-5436-01A	HNSC	7.6
TCGA-BA-4074-01A	HNSC	7.16
TCGA-CR-7395-01A	HNSC	7.02
TCGA-CR-6481-01A	HNSC	6.45
TCGA-CR-5250-01A	HNSC	7.14
TCGA-BA-A8YP-01A	HNSC	7.37
TCGA-CR-5248-01A	HNSC	6.55
TCGA-CN-A499-01A	HNSC	7.05
TCGA-MZ-A6I9-01A	HNSC	6.99
TCGA-CN-6010-01A	HNSC	7.39
TCGA-TN-A7HJ-01A	HNSC	6.96
TCGA-HD-A633-01A	HNSC	7.39
TCGA-CV-7433-01A	HNSC	6.84
TCGA-CR-6493-01A	HNSC	7.93
TCGA-D6-A6ES-01A	HNSC	4.1
TCGA-DQ-5624-01A	HNSC	7.34
TCGA-F7-8298-01A	HNSC	7.04
TCGA-CV-7180-01A	HNSC	7.26
TCGA-IQ-A61E-01A	HNSC	7.2
TCGA-IQ-A61G-01A	HNSC	6.96
TCGA-CV-A6K0-01B	HNSC	7
TCGA-CV-6433-01A	HNSC	7.79
TCGA-IQ-A61J-01A	HNSC	6.67
TCGA-RS-A6TO-01A	HNSC	6.61
TCGA-CQ-5323-01A	HNSC	7.06
TCGA-CV-A45Z-01A	HNSC	7.31

TCGA-CR-7401-01A	HNSC	6.99
TCGA-CQ-6223-01A	HNSC	6.73
TCGA-P3-A6T0-01A	HNSC	6.33
TCGA-HD-A6I0-01A	HNSC	5.86
TCGA-CR-7404-01A	HNSC	7.19
TCGA-D6-A6EN-01A	HNSC	7.45
TCGA-HD-8314-01A	HNSC	7.79
TCGA-CN-6019-01A	HNSC	7.09
TCGA-CV-7568-01A	HNSC	7.82
TCGA-CN-6989-01A	HNSC	7.63
TCGA-BB-7864-01A	HNSC	8.69
TCGA-D6-6823-01A	HNSC	8.43
TCGA-CV-6937-01A	HNSC	7.19
TCGA-CN-4735-01A	HNSC	7.35
TCGA-DQ-5629-01A	HNSC	7.79
TCGA-CV-7421-01A	HNSC	7.46
TCGA-CQ-7067-01A	HNSC	7.01
TCGA-CN-4740-01A	HNSC	7.34
TCGA-CN-4733-01A	HNSC	6.77
TCGA-CQ-6222-01A	HNSC	6.58
TCGA-CV-6941-01A	HNSC	7.11
TCGA-D6-6516-01A	HNSC	6.33
TCGA-CR-7394-01A	HNSC	6.34
TCGA-CV-6955-01A	HNSC	5.8
TCGA-D6-A6EK-01A	HNSC	5.44
TCGA-CR-7377-01A	HNSC	7.48
TCGA-BA-6868-01B	HNSC	6.84
TCGA-WA-A7GZ-01A	HNSC	6.94
TCGA-T2-A6X0-01A	HNSC	6.2
TCGA-CV-7425-01A	HNSC	6.55
TCGA-CV-7177-01A	HNSC	7.79
TCGA-CV-5441-01A	HNSC	7.45
TCGA-CV-7414-01A	HNSC	6.17
TCGA-BA-A4IH-01A	HNSC	7.02
TCGA-T3-A92M-01A	HNSC	6.99
TCGA-BA-A6DL-01A	HNSC	8.07
TCGA-CQ-A4CA-01A	HNSC	6.24
TCGA-CQ-5326-01A	HNSC	7.94
TCGA-CQ-5331-01A	HNSC	6.42
TCGA-QK-A6V9-01A	HNSC	4.18
TCGA-CV-5435-01A	HNSC	8.04
TCGA-IQ-7631-01A	HNSC	6.69
TCGA-CN-5356-01A	HNSC	7.47
TCGA-UF-A71B-01A	HNSC	6.58
TCGA-DQ-7590-01A	HNSC	6.38
TCGA-CR-6487-01A	HNSC	6.98
TCGA-CN-A642-01A	HNSC	7.41
TCGA-CV-A45O-01A	HNSC	4.71
TCGA-CV-7409-01A	HNSC	7.98
TCGA-CR-7385-01A	HNSC	7.03
TCGA-CR-6484-01A	HNSC	6.47
TCGA-CV-A6JU-01A	HNSC	6.43
TCGA-CN-A49C-01A	HNSC	6.7
TCGA-BB-4227-01A	HNSC	7.6
TCGA-CN-5369-01A	HNSC	6.68
TCGA-CV-7432-01A	HNSC	7.7
TCGA-CV-7415-01A	HNSC	6.87
TCGA-D6-6517-01A	HNSC	7.2

TCGA-H7-A6C4-01A	HNSC	6.74
TCGA-CV-6939-01A	HNSC	7.43
TCGA-CV-7243-01A	HNSC	6.28
TCGA-D6-A74Q-01A	HNSC	6.83
TCGA-C9-A480-01A	HNSC	6.78
TCGA-D6-A6EM-01A	HNSC	6.88
TCGA-F7-A623-01A	HNSC	7.28
TCGA-CN-6016-01A	HNSC	6.66
TCGA-T3-A92N-01A	HNSC	8.35
TCGA-CR-7399-01A	HNSC	7.57
TCGA-CR-6471-01A	HNSC	6.76
TCGA-CR-6488-01A	HNSC	7.16
TCGA-CQ-A4CG-01A	HNSC	7.06
TCGA-BB-7862-01A	HNSC	7.5
TCGA-CV-6959-01A	HNSC	7.96
TCGA-HD-7753-01A	HNSC	7.18
TCGA-CR-6473-01A	HNSC	8.43
TCGA-H7-8501-01A	HNSC	8.11
TCGA-H7-7774-01A	HNSC	6.18
TCGA-CN-6011-01A	HNSC	7.04
TCGA-BB-A5HU-01A	HNSC	6.55
TCGA-CR-7382-01A	HNSC	7.74
TCGA-UP-A6WW-01A	HNSC	6.04
TCGA-CR-7371-01A	HNSC	7.88
TCGA-CN-4741-01A	HNSC	7.22
TCGA-CR-7368-01A	HNSC	6.91
TCGA-CR-7373-01A	HNSC	7.38
TCGA-BB-4217-01A	HNSC	7.77
TCGA-QK-A64Z-01A	HNSC	6.91
TCGA-CV-7235-01A	HNSC	7.05
TCGA-CN-6024-01A	HNSC	6.9
TCGA-CN-4730-01A	HNSC	7.84
TCGA-UF-A7JF-01A	HNSC	6.48
TCGA-HD-7832-01A	HNSC	7.9
TCGA-CV-6933-01A	HNSC	7.08
TCGA-CV-A45X-01A	HNSC	6.42
TCGA-CQ-7063-01A	HNSC	6.92
TCGA-CV-7236-01A	HNSC	7.05
TCGA-CN-6995-01A	HNSC	7.94
TCGA-CV-A6K1-01A	HNSC	6.73
TCGA-CN-6998-01A	HNSC	6.72
TCGA-QK-A6VC-01A	HNSC	6.8
TCGA-CN-6988-01A	HNSC	8.04
TCGA-DQ-7589-01A	HNSC	7.12
TCGA-QK-A8Z7-01A	HNSC	7.83
TCGA-CQ-5325-01A	HNSC	6.58
TCGA-CN-4731-01A	HNSC	8.69
TCGA-CV-7247-01A	HNSC	7.43
TCGA-BA-4077-01B	HNSC	7.48
TCGA-CN-5373-01A	HNSC	6.49
TCGA-BB-8596-01A	HNSC	7.34
TCGA-CV-7099-01A	HNSC	7.11
TCGA-CV-7255-01A	HNSC	7.61
TCGA-CR-7380-01A	HNSC	6.82
TCGA-CN-5367-01A	HNSC	6.93
TCGA-CQ-6227-01A	HNSC	7.45
TCGA-DQ-7594-01A	HNSC	7.57
TCGA-CN-5370-01A	HNSC	6.8

TCGA-CV-7434-01A	HNSC	6.88
TCGA-CN-A49A-01A	HNSC	6.24
TCGA-CN-6023-01A	HNSC	7.24
TCGA-CV-7440-01A	HNSC	7.88
TCGA-CV-7411-01A	HNSC	6.24
TCGA-CN-A6UY-01A	HNSC	6.63
TCGA-CV-5431-01A	HNSC	6.19
TCGA-CN-4728-01A	HNSC	7.61
TCGA-CV-A6JZ-01A	HNSC	7.09
TCGA-CN-A63W-01A	HNSC	6.86
TCGA-CN-6992-01A	HNSC	7.92
TCGA-BA-A4IG-01A	HNSC	7.5
TCGA-CQ-A4C6-01A	HNSC	7.08
TCGA-CV-5444-01A	HNSC	6.9
TCGA-CN-6018-01A	HNSC	6.66
TCGA-CN-6994-01A	HNSC	6.99
TCGA-CQ-6218-01A	HNSC	6.67
TCGA-IQ-7632-01A	HNSC	6.26
TCGA-CR-7364-01A	HNSC	7.81
TCGA-CV-A465-01A	HNSC	6.81
TCGA-CN-A641-01A	HNSC	6.59
TCGA-CV-A6JT-01A	HNSC	5.49
TCGA-CN-6013-01A	HNSC	7.08
TCGA-CV-6441-01A	HNSC	8.89
TCGA-BA-5557-01A	HNSC	6.69
TCGA-CN-5365-01A	HNSC	6.69
TCGA-CN-4726-01A	HNSC	7.2
TCGA-CV-A45Q-01A	HNSC	5.45
TCGA-CR-6491-01A	HNSC	7.28
TCGA-CQ-5332-01A	HNSC	8.53
TCGA-UF-A7JJ-01A	HNSC	6.33
TCGA-CR-5247-01A	HNSC	8.58
TCGA-BA-4075-01A	HNSC	7.59
TCGA-C9-A47Z-01A	HNSC	6.28
TCGA-CQ-7071-01A	HNSC	6.7
TCGA-CR-7391-01A	HNSC	6.81
TCGA-CV-A45T-01A	HNSC	6.68
TCGA-QK-A6VB-01A	HNSC	6.56
TCGA-CV-7435-01A	HNSC	7.49
TCGA-CN-5360-01A	HNSC	6.74
TCGA-CQ-A4C9-01A	HNSC	7.6
TCGA-QK-AA3J-01A	HNSC	8.27
TCGA-BB-4224-01A	HNSC	7.66
TCGA-BA-5558-01A	HNSC	7.35
TCGA-DQ-7593-01A	HNSC	6.82
TCGA-CN-A49B-01A	HNSC	8.07
TCGA-BA-6870-01A	HNSC	6.6
TCGA-D6-6824-01A	HNSC	7.62
TCGA-IQ-A6SH-01A	HNSC	6.06
TCGA-CN-A6V7-01A	HNSC	6.81
TCGA-BA-5556-01A	HNSC	7.19
TCGA-MT-A51W-01A	HNSC	6.47
TCGA-HD-A4C1-01A	HNSC	6.89
TCGA-CR-7390-01A	HNSC	7.62
TCGA-D6-6826-01A	HNSC	8.26
TCGA-CX-7085-01A	HNSC	6.43
TCGA-BA-6872-01A	HNSC	6.93
TCGA-CQ-5329-01A	HNSC	8.23

TCGA-P3-A6SW-01A	HNSC	6.5
TCGA-QK-A8ZB-01A	HNSC	7.07
TCGA-CQ-5333-01A	HNSC	6.95
TCGA-QK-AA3K-01A	HNSC	6.35
TCGA-CV-6935-01A	HNSC	7.59
TCGA-CN-6017-01A	HNSC	6.29
TCGA-CV-7101-01A	HNSC	7.12
TCGA-BA-A4II-01A	HNSC	6.86
TCGA-CV-7252-01A	HNSC	7.4
TCGA-CN-4729-01A	HNSC	7.18
TCGA-D6-6827-01A	HNSC	6.59
TCGA-CV-5439-01A	HNSC	6.91
TCGA-BB-A5HY-01A	HNSC	7.17
TCGA-CV-A461-01A	HNSC	7.29
TCGA-P3-A5QF-01A	HNSC	7.69
TCGA-CN-5355-01A	HNSC	8.12
TCGA-D6-6825-01A	HNSC	7.28
TCGA-CV-6961-01A	HNSC	6.04
TCGA-CN-4742-01A	HNSC	6.79
TCGA-CN-A498-01A	HNSC	6.85
TCGA-CN-6996-01A	HNSC	7.74
TCGA-CV-A45Y-01A	HNSC	6.57
TCGA-QK-A652-01A	HNSC	6.53
TCGA-P3-A5Q6-01A	HNSC	7.03
TCGA-CV-7242-01A	HNSC	7.23
TCGA-F7-A624-01A	HNSC	6.57
TCGA-TN-A7HL-01A	HNSC	6.71
TCGA-CV-7102-01A	HNSC	7.42
TCGA-MT-A67F-01A	HNSC	6.59
TCGA-CN-A6V6-01A	HNSC	6.57
TCGA-CN-A63U-01A	HNSC	7.07
TCGA-CN-5363-01A	HNSC	8.06
TCGA-BB-8601-01A	HNSC	7.11
TCGA-D6-A6EP-01A	HNSC	6.45
TCGA-CV-6950-01A	HNSC	7.65
TCGA-UF-A71D-01A	HNSC	8.38
TCGA-DQ-7591-01A	HNSC	7.1
TCGA-CX-7219-01A	HNSC	7.54
TCGA-CR-7386-01A	HNSC	6.99
TCGA-P3-A5Q5-01A	HNSC	7.47
TCGA-CV-7422-01A	HNSC	7.27
TCGA-BB-7863-01A	HNSC	6.51
TCGA-CV-7091-01A	HNSC	7.16
TCGA-QK-A6II-01A	HNSC	5.56
TCGA-CN-4725-01A	HNSC	6.9
TCGA-QK-A6IG-01A	HNSC	7.59
TCGA-BA-5152-01A	HNSC	7.86
TCGA-CV-5442-01A	HNSC	6.81
TCGA-UF-A71A-01A	HNSC	7.36
TCGA-CR-7372-01A	HNSC	6.53
TCGA-D6-A6EO-01A	HNSC	8.69
TCGA-CV-A6JD-01A	HNSC	7.14
TCGA-CR-7392-01A	HNSC	6.76
TCGA-CR-6478-01A	HNSC	7.22
TCGA-WA-A7H4-01A	HNSC	6.89
TCGA-D6-A4Z9-01A	HNSC	7.74
TCGA-H7-A76A-01A	HNSC	6.32
TCGA-CR-7389-01A	HNSC	6.75

TCGA-CQ-5327-01A	HNSC	7.59
TCGA-HD-7229-01A	HNSC	7.72
TCGA-D6-8568-01A	HNSC	7.49
TCGA-CV-6954-01A	HNSC	7.53
TCGA-CV-7089-01A	HNSC	7.6
TCGA-CV-6003-01A	HNSC	7.05
TCGA-CN-5361-01A	HNSC	7.15
TCGA-CV-5977-01A	HNSC	7.35
TCGA-UF-A7JO-01A	HNSC	7.39
TCGA-CR-7388-01A	HNSC	6.84
TCGA-F7-8489-01A	HNSC	7.11
TCGA-CV-7248-01A	HNSC	7.5
TCGA-P3-A6T5-01A	HNSC	6.23
TCGA-CV-7429-01A	HNSC	7.15
TCGA-CV-6934-01A	HNSC	7.02
TCGA-BB-4223-01A	HNSC	7.47
TCGA-CX-7082-01A	HNSC	8.04
TCGA-RS-A6TP-01A	HNSC	7.27
TCGA-KU-A66S-01A	HNSC	6.64
TCGA-CV-7090-01A	HNSC	6.91
TCGA-CQ-7069-01A	HNSC	6.89
TCGA-CV-A6JY-01A	HNSC	6.4
TCGA-MZ-A7D7-01A	HNSC	7.96
TCGA-CR-6472-01A	HNSC	6.81
TCGA-CV-7410-01A	HNSC	6.66
TCGA-CV-7250-01A	HNSC	7.95
TCGA-CR-7393-01A	HNSC	6.86
TCGA-HD-8224-01A	HNSC	7.89
TCGA-CN-A6V1-01A	HNSC	6.01
TCGA-BB-A6UM-01A	HNSC	6.82
TCGA-CR-6482-01A	HNSC	6.89
TCGA-DQ-5625-01A	HNSC	6.98
TCGA-CV-7261-01A	HNSC	7.86
TCGA-UF-A7JC-01A	HNSC	6.88
TCGA-CV-7427-01A	HNSC	6.56
TCGA-CQ-7072-01A	HNSC	6.44
TCGA-BB-4228-01A	HNSC	7.46
TCGA-BA-6871-01A	HNSC	6.79
TCGA-BA-A6DD-01A	HNSC	6.71
TCGA-CV-7430-01A	HNSC	8.05
TCGA-BA-5149-01A	HNSC	8.19
TCGA-CV-A6JE-01A	HNSC	6.28
TCGA-BB-A6UO-01A	HNSC	6.61
TCGA-CV-A460-01A	HNSC	6.43
TCGA-BB-7871-01A	HNSC	7.6
TCGA-CR-7365-01A	HNSC	7.49
TCGA-QK-A8Z8-01A	HNSC	7.24
TCGA-CR-6492-01A	HNSC	6.84
TCGA-CR-7397-01A	HNSC	6.35
TCGA-UF-A719-01A	HNSC	5.94
TCGA-BA-6873-01A	HNSC	7.03
TCGA-CR-6477-01A	HNSC	6.32
TCGA-CV-7446-01A	HNSC	8.16
TCGA-T2-A6WX-01A	HNSC	7.16
TCGA-CV-7238-01A	HNSC	6.45
TCGA-QK-A8Z9-01B	HNSC	6.55
TCGA-DQ-7588-01A	HNSC	8.38
TCGA-CV-A45U-01A	HNSC	7.12

TCGA-CV-6940-01A	HNSC	6.78
TCGA-CN-4734-01A	HNSC	6.79
TCGA-IQ-A6SG-01A	HNSC	6.06
TCGA-CN-5374-01A	HNSC	6.74
TCGA-CN-A63V-01A	HNSC	6.38
TCGA-CV-7095-01A	HNSC	7.08
TCGA-IQ-A61O-01A	HNSC	6.84
TCGA-P3-A6T4-01A	HNSC	6.28
TCGA-DQ-7592-01A	HNSC	7.1
TCGA-CR-5249-01A	HNSC	6.53
TCGA-CQ-6224-01A	HNSC	6.95
TCGA-CV-5978-01A	HNSC	7.24
TCGA-CV-7183-01A	HNSC	6.35
TCGA-BA-4078-01A	HNSC	7.03
TCGA-QK-A6IJ-01A	HNSC	5.98
TCGA-CV-5432-01A	HNSC	6.75
TCGA-CR-7398-01A	HNSC	7.58
TCGA-CV-5973-01A	HNSC	5.34
TCGA-CV-7407-01A	HNSC	7.29
TCGA-CV-A45R-01A	HNSC	6.98
TCGA-KU-A6H8-01A	HNSC	6.52
TCGA-CV-A6JO-01B	HNSC	6.33
TCGA-BA-A6DI-01A	HNSC	7.08
TCGA-BB-7870-01A	HNSC	7.55
TCGA-CV-5434-01A	HNSC	7.08
TCGA-D6-A4ZB-01A	HNSC	6.4
TCGA-CR-6470-01A	HNSC	6.07
TCGA-F7-A620-01A	HNSC	7.55
TCGA-KU-A6H7-01A	HNSC	6.3
TCGA-CV-7413-01A	HNSC	6.85
TCGA-MT-A7BN-01A	HNSC	5.62
TCGA-QK-A6IH-01A	HNSC	5.82
TCGA-CV-A6JM-01A	HNSC	5.91
TCGA-DQ-7596-01A	HNSC	6.61
TCGA-CN-5364-01A	HNSC	7.21
TCGA-KL-8325-01A	KICH	7.96
TCGA-KO-8403-01A	KICH	6.43
TCGA-KL-8341-01A	KICH	7.63
TCGA-KL-8336-01A	KICH	7.87
TCGA-KN-8421-01A	KICH	7.13
TCGA-KN-8422-01A	KICH	6.93
TCGA-KL-8345-01A	KICH	6.77
TCGA-KN-8428-01A	KICH	5.53
TCGA-KM-8476-01A	KICH	5.89
TCGA-KL-8337-01A	KICH	6.61
TCGA-KN-8423-01A	KICH	8.33
TCGA-KL-8340-01A	KICH	7.07
TCGA-KN-8429-01A	KICH	7.34
TCGA-KM-8442-01A	KICH	7.31
TCGA-KL-8328-01A	KICH	6.71
TCGA-KM-8439-01A	KICH	6.92
TCGA-KL-8333-01A	KICH	7.66
TCGA-KO-8407-01A	KICH	6.67
TCGA-KO-8417-01A	KICH	7.85
TCGA-KO-8404-01A	KICH	6.23
TCGA-KL-8330-01A	KICH	7.41
TCGA-KL-8327-01A	KICH	5.41
TCGA-KO-8416-01A	KICH	6.42

TCGA-KL-8326-01A	KICH	7.19
TCGA-KL-8324-01A	KICH	7.57
TCGA-KM-8438-01A	KICH	6.66
TCGA-KO-8413-01A	KICH	7.41
TCGA-KM-8443-01A	KICH	6.99
TCGA-KL-8338-01A	KICH	6.7
TCGA-KL-8332-01A	KICH	7.09
TCGA-KN-8431-01A	KICH	7.53
TCGA-KM-8639-01A	KICH	7.91
TCGA-KN-8424-01A	KICH	7.12
TCGA-KL-8331-01A	KICH	6.73
TCGA-KN-8427-01A	KICH	8.22
TCGA-KN-8430-01A	KICH	7.4
TCGA-KO-8408-01A	KICH	6.65
TCGA-KN-8433-01A	KICH	6.17
TCGA-KL-8346-01A	KICH	6.42
TCGA-KL-8344-01A	KICH	8.99
TCGA-KO-8414-01A	KICH	7.23
TCGA-KN-8418-01A	KICH	7.34
TCGA-KL-8323-01A	KICH	7.18
TCGA-KM-8440-01A	KICH	6.94
TCGA-KO-8406-01A	KICH	7.73
TCGA-KM-8477-01A	KICH	8.26
TCGA-KO-8410-01A	KICH	8.36
TCGA-KL-8342-01A	KICH	7.04
TCGA-KN-8434-01A	KICH	6.79
TCGA-KO-8411-01A	KICH	7.25
TCGA-KN-8437-01A	KICH	6.77
TCGA-KO-8415-01A	KICH	6.91
TCGA-KL-8343-01A	KICH	7.64
TCGA-KL-8335-01A	KICH	7.27
TCGA-KL-8339-01A	KICH	8.38
TCGA-KL-8334-01A	KICH	7.01
TCGA-KN-8432-01A	KICH	8
TCGA-KN-8426-01A	KICH	7.27
TCGA-KN-8425-01A	KICH	7.54
TCGA-KN-8419-01A	KICH	7.57
TCGA-KO-8405-01A	KICH	6.59
TCGA-KL-8329-01A	KICH	7.36
TCGA-KN-8436-01A	KICH	7.27
TCGA-KN-8435-01A	KICH	7.26
TCGA-KM-8441-01A	KICH	7.52
TCGA-KO-8409-01A	KICH	5.89
TCGA-BP-4756-01A	KIRC	7.72
TCGA-BP-4999-01A	KIRC	7.86
TCGA-CJ-5684-01A	KIRC	8.03
TCGA-B0-5692-01A	KIRC	8.28
TCGA-BP-4988-01A	KIRC	7.03
TCGA-BP-5010-01A	KIRC	5.84
TCGA-CZ-5451-01A	KIRC	8.28
TCGA-CJ-4899-01A	KIRC	7.65
TCGA-CW-5590-01A	KIRC	7.59
TCGA-B8-5545-01A	KIRC	8.05
TCGA-CJ-4902-01A	KIRC	7.15
TCGA-B0-5102-01A	KIRC	8.16
TCGA-BP-4801-01A	KIRC	8.23
TCGA-CZ-5460-01A	KIRC	6.74
TCGA-BP-4347-01A	KIRC	8.79

TCGA-CJ-4635-01A	KIRC	6.34
TCGA-A3-A6NJ-01A	KIRC	7.98
TCGA-BP-4962-01A	KIRC	7.24
TCGA-B4-5834-01A	KIRC	7.68
TCGA-BP-4963-01A	KIRC	7.06
TCGA-B0-5116-01A	KIRC	7.75
TCGA-B2-4101-01A	KIRC	7.28
TCGA-B0-5707-01A	KIRC	7.98
TCGA-B0-5108-01A	KIRC	7.32
TCGA-A3-3313-01A	KIRC	6.77
TCGA-B0-4843-01A	KIRC	6.09
TCGA-CW-5585-01A	KIRC	7.69
TCGA-B8-A8YJ-01A	KIRC	7.51
TCGA-B8-5164-01A	KIRC	9.13
TCGA-CJ-4637-01A	KIRC	8
TCGA-B0-4841-01A	KIRC	5.42
TCGA-CJ-4870-01A	KIRC	7.7
TCGA-B0-5400-01A	KIRC	6.94
TCGA-BP-4784-01A	KIRC	7.31
TCGA-A3-3363-01A	KIRC	8.38
TCGA-B8-4621-01A	KIRC	7.47
TCGA-B0-5690-01A	KIRC	7.63
TCGA-B0-5702-01A	KIRC	7.05
TCGA-B0-5083-01A	KIRC	7.01
TCGA-B0-4688-01A	KIRC	6.55
TCGA-BP-4781-01A	KIRC	7.71
TCGA-B4-5832-01A	KIRC	9.14
TCGA-BP-4766-01A	KIRC	7.07
TCGA-B0-5121-01A	KIRC	6.66
TCGA-CJ-4872-01A	KIRC	7.71
TCGA-B2-5633-01B	KIRC	7.24
TCGA-A3-3317-01A	KIRC	7.14
TCGA-CZ-4853-01A	KIRC	7.51
TCGA-BP-4991-01A	KIRC	7.94
TCGA-B0-4691-01A	KIRC	7.65
TCGA-B0-5713-01A	KIRC	8.25
TCGA-CJ-6032-01A	KIRC	7.28
TCGA-B8-4146-01B	KIRC	9.72
TCGA-AK-3445-01A	KIRC	8.22
TCGA-A3-3335-01A	KIRC	7.63
TCGA-BP-4342-01A	KIRC	7.18
TCGA-B8-5163-01A	KIRC	8.17
TCGA-B0-5092-01A	KIRC	7.46
TCGA-B0-4703-01A	KIRC	7.65
TCGA-B8-5162-01A	KIRC	7.05
TCGA-CJ-5677-01A	KIRC	7.78
TCGA-BP-4797-01A	KIRC	7.59
TCGA-B0-4693-01A	KIRC	8.06
TCGA-BP-4985-01A	KIRC	7.04
TCGA-6D-AA2E-01A	KIRC	6.98
TCGA-BP-4770-01A	KIRC	7.11
TCGA-B0-5693-01A	KIRC	8.42
TCGA-BP-4332-01A	KIRC	7.55
TCGA-A3-3382-01A	KIRC	8.14
TCGA-BP-4334-01A	KIRC	6.46
TCGA-BP-4971-01A	KIRC	6.45
TCGA-AK-3428-01A	KIRC	7.75
TCGA-A3-3387-01A	KIRC	7.84

TCGA-BP-4327-01A	KIRC	7.21
TCGA-A3-3306-01A	KIRC	8.37
TCGA-B0-5100-01A	KIRC	7.42
TCGA-BP-4995-01A	KIRC	7.44
TCGA-CJ-4642-01B	KIRC	6.27
TCGA-B2-5641-01A	KIRC	7.53
TCGA-B0-5117-01A	KIRC	6.35
TCGA-CJ-4912-01A	KIRC	6.69
TCGA-CZ-5982-01A	KIRC	7.52
TCGA-MW-A4EC-01A	KIRC	7.84
TCGA-A3-3325-01A	KIRC	7.27
TCGA-CJ-4886-01A	KIRC	7.95
TCGA-B2-4102-01A	KIRC	7.99
TCGA-B0-4699-01A	KIRC	7.36
TCGA-B0-5120-01A	KIRC	8.01
TCGA-B0-4824-01A	KIRC	7.7
TCGA-B0-5077-01A	KIRC	7.42
TCGA-CJ-6033-01A	KIRC	8.06
TCGA-A3-3357-01A	KIRC	8.01
TCGA-A3-3320-01A	KIRC	8.05
TCGA-CJ-4916-01A	KIRC	7.34
TCGA-BP-4346-01A	KIRC	7.51
TCGA-B0-5712-01A	KIRC	9.32
TCGA-CW-5589-01A	KIRC	7.78
TCGA-EU-5906-01A	KIRC	8.56
TCGA-MM-A564-01A	KIRC	6.85
TCGA-BP-4983-01A	KIRC	6.98
TCGA-CZ-4860-01A	KIRC	7.22
TCGA-BP-4765-01A	KIRC	8.13
TCGA-CZ-5455-01A	KIRC	8.32
TCGA-B0-4690-01A	KIRC	7.17
TCGA-CZ-5988-01A	KIRC	8.08
TCGA-B8-A7U6-01A	KIRC	7.92
TCGA-CJ-6031-01A	KIRC	7.14
TCGA-BP-4355-01A	KIRC	7
TCGA-B0-5110-01A	KIRC	8.26
TCGA-DV-5574-01A	KIRC	7.61
TCGA-BP-4176-01A	KIRC	7.65
TCGA-B0-5695-01A	KIRC	8.73
TCGA-DV-5569-01A	KIRC	8.29
TCGA-CZ-5454-01A	KIRC	7.11
TCGA-BP-5183-01A	KIRC	7.33
TCGA-BP-5168-01A	KIRC	8.32
TCGA-B8-4619-01A	KIRC	8.81
TCGA-A3-3378-01A	KIRC	7.58
TCGA-BP-5178-01A	KIRC	6.59
TCGA-B8-5546-01A	KIRC	9.72
TCGA-CZ-5469-01A	KIRC	7.07
TCGA-CJ-4892-01A	KIRC	8.03
TCGA-CJ-4875-01A	KIRC	7.8
TCGA-BP-4326-01A	KIRC	7.75
TCGA-CJ-4920-01A	KIRC	7.79
TCGA-CZ-4863-01A	KIRC	9.44
TCGA-CJ-6030-01A	KIRC	8.18
TCGA-CJ-4634-01A	KIRC	8.08
TCGA-CZ-5459-01A	KIRC	7.91
TCGA-BP-4341-01A	KIRC	7.23
TCGA-CZ-5468-01A	KIRC	7.37

TCGA-BP-5176-01A	KIRC	8.08
TCGA-CZ-5986-01A	KIRC	8.21
TCGA-A3-3346-01A	KIRC	7.29
TCGA-B0-5402-01A	KIRC	6.65
TCGA-CJ-4871-01A	KIRC	7.54
TCGA-BP-4989-01A	KIRC	6.58
TCGA-AK-3447-01A	KIRC	7.93
TCGA-A3-3359-01A	KIRC	7.9
TCGA-B0-5084-01A	KIRC	6.88
TCGA-CJ-5675-01A	KIRC	7.69
TCGA-B0-4845-01A	KIRC	7.32
TCGA-B0-4945-01A	KIRC	8.08
TCGA-A3-3351-01A	KIRC	8.12
TCGA-B8-A54G-01A	KIRC	7.33
TCGA-BP-4961-01A	KIRC	7.43
TCGA-T7-A92I-01A	KIRC	7.74
TCGA-BP-4974-01A	KIRC	7.19
TCGA-BP-5001-01A	KIRC	7.25
TCGA-BP-5199-01A	KIRC	7.72
TCGA-B0-4846-01A	KIRC	8.56
TCGA-B0-5697-01A	KIRC	8.1
TCGA-BP-5185-01A	KIRC	5.95
TCGA-A3-A8OX-01A	KIRC	7.58
TCGA-CZ-5462-01A	KIRC	8.19
TCGA-A3-3323-01A	KIRC	7.62
TCGA-CJ-5689-01A	KIRC	7.28
TCGA-BP-4965-01A	KIRC	8.65
TCGA-BP-5173-01A	KIRC	8.36
TCGA-CJ-4641-01A	KIRC	8.14
TCGA-BP-4981-01A	KIRC	7.24
TCGA-CZ-5457-01A	KIRC	8.27
TCGA-CJ-4643-01A	KIRC	8.28
TCGA-B0-5096-01A	KIRC	7.19
TCGA-CJ-4901-01A	KIRC	6.57
TCGA-DV-5573-01A	KIRC	8.05
TCGA-A3-3362-01A	KIRC	7.92
TCGA-B0-5699-01A	KIRC	7.96
TCGA-B0-5709-01A	KIRC	7.93
TCGA-BP-4970-01A	KIRC	8.49
TCGA-AK-3443-01A	KIRC	7.87
TCGA-A3-3343-01A	KIRC	7.55
TCGA-BP-4964-01A	KIRC	7.9
TCGA-CZ-4857-01A	KIRC	7.61
TCGA-CJ-4894-01A	KIRC	8.84
TCGA-CJ-4891-01A	KIRC	6.18
TCGA-CZ-4859-01A	KIRC	8.99
TCGA-BP-4769-01A	KIRC	7.39
TCGA-AK-3458-01A	KIRC	7.69
TCGA-CJ-4893-01A	KIRC	7.89
TCGA-BP-4354-01A	KIRC	7.85
TCGA-B0-5698-01A	KIRC	7.64
TCGA-B8-4143-01A	KIRC	8.4
TCGA-CJ-4876-01A	KIRC	8.32
TCGA-BP-4161-01A	KIRC	7.29
TCGA-CZ-5458-01A	KIRC	7.45
TCGA-BP-4763-01A	KIRC	8.4
TCGA-CZ-5984-01A	KIRC	7.77
TCGA-B0-4834-01A	KIRC	8.03

TCGA-B8-A54K-01A	KIRC	7.4
TCGA-CJ-4895-01A	KIRC	7.02
TCGA-BP-5191-01A	KIRC	6.05
TCGA-AK-3427-01A	KIRC	9.34
TCGA-BP-5008-01A	KIRC	7.87
TCGA-B8-5549-01A	KIRC	7.92
TCGA-BP-4977-01A	KIRC	7.55
TCGA-AK-3426-01A	KIRC	6.41
TCGA-B4-5377-01A	KIRC	8.34
TCGA-BP-5195-01A	KIRC	7.83
TCGA-BP-4775-01A	KIRC	7.77
TCGA-B8-5165-01A	KIRC	8.09
TCGA-A3-3358-01A	KIRC	7.94
TCGA-B8-A54D-01A	KIRC	7.15
TCGA-B8-4153-01B	KIRC	7.61
TCGA-B8-5159-01A	KIRC	8.52
TCGA-DV-5567-01A	KIRC	7.75
TCGA-A3-A8OW-01A	KIRC	7.26
TCGA-B0-5109-01A	KIRC	7.34
TCGA-A3-A8CQ-01A	KIRC	7.82
TCGA-B0-4822-01A	KIRC	6.67
TCGA-CZ-4862-01A	KIRC	8.37
TCGA-B8-4622-01A	KIRC	7.49
TCGA-BP-4164-01A	KIRC	7.73
TCGA-BP-4776-01A	KIRC	6.69
TCGA-BP-5006-01A	KIRC	7.64
TCGA-B0-5710-01A	KIRC	7.84
TCGA-BP-5180-01A	KIRC	6.51
TCGA-CJ-4897-01A	KIRC	8.18
TCGA-BP-4986-01A	KIRC	7.61
TCGA-BP-4973-01A	KIRC	7.12
TCGA-B0-4823-01A	KIRC	8.09
TCGA-BP-4968-01A	KIRC	7.52
TCGA-CZ-4861-01A	KIRC	7.32
TCGA-AK-3444-01A	KIRC	9
TCGA-A3-3352-01A	KIRC	7.71
TCGA-CJ-4868-01A	KIRC	7.43
TCGA-EU-5907-01A	KIRC	7.01
TCGA-A3-3326-01A	KIRC	7.52
TCGA-A3-3373-01A	KIRC	9.06
TCGA-B0-4842-01A	KIRC	6.61
TCGA-CW-5581-01A	KIRC	8.27
TCGA-BP-4337-01A	KIRC	7.19
TCGA-CZ-4854-01A	KIRC	6.85
TCGA-B0-5097-01A	KIRC	8.08
TCGA-BP-4338-01A	KIRC	7.77
TCGA-A3-3324-01A	KIRC	8.37
TCGA-BP-5009-01A	KIRC	7.01
TCGA-CJ-5682-01A	KIRC	7.98
TCGA-A3-A6NL-01A	KIRC	7.57
TCGA-B8-5550-01A	KIRC	7.28
TCGA-B2-5635-01A	KIRC	8.39
TCGA-CJ-4885-01A	KIRC	8.73
TCGA-B0-4811-01A	KIRC	6.26
TCGA-CZ-5453-01A	KIRC	8.21
TCGA-B4-5836-01A	KIRC	9.1
TCGA-B8-A54F-01A	KIRC	7.86
TCGA-B0-5085-01A	KIRC	6.13

TCGA-B2-3924-01A	KIRC	6.8
TCGA-B4-5378-01A	KIRC	7.47
TCGA-BP-4787-01A	KIRC	7.19
TCGA-AK-3456-01A	KIRC	8.44
TCGA-B0-4707-01A	KIRC	7.4
TCGA-BP-5181-01A	KIRC	8.4
TCGA-CW-6090-01A	KIRC	7.41
TCGA-B8-A54E-01A	KIRC	7.31
TCGA-B0-5703-01A	KIRC	6.58
TCGA-CJ-4873-01A	KIRC	6.44
TCGA-CW-5587-01A	KIRC	8.78
TCGA-BP-5194-01A	KIRC	7.66
TCGA-BP-4993-01A	KIRC	7.63
TCGA-B0-5705-01A	KIRC	8.23
TCGA-A3-3370-01A	KIRC	8.37
TCGA-BP-5198-01A	KIRC	7.27
TCGA-CJ-4644-01A	KIRC	8.56
TCGA-DV-5576-01A	KIRC	7.53
TCGA-AK-3436-01A	KIRC	7.27
TCGA-BP-5202-01A	KIRC	7.84
TCGA-AK-3429-01A	KIRC	8.82
TCGA-BP-4351-01A	KIRC	7.33
TCGA-B0-4817-01A	KIRC	6.55
TCGA-BP-4987-01A	KIRC	7.78
TCGA-BP-4774-01A	KIRC	7.82
TCGA-MM-A84U-01A	KIRC	7.72
TCGA-BP-4325-01A	KIRC	7.85
TCGA-A3-3331-01A	KIRC	7.25
TCGA-B4-5843-01A	KIRC	8.45
TCGA-B4-5844-01A	KIRC	8.65
TCGA-CW-6087-01A	KIRC	7.45
TCGA-BP-4177-01A	KIRC	7.67
TCGA-B0-4714-01A	KIRC	6.77
TCGA-B0-4814-01A	KIRC	7.99
TCGA-A3-3385-01A	KIRC	7.49
TCGA-CJ-4890-01A	KIRC	6.91
TCGA-CZ-5985-01A	KIRC	7.97
TCGA-B0-5081-01A	KIRC	7.01
TCGA-CJ-4882-01A	KIRC	6.59
TCGA-B2-4098-01A	KIRC	6.84
TCGA-B0-4819-01A	KIRC	6.3
TCGA-DV-5566-01A	KIRC	7.77
TCGA-BP-4760-01A	KIRC	7.6
TCGA-B2-5635-01B	KIRC	7.99
TCGA-BP-4173-01A	KIRC	7.56
TCGA-BP-5192-01A	KIRC	8.1
TCGA-AK-3460-01A	KIRC	7.52
TCGA-CJ-4903-01A	KIRC	7.56
TCGA-B0-4818-01A	KIRC	7.84
TCGA-CW-6097-01A	KIRC	8.15
TCGA-BP-4343-01A	KIRC	7.52
TCGA-CZ-4864-01A	KIRC	7.93
TCGA-B0-4852-01A	KIRC	8.32
TCGA-CJ-4874-01A	KIRC	8.22
TCGA-CJ-5678-01A	KIRC	7.25
TCGA-B0-4701-01A	KIRC	8.24
TCGA-B2-5633-01A	KIRC	8.17
TCGA-CW-5580-01A	KIRC	8.34

TCGA-BP-4994-01A	KIRC	7.38
TCGA-BP-4174-01A	KIRC	8.69
TCGA-BP-4782-01A	KIRC	8.85
TCGA-CZ-5463-01A	KIRC	9.02
TCGA-CJ-5671-01A	KIRC	7.18
TCGA-B0-4844-01A	KIRC	7.02
TCGA-CJ-4884-01A	KIRC	7.89
TCGA-BP-4959-01A	KIRC	7.99
TCGA-CZ-5466-01A	KIRC	7.34
TCGA-B0-5700-01A	KIRC	8.13
TCGA-BP-4344-01A	KIRC	7.69
TCGA-B0-4698-01A	KIRC	7.67
TCGA-B0-4836-01A	KIRC	6.73
TCGA-BP-4998-01A	KIRC	8.01
TCGA-BP-4329-01A	KIRC	6.93
TCGA-CJ-5680-01A	KIRC	7.52
TCGA-BP-4777-01A	KIRC	7.18
TCGA-CW-5583-01A	KIRC	8.12
TCGA-B4-5838-01A	KIRC	8.32
TCGA-B0-5711-01A	KIRC	8.01
TCGA-CJ-4878-01A	KIRC	8.04
TCGA-B8-5553-01A	KIRC	8.87
TCGA-B0-5094-01A	KIRC	8.05
TCGA-B8-A54I-01A	KIRC	6.13
TCGA-B0-4813-01A	KIRC	6.91
TCGA-CJ-4905-01A	KIRC	7.66
TCGA-B0-4821-01A	KIRC	6.58
TCGA-A3-3380-01A	KIRC	7.18
TCGA-BP-4795-01A	KIRC	7.54
TCGA-CZ-5461-01A	KIRC	8.22
TCGA-CJ-4907-01A	KIRC	7.37
TCGA-BP-4331-01A	KIRC	7.89
TCGA-BP-5000-01A	KIRC	7.28
TCGA-BP-4969-01A	KIRC	6.73
TCGA-EU-5904-01A	KIRC	7.83
TCGA-A3-3308-01A	KIRC	8.02
TCGA-DV-A4W0-01A	KIRC	6.93
TCGA-CZ-5456-01A	KIRC	7.27
TCGA-B0-4816-01A	KIRC	7.61
TCGA-BP-5004-01A	KIRC	8.79
TCGA-B0-4810-01A	KIRC	7.6
TCGA-BP-4758-01A	KIRC	6.97
TCGA-BP-4352-01A	KIRC	6.72
TCGA-B2-3923-01A	KIRC	7.6
TCGA-CJ-5686-01A	KIRC	8.05
TCGA-GK-A6C7-01A	KIRC	7.89
TCGA-AK-3434-01A	KIRC	7.48
TCGA-B8-4148-01A	KIRC	7.77
TCGA-BP-4976-01A	KIRC	8.51
TCGA-BP-5189-01A	KIRC	8.05
TCGA-BP-4972-01A	KIRC	7.52
TCGA-BP-5175-01A	KIRC	6.53
TCGA-BP-4967-01A	KIRC	7.14
TCGA-3Z-A93Z-01A	KIRC	8.17
TCGA-AK-3454-01A	KIRC	6.57
TCGA-BP-4761-01A	KIRC	8.08
TCGA-CZ-4866-01A	KIRC	8.39
TCGA-B0-5706-01A	KIRC	6.31

TCGA-BP-4166-01A	KIRC	6.84
TCGA-BP-4771-01A	KIRC	6.81
TCGA-A3-3376-01A	KIRC	7.84
TCGA-B0-4710-01A	KIRC	8.12
TCGA-B0-5113-01A	KIRC	7.7
TCGA-CJ-5679-01A	KIRC	6.85
TCGA-BP-5196-01A	KIRC	8.19
TCGA-BP-4759-01A	KIRC	6.88
TCGA-BP-4158-01A	KIRC	7.55
TCGA-CZ-5989-01A	KIRC	8.84
TCGA-B0-4828-01A	KIRC	7.04
TCGA-A3-3329-01A	KIRC	8.17
TCGA-DV-5568-01A	KIRC	7.39
TCGA-A3-3319-01A	KIRC	7.78
TCGA-AK-3455-01A	KIRC	9.08
TCGA-AK-3425-01A	KIRC	7.93
TCGA-BP-5186-01A	KIRC	8.4
TCGA-B0-5696-01A	KIRC	8.32
TCGA-G6-A8L7-01A	KIRC	6.75
TCGA-G6-A8L6-01A	KIRC	7.18
TCGA-BP-5169-01A	KIRC	5.28
TCGA-B0-5691-01A	KIRC	8.39
TCGA-BP-4803-01A	KIRC	7.32
TCGA-A3-3374-01A	KIRC	6.42
TCGA-AK-3450-01A	KIRC	9.01
TCGA-A3-3372-01A	KIRC	7.6
TCGA-BP-4960-01A	KIRC	5.79
TCGA-BP-4165-01A	KIRC	7.22
TCGA-CJ-5676-01A	KIRC	6.64
TCGA-BP-4762-01A	KIRC	7.93
TCGA-B0-4712-01A	KIRC	7.26
TCGA-CJ-4900-01A	KIRC	6.82
TCGA-B0-5115-01A	KIRC	7.4
TCGA-CJ-6027-01A	KIRC	8.22
TCGA-B0-4839-01A	KIRC	7.72
TCGA-B0-4718-01A	KIRC	6.48
TCGA-B0-5095-01A	KIRC	8.04
TCGA-A3-3307-01A	KIRC	8.19
TCGA-CJ-4640-01A	KIRC	7.98
TCGA-BP-4170-01A	KIRC	7.68
TCGA-CW-5588-01A	KIRC	7.79
TCGA-BP-4160-01A	KIRC	8.01
TCGA-G6-A8L8-01A	KIRC	7.28
TCGA-AS-3778-01A	KIRC	8.2
TCGA-B0-5107-01A	KIRC	8.5
TCGA-CZ-5464-01A	KIRC	7.03
TCGA-BP-5177-01A	KIRC	7.35
TCGA-BP-4345-01A	KIRC	6.75
TCGA-B0-5701-01A	KIRC	7.84
TCGA-B0-4849-01A	KIRC	7.58
TCGA-CJ-4908-01A	KIRC	7.33
TCGA-B0-5088-01A	KIRC	7.19
TCGA-B2-3923-01B	KIRC	7.5
TCGA-CJ-4869-01A	KIRC	7.62
TCGA-AK-3461-01A	KIRC	7.26
TCGA-B0-4848-01A	KIRC	7.87
TCGA-B8-A54J-01A	KIRC	7.74
TCGA-AK-3465-01A	KIRC	6.81

TCGA-A3-3316-01A	KIRC	6.35
TCGA-DV-5575-01A	KIRC	8.31
TCGA-B0-4827-01A	KIRC	7.43
TCGA-CJ-4918-01A	KIRC	7.53
TCGA-B0-4706-01A	KIRC	6.95
TCGA-A3-3322-01A	KIRC	7.53
TCGA-A3-A6NI-01A	KIRC	7.79
TCGA-A3-A6NN-01A	KIRC	7.42
TCGA-CJ-4638-01A	KIRC	6.62
TCGA-A3-3349-01A	KIRC	8.45
TCGA-CW-5584-01A	KIRC	8.62
TCGA-A3-3311-01A	KIRC	8.92
TCGA-B0-5694-01A	KIRC	6.6
TCGA-B8-5551-01A	KIRC	7.13
TCGA-B0-4694-01A	KIRC	7.18
TCGA-B0-4696-01A	KIRC	7
TCGA-A3-3365-01A	KIRC	8.59
TCGA-B4-5835-01A	KIRC	7.22
TCGA-EU-5905-01A	KIRC	8.44
TCGA-BP-5201-01A	KIRC	7.26
TCGA-B0-5075-01A	KIRC	8.1
TCGA-B2-5639-01A	KIRC	8.02
TCGA-CZ-5465-01A	KIRC	7.65
TCGA-CJ-5683-01A	KIRC	7.34
TCGA-A3-3367-01A	KIRC	8.01
TCGA-CJ-4881-01A	KIRC	7.65
TCGA-CJ-4923-01A	KIRC	6.66
TCGA-A3-A8OV-01A	KIRC	7.54
TCGA-B0-4697-01A	KIRC	5.55
TCGA-CJ-6028-01A	KIRC	7.18
TCGA-B2-4099-01A	KIRC	7.85
TCGA-CJ-5681-01A	KIRC	7.41
TCGA-BP-5170-01A	KIRC	7.63
TCGA-B0-5812-01A	KIRC	7.79
TCGA-CJ-4636-01A	KIRC	7.3
TCGA-B0-5098-01A	KIRC	6.72
TCGA-B8-A54H-01A	KIRC	8.23
TCGA-BP-4807-01A	KIRC	8.95
TCGA-CW-6093-01A	KIRC	7.47
TCGA-CZ-4865-01A	KIRC	8.07
TCGA-B0-4837-01A	KIRC	6.4
TCGA-BP-4169-01A	KIRC	7.63
TCGA-A3-3347-01A	KIRC	7.36
TCGA-B2-3924-01B	KIRC	6.93
TCGA-DV-A4W0-05A	KIRC	7.26
TCGA-AK-3431-01A	KIRC	8.23
TCGA-BP-4162-01A	KIRC	7.69
TCGA-BP-5174-01A	KIRC	8.32
TCGA-CW-6088-01A	KIRC	8.59
TCGA-CJ-4639-01A	KIRC	8.49
TCGA-BP-5184-01A	KIRC	7.84
TCGA-AK-3440-01A	KIRC	6.99
TCGA-BP-5182-01A	KIRC	8.1
TCGA-G6-A5PC-01A	KIRC	7.85
TCGA-BP-4340-01A	KIRC	9.17
TCGA-A3-3383-01A	KIRC	7.8
TCGA-BP-4975-01A	KIRC	7.68
TCGA-B0-5106-01A	KIRC	7.6

TCGA-BP-5007-01A	KIRC	7.53
TCGA-B2-5636-01A	KIRC	7.42
TCGA-CZ-4858-01A	KIRC	6.98
TCGA-CZ-5452-01A	KIRC	7.38
TCGA-BP-4167-01A	KIRC	6.49
TCGA-BP-4353-01A	KIRC	7.75
TCGA-AK-3451-01A	KIRC	8.12
TCGA-DV-5565-01A	KIRC	6.75
TCGA-BP-4982-01A	KIRC	7.86
TCGA-AK-3433-01A	KIRC	7.27
TCGA-BP-5190-01A	KIRC	6.92
TCGA-BP-4330-01A	KIRC	7.77
TCGA-MM-A563-01A	KIRC	7.28
TCGA-CW-5591-01A	KIRC	7.86
TCGA-B0-4833-01A	KIRC	7.4
TCGA-B0-5119-01A	KIRC	8.33
TCGA-DV-A4VZ-01A	KIRC	6.86
TCGA-BP-5187-01A	KIRC	7.96
TCGA-B0-4815-01A	KIRC	6.62
TCGA-CJ-4888-01A	KIRC	7.19
TCGA-CJ-5672-01A	KIRC	8.15
TCGA-DV-A4VX-01A	KIRC	7.14
TCGA-A3-A8OU-01A	KIRC	8.56
TCGA-B0-4838-01A	KIRC	8.28
TCGA-BP-4163-01A	KIRC	7.62
TCGA-B0-5399-01A	KIRC	7.59
TCGA-BP-4349-01A	KIRC	7.24
TCGA-BP-4159-01A	KIRC	6.77
TCGA-CZ-4856-01A	KIRC	8.47
TCGA-B0-5080-01A	KIRC	7.79
TCGA-BP-5200-01A	KIRC	7.91
TCGA-B2-A4SR-01A	KIRC	7.61
TCGA-CZ-5987-01A	KIRC	7.69
TCGA-CJ-4889-01A	KIRC	8.07
TCGA-AK-3453-01A	KIRC	7.05
TCGA-CJ-4904-01A	KIRC	8.48
TCGA-B0-5104-01A	KIRC	8.05
TCGA-BP-4790-01A	KIRC	7.87
TCGA-B8-5552-01B	KIRC	7.8
TCGA-BP-4335-01A	KIRC	6.91
TCGA-BP-4799-01A	KIRC	6.35
TCGA-B8-5158-01A	KIRC	7.19
TCGA-B0-4700-01A	KIRC	6.75
TCGA-B8-4620-01A	KIRC	7.02
TCGA-AS-3777-01A	KIRC	6.87
TCGA-BP-4804-01A	KIRC	7.18
TCGA-B8-4151-01A	KIRC	8.47
TCGA-BP-4992-01A	KIRC	5.51
TCGA-BP-4789-01A	KIRC	7.54
TCGA-BP-4798-01A	KIRC	7.07
TCGA-CJ-4887-01A	KIRC	7
TCGA-CZ-5467-01A	KIRC	7.87
TCGA-B0-5099-01A	KIRC	8.25
TCGA-B0-4847-01A	KIRC	7.05
TCGA-B8-4154-01A	KIRC	8.26
TCGA-A3-3328-01A	KIRC	8.52
TCGA-B0-4713-01A	KIRC	7.98
TCGA-BP-4768-01A	KIRC	7.75

TCGA-CZ-5470-01A	KIRC	7.29
TCGA-DW-7834-01A	KIRP	8.54
TCGA-2Z-A9J8-01A	KIRP	6.44
TCGA-IA-A83T-01A	KIRP	6.63
TCGA-B9-4114-01A	KIRP	9.05
TCGA-UZ-A9PL-01A	KIRP	7.74
TCGA-2Z-A9JG-01A	KIRP	8.14
TCGA-MH-A561-01A	KIRP	8.3
TCGA-HE-A5NL-01A	KIRP	6.8
TCGA-G7-6790-01A	KIRP	8.79
TCGA-A4-A772-01A	KIRP	8.21
TCGA-KV-A6GE-01A	KIRP	8.49
TCGA-EV-5901-01A	KIRP	7.87
TCGA-EV-5902-01A	KIRP	8.15
TCGA-DW-7841-01A	KIRP	7.64
TCGA-MH-A55Z-01A	KIRP	8.19
TCGA-A4-8517-01A	KIRP	7.2
TCGA-A4-7996-01A	KIRP	9.52
TCGA-B9-A5W7-01A	KIRP	9.54
TCGA-HE-A5NK-01A	KIRP	7.29
TCGA-B9-4117-01A	KIRP	8.39
TCGA-IA-A83S-01A	KIRP	7.1
TCGA-5P-A9K2-01A	KIRP	7.58
TCGA-HE-A5NJ-01A	KIRP	8.08
TCGA-P4-A5E8-01A	KIRP	7.62
TCGA-SX-A71V-01A	KIRP	8.99
TCGA-A4-8310-01A	KIRP	8.55
TCGA-A4-A4ZT-01A	KIRP	9.08
TCGA-DW-5561-01A	KIRP	7.94
TCGA-A4-A7UZ-01A	KIRP	6.7
TCGA-5P-A9JZ-01A	KIRP	7.54
TCGA-AL-3473-01A	KIRP	8.52
TCGA-UN-AAZ9-01A	KIRP	9.35
TCGA-B9-A8YI-01A	KIRP	6.53
TCGA-UZ-A9PZ-01A	KIRP	8.31
TCGA-SX-A71R-01A	KIRP	8.36
TCGA-J7-8537-01A	KIRP	7.93
TCGA-Y8-A8S0-01A	KIRP	9.13
TCGA-J7-A8I2-01A	KIRP	5.51
TCGA-5P-A9KC-01A	KIRP	7.6
TCGA-BQ-5883-01A	KIRP	7.16
TCGA-BQ-7055-01A	KIRP	7.64
TCGA-V9-A7HT-01A	KIRP	7.12
TCGA-BQ-7061-01A	KIRP	7.49
TCGA-AL-3467-01A	KIRP	7.32
TCGA-B1-5398-01A	KIRP	6.98
TCGA-BQ-7053-01A	KIRP	8.02
TCGA-2K-A9WE-01A	KIRP	6.93
TCGA-IA-A40U-01A	KIRP	7.82
TCGA-KV-A74V-01A	KIRP	9.1
TCGA-B9-A8YH-01A	KIRP	9.31
TCGA-AL-3468-01A	KIRP	8.1
TCGA-A4-8312-01A	KIRP	7.29
TCGA-B3-3926-01A	KIRP	8.86
TCGA-EV-5903-01A	KIRP	8.06
TCGA-IA-A40Y-01A	KIRP	6.63
TCGA-J7-6720-01A	KIRP	8.71
TCGA-BQ-5887-01A	KIRP	6.54

TCGA-UZ-A9PO-01A	KIRP	7.86
TCGA-UZ-A9Q0-01A	KIRP	6.62
TCGA-2Z-A9JQ-01A	KIRP	8.48
TCGA-B1-7332-01A	KIRP	8.73
TCGA-BQ-5892-01A	KIRP	7.61
TCGA-SX-A71W-01A	KIRP	8.41
TCGA-G7-A8LC-01A	KIRP	7.63
TCGA-G7-6793-01A	KIRP	7.21
TCGA-P4-A5E6-01A	KIRP	6.92
TCGA-BQ-5882-01A	KIRP	6.82
TCGA-B9-A5W9-01A	KIRP	8.17
TCGA-BQ-7051-01A	KIRP	8.98
TCGA-G7-6789-01A	KIRP	6.18
TCGA-AL-7173-01A	KIRP	7.18
TCGA-4A-A93Y-01A	KIRP	6.1
TCGA-UZ-A9PJ-01A	KIRP	9.17
TCGA-2Z-A9J1-01A	KIRP	8.35
TCGA-MH-A560-01A	KIRP	6.69
TCGA-DZ-6132-01A	KIRP	7.68
TCGA-2Z-A9JR-01A	KIRP	8.04
TCGA-5P-A9JV-01A	KIRP	8.45
TCGA-BQ-7049-01A	KIRP	6.87
TCGA-SX-A7SN-01A	KIRP	8.02
TCGA-IZ-A6M8-01A	KIRP	7.56
TCGA-2Z-A9J2-01A	KIRP	5.89
TCGA-IZ-8195-01A	KIRP	6.8
TCGA-BQ-5880-01A	KIRP	6.57
TCGA-BQ-5879-01A	KIRP	7.42
TCGA-B9-4115-01A	KIRP	8.55
TCGA-P4-A5EB-01A	KIRP	8.12
TCGA-BQ-7062-01A	KIRP	8.14
TCGA-GL-A59R-01A	KIRP	9.46
TCGA-G7-A4TM-01A	KIRP	7.46
TCGA-F9-A8NY-01A	KIRP	7.49
TCGA-AL-3466-01A	KIRP	6.28
TCGA-A4-8098-01A	KIRP	7.37
TCGA-B1-A656-01A	KIRP	6.63
TCGA-SX-A7SS-01A	KIRP	7.97
TCGA-A4-A48D-01A	KIRP	6.56
TCGA-UZ-A9PK-01A	KIRP	8.64
TCGA-HE-A5NH-01A	KIRP	8.82
TCGA-DZ-6133-01A	KIRP	9.52
TCGA-B9-7268-01A	KIRP	9.27
TCGA-MH-A857-01A	KIRP	7.61
TCGA-BQ-5893-01A	KIRP	7.04
TCGA-A4-7286-01A	KIRP	7
TCGA-Y8-A8RY-01A	KIRP	7.94
TCGA-Y8-A896-01A	KIRP	7.37
TCGA-SX-A7SQ-01A	KIRP	8.45
TCGA-UZ-A9PM-01A	KIRP	7.22
TCGA-5P-A9JU-01A	KIRP	5.87
TCGA-G7-6797-01A	KIRP	8.68
TCGA-BQ-7056-01A	KIRP	7.37
TCGA-BQ-7058-01A	KIRP	6.15
TCGA-SX-A7SL-01A	KIRP	8.03
TCGA-HE-7129-01A	KIRP	9.49
TCGA-GL-A4EM-01A	KIRP	5.98
TCGA-BQ-7045-01A	KIRP	7.2

TCGA-PJ-A5Z8-01A	KIRP	7.31
TCGA-WN-A9G9-01A	KIRP	6.54
TCGA-B9-4617-01A	KIRP	8.47
TCGA-4A-A93W-01A	KIRP	8.32
TCGA-BQ-7048-01A	KIRP	6.93
TCGA-BQ-5878-01A	KIRP	9.01
TCGA-A4-8518-01A	KIRP	7.6
TCGA-5P-A9K4-01A	KIRP	7.27
TCGA-A4-A5DU-01A	KIRP	7.67
TCGA-WN-AB4C-01A	KIRP	5.63
TCGA-SX-A7SR-01A	KIRP	9.01
TCGA-MH-A55W-01A	KIRP	8.48
TCGA-A4-A6HP-01A	KIRP	7.67
TCGA-B1-A654-01A	KIRP	8.44
TCGA-B9-5156-01A	KIRP	8.23
TCGA-DW-7836-01A	KIRP	9.02
TCGA-B3-4103-01A	KIRP	8.83
TCGA-GL-A59T-01A	KIRP	8.75
TCGA-A4-A5Y0-01A	KIRP	6.49
TCGA-BQ-7059-01A	KIRP	7.09
TCGA-A4-7997-01A	KIRP	7.71
TCGA-AT-A5NU-01A	KIRP	8.91
TCGA-UZ-A9PR-01A	KIRP	8.16
TCGA-Y8-A8RZ-01A	KIRP	6.24
TCGA-MH-A562-01A	KIRP	7.46
TCGA-2Z-A9J3-01A	KIRP	8.39
TCGA-5P-A9JY-01A	KIRP	8.48
TCGA-P4-AAVM-01A	KIRP	6.77
TCGA-Y8-A894-01A	KIRP	8.67
TCGA-P4-A5EA-01A	KIRP	6.29
TCGA-DW-5560-01A	KIRP	6.9
TCGA-G7-7501-01A	KIRP	7.67
TCGA-BQ-5888-01A	KIRP	6.46
TCGA-B9-5155-01A	KIRP	9.6
TCGA-BQ-7060-01A	KIRP	6.95
TCGA-A4-A5Y1-01A	KIRP	7.47
TCGA-DZ-6131-01A	KIRP	6.95
TCGA-UZ-A9PS-05A	KIRP	7.08
TCGA-B1-A47O-01A	KIRP	7.46
TCGA-B9-4113-01A	KIRP	8.51
TCGA-DW-7963-01B	KIRP	6.57
TCGA-B3-3925-01A	KIRP	8.3
TCGA-B1-A655-01A	KIRP	7.91
TCGA-MH-A856-01A	KIRP	7.41
TCGA-BQ-5875-01A	KIRP	7.18
TCGA-Q2-A5QZ-01A	KIRP	6.67
TCGA-5P-A9KH-01A	KIRP	7.26
TCGA-P4-AAVO-01A	KIRP	6.27
TCGA-UZ-A9PV-01A	KIRP	9
TCGA-B9-4116-01A	KIRP	7.52
TCGA-GL-A9DD-01A	KIRP	6.65
TCGA-GL-A9DE-01A	KIRP	7.22
TCGA-MH-A855-01A	KIRP	7.97
TCGA-A4-7915-01A	KIRP	6.78
TCGA-GL-8500-01A	KIRP	9.16
TCGA-HE-7130-01A	KIRP	7.64
TCGA-P4-AAVL-01A	KIRP	7.77
TCGA-A4-7828-01A	KIRP	7.3

TCGA-SX-A7SU-01A	KIRP	7.1
TCGA-G7-A8LE-01A	KIRP	8.65
TCGA-Y8-A898-01A	KIRP	7.75
TCGA-Y8-A8S1-01A	KIRP	5.79
TCGA-SX-A7SM-01A	KIRP	7.15
TCGA-5P-A9K3-01A	KIRP	7.22
TCGA-BQ-5877-01A	KIRP	7.54
TCGA-AL-3472-01A	KIRP	8.16
TCGA-IA-A83W-01A	KIRP	7.43
TCGA-P4-AAVK-01A	KIRP	7.37
TCGA-F9-A97G-01A	KIRP	7.07
TCGA-BQ-7050-01A	KIRP	8.88
TCGA-B1-A657-01A	KIRP	8.4
TCGA-F9-A4JJ-01A	KIRP	7.33
TCGA-5P-A9KF-01A	KIRP	7.7
TCGA-UZ-A9PN-01A	KIRP	7.03
TCGA-5P-A9KE-01A	KIRP	7.21
TCGA-DW-7840-01A	KIRP	8.12
TCGA-5P-A9K0-01A	KIRP	7.31
TCGA-F9-A7Q0-01A	KIRP	8.67
TCGA-HE-A5NF-01A	KIRP	7.82
TCGA-BQ-5881-01A	KIRP	5.59
TCGA-2Z-A9JN-01A	KIRP	6.53
TCGA-2Z-A9JI-01A	KIRP	8.51
TCGA-G7-6792-01A	KIRP	7.49
TCGA-GL-6846-01A	KIRP	6.63
TCGA-2Z-A9JJ-01A	KIRP	7.33
TCGA-B1-A47M-01A	KIRP	6.74
TCGA-2Z-A9J6-01A	KIRP	8.57
TCGA-DW-7839-01A	KIRP	8.81
TCGA-5P-A9JW-01A	KIRP	6.56
TCGA-SX-A7SO-01A	KIRP	8.83
TCGA-UZ-A9PS-01A	KIRP	8.97
TCGA-IZ-8196-01A	KIRP	9.02
TCGA-A4-7732-01A	KIRP	8.44
TCGA-2Z-A9J9-01A	KIRP	7.41
TCGA-G7-6796-01A	KIRP	8.5
TCGA-2Z-A9JO-01A	KIRP	6.98
TCGA-F9-A7VF-01A	KIRP	9.05
TCGA-GL-7773-01A	KIRP	8.11
TCGA-2Z-A9JT-01A	KIRP	6.91
TCGA-DZ-6135-01A	KIRP	7.52
TCGA-BQ-5889-01A	KIRP	6.62
TCGA-B9-A69E-01A	KIRP	6.31
TCGA-B9-A44B-01A	KIRP	6.26
TCGA-HE-7128-01A	KIRP	7.43
TCGA-UZ-A9PU-01A	KIRP	8.16
TCGA-4A-A93X-01A	KIRP	6.99
TCGA-A4-7288-01A	KIRP	9.23
TCGA-2Z-A9JP-01A	KIRP	6.94
TCGA-DZ-6134-01A	KIRP	7.52
TCGA-B3-4104-01A	KIRP	7.58
TCGA-KV-A6GD-01A	KIRP	7.36
TCGA-A4-7583-01A	KIRP	8.24
TCGA-BQ-5890-01A	KIRP	7.03
TCGA-BQ-5894-01A	KIRP	7.53
TCGA-A4-8311-01A	KIRP	7.29
TCGA-Y8-A895-01A	KIRP	7.92

TCGA-B3-8121-01A	KIRP	8.71
TCGA-2Z-A9J7-01A	KIRP	8.06
TCGA-A4-A57E-01A	KIRP	6.74
TCGA-IA-A83V-01A	KIRP	7.16
TCGA-DW-7838-01A	KIRP	7.82
TCGA-2Z-A9J5-01A	KIRP	8.08
TCGA-BQ-5885-01A	KIRP	7.5
TCGA-HE-A5NI-01A	KIRP	7.28
TCGA-B1-A47N-01A	KIRP	7.22
TCGA-G7-A8LD-01A	KIRP	6.98
TCGA-5P-A9K9-01A	KIRP	6.87
TCGA-PJ-A5Z9-01A	KIRP	9.52
TCGA-AL-3471-01A	KIRP	8.07
TCGA-UZ-A9PX-01A	KIRP	7.86
TCGA-A4-A5XZ-01A	KIRP	8.67
TCGA-2Z-A9JK-01A	KIRP	6.89
TCGA-P4-A5E7-01A	KIRP	7.89
TCGA-BQ-5891-01A	KIRP	8.25
TCGA-2Z-A9JE-01A	KIRP	8.53
TCGA-SX-A71U-01A	KIRP	8.19
TCGA-2Z-A9JD-01A	KIRP	7.23
TCGA-BQ-5884-01A	KIRP	7.9
TCGA-BQ-5886-01A	KIRP	6.63
TCGA-2Z-A9JL-01A	KIRP	7.57
TCGA-SX-A71S-01A	KIRP	9.34
TCGA-SX-A7SP-01A	KIRP	7.45
TCGA-O9-A75Z-01A	KIRP	8.04
TCGA-IZ-A6M9-01A	KIRP	7.5
TCGA-A4-8630-01A	KIRP	8.43
TCGA-A4-7287-01A	KIRP	7.1
TCGA-2Z-A9JS-01A	KIRP	9.18
TCGA-B9-A5W8-01A	KIRP	7.77
TCGA-AL-A5DJ-01A	KIRP	6.69
TCGA-UZ-A9PQ-01A	KIRP	8.06
TCGA-5P-A9K8-01A	KIRP	7.76
TCGA-G7-7502-01A	KIRP	7.09
TCGA-A4-7734-01A	KIRP	8.62
TCGA-MH-A854-01A	KIRP	8.47
TCGA-A4-8515-01A	KIRP	8.91
TCGA-5P-A9K6-01A	KIRP	7.83
TCGA-G7-A8LB-01A	KIRP	7.46
TCGA-B3-A6W5-01A	KIRP	9.25
TCGA-Y8-A897-01A	KIRP	9.32
TCGA-UZ-A9Q1-01A	KIRP	6.88
TCGA-A4-7585-01A	KIRP	7.69
TCGA-BQ-7044-01A	KIRP	6.85
TCGA-A4-7584-01A	KIRP	7.97
TCGA-UZ-A9PP-01A	KIRP	7.89
TCGA-GL-A9DC-01A	KIRP	9.04
TCGA-2Z-A9JM-01A	KIRP	7.58
TCGA-BQ-5876-01A	KIRP	7.22
TCGA-5P-A9KA-01A	KIRP	7
TCGA-BQ-7046-01A	KIRP	7.16
TCGA-P4-A5ED-01A	KIRP	6.7
TCGA-DW-7837-01A	KIRP	8.69
TCGA-A4-8516-01A	KIRP	9.25
TCGA-IA-A40X-01A	KIRP	8.23
TCGA-G7-6795-01A	KIRP	8.18

TCGA-DW-7842-01A	KIRP	8.96
TCGA-GL-7966-01A	KIRP	6.81
TCGA-AB-2937-03A	LAML	5.41
TCGA-AB-2863-03A	LAML	7.49
TCGA-AB-2941-03A	LAML	6.37
TCGA-AB-2910-03A	LAML	7.09
TCGA-AB-2856-03A	LAML	6.85
TCGA-AB-2874-03A	LAML	6.9
TCGA-AB-2812-03A	LAML	6.85
TCGA-AB-2891-03A	LAML	6.86
TCGA-AB-2851-03A	LAML	7.07
TCGA-AB-2811-03B	LAML	6.22
TCGA-AB-2894-03A	LAML	6.62
TCGA-AB-2876-03A	LAML	6.81
TCGA-AB-2975-03A	LAML	6.12
TCGA-AB-2980-03A	LAML	5.83
TCGA-AB-2949-03B	LAML	7.02
TCGA-AB-2909-03A	LAML	7
TCGA-AB-2921-03A	LAML	7.26
TCGA-AB-2936-03A	LAML	6.42
TCGA-AB-2966-03A	LAML	6.06
TCGA-AB-2849-03A	LAML	6.59
TCGA-AB-2998-03A	LAML	6.59
TCGA-AB-2981-03B	LAML	7.2
TCGA-AB-2825-03A	LAML	7.5
TCGA-AB-2927-03A	LAML	6.18
TCGA-AB-2881-03A	LAML	7.19
TCGA-AB-2931-03A	LAML	7.18
TCGA-AB-3007-03A	LAML	6.02
TCGA-AB-2828-03A	LAML	7.33
TCGA-AB-2948-03A	LAML	6.99
TCGA-AB-2963-03A	LAML	7.23
TCGA-AB-2935-03A	LAML	6.85
TCGA-AB-2818-03A	LAML	7.12
TCGA-AB-2883-03A	LAML	5.34
TCGA-AB-2869-03A	LAML	6.79
TCGA-AB-2880-03A	LAML	6.96
TCGA-AB-2834-03A	LAML	6.69
TCGA-AB-2984-03A	LAML	5.09
TCGA-AB-2914-03A	LAML	6.91
TCGA-AB-2861-03A	LAML	6.71
TCGA-AB-2882-03A	LAML	6.32
TCGA-AB-2823-03A	LAML	6.78
TCGA-AB-2813-03A	LAML	6.9
TCGA-AB-2898-03A	LAML	6.46
TCGA-AB-2853-03A	LAML	7.16
TCGA-AB-2893-03A	LAML	6.99
TCGA-AB-2871-03A	LAML	6.52
TCGA-AB-2859-03A	LAML	6.85
TCGA-AB-2920-03B	LAML	5.63
TCGA-AB-2822-03A	LAML	6.71
TCGA-AB-2846-03A	LAML	7.14
TCGA-AB-2885-03A	LAML	7.24
TCGA-AB-2913-03A	LAML	6.36
TCGA-AB-2899-03A	LAML	6.68
TCGA-AB-2970-03A	LAML	6.74
TCGA-AB-2847-03A	LAML	6.53
TCGA-AB-2971-03A	LAML	6.41

TCGA-AB-2986-03A	LAML	6.23
TCGA-AB-2826-03A	LAML	6.86
TCGA-AB-2895-03A	LAML	7.02
TCGA-AB-2924-03A	LAML	7.23
TCGA-AB-2991-03A	LAML	6.07
TCGA-AB-2912-03A	LAML	6.95
TCGA-AB-2911-03A	LAML	5.85
TCGA-AB-2959-03A	LAML	6.72
TCGA-AB-2965-03A	LAML	7.21
TCGA-AB-2872-03A	LAML	4.88
TCGA-AB-2983-03A	LAML	6.41
TCGA-AB-2999-03B	LAML	5.47
TCGA-AB-3002-03A	LAML	6.88
TCGA-AB-2996-03A	LAML	6.45
TCGA-AB-2944-03A	LAML	7.08
TCGA-AB-2930-03A	LAML	6.81
TCGA-AB-2901-03A	LAML	7.8
TCGA-AB-2842-03A	LAML	6.75
TCGA-AB-2890-03A	LAML	6.55
TCGA-AB-2844-03A	LAML	6.89
TCGA-AB-2925-03A	LAML	6.84
TCGA-AB-2933-03A	LAML	6.46
TCGA-AB-2889-03A	LAML	6.83
TCGA-AB-3001-03A	LAML	5.99
TCGA-AB-2940-03A	LAML	6.42
TCGA-AB-2897-03A	LAML	5.5
TCGA-AB-2908-03A	LAML	5.59
TCGA-AB-2987-03A	LAML	7.05
TCGA-AB-2973-03A	LAML	6.68
TCGA-AB-2896-03B	LAML	6.89
TCGA-AB-2841-03B	LAML	5.31
TCGA-AB-3000-03A	LAML	7.04
TCGA-AB-2810-03A	LAML	6.46
TCGA-AB-2943-03A	LAML	6.1
TCGA-AB-2840-03A	LAML	5.67
TCGA-AB-2888-03B	LAML	7.17
TCGA-AB-3008-03A	LAML	6.67
TCGA-AB-2830-03A	LAML	6.86
TCGA-AB-2858-03A	LAML	6.82
TCGA-AB-2815-03A	LAML	7.18
TCGA-AB-2886-03A	LAML	7.01
TCGA-AB-2820-03A	LAML	7.23
TCGA-AB-2942-03A	LAML	6.75
TCGA-AB-2865-03A	LAML	6.95
TCGA-AB-2915-03A	LAML	5.85
TCGA-AB-3012-03A	LAML	5.77
TCGA-AB-2952-03B	LAML	5.99
TCGA-AB-2877-03A	LAML	6.69
TCGA-AB-2873-03A	LAML	6.85
TCGA-AB-2946-03A	LAML	6.68
TCGA-AB-2836-03A	LAML	7.26
TCGA-AB-2988-03B	LAML	7.2
TCGA-AB-2939-03A	LAML	6.74
TCGA-AB-2892-03A	LAML	6.58
TCGA-AB-2839-03A	LAML	6.72
TCGA-AB-2862-03A	LAML	5.88
TCGA-AB-2929-03A	LAML	7.08
TCGA-AB-2916-03A	LAML	6.93

TCGA-AB-2806-03A	LAML	7.08
TCGA-AB-2990-03B	LAML	6.13
TCGA-AB-2995-03A	LAML	6.5
TCGA-AB-2835-03A	LAML	7.47
TCGA-AB-2808-03A	LAML	6.65
TCGA-AB-2843-03A	LAML	6.55
TCGA-AB-3011-03A	LAML	6.79
TCGA-AB-2857-03A	LAML	6.33
TCGA-AB-2884-03A	LAML	7.1
TCGA-AB-2875-03A	LAML	7.25
TCGA-AB-2992-03A	LAML	6.19
TCGA-AB-2866-03A	LAML	7.2
TCGA-AB-2917-03A	LAML	6.65
TCGA-AB-3009-03A	LAML	6.97
TCGA-AB-2870-03A	LAML	7.15
TCGA-AB-2817-03A	LAML	7.05
TCGA-AB-2950-03A	LAML	6.94
TCGA-AB-2932-03A	LAML	7.25
TCGA-AB-2819-03A	LAML	7.08
TCGA-AB-2900-03A	LAML	7.06
TCGA-AB-2928-03A	LAML	7.52
TCGA-AB-2845-03B	LAML	6.52
TCGA-AB-2814-03A	LAML	6.47
TCGA-AB-2821-03A	LAML	6.79
TCGA-AB-2977-03B	LAML	6.08
TCGA-AB-2938-03A	LAML	6.04
TCGA-AB-2867-03A	LAML	7.08
TCGA-AB-2878-03A	LAML	6.02
TCGA-AB-2934-03A	LAML	6.81
TCGA-AB-2919-03A	LAML	6.78
TCGA-AB-2976-03A	LAML	6.49
TCGA-AB-2805-03A	LAML	7.73
TCGA-AB-2994-03A	LAML	6.51
TCGA-AB-2956-03A	LAML	7.46
TCGA-AB-2955-03A	LAML	5.79
TCGA-AB-2979-03B	LAML	6.24
TCGA-AB-2918-03A	LAML	6.99
TCGA-HT-7691-01A	LGG	8.54
TCGA-S9-A7J0-01A	LGG	8.16
TCGA-HT-7605-01A	LGG	8.97
TCGA-HT-A4DS-01A	LGG	8.33
TCGA-P5-A5EW-01A	LGG	10.18
TCGA-QH-A6CS-01A	LGG	7.83
TCGA-DU-6407-02A	LGG	8.13
TCGA-DU-8162-01A	LGG	8.46
TCGA-CS-4944-01A	LGG	9.1
TCGA-TM-A84I-01A	LGG	8.43
TCGA-E1-A7YS-01A	LGG	7.8
TCGA-S9-A6TU-01A	LGG	8.21
TCGA-RY-A83X-01A	LGG	8.95
TCGA-W9-A837-01A	LGG	9.47
TCGA-DU-A5TP-01A	LGG	8.46
TCGA-DU-A5TT-01A	LGG	7.89
TCGA-TM-A84M-01A	LGG	8.94
TCGA-HT-A61C-01A	LGG	6.88
TCGA-HT-7692-01A	LGG	8.5
TCGA-S9-A6U5-01A	LGG	8.91
TCGA-HT-7485-01A	LGG	9.95

TCGA-HT-7884-01B	LGG	9.34
TCGA-FG-6689-01A	LGG	9.54
TCGA-DU-5847-01A	LGG	8.04
TCGA-DH-A7UT-01A	LGG	8.65
TCGA-FG-7636-01A	LGG	9.61
TCGA-TM-A84J-01A	LGG	7.28
TCGA-DH-A7UR-01A	LGG	8.08
TCGA-VM-A8CD-01A	LGG	8.08
TCGA-HT-7603-01A	LGG	9.16
TCGA-QH-A6CZ-01A	LGG	9.1
TCGA-QH-A6X3-01A	LGG	9.75
TCGA-DU-A7TJ-01A	LGG	7.9
TCGA-FG-6690-01A	LGG	9.78
TCGA-S9-A7R3-01A	LGG	8.65
TCGA-QH-A65V-01A	LGG	8.81
TCGA-DB-5270-01A	LGG	9.43
TCGA-HW-8319-01A	LGG	9.7
TCGA-FG-A60J-01A	LGG	9.53
TCGA-WY-A85B-01A	LGG	9.23
TCGA-FG-7637-01A	LGG	8.88
TCGA-DU-7304-02A	LGG	8.18
TCGA-DH-A66F-01A	LGG	9.51
TCGA-VM-A8CA-01A	LGG	8.26
TCGA-CS-6669-01A	LGG	8.45
TCGA-S9-A6UB-01A	LGG	8.7
TCGA-TQ-A7RO-01A	LGG	9.38
TCGA-QH-A6CY-01A	LGG	9.44
TCGA-TQ-A7RQ-01A	LGG	8.66
TCGA-DU-5870-01A	LGG	9.26
TCGA-DU-7018-01A	LGG	8.51
TCGA-DU-5870-02A	LGG	8.74
TCGA-HT-7681-01A	LGG	9.86
TCGA-TM-A84H-01A	LGG	8.75
TCGA-HT-7860-01A	LGG	8.54
TCGA-HT-7467-01A	LGG	8.45
TCGA-TQ-A7RF-01A	LGG	8.84
TCGA-E1-A7YE-01A	LGG	8.34
TCGA-EZ-7264-01A	LGG	8.83
TCGA-RY-A847-01A	LGG	8.92
TCGA-DU-6408-01A	LGG	10.09
TCGA-DU-6393-01A	LGG	7.94
TCGA-DU-A7TI-01A	LGG	9.54
TCGA-FG-7641-01B	LGG	9.11
TCGA-DU-6402-01A	LGG	7
TCGA-HT-A614-01A	LGG	9.97
TCGA-DU-7006-01A	LGG	7.61
TCGA-P5-A72Z-01A	LGG	6.98
TCGA-DB-A4XE-01A	LGG	9.78
TCGA-P5-A737-01A	LGG	8.32
TCGA-RY-A83Y-01A	LGG	8.85
TCGA-DU-5874-01A	LGG	8.22
TCGA-DB-A4XD-01A	LGG	9.72
TCGA-DU-A6S3-01A	LGG	7.98
TCGA-HT-7607-01A	LGG	9.45
TCGA-FG-6691-01A	LGG	9.08
TCGA-WY-A859-01A	LGG	9.29
TCGA-HT-8013-01A	LGG	8.41
TCGA-HW-7493-01A	LGG	9.75

TCGA-DU-A7TD-01A	LGG	7.04
TCGA-HT-7877-01A	LGG	9.34
TCGA-HT-A616-01A	LGG	9.82
TCGA-WY-A85C-01A	LGG	8.02
TCGA-QH-A6X5-01A	LGG	9.13
TCGA-TM-A84B-01A	LGG	8.58
TCGA-FG-A4MY-01A	LGG	8.73
TCGA-DU-8164-01A	LGG	9.72
TCGA-HT-7606-01A	LGG	9.07
TCGA-TM-A84Q-01A	LGG	8.95
TCGA-S9-A89V-01A	LGG	8.24
TCGA-S9-A6WL-01A	LGG	8.19
TCGA-HT-A617-01A	LGG	7.45
TCGA-HT-8105-01A	LGG	8.32
TCGA-P5-A780-01A	LGG	7.86
TCGA-P5-A733-01A	LGG	9.41
TCGA-DU-6397-01A	LGG	8.76
TCGA-DB-A64L-01A	LGG	9.18
TCGA-DB-5277-01A	LGG	8.9
TCGA-DU-5855-01A	LGG	8.69
TCGA-HT-7468-01A	LGG	8.86
TCGA-DU-A7TB-01A	LGG	8.14
TCGA-DU-8166-01A	LGG	9.32
TCGA-DB-A4XB-01A	LGG	9.34
TCGA-HT-7601-01A	LGG	8.93
TCGA-S9-A6WM-01A	LGG	8.17
TCGA-HT-A74H-01A	LGG	7.55
TCGA-CS-4941-01A	LGG	8.34
TCGA-E1-A7YU-01A	LGG	9.32
TCGA-DU-8168-01A	LGG	8.22
TCGA-TM-A84T-01A	LGG	9.48
TCGA-DU-7007-01A	LGG	8.27
TCGA-S9-A7R2-01A	LGG	7.64
TCGA-TM-A84O-01A	LGG	8.67
TCGA-DB-A64Q-01A	LGG	9.25
TCGA-QH-A65S-01A	LGG	9.21
TCGA-DU-6405-01A	LGG	8.31
TCGA-S9-A7R1-01A	LGG	8.57
TCGA-HT-8111-01A	LGG	9.78
TCGA-CS-6668-01A	LGG	8.49
TCGA-TM-A84L-01A	LGG	9.61
TCGA-P5-A781-01A	LGG	9.24
TCGA-HT-7686-01A	LGG	9.42
TCGA-S9-A6WD-01A	LGG	8.55
TCGA-HT-7687-01A	LGG	8.56
TCGA-HT-7695-01A	LGG	9.52
TCGA-E1-5303-01A	LGG	9.04
TCGA-P5-A72U-01A	LGG	6.34
TCGA-S9-A7R4-01A	LGG	8.92
TCGA-HT-7478-01A	LGG	9.77
TCGA-HW-7486-01A	LGG	9.06
TCGA-DU-8167-01A	LGG	8.8
TCGA-CS-6290-01A	LGG	9.04
TCGA-HT-7857-01A	LGG	6.99
TCGA-DU-5849-01A	LGG	9.22
TCGA-HT-7475-01A	LGG	9.29
TCGA-DU-5872-01A	LGG	8.91
TCGA-DU-5872-02A	LGG	6.99

TCGA-TQ-A7RV-02A	LGG	8.85
TCGA-HT-7693-01A	LGG	9.03
TCGA-VM-A8CE-01A	LGG	8.9
TCGA-S9-A6U6-01A	LGG	9.72
TCGA-P5-A77X-01A	LGG	9.08
TCGA-DB-A75P-01A	LGG	8.01
TCGA-HT-7882-01A	LGG	7.06
TCGA-S9-A7QY-01A	LGG	8.79
TCGA-HT-8558-01A	LGG	8.17
TCGA-HT-8108-01A	LGG	9.38
TCGA-E1-A7Z2-01A	LGG	8.66
TCGA-IK-8125-01A	LGG	9.27
TCGA-E1-5305-01A	LGG	9.82
TCGA-S9-A6WO-01A	LGG	9.34
TCGA-E1-A7YO-01A	LGG	8.97
TCGA-E1-A7YH-01A	LGG	9.11
TCGA-DB-A4XA-01A	LGG	8.76
TCGA-HW-A5KK-01A	LGG	7.66
TCGA-CS-6666-01A	LGG	9.37
TCGA-DB-5279-01A	LGG	7.71
TCGA-TQ-A7RI-01A	LGG	8.69
TCGA-DU-A7T6-01A	LGG	7.67
TCGA-DU-A6S8-01A	LGG	7.89
TCGA-E1-A7Z4-01A	LGG	9.44
TCGA-TM-A84C-01A	LGG	9.35
TCGA-DB-5276-01A	LGG	9.57
TCGA-DU-7300-01A	LGG	9.25
TCGA-DB-A64U-01A	LGG	9
TCGA-CS-6186-01A	LGG	8.41
TCGA-FG-A6J3-01A	LGG	7.07
TCGA-P5-A735-01A	LGG	8.94
TCGA-WY-A858-01A	LGG	8.09
TCGA-HT-7879-01A	LGG	9.94
TCGA-DU-7309-01A	LGG	10.05
TCGA-S9-A89Z-01A	LGG	8.28
TCGA-HT-A619-01A	LGG	8.11
TCGA-HT-7473-01A	LGG	8.74
TCGA-DU-7013-01A	LGG	7.59
TCGA-HT-A61B-01A	LGG	9.16
TCGA-R8-A73M-01A	LGG	8.66
TCGA-DU-7012-01A	LGG	8.39
TCGA-DU-5871-01A	LGG	9.06
TCGA-S9-A6TY-01A	LGG	8.61
TCGA-DH-5143-01A	LGG	9.6
TCGA-HT-A5R9-01A	LGG	7.88
TCGA-FG-8186-01A	LGG	9.48
TCGA-P5-A5ET-01A	LGG	9.36
TCGA-FG-A4MT-01A	LGG	9.22
TCGA-FG-A4MW-01A	LGG	7.5
TCGA-FG-A4MT-02A	LGG	9.2
TCGA-TQ-A7RJ-01A	LGG	9.23
TCGA-P5-A77W-01A	LGG	7.91
TCGA-HT-8564-01A	LGG	9.17
TCGA-HT-A61A-01A	LGG	8.74
TCGA-HT-7689-01A	LGG	9.39
TCGA-S9-A6WE-01A	LGG	8.88
TCGA-FG-5964-01A	LGG	8.94
TCGA-DU-6407-02B	LGG	8.24

TCGA-HT-A5R5-01A	LGG	9.06
TCGA-E1-A7YQ-01A	LGG	6.92
TCGA-HT-7688-01A	LGG	9.07
TCGA-DU-7010-01A	LGG	8.96
TCGA-KT-A7W1-01A	LGG	8.01
TCGA-DB-A4XG-01A	LGG	8.98
TCGA-RY-A83Z-01A	LGG	8.69
TCGA-DB-A64P-01A	LGG	8.75
TCGA-DU-A5TU-01A	LGG	8.56
TCGA-DB-5281-01A	LGG	9.34
TCGA-TM-A84R-01A	LGG	9.21
TCGA-WY-A85E-01A	LGG	9.12
TCGA-HT-7476-01A	LGG	9.18
TCGA-DU-7298-01A	LGG	9.27
TCGA-E1-A7YV-01A	LGG	8.41
TCGA-P5-A5EX-01A	LGG	7.86
TCGA-HT-7482-01A	LGG	9.37
TCGA-QH-A65X-01A	LGG	8.34
TCGA-E1-A7YD-01A	LGG	7.92
TCGA-HT-7472-01A	LGG	9.81
TCGA-DB-A4XF-01A	LGG	9.02
TCGA-P5-A72W-01A	LGG	8.94
TCGA-FG-6692-01A	LGG	6.57
TCGA-DB-A64S-01A	LGG	9.93
TCGA-FN-7833-01A	LGG	8.95
TCGA-TQ-A7RR-01A	LGG	9.34
TCGA-FG-8182-01A	LGG	9.49
TCGA-DU-7302-01A	LGG	9.14
TCGA-E1-A7YM-01A	LGG	8.03
TCGA-QH-A65R-01A	LGG	8.39
TCGA-VM-A8C9-01A	LGG	7.14
TCGA-HT-7854-01A	LGG	8.87
TCGA-DU-A5TR-01A	LGG	8.27
TCGA-DU-A5TW-01A	LGG	8.61
TCGA-HT-7471-01A	LGG	8.74
TCGA-VM-A8CH-01A	LGG	9.45
TCGA-HT-A5RA-01A	LGG	8.62
TCGA-CS-6670-01A	LGG	9.03
TCGA-TQ-A7RH-01A	LGG	8.79
TCGA-DB-A4X9-01A	LGG	9.9
TCGA-FG-A60L-01A	LGG	8.62
TCGA-HT-A74O-01A	LGG	8.19
TCGA-R8-A6MK-01A	LGG	9.02
TCGA-FG-5963-02A	LGG	7.27
TCGA-DH-A7US-01A	LGG	8.89
TCGA-TQ-A7RG-01A	LGG	9.2
TCGA-FG-A87Q-01A	LGG	7.3
TCGA-HT-A5RC-01A	LGG	7.89
TCGA-DH-A669-02A	LGG	7.71
TCGA-FG-A87N-01A	LGG	8.55
TCGA-DU-6407-01A	LGG	9.31
TCGA-RY-A840-01A	LGG	9.35
TCGA-TQ-A7RP-01A	LGG	7.39
TCGA-E1-A7YY-01A	LGG	8.31
TCGA-HW-8322-01A	LGG	8.58
TCGA-HT-7474-01A	LGG	9.85
TCGA-FG-5963-01A	LGG	8.06
TCGA-HT-7602-01A	LGG	9.81

TCGA-P5-A5EZ-01A	LGG	7.77
TCGA-QH-A6XC-01A	LGG	7.47
TCGA-P5-A5F0-01A	LGG	9.46
TCGA-DU-7301-01A	LGG	9.39
TCGA-DU-6403-01A	LGG	7.42
TCGA-F6-A8O3-01A	LGG	9.47
TCGA-HT-8012-01A	LGG	8.53
TCGA-HW-A5KJ-01A	LGG	7.74
TCGA-S9-A7IY-01A	LGG	9
TCGA-QH-A6X4-01A	LGG	8.38
TCGA-S9-A7R8-01A	LGG	8.08
TCGA-TM-A7C5-01A	LGG	8.17
TCGA-DU-A6S7-01A	LGG	9.62
TCGA-S9-A6U1-01A	LGG	10.12
TCGA-DB-A64X-01A	LGG	9.5
TCGA-HT-7676-01A	LGG	10.07
TCGA-HT-7479-01A	LGG	9.18
TCGA-VM-A8C8-01A	LGG	9.39
TCGA-P5-A736-01A	LGG	7.91
TCGA-CS-4943-01A	LGG	9.12
TCGA-DH-A7UU-01A	LGG	8.9
TCGA-S9-A7QZ-01A	LGG	8.85
TCGA-DU-A76R-01A	LGG	8.54
TCGA-HT-8019-01A	LGG	8.19
TCGA-FG-A70Z-01A	LGG	8.13
TCGA-HT-8113-01A	LGG	8.99
TCGA-CS-6667-01A	LGG	9.01
TCGA-QH-A86X-01A	LGG	9.44
TCGA-IK-7675-01A	LGG	9.02
TCGA-E1-5322-01A	LGG	10.16
TCGA-E1-A7YW-01A	LGG	9.53
TCGA-F6-A8O4-01A	LGG	9.49
TCGA-E1-5304-01A	LGG	8.83
TCGA-S9-A7QW-01A	LGG	9.9
TCGA-HW-7489-01A	LGG	9.55
TCGA-QH-A6CU-01A	LGG	7.98
TCGA-DH-A669-01A	LGG	7.62
TCGA-HT-A5R7-01A	LGG	9.23
TCGA-E1-5311-01A	LGG	8.83
TCGA-TQ-A7RK-02A	LGG	9.52
TCGA-DU-A76K-01A	LGG	8.35
TCGA-HT-7881-01A	LGG	9
TCGA-S9-A7IZ-01A	LGG	8.48
TCGA-HT-7855-01A	LGG	9.19
TCGA-FG-8191-01A	LGG	9.16
TCGA-S9-A7J3-01A	LGG	9.3
TCGA-P5-A5F1-01A	LGG	9.37
TCGA-S9-A6WH-01A	LGG	7.82
TCGA-S9-A6TV-01A	LGG	8.91
TCGA-HW-A5KL-01A	LGG	8.86
TCGA-HT-7611-01A	LGG	9.92
TCGA-WY-A85D-01A	LGG	8.37
TCGA-VV-A829-01A	LGG	8.29
TCGA-S9-A6WQ-01A	LGG	9.17
TCGA-DU-6396-01A	LGG	8.71
TCGA-CS-4942-01A	LGG	9.53
TCGA-DB-A64V-01A	LGG	8.85
TCGA-P5-A731-01A	LGG	8.59

TCGA-VM-A8CB-01A	LGG	8.33
TCGA-DB-A4XC-01A	LGG	9.8
TCGA-FG-7634-01A	LGG	8.85
TCGA-P5-A5EY-01A	LGG	9.07
TCGA-TQ-A7RK-01A	LGG	9.19
TCGA-QH-A870-01A	LGG	9.33
TCGA-P5-A72X-01A	LGG	8.93
TCGA-RY-A845-01A	LGG	9.31
TCGA-FG-5965-01B	LGG	9.72
TCGA-DU-6410-01A	LGG	7.96
TCGA-HT-7880-01A	LGG	8.75
TCGA-TM-A84F-01A	LGG	9.34
TCGA-P5-A730-01A	LGG	8.67
TCGA-S9-A6WI-01A	LGG	8.55
TCGA-S9-A7J2-01A	LGG	9.08
TCGA-TQ-A8XE-01A	LGG	8.92
TCGA-DU-7019-01A	LGG	9.72
TCGA-R8-A6MO-01A	LGG	8.64
TCGA-QH-A6CV-01A	LGG	7.7
TCGA-P5-A5F6-01A	LGG	6.13
TCGA-S9-A7J1-01A	LGG	9.53
TCGA-DH-5140-01A	LGG	7.96
TCGA-VM-A8CF-01A	LGG	7.86
TCGA-DH-5141-01A	LGG	9.04
TCGA-HT-7477-01B	LGG	8.72
TCGA-S9-A6U9-01A	LGG	8.4
TCGA-CS-6188-01A	LGG	7.95
TCGA-S9-A6TX-01A	LGG	8.27
TCGA-R8-A6ML-01A	LGG	9.07
TCGA-S9-A7QX-01A	LGG	8.9
TCGA-FG-8189-01B	LGG	8.5
TCGA-HT-A74L-01A	LGG	8.8
TCGA-HT-7610-01A	LGG	9.53
TCGA-DU-5851-01A	LGG	8.92
TCGA-FG-A710-01A	LGG	9.04
TCGA-VW-A8FI-01A	LGG	7.62
TCGA-E1-A7YI-01A	LGG	7.52
TCGA-DH-5142-01A	LGG	9.9
TCGA-DB-A75K-01A	LGG	8.52
TCGA-HT-8104-01A	LGG	7.9
TCGA-TQ-A7RM-01A	LGG	8.93
TCGA-RY-A843-01A	LGG	9.25
TCGA-VW-A7QS-01A	LGG	8.39
TCGA-DB-5278-01A	LGG	9.79
TCGA-HT-A4DV-01A	LGG	9.27
TCGA-HW-8321-01A	LGG	9.22
TCGA-HT-7604-01A	LGG	9.41
TCGA-S9-A6U2-01A	LGG	9.74
TCGA-FG-5965-02A	LGG	7.71
TCGA-CS-4938-01B	LGG	9.8
TCGA-DU-7009-01A	LGG	8.98
TCGA-DB-5280-01A	LGG	10.69
TCGA-DU-6400-01A	LGG	8.22
TCGA-FG-8181-01A	LGG	8.12
TCGA-DU-7292-01A	LGG	8.16
TCGA-WY-A85A-01A	LGG	9.97
TCGA-DU-8165-01A	LGG	7.78
TCGA-TQ-A8XE-02A	LGG	8.62

TCGA-DB-5274-01A	LGG	7.85
TCGA-HT-A74J-01A	LGG	8.78
TCGA-P5-A5EU-01A	LGG	8.39
TCGA-QH-A6X9-01A	LGG	9.34
TCGA-VV-A86M-01A	LGG	8.6
TCGA-TM-A7CA-01A	LGG	9.12
TCGA-DU-5853-01A	LGG	9.95
TCGA-TQ-A7RK-02B	LGG	9.24
TCGA-FG-7638-01B	LGG	9.41
TCGA-DU-A5TY-01A	LGG	7.58
TCGA-FG-A711-01A	LGG	9.77
TCGA-DU-7294-01A	LGG	9.28
TCGA-KT-A74X-01A	LGG	8.32
TCGA-HW-7495-01A	LGG	9.22
TCGA-DH-A7UV-01A	LGG	9.51
TCGA-E1-5318-01A	LGG	8.85
TCGA-TQ-A7RS-01A	LGG	8.56
TCGA-HT-7694-01A	LGG	9.36
TCGA-DU-A6S6-01A	LGG	8.67
TCGA-DU-7008-01A	LGG	9.32
TCGA-HT-7608-01A	LGG	9.12
TCGA-QH-A6XA-01A	LGG	9.79
TCGA-DU-6394-01A	LGG	8.47
TCGA-HT-7680-01A	LGG	8.53
TCGA-TQ-A7RV-01A	LGG	8.95
TCGA-DB-A4XH-01A	LGG	9.13
TCGA-DU-A7TC-01A	LGG	9.16
TCGA-TM-A84G-01A	LGG	9.18
TCGA-DU-A6S2-01A	LGG	8.64
TCGA-CS-5396-01A	LGG	7.85
TCGA-HT-8110-01A	LGG	8.3
TCGA-E1-A7YK-01A	LGG	8.03
TCGA-QH-A65Z-01A	LGG	9.36
TCGA-DU-7306-01A	LGG	8.97
TCGA-DU-8161-01A	LGG	7.73
TCGA-TQ-A7RN-01A	LGG	8.92
TCGA-CS-5390-01A	LGG	8.32
TCGA-DU-8163-01A	LGG	9.06
TCGA-HT-8015-01B	LGG	8.27
TCGA-E1-A7Z6-01A	LGG	9.58
TCGA-DU-7299-01A	LGG	9.75
TCGA-CS-5394-01A	LGG	9.46
TCGA-E1-A7YL-01A	LGG	7.03
TCGA-DU-6401-01A	LGG	9.04
TCGA-HT-A618-01A	LGG	9.31
TCGA-FG-A60K-01A	LGG	9.16
TCGA-HW-7491-01A	LGG	9.3
TCGA-FG-A4MX-01A	LGG	7.56
TCGA-S9-A6WG-01A	LGG	8.41
TCGA-DU-5852-01A	LGG	8.26
TCGA-DB-5273-01A	LGG	9.91
TCGA-HT-7616-01A	LGG	7.87
TCGA-S9-A6U0-01A	LGG	7.83
TCGA-S9-A7R7-01A	LGG	8.04
TCGA-FG-7643-01A	LGG	8.47
TCGA-S9-A6WN-01A	LGG	7.47
TCGA-HT-7856-01A	LGG	9.21
TCGA-DU-8158-01A	LGG	7.51

TCGA-S9-A6U8-01A	LGG	8.76
TCGA-E1-A7YJ-01A	LGG	7.77
TCGA-S9-A6TZ-01A	LGG	9.31
TCGA-DU-6404-02A	LGG	8.03
TCGA-HT-7858-01A	LGG	9.04
TCGA-HT-A74K-01A	LGG	7.94
TCGA-HT-8106-01A	LGG	7.9
TCGA-HT-7609-01A	LGG	9.42
TCGA-HT-7481-01A	LGG	9.47
TCGA-E1-5307-01A	LGG	9.59
TCGA-DB-5275-01A	LGG	9.87
TCGA-DU-5854-01A	LGG	7.98
TCGA-HT-7684-01A	LGG	9.3
TCGA-DU-A76O-01A	LGG	9.16
TCGA-E1-A7YN-01A	LGG	7.67
TCGA-TQ-A7RU-01A	LGG	8.66
TCGA-DH-A66B-01A	LGG	9.11
TCGA-DU-6406-01A	LGG	7.58
TCGA-HT-7677-01A	LGG	9.22
TCGA-HT-7480-01A	LGG	8.75
TCGA-DU-A76L-01A	LGG	7.1
TCGA-E1-A7Z3-01A	LGG	8.66
TCGA-FG-5965-02B	LGG	8.35
TCGA-WH-A86K-01A	LGG	9.38
TCGA-HW-7487-01A	LGG	9.25
TCGA-DU-7014-01A	LGG	8.58
TCGA-S9-A7IX-01A	LGG	7.52
TCGA-R8-A6YH-01A	LGG	9.14
TCGA-HW-7490-01A	LGG	9.27
TCGA-DU-6404-02B	LGG	7.59
TCGA-P5-A5F2-01A	LGG	8.19
TCGA-S9-A6TW-01A	LGG	8.21
TCGA-HT-7875-01A	LGG	9.34
TCGA-HT-8011-01A	LGG	7.75
TCGA-DU-6399-01A	LGG	9.69
TCGA-S9-A6TS-01A	LGG	9.59
TCGA-DU-A7TG-01A	LGG	8.79
TCGA-DU-6395-01A	LGG	9.54
TCGA-DB-A64R-01A	LGG	9.12
TCGA-HT-7873-01B	LGG	10.04
TCGA-FG-A70Y-01A	LGG	9.62
TCGA-HT-7469-01A	LGG	8.54
TCGA-DU-6404-01A	LGG	9.03
TCGA-CS-5397-01A	LGG	8.06
TCGA-HT-8109-01A	LGG	9.09
TCGA-DB-A75L-01A	LGG	9.35
TCGA-DU-7011-01A	LGG	9.18
TCGA-HT-7483-01A	LGG	10.06
TCGA-FG-8187-01A	LGG	8.43
TCGA-FG-A4MU-01B	LGG	7.37
TCGA-E1-5302-01A	LGG	9.23
TCGA-HT-8018-01A	LGG	8.98
TCGA-HT-7874-01A	LGG	9.45
TCGA-E1-5319-01A	LGG	9.4
TCGA-HT-7902-01A	LGG	9.49
TCGA-P5-A5F4-01A	LGG	9.52
TCGA-TM-A7CF-01A	LGG	9.36
TCGA-TM-A7CF-02A	LGG	9.48

TCGA-HT-8114-01A	LGG	9.73
TCGA-HT-7620-01A	LGG	8.9
TCGA-HW-A5KM-01A	LGG	9.1
TCGA-HT-A5RB-01A	LGG	9.13
TCGA-S9-A6WP-01A	LGG	8.53
TCGA-HT-8010-01A	LGG	9.35
TCGA-DB-A75M-01A	LGG	9.56
TCGA-DB-A75O-01A	LGG	9.29
TCGA-DU-A7T8-01A	LGG	9.04
TCGA-DB-A64W-01A	LGG	8.65
TCGA-DH-A66G-01A	LGG	9.26
TCGA-DU-A5TS-01A	LGG	8.97
TCGA-DU-6397-02A	LGG	7.68
TCGA-DU-7290-01A	LGG	7.65
TCGA-QH-A6X8-01A	LGG	8.15
TCGA-CS-5393-01A	LGG	8.83
TCGA-DU-A7TA-01A	LGG	9.28
TCGA-DH-A66D-01A	LGG	9.56
TCGA-P5-A5EV-01A	LGG	9.64
TCGA-S9-A7IS-01A	LGG	7.73
TCGA-HT-8107-01A	LGG	8.27
TCGA-QH-A6CW-01A	LGG	8.87
TCGA-FG-8188-01A	LGG	9.44
TCGA-DH-5144-01A	LGG	9.15
TCGA-DB-A64O-01A	LGG	8.04
TCGA-S9-A7IQ-01A	LGG	9.05
TCGA-TM-A84S-01A	LGG	9.23
TCGA-FG-6688-01A	LGG	7.62
TCGA-DU-6542-01A	LGG	9.26
TCGA-CS-6665-01A	LGG	7.84
TCGA-HT-8563-01A	LGG	7.45
TCGA-QH-A6CX-01A	LGG	6.97
TCGA-FG-A6IZ-01A	LGG	8.57
TCGA-TM-A7C3-01A	LGG	7.42
TCGA-HT-A615-01A	LGG	9.03
TCGA-S9-A6UA-01A	LGG	7.42
TCGA-FG-5962-01B	LGG	8.95
TCGA-FG-A6J1-01A	LGG	8.83
TCGA-HW-8320-01A	LGG	9.42
TCGA-HT-7690-01A	LGG	9.18
TCGA-FG-8185-01A	LGG	10.13
TCGA-DU-7015-01A	LGG	8.59
TCGA-DU-7304-01A	LGG	8.82
TCGA-CS-5395-01A	LGG	9.08
TCGA-TM-A7C4-01A	LGG	9.7
TCGA-HT-7470-01A	LGG	9.68
TCGA-TQ-A7RW-01A	LGG	8.96
TCGA-DU-6392-01A	LGG	7.41
TCGA-FG-A713-01A	LGG	8.84
TCGA-RC-A7SF-01A	LIHC	7.36
TCGA-EP-A2KC-01A	LIHC	8.53
TCGA-ES-A2HS-01A	LIHC	8.35
TCGA-CC-5259-01A	LIHC	8.69
TCGA-ED-A97K-01A	LIHC	7.74
TCGA-DD-A3A1-01A	LIHC	7.26
TCGA-G3-A6UC-01A	LIHC	8.44
TCGA-DD-AACU-01A	LIHC	8.86
TCGA-DD-A1EG-01A	LIHC	7.46

TCGA-DD-A4NJ-01A	LIHC	7.26
TCGA-ZS-A9CF-02A	LIHC	8.01
TCGA-BD-A3ER-01A	LIHC	8.42
TCGA-G3-A5SL-01A	LIHC	8.85
TCGA-DD-A73F-01A	LIHC	8.43
TCGA-CC-A3M9-01A	LIHC	7.09
TCGA-ED-A627-01A	LIHC	8.52
TCGA-CC-A8HT-01A	LIHC	6.69
TCGA-UB-A7MF-01A	LIHC	7.74
TCGA-2Y-A9H1-01A	LIHC	7.69
TCGA-DD-AADO-01A	LIHC	9.42
TCGA-ZP-A9CV-01A	LIHC	8.17
TCGA-4R-AA8I-01A	LIHC	7.57
TCGA-DD-A113-01A	LIHC	7.17
TCGA-DD-AADL-01A	LIHC	7.52
TCGA-RC-A7SK-01A	LIHC	7.29
TCGA-DD-AAEA-01A	LIHC	7.77
TCGA-FV-A495-01A	LIHC	7.74
TCGA-EP-A26S-01A	LIHC	9.14
TCGA-DD-AAEG-01A	LIHC	7.68
TCGA-G3-A25S-01A	LIHC	7.75
TCGA-G3-A3CJ-01A	LIHC	7.52
TCGA-G3-A3CI-01A	LIHC	7.67
TCGA-2Y-A9H2-01A	LIHC	6.33
TCGA-DD-A4NG-01A	LIHC	8.65
TCGA-K7-A6G5-01A	LIHC	8.62
TCGA-DD-AADG-01A	LIHC	8.3
TCGA-LG-A9QC-01A	LIHC	9.01
TCGA-DD-AACJ-01A	LIHC	7.32
TCGA-DD-A1EA-01A	LIHC	8.27
TCGA-LG-A9QD-01A	LIHC	8.07
TCGA-ED-A7PY-01A	LIHC	8.61
TCGA-NI-A4U2-01A	LIHC	6.13
TCGA-DD-A1EK-01A	LIHC	8.07
TCGA-DD-AADP-01A	LIHC	7.9
TCGA-DD-AAVS-01A	LIHC	9.19
TCGA-ED-A82E-01A	LIHC	6.74
TCGA-CC-A7II-01A	LIHC	6.33
TCGA-EP-A3JL-01A	LIHC	7.47
TCGA-DD-A3A4-01A	LIHC	7.78
TCGA-DD-AACP-01A	LIHC	9.8
TCGA-2Y-A9H6-01A	LIHC	8.14
TCGA-UB-A7MB-01A	LIHC	7.26
TCGA-UB-A7MD-01A	LIHC	7.49
TCGA-CC-5260-01A	LIHC	6.56
TCGA-DD-A1EH-01A	LIHC	9.49
TCGA-ED-A4XI-01A	LIHC	8.66
TCGA-DD-AADR-01A	LIHC	7.41
TCGA-FV-A496-01A	LIHC	7.21
TCGA-G3-A3CG-01A	LIHC	7.92
TCGA-DD-AACS-01A	LIHC	8.63
TCGA-DD-AAVR-01A	LIHC	8.28
TCGA-BC-A10T-01A	LIHC	7.59
TCGA-3K-AAZ8-01A	LIHC	8.54
TCGA-DD-AAVV-01A	LIHC	7.54
TCGA-2Y-A9GS-01A	LIHC	7.54
TCGA-RC-A6M6-01A	LIHC	7.18
TCGA-G3-AAV6-01A	LIHC	9.64

TCGA-CC-5263-01A	LIHC	6.3
TCGA-ZS-A9CE-01A	LIHC	7.76
TCGA-DD-A4ND-01A	LIHC	9.02
TCGA-DD-AAVX-01A	LIHC	9.02
TCGA-DD-A4NE-01A	LIHC	7.6
TCGA-2Y-A9HB-01A	LIHC	7.95
TCGA-CC-A9FV-01A	LIHC	6.77
TCGA-DD-A39Y-01A	LIHC	6.48
TCGA-CC-A5UC-01A	LIHC	9.88
TCGA-DD-AACB-01A	LIHC	10.04
TCGA-HP-A5N0-01A	LIHC	7.03
TCGA-ED-A8O6-01A	LIHC	9.51
TCGA-ZS-A9CF-01A	LIHC	8.31
TCGA-2Y-A9H4-01A	LIHC	7.31
TCGA-DD-A116-01A	LIHC	8.96
TCGA-G3-A7M5-01A	LIHC	7.59
TCGA-BC-A112-01A	LIHC	6.8
TCGA-DD-A4NV-01A	LIHC	8.6
TCGA-DD-AAE6-01A	LIHC	8.29
TCGA-CC-A7IL-01A	LIHC	7.75
TCGA-5C-AAPD-01A	LIHC	5.85
TCGA-DD-AAC9-01A	LIHC	7.62
TCGA-MI-A75C-01A	LIHC	7.91
TCGA-FV-A23B-01A	LIHC	9.46
TCGA-DD-AAVY-01A	LIHC	8.3
TCGA-DD-A3A6-01A	LIHC	5.89
TCGA-DD-AADD-01A	LIHC	10.66
TCGA-G3-A25U-01A	LIHC	6.43
TCGA-DD-AAE2-01A	LIHC	8
TCGA-DD-AAW3-01A	LIHC	8.72
TCGA-BW-A5NP-01A	LIHC	9.75
TCGA-DD-AA3A-01A	LIHC	6.51
TCGA-XR-A8TD-01A	LIHC	7.6
TCGA-BC-A10S-01A	LIHC	8
TCGA-T1-A6J8-01A	LIHC	7.68
TCGA-KR-A7K0-01A	LIHC	8.61
TCGA-DD-A4NK-01A	LIHC	7.89
TCGA-DD-A3A9-01A	LIHC	8.85
TCGA-K7-A5RG-01A	LIHC	7.73
TCGA-DD-A4NR-01A	LIHC	7.52
TCGA-FV-A4ZP-01A	LIHC	6.68
TCGA-CC-A9FU-01A	LIHC	9.16
TCGA-DD-AACW-01A	LIHC	10.44
TCGA-DD-A1EC-01A	LIHC	7.58
TCGA-WJ-A86L-01A	LIHC	7.45
TCGA-DD-AAEK-01A	LIHC	9.94
TCGA-FV-A4ZQ-01A	LIHC	11.13
TCGA-5C-A9VH-01A	LIHC	8.67
TCGA-DD-AACA-02B	LIHC	8.92
TCGA-DD-AACA-02A	LIHC	9.05
TCGA-DD-AACA-01A	LIHC	9.3
TCGA-FV-A2QQ-01A	LIHC	8.01
TCGA-2Y-A9GX-01A	LIHC	8.16
TCGA-G3-AAUZ-01A	LIHC	6.78
TCGA-DD-AACK-01A	LIHC	9.75
TCGA-DD-A3A5-01A	LIHC	7.17
TCGA-DD-AACY-01A	LIHC	7.46
TCGA-BD-A3EP-01A	LIHC	6.9

TCGA-BC-A10Q-01A	LIHC	6.78
TCGA-CC-A7IF-01A	LIHC	6.54
TCGA-DD-AADY-01A	LIHC	7.28
TCGA-DD-AACI-01A	LIHC	6.56
TCGA-CC-A7IH-01A	LIHC	9.49
TCGA-UB-AA0V-01A	LIHC	9.06
TCGA-DD-AAEB-01A	LIHC	8.92
TCGA-DD-AAEH-01A	LIHC	8.19
TCGA-CC-A8HS-01A	LIHC	8.7
TCGA-MR-A8JO-01A	LIHC	6.1
TCGA-DD-AACV-01A	LIHC	6.86
TCGA-ED-A7PZ-01A	LIHC	7.76
TCGA-EP-A12J-01A	LIHC	10.12
TCGA-2Y-A9H3-01A	LIHC	6.7
TCGA-FV-A3I0-01A	LIHC	6.55
TCGA-DD-AADI-01A	LIHC	8.41
TCGA-DD-A4NS-01A	LIHC	8.36
TCGA-2Y-A9HA-01A	LIHC	9.22
TCGA-DD-AACG-01A	LIHC	9.38
TCGA-DD-A73A-01A	LIHC	8.11
TCGA-KR-A7K7-01A	LIHC	9.03
TCGA-QA-A7B7-01A	LIHC	8.62
TCGA-BC-A110-01A	LIHC	8.07
TCGA-UB-A7ME-01A	LIHC	7.22
TCGA-G3-AAV2-01A	LIHC	9.1
TCGA-DD-A39X-01A	LIHC	7.63
TCGA-DD-AAD3-01A	LIHC	7.35
TCGA-FV-A3R2-01A	LIHC	8.33
TCGA-2Y-A9H7-01A	LIHC	9.44
TCGA-ED-A7XO-01A	LIHC	6.86
TCGA-DD-AAVZ-01A	LIHC	9.55
TCGA-ZP-A9CZ-01A	LIHC	7.69
TCGA-G3-A5SI-01A	LIHC	6.15
TCGA-MR-A520-01A	LIHC	8.2
TCGA-BC-A10U-01A	LIHC	7.72
TCGA-G3-A25Z-01A	LIHC	7.38
TCGA-DD-A39V-01A	LIHC	8.54
TCGA-DD-A4NA-01A	LIHC	7.35
TCGA-FV-A3I1-01A	LIHC	8.69
TCGA-DD-A4NL-01A	LIHC	9
TCGA-DD-A4NQ-01A	LIHC	7.16
TCGA-CC-A7IJ-01A	LIHC	7.53
TCGA-LG-A6GG-01A	LIHC	8.11
TCGA-CC-A123-01A	LIHC	9.61
TCGA-DD-AAE4-01A	LIHC	7.78
TCGA-GJ-A3OU-01A	LIHC	8.57
TCGA-EP-A3RK-01A	LIHC	8.22
TCGA-MI-A75H-01A	LIHC	8.47
TCGA-FV-A2QR-01A	LIHC	7.2
TCGA-ED-A5KG-01A	LIHC	8.3
TCGA-CC-A9FS-01A	LIHC	8.38
TCGA-G3-A25T-01A	LIHC	6.72
TCGA-UB-A7MC-01A	LIHC	8.25
TCGA-YA-A8S7-01A	LIHC	6.71
TCGA-DD-AACN-01A	LIHC	9.79
TCGA-5C-A9VG-01A	LIHC	10.94
TCGA-BC-A10R-01A	LIHC	6.66
TCGA-BC-A3KF-01A	LIHC	8.7

TCGA-CC-A5UE-01A	LIHC	7.82
TCGA-MI-A75E-01A	LIHC	7.62
TCGA-G3-A7M8-01A	LIHC	7.17
TCGA-CC-A5UD-01A	LIHC	8.63
TCGA-CC-A9FW-01A	LIHC	10.59
TCGA-BC-A10W-01A	LIHC	7.38
TCGA-2Y-A9GW-01A	LIHC	7.12
TCGA-WX-AA47-01A	LIHC	7.03
TCGA-DD-AADC-01A	LIHC	9.3
TCGA-DD-AADQ-01A	LIHC	8.27
TCGA-DD-AAE9-01A	LIHC	8.56
TCGA-ZP-A9D1-01A	LIHC	8.13
TCGA-G3-AAV5-01A	LIHC	7.85
TCGA-2Y-A9GZ-01A	LIHC	7.85
TCGA-RG-A7D4-01A	LIHC	9.51
TCGA-EP-A2KB-01A	LIHC	11.07
TCGA-DD-AAW0-01A	LIHC	8.14
TCGA-DD-AACQ-01A	LIHC	7.1
TCGA-CC-A8HU-01A	LIHC	7.81
TCGA-MI-A75G-01A	LIHC	8.39
TCGA-DD-AAD1-01A	LIHC	8.4
TCGA-CC-A8HV-01A	LIHC	6.91
TCGA-DD-A1EJ-01A	LIHC	10.11
TCGA-WQ-A9G7-01A	LIHC	7.8
TCGA-NI-A8LF-01A	LIHC	9.21
TCGA-BC-4072-01B	LIHC	7.23
TCGA-DD-AAD5-01A	LIHC	6.5
TCGA-2Y-A9H5-01A	LIHC	9.08
TCGA-BC-A217-01A	LIHC	7.14
TCGA-DD-A3A8-01A	LIHC	9.25
TCGA-CC-5258-01A	LIHC	8.77
TCGA-BC-A5W4-01A	LIHC	6.57
TCGA-DD-AADF-01A	LIHC	7.49
TCGA-DD-AAE1-01A	LIHC	9.66
TCGA-2V-A95S-01A	LIHC	6.6
TCGA-DD-A73D-01A	LIHC	7.57
TCGA-ZP-A9D0-01A	LIHC	8.22
TCGA-G3-A5SK-01A	LIHC	7.44
TCGA-DD-AADN-01A	LIHC	7.11
TCGA-CC-A3MC-01A	LIHC	6.86
TCGA-DD-AADS-01A	LIHC	7.36
TCGA-DD-AACX-01A	LIHC	8.53
TCGA-5R-AAAM-01A	LIHC	7.7
TCGA-EP-A2KA-01A	LIHC	7.45
TCGA-XR-A8TC-01A	LIHC	9.46
TCGA-DD-A39W-01A	LIHC	11.12
TCGA-G3-A3CH-01A	LIHC	7.82
TCGA-DD-AACO-01A	LIHC	7.36
TCGA-K7-A5RF-01A	LIHC	8.35
TCGA-DD-A4NP-01A	LIHC	9.89
TCGA-ZP-A9CY-01A	LIHC	8.1
TCGA-BC-4073-01B	LIHC	7.13
TCGA-DD-AAEE-01A	LIHC	9.62
TCGA-CC-A3MB-01A	LIHC	7.5
TCGA-RC-A7SH-01A	LIHC	7.49
TCGA-ZP-A9D4-01A	LIHC	7.97
TCGA-ED-A66Y-01A	LIHC	8.05
TCGA-DD-A114-01A	LIHC	7.25

TCGA-5R-AA1C-01A	LIHC	7.77
TCGA-BC-A10X-01A	LIHC	9.52
TCGA-DD-AACT-01A	LIHC	8.31
TCGA-DD-AAE7-01A	LIHC	7.94
TCGA-G3-A3CK-01A	LIHC	8.66
TCGA-BC-A216-01A	LIHC	7.46
TCGA-DD-A39Z-01A	LIHC	7.69
TCGA-DD-AADV-01A	LIHC	8.3
TCGA-DD-AADK-01A	LIHC	8.05
TCGA-2Y-A9H0-01A	LIHC	7.91
TCGA-DD-AAC8-01A	LIHC	8.43
TCGA-CC-A7IG-01A	LIHC	6.9
TCGA-KR-A7K8-01A	LIHC	7.39
TCGA-CC-5264-01A	LIHC	7.98
TCGA-GJ-A9DB-01A	LIHC	7.97
TCGA-DD-AADM-01A	LIHC	7.94
TCGA-CC-A1HT-01A	LIHC	6.83
TCGA-G3-AAV0-01A	LIHC	6.86
TCGA-DD-A1EE-01A	LIHC	7.83
TCGA-DD-A3A7-01A	LIHC	6.59
TCGA-DD-AAED-01A	LIHC	7.96
TCGA-DD-AACF-01A	LIHC	5.98
TCGA-2Y-A9GY-01A	LIHC	7.27
TCGA-DD-A4NO-01A	LIHC	8.15
TCGA-DD-A118-01A	LIHC	6.83
TCGA-O8-A75V-01A	LIHC	7.52
TCGA-DD-AAVQ-01A	LIHC	8.7
TCGA-BC-A69H-01A	LIHC	8.43
TCGA-DD-A73C-01A	LIHC	8
TCGA-ZS-A9CD-01A	LIHC	8.16
TCGA-UB-AA0U-01A	LIHC	8.74
TCGA-DD-AACE-01A	LIHC	7.22
TCGA-ED-A66X-01A	LIHC	10.29
TCGA-BW-A5NQ-01A	LIHC	10.85
TCGA-2Y-A9GU-01A	LIHC	7.4
TCGA-DD-AADU-01A	LIHC	8.87
TCGA-DD-A4NN-01A	LIHC	8.74
TCGA-DD-A73E-01A	LIHC	9.23
TCGA-RC-A6M3-01A	LIHC	6.18
TCGA-DD-AADA-01A	LIHC	8.12
TCGA-G3-AAV3-01A	LIHC	8.62
TCGA-CC-A7IK-01A	LIHC	8.98
TCGA-XR-A8TE-01A	LIHC	10.82
TCGA-2Y-A9GV-01A	LIHC	8.23
TCGA-G3-A25V-01A	LIHC	8.19
TCGA-G3-A5SM-01A	LIHC	8.36
TCGA-BW-A5NO-01A	LIHC	7.34
TCGA-G3-AAV1-01A	LIHC	8.65
TCGA-2Y-A9H9-01A	LIHC	6.92
TCGA-RC-A7S9-01A	LIHC	7.24
TCGA-RC-A6M5-01A	LIHC	7.4
TCGA-WQ-AB4B-01A	LIHC	7.78
TCGA-ED-A8O5-01A	LIHC	10.39
TCGA-2Y-A9GT-01A	LIHC	7.57
TCGA-DD-A3A2-01A	LIHC	7.09
TCGA-DD-AAW1-01A	LIHC	9.01
TCGA-G3-A7M7-01A	LIHC	7.2
TCGA-DD-AACL-01A	LIHC	8.08

TCGA-ZS-A9CG-01A	LIHC	9.77
TCGA-ED-A7PX-01A	LIHC	6.37
TCGA-FV-A3R3-01A	LIHC	8.3
TCGA-DD-A4NH-01A	LIHC	7.88
TCGA-DD-A73G-01A	LIHC	7.75
TCGA-DD-AAD6-01A	LIHC	9.37
TCGA-DD-AACD-01A	LIHC	7.73
TCGA-G3-A25X-01A	LIHC	6.15
TCGA-DD-A11D-01A	LIHC	7.49
TCGA-DD-A4NB-01A	LIHC	7.77
TCGA-CC-A7IE-01A	LIHC	9.09
TCGA-RC-A7SB-01A	LIHC	7.44
TCGA-RC-A6M4-01A	LIHC	8.47
TCGA-DD-A4NF-01A	LIHC	9.92
TCGA-G3-A7M9-01A	LIHC	7.93
TCGA-DD-AAVW-01A	LIHC	7.81
TCGA-PD-A5DF-01A	LIHC	7.13
TCGA-BD-A2L6-01A	LIHC	6.84
TCGA-XR-A8TG-01A	LIHC	7.49
TCGA-XR-A8TF-01A	LIHC	8.82
TCGA-G3-A7M6-01A	LIHC	7.79
TCGA-G3-A25Y-01A	LIHC	8.35
TCGA-UB-A7MA-01A	LIHC	8.03
TCGA-GJ-A6C0-01A	LIHC	8.48
TCGA-DD-AAVP-01A	LIHC	8.88
TCGA-DD-AADW-01A	LIHC	9.22
TCGA-DD-A1EF-01A	LIHC	11.12
TCGA-CC-5261-01A	LIHC	7.54
TCGA-DD-A11B-01A	LIHC	9.65
TCGA-DD-AAD8-01A	LIHC	7.24
TCGA-DD-AAVU-01A	LIHC	7.98
TCGA-CC-5262-01A	LIHC	8.09
TCGA-G3-AAV4-01A	LIHC	9.16
TCGA-DD-AACH-01A	LIHC	6.28
TCGA-DD-AADJ-01A	LIHC	9.05
TCGA-G3-A5SJ-01A	LIHC	6.74
TCGA-K7-AAU7-01A	LIHC	7.04
TCGA-HP-A5MZ-01A	LIHC	8.22
TCGA-DD-A1EL-01A	LIHC	8.96
TCGA-DD-A1EI-01A	LIHC	9.28
TCGA-DD-AAEI-01A	LIHC	7.17
TCGA-2Y-A9H8-01A	LIHC	6.96
TCGA-DD-A11C-01A	LIHC	7.56
TCGA-DD-AAE3-01A	LIHC	6.97
TCGA-WX-AA46-01A	LIHC	8.69
TCGA-DD-AAW2-01A	LIHC	8.34
TCGA-ZP-A9D2-01A	LIHC	7.76
TCGA-KR-A7K2-01A	LIHC	7.52
TCGA-DD-AACZ-01A	LIHC	7.21
TCGA-BC-A8YO-01A	LIHC	7.45
TCGA-DD-A3A3-01A	LIHC	8.1
TCGA-WX-AA44-01A	LIHC	7.99
TCGA-DD-A73B-01A	LIHC	8.56
TCGA-DD-A4NI-01A	LIHC	7.87
TCGA-MI-A75I-01A	LIHC	7.23
TCGA-DD-AAE0-01A	LIHC	7.84
TCGA-DD-AADB-01A	LIHC	9.98
TCGA-ED-A459-01A	LIHC	7.24

TCGA-BC-A10Y-01A	LIHC	5.72
TCGA-DD-A1EB-01A	LIHC	6.99
TCGA-DD-A11A-01A	LIHC	8.17
TCGA-BC-A3KG-01A	LIHC	6.64
TCGA-DD-A119-01A	LIHC	7.05
TCGA-DD-A115-01A	LIHC	6.68
TCGA-G3-AAV7-01A	LIHC	7.75
TCGA-BC-A10Z-01A	LIHC	8.69
TCGA-BC-A69I-01A	LIHC	9.22
TCGA-CC-A3MA-01A	LIHC	6.52
TCGA-ES-A2HT-01A	LIHC	7.74
TCGA-DD-AAD2-01A	LIHC	8.76
TCGA-DD-AAD0-01A	LIHC	7.97
TCGA-DD-A1ED-01A	LIHC	9.35
TCGA-ED-A7XP-01A	LIHC	7.76
TCGA-DD-AACC-01A	LIHC	8.37
TCGA-5R-AA1D-01A	LIHC	7.21
TCGA-69-7980-01A	LUAD	6.6
TCGA-55-1592-01A	LUAD	6.18
TCGA-55-7727-01A	LUAD	7.1
TCGA-55-6972-01A	LUAD	7.38
TCGA-49-AARE-01A	LUAD	6.48
TCGA-86-7713-01A	LUAD	6.93
TCGA-55-7913-01B	LUAD	7.4
TCGA-44-A47G-01A	LUAD	6.37
TCGA-44-2662-01B	LUAD	6.71
TCGA-44-5645-01A	LUAD	7
TCGA-95-A4VK-01A	LUAD	6.35
TCGA-62-A46O-01A	LUAD	6.36
TCGA-62-A46S-01A	LUAD	7.11
TCGA-05-4434-01A	LUAD	7.25
TCGA-53-7626-01A	LUAD	6.98
TCGA-55-8087-01A	LUAD	7.45
TCGA-86-8668-01A	LUAD	6.4
TCGA-55-A493-01A	LUAD	7.09
TCGA-55-8616-01A	LUAD	7.23
TCGA-55-7570-01A	LUAD	6.45
TCGA-64-1678-01A	LUAD	6.5
TCGA-05-4250-01A	LUAD	7.49
TCGA-86-8278-01A	LUAD	6.32
TCGA-75-6203-01A	LUAD	6.73
TCGA-05-4433-01A	LUAD	7.51
TCGA-86-8673-01A	LUAD	6.01
TCGA-NJ-A55A-01A	LUAD	6.75
TCGA-55-8097-01A	LUAD	6.69
TCGA-73-4668-01A	LUAD	6.97
TCGA-MP-A4TE-01A	LUAD	5.71
TCGA-86-8671-01A	LUAD	6.53
TCGA-55-8508-01A	LUAD	5.11
TCGA-44-2659-01A	LUAD	6.37
TCGA-50-5055-01A	LUAD	6.85
TCGA-78-7153-01A	LUAD	6.78
TCGA-05-4415-01A	LUAD	7.02
TCGA-78-7154-01A	LUAD	7.54
TCGA-55-8507-01A	LUAD	6.38
TCGA-55-8091-01A	LUAD	6.58
TCGA-50-5946-01A	LUAD	7.21
TCGA-75-6206-01A	LUAD	7.26

TCGA-78-7536-01A	LUAD	6.97
TCGA-38-4627-01A	LUAD	7.31
TCGA-91-6831-01A	LUAD	6.61
TCGA-55-1595-01A	LUAD	6.51
TCGA-44-5645-01B	LUAD	6.64
TCGA-62-A472-01A	LUAD	7.27
TCGA-67-6215-01A	LUAD	6.68
TCGA-55-6985-01A	LUAD	6.63
TCGA-55-7815-01A	LUAD	7.05
TCGA-78-7148-01A	LUAD	7.16
TCGA-97-A4LX-01A	LUAD	6.26
TCGA-50-5936-01A	LUAD	7.27
TCGA-05-4432-01A	LUAD	6.7
TCGA-55-6984-01A	LUAD	6.91
TCGA-78-7167-01A	LUAD	7.22
TCGA-44-2657-01A	LUAD	6.02
TCGA-49-4510-01A	LUAD	6.47
TCGA-44-7671-01A	LUAD	7.77
TCGA-97-8175-01A	LUAD	6.09
TCGA-69-8254-01A	LUAD	7
TCGA-55-8505-01A	LUAD	6.3
TCGA-62-A46V-01A	LUAD	6.32
TCGA-38-4630-01A	LUAD	6.1
TCGA-44-2665-01B	LUAD	6.83
TCGA-44-2665-01A	LUAD	7.25
TCGA-4B-A93V-01A	LUAD	5.92
TCGA-44-A4SS-01A	LUAD	6.67
TCGA-97-7937-01A	LUAD	6.52
TCGA-64-5774-01A	LUAD	5.94
TCGA-50-5044-01A	LUAD	6.57
TCGA-78-7220-01A	LUAD	6.96
TCGA-L9-A743-01A	LUAD	6.45
TCGA-64-5815-01A	LUAD	6.67
TCGA-55-7574-01A	LUAD	6.33
TCGA-97-8552-01A	LUAD	6.39
TCGA-44-7659-01A	LUAD	6.4
TCGA-93-8067-01A	LUAD	6.74
TCGA-95-7944-01A	LUAD	6.19
TCGA-49-4506-01A	LUAD	6.46
TCGA-55-7995-01A	LUAD	6.71
TCGA-97-8547-01A	LUAD	6.28
TCGA-55-7283-01A	LUAD	7.37
TCGA-44-7661-01A	LUAD	7.56
TCGA-95-7043-01A	LUAD	6.71
TCGA-50-8457-01A	LUAD	6.02
TCGA-MN-A4N4-01A	LUAD	7.17
TCGA-91-6840-01A	LUAD	6.83
TCGA-50-5946-02A	LUAD	7.04
TCGA-80-5611-01A	LUAD	6.93
TCGA-99-AA5R-01A	LUAD	6.4
TCGA-86-6562-01A	LUAD	7.43
TCGA-86-A4JF-01A	LUAD	6.39
TCGA-67-3770-01A	LUAD	6.85
TCGA-62-A470-01A	LUAD	7.03
TCGA-78-7542-01A	LUAD	7.53
TCGA-93-7347-01A	LUAD	6.74
TCGA-91-A4BC-01A	LUAD	5.65
TCGA-44-3398-01A	LUAD	6.7

TCGA-38-6178-01A	LUAD	6.99
TCGA-05-4405-01A	LUAD	7.09
TCGA-49-AAR2-01A	LUAD	5.76
TCGA-78-7155-01A	LUAD	7.82
TCGA-73-4658-01A	LUAD	7.15
TCGA-69-7765-01A	LUAD	7.22
TCGA-49-4487-01A	LUAD	6.17
TCGA-97-A4M1-01A	LUAD	6.12
TCGA-44-2668-01B	LUAD	6.95
TCGA-86-8672-01A	LUAD	6.95
TCGA-91-7771-01A	LUAD	7.28
TCGA-73-4659-01A	LUAD	6.88
TCGA-97-A4M7-01A	LUAD	6.21
TCGA-44-2668-01A	LUAD	7.68
TCGA-86-8073-01A	LUAD	7.51
TCGA-64-5775-01A	LUAD	7.9
TCGA-05-4410-01A	LUAD	6.36
TCGA-44-2662-01A	LUAD	6.3
TCGA-50-6593-01A	LUAD	7.54
TCGA-97-A4M0-01A	LUAD	7.03
TCGA-55-7728-01A	LUAD	6.73
TCGA-44-2666-01B	LUAD	6.55
TCGA-J2-A4AE-01A	LUAD	6.6
TCGA-95-7947-01A	LUAD	7.17
TCGA-55-6543-01A	LUAD	7.2
TCGA-MP-A4TI-01A	LUAD	7.2
TCGA-78-7146-01A	LUAD	7.78
TCGA-95-7039-01A	LUAD	7.91
TCGA-50-6673-01A	LUAD	6.96
TCGA-55-8301-01A	LUAD	6.39
TCGA-93-A4JN-01A	LUAD	6.47
TCGA-86-7701-01A	LUAD	6.51
TCGA-55-A490-01A	LUAD	5.11
TCGA-05-4390-01A	LUAD	6.41
TCGA-44-6779-01A	LUAD	7.91
TCGA-97-8172-01A	LUAD	7.11
TCGA-64-1681-01A	LUAD	6.48
TCGA-78-7160-01A	LUAD	7.72
TCGA-44-A4SU-01A	LUAD	5.63
TCGA-38-4628-01A	LUAD	7.33
TCGA-55-6969-01A	LUAD	6.09
TCGA-44-2666-01A	LUAD	6.73
TCGA-75-7030-01A	LUAD	7.01
TCGA-50-5051-01A	LUAD	6.48
TCGA-J2-A4AG-01A	LUAD	7.09
TCGA-97-7938-01A	LUAD	7.05
TCGA-35-3615-01A	LUAD	6.89
TCGA-44-2661-01A	LUAD	6.38
TCGA-50-5941-01A	LUAD	6.81
TCGA-64-1676-01A	LUAD	7.17
TCGA-50-5932-01A	LUAD	7.36
TCGA-55-8207-01A	LUAD	6.25
TCGA-L9-A50W-01A	LUAD	6.75
TCGA-97-7941-01A	LUAD	7.3
TCGA-97-7554-01A	LUAD	7.46
TCGA-55-7910-01A	LUAD	6.39
TCGA-50-8459-01A	LUAD	6.77
TCGA-44-7662-01A	LUAD	6.47

TCGA-78-7166-01A	LUAD	5.63
TCGA-55-8513-01A	LUAD	6.3
TCGA-49-4514-01A	LUAD	6.85
TCGA-78-7152-01A	LUAD	7.26
TCGA-67-6216-01A	LUAD	6.75
TCGA-55-7907-01A	LUAD	7.31
TCGA-44-6776-01A	LUAD	7.63
TCGA-49-4494-01A	LUAD	6.51
TCGA-05-4403-01A	LUAD	7.99
TCGA-75-5147-01A	LUAD	6.78
TCGA-62-A46R-01A	LUAD	6.92
TCGA-05-4418-01A	LUAD	6.62
TCGA-49-4488-01A	LUAD	7.64
TCGA-69-7973-01A	LUAD	6.74
TCGA-86-8280-01A	LUAD	6.44
TCGA-05-5715-01A	LUAD	6.69
TCGA-93-7348-01A	LUAD	6.31
TCGA-78-7147-01A	LUAD	6.36
TCGA-69-7978-01A	LUAD	6.79
TCGA-55-6712-01A	LUAD	8.37
TCGA-MP-A4TK-01A	LUAD	6.62
TCGA-44-2656-01A	LUAD	7.5
TCGA-75-7031-01A	LUAD	6.05
TCGA-78-7158-01A	LUAD	7.24
TCGA-49-4507-01A	LUAD	6.55
TCGA-55-8092-01A	LUAD	6.34
TCGA-NJ-A4YQ-01A	LUAD	5.76
TCGA-91-8499-01A	LUAD	6.93
TCGA-91-8497-01A	LUAD	6.53
TCGA-49-6761-01A	LUAD	7.01
TCGA-53-7624-01A	LUAD	7.79
TCGA-44-3919-01A	LUAD	6.42
TCGA-86-8281-01A	LUAD	7.04
TCGA-67-3774-01A	LUAD	7.08
TCGA-05-4426-01A	LUAD	7.02
TCGA-MP-A4TD-01A	LUAD	6.14
TCGA-86-8055-01A	LUAD	6.72
TCGA-05-5428-01A	LUAD	7.43
TCGA-75-6214-01A	LUAD	7.67
TCGA-55-6642-01A	LUAD	5.85
TCGA-69-8253-01A	LUAD	6.93
TCGA-L9-A443-01A	LUAD	5.91
TCGA-49-AAR9-01A	LUAD	6.89
TCGA-91-6848-01A	LUAD	6.94
TCGA-MP-A4SV-01A	LUAD	6.74
TCGA-38-4632-01A	LUAD	7.82
TCGA-05-4395-01A	LUAD	7.86
TCGA-49-AAR4-01A	LUAD	5.92
TCGA-95-8039-01A	LUAD	6.62
TCGA-64-1677-01A	LUAD	7.12
TCGA-86-8075-01A	LUAD	6.7
TCGA-05-4427-01A	LUAD	6.92
TCGA-50-6594-01A	LUAD	6.67
TCGA-53-A4EZ-01A	LUAD	6.78
TCGA-38-7271-01A	LUAD	6.39
TCGA-MP-A4TJ-01A	LUAD	6.19
TCGA-86-8585-01A	LUAD	6.98
TCGA-55-6982-01A	LUAD	6.67

TCGA-05-4398-01A	LUAD	6.98
TCGA-97-A4M6-01A	LUAD	6.7
TCGA-44-6775-01A	LUAD	6.75
TCGA-L4-A4E5-01A	LUAD	6.4
TCGA-49-AAR0-01A	LUAD	5.87
TCGA-35-4122-01A	LUAD	7.63
TCGA-O1-A52J-01A	LUAD	6.17
TCGA-NJ-A7XG-01A	LUAD	6.89
TCGA-86-8054-01A	LUAD	7.09
TCGA-50-5931-01A	LUAD	6.67
TCGA-55-8620-01A	LUAD	6.3
TCGA-69-8255-01A	LUAD	6.67
TCGA-62-A46Y-01A	LUAD	6.76
TCGA-80-5607-01A	LUAD	6.92
TCGA-86-A4P8-01A	LUAD	6.71
TCGA-91-8496-01A	LUAD	7
TCGA-93-A4JQ-01A	LUAD	6.79
TCGA-69-A59K-01A	LUAD	5.69
TCGA-55-7281-01A	LUAD	6.32
TCGA-05-4397-01A	LUAD	6.12
TCGA-49-6767-01A	LUAD	7.04
TCGA-35-4123-01A	LUAD	8.16
TCGA-78-8648-01A	LUAD	5.97
TCGA-62-8394-01A	LUAD	6.22
TCGA-55-8204-01A	LUAD	6.88
TCGA-MP-A4T9-01A	LUAD	6.75
TCGA-MN-A4N1-01A	LUAD	6.67
TCGA-50-5942-01A	LUAD	6.55
TCGA-44-6148-01A	LUAD	6.82
TCGA-55-7903-01A	LUAD	6.22
TCGA-NJ-A55O-01A	LUAD	6.61
TCGA-44-6147-01A	LUAD	6.85
TCGA-78-7539-01A	LUAD	6.32
TCGA-55-6971-01A	LUAD	5.92
TCGA-75-5122-01A	LUAD	7.46
TCGA-62-A46U-01A	LUAD	6.48
TCGA-86-8076-01A	LUAD	6.6
TCGA-55-7573-01A	LUAD	6.5
TCGA-67-3772-01A	LUAD	7.31
TCGA-97-7547-01A	LUAD	7.49
TCGA-44-4112-01A	LUAD	7.23
TCGA-44-3917-01B	LUAD	6.59
TCGA-78-7163-01A	LUAD	7.25
TCGA-55-6970-01A	LUAD	7.26
TCGA-99-8032-01A	LUAD	6.27
TCGA-MP-A4T6-01A	LUAD	6.9
TCGA-55-8085-01A	LUAD	6.09
TCGA-78-7537-01A	LUAD	6.56
TCGA-55-7994-01A	LUAD	7.03
TCGA-55-8094-01A	LUAD	6.4
TCGA-05-4425-01A	LUAD	8.02
TCGA-44-3917-01A	LUAD	5.69
TCGA-55-7914-01A	LUAD	6.59
TCGA-55-8514-01A	LUAD	6.24
TCGA-05-4402-01A	LUAD	6.74
TCGA-69-7761-01A	LUAD	6.95
TCGA-50-5933-01A	LUAD	7.17
TCGA-97-A4M5-01A	LUAD	6.97

TCGA-55-1594-01A	LUAD	6.93
TCGA-44-4112-01B	LUAD	6.43
TCGA-44-8117-01A	LUAD	6.43
TCGA-75-7025-01A	LUAD	6.46
TCGA-44-3918-01B	LUAD	6.25
TCGA-73-7499-01A	LUAD	6.21
TCGA-80-5608-01A	LUAD	6.71
TCGA-MP-A4TH-01A	LUAD	6.47
TCGA-05-5429-01A	LUAD	7.14
TCGA-91-6830-01A	LUAD	7.31
TCGA-55-8619-01A	LUAD	6.3
TCGA-55-6979-01A	LUAD	6.47
TCGA-86-8359-01A	LUAD	6.78
TCGA-95-8494-01A	LUAD	6.94
TCGA-50-6590-01A	LUAD	6.9
TCGA-91-6829-01A	LUAD	7.11
TCGA-55-7576-01A	LUAD	7.25
TCGA-55-8615-01A	LUAD	6.9
TCGA-83-5908-01A	LUAD	7.59
TCGA-95-A4VN-01A	LUAD	6.45
TCGA-55-A492-01A	LUAD	6.63
TCGA-44-7672-01A	LUAD	6.3
TCGA-78-7633-01A	LUAD	7.26
TCGA-05-4430-01A	LUAD	7.08
TCGA-55-A48X-01A	LUAD	6.23
TCGA-NJ-A4YP-01A	LUAD	7.28
TCGA-05-4420-01A	LUAD	6.81
TCGA-44-2656-01B	LUAD	6.97
TCGA-44-6146-01A	LUAD	7.46
TCGA-44-6146-01B	LUAD	7.81
TCGA-62-A46P-01A	LUAD	6.22
TCGA-73-4676-01A	LUAD	7.12
TCGA-55-1596-01A	LUAD	6.86
TCGA-55-8511-01A	LUAD	7.5
TCGA-44-5644-01A	LUAD	6.63
TCGA-49-6742-01A	LUAD	6.64
TCGA-55-8512-01A	LUAD	6.72
TCGA-97-8179-01A	LUAD	6.97
TCGA-95-7562-01A	LUAD	6.33
TCGA-50-5072-01A	LUAD	6.9
TCGA-78-8662-01A	LUAD	7.3
TCGA-75-5125-01A	LUAD	7.1
TCGA-MN-A4N5-01A	LUAD	6.17
TCGA-86-8279-01A	LUAD	6.81
TCGA-55-7724-01A	LUAD	7.24
TCGA-73-4662-01A	LUAD	6.16
TCGA-73-A9RS-01A	LUAD	6.62
TCGA-78-8640-01A	LUAD	6.61
TCGA-NJ-A4YI-01A	LUAD	6.25
TCGA-38-4631-01A	LUAD	6.76
TCGA-50-5930-01A	LUAD	6.24
TCGA-50-5066-02A	LUAD	7.3
TCGA-05-4382-01A	LUAD	6.76
TCGA-L9-A5IP-01A	LUAD	6.31
TCGA-64-5779-01A	LUAD	5.77
TCGA-75-6212-01A	LUAD	5.9
TCGA-55-A4DF-01A	LUAD	6.74
TCGA-44-6774-01A	LUAD	6.66

TCGA-78-7149-01A	LUAD	6.65
TCGA-97-A4M2-01A	LUAD	6.58
TCGA-38-A44F-01A	LUAD	6.76
TCGA-38-4629-01A	LUAD	7.31
TCGA-38-4625-01A	LUAD	7.46
TCGA-50-5939-01A	LUAD	6.9
TCGA-55-A48Y-01A	LUAD	6.15
TCGA-MP-A4SW-01A	LUAD	6.75
TCGA-97-8176-01A	LUAD	6.61
TCGA-55-8089-01A	LUAD	7.04
TCGA-64-1680-01A	LUAD	7.31
TCGA-97-7552-01A	LUAD	7.28
TCGA-73-4670-01A	LUAD	7.15
TCGA-93-A4JP-01A	LUAD	6.81
TCGA-69-7764-01A	LUAD	6.91
TCGA-55-8096-01A	LUAD	6.27
TCGA-55-6987-01A	LUAD	6.04
TCGA-S2-AA1A-01A	LUAD	6.1
TCGA-55-6980-01A	LUAD	6.77
TCGA-J2-8192-01A	LUAD	7.11
TCGA-49-6744-01A	LUAD	6.74
TCGA-75-5146-01A	LUAD	7.55
TCGA-50-5935-01A	LUAD	6.69
TCGA-78-7162-01A	LUAD	7.34
TCGA-55-7911-01A	LUAD	6.13
TCGA-L4-A4E6-01A	LUAD	6.53
TCGA-MP-A4T4-01A	LUAD	6.18
TCGA-64-5781-01A	LUAD	6.42
TCGA-93-A4JO-01A	LUAD	6.42
TCGA-05-4422-01A	LUAD	7.61
TCGA-44-3396-01A	LUAD	6.11
TCGA-55-A4DG-01A	LUAD	6.55
TCGA-75-7027-01A	LUAD	7.44
TCGA-55-8506-01A	LUAD	6.69
TCGA-MP-A4T7-01A	LUAD	7.09
TCGA-86-A4D0-01A	LUAD	6.92
TCGA-86-7953-01A	LUAD	6.59
TCGA-78-7540-01A	LUAD	6.46
TCGA-55-7816-01A	LUAD	7.04
TCGA-78-7159-01A	LUAD	6.27
TCGA-44-6778-01A	LUAD	6.59
TCGA-86-8074-01A	LUAD	6.65
TCGA-69-7974-01A	LUAD	6.99
TCGA-78-7535-01A	LUAD	5.78
TCGA-73-4666-01A	LUAD	8.09
TCGA-50-5068-01A	LUAD	7.64
TCGA-35-5375-01A	LUAD	7.31
TCGA-55-7227-01A	LUAD	7.51
TCGA-L9-A7SV-01A	LUAD	6.45
TCGA-91-6835-01A	LUAD	6.76
TCGA-71-8520-01A	LUAD	6.72
TCGA-44-6777-01A	LUAD	6.68
TCGA-97-7553-01A	LUAD	6.83
TCGA-62-8398-01A	LUAD	7.42
TCGA-53-7813-01A	LUAD	6.03
TCGA-44-6775-01C	LUAD	6.72
TCGA-97-8171-01A	LUAD	5.99
TCGA-62-8402-01A	LUAD	6.76

TCGA-55-A57B-01A	LUAD	6.33
TCGA-67-3773-01A	LUAD	6.62
TCGA-95-7567-01A	LUAD	6.71
TCGA-38-4626-01A	LUAD	6.86
TCGA-J2-8194-01A	LUAD	7.11
TCGA-50-5944-01A	LUAD	6.64
TCGA-75-6211-01A	LUAD	6.26
TCGA-50-5066-01A	LUAD	7.3
TCGA-55-6986-01A	LUAD	7.09
TCGA-86-8056-01A	LUAD	7.19
TCGA-73-4677-01A	LUAD	7.15
TCGA-97-A4M3-01A	LUAD	6.86
TCGA-05-4249-01A	LUAD	7.73
TCGA-78-7145-01A	LUAD	7.21
TCGA-55-6978-01A	LUAD	7.79
TCGA-49-AARO-01A	LUAD	6.76
TCGA-67-4679-01B	LUAD	6.57
TCGA-71-6725-01A	LUAD	6.63
TCGA-50-8460-01A	LUAD	6.09
TCGA-86-8358-01A	LUAD	6.14
TCGA-78-7143-01A	LUAD	7.18
TCGA-55-6983-01A	LUAD	6.86
TCGA-49-4486-01A	LUAD	7.25
TCGA-L9-A444-01A	LUAD	5.68
TCGA-86-6851-01A	LUAD	6.06
TCGA-86-7714-01A	LUAD	7.23
TCGA-49-AAR3-01A	LUAD	6.64
TCGA-75-6207-01A	LUAD	7.47
TCGA-44-7669-01A	LUAD	6.59
TCGA-44-6145-01A	LUAD	7.1
TCGA-62-8395-01A	LUAD	6.71
TCGA-05-4424-01A	LUAD	7.22
TCGA-55-8090-01A	LUAD	7.16
TCGA-62-8399-01A	LUAD	6.28
TCGA-NJ-A4YF-01A	LUAD	5.45
TCGA-44-8119-01A	LUAD	6.44
TCGA-55-8299-01A	LUAD	6.45
TCGA-05-5423-01A	LUAD	7.01
TCGA-05-4244-01A	LUAD	6.6
TCGA-95-A4VP-01A	LUAD	5.93
TCGA-86-A4P7-01A	LUAD	6.5
TCGA-50-5045-01A	LUAD	7.17
TCGA-50-5049-01A	LUAD	6.42
TCGA-69-8453-01A	LUAD	7.18
TCGA-05-4396-01A	LUAD	7.87
TCGA-78-7161-01A	LUAD	6.45
TCGA-55-8205-01A	LUAD	8.39
TCGA-55-8203-01A	LUAD	6.27
TCGA-55-A491-01A	LUAD	6.15
TCGA-91-6828-01A	LUAD	6.93
TCGA-55-A494-01A	LUAD	7.13
TCGA-49-AARN-01A	LUAD	6.77
TCGA-55-5899-01A	LUAD	6.57
TCGA-MP-A4TC-01A	LUAD	7.35
TCGA-55-7725-01A	LUAD	7.41
TCGA-05-4384-01A	LUAD	7.12
TCGA-78-7150-01A	LUAD	6.95
TCGA-99-7458-01A	LUAD	7.13

TCGA-49-4512-01A	LUAD	6.24
TCGA-44-7660-01A	LUAD	6.12
TCGA-49-4505-01A	LUAD	6.34
TCGA-50-6597-01A	LUAD	6.24
TCGA-62-A471-01A	LUAD	6.35
TCGA-64-5778-01A	LUAD	7.44
TCGA-55-7726-01A	LUAD	7.58
TCGA-49-AARQ-01A	LUAD	4.91
TCGA-99-8033-01A	LUAD	6.43
TCGA-62-8397-01A	LUAD	5.56
TCGA-50-6591-01A	LUAD	6.45
TCGA-55-8206-01A	LUAD	6.42
TCGA-49-AARR-01A	LUAD	6.44
TCGA-55-8614-01A	LUAD	6.65
TCGA-99-8028-01A	LUAD	6.19
TCGA-91-6847-01A	LUAD	6.47
TCGA-86-7711-01A	LUAD	6.46
TCGA-55-8208-01A	LUAD	6.04
TCGA-05-5420-01A	LUAD	7.11
TCGA-78-7156-01A	LUAD	7.27
TCGA-97-8174-01A	LUAD	6.7
TCGA-MP-A4SY-01A	LUAD	6.52
TCGA-44-6147-01B	LUAD	6.65
TCGA-55-8621-01A	LUAD	6.59
TCGA-91-6849-01A	LUAD	6.84
TCGA-44-7670-01A	LUAD	6.51
TCGA-MP-A4TF-01A	LUAD	6.05
TCGA-86-8669-01A	LUAD	6.59
TCGA-44-8120-01A	LUAD	6.74
TCGA-99-8025-01A	LUAD	6.82
TCGA-NJ-A55R-01A	LUAD	5.93
TCGA-55-8302-01A	LUAD	7.37
TCGA-64-1679-01A	LUAD	7.31
TCGA-86-A456-01A	LUAD	6.63
TCGA-50-6595-01A	LUAD	7.48
TCGA-78-8660-01A	LUAD	6.78
TCGA-50-7109-01A	LUAD	6.28
TCGA-44-A479-01A	LUAD	6.05
TCGA-75-6205-01A	LUAD	7.78
TCGA-05-5425-01A	LUAD	7.08
TCGA-05-4417-01A	LUAD	6.66
TCGA-49-6743-01A	LUAD	7.09
TCGA-69-7979-01A	LUAD	5.86
TCGA-78-8655-01A	LUAD	6.38
TCGA-05-4389-01A	LUAD	7.08
TCGA-55-6975-01A	LUAD	6.64
TCGA-44-A47B-01A	LUAD	6.74
TCGA-75-5126-01A	LUAD	7.5
TCGA-95-7948-01A	LUAD	7.09
TCGA-49-AAQV-01A	LUAD	6.33
TCGA-55-8510-01A	LUAD	6.32
TCGA-L9-A8F4-01A	LUAD	6.65
TCGA-73-7498-01A	LUAD	6.81
TCGA-49-6745-01A	LUAD	7.53
TCGA-73-4675-01A	LUAD	7.32
TCGA-44-3918-01A	LUAD	6.31
TCGA-49-4490-01A	LUAD	7.22
TCGA-55-6968-01A	LUAD	5.92

TCGA-44-A47A-01A	LUAD	6.74
TCGA-44-7667-01A	LUAD	6.67
TCGA-NJ-A4YG-01A	LUAD	6.6
TCGA-MP-A4T8-01A	LUAD	7
TCGA-69-7760-01A	LUAD	5.88
TCGA-MP-A5C7-01A	LUAD	7.18
TCGA-MP-A4TA-01A	LUAD	6.98
TCGA-49-4501-01A	LUAD	7.02
TCGA-55-6981-01A	LUAD	6.27
TCGA-44-2655-01A	LUAD	7.43
TCGA-91-A4BD-01A	LUAD	5.9
TCGA-97-8177-01A	LUAD	6.73
TCGA-67-6217-01A	LUAD	6.26
TCGA-55-7284-01B	LUAD	7.33
TCGA-86-7955-01A	LUAD	6.31
TCGA-91-6836-01A	LUAD	6.85
TCGA-67-3771-01A	LUAD	6.75
TCGA-50-6592-01A	LUAD	6.43
TCGA-86-8674-01A	LUAD	6.19
TCGA-86-7954-01A	LUAD	7.03
TCGA-J2-A4AD-01A	LUAD	6.41
TCGA-97-7546-01A	LUAD	6.97
TCGA-55-A48Z-01A	LUAD	6.37
TCGA-69-7763-01A	LUAD	7.06
TCGA-44-5643-01A	LUAD	8.04
TCGA-92-7340-01A	LUSC	6.84
TCGA-O2-A52Q-01A	LUSC	6.98
TCGA-85-8479-01A	LUSC	6.6
TCGA-22-4594-01A	LUSC	6.34
TCGA-63-A5MB-01A	LUSC	6.62
TCGA-21-5783-01A	LUSC	6.62
TCGA-18-4083-01A	LUSC	6.87
TCGA-85-A53L-01A	LUSC	6.2
TCGA-77-8148-01A	LUSC	7.17
TCGA-77-A5G7-01B	LUSC	6.54
TCGA-22-4593-01A	LUSC	7.37
TCGA-68-8250-01A	LUSC	7.07
TCGA-77-8150-01A	LUSC	6.99
TCGA-66-2793-01A	LUSC	6.61
TCGA-33-A4WN-01A	LUSC	8.05
TCGA-34-8456-01A	LUSC	7.11
TCGA-77-A5FZ-01A	LUSC	6.26
TCGA-34-2596-01A	LUSC	6.99
TCGA-O2-A5IB-01A	LUSC	5.52
TCGA-66-2773-01A	LUSC	7.43
TCGA-43-5670-01A	LUSC	6.99
TCGA-NC-A5HK-01A	LUSC	6.83
TCGA-66-2789-01A	LUSC	6.79
TCGA-85-A50Z-01A	LUSC	4.63
TCGA-22-1017-01A	LUSC	7.84
TCGA-85-A4JB-01A	LUSC	6.07
TCGA-33-AASB-01A	LUSC	5.95
TCGA-NK-A5CR-01A	LUSC	6.73
TCGA-34-5234-01A	LUSC	7.26
TCGA-85-6175-01A	LUSC	6.88
TCGA-77-7338-01A	LUSC	7.48
TCGA-NK-A7XE-01A	LUSC	7.67
TCGA-63-A5MY-01A	LUSC	6.94

TCGA-56-7222-01A	LUSC	7.81
TCGA-33-4586-01A	LUSC	6.49
TCGA-51-4079-01A	LUSC	7.18
TCGA-22-1005-01A	LUSC	5.54
TCGA-22-5485-01A	LUSC	6.62
TCGA-60-2704-01A	LUSC	6.65
TCGA-56-8083-01A	LUSC	7.13
TCGA-85-6561-01A	LUSC	6.58
TCGA-66-2737-01A	LUSC	7.48
TCGA-39-5036-01A	LUSC	7.45
TCGA-21-5784-01A	LUSC	6.73
TCGA-85-8287-01A	LUSC	6.28
TCGA-33-4589-01A	LUSC	7.28
TCGA-60-2707-01A	LUSC	7.89
TCGA-63-5131-01A	LUSC	7.15
TCGA-63-A5MH-01A	LUSC	6.17
TCGA-22-4609-01A	LUSC	6.68
TCGA-90-7766-01A	LUSC	7.26
TCGA-98-A53J-01A	LUSC	6.31
TCGA-58-8388-01A	LUSC	6.02
TCGA-34-2608-01A	LUSC	7.03
TCGA-77-8154-01A	LUSC	7.38
TCGA-56-8623-01A	LUSC	6.76
TCGA-77-8140-01A	LUSC	6.97
TCGA-33-4587-01A	LUSC	5.6
TCGA-NC-A5HP-01A	LUSC	7.82
TCGA-60-2709-01A	LUSC	6.68
TCGA-56-1622-01A	LUSC	7.47
TCGA-NC-A5HQ-01A	LUSC	7.04
TCGA-NC-A5HO-01A	LUSC	6.68
TCGA-85-A4JC-01A	LUSC	5.97
TCGA-66-2792-01A	LUSC	7.83
TCGA-63-A5MT-01A	LUSC	7.05
TCGA-34-A5IX-01A	LUSC	3.06
TCGA-43-6647-01A	LUSC	6.73
TCGA-98-8023-01A	LUSC	6.17
TCGA-43-A56U-01A	LUSC	5.44
TCGA-58-8392-01A	LUSC	6.79
TCGA-22-5492-01A	LUSC	7.35
TCGA-22-5478-01A	LUSC	6.72
TCGA-92-7341-01A	LUSC	7.08
TCGA-60-2708-01A	LUSC	7.09
TCGA-68-A59J-01A	LUSC	5.66
TCGA-94-A4VJ-01A	LUSC	6.57
TCGA-60-2720-01A	LUSC	5.85
TCGA-96-8170-01A	LUSC	6.1
TCGA-94-8035-01A	LUSC	6.45
TCGA-39-5029-01A	LUSC	8
TCGA-85-8052-01A	LUSC	6.66
TCGA-60-2697-01A	LUSC	6.2
TCGA-34-5236-01A	LUSC	6.96
TCGA-39-5024-01A	LUSC	7.31
TCGA-51-4081-01A	LUSC	7.68
TCGA-52-7622-01A	LUSC	6.76
TCGA-60-2726-01A	LUSC	7.6
TCGA-21-1076-01A	LUSC	7.29
TCGA-21-1070-01A	LUSC	7.89
TCGA-56-A5DR-01A	LUSC	7.93

TCGA-22-4605-01A	LUSC	7.15
TCGA-63-7023-01A	LUSC	8.2
TCGA-77-8138-01A	LUSC	6.34
TCGA-85-7843-01A	LUSC	8.24
TCGA-34-8455-01A	LUSC	7.56
TCGA-66-2765-01A	LUSC	7.73
TCGA-56-8624-01A	LUSC	6.55
TCGA-43-2578-01A	LUSC	6.22
TCGA-63-7020-01A	LUSC	6.89
TCGA-85-8481-01A	LUSC	7.21
TCGA-43-6770-01A	LUSC	6.4
TCGA-77-7142-01A	LUSC	8.09
TCGA-94-7033-01A	LUSC	6.73
TCGA-18-3421-01A	LUSC	7.66
TCGA-85-8666-01A	LUSC	7.09
TCGA-22-1016-01A	LUSC	7.02
TCGA-22-5480-01A	LUSC	7.01
TCGA-79-5596-01A	LUSC	7.22
TCGA-NC-A5HJ-01A	LUSC	6.64
TCGA-21-1081-01A	LUSC	6.73
TCGA-22-4613-01A	LUSC	6.82
TCGA-18-5592-01A	LUSC	7.03
TCGA-77-8153-01A	LUSC	6.39
TCGA-39-5037-01A	LUSC	6.97
TCGA-63-A5MW-01A	LUSC	6.61
TCGA-56-8201-01A	LUSC	7.44
TCGA-56-7223-01A	LUSC	6.57
TCGA-68-7755-01A	LUSC	7.54
TCGA-18-4086-01A	LUSC	7.76
TCGA-66-2756-01A	LUSC	6.68
TCGA-85-8353-01A	LUSC	6.72
TCGA-96-7545-01A	LUSC	7.62
TCGA-77-8008-01A	LUSC	6.66
TCGA-66-2800-01A	LUSC	7.47
TCGA-NC-A5HF-01A	LUSC	7.17
TCGA-39-5011-01A	LUSC	7.46
TCGA-98-A53C-01A	LUSC	6.25
TCGA-18-5595-01A	LUSC	7.62
TCGA-22-4601-01A	LUSC	7.74
TCGA-22-5491-01A	LUSC	8.07
TCGA-94-A5I4-01A	LUSC	6.71
TCGA-33-4547-01A	LUSC	7.24
TCGA-92-8063-01A	LUSC	7.57
TCGA-33-AASI-01A	LUSC	7.91
TCGA-37-4141-01A	LUSC	7.13
TCGA-34-5239-01A	LUSC	7.29
TCGA-85-8070-01A	LUSC	7.24
TCGA-66-2769-01A	LUSC	7.07
TCGA-NC-A5HL-01A	LUSC	7.69
TCGA-63-A5MV-01A	LUSC	6.84
TCGA-85-A4CL-01A	LUSC	6.85
TCGA-85-A50M-01A	LUSC	7.57
TCGA-60-2716-01A	LUSC	7.46
TCGA-6A-AB49-01A	LUSC	6.79
TCGA-85-A513-01A	LUSC	5.23
TCGA-34-5928-01A	LUSC	7.33
TCGA-90-7769-01A	LUSC	7.14
TCGA-O2-A52V-01A	LUSC	6.69

TCGA-60-2722-01A	LUSC	8.04
TCGA-90-A4ED-01A	LUSC	6.3
TCGA-39-5030-01A	LUSC	5.79
TCGA-85-6560-01A	LUSC	6.81
TCGA-56-A62T-01A	LUSC	6.72
TCGA-51-4080-01A	LUSC	7.87
TCGA-56-8304-01A	LUSC	6.86
TCGA-66-2780-01A	LUSC	7.89
TCGA-85-A4CN-01A	LUSC	6.52
TCGA-O2-A52W-01A	LUSC	6.09
TCGA-56-A49D-01A	LUSC	7.82
TCGA-66-2771-01A	LUSC	7.42
TCGA-85-8288-01A	LUSC	7.02
TCGA-LA-A446-01A	LUSC	6.84
TCGA-56-7221-01A	LUSC	7.24
TCGA-66-2763-01A	LUSC	6.89
TCGA-96-7544-01A	LUSC	7.43
TCGA-77-7465-01A	LUSC	6.98
TCGA-63-A5MI-01A	LUSC	6.28
TCGA-77-8139-01A	LUSC	6.33
TCGA-22-4591-01A	LUSC	8.14
TCGA-37-4132-01A	LUSC	6.46
TCGA-52-7811-01A	LUSC	7.54
TCGA-98-8021-01A	LUSC	6.92
TCGA-70-6723-01A	LUSC	7.36
TCGA-60-2711-01A	LUSC	7.44
TCGA-18-4721-01A	LUSC	7.24
TCGA-56-7579-01A	LUSC	7.22
TCGA-98-A53D-01A	LUSC	6.89
TCGA-56-8629-01A	LUSC	6.14
TCGA-37-A5EM-01A	LUSC	7.23
TCGA-56-8625-01A	LUSC	6.35
TCGA-66-2777-01A	LUSC	7.72
TCGA-56-8628-01A	LUSC	6.4
TCGA-66-2744-01A	LUSC	7.05
TCGA-77-A5G6-01A	LUSC	7.32
TCGA-66-2767-01A	LUSC	6.72
TCGA-39-5016-01A	LUSC	8.19
TCGA-58-8386-01A	LUSC	7.07
TCGA-39-5019-01A	LUSC	7.03
TCGA-68-A59I-01A	LUSC	6.29
TCGA-58-A46L-01A	LUSC	8.01
TCGA-85-7844-01A	LUSC	6.4
TCGA-37-4135-01A	LUSC	6.81
TCGA-LA-A7SW-01A	LUSC	6.49
TCGA-43-8115-01A	LUSC	6.84
TCGA-96-A4JL-01A	LUSC	6.89
TCGA-85-7699-01A	LUSC	6.86
TCGA-21-1082-01A	LUSC	6.58
TCGA-60-2723-01A	LUSC	7.78
TCGA-O2-A52N-01A	LUSC	6.87
TCGA-21-1083-01A	LUSC	7.64
TCGA-18-3419-01A	LUSC	7.58
TCGA-77-A5GF-01A	LUSC	7.29
TCGA-34-8454-01A	LUSC	7.26
TCGA-21-1078-01A	LUSC	7.74
TCGA-22-4604-01A	LUSC	7.51
TCGA-63-A5MG-01A	LUSC	5.78

TCGA-90-6837-01A	LUSC	6.53
TCGA-43-2576-01A	LUSC	6.07
TCGA-56-8082-01A	LUSC	6.92
TCGA-63-7021-01A	LUSC	6.55
TCGA-66-2734-01A	LUSC	6.91
TCGA-22-1011-01A	LUSC	6.22
TCGA-58-8393-01A	LUSC	6.65
TCGA-77-7140-01A	LUSC	3.55
TCGA-85-8352-01A	LUSC	8.79
TCGA-34-5927-01A	LUSC	6.43
TCGA-NC-A5HR-01A	LUSC	6.38
TCGA-85-8354-01A	LUSC	6.69
TCGA-43-3920-01A	LUSC	6.65
TCGA-37-3783-01A	LUSC	7.39
TCGA-56-7730-01A	LUSC	7.24
TCGA-NK-A5D1-01A	LUSC	6.36
TCGA-43-8116-01A	LUSC	7.2
TCGA-63-A5MR-01A	LUSC	7.53
TCGA-37-4129-01A	LUSC	7.42
TCGA-18-3410-01A	LUSC	6.53
TCGA-J1-A4AH-01A	LUSC	7.02
TCGA-33-A5GW-01A	LUSC	6.23
TCGA-77-8156-01A	LUSC	6.85
TCGA-77-8145-01A	LUSC	6.82
TCGA-22-1000-01A	LUSC	6.63
TCGA-77-8144-01A	LUSC	7.37
TCGA-90-A59Q-01A	LUSC	6.42
TCGA-85-8048-01A	LUSC	6.71
TCGA-52-7810-01A	LUSC	7.22
TCGA-66-2782-01A	LUSC	7.27
TCGA-22-5474-01A	LUSC	7.27
TCGA-66-2794-01A	LUSC	7.85
TCGA-77-8130-01A	LUSC	7.04
TCGA-85-8277-01A	LUSC	7.06
TCGA-NC-A5HT-01A	LUSC	6.56
TCGA-18-3406-01A	LUSC	6.58
TCGA-56-8504-01A	LUSC	7.5
TCGA-22-4607-01A	LUSC	7.45
TCGA-37-4133-01A	LUSC	8.28
TCGA-77-7463-01A	LUSC	6.79
TCGA-56-8308-01A	LUSC	6.65
TCGA-18-3407-01A	LUSC	6.73
TCGA-37-3792-01A	LUSC	6.97
TCGA-51-6867-01A	LUSC	7.92
TCGA-63-A5MS-01A	LUSC	6.4
TCGA-77-7141-01A	LUSC	8.07
TCGA-77-8146-01A	LUSC	7.25
TCGA-60-2719-01A	LUSC	7.15
TCGA-90-7964-01A	LUSC	6.62
TCGA-37-5819-01A	LUSC	6.74
TCGA-18-3416-01A	LUSC	7.6
TCGA-33-4532-01A	LUSC	7.72
TCGA-77-A5G8-01B	LUSC	6.74
TCGA-85-8584-01A	LUSC	6.68
TCGA-22-1012-01A	LUSC	7.45
TCGA-21-1072-01A	LUSC	7.61
TCGA-63-A5MJ-01A	LUSC	6.93
TCGA-46-3767-01A	LUSC	7.2

TCGA-58-8390-01A	LUSC	6.9
TCGA-22-A5C4-01A	LUSC	7.51
TCGA-85-A4PA-01A	LUSC	6.23
TCGA-22-0940-01A	LUSC	7.16
TCGA-77-6842-01A	LUSC	6.77
TCGA-52-7812-01A	LUSC	7.38
TCGA-66-2754-01A	LUSC	6.4
TCGA-52-7809-01A	LUSC	6.74
TCGA-85-8580-01A	LUSC	6.73
TCGA-58-8387-01A	LUSC	6.86
TCGA-77-8131-01A	LUSC	6.8
TCGA-63-A5MU-01A	LUSC	7.53
TCGA-56-5898-01A	LUSC	6.88
TCGA-66-2727-01A	LUSC	7.18
TCGA-85-6798-01A	LUSC	6.67
TCGA-46-6025-01A	LUSC	6.11
TCGA-94-8490-01A	LUSC	7.68
TCGA-60-2714-01A	LUSC	7.37
TCGA-34-5241-01A	LUSC	8.33
TCGA-NK-A5CX-01A	LUSC	7.23
TCGA-56-7823-01B	LUSC	6.77
TCGA-94-A5I6-01A	LUSC	6.56
TCGA-96-A4JK-01A	LUSC	6.48
TCGA-66-2742-01A	LUSC	7.37
TCGA-43-6773-01A	LUSC	6.08
TCGA-60-2724-01A	LUSC	7.95
TCGA-46-3768-01A	LUSC	7.33
TCGA-22-1002-01A	LUSC	7.43
TCGA-85-7698-01A	LUSC	7.15
TCGA-90-7767-01A	LUSC	7.19
TCGA-56-8503-01A	LUSC	6.21
TCGA-43-7656-01A	LUSC	6.9
TCGA-37-A5EL-01A	LUSC	7.49
TCGA-21-1079-01A	LUSC	7.04
TCGA-85-8350-01A	LUSC	6.92
TCGA-94-7943-01A	LUSC	7.42
TCGA-NC-A5HH-01A	LUSC	7.48
TCGA-43-A474-01A	LUSC	7.65
TCGA-39-5034-01A	LUSC	6.85
TCGA-77-8007-01A	LUSC	7.83
TCGA-34-7107-01A	LUSC	7.51
TCGA-98-A539-01A	LUSC	6.44
TCGA-85-8351-01A	LUSC	7.2
TCGA-56-A4ZJ-01A	LUSC	6.89
TCGA-66-2753-01A	LUSC	7.48
TCGA-60-2696-01A	LUSC	8.21
TCGA-56-8305-01A	LUSC	6.73
TCGA-22-5482-01A	LUSC	7.96
TCGA-66-2781-01A	LUSC	7.59
TCGA-39-5027-01A	LUSC	6.72
TCGA-NC-A5HM-01A	LUSC	6.92
TCGA-63-6202-01A	LUSC	6.79
TCGA-60-2713-01A	LUSC	7.16
TCGA-O2-A52S-01A	LUSC	7.17
TCGA-77-7138-01A	LUSC	7.3
TCGA-43-7657-01A	LUSC	7.47
TCGA-22-5489-01A	LUSC	5.84
TCGA-66-2759-01A	LUSC	7.09

TCGA-92-8064-01A	LUSC	6.81
TCGA-98-A53H-01A	LUSC	6.62
TCGA-34-5929-01A	LUSC	6.26
TCGA-33-AASD-01A	LUSC	6.47
TCGA-92-8065-01A	LUSC	6.73
TCGA-56-A5DS-01A	LUSC	6.46
TCGA-98-A53I-01A	LUSC	6.76
TCGA-21-5786-01A	LUSC	7.41
TCGA-MF-A522-01A	LUSC	7.53
TCGA-43-7658-01A	LUSC	5.86
TCGA-60-2695-01A	LUSC	6.51
TCGA-39-5022-01A	LUSC	5.93
TCGA-63-7022-01A	LUSC	5.65
TCGA-56-7582-01A	LUSC	7.24
TCGA-77-8128-01A	LUSC	7.83
TCGA-33-AASL-01A	LUSC	7.4
TCGA-56-A4BW-01A	LUSC	7.14
TCGA-43-6771-01A	LUSC	6.71
TCGA-33-4533-01A	LUSC	7.39
TCGA-22-5471-01A	LUSC	7.12
TCGA-85-A512-01A	LUSC	7.34
TCGA-21-1080-01A	LUSC	8.23
TCGA-85-A5B5-01A	LUSC	6.91
TCGA-46-3769-01A	LUSC	7.93
TCGA-56-A4BY-01A	LUSC	6.92
TCGA-96-8169-01A	LUSC	6.36
TCGA-63-5128-01A	LUSC	6.26
TCGA-22-4596-01A	LUSC	6.9
TCGA-34-5232-01A	LUSC	6.57
TCGA-21-5787-01A	LUSC	7.29
TCGA-46-6026-01A	LUSC	7.41
TCGA-18-3417-01A	LUSC	7.24
TCGA-46-3765-01A	LUSC	7.27
TCGA-22-5473-01A	LUSC	6.83
TCGA-34-5240-01A	LUSC	7.71
TCGA-60-2725-01A	LUSC	6.73
TCGA-66-2790-01A	LUSC	7.31
TCGA-56-A4BX-01A	LUSC	7.63
TCGA-60-2698-01A	LUSC	7.29
TCGA-21-1075-01A	LUSC	7.77
TCGA-58-A46N-01A	LUSC	6.94
TCGA-L3-A524-01A	LUSC	7.11
TCGA-98-8020-01A	LUSC	6.74
TCGA-37-4130-01A	LUSC	9.93
TCGA-66-2757-01A	LUSC	6.14
TCGA-94-8491-01A	LUSC	7.65
TCGA-66-2786-01A	LUSC	8.17
TCGA-43-2581-01A	LUSC	7.21
TCGA-18-3409-01A	LUSC	7.34
TCGA-77-A5GH-01A	LUSC	6.96
TCGA-90-A4EE-01A	LUSC	6.44
TCGA-22-5481-01A	LUSC	6.31
TCGA-77-8143-01A	LUSC	7.17
TCGA-85-8582-01A	LUSC	7.31
TCGA-43-A475-01A	LUSC	6.65
TCGA-85-7696-01A	LUSC	7.41
TCGA-18-3412-01A	LUSC	8.43
TCGA-43-3394-01A	LUSC	7.56

TCGA-60-2721-01A	LUSC	7.52
TCGA-22-5477-01A	LUSC	8.13
TCGA-85-8071-01A	LUSC	7.27
TCGA-85-A510-01A	LUSC	5.98
TCGA-60-2703-01A	LUSC	7.02
TCGA-85-7697-01A	LUSC	7.49
TCGA-56-6546-01A	LUSC	6.77
TCGA-85-8355-01A	LUSC	7.23
TCGA-18-3408-01A	LUSC	8.94
TCGA-L3-A4E7-01A	LUSC	6.51
TCGA-22-4599-01A	LUSC	6.8
TCGA-85-8072-01A	LUSC	6.69
TCGA-60-2712-01A	LUSC	6.56
TCGA-56-8622-01A	LUSC	6.71
TCGA-NC-A5HD-01A	LUSC	6.83
TCGA-56-8309-01A	LUSC	6.24
TCGA-21-1077-01A	LUSC	7.3
TCGA-56-8626-01A	LUSC	6.7
TCGA-98-7454-01A	LUSC	6.65
TCGA-66-2791-01A	LUSC	7.14
TCGA-22-5483-01A	LUSC	6.8
TCGA-98-8022-01A	LUSC	6.69
TCGA-56-5897-01A	LUSC	6.39
TCGA-39-5035-01A	LUSC	7.37
TCGA-60-2706-01A	LUSC	7.28
TCGA-66-2766-01A	LUSC	7.93
TCGA-77-6843-01A	LUSC	7.03
TCGA-NC-A5HI-01A	LUSC	6.67
TCGA-56-8307-01A	LUSC	6.57
TCGA-77-A5G1-01A	LUSC	6.43
TCGA-33-6738-01A	LUSC	6.01
TCGA-98-A538-01A	LUSC	6.91
TCGA-58-A46K-01A	LUSC	6.88
TCGA-66-2768-01A	LUSC	7.68
TCGA-85-A511-01A	LUSC	6.78
TCGA-NC-A5HE-01A	LUSC	6.7
TCGA-56-7580-01A	LUSC	6.41
TCGA-66-2755-01A	LUSC	7.1
TCGA-39-5028-01A	LUSC	7.99
TCGA-37-A5EN-01A	LUSC	7.89
TCGA-34-2600-01A	LUSC	7.99
TCGA-58-A46J-01A	LUSC	6.83
TCGA-XC-AA0X-01A	LUSC	6.93
TCGA-66-2795-01A	LUSC	8.33
TCGA-66-2787-01A	LUSC	7.35
TCGA-66-2758-01A	LUSC	7.26
TCGA-70-6722-01A	LUSC	7.16
TCGA-58-A46M-01A	LUSC	6.59
TCGA-60-2715-01A	LUSC	6.75
TCGA-39-5040-01A	LUSC	8.08
TCGA-63-A5M9-01A	LUSC	6.2
TCGA-77-6844-01A	LUSC	7.22
TCGA-33-AASJ-01A	LUSC	7.22
TCGA-37-3789-01A	LUSC	7.93
TCGA-56-7822-01A	LUSC	7.24
TCGA-66-2770-01A	LUSC	7.1
TCGA-68-7757-01B	LUSC	5.91
TCGA-18-3415-01A	LUSC	6.48

TCGA-66-2778-01A	LUSC	7.81
TCGA-85-7710-01A	LUSC	7.18
TCGA-58-8391-01A	LUSC	6.38
TCGA-43-8118-01A	LUSC	7.85
TCGA-85-8276-01A	LUSC	6.62
TCGA-60-2710-01A	LUSC	7.65
TCGA-18-3414-01A	LUSC	7.95
TCGA-NC-A5HG-01A	LUSC	6.92
TCGA-33-6737-01A	LUSC	6.39
TCGA-43-A56V-01A	LUSC	6.68
TCGA-77-8136-01A	LUSC	6.41
TCGA-22-5472-01A	LUSC	8.2
TCGA-68-7756-01A	LUSC	7.78
TCGA-77-A5GA-01A	LUSC	6.98
TCGA-66-2783-01A	LUSC	6.69
TCGA-63-A5MN-01A	LUSC	6.36
TCGA-98-A53B-01A	LUSC	6.89
TCGA-21-5782-01A	LUSC	6.09
TCGA-22-0944-01A	LUSC	7.51
TCGA-43-5668-01A	LUSC	6.15
TCGA-85-8664-01A	LUSC	6.55
TCGA-94-7557-01A	LUSC	7.8
TCGA-77-7139-01A	LUSC	8.03
TCGA-39-5021-01A	LUSC	7.37
TCGA-77-6845-01A	LUSC	7.51
TCGA-66-2788-01A	LUSC	7.33
TCGA-63-A5MP-01A	LUSC	7.28
TCGA-85-8049-01A	LUSC	6.23
TCGA-33-4566-01A	LUSC	7.87
TCGA-39-5031-01A	LUSC	7.22
TCGA-56-A4ZK-01A	LUSC	7.12
TCGA-21-A5DI-01A	LUSC	6.24
TCGA-33-AAS8-01A	LUSC	6.53
TCGA-63-A5MM-01A	LUSC	6.9
TCGA-77-8009-01A	LUSC	6.72
TCGA-43-6143-01A	LUSC	7.03
TCGA-77-7335-01A	LUSC	7.66
TCGA-22-4595-01A	LUSC	6.5
TCGA-56-6545-01A	LUSC	6.89
TCGA-77-A5G3-01A	LUSC	7.31
TCGA-77-8133-01A	LUSC	7.18
TCGA-56-7731-01A	LUSC	6.17
TCGA-66-2785-01A	LUSC	6.25
TCGA-33-4538-01A	LUSC	7.13
TCGA-77-7337-01A	LUSC	6.81
TCGA-98-A53A-01A	LUSC	5.98
TCGA-68-8251-01A	LUSC	6.21
TCGA-63-A5ML-01A	LUSC	7.14
TCGA-85-A4QQ-01A	LUSC	7.04
TCGA-21-1071-01A	LUSC	7.17
TCGA-85-7950-01A	LUSC	6.16
TCGA-NK-A5CT-01A	LUSC	7.06
TCGA-33-4583-01A	LUSC	7.13
TCGA-NC-A5HN-01A	LUSC	7.38
TCGA-34-5231-01A	LUSC	7.23
TCGA-22-5479-01A	LUSC	7.1
TCGA-46-3766-01A	LUSC	7.15
TCGA-18-3411-01A	LUSC	7.41

TCGA-39-5039-01A	LUSC	7.61
TCGA-85-A4QR-01A	LUSC	7.24
TCGA-33-4582-01A	LUSC	7.31
TCGA-77-A5GB-01B	LUSC	6.14
TCGA-NQ-A638-01A	MESO	6.92
TCGA-LK-A4O4-01A	MESO	6.8
TCGA-TS-A7P6-01A	MESO	6.74
TCGA-TS-A8AS-01A	MESO	6.59
TCGA-UD-AAC4-01A	MESO	5.44
TCGA-MQ-A4LI-01A	MESO	6.31
TCGA-MQ-A6BN-01A	MESO	5.17
TCGA-MQ-A6BS-01A	MESO	6.02
TCGA-TS-A7P1-01A	MESO	6.5
TCGA-ZN-A9VW-01A	MESO	6.23
TCGA-SC-A6LQ-01A	MESO	6.4
TCGA-YS-AA4M-01A	MESO	6.79
TCGA-3U-A98F-01A	MESO	7.09
TCGA-3H-AB3O-01A	MESO	6.33
TCGA-3H-AB3T-01A	MESO	7.31
TCGA-3U-A98H-01A	MESO	5.94
TCGA-ZN-A9VS-01A	MESO	6.64
TCGA-UT-A88E-01B	MESO	6.3
TCGA-TS-A7P3-01A	MESO	6.84
TCGA-MQ-A4LM-01A	MESO	7.19
TCGA-UD-AAC7-01A	MESO	6.75
TCGA-SC-AA5Z-01A	MESO	6.77
TCGA-NQ-A57I-01A	MESO	7.01
TCGA-MQ-A6BL-01A	MESO	6.47
TCGA-UT-A88D-01B	MESO	6.59
TCGA-TS-A8AY-01A	MESO	7.33
TCGA-TS-A7OY-01A	MESO	6.99
TCGA-SC-A6LN-01A	MESO	6.11
TCGA-YS-A95B-01A	MESO	6.88
TCGA-3H-AB3S-01A	MESO	6.25
TCGA-LK-A4O0-01A	MESO	7.73
TCGA-LK-A4NW-01A	MESO	6.21
TCGA-TS-A7P7-01A	MESO	8.14
TCGA-SH-A9CT-01A	MESO	6.2
TCGA-SH-A9CU-01A	MESO	6.7
TCGA-ZN-A9VO-01A	MESO	5.67
TCGA-UD-AABZ-01A	MESO	5.53
TCGA-LK-A4O5-01A	MESO	6.81
TCGA-3H-AB3L-01A	MESO	6.52
TCGA-3U-A98G-01A	MESO	6.67
TCGA-MQ-A4LJ-01A	MESO	6.68
TCGA-MQ-A4LP-01A	MESO	6.14
TCGA-SH-A7BH-01A	MESO	5.04
TCGA-LK-A4O2-01A	MESO	6.53
TCGA-SH-A7BD-01A	MESO	4.79
TCGA-TS-A7OU-01B	MESO	5.71
TCGA-TS-A8AI-01A	MESO	5.41
TCGA-SC-A6LR-01A	MESO	5.72
TCGA-3U-A98D-01A	MESO	7.02
TCGA-3U-A98I-01A	MESO	6.97
TCGA-TS-A7PB-01A	MESO	5.48
TCGA-MQ-A6BQ-01A	MESO	5.6
TCGA-MQ-A4LC-01A	MESO	5.89
TCGA-ZN-A9VU-01A	MESO	6.57

TCGA-UT-A88G-01B	MESO	6.14
TCGA-MQ-A4KX-01A	MESO	6.02
TCGA-UT-A88C-01B	MESO	6.43
TCGA-MQ-A4LV-01A	MESO	5.95
TCGA-TS-A8AF-01A	MESO	6.7
TCGA-3H-AB3U-01A	MESO	5.99
TCGA-YS-A95C-01A	MESO	6.31
TCGA-UD-AAC5-01A	MESO	6.28
TCGA-LK-A4NY-01A	MESO	6.39
TCGA-LK-A4NZ-01A	MESO	5.89
TCGA-UD-AAC1-01A	MESO	6.55
TCGA-TS-A7P8-01A	MESO	6.37
TCGA-UD-AAC6-01A	MESO	6.43
TCGA-UD-AABY-01A	MESO	6.45
TCGA-SH-A7BC-01A	MESO	6.99
TCGA-3H-AB3X-01A	MESO	6.66
TCGA-LK-A4O6-01A	MESO	5.89
TCGA-SC-A6LP-01A	MESO	6.8
TCGA-3U-A98E-01A	MESO	5.85
TCGA-TS-A7P0-01A	MESO	6.74
TCGA-3H-AB3K-01A	MESO	6.01
TCGA-TS-A8AV-01A	MESO	7.03
TCGA-XT-AASU-01A	MESO	6.84
TCGA-3U-A98J-01A	MESO	6.27
TCGA-UT-A97Y-01A	MESO	6.61
TCGA-ZN-A9VV-01A	MESO	7.05
TCGA-LK-A4O7-01A	MESO	6.25
TCGA-SC-A6LM-01A	MESO	5.77
TCGA-TS-A7OZ-01A	MESO	7.01
TCGA-3H-AB3M-01A	MESO	6.9
TCGA-ZN-A9VP-01A	MESO	7.36
TCGA-ZN-A9VQ-01A	MESO	5.91
TCGA-MQ-A6BR-01A	MESO	6.36
TCGA-10-0933-01A	OV	5.06
TCGA-23-1024-01A	OV	6.48
TCGA-13-0886-01A	OV	5.99
TCGA-29-1769-01A	OV	6.22
TCGA-13-1505-01A	OV	6.71
TCGA-61-1736-01B	OV	7.18
TCGA-61-1737-01A	OV	5.87
TCGA-25-1871-01A	OV	6.17
TCGA-29-1705-02A	OV	6.4
TCGA-29-1705-01A	OV	6.93
TCGA-24-1104-01A	OV	5.85
TCGA-13-0720-01A	OV	6.43
TCGA-61-1728-01A	OV	6.46
TCGA-WR-A838-01A	OV	6.88
TCGA-23-1122-01A	OV	6.55
TCGA-13-1487-01A	OV	6.34
TCGA-23-1027-01A	OV	6.22
TCGA-29-1774-01A	OV	7.12
TCGA-30-1866-01A	OV	7.34
TCGA-23-1030-01A	OV	6.65
TCGA-25-1627-01A	OV	6.64
TCGA-13-1410-01A	OV	6.22
TCGA-24-1564-01A	OV	6.98
TCGA-10-0926-01A	OV	6.17
TCGA-13-1510-01A	OV	5.82

TCGA-31-1956-01A	OV	6.32
TCGA-24-2020-01A	OV	5.97
TCGA-23-1028-01A	OV	6.5
TCGA-13-1407-01A	OV	6.16
TCGA-04-1655-01A	OV	6.12
TCGA-09-1661-01B	OV	5.55
TCGA-24-1416-01A	OV	5.88
TCGA-61-1911-01A	OV	6.3
TCGA-61-1995-01A	OV	6.77
TCGA-10-0927-01A	OV	6.27
TCGA-20-1684-01A	OV	6.75
TCGA-31-1951-01A	OV	4.38
TCGA-13-1411-01A	OV	6.3
TCGA-24-1425-01A	OV	6.63
TCGA-09-0367-01A	OV	6.87
TCGA-30-1718-01A	OV	6.57
TCGA-20-1687-01A	OV	6.28
TCGA-61-2094-01A	OV	5.68
TCGA-61-1743-01A	OV	5.41
TCGA-30-1892-01A	OV	6.7
TCGA-09-1665-01B	OV	6.68
TCGA-13-0801-01A	OV	5.85
TCGA-04-1343-01A	OV	6.06
TCGA-61-2092-01A	OV	6.55
TCGA-5X-AA5U-01A	OV	6.31
TCGA-61-1740-01A	OV	6.06
TCGA-09-1674-01A	OV	7.52
TCGA-61-2088-01A	OV	5.34
TCGA-24-1567-01A	OV	6.13
TCGA-25-1633-01A	OV	6.88
TCGA-23-2084-01A	OV	6.38
TCGA-59-2348-01A	OV	6.66
TCGA-OY-A56P-01A	OV	5.77
TCGA-24-1544-01A	OV	6.21
TCGA-04-1338-01A	OV	6.81
TCGA-36-1576-01A	OV	6.88
TCGA-13-0885-01A	OV	6.65
TCGA-13-0913-01A	OV	5.74
TCGA-13-0724-01A	OV	5.88
TCGA-09-1669-01A	OV	6.61
TCGA-20-1683-01A	OV	6.32
TCGA-23-1107-01A	OV	4.98
TCGA-13-0795-01A	OV	5.94
TCGA-25-2399-01A	OV	6.58
TCGA-29-1703-01A	OV	6.48
TCGA-24-1560-01A	OV	6.55
TCGA-29-2414-02A	OV	6.38
TCGA-24-2289-01A	OV	6.02
TCGA-61-1725-01A	OV	6.42
TCGA-25-1632-01A	OV	6.96
TCGA-13-1488-01A	OV	5.35
TCGA-24-2036-01A	OV	3.03
TCGA-13-1497-01A	OV	5.38
TCGA-59-2354-01A	OV	7.04
TCGA-24-1474-01A	OV	6.79
TCGA-24-1426-01A	OV	6.03
TCGA-13-1496-01A	OV	5.98
TCGA-13-0890-01A	OV	6.37

TCGA-04-1514-01A	OV	6.58
TCGA-36-1577-01A	OV	6.81
TCGA-59-2350-01A	OV	6.47
TCGA-29-A5NZ-01A	OV	6.39
TCGA-61-2098-01A	OV	7.35
TCGA-23-2077-01A	OV	6.23
TCGA-23-1120-01A	OV	6.7
TCGA-24-1565-01A	OV	6.59
TCGA-24-1423-01A	OV	6.83
TCGA-61-1900-01A	OV	5.99
TCGA-29-1781-01A	OV	5.48
TCGA-20-1685-01A	OV	7.22
TCGA-09-2054-01A	OV	6.62
TCGA-25-1313-01A	OV	6.95
TCGA-13-0760-01A	OV	6.3
TCGA-10-0931-01A	OV	6.05
TCGA-13-0768-01A	OV	5.56
TCGA-3P-A9WA-01A	OV	6.32
TCGA-24-1545-01A	OV	6.55
TCGA-13-0906-01A	OV	6.95
TCGA-13-1482-01A	OV	6.05
TCGA-36-1581-01A	OV	6.1
TCGA-29-1711-01A	OV	6.29
TCGA-13-1499-01A	OV	5.72
TCGA-23-1023-01A	OV	5.31
TCGA-29-2428-01A	OV	6.38
TCGA-24-1844-01A	OV	7.09
TCGA-25-1635-01A	OV	6.29
TCGA-23-1114-01B	OV	6.11
TCGA-OY-A56Q-01A	OV	6.72
TCGA-31-1946-01A	OV	6.04
TCGA-23-2078-01A	OV	6.56
TCGA-61-2109-01A	OV	7.76
TCGA-57-1584-01A	OV	6.95
TCGA-25-2409-01A	OV	6.37
TCGA-04-1347-01A	OV	5.49
TCGA-36-1568-01A	OV	6.58
TCGA-09-2056-01B	OV	7.08
TCGA-24-2261-01A	OV	7.2
TCGA-24-1471-01A	OV	6.88
TCGA-13-0893-01B	OV	6.18
TCGA-25-1634-01A	OV	6.57
TCGA-25-1870-01A	OV	6.22
TCGA-13-0797-01A	OV	6.49
TCGA-31-1959-01A	OV	6.6
TCGA-57-1993-01A	OV	6.95
TCGA-13-1409-01A	OV	6.24
TCGA-09-1668-01B	OV	6.2
TCGA-13-1477-01A	OV	5.93
TCGA-24-1418-01A	OV	6.73
TCGA-13-0887-01A	OV	5.85
TCGA-13-2060-01A	OV	6.64
TCGA-24-2288-01A	OV	6.65
TCGA-29-1784-01A	OV	6.42
TCGA-57-1585-01A	OV	6.42
TCGA-13-0916-01A	OV	6.28
TCGA-09-1670-01A	OV	7.54
TCGA-57-1994-01A	OV	6.84

TCGA-23-1123-01A	OV	6.83
TCGA-24-0968-01A	OV	5.68
TCGA-25-1321-01A	OV	7.33
TCGA-24-1930-01A	OV	6.77
TCGA-29-1693-01A	OV	7.19
TCGA-30-1853-01A	OV	6.13
TCGA-61-2016-01A	OV	7.16
TCGA-23-1118-01A	OV	6.44
TCGA-24-1604-01A	OV	7.06
TCGA-23-1109-01A	OV	6.27
TCGA-25-1631-01A	OV	6.45
TCGA-13-0897-01A	OV	7.12
TCGA-24-0975-01A	OV	6.14
TCGA-61-1738-01A	OV	5.88
TCGA-61-2008-02A	OV	7.19
TCGA-61-2008-01A	OV	6.87
TCGA-24-1549-01A	OV	7.32
TCGA-25-2401-01A	OV	6.74
TCGA-29-1761-01A	OV	5.86
TCGA-09-2045-01A	OV	5.62
TCGA-29-2425-01A	OV	7.37
TCGA-24-1551-01A	OV	6.88
TCGA-13-1498-01A	OV	5.76
TCGA-25-1316-01A	OV	6.27
TCGA-25-1320-01A	OV	6.42
TCGA-30-1891-01A	OV	6.85
TCGA-24-1434-01A	OV	6.82
TCGA-13-1507-01A	OV	6.46
TCGA-29-1707-02A	OV	5.23
TCGA-25-1623-01A	OV	7.09
TCGA-04-1364-01A	OV	6.05
TCGA-25-2042-01A	OV	6.95
TCGA-13-1512-01A	OV	6.01
TCGA-24-1923-01A	OV	6.58
TCGA-13-1404-01A	OV	5.93
TCGA-61-2102-01A	OV	6.9
TCGA-VG-A8LO-01A	OV	6.95
TCGA-04-1356-01A	OV	6.65
TCGA-29-1701-01A	OV	6.56
TCGA-13-0900-01B	OV	5.69
TCGA-29-1768-01A	OV	6.33
TCGA-57-1583-01A	OV	6.08
TCGA-13-0765-01A	OV	6
TCGA-24-2298-01A	OV	5.95
TCGA-61-2111-01A	OV	6.18
TCGA-29-1696-01A	OV	5.75
TCGA-24-2267-01A	OV	6.07
TCGA-13-0727-01A	OV	6.35
TCGA-20-1682-01A	OV	6.07
TCGA-13-0911-01A	OV	5.47
TCGA-20-0991-01A	OV	5.59
TCGA-57-1586-01A	OV	6.35
TCGA-24-1417-01A	OV	6.98
TCGA-24-1464-01A	OV	6.68
TCGA-25-2397-01A	OV	6.52
TCGA-13-1485-01A	OV	6.34
TCGA-30-1857-01A	OV	6.51
TCGA-25-2400-01A	OV	6.53

TCGA-29-1762-01A	OV	7.23
TCGA-57-1582-01A	OV	7.29
TCGA-23-1113-01A	OV	6.66
TCGA-24-1552-01A	OV	7.4
TCGA-25-2396-01A	OV	6.44
TCGA-25-1626-01A	OV	6.73
TCGA-29-1690-01A	OV	6.8
TCGA-25-1315-01A	OV	6.99
TCGA-13-0884-01B	OV	5.99
TCGA-09-1659-01B	OV	6.12
TCGA-25-2392-01A	OV	6.61
TCGA-25-1312-01A	OV	6.06
TCGA-13-0888-01A	OV	5.19
TCGA-29-1776-01A	OV	7.47
TCGA-13-1495-01A	OV	6.61
TCGA-24-1846-01A	OV	6.56
TCGA-09-2044-01B	OV	6.94
TCGA-29-1770-02A	OV	6.22
TCGA-13-1483-01A	OV	6.3
TCGA-31-1953-01A	OV	6.99
TCGA-10-0934-01A	OV	5.63
TCGA-23-1111-01A	OV	6.4
TCGA-59-2355-01A	OV	6.28
TCGA-04-1331-01A	OV	4.91
TCGA-24-1556-01A	OV	5.88
TCGA-36-1569-01A	OV	6.73
TCGA-24-1557-01A	OV	6.39
TCGA-61-2002-01A	OV	6.76
TCGA-61-1914-01A	OV	6.19
TCGA-13-0804-01A	OV	5.91
TCGA-13-1408-01A	OV	5.43
TCGA-24-0979-01A	OV	6.26
TCGA-24-2254-01A	OV	6.69
TCGA-25-1319-01A	OV	5.85
TCGA-04-1341-01A	OV	5.62
TCGA-25-1328-01A	OV	6.89
TCGA-24-1105-01A	OV	6.62
TCGA-61-1907-01A	OV	6.09
TCGA-61-2110-01A	OV	6.93
TCGA-25-1625-01A	OV	7.33
TCGA-23-1023-01R	OV	5.67
TCGA-24-1843-01A	OV	6.8
TCGA-24-1849-01A	OV	7.06
TCGA-29-1763-01A	OV	5.68
TCGA-04-1536-01A	OV	6.42
TCGA-24-2026-01A	OV	6.15
TCGA-61-1918-01A	OV	6.98
TCGA-24-1427-01A	OV	7.21
TCGA-24-2290-01A	OV	6.27
TCGA-24-1430-01A	OV	5.98
TCGA-24-1431-01A	OV	6.68
TCGA-13-0726-01A	OV	6.46
TCGA-24-2297-01A	OV	2.62
TCGA-04-1517-01A	OV	6.92
TCGA-59-2351-01A	OV	6.63
TCGA-25-1314-01A	OV	7.47
TCGA-13-1506-01A	OV	7
TCGA-04-1350-01A	OV	6.17

TCGA-24-1463-01A	OV	7.22
TCGA-24-2262-01A	OV	6.61
TCGA-13-0714-01A	OV	5.35
TCGA-09-2048-01A	OV	6.33
TCGA-29-1702-01A	OV	6.01
TCGA-25-1318-01A	OV	7.01
TCGA-24-2038-01A	OV	6.52
TCGA-09-0366-01A	OV	6.47
TCGA-61-1721-01A	OV	7.14
TCGA-61-2012-01A	OV	6.26
TCGA-13-0905-01B	OV	6.15
TCGA-24-1847-01A	OV	6.72
TCGA-24-2027-01A	OV	7.46
TCGA-24-1924-01A	OV	6.7
TCGA-61-2000-01A	OV	6.36
TCGA-25-2404-01A	OV	7.64
TCGA-10-0928-01A	OV	7.28
TCGA-24-1467-01A	OV	6.46
TCGA-13-1403-01A	OV	6.37
TCGA-04-1362-01A	OV	6.72
TCGA-13-0901-01B	OV	6.54
TCGA-24-0982-01A	OV	6.62
TCGA-29-1785-01A	OV	6.37
TCGA-24-1550-01A	OV	5.98
TCGA-29-1698-01A	OV	6.49
TCGA-09-0369-01A	OV	5.93
TCGA-29-1694-01A	OV	6.66
TCGA-25-1630-01A	OV	6.7
TCGA-13-0730-01A	OV	6.29
TCGA-09-2051-01A	OV	6.31
TCGA-20-0987-01A	OV	6.03
TCGA-13-0913-02A	OV	5.2
TCGA-04-1365-01A	OV	5.36
TCGA-09-1667-01C	OV	6.35
TCGA-61-1724-01A	OV	8.16
TCGA-29-1695-01A	OV	6.88
TCGA-13-0800-01A	OV	5.45
TCGA-23-1119-01A	OV	6.09
TCGA-29-1766-01A	OV	6.23
TCGA-25-1323-01A	OV	6.89
TCGA-13-1405-01A	OV	5.95
TCGA-25-1628-01A	OV	6.73
TCGA-59-2363-01A	OV	6.08
TCGA-04-1519-01A	OV	6.68
TCGA-09-2053-01C	OV	5.99
TCGA-13-A5FT-01A	OV	6.08
TCGA-61-1919-01A	OV	6.73
TCGA-04-1357-01A	OV	6.78
TCGA-24-0966-01A	OV	5.69
TCGA-20-1686-01A	OV	6.03
TCGA-23-1032-01A	OV	6.68
TCGA-04-1337-01A	OV	6.21
TCGA-04-1348-01A	OV	5.72
TCGA-13-0924-01A	OV	5.42
TCGA-25-2398-01A	OV	6
TCGA-23-1110-01A	OV	6.86
TCGA-24-1428-01A	OV	6.92
TCGA-61-2009-01A	OV	6.77

TCGA-10-0937-01A	OV	6.15
TCGA-24-1928-01A	OV	6.51
TCGA-04-1332-01A	OV	6.23
TCGA-24-2293-01A	OV	6.91
TCGA-25-1322-01A	OV	6.98
TCGA-23-1021-01B	OV	5.47
TCGA-24-1845-01A	OV	5.2
TCGA-13-0799-01A	OV	6.35
TCGA-24-1558-01A	OV	6.65
TCGA-23-1026-01B	OV	6.28
TCGA-25-1324-01A	OV	6.99
TCGA-24-2271-01A	OV	6.17
TCGA-29-1691-01A	OV	6.18
TCGA-29-2414-01A	OV	6.32
TCGA-04-1361-01A	OV	6.52
TCGA-25-2393-01A	OV	6.48
TCGA-24-1470-01A	OV	6.38
TCGA-25-1329-01A	OV	6.16
TCGA-24-2019-01A	OV	6.41
TCGA-24-2280-01A	OV	5.89
TCGA-23-1809-01A	OV	6.27
TCGA-24-1850-01A	OV	6.47
TCGA-24-1424-01A	OV	6.56
TCGA-13-0920-01A	OV	6.86
TCGA-09-1662-01A	OV	6.96
TCGA-59-A5PD-01A	OV	7.53
TCGA-13-0766-01A	OV	6.57
TCGA-24-2033-01A	OV	6.33
TCGA-24-1546-01A	OV	6.41
TCGA-36-1574-01A	OV	6.73
TCGA-24-1103-01A	OV	6.04
TCGA-25-2391-01A	OV	6.17
TCGA-04-1648-01A	OV	5.3
TCGA-29-1699-01A	OV	6.56
TCGA-23-1022-01A	OV	6.01
TCGA-13-1509-01A	OV	5.42
TCGA-36-1578-01A	OV	8.13
TCGA-31-1944-01A	OV	5.82
TCGA-24-1616-01A	OV	6.27
TCGA-09-0364-01A	OV	6.49
TCGA-23-1116-01A	OV	6.6
TCGA-13-1511-01A	OV	6.6
TCGA-24-1436-01A	OV	6.45
TCGA-61-2113-01A	OV	6.31
TCGA-59-2352-01A	OV	5.87
TCGA-61-1910-01A	OV	6.96
TCGA-24-1413-01A	OV	6.68
TCGA-29-1697-01A	OV	6.85
TCGA-29-1688-01A	OV	6
TCGA-61-2003-01A	OV	7.18
TCGA-61-2104-01A	OV	7.11
TCGA-29-1710-01A	OV	7.6
TCGA-24-2023-01A	OV	7.09
TCGA-24-1562-01A	OV	6.67
TCGA-61-1998-01A	OV	6.57
TCGA-31-1950-01A	OV	6.76
TCGA-29-1783-01A	OV	6.67
TCGA-61-1733-01A	OV	6.96

TCGA-29-1778-01A	OV	6.02
TCGA-13-1501-01A	OV	5.43
TCGA-30-1860-01A	OV	6.02
TCGA-24-1563-01A	OV	6.53
TCGA-36-1580-01A	OV	6.57
TCGA-61-2097-01A	OV	6.53
TCGA-30-1862-01A	OV	6.61
TCGA-04-1651-01A	OV	6.02
TCGA-23-2081-01A	OV	6.41
TCGA-13-0923-01A	OV	5.18
TCGA-13-0725-01A	OV	5.15
TCGA-04-1530-01A	OV	5.82
TCGA-13-0883-01A	OV	7.05
TCGA-09-1666-01A	OV	7.03
TCGA-13-0899-01A	OV	5.14
TCGA-24-2281-01A	OV	5.95
TCGA-29-1777-01A	OV	6.44
TCGA-61-2101-01A	OV	7.18
TCGA-24-1555-01A	OV	7.31
TCGA-24-1548-01A	OV	7.41
TCGA-13-1492-01A	OV	5.08
TCGA-13-0762-01A	OV	5.77
TCGA-24-1419-01A	OV	8
TCGA-25-1317-01A	OV	6.46
TCGA-10-0938-01A	OV	5.22
TCGA-36-1575-01A	OV	6.98
TCGA-29-2427-01A	OV	6.71
TCGA-30-1855-01A	OV	6.15
TCGA-13-0919-01A	OV	6.19
TCGA-24-1435-01A	OV	6.7
TCGA-23-1029-01B	OV	7.27
TCGA-09-1673-01A	OV	6.88
TCGA-29-1770-01A	OV	6.23
TCGA-10-0936-01A	OV	5.54
TCGA-61-2095-01A	OV	6.49
TCGA-13-1489-01A	OV	6.29
TCGA-13-1489-02A	OV	6.11
TCGA-36-1571-01A	OV	7.01
TCGA-61-1741-01A	OV	6.7
TCGA-24-1603-01A	OV	7.49
TCGA-13-1481-01A	OV	6.25
TCGA-24-0970-01B	OV	6.27
TCGA-24-1469-01A	OV	6.05
TCGA-24-1422-01A	OV	6.91
TCGA-24-2035-01A	OV	7.14
TCGA-24-1553-01A	OV	7.25
TCGA-30-1861-01A	OV	6.91
TCGA-13-0908-01B	OV	6.18
TCGA-25-1877-01A	OV	6.6
TCGA-04-1542-01A	OV	6.72
TCGA-25-1326-01A	OV	7.36
TCGA-24-2024-01A	OV	6.68
TCGA-36-1570-01A	OV	5.97
TCGA-30-1714-01A	OV	7.65
TCGA-13-0891-01A	OV	5.95
TCGA-24-1842-01A	OV	6.89
TCGA-FB-AAPP-01A	PAAD	6.93
TCGA-HZ-7289-01A	PAAD	6.49

TCGA-2L-AAQL-01A	PAAD	7.08
TCGA-3A-A9IL-01A	PAAD	6.42
TCGA-FB-AAPS-01A	PAAD	6.63
TCGA-2J-AAB8-01A	PAAD	7.51
TCGA-2J-AABT-01A	PAAD	6.93
TCGA-OE-A75W-01A	PAAD	6.89
TCGA-IB-8127-01A	PAAD	7.46
TCGA-HZ-8002-01A	PAAD	7.32
TCGA-HV-A5A6-01A	PAAD	6.83
TCGA-US-A779-01A	PAAD	6.87
TCGA-3A-A9IR-01A	PAAD	6.97
TCGA-HZ-8637-01A	PAAD	6.22
TCGA-HZ-7923-01A	PAAD	6.96
TCGA-IB-AAUN-01A	PAAD	7.25
TCGA-LB-A7SX-01A	PAAD	6.74
TCGA-HV-A7OP-01A	PAAD	7.08
TCGA-IB-A5ST-01A	PAAD	6.62
TCGA-3A-A9IC-01A	PAAD	6.83
TCGA-2J-AAB6-01A	PAAD	7.14
TCGA-HZ-8003-01A	PAAD	5.99
TCGA-H6-8124-01A	PAAD	7.29
TCGA-HZ-A9TJ-01A	PAAD	6.53
TCGA-FB-AAPY-01A	PAAD	6.07
TCGA-FB-A545-01A	PAAD	6.87
TCGA-2J-AABH-01A	PAAD	6.98
TCGA-HZ-8317-01A	PAAD	6.54
TCGA-2J-AABO-01A	PAAD	6.78
TCGA-3A-A9IS-01A	PAAD	6.72
TCGA-IB-7891-01A	PAAD	6.94
TCGA-2J-AABA-01A	PAAD	6.52
TCGA-US-A776-01A	PAAD	5.85
TCGA-IB-7886-01A	PAAD	8.02
TCGA-PZ-A5RE-01A	PAAD	7.25
TCGA-XD-AAUG-01A	PAAD	6.87
TCGA-HV-A5A3-01A	PAAD	7.14
TCGA-2L-AAQJ-01A	PAAD	7.01
TCGA-HZ-A77O-01A	PAAD	6.21
TCGA-FB-AAQ2-01A	PAAD	7.31
TCGA-FB-AAQ1-01A	PAAD	6.5
TCGA-HV-AA8V-01A	PAAD	6.52
TCGA-3A-A9IH-01A	PAAD	7.42
TCGA-3A-A9IO-01A	PAAD	6.81
TCGA-IB-AAUW-01A	PAAD	7.1
TCGA-HV-AA8X-01A	PAAD	7.15
TCGA-F2-A7TX-01A	PAAD	7.46
TCGA-IB-7644-01A	PAAD	7.56
TCGA-IB-A5SS-01A	PAAD	7.79
TCGA-IB-7897-01A	PAAD	7.27
TCGA-IB-7889-01A	PAAD	7.13
TCGA-US-A77J-01A	PAAD	6.61
TCGA-3A-A9IZ-01A	PAAD	6.72
TCGA-IB-8126-01A	PAAD	5.81
TCGA-HZ-8001-01A	PAAD	6.86
TCGA-2J-AABR-01A	PAAD	6.95
TCGA-F2-A44H-01A	PAAD	6.36
TCGA-Z5-AAPL-01A	PAAD	6.97
TCGA-IB-AAUR-01A	PAAD	6.6
TCGA-F2-A8YN-01A	PAAD	7.23

TCGA-3A-A9IJ-01A	PAAD	5.98
TCGA-US-A77G-01A	PAAD	7.15
TCGA-FB-AAPU-01A	PAAD	6.84
TCGA-IB-7885-01A	PAAD	6.57
TCGA-IB-AAUO-01A	PAAD	6.75
TCGA-IB-7645-01A	PAAD	7.15
TCGA-F2-6879-01A	PAAD	7.76
TCGA-FB-AAQ6-01A	PAAD	7.28
TCGA-HZ-A9TJ-06A	PAAD	6.8
TCGA-IB-AAUU-01A	PAAD	7.31
TCGA-HZ-7924-01A	PAAD	7.1
TCGA-XD-AAUH-01A	PAAD	6.99
TCGA-IB-AAUM-01A	PAAD	5.91
TCGA-S4-A8RM-01A	PAAD	7.29
TCGA-S4-A8RP-01A	PAAD	6.9
TCGA-HZ-A49I-01A	PAAD	7.02
TCGA-2J-AABI-01A	PAAD	6.78
TCGA-3A-A9I9-01A	PAAD	5.99
TCGA-HZ-8636-01A	PAAD	7.21
TCGA-HZ-8005-01A	PAAD	7.5
TCGA-HV-A5A4-01A	PAAD	6.8
TCGA-2J-AAB4-01A	PAAD	7.03
TCGA-HV-A7OL-01A	PAAD	6.49
TCGA-IB-AAUP-01A	PAAD	6.67
TCGA-FB-A5VM-01A	PAAD	7.25
TCGA-IB-A5SQ-01A	PAAD	7.03
TCGA-2J-AABE-01A	PAAD	6.96
TCGA-YH-A8SY-01A	PAAD	6.71
TCGA-HZ-8638-01A	PAAD	6.92
TCGA-IB-A6UF-01A	PAAD	7.6
TCGA-HZ-7918-01A	PAAD	6.75
TCGA-US-A77E-01A	PAAD	6.8
TCGA-RB-A7B8-01A	PAAD	6.38
TCGA-IB-A7M4-01A	PAAD	7.75
TCGA-IB-7654-01A	PAAD	6.37
TCGA-HZ-7922-01A	PAAD	7.72
TCGA-2J-AAB1-01A	PAAD	7.05
TCGA-IB-AAUS-01A	PAAD	6.68
TCGA-IB-AAUT-01A	PAAD	6.74
TCGA-2L-AAQM-01A	PAAD	5.93
TCGA-3A-A9IX-01A	PAAD	6.7
TCGA-2L-AAQE-01A	PAAD	7.23
TCGA-3A-A9I5-01A	PAAD	6.49
TCGA-FB-AAQ0-01A	PAAD	7.37
TCGA-2J-AABP-01A	PAAD	6.68
TCGA-FB-A4P6-01A	PAAD	7.11
TCGA-XD-AAUI-01A	PAAD	6.8
TCGA-3E-AAAZ-01A	PAAD	7.47
TCGA-RB-AA9M-01A	PAAD	7.14
TCGA-3E-AAAY-01A	PAAD	6.74
TCGA-FB-A4P5-01A	PAAD	6.78
TCGA-RL-AAAS-01A	PAAD	6.85
TCGA-S4-A8RO-01A	PAAD	6.42
TCGA-YB-A89D-01A	PAAD	7.21
TCGA-IB-A5SO-01A	PAAD	7.02
TCGA-HZ-A8P1-01A	PAAD	7.1
TCGA-XN-A8T3-01A	PAAD	6.78
TCGA-IB-A5SP-01A	PAAD	7.18

TCGA-IB-7646-01A	PAAD	7.22
TCGA-3A-A9IU-01A	PAAD	6.5
TCGA-2J-AABV-01A	PAAD	4.9
TCGA-3A-A9IV-01A	PAAD	6.58
TCGA-Q3-AA2A-01A	PAAD	6.93
TCGA-F2-A44G-01A	PAAD	7.25
TCGA-IB-7652-01A	PAAD	7.07
TCGA-LB-A9Q5-01A	PAAD	5.57
TCGA-FB-A78T-01A	PAAD	6.98
TCGA-IB-7893-01A	PAAD	7.31
TCGA-3A-A9IB-01A	PAAD	7.22
TCGA-IB-A6UG-01A	PAAD	6.79
TCGA-HZ-A49G-01A	PAAD	6.63
TCGA-IB-7651-01A	PAAD	7.13
TCGA-F2-7273-01A	PAAD	7.24
TCGA-IB-7890-01A	PAAD	7.24
TCGA-3A-A9I7-01A	PAAD	6.7
TCGA-FB-AAQ3-01A	PAAD	7.38
TCGA-HZ-A77P-01A	PAAD	6.85
TCGA-HZ-A4BK-01A	PAAD	7.01
TCGA-XD-AAUL-01A	PAAD	7.26
TCGA-IB-AAUV-01A	PAAD	6.85
TCGA-H6-A45N-01A	PAAD	6.85
TCGA-HZ-8519-01A	PAAD	7.07
TCGA-2J-AABF-01A	PAAD	6.76
TCGA-IB-A7LX-01A	PAAD	8.34
TCGA-3A-A9J0-01A	PAAD	6.62
TCGA-HZ-A8P0-01A	PAAD	5.57
TCGA-2J-AAB9-01A	PAAD	6.66
TCGA-IB-AAUQ-01A	PAAD	6.49
TCGA-2L-AAQI-01A	PAAD	7.11
TCGA-F2-6880-01A	PAAD	4.8
TCGA-L1-A7W4-01A	PAAD	6.66
TCGA-IB-7647-01A	PAAD	7.61
TCGA-F2-7276-01A	PAAD	7.16
TCGA-FB-A7DR-01A	PAAD	6.95
TCGA-IB-7649-01A	PAAD	6.95
TCGA-HZ-7925-01A	PAAD	7.15
TCGA-2L-AAQA-01A	PAAD	7.34
TCGA-YY-A8LH-01A	PAAD	7.05
TCGA-M8-A5N4-01A	PAAD	6.8
TCGA-IB-7887-01A	PAAD	6.27
TCGA-FB-AAPZ-01A	PAAD	7.46
TCGA-HV-A5A5-01A	PAAD	6.71
TCGA-HZ-A49H-01A	PAAD	6.52
TCGA-Q3-A5QY-01A	PAAD	6.31
TCGA-HZ-7920-01A	PAAD	7.35
TCGA-HZ-7919-01A	PAAD	7.68
TCGA-HZ-8315-01A	PAAD	7.23
TCGA-HZ-7926-01A	PAAD	7.59
TCGA-IB-7888-01A	PAAD	6.87
TCGA-HZ-A4BH-01A	PAAD	7.1
TCGA-US-A774-01A	PAAD	6.57
TCGA-H8-A6C1-01A	PAAD	6.5
TCGA-XN-A8T5-01A	PAAD	6.97
TCGA-2J-AABU-01A	PAAD	6.65
TCGA-2J-AABK-01A	PAAD	6.72
TCGA-LB-A8F3-01A	PAAD	6.31

TCGA-HZ-A77Q-01A	PAAD	6.88
TCGA-FB-AAPQ-01A	PAAD	6.96
TCGA-3A-A9IN-01A	PAAD	6.16
TCGA-QR-A70H-01A	PCPG	6.41
TCGA-QR-A70X-01A	PCPG	6.19
TCGA-S7-A7WW-01A	PCPG	5.56
TCGA-PR-A5PF-01A	PCPG	7.14
TCGA-RW-A689-01A	PCPG	6.66
TCGA-QR-A6GR-01A	PCPG	6.4
TCGA-QR-A6GZ-05A	PCPG	6.31
TCGA-RW-A681-01A	PCPG	6.27
TCGA-WB-A81W-01A	PCPG	6.87
TCGA-TT-A6YO-01A	PCPG	7.64
TCGA-WB-A81J-01A	PCPG	6.42
TCGA-QR-A6GT-01A	PCPG	6.52
TCGA-RW-A68G-01A	PCPG	6.17
TCGA-RW-A68D-01A	PCPG	6.96
TCGA-WB-A81R-01A	PCPG	7.25
TCGA-RW-A68C-01A	PCPG	6.95
TCGA-QR-A70U-01A	PCPG	6.25
TCGA-WB-A81K-01A	PCPG	7.24
TCGA-QR-A70O-01A	PCPG	4.68
TCGA-TT-A6YJ-01A	PCPG	7.05
TCGA-RW-A68B-01A	PCPG	7.31
TCGA-RW-A68A-01A	PCPG	7.13
TCGA-S7-A7WL-01A	PCPG	6.78
TCGA-QR-A6H2-01A	PCPG	5.85
TCGA-QT-A5XO-01A	PCPG	7.03
TCGA-RT-A6Y9-01A	PCPG	6.75
TCGA-WB-A80K-01A	PCPG	6.11
TCGA-SR-A6N0-01A	PCPG	7.42
TCGA-SR-A6MX-05A	PCPG	5.86
TCGA-WB-A81M-01A	PCPG	6.81
TCGA-TT-A6YN-01A	PCPG	6.94
TCGA-S7-A7WP-01A	PCPG	6.92
TCGA-QR-A707-01A	PCPG	6.58
TCGA-QR-A6GS-01A	PCPG	6.47
TCGA-WB-A80V-01A	PCPG	6.18
TCGA-RW-A67X-01A	PCPG	6.14
TCGA-QR-A7IP-01A	PCPG	6.63
TCGA-SR-A6MR-01A	PCPG	6.21
TCGA-QT-A5XP-01A	PCPG	6.68
TCGA-S7-A7WM-01A	PCPG	6.28
TCGA-RW-A68F-01A	PCPG	5.83
TCGA-WB-A822-01A	PCPG	6.19
TCGA-WB-A815-01A	PCPG	6.62
TCGA-XG-A823-01A	PCPG	5.74
TCGA-W2-A7H7-01A	PCPG	5.9
TCGA-S7-A7WX-01A	PCPG	6.67
TCGA-SP-A6QF-01A	PCPG	6.41
TCGA-QR-A70J-01A	PCPG	6.76
TCGA-QR-A708-01A	PCPG	4.73
TCGA-SQ-A6I4-01A	PCPG	6.51
TCGA-WB-A81N-01A	PCPG	6.89
TCGA-QR-A6H1-01A	PCPG	7.15
TCGA-QR-A6ZZ-01A	PCPG	6.7
TCGA-S7-A7WU-01A	PCPG	6.08
TCGA-QR-A70G-01B	PCPG	6.61

TCGA-QT-A5XN-01A	PCPG	6.83
TCGA-QT-A5XL-01A	PCPG	6.46
TCGA-QR-A703-01A	PCPG	6.54
TCGA-W2-A7HD-01A	PCPG	6.97
TCGA-QT-A69Q-01A	PCPG	7.01
TCGA-QR-A70R-01A	PCPG	5.54
TCGA-WB-A816-01A	PCPG	6.85
TCGA-RT-A6YA-01A	PCPG	6.3
TCGA-WB-A820-01A	PCPG	7.67
TCGA-QR-A70V-01A	PCPG	6.43
TCGA-S7-A7WQ-01A	PCPG	6.42
TCGA-QR-A702-01A	PCPG	6.65
TCGA-QR-A70W-01A	PCPG	5.76
TCGA-QR-A6H0-01A	PCPG	6.26
TCGA-SP-A6QK-01A	PCPG	6.75
TCGA-QR-A70D-01A	PCPG	6.14
TCGA-SR-A6MT-01A	PCPG	6.27
TCGA-WB-A81F-01A	PCPG	6.9
TCGA-QR-A70Q-01A	PCPG	4.87
TCGA-W2-A7HF-01A	PCPG	6.77
TCGA-WB-A81S-01A	PCPG	6.37
TCGA-SR-A6MU-01A	PCPG	6.45
TCGA-QT-A5XM-01A	PCPG	6.75
TCGA-WB-A81A-01A	PCPG	6.8
TCGA-SR-A6MX-01A	PCPG	5.78
TCGA-QR-A70M-01A	PCPG	6.96
TCGA-SR-A6MP-01A	PCPG	7.13
TCGA-WB-A80P-01A	PCPG	6.2
TCGA-RW-A8AZ-01A	PCPG	6.76
TCGA-QR-A6GO-01A	PCPG	6.68
TCGA-QR-A6H5-01A	PCPG	6.87
TCGA-WB-A81V-01A	PCPG	6.3
TCGA-QR-A70I-01A	PCPG	6.52
TCGA-PR-A5PH-01A	PCPG	6.91
TCGA-QR-A6GX-01A	PCPG	5.84
TCGA-WB-A81D-01A	PCPG	6.94
TCGA-RW-A67Y-01A	PCPG	7.7
TCGA-RW-A67V-01A	PCPG	5.72
TCGA-W2-A7HE-01A	PCPG	7.21
TCGA-W2-A7HA-01B	PCPG	7.11
TCGA-SR-A6MZ-01A	PCPG	6.73
TCGA-QR-A6GZ-01A	PCPG	6.5
TCGA-P7-A5NX-01A	PCPG	6.85
TCGA-QR-A6H3-01A	PCPG	5.7
TCGA-SR-A6MQ-01A	PCPG	6.95
TCGA-TT-A6YK-01A	PCPG	6.38
TCGA-S7-A7WT-01A	PCPG	6.76
TCGA-S7-A7X2-01A	PCPG	6.81
TCGA-SP-A6QJ-01A	PCPG	6.78
TCGA-P7-A5NY-01A	PCPG	6.75
TCGA-RW-A680-01A	PCPG	5.29
TCGA-RW-A67W-01A	PCPG	6.11
TCGA-QR-A6H4-01A	PCPG	6.74
TCGA-S7-A7WV-01A	PCPG	6.55
TCGA-WB-A817-01A	PCPG	7.46
TCGA-SR-A6MX-06A	PCPG	6.05
TCGA-S7-A7X1-01A	PCPG	6.51
TCGA-W2-A7UY-01A	PCPG	6.15

TCGA-TT-A6YP-01A	PCPG	6.95
TCGA-WB-A80N-01A	PCPG	6.64
TCGA-WB-A80Q-01A	PCPG	6.38
TCGA-SR-A6MY-01A	PCPG	7.01
TCGA-QR-A6GY-01A	PCPG	6.4
TCGA-SP-A6QH-01A	PCPG	6.77
TCGA-S7-A7WN-01A	PCPG	6.67
TCGA-S7-A7WO-01A	PCPG	7.51
TCGA-P8-A5KD-01A	PCPG	7.16
TCGA-W2-A7HB-01A	PCPG	7.44
TCGA-RW-A684-01A	PCPG	6.48
TCGA-PR-A5PG-01A	PCPG	7.14
TCGA-RM-A68T-01A	PCPG	6.02
TCGA-SR-A6MS-01A	PCPG	6.55
TCGA-WB-A80O-01A	PCPG	6.53
TCGA-QT-A5XJ-01A	PCPG	6.99
TCGA-P7-A5NY-05A	PCPG	7.3
TCGA-W2-A7HH-01A	PCPG	6.8
TCGA-P8-A6RY-01A	PCPG	5.07
TCGA-RM-A68W-01A	PCPG	5.81
TCGA-RW-A685-01A	PCPG	6.14
TCGA-WB-A81T-01A	PCPG	6.77
TCGA-QR-A6GU-01A	PCPG	5.86
TCGA-QR-A70T-01A	PCPG	6.1
TCGA-RW-A7D0-01A	PCPG	6.58
TCGA-QR-A70E-01A	PCPG	6.16
TCGA-RW-A7CZ-01A	PCPG	6.06
TCGA-WB-A81I-01A	PCPG	6.5
TCGA-QR-A70K-01A	PCPG	6.62
TCGA-SP-A6QD-01A	PCPG	5.22
TCGA-QR-A7IN-01A	PCPG	6.39
TCGA-S7-A7X0-01A	PCPG	6.59
TCGA-WB-A81P-01A	PCPG	6.83
TCGA-QR-A705-01A	PCPG	5.1
TCGA-W2-A7H5-01B	PCPG	7.29
TCGA-RT-A6YC-01A	PCPG	7.13
TCGA-QR-A706-01A	PCPG	5.86
TCGA-QR-A700-01A	PCPG	5.61
TCGA-RX-A8JQ-01A	PCPG	6.53
TCGA-QR-A70C-01A	PCPG	5.92
TCGA-WB-A818-01A	PCPG	7.19
TCGA-WB-A81Q-01A	PCPG	6.49
TCGA-SQ-A6I6-01A	PCPG	7.23
TCGA-WB-A819-01A	PCPG	6.89
TCGA-W2-A7HC-01A	PCPG	6.79
TCGA-QT-A5XK-01A	PCPG	6.57
TCGA-SR-A6MV-01A	PCPG	6.37
TCGA-WB-A81H-01A	PCPG	7
TCGA-WB-A81G-01A	PCPG	6.2
TCGA-WB-A80Y-01A	PCPG	6.22
TCGA-QR-A6GW-01A	PCPG	7.32
TCGA-QR-A70A-01A	PCPG	5.54
TCGA-SP-A6QG-01A	PCPG	6.53
TCGA-RW-A686-01A	PCPG	6.42
TCGA-QR-A70P-01A	PCPG	5.88
TCGA-SP-A6QC-01A	PCPG	6.61
TCGA-P8-A6RX-01A	PCPG	6.75
TCGA-SA-A6C2-01A	PCPG	7.23

TCGA-WB-A814-01A	PCPG	6.5
TCGA-P8-A5KC-01A	PCPG	7.41
TCGA-RW-A686-06A	PCPG	6.27
TCGA-QR-A70N-01A	PCPG	6.35
TCGA-RW-A688-01A	PCPG	7.08
TCGA-QT-A7U0-01A	PCPG	6.58
TCGA-WB-A81E-01A	PCPG	6.69
TCGA-WB-A821-01A	PCPG	6.97
TCGA-WB-A80M-01A	PCPG	6.75
TCGA-QR-A6H6-01A	PCPG	5.96
TCGA-S7-A7WR-01A	PCPG	7.12
TCGA-SP-A6QI-01A	PCPG	6.53
TCGA-WB-A80L-01A	PCPG	7.22
TCGA-J9-A8CL-01A	PRAD	8.71
TCGA-EJ-A46B-01A	PRAD	8.76
TCGA-EJ-5509-01A	PRAD	8.44
TCGA-EJ-7782-01A	PRAD	9.35
TCGA-J9-A52C-01A	PRAD	7.8
TCGA-G9-6356-01A	PRAD	7.54
TCGA-EJ-7783-01A	PRAD	8.54
TCGA-KK-A8IG-01A	PRAD	8.58
TCGA-EJ-5505-01A	PRAD	9.36
TCGA-EJ-7789-01A	PRAD	8.95
TCGA-XK-AAK1-01A	PRAD	8.83
TCGA-YL-A9WH-01A	PRAD	6.6
TCGA-EJ-5516-01A	PRAD	8.15
TCGA-YL-A8SL-01B	PRAD	7.64
TCGA-YL-A8SQ-01B	PRAD	8.42
TCGA-J4-A83K-01A	PRAD	8.35
TCGA-KK-A6E7-01A	PRAD	7.34
TCGA-KK-A7B2-01A	PRAD	7.51
TCGA-HC-7213-01A	PRAD	4.62
TCGA-HC-8257-01A	PRAD	8.98
TCGA-G9-6343-01A	PRAD	8.1
TCGA-HC-A8D1-01A	PRAD	8.15
TCGA-YL-A8HK-01A	PRAD	7.21
TCGA-XK-AAJP-01A	PRAD	8.77
TCGA-CH-5751-01A	PRAD	8.6
TCGA-KK-A7B1-01A	PRAD	7.99
TCGA-G9-6354-01A	PRAD	8.34
TCGA-G9-6333-01A	PRAD	9.19
TCGA-EJ-5498-01A	PRAD	8.8
TCGA-KK-A8ID-01A	PRAD	8.63
TCGA-V1-A8ML-01A	PRAD	8.57
TCGA-EJ-A65G-01A	PRAD	8.63
TCGA-KC-A7F3-01A	PRAD	8.74
TCGA-G9-7522-01A	PRAD	8.51
TCGA-KC-A7FD-01A	PRAD	8.35
TCGA-EJ-5501-01A	PRAD	8.18
TCGA-HC-A6AO-01A	PRAD	7.73
TCGA-CH-5744-01A	PRAD	7.13
TCGA-KC-A7FA-01A	PRAD	7.74
TCGA-V1-A9O7-01A	PRAD	8.11
TCGA-EJ-7328-01A	PRAD	7.91
TCGA-HC-8213-01A	PRAD	8.66
TCGA-CH-5737-01A	PRAD	7.92
TCGA-EJ-5525-01A	PRAD	9.13
TCGA-HC-A9TH-01A	PRAD	6.71

TCGA-X4-A8KQ-01A	PRAD	7.36
TCGA-XJ-A83H-01A	PRAD	8.06
TCGA-4L-AA1F-01A	PRAD	6.81
TCGA-XJ-A9DK-01A	PRAD	7.87
TCGA-EJ-5542-01A	PRAD	8.42
TCGA-J9-A8CP-01A	PRAD	8.76
TCGA-EJ-5496-01A	PRAD	9.06
TCGA-HI-7170-01A	PRAD	7.84
TCGA-J4-A67M-01A	PRAD	7.5
TCGA-EJ-7791-01A	PRAD	8.2
TCGA-KK-A8IM-01A	PRAD	7.5
TCGA-M7-A720-01A	PRAD	8.23
TCGA-EJ-7325-01B	PRAD	7.39
TCGA-2A-A8VL-01A	PRAD	8.64
TCGA-XQ-A8TB-01A	PRAD	7.35
TCGA-YL-A8S8-01A	PRAD	8.03
TCGA-HC-7075-01A	PRAD	8.75
TCGA-CH-5738-01A	PRAD	8.52
TCGA-EJ-7318-01B	PRAD	8.26
TCGA-EJ-7784-01A	PRAD	8.88
TCGA-ZG-A9L2-01A	PRAD	7.41
TCGA-KK-A59X-01A	PRAD	8.56
TCGA-KK-A59Y-01A	PRAD	8.41
TCGA-J4-AATZ-01A	PRAD	7.7
TCGA-VN-A88P-01A	PRAD	8.13
TCGA-HC-A6AS-01A	PRAD	7.59
TCGA-EJ-AB20-01A	PRAD	7.05
TCGA-KK-A8I6-01A	PRAD	7.32
TCGA-VN-A88L-01A	PRAD	7.61
TCGA-G9-6361-01A	PRAD	7.83
TCGA-VP-A87C-01A	PRAD	8.31
TCGA-HC-7738-01A	PRAD	9.05
TCGA-HC-8216-01A	PRAD	8.78
TCGA-EJ-7321-01A	PRAD	8.69
TCGA-HC-7077-01A	PRAD	8.39
TCGA-KK-A7B0-01A	PRAD	7.46
TCGA-HC-7737-01A	PRAD	7.91
TCGA-2A-A8VV-01A	PRAD	8.53
TCGA-FC-A66V-01A	PRAD	7.81
TCGA-CH-5739-01A	PRAD	8.63
TCGA-H9-A6BY-01A	PRAD	7.91
TCGA-G9-7509-01A	PRAD	8.84
TCGA-EJ-7317-01A	PRAD	8.69
TCGA-KK-A7AZ-01A	PRAD	7.95
TCGA-V1-A9ZK-01A	PRAD	8.54
TCGA-J4-8200-01A	PRAD	8.66
TCGA-CH-5761-01A	PRAD	9.42
TCGA-KC-A7FE-01A	PRAD	8.39
TCGA-EJ-A65F-01A	PRAD	8.88
TCGA-CH-5767-01A	PRAD	9.11
TCGA-EJ-A65J-01A	PRAD	7.77
TCGA-ZG-A9LM-01A	PRAD	7.58
TCGA-YJ-A8SW-01A	PRAD	8.08
TCGA-VP-A87H-01A	PRAD	8.33
TCGA-V1-A9OH-01A	PRAD	7.81
TCGA-TP-A8TV-01A	PRAD	8.59
TCGA-HC-7745-01A	PRAD	7.75
TCGA-VP-A876-01A	PRAD	8.55

TCGA-YL-A8HJ-01A	PRAD	7.88
TCGA-KK-A8I5-01A	PRAD	7.59
TCGA-CH-5791-01A	PRAD	8.37
TCGA-EJ-7330-01A	PRAD	8.86
TCGA-FC-A8O0-01A	PRAD	7.4
TCGA-HC-8256-01A	PRAD	8.93
TCGA-J9-A52D-01A	PRAD	9.25
TCGA-EJ-5512-01A	PRAD	8.98
TCGA-HC-A8CY-01A	PRAD	9.15
TCGA-EJ-AB27-01A	PRAD	8.71
TCGA-G9-6373-01A	PRAD	7.58
TCGA-G9-6332-01A	PRAD	7.92
TCGA-EJ-8474-01A	PRAD	8.37
TCGA-J4-A83M-01A	PRAD	7.85
TCGA-G9-7521-01A	PRAD	7.71
TCGA-XJ-A83F-01A	PRAD	8.01
TCGA-EJ-7218-01B	PRAD	8.77
TCGA-EJ-5521-01A	PRAD	7.84
TCGA-CH-5750-01A	PRAD	8.6
TCGA-YL-A9WY-01A	PRAD	7.08
TCGA-V1-A9OF-01A	PRAD	9.02
TCGA-V1-A8WW-01A	PRAD	7.47
TCGA-EJ-A65M-01A	PRAD	8.68
TCGA-XJ-A83G-01A	PRAD	8.48
TCGA-XK-AAJA-01A	PRAD	8.38
TCGA-EJ-5497-01A	PRAD	8.89
TCGA-EJ-A7NK-01A	PRAD	8.06
TCGA-EJ-A8FO-01A	PRAD	8.73
TCGA-YL-A8HO-01A	PRAD	7.76
TCGA-G9-6498-01A	PRAD	7.21
TCGA-VN-A88I-01A	PRAD	7.75
TCGA-EJ-5527-01A	PRAD	8.38
TCGA-G9-6353-01A	PRAD	8.27
TCGA-HC-A9TE-01A	PRAD	6.26
TCGA-QU-A6IM-01A	PRAD	7.88
TCGA-EJ-A7NN-01A	PRAD	7.87
TCGA-KK-A8II-01A	PRAD	8.17
TCGA-CH-5789-01A	PRAD	8.63
TCGA-YL-A8SR-01B	PRAD	8.81
TCGA-V1-A8WS-01A	PRAD	8.22
TCGA-KK-A6E4-01A	PRAD	8.16
TCGA-EJ-5506-01A	PRAD	8.78
TCGA-G9-6369-01A	PRAD	9.02
TCGA-J4-A83J-01A	PRAD	7.48
TCGA-KK-A8IJ-01A	PRAD	8.57
TCGA-G9-6348-01A	PRAD	7.51
TCGA-G9-6329-01A	PRAD	7.97
TCGA-HC-7752-01A	PRAD	6.55
TCGA-HC-7736-01A	PRAD	8.69
TCGA-VN-A88K-01A	PRAD	8.2
TCGA-J4-8198-01A	PRAD	8.56
TCGA-MG-AAMC-01A	PRAD	7.16
TCGA-KK-A59V-01A	PRAD	8.11
TCGA-XK-AAJR-01A	PRAD	8.61
TCGA-EJ-A65D-01A	PRAD	7.53
TCGA-V1-A8WV-01A	PRAD	8.07
TCGA-HC-7231-01A	PRAD	7.7
TCGA-EJ-5531-01A	PRAD	8.71

TCGA-YL-A9WK-01A	PRAD	7.83
TCGA-CH-5741-01A	PRAD	8.6
TCGA-WW-A8ZI-01A	PRAD	7.97
TCGA-VN-A88O-01A	PRAD	8.66
TCGA-ZG-A9LB-01A	PRAD	8.14
TCGA-YL-A8HM-01A	PRAD	9.02
TCGA-VP-A875-01A	PRAD	8.63
TCGA-EJ-7327-01A	PRAD	8.2
TCGA-VN-A88N-01A	PRAD	8.42
TCGA-KK-A59Z-01A	PRAD	7.64
TCGA-HC-7747-01A	PRAD	8.48
TCGA-2A-A8W1-01A	PRAD	9.29
TCGA-V1-A9O5-01A	PRAD	7.16
TCGA-EJ-A7NF-01A	PRAD	8.24
TCGA-HC-8266-01A	PRAD	7.93
TCGA-KK-A7AY-01A	PRAD	6.82
TCGA-HC-7744-01A	PRAD	8.51
TCGA-V1-A9O9-01A	PRAD	7.79
TCGA-V1-A8MJ-01A	PRAD	7.86
TCGA-HC-A4ZV-01A	PRAD	8.77
TCGA-EJ-5532-01A	PRAD	9.53
TCGA-ZG-A8QX-01A	PRAD	8.37
TCGA-XQ-A8TA-01A	PRAD	6.49
TCGA-FC-7961-01A	PRAD	7.61
TCGA-G9-6347-01A	PRAD	7.3
TCGA-ZG-A8QZ-01A	PRAD	7.29
TCGA-KK-A7AQ-01A	PRAD	6.87
TCGA-EJ-7115-01A	PRAD	8.76
TCGA-J4-A67R-01A	PRAD	7.3
TCGA-HC-A631-01A	PRAD	7.65
TCGA-VP-A87E-01A	PRAD	8.08
TCGA-CH-5740-01A	PRAD	8.94
TCGA-KK-A6E1-01A	PRAD	6.61
TCGA-G9-6385-01A	PRAD	8.46
TCGA-HC-7211-01A	PRAD	8.96
TCGA-ZG-A9L5-01A	PRAD	8.65
TCGA-HC-8261-01B	PRAD	7.3
TCGA-EJ-A8FN-01A	PRAD	8.41
TCGA-QU-A6IL-01A	PRAD	8.33
TCGA-HC-A632-01A	PRAD	8.71
TCGA-HC-A6HX-01A	PRAD	7.28
TCGA-J4-AATV-01A	PRAD	7.63
TCGA-M7-A724-01A	PRAD	8.32
TCGA-YL-A8HL-01A	PRAD	7.64
TCGA-G9-6364-01A	PRAD	7.7
TCGA-EJ-7331-01A	PRAD	9.09
TCGA-KC-A4BV-01A	PRAD	7.8
TCGA-J4-A67K-01A	PRAD	7.3
TCGA-YL-A8SP-01B	PRAD	8.31
TCGA-EJ-7794-01A	PRAD	8.83
TCGA-YL-A8SI-01A	PRAD	8.37
TCGA-EJ-A46H-01A	PRAD	8.07
TCGA-HC-7819-01A	PRAD	8.71
TCGA-G9-6371-01A	PRAD	8.57
TCGA-ZG-A9L9-01A	PRAD	6.71
TCGA-EJ-5508-01A	PRAD	8.63
TCGA-G9-6339-01A	PRAD	7.96
TCGA-YL-A9WL-01A	PRAD	7.65

TCGA-V1-A8WN-01A	PRAD	8.58
TCGA-KK-A6E0-01A	PRAD	9.25
TCGA-Y6-A9XI-01A	PRAD	8.39
TCGA-G9-6496-01A	PRAD	8.22
TCGA-HC-7232-01A	PRAD	8.05
TCGA-ZG-A9L4-01A	PRAD	7.1
TCGA-G9-6379-01A	PRAD	7.21
TCGA-ZG-A9LZ-01A	PRAD	8.14
TCGA-QU-A6IN-01A	PRAD	7.85
TCGA-KK-A8IK-01A	PRAD	8.92
TCGA-CH-5752-01A	PRAD	8.57
TCGA-KK-A7AV-01A	PRAD	8.46
TCGA-V1-A9ZG-01A	PRAD	7.29
TCGA-J9-A8CM-01A	PRAD	7.39
TCGA-YL-A8SF-01A	PRAD	7.04
TCGA-EJ-5530-01A	PRAD	8.86
TCGA-V1-A9O5-06A	PRAD	6.83
TCGA-EJ-A7NJ-01A	PRAD	8.67
TCGA-KC-A7F6-01A	PRAD	8.06
TCGA-EJ-7797-01A	PRAD	8.64
TCGA-VN-A88R-01A	PRAD	9.07
TCGA-XK-AAJ3-01A	PRAD	8.96
TCGA-HC-8259-01A	PRAD	8.86
TCGA-G9-6494-01A	PRAD	8.87
TCGA-G9-A9S0-01A	PRAD	8.03
TCGA-EJ-A46F-01A	PRAD	6.55
TCGA-XK-AAIV-01A	PRAD	7.24
TCGA-KK-A6E5-01A	PRAD	8.56
TCGA-YL-A8SK-01B	PRAD	7.84
TCGA-HC-7749-01A	PRAD	8.74
TCGA-J9-A8CN-01A	PRAD	8.73
TCGA-EJ-5515-01A	PRAD	9.12
TCGA-EJ-8470-01A	PRAD	8.46
TCGA-EJ-A8FU-01A	PRAD	7.88
TCGA-ZG-A9LN-01A	PRAD	8.52
TCGA-YL-A8SB-01A	PRAD	8.14
TCGA-XK-AAIW-01A	PRAD	8.08
TCGA-ZG-A9N3-01A	PRAD	7.57
TCGA-EJ-7785-01A	PRAD	8.65
TCGA-J4-A67L-01A	PRAD	7.62
TCGA-V1-A9ZR-01A	PRAD	7.21
TCGA-KK-A8IL-01A	PRAD	8.19
TCGA-ZG-A9ND-01A	PRAD	8.61
TCGA-G9-6377-01A	PRAD	8.09
TCGA-M7-A722-01A	PRAD	8.06
TCGA-XJ-A9DI-01A	PRAD	7.35
TCGA-HC-8265-01A	PRAD	8.29
TCGA-KC-A7F5-01A	PRAD	8.73
TCGA-YL-A8SC-01A	PRAD	7.96
TCGA-G9-6338-01A	PRAD	7.62
TCGA-HI-7168-01A	PRAD	8.08
TCGA-KK-A8I9-01A	PRAD	8.32
TCGA-EJ-7315-01A	PRAD	7.43
TCGA-HC-7750-01A	PRAD	8.29
TCGA-V1-A8MG-01A	PRAD	8.33
TCGA-CH-5771-01A	PRAD	8.21
TCGA-KK-A6E3-01A	PRAD	7.96
TCGA-EJ-5518-01A	PRAD	8.71

TCGA-V1-A9ZI-01A	PRAD	7.76
TCGA-EJ-7312-01B	PRAD	8.53
TCGA-HC-8260-01A	PRAD	8.55
TCGA-HC-8265-01B	PRAD	7.43
TCGA-CH-5743-01A	PRAD	7.61
TCGA-FC-A4JI-01A	PRAD	8.59
TCGA-EJ-7792-01A	PRAD	8.48
TCGA-EJ-7781-01A	PRAD	9.11
TCGA-ZG-A9L0-01A	PRAD	8.37
TCGA-KK-A7AU-01A	PRAD	7.52
TCGA-HC-7081-01A	PRAD	8.01
TCGA-G9-6342-01A	PRAD	7.94
TCGA-HC-A6AQ-01A	PRAD	7.66
TCGA-HC-A6AN-01A	PRAD	8.04
TCGA-J4-A67N-01A	PRAD	6.93
TCGA-KC-A4BR-01A	PRAD	7.84
TCGA-VP-A879-01A	PRAD	8.02
TCGA-CH-5754-01A	PRAD	6.89
TCGA-M7-A71Y-01A	PRAD	8.06
TCGA-V1-A9OQ-01A	PRAD	8.52
TCGA-G9-6378-01A	PRAD	8.19
TCGA-VP-A87B-01A	PRAD	8.13
TCGA-CH-5763-01A	PRAD	8.39
TCGA-HI-7169-01A	PRAD	6.34
TCGA-G9-7519-01A	PRAD	8.63
TCGA-V1-A9OT-01A	PRAD	7
TCGA-HC-A76X-01A	PRAD	8.71
TCGA-J4-A67Q-01A	PRAD	7.6
TCGA-FC-7708-01A	PRAD	8
TCGA-EJ-5522-01A	PRAD	8.9
TCGA-VP-A87K-01A	PRAD	8.55
TCGA-SU-A7E7-01A	PRAD	7.83
TCGA-YL-A9WI-01A	PRAD	8.09
TCGA-J9-A52E-01A	PRAD	7.47
TCGA-G9-A9S7-01A	PRAD	7.36
TCGA-VN-A88M-01A	PRAD	8.09
TCGA-KK-A8I8-01A	PRAD	8.24
TCGA-J9-A52B-01A	PRAD	7.9
TCGA-YL-A8SA-01A	PRAD	7.88
TCGA-KK-A7AW-01A	PRAD	7.49
TCGA-EJ-A65E-01A	PRAD	8.97
TCGA-EJ-8468-01A	PRAD	9.09
TCGA-H9-A6BX-01A	PRAD	7.71
TCGA-HC-A6HY-01A	PRAD	8.39
TCGA-CH-5790-01A	PRAD	8.41
TCGA-KK-A8IF-01A	PRAD	8.71
TCGA-ZG-A9LY-01A	PRAD	7.98
TCGA-EJ-A8FP-01A	PRAD	8.04
TCGA-J4-A67T-01A	PRAD	8.47
TCGA-KK-A8I4-01A	PRAD	7.77
TCGA-EJ-A46D-01A	PRAD	8.37
TCGA-G9-6367-01A	PRAD	8.62
TCGA-2A-A8VT-01A	PRAD	8.17
TCGA-HC-7078-01A	PRAD	8.47
TCGA-HI-7171-01A	PRAD	8.66
TCGA-J4-A67S-01A	PRAD	7.07
TCGA-ZG-A8QY-01A	PRAD	8.66
TCGA-EJ-A46G-01A	PRAD	7.88

TCGA-G9-A9S4-01A	PRAD	8.96
TCGA-KK-A8IB-01A	PRAD	7.41
TCGA-EJ-7314-01A	PRAD	8.3
TCGA-V1-A8MK-01A	PRAD	8.55
TCGA-TP-A8TT-01A	PRAD	7.27
TCGA-QU-A6IP-01A	PRAD	8.3
TCGA-V1-A9Z8-01A	PRAD	7.06
TCGA-CH-5753-01A	PRAD	8.95
TCGA-HC-7821-01A	PRAD	8.81
TCGA-G9-6384-01A	PRAD	8.56
TCGA-ZG-A9L6-01A	PRAD	8.09
TCGA-CH-5745-01A	PRAD	8.31
TCGA-V1-A8MU-01A	PRAD	8.46
TCGA-EJ-7125-01A	PRAD	9.08
TCGA-EJ-7123-01A	PRAD	8.91
TCGA-G9-6363-01A	PRAD	8.1
TCGA-EJ-7786-01A	PRAD	9.08
TCGA-J4-A6G3-01A	PRAD	8.15
TCGA-EJ-A46I-01A	PRAD	8.5
TCGA-VN-A943-01A	PRAD	9.08
TCGA-ZG-A9M4-01A	PRAD	7.62
TCGA-YL-A9WX-01A	PRAD	7.97
TCGA-Y6-A8TL-01A	PRAD	8.64
TCGA-2A-A8VX-01A	PRAD	8.75
TCGA-2A-AAYU-01A	PRAD	7.68
TCGA-EJ-5503-01A	PRAD	8.43
TCGA-CH-5766-01A	PRAD	7.76
TCGA-EJ-A46E-01A	PRAD	7.71
TCGA-KK-A6DY-01A	PRAD	7.9
TCGA-KK-A7B3-01A	PRAD	7.85
TCGA-V1-A9OA-01A	PRAD	7.53
TCGA-VN-A88Q-01A	PRAD	8.15
TCGA-J4-A67O-01A	PRAD	7.67
TCGA-EJ-A6RA-01A	PRAD	8.39
TCGA-KK-A7B4-01A	PRAD	7.43
TCGA-TK-A8OK-01A	PRAD	7.57
TCGA-KK-A5A1-01A	PRAD	9.13
TCGA-HC-7209-01A	PRAD	7.31
TCGA-KK-A6E2-01A	PRAD	8.83
TCGA-KC-A4BN-01A	PRAD	9.03
TCGA-V1-A8MF-01A	PRAD	7.85
TCGA-EJ-5519-01A	PRAD	7.56
TCGA-QU-A6IO-01A	PRAD	7.59
TCGA-EJ-5510-01A	PRAD	8.37
TCGA-2A-A8W3-01A	PRAD	8.46
TCGA-HC-A76W-01A	PRAD	7.97
TCGA-HC-7820-01A	PRAD	8.72
TCGA-G9-7523-01A	PRAD	7.71
TCGA-V1-A8MM-01A	PRAD	8.26
TCGA-VP-A87D-01A	PRAD	8.64
TCGA-EJ-A7NM-01A	PRAD	7.86
TCGA-CH-5769-01A	PRAD	7.36
TCGA-ZG-A9LS-01A	PRAD	7.95
TCGA-EJ-A8FS-01A	PRAD	9.21
TCGA-EJ-5495-01A	PRAD	7.74
TCGA-ZG-A9KY-01A	PRAD	7.93
TCGA-EJ-8472-01A	PRAD	8.36
TCGA-ZG-A9NI-01A	PRAD	7.69

TCGA-HC-7230-01A	PRAD	8.2
TCGA-EJ-5524-01A	PRAD	8.6
TCGA-V1-A9Z7-01A	PRAD	7.77
TCGA-VP-A878-01A	PRAD	8.45
TCGA-2A-AAYF-01A	PRAD	8.97
TCGA-J4-A83I-01A	PRAD	7.81
TCGA-VP-A87J-01A	PRAD	7.28
TCGA-EJ-5502-01A	PRAD	8.39
TCGA-EJ-5517-01A	PRAD	9.23
TCGA-CH-5746-01A	PRAD	8.56
TCGA-XK-AAJT-01A	PRAD	7.44
TCGA-KK-A8IC-01A	PRAD	7.82
TCGA-G9-6365-01A	PRAD	8.21
TCGA-XJ-A9DX-01A	PRAD	8.42
TCGA-CH-5765-01A	PRAD	8.58
TCGA-ZG-A9MC-01A	PRAD	6.3
TCGA-V1-A8X3-01A	PRAD	8.62
TCGA-VP-A872-01A	PRAD	6.29
TCGA-2A-A8VO-01A	PRAD	8.21
TCGA-HC-7740-01B	PRAD	7.38
TCGA-KK-A8IH-01A	PRAD	6.95
TCGA-CH-5792-01A	PRAD	7.71
TCGA-XA-A8JR-01A	PRAD	8.48
TCGA-ZG-A9LU-01A	PRAD	7.5
TCGA-YL-A8SO-01B	PRAD	7.85
TCGA-XJ-A9DQ-01A	PRAD	8.22
TCGA-M7-A71Z-01A	PRAD	7.59
TCGA-M7-A721-01A	PRAD	7.78
TCGA-EJ-5507-01A	PRAD	8.1
TCGA-EJ-A7NG-01A	PRAD	8.28
TCGA-KK-A6E8-01A	PRAD	9.03
TCGA-2A-AAYO-01A	PRAD	8.3
TCGA-V1-A9OY-01A	PRAD	8.03
TCGA-HC-7210-01A	PRAD	7.88
TCGA-HC-7740-01A	PRAD	8.1
TCGA-J4-A83L-01A	PRAD	8.55
TCGA-HC-8264-01B	PRAD	8.77
TCGA-YL-A8SH-01B	PRAD	8.19
TCGA-J4-A83N-01A	PRAD	8.07
TCGA-CH-5788-01A	PRAD	9.06
TCGA-EJ-7793-01A	PRAD	8.78
TCGA-HC-8258-01B	PRAD	6.61
TCGA-HC-8258-01A	PRAD	8.67
TCGA-EJ-5526-01A	PRAD	9
TCGA-J9-A8CK-01A	PRAD	7.65
TCGA-HC-7818-01A	PRAD	8.32
TCGA-J4-A6M7-01A	PRAD	9
TCGA-CH-5764-01A	PRAD	8.5
TCGA-HC-7233-01A	PRAD	8.81
TCGA-VP-AA1N-01A	PRAD	7.04
TCGA-G9-7510-01A	PRAD	8.34
TCGA-G9-6362-01A	PRAD	8.66
TCGA-EJ-5511-01A	PRAD	8.8
TCGA-HC-7748-01A	PRAD	7.9
TCGA-G9-7525-01A	PRAD	7.64
TCGA-HC-8262-01A	PRAD	8.22
TCGA-M7-A725-01A	PRAD	8.48
TCGA-CH-5768-01A	PRAD	8.66

TCGA-V1-A9OL-01A	PRAD	7.41
TCGA-J4-A6G1-01A	PRAD	7.39
TCGA-KK-A8I7-01A	PRAD	8.3
TCGA-ZG-A9L1-01A	PRAD	8.37
TCGA-HC-7742-01A	PRAD	7.89
TCGA-HC-A8D0-01A	PRAD	7.95
TCGA-HC-7079-01A	PRAD	6.93
TCGA-YL-A9WJ-01A	PRAD	8.6
TCGA-EJ-A7NH-01A	PRAD	8.32
TCGA-V1-A9Z9-01A	PRAD	7.58
TCGA-H9-7775-01A	PRAD	8.7
TCGA-EJ-5504-01A	PRAD	8.85
TCGA-ZG-A8QW-01A	PRAD	7.71
TCGA-CH-5772-01A	PRAD	9.16
TCGA-J4-AAU2-01A	PRAD	8.43
TCGA-M7-A723-01A	PRAD	7.84
TCGA-XK-AAJU-01A	PRAD	8.09
TCGA-EJ-A65B-01A	PRAD	7.98
TCGA-G9-6366-01A	PRAD	8.16
TCGA-EJ-8469-01A	PRAD	9.35
TCGA-EJ-7788-01A	PRAD	8.53
TCGA-HC-A6AP-01A	PRAD	8.01
TCGA-KK-A6E6-01A	PRAD	8.14
TCGA-EJ-5514-01A	PRAD	7.33
TCGA-KK-A7AP-01A	PRAD	8.13
TCGA-G9-6370-01A	PRAD	8.36
TCGA-FC-A6HD-01A	PRAD	7.83
TCGA-FC-A5OB-01A	PRAD	9.6
TCGA-V1-A8WL-01A	PRAD	8.78
TCGA-EJ-5494-01A	PRAD	8.94
TCGA-HC-8261-01A	PRAD	8.41
TCGA-HC-7817-01B	PRAD	8.01
TCGA-G9-6351-01A	PRAD	8.6
TCGA-HC-A48F-01A	PRAD	8.26
TCGA-YL-A8SJ-01B	PRAD	7.48
TCGA-CH-5794-01A	PRAD	7.45
TCGA-G9-6499-01A	PRAD	8.43
TCGA-YL-A8S9-01A	PRAD	8.94
TCGA-EJ-A6RC-01A	PRAD	8.31
TCGA-HC-7212-01A	PRAD	8.51
TCGA-HC-A6AL-01A	PRAD	7.67
TCGA-CH-5762-01A	PRAD	8.3
TCGA-KC-A4BL-01A	PRAD	7.7
TCGA-X4-A8KS-01A	PRAD	7.72
TCGA-G9-6336-01A	PRAD	8.05
TCGA-EJ-5499-01A	PRAD	7.81
TCGA-HC-7080-01A	PRAD	9.09
TCGA-CH-5748-01A	PRAD	9.18
TCGA-KK-A8IA-01A	PRAD	7.75
TCGA-V1-A9OX-01A	PRAD	8
TCGA-XK-AAIR-01A	PRAD	8.81
TCGA-G5-6572-01A	READ	6.5
TCGA-AG-A023-01A	READ	6.27
TCGA-DC-5337-01A	READ	7.41
TCGA-AF-2693-01A	READ	7.07
TCGA-AG-3898-01A	READ	6.73
TCGA-AG-3894-01A	READ	6.82
TCGA-EI-6885-01A	READ	7.14

TCGA-EI-6510-01A	READ	6.91
TCGA-F5-6810-01A	READ	7.42
TCGA-AG-3575-01A	READ	7.93
TCGA-AF-6672-01A	READ	6.58
TCGA-AG-3896-01A	READ	7.11
TCGA-AG-A011-01A	READ	5.34
TCGA-DC-6154-01A	READ	7.04
TCGA-AG-3581-01A	READ	6.57
TCGA-DT-5265-01A	READ	7.26
TCGA-AG-3578-01A	READ	6.5
TCGA-AG-3583-01A	READ	7.43
TCGA-AF-5654-01A	READ	6.84
TCGA-AG-A00C-01A	READ	6.46
TCGA-DC-6155-01A	READ	5.9
TCGA-AG-A036-01A	READ	7.05
TCGA-AF-A56K-01A	READ	6.48
TCGA-AG-3602-01A	READ	7.19
TCGA-AH-6547-01A	READ	6.69
TCGA-AH-6644-01A	READ	7.31
TCGA-EI-6917-01A	READ	7.15
TCGA-AG-3574-01A	READ	6.98
TCGA-AG-A032-01A	READ	6.61
TCGA-CL-5918-01A	READ	6.8
TCGA-AF-2687-01A	READ	6.82
TCGA-AG-3611-01A	READ	6.84
TCGA-EI-6507-01A	READ	6.9
TCGA-EI-6508-01A	READ	7.3
TCGA-AF-A56L-01A	READ	6.79
TCGA-F5-6811-01A	READ	7.36
TCGA-EI-6511-01A	READ	7.19
TCGA-EI-6883-01A	READ	7.13
TCGA-EF-5831-01A	READ	7.25
TCGA-DY-A1DE-01A	READ	6.68
TCGA-F5-6464-01A	READ	7.14
TCGA-F5-6863-01A	READ	7.08
TCGA-AG-3887-01A	READ	6.62
TCGA-CL-4957-01A	READ	6.8
TCGA-CI-6623-01B	READ	7.62
TCGA-BM-6198-01A	READ	6.96
TCGA-EI-6506-01A	READ	7.16
TCGA-AG-3594-01A	READ	6.69
TCGA-AG-A002-01A	READ	7.08
TCGA-CL-5917-01A	READ	7.5
TCGA-AG-A014-01A	READ	6.08
TCGA-AG-3598-01A	READ	6.28
TCGA-AF-A56N-01A	READ	7.39
TCGA-G5-6641-01A	READ	6.84
TCGA-DY-A1DD-01A	READ	6.46
TCGA-EF-5830-01A	READ	6.63
TCGA-AG-3609-01A	READ	6.77
TCGA-CI-6624-01C	READ	6.93
TCGA-AG-A016-01A	READ	6.74
TCGA-AG-A01L-01A	READ	6.53
TCGA-AG-3732-01A	READ	6.9
TCGA-AG-A00H-01A	READ	6.51
TCGA-AG-4007-01A	READ	6.86
TCGA-AG-3591-01A	READ	7.46
TCGA-AG-A02X-01A	READ	6.65

TCGA-DY-A1DF-01A	READ	5.37
TCGA-EI-6884-01A	READ	7.51
TCGA-AG-3902-01A	READ	7.64
TCGA-AG-A015-01A	READ	6.65
TCGA-AF-4110-01A	READ	6.83
TCGA-AF-2691-01A	READ	6.77
TCGA-AG-3731-01A	READ	6.72
TCGA-DY-A1DG-01A	READ	7.13
TCGA-CI-6619-01B	READ	7.12
TCGA-DC-6157-01A	READ	7.12
TCGA-DC-4745-01A	READ	7.88
TCGA-AG-3584-01A	READ	6.81
TCGA-AG-4022-01A	READ	7.07
TCGA-AG-3893-01A	READ	7.1
TCGA-AG-3881-01A	READ	5.9
TCGA-DY-A1H8-01A	READ	6.52
TCGA-AG-3883-01A	READ	5.66
TCGA-AG-3882-01A	READ	5.94
TCGA-AG-3601-01A	READ	7.06
TCGA-F5-6813-01A	READ	7.28
TCGA-AG-3608-01A	READ	6.58
TCGA-F5-6702-01A	READ	6.96
TCGA-AH-6643-01A	READ	7.22
TCGA-CI-6622-01A	READ	6.16
TCGA-G5-6233-01A	READ	7.34
TCGA-AG-3742-01A	READ	7.58
TCGA-AG-3725-01A	READ	7.12
TCGA-AG-A00Y-01A	READ	6.6
TCGA-AG-3878-01A	READ	5.81
TCGA-AG-3582-01A	READ	7.14
TCGA-AG-3892-01A	READ	6.46
TCGA-AG-3587-01A	READ	6.58
TCGA-AF-3400-01A	READ	6.81
TCGA-AG-3890-01A	READ	6.44
TCGA-EI-6882-01A	READ	7.79
TCGA-F5-6812-01A	READ	6.49
TCGA-DC-5869-01A	READ	7.87
TCGA-AG-3885-01A	READ	6.37
TCGA-AF-6655-01A	READ	7.02
TCGA-AG-3727-01A	READ	5.67
TCGA-AG-A008-01A	READ	6.66
TCGA-F5-6465-01A	READ	6.56
TCGA-AG-4001-01A	READ	6.75
TCGA-F5-6861-01A	READ	7.22
TCGA-AG-4021-01A	READ	6.91
TCGA-AG-3580-01A	READ	6.99
TCGA-AG-3999-01A	READ	7.47
TCGA-AF-2692-01A	READ	7.11
TCGA-AG-4008-01A	READ	6.91
TCGA-AH-6544-01A	READ	7.31
TCGA-AG-A025-01A	READ	5.98
TCGA-AG-3728-01A	READ	5.71
TCGA-DY-A0XA-01A	READ	7.11
TCGA-F5-6571-01A	READ	7.19
TCGA-AG-A01W-01A	READ	7.48
TCGA-EI-6513-01A	READ	6.29
TCGA-AG-A01J-01A	READ	6.04
TCGA-AG-A01Y-01A	READ	6.72

TCGA-AG-3593-01A	READ	6.89
TCGA-AH-6549-01A	READ	7.3
TCGA-AF-3911-01A	READ	7.32
TCGA-AG-4015-01A	READ	6.12
TCGA-DC-6681-01A	READ	7.38
TCGA-F5-6864-01A	READ	6.67
TCGA-AF-2690-01A	READ	6.73
TCGA-EI-6509-01A	READ	6.44
TCGA-AG-A01N-01A	READ	6.3
TCGA-AG-3600-01A	READ	6.67
TCGA-AG-3599-01A	READ	6.93
TCGA-AG-A020-01A	READ	6.36
TCGA-EI-7002-01A	READ	6.96
TCGA-CI-6621-01A	READ	7.04
TCGA-DC-6160-01A	READ	7.91
TCGA-AG-3726-01A	READ	5.9
TCGA-G5-6572-02A	READ	7.89
TCGA-EI-6512-01A	READ	7.17
TCGA-AF-3913-01A	READ	7.31
TCGA-DC-6682-01A	READ	7
TCGA-AH-6897-01A	READ	7.22
TCGA-AG-A026-01A	READ	6.58
TCGA-AF-6136-01A	READ	7.26
TCGA-EI-6514-01A	READ	6.52
TCGA-AG-3901-01A	READ	6.41
TCGA-DC-6683-01A	READ	7.37
TCGA-DC-6158-01A	READ	7.61
TCGA-AG-A02G-01A	READ	6.46
TCGA-DC-6156-01A	READ	7.24
TCGA-AG-3592-01A	READ	6.8
TCGA-AG-3612-01A	READ	7.02
TCGA-AH-6903-01A	READ	7.28
TCGA-DY-A1DC-01A	READ	7.17
TCGA-AG-4005-01A	READ	7
TCGA-EI-6881-01A	READ	7.5
TCGA-AG-A02N-01A	READ	6.96
TCGA-AG-3605-01A	READ	6.66
TCGA-AG-3909-01A	READ	6.74
TCGA-AG-3586-01A	READ	6.68
TCGA-F5-6814-01A	READ	7.34
TCGA-EI-7004-01A	READ	6.8
TCGA-DC-4749-01A	READ	7.66
TCGA-G5-6235-01A	READ	7.1
TCGA-CI-6620-01A	READ	6.95
TCGA-HS-A5N7-01A	SARC	6.14
TCGA-DX-A3LS-01A	SARC	7.21
TCGA-IE-A3OV-01A	SARC	6.87
TCGA-FX-A2QS-01A	SARC	7.28
TCGA-HS-A5N9-01A	SARC	6.69
TCGA-FX-A8OO-01A	SARC	7.23
TCGA-SI-AA8B-01A	SARC	7.2
TCGA-DX-AB2W-01A	SARC	6.62
TCGA-K1-A6RT-01A	SARC	6.16
TCGA-MB-A5YA-01A	SARC	6.65
TCGA-UE-A6QU-01A	SARC	7.14
TCGA-DX-A3U9-01A	SARC	6.56
TCGA-DX-AB37-01A	SARC	6.81
TCGA-DX-A6YR-01A	SARC	6.64

TCGA-HS-A5NA-01A	SARC	7.28
TCGA-QQ-A8VH-01A	SARC	6.39
TCGA-IS-A3K7-01A	SARC	7.14
TCGA-MJ-A68H-01A	SARC	6.41
TCGA-DX-A8BK-01A	SARC	6.54
TCGA-DX-AATS-01A	SARC	6.61
TCGA-IF-A3RQ-01A	SARC	7.6
TCGA-IE-A6BZ-01A	SARC	7.59
TCGA-DX-A1L0-01A	SARC	7.23
TCGA-X6-A7WA-01A	SARC	6.41
TCGA-DX-AB3B-01A	SARC	7.47
TCGA-DX-A8BJ-01A	SARC	6.77
TCGA-X6-A8C2-01A	SARC	7.03
TCGA-WK-A8XQ-01A	SARC	7.09
TCGA-QQ-A8VD-01A	SARC	6.93
TCGA-K1-A42X-01A	SARC	6.62
TCGA-HS-A5N8-01A	SARC	6.32
TCGA-DX-A1L2-01A	SARC	7.55
TCGA-DX-A3UB-01A	SARC	5.84
TCGA-MO-A47P-01A	SARC	7.67
TCGA-PC-A5DO-01A	SARC	6.08
TCGA-DX-AB2F-01A	SARC	6.73
TCGA-FX-A3RE-01A	SARC	6.07
TCGA-DX-A8BG-01A	SARC	6.03
TCGA-DX-A23Y-01A	SARC	7.47
TCGA-QC-A7B5-01A	SARC	6.51
TCGA-3B-A9HZ-01A	SARC	6.21
TCGA-X6-A8C5-01A	SARC	6.26
TCGA-X6-A8C3-01A	SARC	7.47
TCGA-3B-A9HI-01A	SARC	6.42
TCGA-DX-A8BS-01A	SARC	6.59
TCGA-DX-A3LY-01B	SARC	7.31
TCGA-DX-A6BB-01A	SARC	5.86
TCGA-VT-AB3D-01A	SARC	6.31
TCGA-DX-A7EN-01A	SARC	7.29
TCGA-VT-A80J-02A	SARC	7.19
TCGA-DX-AB2P-01A	SARC	7.06
TCGA-3B-A9HV-01A	SARC	6.27
TCGA-DX-A8BR-01A	SARC	7.05
TCGA-QQ-A8VB-01A	SARC	6.98
TCGA-DX-A1KZ-01A	SARC	6.81
TCGA-DX-A2J0-01A	SARC	7.09
TCGA-IS-A3KA-01A	SARC	5.03
TCGA-K1-A6RU-01A	SARC	7.33
TCGA-3B-A9HS-01A	SARC	6.82
TCGA-LI-A67I-01A	SARC	6.81
TCGA-WK-A8XY-01A	SARC	6.35
TCGA-IW-A3M6-01A	SARC	6.83
TCGA-WK-A8XX-01A	SARC	7.42
TCGA-DX-A3UF-01A	SARC	6.4
TCGA-DX-A8BZ-01A	SARC	6.65
TCGA-DX-AB2J-01A	SARC	6.53
TCGA-WP-A9GB-01A	SARC	6.39
TCGA-IE-A4EJ-01A	SARC	7.12
TCGA-Z4-A9VC-01A	SARC	7.03
TCGA-DX-A6YQ-01A	SARC	7.18
TCGA-IE-A4EK-01A	SARC	6.19
TCGA-DX-AB2H-01A	SARC	7.63

TCGA-HB-A3L4-01A	SARC	7.14
TCGA-X2-A95T-01A	SARC	7.22
TCGA-K1-A42W-01A	SARC	6.54
TCGA-DX-A48J-01A	SARC	6.78
TCGA-3B-A9HQ-01A	SARC	5.96
TCGA-X6-A7WB-01A	SARC	5.39
TCGA-QQ-A5VA-01A	SARC	6.61
TCGA-DX-AB36-01A	SARC	7.43
TCGA-DX-A7EF-01A	SARC	7.72
TCGA-VT-A80G-01A	SARC	7.48
TCGA-N1-A6IA-01A	SARC	6.85
TCGA-K1-A42X-02A	SARC	6.65
TCGA-X6-A8C6-01A	SARC	6.91
TCGA-DX-A6B8-01A	SARC	3.77
TCGA-DX-A7ES-01A	SARC	5.94
TCGA-DX-AB2X-01A	SARC	7.62
TCGA-QQ-A8VG-01A	SARC	7.08
TCGA-DX-A48K-01A	SARC	6.32
TCGA-IE-A4EH-01A	SARC	6.62
TCGA-DX-A6BH-01A	SARC	6.92
TCGA-DX-AB2G-01A	SARC	7.5
TCGA-SI-A71P-01A	SARC	6.98
TCGA-DX-A23Z-01A	SARC	6.85
TCGA-DX-A240-01A	SARC	6.85
TCGA-IF-A4AJ-01A	SARC	5.8
TCGA-PT-A8TR-01A	SARC	6.39
TCGA-Z4-AAPG-01A	SARC	7.07
TCGA-DX-A3U6-01A	SARC	6.78
TCGA-DX-AB30-01A	SARC	6.88
TCGA-DX-A2IZ-01A	SARC	7.87
TCGA-DX-A1KX-01A	SARC	7.1
TCGA-DX-A3M2-01A	SARC	6.61
TCGA-MO-A47R-01A	SARC	6.77
TCGA-SI-AA8C-01A	SARC	6.96
TCGA-LI-A9QH-01A	SARC	7.51
TCGA-MB-A8JL-01A	SARC	7.53
TCGA-FX-A3NJ-01A	SARC	7.03
TCGA-DX-AB3C-01A	SARC	7.7
TCGA-DX-A6BG-01A	SARC	6.87
TCGA-UE-A6QT-01A	SARC	6.65
TCGA-DX-A6YV-01A	SARC	7.3
TCGA-MB-A8JK-01A	SARC	6.44
TCGA-DX-A48N-01A	SARC	6.13
TCGA-DX-A3UC-01A	SARC	6.02
TCGA-DX-A1L4-01A	SARC	7.01
TCGA-DX-A23U-01A	SARC	7.4
TCGA-X9-A973-01A	SARC	6.89
TCGA-PC-A5DL-01A	SARC	7.23
TCGA-DX-A3U5-01A	SARC	7.56
TCGA-DX-A8BP-01A	SARC	7.45
TCGA-DX-A1L3-01A	SARC	7.17
TCGA-DX-AB2Z-01A	SARC	7.36
TCGA-DX-A3UD-01A	SARC	5.71
TCGA-DX-A48R-01A	SARC	6.19
TCGA-DX-A7EO-01A	SARC	6.78
TCGA-QQ-A5V9-01A	SARC	6.26
TCGA-SI-A71O-06A	SARC	6.85
TCGA-QQ-A5V2-01A	SARC	6.8

TCGA-DX-A6Z0-01A	SARC	7
TCGA-KF-A41W-01A	SARC	6.91
TCGA-KD-A5QT-01A	SARC	6.62
TCGA-3B-A9HU-01A	SARC	6.24
TCGA-DX-AB3A-01A	SARC	7.27
TCGA-DX-A6B9-01A	SARC	6.24
TCGA-3B-A9I3-01A	SARC	6.74
TCGA-PC-A5DK-01A	SARC	7.86
TCGA-DX-A1KW-01A	SARC	7.15
TCGA-JV-A5VF-01A	SARC	6.46
TCGA-X6-A8C7-01A	SARC	7.79
TCGA-Z4-AAPF-01A	SARC	6.53
TCGA-PC-A5DN-01A	SARC	7
TCGA-HB-A5W3-01A	SARC	5.86
TCGA-X9-A971-01A	SARC	6.11
TCGA-IE-A4EI-01A	SARC	6.1
TCGA-IS-A3K8-01A	SARC	7.03
TCGA-X6-A8C4-01A	SARC	7.78
TCGA-DX-AB2Q-01A	SARC	6.79
TCGA-Z4-A8JB-01A	SARC	6.7
TCGA-MB-A5Y9-01A	SARC	6.29
TCGA-DX-A23T-01A	SARC	6.71
TCGA-3B-A9I0-01A	SARC	5.71
TCGA-3B-A9HJ-01A	SARC	5.94
TCGA-3B-A9HL-01A	SARC	7.63
TCGA-JV-A5VE-01A	SARC	6.16
TCGA-DX-A23R-01A	SARC	7.51
TCGA-KD-A5QS-01A	SARC	6.79
TCGA-K1-A3PN-02A	SARC	5.93
TCGA-WK-A8XO-01A	SARC	6.57
TCGA-IS-A3K6-01A	SARC	5.39
TCGA-DX-A7EM-01A	SARC	6.45
TCGA-DX-A6YX-01A	SARC	7.41
TCGA-DX-A8BL-01A	SARC	7.07
TCGA-3B-A9HR-01A	SARC	6.18
TCGA-DX-A6BF-01A	SARC	6.65
TCGA-FX-A3NK-01A	SARC	6.88
TCGA-3B-A9I1-01A	SARC	6.43
TCGA-PC-A5DM-01A	SARC	6.98
TCGA-QC-AA9N-01A	SARC	6.44
TCGA-DX-A7ER-01A	SARC	7.06
TCGA-SI-A71O-01A	SARC	6.73
TCGA-RN-A68Q-01A	SARC	6.6
TCGA-WK-A8Y0-01A	SARC	6.42
TCGA-SG-A849-01A	SARC	6.57
TCGA-IW-A3M5-01A	SARC	6.44
TCGA-JV-A75J-01A	SARC	5.19
TCGA-WK-A8XS-01A	SARC	6.39
TCGA-DX-A8BQ-01A	SARC	7.34
TCGA-DX-A48L-01A	SARC	6.15
TCGA-DX-AB32-01A	SARC	7.25
TCGA-DX-AB35-01A	SARC	7.69
TCGA-QQ-A5VD-01A	SARC	6.58
TCGA-DX-A3U7-01A	SARC	7.14
TCGA-3B-A9HO-01A	SARC	6.73
TCGA-DX-AB2O-01A	SARC	7.12
TCGA-DX-A7EL-01A	SARC	6.63
TCGA-DX-A2J4-01A	SARC	6.3

TCGA-DX-A6YT-01A	SARC	7.34
TCGA-DX-A6BE-01A	SARC	6.49
TCGA-DX-A3U8-01A	SARC	7.61
TCGA-DX-AB2E-01A	SARC	7.44
TCGA-DX-A3UA-01A	SARC	7.06
TCGA-DX-A7EU-01A	SARC	6.91
TCGA-X6-A7WD-01A	SARC	6.16
TCGA-DX-A48P-01A	SARC	6.29
TCGA-DX-A1KU-01A	SARC	7.56
TCGA-SI-A71Q-01A	SARC	7.6
TCGA-DX-A3M1-01A	SARC	7.12
TCGA-RN-AAAQ-01A	SARC	5.91
TCGA-DX-A6YS-01A	SARC	7.48
TCGA-X6-A7W8-01A	SARC	6.09
TCGA-DX-A48U-01A	SARC	6.6
TCGA-3R-A8YX-01A	SARC	7.7
TCGA-K1-A3PN-01A	SARC	6.58
TCGA-DX-A48O-01A	SARC	6.37
TCGA-DX-A3LU-01A	SARC	6.22
TCGA-DX-A3LT-01A	SARC	7.52
TCGA-DX-AB2S-01A	SARC	6.45
TCGA-DX-A8BT-01A	SARC	6.8
TCGA-DX-A1KY-01A	SARC	7.57
TCGA-PC-A5DP-01A	SARC	6.39
TCGA-3B-A9HP-01A	SARC	6.2
TCGA-K1-A6RV-01A	SARC	6.53
TCGA-IF-A4AK-01A	SARC	6.24
TCGA-DX-A8BM-01A	SARC	8.15
TCGA-3B-A9HX-01A	SARC	6.08
TCGA-HB-A43Z-01A	SARC	5.68
TCGA-HB-A2OT-01A	SARC	6.75
TCGA-QC-A6FX-01A	SARC	6.63
TCGA-DX-AB2V-01A	SARC	6.34
TCGA-WK-A8XZ-01A	SARC	4.58
TCGA-DX-A6B7-01A	SARC	6.85
TCGA-FX-A48G-01A	SARC	5.74
TCGA-DX-A8BO-01A	SARC	6.57
TCGA-IW-A3M4-01A	SARC	6.95
TCGA-MJ-A850-01A	SARC	7.04
TCGA-K1-A3PO-01A	SARC	6.98
TCGA-DX-AB2T-01A	SARC	7.52
TCGA-DX-A3LW-01A	SARC	7.05
TCGA-MJ-A68J-01A	SARC	7.25
TCGA-DX-A7EQ-01A	SARC	6.61
TCGA-FX-A3TO-01A	SARC	7.58
TCGA-X6-A7WC-01A	SARC	6.42
TCGA-QQ-A5VB-01A	SARC	6.58
TCGA-DX-A23V-01A	SARC	7.4
TCGA-DX-A2J1-01A	SARC	6.93
TCGA-SG-A6Z7-01A	SARC	6.81
TCGA-DX-A8BV-01A	SARC	7.38
TCGA-DX-A6YU-01A	SARC	6.95
TCGA-SG-A6Z4-01A	SARC	7.04
TCGA-HB-A3YV-01A	SARC	6.83
TCGA-DX-A6Z2-01A	SARC	6.42
TCGA-DX-AB2L-01A	SARC	6.72
TCGA-FX-A76Y-01A	SARC	7.15
TCGA-QQ-A8VF-01A	SARC	7.54

TCGA-WK-A8XT-01A	SARC	7.35
TCGA-DX-A7ET-01A	SARC	6.42
TCGA-MB-A5Y8-01A	SARC	6.86
TCGA-DX-A6YZ-01A	SARC	6.62
TCGA-DX-A8BX-01A	SARC	7.06
TCGA-3B-A9HY-01A	SARC	6.16
TCGA-DX-A7EI-01A	SARC	6.93
TCGA-KD-A5QU-01A	SARC	6.88
TCGA-DX-A6BA-01A	SARC	6.37
TCGA-QQ-A5VC-01A	SARC	6.53
TCGA-DX-A8BN-01A	SARC	7.46
TCGA-VT-A80J-01A	SARC	7.51
TCGA-DX-A8BU-01A	SARC	7.33
TCGA-DX-A3UE-01A	SARC	5.26
TCGA-DX-A8BH-01A	SARC	6.91
TCGA-DX-A1L1-01A	SARC	7.69
TCGA-3B-A9HT-01A	SARC	7.59
TCGA-LH-A9QB-06A	SKCM	7.27
TCGA-GN-A268-06A	SKCM	7.62
TCGA-FR-A69P-06A	SKCM	6.52
TCGA-EE-A2A1-06A	SKCM	6.78
TCGA-YD-A9TA-06A	SKCM	6.48
TCGA-D3-A1QB-06A	SKCM	7.19
TCGA-EE-A2A6-06A	SKCM	6.59
TCGA-FS-A1YY-06A	SKCM	6.81
TCGA-D3-A5GN-06A	SKCM	6.26
TCGA-RP-A690-06A	SKCM	6.18
TCGA-EE-A2ME-06A	SKCM	6.38
TCGA-WE-AAA3-06A	SKCM	5.7
TCGA-QB-A6FS-06A	SKCM	4.74
TCGA-EE-A17X-06A	SKCM	6.3
TCGA-GN-A4U4-06A	SKCM	6.41
TCGA-XV-AAZW-01A	SKCM	5.69
TCGA-D3-A8GK-06A	SKCM	6.65
TCGA-WE-A8ZR-06A	SKCM	6.2
TCGA-GN-A8LL-06A	SKCM	6.28
TCGA-EB-A551-01A	SKCM	6.36
TCGA-GN-A264-06A	SKCM	7.23
TCGA-FS-A4FB-06A	SKCM	5.59
TCGA-WE-A8ZQ-06A	SKCM	6.49
TCGA-EE-A2GJ-06A	SKCM	6.73
TCGA-EE-A3AE-06A	SKCM	6.65
TCGA-EE-A2GL-06A	SKCM	7.09
TCGA-FS-A1ZH-06A	SKCM	5.8
TCGA-EE-A185-06A	SKCM	5.85
TCGA-FS-A1ZM-06A	SKCM	6.48
TCGA-EB-A5SH-06A	SKCM	5.15
TCGA-EB-A44N-01A	SKCM	6.12
TCGA-FS-A1ZC-06A	SKCM	6.68
TCGA-EE-A3AB-06A	SKCM	6.38
TCGA-BF-A1PX-01A	SKCM	5.96
TCGA-D3-A8GL-06A	SKCM	7.31
TCGA-D3-A8GC-06A	SKCM	7.35
TCGA-D3-A2JO-06A	SKCM	7.28
TCGA-EB-A57M-01A	SKCM	6.14
TCGA-EE-A2A2-06A	SKCM	7.02
TCGA-D3-A8GS-06A	SKCM	5.83
TCGA-EE-A2M6-06A	SKCM	7.41

TCGA-Z2-A8RT-06A	SKCM	6.91
TCGA-WE-A8ZT-06A	SKCM	5.28
TCGA-EB-A82C-01A	SKCM	7.09
TCGA-EE-A183-06A	SKCM	5.86
TCGA-EB-A44R-06A	SKCM	6.52
TCGA-EE-A2GD-06A	SKCM	6.66
TCGA-EE-A29S-06A	SKCM	7.61
TCGA-D3-A5GU-06A	SKCM	6.09
TCGA-EB-A44P-01A	SKCM	5.82
TCGA-ER-A2NH-06A	SKCM	6.21
TCGA-EB-A3XF-01A	SKCM	6.23
TCGA-D3-A3CE-06A	SKCM	6.27
TCGA-EE-A182-06A	SKCM	4.51
TCGA-D9-A3Z3-06A	SKCM	5.37
TCGA-Z2-AA3S-06A	SKCM	6.39
TCGA-FS-A4FD-06A	SKCM	7.08
TCGA-D3-A8GD-06A	SKCM	5.98
TCGA-FR-A3R1-01A	SKCM	7.22
TCGA-XV-A9W5-01A	SKCM	5.43
TCGA-EE-A2M7-06A	SKCM	6.45
TCGA-DA-A1I4-06A	SKCM	6.29
TCGA-EE-A2MJ-06A	SKCM	6.57
TCGA-ER-A42K-06A	SKCM	6.15
TCGA-DA-A1I1-06A	SKCM	6.28
TCGA-EE-A2MT-06A	SKCM	7.03
TCGA-ER-A19O-06A	SKCM	5.13
TCGA-EE-A29N-06A	SKCM	7.06
TCGA-YD-A9TB-06A	SKCM	6.7
TCGA-W3-AA1Q-06A	SKCM	6.39
TCGA-YD-A89C-06A	SKCM	6.19
TCGA-D3-A1Q1-06A	SKCM	6.53
TCGA-BF-A5EO-01A	SKCM	4.77
TCGA-D3-A1Q8-06A	SKCM	6.72
TCGA-EE-A29E-06A	SKCM	7.73
TCGA-D3-A3MO-06A	SKCM	6.01
TCGA-EE-A29G-06A	SKCM	6.33
TCGA-DA-A1IB-06A	SKCM	5.95
TCGA-BF-AAP2-01A	SKCM	7.22
TCGA-FS-A1ZU-06A	SKCM	6.74
TCGA-EE-A2GO-06A	SKCM	6.01
TCGA-FS-A1ZR-06A	SKCM	6.13
TCGA-WE-AAA4-06A	SKCM	4.8
TCGA-EE-A20I-06A	SKCM	6.52
TCGA-GN-A26D-06A	SKCM	7.6
TCGA-EB-A5VU-01A	SKCM	7.3
TCGA-D3-A51N-06A	SKCM	5.51
TCGA-D3-A3CB-06A	SKCM	6.52
TCGA-ER-A19T-06A	SKCM	6.62
TCGA-EE-A3J3-06A	SKCM	6.28
TCGA-EE-A2GI-06A	SKCM	6.19
TCGA-FS-A1ZQ-06A	SKCM	5.46
TCGA-D9-A4Z6-06A	SKCM	6.65
TCGA-ER-A3ES-06A	SKCM	6.82
TCGA-ER-A2NC-06A	SKCM	6.83
TCGA-FS-A1ZY-06A	SKCM	5.45
TCGA-FS-A4FC-06A	SKCM	7.89
TCGA-EB-A550-01A	SKCM	6.51
TCGA-D3-A3BZ-06A	SKCM	6.7

TCGA-ER-A196-01A	SKCM	6.99
TCGA-D3-A3C6-06A	SKCM	6.69
TCGA-GF-A769-01A	SKCM	6.77
TCGA-EE-A3AC-06A	SKCM	7.36
TCGA-D3-A2JF-06A	SKCM	6.61
TCGA-DA-A95X-06A	SKCM	7.38
TCGA-EE-A29H-06A	SKCM	5.75
TCGA-EE-A29D-06A	SKCM	7.23
TCGA-EE-A2GK-06A	SKCM	6.77
TCGA-EE-A29P-06A	SKCM	6.41
TCGA-EB-A5SE-01A	SKCM	7.9
TCGA-D9-A4Z2-01A	SKCM	5.97
TCGA-D3-A8GR-06A	SKCM	5.53
TCGA-ER-A19P-06A	SKCM	6.2
TCGA-D9-A148-06A	SKCM	6.1
TCGA-WE-A8ZM-06A	SKCM	5.69
TCGA-GF-A3OT-06A	SKCM	6.32
TCGA-ER-A19K-01A	SKCM	6.87
TCGA-GN-A26C-01A	SKCM	6.31
TCGA-ER-A19S-06A	SKCM	5.88
TCGA-D9-A1JX-06A	SKCM	6.86
TCGA-FS-A1ZG-06A	SKCM	7.65
TCGA-FR-A729-06A	SKCM	5.98
TCGA-D3-A3MV-06A	SKCM	6.26
TCGA-EB-A5UL-06A	SKCM	6.39
TCGA-EE-A20B-06A	SKCM	6.41
TCGA-FS-A1ZZ-06A	SKCM	7.39
TCGA-D9-A6EA-06A	SKCM	6.28
TCGA-D3-A8GO-06A	SKCM	4.64
TCGA-ER-A19C-06A	SKCM	5.58
TCGA-EB-A44Q-06A	SKCM	7.09
TCGA-EB-A3XE-01A	SKCM	5.85
TCGA-D3-A8GE-06A	SKCM	7.06
TCGA-EE-A29R-06A	SKCM	6.78
TCGA-FR-A8YC-06A	SKCM	6.46
TCGA-DA-A1I5-06A	SKCM	6.18
TCGA-D3-A1Q4-06A	SKCM	6.4
TCGA-EE-A181-06A	SKCM	6.72
TCGA-BF-A3DN-01A	SKCM	6.56
TCGA-D3-A2J8-06A	SKCM	7.2
TCGA-ER-A19W-06A	SKCM	6.32
TCGA-GF-A4EO-06A	SKCM	5.33
TCGA-W3-A824-06A	SKCM	6.8
TCGA-FR-A2OS-01A	SKCM	6.38
TCGA-EB-A41A-01A	SKCM	6.33
TCGA-EE-A29C-06A	SKCM	7.07
TCGA-EE-A2MD-06A	SKCM	6.5
TCGA-ER-A2NF-01A	SKCM	5.97
TCGA-ER-A2NF-06A	SKCM	5.72
TCGA-EB-A3Y7-01A	SKCM	4.27
TCGA-GN-A4U8-06A	SKCM	6.03
TCGA-EB-A430-01A	SKCM	5.64
TCGA-EB-A41B-01A	SKCM	7.08
TCGA-EE-A2MM-06A	SKCM	7.16
TCGA-D3-A2JC-06A	SKCM	6.12
TCGA-W3-AA1O-06A	SKCM	6.63
TCGA-FS-A1ZF-06A	SKCM	5.68
TCGA-D3-A51K-06A	SKCM	4.21

TCGA-ER-A19H-06A	SKCM	6.91
TCGA-ER-A199-06A	SKCM	6.15
TCGA-DA-A95V-06A	SKCM	4.82
TCGA-ER-A3EV-06A	SKCM	5.29
TCGA-EB-A4P0-01A	SKCM	6.3
TCGA-GN-A262-06A	SKCM	5.78
TCGA-FR-A7UA-06A	SKCM	6.07
TCGA-EB-A24D-01A	SKCM	5.99
TCGA-GN-A9SD-06A	SKCM	6.19
TCGA-EE-A17Z-06A	SKCM	5.64
TCGA-ER-A198-06A	SKCM	7.51
TCGA-ER-A193-06A	SKCM	6.51
TCGA-EB-A6QZ-01A	SKCM	6.09
TCGA-XV-A9W2-01A	SKCM	6.2
TCGA-DA-A1IC-06A	SKCM	6.95
TCGA-EE-A29B-06A	SKCM	6.5
TCGA-ER-A19G-06A	SKCM	6.86
TCGA-D3-A8GP-06A	SKCM	6.32
TCGA-EB-A553-01A	SKCM	4.62
TCGA-XV-AAZV-01A	SKCM	5.95
TCGA-GN-A8LN-01A	SKCM	6.53
TCGA-BF-AAP6-01A	SKCM	6.19
TCGA-FR-A8YE-06A	SKCM	6.19
TCGA-XV-AAZY-01A	SKCM	4.88
TCGA-D3-A2JD-06A	SKCM	5.19
TCGA-EE-A2A0-06A	SKCM	6.91
TCGA-EB-A3Y6-01A	SKCM	6
TCGA-EB-A6QY-01A	SKCM	6.53
TCGA-FS-A4F5-06A	SKCM	6.39
TCGA-EE-A2GS-06A	SKCM	6.14
TCGA-EE-A180-06A	SKCM	6.9
TCGA-ER-A3PL-06A	SKCM	6.11
TCGA-EE-A29T-06A	SKCM	6.02
TCGA-EB-A431-01A	SKCM	6.98
TCGA-EE-A2GT-06A	SKCM	6.53
TCGA-W3-AA1V-06B	SKCM	8.55
TCGA-DA-A3F2-06A	SKCM	4.33
TCGA-GN-A267-06A	SKCM	5.69
TCGA-ER-A19N-06A	SKCM	6.61
TCGA-WE-A8ZO-06A	SKCM	6.89
TCGA-WE-A8K1-06A	SKCM	6.15
TCGA-ER-A19T-01A	SKCM	7.33
TCGA-EE-A3JD-06A	SKCM	7.12
TCGA-BF-A5ER-01A	SKCM	3.98
TCGA-3N-A9WD-06A	SKCM	6.45
TCGA-W3-A828-06A	SKCM	7.16
TCGA-EE-A20C-06A	SKCM	6.81
TCGA-EE-A2MH-06A	SKCM	6.33
TCGA-ER-A2NB-01A	SKCM	4.56
TCGA-EB-A3XB-01A	SKCM	5.84
TCGA-D3-A5GR-06A	SKCM	6.28
TCGA-D3-A8GI-06A	SKCM	4.95
TCGA-WE-AAA0-06A	SKCM	7.11
TCGA-RP-A694-06A	SKCM	6.82
TCGA-EE-A29W-06A	SKCM	6.77
TCGA-EB-A3HV-01A	SKCM	5.48
TCGA-D9-A4Z5-01A	SKCM	7.04
TCGA-EE-A2MQ-06A	SKCM	7.33

TCGA-EE-A2M5-06A	SKCM	6.62
TCGA-EE-A29L-06A	SKCM	6.53
TCGA-DA-A3F8-06A	SKCM	6.39
TCGA-D3-A5GS-06A	SKCM	5.96
TCGA-GN-A4U3-06A	SKCM	5.63
TCGA-WE-A8K6-06A	SKCM	5.82
TCGA-GN-A8LK-06A	SKCM	5.89
TCGA-FW-A5DX-01A	SKCM	7.02
TCGA-BF-AAP8-01A	SKCM	6.58
TCGA-DA-A960-01A	SKCM	3.58
TCGA-EB-A82B-01A	SKCM	5.44
TCGA-D3-A51T-06A	SKCM	6.92
TCGA-EB-A4XL-01A	SKCM	6.33
TCGA-IH-A3EA-01A	SKCM	6.02
TCGA-EE-A3AA-06A	SKCM	7.26
TCGA-FS-A1ZN-01A	SKCM	5.88
TCGA-FW-A5DY-06A	SKCM	5.45
TCGA-DA-A3F5-06A	SKCM	5.8
TCGA-EB-A5UN-06A	SKCM	5.96
TCGA-EE-A3J5-06A	SKCM	7.12
TCGA-BF-AAOU-01A	SKCM	5.58
TCGA-D3-A2JB-06A	SKCM	6.73
TCGA-WE-A8K4-01A	SKCM	6.71
TCGA-D3-A1Q7-06A	SKCM	6.37
TCGA-FS-A1Z7-06A	SKCM	6.28
TCGA-DA-A1I8-06A	SKCM	5.75
TCGA-D3-A5GO-06A	SKCM	6.15
TCGA-EE-A2GH-06A	SKCM	5.86
TCGA-EE-A2GR-06A	SKCM	8.02
TCGA-EE-A2A5-06A	SKCM	6.31
TCGA-EB-A299-01A	SKCM	6.26
TCGA-D3-A51E-06A	SKCM	6.69
TCGA-D9-A1JW-06A	SKCM	6.65
TCGA-BF-A5EP-01A	SKCM	5.94
TCGA-EE-A3AH-06A	SKCM	6.41
TCGA-D3-A5GL-06A	SKCM	6.95
TCGA-EE-A2MG-06A	SKCM	6.59
TCGA-ER-A42H-01A	SKCM	7.01
TCGA-D3-A2JP-06A	SKCM	6.49
TCGA-EE-A2GB-06A	SKCM	7.1
TCGA-D3-A2JK-06A	SKCM	4.73
TCGA-OD-A75X-06A	SKCM	6.19
TCGA-EB-A85J-01A	SKCM	6.86
TCGA-FR-A3YN-06A	SKCM	5.57
TCGA-D9-A1X3-06A	SKCM	7.61
TCGA-EE-A2GP-06A	SKCM	6.92
TCGA-EB-A5VV-06A	SKCM	6.69
TCGA-EE-A3J8-06A	SKCM	7.41
TCGA-GN-A4U7-06A	SKCM	6.54
TCGA-EE-A3AD-06A	SKCM	6.21
TCGA-EB-A5SG-06A	SKCM	5.73
TCGA-FS-A1YX-06A	SKCM	6.01
TCGA-DA-A95Y-06A	SKCM	6.62
TCGA-ER-A19A-06A	SKCM	7.49
TCGA-D9-A3Z1-06A	SKCM	7.15
TCGA-DA-A95W-06A	SKCM	5.62
TCGA-EE-A2MR-06A	SKCM	6.96
TCGA-D3-A8GM-06A	SKCM	6.73

TCGA-D3-A51G-06A	SKCM	6.72
TCGA-BF-A1PZ-01A	SKCM	7.05
TCGA-FR-A726-01A	SKCM	6.95
TCGA-D3-A2JG-06A	SKCM	6.87
TCGA-D3-A8GB-06A	SKCM	5.19
TCGA-D3-A8GJ-06A	SKCM	6.17
TCGA-EE-A2GE-06A	SKCM	6.16
TCGA-D3-A51H-06A	SKCM	6.23
TCGA-BF-A5EQ-01A	SKCM	6.52
TCGA-EB-A6R0-01A	SKCM	6.27
TCGA-EE-A29Q-06A	SKCM	7.05
TCGA-FS-A1ZB-06A	SKCM	6.96
TCGA-FS-A1Z0-06A	SKCM	7.01
TCGA-D3-A2J6-06A	SKCM	6.71
TCGA-EE-A2MP-06A	SKCM	5.82
TCGA-EB-A3XD-01A	SKCM	6.24
TCGA-WE-A8ZY-06A	SKCM	6.47
TCGA-BF-A3DJ-01A	SKCM	6.35
TCGA-EB-A4OY-01A	SKCM	4.92
TCGA-DA-A1HW-06A	SKCM	6.61
TCGA-Z2-AA3V-06A	SKCM	3.4
TCGA-GN-A26A-06A	SKCM	6.03
TCGA-W3-AA1R-06A	SKCM	6.85
TCGA-EB-A51B-01A	SKCM	6.79
TCGA-D3-A8GN-06A	SKCM	4.72
TCGA-EE-A20F-06A	SKCM	5.89
TCGA-3N-A9WB-06A	SKCM	6.35
TCGA-EB-A5UM-01A	SKCM	5.92
TCGA-FR-A3YO-06A	SKCM	4.15
TCGA-DA-A95Z-06A	SKCM	6
TCGA-FS-A4F4-06A	SKCM	4.93
TCGA-DA-A1I0-06A	SKCM	5.81
TCGA-FS-A1Z4-06A	SKCM	6.65
TCGA-RP-A693-06A	SKCM	6.28
TCGA-EE-A3AF-06A	SKCM	6.4
TCGA-EE-A3JA-06A	SKCM	6.92
TCGA-XV-A9VZ-01A	SKCM	7.41
TCGA-EE-A29V-06A	SKCM	6.4
TCGA-EB-A85I-01A	SKCM	5.43
TCGA-EE-A29A-06A	SKCM	6.41
TCGA-FW-A3TU-06A	SKCM	4.07
TCGA-RP-A6K9-06A	SKCM	6.2
TCGA-ER-A19L-06A	SKCM	6.01
TCGA-D3-A5GT-01A	SKCM	4.86
TCGA-HR-A2OH-06A	SKCM	6.13
TCGA-WE-A8JZ-06A	SKCM	6.05
TCGA-EE-A3JB-06A	SKCM	6.96
TCGA-BF-AAP1-01A	SKCM	5.93
TCGA-D3-A1Q9-06A	SKCM	6.24
TCGA-EE-A2M8-06A	SKCM	6.2
TCGA-BF-AAP0-06A	SKCM	6.28
TCGA-3N-A9WC-06A	SKCM	5.76
TCGA-D3-A3MU-06A	SKCM	5.62
TCGA-BF-A1Q0-01A	SKCM	6.69
TCGA-BF-AAP4-01A	SKCM	6.52
TCGA-ER-A19M-06A	SKCM	6.68
TCGA-ER-A19Q-06A	SKCM	6.48
TCGA-FS-A1ZW-06A	SKCM	5.86

TCGA-D3-A2JH-06A	SKCM	6.35
TCGA-EE-A2MK-06A	SKCM	6.78
TCGA-W3-AA21-06A	SKCM	5.78
TCGA-BF-A1PU-01A	SKCM	6.54
TCGA-ER-A42L-06A	SKCM	7.01
TCGA-FW-A3I3-06A	SKCM	6.42
TCGA-FS-A4F2-06A	SKCM	7.23
TCGA-EB-A97M-01A	SKCM	5.18
TCGA-XV-AB01-06A	SKCM	4.39
TCGA-DA-A1HY-06A	SKCM	6.59
TCGA-DA-A1I2-06A	SKCM	6.83
TCGA-HR-A2OG-06A	SKCM	5.32
TCGA-EE-A3J7-06A	SKCM	7.13
TCGA-BF-AAP7-01A	SKCM	5.35
TCGA-ER-A3ET-06A	SKCM	7.35
TCGA-ER-A197-06A	SKCM	7.06
TCGA-FS-A1ZT-06A	SKCM	6.91
TCGA-FS-A4F0-06A	SKCM	8.24
TCGA-FS-A1YW-06A	SKCM	6.54
TCGA-GF-A6C9-06A	SKCM	6.44
TCGA-D3-A3C7-06A	SKCM	5.7
TCGA-EE-A2GM-06B	SKCM	7.14
TCGA-EE-A2MS-06A	SKCM	6.65
TCGA-EE-A2ML-06A	SKCM	7.17
TCGA-FS-A1ZP-06A	SKCM	6.74
TCGA-ER-A195-06A	SKCM	6.79
TCGA-FW-A3TV-06A	SKCM	6.25
TCGA-EB-A4IQ-01A	SKCM	5.87
TCGA-EB-A3XC-01A	SKCM	6.68
TCGA-GN-A265-06A	SKCM	6.15
TCGA-EE-A2MN-06A	SKCM	7.12
TCGA-FS-A4F9-06A	SKCM	6.85
TCGA-GF-A6C8-06A	SKCM	6.08
TCGA-EE-A2GC-06A	SKCM	7.1
TCGA-EB-A5KH-06A	SKCM	5.56
TCGA-ER-A19F-06A	SKCM	7.05
TCGA-FS-A1ZK-06A	SKCM	6.89
TCGA-FR-A44A-06A	SKCM	6.51
TCGA-BF-A9VF-01A	SKCM	5.98
TCGA-YG-AA3O-06A	SKCM	6.54
TCGA-D3-A1Q6-06A	SKCM	6.46
TCGA-ER-A2ND-06A	SKCM	6.86
TCGA-FR-A7U9-06A	SKCM	7.21
TCGA-BF-A3DM-01A	SKCM	5.77
TCGA-D9-A6E9-06A	SKCM	5.96
TCGA-D3-A2JN-06A	SKCM	6.33
TCGA-FS-A1ZJ-06A	SKCM	6.3
TCGA-D3-A3CC-06A	SKCM	7.36
TCGA-DA-A1HV-06A	SKCM	6.6
TCGA-D3-A51F-06A	SKCM	6.31
TCGA-FR-A7U8-06A	SKCM	5.76
TCGA-GF-A2C7-01A	SKCM	5.7
TCGA-W3-A825-06A	SKCM	6.26
TCGA-D3-A1Q5-06A	SKCM	7.56
TCGA-D3-A3C1-06A	SKCM	5.76
TCGA-FS-A1ZS-06A	SKCM	8.5
TCGA-EE-A2MC-06A	SKCM	7.22
TCGA-RP-A695-06A	SKCM	5.64

TCGA-EE-A2MU-06A	SKCM	6.32
TCGA-EE-A2MF-06A	SKCM	6.14
TCGA-WE-AA9Y-06A	SKCM	6.2
TCGA-GN-A263-01A	SKCM	7.33
TCGA-D9-A149-06A	SKCM	7.31
TCGA-D3-A3CF-06A	SKCM	6.67
TCGA-WE-A8K5-06A	SKCM	5.88
TCGA-D3-A3C8-06A	SKCM	6.7
TCGA-FS-A1ZA-06A	SKCM	6.65
TCGA-YG-AA3N-01A	SKCM	5.97
TCGA-EE-A3JI-06A	SKCM	8.25
TCGA-FS-A1Z3-06A	SKCM	6.26
TCGA-ER-A194-01A	SKCM	4.67
TCGA-DA-A1IA-06A	SKCM	4.14
TCGA-HR-A5NC-01A	SKCM	6.38
TCGA-EB-A24C-01A	SKCM	6.97
TCGA-EB-A1NK-01A	SKCM	6.42
TCGA-D3-A51R-06A	SKCM	6.82
TCGA-QB-AA9O-06A	SKCM	7.17
TCGA-D3-A2JE-06A	SKCM	7.37
TCGA-EB-A5SF-01A	SKCM	4.07
TCGA-D3-A51J-06A	SKCM	6.32
TCGA-D3-A2JA-06A	SKCM	6.38
TCGA-D3-A3ML-06A	SKCM	7.46
TCGA-EB-A4OZ-01A	SKCM	5.08
TCGA-ER-A19D-06A	SKCM	6.48
TCGA-GN-A266-06A	SKCM	6.61
TCGA-EB-A5FP-01A	SKCM	6.16
TCGA-W3-AA1W-06A	SKCM	6.33
TCGA-EE-A184-06A	SKCM	6.3
TCGA-FS-A1ZE-06A	SKCM	8.08
TCGA-EE-A3JH-06A	SKCM	5.35
TCGA-EE-A20H-06A	SKCM	6.73
TCGA-D3-A8GV-06A	SKCM	5.82
TCGA-D3-A8GQ-06A	SKCM	6.1
TCGA-ER-A2NE-06A	SKCM	6.29
TCGA-D3-A3MR-06A	SKCM	5.17
TCGA-ER-A1A1-06A	SKCM	6.64
TCGA-D3-A2J7-06A	SKCM	7.06
TCGA-EE-A29M-06A	SKCM	6.06
TCGA-EB-A4IS-01A	SKCM	6.26
TCGA-EE-A2GU-06A	SKCM	6.72
TCGA-D9-A4Z3-01A	SKCM	6.76
TCGA-EE-A29X-06A	SKCM	5.4
TCGA-FS-A4F8-06A	SKCM	6.75
TCGA-FR-A8YD-06A	SKCM	5.03
TCGA-DA-A3F3-06A	SKCM	6.85
TCGA-BF-AAOX-01A	SKCM	6.23
TCGA-ER-A19E-06A	SKCM	6.47
TCGA-D3-A2JL-06A	SKCM	5.85
TCGA-EE-A3J4-06A	SKCM	6
TCGA-D3-A1Q3-06A	SKCM	7.34
TCGA-EE-A3AG-06A	SKCM	6.86
TCGA-BF-A5ES-01A	SKCM	6.28
TCGA-ER-A19B-06A	SKCM	6.48
TCGA-ER-A2NG-06A	SKCM	6.14
TCGA-EB-A6L9-06A	SKCM	5.35
TCGA-D3-A1QA-06A	SKCM	7.57

TCGA-FW-A3R5-06A	SKCM	7.32
TCGA-D3-A3C3-06A	SKCM	7.4
TCGA-WE-A8ZX-06A	SKCM	6.5
TCGA-EB-A42Z-01A	SKCM	3.7
TCGA-YG-AA3P-06A	SKCM	6.44
TCGA-FS-A1ZD-06A	SKCM	6.6
TCGA-GN-A4U5-01A	SKCM	5.81
TCGA-EE-A2GN-06A	SKCM	5.87
TCGA-GN-A4U9-06A	SKCM	6.36
TCGA-EE-A3JE-06A	SKCM	6.3
TCGA-D9-A3Z4-01A	SKCM	6.4
TCGA-EE-A2MI-06A	SKCM	6.19
TCGA-ER-A19J-06A	SKCM	6.91
TCGA-D9-A6EG-06A	SKCM	5.71
TCGA-EB-A44O-01A	SKCM	6.7
TCGA-D9-A6EC-06A	SKCM	6.96
TCGA-EE-A17Y-06A	SKCM	7.01
TCGA-EB-A42Y-01A	SKCM	7
TCGA-FR-A728-01A	SKCM	5.4
TCGA-DA-A1I7-06A	SKCM	6.27
TCGA-D3-A2J9-06A	SKCM	6.6
TCGA-BF-A3DL-01A	SKCM	5.02
TCGA-BF-A1PV-01A	SKCM	7.24
TCGA-WE-A8ZN-06A	SKCM	5
TCGA-D3-A1QA-07A	SKCM	6.58
TCGA-BR-6457-01A	STAD	5.7
TCGA-BR-A4QM-01A	STAD	4.78
TCGA-CG-5723-01A	STAD	6.7
TCGA-BR-8591-01A	STAD	6.8
TCGA-BR-7957-01A	STAD	7.33
TCGA-BR-8368-01A	STAD	7.02
TCGA-BR-A4J2-01A	STAD	6.13
TCGA-CD-5813-01A	STAD	5.8
TCGA-FP-7735-01A	STAD	5.85
TCGA-CD-8528-01A	STAD	6.79
TCGA-D7-8570-01A	STAD	5.69
TCGA-BR-8485-01A	STAD	6.5
TCGA-BR-8060-01A	STAD	6.25
TCGA-RD-A8N4-01A	STAD	6.53
TCGA-VQ-A8PC-01A	STAD	6.68
TCGA-BR-7958-01A	STAD	6.23
TCGA-CD-8527-01A	STAD	6.7
TCGA-BR-6456-01A	STAD	7.12
TCGA-F1-6875-01A	STAD	7.02
TCGA-IN-A6RJ-01A	STAD	6.18
TCGA-BR-7703-01A	STAD	6.8
TCGA-BR-A4CS-01A	STAD	5.36
TCGA-CG-4469-01A	STAD	6.6
TCGA-FP-8211-01A	STAD	5.28
TCGA-CG-5724-01A	STAD	7.21
TCGA-HU-A4GJ-01A	STAD	5.75
TCGA-RD-A8MW-01A	STAD	5.99
TCGA-BR-7851-01A	STAD	7.23
TCGA-BR-A4PF-01A	STAD	7.35
TCGA-FP-A4BF-01A	STAD	5.82
TCGA-F1-6874-01A	STAD	7
TCGA-RD-A8N2-01A	STAD	6.33
TCGA-HU-A4G8-01A	STAD	7.09

TCGA-D7-A4YY-01A	STAD	7.17
TCGA-CG-4477-01A	STAD	6.81
TCGA-D7-8575-01A	STAD	7.47
TCGA-BR-8081-01A	STAD	6.48
TCGA-BR-8291-01A	STAD	6.02
TCGA-CD-A4MH-01A	STAD	5.37
TCGA-IN-A7NU-01A	STAD	6.09
TCGA-VQ-A8PB-01A	STAD	6.86
TCGA-VQ-A91K-01A	STAD	6.91
TCGA-BR-4361-01A	STAD	7.04
TCGA-IN-A6RO-01A	STAD	7.02
TCGA-FP-8631-01A	STAD	5.9
TCGA-BR-6452-01A	STAD	7.32
TCGA-CG-5719-01A	STAD	6.14
TCGA-RD-A7BT-01A	STAD	7.28
TCGA-BR-8296-01A	STAD	6.42
TCGA-CD-A4MJ-01A	STAD	7.19
TCGA-CG-4438-01A	STAD	7.02
TCGA-D7-A4YX-01A	STAD	7.16
TCGA-HU-A4HB-01A	STAD	6.41
TCGA-B7-A5TI-01A	STAD	6.84
TCGA-D7-A74A-01A	STAD	7.43
TCGA-VQ-A8PE-01A	STAD	6.94
TCGA-BR-4253-01A	STAD	7.02
TCGA-MX-A5UJ-01A	STAD	6.3
TCGA-HU-A4G2-01A	STAD	6.66
TCGA-HU-A4GY-01A	STAD	6.4
TCGA-EQ-8122-01A	STAD	6.17
TCGA-CG-4476-01A	STAD	6.42
TCGA-VQ-AA6I-01A	STAD	7.16
TCGA-RD-A8MV-01A	STAD	5.7
TCGA-BR-8678-01A	STAD	6.84
TCGA-BR-6801-01A	STAD	5.86
TCGA-IN-AB1X-01A	STAD	7.33
TCGA-D7-A6EX-01A	STAD	7.13
TCGA-BR-7196-01A	STAD	6.32
TCGA-KB-A6F7-01A	STAD	6.58
TCGA-VQ-A91A-01A	STAD	6.5
TCGA-BR-8371-01A	STAD	6.38
TCGA-VQ-AA6B-01A	STAD	7
TCGA-IN-A7NT-01A	STAD	7.61
TCGA-D7-6520-01A	STAD	6.59
TCGA-HF-7134-01A	STAD	6.56
TCGA-RD-A8N0-01A	STAD	5.74
TCGA-D7-A6EZ-01A	STAD	6.53
TCGA-FP-8209-01A	STAD	6.77
TCGA-VQ-A922-01A	STAD	6.4
TCGA-ZA-A8F6-01A	STAD	6.35
TCGA-BR-7901-01A	STAD	6.16
TCGA-BR-8680-01A	STAD	7.05
TCGA-VQ-A94R-01A	STAD	6.5
TCGA-BR-6564-01A	STAD	6.3
TCGA-BR-6454-01A	STAD	6.06
TCGA-HU-8238-01A	STAD	6.13
TCGA-FP-7998-01A	STAD	6.4
TCGA-BR-8367-01A	STAD	6.9
TCGA-CD-A48A-01A	STAD	5.86
TCGA-R5-A7ZI-01A	STAD	5.18

TCGA-BR-7959-01A	STAD	6.84
TCGA-BR-8059-01A	STAD	7.07
TCGA-BR-7723-01A	STAD	7.02
TCGA-VQ-AA6G-01A	STAD	6.03
TCGA-HU-A4H2-01A	STAD	5.93
TCGA-R5-A7ZE-01B	STAD	6.08
TCGA-BR-8686-01A	STAD	6.3
TCGA-D7-A6EV-01A	STAD	6.98
TCGA-BR-6802-01A	STAD	6.48
TCGA-CG-4301-01A	STAD	6.82
TCGA-HU-A4GX-01A	STAD	5.97
TCGA-HU-8243-01A	STAD	6.05
TCGA-VQ-A927-01A	STAD	6.43
TCGA-HJ-7597-01A	STAD	7.27
TCGA-BR-7707-01A	STAD	6.89
TCGA-BR-8289-01A	STAD	6.53
TCGA-CG-4440-01A	STAD	7.31
TCGA-BR-A4J4-01A	STAD	7.26
TCGA-HU-A4G9-01A	STAD	6.81
TCGA-D7-8579-01A	STAD	6.36
TCGA-FP-8210-01A	STAD	6.42
TCGA-BR-8361-01A	STAD	6.81
TCGA-VQ-A94U-01A	STAD	6.86
TCGA-CG-4442-01A	STAD	7.2
TCGA-HU-A4GD-01A	STAD	5.79
TCGA-BR-4255-01A	STAD	6.87
TCGA-BR-6563-01A	STAD	6.09
TCGA-BR-7704-01A	STAD	6.16
TCGA-BR-4294-01A	STAD	6.73
TCGA-BR-8588-01A	STAD	5.95
TCGA-D7-8574-01A	STAD	5.72
TCGA-SW-A7EB-01A	STAD	6.45
TCGA-BR-8284-01A	STAD	6.88
TCGA-D7-6526-01A	STAD	6.98
TCGA-VQ-A8E2-01A	STAD	6.46
TCGA-BR-8487-01A	STAD	6.95
TCGA-BR-A4QL-01A	STAD	6.12
TCGA-BR-8690-01A	STAD	5.71
TCGA-CD-5803-01A	STAD	6.66
TCGA-VQ-A91D-01A	STAD	7.36
TCGA-BR-A4IV-01A	STAD	6.29
TCGA-D7-6528-01A	STAD	6.17
TCGA-BR-8297-01A	STAD	6.49
TCGA-FP-A9TM-01A	STAD	6.11
TCGA-CG-5734-01A	STAD	4.86
TCGA-VQ-AA68-01A	STAD	6.96
TCGA-BR-A4J7-01A	STAD	6.3
TCGA-HU-8604-01A	STAD	6.06
TCGA-BR-8058-01A	STAD	6.14
TCGA-R5-A7O7-01A	STAD	5.07
TCGA-CG-4460-01A	STAD	5.37
TCGA-CG-5720-01A	STAD	5.97
TCGA-D7-6525-01A	STAD	7.27
TCGA-IN-7806-01A	STAD	6.94
TCGA-BR-8683-01A	STAD	6.75
TCGA-BR-7197-01A	STAD	6.63
TCGA-CD-5804-01A	STAD	5.75
TCGA-BR-6706-01A	STAD	5.37

TCGA-BR-8381-01A	STAD	6.5
TCGA-R5-A7ZF-01A	STAD	6.88
TCGA-BR-A4IU-01A	STAD	6.66
TCGA-IN-8462-01A	STAD	6.02
TCGA-VQ-A94P-01A	STAD	6.69
TCGA-D7-A748-01A	STAD	6.03
TCGA-D7-A6F0-01A	STAD	6.29
TCGA-IN-A6RS-01A	STAD	6.21
TCGA-VQ-A924-01A	STAD	7.07
TCGA-VQ-AA6K-01A	STAD	6.97
TCGA-BR-A44U-01A	STAD	6.03
TCGA-VQ-A8DT-01A	STAD	6.23
TCGA-BR-7722-01A	STAD	5.9
TCGA-CG-5726-01A	STAD	6.24
TCGA-VQ-A8E7-01B	STAD	6.62
TCGA-BR-4191-01A	STAD	6.2
TCGA-HU-A4G3-01A	STAD	5.56
TCGA-D7-6524-01A	STAD	6.79
TCGA-SW-A7EA-01A	STAD	6.54
TCGA-VQ-A91Y-01A	STAD	6.31
TCGA-D7-8572-01A	STAD	6.8
TCGA-BR-4201-01A	STAD	6.85
TCGA-CG-4443-01A	STAD	3.31
TCGA-3M-AB47-01A	STAD	6.48
TCGA-BR-A44T-01A	STAD	5.54
TCGA-CG-4304-01A	STAD	5.16
TCGA-VQ-A8P2-01A	STAD	7.1
TCGA-BR-6455-01A	STAD	7.31
TCGA-BR-8286-01A	STAD	7.05
TCGA-CD-A489-01A	STAD	6.67
TCGA-VQ-A91W-01A	STAD	6.74
TCGA-VQ-A8PD-01A	STAD	6.79
TCGA-RD-A8N9-01A	STAD	6.48
TCGA-HU-A4H8-01A	STAD	6.85
TCGA-D7-6522-01A	STAD	5.8
TCGA-BR-7717-01A	STAD	5.83
TCGA-BR-A4QI-01A	STAD	5.92
TCGA-CD-8532-01A	STAD	6.66
TCGA-BR-A4IY-01A	STAD	6.11
TCGA-CD-8536-01A	STAD	7.7
TCGA-BR-8589-01A	STAD	7.03
TCGA-HF-A5NB-01A	STAD	6.99
TCGA-BR-6453-01A	STAD	5.62
TCGA-BR-4357-01A	STAD	6.1
TCGA-RD-A7BW-01A	STAD	6.7
TCGA-VQ-A8PM-01A	STAD	6.3
TCGA-VQ-AA6J-01A	STAD	7.27
TCGA-D7-6818-01A	STAD	6.31
TCGA-BR-A4IZ-01A	STAD	6.83
TCGA-VQ-A91Z-01A	STAD	6.71
TCGA-VQ-A8PU-01A	STAD	7.28
TCGA-D7-6527-01A	STAD	5.4
TCGA-BR-4367-01A	STAD	7.18
TCGA-VQ-A91S-01A	STAD	7.04
TCGA-RD-A7C1-01A	STAD	6.3
TCGA-BR-8080-01A	STAD	5.95
TCGA-CG-4465-01A	STAD	6.82
TCGA-BR-7716-01A	STAD	6.52

TCGA-HU-8249-01A	STAD	6.5
TCGA-BR-8382-01A	STAD	6.83
TCGA-CD-5798-01A	STAD	6.78
TCGA-VQ-A8PX-01A	STAD	6.86
TCGA-CD-A48C-01A	STAD	6.32
TCGA-CG-5722-01A	STAD	5.79
TCGA-IN-7808-01A	STAD	5.4
TCGA-RD-A7BS-01A	STAD	6.54
TCGA-VQ-A8PF-01A	STAD	6.62
TCGA-VQ-A92D-01A	STAD	6.52
TCGA-BR-8380-01A	STAD	6.35
TCGA-BR-8592-01A	STAD	6.48
TCGA-BR-6705-01A	STAD	6.24
TCGA-BR-8676-01A	STAD	5.6
TCGA-HU-8608-01A	STAD	7.09
TCGA-CG-5717-01A	STAD	6.8
TCGA-BR-4257-01A	STAD	6.96
TCGA-BR-A4PD-01A	STAD	6.79
TCGA-BR-8590-01A	STAD	6.77
TCGA-KB-A93H-01A	STAD	5.95
TCGA-3M-AB46-01A	STAD	5
TCGA-VQ-AA6F-01A	STAD	6.4
TCGA-MX-A663-01A	STAD	7.88
TCGA-BR-6707-01A	STAD	5.74
TCGA-BR-8682-01A	STAD	6.9
TCGA-ZQ-A9CR-01A	STAD	5.95
TCGA-VQ-A8E0-01A	STAD	6.67
TCGA-VQ-A91N-01A	STAD	6.04
TCGA-BR-4371-01A	STAD	6.37
TCGA-F1-6177-01A	STAD	6.92
TCGA-VQ-A8DV-01A	STAD	9.63
TCGA-F1-A448-01A	STAD	6.05
TCGA-HU-A4HD-01A	STAD	6.41
TCGA-CD-A4MI-01A	STAD	6.05
TCGA-HU-A4GP-01A	STAD	6.42
TCGA-CD-5799-01A	STAD	6.06
TCGA-IN-A6RL-01A	STAD	6.35
TCGA-IN-A6RN-01A	STAD	5.54
TCGA-D7-A4Z0-01A	STAD	6.29
TCGA-IN-8663-01A	STAD	8.23
TCGA-CG-4475-01A	STAD	7.18
TCGA-BR-8483-01A	STAD	5.76
TCGA-BR-6458-01A	STAD	6.94
TCGA-CD-5800-01A	STAD	5.86
TCGA-R5-A7ZR-01A	STAD	5.87
TCGA-BR-8078-01A	STAD	7.36
TCGA-B7-5816-01A	STAD	6.56
TCGA-D7-8573-01A	STAD	6.7
TCGA-BR-4369-01A	STAD	6.28
TCGA-FP-A8CX-01A	STAD	6.04
TCGA-BR-8679-01A	STAD	6.72
TCGA-VQ-A8P8-01A	STAD	6.47
TCGA-CD-A4MG-01A	STAD	5.85
TCGA-VQ-A94T-01A	STAD	6.32
TCGA-BR-8677-01A	STAD	6.51
TCGA-BR-A4J1-01A	STAD	7.06
TCGA-VQ-A8E3-01A	STAD	7.87
TCGA-D7-A6F2-01A	STAD	6.04

TCGA-MX-A5UG-01A	STAD	6.82
TCGA-IN-A7NR-01A	STAD	5.94
TCGA-RD-A8NB-01A	STAD	6.99
TCGA-HU-A4GN-01A	STAD	7.2
TCGA-BR-8077-01A	STAD	6.5
TCGA-IN-A6RI-01A	STAD	5.43
TCGA-IP-7968-01A	STAD	7.27
TCGA-VQ-AA64-01A	STAD	5.12
TCGA-BR-A4PE-01A	STAD	6.53
TCGA-HU-A4H5-01A	STAD	5.24
TCGA-HU-A4GH-01A	STAD	5.78
TCGA-CG-5718-01A	STAD	4.71
TCGA-D7-6822-01A	STAD	6.44
TCGA-CD-5801-01A	STAD	6.52
TCGA-BR-4280-01A	STAD	6.55
TCGA-HU-8244-01A	STAD	5.93
TCGA-MX-A666-01A	STAD	6.95
TCGA-BR-8362-01A	STAD	7.01
TCGA-BR-6565-01A	STAD	7.02
TCGA-VQ-A94O-01A	STAD	6.71
TCGA-VQ-A8PP-01A	STAD	7.63
TCGA-HU-8602-01A	STAD	8.56
TCGA-HU-A4GF-01A	STAD	6.46
TCGA-CG-4462-01A	STAD	6.84
TCGA-D7-6519-01A	STAD	7.08
TCGA-RD-A8N6-01A	STAD	6.29
TCGA-CD-8531-01A	STAD	6.29
TCGA-VQ-AA6A-01A	STAD	6.12
TCGA-FP-7916-01A	STAD	6.44
TCGA-IN-AB1V-01A	STAD	6.94
TCGA-KB-A93G-01A	STAD	6.45
TCGA-BR-8369-01A	STAD	8.34
TCGA-CG-5732-01A	STAD	5.63
TCGA-VQ-A91Q-01A	STAD	7.19
TCGA-CD-A487-01A	STAD	5.6
TCGA-BR-4370-01A	STAD	7.08
TCGA-VQ-A8DZ-01A	STAD	6.54
TCGA-CD-A486-01A	STAD	5.37
TCGA-D7-5578-01A	STAD	6.23
TCGA-BR-8486-01A	STAD	6.22
TCGA-CD-8533-01A	STAD	7.18
TCGA-HU-A4GU-01A	STAD	6.79
TCGA-BR-4368-01A	STAD	7.92
TCGA-VQ-A8P3-01A	STAD	5.88
TCGA-BR-7715-01A	STAD	6.67
TCGA-BR-8295-01A	STAD	5.08
TCGA-F1-A72C-01A	STAD	6.35
TCGA-HF-7133-01A	STAD	6.57
TCGA-D7-8578-01A	STAD	6.1
TCGA-BR-4292-01A	STAD	6.7
TCGA-BR-4366-01A	STAD	6.66
TCGA-HU-A4GQ-01A	STAD	6.42
TCGA-VQ-A8PH-01A	STAD	6.63
TCGA-B7-5818-01A	STAD	6.93
TCGA-VQ-A8PQ-01A	STAD	6.82
TCGA-RD-A8N5-01A	STAD	5.85
TCGA-D7-5577-01A	STAD	6.55
TCGA-HU-A4G6-01A	STAD	6.71

TCGA-VQ-A91E-01A	STAD	7.05
TCGA-CD-8525-01A	STAD	4.87
TCGA-VQ-A928-01A	STAD	6.32
TCGA-D7-A4YU-01A	STAD	7.73
TCGA-CG-4441-01A	STAD	5.37
TCGA-D7-A747-01A	STAD	6.17
TCGA-D7-6518-01A	STAD	5.89
TCGA-VQ-AA69-01A	STAD	7.28
TCGA-RD-A8N1-01A	STAD	5.97
TCGA-D7-A4YV-01A	STAD	6.76
TCGA-HU-A4H6-01A	STAD	5.84
TCGA-FP-A4BE-01A	STAD	6.72
TCGA-BR-6852-01A	STAD	6.58
TCGA-CG-5716-01A	STAD	5.62
TCGA-VQ-A8PO-01A	STAD	7.07
TCGA-VQ-A91V-01A	STAD	6.75
TCGA-B7-A5TK-01A	STAD	6.89
TCGA-BR-A4J5-01A	STAD	6.12
TCGA-BR-8372-01A	STAD	8.09
TCGA-CD-8529-01A	STAD	6.12
TCGA-B7-A5TJ-01A	STAD	6.61
TCGA-VQ-A91X-01A	STAD	6.26
TCGA-CG-4437-01A	STAD	6.07
TCGA-BR-8364-01A	STAD	6.73
TCGA-VQ-A8DU-01A	STAD	5.95
TCGA-CG-5721-01A	STAD	6.46
TCGA-KB-A93J-01A	STAD	6.39
TCGA-VQ-A91U-01A	STAD	6.55
TCGA-VQ-A923-01A	STAD	6.51
TCGA-BR-6566-01A	STAD	5.64
TCGA-BR-8365-01A	STAD	6.54
TCGA-BR-A4J8-01A	STAD	5.78
TCGA-HU-A4H4-01A	STAD	6.48
TCGA-VQ-A925-01A	STAD	6.09
TCGA-BR-6710-01A	STAD	6.11
TCGA-CG-4466-01A	STAD	5.79
TCGA-BR-6803-01A	STAD	6.25
TCGA-CG-4444-01A	STAD	6.05
TCGA-BR-8687-01A	STAD	6.35
TCGA-BR-8366-01A	STAD	6.56
TCGA-CD-8535-01A	STAD	7.66
TCGA-D7-6817-01A	STAD	6.62
TCGA-BR-A4J9-01A	STAD	6.08
TCGA-BR-4363-01A	STAD	6.81
TCGA-B7-A5TN-01A	STAD	6.78
TCGA-BR-8373-01A	STAD	7.26
TCGA-VQ-AA6D-01A	STAD	6.9
TCGA-BR-8384-01A	STAD	6.81
TCGA-CD-8526-01A	STAD	6.24
TCGA-HF-7136-01A	STAD	7.02
TCGA-FP-8099-01A	STAD	6.69
TCGA-D7-A4YT-01A	STAD	5.64
TCGA-BR-8363-01A	STAD	6.86
TCGA-D7-6815-01A	STAD	6.78
TCGA-CD-8524-01A	STAD	6.14
TCGA-FP-7829-01A	STAD	6.78
TCGA-HU-A4H3-01A	STAD	6.79
TCGA-VQ-A8PK-01A	STAD	5.94

TCGA-BR-6709-01A	STAD	6.25
TCGA-R5-A805-01A	STAD	5.66
TCGA-CD-8534-01A	STAD	5.98
TCGA-D7-A6EY-01A	STAD	6.8
TCGA-IN-A6RR-01A	STAD	4.09
TCGA-BR-A452-01A	STAD	6.65
TCGA-HF-7132-01A	STAD	7.17
TCGA-D7-6521-01A	STAD	6.16
TCGA-CG-4306-01A	STAD	7.15
TCGA-BR-4256-01A	STAD	6.92
TCGA-BR-A4J6-01A	STAD	6.67
TCGA-BR-A453-01A	STAD	6.36
TCGA-BR-4279-01A	STAD	6.79
TCGA-BR-4187-01A	STAD	6.39
TCGA-CG-5725-01A	STAD	5.9
TCGA-HU-8610-01A	STAD	6.68
TCGA-HU-A4GC-01A	STAD	5.96
TCGA-VQ-A8P5-01A	STAD	7.15
TCGA-D7-6820-01A	STAD	6.61
TCGA-CG-4436-01A	STAD	6.12
TCGA-BR-A4CR-01A	STAD	6.36
TCGA-HU-A4GT-01A	STAD	6.8
TCGA-VQ-A8PJ-01A	STAD	8.5
TCGA-HF-7131-01A	STAD	7.29
TCGA-BR-4267-01A	STAD	7.2
TCGA-CD-8530-01A	STAD	6.06
TCGA-CG-4305-01A	STAD	7.47
TCGA-BR-8484-01A	STAD	6.63
TCGA-EQ-A4SO-01A	STAD	7.58
TCGA-HU-A4H0-01A	STAD	7.24
TCGA-D7-8576-01A	STAD	6.72
TCGA-2G-AAFE-01A	TGCT	7.11
TCGA-S6-A8JY-01A	TGCT	7.3
TCGA-2G-AAGY-01A	TGCT	6.18
TCGA-2G-AAG0-01A	TGCT	8.27
TCGA-2X-A9D6-01A	TGCT	7.21
TCGA-2G-AAKO-01A	TGCT	7.36
TCGA-2G-AAG9-01A	TGCT	6.82
TCGA-4K-AA1H-01A	TGCT	6.73
TCGA-2G-AAGT-01A	TGCT	6.93
TCGA-2G-AAFH-01A	TGCT	6.57
TCGA-2G-AALT-01A	TGCT	6.05
TCGA-VF-A8AC-01A	TGCT	7.56
TCGA-2G-AAKG-05A	TGCT	5.46
TCGA-XY-A8S3-01B	TGCT	7.61
TCGA-XE-AANV-01A	TGCT	7.28
TCGA-W4-A7U2-01A	TGCT	7.17
TCGA-2X-A9D5-01A	TGCT	6.84
TCGA-2G-AAH2-01A	TGCT	6.79
TCGA-VF-A8A8-01A	TGCT	7.86
TCGA-2G-AAG6-01A	TGCT	6.6
TCGA-2G-AALS-01A	TGCT	6.47
TCGA-2G-AAGW-01A	TGCT	7.52
TCGA-2G-AAKG-01A	TGCT	7.87
TCGA-ZM-AA0F-01A	TGCT	6.36
TCGA-2G-AAFO-01A	TGCT	7.13
TCGA-2G-AAGA-01A	TGCT	7.92
TCGA-XE-AAOD-01A	TGCT	7.01

TCGA-WZ-A8D5-01A	TGCT	7.4
TCGA-2G-AAFL-01A	TGCT	6.88
TCGA-YU-A912-01A	TGCT	6.85
TCGA-2G-AAFJ-01A	TGCT	6.92
TCGA-2G-AAGN-01A	TGCT	7.68
TCGA-2G-AAHL-01A	TGCT	7.04
TCGA-XE-AAOF-01A	TGCT	6.88
TCGA-2G-AAL7-01A	TGCT	7.88
TCGA-XE-A8H5-01A	TGCT	7.41
TCGA-2G-AALQ-01A	TGCT	6.27
TCGA-ZM-AA0D-01A	TGCT	6.6
TCGA-W4-A7U3-01A	TGCT	6.89
TCGA-2G-AAFN-01A	TGCT	7.03
TCGA-2G-AAEW-01A	TGCT	7.82
TCGA-XE-AAO4-01A	TGCT	7.02
TCGA-YU-AA61-01A	TGCT	7.08
TCGA-SN-A6IS-01A	TGCT	7.1
TCGA-SN-A84X-01A	TGCT	6.49
TCGA-2G-AAGV-01A	TGCT	6.46
TCGA-SN-A84W-01A	TGCT	6.38
TCGA-2G-AAGE-01A	TGCT	7.78
TCGA-2G-AAH8-01A	TGCT	7.46
TCGA-XE-A9SE-01A	TGCT	6.7
TCGA-2G-AAGI-05A	TGCT	7.34
TCGA-XE-A8H4-01A	TGCT	7.37
TCGA-XY-A9T9-01A	TGCT	7.02
TCGA-2G-AAHT-01A	TGCT	7.42
TCGA-SN-A84Y-01A	TGCT	8.06
TCGA-2G-AALG-01A	TGCT	5.94
TCGA-2G-AAGG-01A	TGCT	6.43
TCGA-VF-A8A9-01A	TGCT	7.13
TCGA-XE-AAOB-01A	TGCT	6.21
TCGA-XE-A8H1-01A	TGCT	8.46
TCGA-SB-A76C-01A	TGCT	8.32
TCGA-2G-AAGX-01A	TGCT	7.18
TCGA-S6-A8JW-01A	TGCT	6.97
TCGA-ZM-AA0E-01A	TGCT	6.54
TCGA-2G-AAHC-01A	TGCT	7.53
TCGA-2G-AAF8-01A	TGCT	7.36
TCGA-2G-AAKD-01A	TGCT	7.33
TCGA-2G-AAGY-05A	TGCT	8.12
TCGA-2G-AAKO-05A	TGCT	7.18
TCGA-2G-AAEX-01A	TGCT	6.91
TCGA-2G-AAF4-01A	TGCT	7.45
TCGA-2G-AAGC-01A	TGCT	7.69
TCGA-YU-AA4L-01A	TGCT	7
TCGA-ZM-AA05-01A	TGCT	5.27
TCGA-4K-AAAL-01A	TGCT	7.06
TCGA-ZM-AA06-01A	TGCT	5.86
TCGA-XE-AAO6-01A	TGCT	6.72
TCGA-2G-AAFI-01A	TGCT	8.26
TCGA-2G-AAFM-01A	TGCT	7.13
TCGA-2G-AAG3-01A	TGCT	7.19
TCGA-2G-AAGO-01A	TGCT	8.64
TCGA-2G-AAM3-01A	TGCT	6.01
TCGA-2G-AAM4-01A	TGCT	6.67
TCGA-YU-A90W-01A	TGCT	7.14
TCGA-2G-AAFG-05A	TGCT	7.79

TCGA-2G-AAFG-01A	TGCT	7.65
TCGA-XE-AAOC-01A	TGCT	7.24
TCGA-YU-A90Q-01A	TGCT	7.58
TCGA-SB-A6J6-01A	TGCT	7.23
TCGA-ZM-AA0H-01A	TGCT	5.63
TCGA-2G-AAGK-01A	TGCT	8.03
TCGA-WZ-A7V4-01A	TGCT	6.07
TCGA-XE-AANJ-01A	TGCT	7.9
TCGA-2G-AALO-01A	TGCT	6.67
TCGA-2G-AAFY-01A	TGCT	7.48
TCGA-2G-AALY-01A	TGCT	7.01
TCGA-YU-A90S-01A	TGCT	6.66
TCGA-2G-AAG5-01A	TGCT	6.93
TCGA-2G-AAL5-01A	TGCT	6.67
TCGA-2G-AAGJ-01A	TGCT	8.18
TCGA-XY-A8S2-01A	TGCT	8.11
TCGA-2G-AAGZ-01A	TGCT	6.8
TCGA-2G-AAFV-01A	TGCT	6.78
TCGA-2G-AAF1-01A	TGCT	7.53
TCGA-YU-A94D-01A	TGCT	6.26
TCGA-W4-A7U4-01A	TGCT	8.28
TCGA-YU-A90P-01A	TGCT	7.44
TCGA-2G-AAH3-01A	TGCT	7.44
TCGA-VF-A8AA-01A	TGCT	7.38
TCGA-2G-AALZ-01A	TGCT	6.22
TCGA-2G-AAFZ-01A	TGCT	7.59
TCGA-2G-AAHG-01A	TGCT	7.34
TCGA-VF-A8AE-01A	TGCT	6.61
TCGA-2G-AAGP-01A	TGCT	6.54
TCGA-ZM-AA0B-01A	TGCT	7.14
TCGA-2G-AAHA-01A	TGCT	6.96
TCGA-4K-AA1G-01A	TGCT	8.34
TCGA-VF-A8AB-01A	TGCT	7.25
TCGA-2G-AALN-01A	TGCT	6.98
TCGA-YU-A94I-01A	TGCT	6.97
TCGA-2G-AAGM-01A	TGCT	7.48
TCGA-ZM-AA0N-01A	TGCT	6.48
TCGA-VF-A8AD-01A	TGCT	7.82
TCGA-2G-AAHP-05A	TGCT	8.01
TCGA-2G-AAG7-01A	TGCT	7.42
TCGA-WZ-A7V5-01A	TGCT	6.45
TCGA-2G-AALP-01A	TGCT	6.7
TCGA-2G-AAKM-01A	TGCT	7.82
TCGA-XE-AANI-01A	TGCT	6.74
TCGA-XE-AAOJ-01A	TGCT	6.88
TCGA-2G-AAG8-01A	TGCT	7.44
TCGA-XE-AANR-01A	TGCT	7.55
TCGA-2G-AALR-01A	TGCT	6.86
TCGA-2G-AAKL-01A	TGCT	7.98
TCGA-XY-A89B-01A	TGCT	7.02
TCGA-2G-AAH0-01A	TGCT	7.73
TCGA-2G-AAH4-01A	TGCT	6.25
TCGA-2G-AAGS-01A	TGCT	7.6
TCGA-SO-A8JP-01A	TGCT	7.74
TCGA-XE-AAOL-01A	TGCT	4.76
TCGA-XE-AAO3-01A	TGCT	7.25
TCGA-2G-AAGF-01A	TGCT	7.17
TCGA-2G-AAGI-01A	TGCT	7.45

TCGA-YU-A90Y-01A	TGCT	7.87
TCGA-2G-AALX-01A	TGCT	6.49
TCGA-2G-AAM2-01A	TGCT	7.38
TCGA-2G-AAHP-01A	TGCT	6.85
TCGA-2G-AALF-01A	TGCT	6.44
TCGA-2G-AAF6-01A	TGCT	8.09
TCGA-2G-AALW-01A	TGCT	6.96
TCGA-WZ-A7V3-01A	TGCT	7.63
TCGA-2G-AAKH-01A	TGCT	7.31
TCGA-2G-AAHN-01A	TGCT	6.76
TCGA-4K-AA1I-01A	TGCT	7.57
TCGA-X3-A8G4-01A	TGCT	7.18
TCGA-S6-A8JX-01A	TGCT	6.83
TCGA-EM-A2CO-01A	THCA	5.91
TCGA-EL-A3ZM-01A	THCA	7.05
TCGA-BJ-A190-01A	THCA	6.42
TCGA-DJ-A2PP-01A	THCA	7.32
TCGA-DJ-A2Q9-01A	THCA	7.22
TCGA-E8-A437-01A	THCA	6.59
TCGA-BJ-A2N9-01A	THCA	6.23
TCGA-DJ-A3VI-01A	THCA	6.64
TCGA-DJ-A3VM-01A	THCA	6.21
TCGA-EL-A3ZN-01A	THCA	7.05
TCGA-EL-A3CM-01A	THCA	6.97
TCGA-EM-A1CW-01A	THCA	6.81
TCGA-FE-A3PB-01A	THCA	6.46
TCGA-EL-A3CT-01A	THCA	7.69
TCGA-BJ-A0ZA-01A	THCA	7.02
TCGA-BJ-A3PU-01A	THCA	6.75
TCGA-DE-A0XZ-01A	THCA	6.89
TCGA-IM-A3EB-01A	THCA	7.56
TCGA-EL-A3GQ-01A	THCA	6.98
TCGA-EM-A3FN-01A	THCA	6.47
TCGA-FY-A3TY-01A	THCA	7.08
TCGA-DJ-A3UY-01A	THCA	7.17
TCGA-BJ-A28S-01A	THCA	7.07
TCGA-EL-A3CN-01A	THCA	6.83
TCGA-EM-A4FQ-01A	THCA	6.77
TCGA-J8-A3O0-01A	THCA	7.44
TCGA-EM-A4FV-01A	THCA	7.15
TCGA-ET-A3DS-01A	THCA	7.71
TCGA-EL-A4KH-01A	THCA	6.53
TCGA-DJ-A3UW-01A	THCA	6.13
TCGA-DJ-A3V3-01A	THCA	6.69
TCGA-FE-A231-01A	THCA	7.3
TCGA-EM-A2CU-01A	THCA	7.17
TCGA-DJ-A3V2-01A	THCA	6.73
TCGA-ET-A40P-01A	THCA	6.77
TCGA-DJ-A3UN-01A	THCA	7.17
TCGA-ET-A25M-01A	THCA	6.95
TCGA-EM-A2P0-01A	THCA	7.91
TCGA-ET-A2N5-01A	THCA	6.45
TCGA-FY-A76V-01A	THCA	6.95
TCGA-GE-A2C6-01A	THCA	7.08
TCGA-FY-A3W9-01A	THCA	6.79
TCGA-EM-A2CQ-01A	THCA	7.17
TCGA-DJ-A3V6-01A	THCA	7.04
TCGA-BJ-A0ZJ-01A	THCA	7.08

TCGA-EL-A3MY-01A	THCA	7.58
TCGA-BJ-A0ZH-01A	THCA	6.96
TCGA-EL-A3CU-01A	THCA	6.83
TCGA-KS-A4ID-01A	THCA	6.4
TCGA-EM-A2CK-01A	THCA	7.03
TCGA-EM-A4FR-01A	THCA	6.08
TCGA-DJ-A4UL-01A	THCA	7.03
TCGA-DJ-A2Q0-01A	THCA	6.13
TCGA-EL-A3CX-01A	THCA	6.63
TCGA-FY-A3ON-01A	THCA	7.77
TCGA-KS-A4IC-01A	THCA	6.59
TCGA-EL-A3ZO-01A	THCA	6.82
TCGA-FY-A4B3-01A	THCA	8.38
TCGA-J8-A3YF-01A	THCA	6.75
TCGA-EL-A3ZK-01A	THCA	6.58
TCGA-DE-A4MB-01A	THCA	6.67
TCGA-ET-A25L-01A	THCA	6.95
TCGA-EL-A4JW-01A	THCA	7.5
TCGA-EL-A4K6-01A	THCA	5.76
TCGA-EM-A4FU-01A	THCA	6.43
TCGA-ET-A2MX-01A	THCA	7.17
TCGA-EL-A3ZT-01A	THCA	7.54
TCGA-EM-A2CS-06A	THCA	7.57
TCGA-EM-A2CS-01A	THCA	7.65
TCGA-EL-A4K0-01A	THCA	6.8
TCGA-DJ-A13T-01A	THCA	7.77
TCGA-ET-A25R-01A	THCA	6.5
TCGA-BJ-A18Z-01A	THCA	6.77
TCGA-ET-A25P-01A	THCA	7.43
TCGA-EM-A3FK-01A	THCA	8.51
TCGA-ET-A39J-01A	THCA	7.92
TCGA-DJ-A2QC-01A	THCA	6.65
TCGA-EM-A3AO-01A	THCA	6.65
TCGA-DJ-A3V5-01A	THCA	7.17
TCGA-EL-A4JX-01A	THCA	6.09
TCGA-FK-A4UB-01A	THCA	6.77
TCGA-FE-A237-01A	THCA	7.03
TCGA-H2-A421-01A	THCA	6.39
TCGA-CE-A483-01A	THCA	7.48
TCGA-CE-A27D-01A	THCA	7.01
TCGA-ET-A4KN-01A	THCA	5.35
TCGA-IM-A3U2-01A	THCA	7.19
TCGA-FK-A3SG-01A	THCA	7.32
TCGA-EM-A3AR-01A	THCA	7.95
TCGA-EL-A3T0-01A	THCA	7.13
TCGA-BJ-A2N8-01A	THCA	7.56
TCGA-BJ-A45J-01A	THCA	7.34
TCGA-DE-A4M9-01A	THCA	7.49
TCGA-BJ-A45H-01A	THCA	7.28
TCGA-FY-A40M-01A	THCA	7.4
TCGA-FK-A3SB-01A	THCA	7.15
TCGA-FY-A40K-01A	THCA	6.44
TCGA-DE-A2OL-01A	THCA	7.28
TCGA-DJ-A13S-01A	THCA	6.31
TCGA-ET-A39K-01A	THCA	7.12
TCGA-BJ-A0Z9-01A	THCA	8.01
TCGA-EL-A4K9-01A	THCA	6.97
TCGA-IM-A41Z-01A	THCA	7.3

TCGA-DJ-A3V7-01A	THCA	6.84
TCGA-ET-A39T-01A	THCA	7.61
TCGA-E8-A417-01A	THCA	6.61
TCGA-EL-A3T6-01A	THCA	7.19
TCGA-FY-A40L-01A	THCA	6.47
TCGA-EL-A3H5-01A	THCA	5.57
TCGA-E8-A242-01A	THCA	7.08
TCGA-EM-A1CU-01A	THCA	7.87
TCGA-E3-A3DZ-01A	THCA	6.97
TCGA-ET-A25J-01A	THCA	6.63
TCGA-ET-A3BQ-01B	THCA	7.42
TCGA-EM-A4FH-01A	THCA	7.27
TCGA-FK-A3SD-01A	THCA	7.38
TCGA-EM-A3AK-01A	THCA	7.06
TCGA-J8-A42S-01A	THCA	7.43
TCGA-EL-A3D4-01A	THCA	7.17
TCGA-EL-A3CL-01A	THCA	7.48
TCGA-KS-A4I1-01A	THCA	7.13
TCGA-KS-A41I-01A	THCA	5.08
TCGA-BJ-A0ZG-01A	THCA	6.73
TCGA-FE-A3PC-01A	THCA	6.41
TCGA-BJ-A0ZE-01A	THCA	5.87
TCGA-DJ-A3V8-01A	THCA	7.31
TCGA-E8-A432-01A	THCA	6.54
TCGA-BJ-A3F0-01A	THCA	7.08
TCGA-DJ-A3UZ-01A	THCA	7.08
TCGA-DJ-A2Q2-01A	THCA	7.22
TCGA-EM-A2OX-01A	THCA	7.21
TCGA-EL-A3T7-01A	THCA	7.38
TCGA-EM-A3FO-01A	THCA	8.43
TCGA-L6-A4EU-01A	THCA	6.87
TCGA-FY-A3NN-01A	THCA	6.2
TCGA-H2-A3RI-01A	THCA	7
TCGA-FY-A3BL-01A	THCA	6.72
TCGA-DO-A2HM-01B	THCA	7.41
TCGA-DE-A69K-01A	THCA	7.26
TCGA-EL-A3GX-01A	THCA	7.14
TCGA-DE-A4MC-01A	THCA	6.92
TCGA-EM-A22Q-01A	THCA	5.97
TCGA-E8-A416-01A	THCA	6.97
TCGA-EL-A3CS-01A	THCA	7.09
TCGA-KS-A41F-01A	THCA	6.77
TCGA-FK-A3S3-01A	THCA	7.17
TCGA-E3-A3E1-01A	THCA	7.02
TCGA-FE-A22Z-01A	THCA	6.74
TCGA-BJ-A45K-01A	THCA	7.16
TCGA-E8-A2EA-01A	THCA	7.14
TCGA-ET-A3BP-01A	THCA	6.44
TCGA-EM-A1CV-01A	THCA	7.43
TCGA-BJ-A0Z0-01A	THCA	6.27
TCGA-BJ-A28Z-01A	THCA	6.29
TCGA-DE-A4MA-01A	THCA	6.49
TCGA-DJ-A13O-01A	THCA	7.05
TCGA-ET-A3BX-01A	THCA	8.46
TCGA-DJ-A13U-01A	THCA	7
TCGA-EM-A1YD-01A	THCA	6.89
TCGA-EM-A3FM-01A	THCA	6.91
TCGA-EL-A3T1-01A	THCA	6.48

TCGA-EM-A2P1-01A	THCA	6.5
TCGA-J8-A3YH-01A	THCA	7.19
TCGA-J8-A3YH-06A	THCA	6.88
TCGA-EL-A3TB-01A	THCA	7.04
TCGA-BJ-A45C-01A	THCA	7.36
TCGA-CE-A13K-01A	THCA	7.02
TCGA-DJ-A2PX-01A	THCA	6.89
TCGA-EL-A3ZS-01A	THCA	7.38
TCGA-DJ-A3UX-01A	THCA	7.11
TCGA-EM-A2P1-06A	THCA	6.93
TCGA-EM-A22M-01A	THCA	5.95
TCGA-L6-A4EP-01A	THCA	7.31
TCGA-BJ-A18Y-01A	THCA	6.84
TCGA-EL-A4K2-01A	THCA	6.88
TCGA-DJ-A4UT-01A	THCA	7.05
TCGA-DJ-A2Q3-01A	THCA	7.28
TCGA-FK-A3SE-01A	THCA	8.91
TCGA-FY-A2QD-01A	THCA	8.38
TCGA-ET-A3DR-01A	THCA	8.42
TCGA-DJ-A13M-01A	THCA	6.97
TCGA-ET-A25O-01A	THCA	6.81
TCGA-DJ-A4V4-01A	THCA	6.54
TCGA-EL-A3CZ-01A	THCA	7.33
TCGA-EM-A4FF-01A	THCA	6.97
TCGA-FY-A3R7-01A	THCA	7.16
TCGA-BJ-A4O9-01A	THCA	6.69
TCGA-EL-A3GW-01A	THCA	7.39
TCGA-DJ-A13P-01A	THCA	6.82
TCGA-EM-A3FL-01A	THCA	6.9
TCGA-EM-A2CT-01A	THCA	7.54
TCGA-IM-A3ED-01A	THCA	7.55
TCGA-KS-A4IB-01A	THCA	7
TCGA-DJ-A2PW-01A	THCA	7.37
TCGA-L6-A4EQ-01A	THCA	7.04
TCGA-ET-A3BO-01A	THCA	7.13
TCGA-DJ-A3VD-01A	THCA	7.02
TCGA-IM-A3U3-01A	THCA	6.78
TCGA-EM-A2CJ-01A	THCA	7.72
TCGA-DJ-A2Q1-01A	THCA	6.67
TCGA-EL-A3MW-01A	THCA	7.2
TCGA-FE-A233-01A	THCA	7.16
TCGA-BJ-A0Z2-01A	THCA	6.74
TCGA-EL-A3CV-01A	THCA	7.8
TCGA-BJ-A2NA-01A	THCA	7.29
TCGA-DJ-A13L-01A	THCA	6.67
TCGA-ET-A39L-01A	THCA	7.49
TCGA-BJ-A2N7-01A	THCA	7.06
TCGA-ET-A3BS-01A	THCA	6.63
TCGA-ET-A39O-01A	THCA	7.27
TCGA-FY-A3YR-01A	THCA	6.31
TCGA-EL-A3TA-01A	THCA	7.09
TCGA-J8-A3YE-01A	THCA	6.78
TCGA-DJ-A3UR-01A	THCA	6.71
TCGA-BJ-A0ZB-01A	THCA	6.76
TCGA-FE-A238-01A	THCA	6.84
TCGA-E8-A413-01A	THCA	6.99
TCGA-DJ-A3UK-01A	THCA	7.11
TCGA-KS-A4I7-01A	THCA	6.78

TCGA-EL-A3ZL-01A	THCA	6.59
TCGA-IM-A420-01A	THCA	7.02
TCGA-KS-A4I5-01A	THCA	7.45
TCGA-DJ-A3VE-01A	THCA	7.26
TCGA-DJ-A1QL-01A	THCA	6.9
TCGA-EM-A1YE-01A	THCA	5.98
TCGA-FE-A234-01A	THCA	7.25
TCGA-DJ-A1QH-01A	THCA	7.17
TCGA-E3-A3E2-01A	THCA	7.06
TCGA-EM-A4G1-01A	THCA	6.97
TCGA-DE-A4MD-06A	THCA	6.18
TCGA-DE-A4MD-01A	THCA	6.59
TCGA-FY-A3I4-01A	THCA	7.7
TCGA-DJ-A3VL-01A	THCA	6.53
TCGA-FE-A235-01A	THCA	7.1
TCGA-DJ-A1QG-01A	THCA	6.08
TCGA-EM-A2OY-01A	THCA	6.4
TCGA-DJ-A4UR-01A	THCA	6.59
TCGA-ET-A40S-01A	THCA	7.5
TCGA-BJ-A45I-01A	THCA	7.08
TCGA-DJ-A3UO-01A	THCA	7.27
TCGA-E8-A436-01A	THCA	6.81
TCGA-ET-A3BT-01A	THCA	7.11
TCGA-4C-A93U-01A	THCA	7.33
TCGA-EM-A3AJ-01A	THCA	7.27
TCGA-DJ-A1QE-01A	THCA	7.41
TCGA-L6-A4ET-01A	THCA	7.25
TCGA-BJ-A45F-01A	THCA	5.55
TCGA-MK-A4N6-01A	THCA	7.59
TCGA-DJ-A1QD-01A	THCA	7.44
TCGA-ET-A25N-01A	THCA	8.14
TCGA-EL-A4K7-01A	THCA	6.83
TCGA-DJ-A3VJ-01A	THCA	7.54
TCGA-EL-A3D5-01A	THCA	7.27
TCGA-H2-A26U-01A	THCA	6.8
TCGA-FY-A3R6-01A	THCA	6.45
TCGA-E8-A419-01A	THCA	6.48
TCGA-EL-A3H1-01A	THCA	6.78
TCGA-DE-A4M8-01A	THCA	6.04
TCGA-ET-A2N3-01B	THCA	6.48
TCGA-DE-A69J-01A	THCA	7.6
TCGA-EL-A3GO-01A	THCA	7.36
TCGA-EL-A4K1-01A	THCA	5.77
TCGA-EM-A2OW-01A	THCA	6.66
TCGA-DJ-A2PS-01A	THCA	7.55
TCGA-ET-A3DT-01A	THCA	6.5
TCGA-DJ-A2QA-01A	THCA	7.13
TCGA-EL-A3T8-01A	THCA	7.19
TCGA-DJ-A3VF-01A	THCA	6.81
TCGA-EL-A3CY-01A	THCA	6.93
TCGA-EL-A3GR-01A	THCA	8.26
TCGA-BJ-A192-01A	THCA	7.17
TCGA-DJ-A3VB-01A	THCA	6.97
TCGA-EM-A3FP-01A	THCA	6.28
TCGA-DJ-A2QB-01A	THCA	6.29
TCGA-DJ-A4UW-01A	THCA	7.3
TCGA-BJ-A28V-01A	THCA	7.14
TCGA-DE-A0Y3-01B	THCA	7.74

TCGA-EM-A22N-01A	THCA	6.28
TCGA-EM-A3FJ-01A	THCA	7.12
TCGA-DJ-A3VG-01A	THCA	7.14
TCGA-BJ-A291-01A	THCA	6.58
TCGA-CE-A3ME-01A	THCA	7.36
TCGA-DJ-A3UM-01A	THCA	7.06
TCGA-E3-A3DY-01A	THCA	6.96
TCGA-FY-A3R9-01A	THCA	6.03
TCGA-EL-A3GP-01A	THCA	6.5
TCGA-EL-A3GV-01A	THCA	7.15
TCGA-EM-A22I-01A	THCA	6.8
TCGA-DJ-A3UV-01A	THCA	7.23
TCGA-DJ-A1QF-01A	THCA	6.45
TCGA-BJ-A45D-01A	THCA	6.28
TCGA-EL-A3ZP-01A	THCA	7.16
TCGA-E3-A3E5-01A	THCA	7.21
TCGA-BJ-A28T-01A	THCA	6.88
TCGA-DJ-A3UT-01A	THCA	6.43
TCGA-CE-A482-01A	THCA	7.15
TCGA-E8-A44K-01A	THCA	6.61
TCGA-DJ-A2Q6-01A	THCA	7.3
TCGA-EM-A22L-01A	THCA	6.83
TCGA-EM-A3SY-01A	THCA	6.44
TCGA-EM-A2P2-01A	THCA	7.69
TCGA-ET-A3BV-01A	THCA	7.53
TCGA-EL-A3D1-01A	THCA	7.26
TCGA-BJ-A28X-01A	THCA	6.98
TCGA-EM-A1YB-01A	THCA	6.08
TCGA-DJ-A3V0-01A	THCA	6.83
TCGA-EM-A22J-01A	THCA	6.17
TCGA-EM-A2P3-01A	THCA	7.28
TCGA-ET-A3DU-01A	THCA	6.11
TCGA-EL-A4KG-01A	THCA	7.15
TCGA-EM-A3ST-01A	THCA	6.89
TCGA-ET-A39S-01A	THCA	7.67
TCGA-DJ-A2PO-01A	THCA	6.96
TCGA-ET-A25G-01A	THCA	7.61
TCGA-EL-A3H4-01A	THCA	7.97
TCGA-EL-A3GU-01A	THCA	7
TCGA-EM-A1CS-01A	THCA	6.67
TCGA-DJ-A2PQ-01A	THCA	7.1
TCGA-EM-A4FO-01A	THCA	6.27
TCGA-BJ-A0YZ-01A	THCA	7.32
TCGA-BJ-A28W-01A	THCA	5.98
TCGA-FE-A230-01A	THCA	6.84
TCGA-EL-A3ZR-01A	THCA	6.49
TCGA-ET-A39R-01A	THCA	6.99
TCGA-EM-A3O8-01A	THCA	6.76
TCGA-DO-A1K0-01A	THCA	7.05
TCGA-ET-A3DV-01A	THCA	6.97
TCGA-EL-A3H2-01A	THCA	7.38
TCGA-ET-A40Q-01A	THCA	6.25
TCGA-DJ-A4V5-01A	THCA	7.12
TCGA-ET-A39N-01A	THCA	7.54
TCGA-BJ-A0ZF-01A	THCA	7.08
TCGA-EL-A3T9-01A	THCA	6.76
TCGA-EL-A3T2-01A	THCA	7.03
TCGA-EM-A3OB-01A	THCA	6.65

TCGA-DJ-A2Q7-01A	THCA	7
TCGA-DJ-A3VK-01A	THCA	6.91
TCGA-DJ-A13V-01A	THCA	7.11
TCGA-DJ-A1QQ-01A	THCA	8.01
TCGA-J8-A3YG-01A	THCA	7.01
TCGA-BJ-A3EZ-01A	THCA	7.03
TCGA-QD-A8IV-01A	THCA	6.56
TCGA-E8-A415-01A	THCA	7.08
TCGA-BJ-A45G-01A	THCA	6.84
TCGA-EL-A4KD-01A	THCA	6.68
TCGA-FY-A3R8-01A	THCA	7.08
TCGA-DJ-A4V2-01A	THCA	7.42
TCGA-CE-A484-01A	THCA	7.03
TCGA-EM-A22K-01A	THCA	6.47
TCGA-FK-A3SH-01A	THCA	7.75
TCGA-ET-A39M-01A	THCA	6.1
TCGA-EL-A3MX-01A	THCA	6.74
TCGA-E8-A3X7-01A	THCA	6.52
TCGA-FE-A239-01A	THCA	6.32
TCGA-J8-A3YD-01A	THCA	7.39
TCGA-J8-A3NZ-01A	THCA	7.07
TCGA-ET-A39P-01A	THCA	7.04
TCGA-EL-A4JZ-01A	THCA	6.85
TCGA-EL-A3CW-01A	THCA	7.17
TCGA-FY-A3NP-01A	THCA	7.22
TCGA-H2-A3RH-01A	THCA	7.24
TCGA-EM-A1YA-01A	THCA	6.47
TCGA-J8-A3O2-06A	THCA	6.97
TCGA-EM-A2CN-01A	THCA	7.2
TCGA-BJ-A28R-01A	THCA	7.31
TCGA-EM-A3AI-01A	THCA	7
TCGA-EM-A22O-01A	THCA	6.99
TCGA-BJ-A0ZC-01A	THCA	6.1
TCGA-DE-A0Y2-01A	THCA	8
TCGA-FY-A4B0-01A	THCA	6.74
TCGA-DJ-A1QN-01A	THCA	6.82
TCGA-EL-A3D6-01A	THCA	7.06
TCGA-ET-A2N0-01A	THCA	6.48
TCGA-EM-A3FR-01A	THCA	8.03
TCGA-ET-A4KQ-01A	THCA	7.08
TCGA-ET-A3BW-01A	THCA	7.64
TCGA-EL-A3ZG-01A	THCA	6.83
TCGA-EL-A3N2-01A	THCA	7.01
TCGA-J8-A4HY-01A	THCA	6.6
TCGA-EM-A3OA-01A	THCA	6.75
TCGA-DE-A3KN-01A	THCA	6.26
TCGA-EM-A3AL-01A	THCA	7.36
TCGA-EM-A3AP-01A	THCA	7.12
TCGA-ET-A3DO-01A	THCA	7.05
TCGA-EM-A4FK-01A	THCA	7.41
TCGA-DJ-A2PU-01A	THCA	7.09
TCGA-BJ-A0Z5-01A	THCA	6.39
TCGA-ET-A25I-01A	THCA	5.95
TCGA-DJ-A2Q5-01A	THCA	6.67
TCGA-EM-A3SX-01A	THCA	7.3
TCGA-EM-A2OZ-01A	THCA	7.54
TCGA-BJ-A191-01A	THCA	8.21
TCGA-ET-A40R-01A	THCA	6.92

TCGA-DJ-A4UP-01A	THCA	6.83
TCGA-CE-A481-01A	THCA	7.09
TCGA-DJ-A3UP-01A	THCA	7.11
TCGA-EL-A3GZ-01A	THCA	7.57
TCGA-EM-A3O9-01A	THCA	5.4
TCGA-EL-A3N3-01A	THCA	7.15
TCGA-EM-A3O6-01A	THCA	5.75
TCGA-EM-A2CP-01A	THCA	6.76
TCGA-ET-A25K-01A	THCA	7.45
TCGA-EL-A3H3-01A	THCA	7.07
TCGA-DJ-A1QM-01A	THCA	7.13
TCGA-DJ-A2PT-01A	THCA	7.02
TCGA-BJ-A290-01A	THCA	7.17
TCGA-EM-A3O7-01A	THCA	6.61
TCGA-ET-A3DW-01A	THCA	7.19
TCGA-KS-A4I9-01A	THCA	6.61
TCGA-IM-A41Y-01A	THCA	7.09
TCGA-FE-A232-01A	THCA	6.97
TCGA-ET-A2MZ-01A	THCA	7.77
TCGA-DJ-A3UU-01A	THCA	7.28
TCGA-ET-A3BU-01A	THCA	7.79
TCGA-J8-A3O2-01A	THCA	7.29
TCGA-EL-A3D0-01A	THCA	6.89
TCGA-FY-A3I5-01B	THCA	5.72
TCGA-EM-A3AN-01A	THCA	7.26
TCGA-DJ-A2PZ-01A	THCA	6.87
TCGA-E3-A3E3-01A	THCA	6.96
TCGA-DJ-A4UQ-01A	THCA	7.69
TCGA-BJ-A3PT-01A	THCA	6.32
TCGA-KS-A41J-01A	THCA	6.81
TCGA-DJ-A3UQ-01A	THCA	7.24
TCGA-E8-A414-01A	THCA	7.09
TCGA-IM-A4EB-01A	THCA	7.27
TCGA-EL-A3GS-01A	THCA	7.89
TCGA-DJ-A1QI-01A	THCA	7.36
TCGA-ET-A3BN-01A	THCA	8.15
TCGA-FE-A236-01A	THCA	7.12
TCGA-E8-A44M-01A	THCA	6.73
TCGA-EM-A3O3-01A	THCA	6.42
TCGA-EM-A2CM-01A	THCA	5.84
TCGA-ET-A39I-01A	THCA	7.27
TCGA-EL-A3CP-01A	THCA	7.47
TCGA-FE-A3PD-01A	THCA	6.8
TCGA-ET-A2N4-01A	THCA	7.1
TCGA-FY-A40N-01A	THCA	7.18
TCGA-DJ-A13R-01A	THCA	5.17
TCGA-EL-A4K4-01A	THCA	7.4
TCGA-DJ-A2PR-01A	THCA	7.81
TCGA-BJ-A0Z3-01A	THCA	6.89
TCGA-BJ-A45E-01A	THCA	5.45
TCGA-DJ-A13X-01A	THCA	7.21
TCGA-DJ-A13W-01A	THCA	4.02
TCGA-MK-A4N9-01A	THCA	7
TCGA-EM-A2CL-01A	THCA	7.13
TCGA-DJ-A3VA-01A	THCA	6.73
TCGA-EL-A3CR-01A	THCA	6.56
TCGA-H2-A2K9-01A	THCA	7.45
TCGA-EM-A1CT-01A	THCA	6.67

TCGA-ET-A40T-01A	THCA	6.99
TCGA-DE-A7U5-01A	THCA	7.4
TCGA-EL-A3ZQ-01A	THCA	5.78
TCGA-DJ-A3V9-01A	THCA	7.5
TCGA-DJ-A3V4-01A	THCA	7.04
TCGA-EM-A3SZ-01A	THCA	6.75
TCGA-FE-A3PA-01A	THCA	6.4
TCGA-DO-A1JZ-01A	THCA	7.43
TCGA-EL-A3ZH-01A	THCA	7.42
TCGA-ET-A3DP-01A	THCA	7.3
TCGA-EM-A2CR-01A	THCA	6.73
TCGA-MK-A84Z-01A	THCA	6.44
TCGA-EM-A22P-01A	THCA	7.32
TCGA-EL-A4KI-01A	THCA	6.28
TCGA-E8-A2JQ-01A	THCA	7.06
TCGA-FY-A3NM-01A	THCA	7.32
TCGA-EL-A3MZ-01A	THCA	6.93
TCGA-EM-A4FN-01A	THCA	6.18
TCGA-ET-A3DQ-01A	THCA	6.49
TCGA-BJ-A3PR-01A	THCA	6.75
TCGA-EM-A3FQ-01A	THCA	7.4
TCGA-ET-A2MY-01A	THCA	7.21
TCGA-E8-A418-01A	THCA	6.25
TCGA-DJ-A4V0-01A	THCA	6.87
TCGA-DJ-A2PY-01A	THCA	7.4
TCGA-J8-A4HW-06A	THCA	7.23
TCGA-KS-A4I3-01A	THCA	7.09
TCGA-EL-A3H7-01A	THCA	7.27
TCGA-BJ-A2P4-01A	THCA	6.26
TCGA-J8-A4HW-01A	THCA	8.29
TCGA-EL-A3CO-01A	THCA	7.63
TCGA-FE-A23A-01A	THCA	6.45
TCGA-EM-A3SU-06A	THCA	6.85
TCGA-EM-A3SU-01A	THCA	6.63
TCGA-E8-A434-01A	THCA	7.14
TCGA-DJ-A2Q4-01A	THCA	8
TCGA-EL-A3H8-01A	THCA	7.75
TCGA-MK-A4N7-01A	THCA	7.47
TCGA-EM-A3AQ-01A	THCA	6.98
TCGA-DJ-A1QO-01A	THCA	7.73
TCGA-DJ-A2PN-01A	THCA	7.6
TCGA-EM-A2OV-01A	THCA	6.94
TCGA-DJ-A2PV-01A	THCA	7.53
TCGA-E3-A3E0-01A	THCA	7.88
TCGA-EL-A3T3-01A	THCA	7.28
TCGA-FY-A4B4-01A	THCA	6.87
TCGA-FY-A3RA-01A	THCA	7.32
TCGA-FY-A3WA-01A	THCA	6.87
TCGA-KS-A41L-01A	THCA	6.43
TCGA-E8-A433-01A	THCA	6.67
TCGA-EL-A4JV-01A	THCA	6.65
TCGA-DJ-A3US-01A	THCA	6.97
TCGA-H2-A422-01A	THCA	7.17
TCGA-CE-A485-01A	THCA	7.38
TCGA-EM-A1YC-01A	THCA	5.75
TCGA-EM-A4FM-01A	THCA	6.79
TCGA-EL-A3GY-01A	THCA	7.31
TCGA-CE-A3MD-01A	THCA	6.7

TCGA-BJ-A4O8-01A	THCA	6.84
TCGA-EM-A3FQ-06A	THCA	7.02
TCGA-E8-A438-01A	THCA	6.43
TCGA-J8-A3O1-01A	THCA	7.8
TCGA-X7-A8D7-01A	THYM	6.64
TCGA-XM-A8RH-01A	THYM	7.06
TCGA-X7-A8DE-01A	THYM	6.7
TCGA-ZB-A96G-01A	THYM	6.53
TCGA-5G-A9ZZ-01A	THYM	6.84
TCGA-4V-A9QR-01A	THYM	5.96
TCGA-4V-A9QU-01A	THYM	7.18
TCGA-XM-A8RF-01A	THYM	6.93
TCGA-3T-AA9L-01A	THYM	6.66
TCGA-4X-A9F9-01A	THYM	6.12
TCGA-ZB-A96M-01A	THYM	6.88
TCGA-XU-AAXW-01A	THYM	6.65
TCGA-3G-AB0Q-01A	THYM	6.78
TCGA-ZT-A8OM-01A	THYM	6.01
TCGA-ZB-A96H-01A	THYM	6.63
TCGA-ZB-A961-01A	THYM	6.03
TCGA-5U-AB0F-01A	THYM	6.01
TCGA-X7-A8DB-01A	THYM	6.64
TCGA-XM-AAZ1-01A	THYM	6.03
TCGA-XM-A8RE-01A	THYM	6.62
TCGA-X7-A8DG-01A	THYM	6.34
TCGA-3G-AB14-01A	THYM	6.38
TCGA-4X-A9FA-01A	THYM	6.53
TCGA-ZB-A96C-01A	THYM	7.19
TCGA-XH-A853-01A	THYM	6.92
TCGA-ZB-A96D-01A	THYM	6.91
TCGA-YT-A95F-01A	THYM	7.01
TCGA-X7-A8D9-01A	THYM	6.98
TCGA-XU-A92O-01A	THYM	6.26
TCGA-X7-A8M8-01A	THYM	6.57
TCGA-5V-A9RR-01A	THYM	6.42
TCGA-4X-A9FC-01A	THYM	7.1
TCGA-XU-A92W-01A	THYM	6.71
TCGA-ZB-A96P-01A	THYM	6.93
TCGA-ZC-AAAF-01A	THYM	6.73
TCGA-4V-A9QI-01A	THYM	6.48
TCGA-ZB-A96Q-01A	THYM	7.38
TCGA-4V-A9QX-01A	THYM	7.06
TCGA-ZB-A96F-01A	THYM	5.92
TCGA-X7-A8M1-01A	THYM	7.18
TCGA-X7-A8M4-01A	THYM	6.9
TCGA-XM-AAZ3-01A	THYM	6.97
TCGA-ZB-A964-01A	THYM	6.62
TCGA-ZL-A9V6-01A	THYM	7.02
TCGA-ZB-A965-01A	THYM	6.62
TCGA-XU-AAY1-01A	THYM	6.5
TCGA-4V-A9QT-01A	THYM	6.85
TCGA-X7-A8M7-01A	THYM	6.3
TCGA-XU-AAXZ-01A	THYM	4.77
TCGA-XM-AAZ2-01A	THYM	6.84
TCGA-X7-A8M0-01A	THYM	6.44
TCGA-XM-A8R9-01A	THYM	6.49
TCGA-X7-A8M5-01A	THYM	6.23
TCGA-XM-A8R8-01A	THYM	5.8

TCGA-YT-A95H-01A	THYM	6.87
TCGA-ZB-A966-01A	THYM	6.26
TCGA-ZC-AAAH-01A	THYM	6.28
TCGA-4V-A9QN-01A	THYM	5.42
TCGA-3G-AB0O-01A	THYM	6.76
TCGA-ZB-A96V-01A	THYM	6.41
TCGA-4V-A9QJ-01A	THYM	6.23
TCGA-XM-A8RC-01A	THYM	6.5
TCGA-4V-A9QW-01A	THYM	6.34
TCGA-X7-A8DD-01A	THYM	6.51
TCGA-XM-A8RL-01A	THYM	6.86
TCGA-XU-A92T-01A	THYM	6.91
TCGA-ZB-A96A-01A	THYM	6.74
TCGA-XU-A92U-01A	THYM	6.98
TCGA-3Q-A9WF-01A	THYM	6.74
TCGA-XM-A8RG-01A	THYM	6.2
TCGA-XU-A92Y-01A	THYM	6.3
TCGA-ZB-A96L-01A	THYM	6.81
TCGA-XU-A92X-01A	THYM	5.54
TCGA-X7-A8DJ-01A	THYM	7.01
TCGA-ZB-A962-01A	THYM	6.96
TCGA-XM-A8RB-01A	THYM	6.63
TCGA-XU-A930-01A	THYM	6.13
TCGA-XU-A932-01A	THYM	6.12
TCGA-4X-A9FD-01A	THYM	6.51
TCGA-3S-AAYX-01A	THYM	6.74
TCGA-ZB-A96B-01A	THYM	6.73
TCGA-XU-A92V-01A	THYM	7.22
TCGA-ZC-AAAA-01A	THYM	6.37
TCGA-X7-A8M3-01A	THYM	5.66
TCGA-XU-AAXY-01A	THYM	6.1
TCGA-XU-AAXX-01A	THYM	6.53
TCGA-X7-A8M6-01A	THYM	5.96
TCGA-XM-A8RI-01A	THYM	6.12
TCGA-5K-AAAP-01A	THYM	6.66
TCGA-YT-A95G-01A	THYM	6.55
TCGA-XU-AAY0-01A	THYM	6.43
TCGA-ZC-AAA7-01A	THYM	5.67
TCGA-YT-A95D-01A	THYM	7.06
TCGA-5U-AB0D-01A	THYM	6.13
TCGA-3S-A8YW-01A	THYM	6.76
TCGA-XU-A936-01A	THYM	5.87
TCGA-5U-AB0E-01A	THYM	5.72
TCGA-4X-A9FB-01A	THYM	6.86
TCGA-X7-A8D6-01A	THYM	7.26
TCGA-4V-A9QM-01A	THYM	7.02
TCGA-ZB-A96K-01A	THYM	7.21
TCGA-3G-AB0T-01A	THYM	6.2
TCGA-ZB-A96O-01A	THYM	6.42
TCGA-X7-A8D8-01A	THYM	6.87
TCGA-XM-A8RD-01A	THYM	6.13
TCGA-ZB-A96R-01A	THYM	6.31
TCGA-XU-AAXV-01A	THYM	6.53
TCGA-X7-A8DF-01A	THYM	6.66
TCGA-XU-A931-01A	THYM	5.64
TCGA-3G-AB19-01A	THYM	6.94
TCGA-ZB-A96E-01A	THYM	6.48
TCGA-XU-A92Z-01A	THYM	7.03

TCGA-ZB-A96I-01A	THYM	6.06
TCGA-XU-A933-01A	THYM	7.16
TCGA-4V-A9QS-01A	THYM	6.31
TCGA-YT-A95E-01A	THYM	7.03
TCGA-4V-A9QL-01A	THYM	6.6
TCGA-ZB-A969-01A	THYM	7.1
TCGA-XU-A92Q-01A	THYM	6.42
TCGA-ZB-A963-01A	THYM	6.85
TCGA-D1-A17H-01A	UCEC	5.05
TCGA-D1-A176-01A	UCEC	5.69
TCGA-AX-A3G8-01A	UCEC	6.19
TCGA-AX-A3FX-01A	UCEC	5.33
TCGA-D1-A17C-01A	UCEC	4.99
TCGA-D1-A2G0-01A	UCEC	7.43
TCGA-AX-A2HK-01A	UCEC	4.84
TCGA-AJ-A23O-01A	UCEC	5.86
TCGA-EY-A1GR-01A	UCEC	5.25
TCGA-AJ-A3NH-01A	UCEC	5.21
TCGA-EO-A1Y7-01A	UCEC	6.15
TCGA-AX-A3G7-01A	UCEC	6.65
TCGA-A5-A0GA-01A	UCEC	5.82
TCGA-AP-A056-01A	UCEC	6.77
TCGA-AJ-A23N-01A	UCEC	6.49
TCGA-B5-A0JX-01A	UCEC	6.45
TCGA-BG-A0MO-01A	UCEC	5.81
TCGA-EO-A3KW-01A	UCEC	6.39
TCGA-AP-A0LV-01A	UCEC	5.51
TCGA-D1-A16F-01A	UCEC	6.57
TCGA-AP-A0LF-01A	UCEC	5.89
TCGA-KP-A3W4-01A	UCEC	6.15
TCGA-AJ-A3BG-01A	UCEC	7.08
TCGA-A5-A0GR-01A	UCEC	4.91
TCGA-AP-A0LH-01A	UCEC	6.46
TCGA-D1-A3DG-01A	UCEC	5.46
TCGA-AX-A2IO-01A	UCEC	5.89
TCGA-A5-AB3J-01A	UCEC	6.37
TCGA-B5-A0JU-01B	UCEC	6.33
TCGA-A5-A0G3-01A	UCEC	5.78
TCGA-AX-A2HG-01A	UCEC	6.91
TCGA-EY-A1GQ-01A	UCEC	5.79
TCGA-DF-A2KU-01A	UCEC	6.18
TCGA-BG-A0MG-01A	UCEC	6.35
TCGA-BK-A0C9-01A	UCEC	5.61
TCGA-EY-A72D-01A	UCEC	7.18
TCGA-AX-A2H2-01A	UCEC	6.14
TCGA-D1-A16E-01A	UCEC	6.1
TCGA-E6-A8L9-01A	UCEC	6.15
TCGA-B5-A3FC-01A	UCEC	6.61
TCGA-AX-A0J1-01A	UCEC	6.15
TCGA-BG-A0VZ-01A	UCEC	6.71
TCGA-DI-A2QT-01A	UCEC	7.02
TCGA-B5-A3FD-01A	UCEC	5.27
TCGA-AX-A063-01A	UCEC	5.76
TCGA-D1-A17T-01A	UCEC	6.22
TCGA-D1-A177-01A	UCEC	6.64
TCGA-EY-A210-01A	UCEC	6.33
TCGA-EC-A1QX-01A	UCEC	5.83
TCGA-BG-A2AE-01A	UCEC	6.88

TCGA-D1-A1NZ-01A	UCEC	5.93
TCGA-AX-A06L-01A	UCEC	5.31
TCGA-D1-A15V-01A	UCEC	7.67
TCGA-D1-A17B-01A	UCEC	5.66
TCGA-EY-A1GW-01A	UCEC	4.39
TCGA-D1-A102-01A	UCEC	6.33
TCGA-BS-A0U8-01A	UCEC	6.25
TCGA-AX-A1CI-01A	UCEC	4.79
TCGA-BG-A0MA-01A	UCEC	5.73
TCGA-D1-A174-01A	UCEC	5.29
TCGA-H5-A2HR-01A	UCEC	7.02
TCGA-EY-A4KR-01A	UCEC	6.66
TCGA-AX-A0IW-01A	UCEC	5.32
TCGA-PG-A914-01A	UCEC	6.12
TCGA-B5-A11O-01A	UCEC	6.45
TCGA-AP-A0L8-01A	UCEC	6.12
TCGA-D1-A16O-01A	UCEC	5.98
TCGA-BG-A0MU-01A	UCEC	5.1
TCGA-A5-A7WJ-01A	UCEC	7.01
TCGA-EY-A1GH-01A	UCEC	7.13
TCGA-4E-A92E-01A	UCEC	6.4
TCGA-B5-A0K4-01A	UCEC	5.09
TCGA-AP-A052-01A	UCEC	5.08
TCGA-FI-A2CY-01A	UCEC	6.93
TCGA-B5-A1MS-01B	UCEC	5.41
TCGA-BG-A0MH-01A	UCEC	6.35
TCGA-BK-A26L-01A	UCEC	6.96
TCGA-D1-A0ZO-01A	UCEC	6.21
TCGA-D1-A16B-01A	UCEC	5.89
TCGA-AP-A0LS-01A	UCEC	6.14
TCGA-D1-A15Z-01A	UCEC	6.58
TCGA-A5-A2K5-01A	UCEC	6.16
TCGA-AP-A1DM-01A	UCEC	6.58
TCGA-E6-A2P8-01A	UCEC	7.17
TCGA-D1-A169-01A	UCEC	5.25
TCGA-EO-A22S-01A	UCEC	6.51
TCGA-B5-A0JR-01A	UCEC	6.19
TCGA-AX-A3FS-01A	UCEC	7.7
TCGA-D1-A1NS-01A	UCEC	5.81
TCGA-AX-A1CJ-01A	UCEC	5.68
TCGA-AX-A2HD-01A	UCEC	6.18
TCGA-A5-A0RA-01A	UCEC	6.44
TCGA-AP-A0LJ-01A	UCEC	5.7
TCGA-EC-A24G-01A	UCEC	6.73
TCGA-AP-A051-01A	UCEC	6.65
TCGA-BS-A0TA-01A	UCEC	5.82
TCGA-BS-A0UV-01A	UCEC	5.44
TCGA-EY-A2ON-01A	UCEC	7.4
TCGA-EO-A2CG-01A	UCEC	6.47
TCGA-EC-A1NJ-01A	UCEC	5.75
TCGA-AX-A1C8-01A	UCEC	6.35
TCGA-AJ-A5DW-01A	UCEC	6.58
TCGA-AX-A1CC-01A	UCEC	6.71
TCGA-A5-A2K4-01A	UCEC	7.07
TCGA-SL-A6J9-01A	UCEC	5.89
TCGA-AP-A0LL-01A	UCEC	3.59
TCGA-A5-A0GQ-01A	UCEC	5.45
TCGA-B5-A11Z-01A	UCEC	6.34

TCGA-D1-A15W-01A	UCEC	6.53
TCGA-D1-A0ZU-01A	UCEC	6.59
TCGA-DI-A1BY-01A	UCEC	5.21
TCGA-AP-A054-01A	UCEC	6.43
TCGA-BG-A0MS-01A	UCEC	6.32
TCGA-BG-A0LX-01A	UCEC	6.28
TCGA-AJ-A3BH-01A	UCEC	7.46
TCGA-EY-A1GX-01A	UCEC	6.89
TCGA-B5-A0JN-01A	UCEC	5.8
TCGA-BS-A0V6-01A	UCEC	6.07
TCGA-PG-A917-01A	UCEC	6.69
TCGA-BG-A0MI-01A	UCEC	5.97
TCGA-B5-A11G-01A	UCEC	4.76
TCGA-KP-A3W0-01A	UCEC	6.48
TCGA-B5-A11S-01A	UCEC	6.61
TCGA-BS-A0U5-01A	UCEC	5.78
TCGA-EY-A1H0-01A	UCEC	6.56
TCGA-DI-A1NO-01A	UCEC	6.97
TCGA-AP-A05A-01A	UCEC	4.65
TCGA-EY-A1GL-01A	UCEC	5.61
TCGA-B5-A0JT-01A	UCEC	7
TCGA-AJ-A3NF-01A	UCEC	7.22
TCGA-BG-A0VT-01A	UCEC	6.09
TCGA-EY-A1G8-01A	UCEC	5.64
TCGA-D1-A17N-01A	UCEC	4.64
TCGA-BG-A0M6-01A	UCEC	6.44
TCGA-BK-A0CC-01A	UCEC	5.86
TCGA-D1-A2G5-01A	UCEC	6.62
TCGA-D1-A1O7-01A	UCEC	5.6
TCGA-AP-A05J-01A	UCEC	5.04
TCGA-EY-A214-01A	UCEC	5.46
TCGA-B5-A11J-01A	UCEC	6.15
TCGA-DI-A0WH-01A	UCEC	5.65
TCGA-AJ-A6NU-01A	UCEC	6.82
TCGA-B5-A0K9-01A	UCEC	6.38
TCGA-EO-A3L0-01A	UCEC	6.44
TCGA-AW-A1PO-01A	UCEC	5.34
TCGA-AP-A1E3-01A	UCEC	7
TCGA-D1-A3JP-01A	UCEC	6.38
TCGA-AP-A1DK-01A	UCEC	5.55
TCGA-AJ-A8CV-01A	UCEC	6.15
TCGA-AX-A1C4-01A	UCEC	7.46
TCGA-AX-A3FZ-01A	UCEC	6.76
TCGA-D1-A16I-01A	UCEC	5.36
TCGA-DF-A2KY-01A	UCEC	6.08
TCGA-B5-A0K6-01A	UCEC	5.02
TCGA-EY-A548-01A	UCEC	6.1
TCGA-BS-A0TE-01A	UCEC	6.71
TCGA-EO-A2CH-01A	UCEC	5.62
TCGA-EO-A22X-01A	UCEC	6.43
TCGA-AP-A1E4-01A	UCEC	6.24
TCGA-D1-A0ZR-01A	UCEC	6.09
TCGA-AJ-A3EK-01A	UCEC	7.1
TCGA-B5-A1MV-01A	UCEC	6.31
TCGA-AX-A3G3-01A	UCEC	6.19
TCGA-5B-A90C-01A	UCEC	6.55
TCGA-AX-A1CF-01A	UCEC	6.77
TCGA-D1-A16G-01A	UCEC	6.38

TCGA-D1-A160-01A	UCEC	5.9
TCGA-AX-A05T-01A	UCEC	6.7
TCGA-BK-A0CC-01B	UCEC	6.12
TCGA-SJ-A6ZI-01A	UCEC	6.77
TCGA-KP-A3W3-01A	UCEC	5.95
TCGA-D1-A168-01A	UCEC	5.48
TCGA-B5-A1MR-01A	UCEC	5.57
TCGA-DI-A1C3-01A	UCEC	5.89
TCGA-BK-A0CA-01A	UCEC	6.59
TCGA-FI-A2EU-01A	UCEC	5.85
TCGA-AP-A059-01A	UCEC	6.85
TCGA-D1-A16R-01A	UCEC	5.84
TCGA-AP-A05O-01A	UCEC	6.24
TCGA-AX-A1CK-01A	UCEC	5.26
TCGA-BG-A2AD-01A	UCEC	6.55
TCGA-BS-A0T9-01A	UCEC	4.98
TCGA-FI-A2D2-01A	UCEC	5.51
TCGA-DF-A2KS-01A	UCEC	6.21
TCGA-PG-A916-01A	UCEC	6.27
TCGA-B5-A0JY-01A	UCEC	5.96
TCGA-D1-A16D-01A	UCEC	6.08
TCGA-AX-A1CP-01A	UCEC	6.56
TCGA-BS-A0UT-01A	UCEC	5.54
TCGA-B5-A1MW-01A	UCEC	7.26
TCGA-AX-A3G6-01A	UCEC	5.35
TCGA-E6-A1M0-01A	UCEC	6.52
TCGA-EY-A1GV-01A	UCEC	5.39
TCGA-BS-A0V4-01A	UCEC	6.09
TCGA-EY-A1GC-01A	UCEC	6.01
TCGA-BK-A13B-01A	UCEC	6.48
TCGA-BG-A221-01A	UCEC	6.13
TCGA-AP-A0LP-01A	UCEC	5.82
TCGA-PG-A6IB-01A	UCEC	6.38
TCGA-B5-A11R-01A	UCEC	6.65
TCGA-DI-A2QU-01A	UCEC	5.41
TCGA-D1-A1O5-01A	UCEC	6.32
TCGA-D1-A175-01A	UCEC	6.34
TCGA-PG-A915-01A	UCEC	7.28
TCGA-AX-A06F-01A	UCEC	6.79
TCGA-BG-A0RY-01A	UCEC	5.71
TCGA-B5-A11W-01A	UCEC	5.62
TCGA-A5-A2K7-01A	UCEC	6
TCGA-B5-A11I-01A	UCEC	6.38
TCGA-QF-A5YT-01A	UCEC	5.68
TCGA-B5-A1MU-01A	UCEC	6.03
TCGA-B5-A11X-01A	UCEC	6.27
TCGA-D1-A1NU-01A	UCEC	6.36
TCGA-EY-A2OP-01A	UCEC	7.49
TCGA-BS-A0TJ-01A	UCEC	6.43
TCGA-AJ-A3I9-01A	UCEC	6.3
TCGA-B5-A0KB-01B	UCEC	3.12
TCGA-AP-A05D-01A	UCEC	4.8
TCGA-SJ-A6ZJ-01A	UCEC	6.1
TCGA-B5-A1N2-01A	UCEC	6.45
TCGA-BG-A0YV-01A	UCEC	5.82
TCGA-AP-A05H-01A	UCEC	5.05
TCGA-BS-A0TI-01A	UCEC	5.81
TCGA-BG-A18A-01A	UCEC	5.55

TCGA-FI-A2CX-01A	UCEC	6.39
TCGA-BG-A0M8-01A	UCEC	4.48
TCGA-BS-A0TD-01A	UCEC	5.82
TCGA-BS-A0WQ-01A	UCEC	6.64
TCGA-EY-A1GT-01A	UCEC	6.58
TCGA-AJ-A2QO-01A	UCEC	7.03
TCGA-BG-A0MT-01A	UCEC	6.31
TCGA-EY-A1GO-01A	UCEC	6.62
TCGA-FI-A2EW-01A	UCEC	5.78
TCGA-DI-A1NN-01A	UCEC	6.33
TCGA-A5-A0GJ-01A	UCEC	3.64
TCGA-BG-A0YU-01A	UCEC	5.84
TCGA-2E-A9G8-01A	UCEC	5.75
TCGA-AX-A2H7-01A	UCEC	5.63
TCGA-BS-A0UA-01A	UCEC	5.94
TCGA-EY-A1GE-01A	UCEC	6.5
TCGA-A5-A0GN-01A	UCEC	4.9
TCGA-AP-A5FX-01A	UCEC	4.99
TCGA-QS-A744-01A	UCEC	6.18
TCGA-A5-A1OH-01A	UCEC	6.37
TCGA-AJ-A2QL-01A	UCEC	6.6
TCGA-AX-A06D-01A	UCEC	6.36
TCGA-A5-A0GE-01A	UCEC	6.43
TCGA-A5-A0GG-01A	UCEC	5.41
TCGA-AP-A053-01A	UCEC	6.05
TCGA-B5-A11Q-01A	UCEC	5.02
TCGA-BS-A0UM-01A	UCEC	5.96
TCGA-B5-A3FA-01A	UCEC	6.06
TCGA-AP-A1DQ-01A	UCEC	6.56
TCGA-AX-A06J-01A	UCEC	5.78
TCGA-A5-A0GU-01A	UCEC	6.63
TCGA-B5-A5OC-01A	UCEC	6.68
TCGA-BK-A26L-01C	UCEC	6.39
TCGA-AJ-A3NC-01A	UCEC	6.9
TCGA-BK-A13C-01A	UCEC	6.65
TCGA-EO-A3AV-01A	UCEC	6.3
TCGA-EY-A1GS-01A	UCEC	7.35
TCGA-AJ-A23M-01A	UCEC	6.66
TCGA-D1-A101-01A	UCEC	5.67
TCGA-A5-A2K3-01A	UCEC	5.61
TCGA-B5-A0JZ-01A	UCEC	5.66
TCGA-D1-A17Q-01A	UCEC	6.04
TCGA-AX-A05Y-01A	UCEC	4.98
TCGA-B5-A11P-01B	UCEC	5.45
TCGA-AX-A2H5-01A	UCEC	7.23
TCGA-B5-A5OE-01A	UCEC	7.34
TCGA-K6-A3WQ-01A	UCEC	4.91
TCGA-D1-A1NW-01A	UCEC	6.9
TCGA-D1-A1NX-01A	UCEC	6.11
TCGA-AP-A0LI-01A	UCEC	6.71
TCGA-EY-A1GK-01A	UCEC	5.66
TCGA-BG-A0M2-01A	UCEC	6.19
TCGA-A5-A0GM-01A	UCEC	5.02
TCGA-BG-A3EW-01A	UCEC	6.54
TCGA-BK-A6W4-01A	UCEC	6.86
TCGA-D1-A165-01A	UCEC	6.24
TCGA-D1-A163-01A	UCEC	6.49
TCGA-A5-A3LP-01A	UCEC	5.4

TCGA-AX-A3G1-01A	UCEC	5.14
TCGA-AX-A2H4-01A	UCEC	5.79
TCGA-D1-A0ZP-01A	UCEC	6.58
TCGA-AP-A1DP-01A	UCEC	6.25
TCGA-D1-A1O0-01A	UCEC	5.83
TCGA-B5-A0K2-01A	UCEC	6.06
TCGA-AX-A3GB-01A	UCEC	6.18
TCGA-AJ-A3BD-01A	UCEC	5.87
TCGA-AX-A05Z-01A	UCEC	4.97
TCGA-B5-A11Y-01A	UCEC	6.19
TCGA-AP-A0LG-01A	UCEC	6.64
TCGA-B5-A11N-01A	UCEC	5.34
TCGA-D1-A17A-01A	UCEC	5.56
TCGA-BS-A0V7-01A	UCEC	5.2
TCGA-AX-A1C5-01A	UCEC	5.8
TCGA-AX-A060-01A	UCEC	5.79
TCGA-BG-A3PP-01A	UCEC	5.56
TCGA-AX-A3FV-01A	UCEC	6.3
TCGA-EO-A3KU-01A	UCEC	7.38
TCGA-AX-A3GI-01A	UCEC	6.43
TCGA-FI-A3PX-01A	UCEC	6.65
TCGA-B5-A11F-01A	UCEC	6.25
TCGA-EY-A549-01A	UCEC	6.42
TCGA-AX-A1CA-01A	UCEC	5.76
TCGA-B5-A5OD-01A	UCEC	6.67
TCGA-BK-A0CA-01B	UCEC	6.73
TCGA-B5-A0K1-01A	UCEC	5.83
TCGA-AX-A1CE-01A	UCEC	6.92
TCGA-A5-A0G9-01A	UCEC	5.46
TCGA-D1-A2G6-01A	UCEC	6.04
TCGA-A5-A0G1-01A	UCEC	6.6
TCGA-BG-A18C-01A	UCEC	5.33
TCGA-BK-A4ZD-01A	UCEC	6.75
TCGA-AX-A0J0-01A	UCEC	6.36
TCGA-BG-A0M7-01A	UCEC	5.46
TCGA-B5-A0K8-01A	UCEC	6.92
TCGA-BK-A56F-01A	UCEC	6.55
TCGA-EO-A22U-01A	UCEC	6.25
TCGA-AX-A1C9-01A	UCEC	6.31
TCGA-AX-A0IS-01A	UCEC	6.26
TCGA-AX-A2HJ-01A	UCEC	6.14
TCGA-A5-A0GH-01A	UCEC	6.73
TCGA-SL-A6JA-01A	UCEC	6.93
TCGA-EY-A2OM-01A	UCEC	6.49
TCGA-B5-A3S1-01A	UCEC	6.5
TCGA-KP-A3W1-01A	UCEC	6.38
TCGA-EY-A2OO-01A	UCEC	6.6
TCGA-AP-A05N-01A	UCEC	6.41
TCGA-D1-A0ZV-01A	UCEC	5.86
TCGA-B5-A3FB-01A	UCEC	6.2
TCGA-D1-A1NY-01A	UCEC	5.95
TCGA-BK-A6W3-01A	UCEC	6.61
TCGA-AP-A1DR-01A	UCEC	6.14
TCGA-A5-A1OJ-01A	UCEC	5.31
TCGA-B5-A121-01A	UCEC	6.34
TCGA-AJ-A3NG-01A	UCEC	6.72
TCGA-B5-A0JV-01A	UCEC	5.85
TCGA-D1-A17S-01A	UCEC	4.92

TCGA-D1-A16X-01A	UCEC	5.14
TCGA-B5-A0K7-01A	UCEC	5.96
TCGA-A5-A1OF-01A	UCEC	5.47
TCGA-DF-A2KZ-01A	UCEC	6.27
TCGA-D1-A0ZQ-01A	UCEC	5.99
TCGA-JU-AAVI-01A	UCEC	6.11
TCGA-A5-A0R6-01A	UCEC	6.39
TCGA-AP-A05P-01A	UCEC	6.82
TCGA-D1-A167-01A	UCEC	7.44
TCGA-BG-A222-01A	UCEC	6.59
TCGA-AJ-A8CW-01A	UCEC	6.74
TCGA-D1-A162-01A	UCEC	5.96
TCGA-FI-A2F4-01A	UCEC	6.28
TCGA-AJ-A3BI-01A	UCEC	5.51
TCGA-D1-A16J-01A	UCEC	5.22
TCGA-AP-A0LT-01A	UCEC	6.47
TCGA-A5-A0GW-01A	UCEC	6.18
TCGA-A5-A0G2-01A	UCEC	7.5
TCGA-AJ-A3BK-01A	UCEC	5.77
TCGA-PG-A7D5-01A	UCEC	6.32
TCGA-QS-A8F1-01A	UCEC	6.36
TCGA-A5-A0GX-01A	UCEC	4.49
TCGA-AX-A062-01A	UCEC	6.04
TCGA-D1-A17K-01A	UCEC	5.01
TCGA-BG-A220-01A	UCEC	6.6
TCGA-AX-A05S-01A	UCEC	5.65
TCGA-EY-A2OQ-01A	UCEC	7.33
TCGA-AP-A0LM-01A	UCEC	6.07
TCGA-AJ-A3BF-01A	UCEC	6.11
TCGA-A5-A0G5-01A	UCEC	5.31
TCGA-EY-A215-01A	UCEC	6.4
TCGA-FI-A2D4-01A	UCEC	6.11
TCGA-E6-A1LX-01A	UCEC	6.08
TCGA-AX-A2HH-01A	UCEC	6.44
TCGA-BG-A0M9-01A	UCEC	5.61
TCGA-EO-A22R-01A	UCEC	6.99
TCGA-BS-A0UL-01A	UCEC	5.97
TCGA-AP-A1DO-01A	UCEC	6.71
TCGA-B5-A0K0-01A	UCEC	6.2
TCGA-BS-A0VI-01A	UCEC	5.1
TCGA-EY-A547-01A	UCEC	5.98
TCGA-FI-A2EX-01A	UCEC	7.63
TCGA-BS-A0V8-01A	UCEC	5.8
TCGA-EO-A3AZ-01A	UCEC	6.13
TCGA-EO-A3B0-01A	UCEC	6.85
TCGA-D1-A0ZZ-01A	UCEC	6.2
TCGA-DI-A1BU-01A	UCEC	7.03
TCGA-AX-A2HC-01A	UCEC	6.92
TCGA-A5-A1OG-01A	UCEC	5.59
TCGA-QS-A5YR-01A	UCEC	5.52
TCGA-EO-A1Y5-01A	UCEC	6
TCGA-EO-A3AS-01A	UCEC	5.3
TCGA-AJ-A3EL-01A	UCEC	6.02
TCGA-EO-A3B1-01A	UCEC	7.37
TCGA-DF-A2KR-01A	UCEC	6.01
TCGA-D1-A2G7-01A	UCEC	6.32
TCGA-EY-A1GD-01A	UCEC	6.18
TCGA-A5-A3LO-01A	UCEC	6.54

TCGA-BS-A0TG-01A	UCEC	5.75
TCGA-A5-A0R8-01A	UCEC	5.5
TCGA-B5-A0JS-01A	UCEC	6.02
TCGA-A5-A0GD-01A	UCEC	5.83
TCGA-BG-A187-01A	UCEC	5.85
TCGA-KJ-A3U4-01A	UCEC	5.72
TCGA-BG-A0MK-01A	UCEC	6.42
TCGA-BS-A0U9-01B	UCEC	4.95
TCGA-BG-A0M4-01A	UCEC	5.41
TCGA-BK-A0CB-01A	UCEC	5.42
TCGA-D1-A103-01A	UCEC	6.21
TCGA-AJ-A3EJ-01A	UCEC	6.34
TCGA-FI-A2EY-01A	UCEC	5.14
TCGA-D1-A3DH-01A	UCEC	5.69
TCGA-FI-A2D6-01A	UCEC	6.57
TCGA-B5-A0K3-01A	UCEC	7.03
TCGA-AX-A2HF-01A	UCEC	6.15
TCGA-EY-A1G7-01A	UCEC	6.63
TCGA-D1-A16Q-01A	UCEC	4.75
TCGA-BK-A139-02A	UCEC	4.88
TCGA-AJ-A3OL-01A	UCEC	6.54
TCGA-D1-A0ZN-01A	UCEC	6.54
TCGA-EY-A3QX-01A	UCEC	6.84
TCGA-AX-A06B-01A	UCEC	5.88
TCGA-BK-A139-01C	UCEC	6.2
TCGA-AX-A2HA-01A	UCEC	5.46
TCGA-EO-A3AY-01A	UCEC	5.68
TCGA-B5-A11U-01A	UCEC	6.18
TCGA-D1-A16Y-01A	UCEC	5.15
TCGA-E6-A2P9-01A	UCEC	7.07
TCGA-D1-A15X-01A	UCEC	6.35
TCGA-B5-A11L-01B	UCEC	6.61
TCGA-AP-A0LD-01A	UCEC	5.61
TCGA-AJ-A3NE-01A	UCEC	6.75
TCGA-AP-A1DV-01A	UCEC	6.8
TCGA-B5-A1MY-01A	UCEC	6.16
TCGA-D1-A17R-01A	UCEC	5.65
TCGA-DF-A2KV-01A	UCEC	5.76
TCGA-FI-A2F8-01A	UCEC	5.21
TCGA-A5-A0GV-01A	UCEC	5.56
TCGA-EY-A1GP-01A	UCEC	6.3
TCGA-FI-A3PV-01A	UCEC	6.38
TCGA-DF-A2L0-01A	UCEC	4.76
TCGA-AP-A0LN-01A	UCEC	6.02
TCGA-AX-A1C7-01A	UCEC	5.93
TCGA-AP-A0L9-01A	UCEC	5.73
TCGA-BG-A0LW-01A	UCEC	5.93
TCGA-D1-A17D-01A	UCEC	5.11
TCGA-DI-A2QY-01A	UCEC	5.8
TCGA-AX-A0IU-01A	UCEC	7.44
TCGA-E6-A1LZ-01A	UCEC	6.24
TCGA-BK-A139-01A	UCEC	6.99
TCGA-BG-A0MC-01A	UCEC	4.98
TCGA-EY-A1GU-01A	UCEC	6.25
TCGA-BG-A18B-01A	UCEC	5.86
TCGA-AJ-A3OJ-01A	UCEC	6.38
TCGA-BS-A0TC-01A	UCEC	6.39
TCGA-AJ-A3IA-01A	UCEC	5.65

TCGA-D1-A16V-01A	UCEC	6.24
TCGA-AX-A3FT-01A	UCEC	6.88
TCGA-BG-A0VX-01A	UCEC	5.91
TCGA-BG-A0M0-01A	UCEC	5.93
TCGA-EO-A3KX-01A	UCEC	6.23
TCGA-AX-A2IN-01A	UCEC	6.38
TCGA-AJ-A2QM-01A	UCEC	5.06
TCGA-AX-A2H8-01A	UCEC	6.7
TCGA-AJ-A2QN-01A	UCEC	6.31
TCGA-BS-A0UJ-01A	UCEC	4.7
TCGA-D1-A3DA-01A	UCEC	5.97
TCGA-FI-A2D5-01A	UCEC	6.42
TCGA-AP-A0LE-01A	UCEC	5.41
TCGA-EO-A1Y8-01A	UCEC	5.58
TCGA-AJ-A2QK-01A	UCEC	6.39
TCGA-A5-A7WK-01A	UCEC	5.42
TCGA-B5-A11H-01A	UCEC	5.99
TCGA-BG-A0W2-01A	UCEC	6.11
TCGA-AX-A05U-01A	UCEC	6.88
TCGA-AX-A3G9-01A	UCEC	6.88
TCGA-AX-A06H-01A	UCEC	6.39
TCGA-BS-A0UF-01A	UCEC	5.55
TCGA-EO-A22Y-01A	UCEC	6.36
TCGA-B5-A11M-01A	UCEC	6.36
TCGA-BG-A186-01A	UCEC	5.37
TCGA-BG-A0VW-01A	UCEC	4.94
TCGA-EY-A1GF-01A	UCEC	6.93
TCGA-EO-A22T-01A	UCEC	6.79
TCGA-AX-A064-01A	UCEC	5.53
TCGA-D1-A17L-01A	UCEC	4.86
TCGA-AX-A3G4-01A	UCEC	5.64
TCGA-KP-A3VZ-01A	UCEC	6.8
TCGA-5S-A9Q8-01A	UCEC	5.41
TCGA-BG-A0M3-01A	UCEC	5.52
TCGA-EY-A54A-01A	UCEC	7.08
TCGA-BG-A0VV-01A	UCEC	5.72
TCGA-B5-A1MZ-01A	UCEC	6.28
TCGA-D1-A17U-01A	UCEC	6.24
TCGA-AJ-A3QS-01A	UCEC	6.94
TCGA-A5-A2K2-01A	UCEC	7.04
TCGA-AJ-A8CT-01A	UCEC	5.83
TCGA-AP-A1E0-01A	UCEC	6.77
TCGA-D1-A0ZS-01A	UCEC	6.59
TCGA-EO-A3AU-01A	UCEC	7.12
TCGA-AX-A05W-01A	UCEC	5.25
TCGA-FI-A2F9-01A	UCEC	6.37
TCGA-BG-A0W1-01A	UCEC	5.39
TCGA-A5-A0VP-01A	UCEC	6.33
TCGA-B5-A3F9-01A	UCEC	6.48
TCGA-D1-A1O8-01A	UCEC	4.99
TCGA-BS-A0U7-01A	UCEC	6.18
TCGA-PG-A5BC-01A	UCEC	6.83
TCGA-A5-A0GB-01A	UCEC	6.74
TCGA-A5-A1OK-01A	UCEC	6.28
TCGA-B5-A1MX-01A	UCEC	6.11
TCGA-D1-A179-01A	UCEC	6.24
TCGA-AX-A3FW-01A	UCEC	6.12
TCGA-B5-A11E-01A	UCEC	6.28

TCGA-AP-A1E1-01A	UCEC	6.27
TCGA-A5-A0R7-01A	UCEC	5.42
TCGA-D1-A17M-01A	UCEC	6.25
TCGA-D1-A161-01A	UCEC	6.46
TCGA-QS-A5YQ-01A	UCEC	5.54
TCGA-D1-A3JQ-01A	UCEC	7.06
TCGA-AP-A1DH-01A	UCEC	5.63
TCGA-A5-A0GP-01A	UCEC	5.9
TCGA-A5-A0VO-01A	UCEC	6.33
TCGA-EY-A3L3-01A	UCEC	6.98
TCGA-B5-A11V-01A	UCEC	6.34
TCGA-AJ-A5DV-01A	UCEC	6.05
TCGA-AJ-A3TW-01A	UCEC	6.72
TCGA-DF-A2KN-01A	UCEC	6.51
TCGA-AJ-A3EM-01A	UCEC	6.01
TCGA-AX-A1CN-01A	UCEC	6.05
TCGA-AP-A0LO-01A	UCEC	5.93
TCGA-D1-A16N-01A	UCEC	4.56
TCGA-QF-A5YS-01A	UCEC	6.17
TCGA-D1-A17F-01A	UCEC	5.46
TCGA-EY-A1GM-01A	UCEC	5.49
TCGA-BG-A0MQ-01A	UCEC	6.54
TCGA-AJ-A3OK-01A	UCEC	5.77
TCGA-EY-A1GI-01A	UCEC	6.79
TCGA-FI-A2D0-01A	UCEC	6.59
TCGA-B5-A3FH-01A	UCEC	6.17
TCGA-BG-A2L7-01A	UCEC	6.4
TCGA-A5-A0VQ-01A	UCEC	6.18
TCGA-D1-A16S-01A	UCEC	5.86
TCGA-AX-A0IZ-01A	UCEC	5.81
TCGA-EY-A5W2-01A	UCEC	6.22
TCGA-AP-A3K1-01A	UCEC	6.5
TCGA-A5-A0R9-01A	UCEC	6.05
TCGA-EY-A212-01A	UCEC	6.59
TCGA-AX-A1CR-01A	UCEC	5.05
TCGA-A5-A0GI-01A	UCEC	5.34
TCGA-N6-A4VF-01A	UCS	6.12
TCGA-N9-A4PZ-01A	UCS	5.63
TCGA-N9-A4Q8-01A	UCS	6.68
TCGA-N8-A4PQ-01A	UCS	7.24
TCGA-N5-A4RO-01A	UCS	6.74
TCGA-QM-A5NM-01A	UCS	6.27
TCGA-N5-A4RJ-01A	UCS	6.51
TCGA-NA-A4QW-01A	UCS	6.82
TCGA-N5-A4RS-01A	UCS	6.06
TCGA-NF-A4WX-01A	UCS	6.19
TCGA-NG-A4VU-01A	UCS	7.34
TCGA-NA-A4QX-01A	UCS	6.84
TCGA-ND-A4WF-01A	UCS	6.77
TCGA-N6-A4V9-01A	UCS	6.31
TCGA-ND-A4WC-01A	UCS	6.46
TCGA-ND-A4WA-01A	UCS	6.97
TCGA-N5-A4RF-01A	UCS	6.33
TCGA-N9-A4Q4-01A	UCS	6.6
TCGA-N6-A4VE-01A	UCS	6
TCGA-N8-A56S-01A	UCS	6.71
TCGA-NA-A5I1-01A	UCS	6.72
TCGA-N5-A4RU-01A	UCS	6.71

TCGA-N5-A4RV-01A	UCS	6.87
TCGA-N5-A4RA-01A	UCS	5.76
TCGA-N5-A4RM-01A	UCS	6.46
TCGA-N8-A4PM-01A	UCS	5.71
TCGA-N6-A4VC-01A	UCS	6.51
TCGA-N5-A59F-01A	UCS	6.83
TCGA-N7-A4Y5-01A	UCS	6.71
TCGA-N5-A4RT-01A	UCS	6.69
TCGA-N7-A59B-01A	UCS	5.79
TCGA-N8-A4PO-01A	UCS	6.66
TCGA-N5-A4RN-01A	UCS	6.6
TCGA-NF-A4X2-01A	UCS	6.77
TCGA-N7-A4Y8-01A	UCS	5.94
TCGA-N8-A4PL-01A	UCS	7.24
TCGA-N9-A4Q1-01A	UCS	6.39
TCGA-N5-A4RD-01A	UCS	5.71
TCGA-N5-A4R8-01A	UCS	7.5
TCGA-N8-A4PN-01A	UCS	6.85
TCGA-QN-A5NN-01A	UCS	6.69
TCGA-NA-A4R1-01A	UCS	6.67
TCGA-N9-A4Q7-01A	UCS	6.13
TCGA-NF-A5CP-01A	UCS	6.8
TCGA-N8-A4PI-01A	UCS	6.28
TCGA-NA-A4R0-01A	UCS	6.23
TCGA-N5-A59E-01A	UCS	6.75
TCGA-NG-A4VW-01A	UCS	6.98
TCGA-N6-A4VG-01A	UCS	6.68
TCGA-NA-A4QV-01A	UCS	6.35
TCGA-N6-A4VD-01A	UCS	6.61
TCGA-ND-A4W6-01A	UCS	6.3
TCGA-N8-A4PP-01A	UCS	6.74
TCGA-NF-A4WU-01A	UCS	5.91
TCGA-N9-A4Q3-01A	UCS	6.27
TCGA-N7-A4Y0-01A	UCS	7.65
TCGA-NA-A4QY-01A	UCS	6.7
TCGA-VD-A8KJ-01A	UVM	6.43
TCGA-WC-A87W-01A	UVM	7.33
TCGA-V4-A9EW-01A	UVM	5.91
TCGA-V4-A9F8-01A	UVM	5.09
TCGA-YZ-A984-01A	UVM	4.75
TCGA-WC-A888-01A	UVM	6.82
TCGA-VD-A8KH-01A	UVM	6.32
TCGA-V4-A9EZ-01A	UVM	3.5
TCGA-V4-A9F2-01A	UVM	5.96
TCGA-V4-A9F4-01A	UVM	6.54
TCGA-WC-A881-01A	UVM	5.6
TCGA-WC-A87U-01A	UVM	6.4
TCGA-WC-A88A-01A	UVM	6.23
TCGA-V4-A9ET-01A	UVM	5.07
TCGA-V3-A9ZX-01A	UVM	6.66
TCGA-V4-A9E9-01A	UVM	4
TCGA-V4-A9EK-01A	UVM	6.59
TCGA-WC-AA9E-01A	UVM	6.18
TCGA-VD-A8KF-01A	UVM	6.63
TCGA-V4-A9EM-01A	UVM	5.73
TCGA-VD-A8KD-01A	UVM	6.88
TCGA-V4-A9EH-01A	UVM	5.9
TCGA-V4-A9F7-01A	UVM	6.43

TCGA-V4-A9E5-01A	UVM	4.7
TCGA-V4-A9EY-01A	UVM	3.15
TCGA-VD-A8KM-01A	UVM	6.08
TCGA-VD-A8KO-01A	UVM	6.16
TCGA-VD-AA8N-01A	UVM	4.89
TCGA-VD-A8KB-01A	UVM	5.3
TCGA-V4-A9ED-01A	UVM	5.46
TCGA-WC-A87Y-01A	UVM	7.15
TCGA-V3-A9ZY-01A	UVM	4.96
TCGA-VD-A8KI-01A	UVM	5.27
TCGA-RZ-AB0B-01A	UVM	6.93
TCGA-WC-A87T-01A	UVM	6.21
TCGA-VD-AA8Q-01A	UVM	4.42
TCGA-V4-A9F0-01A	UVM	6.37
TCGA-VD-AA8M-01A	UVM	5.24
TCGA-WC-A884-01A	UVM	5.53
TCGA-V4-A9EX-01A	UVM	7.2
TCGA-V4-A9EL-01A	UVM	6.7
TCGA-WC-A882-01A	UVM	4.2
TCGA-VD-AA8S-01B	UVM	5.2
TCGA-VD-AA8O-01A	UVM	5.93
TCGA-V4-A9E7-01A	UVM	5.9
TCGA-V4-A9ES-01A	UVM	6.54
TCGA-YZ-A983-01A	UVM	5.75
TCGA-V4-A9F1-01A	UVM	7.57
TCGA-WC-A883-01A	UVM	6.18
TCGA-VD-A8KL-01A	UVM	5.03
TCGA-VD-A8KN-01A	UVM	6.31
TCGA-V4-A9EO-01A	UVM	6.48
TCGA-WC-A880-01A	UVM	5.7
TCGA-V4-A9EV-01A	UVM	7.07
TCGA-V4-A9E8-01A	UVM	4.94
TCGA-V4-A9EE-01A	UVM	7.07
TCGA-WC-A885-01A	UVM	4.54
TCGA-VD-AA8T-01A	UVM	6.78
TCGA-VD-A8K8-01A	UVM	6.79
TCGA-V4-A9EF-01A	UVM	6.41
TCGA-V4-A9EA-01A	UVM	3.31
TCGA-VD-A8KA-01B	UVM	5.76
TCGA-VD-AA8P-01A	UVM	6.38
TCGA-YZ-A980-01A	UVM	6.82
TCGA-VD-A8K9-01A	UVM	5.9
TCGA-VD-A8KG-01A	UVM	5.54
TCGA-V4-A9EQ-01A	UVM	5.01
TCGA-V4-A9F5-01A	UVM	5.41
TCGA-YZ-A982-01A	UVM	6.4
TCGA-V4-A9EJ-01A	UVM	5.45
TCGA-V4-A9EU-01A	UVM	6.92
TCGA-YZ-A985-01A	UVM	4.81
TCGA-VD-A8KK-01A	UVM	5.29
TCGA-VD-AA8R-01A	UVM	6.23
TCGA-V4-A9EI-01A	UVM	7.39
TCGA-VD-A8KE-01A	UVM	5.18
TCGA-V4-A9F3-01A	UVM	6.77
TCGA-V4-A9EC-01A	UVM	4.69
TCGA-VD-A8K7-01B	UVM	5.8
TCGA-WC-AA9A-01A	UVM	6.34

Fig.5 A

Gene id	Gene name	Gene desc	FC(A02/A0	Log2FC(A(	Pvalue	Padjust	Significant	Regulate
ENSG0000	SOX8	SRY-box tr	0.205648	-2.281753	0.000117	0.002708	yes	down
ENSG0000	STAB1	stabilin 1 [S	0.191938	-2.381289	0.001536	0.025433	yes	down
ENSG0000	FYN	FYN proto-	2.303254	1.203673	2.00E-10	1.38E-08	yes	up
ENSG0000	NME1-NME	NME1-NME	0.366667	-1.447458	2.49E-24	5.11E-22	yes	down
ENSG0000	SNAI2	snail family	3.454881	1.788636	0.000529	0.010128	yes	up
ENSG0000	ADRB1	adrenocept	0.460651	-1.118255	0.00121	0.020743	yes	down
ENSG0000	EPHA3	EPH recept	0.460651	-1.118255	5.73E-05	0.001442	yes	down
ENSG0000	MAGEC2	MAGE fami	3.646818	1.866638	0.002761	0.041636	yes	up
ENSG0000	ARAP2	ArfGAP witl	2.188091	1.129673	4.18E-06	0.000139	yes	up
ENSG0000	KITLG	KIT ligand [	2.169084	1.117086	3.04E-20	4.84E-18	yes	up
ENSG0000	MCF2L2	MCF.2 cell	2.55783	1.354921	0.00039	0.007762	yes	up
ENSG0000	NRIP2	nuclear rec	15.56253	3.960004	8.65E-05	0.002076	yes	up
ENSG0000	FOXC1	forkhead bc	2.988005	1.579183	2.19E-09	1.31E-07	yes	up
ENSG0000	LAMC2	laminin sub	8.061388	3.011028	0.000676	0.012497	yes	up
ENSG0000	RASGRF1	Ras protein	0.411295	-1.281753	0.000274	0.005702	yes	down
ENSG0000	AHRR	aryl hydroca	3.042683	1.605344	6.84E-06	0.000219	yes	up
ENSG0000	FGFR2	fibroblast gı	2.112985	1.079283	3.83E-11	2.91E-09	yes	up
ENSG0000	TRIB2	tribbles pse	0.36754	-1.444025	2.54E-10	1.72E-08	yes	down
ENSG0000	MOV10L1	Mov10 like	4.390578	2.134411	4.78E-09	2.75E-07	yes	up
ENSG0000	ALPK1	alpha kinas	2.438739	1.286136	6.74E-19	9.81E-17	yes	up
ENSG0000	ATP2A3	ATPase sa	0.287907	-1.796327	0.00226	0.035217	yes	down
ENSG0000	PLD1	phospholip	0.297194	-1.750523	0.000652	0.012087	yes	down
ENSG0000	LAMP3	lysosomal a	3.222442	1.688154	4.74E-29	1.28E-26	yes	up
ENSG0000	PPIE	peptidylprol	0.455543	-1.13434	3.28E-61	2.33E-58	yes	down
ENSG0000	COL16A1	collagen tyr	2.409763	1.268891	4.10E-44	1.88E-41	yes	up
ENSG0000	ABCB1	ATP binding	10.36464	3.373598	6.81E-07	2.67E-05	yes	up
ENSG0000	AKR1B1	aldo-keto re	5.37202	2.425465	0.001197	0.020554	yes	up
ENSG0000	PPP1R15A	protein pho	2.178379	1.123255	1.96E-24	4.10E-22	yes	up
ENSG0000	OAS1	2'-5'-oligoa	2.252454	1.171497	0.00086	0.015457	yes	up
ENSG0000	CAPN3	calpain 3 [S	2.538678	1.344077	1.55E-05	0.000454	yes	up
ENSG0000	TGFB2	transformin	2.014804	1.010639	0.000204	0.0044	yes	up
ENSG0000	ADA2	adenosine	2.025902	1.018564	1.43E-10	1.00E-08	yes	up
ENSG0000	PTGS1	prostagland	0.457264	-1.128902	0.000287	0.005947	yes	down
ENSG0000	YPEL1	yippee like	2.259455	1.175975	0.002421	0.037346	yes	up
ENSG0000	LGALS1	galectin 1 [	0.36754	-1.444025	0.000261	0.005465	yes	down
ENSG0000	DHRS2	dehydroger	0.435059	-1.200717	0.001942	0.030973	yes	down
ENSG0000	LAMA1	laminin sub	2.015347	1.011028	0.002531	0.038679	yes	up
ENSG0000	FA2H	fatty acid 2-	0.393238	-1.346524	0.001219	0.020881	yes	down
ENSG0000	TUBB4A	tubulin beta	0.131615	-2.92561	7.87E-07	3.03E-05	yes	down
ENSG0000	TNNT1	troponin T1	0.347661	-1.524247	5.43E-05	0.001372	yes	down
ENSG0000	TMEM59L	transmemb	0.338714	-1.561861	0.000997	0.017574	yes	down
ENSG0000	ITGB8	integrin sub	12.89822	3.6891	0.000927	0.016453	yes	up
ENSG0000	LFNG	LFNG O-fu	0.429712	-1.21856	0.00013	0.002965	yes	down
ENSG0000	AGR2	anterior gra	2.303254	1.203673	5.30E-06	0.000173	yes	up
ENSG0000	APBA1	amyloid bet	0.454163	-1.138719	0.00019	0.004142	yes	down
ENSG0000	SH3PXD2A	SH3 and P)	2.111316	1.078142	0.002561	0.039021	yes	up
ENSG0000	SEPTIN4	septin 4 [Sc	0.394844	-1.340647	0.002898	0.043407	yes	down
ENSG0000	TRIM16L	tripartite mc	0.347463	-1.525071	4.84E-05	0.001246	yes	down
ENSG0000	ABCC3	ATP binding	4.030694	2.011028	1.97E-05	0.000562	yes	up
ENSG0000	MAP2K6	mitogen-ac	0.40349	-1.309396	1.21E-23	2.36E-21	yes	down
ENSG0000	UGT2B10	UDP glucur	0.418575	-1.256443	4.39E-28	1.11E-25	yes	down
ENSG0000	ASIC1	acid sensin	0.475318	-1.073036	3.59E-18	4.97E-16	yes	down
ENSG0000	PDE10A	phosphodie	2.263237	1.178388	4.97E-05	0.001277	yes	up
ENSG0000	VEGFA	vascular en	2.080688	1.05706	3.96E-135	9.27E-132	yes	up
ENSG0000	BCHE	butyrylcholi	0.18426	-2.440183	8.54E-08	3.93E-06	yes	down
ENSG0000	CLIP4	CAP-Gly dc	3.725852	1.89757	2.61E-07	1.11E-05	yes	up

ENSG0000	ACADL	acyl-CoA de	2.345907	1.230146	0.000179	0.003915	yes	up
ENSG0000	DLEU2L	deleted in ly	3.469265	1.79463	1.87E-05	0.000536	yes	up
ENSG0000	SLC35D1	solute carri	3.358912	1.747994	0.000103	0.002423	yes	up
ENSG0000	HPCAL4	hippocalcin	0.463154	-1.110435	3.15E-05	0.000856	yes	down
ENSG0000	PADI2	peptidyl arg	0.487977	-1.035113	0.001644	0.026904	yes	down
ENSG0000	GBP3	guanylate b	3.166974	1.663105	0.002952	0.044083	yes	up
ENSG0000	GBP1	guanylate b	5.758134	2.525601	5.63E-06	0.000183	yes	up
ENSG0000	CCN2	cellular com	9.404953	3.233421	5.01E-11	3.73E-09	yes	up
ENSG0000	PLEKHG1	pleckstrin h	2.438739	1.286136	9.01E-06	0.00028	yes	up
ENSG0000	LHX4	LIM homeo	2.259108	1.175753	9.63E-06	0.000296	yes	up
ENSG0000	CCR2	C-C motif c	5.326274	2.413127	9.02E-07	3.43E-05	yes	up
ENSG0000	TAF1L	TATA-box b	2.375462	1.248208	4.24E-05	0.001108	yes	up
ENSG0000	PREX1	phosphatidy	0.395872	-1.336895	5.85E-05	0.00147	yes	down
ENSG0000	FOSB	FosB proto-	3.14778	1.654335	5.28E-05	0.001341	yes	up
ENSG0000	CHURC1-F	CHURC1-F	0.16277	-2.61909	0.000246	0.005193	yes	down
ENSG0000	CTAG2	cancer/testi	0.448028	-1.158339	0.000557	0.010564	yes	down
ENSG0000	EDN2	endothelin 2	0.472773	-1.08078	3.50E-05	0.000937	yes	down
ENSG0000	BCL11B	BCL11 tran	2.303254	1.203673	1.52E-09	9.34E-08	yes	up
ENSG0000	KLHDC7B	kelch doma	2.373049	1.246742	2.54E-05	0.000708	yes	up
ENSG0000	HSD17B3	hydroxyster	11.87327	3.569646	0.001695	0.027617	yes	up
ENSG0000	GJA9	gap junctior	20.26863	4.341177	0.002032	0.032147	yes	up
ENSG0000	KREMEN2	kringle cont	0.352099	-1.505946	5.38E-05	0.001364	yes	down
ENSG0000	SLX1A	SLX1 homo	0.438459	-1.189486	1.52E-10	1.07E-08	yes	down
ENSG0000	CARD6	caspase re	3.556495	1.830456	3.83E-12	3.29E-10	yes	up
ENSG0000	NES	nestin [Sou	0.379016	-1.399668	3.76E-06	0.000126	yes	down
ENSG0000	DMGDH	dimethylgly	2.177295	1.122537	2.37E-07	1.01E-05	yes	up
ENSG0000	PDZD2	PDZ domai	0.445167	-1.167582	8.29E-07	3.17E-05	yes	down
ENSG0000	LRRCC1	leucine rich	3.310927	1.727235	1.01E-05	0.000309	yes	up
ENSG0000	HMGCS2	3-hydroxy-3	2.069231	1.049094	1.75E-170	5.54E-167	yes	up
ENSG0000	LMO2	LIM domair	2.111316	1.078142	5.69E-07	2.26E-05	yes	up
ENSG0000	NIBAN1	niban apopt	3.184511	1.671072	1.15E-193	4.85E-190	yes	up
ENSG0000	DOCK10	dedicator of	0.101614	-3.298827	2.68E-07	1.13E-05	yes	down
ENSG0000	RCBTB2	RCC1 and	2.204543	1.14048	9.83E-05	0.002329	yes	up
ENSG0000	KLF4	KLF transcr	2.212817	1.145884	2.28E-11	1.78E-09	yes	up
ENSG0000	KIF12	kinesin fam	0.361295	-1.468752	0.000117	0.002703	yes	down
ENSG0000	BRDT	bromodoma	0.383504	-1.382685	6.58E-07	2.58E-05	yes	down
ENSG0000	IFI44	interferon ir	2.334379	1.223039	1.32E-05	0.000394	yes	up
ENSG0000	DUSP5	dual specifi	2.657601	1.410124	1.37E-05	0.000406	yes	up
ENSG0000	MYPN	myopalladir	5.758134	2.525601	0.001327	0.022497	yes	up
ENSG0000	IDH1	isocitrate de	0.103205	-3.276412	0	0	yes	down
ENSG0000	PDE5A	phosphodie	2.129058	1.090215	0.002339	0.036239	yes	up
ENSG0000	EGF	epidermal g	2.259172	1.175794	5.49E-23	1.02E-20	yes	up
ENSG0000	CCDC65	coiled-coil c	0.060612	-4.044254	3.42E-05	0.000918	yes	down
ENSG0000	MORN3	MORN repe	2.367233	1.243202	0.00194	0.030956	yes	up
ENSG0000	CDH24	cadherin 24	0.444328	-1.170301	3.38E-07	1.40E-05	yes	down
ENSG0000	RTN1	reticulon 1 [	2.217948	1.149226	0.000556	0.010556	yes	up
ENSG0000	CELF6	CUGBP Ela	0.345281	-1.534156	0.003282	0.04818	yes	down
ENSG0000	SH3GL3	SH3 domai	2.040025	1.028587	0.000571	0.010794	yes	up
ENSG0000	IGLON5	IgLON fami	4.376182	2.129673	0.001103	0.019191	yes	up
ENSG0000	PADI1	peptidyl arg	7.485575	2.904113	0.00129	0.021986	yes	up
ENSG0000	CCN1	cellular com	2.385147	1.254078	1.67E-28	4.38E-26	yes	up
ENSG0000	SUSD4	sushi doma	0.335891	-1.573934	8.40E-05	0.002019	yes	down
ENSG0000	GALNT13	polypeptide	0.360509	-1.471892	2.53E-16	3.12E-14	yes	down
ENSG0000	FAM171B	family with s	3.224555	1.6891	0.000697	0.01284	yes	up
ENSG0000	ALDH1L1	aldehyde de	2.193575	1.133284	2.29E-05	0.000642	yes	up
ENSG0000	ATP10D	ATPase ph	2.663137	1.413127	0.000625	0.011646	yes	up
ENSG0000	GC	GC vitamin	0.489253	-1.031347	4.32E-07	1.77E-05	yes	down
ENSG0000	DDIT4L	DNA damag	0.497503	-1.007223	7.46E-06	0.000236	yes	down

ENSG0000	SLC25A48	solute carri	0.265265	-1.914496	0.000333	0.006756	yes	down
ENSG0000	TENM2	teneurin tra	0.209387	-2.255758	3.29E-05	0.00089	yes	down
ENSG0000	SLC22A3	solute carri	0.212142	-2.236899	9.28E-06	0.000288	yes	down
ENSG0000	CREB5	cAMP resp	2.550031	1.350515	0.002486	0.038139	yes	up
ENSG0000	DOCK11	dedicator o	2.303254	1.203673	7.08E-05	0.001741	yes	up
ENSG0000	CDKN2B	cyclin depe	2.591161	1.373598	1.78E-07	7.70E-06	yes	up
ENSG0000	ANKRD1	ankyrin rep	5.182321	2.373598	8.10E-08	3.73E-06	yes	up
ENSG0000	C20orf144	chromosom	3.339718	1.739726	0.000426	0.008394	yes	up
ENSG0000	KLHL1	kelch like fa	0.444001	-1.171366	3.68E-05	0.000978	yes	down
ENSG0000	PRSS23	serine prote	2.243649	1.165847	1.11E-36	4.02E-34	yes	up
ENSG0000	SLC7A11	solute carri	2.759163	1.464231	0	0	yes	up
ENSG0000	MAT1A	methionine	2.245672	1.167148	1.86E-05	0.000536	yes	up
ENSG0000	AKR1C2	aldo-keto re	3.173438	1.666046	2.24E-24	4.62E-22	yes	up
ENSG0000	OLAH	oleoyl-ACP	4.552092	2.18653	1.26E-12	1.16E-10	yes	up
ENSG0000	DNAI4	dynein axor	2.80997	1.490555	4.85E-06	0.000159	yes	up
ENSG0000	JAKMIP1	janus kinas	0.499462	-1.001554	7.56E-12	6.24E-10	yes	down
ENSG0000	ADGRF4	adhesion G	11.13239	3.476692	1.88E-07	8.12E-06	yes	up
ENSG0000	AK5	adenylate k	0.437943	-1.191186	0.00011	0.002561	yes	down
ENSG0000	NRGN	neurogranir	0.334343	-1.580598	0.001524	0.025267	yes	down
ENSG0000	LY96	lymphocyte	2.751109	1.460013	0.000157	0.003499	yes	up
ENSG0000	NCAM2	neural cell a	0.397659	-1.330397	2.78E-73	2.54E-70	yes	down
ENSG0000	PPM1J	protein pho	0.487227	-1.037335	0.000288	0.005954	yes	down
ENSG0000	HKDC1	hexokinase	5.470228	2.451601	0.000382	0.007625	yes	up
ENSG0000	CFAP107	cilia and fla	3.685206	1.881745	9.40E-05	0.002236	yes	up
ENSG0000	COLEC12	collectin su	0.438336	-1.189892	6.10E-48	3.04E-45	yes	down
ENSG0000	MPZ	myelin prote	6.909761	2.788636	0.002454	0.037739	yes	up
ENSG0000	CCDC17	coiled-coil c	3.886741	1.958561	0.000229	0.004871	yes	up
ENSG0000	NPR2	natriuretic p	7.600737	2.926139	2.65E-07	1.12E-05	yes	up
ENSG0000	ABCG1	ATP binding	0.477886	-1.065262	2.58E-24	5.25E-22	yes	down
ENSG0000	RNASEK-C	RNASEK-C	0.226372	-2.14323	3.66E-06	0.000123	yes	down
ENSG0000	SLC25A34	solute carri	2.687129	1.426066	0.000808	0.014641	yes	up
ENSG0000	MEGF6	multiple EG	0.406457	-1.298827	5.10E-05	0.001302	yes	down
ENSG0000	GBP2	guanylate b	2.303254	1.203673	3.18E-08	1.59E-06	yes	up
ENSG0000	ATF3	activating tr	2.966312	1.56867	1.04E-131	2.26E-128	yes	up
ENSG0000	KCNJ3	potassium i	0.49367	-1.018381	1.53E-52	8.41E-50	yes	down
ENSG0000	SPATA18	spermatoge	2.015347	1.011028	9.88E-15	1.08E-12	yes	up
ENSG0000	ANKRD23	ankyrin rep	0.357716	-1.483115	0.001838	0.029545	yes	down
ENSG0000	IFI16	interferon g	4.894414	2.291136	0.001243	0.021265	yes	up
ENSG0000	ALB	albumin [So	0.248043	-2.01134	5.92E-08	2.81E-06	yes	down
ENSG0000	NOS3	nitric oxide	2.915073	1.543532	7.05E-22	1.24E-19	yes	up
ENSG0000	GEM	GTP bindin	2.788149	1.479308	2.38E-08	1.21E-06	yes	up
ENSG0000	TMC1	transmemb	2.102971	1.072429	0.003346	0.048936	yes	up
ENSG0000	PKNOX2	PBX/knotte	0.239922	-2.059361	0.000823	0.014887	yes	down
ENSG0000	AK8	adenylate k	0.46508	-1.104449	0.001835	0.029517	yes	down
ENSG0000	SPIC	Spi-C trans	0.219358	-2.188644	0.001148	0.019857	yes	down
ENSG0000	NUDT13	nudix hydro	2.370997	1.245494	0.002538	0.038768	yes	up
ENSG0000	NETO1	neuropilin a	0.386947	-1.369791	4.34E-15	4.86E-13	yes	down
ENSG0000	SERPINB8	serpin famil	2.02628	1.018834	3.08E-07	1.29E-05	yes	up
ENSG0000	ZMAT1	zinc finger r	2.467772	1.303209	0.000391	0.007775	yes	up
ENSG0000	STAT6	signal trans	4.090129	2.032146	3.97E-05	0.001044	yes	up
ENSG0000	MAP1A	microtubule	0.403069	-1.3109	9.61E-06	0.000296	yes	down
ENSG0000	UGT1A6	UDP glucur	3.154399	1.657365	7.92E-08	3.66E-06	yes	up
ENSG0000	FN3K	fructosamir	2.209879	1.143967	5.84E-05	0.001468	yes	up
ENSG0000	TTYH1	tweety fami	2.687129	1.426066	0.002733	0.041295	yes	up
ENSG0000	KRT80	keratin 80 [	2.879067	1.525601	6.93E-11	5.07E-09	yes	up
ENSG0000	-	novel trans	0.027911	-5.162999	5.61E-06	0.000182	yes	down
ENSG0000	BEST1	bestrophin	4.070945	2.025364	3.28E-74	3.10E-71	yes	up
ENSG0000	PXDC1	PX domain	2.418019	1.273826	1.84E-09	1.11E-07	yes	up

ENSG0000	SH3TC2	SH3 domai	3.166974	1.663105	0.002952	0.044083	yes	up
ENSG0000	STK32A	serine/threo	2.303254	1.203673	0.000119	0.00275	yes	up
ENSG0000	MUC15	mucin 15, c	19.57766	4.291136	1.90E-05	0.000546	yes	up
ENSG0000	USP50	ubiquitin sp	4.036433	2.013081	0.000911	0.016219	yes	up
ENSG0000	FOS	Fos proto-o	15.62922	3.966174	4.89E-23	9.09E-21	yes	up
ENSG0000	KRT75	keratin 75 [	2.591161	1.373598	3.83E-14	3.99E-12	yes	up
ENSG0000	PKIA	cAMP-depe	0.287907	-1.796327	0.000422	0.008311	yes	down
ENSG0000	FUT3	fucosyltrans	5.134642	2.360264	0.000259	0.00544	yes	up
ENSG0000	APLN	apelin [Sou	0.450637	-1.149964	1.70E-06	6.09E-05	yes	down
ENSG0000	COL24A1	collagen typ	2.037494	1.026796	0.003044	0.045201	yes	up
ENSG0000	PTGER4	prostagland	2.116503	1.081683	0.00017	0.003753	yes	up
ENSG0000	FRMD5	FERM dom	0.490857	-1.026624	0.001476	0.024571	yes	down
ENSG0000	ZNF554	zinc finger p	2.490506	1.316439	1.19E-10	8.47E-09	yes	up
ENSG0000	SNTB1	syntrophin b	11.51627	3.525601	0.002093	0.032985	yes	up
ENSG0000	ISG20	interferon s	2.272948	1.184565	2.16E-05	0.00061	yes	up
ENSG0000	RCAN2	regulator of	2.344383	1.229208	0.000137	0.003111	yes	up
ENSG0000	GTPBP2	GTP bindin	2.046837	1.033396	1.48E-116	2.75E-113	yes	up
ENSG0000	HSPA6	heat shock	6.417219	2.681948	6.29E-25	1.36E-22	yes	up
ENSG0000	AHSA2P	activator of	40.99792	5.357479	5.60E-06	0.000182	yes	up
ENSG0000	SPDYE2B	speedy/RIN	28.33002	4.82426	0.000193	0.004191	yes	up
ENSG0000	-	------	4.004572	2.001648	0.002525	0.038637	yes	up
ENSG0000	FAM3C2P	family with s	0.388163	-1.365267	5.46E-10	3.54E-08	yes	down
ENSG0000	TLR6	toll like rece	3.915531	1.969208	0.003344	0.048935	yes	up
ENSG0000	DDIT3	DNA damag	2.091443	1.064499	8.16E-80	8.74E-77	yes	up
ENSG0000	CHST1	carbohydra	4.894414	2.291136	0.001243	0.021265	yes	up
ENSG0000	ISX	intestine sp	2.242642	1.165199	0.003236	0.047563	yes	up
ENSG0000	NR2F1	nuclear rec	0.457179	-1.12917	5.47E-10	3.54E-08	yes	down
ENSG0000	CNTD1	cyclin N-ter	2.038992	1.027856	0.001735	0.028193	yes	up
ENSG0000	TCIM	transcription	3.768961	1.914167	2.78E-05	0.000767	yes	up
ENSG0000	FIBIN	fin bud initia	4.468312	2.15973	1.03E-13	1.04E-11	yes	up
ENSG0000	DLGAP1-A	DLGAP1 ar	2.199909	1.137444	1.07E-05	0.000325	yes	up
ENSG0000	SAMD9L	sterile alpha	19.57766	4.291136	1.90E-05	0.000546	yes	up
ENSG0000	STX19	syntaxin 19	2.519184	1.332956	0.001462	0.024371	yes	up
ENSG0000	FAM133A	family with s	3.262943	1.706174	0.000168	0.003705	yes	up
ENSG0000	NLRP11	NLR family	4.606508	2.203673	0.002223	0.03469	yes	up
ENSG0000	SRP9P1	signal recog	24.71391	4.627252	0.000548	0.010433	yes	up
ENSG0000	SHISA2	shisa family	2.648742	1.405307	0.000146	0.003274	yes	up
ENSG0000	DDX60L	DExD/H-bo	2.006807	1.004902	9.03E-08	4.13E-06	yes	up
ENSG0000	MBD3L3	methyl-CpG	0.21084	-2.245781	7.32E-05	0.001794	yes	down
ENSG0000	CRIP2	cysteine ric	0.371493	-1.428595	3.18E-05	0.000863	yes	down
ENSG0000	BEGAIN	brain enrich	0.104693	-3.255758	3.87E-05	0.001022	yes	down
ENSG0000	-	nucleolar pr	6.525886	2.706174	0.000387	0.007709	yes	up
ENSG0000	-	novel transc	2.847264	1.509576	2.44E-05	0.000682	yes	up
ENSG0000	SPDYE12	speedy/RIN	19.46249	4.282625	0.002586	0.039327	yes	up
ENSG0000	DDX53	DEAD-box	2.144409	1.10058	0.00068	0.012553	yes	up
ENSG0000	DYNLT2	dynein light	2.268356	1.181647	7.93E-05	0.001924	yes	up
ENSG0000	FMNL1	formin like 1	6.058637	2.598993	1.03E-06	3.84E-05	yes	up
ENSG0000	RFX6	regulatory f	0.140442	-2.831951	1.45E-07	6.40E-06	yes	down
ENSG0000	TNFAIP2	TNF alpha	0.490508	-1.027652	0.002755	0.041571	yes	down
ENSG0000	HS6ST3	heparan su	0.143953	-2.796327	0.001099	0.019139	yes	down
ENSG0000	FAM131C	family with s	0.156969	-2.671451	0.000186	0.004064	yes	down
ENSG0000	CYP4Z1	cytochrome	3.546772	1.826507	0.000225	0.00481	yes	up
ENSG0000	CIDECP1	CIDEC pse	0.046693	-4.420635	5.71E-07	2.27E-05	yes	down
ENSG0000	DEFB132	defensin be	0.450316	-1.150992	2.42E-08	1.23E-06	yes	down
ENSG0000	C3orf70	chromosom	2.303254	1.203673	0.002199	0.034366	yes	up
ENSG0000	AKR1C1	aldo-keto re	2.573372	1.36366	7.83E-12	6.46E-10	yes	up
ENSG0000	MAGED4B	MAGE fami	0.469022	-1.092272	3.79E-16	4.62E-14	yes	down
ENSG0000	LUZP2	leucine zipp	0.346956	-1.527176	2.66E-10	1.80E-08	yes	down

ENSG0000	SAMD11	sterile alpha	0.418765	-1.255788	3.75E-26	8.59E-24	yes	down
ENSG0000	THSD4	thrombospo	2.175295	1.121211	6.42E-09	3.63E-07	yes	up
ENSG0000	PLA2G4E	phospholipa	0.094473	-3.403953	0.001801	0.029041	yes	down
ENSG0000	NUTM2B	NUT family	2.219379	1.150156	0.002381	0.036805	yes	up
ENSG0000	ZNF793	zinc finger p	2.224358	1.153389	3.05E-10	2.05E-08	yes	up
ENSG0000	MIR3667H(	MIR3667 h	0.261733	-1.93383	0.002116	0.033278	yes	down
ENSG0000	NPIPP1	nuclear por	2.333604	1.22256	0.001194	0.020527	yes	up
ENSG0000	FAM72B	family with s	2.386107	1.254658	1.11E-11	9.01E-10	yes	up
ENSG0000	GUSBP15	GUSB pseu	0.217314	-2.202146	0.000373	0.007468	yes	down
ENSG0000	SLC6A9	solute carri	2.00867	1.006241	4.87E-27	1.16E-24	yes	up
ENSG0000	H2AC7	H2A cluster	0.175503	-2.510434	0.000651	0.012069	yes	down
ENSG0000	ZNF300P1	zinc finger p	0.039657	-4.656296	0.000176	0.003874	yes	down
ENSG0000	H4C11	H4 clustere	0.033871	-4.883789	4.27E-05	0.001115	yes	down
ENSG0000	ZNF165	zinc finger p	2.127917	1.089442	7.58E-11	5.53E-09	yes	up
ENSG0000	IRF1-AS1	IRF1 antise	3.086594	1.626016	5.05E-05	0.001293	yes	up
ENSG0000	DMBX1	diencephalo	0.372585	-1.424358	0.002113	0.033247	yes	down
ENSG0000	AKR1B10	aldo-keto re	3.140801	1.651132	1.04E-06	3.88E-05	yes	up
ENSG0000	TMEM229E	transmemb	0.442933	-1.174838	3.65E-06	0.000123	yes	down
ENSG0000	ZNF876P	zinc finger p	2.174631	1.12077	0.001943	0.030977	yes	up
ENSG0000	PEG3	paternally e	0.416668	-1.263029	4.59E-10	3.02E-08	yes	down
ENSG0000	ATL1	atlastin GTI	2.118013	1.082712	0.000239	0.005074	yes	up
ENSG0000	ARMH1	armadillo lik	2.193575	1.133284	0.002753	0.041551	yes	up
ENSG0000	ZNF534	zinc finger p	2.08999	1.063496	0.001646	0.026937	yes	up
ENSG0000	ABCA4	ATP binding	8.829139	3.142273	8.82E-06	0.000275	yes	up
ENSG0000	STYXL2	serine/threo	3.043585	1.605772	1.06E-07	4.80E-06	yes	up
ENSG0000	SFMBT2	Scm like wi	5.247221	2.391554	0.000463	0.009017	yes	up
ENSG0000	OR2L2	olfactory re	21.44329	4.422455	0.001433	0.023951	yes	up
ENSG0000	BVES-AS1	BVES antis	0.342311	-1.54662	0.002545	0.038848	yes	down
ENSG0000	FOXO6	forkhead bo	0.442933	-1.174838	0.00106	0.018561	yes	down
ENSG0000	CD177	CD177 mol	0.052347	-4.255758	0.001382	0.02327	yes	down
ENSG0000	FRG2	FSHD regio	9.229868	3.20631	0.00015	0.003364	yes	up
ENSG0000	SAMD9	sterile alpha	2.103638	1.072887	1.15E-07	5.18E-06	yes	up
ENSG0000	EIF3CL	eukaryotic t	43.37027	5.438634	2.94E-06	0.000101	yes	up
ENSG0000	-	novel trans	2.879067	1.525601	0.002911	0.043582	yes	up
ENSG0000	ZCWPW2	zinc finger (	2.334635	1.223197	0.001584	0.026109	yes	up
ENSG0000	RPL23AP4	ribosomal p	23.56229	4.558408	0.000768	0.014005	yes	up
ENSG0000	VDAC1	voltage dep	0.469653	-1.090332	0	0	yes	down
ENSG0000	SULT1A4	sulfotransfe	0.023552	-5.407998	4.78E-12	4.04E-10	yes	down
ENSG0000	EML6	EMAP like (	2.096552	1.068018	0.000144	0.003234	yes	up
ENSG0000	-	alpha-2-ma	3.123437	1.643134	0.003045	0.045197	yes	up
ENSG0000	NPIPA8	nuclear por	0.340771	-1.553126	0.002776	0.041828	yes	down
ENSG0000	CXorf49B	chromosom	0.471794	-1.08377	2.34E-13	2.26E-11	yes	down
ENSG0000	LINC02449	long interge	4.308574	2.107211	0.000413	0.00816	yes	up
ENSG0000	-	novel trans	19.16307	4.260257	0.002828	0.042487	yes	up
ENSG0000	HNRNPA1I	heterogene	0.042433	-4.558675	0.000306	0.006251	yes	down
ENSG0000	RSC1A1	regulator of	2.789731	1.480126	0.000644	0.01197	yes	up
ENSG0000	-	novel trans	2.236814	1.161445	1.22E-06	4.51E-05	yes	up
ENSG0000	APOL6	apolipoprot	2.763905	1.466708	8.59E-15	9.40E-13	yes	up
ENSG0000	LINC01778	long interge	2.214667	1.14709	0.000724	0.013277	yes	up
ENSG0000	RPS10P7	ribosomal p	6.419707	2.682508	0.000682	0.012586	yes	up
ENSG0000	POTEF-AS	POTEF ant	0.460651	-1.118255	0.002029	0.032101	yes	down
ENSG0000	LINC01133	long interge	2.967654	1.569323	6.51E-07	2.55E-05	yes	up
ENSG0000	MSL3P1	MSL compl	3.028146	1.598435	4.78E-05	0.001235	yes	up
ENSG0000	PINCR	p53-induce	3.742787	1.904113	0.000387	0.007713	yes	up
ENSG0000	NPIPA3	nuclear por	0.072118	-3.793488	2.70E-06	9.37E-05	yes	down
ENSG0000	RNF32-AS	RNF32 anti	2.461745	1.299681	0.000528	0.010112	yes	up
ENSG0000	-	novel trans	3.310927	1.727235	0.001797	0.029019	yes	up
ENSG0000	ZBTB45P1	zinc finger a	2.214821	1.14719	0.001458	0.024307	yes	up

ENSG0000	TMEM30A-	TMEM30A	2.009788	1.007043	0.000366	0.007354	yes		up
ENSG0000	-	novel trans	2.879067	1.525601	1.58E-06	5.72E-05	yes		up
ENSG0000	-	ribosomal p	0.052347	-4.255758	0.001382	0.02327	yes		down
ENSG0000	POLR2J2	RNA polym	6.036218	2.593645	1.06E-07	4.80E-06	yes		up
ENSG0000	LINC01004	long interge	2.260601	1.176706	0.000383	0.007637	yes		up
ENSG0000	SDAD1P1	SDA1 doma	0.012472	-6.32521	1.02E-11	8.29E-10	yes		down
ENSG0000	RHEBP1	RHEB pseu	0.141907	-2.816986	0.000168	0.003714	yes		down
ENSG0000	ATXN1-AS	ATXN1 anti	2.879067	1.525601	0.002911	0.043582	yes		up
ENSG0000	PRAMEF36	PRAME far	36.02289	5.170842	2.20E-05	0.000619	yes		up
ENSG0000	LINC01756	long interge	0.057581	-4.118255	0.00251	0.038441	yes		down
ENSG0000	ITPR1-DT	ITPR1 dive	2.123312	1.086316	0.000441	0.008643	yes		up
ENSG0000	IFT122P3	IFT122 pse	2.336157	1.224137	2.22E-05	0.000624	yes		up
ENSG0000	FAM225A	family with	34.54881	5.110564	3.32E-05	0.000897	yes		up
ENSG0000	SHQ1P1	SHQ1, H/A	4.367084	2.12667	0.001403	0.023539	yes		up
ENSG0000	-	novel trans	2.311587	1.208884	0.001304	0.02218	yes		up
ENSG0000	SAT1-DT	SAT1 diver	6.909761	2.788636	0.002454	0.037739	yes		up
ENSG0000	-	zinc finger	20.78885	4.377738	0.000745	0.013622	yes		up
ENSG0000	SNRK-AS1	SNRK antis	5.954468	2.573973	0.000159	0.00354	yes		up
ENSG0000	LINC01287	long interge	3.838756	1.940639	0.001621	0.026612	yes		up
ENSG0000	ECI2-DT	ECI2 diverg	3.646818	1.866638	0.002761	0.041636	yes		up
ENSG0000	LINC02966	long interge	2.245672	1.167148	0.002471	0.037942	yes		up
ENSG0000	LINC01967	long interge	4.53275	2.180387	7.81E-05	0.001897	yes		up
ENSG0000	GUSBP17	GUSB pseu	20.29167	4.342815	0.002018	0.031959	yes		up
ENSG0000	-	proteasome	2.044138	1.031492	0.00022	0.004704	yes		up
ENSG0000	RPL13AP5	ribosomal p	0.168729	-2.567219	0.000207	0.004457	yes		down
ENSG0000	PRPF31-AS	PRPF31 ar	2.255384	1.173373	0.000656	0.012151	yes		up
ENSG0000	-	novel trans	6.443332	2.687807	0.000703	0.012933	yes		up
ENSG0000	SMG1P1	SMG1 pseu	0.003948	-7.984503	7.30E-05	0.001791	yes		down
ENSG0000	OXCT2P1	3-oxoacid C	0.096827	-3.368442	0.000267	0.005581	yes		down
ENSG0000	LINC02984	long interge	6.14201	2.618711	0.000718	0.013182	yes		up
ENSG0000	KLHL41	kelch like fa	6.288564	2.652731	6.08E-05	0.001521	yes		up
ENSG0000	UGT1A1	UDP glucur	2.386982	1.255188	5.36E-10	3.48E-08	yes		up
ENSG0000	HLA-DMB	major histo	0.43422	-1.203502	0.000301	0.00617	yes		down
ENSG0000	ZNF702P	zinc finger	44.84435	5.486854	1.97E-06	6.99E-05	yes		up
ENSG0000	RPLP0P2	ribosomal p	17.51326	4.130376	3.31E-06	0.000113	yes		up
ENSG0000	GSTA2	glutathione	0.15704	-2.670796	0.000187	0.004083	yes		down
ENSG0000	PKD1P1	polycystin 1	0.490859	-1.026619	6.06E-05	0.001519	yes		down
ENSG0000	ETV5	ETS variant	3.071005	1.618711	0.000437	0.008571	yes		up
ENSG0000	C4A	complemer	2.674426	1.419229	0.001348	0.022783	yes		up
ENSG0000	ASAH1-AS	ASAH1 anti	4.870696	2.284128	6.32E-06	0.000204	yes		up
ENSG0000	LINC02709	long interge	3.310927	1.727235	0.001797	0.029019	yes		up
ENSG0000	NSMCE1-D	NSMCE1 d	6.909761	2.788636	0.002454	0.037739	yes		up
ENSG0000	SBF2-AS1	SBF2 antis	3.387555	1.760244	4.53E-05	0.001177	yes		up
ENSG0000	-	novel trans	2.217948	1.149226	0.000556	0.010556	yes		up
ENSG0000	-	novel trans	2.687129	1.426066	7.38E-05	0.001807	yes		up
ENSG0000	MISFA	mitochondr	0.055527	-4.170671	0.002012	0.031888	yes		down
ENSG0000	FAM13A-AS	FAM13A ar	2.613916	1.386213	1.51E-09	9.33E-08	yes		up
ENSG0000	-	novel trans	5.470228	2.451601	0.000382	0.007625	yes		up
ENSG0000	LUCAT1	lung cancer	2.648193	1.405009	9.99E-05	0.002363	yes		up
ENSG0000	FLJ42969	uncharacte	6.909761	2.788636	0.002454	0.037739	yes		up
ENSG0000	-	novel trans	0.287994	-1.795888	0.000353	0.007116	yes		down
ENSG0000	-	novel trans	19.71585	4.301284	0.002397	0.037008	yes		up
ENSG0000	MCPH1-AS	MCPH1 ant	3.477462	1.798035	9.85E-17	1.26E-14	yes		up
ENSG0000	-	novel protei	0.02914	-5.100838	9.08E-06	0.000282	yes		down
ENSG0000	-	novel trans	0.152203	-2.715928	4.70E-05	0.001217	yes		down
ENSG0000	-	novel trans	2.08328	1.058857	1.31E-08	6.97E-07	yes		up
ENSG0000	FOXD1	forkhead bo	14.52202	3.86017	0.000357	0.00719	yes		up
ENSG0000	-	novel tripar	5.641144	2.495988	0.00122	0.020892	yes		up

ENSG0000	-	novel transc	0.09062	-3.46403	1.43E-08	7.57E-07	yes	down
ENSG0000	-	novel transc	4.836833	2.274063	0.000354	0.007133	yes	up
ENSG0000	SLC10A5	solute carrie	2.99423	1.582185	0.001822	0.029348	yes	up
ENSG0000	-	novel transc	9.213015	3.203673	4.66E-06	0.000153	yes	up
ENSG0000	-	Family with	0.045233	-4.466487	0.000499	0.009617	yes	down
ENSG0000	-	novel protei	79.278	6.308849	2.87E-10	1.93E-08	yes	up
ENSG0000	-	glucuronida	19.02488	4.249815	0.002948	0.044045	yes	up
ENSG0000	-	novel transc	3.798335	1.925367	1.03E-06	3.86E-05	yes	up
ENSG0000	NPIPA2	nuclear por	7.41878	2.891182	4.32E-07	1.77E-05	yes	up
ENSG0000	-	novel transc	2.085423	1.06034	0.000341	0.006904	yes	up
ENSG0000	LINC02489	long interge	5.374259	2.426066	0.002439	0.037559	yes	up
ENSG0000	EID3	EP300 inter	11.51627	3.525601	0.002093	0.032985	yes	up
ENSG0000	-	novel protei	0.103495	-3.27237	0.000238	0.00505	yes	down
ENSG0000	-	novel transc	0.472229	-1.082443	0.001733	0.028167	yes	down
ENSG0000	-	novel protei	4.852497	2.278727	9.46E-13	8.76E-11	yes	up
ENSG0000	GOLGA2P1	GOLGA2 p	72.2761	6.175447	1.62E-09	9.95E-08	yes	up
ENSG0000	-	novel transc	3.19857	1.677427	0.000906	0.016133	yes	up
ENSG0000	LINC02552	long interge	25.33579	4.663105	0.000458	0.008926	yes	up
ENSG0000	-	novel protei	0.009284	-6.750988	5.37E-15	5.98E-13	yes	down
ENSG0000	LIMS3	LIM zinc fin	6.613406	2.725393	1.31E-05	0.000391	yes	up
ENSG0000	ZNF271P	zinc finger p	2.131749	1.092037	0.000203	0.004388	yes	up
ENSG0000	ZBED6	zinc finger B	2.03363	1.024057	1.74E-11	1.37E-09	yes	up
ENSG0000	-	novel protei	55.02473	5.782008	1.33E-07	5.90E-06	yes	up
ENSG0000	-	novel protei	34.54881	5.110564	3.32E-05	0.000897	yes	up
ENSG0000	PPP1R12A	PPP1R12A	2.630214	1.39518	7.34E-05	0.001799	yes	up
ENSG0000	-	novel transc	2.481688	1.311322	8.79E-05	0.002107	yes	up
ENSG0000	-	novel protei	39.75416	5.313034	7.86E-06	0.000248	yes	up
ENSG0000	-	novel transc	0.451618	-1.146824	6.86E-06	0.000219	yes	down
ENSG0000	-	FGFR1 onc	3.915531	1.969208	0.003344	0.048935	yes	up
ENSG0000	-	novel transc	0.140729	-2.829004	0.000199	0.004312	yes	down
ENSG0000	BCL2L2-PA	BCL2L2-PA	0.481053	-1.055733	7.95E-05	0.001927	yes	down
ENSG0000	LINC00638	long interge	3.82225	1.934422	5.08E-05	0.001299	yes	up
ENSG0000	PRC1-AS1	PRC1 antis	3.066344	1.61652	0.00058	0.010938	yes	up
ENSG0000	-	novel transc	0.128315	-2.962239	0.000373	0.00747	yes	down
ENSG0000	RNASE4	ribonucleas	0.387856	-1.366408	7.81E-05	0.001897	yes	down
ENSG0000	-	novel transc	2.99423	1.582185	0.001822	0.029348	yes	up
ENSG0000	UBE2F-SC	UBE2F-SC	0.042339	-4.561861	0.0003	0.006165	yes	down
ENSG0000	-	novel protei	2.784985	1.47767	0.000515	0.009885	yes	up
ENSG0000	-	novel protei	26.51045	4.728489	0.000326	0.006618	yes	up
ENSG0000	SNRPA1-D	SNRPA1 di	105.5351	6.721579	5.33E-13	5.05E-11	yes	up
ENSG0000	-	novel transc	20.15347	4.332956	0.002103	0.033113	yes	up
ENSG0000	ZHX1-C8or	ZHX1-C8or	12.84025	3.682601	6.23E-05	0.001555	yes	up
ENSG0000	TGIF2-RAE	TGIF2-RAE	0.037635	-4.731786	0.000113	0.002617	yes	down
ENSG0000	DUT-AS1	DUT antise	2.602547	1.379924	0.00092	0.016355	yes	up
ENSG0000	-	novel protei	0.053267	-4.230621	0.001548	0.025603	yes	down
ENSG0000	-	novel transc	6.909761	2.788636	0.002454	0.037739	yes	up
ENSG0000	-	novel transc	23.26756	4.540248	0.000944	0.01674	yes	up
ENSG0000	CDK13-DT	CDK13 dive	2.574225	1.364138	0.000738	0.013518	yes	up
ENSG0000	-	novel transc	108.322	6.759183	2.79E-13	2.68E-11	yes	up
ENSG0000	LINC02846	long interge	2.460294	1.298831	0.000301	0.006174	yes	up
ENSG0000	SLX1B-SUI	SLX1B-SUI	38.62974	5.27164	6.09E-27	1.45E-24	yes	up
ENSG0000	-	novel transc	2.365371	1.242066	7.99E-06	0.000252	yes	up
ENSG0000	-	novel transc	4.619624	2.207775	0.001315	0.022335	yes	up
ENSG0000	-	novel transc	4.606508	2.203673	1.17E-07	5.25E-06	yes	up
ENSG0000	HERC2P5	HERC2 pse	2.817502	1.494417	0.002214	0.034564	yes	up
ENSG0000	-	novel transc	0.436824	-1.194876	0.000457	0.008923	yes	down
ENSG0000	-	novel protei	5.009577	2.324689	5.57E-06	0.000181	yes	up
ENSG0000	CES1P2	carboxylest	20.72928	4.373598	0.001772	0.028695	yes	up

ENSG0000	AGAP11	ArfGAP witl	0.342005	-1.547911	0.000279	0.005798	yes	down
ENSG0000	-	novel trans	2.34454	1.229305	9.21E-08	4.21E-06	yes	up
ENSG0000	-	novel trans	2.811708	1.491447	0.002895	0.043381	yes	up
ENSG0000	DNAAF4-C	DNAAF4-C	2.763364	1.466426	0.001359	0.022936	yes	up
ENSG0000	DLGAP1-A	DLGAP1 ar	2.80781	1.489445	2.73E-13	2.64E-11	yes	up
ENSG0000	HNRNPA1I	heterogene	2.938808	1.555231	0.000114	0.002646	yes	up
ENSG0000	FAM72C	family with	2.884904	1.528523	1.31E-10	9.28E-09	yes	up
ENSG0000	-	novel protei	0.031569	-4.98536	2.12E-05	0.0006	yes	down
ENSG0000	-	novel trans	20.72928	4.373598	0.001772	0.028695	yes	up
ENSG0000	-	inositol poly	5.758134	2.525601	0.00021	0.004515	yes	up
ENSG0000	-	novel trans	0.023677	-5.400398	7.46E-07	2.90E-05	yes	down
ENSG0000	-	novel trans	0.375562	-1.412876	1.05E-06	3.91E-05	yes	down
ENSG0000	RNF157-AS	RNF157 an	9.623762	3.266601	2.35E-06	8.22E-05	yes	up
ENSG0000	ATF7-NPFI	ATF7-NPFI	0.040182	-4.637293	0.000197	0.004263	yes	down
ENSG0000	-	novel trans	5.758134	2.525601	0.001327	0.022497	yes	up
ENSG0000	-	novel protei	0.166657	-2.58505	4.15E-06	0.000138	yes	down
ENSG0000	ZSCAN16-/	ZSCAN16 a	2.043134	1.030784	0.001427	0.023873	yes	up
ENSG0000	-	novel trans	0.029559	-5.080248	1.06E-05	0.000323	yes	down
ENSG0000	-	ribosomal p	9.325369	3.221161	0.003103	0.045901	yes	up
ENSG0000	-	novel trans	3.860462	1.948773	0.001284	0.021889	yes	up
ENSG0000	TSNAX-DIS	TSNAX-DIS	0.10996	-3.184953	5.68E-05	0.001431	yes	down
ENSG0000	-	novel trans	2.777453	1.473763	0.000202	0.004371	yes	up
ENSG0000	-	novel trans	0.257233	-1.95885	0.000565	0.010699	yes	down
ENSG0000	UBE2CP5	ubiquitin co	7.440653	2.895429	0.000142	0.00321	yes	up
ENSG0000	-	novel trans	19.74218	4.303209	0.002631	0.039915	yes	up
ENSG0000	-	novel trans	0.413259	-1.274881	0.001551	0.025647	yes	down
ENSG0000	-	novel trans	53.03545	5.728885	0.001411	0.023659	yes	up
ENSG0000	CASC15	cancer sus	2.826721	1.499129	0.002258	0.035191	yes	up
ENSG0000	-	novel trans	3.140801	1.651132	0.000553	0.010515	yes	up
ENSG0000	FAM47E-S	FAM47E-S	59.00936	5.882872	4.72E-08	2.28E-06	yes	up
ENSG0000	-	novel trans	2.31766	1.212669	0.000337	0.006828	yes	up
ENSG0000	-	novel trans	30.05746	4.909651	0.000118	0.002718	yes	up
ENSG0000	TBC1D8-AS	TBC1D8 ar	3.204527	1.680111	3.27E-07	1.36E-05	yes	up
ENSG0000	ARL2-SNX	ARL2-SNX	5.211001	2.381561	9.84E-05	0.00233	yes	up
ENSG0000	-	novel trans	0.059118	-4.080248	0.002934	0.043859	yes	down
ENSG0000	-	novel trans	5.374259	2.426066	0.002439	0.037559	yes	up
ENSG0000	C1QTNF3-/	C1QTNF3-/	0.018892	-5.726118	2.82E-08	1.42E-06	yes	down
ENSG0000	DGCR11	DiGeorge s	0.245027	-2.028987	3.55E-06	0.00012	yes	down
ENSG0000	-	novel trans	0.17591	-2.507094	5.50E-09	3.14E-07	yes	down
ENSG0000	-	novel trans	0.048226	-4.374058	0.000793	0.014391	yes	down
ENSG0000	-	novel trans	2.610354	1.384246	0.001246	0.021282	yes	up
ENSG0000	-	ring finger p	0.407154	-1.296352	1.74E-05	0.000504	yes	down
ENSG0000	PMS2P8	PMS1 hom	0.029651	-5.075798	1.10E-05	0.000334	yes	down
ENSG0000	LINC01138	long interge	2.309689	1.207699	3.91E-05	0.001032	yes	up
ENSG0000	-	------	36.96722	5.208175	1.69E-05	0.000492	yes	up
ENSG0000	NPEPPSP1	NPEPPS p	0.053465	-4.225273	0.001585	0.026117	yes	down
ENSG0000	FGD5P1	FYVE, Rho	2.767421	1.468542	0.001164	0.020098	yes	up
ENSG0000	-	novel trans	2.605347	1.381476	0.000155	0.003457	yes	up
ENSG0000	-	novel trans	0.179755	-2.475893	2.15E-20	3.52E-18	yes	down
ENSG0000	HERC2P2	HERC2 pse	0.008952	-6.803522	1.85E-15	2.16E-13	yes	down
ENSG0000	CSPG4P10	chondroitin	6.554587	2.712505	2.10E-05	0.000596	yes	up
ENSG0000	-	novel trans	7.485575	2.904113	0.00129	0.021986	yes	up
ENSG0000	-	novel trans	2.922389	1.547148	0.000619	0.011565	yes	up
ENSG0000	PMS2P14	PMS1 hom	4.248439	2.086933	0.001957	0.031154	yes	up
ENSG0000	CHD9NB	CHD9 neig	2.367233	1.243202	0.00194	0.030956	yes	up
ENSG0000	PRAMEF34	PRAME far	0.176179	-2.504885	2.51E-10	1.70E-08	yes	down
ENSG0000	-	novel trans	34.54881	5.110564	3.32E-05	0.000897	yes	up
ENSG0000	-	novel trans	17.66496	4.142818	1.05E-09	6.58E-08	yes	up

ENSG0000	RDM1	RAD52 mot	2.12608	1.088196	0.001503	0.024947	yes	up
ENSG0000	TP53TG3F	TP53 targe	0.016175	-5.950132	2.00E-09	1.20E-07	yes	down
ENSG0000	-	TEC	19.60069	4.292833	0.002481	0.038072	yes	up
ENSG0000	PCDHB1-A	PCDHB1 ar	5.702298	2.511543	0.000838	0.015135	yes	up
ENSG0000	LINC01670	long interge	4.858143	2.280405	0.000301	0.00617	yes	up
ENSG0000	-	novel trans	0.478762	-1.062621	5.53E-08	2.64E-06	yes	down
ENSG0000	-	ribosomal p	0.049128	-4.347316	7.59E-07	2.94E-05	yes	down
ENSG0000	-	TEC	3.166974	1.663105	0.002952	0.044083	yes	up
ENSG0000	-	tec	2.63229	1.396318	0.001619	0.026597	yes	up
ENSG0000	-	novel ankyr	4.583475	2.196442	0.002328	0.036115	yes	up
ENSG0000	-	novel trans	0.255844	-1.966664	1.60E-06	5.77E-05	yes	down
ENSG0000	-	novel trans	21.02871	4.394288	0.001621	0.02662	yes	up
ENSG0000	-	TEC	3.549105	1.827455	8.12E-05	0.00196	yes	up
ENSG0000	-	TEC	3.867243	1.951305	1.27E-08	6.78E-07	yes	up
ENSG0000	-	novel trans	0.400566	-1.319889	0.000742	0.013575	yes	down
ENSG0000	-	novel pseu	25.49702	4.672257	0.000437	0.00856	yes	up
ENSG0000	FRG1CP	FSHD regic	0.402143	-1.314221	0.00117	0.020166	yes	down
ENSG0000	-	novel trans	0.011498	-6.442511	6.64E-37	2.43E-34	yes	down
ENSG0000	RN7SK	RNA comp	0.375531	-1.412998	0.00038	0.007584	yes	down
ENSG0000	-	novel trans	8.061388	3.011028	0.000676	0.012497	yes	up
ENSG0000	PLD5P1	PLD5 pseu	10.07806	3.333146	3.41E-05	0.000916	yes	up
ENSG0000	SMIM39	small integr	34.84823	5.123013	3.05E-05	0.000832	yes	up
ENSG0000	THSD8	thrombospo	26.78684	4.743453	0.000301	0.006168	yes	up
ENSG0000	-	novel protei	2.863632	1.517846	1.75E-09	1.07E-07	yes	up
ENSG0000	-	novel trans	0.165852	-2.592032	3.40E-49	1.74E-46	yes	down
ENSG0000	-	novel protei	76.19164	6.251561	6.14E-10	3.95E-08	yes	up
ENSG0000	-	novel protei	15.60928	3.964332	1.40E-08	7.43E-07	yes	up
ENSG0000	-	novel protei	0.147194	-2.764207	1.09E-17	1.46E-15	yes	down
ENSG0000	-	novel protei	2.060951	1.043311	0.000287	0.005947	yes	up
ENSG0000	-	novel trans	0.225025	-2.151844	0.000754	0.013766	yes	down
ENSG0000	-	novel trans	4.798445	2.262567	0.000102	0.002404	yes	up
ENSG0000	-	novel trans	60.34525	5.915168	3.34E-08	1.67E-06	yes	up
ENSG0000	-	novel trans	2.516311	1.331311	7.89E-10	5.02E-08	yes	up
ENSG0000	LCAL1	lung cancer	2.336157	1.224137	2.22E-05	0.000624	yes	up
ENSG0000	-	novel protei	59.8155	5.902447	3.83E-08	1.89E-06	yes	up
ENSG0000	-	------	40.85972	5.352607	5.81E-06	0.000188	yes	up
ENSG0000	-	novel protei	24.29933	4.602844	0.000619	0.011562	yes	up
ENSG0000	-	novel trans	25.01334	4.644626	0.000503	0.009678	yes	up
ENSG0000	-	novel trans	20.72928	4.373598	0.001772	0.028695	yes	up
ENSG0000	-	novel trans	2.079259	1.056069	0.000547	0.010407	yes	up
ENSG0000	-	novel trans	0.010573	-6.563452	1.87E-13	1.82E-11	yes	down
ENSG0000	-	novel trans	20.72928	4.373598	0.001772	0.028695	yes	up
ENSG0000	-	novel trans	3.897215	1.962443	0.001496	0.024847	yes	up
ENSG0000	-	novel trans	27.63905	4.788636	0.000235	0.004997	yes	up
ENSG0000	-	novel trans	16.12278	4.011028	0.00014	0.00317	yes	up
ENSG0000	-	novel trans	0.047237	-4.403953	0.000684	0.012626	yes	down
ENSG0000	HERC3	HECT and	4.694773	2.231055	1.36E-39	5.44E-37	yes	up
ENSG0000	-	novel protei	0.302455	-1.725207	4.68E-05	0.001212	yes	down
ENSG0000	-	novel trans	6.525886	2.706174	0.000387	0.007709	yes	up
ENSG0000	-	novel protei	13.46952	3.751626	0.000585	0.011012	yes	up
ENSG0000	-	novel protei	2.653115	1.407687	0.00133	0.022525	yes	up
ENSG0000	C8orf44-SC	C8orf44-SC	7.369356	2.881538	0.000898	0.016017	yes	up
ENSG0000	PDCD6-AH	PDCD6-AH	0.008327	-6.907984	2.03E-16	2.52E-14	yes	down
ENSG0000	-	novel trans	23.10164	4.529923	0.000879	0.015746	yes	up
ENSG0000	UGT1A3	UDP glucur	2.46175	1.299684	0.000481	0.009328	yes	up
ENSG0000	-	novel trans	5.758134	2.525601	0.001327	0.022497	yes	up
ENSG0000	-	novel trans	3.646818	1.866638	0.002761	0.041636	yes	up
ENSG0000	-	novel trans	3.915531	1.969208	0.003344	0.048935	yes	up

ENSG0000	-	novel trans(	28.42215	4.828944	0.000188	0.004094	yes		up
ENSG0000	-	novel trans(	3.838756	1.940639	0.001621	0.026612	yes		up
ENSG0000	-	novel trans(	0.391553	-1.35272	0.000338	0.00684	yes		down
ENSG0000	-	novel trans(	6.909761	2.788636	0.002454	0.037739	yes		up
ENSG0000	-	novel trans(	0.345488	-1.533292	0.000428	0.008426	yes		down
ENSG0000	-	novel trans(	14.97115	3.904113	0.000275	0.005715	yes		up
ENSG0000	TARP	TCR gamm	2.000289	1.000208	0.001452	0.024229	yes		up
ENSG0000	-	novel trans(	0.050333	-4.312342	0.001065	0.018627	yes		down
ENSG0000	-	novel protei	0.046549	-4.4251	0.000616	0.011514	yes		down
ENSG0000	-	novel protei	2.050027	1.035643	0.00131	0.022252	yes		up
ENSG0000	-	novel trans(	6.904355	2.787507	0.001784	0.028843	yes		up
ENSG0000	-	novel trans(	2.264075	1.178922	1.73E-07	7.51E-06	yes		up
ENSG0000	-	novel trans(	55.30112	5.789237	1.23E-07	5.51E-06	yes		up
ENSG0000	-	novel trans(	2.109342	1.076793	7.98E-11	5.80E-09	yes		up
ENSG0000	-	novel trans(	0.057035	-4.132002	0.003211	0.047242	yes		down
ENSG0000	-	novel trans(	0.037684	-4.729899	0.000114	0.002643	yes		down
ENSG0000	NMRAL2P	NmrA like r	5.403788	2.433971	2.32E-10	1.58E-08	yes		up
ENSG0000	-	novel trans(	5.053298	2.337225	3.50E-06	0.000118	yes		up
ENSG0000	-	novel trans(	2.156162	1.108466	7.09E-05	0.001743	yes		up
ENSG0000	HLA-L	major histo(	2.803828	1.487398	0.000414	0.008167	yes		up
ENSG0000	-	------	2.687129	1.426066	7.38E-05	0.001807	yes		up
ENSG0000	RP9P	RP9 pseud(	0.102983	-3.279522	0.002688	0.040677	yes		down
ENSG0000	-	novel trans(	16.47978	4.042625	0.000114	0.002647	yes		up
ENSG0000	A2MP1	alpha-2-ma	24.62178	4.621863	0.000563	0.010675	yes		up
ENSG0000	-	novel trans(	5.839582	2.545865	3.79E-06	0.000127	yes		up
ENSG0000	-	novel trans(	0.106474	-3.231427	2.82E-05	0.000777	yes		down
ENSG0000	-	novel trans(	27.06323	4.758262	0.000278	0.005765	yes		up
ENSG0000	SMG1P7	SMG1 pseu	2.290247	1.195503	0.00132	0.022396	yes		up
ENSG0000	-	novel trans(	0.17783	-2.491426	2.99E-05	0.000817	yes		down
ENSG0000	PLCXD1	phosphatid	0.006561	-7.251896	9.25E-08	4.22E-06	yes		down
ENSG0000	ZBED1	zinc finger I	19.98166	4.320605	2.53E-24	5.16E-22	yes		up
ENSG0000	-	novel trans(	2.697688	1.431723	0.000682	0.012589	yes		up
ENSG0000	TPI1P2	triosephosp	0.471495	-1.084685	0.000167	0.003687	yes		down
ENSG0000	-	novel trans(	24.69088	4.625906	0.000552	0.010494	yes		up
ENSG0000	TEX14BP	testis expre	0.15355	-2.703217	0.001905	0.030493	yes		down

Fig.S1 B

caseid	Cancer	Group	GLUD1[log2(TPM+1)]
TCGA-BL-A13J-01A	BLCA	Tumor	6.98
TCGA-BL-A13J-11A	BLCA	Normal	7.43
TCGA-BT-A20N-01A	BLCA	Tumor	7.51
TCGA-BT-A20N-11A	BLCA	Normal	7.14
TCGA-BT-A20Q-01A	BLCA	Tumor	6.81
TCGA-BT-A20Q-11A	BLCA	Normal	7.38
TCGA-BT-A20R-01A	BLCA	Tumor	6.53
TCGA-BT-A20R-11A	BLCA	Normal	7.19
TCGA-BT-A20U-01A	BLCA	Tumor	7.2
TCGA-BT-A20U-11A	BLCA	Normal	6.8
TCGA-BT-A20W-01A	BLCA	Tumor	6.94
TCGA-BT-A20W-11A	BLCA	Normal	7.4
TCGA-BT-A2LA-01A	BLCA	Tumor	6.98
TCGA-BT-A2LA-11A	BLCA	Normal	6.99
TCGA-BT-A2LB-01A	BLCA	Tumor	7.24
TCGA-BT-A2LB-11A	BLCA	Normal	6.87
TCGA-CU-A0YN-01A	BLCA	Tumor	6.76
TCGA-CU-A0YN-11A	BLCA	Normal	7.22
TCGA-CU-A0YR-01A	BLCA	Tumor	7.6
TCGA-CU-A0YR-11A	BLCA	Normal	7.36
TCGA-GC-A3BM-01A	BLCA	Tumor	7.07
TCGA-GC-A3BM-11A	BLCA	Normal	7.04
TCGA-GC-A3WC-01A	BLCA	Tumor	6.39
TCGA-GC-A3WC-11A	BLCA	Normal	7.15
TCGA-GC-A6I3-01A	BLCA	Tumor	5.54
TCGA-GC-A6I3-11A	BLCA	Normal	5.78
TCGA-GD-A2C5-01A	BLCA	Tumor	7.48
TCGA-GD-A2C5-11A	BLCA	Normal	6.75
TCGA-GD-A3OP-01A	BLCA	Tumor	6.72
TCGA-GD-A3OP-11A	BLCA	Normal	7.06
TCGA-GD-A3OQ-01A	BLCA	Tumor	7.22
TCGA-GD-A3OQ-11A	BLCA	Normal	7.12
TCGA-K4-A3WV-01A	BLCA	Tumor	6.67
TCGA-K4-A3WV-11A	BLCA	Normal	7.19
TCGA-K4-A54R-01A	BLCA	Tumor	6.58
TCGA-K4-A54R-11A	BLCA	Normal	7.31
TCGA-K4-A5RI-01A	BLCA	Tumor	7.09
TCGA-K4-A5RI-11A	BLCA	Normal	7.03
TCGA-A7-A0CE-01A	BRCA	Tumor	7.21
TCGA-A7-A0CE-11A	BRCA	Normal	8.08
TCGA-A7-A0CH-01A	BRCA	Tumor	8.26
TCGA-A7-A0CH-11A	BRCA	Normal	7.39
TCGA-A7-A0D9-01A	BRCA	Tumor	7.9
TCGA-A7-A0D9-11A	BRCA	Normal	7.06
TCGA-A7-A0DB-01A	BRCA	Tumor	7.04
TCGA-A7-A0DB-11A	BRCA	Normal	7.46
TCGA-A7-A0DC-01A	BRCA	Tumor	8.36
TCGA-A7-A0DC-11A	BRCA	Normal	8.13
TCGA-A7-A13E-01A	BRCA	Tumor	7.74
TCGA-A7-A13E-11A	BRCA	Normal	7.41
TCGA-A7-A13F-01A	BRCA	Tumor	7.36
TCGA-A7-A13F-11A	BRCA	Normal	7.32
TCGA-A7-A13G-01B	BRCA	Tumor	6.83
TCGA-A7-A13G-11A	BRCA	Normal	7.07
TCGA-AC-A23H-01A	BRCA	Tumor	7.55
TCGA-AC-A23H-11A	BRCA	Normal	7.93

TCGA-AC-A2FB-01A	BRCA	Tumor	7.52
TCGA-AC-A2FB-11A	BRCA	Normal	7.81
TCGA-AC-A2FF-01A	BRCA	Tumor	7.83
TCGA-AC-A2FF-11A	BRCA	Normal	7.46
TCGA-AC-A2FM-01A	BRCA	Tumor	8.15
TCGA-AC-A2FM-11B	BRCA	Normal	7.42
TCGA-BH-A0AU-01A	BRCA	Tumor	8.63
TCGA-BH-A0AU-11A	BRCA	Normal	7.63
TCGA-BH-A0AY-01A	BRCA	Tumor	7.09
TCGA-BH-A0AY-11A	BRCA	Normal	7.82
TCGA-BH-A0AZ-01A	BRCA	Tumor	7.19
TCGA-BH-A0AZ-11A	BRCA	Normal	8.08
TCGA-BH-A0B3-01A	BRCA	Tumor	6.27
TCGA-BH-A0B3-11B	BRCA	Normal	8.37
TCGA-BH-A0B5-01A	BRCA	Tumor	7.67
TCGA-BH-A0B5-11A	BRCA	Normal	7.14
TCGA-BH-A0B7-01A	BRCA	Tumor	6.97
TCGA-BH-A0B7-11A	BRCA	Normal	8.03
TCGA-BH-A0B8-01A	BRCA	Tumor	8.62
TCGA-BH-A0B8-11A	BRCA	Normal	7.09
TCGA-BH-A0BA-01A	BRCA	Tumor	7.73
TCGA-BH-A0BA-11A	BRCA	Normal	7.78
TCGA-BH-A0BC-01A	BRCA	Tumor	7.83
TCGA-BH-A0BC-11A	BRCA	Normal	7.78
TCGA-BH-A0BJ-01A	BRCA	Tumor	7.22
TCGA-BH-A0BJ-11A	BRCA	Normal	7.75
TCGA-BH-A0BM-01A	BRCA	Tumor	7.75
TCGA-BH-A0BM-11A	BRCA	Normal	7.56
TCGA-BH-A0BQ-01A	BRCA	Tumor	7.45
TCGA-BH-A0BQ-11A	BRCA	Normal	7.77
TCGA-BH-A0BS-01A	BRCA	Tumor	8.54
TCGA-BH-A0BS-11A	BRCA	Normal	8.21
TCGA-BH-A0BT-01A	BRCA	Tumor	8.49
TCGA-BH-A0BT-11A	BRCA	Normal	8.47
TCGA-BH-A0BV-01A	BRCA	Tumor	7.23
TCGA-BH-A0BV-11A	BRCA	Normal	7.92
TCGA-BH-A0BW-01A	BRCA	Tumor	6.7
TCGA-BH-A0BW-11A	BRCA	Normal	7.21
TCGA-BH-A0BZ-01A	BRCA	Tumor	7.24
TCGA-BH-A0BZ-11A	BRCA	Normal	7.62
TCGA-BH-A0C0-01A	BRCA	Tumor	6.88
TCGA-BH-A0C0-11A	BRCA	Normal	8.16
TCGA-BH-A0C3-01A	BRCA	Tumor	7.14
TCGA-BH-A0C3-11A	BRCA	Normal	7.46
TCGA-BH-A0DD-01A	BRCA	Tumor	7.75
TCGA-BH-A0DD-11A	BRCA	Normal	7.11
TCGA-BH-A0DG-01A	BRCA	Tumor	7.34
TCGA-BH-A0DG-11A	BRCA	Normal	8.23
TCGA-BH-A0DH-01A	BRCA	Tumor	7.47
TCGA-BH-A0DH-11A	BRCA	Normal	7.4
TCGA-BH-A0DK-01A	BRCA	Tumor	6.71
TCGA-BH-A0DK-11A	BRCA	Normal	8.13
TCGA-BH-A0DL-01A	BRCA	Tumor	6.29
TCGA-BH-A0DL-11A	BRCA	Normal	7.47
TCGA-BH-A0DO-01B	BRCA	Tumor	8.16
TCGA-BH-A0DO-11A	BRCA	Normal	7.78
TCGA-BH-A0DP-01A	BRCA	Tumor	8.26
TCGA-BH-A0DP-11A	BRCA	Normal	8.6

TCGA-BH-A0DQ-01A	BRCA	Tumor	7.51
TCGA-BH-A0DQ-11A	BRCA	Normal	8.57
TCGA-BH-A0DT-01A	BRCA	Tumor	8.2
TCGA-BH-A0DT-11A	BRCA	Normal	8.19
TCGA-BH-A0DV-01A	BRCA	Tumor	7.82
TCGA-BH-A0DV-11A	BRCA	Normal	8.56
TCGA-BH-A0DZ-01A	BRCA	Tumor	7.54
TCGA-BH-A0DZ-11A	BRCA	Normal	7.38
TCGA-BH-A0E0-01A	BRCA	Tumor	6.32
TCGA-BH-A0E0-11A	BRCA	Normal	7.88
TCGA-BH-A0E1-01A	BRCA	Tumor	7.45
TCGA-BH-A0E1-11A	BRCA	Normal	7.77
TCGA-BH-A0H5-01A	BRCA	Tumor	7.73
TCGA-BH-A0H5-11A	BRCA	Normal	7.94
TCGA-BH-A0H7-01A	BRCA	Tumor	7.32
TCGA-BH-A0H7-11A	BRCA	Normal	7.68
TCGA-BH-A0H9-01A	BRCA	Tumor	7.58
TCGA-BH-A0H9-11A	BRCA	Normal	7.14
TCGA-BH-A0HA-01A	BRCA	Tumor	7.19
TCGA-BH-A0HA-11A	BRCA	Normal	7.52
TCGA-BH-A0HK-01A	BRCA	Tumor	7.09
TCGA-BH-A0HK-11A	BRCA	Normal	8.04
TCGA-BH-A18J-01A	BRCA	Tumor	7.4
TCGA-BH-A18J-11A	BRCA	Normal	7.66
TCGA-BH-A18K-01A	BRCA	Tumor	7.8
TCGA-BH-A18K-11A	BRCA	Normal	7.91
TCGA-BH-A18L-01A	BRCA	Tumor	8.3
TCGA-BH-A18L-11A	BRCA	Normal	7.74
TCGA-BH-A18M-01A	BRCA	Tumor	7.6
TCGA-BH-A18M-11A	BRCA	Normal	7.66
TCGA-BH-A18N-01A	BRCA	Tumor	8.22
TCGA-BH-A18N-11A	BRCA	Normal	7.83
TCGA-BH-A18P-01A	BRCA	Tumor	8.01
TCGA-BH-A18P-11A	BRCA	Normal	7.32
TCGA-BH-A18Q-01A	BRCA	Tumor	7.41
TCGA-BH-A18Q-11A	BRCA	Normal	8.13
TCGA-BH-A18R-01A	BRCA	Tumor	8.61
TCGA-BH-A18R-11A	BRCA	Normal	7.78
TCGA-BH-A18S-01A	BRCA	Tumor	8.58
TCGA-BH-A18S-11A	BRCA	Normal	7.56
TCGA-BH-A18U-01A	BRCA	Tumor	7.28
TCGA-BH-A18U-11A	BRCA	Normal	7.89
TCGA-BH-A18V-06A	BRCA	Tumor	6.56
TCGA-BH-A18V-11A	BRCA	Normal	7.26
TCGA-BH-A1EN-01A	BRCA	Tumor	8.07
TCGA-BH-A1EN-11A	BRCA	Normal	7.38
TCGA-BH-A1EO-01A	BRCA	Tumor	7.96
TCGA-BH-A1EO-11A	BRCA	Normal	7.62
TCGA-BH-A1ET-01A	BRCA	Tumor	8.5
TCGA-BH-A1ET-11B	BRCA	Normal	8.08
TCGA-BH-A1EU-01A	BRCA	Tumor	7.73
TCGA-BH-A1EU-11A	BRCA	Normal	7.91
TCGA-BH-A1EV-01A	BRCA	Tumor	7.52
TCGA-BH-A1EV-11A	BRCA	Normal	8
TCGA-BH-A1EW-01A	BRCA	Tumor	7.95
TCGA-BH-A1EW-11B	BRCA	Normal	8.02
TCGA-BH-A1F0-01A	BRCA	Tumor	6.98
TCGA-BH-A1F0-11B	BRCA	Normal	7.71

TCGA-BH-A1F2-01A	BRCA	Tumor	7.39
TCGA-BH-A1F2-11A	BRCA	Normal	7.23
TCGA-BH-A1F6-01A	BRCA	Tumor	6.72
TCGA-BH-A1F6-11B	BRCA	Normal	7.7
TCGA-BH-A1F8-01A	BRCA	Tumor	7.84
TCGA-BH-A1F8-11B	BRCA	Normal	7.73
TCGA-BH-A1FB-01A	BRCA	Tumor	8.04
TCGA-BH-A1FB-11A	BRCA	Normal	7.91
TCGA-BH-A1FC-01A	BRCA	Tumor	6.94
TCGA-BH-A1FC-11A	BRCA	Normal	8
TCGA-BH-A1FD-01A	BRCA	Tumor	7.96
TCGA-BH-A1FD-11B	BRCA	Normal	7.84
TCGA-BH-A1FE-06A	BRCA	Tumor	7.34
TCGA-BH-A1FE-11B	BRCA	Normal	7.65
TCGA-BH-A1FG-01A	BRCA	Tumor	8.18
TCGA-BH-A1FG-11B	BRCA	Normal	8.11
TCGA-BH-A1FH-01A	BRCA	Tumor	7.31
TCGA-BH-A1FH-11B	BRCA	Normal	8.03
TCGA-BH-A1FJ-01A	BRCA	Tumor	8.93
TCGA-BH-A1FJ-11B	BRCA	Normal	8.65
TCGA-BH-A1FM-01A	BRCA	Tumor	7.12
TCGA-BH-A1FM-11B	BRCA	Normal	7.82
TCGA-BH-A1FN-01A	BRCA	Tumor	8.34
TCGA-BH-A1FN-11A	BRCA	Normal	7.65
TCGA-BH-A1FR-01A	BRCA	Tumor	8.54
TCGA-BH-A1FR-11B	BRCA	Normal	7.79
TCGA-BH-A1FU-01A	BRCA	Tumor	7.28
TCGA-BH-A1FU-11A	BRCA	Normal	7.72
TCGA-BH-A203-01A	BRCA	Tumor	7.77
TCGA-BH-A203-11A	BRCA	Normal	7.62
TCGA-BH-A204-01A	BRCA	Tumor	8.81
TCGA-BH-A204-11A	BRCA	Normal	7.18
TCGA-BH-A208-01A	BRCA	Tumor	7.16
TCGA-BH-A208-11A	BRCA	Normal	7.56
TCGA-BH-A209-01A	BRCA	Tumor	7.35
TCGA-BH-A209-11A	BRCA	Normal	8.01
TCGA-E2-A153-01A	BRCA	Tumor	7.53
TCGA-E2-A153-11A	BRCA	Normal	7.93
TCGA-E2-A158-01A	BRCA	Tumor	8.01
TCGA-E2-A158-11A	BRCA	Normal	7.46
TCGA-E2-A15I-01A	BRCA	Tumor	8.24
TCGA-E2-A15I-11A	BRCA	Normal	7.08
TCGA-E2-A15K-06A	BRCA	Tumor	8.28
TCGA-E2-A15K-11A	BRCA	Normal	6.82
TCGA-E2-A15M-01A	BRCA	Tumor	7.66
TCGA-E2-A15M-11A	BRCA	Normal	7.72
TCGA-E2-A1BC-01A	BRCA	Tumor	8.02
TCGA-E2-A1BC-11A	BRCA	Normal	6.97
TCGA-E2-A1IG-01A	BRCA	Tumor	7.44
TCGA-E2-A1IG-11A	BRCA	Normal	7.48
TCGA-E2-A1L7-01A	BRCA	Tumor	6.75
TCGA-E2-A1L7-11A	BRCA	Normal	7.72
TCGA-E2-A1LB-01A	BRCA	Tumor	8.21
TCGA-E2-A1LB-11A	BRCA	Normal	7.75
TCGA-E2-A1LH-01A	BRCA	Tumor	5.69
TCGA-E2-A1LH-11A	BRCA	Normal	8.29
TCGA-E2-A1LS-01A	BRCA	Tumor	5.73
TCGA-E2-A1LS-11A	BRCA	Normal	6.87

TCGA-E9-A1N4-01A	BRCA	Tumor	8.16
TCGA-E9-A1N4-11A	BRCA	Normal	7.85
TCGA-E9-A1N5-01A	BRCA	Tumor	7.73
TCGA-E9-A1N5-11A	BRCA	Normal	7.57
TCGA-E9-A1N6-01A	BRCA	Tumor	7.05
TCGA-E9-A1N6-11A	BRCA	Normal	7.67
TCGA-E9-A1N9-01A	BRCA	Tumor	7.91
TCGA-E9-A1N9-11A	BRCA	Normal	7.77
TCGA-E9-A1NA-01A	BRCA	Tumor	7.14
TCGA-E9-A1NA-11A	BRCA	Normal	7.44
TCGA-E9-A1ND-01A	BRCA	Tumor	6.09
TCGA-E9-A1ND-11A	BRCA	Normal	7.18
TCGA-E9-A1NF-01A	BRCA	Tumor	7.38
TCGA-E9-A1NF-11A	BRCA	Normal	7.69
TCGA-E9-A1NG-01A	BRCA	Tumor	7.85
TCGA-E9-A1NG-11A	BRCA	Normal	7.68
TCGA-E9-A1R7-01A	BRCA	Tumor	6.63
TCGA-E9-A1R7-11A	BRCA	Normal	7.61
TCGA-E9-A1RB-01A	BRCA	Tumor	7.49
TCGA-E9-A1RB-11A	BRCA	Normal	7.39
TCGA-E9-A1RC-01A	BRCA	Tumor	7.75
TCGA-E9-A1RC-11A	BRCA	Normal	7.14
TCGA-E9-A1RD-01A	BRCA	Tumor	8.31
TCGA-E9-A1RD-11A	BRCA	Normal	7.51
TCGA-E9-A1RF-01A	BRCA	Tumor	6.46
TCGA-E9-A1RF-11A	BRCA	Normal	7.62
TCGA-E9-A1RH-01A	BRCA	Tumor	6.91
TCGA-E9-A1RH-11A	BRCA	Normal	7.17
TCGA-E9-A1RI-01A	BRCA	Tumor	8.29
TCGA-E9-A1RI-11A	BRCA	Normal	7.44
TCGA-GI-A2C8-01A	BRCA	Tumor	7.93
TCGA-GI-A2C8-11A	BRCA	Normal	5.47
TCGA-GI-A2C9-01A	BRCA	Tumor	6.96
TCGA-GI-A2C9-11A	BRCA	Normal	7.9
TCGA-A6-2671-01A	COAD	Tumor	6.16
TCGA-A6-2671-11A	COAD	Normal	6.85
TCGA-A6-2675-01A	COAD	Tumor	7.24
TCGA-A6-2675-11A	COAD	Normal	6.91
TCGA-A6-2678-01A	COAD	Tumor	7.74
TCGA-A6-2678-11A	COAD	Normal	7.1
TCGA-A6-2679-01A	COAD	Tumor	4.96
TCGA-A6-2679-11A	COAD	Normal	6.69
TCGA-A6-2680-01A	COAD	Tumor	6.73
TCGA-A6-2680-11A	COAD	Normal	7.81
TCGA-A6-2682-01A	COAD	Tumor	6.11
TCGA-A6-2682-11A	COAD	Normal	7
TCGA-A6-2683-01A	COAD	Tumor	6.34
TCGA-A6-2683-11A	COAD	Normal	7.24
TCGA-A6-2684-01A	COAD	Tumor	7.03
TCGA-A6-2684-11A	COAD	Normal	6.95
TCGA-A6-2685-01A	COAD	Tumor	6.63
TCGA-A6-2685-11A	COAD	Normal	6.84
TCGA-A6-2686-01A	COAD	Tumor	7.38
TCGA-A6-2686-11A	COAD	Normal	7.05
TCGA-A6-5659-01A	COAD	Tumor	7.32
TCGA-A6-5659-11A	COAD	Normal	7.19
TCGA-A6-5662-01A	COAD	Tumor	7.79
TCGA-A6-5662-11A	COAD	Normal	7.01

TCGA-A6-5665-01A	COAD	Tumor	7.93
TCGA-A6-5665-11A	COAD	Normal	6.97
TCGA-A6-5667-01A	COAD	Tumor	6.94
TCGA-A6-5667-11A	COAD	Normal	6.79
TCGA-AA-3489-01A	COAD	Tumor	7.1
TCGA-AA-3489-11A	COAD	Normal	7.33
TCGA-AA-3496-01A	COAD	Tumor	6.66
TCGA-AA-3496-11A	COAD	Normal	7.41
TCGA-AA-3511-01A	COAD	Tumor	6.66
TCGA-AA-3511-11A	COAD	Normal	6.88
TCGA-AA-3514-01A	COAD	Tumor	6.09
TCGA-AA-3514-11A	COAD	Normal	7.22
TCGA-AA-3516-01A	COAD	Tumor	7.32
TCGA-AA-3516-11A	COAD	Normal	6.77
TCGA-AA-3517-01A	COAD	Tumor	6.65
TCGA-AA-3517-11A	COAD	Normal	6.96
TCGA-AA-3518-01A	COAD	Tumor	7.34
TCGA-AA-3518-11A	COAD	Normal	7.04
TCGA-AA-3520-01A	COAD	Tumor	7.42
TCGA-AA-3520-11A	COAD	Normal	7.14
TCGA-AA-3522-01A	COAD	Tumor	8.08
TCGA-AA-3522-11A	COAD	Normal	7.29
TCGA-AA-3525-01A	COAD	Tumor	6.83
TCGA-AA-3525-11A	COAD	Normal	6.95
TCGA-AA-3527-01A	COAD	Tumor	5.49
TCGA-AA-3527-11A	COAD	Normal	7.55
TCGA-AA-3531-01A	COAD	Tumor	7
TCGA-AA-3531-11A	COAD	Normal	6.87
TCGA-AA-3534-01A	COAD	Tumor	6.84
TCGA-AA-3534-11A	COAD	Normal	6.95
TCGA-AA-3655-01A	COAD	Tumor	7.26
TCGA-AA-3655-11A	COAD	Normal	7.4
TCGA-AA-3660-01A	COAD	Tumor	6.97
TCGA-AA-3660-11A	COAD	Normal	7.08
TCGA-AA-3662-01A	COAD	Tumor	6.64
TCGA-AA-3662-11A	COAD	Normal	7.12
TCGA-AA-3663-01A	COAD	Tumor	7.67
TCGA-AA-3663-11A	COAD	Normal	7.1
TCGA-AA-3697-01A	COAD	Tumor	7.29
TCGA-AA-3697-11A	COAD	Normal	6.97
TCGA-AA-3712-01A	COAD	Tumor	7.43
TCGA-AA-3712-11A	COAD	Normal	6.92
TCGA-AA-3713-01A	COAD	Tumor	7.48
TCGA-AA-3713-11A	COAD	Normal	7.98
TCGA-AZ-6598-01A	COAD	Tumor	7.55
TCGA-AZ-6598-11A	COAD	Normal	6.77
TCGA-AZ-6599-01A	COAD	Tumor	7.25
TCGA-AZ-6599-11A	COAD	Normal	7.19
TCGA-AZ-6600-01A	COAD	Tumor	7.47
TCGA-AZ-6600-11A	COAD	Normal	7.01
TCGA-AZ-6601-01A	COAD	Tumor	7.22
TCGA-AZ-6601-11A	COAD	Normal	6.72
TCGA-AZ-6603-01A	COAD	Tumor	6.85
TCGA-AZ-6603-11A	COAD	Normal	6.72
TCGA-AZ-6605-01A	COAD	Tumor	6.92
TCGA-AZ-6605-11A	COAD	Normal	7.03
TCGA-F4-6704-01A	COAD	Tumor	6.98
TCGA-F4-6704-11A	COAD	Normal	6.96

TCGA-IC-A6RE-01A	ESCA	Tumor	6.68
TCGA-IC-A6RE-11A	ESCA	Normal	6.63
TCGA-IC-A6RF-01A	ESCA	Tumor	8.56
TCGA-IC-A6RF-11A	ESCA	Normal	7.38
TCGA-IG-A3I8-01A	ESCA	Tumor	7.39
TCGA-IG-A3I8-11A	ESCA	Normal	7.54
TCGA-L5-A43C-01A	ESCA	Tumor	6.44
TCGA-L5-A43C-11A	ESCA	Normal	5.97
TCGA-L5-A4OF-01A	ESCA	Tumor	7.69
TCGA-L5-A4OF-11A	ESCA	Normal	6.27
TCGA-L5-A4OG-01A	ESCA	Tumor	6.93
TCGA-L5-A4OG-11A	ESCA	Normal	6.45
TCGA-L5-A4OJ-01A	ESCA	Tumor	6.1
TCGA-L5-A4OJ-11A	ESCA	Normal	6.11
TCGA-L5-A4OM-01A	ESCA	Tumor	7.9
TCGA-L5-A4OM-11A	ESCA	Normal	6.96
TCGA-L5-A4OO-01A	ESCA	Tumor	5.8
TCGA-L5-A4OO-11A	ESCA	Normal	6.63
TCGA-L5-A4OQ-01A	ESCA	Tumor	6.12
TCGA-L5-A4OQ-11A	ESCA	Normal	7.27
TCGA-L5-A4OR-01A	ESCA	Tumor	5.69
TCGA-L5-A4OR-11A	ESCA	Normal	5.84
TCGA-V5-A7RE-01A	ESCA	Tumor	6.36
TCGA-V5-A7RE-11A	ESCA	Normal	6.58
TCGA-V5-AASX-01A	ESCA	Tumor	7.2
TCGA-V5-AASX-11A	ESCA	Normal	7.11
TCGA-CV-6933-01A	HNSC	Tumor	7.08
TCGA-CV-6933-11A	HNSC	Normal	5.76
TCGA-CV-6934-01A	HNSC	Tumor	7.02
TCGA-CV-6934-11A	HNSC	Normal	7.01
TCGA-CV-6935-01A	HNSC	Tumor	7.59
TCGA-CV-6935-11A	HNSC	Normal	6.98
TCGA-CV-6936-01A	HNSC	Tumor	6.26
TCGA-CV-6936-11A	HNSC	Normal	6.8
TCGA-CV-6938-01A	HNSC	Tumor	7.5
TCGA-CV-6938-11A	HNSC	Normal	7.03
TCGA-CV-6939-01A	HNSC	Tumor	7.43
TCGA-CV-6939-11A	HNSC	Normal	5.66
TCGA-CV-6943-01A	HNSC	Tumor	6.97
TCGA-CV-6943-11A	HNSC	Normal	7.16
TCGA-CV-6955-01A	HNSC	Tumor	5.8
TCGA-CV-6955-11A	HNSC	Normal	6.51
TCGA-CV-6956-01A	HNSC	Tumor	8.62
TCGA-CV-6956-11A	HNSC	Normal	6.45
TCGA-CV-6959-01A	HNSC	Tumor	7.96
TCGA-CV-6959-11A	HNSC	Normal	6.56
TCGA-CV-6960-01A	HNSC	Tumor	6.41
TCGA-CV-6960-11A	HNSC	Normal	7.27
TCGA-CV-6961-01A	HNSC	Tumor	6.04
TCGA-CV-6961-11A	HNSC	Normal	6.53
TCGA-CV-6962-01A	HNSC	Tumor	6.96
TCGA-CV-6962-11A	HNSC	Normal	7.05
TCGA-CV-7091-01A	HNSC	Tumor	7.16
TCGA-CV-7091-11A	HNSC	Normal	5.99
TCGA-CV-7097-01A	HNSC	Tumor	7.01
TCGA-CV-7097-11A	HNSC	Normal	6.69
TCGA-CV-7101-01A	HNSC	Tumor	7.12
TCGA-CV-7101-11A	HNSC	Normal	7.68

TCGA-CV-7103-01A	HNSC	Tumor	7.63
TCGA-CV-7103-11A	HNSC	Normal	6.65
TCGA-CV-7177-01A	HNSC	Tumor	7.79
TCGA-CV-7177-11A	HNSC	Normal	7.39
TCGA-CV-7178-01A	HNSC	Tumor	6.85
TCGA-CV-7178-11A	HNSC	Normal	6.81
TCGA-CV-7183-01A	HNSC	Tumor	6.35
TCGA-CV-7183-11A	HNSC	Normal	7.29
TCGA-CV-7235-01A	HNSC	Tumor	7.05
TCGA-CV-7235-11A	HNSC	Normal	7.05
TCGA-CV-7238-01A	HNSC	Tumor	6.45
TCGA-CV-7238-11A	HNSC	Normal	6.59
TCGA-CV-7242-01A	HNSC	Tumor	7.23
TCGA-CV-7242-11A	HNSC	Normal	7.02
TCGA-CV-7245-01A	HNSC	Tumor	6.87
TCGA-CV-7245-11A	HNSC	Normal	6.83
TCGA-CV-7250-01A	HNSC	Tumor	7.95
TCGA-CV-7250-11A	HNSC	Normal	7.12
TCGA-CV-7252-01A	HNSC	Tumor	7.4
TCGA-CV-7252-11A	HNSC	Normal	6.96
TCGA-CV-7255-01A	HNSC	Tumor	7.61
TCGA-CV-7255-11A	HNSC	Normal	7.5
TCGA-CV-7261-01A	HNSC	Tumor	7.86
TCGA-CV-7261-11A	HNSC	Normal	6.98
TCGA-CV-7406-01A	HNSC	Tumor	6.63
TCGA-CV-7406-11A	HNSC	Normal	6.81
TCGA-CV-7416-01A	HNSC	Tumor	7.54
TCGA-CV-7416-11A	HNSC	Normal	6.93
TCGA-CV-7423-01A	HNSC	Tumor	6.75
TCGA-CV-7423-11A	HNSC	Normal	6.99
TCGA-CV-7424-01A	HNSC	Tumor	6.63
TCGA-CV-7424-11A	HNSC	Normal	7.58
TCGA-CV-7425-01A	HNSC	Tumor	6.55
TCGA-CV-7425-11A	HNSC	Normal	6.25
TCGA-CV-7432-01A	HNSC	Tumor	7.7
TCGA-CV-7432-11A	HNSC	Normal	6.57
TCGA-CV-7434-01A	HNSC	Tumor	6.88
TCGA-CV-7434-11A	HNSC	Normal	6.92
TCGA-CV-7437-01A	HNSC	Tumor	7.17
TCGA-CV-7437-11A	HNSC	Normal	7.65
TCGA-CV-7438-01A	HNSC	Tumor	6.88
TCGA-CV-7438-11A	HNSC	Normal	6.89
TCGA-CV-7440-01A	HNSC	Tumor	7.88
TCGA-CV-7440-11A	HNSC	Normal	8.27
TCGA-H7-A6C4-01A	HNSC	Tumor	6.74
TCGA-H7-A6C4-11A	HNSC	Normal	5.76
TCGA-HD-8635-01A	HNSC	Tumor	7.26
TCGA-HD-8635-11A	HNSC	Normal	7.02
TCGA-HD-A6HZ-01A	HNSC	Tumor	7.1
TCGA-HD-A6HZ-11A	HNSC	Normal	5.6
TCGA-HD-A6I0-01A	HNSC	Tumor	5.86
TCGA-HD-A6I0-11A	HNSC	Normal	5.54
TCGA-WA-A7GZ-01A	HNSC	Tumor	6.94
TCGA-WA-A7GZ-11A	HNSC	Normal	6.39
TCGA-KL-8324-01A	KICH	Tumor	7.57
TCGA-KL-8324-11A	KICH	Normal	8.59
TCGA-KL-8326-01A	KICH	Tumor	7.19
TCGA-KL-8326-11A	KICH	Normal	7.92

TCGA-KL-8329-01A	KICH	Tumor	7.36
TCGA-KL-8329-11A	KICH	Normal	8.24
TCGA-KL-8332-01A	KICH	Tumor	7.09
TCGA-KL-8332-11A	KICH	Normal	8.93
TCGA-KL-8336-01A	KICH	Tumor	7.87
TCGA-KL-8336-11A	KICH	Normal	7.46
TCGA-KL-8339-01A	KICH	Tumor	8.38
TCGA-KL-8339-11A	KICH	Normal	8.82
TCGA-KN-8419-01A	KICH	Tumor	7.57
TCGA-KN-8419-11A	KICH	Normal	8.37
TCGA-KN-8422-01A	KICH	Tumor	6.93
TCGA-KN-8422-11A	KICH	Normal	7.59
TCGA-KN-8423-01A	KICH	Tumor	8.33
TCGA-KN-8423-11A	KICH	Normal	7.87
TCGA-KN-8424-01A	KICH	Tumor	7.12
TCGA-KN-8424-11A	KICH	Normal	7.92
TCGA-KN-8425-01A	KICH	Tumor	7.54
TCGA-KN-8425-11A	KICH	Normal	8.36
TCGA-KN-8426-01A	KICH	Tumor	7.27
TCGA-KN-8426-11A	KICH	Normal	7.87
TCGA-KN-8427-01A	KICH	Tumor	8.22
TCGA-KN-8427-11A	KICH	Normal	8.05
TCGA-KN-8428-01A	KICH	Tumor	5.53
TCGA-KN-8428-11A	KICH	Normal	8.31
TCGA-KN-8429-01A	KICH	Tumor	7.34
TCGA-KN-8429-11A	KICH	Normal	8.1
TCGA-KN-8430-01A	KICH	Tumor	7.4
TCGA-KN-8430-11A	KICH	Normal	7.28
TCGA-KN-8431-01A	KICH	Tumor	7.53
TCGA-KN-8431-11A	KICH	Normal	7.9
TCGA-KN-8432-01A	KICH	Tumor	8
TCGA-KN-8432-11A	KICH	Normal	8.36
TCGA-KN-8433-01A	KICH	Tumor	6.17
TCGA-KN-8433-11A	KICH	Normal	8.2
TCGA-KN-8434-01A	KICH	Tumor	6.79
TCGA-KN-8434-11A	KICH	Normal	8.59
TCGA-KN-8435-01A	KICH	Tumor	7.26
TCGA-KN-8435-11A	KICH	Normal	8.5
TCGA-KN-8436-01A	KICH	Tumor	7.27
TCGA-KN-8436-11A	KICH	Normal	7.26
TCGA-KN-8437-01A	KICH	Tumor	6.77
TCGA-KN-8437-11A	KICH	Normal	7.59
TCGA-KO-8403-01A	KICH	Tumor	6.43
TCGA-KO-8403-11A	KICH	Normal	7.47
TCGA-KO-8415-01A	KICH	Tumor	6.91
TCGA-KO-8415-11A	KICH	Normal	8.4
TCGA-A3-3358-01A	KIRC	Tumor	7.94
TCGA-A3-3358-11A	KIRC	Normal	8.44
TCGA-A3-3387-01A	KIRC	Tumor	7.84
TCGA-A3-3387-11A	KIRC	Normal	8.33
TCGA-B0-4700-01A	KIRC	Tumor	6.75
TCGA-B0-4700-11A	KIRC	Normal	7.54
TCGA-B0-4712-01A	KIRC	Tumor	7.26
TCGA-B0-4712-11A	KIRC	Normal	8.13
TCGA-B0-5402-01A	KIRC	Tumor	6.65
TCGA-B0-5402-11A	KIRC	Normal	7.96
TCGA-B0-5690-01A	KIRC	Tumor	7.63
TCGA-B0-5690-11A	KIRC	Normal	8.33

TCGA-B0-5691-01A	KIRC	Tumor	8.39
TCGA-B0-5691-11A	KIRC	Normal	8.2
TCGA-B0-5694-01A	KIRC	Tumor	6.6
TCGA-B0-5694-11A	KIRC	Normal	7.89
TCGA-B0-5696-01A	KIRC	Tumor	8.32
TCGA-B0-5696-11A	KIRC	Normal	8.64
TCGA-B0-5697-01A	KIRC	Tumor	8.1
TCGA-B0-5697-11A	KIRC	Normal	8.27
TCGA-B0-5699-01A	KIRC	Tumor	7.96
TCGA-B0-5699-11A	KIRC	Normal	8.34
TCGA-B0-5701-01A	KIRC	Tumor	7.84
TCGA-B0-5701-11A	KIRC	Normal	7.76
TCGA-B0-5703-01A	KIRC	Tumor	6.58
TCGA-B0-5703-11A	KIRC	Normal	7.99
TCGA-B0-5705-01A	KIRC	Tumor	8.23
TCGA-B0-5705-11A	KIRC	Normal	7.7
TCGA-B0-5706-01A	KIRC	Tumor	6.31
TCGA-B0-5706-11A	KIRC	Normal	7.77
TCGA-B0-5709-01A	KIRC	Tumor	7.93
TCGA-B0-5709-11A	KIRC	Normal	8.59
TCGA-B0-5711-01A	KIRC	Tumor	8.01
TCGA-B0-5711-11A	KIRC	Normal	8.53
TCGA-B0-5712-01A	KIRC	Tumor	9.32
TCGA-B0-5712-11A	KIRC	Normal	7.53
TCGA-B2-5636-01A	KIRC	Tumor	7.42
TCGA-B2-5636-11A	KIRC	Normal	8.17
TCGA-B2-5641-01A	KIRC	Tumor	7.53
TCGA-B2-5641-11A	KIRC	Normal	7.53
TCGA-B8-4619-01A	KIRC	Tumor	8.81
TCGA-B8-4619-11A	KIRC	Normal	8.95
TCGA-B8-4620-01A	KIRC	Tumor	7.02
TCGA-B8-4620-11A	KIRC	Normal	7.53
TCGA-B8-4622-01A	KIRC	Tumor	7.49
TCGA-B8-4622-11A	KIRC	Normal	8.31
TCGA-B8-5549-01A	KIRC	Tumor	7.92
TCGA-B8-5549-11A	KIRC	Normal	7.83
TCGA-B8-5552-01B	KIRC	Tumor	7.8
TCGA-B8-5552-11A	KIRC	Normal	7.78
TCGA-CJ-5672-01A	KIRC	Tumor	8.15
TCGA-CJ-5672-11A	KIRC	Normal	8
TCGA-CJ-5676-01A	KIRC	Tumor	6.64
TCGA-CJ-5676-11A	KIRC	Normal	8.18
TCGA-CJ-5677-01A	KIRC	Tumor	7.78
TCGA-CJ-5677-11A	KIRC	Normal	8.53
TCGA-CJ-5678-01A	KIRC	Tumor	7.25
TCGA-CJ-5678-11A	KIRC	Normal	7.73
TCGA-CJ-5679-01A	KIRC	Tumor	6.85
TCGA-CJ-5679-11A	KIRC	Normal	8.02
TCGA-CJ-5680-01A	KIRC	Tumor	7.52
TCGA-CJ-5680-11A	KIRC	Normal	7.87
TCGA-CJ-5681-01A	KIRC	Tumor	7.41
TCGA-CJ-5681-11A	KIRC	Normal	8.07
TCGA-CJ-5689-01A	KIRC	Tumor	7.28
TCGA-CJ-5689-11A	KIRC	Normal	7.96
TCGA-CJ-6030-01A	KIRC	Tumor	8.18
TCGA-CJ-6030-11A	KIRC	Normal	8.5
TCGA-CJ-6033-01A	KIRC	Tumor	8.06
TCGA-CJ-6033-11A	KIRC	Normal	7.5

TCGA-CW-5580-01A	KIRC	Tumor	8.34
TCGA-CW-5580-11A	KIRC	Normal	7.97
TCGA-CW-5581-01A	KIRC	Tumor	8.27
TCGA-CW-5581-11A	KIRC	Normal	8.58
TCGA-CW-5584-01A	KIRC	Tumor	8.62
TCGA-CW-5584-11A	KIRC	Normal	7.74
TCGA-CW-5585-01A	KIRC	Tumor	7.69
TCGA-CW-5585-11A	KIRC	Normal	8.72
TCGA-CW-5587-01A	KIRC	Tumor	8.78
TCGA-CW-5587-11A	KIRC	Normal	8.95
TCGA-CW-5589-01A	KIRC	Tumor	7.78
TCGA-CW-5589-11A	KIRC	Normal	8.43
TCGA-CW-5591-01A	KIRC	Tumor	7.86
TCGA-CW-5591-11A	KIRC	Normal	7.34
TCGA-CW-6087-01A	KIRC	Tumor	7.45
TCGA-CW-6087-11A	KIRC	Normal	7.68
TCGA-CW-6088-01A	KIRC	Tumor	8.59
TCGA-CW-6088-11A	KIRC	Normal	7.9
TCGA-CW-6090-01A	KIRC	Tumor	7.41
TCGA-CW-6090-11A	KIRC	Normal	8.65
TCGA-CZ-4863-01A	KIRC	Tumor	9.44
TCGA-CZ-4863-11A	KIRC	Normal	7.69
TCGA-CZ-4864-01A	KIRC	Tumor	7.93
TCGA-CZ-4864-11A	KIRC	Normal	8.22
TCGA-CZ-4865-01A	KIRC	Tumor	8.07
TCGA-CZ-4865-11A	KIRC	Normal	7.88
TCGA-CZ-5451-01A	KIRC	Tumor	8.28
TCGA-CZ-5451-11A	KIRC	Normal	8.64
TCGA-CZ-5452-01A	KIRC	Tumor	7.38
TCGA-CZ-5452-11A	KIRC	Normal	8.06
TCGA-CZ-5453-01A	KIRC	Tumor	8.21
TCGA-CZ-5453-11A	KIRC	Normal	7.91
TCGA-CZ-5454-01A	KIRC	Tumor	7.11
TCGA-CZ-5454-11A	KIRC	Normal	8.15
TCGA-CZ-5455-01A	KIRC	Tumor	8.32
TCGA-CZ-5455-11A	KIRC	Normal	7.55
TCGA-CZ-5456-01A	KIRC	Tumor	7.27
TCGA-CZ-5456-11A	KIRC	Normal	7.55
TCGA-CZ-5457-01A	KIRC	Tumor	8.27
TCGA-CZ-5457-11A	KIRC	Normal	7.34
TCGA-CZ-5458-01A	KIRC	Tumor	7.45
TCGA-CZ-5458-11A	KIRC	Normal	7.87
TCGA-CZ-5461-01A	KIRC	Tumor	8.22
TCGA-CZ-5461-11A	KIRC	Normal	8.44
TCGA-CZ-5462-01A	KIRC	Tumor	8.19
TCGA-CZ-5462-11A	KIRC	Normal	8.24
TCGA-CZ-5463-01A	KIRC	Tumor	9.02
TCGA-CZ-5463-11A	KIRC	Normal	8.57
TCGA-CZ-5465-01A	KIRC	Tumor	7.65
TCGA-CZ-5465-11A	KIRC	Normal	7.99
TCGA-CZ-5466-01A	KIRC	Tumor	7.34
TCGA-CZ-5466-11A	KIRC	Normal	8.49
TCGA-CZ-5467-01A	KIRC	Tumor	7.87
TCGA-CZ-5467-11A	KIRC	Normal	7.45
TCGA-CZ-5468-01A	KIRC	Tumor	7.37
TCGA-CZ-5468-11A	KIRC	Normal	7.76
TCGA-CZ-5469-01A	KIRC	Tumor	7.07
TCGA-CZ-5469-11A	KIRC	Normal	7.83

TCGA-CZ-5470-01A	KIRC	Tumor	7.29
TCGA-CZ-5470-11A	KIRC	Normal	7.91
TCGA-CZ-5982-01A	KIRC	Tumor	7.52
TCGA-CZ-5982-11A	KIRC	Normal	8.18
TCGA-CZ-5984-01A	KIRC	Tumor	7.77
TCGA-CZ-5984-11A	KIRC	Normal	8.03
TCGA-CZ-5985-01A	KIRC	Tumor	7.97
TCGA-CZ-5985-11A	KIRC	Normal	7.88
TCGA-CZ-5986-01A	KIRC	Tumor	8.21
TCGA-CZ-5986-11A	KIRC	Normal	8.16
TCGA-CZ-5987-01A	KIRC	Tumor	7.69
TCGA-CZ-5987-11A	KIRC	Normal	8.05
TCGA-CZ-5988-01A	KIRC	Tumor	8.08
TCGA-CZ-5988-11A	KIRC	Normal	8.11
TCGA-CZ-5989-01A	KIRC	Tumor	8.84
TCGA-CZ-5989-11A	KIRC	Normal	7.55
TCGA-A4-A4ZT-01A	KIRP	Tumor	9.08
TCGA-A4-A4ZT-11A	KIRP	Normal	7.62
TCGA-A4-A57E-01A	KIRP	Tumor	6.74
TCGA-A4-A57E-11A	KIRP	Normal	7.21
TCGA-B9-4115-01A	KIRP	Tumor	8.55
TCGA-B9-4115-11A	KIRP	Normal	8.24
TCGA-BQ-5875-01A	KIRP	Tumor	7.18
TCGA-BQ-5875-11A	KIRP	Normal	7.91
TCGA-BQ-5877-01A	KIRP	Tumor	7.54
TCGA-BQ-5877-11A	KIRP	Normal	8.35
TCGA-BQ-5878-01A	KIRP	Tumor	9.01
TCGA-BQ-5878-11A	KIRP	Normal	8.71
TCGA-BQ-5879-01A	KIRP	Tumor	7.42
TCGA-BQ-5879-11A	KIRP	Normal	8.63
TCGA-BQ-5882-01A	KIRP	Tumor	6.82
TCGA-BQ-5882-11A	KIRP	Normal	8.7
TCGA-BQ-5884-01A	KIRP	Tumor	7.9
TCGA-BQ-5884-11A	KIRP	Normal	8.39
TCGA-BQ-5887-01A	KIRP	Tumor	6.54
TCGA-BQ-5887-11A	KIRP	Normal	7.68
TCGA-BQ-5888-01A	KIRP	Tumor	6.46
TCGA-BQ-5888-11A	KIRP	Normal	7.45
TCGA-BQ-5890-01A	KIRP	Tumor	7.03
TCGA-BQ-5890-11A	KIRP	Normal	8.28
TCGA-BQ-5891-01A	KIRP	Tumor	8.25
TCGA-BQ-5891-11A	KIRP	Normal	8.32
TCGA-BQ-5894-01A	KIRP	Tumor	7.53
TCGA-BQ-5894-11A	KIRP	Normal	9.01
TCGA-BQ-7044-01A	KIRP	Tumor	6.85
TCGA-BQ-7044-11A	KIRP	Normal	8.25
TCGA-BQ-7045-01A	KIRP	Tumor	7.2
TCGA-BQ-7045-11A	KIRP	Normal	8.26
TCGA-BQ-7046-01A	KIRP	Tumor	7.16
TCGA-BQ-7046-11A	KIRP	Normal	8.23
TCGA-BQ-7051-01A	KIRP	Tumor	8.98
TCGA-BQ-7051-11A	KIRP	Normal	7.87
TCGA-BQ-7055-01A	KIRP	Tumor	7.64
TCGA-BQ-7055-11A	KIRP	Normal	7.78
TCGA-BQ-7059-01A	KIRP	Tumor	7.09
TCGA-BQ-7059-11A	KIRP	Normal	8.28
TCGA-BQ-7061-01A	KIRP	Tumor	7.49
TCGA-BQ-7061-11A	KIRP	Normal	7.97

TCGA-DZ-6131-01A	KIRP	Tumor	6.95
TCGA-DZ-6131-11A	KIRP	Normal	7.36
TCGA-DZ-6132-01A	KIRP	Tumor	7.68
TCGA-DZ-6132-11A	KIRP	Normal	7.69
TCGA-DZ-6133-01A	KIRP	Tumor	9.52
TCGA-DZ-6133-11A	KIRP	Normal	7.93
TCGA-DZ-6134-01A	KIRP	Tumor	7.52
TCGA-DZ-6134-11A	KIRP	Normal	7.84
TCGA-GL-6846-01A	KIRP	Tumor	6.63
TCGA-GL-6846-11A	KIRP	Normal	7.89
TCGA-GL-7966-01A	KIRP	Tumor	6.81
TCGA-GL-7966-11A	KIRP	Normal	7.92
TCGA-GL-A59R-01A	KIRP	Tumor	9.46
TCGA-GL-A59R-11A	KIRP	Normal	7.72
TCGA-GL-A9DE-01A	KIRP	Tumor	7.22
TCGA-GL-A9DE-11A	KIRP	Normal	7.64
TCGA-P4-A5E8-01A	KIRP	Tumor	7.62
TCGA-P4-A5E8-11A	KIRP	Normal	7.07
TCGA-P4-A5ED-01A	KIRP	Tumor	6.7
TCGA-P4-A5ED-11A	KIRP	Normal	7.82
TCGA-Y8-A8RY-01A	KIRP	Tumor	7.94
TCGA-Y8-A8RY-11A	KIRP	Normal	7.64
TCGA-BC-A10Q-01A	LIHC	Tumor	6.78
TCGA-BC-A10Q-11A	LIHC	Normal	9.06
TCGA-BC-A10R-01A	LIHC	Tumor	6.66
TCGA-BC-A10R-11A	LIHC	Normal	8.85
TCGA-BC-A10T-01A	LIHC	Tumor	7.59
TCGA-BC-A10T-11A	LIHC	Normal	8.46
TCGA-BC-A10U-01A	LIHC	Tumor	7.72
TCGA-BC-A10U-11A	LIHC	Normal	8.74
TCGA-BC-A10W-01A	LIHC	Tumor	7.38
TCGA-BC-A10W-11A	LIHC	Normal	8.78
TCGA-BC-A10X-01A	LIHC	Tumor	9.52
TCGA-BC-A10X-11A	LIHC	Normal	9.34
TCGA-BC-A10Y-01A	LIHC	Tumor	5.72
TCGA-BC-A10Y-11A	LIHC	Normal	8.61
TCGA-BC-A10Z-01A	LIHC	Tumor	8.69
TCGA-BC-A10Z-11A	LIHC	Normal	7.98
TCGA-BC-A110-01A	LIHC	Tumor	8.07
TCGA-BC-A110-11A	LIHC	Normal	8.62
TCGA-BC-A216-01A	LIHC	Tumor	7.46
TCGA-BC-A216-11A	LIHC	Normal	7.7
TCGA-BD-A2L6-01A	LIHC	Tumor	6.84
TCGA-BD-A2L6-11A	LIHC	Normal	7.72
TCGA-BD-A3EP-01A	LIHC	Tumor	6.9
TCGA-BD-A3EP-11A	LIHC	Normal	8.95
TCGA-DD-A113-01A	LIHC	Tumor	7.17
TCGA-DD-A113-11A	LIHC	Normal	8.69
TCGA-DD-A114-01A	LIHC	Tumor	7.25
TCGA-DD-A114-11A	LIHC	Normal	8.95
TCGA-DD-A116-01A	LIHC	Tumor	8.96
TCGA-DD-A116-11A	LIHC	Normal	8.6
TCGA-DD-A118-01A	LIHC	Tumor	6.83
TCGA-DD-A118-11A	LIHC	Normal	8.95
TCGA-DD-A119-01A	LIHC	Tumor	7.05
TCGA-DD-A119-11A	LIHC	Normal	8.56
TCGA-DD-A11A-01A	LIHC	Tumor	8.17
TCGA-DD-A11A-11A	LIHC	Normal	8.58

TCGA-DD-A11B-01A	LIHC	Tumor	9.65
TCGA-DD-A11B-11A	LIHC	Normal	8.42
TCGA-DD-A11C-01A	LIHC	Tumor	7.56
TCGA-DD-A11C-11A	LIHC	Normal	9.2
TCGA-DD-A11D-01A	LIHC	Tumor	7.49
TCGA-DD-A11D-11A	LIHC	Normal	8.98
TCGA-DD-A1EB-01A	LIHC	Tumor	6.99
TCGA-DD-A1EB-11A	LIHC	Normal	9.24
TCGA-DD-A1EC-01A	LIHC	Tumor	7.58
TCGA-DD-A1EC-11A	LIHC	Normal	8.62
TCGA-DD-A1EE-01A	LIHC	Tumor	7.83
TCGA-DD-A1EE-11A	LIHC	Normal	8.36
TCGA-DD-A1EG-01A	LIHC	Tumor	7.46
TCGA-DD-A1EG-11A	LIHC	Normal	8.61
TCGA-DD-A1EH-01A	LIHC	Tumor	9.49
TCGA-DD-A1EH-11A	LIHC	Normal	9.04
TCGA-DD-A1EI-01A	LIHC	Tumor	9.28
TCGA-DD-A1EI-11A	LIHC	Normal	9.5
TCGA-DD-A1EJ-01A	LIHC	Tumor	10.11
TCGA-DD-A1EJ-11A	LIHC	Normal	8.75
TCGA-DD-A1EL-01A	LIHC	Tumor	8.96
TCGA-DD-A1EL-11A	LIHC	Normal	9.21
TCGA-DD-A39V-01A	LIHC	Tumor	8.54
TCGA-DD-A39V-11A	LIHC	Normal	8.68
TCGA-DD-A39W-01A	LIHC	Tumor	11.12
TCGA-DD-A39W-11A	LIHC	Normal	9.03
TCGA-DD-A39X-01A	LIHC	Tumor	7.63
TCGA-DD-A39X-11A	LIHC	Normal	9.32
TCGA-DD-A39Z-01A	LIHC	Tumor	7.69
TCGA-DD-A39Z-11A	LIHC	Normal	8.07
TCGA-DD-A3A1-01A	LIHC	Tumor	7.26
TCGA-DD-A3A1-11A	LIHC	Normal	8.21
TCGA-DD-A3A2-01A	LIHC	Tumor	7.09
TCGA-DD-A3A2-11A	LIHC	Normal	8.63
TCGA-DD-A3A3-01A	LIHC	Tumor	8.1
TCGA-DD-A3A3-11A	LIHC	Normal	7.72
TCGA-DD-A3A4-01A	LIHC	Tumor	7.78
TCGA-DD-A3A4-11A	LIHC	Normal	8.59
TCGA-DD-A3A5-01A	LIHC	Tumor	7.17
TCGA-DD-A3A5-11A	LIHC	Normal	8.8
TCGA-DD-A3A6-01A	LIHC	Tumor	5.89
TCGA-DD-A3A6-11A	LIHC	Normal	8.13
TCGA-DD-A3A8-01A	LIHC	Tumor	9.25
TCGA-DD-A3A8-11A	LIHC	Normal	8.49
TCGA-EP-A12J-01A	LIHC	Tumor	10.12
TCGA-EP-A12J-11A	LIHC	Normal	9.17
TCGA-EP-A26S-01A	LIHC	Tumor	9.14
TCGA-EP-A26S-11A	LIHC	Normal	9.08
TCGA-EP-A3RK-01A	LIHC	Tumor	8.22
TCGA-EP-A3RK-11A	LIHC	Normal	9.07
TCGA-ES-A2HT-01A	LIHC	Tumor	7.74
TCGA-ES-A2HT-11A	LIHC	Normal	8.58
TCGA-FV-A23B-01A	LIHC	Tumor	9.46
TCGA-FV-A23B-11A	LIHC	Normal	9.1
TCGA-FV-A2QR-01A	LIHC	Tumor	7.2
TCGA-FV-A2QR-11A	LIHC	Normal	7.87
TCGA-FV-A3I0-01A	LIHC	Tumor	6.55
TCGA-FV-A3I0-11A	LIHC	Normal	8.68

TCGA-FV-A3I1-01A	LIHC	Tumor	8.69
TCGA-FV-A3I1-11A	LIHC	Normal	8.96
TCGA-FV-A3R2-01A	LIHC	Tumor	8.33
TCGA-FV-A3R2-11A	LIHC	Normal	7.91
TCGA-G3-A3CH-01A	LIHC	Tumor	7.82
TCGA-G3-A3CH-11A	LIHC	Normal	9.17
TCGA-38-4625-01A	LUAD	Tumor	7.46
TCGA-38-4625-11A	LUAD	Normal	7.01
TCGA-38-4626-01A	LUAD	Tumor	6.86
TCGA-38-4626-11A	LUAD	Normal	7.24
TCGA-38-4627-01A	LUAD	Tumor	7.31
TCGA-38-4627-11A	LUAD	Normal	7.62
TCGA-38-4632-01A	LUAD	Tumor	7.82
TCGA-38-4632-11A	LUAD	Normal	7.09
TCGA-44-2655-01A	LUAD	Tumor	7.43
TCGA-44-2655-11A	LUAD	Normal	7.18
TCGA-44-2657-01A	LUAD	Tumor	6.02
TCGA-44-2657-11A	LUAD	Normal	6.66
TCGA-44-2661-01A	LUAD	Tumor	6.38
TCGA-44-2661-11A	LUAD	Normal	7.02
TCGA-44-2662-01B	LUAD	Tumor	6.71
TCGA-44-2662-11A	LUAD	Normal	6.87
TCGA-44-2665-01B	LUAD	Tumor	6.83
TCGA-44-2665-11A	LUAD	Normal	6.74
TCGA-44-2668-01B	LUAD	Tumor	6.95
TCGA-44-2668-11A	LUAD	Normal	7.03
TCGA-44-3396-01A	LUAD	Tumor	6.11
TCGA-44-3396-11A	LUAD	Normal	7.18
TCGA-44-3398-01A	LUAD	Tumor	6.7
TCGA-44-3398-11B	LUAD	Normal	7.2
TCGA-44-5645-01A	LUAD	Tumor	7
TCGA-44-5645-11A	LUAD	Normal	7.11
TCGA-44-6145-01A	LUAD	Tumor	7.1
TCGA-44-6145-11A	LUAD	Normal	6.88
TCGA-44-6146-01A	LUAD	Tumor	7.46
TCGA-44-6146-11A	LUAD	Normal	7.33
TCGA-44-6147-01A	LUAD	Tumor	6.85
TCGA-44-6147-11A	LUAD	Normal	7.15
TCGA-44-6148-01A	LUAD	Tumor	6.82
TCGA-44-6148-11A	LUAD	Normal	7.14
TCGA-44-6776-01A	LUAD	Tumor	7.63
TCGA-44-6776-11A	LUAD	Normal	7.04
TCGA-44-6777-01A	LUAD	Tumor	6.68
TCGA-44-6777-11A	LUAD	Normal	6.95
TCGA-44-6778-01A	LUAD	Tumor	6.59
TCGA-44-6778-11A	LUAD	Normal	7.11
TCGA-49-4490-01A	LUAD	Tumor	7.22
TCGA-49-4490-11A	LUAD	Normal	7.61
TCGA-49-4512-01A	LUAD	Tumor	6.24
TCGA-49-4512-11A	LUAD	Normal	7.38
TCGA-49-6742-01A	LUAD	Tumor	6.64
TCGA-49-6742-11A	LUAD	Normal	6.84
TCGA-49-6743-01A	LUAD	Tumor	7.09
TCGA-49-6743-11A	LUAD	Normal	7.31
TCGA-49-6744-01A	LUAD	Tumor	6.74
TCGA-49-6744-11A	LUAD	Normal	7.37
TCGA-49-6745-01A	LUAD	Tumor	7.53
TCGA-49-6745-11A	LUAD	Normal	7.43

TCGA-49-6761-01A	LUAD	Tumor	7.01
TCGA-49-6761-11A	LUAD	Normal	7.06
TCGA-50-5930-01A	LUAD	Tumor	6.24
TCGA-50-5930-11A	LUAD	Normal	6.95
TCGA-50-5931-01A	LUAD	Tumor	6.67
TCGA-50-5931-11A	LUAD	Normal	6.95
TCGA-50-5932-01A	LUAD	Tumor	7.36
TCGA-50-5932-11A	LUAD	Normal	7.53
TCGA-50-5933-01A	LUAD	Tumor	7.17
TCGA-50-5933-11A	LUAD	Normal	7.26
TCGA-50-5935-01A	LUAD	Tumor	6.69
TCGA-50-5935-11A	LUAD	Normal	7.18
TCGA-50-5936-01A	LUAD	Tumor	7.27
TCGA-50-5936-11A	LUAD	Normal	7.01
TCGA-50-5939-01A	LUAD	Tumor	6.9
TCGA-50-5939-11A	LUAD	Normal	6.93
TCGA-50-6595-01A	LUAD	Tumor	7.48
TCGA-50-6595-11A	LUAD	Normal	7.23
TCGA-55-6968-01A	LUAD	Tumor	5.92
TCGA-55-6968-11A	LUAD	Normal	6.58
TCGA-55-6969-01A	LUAD	Tumor	6.09
TCGA-55-6969-11A	LUAD	Normal	6.74
TCGA-55-6970-01A	LUAD	Tumor	7.26
TCGA-55-6970-11A	LUAD	Normal	7.11
TCGA-55-6971-01A	LUAD	Tumor	5.92
TCGA-55-6971-11A	LUAD	Normal	6.81
TCGA-55-6972-01A	LUAD	Tumor	7.38
TCGA-55-6972-11A	LUAD	Normal	7.06
TCGA-55-6975-01A	LUAD	Tumor	6.64
TCGA-55-6975-11A	LUAD	Normal	6.82
TCGA-55-6978-01A	LUAD	Tumor	7.79
TCGA-55-6978-11A	LUAD	Normal	7.25
TCGA-55-6979-01A	LUAD	Tumor	6.47
TCGA-55-6979-11A	LUAD	Normal	6.64
TCGA-55-6980-01A	LUAD	Tumor	6.77
TCGA-55-6980-11A	LUAD	Normal	6.89
TCGA-55-6981-01A	LUAD	Tumor	6.27
TCGA-55-6981-11A	LUAD	Normal	6.83
TCGA-55-6982-01A	LUAD	Tumor	6.67
TCGA-55-6982-11A	LUAD	Normal	7.16
TCGA-55-6983-01A	LUAD	Tumor	6.86
TCGA-55-6983-11A	LUAD	Normal	6.93
TCGA-55-6984-01A	LUAD	Tumor	6.91
TCGA-55-6984-11A	LUAD	Normal	6.96
TCGA-55-6985-01A	LUAD	Tumor	6.63
TCGA-55-6985-11A	LUAD	Normal	6.95
TCGA-55-6986-01A	LUAD	Tumor	7.09
TCGA-55-6986-11A	LUAD	Normal	7.07
TCGA-73-4676-01A	LUAD	Tumor	7.12
TCGA-73-4676-11A	LUAD	Normal	7.28
TCGA-91-6828-01A	LUAD	Tumor	6.93
TCGA-91-6828-11A	LUAD	Normal	7.1
TCGA-91-6829-01A	LUAD	Tumor	7.11
TCGA-91-6829-11A	LUAD	Normal	6.85
TCGA-91-6831-01A	LUAD	Tumor	6.61
TCGA-91-6831-11A	LUAD	Normal	7.26
TCGA-91-6835-01A	LUAD	Tumor	6.76
TCGA-91-6835-11A	LUAD	Normal	7.25

TCGA-91-6836-01A	LUAD	Tumor	6.85
TCGA-91-6836-11A	LUAD	Normal	6.88
TCGA-91-6847-01A	LUAD	Tumor	6.47
TCGA-91-6847-11A	LUAD	Normal	7.01
TCGA-91-6849-01A	LUAD	Tumor	6.84
TCGA-91-6849-11A	LUAD	Normal	7.23
TCGA-22-4593-01A	LUSC	Tumor	7.37
TCGA-22-4593-11A	LUSC	Normal	7.29
TCGA-22-4609-01A	LUSC	Tumor	6.68
TCGA-22-4609-11A	LUSC	Normal	6.86
TCGA-22-5471-01A	LUSC	Tumor	7.12
TCGA-22-5471-11A	LUSC	Normal	7.13
TCGA-22-5472-01A	LUSC	Tumor	8.2
TCGA-22-5472-11A	LUSC	Normal	7.51
TCGA-22-5478-01A	LUSC	Tumor	6.72
TCGA-22-5478-11A	LUSC	Normal	7.17
TCGA-22-5481-01A	LUSC	Tumor	6.31
TCGA-22-5481-11A	LUSC	Normal	6.61
TCGA-22-5482-01A	LUSC	Tumor	7.96
TCGA-22-5482-11A	LUSC	Normal	7.47
TCGA-22-5483-01A	LUSC	Tumor	6.8
TCGA-22-5483-11A	LUSC	Normal	6.88
TCGA-22-5489-01A	LUSC	Tumor	5.84
TCGA-22-5489-11A	LUSC	Normal	6.94
TCGA-22-5491-01A	LUSC	Tumor	8.07
TCGA-22-5491-11A	LUSC	Normal	6.65
TCGA-33-4587-01A	LUSC	Tumor	5.6
TCGA-33-4587-11A	LUSC	Normal	7.35
TCGA-33-6737-01A	LUSC	Tumor	6.39
TCGA-33-6737-11A	LUSC	Normal	7.67
TCGA-34-7107-01A	LUSC	Tumor	7.51
TCGA-34-7107-11A	LUSC	Normal	6.19
TCGA-34-8454-01A	LUSC	Tumor	7.26
TCGA-34-8454-11A	LUSC	Normal	6.96
TCGA-39-5040-01A	LUSC	Tumor	8.08
TCGA-39-5040-11A	LUSC	Normal	7.44
TCGA-43-3394-01A	LUSC	Tumor	7.56
TCGA-43-3394-11A	LUSC	Normal	6.81
TCGA-43-5670-01A	LUSC	Tumor	6.99
TCGA-43-5670-11A	LUSC	Normal	7.14
TCGA-43-6143-01A	LUSC	Tumor	7.03
TCGA-43-6143-11A	LUSC	Normal	6.35
TCGA-43-6647-01A	LUSC	Tumor	6.73
TCGA-43-6647-11A	LUSC	Normal	7.09
TCGA-43-6771-01A	LUSC	Tumor	6.71
TCGA-43-6771-11A	LUSC	Normal	7.1
TCGA-43-6773-01A	LUSC	Tumor	6.08
TCGA-43-6773-11A	LUSC	Normal	7.11
TCGA-43-7657-01A	LUSC	Tumor	7.47
TCGA-43-7657-11A	LUSC	Normal	7
TCGA-43-7658-01A	LUSC	Tumor	5.86
TCGA-43-7658-11A	LUSC	Normal	6.97
TCGA-51-4079-01A	LUSC	Tumor	7.18
TCGA-51-4079-11A	LUSC	Normal	6.79
TCGA-51-4080-01A	LUSC	Tumor	7.87
TCGA-51-4080-11A	LUSC	Normal	7.06
TCGA-51-4081-01A	LUSC	Tumor	7.68
TCGA-51-4081-11A	LUSC	Normal	6.36

TCGA-56-7222-01A	LUSC	Tumor	7.81
TCGA-56-7222-11A	LUSC	Normal	7.01
TCGA-56-7579-01A	LUSC	Tumor	7.22
TCGA-56-7579-11A	LUSC	Normal	7.29
TCGA-56-7580-01A	LUSC	Tumor	6.41
TCGA-56-7580-11A	LUSC	Normal	7.08
TCGA-56-7582-01A	LUSC	Tumor	7.24
TCGA-56-7582-11A	LUSC	Normal	7.23
TCGA-56-7730-01A	LUSC	Tumor	7.24
TCGA-56-7730-11A	LUSC	Normal	6.65
TCGA-56-7731-01A	LUSC	Tumor	6.17
TCGA-56-7731-11A	LUSC	Normal	6.91
TCGA-56-7823-01B	LUSC	Tumor	6.77
TCGA-56-7823-11A	LUSC	Normal	6.98
TCGA-56-8082-01A	LUSC	Tumor	6.92
TCGA-56-8082-11A	LUSC	Normal	7.44
TCGA-56-8083-01A	LUSC	Tumor	7.13
TCGA-56-8083-11A	LUSC	Normal	6.95
TCGA-56-8201-01A	LUSC	Tumor	7.44
TCGA-56-8201-11A	LUSC	Normal	6.68
TCGA-56-8309-01A	LUSC	Tumor	6.24
TCGA-56-8309-11A	LUSC	Normal	7.01
TCGA-56-8623-01A	LUSC	Tumor	6.76
TCGA-56-8623-11A	LUSC	Normal	6.73
TCGA-58-8386-01A	LUSC	Tumor	7.07
TCGA-58-8386-11A	LUSC	Normal	6.47
TCGA-60-2709-01A	LUSC	Tumor	6.68
TCGA-60-2709-11A	LUSC	Normal	7.51
TCGA-77-7138-01A	LUSC	Tumor	7.3
TCGA-77-7138-11A	LUSC	Normal	7.08
TCGA-77-7142-01A	LUSC	Tumor	8.09
TCGA-77-7142-11A	LUSC	Normal	7.28
TCGA-77-7335-01A	LUSC	Tumor	7.66
TCGA-77-7335-11A	LUSC	Normal	7.86
TCGA-77-7337-01A	LUSC	Tumor	6.81
TCGA-77-7337-11A	LUSC	Normal	7.4
TCGA-77-7338-01A	LUSC	Tumor	7.48
TCGA-77-7338-11A	LUSC	Normal	7.59
TCGA-77-8007-01A	LUSC	Tumor	7.83
TCGA-77-8007-11A	LUSC	Normal	7.17
TCGA-77-8008-01A	LUSC	Tumor	6.66
TCGA-77-8008-11A	LUSC	Normal	6.94
TCGA-85-7710-01A	LUSC	Tumor	7.18
TCGA-85-7710-11A	LUSC	Normal	6.96
TCGA-90-6837-01A	LUSC	Tumor	6.53
TCGA-90-6837-11A	LUSC	Normal	6.99
TCGA-90-7767-01A	LUSC	Tumor	7.19
TCGA-90-7767-11A	LUSC	Normal	7.19
TCGA-92-7340-01A	LUSC	Tumor	6.84
TCGA-92-7340-11A	LUSC	Normal	7.38
TCGA-CH-5761-01A	PRAD	Tumor	9.42
TCGA-CH-5761-11A	PRAD	Normal	8.18
TCGA-CH-5767-01A	PRAD	Tumor	9.11
TCGA-CH-5767-11B	PRAD	Normal	7.56
TCGA-CH-5768-01A	PRAD	Tumor	8.66
TCGA-CH-5768-11A	PRAD	Normal	8.68
TCGA-CH-5769-01A	PRAD	Tumor	7.36
TCGA-CH-5769-11A	PRAD	Normal	7.34

TCGA-EJ-7115-01A	PRAD	Tumor	8.76
TCGA-EJ-7115-11A	PRAD	Normal	8.69
TCGA-EJ-7123-01A	PRAD	Tumor	8.91
TCGA-EJ-7123-11A	PRAD	Normal	7.75
TCGA-EJ-7125-01A	PRAD	Tumor	9.08
TCGA-EJ-7125-11A	PRAD	Normal	9.11
TCGA-EJ-7314-01A	PRAD	Tumor	8.3
TCGA-EJ-7314-11A	PRAD	Normal	8.57
TCGA-EJ-7315-01A	PRAD	Tumor	7.43
TCGA-EJ-7315-11A	PRAD	Normal	8.39
TCGA-EJ-7317-01A	PRAD	Tumor	8.69
TCGA-EJ-7317-11A	PRAD	Normal	9.22
TCGA-EJ-7321-01A	PRAD	Tumor	8.69
TCGA-EJ-7321-11A	PRAD	Normal	8.59
TCGA-EJ-7327-01A	PRAD	Tumor	8.2
TCGA-EJ-7327-11A	PRAD	Normal	9.32
TCGA-EJ-7328-01A	PRAD	Tumor	7.91
TCGA-EJ-7328-11A	PRAD	Normal	8.2
TCGA-EJ-7330-01A	PRAD	Tumor	8.86
TCGA-EJ-7330-11A	PRAD	Normal	7.98
TCGA-EJ-7331-01A	PRAD	Tumor	9.09
TCGA-EJ-7331-11A	PRAD	Normal	8.77
TCGA-EJ-7781-01A	PRAD	Tumor	9.11
TCGA-EJ-7781-11A	PRAD	Normal	8.26
TCGA-EJ-7782-01A	PRAD	Tumor	9.35
TCGA-EJ-7782-11A	PRAD	Normal	9.12
TCGA-EJ-7783-01A	PRAD	Tumor	8.54
TCGA-EJ-7783-11A	PRAD	Normal	7.86
TCGA-EJ-7784-01A	PRAD	Tumor	8.88
TCGA-EJ-7784-11A	PRAD	Normal	8.76
TCGA-EJ-7785-01A	PRAD	Tumor	8.65
TCGA-EJ-7785-11A	PRAD	Normal	8.97
TCGA-EJ-7786-01A	PRAD	Tumor	9.08
TCGA-EJ-7786-11A	PRAD	Normal	8.36
TCGA-EJ-7789-01A	PRAD	Tumor	8.95
TCGA-EJ-7789-11A	PRAD	Normal	8.98
TCGA-EJ-7792-01A	PRAD	Tumor	8.48
TCGA-EJ-7792-11A	PRAD	Normal	8.77
TCGA-EJ-7793-01A	PRAD	Tumor	8.78
TCGA-EJ-7793-11A	PRAD	Normal	8.69
TCGA-EJ-7794-01A	PRAD	Tumor	8.83
TCGA-EJ-7794-11A	PRAD	Normal	8.26
TCGA-EJ-7797-01A	PRAD	Tumor	8.64
TCGA-EJ-7797-11A	PRAD	Normal	8.84
TCGA-EJ-A8FO-01A	PRAD	Tumor	8.73
TCGA-EJ-A8FO-11A	PRAD	Normal	7.46
TCGA-G9-6333-01A	PRAD	Tumor	9.19
TCGA-G9-6333-11A	PRAD	Normal	7.49
TCGA-G9-6342-01A	PRAD	Tumor	7.94
TCGA-G9-6342-11A	PRAD	Normal	8.55
TCGA-G9-6348-01A	PRAD	Tumor	7.51
TCGA-G9-6348-11A	PRAD	Normal	7.64
TCGA-G9-6351-01A	PRAD	Tumor	8.6
TCGA-G9-6351-11A	PRAD	Normal	8.24
TCGA-G9-6356-01A	PRAD	Tumor	7.54
TCGA-G9-6356-11A	PRAD	Normal	8.29
TCGA-G9-6362-01A	PRAD	Tumor	8.66
TCGA-G9-6362-11A	PRAD	Normal	8.46

TCGA-G9-6363-01A	PRAD	Tumor	8.1
TCGA-G9-6363-11A	PRAD	Normal	7.84
TCGA-G9-6365-01A	PRAD	Tumor	8.21
TCGA-G9-6365-11A	PRAD	Normal	7.49
TCGA-G9-6384-01A	PRAD	Tumor	8.56
TCGA-G9-6384-11A	PRAD	Normal	8.23
TCGA-G9-6496-01A	PRAD	Tumor	8.22
TCGA-G9-6496-11A	PRAD	Normal	7.85
TCGA-G9-6499-01A	PRAD	Tumor	8.43
TCGA-G9-6499-11A	PRAD	Normal	7.51
TCGA-HC-7211-01A	PRAD	Tumor	8.96
TCGA-HC-7211-11A	PRAD	Normal	5.7
TCGA-HC-7737-01A	PRAD	Tumor	7.91
TCGA-HC-7737-11A	PRAD	Normal	6.34
TCGA-HC-7738-01A	PRAD	Tumor	9.05
TCGA-HC-7738-11A	PRAD	Normal	5.93
TCGA-HC-7740-01B	PRAD	Tumor	7.38
TCGA-HC-7740-11A	PRAD	Normal	5.86
TCGA-HC-7742-01A	PRAD	Tumor	7.89
TCGA-HC-7742-11A	PRAD	Normal	8.4
TCGA-HC-7745-01A	PRAD	Tumor	7.75
TCGA-HC-7745-11A	PRAD	Normal	6.08
TCGA-HC-7747-01A	PRAD	Tumor	8.48
TCGA-HC-7747-11A	PRAD	Normal	5.65
TCGA-HC-7752-01A	PRAD	Tumor	6.55
TCGA-HC-7752-11A	PRAD	Normal	7.63
TCGA-HC-7819-01A	PRAD	Tumor	8.71
TCGA-HC-7819-11A	PRAD	Normal	8.76
TCGA-HC-8258-01B	PRAD	Tumor	6.61
TCGA-HC-8258-11A	PRAD	Normal	5.72
TCGA-HC-8259-01A	PRAD	Tumor	8.86
TCGA-HC-8259-11A	PRAD	Normal	8.44
TCGA-HC-8260-01A	PRAD	Tumor	8.55
TCGA-HC-8260-11A	PRAD	Normal	8.87
TCGA-HC-8262-01A	PRAD	Tumor	8.22
TCGA-HC-8262-11A	PRAD	Normal	7.7
TCGA-J4-A83J-01A	PRAD	Tumor	7.48
TCGA-J4-A83J-11A	PRAD	Normal	7.66
TCGA-BR-6453-01A	STAD	Tumor	5.62
TCGA-BR-6453-11A	STAD	Normal	5.36
TCGA-BR-6454-01A	STAD	Tumor	6.06
TCGA-BR-6454-11A	STAD	Normal	5.37
TCGA-BR-6457-01A	STAD	Tumor	5.7
TCGA-BR-6457-11A	STAD	Normal	5.66
TCGA-BR-6458-01A	STAD	Tumor	6.94
TCGA-BR-6458-11A	STAD	Normal	5.67
TCGA-BR-6564-01A	STAD	Tumor	6.3
TCGA-BR-6564-11A	STAD	Normal	6.11
TCGA-BR-6802-01A	STAD	Tumor	6.48
TCGA-BR-6802-11A	STAD	Normal	5.39
TCGA-BR-6852-01A	STAD	Tumor	6.58
TCGA-BR-6852-11A	STAD	Normal	5.71
TCGA-BR-7703-01A	STAD	Tumor	6.8
TCGA-BR-7703-11A	STAD	Normal	6.31
TCGA-BR-7704-01A	STAD	Tumor	6.16
TCGA-BR-7704-11A	STAD	Normal	6.54
TCGA-BR-7715-01A	STAD	Tumor	6.67
TCGA-BR-7715-11A	STAD	Normal	5.67

TCGA-BR-7716-01A	STAD	Tumor	6.52
TCGA-BR-7716-11A	STAD	Normal	6.35
TCGA-BR-7717-01A	STAD	Tumor	5.83
TCGA-BR-7717-11A	STAD	Normal	5.74
TCGA-BR-7851-01A	STAD	Tumor	7.23
TCGA-BR-7851-11A	STAD	Normal	6.21
TCGA-BR-8060-01A	STAD	Tumor	6.25
TCGA-BR-8060-11A	STAD	Normal	5.77
TCGA-CG-5720-01A	STAD	Tumor	5.97
TCGA-CG-5720-11A	STAD	Normal	5.25
TCGA-CG-5721-01A	STAD	Tumor	6.46
TCGA-CG-5721-11A	STAD	Normal	4.74
TCGA-CG-5722-01A	STAD	Tumor	5.79
TCGA-CG-5722-11A	STAD	Normal	5.36
TCGA-CG-5734-01A	STAD	Tumor	4.86
TCGA-CG-5734-11A	STAD	Normal	5.28
TCGA-FP-7735-01A	STAD	Tumor	5.85
TCGA-FP-7735-11A	STAD	Normal	6.53
TCGA-FP-7829-01A	STAD	Tumor	6.78
TCGA-FP-7829-11A	STAD	Normal	7.04
TCGA-HU-8238-01A	STAD	Tumor	6.13
TCGA-HU-8238-11A	STAD	Normal	7.06
TCGA-HU-A4GC-01A	STAD	Tumor	5.96
TCGA-HU-A4GC-11A	STAD	Normal	6.17
TCGA-HU-A4GH-01A	STAD	Tumor	5.78
TCGA-HU-A4GH-11A	STAD	Normal	6.44
TCGA-HU-A4GN-01A	STAD	Tumor	7.2
TCGA-HU-A4GN-11A	STAD	Normal	6.36
TCGA-HU-A4GP-01A	STAD	Tumor	6.42
TCGA-HU-A4GP-11A	STAD	Normal	6.25
TCGA-HU-A4GY-01A	STAD	Tumor	6.4
TCGA-HU-A4GY-11A	STAD	Normal	6.38
TCGA-HU-A4HB-01A	STAD	Tumor	6.41
TCGA-HU-A4HB-11A	STAD	Normal	6.25
TCGA-IN-7806-01A	STAD	Tumor	6.94
TCGA-IN-7806-11A	STAD	Normal	6.48
TCGA-IN-8462-01A	STAD	Tumor	6.02
TCGA-IN-8462-11A	STAD	Normal	6.73
TCGA-IN-8663-01A	STAD	Tumor	8.23
TCGA-IN-8663-11A	STAD	Normal	6.46
TCGA-IN-AB1V-01A	STAD	Tumor	6.94
TCGA-IN-AB1V-11A	STAD	Normal	7.13
TCGA-IN-AB1X-01A	STAD	Tumor	7.33
TCGA-IN-AB1X-11A	STAD	Normal	6.54
TCGA-IP-7968-01A	STAD	Tumor	7.27
TCGA-IP-7968-11A	STAD	Normal	6.5
TCGA-BJ-A28R-01A	THCA	Tumor	7.31
TCGA-BJ-A28R-11A	THCA	Normal	7.16
TCGA-BJ-A28W-01A	THCA	Tumor	5.98
TCGA-BJ-A28W-11A	THCA	Normal	5.62
TCGA-BJ-A28X-01A	THCA	Tumor	6.98
TCGA-BJ-A28X-11A	THCA	Normal	6.9
TCGA-BJ-A290-01A	THCA	Tumor	7.17
TCGA-BJ-A290-11A	THCA	Normal	7.38
TCGA-BJ-A2N7-01A	THCA	Tumor	7.06
TCGA-BJ-A2N7-11A	THCA	Normal	7.64
TCGA-BJ-A2N8-01A	THCA	Tumor	7.56
TCGA-BJ-A2N8-11A	THCA	Normal	7.71

TCGA-BJ-A2N9-01A	THCA	Tumor	6.23
TCGA-BJ-A2N9-11A	THCA	Normal	6.89
TCGA-BJ-A2NA-01A	THCA	Tumor	7.29
TCGA-BJ-A2NA-11A	THCA	Normal	7.26
TCGA-BJ-A3PR-01A	THCA	Tumor	6.75
TCGA-BJ-A3PR-11A	THCA	Normal	7.16
TCGA-BJ-A3PU-01A	THCA	Tumor	6.75
TCGA-BJ-A3PU-11A	THCA	Normal	7.73
TCGA-DO-A1JZ-01A	THCA	Tumor	7.43
TCGA-DO-A1JZ-11A	THCA	Normal	7.33
TCGA-E8-A2JQ-01A	THCA	Tumor	7.06
TCGA-E8-A2JQ-11A	THCA	Normal	7.43
TCGA-EL-A3GZ-01A	THCA	Tumor	7.57
TCGA-EL-A3GZ-11A	THCA	Normal	7.84
TCGA-EL-A3H1-01A	THCA	Tumor	6.78
TCGA-EL-A3H1-11A	THCA	Normal	7.41
TCGA-EL-A3H2-01A	THCA	Tumor	7.38
TCGA-EL-A3H2-11A	THCA	Normal	7.48
TCGA-EL-A3H7-01A	THCA	Tumor	7.27
TCGA-EL-A3H7-11A	THCA	Normal	8.4
TCGA-EL-A3MW-01A	THCA	Tumor	7.2
TCGA-EL-A3MW-11A	THCA	Normal	7.66
TCGA-EL-A3MX-01A	THCA	Tumor	6.74
TCGA-EL-A3MX-11A	THCA	Normal	7.2
TCGA-EL-A3MY-01A	THCA	Tumor	7.58
TCGA-EL-A3MY-11A	THCA	Normal	7.71
TCGA-EL-A3N2-01A	THCA	Tumor	7.01
TCGA-EL-A3N2-11A	THCA	Normal	7.34
TCGA-EL-A3N3-01A	THCA	Tumor	7.15
TCGA-EL-A3N3-11A	THCA	Normal	8.1
TCGA-EL-A3T0-01A	THCA	Tumor	7.13
TCGA-EL-A3T0-11A	THCA	Normal	7.1
TCGA-EL-A3T1-01A	THCA	Tumor	6.48
TCGA-EL-A3T1-11A	THCA	Normal	7.72
TCGA-EL-A3T2-01A	THCA	Tumor	7.03
TCGA-EL-A3T2-11A	THCA	Normal	7.12
TCGA-EL-A3T3-01A	THCA	Tumor	7.28
TCGA-EL-A3T3-11A	THCA	Normal	7.36
TCGA-EL-A3T6-01A	THCA	Tumor	7.19
TCGA-EL-A3T6-11A	THCA	Normal	7.85
TCGA-EL-A3T7-01A	THCA	Tumor	7.38
TCGA-EL-A3T7-11A	THCA	Normal	6.88
TCGA-EL-A3T8-01A	THCA	Tumor	7.19
TCGA-EL-A3T8-11A	THCA	Normal	7.54
TCGA-EL-A3TA-01A	THCA	Tumor	7.09
TCGA-EL-A3TA-11A	THCA	Normal	7.5
TCGA-EL-A3TB-01A	THCA	Tumor	7.04
TCGA-EL-A3TB-11A	THCA	Normal	7.35
TCGA-EL-A3ZG-01A	THCA	Tumor	6.83
TCGA-EL-A3ZG-11A	THCA	Normal	7.85
TCGA-EL-A3ZH-01A	THCA	Tumor	7.42
TCGA-EL-A3ZH-11A	THCA	Normal	7.39
TCGA-EL-A3ZK-01A	THCA	Tumor	6.58
TCGA-EL-A3ZK-11A	THCA	Normal	7.14
TCGA-EL-A3ZL-01A	THCA	Tumor	6.59
TCGA-EL-A3ZL-11A	THCA	Normal	7.5
TCGA-EL-A3ZM-01A	THCA	Tumor	7.05
TCGA-EL-A3ZM-11A	THCA	Normal	6.82

TCGA-EL-A3ZO-01A	THCA	Tumor	6.82
TCGA-EL-A3ZO-11A	THCA	Normal	7.54
TCGA-EL-A3ZP-01A	THCA	Tumor	7.16
TCGA-EL-A3ZP-11A	THCA	Normal	6.85
TCGA-EL-A3ZQ-01A	THCA	Tumor	5.78
TCGA-EL-A3ZQ-11A	THCA	Normal	6.98
TCGA-EL-A3ZR-01A	THCA	Tumor	6.49
TCGA-EL-A3ZR-11A	THCA	Normal	7.23
TCGA-EL-A3ZS-01A	THCA	Tumor	7.38
TCGA-EL-A3ZS-11A	THCA	Normal	6.1
TCGA-EL-A3ZT-01A	THCA	Tumor	7.54
TCGA-EL-A3ZT-11A	THCA	Normal	7.11
TCGA-EM-A1CS-01A	THCA	Tumor	6.67
TCGA-EM-A1CS-11A	THCA	Normal	7.34
TCGA-EM-A1CT-01A	THCA	Tumor	6.67
TCGA-EM-A1CT-11A	THCA	Normal	7.42
TCGA-EM-A1CU-01A	THCA	Tumor	7.87
TCGA-EM-A1CU-11A	THCA	Normal	7.82
TCGA-EM-A1CV-01A	THCA	Tumor	7.43
TCGA-EM-A1CV-11A	THCA	Normal	7.7
TCGA-EM-A1CW-01A	THCA	Tumor	6.81
TCGA-EM-A1CW-11A	THCA	Normal	7.96
TCGA-EM-A1YC-01A	THCA	Tumor	5.75
TCGA-EM-A1YC-11A	THCA	Normal	6.36
TCGA-EM-A3ST-01A	THCA	Tumor	6.89
TCGA-EM-A3ST-11A	THCA	Normal	7.35
TCGA-ET-A2MX-01A	THCA	Tumor	7.17
TCGA-ET-A2MX-11C	THCA	Normal	7.04
TCGA-ET-A2N5-01A	THCA	Tumor	6.45
TCGA-ET-A2N5-11B	THCA	Normal	7.69
TCGA-ET-A3DP-01A	THCA	Tumor	7.3
TCGA-ET-A3DP-11A	THCA	Normal	7.85
TCGA-ET-A3DW-01A	THCA	Tumor	7.19
TCGA-ET-A3DW-11A	THCA	Normal	7.97
TCGA-FY-A3TY-01A	THCA	Tumor	7.08
TCGA-FY-A3TY-11A	THCA	Normal	7.35
TCGA-GE-A2C6-01A	THCA	Tumor	7.08
TCGA-GE-A2C6-11A	THCA	Normal	7.14
TCGA-H2-A2K9-01A	THCA	Tumor	7.45
TCGA-H2-A2K9-11A	THCA	Normal	7.6
TCGA-H2-A3RI-01A	THCA	Tumor	7
TCGA-H2-A3RI-11A	THCA	Normal	7.3
TCGA-KS-A41I-01A	THCA	Tumor	5.08
TCGA-KS-A41I-11A	THCA	Normal	6.98
TCGA-KS-A41J-01A	THCA	Tumor	6.81
TCGA-KS-A41J-11A	THCA	Normal	7.38
TCGA-KS-A41L-01A	THCA	Tumor	6.43
TCGA-KS-A41L-11A	THCA	Normal	7.04
TCGA-AJ-A2QL-01A	UCEC	Tumor	6.6
TCGA-AJ-A2QL-11A	UCEC	Normal	6.77
TCGA-AJ-A3NC-01A	UCEC	Tumor	6.9
TCGA-AJ-A3NC-11A	UCEC	Normal	7.12
TCGA-AJ-A3NE-01A	UCEC	Tumor	6.75
TCGA-AJ-A3NE-11A	UCEC	Normal	6.69
TCGA-AJ-A3NH-01A	UCEC	Tumor	5.21
TCGA-AJ-A3NH-11A	UCEC	Normal	7.26
TCGA-AX-A05Y-01A	UCEC	Tumor	4.98
TCGA-AX-A05Y-11A	UCEC	Normal	7.39

TCGA-AX-A0IZ-01A	UCEC	Tumor	5.81
TCGA-AX-A0IZ-11A	UCEC	Normal	7.58
TCGA-AX-A0J0-01A	UCEC	Tumor	6.36
TCGA-AX-A0J0-11A	UCEC	Normal	7.28
TCGA-AX-A1CF-01A	UCEC	Tumor	6.77
TCGA-AX-A1CF-11A	UCEC	Normal	6.74
TCGA-AX-A1CI-01A	UCEC	Tumor	4.79
TCGA-AX-A1CI-11A	UCEC	Normal	7.04
TCGA-AX-A1CK-01A	UCEC	Tumor	5.26
TCGA-AX-A1CK-11A	UCEC	Normal	7
TCGA-AX-A2H8-01A	UCEC	Tumor	6.7
TCGA-AX-A2H8-11A	UCEC	Normal	7.09
TCGA-AX-A2HA-01A	UCEC	Tumor	5.46
TCGA-AX-A2HA-11A	UCEC	Normal	6.6
TCGA-AX-A2HC-01A	UCEC	Tumor	6.92
TCGA-AX-A2HC-11A	UCEC	Normal	7.2
TCGA-AX-A2HD-01A	UCEC	Tumor	6.18
TCGA-AX-A2HD-11A	UCEC	Normal	6.95
TCGA-BG-A2AD-01A	UCEC	Tumor	6.55
TCGA-BG-A2AD-11A	UCEC	Normal	7.11
TCGA-BG-A3EW-01A	UCEC	Tumor	6.54
TCGA-BG-A3EW-11A	UCEC	Normal	7.21
TCGA-BG-A3PP-01A	UCEC	Tumor	5.56
TCGA-BG-A3PP-11A	UCEC	Normal	7.16
TCGA-BK-A0CB-01A	UCEC	Tumor	5.42
TCGA-BK-A0CB-11A	UCEC	Normal	6.65
TCGA-BK-A13C-01A	UCEC	Tumor	6.65
TCGA-BK-A13C-11A	UCEC	Normal	7.17
TCGA-BK-A4ZD-01A	UCEC	Tumor	6.75
TCGA-BK-A4ZD-11A	UCEC	Normal	7.16
TCGA-DI-A2QU-01A	UCEC	Tumor	5.41
TCGA-DI-A2QU-11A	UCEC	Normal	6.59
TCGA-DI-A2QY-01A	UCEC	Tumor	5.8
TCGA-DI-A2QY-11A	UCEC	Normal	7.21
TCGA-E6-A1M0-01A	UCEC	Tumor	6.52
TCGA-E6-A1M0-11A	UCEC	Normal	7.76
