# Supplementary material for: c-Fos-driven metabolic switch of α-ketoglutarate orchestrates progression in prostate cancer
Source: Cell Death Dis. 2026 May 31;17(1):574. doi: 10.1038/s41419-026-08918-4 (PMC13273188; doi:10.1038/s41419-026-08918-4)
Supplement: Supplementary file 3 — Supplementary Figure legends [file 41419_2026_8918_MOESM3_ESM.docx]

**Supplementary Figure Legends**

**Fig. S1**

A: Schematic diagram of the digestion experiment.

B: GLUD1 expression levels in paired tumor and benign tissues from the TCGA pan-cancer analysis.

C: Prognostic analysis of patients stratified by IDH1 expression levels in the TCGA database.

D: Ratio of relative RNA expression levels of GLUD1 to IDH1 in PC-3 cells.

ns p>0.05, *p<0.05, **p<0.01, ***p<0.001, ****p<0.0001
